# Deficiencies in clinical reasoning of LLMs in low back pain management and remediation via prompt engineering: from performance evaluation to error diagnosis

**DOI:** 10.3389/frai.2026.1811701

**Published:** 2026-05-25

**Authors:** Jia-Hui Luo, Yi-Lin Wang, Min-Jun Zhao, Jian-Li Yin, Dao-Fang Ding, Xu-Bo Wu

**Affiliations:** 1 School of Rehabilitation Science, Shanghai University of Traditional Chinese Medicine, Shanghai, China; 2 Department of Rehabilitation Medicine of Shanghai Pudong New Area People's Hospital, Shanghai, China; 3 Department of Rehabilitation Therapy, The Second Rehabilitation Hospital of Shanghai, Shanghai, China; 4 Department of Orthopedic, Guanghua Hospital of Integrative Chinese and Western Medicine, Shanghai, China

**Keywords:** clinical decision support, large language models, low back pain, prompt engineering, rehabilitation management

## Abstract

**Background:**

Large language models show promise in medical tasks, but their systematic error patterns in high-stakes clinical settings remain poorly understood, limiting safe deployment.

**Methods:**

A three-phase simulation study was conducted. In phase 1, researchers selected 103 multiple-choice questions and 30 clinical scenario questions, derived from an LBP examination question bank and clinical guidelines and systematically evaluated five mainstream LLMs (GPT-5, GPT-4o, GPT-o3, Deepseek-V2.5, and Grok-4) across six dimensions: accuracy, completeness, practicality, readability, safety, and output stability. In Phase 2, two clinical coders independently performed qualitative content analysis on responses with low scores from Phase 1 (≤ 3 any dimension), classified error types, and calculated inter-rater reliability (Cohen's κ = 0.84); consensus was reached through discussion. In Phase 3, targeted safety-oriented prompts were designed for the high-risk error categories identified in Phase 2, and a separate linear mixed model was fitted for each of the five evaluation dimensions (*n* = 7 questions). Given the small sample size, the effect size (Hedges' g) serves as the primary indicator of practical significance.

**Results:**

All five models achieved accuracy rates exceeding 90% on the general LBP knowledge test, demonstrating solid foundational knowledge. GPT-4o exhibited the highest overall clinical quality score and output stability. Error attribution revealed that lower-performing models, particularly Deepseek-V2.5, produced more safety-critical errors, including factual hallucinations and omissions of safety warnings. Targeted prompt engineering produced significant improvements for Deepseek-V2.5 across all five evaluation dimensions (*p* < 0.001), with the largest gains in safety and completeness. GPT-4o showed significant improvements in four dimensions but not in safety (*p* = 0.227). A significant model × condition interaction was observed only for safety (*p* = 0.002).

**Conclusion:**

Although all models demonstrated strong foundational medical knowledge, their ability to translate this knowledge into reliable clinical guidance varied substantially. Critical concerns extend beyond factual accuracy to encompass safety and completeness in real-world clinical contexts. Human oversight remains indispensable. Clinicians should recognize the distinct strengths and limitations of different models and select tools according to their specific clinical use cases. Structured prompt design and systematic fact-checking represent the most practical and scalable approaches to enhancing safety, particularly in resource-limited settings. This study contributes to a more nuanced understanding of LLMs' capabilities and risks in chronic disease management and provides a replicable methodological foundation for future clinical AI evaluation.

## 1 Introduction

Low back pain (LBP) is the leading cause of disability and productivity loss worldwide, posing a significant public health challenge. According to the Global Burden of Disease study, approximately 619 million people were affected by LBP globally in 2020, with the number projected to reach 843 million by 2050 (GBD 2021 Other Musculoskeletal Disorders Collaborators, 2023). More concerningly, recent evidence indicates that the burden of LBP is rising significantly among working-age populations. A 2025 study reported that risk factors such as high body mass index continue to drive increases in disability-adjusted life years, with the overall disease burden of LBP expected to further rise by 2035 (Wu et al., 2025). From a clinical perspective, effective LBP management requires a multimodal approach rather than reliance on a single treatment modality, emphasizing evidence-based patient education, lifestyle modifications, and long-term, adaptive rehabilitation guidance. Ideally, these interventions should be individualized and continuously adjusted in response to disease progression. However, in real-world clinical settings, healthcare providers are often constrained by time, workforce shortages, and the rapid expansion of medical knowledge, making it challenging to consistently deliver timely, accurate, and highly personalized care to all patients. Against this backdrop, the rapid advancement of LLMs has garnered widespread attention. With advanced capabilities in natural language understanding and generation, LLMs are increasingly viewed as highly scalable tools with potential applications in clinical practice. Emerging evidence indicates that these models perform well in medical knowledge question-answering, clinical decision support, and patient education. In certain standardized assessment scenarios, their performance has matched or even surpassed that of human experts (Kung et al., 2023). Models such as ChatGPT can generate structured patient education materials and provide seemingly plausible explanations and recommendations for common clinical queries. However, deeper analyses reveal that their responses still exhibit significant variability in accuracy, information completeness, and readability (Singhal et al., 2025). Recent studies have begun exploring the application of LLMs in chronic pain management, such as understanding patients' pain experiences or assisting in developing personalized intervention plans. However, research also indicates that model performance may significantly decline when applied to high-risk or complex clinical scenarios (Li et al., 2025).

These limitations highlight critical challenges in translating LLMs into reliable clinical tools. Current evaluation efforts primarily focus on overall accuracy or general benchmarking (Agrawal et al., 2025), frequently overlooking more subtle yet higher-risk issues inherent in clinical reasoning, such as the generation of hallucinations, the omission of critical information, and the provision of potentially unsafe recommendations (Wang et al., 2025).

In real-world practice, failing to identify potential risks or providing misleading rehabilitation guidance may be more severe than simple factual errors (Normand et al., 2025). Prompt engineering is a strategy designed to mitigate these shortcomings. It involves carefully crafting target inputs to guide the model's output. Evidence suggests this approach can enhance the accuracy and safety of medical tasks. However, systematic evaluations of LLM applications in LBP rehabilitation remain limited. Therefore, this study focuses on the prevalent and complex clinical domain of chronic LBP rehabilitation management, proposing a three-stage research paradigm encompassing assessment, attribution, and prompt-based targeted correction.

The study first conducted multidimensional benchmarking of multiple mainstream LLMs, comparing their performance differences across clinically relevant tasks. It then performs systematic error attribution analysis to characterize the distribution and nature of errors generated in clinical contexts. Building upon this foundation, the research designed and validated targeted safety prompt engineering interventions tailored to specific risk characteristics, exploring feasible pathways to reduce high-risk errors. The error classification system, five-dimensional clinical assessment framework, and minimized safety prompt strategy developed here hold potential for transferability to other guideline-driven chronic disease management domains, such as heart failure and diabetes. We have not only more clearly defined the capability boundaries and potential risks of current LLMs in chronic LBP rehabilitation guidance, but also provided a replicable methodological framework that lays the groundwork for the safer and more reliable integration of LLMs into clinical practice in the future.

## 2 Materials and methods

### 2.1 Study design

**Figure 1:**
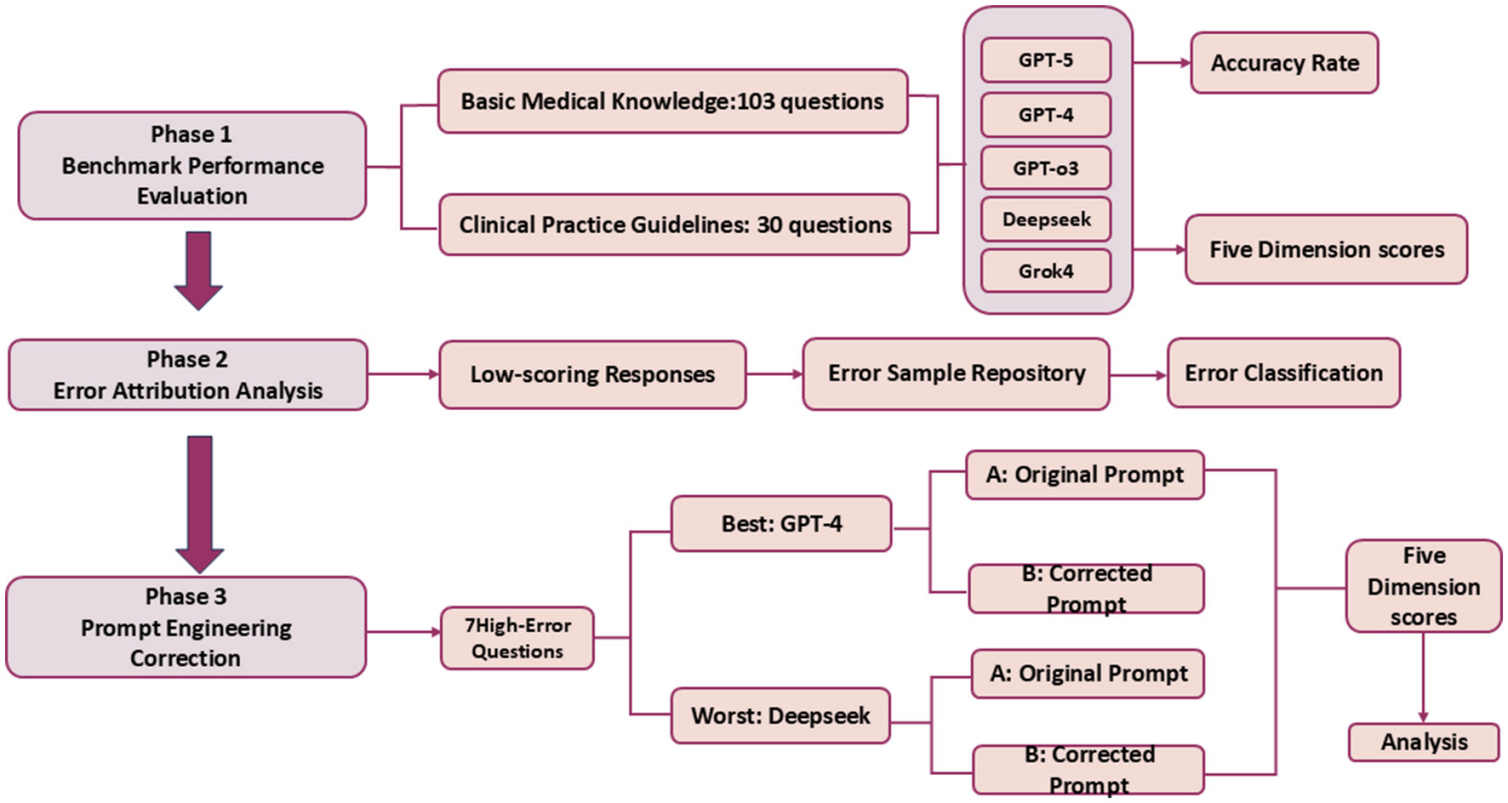
Five LLMs were selected. In the first phase, experts evaluated their performance on medical knowledge and clinical practice guidelines for LBP across five dimensions and tested their output stability. In the second phase, an error sample repository was constructed based on their low-scoring performance. In the third phase, the worst-performing and best-performing models were selected for comparative experiments before and after prompt modification.

This study employed a three-phase simulation design aimed at evaluating the performance of LLMs in providing clinical information related to LBP. In Phase 1, baseline performance was assessed with respect to foundational medical knowledge and clinical practice guidelines. Phase 2 focused on error attribution analysis of responses that received low scores in Phase 1. Phase 3 was designed as an exploratory proof-of-concept study to investigate whether targeted prompt engineering, tailored to the common error types identified in Phase 2, could improve response quality. Phase 3 primarily focuses on improvement trends and effect sizes. The overall workflow is illustrated in Figure 1.

### 2.2 Models

Five mainstream, publicly accessible LLMs were selected: GPT-5, GPT-4o, GPT-o3, Deepseek-V2.5, and Grok-4. All models were accessed via their respective official web interfaces without additional fine-tuning. Specific model version information is provided in Supplementary Material 1.

### 2.3 Evaluation materials

#### 2.3.1 Phase 1: benchmark performance evaluation

1) Source

Multiple-choice questions: a total of 103 multiple-choice questions were selected from the past exam question banks of the National Medical Licensing Examination (2018–2025) for the specialties of Rehabilitation Medicine and the Orthopedic Surgery specialty, as presented in Supplementary Material 2. The model was assigned the following role definition: “focus on medical knowledge related to LBP and answer the following questions.” All questions were input into the model's online interface, and the generated answers were collected and compared with the standard answers to calculate overall and domain-specific accuracy. To reduce bias caused by fixed answer positions, the order of the options (A, B, C, D) was randomized using a computer-generated random number table. Each question was tested three times, yielding in a total of 309 tests per model. The random sequences were generated by personnel outside the research team.

Clinical Scenario Questions: a total of 30 standardized, clinically relevant questions were developed by two experienced LBP specialists based on both domestic and international clinical guidelines, including the ACP Guidelines (Qaseem et al., 2017), NICE Guidelines [National Guideline Centre (UK), 2016], European Guidelines (Airaksinen et al., 2006), WHO Guidelines (Moser et al., 2024), and the Chinese Guides to Lower Back Pain. Discrepancies during question development were resolved through consultation with a third senior expert. Each question was finalized through independent discussion sessions. Before the session began, the model was instructed: “as an LBP rehabilitation specialist, please answer the following questions based on the latest clinical guidelines for LBP.” The 30 questions were evaluated over three independent rounds of testing. In each round, a new, isolated chat session was initiated. After providing the initial role-definition prompt, the 30 clinical scenario questions were presented sequentially within that single session, and the model's responses were collected. This process was repeated three times (Round 1, 2, and 3) using completely separate chat sessions, resulting in three independently generated response sets per question.

2) Screening process and inclusion criteria

Multiple-choice questions were independently screened by two rehabilitation physicians specializing in LBP. Inclusion criteria were as follows: (1) the question content is directly related to core knowledge in the clinical diagnosis and treatment of LBP; (2) the correct answer is clearly supported by the latest clinical guidelines; and (3) the question is clearly worded and unambiguous. Exclusion criteria included: (1) questions involving highly specialized content; (2) questions based on research that is clearly outdated; (3) questions that are highly controversial or the presence of multiple correct answers. After an independent assessment, discrepancies between the two screeners were resolved through discussion until a consensus was reached. A total of 103 questions were ultimately retained and categorized into seven sub-domains based on clinical diagnostic and therapeutic frameworks: definitions (6 questions), Etiology (14 questions), Clinical Manifestations (21 questions), Clinical Examination (12 questions), Imaging Studies (16 questions), Clinical Treatment (13 questions), and Rehabilitation Therapy (22 questions).

Clinical scenario questions: two rehabilitation physicians specializing in LBP initially developed 45 clinical scenario questions based on the latest clinical guidelines for LBP. The clinical relevance of each scenario was subsequently evaluated using a 5-point Likert scale (1 = not at all relevant, 5 = highly relevant), assessing whether the scenario represented a typical and clinically important issue in LBP management. Inclusion criteria were defined as follows: (1) the mean clinical relevance score ≥4 across three experts and a score ≥4 from at least two experts. Based on these criteria, a final set of 30 clinical scenario questions was selected. Detailed question and corresponding LLM-generated content are provided in Supplementary Material 3.

#### 2.3.2 Phase 2: attribution of errors

A total of 30 clinical scenario questions from Phase 1 were selected for content scoring analysis. Responses in which any single evaluation dimension received a score of ≤ 3 were identified as low-quality outputs. From a clinical perspective, we chose 3 as the cutoff value. On a 5-point Likert scale, a score of 3 represents “average,” which may indicate clinically meaningful deficiencies, such as omitted safety warnings, incomplete information, or recommendations lacking practical applicability, thereby highlighting and clearly identifying areas for requiring improvement. In contrast, scores of 4 or 5 represent “Good” or “Excellent,” indicating relatively minor deficiencies, making it difficult to detect the effects of interventions. Finally, all eligible low-scoring content was compiled into an error sample database. Based on clinical reasoning processes and patient safety principles, a five-dimensional error classification framework was developed in collaboration with two senior clinical experts (Appendix 1). This error sample repository served as the source pool for selecting questions in the subsequent exploratory prompt-engineering phase (Phase 3). Specific inclusion criteria for Phase 3 are detailed in the following section.

#### 2.3.3 Exploration phase: rapid engineering validation testing

In Phase 3, seven issues were selected from the Phase 2 error sample repository for intervention testing. Selection criteria required that each issue received a scored ≤ 3 in at least three of the five evaluation dimensions (accuracy, completeness, practicality, safety, and readability), thereby representing high-risk and systematically deficient cases. Targeted correction prompts were then developed to address the common and high-risk error types identified in Phase 2. The prompts emphasized the following principles: “Verify and disclose all safety precautions, ensure that all medical claims are supported by evidence, report uncertainties accurately, and provide specific, actionable details.”

### 2.4 Process

#### 2.4.1 Phase 1: scoring process for clinical guidelines multiple-choice questions

Three orthopedic rehabilitation specialists independently conducted blinded evaluations of the model-generated responses using a five-point Likert scale (1 = very poor, 5 = excellent) across five predefined dimensions: accuracy, completeness, safety, readability, and practicality. Detailed definitions and scoring criteria specific to clinical LBP are provided in Supplementary Material 4.

a) Pre-scoring training (conducted 1 week before formal evaluation):
  1. Guideline review: participants collectively reviewed the core recommendations of the NICE NG59, ACP, and WHO LBP guidelines to ensure alignment with current clinical standards.
  2. Explanation of scoring criteria: the principal investigator provided a structured explanation of each evaluation dimension and the corresponding scoring criteria (1–5 scale).
  3. Preliminary scoring: 10 informal test questions (excluded in the final analysis) were independently scored by three raters, Discrepancies were then discussed collectively to achieve consensus and calibrate scoring consistency, if κ ≥ 0.80, proceed to the formal evaluation; if κ = 0.70–0.79, resolve discrepancies through consultation; if κ = 0.60–0.69, recalibrate scoring criteria and repeat pilot evaluation; if κ < 0.60, restart the training process. Particular attention was given to dimensions with the greatest disagreement, and ambiguous scoring criteria were revised accordingly.
b) Formal scoring

The five content files generated by the LLMs were assigned randomized labels and distributed to experts for blind evaluation.

c) Post-scoring

Score data across five dimensions were collected for subsequent statistical analysis.

Additionally, as both this study was conducted entirely in Chinese and the generated text was conducted in Chinese, the LDU-TGP platform was utilized to calculate three additional objective metrics based on Chinese text features: text difficulty score, educational level, and recommended reading age (http://120.27.70.114:8000/analysis_a). LDU-TGP provides a standardized assessment of the complexity of Chinese texts, with higher scores indicating lower comprehensibility (Cheng et al., 2020).

#### 2.4.2 Phase 2: attribution of errors

The research team reviewed existing literature on error patterns and misclassification in LLM-based clinical evaluations (Seo et al., 2024). Based on commonly reported error types and in alignment with patient safety and data integrity principles emphasized in LBP clinical guidelines, a preliminary five-dimensional error classification framework was developed (Altermatt et al., 2025). This five-dimensional framework was further operationalized into a detailed 15-category error taxonomy, which is presented in full in Appendix 1.

Two coders underwent a standardized 2 h training session before formal coding. The training included: (1) a detailed review of operational definitions and classification criteria for each dimension; (2) group discussion to clarify ambiguities; (3) independent coding of five pilot responses; and (4) calibration of coding results to ensure consistency. Cohen's kappa coefficient was used to assess the inter-rater agreement between the two coders' classifications across the five dimensions, yielding an overall mean kappa coefficient of 0.84 to which indicates strong agreement and supports the reliability of independent coding(≥0.80). During the formal coding process, discrepancies in classification were resolved through a structured consensus procedure. Specifically, each coder re-examined the original responses and corresponding classification criteria, followed by discussion to reconcile differences. If consensus could not be achieved, a third senior expert was consulted to make the final determination.

#### 2.4.3 Phase 3: rapid engineering validation testing

Based on the results of Phase 1, we selected GPT-4o (the best-performing model) and Deepseek-V2.5 (the worst-performing model) to test the improved prompts. A paired design was employed using seven selected clinical questions. In the control condition (Group A), the original prompt was “Please answer the following questions based on the latest clinical guidelines for low back pain”. In the intervention condition (Group B), a modified prompt “Original prompt, plus verification and communication of all safety precautions, ensuring all medical statements are based on evidence-based medicine, accurately disclosing uncertainties, and providing actionable details” (see Supplementary material 5) was applied. During the response generation process, each model generated content under the original and modified prompts in separate chat windows, based on the original and modified prompts. respectively, with all seven questions were generated within the same chat window session to maintain contextual consistency. As given the exploratory nature of this phase this phase was exploratory, only one response per question under each condition was generated, per question and condition, yielding a total of 28 responses. All generation tasks were performed by the same research assistant to ensure operational consistency. Subsequently, responses from both groups were mixed, anonymized, and randomized before evaluation. Detailed procedures for blinding and scoring are consistent with those described in the Phase 1 clinical scenario assessment. Specific personnel and procedures are detailed in the scoring process for Phase 1 clinical guideline questions.

### 2.5 Statistical analysis

In the clinical scenario questions of the first phase, inter-rater reliability was assessed using a two-way random-effects Intraclass Correlation Coefficient (ICC) for absolute agreement. The average-measures ICC was 0.62, indicating moderate agreement among the three raters. Given this moderate level of reliability, a linear mixed model was used to compare scores for different models within each evaluation dimension, with “clinical questions” and “raters” specified as random effects. *Post hoc* pairwise comparisons were conducted based on the model-estimated marginal means, with Bonferroni correction applied to control for Type I error inflation due to multiple comparisons. All pairwise comparison results are presented as corrected *p*-values; statistical significance was defined as *p* < 0.05. Data obtained from the LDU-TGP platform were analyzed using the same statistical methods as those applied in the clinical scenario scores.

To assess the output stability in Phase 1, the variance of scores obtained from three independent answer generations across all clinical questions was calculated for each model. Subsequently, nonparametric paired comparisons of score variances between all models were performed using the Wilcoxon signed-rank test. Given that a total of 10 pairwise comparisons were conducted, to control for Type I error inflation resulting from multiple comparisons, all *p*-values were Bonferroni-corrected, with the corrected significance level set at α' = 0.005. A statistically significant difference in output stability between two models was defined as the corrected *p*-value < 0.005. In Phase 3, a separate linear mixed model was fitted for each of the five evaluation dimensions. In each model, the dimension-specific score served as the dependent variable. Fixed effects included Model (GPT-4o vs. DeepSeek-V2.5), Condition (baseline vs. corrected prompt), and their interaction. Random intercepts were specified for QuestionID and RaterID to account for the nested data structure. Given the exploratory nature and small sample size (*n* = 7), effect sizes (Hedges' g) are reported as the primary metric of interest, with *p*-values from the mixed models reported descriptively. For the first stage, which had a larger sample size, Cohen's d was used as the effect size measure. Effect sizes were interpreted using standard thresholds: |d| or |g| ≥0.2 indicates a small effect, ≥0.5 indicates a moderate effect, and ≥0.8 indicates a large effect. All statistical analyses were conducted using SPSS 27.0 software, with a two-sided significance level of α = 0.05.

## 3 Results

### 3.1 Multi-model benchmark performance evaluation

#### 3.1.1 General medical knowledge test on LBP

**Figure 2:**
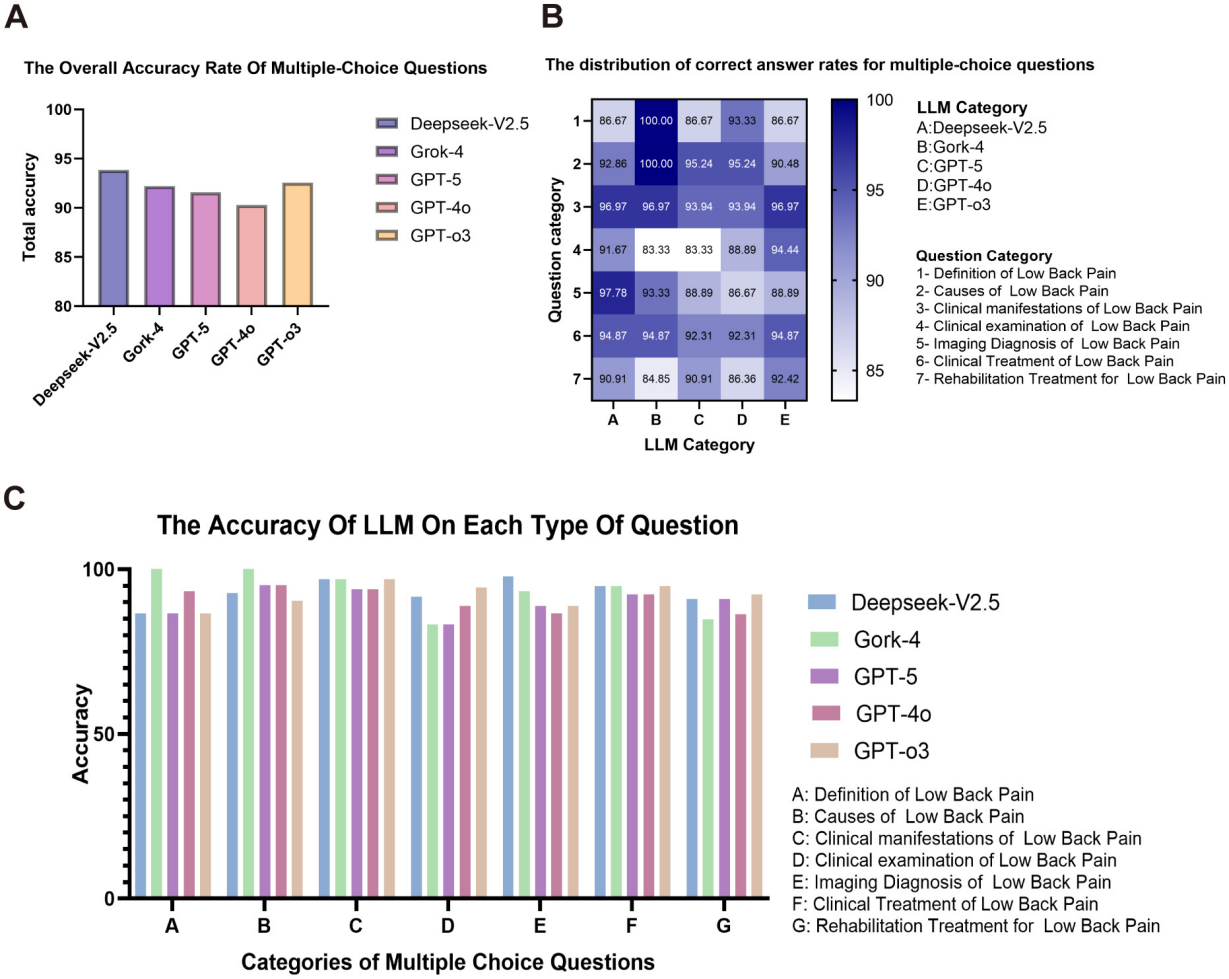
Results of the general LBP medical knowledge test. **(A)** displays the overall accuracy rates of each model in the medical knowledge test on LBP, with the Y-axis showing accuracy percentages. **(B)** illustrates the varying error patterns across the seven major clinical subfields for each large model, where darker colors indicate higher error rates and poorer performance in that category. **(C)** visually compares the performance of five models across the seven major clinical subfields.

Figure 2A shows the overall accuracy of all evaluated models on multiple-choice questions. All five models achieved accuracy rates exceeding 90%, indicating a solid foundation of LBP. Deepseek-V2.5 achieved the highest overall accuracy (93.85%), followed by GPT-o3 (92.55%), Grok-4 (92.23%), GPT-5 (91.58%), and GPT-4o (90.29%).

When performance was disaggregated across the seven clinical subdomains (Figures 2B, C), a more nuanced pattern emerged. Accuracy remained above 85% across all models for questions concerning LBP etiology (Category B), clinical manifestations (Category C), and treatment (Category F), indicating relatively robust performance in these domains. In contrast, comparatively lower accuracy was observed in domains involving clinical definition (Category A), clinical examination (Category D), and imaging analysis (Category E), suggesting potential weakness in areas requiring more precise model reasoning and comprehension.

#### 3.1.2 Clinical guideline adherence and output quality assessment

**Figure 3:**
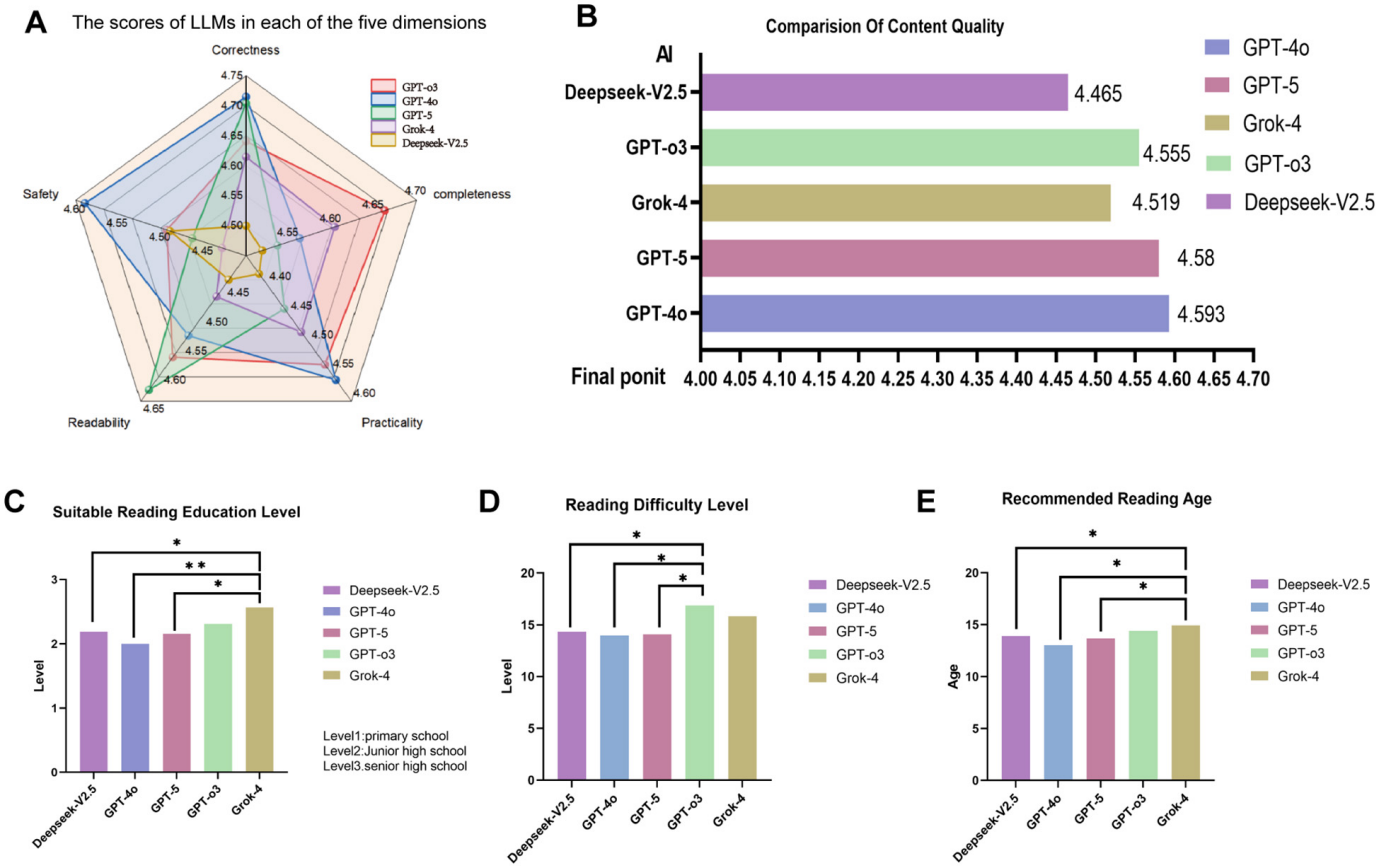
Assessment of clinical guideline adherence and outcome quality. **(A)** presents a comprehensive comparison of outputs generated by various LLMs based on clinical guidelines across dimensions of accuracy, completeness, readability, practicality, and safety. **(B)** displays the overall quality scores of outputs generated by each large language model based on clinical guidelines. **(C)** indicates the age groups for which each large language model's outputs are most suitable. **(D)** shows the corresponding reading difficulty levels of outputs from each large language model. **(E)** indicates the educational level corresponding to the output content of each large model.

Blinded expert evaluation of model-generated responses to the clinical scenario questions showed that all five models achieved mean composite scores exceeding 4.3 across the five evaluation dimensions (Figure 3A and Table 1), indicating generally high overall clinical response quality. As shown in Figure 3B, GPT-4o achieved the highest composite score (approximately 4.593), followed by GPT-5 (4.580), GPT-o3 (4.555), Grok-4 (4.519), and Deepseek-V2.5 (4.465). The highest-scoring model on each dimension was as follows: GPT-4o for accuracy (4.715); GPT-5 for completeness (4.663); GPT-4o for practicality (4.563); GPT-o3 for readability (4.630); GPT-4o for safety (4.589).

**Table 1:** Estimated Marginal Means of each LLM across the five-dimensional evaluation.

*n* = 30 questions × 5 models × 3 generations	Deepseek-V2.5	GPT-5	GPT-4o	GPT-o3	Grok-4
Accuracy	4.500	4.641	4.715	4.704	4.615
Completeness	4.519	4.663	4.563	4.537	4.604
Practicality	4.381	4.537	4.563	4.441	4.481
Readability	4.441	4.574	4.537	4.630	4.470
Safety	4.489	4.493	4.589	4.463	4.428

As summarized in Table 2, Deepseek-V2.5 scored the lowest on most dimensions. Compared to the GPT series models, statistically significant differences were found in accuracy (*p* < 0.001, Cohen's *d* = 0.22–0.33), practicality (*p* < 0.05, Cohen's *d* = 0.24), and readability (*p* < 0.05, Cohen's *d* = 0.22–0.32). No statistically significant differences were observed between models in the safety dimension (*p* > 0.05).

**Table 2:** Pairwise comparison results of LLMs based on five dimensions of evaluation.

Comparison (A vs. B)	Accuracy	Completeness	Practicality	Readability	Safety
Deepseek-V2.5 vs. GPT-4o	−0.215^*^	−0.041	−0.178^*^	−0.107	−0.100
Deepseek-V2.5 vs. GPT-5	−0.204^*^	−0.015	−0.056	−0.200^*^	0.026
Deepseek-V2.5 vs. GPT-o3	−0.141^*^	−0.137	−0.152	−0.141^*^	−0.004
Deepseek-V2.5 vs. Grok-4	−0.115	−0.085	−0.093	−0.037	0.063
GPT-4o vs. GPT-5	0.011	0.026	0.122	−0.093	0.126
GPT-4o vs. GPT-o3	0.074	−0.096	0.026	−0.033	0.096
GPT-4o vs. Grok-4	0.100	−0.044	0.085	0.070	0.163
GPT-5 vs. GPT-o3	0.063	−0.122	−0.096	0.059	−0.030
GPT-5 vs. Grok-4	0.089	−0.070	−0.037	0.163^*^	0.037
GPT-o3 vs. Grok-4	0.026	0.052	0.059	0.104	0.067

Values are mean differences (**A**–**B**). Values marked with an “^*^” indicate significant differences (*p* < 0.05).

Objective readability was assessed using the LDU-TGP platform (Figures 3C–E). Deepseek-V2.5 received the highest reading difficulty score (16.871), indicating that its outputs require a higher level of text comprehension. followed by Grok-4(15.827). In contrast, GPT-5, GPT-4o, and GPT-o3 demonstrated comparatively lower difficulty scores. In terms of recommended reading age, Grok-4 received the highest value (14.933), followed by Deepseek-V2.5 (14.422) and GPT-4o (13.022). Overall, outputs from all models corresponded to secondary-school-level educational materials. Regarding educational level suitability, Grok-4 was rated at the senior high school level, whereas the remaining models were at the junior high school level, with GPT-4o receiving the lowest value within this range.

#### 3.1.3 Output stability assessment

Output stability was assessed by calculating the mean square error of repeated responses across 30 questions. Among all five models, GPT-4o exhibited the lowest overall variance, indicating the most consistent output. Deepseek-V2.5 exhibited the highest variance, reflecting the most unstable performance. As shown in Table 3, GPT-4o's variance was significantly lower than that of other models in terms of accuracy, completeness, safety, and practicality, confirming its significant stability advantage across these dimensions. GPT-5 exhibited the lowest variance in terms of readability.

**Table 3:** LLM's five-dimensional stability score: Wilcoxon rank-sum average.

*n* = 30	Accuracy	Completeness	Practicality	Readability	Safety
GPT-5	2.830	2.890	3.040	2.690^*^	3.110
GPT-4o	2.640^*^	2.790	3.190	2.930	2.610^*^
GPT-o3	3.090	3.100	2.780	3.060	3.170^*^
Deepseek-V2.5	3.330^*^	3.240	3.190	3.050	3.020
Grok-4	3.120	2.970	2.780	3.270^*^	3.100

(^*^) indicates significant differences between models within the same dimension, with statistical significance defined as a corrected *p*-value of less than 0.005. Models marked with (^*^) have the smallest or largest variance in that dimension.

### 3.2 Error diagnosis analysis

**Figure 4:**
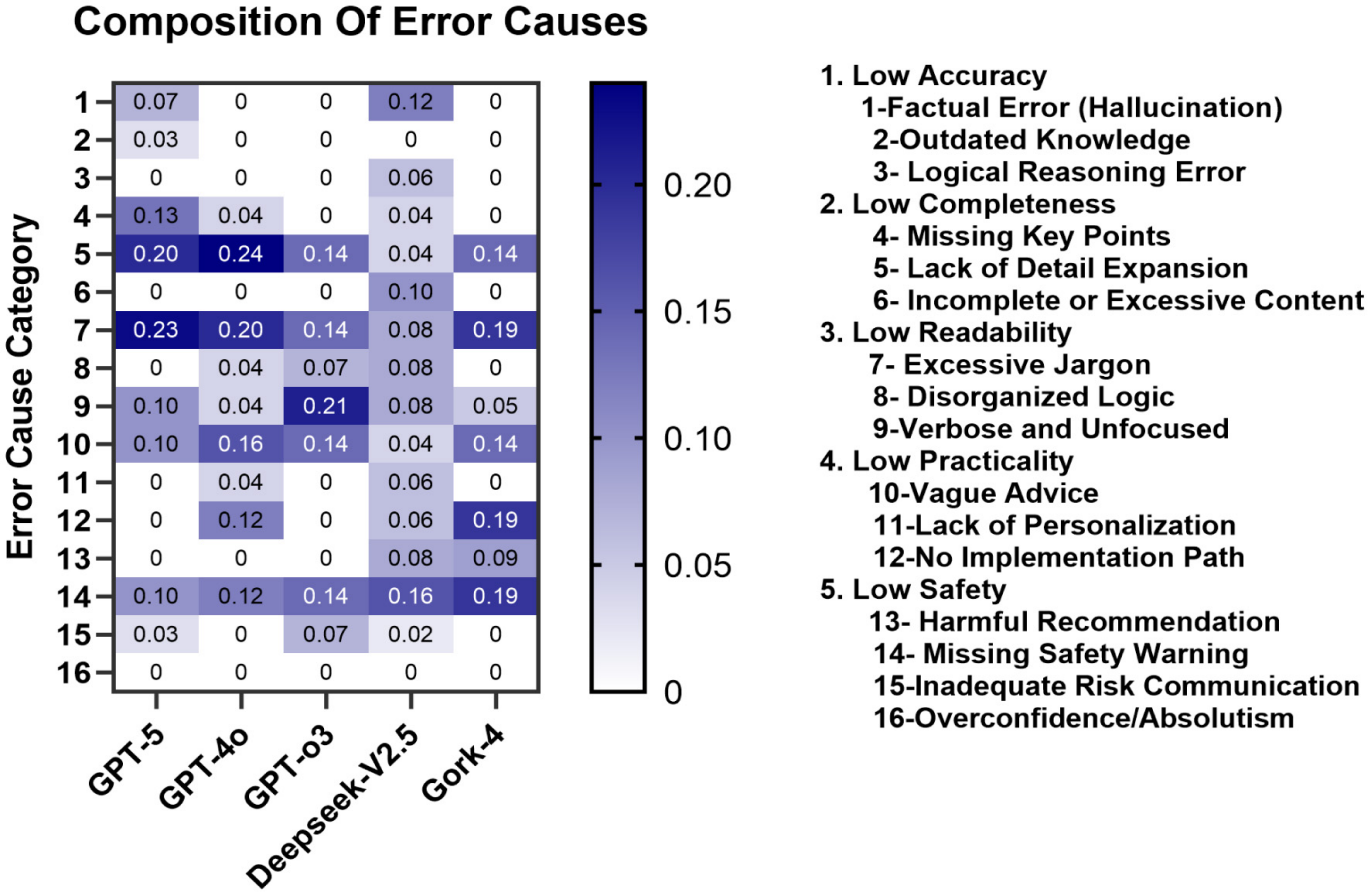
Error attribution results diagram. The Y-axis represents error category IDs, with the error classifications listed on the right. Darker cells indicate a higher proportion of that error category within a specific model's set of low-scoring responses. The total number of low-scoring responses (denominator) for each model is as follows: GPT-5, *n* = 30; GPT-4o, *n* = 25; GPT-o3, *n* = 14; Deepseek-V2.5, *n* = 51; Grok-4, *n* = 21. Due to differences in the denominators across models, caution should be exercised when making direct comparisons of error percentages between models. This figure is intended to illustrate the relative distribution of error types within each model, rather than to provide a statistical comparison of error rates across models.

An error attribution analysis was conducted on all low-scoring responses across the five models (Figure 4), revealing distinct patterns in error distribution between higher- and lower-performing models. For GPT-5 (*n* = 30 low-scoring responses), the most common error causes were: excessive jargon (Category 7: 23%, 7/30), lack of elaboration (Category 5: 20%, 6/30), and omission of key points (Category 4: 13%,4/30). GPT-4o (*n* = 25 low-scoring responses) exhibited similar error characteristics, with lack of elaboration (Category 5: 24%, 6/25), excessive jargon (Category 7: 20%, 5/25), and vague recommendations (Category 10: 16%, 4/25) being the most common issues. Safety warnings were missing in 12% (3/25) of the low-scoring responses. GPT-o3 (*n* = 14, low-scoring responses) exhibited a slightly different distribution. The most prominent error was verbosity and lack of focus (Category 9: 21%, 3/14), followed by excessive jargon (Category 7: 14%, 2/14), lack of detailed elaboration (Category 5: 14%, 2/14), vague recommendations (Category 10: 14%, 2/14), and lack of safety warnings (Category 14: 14%, 2/14). Harmful recommendations appeared in 7% (1/14) of the responses. The error composition of Grok-4 (*n* = 21 low-scoring responses) was markedly different. The most common errors were lack of an actionable implementation path (Category 12: 19%, 4/21), excessive jargon (Category 7: 19%, 4/21), and lack of safety warnings (Category 14: 19%, 4/21), followed by vague recommendations (Category 10: 14%, 3/21) and lack of detailed elaboration (Category 5: 14%, 3/21). Deepseek-V2.5 generated the largest number of low-scoring responses (*n* = 51) and exhibited distinctly different, more clinically significant error characteristics. The most prominent causes of errors included: lack of safety warnings (Category 14: 16%, 8/51), factual errors/hallucinations (Category 1: 12%, 6/51), and incomplete or excessive content (Category 6: 10%, 5/51).

### 3.3 Rapid engineering validation testing

**Figure 5:**
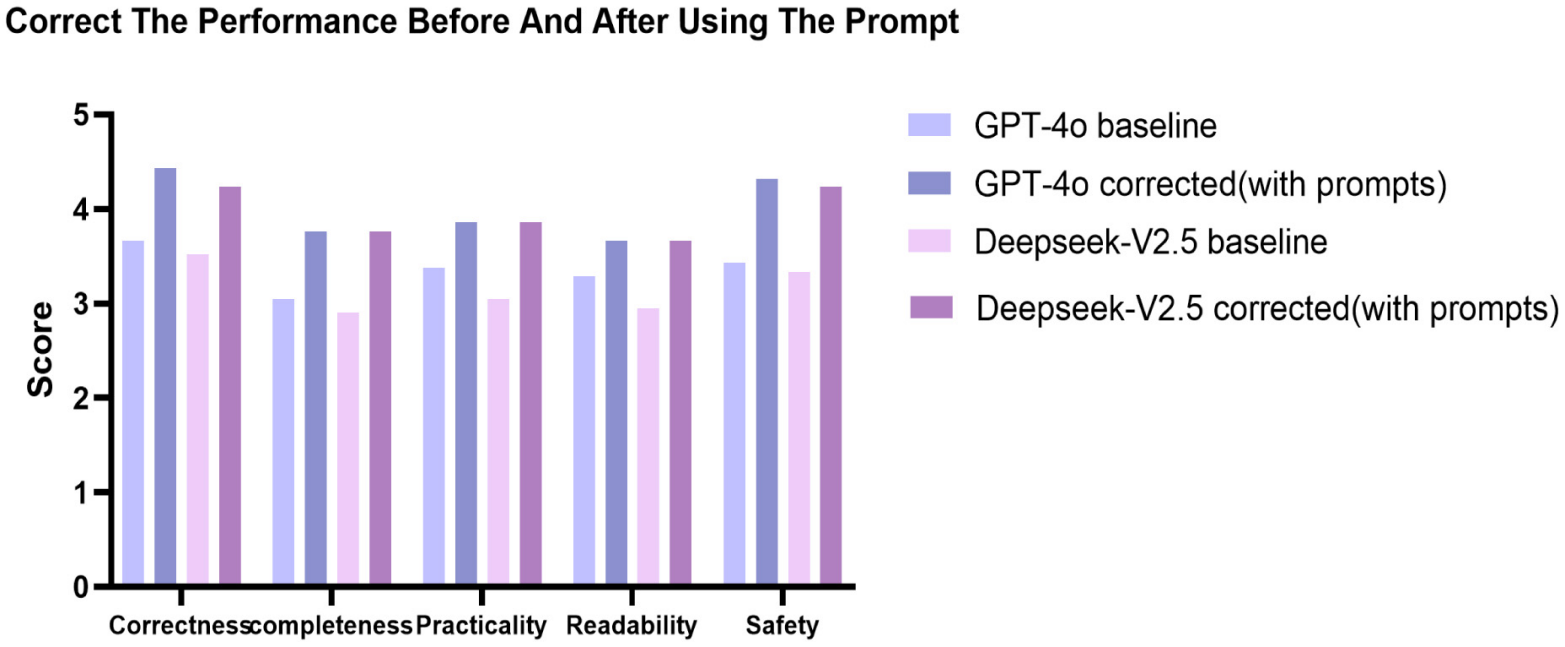
Comparison of output content before and after prompt intervention. The Y-axis represents the scores of various LLMs on each dimension, using a five-point Likert scale as the evaluation criterion.

Linear mixed model estimates of marginal means (Figure 5 and Table 4) showed that the corrected prompt significantly improved Deepseek-V2.5 across all five dimensions (*p* < 0.001), with the largest gains in Safety (*B* = 0.91, from 3.33 to 4.24) and Completeness (*B* = 0.86, from 2.91 to 3.76). GPT-4o also exhibited significant improvements in Accuracy (*B* = 0.76, *p* < 0.001), Completeness (*B* = 0.71, *p* < 0.001), Practicality (*B* = 0.48, *p* = 0.004), and Readability (*B* = 0.38, *p* = 0.023), whereas Safety showed only a modest, non-significant increase (*B* = 0.19, *p* = 0.227). A significant Model × Condition interaction was detected only for Safety (*p* = 0.002), underscoring the differential efficacy of the corrective prompt in this critical dimension. Combined with moderate-to-large Hedges' g effect sizes, these findings provide preliminary evidence of the value of targeted prompt engineering, particularly for lower-performing models.

**Table 4:** Comparison of five-dimensional scores of GPT-4o and Deepseek-V2.5 before and after prompt correction.

Dimension	Model	*B*	SE	df	*T*	*P*	Hedges' g [95% CI]
Accuracy	GPT-4o	0.762	0.158	80	4.835	< 0.001	0.62 [−0.69, 0.69]
	Deepseek-V2.5	0.714	0.158	80	4.533	< 0.001	0.80 [−0.31, 1.17]
Completeness	GPT-4o	0.714	0.124	80	5.752	< 0.001	0.52 [−0.33, 1.13]
	Deepseek-V2.5	0.857	0.124	80	6.903	< 0.001	0.65 [−0.01, 1.70]
Practicality	GPT-4o	0.476	0.163	80	2.924	0.004	0.52 [−0.23, 1.29]
	Deepseek-V2.5	0.810	0.163	80	4.970	< 0.001	0.76 [−0.14, 1.43]
Readability	GPT-4o	0.381	0.164	80	2.319	0.023	0.61 [−0.38, 1.06]
	Deepseek-V2.5	0.714	0.164	80	4.348	< 0.001	0.67 [−0.11, 1.48]
Safety	GPT-4o	0.190	0.156	80	1.217	0.227	0.42 [−0.13, 1.45]
	Deepseek-V2.5	0.905	0.156	80	5.782	< 0.001	0.57 [−0.08, 1.54]

B, unstandardized regression coefficient representing mean score improvement (corrected prompt minus baseline). *p*-values are derived from a linear mixed model with random intercepts for Question ID and Rater ID.

## 4 Discussion

This study systematically evaluated five mainstream large language models in the context of chronic low back pain rehabilitation guidance. Overall, although all models demonstrated strong foundational medical knowledge with overall multiple-choice question accuracy exceeding 90%, substantial variability emerged in guideline adherence, output completeness, practicality, safety, and readability when applied to realistic clinical scenarios. These discrepancies highlight a critical gap between knowledge possession and clinically reliable knowledge application, with direct implications for safe deployment.

During the evaluation phase, models with comparable foundational knowledge differed markedly in their capacity to translate knowledge into reliable clinical guidance. GPT-4o demonstrated the most consistent composite performance, achieving high scores across accuracy, practicality, and safety, suggesting relatively mature knowledge integration and contextualized output capabilities. In contrast, Deepseek-V2.5 showed broader theoretical knowledge coverage but was weaker in clinical reasoning and safety control, with higher rates of safety-critical failures, including factual hallucinations, unsupported recommendations, and omission of safety warnings. These findings suggest that the breadth of knowledge coverage does not equate to reliable clinical decision support. In specific clinical scenarios, the ability to recognize risk and provide qualified recommendations may be more important than answering questions correctly (Bean et al., 2025). Consistent with prior studies, LLMs may approach expert-level performance on standardized assessments, yet remain vulnerable to omissions and incomplete reasoning in open-ended and complex clinical settings (Li et al., 2025).

Error attribution analysis revealed both universal tendencies and model-specific differences. A near-universal pattern was the overuse of technical terminology, likely reflecting training biases toward guideline texts and specialist corpora during training. More clinically consequential was the divergence in error direction across models. Consistent with previous findings (Asgari et al., 2025), errors generated by high-performance models (GPT-5 and GPT-4o) are generally considered low-risk and clinically tolerable in specific contexts, primarily manifesting as missing details, misuse of terminology, and ambiguous recommendations. In contrast, among the low-scoring responses generated by DeepSeek-V2.5 (*n* = 51), the proportion of high-risk error categories was higher; Grok-4 (*n* = 21) also exhibited a similar pattern (albeit to a lesser extent), including fabrication of facts (Category 1), recommendations exceeding the scope of guidelines (Category 2), and omission of critical safety warnings (Category 14). This descriptive pattern stems from a validated 15-category error taxonomy (Appendix 1) and is based on reported denominator data, suggesting that underperforming models may possess unique risk characteristics warranting more detailed clinical review. However, it should be noted that these findings are based on error distributions observed in specific benchmark tests; in the absence of prospective validation, they should not be interpreted as conclusive evidence of inherent differences in model safety. These differentiated risk characteristics should inform clinicians' decision-making when selecting AI-assisted tools. For tasks requiring the highest safety standards, such as patient education or red flag symptom screening, models with more stable and low-risk outputs (e.g., GPT-4o) should be prioritized. In cost-sensitive, or high-throughput contexts, lower-cost models such as Deepseek-V2.5 may be considered, but only with stringent human oversight, targeted safety prompting, and other risk-mitigating strategies (Jarchow et al., 2025). Crucially, even following prompt engineering, overall safety improvements did not eliminate residual risks; therefore, ongoing monitoring and additional safeguards remain necessary for LLMs' use in high-risk clinical environments.

This study also provides preliminary evidence supporting the utility of targeted prompt engineering. The intervention strategy was straightforward, focusing on requiring explicit verification of safety considerations, evidence-based justification, and actionable specificity. Results from this exploratory analysis suggest that such intervention can meaningfully improve response quality, particularly for lower-performing models.

These findings suggest that the effectiveness of prompt engineering is substantially mediated by a model's baseline capabilities. Lower-cost or open-source models may benefit more substantially from structured prompting to enhanced clinical usability, although this requires validation in larger studies. For higher-performing models, prompt engineering may be more applicable to optimizing response quality and reducing edge-case risks (Vilakati, 2025). Overall, structured prompting combined with systematic fact-checking represents the most pragmatic and scalable near-term safety reinforcement strategy, particularly in resource-limited clinical settings.

Several limitations in this study should be considered. First, although the three rounds of response generation were conducted in independent chat sessions, the 30 questions within each round were presented in a fixed sequential order. This may introduce a limited contextual priming effect across questions within the same round. Future studies could mitigate this by randomizing question order across rounds. Second, only the overall intraclass correlation coefficient (ICC, 0.62) was reported; dimension-specific reliability coefficients could provide a more nuanced reflection of rater consistency across different evaluation domains. In this study, although the ICC was moderate, statistically significant inter-model differences were still observed, suggesting that true performance disparities may be underestimated rather than inflated. Systematic biases have been controlled by using a linear mixed-effects model. Therefore, the core conclusions are unlikely to be fundamentally overturned due to disagreements among evaluators. Nevertheless, caution is warranted when interpreting absolute scores, particularly for models with high output variability, such as Deepseek-V2.5. Third, the prompt engineering tests used only a small set of questions drawn from the same error pool used for error attribution, which may lead to circular reasoning and an overestimation of the intervention's effectiveness; future research should employ an independent validation set. Fourth, the prompt strategies were relatively simple; more complex or iterative methods remain to be explored. Fifth, prompt engineering was tested only on the highest- and lowest-performing models, while intermediate models were not evaluated. Sixth, all experiments relied on specific model versions at specific points in time; rapid LLM iteration means that the temporal generalisability of certain findings may be limited. Seventh, all prompts and model outputs in this study were generated and evaluated in Chinese. Although the evaluated large language models are capable of multilingual generation, their training data distributions and fine-tuning strategies differ across languages. Some models (e.g., GPT 4o, GPT 5) are trained predominantly on English corpora, while others (e.g., Deepseek V2.5) are optimized for Chinese. The observed performance differences may therefore reflect not only inherent reasoning capabilities but also language-specific proficiency. The findings should be interpreted within the context of Chinese clinical communication, and cross-linguistic validation is warranted before generalizing these results to other language settings. Finally, all evaluations were conducted in simulated, static scenarios, which do not capture the complexity of real-world clinical environments, including patient heterogeneity, dynamic interaction, and multimodal data integration.

## 5 Conclusion

This study, through an assess-attribute-correct three-phase paradigm, systematically characterized the capability boundaries, risk profiles, and intervention potential of current LLMs in chronic LBP rehabilitation guidance. The findings provide clinicians with a practical framework centered on model-specific risk awareness and targeted safety reinforcement. Until LLMs achieve sufficiently high reliability for autonomous clinical use, careful model selection, explicit prompt constraints, and sustained human oversight remain indispensable for ensuring patient safety.

## Data Availability

The original contributions presented in the study are included in the article/Supplementary material, further inquiries can be directed to the corresponding author/s.
