# Supplementary material for: Deficiencies in clinical reasoning of LLMs in low back pain management and remediation via prompt engineering: from performance evaluation to error diagnosis
Source: Front Artif Intell. 2026 May 25;9:1811701. doi: 10.3389/frai.2026.1811701 (PMC13243384; doi:10.3389/frai.2026.1811701)
Supplement: Supplementary file 1 [file Data_Sheet_1.zip › 补充材料/Appendix 1/Appendix 1.docx]

**Appendix 1:**

| **Dimension** | **Subcategory** | **Operational Definition** | **Judging Criteria** | **Example** | **Risk Level** |
| --- | --- | --- | --- | --- | --- |
| **Low Accuracy** | Factual Error (Hallucination) | Generated content contradicts established medical facts or clinical guidelines | Explicitly inconsistent with NICE/ACP/WHO guidelines; verifiable factual error | “Recommend lumbar rotation exercises (90°) for acute disc herniation.” | High |
|  | Outdated Knowledge | Information has been superseded by recent guidelines or high‑quality evidence | References are no longer recommended in guidelines from the last 5 years | “Use X‑ray for routine screening of lumbar disc herniation.” (Not recommended in current guidelines) | Moderate |
|  | Logical Reasoning Error | Broken causal chain, reversed logic, or violation of clinical reasoning | No causal link between premise and conclusion; violates basic decision logic | “Patient with low back pain and fever; continue rehabilitation and observe.” (Misses red‑flag screening) | High |
| **Low Completeness** | Missing Key Points | Absence of core information explicitly required by guidelines or standard answers | Missing ≥1 item from the guideline “checklist of key information.” | No mention of cauda equina red‑flag symptoms (e.g., sphincter disturbance, saddle anesthesia) | High |
|  | Lack of Detail Expansion | Key point mentioned, but necessary details (rationale, timing, precautions) omitted | Only the conclusion or action name is given, without any explanation | “Perform core stability training.” (No specific exercises, frequency, or intensity) | Moderate |
|  | Incomplete or Excessive Content | Too little relevant information or inclusion of irrelevant content | <2 valid points directly related to the question, or a large amount of off‑topic background | “Consult a professional physician.” (Single sentence, no actionable advice) | Low |
| **Low Readability** | Excessive Jargon | Overuse of technical terms without explanation | ≥3 technical terms in a single sentence without definition or simplification | “Activate the transversus abdominis, multifidus, and quadratus lumborum in a coordinated contraction pattern.” | Low |
|  | Disorganized Logic | Lack of clear logical flow between sentences or paragraphs | Confusion between coordination and subordination; no transition words; unclear cause‑and effect | “Avoid prolonged sitting. Then apply heat. If pain is severe, surgery is needed.” | Moderate |
|  | Verbose and Unfocused | Wordy, repetitive, core message buried | >200 words without directly answering the core question; repeated statements | Uses 300 words to describe the epidemiology of low back pain, but only ends with “get some rest.” | Low |
| **Low Practicality** | Vague Advice | No specific action instruction; the patient cannot follow | Uses vague verbs (e.g., “strengthen,” “pay attention to,” “properly”) without concrete actions | “Strengthen your back muscles.” | Moderate |
|  | Lack of Personalization | Ignores patient‑specific factors (age, comorbidities, exercise capacity) | Uniform advice without indication of adjustment for individual conditions | “All low back pain patients should perform planks.” | Low |
|  | No Implementation Path | No method, frequency, intensity, duration, or precautions provided | Only states the goal without any plan to achieve it | “Need to improve core stability.” (No description of how to achieve it) | Moderate |
| **Low Safety** | Harmful Recommendation | Explicitly recommends actions that could cause harm | Recommends contraindicated maneuvers or advises delaying medical care | “Perform toe‑touch stretching during acute low back pain.” | High |
|  | Missing Safety Warning | No provision of necessary risk warnings or indications for seeking care | Does not mention cauda equina red‑flag symptoms, or does not advise when to seek medical help | Provides only exercise instructions without any warning, such as “seek immediate care if lower limb weakness occurs.” | High |
|  | Inadequate Risk Communication | Mentions risks but in a vague or incomplete manner | “Seek care if you feel unwell” (no specification of which symptoms or when) | “Be careful.” | Moderate |
|  | Overconfidence/Absolutism | Uses absolute language, ignoring uncertainty or individual variation | Uses words like “all,” “absolutely,” “must”; or claims 100% efficacy | “This method works for everyone.” | Moderate |
