# Supplementary material for: Deficiencies in clinical reasoning of LLMs in low back pain management and remediation via prompt engineering: from performance evaluation to error diagnosis
Source: Front Artif Intell. 2026 May 25;9:1811701. doi: 10.3389/frai.2026.1811701 (PMC13243384; doi:10.3389/frai.2026.1811701)
Supplement: Supplementary file 1 [file Data_Sheet_1.zip › 补充材料/Supplementary Materials 1/Model Version.docx]

| Model | Specific Version | Official Release | Availability (Oct 2025) | Interface Settings | Access Interface |
| --- | --- | --- | --- | --- | --- |
| GPT-5 | Official Release: August 2025(version undisclosed) | Aug 7, 2025  [OpenAI正式发布GPT-5模型：面向所有用户开放 更像人类专家_热点播报_太平洋科技资讯中心](https://news.pconline.com.cn/1960/19608272.html) | Available | Official ChatGPT web interface; default standard mode; web browsing and enhanced reasoning disabled | chat.openai.com |
| GPT-4o | GPT-4o-latest identifier | May 2024 (relisted Aug 9, 2025)  [受 GPT-5 差评影响，OpenAI 重新上架 GPT-4o模型-品玩](https://www.pingwest.com/w/306755) | Available (enable 'show legacy models') | Official ChatGPT web interface; default standard mode; web browsing and enhanced reasoning disabled | chat.openai.com |
| GPT-o3 | GPT-o3-2025-04-16 | Apr 17, 2025  [OpenAI更新大模型 能“用图思考”并调用工具_财新网_财新网](https://www.caixin.com/2025-04-17/102310422.html?sourceEntityId=102342497) | Available | Official ChatGPT web interface; default standard mode; web browsing and enhanced reasoning disabled | chat.openai.com |
| Deepseek-V2.5 | Deepseek-V2.5-0905 (open-source) | Feb 4, 2025  [DeepSeek系列新模型正式上线昇腾社区](https://www.stcn.com/article/detail/1515062.html) | Available | DeepSeek official interface; web browsing disabled; default mode; temperature=1.0, top_p=1.0 | chat.deepseek.com |
| Grok-4 | Grok-4-20250709 | Jul 10, 2025  [馬斯克宣布Grok 4免費開放_香港商报数字报](https://ebook.hkcd.com.hk/PC/content/202508/12/content_3370764.html) | Available | xAI official interface; default standard mode; reasoning enhancement disabled | grok.x.ai |
