# Supplementary material for: Deficiencies in clinical reasoning of LLMs in low back pain management and remediation via prompt engineering: from performance evaluation to error diagnosis
Source: Front Artif Intell. 2026 May 25;9:1811701. doi: 10.3389/frai.2026.1811701 (PMC13243384; doi:10.3389/frai.2026.1811701)
Supplement: Supplementary file 1 [file Data_Sheet_1.zip › 补充材料/Supplementary materials 2/Table S1.docx]

**A. Definition of Low Back Pain Injuries (G:6/5 C3:5/5 C4:5/5 D:6/5)**

1. Which of the following descriptions of lumbar spine anatomy is correct?

A. The lumbar spine has 6 vertebral bodies

B. Intervertebral discs connect all lumbar vertebral bodies

C. The intervertebral disc has no nerves, only blood vessels

D. The intervertebral disc does not deform under pressure

E. The intervertebral disc decreases in volume under pressure

Grok-4: B GPT-o3: E GPT-4o: E DeepSeek-V2.5: B GPT-5: E

21. Which of the following descriptions of acute lumbar sprain is correct?

A. Clinically more common in females

B. Acute lumbar sprain has many causes and patients often cannot describe the causative factor

C. When lumbar muscles, fascia, and ligaments sustain overload during labor or exercise, causing varying degrees of fiber tears and a series of clinical symptoms, this is called acute lumbar sprain

D. X-ray may reveal straightening of the spine or protective lateral deviation

E. Positive neurological signs in both lower limbs

Grok-4: C GPT-o3: C GPT-4o: C DeepSeek-V2.5: C GPT-5: B

54. Regarding lumbar spondylolysis (isthmic defect), which description is incorrect? ( )

A. Refers to fracture of the isthmus between the superior and inferior articular processes of the vertebral arch

B. Lower back pain or lower limb soreness often appears after trauma, fatigue, or exercise

C. Symptoms worsen when standing, walking, or bending under load, and ease with rest

D. Often combined with posterior slippage of the superior vertebral body

E. May present with nerve irritation symptoms

Grok-4: D GPT-o3: D GPT-4o: D DeepSeek-V2.5: D GPT-5: D

65. Which of the following descriptions of lumbar myofascitis is incorrect? ( )

A. There are tender points

B. Cold and humidity are triggering factors

C. Myofascial edema occurs

D. Disc herniation

E. Myofascial fibrosis

Grok-4: D GPT-o3: D GPT-4o: D DeepSeek-V2.5: D GPT-5: D

66. Which of the following descriptions of lumbar myofascitis is incorrect? ( )

A. Often has a history of cold, humidity, or chronic injury

B. Symptoms are mainly pain

C. There are clear tender points

D. Cord-like nodules can be felt

E. Pain is relieved when stretching back muscles

Grok-4: E GPT-o3: E GPT-4o: E DeepSeek-V2.5: E GPT-5: E

**B. Etiology of Low Back Pain (G:13/14 C3:13/14 C4:12/14 D:14/14)**

2. The most fundamental cause of lumbar disc herniation is:

A. Trauma and overuse

B. Obesity

C. Cold

D. Degenerative changes of the intervertebral disc

E. Diabetes

Grok-4: D GPT-o3: D GPT-4o: D DeepSeek-V2.5: D GPT-5: D

11. Which of the following is NOT a cause of sacroiliac joint dysfunction?

A. Female menstrual period

B. Hormonal changes in pregnant/postpartum women

C. Pelvic ligament laxity

D. Acute sprain

E. Chronic sprain

Grok-4: A GPT-o3: A GPT-4o: A DeepSeek-V2.5: A GPT-5: A

19. Which of the following causes of low back pain is attributable to visceral organ disease?

A. Calculi (stones)

B. Spinal tuberculosis

C. Acute myelitis

D. Spinal tumor

E. Nerve root inflammation

Grok-4: A GPT-o3: A GPT-4o: A DeepSeek-V2.5: A GPT-5: A

27. The posture with the highest lumbar intervertebral disc pressure is:

A. Standing

B. Sitting

C. Semi-recumbent

D. Supine

E. Hyperextension

Grok-4: B GPT-o3: B GPT-4o: B DeepSeek-V2.5: B GPT-5: B

35. Which of the following clinical manifestations is NOT associated with L5-S1 disc herniation?

A. Low back and leg pain

B. Positive straight leg raise test and reinforcement test

C. Abnormal knee reflex

D. Reduced sensation at the lateral malleolus and dorsolateral foot

E. Abnormal ankle reflex

Grok-4: C GPT-o3: C GPT-4o: C DeepSeek-V2.5: C GPT-5: C

36. When lumbar disc herniation is located lateral to the nerve root, the functional lumbar scoliosis should be:

A. Lumbar convexity toward the healthy side

B. Lumbar convexity toward the affected side

C. No change in lumbar lateral deviation

D. Direction of lumbar deviation is indeterminate

E. Increased lumbar lordosis

Grok-4: A GPT-o3: B GPT-4o: A DeepSeek-V2.5: B GPT-5: B

42. Which of the following is NOT a predisposing factor for lumbar disc herniation? ( )

A. Degenerative changes

B. Sprain

C. Heavy lifting

D. Cold

E. Jogging

Grok-4: E GPT-o3: E GPT-4o: E DeepSeek-V2.5: E GPT-5: E

43. Lumbar degenerative changes first appear in: ( )

A. Facet joints

B. Intervertebral disc

C. Ligamentum flavum

D. Posterior longitudinal ligament

E. Anterior longitudinal ligament

Grok-4: B GPT-o3: B GPT-4o: B DeepSeek-V2.5: B GPT-5: B

64. Which of the following statements about the etiology and pathology of lumbar myofascitis is incorrect? ( )

A. Chronic overuse can cause fibrotic changes in muscles and fascia

B. Cold can cause vasoconstriction and edema in lumbar muscles, leading to local fibrinous exudate

C. Humidity can slow blood flow in the subcutaneous and fascial areas, ultimately causing fibromyositis

D. Viral infection is one of the predisposing factors

E. The tender points of lumbar myofascitis are mostly located at the muscle insertion sites

Grok-4: E GPT-o3: D GPT-4o: D DeepSeek-V2.5: E GPT-5: D

84. Which of the following diseases generally does NOT cause low back pain?

A. Lumbar disc herniation

B. Cholecystitis

C. Kidney stones

D. Lumbar muscle strain

Grok-4: B GPT-o3: B GPT-4o: B DeepSeek-V2.5: B GPT-5: B

85. The most common cause of low back pain with radiating lower limb pain is:

A. Lumbar osteophyte formation

B. Lumbar spinal stenosis

C. Lumbar disc herniation

D. Piriformis syndrome

Grok-4: C GPT-o3: C GPT-4o: C DeepSeek-V2.5: C GPT-5: C

92. What are the common causes of low back pain?

A. Muscle strain

B. Disc herniation

C. Kidney stones

D. All of the above

Grok-4: D GPT-o3: D GPT-4o: D DeepSeek-V2.5: D GPT-5: D

102. Which of the following diseases is most likely to cause low back pain? ( )

A. Periarthritis of the shoulder

B. Lumbar disc herniation

C. Tenosynovitis

D. Cervical spondylosis

Grok-4: B GPT-o3: B GPT-4o: B DeepSeek-V2.5: B GPT-5: B

104. Which of the following is NOT a reason for low back pain caused by osteoporosis? ( )

A. Vertebral body compression deformity

B. Fracture stimulating surrounding tissues

C. Muscle spasm

D. Disc herniation

Grok-4: D GPT-o3: D GPT-4o: D DeepSeek-V2.5: D GPT-5: D

**C. Clinical Manifestations and Signs of Low Back Pain (G:21/21 C3:20/21 C4:20/21 D:21/21)**

3. Which of the following is NOT a characteristic of lumbar disc herniation?

A. More common in young and middle-aged people

B. Cold and humidity are predisposing factors

C. More common in males than females

D. Often has a history of heavy lifting or sprain

E. Often associated with lumbar vertebral destruction

Grok-4: E GPT-o3: E GPT-4o: E DeepSeek-V2.5: E GPT-5: E

4. Which of the following diseases cannot cause sciatica symptoms?

A. Lumbar disc herniation

B. Lumbar back fasciitis

C. Lumbar spinal stenosis

D. Piriformis syndrome

E. Sciatic neuritis

Grok-4: B GPT-o3: B GPT-4o: B DeepSeek-V2.5: B GPT-5: B

10. The time boundary between acute and chronic low back pain is:

A. 1 month

B. 3 months

C. 6 months

D. 9 months

E. 1 year

Grok-4: B GPT-o3: B GPT-4o: B DeepSeek-V2.5: B GPT-5: B

22. In acute lumbar sprain, the most commonly affected lumbar muscle is:

A. Rectus abdominis

B. Erector spinae

C. Internal oblique abdominis

D. Psoas major

E. Iliopsoas

Grok-4: B GPT-o3: B GPT-4o: B DeepSeek-V2.5: B GPT-5: B

24. Tenderness can appear in the gluteal region, sciatic notch, popliteal fossa midline, and posterior leg in:

A. Sacroiliac joint dysfunction

B. Lumbar disc herniation

C. Third lumbar transverse process syndrome

D. Acute lumbar sprain

E. Spinal osteoporosis

Grok-4: B GPT-o3: B GPT-4o: B DeepSeek-V2.5: B GPT-5: C

37. A patient with lumbar disc herniation shows reduced toe dorsiflexion strength and decreased sensation in the lateral leg and toe roots. The lesion is localized to:

A. L2-3

B. L3-4

C. L4-5

D. L5-S1

E. S1-2

Grok-4: C GPT-o3: C GPT-4o: D DeepSeek-V2.5: C GPT-5: C

40. The typical symptom of lumbar disc herniation is:

A. Low back pain

B. Lower limb weakness

C. Low back pain with sciatic nerve pain

D. Sciatic nerve pain

E. Restricted lumbar movement

Grok-4: C GPT-o3: C GPT-4o: C DeepSeek-V2.5: C GPT-5: C

41. The clinical manifestation of lumbar muscle strain is:

A. Positive straight leg raise test, positive reinforcement test

B. Reduced sensation in the posterior thigh, lateral leg, and dorsolateral foot

C. Pain relieved at rest, pain relieved by percussion of the lumbar area

D. Abnormal knee and ankle reflexes

Grok-4: C GPT-o3: C GPT-4o: C DeepSeek-V2.5: C GPT-5: C

44. The most common presentation of lumbar disc herniation is: ( )

A. Upper limb numbness

B. Four-limb weakness

C. Pain

D. Foot drop

E. Urinary incontinence

Grok-4: C GPT-o3: C GPT-4o: C DeepSeek-V2.5: C GPT-5: C

46. For sciatica caused by lumbar disc herniation, the typical radiation area to the lower limb is: ( )

A. The entire leg below the buttock

B. Posterior thigh and medial leg

C. Anterior thigh, medial leg, and medial dorsum of foot

D. Posterior thigh, lateral leg, and lateral dorsum of foot

E. Posterior leg and sole of foot

Grok-4: D GPT-o3: D GPT-4o: D DeepSeek-V2.5: D GPT-5: D

56. The clinical manifestations of degenerative lumbar spondylolisthesis do NOT include: ( )

A. Chronic low back pain history

B. Lumbar soreness and distension

C. Reduced superficial sensation in the lower limbs

D. Hemiplegia

E. Weakened Achilles tendon reflex

Grok-4: D GPT-o3: D GPT-4o: D DeepSeek-V2.5: D GPT-5: D

57. After lumbar spondylolysis, the most likely complication is: ( )

A. Lumbar spondylolisthesis

B. Cauda equina nerve injury

C. Sciatic nerve rupture

D. Bladder and bowel incontinence

E. Bilateral lower limb paralysis

Grok-4: A GPT-o3: A GPT-4o: A DeepSeek-V2.5: A GPT-5: A

59. The clinical features of lumbar facet joint synovial entrapment do NOT include: ( )

A. Coughing aggravates lumbar pain

B. Positive straight leg raise test and reinforcement test

C. Lumbar scoliosis and paraspinal muscle spasm

D. Local facet joint capsule block can relieve pain

E. X-ray AP and lateral views show: straightening of lumbar curvature or lumbar scoliosis

Grok-4: B GPT-o3: B GPT-4o: B DeepSeek-V2.5: B GPT-5: B

67. The pain characteristics of lumbar myofascitis are: ( )

A. Pain in the morning and during the day, relieved in the evening

B. Morning pain, lighter during the day, worsened again in the evening

C. Light in the morning, pain during the day, light in the evening

D. Intermittent onset, no regularity

E. Persistent dull pain

Grok-4: B GPT-o3: B GPT-4o: B DeepSeek-V2.5: B GPT-5: E

82. Low back pain that is relieved by activity but worsened after fatigue is most likely:

A. Lumbar tuberculosis

B. Lumbar fracture

C. Lumbar spondylolisthesis

D. Lumbar muscle strain

Answer: D

Grok-4: D GPT-o3: D GPT-4o: D DeepSeek-V2.5: D GPT-5: D

83. The typical symptom of lumbar spinal stenosis is:

A. Low back pain with lower limb numbness

B. Intermittent claudication

C. Lower limb weakness

D. Bladder and bowel incontinence

Grok-4: B GPT-o3: B GPT-4o: B DeepSeek-V2.5: B GPT-5: B

86. The main sign of acute lumbar sprain is:

A. Positive straight leg raise test

B. Reduced lower limb sensation

C. Marked lumbar tenderness

D. Weakened tendon reflexes

Grok-4: C GPT-o3: C GPT-4o: C DeepSeek-V2.5: C GPT-5: C

87. Lumbar disc herniation most commonly occurs at:

A. L1-L2

B. L2-L3

C. L3-L4

D. L4-L5

Grok-4: D GPT-o3: D GPT-4o: D DeepSeek-V2.5: D GPT-5: D

88. Which of the following descriptions of lumbar muscle strain is incorrect?

A. Long-term repeated chronic lumbar injury

B. Pain can be relieved with rest

C. Lumbar movement is significantly restricted

D. Pain is often in the lumbosacral area

Grok-4: C GPT-o3: C GPT-4o: C DeepSeek-V2.5: C GPT-5: C

105. A patient with low back pain and radiating lower limb pain most likely has: ( )

A. Lumbar muscle strain

B. Lumbar spinal stenosis

C. Third lumbar transverse process syndrome

D. Lumbar tuberculosis

Grok-4: B GPT-o3: B GPT-4o: B DeepSeek-V2.5: B GPT-5: B

109. Saddle area sensory disturbance in a patient with lumbar disc herniation suggests: ( )

A. Mild nerve root compression

B. Cauda equina nerve compression

C. Spinal cord compression

D. Dural sac compression

Grok-4: B GPT-o3: B GPT-4o: B DeepSeek-V2.5: B GPT-5: B

55. Degenerative spondylolisthesis must be differentiated from isthmic spondylolisthesis. The characteristics of the latter do NOT include: ( )

A. Slippage degree is often more severe

B. Often combined with disc herniation

C. Unilateral or bilateral fracture of the vertebral arch isthmus

D. Significant widening of the anteroposterior diameter of the spinal canal

Grok-4: B GPT-o3: D GPT-4o: B DeepSeek-V2.5: B GPT-5: B

**D. Clinical Examination of Low Back Pain (G:10/12 C3:11/12 C4:12/12 D:12/12)**

8. The most commonly used physical examination for lumbar disc herniation is:

A. Pelvic separation test

B. Figure-4 test

C. Tinel's sign

D. Straight leg raise test and reinforcement test

E. Drawer test

Grok-4: D GPT-o3: D GPT-4o: D DeepSeek-V2.5: D GPT-5: D

23. In the clinical examination of sacroiliac joint dysfunction, the positive test is:

A. Iliac separation test and Gaenslen's test

B. Straight leg raise test

C. Reinforcement test

D. Thomas test

E. Drawer test

Grok-4: A GPT-o3: A GPT-4o: A DeepSeek-V2.5: A GPT-5: A

25. In assessing lumbar range of motion in patients with low back pain, the most important range of motion is:

A. Flexion

B. Extension

C. Left lateral flexion

D. Right lateral flexion

E. Rotation

Grok-4: A GPT-o3: A GPT-4o: A DeepSeek-V2.5: A GPT-5: A

26. When measuring lumbar flexion/extension and lateral flexion range of motion with a goniometer, the axis is placed at:

A. T12 spinous process

B. L1 spinous process

C. L2 spinous process

D. L5 spinous process

E. Posterior sacral promontory

Grok-4: D GPT-o3: D GPT-4o: D DeepSeek-V2.5: D GPT-5: C

31. Which of the following is an incorrect statement about the clinical features of lumbar facet joint synovial entrapment?

A. Coughing and vibration both aggravate lumbar pain

B. Positive straight leg raise test and reinforcement test

C. Patients may have lumbar scoliosis and paraspinal muscle spasm

D. Local facet joint capsule block can relieve pain

E. X-ray AP and lateral views show: straightening of lumbar physiological curvature or lumbar scoliosis

Grok-4: B GPT-o3: B GPT-4o: B DeepSeek-V2.5: B GPT-5: B

32. Regarding the clinical manifestations of pelvic outlet narrowing syndrome and piriformis syndrome, which is incorrect?

A. Sensory disturbance in the buttock, inner and outer thigh, inner leg, dorsum of foot, and sole

B. Tenderness at the sciatic nerve outlet with radiating pain along the sciatic nerve route

C. Varying degrees of muscle atrophy in the buttock compared to the healthy side

D. Internal rotation test of the lower limb can induce sciatica; straight leg raise test is generally positive

E. X-ray generally shows no positive findings

Grok-4: D GPT-o3: A GPT-4o: A DeepSeek-V2.5: A GPT-5: A

33. Regarding the clinical manifestations of lumbar myofascitis, which is incorrect?

A. Lumbosacral soreness, dull pain, lighter at rest, heavier after fatigue

B. Cannot sustain bent-over work; when severe, may extend to buttocks and posterior thigh; lower back drooping after prolonged standing, no lower limb radicular pain

C. Tender points are often not localized

D. Positive straight leg raise test

E. X-ray plain film is mostly normal

Grok-4: D GPT-o3: D GPT-4o: D DeepSeek-V2.5: D GPT-5: D

39. A positive straight leg raise test indicates:

A. Schmorl's node

B. Cauda equina injury

C. Chronic lumbar muscle strain

D. L3-4 disc bulging

E. L4-5 or L5-S1 disc herniation

Grok-4: E GPT-o3: E GPT-4o: E DeepSeek-V2.5: E GPT-5: E

47. To differentiate dry (trunk) type from root type sciatica, the first test to consider is: ( )

A. Figure-4 test

B. Piriformis tension test

C. Bedside test

D. Straight leg raise reinforcement test

E. Straight leg raise test

Grok-4: E GPT-o3: E GPT-4o: D DeepSeek-V2.5: E GPT-5: E

48. The most valuable physical examination for diagnosing lumbar disc herniation is: ( )

A. Figure-4 test

B. Gait examination

C. Pelvic compression test

D. Straight leg raise test

E. Lumbar range of motion examination

Grok-4: D GPT-o3: D GPT-4o: D DeepSeek-V2.5: D GPT-5: D

49. In lumbar disc herniation, a positive femoral nerve stretch test is most likely at which level? ( )

A. L2-3

B. L4-5

C. L1-2

D. L3-4

E. L5-S1

Grok-4: D GPT-o3: D GPT-4o: D DeepSeek-V2.5: D GPT-5: D

68. The positive physical sign of lumbar myofascitis is: ( )

A. Straight leg raise test

B. Mill's test

C. Back muscle stretch test

D. Lateral stress test

E. Thomas sign

Grok-4: C GPT-o3: C GPT-4o: C DeepSeek-V2.5: C GPT-5: C

**E. Imaging Examination for Low Back Pain (G:16/16 C3:14/16 C4:14/16 D:15/16)**

5. The most direct imaging examination for diagnosing disc herniation is:

A. X-ray examination

B. CT examination

C. Bone scan

D. Ultrasound examination

E. Infrared thermal imaging

Grok-4: B GPT-o3: B GPT-4o: B DeepSeek-V2.5: B GPT-5: B

12. For diagnosing lumbar spinal stenosis, the CT measurement of the sagittal diameter of the spinal canal is:

A. 6mm

B. 7mm

C. 8mm

D. 9mm

E. 10mm

Grok-4: E GPT-o3: A GPT-4o: E DeepSeek-V2.5: E GPT-5: B

17. The spinal index for diagnosing spinal osteoporosis is:

A. 60

B. 70

C. 80

D. 90

E. 50

Grok-4: C GPT-o3: C GPT-4o: B DeepSeek-V2.5: C GPT-5: C

38. Male, 60 years old. Intermittent claudication for 6 years. Pain is relieved when squatting, can ride a bicycle normally. Straight leg raise test is negative. X-ray shows significant lumbar osteophyte formation. The most likely diagnosis is:

A. Lumbar spinal stenosis

B. Lumbar disc herniation

C. Chronic lumbar muscle strain

D. Interspinous ligament injury

E. Lumbar tuberculosis

Grok-4: A GPT-o3: A GPT-4o: A DeepSeek-V2.5: A GPT-5: A

50. X-ray findings of lumbar disc herniation do NOT include: ( )

A. Lumbar scoliosis

B. Decreased or absent lumbar lordosis

C. Shallow arc-shaped impression on the posterior-inferior margin of the vertebral body

D. Herniated mass compressing the dural sac

E. Intervertebral space with equal or narrower anterior width compared to posterior width

Grok-4: D GPT-o3: D GPT-4o: D DeepSeek-V2.5: D GPT-5: D

51. The most appropriate examination for diagnosing lumbar disc herniation is: ( )

A. CT

B. Ultrasound

C. X-ray

D. MRA

E. DSA

Grok-4: A GPT-o3: A GPT-4o: A GPT-5: A

60. The typical imaging manifestation of lumbar facet joint disease is: ( )

A. Loss of lumbar physiological curvature

B. Lumbar osteophyte formation

C. Decreased lumbar bone density

D. Narrowing of the intervertebral foramen

E. Bamboo spine appearance of the lumbar spine

Grok-4: B GPT-o3: D GPT-4o: B DeepSeek-V2.5: A GPT-5: D

61. Lumbar CT shows narrowed facet joint space and articular surface proliferation, with all other examinations normal. The most likely diagnosis is: ( )

A. Rheumatoid arthritis

B. Ankylosing spondylitis

C. Tuberculous arthritis

D. Suppurative arthritis

E. Lumbar facet joint disease

Grok-4: E GPT-o3: E GPT-4o: E DeepSeek-V2.5: E GPT-5: E

69. The best imaging examination for diagnosing lumbar disc herniation is: ( )

A. MRI

B. Infrared thermal imaging

C. X-ray

D. Myelography

E. Ultrasound

Grok-4: A GPT-o3: A GPT-4o: A DeepSeek-V2.5: A GPT-5: A

70. To determine whether a patient with lumbar disc herniation has nerve damage, the most appropriate examination is: ( )

A. X-ray

B. MRI

C. Electromyography

D. Ultrasound

E. PET

Grok-4: C GPT-o3: C GPT-4o: C DeepSeek-V2.5: C GPT-5: C

89. Which examination is most valuable for diagnosing lumbar disc herniation?

A. X-ray

B. CT

C. MRI

D. Ultrasound

Grok-4: C GPT-o3: C GPT-4o: C DeepSeek-V2.5: C GPT-5: C

90. Low back pain that is relieved by activity but worsened after fatigue is most likely:

A. Lumbar tuberculosis

B. Lumbar fracture

C. Lumbar spondylolisthesis

D. Lumbar muscle strain

Grok-4: D GPT-o3: D GPT-4o: D DeepSeek-V2.5: D GPT-5: D

91. The typical symptom of lumbar spinal stenosis is:

A. Low back pain with lower limb numbness

B. Intermittent claudication

C. Lower limb weakness

D. Bladder and bowel incontinence

Grok-4: B GPT-o3: B GPT-4o: B DeepSeek-V2.5: B GPT-5: B

95. Diagnosis of low back pain usually requires which examinations?

A. X-ray

B. MRI

C. CT scan

D. All of the above

Grok-4: D GPT-o3: D GPT-4o: D DeepSeek-V2.5: D GPT-5: D

110. Regarding imaging examinations for low back pain, which statement is correct? ( )

A. X-ray can clearly show the condition of intervertebral discs

B. CT has better soft tissue resolution than MRI

C. MRI can better display soft tissues such as the spinal cord and nerves

D. Ultrasound is the first-line method for diagnosing low back pain

Grok-4: C GPT-o3: C GPT-4o: C DeepSeek-V2.5: C GPT-5: C

**F. Clinical Treatment or Drug Therapy for Low Back Pain (G:13/13 C3:11/13 C4:12/13 D:13/13)**

14. Contraindications for sacral hiatus epidural injection include:

A. Lumbar spinal stenosis

B. Low back and leg pain caused by spinal tuberculosis

C. Degenerative lumbar instability

D. Lumbar osteoarthritis

E. Lower lumbar disc herniation

Grok-4: B GPT-o3: B GPT-4o: B DeepSeek-V2.5: B GPT-5: B

58. For patients with low back pain caused by grade I spondylolisthesis from isthmic defect, which treatment is inappropriate? ( )

A. Wearing a lumbar brace

B. Drug analgesia

C. Medium-frequency electrotherapy

D. Rotational manipulation reduction

E. Lumbar and abdominal muscle training

Grok-4: D GPT-o3: D GPT-4o: D DeepSeek-V2.5: D GPT-5: D

62. Which statement about the clinical management of lumbar facet joint disease is incorrect? ( )

A. Patients with articular dysfunction can be treated with traction

B. During the acute phase, physiotherapy can be used to relieve pain

C. During the recovery phase, the main approach is strengthening lumbar functional training

D. For joint dysfunction, intra-articular injection can be performed

E. The treatment plan is mainly surgical treatment

Grok-4: E GPT-o3: E GPT-4o: E DeepSeek-V2.5: E GPT-5: E

71. The most basic treatment for patients with acute lumbar disc herniation during the acute phase is: ( )

A. Lumbar traction

B. Anti-inflammatory analgesic medication

C. Bed rest

D. Local pain point block

E. Ultrashort wave thermal treatment

Grok-4: C GPT-o3: C GPT-4o: C DeepSeek-V2.5: C GPT-5: C

74. Contraindications for rapid traction treatment of lumbar disc herniation include: ( )

A. Lumbar facet joint dysfunction

B. Severe osteoporosis

C. Lumbar pseudospondylolisthesis

D. Early ankylosing spondylitis

E. All of the above

Grok-4: B GPT-o3: E GPT-4o: E DeepSeek-V2.5: B GPT-5: E

75. Elderly patients with lumbar disc herniation, especially those with cardiopulmonary diseases, should be particularly cautious about: ( )

A. Lumbar traction

B. Short-wave therapy

C. Medium-frequency electrotherapy

D. Bed rest

E. Exercise therapy

Grok-4: A GPT-o3: A GPT-4o: A DeepSeek-V2.5: A GPT-5: A

80. For lumbar facet joint disease with synovial entrapment and severe low back pain, the preferred treatment is: ( )

A. Surgical treatment

B. Manual therapy

C. Acupuncture

D. External Chinese herbal medicine

E. Wax therapy

Grok-4: B GPT-o3: B GPT-4o: B DeepSeek-V2.5: B GPT-5: B

81. Lumbar fibromyositis does NOT require: ( )

A. Paraffin therapy

B. High-frequency electrotherapy

C. Low-frequency electrotherapy

D. Massage treatment

E. Traction treatment

Grok-4: E GPT-o3: E GPT-4o: E DeepSeek-V2.5: E GPT-5: E

96. What are the non-drug treatment methods for low back pain?

A. Physical therapy

B. Massage

C. Hot compress

D. All of the above

Grok-4: D GPT-o3: D GPT-4o: D DeepSeek-V2.5: D GPT-5: D

97. Drug treatment for low back pain usually includes which medications?

A. NSAIDs (Non-steroidal anti-inflammatory drugs)

B. Muscle relaxants

C. Analgesics

D. All of the above

Grok-4: D GPT-o3: D GPT-4o: D DeepSeek-V2.5: D GPT-5: D

100. Surgical treatment for low back pain is usually indicated in which situations?

A. Conservative treatment is ineffective

B. Disc herniation compressing nerves

C. Low back pain seriously affects quality of life

D. All of the above

Grok-4: D GPT-o3: D GPT-4o: D DeepSeek-V2.5: D GPT-5: D

103. After acute lumbar sprain, which of the following management methods is correct? ( )

A. Apply heat immediately

B. Start vigorous exercise immediately

C. Apply cold first, then hot compress after 24-48 hours

D. Self-massage the lumbar area

Grok-4: C GPT-o3: C GPT-4o: C DeepSeek-V2.5: C GPT-5: C

107. Which of the following drugs is commonly used as a non-steroidal anti-inflammatory drug to relieve low back pain? ( )

A. Morphine

B. Aspirin

C. Dexamethasone

D. Mecobalamin

Grok-4: B GPT-o3: B GPT-4o: B DeepSeek-V2.5: B GPT-5: B

**G. Rehabilitation and Patient Education for Low Back Pain (G:18/22 C3:20/22 C4:20/22 D:19/22)**

6. Which of the following statements about the mechanism of lumbar traction therapy is incorrect?

A. The intervertebral space widens, reducing pressure on the intervertebral disc

B. The facet joints slide superiorly and inferiorly, correcting joint subluxation

C. The herniated nucleus pulposus is completely reduced

D. The tension of the posterior longitudinal ligament is significantly increased, and the ligamentum flavum is stretched

E. Adhesions around nerve roots are released

Grok-4: C GPT-o3: C GPT-4o: C DeepSeek-V2.5: C GPT-5: C

7. A patient with lumbar disc herniation who has an implanted cardiac pacemaker — which physical therapy method is inappropriate?

A. Lumbar traction

B. Short-wave therapy

C. Paraffin therapy

D. Infrared light treatment

Grok-4: B GPT-o3: B GPT-4o: B DeepSeek-V2.5: B GPT-5: B

9. Which of the following descriptions of lumbar traction therapy is incorrect?

A. The weight starts from 80% of body weight and gradually increases to 150%

B. Increases intervertebral space and intervertebral foramen

C. Reduces the degree of nerve root compression

D. Stretches contracted muscle groups and improves blood circulation

E. Reduces nerve root edema

Grok-4: A GPT-o3: A GPT-4o: A DeepSeek-V2.5: A GPT-5: A

13. To avoid low back pain, good lifestyle habits should be established. Which description is correct?

A. The pressure on the spine is minimal when standing

B. When sneezing or coughing, it is easy to strain the back muscles and increase lumbar disc pressure; at this time, slightly extend the knees and hip joints

C. When turning, do not only twist the upper body; try to rotate the entire body

D. Avoid prolonged sitting; if prolonged sitting is necessary, use a cushion to support the lower back and use a low-back chair

E. When standing, maintain appropriate lumbar posterior angulation

Grok-4: C GPT-o3: C GPT-4o: C DeepSeek-V2.5: C GPT-5: C

15. Which description of the prognosis of low back pain is correct?

A. The prognosis of acute low back pain is very good; it is self-limiting in most cases, usually less than 1 month

B. Most of the time, acute low back pain does not recur

C. Most patients with chronic low back pain can be fully cured

D. Biomedical factors are more important to prognosis than social and psychological factors

E. Fear-avoidance beliefs during work and physical activity, as well as lack of confidence in coping with pain, are closely related to the prognosis of acute low back pain patients

Grok-4: A GPT-o3: A GPT-4o: A DeepSeek-V2.5: E GPT-5: E

18. The general recommended bed rest duration during the early stage of acute low back pain is:

A. 1-2 days

B. 2-3 days

C. 3-4 days

D. 4-5 days

E. 5-6 days

Grok-4: A GPT-o3: A GPT-4o: B DeepSeek-V2.5: A GPT-5: B

28. Which description of manual therapy for low back pain is correct?

A. The main role is to relieve pain and improve spinal mobility

B. Various manual therapies do not have their own systems

C. Various manual therapies have no unique operating methods

D. Maitland's spinal joint mobilization is the first choice

E. McKenzie's spinal mechanical therapy is the first choice

Grok-4: A GPT-o3: A GPT-4o: A DeepSeek-V2.5: A GPT-5: A

30. Regarding the mechanism of physical agent therapy for low back pain, which is incorrect?

A. Promotes reduction of herniated intervertebral discs

B. Promotes local blood circulation

C. Relieves local aseptic inflammation

D. Reduces edema and congestion, relieves pain

E. Releases adhesions, promotes tissue regeneration

Grok-4: A GPT-o3: A GPT-4o: A DeepSeek-V2.5: A GPT-5: A

34. Regarding the mechanism of slow lumbar traction, which is incorrect?

A. Can produce an instantaneous traction force on the posterior longitudinal ligament, significantly increasing its tensile stress

B. Is a sustained traction; has obvious effect on relieving lumbar back muscle spasm, and pain reduces after spasm is relieved

C. During sustained traction, widening of the lumbar intervertebral space can partially reduce the herniation, decreasing mechanical stimulation to the nerve root

D. Can increase the area of the intervertebral foramen and the gap between upper and lower facet joints, reducing compression on the joint synovium

E. Releases nerve root adhesions

Grok-4: A GPT-o3: A GPT-4o: A DeepSeek-V2.5: A GPT-5: A

52. The correct posture for a patient with low back pain when lifting an object from the ground is: ( )

A. Bend the waist and round the back, bring the object as close to the body as possible

B. Bend the waist and round the back, hold the object as far from the body as possible

C. Bend the knees and keep the back straight, bring the object as close to the body as possible

D. Bend the knees and keep the back straight, hold the object as far from the body as possible

E. Bend the knees and lift the object

Grok-4: C GPT-o3: C GPT-4o: C DeepSeek-V2.5: C GPT-5: C

63. The most appropriate method to prevent lumbar facet joint dysfunction is: ( )

A. Lumbar oblique thrust manipulation

B. Lumbar traction

C. Heat therapy

D. Magnetic therapy

E. Lumbar back muscle functional exercise

Grok-4: E GPT-o3: E GPT-4o: E DeepSeek-V2.5: E GPT-5: E

72. The optimal rest position during the acute phase for a patient with lumbar disc herniation is: ( )

A. Supine position with slight hip and knee flexion

B. Lateral decubitus on the affected side

C. Lateral decubitus on the healthy side

D. Sitting position

E. Prone position

Grok-4: A GPT-o3: A GPT-4o: A DeepSeek-V2.5: A GPT-5: A

73. The reasonable bed rest duration for patients with lumbar disc herniation is:

A. 1-2 days

B. 2-4 days

C. 4-7 days

D. 7-14 days

E. 14-21 days

Grok-4: D GPT-o3: C GPT-4o: C DeepSeek-V2.5: C GPT-5: C

77. The initial weight for lumbar traction should generally not be less than what percentage of body weight? ( )

A. 20%

B. 30%

C. 40%

D. 60%

E. 100%

Grok-4: C GPT-o3: C GPT-4o: B DeepSeek-V2.5: B GPT-5: B

78. The maximum continuous use time of a lumbar brace for patients with lumbar disc herniation should not exceed: ( )

A. 1 week

B. Half a month

C. 1 month

D. 2 months

E. 3 months

Grok-4: C GPT-o3: C GPT-4o: C DeepSeek-V2.5: E GPT-5: C

79. Most lumbar disc herniations are caused by degeneration and chronic injury. Which of the following is NOT an appropriate preventive measure? ( )

A. Appropriate intensity physical exercise

B. Avoid bent-over work

C. Long-term use of lumbar support brace

D. Strengthening lumbar back muscle exercise

E. Standing posterior leg extension exercise

Grok-4: C GPT-o3: C GPT-4o: C DeepSeek-V2.5: C GPT-5: C

94. Which activities should low back pain patients avoid?

A. Prolonged standing

B. Heavy lifting

C. Prolonged sitting

D. All of the above

Grok-4: D GPT-o3: D GPT-4o: D DeepSeek-V2.5: D GPT-5: D

98. Daily nursing recommendations for low back pain patients include:

A. Maintain good sitting posture

B. Regularly perform lumbar stretching exercises

C. Avoid maintaining the same posture for a long time

D. All of the above

Grok-4: D GPT-o3: D GPT-4o: D DeepSeek-V2.5: D GPT-5: D

99. What are the preventive measures for low back pain?

A. Strengthen lumbar muscle exercise

B. Keep body weight within normal range

C. Avoid repetitive lumbar loading

D. All of the above

Grok-4: D GPT-o3: D GPT-4o: D DeepSeek-V2.5: D GPT-5: D

101. What dietary considerations should low back pain patients keep in mind in daily life?

A. Increase calcium intake

B. Reduce caffeine intake

C. Maintain a balanced diet

D. All of the above

Grok-4: D GPT-o3: D GPT-4o: D DeepSeek-V2.5: D GPT-5: D

106. When a low back pain patient performs rehabilitation exercises, which of the following exercises is NOT appropriate? ( )

A. Superman exercise (small flying swallow)

B. Sit-ups

C. Swimming

D. Plank exercise

Grok-4: B GPT-o3: B GPT-4o: B DeepSeek-V2.5: B GPT-5: B

108. Which of the following is NOT a postural factor causing low back pain? ( )

A. Long-term bent-over work

B. Prolonged sitting

C. Sleeping on a hard board bed

D. Crossing legs

Grok-4: C GPT-o3: C GPT-4o: C DeepSeek-V2.5: C GPT-5: C
