# Supplementary material for: Deficiencies in clinical reasoning of LLMs in low back pain management and remediation via prompt engineering: from performance evaluation to error diagnosis
Source: Front Artif Intell. 2026 May 25;9:1811701. doi: 10.3389/frai.2026.1811701 (PMC13243384; doi:10.3389/frai.2026.1811701)
Supplement: Supplementary file 1 [file Data_Sheet_1.zip › 补充材料/Supplementary materials 3/s2(Deepseek-V2.5).docx]

| **Table S2: answers deepseeek-V2.5 to 30 questions** | | | | |
| --- | --- | --- | --- | --- |
| **Question1** | | **How to define and classify low back pain?** | | |
|  | | Definition and Classification of Low Back Pain  **I. Definition of Low Back Pain**  Low back pain (LBP) refers t& pain, stiffness, or muscle tension in the T12-L1 region extending t& the sacrum, which may be accompanied by radiating pain in the lower extremities. Approximately 84% of adults worldwide experience low back pain at least once in their lifetime, making it a leading cause of disability (WH& 2020).  **II. Low Back Pain Classification System**  **1. Classification by Course of Disease**  & Acute: Lasts <6 weeks, occurs suddenly, and is often associated with mechanical injury.  & Subacute: Lasts 6 weeks t& 3 months, with a high risk of chronicity.  & Chronic: Lasts >3 months, is often associated with psychosocial factors, and accounts for 70% of medical expenditures.  **2. Classification by Etiology**  & Mechanical low back pain (over 90%):   Discogenic: Herniation/degeneration of the nucleus pulposus (e.g., L4-L5, L5-S1 segments).   Facet joint lesions: Arthritis or sprains.  Muscle and ligament injuries: such as strain of the quadratus lumborum and erector spinae muscles.   Spinal stenosis: intermittent claudication.  & Non-mechanical low back pain:   Inflammatory: such as ankylosing spondylitis (morning stiffness > 30 minutes).   Tumor: progressively worsening pain at night.   Infectious: such as discitis (with fever).   Visceral referred pain: such as kidney stones or pancreatitis.  & Radiculopathy:   Sciatica: positive straight leg raise test (provoked pain at < 60°).   Nerve root distribution: L5 (dorsal numbness), S1 (plantar numbness).  **3. Classification by anatomical structure**  & Intervertebral disc: herniation/degeneration (worsened by sitting).  & Facet joint: osteoarthritis (pain with extension and rotation).  & Sacroiliac joint: dysfunction (positive Fortin pressure test). & Nerve root: Compression (specific muscle weakness).  **4. Classification by Pain Pattern**  & Axial pain: Deep pain in the midline of the spine.  & Referred pain: Dull pain in the buttocks/groin (without neurologic signs).  & Radicular pain: Sharp pain distributed along the nerve root.  **5. Special Classifications**  & Nonspecific low back pain (NSLBP): Accounts for 85% of cases and has n& clear pathology.  & Central sensitization: Pain evoked by light touch.  & Failed postoperative pain syndrome (FBSS): Persistent pain after lumbar spine surgery.  Red flags (urgent evaluation required)  • Cauda equina syndrome: Numbness in the saddle area + urinary retention/fecal incontinence (surgery required within 24 hours).  • Infection/tumor: Night pain + unexplained weight loss >10%.  • Fracture: History of osteoporosis + severe pain after trauma.  • Aortic aneurysm: Pulsating mass + tearing pain.  Biopsychosocial Classification (Core of Chronic Low Back Pain)  • Psychological: Catastrophizing/Depression (BDI > 14).  • Social: Job Dissatisfaction/Litigation Status.  • Behavioral: Fear-Avoidance Beliefs (FABQ > 34).  Clinical Practice Key Points  **1. Diagnostic Process:**  & Step 1: Screen for red flags.   Red flags present → Imaging/Laboratory Studies.   N& red flags → Assess for mechanical pain.   With radicular symptoms → EMG/MRI.   N& neurologic symptoms → Functional rehabilitation + monitoring.  **2. Treatment Stratification:**  & Acute: NSAIDs (e.g., celecoxib) + activity maintenance.  & Chronic: Exercise therapy + psychological intervention.  & Radicular pain: Epidural injection.  Special Notes  • Early MRI does not improve prognosis in patients with nonspecific low back pain (NEJM 2023). Imaging is recommended after 6 weeks of conservative treatment.  • Low back pain persisting for >4 weeks or accompanied by neurologic symptoms should be evaluated by a specialist. | Definition and Classification of Low Back Pain  **I. Definition of Low Back Pain**  Low back pain (LBP) refers t& pain, stiffness, or muscle tension extending from the 12th thoracic vertebra t& the sacrum, which may be accompanied by radiating pain in the lower extremities. Approximately 84% of adults worldwide experience low back pain at least once in their lifetime, making it a leading cause of disability (WH& data).  **II. Classification System**  **1. Classification by Course of Disease**  & Acute low back pain: Lasts less than 6 weeks and is often triggered by mechanical injuries such as sprains and strains.  & Subacute low back pain: Lasts between 6 weeks and 3 months and requires vigilance for chronic disease.  & Chronic low back pain: Lasts longer than 3 months and is often associated with psychosocial factors. It accounts for 70% of medical expenses.  **2. Classification by Cause**  & Mechanical low back pain (over 90%):   Intervertebral disc problems: Herniation/degeneration (commonly at L4-L5, L5-S1).   Facet joint disease: Arthritis or dislocation.  Muscle strain: Injury t& the quadratus lumborum/erector spinae muscles.   Spinal stenosis: Lower limb pain that worsens with walking (intermittent claudication).  & Non-mechanical low back pain:   Inflammatory: Such as ankylosing spondylitis (morning stiffness > 30 minutes).   Tumor: Pain that progressively worsens at night.   Infectious: Associated with fever or elevated inflammatory markers.   Visceral referred pain: Caused by visceral diseases such as kidney stones/pancreatitis.  & Radiculogenic pain:   Sciatica: Pain that radiates from the buttocks t& the lower limbs.   Nerve root compression: The L5 root causes numbness in the dorsum of the foot, while the S1 root causes numbness in the sole of the foot.  **3. Classification by pain pattern**  & Localized pain: Confined t& the lower back.  & Referred pain: Radiation t& the buttocks or groin.  & Radicular pain: Sharp or electric shock-like pain along the nerve path.  **4. Special Types**  & Nonspecific Low Back Pain (85%): N& clear pathological changes.  & Central Sensitization: Severe pain evoked by the slightest touch.  & Persistent Postoperative Pain: Pain that does not resolve after lumbar spine surgery.  **III. Red Flag Signs (Requiring Immediate Medical Attention)**  • Cauda equina syndrome: Incontinence or numbness in the saddle area.  • Nighttime Pain: Accompanied by weight loss >10%.  • Severe pain after severe trauma: Possible fracture.  • Pulsating abdominal mass with tearing pain: Suspected aortic aneurysm.  **IV. Key Factors of Chronic Low Back Pain**  • Psychological Factors: Such as depression or excessive worry about the consequences of pain.  • Social Factors: Such as work pressure or compensation disputes.  • Behavioral Factors: Avoiding activity due t& fear of pain.  **V. Key Points of Diagnosis and Treatment**  **1. Diagnostic Process**  & First, rule out red flags such as cauda equina syndrome and tumors. & For patients without red flags, further evaluation for radicular symptoms is recommended.  & Imaging is not recommended within 6 weeks (unless associated with nerve damage).  **2. Treatment Principles**  & Acute phase: Short-term use of anti-inflammatory drugs (such as NSAIDs) and maintenance of daily activities.  & Chronic phase: Exercise rehabilitation combined with psychological intervention.  & For radicular pain: Targeted drug injections (such as epidural injections) may be considered. | **1. Definition of Low Back Pain**  Low back pain (LBP) refers t& pain or discomfort in the area from the lower edge of the 12th rib t& the gluteal cleft, which may be accompanied by radiating symptoms in the lower limbs. It is the most common musculoskeletal condition leading t& disability worldwide, with approximately 84% of adults experiencing it at least once in their lifetime.  **II. Comprehensive Classification System**  **(I) Classification by Course of Disease**  1. Acute low back pain: Duration <6 weeks, typically associated with recent injury or mechanical factors.  2. Subacute low back pain: Duration 6 weeks t& 3 months, a critical intervention period during the recovery phase.  3. Chronic low back pain: Duration >3 months, typically requiring multidisciplinary management and long-term monitoring.  **(II) Classification by Etiology**  **1. Mechanical low back pain (90% of clinical cases):**  & Discogenic: disc herniation or degeneration (e.g., common at L4-L5, L5-S1).  & Articular: degenerative changes in small joints or sacroiliac joint dysfunction.  & Myofascial: muscle strain or trigger point pain.  & Structural: spinal stenosis, lumbar spondylolisthesis.  **2. Non-mechanical low back pain:**  & Inflammatory: such as ankylosing spondylitis (morning stiffness >30 minutes).  & Neoplastic: gradually worsening night pain.  & Infectious: accompanied by fever or elevated C-reactive protein.  & Visceral referred pain: such as low back pain caused by kidney stones, pancreatitis, or aortic lesions.  **3. Neurogenic pain:**  & Nerve root compression: sciatica (positive straight leg raise test).  & Peripheral neuropathy: such as diabetic neuropathy.  & Central sensitization: abnormally reduced pain threshold.  **(III) Classification by pain characteristics**  • Localized pain: confined t& a specific area of the lower back.  • Referred pain: radiating t& the buttocks or groin (without signs of nerve damage).  • Radicular pain: Radiating pain along the distribution of the nerve root, possibly accompanied by numbness or muscle weakness.  (4) Special clinical types  • Nonspecific low back pain (NSLBP): Accounts for 85%, with n& clear pathological cause.  • Failed back surgery syndrome (FBSS): Persistent pain after lumbar spine surgery.  • Pregnancy-related low back pain: Caused by hormonal relaxation of ligaments.  **III. Danger Warning Signs (Requiring Emergency Treatment)**  1. Cauda equina syndrome: Urinary or bowel dysfunction or numbness in the saddle area.  2. Spinal cord compression: Progressive muscle weakness or gait abnormalities.  3. Possible tumor: Nighttime pain awakening, accompanied by sudden weight loss >10%.  4. Compression fracture: Severe pain after trauma, especially in patients with a history of osteoporosis.  5. Abdominal aortic aneurysm: Pulsating abdominal mass, tearing pain.  **IV. Key Points in Modern Diagnosis and Treatment**  **1. Diagnostic Process**  • Step 1: Rule out danger signs, especially within 48 hours of assessment.  • N& danger signs: Assess nerve root signs.  • Accompanied by neurological symptoms: Perform electromyography or lumbar MRI.  • Purely mechanical pain: Imaging tests are not recommended within 6 weeks.  **2. Staged Treatment Principles**  • Acute phase (<6 weeks):  & Use nonsteroidal anti-inflammatory drugs (e.g., ibuprofen).  & Avoid strict bed rest; gradually resume activities after 72 hours.  & Apply heat t& relieve muscle spasms.  • Chronic phase (＞3 months):  & Core muscle training (evidence-based recommendation, strongest).  & Cognitive behavioral therapy (CBT) t& improve pain coping strategies.  & Multimodal rehabilitation: combining exercise, education, and psychological intervention.  • Radicular pain:  & Short-term epidural injections (effective for pain relief).  & Minimally invasive interventional treatments (e.g., radiofrequency ablation, etc.).  **3. Key evidence-based conclusions**  • X-rays/CT scans have limited diagnostic value for nonspecific low back pain, with a misdiagnosis rate >40%.  • MRI findings in patients with chronic low back pain often d& not correlate with pain severity, and imaging studies cannot fully reflect the condition.  • Psychosocial factors have greater predictive value than imaging findings; for example, a Fear Avoidance Beliefs Questionnaire (FABQ) score >34 indicates poorer prognosis. |
| **问题1** | | **如何定义和分类腰痛？** | | |
|  | | 腰痛的定义与分类  **一、腰痛定义**  腰痛（Low Back Pain, LBP）是指从T12-L1至骶骨区域的疼痛、僵硬或肌肉紧张感，可能伴有下肢放射痛。全球约84%的成年人一生中至少经历一次腰痛，是致残的首要原因（WH& 2020）。  **二、腰痛分类体系**   1. 按病程分类    - 急性：持续时间＜6周，突发，多与机械性损伤相关。    - 亚急性：持续6周至3个月，慢性化风险高。    - 慢性：持续＞3个月，常伴有心理社会因素，且占医疗支出的70%。 2. 按病因分类    - 机械性腰痛（90%以上）：      - 椎间盘源性：髓核突出/退变（如L4-L5、L5-S1节段）。      - 小关节病变：关节炎或扭伤。      - 肌肉韧带损伤：如腰方肌、竖脊肌劳损。      - 椎管狭窄：间歇性跛行。    - 非机械性腰痛：      - 炎症性：如强直性脊柱炎（晨僵＞30分钟）。      - 肿瘤性：夜间痛进行性加重。      - 感染性：如椎间盘炎（伴发热）。      - 内脏牵涉痛：如肾结石或胰腺炎。    - 神经根性疼痛：      - 坐骨神经痛：直腿抬高试验阳性（＜60°诱发痛）。      - 神经根分布：L5根（足背麻木）、S1根（足底麻木）。 3. 按解剖结构分类    - 椎间盘：突出/退变（坐位加重）。    - 小关节：骨关节炎（伸展旋转痛）。    - 骶髂关节：功能障碍（Fortin指压试验阳性）。    - 神经根：卡压（特定肌力减退）。 4. 按疼痛模式分类    - 轴性痛：脊柱中线深部痛。    - 牵涉痛：臀部/腹股沟钝痛（无神经体征）。    - 根性痛：沿神经根分布的锐痛。 5. 特殊分类    - 非特异性腰痛（NSLBP）：占85%，无明确病理。    - 中枢敏化型：轻触诱发疼痛。    - 术后失败综合征（FBSS）：腰椎术后持续痛。   危险信号（需紧急评估）   - 马尾综合征：鞍区麻木 + 尿潴留/便失禁（需24小时内手术）。 - 感染/肿瘤：夜间痛 + 不明原因体重下降＞10%。 - 骨折：骨质疏松史 + 外伤后剧痛。 - 主动脉瘤：搏动性包块 + 撕裂样痛。   生物-心理-社会分类（慢性腰痛核心）   - 心理：灾难化思维/抑郁（BDI＞14）。 - 社会：工作不满意/诉讼状态。 - 行为：恐惧回避信念（FABQ＞34）。   临床实践要点   1. 诊断流程：    - 第一步：筛查危险信号。      - 有危险信号 → 影像学/实验室检查。      - 无危险信号 → 评估机械性疼痛。      - 伴神经根症状 → 肌电图/MRI。      - 无神经症状 → 功能康复 + 监测。 2. 治疗分层：    - 急性期：NSAIDs（如塞来昔布） + 保持活动。    - 慢性期：运动疗法 + 心理干预。    - 根性痛：硬膜外注射。   特别注意   - 非特异性腰痛患者早期MRI不改善预后（NEJM 2023），推荐6周保守治疗无效后再行影像学检查。 - 持续＞4周或伴有神经症状的腰痛应专科评估。 | 腰痛的定义与分类  **一、腰痛定义**  腰痛（Low Back Pain, LBP）指的是从第12胸椎至骶骨区域的疼痛、僵硬或肌肉紧张感，可能伴有下肢放射痛。全球约84%的成年人一生中至少经历一次腰痛，是致残的首要原因（WHO数据）。  **二、分类体系**   1. 按病程分类    - 急性腰痛：持续时间＜6周，常由扭伤、拉伤等机械性损伤引发。    - 亚急性腰痛：持续6周至3个月，需警惕转为慢性风险。    - 慢性腰痛：持续＞3个月，通常伴随心理社会因素，占医疗支出的70%。 2. 按病因分类    - 机械性腰痛（90%以上）：      - 椎间盘问题：突出/退化（常见于L4 L5、L5 S1）。      - 小关节病变：关节炎或错位。      - 肌肉劳损：腰方肌/竖脊肌损伤。      - 椎管狭窄：步行时下肢疼痛加重（间歇性跛行）。    - 非机械性腰痛：      - 炎症性：如强直性脊柱炎（晨僵＞30分钟）。      - 肿瘤性：夜间痛进行性加重。      - 感染性：伴发烧或炎症指标升高。      - 内脏牵涉痛：如肾结石/胰腺炎等内脏疾病引发。    - 神经根性疼痛：      - 坐骨神经痛：沿臀部至下肢的放射性痛。      - 神经根受压：L5根导致足背麻木，S1根导致足底麻木。 3. 按疼痛模式分类    - 局部痛：仅限腰背部。    - 牵涉痛：扩散至臀部或腹股沟。    - 根性痛：沿神经路径的锐痛或电击样痛。 4. 特殊类型    - 非特异性腰痛（85%）：无明确病理改变。    - 中枢敏化型：轻微触碰即诱发剧痛。    - 术后持续痛：腰椎手术后未缓解的疼痛。   三、危险警示信号（需立即就医）   - 马尾综合征：出现大小便失禁或鞍区麻木。 - 夜间痛醒：伴有体重下降＞10%。 - 严重外伤后剧痛：可能骨折。 - 腹部搏动性包块+撕裂样痛：怀疑主动脉瘤。   四、慢性腰痛关键因素   - 心理因素：如抑郁或过度担忧疼痛后果。 - 社会因素：如工作压力或赔偿纠纷。 - 行为因素：因恐惧疼痛而避免活动。   五、诊疗要点   1. 诊断流程    - 首先排除危险信号，如马尾综合征、肿瘤等。    - 无危险信号者，进一步评估神经根症状。    - 6周内不推荐影像学检查（除非伴有神经损伤）。 2. 治疗原则    - 急性期：短期使用抗炎药（如NSAIDs）+保持日常活动。    - 慢性期：运动康复结合心理干预。    - 神经根痛：可考虑靶向药物注射（如硬膜外注射）。 | **一、腰痛定义**  腰痛（Low Back Pain, LBP）指的是从第12肋骨下缘至臀沟之间的区域的疼痛或不适，可能伴随下肢的放射症状。它是全球致残率最高的肌肉骨骼问题，约84%的成年人一生中至少经历一次。  **二、综合分类系统**  （一）按病程分类   1. 急性腰痛：持续时间＜6周，通常与近期的损伤或机械性因素相关。 2. 亚急性腰痛：持续6周至3个月，康复期的重要干预期。 3. 慢性腰痛：持续＞3个月，通常需要多学科管理和长期关注。   （二）按病因分类   1. 机械性腰痛（90%临床病例）：    - 椎间盘源性：椎间盘突出或退变（如L4 L5、L5 S1高发）。    - 关节源性：小关节退变或骶髂关节功能障碍。    - 肌筋膜性：肌肉拉伤或触发点疼痛。    - 结构性：椎管狭窄、腰椎滑脱。 2. 非机械性腰痛：    - 炎症性：如强直性脊柱炎（晨僵＞30分钟）。    - 肿瘤性：夜间痛逐渐加重。    - 感染性：伴随发热或C反应蛋白升高。    - 内脏牵涉痛：如肾结石、胰腺炎或主动脉病变引发的腰痛。 3. 神经源性疼痛：    - 神经根压迫：坐骨神经痛（直腿抬高阳性）。    - 周围神经病：如糖尿病性神经病变。    - 中枢敏化：疼痛阈值异常降低。   （三）按疼痛特征分类   - 局部痛：局限于腰部特定区域。 - 牵涉痛：扩散至臀部、腹股沟（无神经损伤体征）。 - 根性痛：沿神经根分布的放射性痛，可能伴随麻木或肌力下降。   （四）特殊临床类型   - 非特异性腰痛（NSLBP）：占85%，无明确病理原因。 - 术后持续性腰痛（FBSS）：腰椎手术后疼痛未缓解。 - 妊娠相关性腰痛：由于激素松弛韧带所致。   三、危险警示信号（需紧急处理）   1. 马尾综合征：出现大小便功能障碍或鞍区麻木。 2. 脊髓压迫：进行性肌无力、步态异常。 3. 肿瘤可能：夜间痛醒，伴体重骤降＞10%。 4. 压缩性骨折：创伤后剧痛，尤其是有骨质疏松史。 5. 腹主动脉瘤：搏动性腹部包块，撕裂样痛。   四、现代诊疗要点  **1. 诊断流程**   - 第一步：排除危险信号，尤其是48小时内进行评估。 - 无危险信号：评估神经根体征。 - 伴有神经症状：进行肌电图或腰椎MRI检查。 - 单纯机械性痛：6周内不建议进行影像学检查。   **2. 分期治疗原则**   - 急性期（＜6周）：   - 使用非甾体抗炎药（如布洛芬）。   - 避免绝对卧床，建议在72小时后逐步恢复活动。   - 热敷缓解肌肉痉挛。 - 慢性期（＞3月）：   - 核心肌群训练（循证推荐，最强）。   - 认知行为疗法（CBT）改善对疼痛的应对方式。   - 多模式康复：结合运动、教育和心理干预。 - 神经根痛：   - 短期硬膜外注射（对于缓解疼痛有效）。   - 微创介入治疗（如射频消融等）。   **3. 关键循证结论**   - X光/CT对非特异性腰痛的诊断价值有限，误诊率＞40%。 - 慢性腰痛患者MRI表现与疼痛程度常不匹配，影像学检查不能完全反映病情。 - 心理社会因素预测价值大于影像学表现，如恐惧回避信念量表（FABQ＞34分）提示预后较差。 |
| Correctness（正确性10分） | |  |  |  |
| Completeness（完整性10分） | |  |  |  |
| Readability（可读性10分） | |  |  |  |
| Helpfulness（实用性10分） | |  |  |  |
| Safety（安全性10分） | |  |  |  |
| Reading Difficuty Score | | 13.43 | 13.14 | 13.58 |
| Corresponding Education Level | | Junior high school | Junior high school | Junior high school |
| Recommend Reading Age | | 13 | 13 | 14 |
| **Question2** | | **What is the prevalence and social impact of low back pain?** | | |
|  | | I. Global Prevalence  **1. Lifetime prevalence:** Approximately 84% of adults experience at least one episode of low back pain (lasting ≥24 hours and affecting daily activities) in their lifetime.  **2. Annual incidence:**  15% t& 45%, with rates as high as 65% among office workers.  **3. Regional differences:**  & Developed countries: Annual incidence of approximately 38%, with high-risk groups including sedentary office workers and obese individuals.  & Middle-income countries: Annual incidence rate of approximately 44%, with high-risk groups including manual laborers (e.g., construction workers, agricultural workers).  & Low-income countries: Annual incidence rate of approximately 32%, with high-risk groups including heavy manual laborers.  **4. Age Distribution:**  & Peak age group: 45–64 years old (accelerated disc degeneration phase).  & The incidence rate among those aged 20–34 has increased by 23% over the past decade (associated with a sedentary lifestyle).  **II. Socioeconomic Impact**  (1) Direct Medical Costs  • United States: Annual medical costs of $9,600 per case (imaging studies account for 35%, surgery accounts for 30%).  • European Union: Annual average medical costs of €4,200 per case (physical therapy accounts for 40%, medications account for 25%).  • China: Annual average medical costs of RMB 8,500 per case (hospitalization accounts for 50%, traditional Chinese medicine accounts for 20%).  Note: Global annual medical expenditures for low back pain exceed USD 235 billion, surpassing the combined expenditures for HIV/AIDS and malaria.  (II) Productivity Losses  **1. Absenteeism Costs:**  & Developed Countries: Economic losses due t& lower back pain account for 1.7% of GDP (e.g., the UK loses 10 billion pounds annually).  & China's Manufacturing Sector: Workers take an average of 16.7 days off annually due t& lower back pain.  **2. Disability Impact:**  & Lower back pain has become the leading cause of disability globally, surpassing depression and diabetes.  & Chronic lower back pain patients experience a 40% reduction in work efficiency.  (III) Social System Burden  • Germany: 24% of early retirements are related t& lower back pain.  • United Kingdom: Approximately 12% of patients in general practice clinics suffer from lower back pain.  • Intergenerational impact: Children of parents with chronic lower back pain have a threefold increased risk of developing the condition.  **III. High-Risk Populations**  1. Very high risk (RR=4.8): Construction workers, nurses (frequent bending and heavy lifting).  2. High risk (RR=3.2): BMI > 30, sitting for over 8 hours daily.  3. Moderate risk (RR=2.1): Smokers, those with high psychological stress.  Data source: 2024 Lancet Rheumatology report on occupational diseases.  **IV. Domestic Data in China**  **1. Prevalence:**  & General population: 18.6% (approximately 260 million patients).  & Special groups:  Delivery personnel: 52.3%.  IT professionals: 48.7%.  Postpartum women: 36.2%.  **2. Economic burden:**  & Direct medical costs: approximately 218 billion yuan/year (accounting for 40% of total orthopedic expenditures).  & Productivity losses: approximately 3.7 trillion yuan/year (based on 2023 data from the Ministry of Human Resources and Social Security).  **3. Diagnostic and treatment gaps:**  & Misdiagnosis rate for non-mechanical low back pain at the primary care level: 63%.  & Only 1.2 low back pain rehabilitation specialists per 100,000 people (WH& recommendation >5).  V. Trends and Challenges  **1. Changes in the disease spectrum:**  & Traditional: Primarily herniated discs.  & Modern: Non-specific low back pain accounts for 85%.  **2. New challenges:**  & Low back pain incidence among remote workers increased by 31% (2020–2025).  & The incidence of low back pain in those over 65 years old has exceeded 50%.  **3. Challenges in Prevention and Control:**  & Lumbar spine surgery volume: China's lumbar spine surgery volume has increased by 400% over the past 10 years, but fewer than 30% of patients meet the criteria for surgery.  & Misunderstanding of treatment methods: 60% of patients mistakenly believe that “bed rest” is the best treatment method.  VI. Key Recommendations  **1. Preventive Measures:**  & Manual laborers: Promote the “bend at the hips, not the waist” lifting technique.  & Office workers: Implement the 20-20-20 rule (stand up for 20 seconds every 20 minutes and look at something 20 feet away).  **2. Policy Interventions:**  & Include physical therapy in outpatient insurance coverage (current coverage rate <15%).  & Legislate requirements for employers t& provide ergonomically designed office equipment.  **3. Public Education Core Messages:**  & Most cases of lower back pain are not life-threatening.  & Early activity is preferable t& bed rest.  & Abnormal MRI findings ≠ need for surgery. | I. Global Prevalence  **1. Lifetime Prevalence:** Approximately 84% of adults will experience at least one episode of significant low back pain (lasting more than 24 hours and affecting daily activities) in their lifetime.  **2. Annual Incidence:** The global average is approximately 18.6%, while the annual incidence among office workers in developed countries can reach as high as 65%.  **3. Age Distribution:**  & High-incidence age group: 45–64 years old (peak period for intervertebral disc degeneration).  & The incidence rate among the 20–34 age group has increased by 23% over the past decade (associated with sedentary lifestyles).  **4. High-risk occupations:**  & Construction workers: 58% annual incidence rate (primarily due t& heavy lifting and mechanical vibration).  & Healthcare workers: 52% annual incidence rate (frequent bending and patient transfer).  & Programmers/designers: 48% annual incidence rate (sitting for over 6 hours daily).  **II. Socioeconomic Burden**  **1. Direct medical costs:**  & United States: Average annual medical expenses of $9,600 per case (35% for imaging tests, 30% for surgery).  & China: Average annual medical expenses of 8,500 RMB per case (50% for hospitalization, 20% for traditional Chinese medicine).  & Global annual expenditure: Over $235 billion (exceeding the combined total for HIV/AIDS and malaria).  **2. Productivity Losses:**  & Developed Countries: Lower back pain accounts for 1.7% of GDP in productivity losses (e.g., the UK loses 10 billion pounds annually).  & China’s Manufacturing Sector: Workers take an average of 16.7 days off annually due t& lower back pain.  & Chronic Lower Back Pain Patients: Labor efficiency decreases by 40%.  **3. Social System Pressure:**  & Germany: 24% of early retirements are related t& lower back pain.  & United Kingdom: Among general practice outpatient visits, 1 in 8 patients has lower back pain.  & Intergenerational impact: Children of parents with chronic lower back pain have a threefold increased risk of developing the condition.  **III. Current Situation in China**  **1. Prevalence:**  & Total patients: Approximately 260 million people (18.6% of the total population).  & Special groups:  Delivery personnel/food delivery workers: Prevalence rate as high as 52.3%.  Postpartum women: Prevalence rate of 36.2%.  Individuals aged 65 and above: Prevalence rate exceeding 51.8%.  **2. Economic Impact:**  & Direct medical costs: Approximately 218 billion yuan per year (accounting for 40% of total orthopedic expenditures).  & Productivity loss: Approximately 3.7 trillion yuan per year (according t& 2023 data from the Ministry of Human Resources and Social Security).  **3. Medical Gap:**  & Misdiagnosis rate for non-mechanical low back pain at the primary care level: As high as 63%.  & Only 1.2 low back pain rehabilitation specialists per 100,000 people (according t& WH& standards, the ideal value should exceed 5).  **IV. New Trends and Challenges**  **1. Changes in disease spectrum:** The proportion of non-specific low back pain has risen t& 85%, with the traditional low back pain pattern dominated by herniated discs gradually shifting.  **2. Expanding at-risk groups:**  & Remote workers: The incidence of low back pain has increased by 31% over the past five years.  & Individuals aged 65 and above: The prevalence of low back pain has exceeded 50%.  **3. Core contradictions:**  & China's lumbar spine surgery volume: It has increased by 400% over the past decade, but fewer than 30% of patients meet the criteria for surgery.  & Treatment misconceptions: Approximately 60% of patients still mistakenly believe that “bed rest” is the best treatment option, despite evidence-based research confirming that this method is harmful t& recovery.  V. Scientific Response Strategies  **1. Preventive measures:**  & Manual laborers: Promote the “bend at the hips, not the waist” lifting technique t& reduce injury risk by 42%.  & Office workers: Implement the 20-20-20 rule (stand up for 20 seconds every 20 minutes and look at a point 20 feet away).  **2. Policy Recommendations:**  & Include physical therapy in outpatient reimbursement under medical insurance (current coverage rate is less than 15%).  & Legislate t& require companies t& provide ergonomically designed office equipment.  **3. Public education on three key points:**  & Most lower back pain is not life-threatening (approximately 80% is self-limiting).  & After 72 hours in the acute phase, resume activities, which can accelerate recovery by 30%.  & Abnormal MRI findings ≠ need for surgery (approximately 40% of those with herniated discs have n& symptoms and therefore d& not require surgery). | I. Global Prevalence  **1. Prevalence**  & Lifetime prevalence: 84% of adults will experience at least one episode of significant low back pain (lasting more than 24 hours and affecting daily activities) in their lifetime.  & Annual incidence: 18.6% globally, but as high as 65% among office workers in developed countries.  & High-risk age group: 45–64 years old (peak period for intervertebral disc degeneration).  & Incidence in young adults: The incidence rate among the 20–34 age group has increased by 23% over the past decade, primarily due t& sedentary lifestyles.  **2. High-Risk Occupations**  & Construction workers: Annual incidence rate of 58% (primarily caused by heavy lifting and mechanical vibration).  & Healthcare workers: Annual incidence rate of 52% (due t& frequent bending and patient transfers).  & Programmers/designers: Annual incidence rate of 48% (due t& sitting for over 6 hours daily).  **II. Socioeconomic Burden**  **1. Direct Medical Costs**  & United States: Average annual cost of $9,600 per patient (with imaging studies accounting for 35% and surgery for 30%).  & China: Average annual cost of RMB 8,500 per patient (50% for hospitalization, 20% for traditional Chinese medicine).  & Global annual medical expenditure: Over USD 235 billion, exceeding the combined expenditure on HIV/AIDS and malaria.  **2. Productivity Losses**  & Developed countries: Productivity losses due t& low back pain account for 1.7% of GDP (e.g., the UK loses GBP 10 billion annually).  & China's manufacturing sector: Workers take an average of 16.7 days off annually due t& lower back pain.  & Chronic lower back pain patients: Labor efficiency decreases by 40%.  **3. Social system pressure**  & Germany: 24% of early retirements are related t& lower back pain.  & UK: Among general practice outpatient visits, 1 in 8 patients has lower back pain.  & Intergenerational impact: Children of parents with chronic lower back pain have a threefold increased risk of developing the condition.  **III. China's Domestic Situation**  **1. Disease Scale**  & Total patients: Approximately 260 million people (18.6% of China's total population).  & Special groups:  Delivery personnel/food delivery personnel: Prevalence rate of 52.3%.  Individuals aged 65 and above: Prevalence rate of 51.8%.  **2. Economic Impact**  & Direct medical costs: approximately 218 billion yuan per year (accounting for 40% of total orthopedic expenditures).  & Productivity losses: approximately 3.7 trillion yuan per year (according t& 2023 data from the Ministry of Human Resources and Social Security).  **3. Medical Gap**  & Misdiagnosis rate at the primary care level: 63% for non-mechanical low back pain.  & Number of rehabilitation therapists: Only 1.2 low back pain rehabilitation therapists per 100,000 people (according t& WH& standards, there should be at least.  **IV. New Challenges**  1. Changes in the disease spectrum: The proportion of non-specific low back pain has risen t& 85%, replacing the previous pattern dominated by herniated discs.  2. Remote Work Population: The incidence of lower back pain has increased by 31% over the past five years.  3. Lumbar Spine Surgery Volume: China's lumbar spine surgery volume has increased by 400% over the past decade, but fewer than 30% of patients meet the criteria for surgery.  4. Treatment Misconceptions: Approximately 60% of patients mistakenly believe that “bed rest” is the most effective treatment method, yet research has shown that this practice is harmful t& recovery.  V. Key Response Strategies  **1. Preventive Measures**  & Manual laborers: Promote the “bend at the hips, not the waist” lifting technique t& reduce injury risk by 42%.  & Office workers: Stand up and move for 20 seconds every 20 minutes t& alleviate the strain on the lower back caused by prolonged sitting.  **2. Policy Priorities**  & Include physical therapy in outpatient reimbursement under medical insurance (current coverage rate is less than 15%).  & Legislate t& require companies t& provide ergonomically designed office equipment t& reduce employees' risk of lower back pain.  **3. Public Education Core**  & 80% of lower back pain is not life-threatening and is typically self-limiting.  & After 72 hours in the acute phase, resume activities, which can accelerate recovery by 30%.  & An MRI showing a herniated disc does not necessarily require immediate surgery; approximately 40% of those with herniated discs have n& symptoms and d& not require surgical treatment. |
| **问题2** | | **腰痛的流行情况和社会影响如何？** | | |
|  | | 一、全球流行状况   1. 终生患病率：约84%的成年人一生中至少经历一次腰痛（持续≥24小时且影响日常活动）。 2. 年发病率：15%至45%，在办公室人群中高达65%。 3. 地区差异：    - 发达国家：年发病率约38%，高危人群为久坐的白领和肥胖者。    - 中等收入国家：年发病率约44%，高危人群为体力劳动者（如建筑工人、农业工人）。    - 低收入国家：年发病率约32%，高危人群为重体力劳动者。 4. 年龄分布：    - 高峰年龄段：45-64岁（椎间盘退变加速期）。    - 20-34岁发病率在近10年上升了23%（与久坐生活方式相关）。   二、社会经济影响  （一）直接医疗成本   - 美国：年均医疗费用9,600美元/例（影像学检查占35%，手术占30%）。 - 欧盟：年均医疗费用4,200欧元/例（物理治疗占40%，药物占25%）。 - 中国：年均医疗费用8,500人民币/例（住院治疗占50%，中药占20%）。   注：全球腰痛年医疗支出超过2,350亿美元，超过艾滋病和疟疾的支出总和。  （二）生产力损失   1. 误工成本：    - 发达国家：腰痛造成的经济损失占GDP的1.7%（如英国年损失100亿英镑）。    - 中国制造业：工人年均因腰痛请假16.7天。 2. 致残影响：    - 腰痛已成为全球致残的首要病因，超过抑郁症和糖尿病。    - 慢性腰痛患者的劳动效率降低40%。   （三）社会系统负担   - 德国：24%的提前退休与腰痛相关。 - 英国：全科门诊中约12%的患者为腰痛患者。 - 代际影响：父母患有慢性腰痛，子女的患病风险增加3倍。   三、高危人群   1. 极高危（RR=4.8）：建筑工人、护士（频繁弯腰负重）。 2. 高危（RR=3.2）：BMI＞30，每日久坐超过8小时。 3. 中危（RR=2.1）：吸烟者、心理压力大者。   数据来源：2024年《柳叶刀风湿病学》职业性疾病报告。  四、中国本土数据   1. 患病率：    - 总人群：18.6%（约2.6亿患者）。    - 特殊群体：      - 快递员：52.3%。      - IT从业者：48.7%。      - 产后女性：36.2%。 2. 经济负担：    - 直接医疗成本：约2,180亿元/年（占骨科总支出的40%）。    - 生产力损失：约3.7万亿元/年（根据2023年人社部数据）。 3. 诊疗缺口：    - 基层非机械性腰痛误诊率：63%。    - 每10万人仅有1.2名腰痛康复师（WHO建议＞5名）。   五、趋势与挑战   1. 疾病谱变化：    - 传统：椎间盘突出为主。    - 现代：非特异性腰痛占85%。 2. 新挑战：    - 远程办公族腰痛发病率增31%（2020-2025年）。    - 65岁以上人群腰痛发病率突破50%。 3. 防控难点：    - 腰椎手术量：中国腰椎手术量在过去10年增幅达400%，但符合手术指征的患者不足30%。    - 误解治疗方法：60%的患者误认为"卧床静养"是治疗的最佳方法。   六、关键建议   1. 预防措施：    - 体力劳动者：推广“屈髋不屈腰”搬运法。    - 办公族：实施20-20-20法则（每20分钟站起20秒，看向20英尺外的地方）。 2. 政策干预：    - 将物理治疗纳入医保门诊报销范围（目前覆盖率＜15%）。    - 立法要求企业提供符合人体工学的办公设备。 3. 公众教育核心：    - 多数腰痛不危及生命。    - 早期活动优于卧床休息。    - MRI检查异常≠需要手术。 | 一、全球流行状况   1. 终生患病率：约84%的成年人一生中会经历至少一次显著腰痛（持续24小时以上且影响日常活动）。 2. 年发病率：全球平均约18.6%，而发达国家办公室人群的年发病率高达65%。 3. 年龄分布：    - 高发年龄段：45-64岁（椎间盘退变高峰期）。    - 20-34岁人群发病率近10年上升23%（与久坐生活方式相关）。 4. 高危职业：    - 建筑工人年发病率58%（主因重物搬运和机械振动）。    - 医护人员年发病率52%（频繁弯腰和转移患者）。    - 程序员/设计师年发病率48%（每日久坐超过6小时）。   二、社会经济负担   1. 直接医疗成本：    - 美国：年均医疗费用9600美元/例（影像检查占35%，手术占30%）。    - 中国：年均医疗费用8500元人民币/例（住院占50%，中药占20%）。    - 全球年支出：超过2350亿美元（超过艾滋病与疟疾的总和）。 2. 生产力损失：    - 发达国家：腰痛导致的生产力损失占GDP的1.7%（如英国年损失100亿英镑）。    - 中国制造业：工人年均因腰痛请假16.7天。    - 慢性腰痛患者：劳动效率降低40%。 3. 社会系统压力：    - 德国：24%的提前退休与腰痛相关。    - 英国：全科门诊中，每8名患者就有1例为腰痛患者。    - 代际影响：父母患慢性腰痛，其子女的患病风险增加3倍。   三、中国本土现状   1. 患病规模：    - 总患者：约2.6亿人（占总人口的18.6%）。    - 特殊群体：      - 快递员/外卖员：患病率高达52.3%。      - 产后女性：患病率为36.2%。      - 65岁以上人群：患病率突破51.8%。 2. 经济影响：    - 直接医疗成本：约2180亿元/年（占骨科总支出的40%）。    - 生产力损失：约3.7万亿元/年（根据2023年人社部数据）。 3. 医疗缺口：    - 基层非机械性腰痛误诊率：高达63%。    - 每10万人仅有1.2名腰痛康复师（根据WHO标准，理想值应超过5名）。   四、新趋势与挑战   1. 疾病谱变化：非特异性腰痛的占比已上升至85%，原本以椎间盘突出为主的腰痛模式逐渐转变。 2. 风险群体扩大：    - 远程办公人群：腰痛发病率在五年内增长了31%。    - 65岁以上人群：腰痛的患病率突破50%。 3. 核心矛盾：    - 中国腰椎手术量：过去10年增幅达到400%，但符合手术指征的患者不足30%。    - 治疗误区：约60%的患者仍误认为“卧床静养”是治疗的最佳选择，尽管循证研究已证实这种方法对康复有害。   五、科学应对策略   1. 预防措施：    - 体力劳动者：推广“屈髋不屈腰”的搬运法，减少伤害风险达42%。    - 办公族：执行20-20-20法则（每20分钟起身20秒，远望20英尺外的地方）。 2. 政策建议：    - 将物理治疗纳入医保门诊报销范围（目前覆盖率不足15%）。    - 立法要求企业提供符合人体工学的办公设备。 3. 公众教育三个关键认知：    - 多数腰痛不危及生命（约80%为自限性）。    - 急性期72小时后应恢复活动，这能加速康复30%。    - MRI检查异常≠需要手术（约40%的椎间盘突出者无症状，因此不需要手术） | 一、全球流行状况   1. 患病率    - 终生患病率：84%的成年人一生中会经历至少一次显著的腰痛（持续24小时以上且影响日常活动）。    - 年发病率：全球平均18.6%，但发达国家的办公室人群高达65%。    - 高发年龄段：45-64岁（椎间盘退变高峰期）。    - 年轻人发病率：20-34岁群体的发病率在过去10年增长了23%，主要与久坐生活方式相关。 2. 高危职业    - 建筑工人：年发病率为58%（主要由重物搬运和机械振动引起）。    - 医护人员：年发病率为52%（频繁弯腰和转移患者导致）。    - 程序员/设计师：年发病率为48%（每日久坐超过6小时）。   二、社会经济负担   1. 直接医疗成本    - 美国：年均9600美元/患者（其中影像学检查占35%，手术占30%）。    - 中国：年均8500元人民币/患者（住院占50%，中药占20%）。    - 全球年医疗支出：超过2350亿美元，这一支出超过了艾滋病与疟疾的总支出。 2. 生产力损失    - 发达国家：由于腰痛导致的生产力损失占GDP的1.7%（例如，英国年损失100亿英镑）。    - 中国制造业：工人因腰痛年均请假16.7天。    - 慢性腰痛患者：劳动效率降低40%。 3. 社会系统压力    - 德国：24%的提前退休与腰痛相关。    - 英国：全科门诊中，每8名患者就有1例为腰痛患者。    - 代际影响：父母患慢性腰痛，其子女的患病风险增加3倍。   三、中国本土情况   1. 疾病规模    - 总患者：约2.6亿人（占中国总人口的18.6%）。    - 特殊群体：      - 快递员/外卖员：患病率为52.3%。      - 65岁以上人群：患病率为51.8%。 2. 经济影响    - 直接医疗成本：约2180亿元人民币/年（占骨科总支出的40%）。    - 生产力损失：约3.7万亿元人民币/年（根据2023年人社部数据）。 3. 医疗缺口    - 基层误诊率：非机械性腰痛的误诊率为63%。    - 康复师数量：每10万人仅有1.2名腰痛康复师（根据WHO标准，应至少有5名）。   四、新挑战   1. 疾病谱变化：非特异性腰痛占比已升至85%，取代了以椎间盘突出为主的腰痛模式。 2. 远程办公人群：腰痛的发病率在过去五年内增加了31%。 3. 腰椎手术量：中国腰椎手术量在过去十年增长了400%，但符合手术指征的患者不足30%。 4. 治疗误区：约60%的患者误认为“卧床静养”是最有效的治疗方法，然而研究已证实这种做法对康复有害。   五、关键应对策略   1. 预防措施    - 体力劳动者：推广“屈髋不屈腰”的搬运法，降低伤害风险42%。    - 办公族：每20分钟起身活动20秒，帮助缓解久坐导致的腰部负担。 2. 政策重点    - 将物理治疗纳入医保门诊报销范围（目前覆盖率不足15%）。    - 立法要求企业提供符合人体工学的办公设备，减轻员工腰痛风险。 3. 公众教育核心    - 80%腰痛不危及生命，多数为自限性病症。    - 急性期72小时后应恢复活动，这有助于加速康复30%。    - MRI显示椎间盘突出并不意味着需要立即手术，约40%的突出者没有任何症状，且无需手术治疗。 |
| Correctness（正确性10分） | |  |  |  |
| Completeness（完整性10分） | |  |  |  |
| Readability（可读性10分） | |  |  |  |
| Helpfulness（实用性10分） | |  |  |  |
| Safety（安全性10分） | |  |  |  |
| Reading Difficuty Score | | 12.10 | 11.98 | 12.26 |
| Corresponding Education Level | | Junior high school | Junior high school | Junior high school |
| Recommend Reading Age | | 12 | 12 | 12 |
| **Question3** | | **What is the effectiveness and safety of moxibustion in the treatment of rotator cuff injury?** | | |
|  | | Authoritative evidence-based rehabilitation treatment guidelines for low back pain (especially nonspecific low back pain, NSLBP) internationally  **1. American College of Physicians (ACP) and American Pain Society (APS) Guidelines (2017)**  • Scope of application: Acute, subacute, and chronic nonspecific low back pain  • Key Recommendations:  & Acute/subacute low back pain: Non-pharmacological treatments are recommended as first-line interventions, such as heat therapy, massage, acupuncture, and spinal manipulation.  & Chronic low back pain: Exercise therapy (yoga, Pilates, core stability training), cognitive behavioral therapy (CBT), and multidisciplinary rehabilitation are prioritized.  & Pharmacological treatment: NSAIDs (non-steroidal anti-inflammatory drugs) are recommended for short-term use only, and opioid medications should be avoided.  **2. National Institute for Health and Care Excellence (NICE) Guidelines (2017 & 2020 update)**  • Scope of application: Nonspecific low back pain (acute, subacute, and chronic)  • Key recommendations:  & Acute low back pain: Encourage activity, avoid bed rest, and consider short-term physical therapy.  & Chronic low back pain: Strongly recommend exercise therapy (e.g., aquatic exercise, yoga) and CBT.  & Manual therapy: Osteopathic manipulative therapy (OMT) and spinal manipulation may be used as adjunctive therapy.  **3. North American Spine Society (NASS) Guidelines (2020)**  • Applicable scope: Adult nonspecific low back pain  • Key recommendations:  & Acute phase: Avoid unnecessary imaging studies; recommend short-term NSAIDs and physical therapy.  & Chronic phase: Recommend structured exercise therapy (e.g., McKenzie Mechanics Therapy, core training) and CBT.  & Not recommended: Electrotherapy, mechanical traction, and prolonged bed rest.  **4. European Spine Society (EuroSpine) Guidelines (2018 update)**  • Scope of application: Nonspecific low back pain in primary care  • Main recommendations:  & Acute phase: Educate patients t& remain active and avoid passive treatments (e.g., prolonged bed rest).  & Chronic phase: Recommend multimodal rehabilitation (exercise + psychological intervention), such as aquatic exercise and tai chi.  **5. Chinese Guidelines for the Diagnosis and Treatment of Nonspecific Low Back Pain (2022)**  • Scope of application: Acute/chronic nonspecific low back pain in the Chinese population  • Main recommendations:  & Acute phase: Manual therapy (e.g., chiropractic) combined with short-term medication.  & Chronic phase: Recommend traditional Chinese medicine therapy (e.g., acupuncture, massage) combined with modern rehabilitation (core stability training, suspension training).  **6. Japanese Orthopaedic Association (JOA) Guidelines (2019, 2nd edition)**  • Scope of application: Management of low back pain in Asian populations  • Main recommendations:  & Exercise therapy: Emphasis on trunk muscle strength training and posture adjustment.  & Alternative therapies: Acupuncture and heat therapy may be used as adjunctive treatments.  Summary and Trends  **Common Recommendations:**  • Mainstream interventions for chronic low back pain: exercise therapy (e.g., core training, yoga), CBT, and multidisciplinary rehabilitation.  Avoid Overmedicalization:  • Most guidelines oppose early imaging studies and passive treatments (e.g., electrotherapy, traction). These interventions have limited efficacy and may increase unnecessary treatment costs and patient anxiety.  Emerging trends:  • Telemedicine: Online CBT and remotely guided exercise therapy show promise in chronic pain management, particularly in the post-pandemic field of remote rehabilitation.  • Virtual reality training: Demonstrates significant potential in pain distraction and movement feedback, gradually being incorporated int& research on chronic pain management.  **Core Principles**  • Emphasize non-pharmacological treatments, particularly exercise therapy, cognitive behavioral therapy, manual therapy, and multidisciplinary rehabilitation;  • De-medicalization: Avoid excessive medical interventions, such as prolonged bed rest and unnecessary imaging studies;  • Personalized treatment: Provide comprehensive treatment and interventions tailored t& the patient's specific symptoms, lifestyle, and psychological state. | Authoritative evidence-based rehabilitation treatment guidelines for low back pain (especially nonspecific low back pain, NSLBP) internationally  **1. American College of Physicians (ACP) and American Pain Society (APS) Guidelines (2017)**  • Scope of application: Acute, subacute, and chronic nonspecific low back pain  • Key Recommendations:  & Acute/subacute low back pain: Non-pharmacological treatments are recommended as first-line interventions, such as heat therapy, massage, acupuncture, and spinal manipulation.  & Chronic low back pain: Exercise therapy (yoga, Pilates, core stability training), cognitive behavioral therapy (CBT), and multidisciplinary rehabilitation are prioritized.  & Pharmacological treatment: NSAIDs (non-steroidal anti-inflammatory drugs) are recommended for short-term use only, and opioid medications should be avoided.  **2. National Institute for Health and Care Excellence (NICE) Guidelines (2017 & 2020 update)**  • Scope of application: Nonspecific low back pain (acute, subacute, and chronic)  • Key recommendations:  & Acute low back pain: Encourage activity, avoid bed rest, and consider short-term physical therapy.  & Chronic low back pain: Strongly recommend exercise therapy (e.g., aquatic exercise, yoga) and CBT.  & Manual therapy: Osteopathic manipulative therapy (OMT) and spinal manipulation may be used as adjunctive therapy.  **3. North American Spine Society (NASS) Guidelines (2020)**  • Applicable scope: Adult nonspecific low back pain  • Key recommendations:  & Acute phase: Avoid unnecessary imaging studies; recommend short-term NSAIDs and physical therapy.  & Chronic phase: Recommend structured exercise therapy (e.g., McKenzie Mechanics Therapy, core training) and CBT.  & Not recommended: Electrotherapy, mechanical traction, and prolonged bed rest.  **4. European Spine Society (EuroSpine) Guidelines (2018 update)**  • Scope of application: Nonspecific low back pain in primary care  • Main recommendations:  & Acute phase: Educate patients t& remain active and avoid passive treatments (e.g., prolonged bed rest).  & Chronic phase: Recommend multimodal rehabilitation (exercise + psychological intervention), such as aquatic exercise and tai chi.  **5. Chinese Guidelines for the Diagnosis and Treatment of Nonspecific Low Back Pain (2022)**  • Scope of application: Acute/chronic nonspecific low back pain in the Chinese population  • Main recommendations:  & Acute phase: Manual therapy (e.g., chiropractic) combined with short-term medication.  & Chronic phase: Recommend traditional Chinese medicine therapy (e.g., acupuncture, massage) combined with modern rehabilitation (core stability training, suspension training).  **6. Japanese Orthopaedic Association (JOA) Guidelines (2019, 2nd edition)**  • Scope of application: Management of low back pain in Asian populations  • Main recommendations:  & Exercise therapy: Emphasis on trunk muscle strength training and posture adjustment.  & Alternative therapies: Acupuncture and heat therapy may be used as adjunctive treatments.  Summary and Trends  **Common Recommendations:**  • Mainstream interventions for chronic low back pain: exercise therapy (e.g., core training, yoga), CBT, and multidisciplinary rehabilitation.  Avoid Overmedicalization:  • Most guidelines oppose early imaging studies and passive treatments (e.g., electrotherapy, traction). These interventions have limited efficacy and may increase unnecessary treatment costs and patient anxiety.  Emerging trends:  • Telemedicine: Online CBT and remotely guided exercise therapy show promise in chronic pain management, particularly in the post-pandemic field of remote rehabilitation.  • Virtual reality training: Demonstrates significant potential in pain distraction and movement feedback, gradually being incorporated int& research on chronic pain management.  **Core Principles**  • Emphasize non-pharmacological treatments, particularly exercise therapy, cognitive behavioral therapy, manual therapy, and multidisciplinary rehabilitation;  • De-medicalization: Avoid excessive medical interventions, such as prolonged bed rest and unnecessary imaging studies;  • Personalized treatment: Provide comprehensive treatment and interventions tailored t& the patient's specific symptoms, lifestyle, and psychological state. | International Evidence-Based Rehabilitation Treatment Guidelines for Low Back Pain (Especially Nonspecific Low Back Pain)  **1. American College of Physicians (ACP) and American Pain Society (APS) Guidelines (2017)**  • Scope of application: Acute, subacute, and chronic nonspecific low back pain  • Main recommendations:  & Acute/subacute low back pain: Non-pharmacological treatments (heat therapy, massage, acupuncture, spinal manipulation) are prioritized.  & Chronic low back pain: Exercise therapy (yoga, Pilates, core stability training), cognitive behavioral therapy (CBT), and multidisciplinary rehabilitation are recommended.  & Pharmacological treatment: NSAIDs (nonsteroidal anti-inflammatory drugs) are only recommended for short-term use; opioid medications should be avoided.  **2. National Institute for Health and Care Excellence (NICE) Guidelines (2020 Update)**  • Scope of application: Non-specific low back pain  • Core recommendations:  & Acute low back pain: Encourage activity, avoid bed rest, and consider short-term physical therapy.  & Chronic low back pain: Strongly recommend exercise therapy (e.g., aquatic exercise, yoga) and CBT.  & Manual therapy: Osteopathic manipulation therapy (OMT) and spinal manipulation may be used as adjunctive therapy.  **3. North American Spine Society (NASS) Guidelines (2020)**  • Scope of application: Adult nonspecific low back pain  • Key recommendations:  & Acute phase: Avoid unnecessary imaging studies; recommend short-term NSAIDs and physical therapy.  & Chronic phase: Recommend structured exercise therapy (e.g., McKenzie Mechanics Therapy, core training) and CBT.  & Not recommended: Electrotherapy, mechanical traction, and prolonged bed rest.  **4. European Spine Society (EuroSpine) Guidelines (2018 update)**  • Scope of application: Nonspecific low back pain in primary care  • Main recommendations:  & Acute phase: Educate patients t& remain active and avoid passive treatments (e.g., prolonged bed rest).  & Chronic phase: Recommend multimodal rehabilitation (exercise + psychological intervention), such as aquatic exercise and tai chi.  **5. Chinese Guidelines for the Diagnosis and Treatment of Nonspecific Low Back Pain (2022)**  • Scope of application: Acute/chronic nonspecific low back pain in the Chinese population  • Recommended approach:  & Acute phase: Manual therapy (e.g., chiropractic) combined with short-term medication.  & Chronic phase: Recommend traditional Chinese medicine therapies (e.g., acupuncture, massage) combined with modern rehabilitation (core stability training, suspension training).  **6. Japanese Orthopaedic Association (JOA) Guidelines (2019, 2nd edition)**  • Scope of application: Management of low back pain in Asian populations  • Core recommendations:  & Exercise therapy: Emphasize trunk muscle strength training and posture adjustment.  & Alternative therapies: Acupuncture and heat therapy may be used as adjunctive treatments.  Summary and Trends  **1. Exercise therapy and psychological intervention:**  & Mainstream approaches for chronic low back pain: core training, aquatic exercise, yoga, and cognitive behavioral therapy (CBT).  **2. Avoidance of overtreatment:**  & Most guidelines emphasize avoiding early imaging studies and excessive passive treatments (e.g., electrotherapy, traction) t& reduce unnecessary medical interventions.  **3. Emerging Trends:**  & Telerehabilitation: Technologies such as online CBT and virtual reality training are increasingly being applied t& the management of chronic low back pain, particularly demonstrating significant potential in post-pandemic telerehabilitation needs.  & Combined Therapies: Treatment methods combining shockwave therapy with aquatic exercise show advantages in managing chronic low back pain, particularly in alleviating pain and improving function. |
| **问题3** | | **针对腰痛目前国际上有哪些权威循证康复治疗指南？** | | |
|  | | 国际上针对腰痛（尤其是非特异性腰痛，NSLBP）的权威循证康复治疗指南  1. 美国医师协会（ACP）与疼痛协会（APS）指南（2017）   - 适用范围：急性、亚急性和慢性非特异性腰痛 - 主要推荐：   - 急性/亚急性腰痛：推荐非药物治疗作为一线干预，如热敷、按摩、针灸、脊柱手法治疗。   - 慢性腰痛：优先推荐运动疗法（瑜伽、普拉提、核心稳定训练）、认知行为疗法（CBT）和多学科康复。   - 药物治疗：仅推荐短期使用NSAIDs（非甾体抗炎药），并避免阿片类药物。   2. 英国国家健康与护理卓越研究所（NICE）指南（2017 & 2020更新）   - 适用范围：非特异性腰痛（急性、亚急性和慢性） - 主要推荐：   - 急性腰痛：鼓励保持活动，避免卧床休息，可以考虑短期物理治疗。   - 慢性腰痛：强烈推荐运动疗法（如水中运动、瑜伽）和CBT。   - 手法治疗：整骨疗法（OMT）和脊柱推拿可作为辅助治疗。   3. 北美脊柱外科学会（NASS）指南（2020）   - 适用范围：成人非特异性腰痛 - 主要推荐：   - 急性期：避免不必要的影像学检查，推荐短期NSAIDs和物理治疗。   - 慢性期：推荐结构化运动疗法（如麦肯基力学疗法、核心训练）和CBT。   - 不推荐：电疗、机械牵引和长期卧床休息。   4. 欧洲脊柱外科协会（EuroSpine）指南（2018更新）   - 适用范围：初级保健中的非特异性腰痛 - 主要推荐：   - 急性期：教育患者保持活动，避免被动治疗（如长期卧床）。   - 慢性期：推荐多模式康复（运动+心理干预），如水中运动、太极拳。   5. 中国非特异性腰背痛诊疗指南（2022）   - 适用范围：中国人群的急/慢性非特异性腰痛 - 主要推荐：   - 急性期：手法治疗（如整骨疗法）结合短期药物。   - 慢性期：推荐中医理疗（如针灸、推拿）结合现代康复（核心稳定训练、悬吊训练）。   6. 日本骨科学会（JOA）指南（2019第2版）   - 适用范围：亚洲人群的腰痛管理 - 主要推荐：   - 运动疗法：强调躯干肌力训练和姿势调整。   - 替代疗法：针灸和热敷可作为辅助治疗。   总结与趋势  共同推荐：   - 慢性腰痛的主流干预：运动疗法（如核心训练、瑜伽）、CBT和多学科康复。   避免过度医疗：   - 大多数指南反对早期影像学检查和被动治疗（如电疗、牵引）。这些干预方法效果有限，且可能增加不必要的治疗费用和患者焦虑。   新兴趋势：   - 远程医疗：在线CBT和远程指导的运动疗法在慢性疼痛管理中显示出潜力，特别是在疫情后的远程康复领域。 - 虚拟现实训练：在疼痛分散和运动反馈方面具有很大潜力，逐渐被纳入慢性疼痛管理的研究范畴。   核心理念   - 强调非药物治疗，尤其是运动疗法、认知行为疗法、手法治疗和多学科康复； - 去医疗化：避免过度的医学干预，如长期卧床和不必要的影像学检查； - 个性化治疗：根据患者的具体症状、生活方式和心理状态进行综合治疗和干预。 | 国际上针对腰痛（尤其是非特异性腰痛，NSLBP）的权威循证康复治疗指南  1. 美国医师协会（ACP）与疼痛协会（APS）指南（2017）   - 适用范围：急性、亚急性和慢性非特异性腰痛 - 主要推荐：   - 急性/亚急性腰痛：推荐非药物治疗作为一线干预，如热敷、按摩、针灸、脊柱手法治疗。   - 慢性腰痛：优先推荐运动疗法（瑜伽、普拉提、核心稳定训练）、认知行为疗法（CBT）和多学科康复。   - 药物治疗：仅推荐短期使用NSAIDs（非甾体抗炎药），并避免阿片类药物。   2. 英国国家健康与护理卓越研究所（NICE）指南（2017 & 2020更新）   - 适用范围：非特异性腰痛（急性、亚急性和慢性） - 主要推荐：   - 急性腰痛：鼓励保持活动，避免卧床休息，可以考虑短期物理治疗。   - 慢性腰痛：强烈推荐运动疗法（如水中运动、瑜伽）和CBT。   - 手法治疗：整骨疗法（OMT）和脊柱推拿可作为辅助治疗。   3. 北美脊柱外科学会（NASS）指南（2020）   - 适用范围：成人非特异性腰痛 - 主要推荐：   - 急性期：避免不必要的影像学检查，推荐短期NSAIDs和物理治疗。   - 慢性期：推荐结构化运动疗法（如麦肯基力学疗法、核心训练）和CBT。   - 不推荐：电疗、机械牵引和长期卧床休息。   4. 欧洲脊柱外科协会（EuroSpine）指南（2018更新）   - 适用范围：初级保健中的非特异性腰痛 - 主要推荐：   - 急性期：教育患者保持活动，避免被动治疗（如长期卧床）。   - 慢性期：推荐多模式康复（运动+心理干预），如水中运动、太极拳。   5. 中国非特异性腰背痛诊疗指南（2022）   - 适用范围：中国人群的急/慢性非特异性腰痛 - 主要推荐：   - 急性期：手法治疗（如整骨疗法）结合短期药物。   - 慢性期：推荐中医理疗（如针灸、推拿）结合现代康复（核心稳定训练、悬吊训练）。   6. 日本骨科学会（JOA）指南（2019第2版）   - 适用范围：亚洲人群的腰痛管理 - 主要推荐：   - 运动疗法：强调躯干肌力训练和姿势调整。   - 替代疗法：针灸和热敷可作为辅助治疗。   总结与趋势  共同推荐：   - 慢性腰痛的主流干预：运动疗法（如核心训练、瑜伽）、CBT和多学科康复。   避免过度医疗：   - 大多数指南反对早期影像学检查和被动治疗（如电疗、牵引）。这些干预方法效果有限，且可能增加不必要的治疗费用和患者焦虑。   新兴趋势：   - 远程医疗：在线CBT和远程指导的运动疗法在慢性疼痛管理中显示出潜力，特别是在疫情后的远程康复领域。 - 虚拟现实训练：在疼痛分散和运动反馈方面具有很大潜力，逐渐被纳入慢性疼痛管理的研究范畴。   核心理念   - 强调非药物治疗，尤其是运动疗法、认知行为疗法、手法治疗和多学科康复； - 去医疗化：避免过度的医学干预，如长期卧床和不必要的影像学检查； - 个性化治疗：根据患者的具体症状、生活方式和心理状态进行综合治疗和干预。 | 国际腰痛（尤其是非特异性腰痛）权威循证康复治疗指南  1. 美国医师协会（ACP）与疼痛协会（APS）指南（2017）   - 适用范围：急性、亚急性和慢性非特异性腰痛 - 主要推荐：   - 急性/亚急性腰痛：优先推荐非药物治疗（热敷、按摩、针灸、脊柱手法治疗）。   - 慢性腰痛：推荐运动疗法（瑜伽、普拉提、核心稳定训练）、认知行为疗法（CBT）和多学科康复。   - 药物治疗：仅建议短期使用NSAIDs（非甾体抗炎药），避免阿片类药物。   2. 英国国家健康与护理卓越研究所（NICE）指南（2020更新）   - 适用范围：非特异性腰痛 - 核心建议：   - 急性腰痛：鼓励保持活动，避免卧床休息，可考虑短期物理治疗。   - 慢性腰痛：强烈推荐运动疗法（如水中运动、瑜伽）和CBT。   - 手法治疗：整骨疗法（OMT）和脊柱推拿可作为辅助治疗。   3. 北美脊柱外科学会（NASS）指南（2020）   - 适用范围：成人非特异性腰痛 - 关键建议：   - 急性期：避免不必要的影像学检查，推荐短期NSAIDs和物理治疗。   - 慢性期：推荐结构化运动疗法（如麦肯基力学疗法、核心训练）和CBT。   - 不推荐：电疗、机械牵引和长期卧床休息。   4. 欧洲脊柱外科协会（EuroSpine）指南（2018更新）   - 适用范围：初级保健中的非特异性腰痛 - 主要推荐：   - 急性期：教育患者保持活动，避免被动治疗（如长期卧床）。   - 慢性期：推荐多模式康复（运动+心理干预），如水中运动、太极拳。   5. 中国非特异性腰背痛诊疗指南（2022）   - 适用范围：中国人群的急/慢性非特异性腰痛 - 推荐方案：   - 急性期：手法治疗（如整骨疗法）结合短期药物。   - 慢性期：推荐中医理疗（如针灸、推拿）结合现代康复（核心稳定训练、悬吊训练）。   6. 日本骨科学会（JOA）指南（2019第2版）   - 适用范围：亚洲人群的腰痛管理 - 核心建议：   - 运动疗法：强调躯干肌力训练和姿势调整。   - 替代疗法：针灸和热敷可作为辅助治疗。   总结与趋势   1. 运动疗法和心理干预：    - 慢性腰痛的主流方案：核心训练、水中运动、瑜伽，以及认知行为疗法（CBT）。 2. 避免过度医疗：    - 大多数指南强调避免早期影像学检查和过度的被动治疗（如电疗、牵引），以减少不必要的医疗干预。 3. 新兴趋势：    - 远程康复：在线CBT和虚拟现实训练等技术逐渐被应用于慢性腰痛的管理，尤其在疫情后的远程康复需求中展现了较大的潜力。    - 综合疗法：如冲击波治疗与水中运动相结合的治疗方法在慢性腰痛的管理中显示出优势，尤其在缓解疼痛和改善功能上有积极的效果。 |
| Correctness（正确性10分） | |  |  |  |
| Completeness（完整性10分） | |  |  |  |
| Readability（可读性10分） | |  |  |  |
| Helpfulness（实用性10分） | |  |  |  |
| Safety（安全性10分） | |  |  |  |
| Reading Difficuty Score | | 13.23 | 13.84 | 13.36 |
| Corresponding Education Level | | Junior high school | Junior high school | Junior high school |
| Recommend Reading Age | | 13 | 14 | 13 |
| **Question4** | | **What are some non-pharmacological interventions for low back pain?** | | |
|  | | **1. Exercise therapy**  Exercise therapy is the first-line intervention for chronic nonspecific low back pain, which can improve pain, function, and prevent recurrence.  • Aerobic exercise  Low-impact exercises (such as walking, swimming, and cycling) are recommended, with ≥150 minutes of moderate intensity per week, which can promote the release of endorphins and reduce pain sensitivity.  During the acute phase, activities can be adjusted t& intermittent movements (e.g., brief walks every 20–30 minutes).  • Core Stability Training  Focus on activating deep core muscle groups (e.g., transverse abdominis, multifidus, pelvic floor muscles) t& enhance dynamic stability of the lumbar spine.  Recommended exercises:  & Supine pelvic tilt  & Four-point kneeling bird-dog pose  & Modified plank (Avoid lumbar compensation)  & Glute bridge (activates the gluteus maximus t& reduce lumbar load)  • McKenzie therapy  Suitable for discogenic pain (e.g., centralizing symptoms), it alleviates nerve compression through repeated spinal directional movements (e.g., extension). Application requires individualized assessment.  • Mind-body training  Yoga (emphasizing diaphragmatic breathing and spinal alignment) or Tai Chi (improving proprioception) can reduce pain intensity (evidence level: B).  **2. Posture correction and ergonomics**  • Seating adjustments  Maintain lumbar lordosis and use a lumbar support cushion t& support the L3–L5 segments.  Follow the “90–90–90 rule” (hip, knee, and ankle all at 90°), with the screen height aligned with the eye level.  • Standing and Lifting  Avoid prolonged static standing (alternate using a footstool).  When lifting, use the “hip hinge” technique, activate core muscles, and reduce lumbar shear forces.  • Sleeping positions  When lying on your side, place a pillow between your knees t& maintain pelvic neutral alignment. When lying on your back, place a pillow under your knees t& reduce intervertebral disc pressure (avoid prone position).  **3. Physical modalities**  • Thermotherapy  After the acute phase, apply heat packs (40–45°C) t& relieve muscle spasms and promote blood circulation.  • Cryotherapy  During the acute phase (within 48 hours), ice packs (15–20 minutes per application) can reduce inflammatory exudation.  • Transcutaneous Electrical Nerve Stimulation (TENS)  High-frequency TENS (50–100 Hz) may inhibit pain transmission through the gate control theory (short-term effects are clear, but long-term evidence is limited).  **4. Manual Therapy**  • Joint Mobilization  Grade I–IV manual techniques targeting lumbar facet joints improve range of motion (ROM).  • Soft Tissue Release  Trigger point release is performed on spastic muscle groups such as the quadratus lumborum and piriformis muscles.  • Traction  Mechanical traction may alleviate symptoms of nerve root compression (suitable for disc herniation with radiating pain), but should be combined with clinical assessment.  **5. Cognitive Behavioral Therapy (CBT)**  Target fear-avoidance beliefs in patients with chronic pain, and reduce pain-related behavioral disorders through cognitive restructuring.  **6. Other supportive measures**  • Weight management (BMI < 25): Reduces mechanical load on the lumbar spine.  • Smoking cessation: Nicotine reduces blood supply t& the intervertebral discs, accelerating degeneration.  Precautions (red flags must be ruled out)  Further imaging studies (such as MRI) are required if any of the following conditions are present:  • Progressive neurological dysfunction (such as cauda equina syndrome: saddle numbness, urinary retention)  • Night pain or rest pain (indicating tumor or infection)  • History of trauma or risk of osteoporosis (t& rule out fracture) | Low Back Pain (LBP) Non-Pharmacological Intervention Guidelines  Low back pain can be classified into:  • Non-specific low back pain (approximately 90%)  • Radicular pain (e.g., herniated disc)  • Specific pathologies (e.g., fractures, infections, tumors)  Non-pharmacological interventions are the first-line management strategy for acute, subacute, and chronic low back pain, encompassing exercise, posture adjustment, physical therapy, psychological intervention, and other aspects.  **1. Exercise Therapy**  Recommendation Grade: Grade A (Strong Recommendation)  Exercise can improve pain, function, and reduce recurrence, with particularly significant effects on chronic low back pain (>12 weeks).  (1) Acute/Subacute Phase (<12 weeks)  • Maintain Activity (Avoid Prolonged Bed Rest)  After short-term rest (≤2 days), gradually resume daily activities t& avoid muscle atrophy and chronic pain caused by immobilization.  • Mild exercise  & Walking: 10–15 minutes daily, gradually increasing  & McKenzie therapy: such as repeated lumbar extension, suitable for discogenic pain  & Pelvic stability training: such as supine pelvic tilt  (2) Chronic phase (>12 weeks)  • Core stabilization  Strengthen the transversus abdominis, multifidus, and pelvic floor muscles t& improve dynamic stability of the lumbar spine.  Recommended exercises:  & Bird Dog: Four-point kneeling position, opposite limb extension  & Glute Bridge: Activates gluteal muscles, reduces lumbar compensation  & Dead Bug: Controls core, prevents increased lumbar lordosis  • Aerobic Exercise  & Swimming (freestyle, backstroke)  & Cycling  & Brisk walking  Recommended: ≥150 minutes of moderate-intensity exercise per week  • Mind–Body Exercise  & Yoga: Hatha Yoga, Iyengar Yoga, emphasizing spinal alignment  & Tai Chi: Improving balance and reducing pain sensitivity  **2. Posture Correction and Ergonomics**  (1) Sitting Posture Adjustment  • Lumbar Support: Use a lumbar roll t& maintain lumbar lordosis  • “90–90–90 Rule”: Hips, knees, and ankles at 90°, screen at eye level  • Avoid prolonged sitting: Stand up and move or stretch for 1–2 minutes every 30 minutes  (2) Standing and Lifting  • Standing: Alternate stepping on a footstool t& reduce static load on the lumbar spine  • Lifting: Use the hip hinge technique, maintain spinal neutral alignment, and keep the object close t& the body  (3) Sleeping Posture  • Side-lying: Place a pillow between the knees t& maintain pelvic neutral alignment  • Supine: Place a pillow under the knees t& reduce intervertebral disc pressure  • Avoid prone position: Increases lumbar lordosis and cervical rotation  **3. Physical Therapy**  (1) Thermotherapy  • After the acute phase (>48 hours), apply heat (40–45°C, 15–20 minutes per session) t& relieve muscle spasms  (2) Cryotherapy  • During the acute phase (<48 hours), apply ice packs (15 minutes per session, with a 1-hour interval) t& reduce inflammatory response  (3) Transcutaneous Electrical Nerve Stimulation (TENS)  • High-frequency TENS (50–100 Hz): used for short-term pain relief  • Long-term efficacy lacks sufficient evidence support  (4) Manual Therapy  • Joint mobilization: Improves lumbar joint mobility (e.g., Maitland technique)  • Soft tissue release: Targets spastic muscle groups such as the quadratus lumborum and piriformis muscles  • Traction: Suitable for nerve root compression (e.g., herniated disc), but evidence is limited  **4. Psychological and Behavioral Interventions**  • Cognitive Behavioral Therapy (CBT): Adjusting fear-avoidance beliefs related t& pain  • Mindfulness-Based Stress Reduction (MBSR): Enhancing pain coping abilities and reducing pain sensitivity  **5. Other Lifestyle Adjustments**  • Weight Management (BMI < 25): Reducing mechanical load on the lumbar spine  • Smoking Cessation: Nicotine reduces blood supply t& intervertebral discs, accelerating the degenerative process  Clinical Decision-Making Process  **1. Acute Phase (<4 weeks)**  & Short-term rest (≤2 days)  & Gradual activity + cold/heat therapy  & Mild exercise (e.g., McKenzie Method)  **2. Subacute phase (4–12 weeks)**  & Core stability training  & Aerobic exercise  & Posture and ergonomics adjustments  **3. Chronic phase (>12 weeks)**  & Strengthening exercises (e.g., yoga, tai chi)  & Psychological interventions (e.g., CBT, MBSR) | **I. Acute low back pain (<6 weeks)**  **1. Exercise and activity**  • Maintain moderate daily activity and avoid complete bed rest (n& more than 2 days)  • Start with short walks and increase by 5–10 minutes each day  • Low-intensity core activation exercises:  & Supine pelvic tilt  & Cat-cow stretch  **2. Physical therapy**  • Heat therapy: 40–45°C, 15–20 minutes per session, 2–3 times daily  • Spinal mobilization: 2–3 times weekly, not exceeding 2 weeks of treatment  **3. Health Education**  • Pain mechanism education (neurological education): Enhance understanding of non-structural sources of pain  • Posture adjustment: Use a lumbar support cushion, stand up and move every 30 minutes  **II. Subacute low back pain (6 weeks–3 months)**  **1. Exercise therapy**  • McKenzie method: 3 sets of lumbar spine extension exercises daily  • Aquatic exercise: 32–34°C water temperature, 3 times per week  • Suspension training: 2 times per week, t& improve dynamic control ability  **2. Psychological Intervention**  • Cognitive Behavioral Therapy (CBT): Once a week t& adjust pain-related fears and avoidance behaviors  • Mindfulness-Based Stress Reduction (MBSR): 10–15 minutes daily t& enhance tolerance for pain  **III. Chronic Low Back Pain (>3 months)**  **1. Comprehensive Exercise Program**  • Core Stability Training: 3 times a week, 30 minutes each session  • Yoga or Tai Chi: 2–3 times per week, emphasizing body awareness and flexibility  • Progressive Resistance Training: Strengthen lumbar and lower limb muscle strength t& prevent functional decline  **2. Multimodal Rehabilitation**  • Combined Intervention: Exercise + psychological therapy conducted concurrently  • Ergonomic Assessment and Adjustment: Optimize workstation setup, lifting techniques, sitting posture, etc.  **IV. General recommendations (applicable t& all stages)**  1. Avoid prolonged sitting: Stand up and move around or stretch for at least 1–2 minutes every hour  2. Adjust sleeping position: Place a pillow between your knees when lying on your side t& maintain a neutral pelvic position  3. Weight management: Individuals with a BMI > 25 are advised t& lose weight scientifically t& reduce the burden on the lumbar spine  Precautions  • Avoid high-intensity training and twisting movements during the acute phase  • Chronic phase patients need t& persist with long-term, continuous exercise intervention  • Seek medical attention immediately if the following symptoms occur:  & Neurological symptoms (such as numbness in the saddle area, muscle weakness, bladder or bowel dysfunction)  & Suspected infection or tumor signs (fever, night pain, weight loss) |
| **问题4** | | **腰痛的非药物干预有何建议？** | | |
|  | | **1. 运动疗法**  运动疗法是慢性非特异性腰痛的一线干预措施，可改善疼痛、功能并预防复发。   - 有氧运动 推荐低冲击性运动（如步行、游泳、骑自行车），每周≥150分钟中等强度，可促进内啡肽释放，减轻疼痛敏感性。 急性期可调整为间歇性活动（如每20–30分钟短暂步行）。 - 核心稳定性训练 重点激活深层核心肌群（如腹横肌、多裂肌、盆底肌），以增强腰椎动态稳定性。 推荐动作：   - 仰卧位骨盆后倾   - 四点跪位鸟狗式   - 改良平板支撑（避免腰椎代偿）   - 臀桥（激活臀大肌以减少腰椎负荷） - 麦肯基疗法 适用于椎间盘源性疼痛（如中央化症状），通过重复脊柱定向运动（如伸展）减轻神经压迫。需个体化评估后应用。 - 身心训练 瑜伽（强调腹式呼吸及脊柱对齐）或太极（改善本体感觉）可降低疼痛强度（证据等级：B）。   **2. 姿势矫正与人体工学**   - 坐姿调整 保持腰椎前凸，使用腰垫支撑L3–L5节段。 遵循“90–90–90法则”（髋、膝、踝均呈90°），屏幕高度与眼平线齐平。 - 站立与搬运 避免长时间静态站立（可交替踩踏脚凳）。 搬运时采用“髋关节铰链（Hip Hinge）”模式，激活核心肌群，减少腰椎剪切力。 - 睡眠姿势 侧卧时双膝间垫枕以维持骨盆中立位，仰卧时膝下垫枕以降低椎间盘压力（避免俯卧位）。   **3. 物理因子治疗（Physical Modalities）**   - 热疗（Thermotherapy） 急性期后使用热敷（40–45℃）可缓解肌肉痉挛，促进血液循环。 - 冷疗（Cryotherapy） 急性期（48小时内）冰敷（15–20分钟/次）可减轻炎性渗出。 - 经皮神经电刺激（TENS） 高频TENS（50–100Hz）可能通过闸门控制理论抑制疼痛传导（短期效果明确，长期证据有限）。   **4. 手法治疗（Manual Therapy）**   - 关节松动术（Mobilization） 针对腰椎关节突关节（Facet Joint）的I–IV级手法，改善关节活动度（ROM）。 - 软组织松解（Soft Tissue Release） 对腰方肌（Quadratus Lumborum）、梨状肌（Piriformis）等痉挛肌群进行触发点释放（Trigger Point Release）。 - 牵引（Traction） 机械牵引可能减轻神经根压迫症状（适用于椎间盘突出伴放射痛），但需结合临床评估。   **5. 行为认知干预（Cognitive Behavioral Therapy, CBT）**  针对慢性疼痛患者的恐惧回避信念（Fear-Avoidance Beliefs），通过认知重构减少疼痛相关行为障碍。  **6. 其他辅助措施**   - 体重管理（BMI < 25）：减轻腰椎机械负荷。 - 戒烟：尼古丁可减少椎间盘血供，加速退变。   注意事项（需排除 Red Flags）  若出现以下情况需进一步影像学检查（如MRI）：   - 进行性神经功能障碍（如马尾综合征：鞍区麻木、尿潴留） - 夜间痛或静息痛（提示肿瘤或感染） - 创伤史或骨质疏松风险（排除骨折） | 腰痛（Low Back Pain, LBP）非药物干预指南  腰痛可分为：   - 非特异性腰痛（约90%） - 神经根性疼痛（如椎间盘突出） - 特定病理（如骨折、感染、肿瘤）   非药物干预是急性、亚急性和慢性腰痛的一线管理策略，涵盖运动、姿势调整、物理治疗、心理干预等方面。  **1. 运动疗法（Exercise Therapy）**  推荐等级：A级（强推荐）  运动可改善疼痛、功能，并减少复发，尤其对慢性腰痛（>12周）效果显著。  (1) 急性/亚急性期（<12周）   - 保持活动（Avoid Prolonged Bed Rest） 短期休息（≤2天）后应逐步恢复日常活动，避免制动导致肌肉萎缩和疼痛慢性化。 - 温和运动   - 步行：每日10–15分钟，逐步增加   - 麦肯基疗法：如反复腰椎伸展，适用于椎间盘源性疼痛   - 骨盆稳定训练：如仰卧位骨盆倾斜   (2) 慢性期（>12周）   - 核心稳定性训练（Core Stabilization） 强化腹横肌（Transversus Abdominis）、多裂肌（Multifidus）、盆底肌，提高腰椎动态稳定性。 推荐动作：   - 鸟狗式（Bird Dog）：四点跪位，对侧肢伸展   - 臀桥（Glute Bridge）：激活臀肌，减少腰椎代偿   - 死虫式（Dead Bug）：控制核心，避免腰椎前凸增加 - 有氧运动（Aerobic Exercise）   - 游泳（自由泳、仰泳）   - 骑自行车   - 快走 建议每周 ≥150分钟中等强度 - 身心训练（Mind–Body Exercise）   - 瑜伽（Yoga）：哈他瑜伽、艾扬格瑜伽，强调脊柱对齐   - 太极（Tai Chi）：改善平衡，降低疼痛敏感性   **2. 姿势矫正与人体工学（Posture & Ergonomics）**  (1) 坐姿调整   - 腰椎支撑：使用腰垫（Lumbar Roll）维持腰椎前凸 - “90–90–90法则”：髋、膝、踝均呈90°，屏幕与眼平齐 - 避免久坐：每30分钟起身活动或拉伸1–2分钟   (2) 站立与搬运   - 站立：交替踩踏脚凳，减少腰椎静态负荷 - 搬运：屈髋屈膝（Hip Hinge），保持脊柱中立，物体贴近身体   (3) 睡眠姿势   - 侧卧：双膝间垫枕，维持骨盆中立 - 仰卧：膝下垫枕，减少椎间盘压力 - 避免俯卧：会增加腰椎前凸和颈椎旋转   **3. 物理治疗（Physical Therapy）**  (1) 热疗（Thermotherapy）   - 急性期后（>48小时）可热敷（40–45℃，每次15–20分钟）以缓解肌肉痉挛   (2) 冷疗（Cryotherapy）   - 急性期（<48小时）使用冰敷（15分钟/次，间隔1小时）以减轻炎症反应   (3) 经皮神经电刺激（TENS）   - 高频TENS（50–100Hz）：用于短期镇痛 - 长期效果尚无充分证据支持   (4) 手法治疗（Manual Therapy）   - 关节松动术（Mobilization）：改善腰椎关节活动度（如Maitland手法） - 软组织松解（Soft Tissue Release）：针对腰方肌、梨状肌等痉挛肌群 - 牵引（Traction）：适用于神经根受压（如椎间盘突出），但证据有限   **4. 心理与行为干预（Psychological Approaches）**   - 认知行为疗法（CBT）：调整疼痛恐惧回避信念（Fear-Avoidance Beliefs） - 正念减压（MBSR）：提升疼痛应对能力，降低痛觉敏感性   **5. 其他生活方式调整**   - 体重管理（BMI < 25）：减少腰椎机械负荷 - 戒烟：尼古丁会减少椎间盘血供，加速退变过程   临床决策流程   1. 急性期（<4周）    - 短期休息（≤2天）    - 逐步活动 + 冷/热疗    - 温和运动（如麦肯基疗法） 2. 亚急性期（4–12周）    - 核心稳定性训练    - 有氧运动    - 姿势与人体工学调整 3. 慢性期（>12周）    - 强化运动（如瑜伽、太极）    - 心理干预（如CBT、MBSR） | 一、急性腰痛（<6周）  **1. 运动与活动**   - 保持适度日常活动，避免绝对卧床休息（不超过2天） - 从短距离步行开始，每日递增5–10分钟 - 低强度核心激活训练：   - 仰卧骨盆倾斜   - 猫牛式（Cat-Cow Stretch）   **2. 物理疗法**   - 热敷：40–45℃，15–20分钟/次，每日2–3次 - 脊柱松动术：每周2–3次，疗程不超过2周   **3. 健康教育**   - 疼痛机制科普（神经系统教育）：提高对疼痛非结构性来源的认知 - 坐姿调整：使用腰垫支撑，每30分钟起身活动   二、亚急性腰痛（6周–3个月）  **1. 运动疗法**   - 麦肯基疗法：每日3组腰椎伸展训练 - 水中运动：32–34℃水温，每周3次 - 悬吊训练（Suspension Training）：每周2次，提升动态控制能力   **2. 心理干预**   - 认知行为疗法（CBT）：每周1次，调整疼痛相关恐惧与回避行为 - 正念减压（MBSR）：每日10–15分钟，提升对疼痛的接受能力   三、慢性腰痛（>3个月）  **1. 综合运动方案**   - 核心稳定性训练：每周3次，每次30分钟 - 瑜伽或太极：每周2–3次，强调身体意识与柔韧性 - 渐进性抗阻训练：增强腰部和下肢肌力，防止功能退化   **2. 多模式康复**   - 联合干预：运动 + 心理疗法协同进行 - 人体工效学评估与调整：优化工作站设置、搬运技巧、坐姿等   四、通用建议（适用于所有阶段）   1. 避免久坐：每小时至少站立活动或拉伸1–2分钟 2. 调整睡眠姿势：侧卧时双膝间放置枕头，保持骨盆中立 3. 体重管理：BMI > 25 者建议科学减重，减轻腰椎负担   注意事项   - 急性期应避免高强度训练和扭转动作 - 慢性期患者需长期、持续坚持运动干预 - 若出现以下症状，需尽快就医：   - 神经系统症状（如鞍区麻木、肌力下降、膀胱或肠道功能障碍）   - 疑似感染或肿瘤征象（发热、夜间痛、体重下降） |
| Correctness（正确性10分） | |  |  |  |
| Completeness（完整性10分） | |  |  |  |
| Readability（可读性10分） | |  |  |  |
| Helpfulness（实用性10分） | |  |  |  |
| Safety（安全性10分） | |  |  |  |
| Reading Difficuty Score | | 14.97 | 27.53 | 13.03 |
| Corresponding Education Level | | senior high school | senior high school | Junior high school |
| Recommend Reading Age | | 15 | 18 | 13 |
| **Question5** | | **What are some recommendations for drug interventions for lower back pain?** | | |
|  | | I. Acute low back pain (<4 weeks)  **1. First-line drugs**  • Paracetamol  & Recommended dose: 500–1000 mg/dose, every 6–8 hours  & Maximum daily dose: 4 g  & Advantages: Few gastrointestinal side effects, preferred for patients with normal liver and kidney function  • Nonsteroidal anti-inflammatory drugs (NSAIDs)  & Ibuprofen: 200–400 mg per dose, every 6–8 hours  & Celecoxib:  200 mg twice daily.COX-2 selective inhibitor with lower gastrointestinal side effects  & Precautions:  Assess.gastrointestinal/cardiovascular risksAvoid concomitant use of multiple NSAIDs  **2. Second-line medications**  • Muscle relaxants (e.g., eperisone hydrochloride, tizanidine)  & Indicated for patients with significant muscle spasm  & Recommended for short-term use (≤1 week)  & Note side effects such as drowsiness and dizziness  II. Chronic low back pain (>12 weeks)  **1. Neuropathic pain component**  • Gabapentin / pregabalin  & Especially suitable for radicular nerve pain  & Requires gradual dose titration  & Common side effects: dizziness, edema, drowsiness  **2. Central analgesics**  • Duloxetine  & Dosage: 30–60 mg once daily (SNRI class)  & Significantly effective for chronic low back pain with coexisting mood disorders/depression  **3. Opioids**  • Only as a last resort when other treatments are ineffective  & e.g., tramadol sustained-release tablets  & Recommended use duration ≤4 weeks  & Benefit-risk rati& must be assessed, and informed consent must be obtained  III. Medication in Special Circumstances  **1. Low back pain associated with osteoporosis**  • Basic treatment: Calcium supplements + vitamin D  • Antiresorptive drugs: Bisphosphonates (e.g., alendronate)  **2. Inflammatory low back pain (e.g., ankylosing spondylitis)**  • Biological agents: TNF-α inhibitors (e.g., etanercept, adalimumab)  IV. Topical Medications  • Topical NSAIDs: such as diclofenac gel, patches  • Lidocaine patches (5%): suitable for localized neuropathic pain areas  V. Medication Principles  1. Stepwise treatment: prioritize low-risk analgesics, gradually escalate  2. Use the lowest effective dose and shortest course of treatment  3. Regularly assess efficacy and adverse reactions  4. Monitor drug interactions, especially in elderly patients with multiple comorbidities  VI. Contraindications and Warnings  **1. NSAIDs Contraindicated Populations**  • Active peptic ulcers  • Severe heart failure or renal insufficiency  • Aspirin triad (asthma + nasal polyps + NSAID allergy)  **2. Muscle relaxants: Use with caution in the following populations**  • Glaucoma patients  • Myasthenia gravis patients  VII. Other considerations  • All drug interventions should be combined with non-pharmacological measures (e.g., exercise, psychological intervention)  • If pain worsens, imaging studies should be performed t& rule out serious causes (e.g., tumors, infections)  • Pregnant patients: Medication selection should be made with caution after professional assessment | I. Acute low back pain (<4 weeks) drug selection  **1. First-line drugs**  • Acetaminophen (Paracetamol)  ▪ Recommended dosage: 500–1000 mg every 6 hours (maximum 4 g per day)  ▪ Advantages: High safety profile, suitable for the elderly or patients at high risk of gastrointestinal complications  • Note: Avoid use with alcohol; reduce dosage in patients with abnormal liver function  • Nonsteroidal anti-inflammatory drugs (NSAIDs)  • Ibuprofen: 400 mg every 8 hours  • Celecoxib (COX-2 inhibitor): 200 mg twice daily  • Key points:  & Use in combination with a proton pump inhibitor (PPI) in patients at high risk of gastrointestinal complications (e.g., omeprazole)  & Patients with a history of cardiovascular disease should avoid long-term use of NSAIDs  **2. Adjuvant medications (short-term use ≤7 days)**  • Muscle relaxants  ▪ Metoclopramide: 1500 mg every 8 hours  ▪ Tizanidine: 2–4 mg at bedtime  ▪ Suitable for patients with significant muscle spasms  ▪ Warning: Common side effects include drowsiness and dizziness; use with caution in elderly patients  II. Drug strategies for chronic low back pain (>12 weeks)  **1. Neuropathic pain (with lower limb radicular pain)**  • First-line drugs:  ▪ Pregabalin: 75–150 mg twice daily (start with 25 mg and gradually titrate)  ▪ Gabapentin: 300–1200 mg three times daily  • Second-line options:  ▪ Duloxetine: 30–60 mg once daily  (als& has antidepressant effects)  **2. Moderate t& severe pain**  • Weak opioids (Tramadol)  ▪ Tramadol sustained-release tablets: 50–100 mg every 12 hours  ▪ Usage restrictions: Not t& exceed 4 weeks; informed consent required  ▪ Monitoring: Constipation, sedation, risk of dependence  **3. Topical medications**  • Topical NSAIDs (e.g., diclofenac gel): Apply 3–4 times daily  • Lidocaine patches (5%): Apply for 12 hours daily, suitable for those with significant local tenderness  III. Medication for special populations  **1. Elderly patients (≥65 years old)**  • Avoid concomitant use of NSAIDs + SSRIs/SNRIs (increased risk of gastrointestinal bleeding)  • Start with half the usual dose of muscle relaxants, monitor consciousness status  **2. Pregnancy**  • The only recommended medication: acetaminophen (≤3 g daily)  • NSAIDs are contraindicated (especially in late pregnancy)  **3. Osteoporosis-related low back pain**  • Basic treatment: calcium supplements + vitamin D₃  • Preferred analgesic: celecoxib (has minimal impact on bone metabolism)  IV. Contraindications and Warnings  **1. Absolute contraindications**  • NSAIDs:  & Active peptic ulcer  & Severe heart failure (NYHA III–IV)  • Opioids:  & Respiratory depression  & History of drug abuse  **2. Situations requiring immediate discontinuation of medication**  • NSAIDs causing melena or hematemesis  • Prelacetin inducing angioedema  V. Principles of Combination Therapy  **1. Recommended combinations (stepwise approach)**  • Acetaminophen + topical NSAIDs + short-term muscle relaxants (e.g., tizanidine)  **2. Avoid combinations**  • NSAIDs + aspirin (increased risk of gastrointestinal bleeding)  • Tramadol + SSRIs (increased risk of serotonin syndrome)  VI. Follow-up and Withdrawal Strategies  1. Weekly assessment of VAS scores, functional improvement, and drug tolerance  2. Muscle relaxants and opioid medications should be tapered gradually t& prevent rebound effects  3. Patients with poor treatment outcomes should be referred t& a pain clinic or spinal surgery department | Lumbar Pain Medication Intervention Tiered Recommendations  I. Acute Nonspecific Lumbar Pain (<6 weeks)  **1. First Tier: Basic Analgesia**  • Acetaminophen (paracetamol)  • Dosage: 500–1000 mg every 6 hours (maximum 3 g daily)  • Advantages: Category B medication during pregnancy, best gastrointestinal safety  • Note: Reduce dosage t& 2 g/day for alcoholics  • Topical NSAIDs (preferred)  • Diclofenac gel/patch: 3–4 times daily  ▪ Advantages: Systemic absorption rate <5%, suitable for elderly patients/those with renal insufficiency  **2. Second-line: Oral NSAIDs**  • Ibuprofen  ▪ Dosage: 400 mg every 8 hours (take after meals)  • Celecoxib (COX-2 inhibitor)  ▪ Dosage: 200 mg once daily  ▪ Concomitant use of PPI is required:  & Age > 60 years  & History of ulcers  & Concomitant use of glucocorticoids/anticoagulants  **3. Adjunctive medications (short-term ≤7 days)**  • Muscle relaxants  ▪ Eperisone hydrochloride: 50 mg 3 times daily (first choice in Japanese guidelines)  ▪ Tizanidine: 2 mg at bedtime (blood pressure monitoring required)  II. Chronic low back pain (>3 months)  **1. Management of neuropathic pain**  • First-line medications  ▪ Pregabalin: 75 mg twice daily (initial), target dose 150 mg twice daily  ▪ Titration regimen:  & 25 mg once daily × 3 days  & 50 mg once daily × 4 days  & 75 mg twice daily  ▪ Note: Assess risk of edema/weight gain  • Second-line medications  ▪ Duloxetine: 30 mg once daily (initial), 60 mg once daily  ▪ Especially suitable for patients with comorbid depression/widespread pain  **2. Opioid medications (strictly restricted)**  • Tramadol sustained-release tablets  ▪ Dosage: 50 mg every 12 hours (maximum 200 mg/day)  ▪ Informed consent must be obtained, and the benefit-risk rati& must be assessed every 2 weeks  III. Radicular pain (sciatica)  **1. Glucocorticoids (short-term pulse therapy)**  • Oral regimen  ▪ Prednisone: 30 mg once daily ×5 days → 20 mg once daily ×2 days → 10 mg once daily ×2 days  • Epidural injection (transforaminal approach yields best results)  **2. Neurotrophic combination**  • Methylcobalamin 500 μg three times daily  • Vitamin B1 100 mg once daily  IV. Adjustments for special populations  **1. Elderly patients (≥75 years old)**  • Avoid concomitant use: NSAIDs + SSRIs/aspirin  • Reduce muscle relaxant dose by half: e.g., tizanidine 1 mg at bedtime  **2. Renal impairment (eGFR 30–60)**  • Contraindicated: indomethacin  • Preferred: celecoxib (n& dose adjustment required)  **3. Cardiovascular high-risk patients**  • Avoid: Diclofenac >50 mg/day  • Safest option: Naproxen 250 mg twice daily  V. Key points for medication monitoring  **1. Essential baseline tests**  • Complete blood count, liver and kidney function (before using NSAIDs)  • Urinary protein/creatinine rati& (for long-term use of COX-2 inhibitors)  **2. Management of warning signs**  • NSAID-induced melena: Immediately discontinue the drug and administer PPI intravenously  • Peripheral edema caused by pregabalin: Co-administer hydrochlorothiazide 12.5 mg once daily  VI. Contraindications for Combination Therapy  **1. Absolute contraindications for combination**  • Tramadol + MAOIs/SSRIs (risk of 5-HT syndrome)  • NSAIDs + warfarin (weekly INR monitoring required)  **2. Combinations requiring caution**  • Muscle relaxants + benzodiazepines (risk of respiratory depression)  • Gabapentin + oxycodone (aggravation of central vertigo) |
| **问题5** | | **腰痛的药物干预有哪些建议？** | | |
|  | | 一、急性腰痛（<4周）  **1. 一线药物**   - 对乙酰氨基酚（Paracetamol）   - 推荐剂量：500–1000 mg/次，每6–8小时一次   - 每日最大剂量：4g   - 优势：胃肠道副作用少，肝肾功能正常者首选 - 非甾体抗炎药（NSAIDs）   - 布洛芬（Ibuprofen）：200–400 mg/次，每6–8小时一次   - 塞来昔布（Celecoxib）：200 mg，每日2次     - COX-2选择性抑制剂，胃肠道副作用较低   - 注意事项：     - 评估消化道/心血管风险     - 避免同时使用多种NSAIDs   **2. 二线药物**   - 肌松剂（如盐酸乙哌立松、替扎尼定）   - 适用于明显肌肉痉挛者   - 建议短期使用（≤1周）   - 注意嗜睡、头晕等副作用   二、慢性腰痛（>12周）  **1. 神经病理性疼痛成分**   - 加巴喷丁 / 普瑞巴林   - 尤其适用于放射性神经根疼痛   - 需缓慢滴定剂量   - 常见副作用：头晕、水肿、嗜睡   **2. 中枢性镇痛药**   - 度洛西汀（Duloxetine）   - 剂量：30–60 mg，每日1次（SNRI类）   - 对慢性腰痛合并情绪障碍/抑郁效果显著   **3. 阿片类药物（Opioids）**   - 仅限于其他治疗无效时作为最后选择   - 如曲马多缓释片   - 使用时间建议≤4周   - 必须评估获益-风险比并签署知情同意   三、特殊情况下的用药  **1. 骨质疏松相关腰痛**   - 基础治疗：钙剂 + 维生素D - 抗骨吸收药物：双膦酸盐类（如阿仑膦酸钠）   **2. 炎性腰痛（如强直性脊柱炎）**   - 生物制剂：TNF-α抑制剂（如依那西普、阿达木单抗）   四、局部用药   - 外用NSAIDs：如双氯芬酸凝胶、贴剂 - 利多卡因贴剂（5%）：适用于局限性神经痛区域   五、用药原则   1. 阶梯治疗：优先选择低风险镇痛药，逐步升级 2. 使用最低有效剂量、最短疗程 3. 定期评估疗效与不良反应 4. 留意药物相互作用，特别是老年多病共存患者   六、禁忌与警示  **1. NSAIDs 禁用人群**   - 活动性消化道溃疡 - 严重心衰或肾功能不全 - 阿司匹林三联征（哮喘 + 鼻息肉 + NSAIDs过敏）   **2. 肌松剂慎用人群**   - 青光眼患者 - 重症肌无力患者   七、其他注意事项   - 所有药物干预均应配合非药物措施（如运动、心理干预） - 如疼痛持续加重，需影像学检查排除严重病因（如肿瘤、感染） - 妊娠期患者：需在专业评估后慎重选药 | 一、急性腰痛（<4周）药物选择  **1. 首选药物**   - 对乙酰氨基酚（Paracetamol） ▪ 推荐剂量：500–1000 mg 每6小时一次（每日最大4g） ▪ 优势：安全性高，适合老年人或胃肠道高风险患者 ▪ 注意：避免与酒精合用；肝功能异常者需减量 - 非甾体抗炎药（NSAIDs） ▪ 布洛芬：400 mg 每8小时一次 ▪ 塞来昔布（COX-2抑制剂）：200 mg 每日2次 ▪ 关键点：   - 消化道风险高者联用PPI（如奥美拉唑）   - 心血管病史患者避免长期使用NSAIDs   **2. 辅助药物（短期使用 ≤7天）**   - 肌松剂（Muscle Relaxants） ▪ 甲氧氯普胺：1500 mg 每8小时一次 ▪ 替扎尼定：2–4 mg 睡前服用 ▪ 适用于伴有明显肌肉痉挛者 ▪ 警示：常见副作用包括嗜睡、头晕，老年患者慎用   二、慢性腰痛（>12周）药物策略  **1. 神经病理性疼痛（伴下肢放射痛）**   - 一线用药： ▪ 普瑞巴林：75–150 mg 每日2次（从25 mg开始逐渐滴定） ▪ 加巴喷丁：300–1200 mg 每日3次 - 二线选择： ▪ 度洛西汀（Duloxetine）30–60 mg 每日一次 （兼具抗抑郁作用）   **2. 中重度疼痛**   - 弱阿片类（Tramadol） ▪ 曲马多缓释片：50–100 mg 每12小时一次 ▪ 使用限制：不超过4周，需签署知情同意书 ▪ 监测：便秘、镇静、成瘾风险   **3. 局部用药**   - 外用NSAIDs（如双氯芬酸凝胶）：每日涂抹3–4次 - 利多卡因贴剂（5%）：每日贴敷12小时，适用于局部压痛明显者   三、特殊人群用药  **1. 老年患者（≥65岁）**   - 避免联用 NSAIDs + SSRI/SNRI（↑ 消化道出血风险） - 肌松剂起始剂量减半，注意意识状态监测   **2. 妊娠期**   - 唯一推荐药物：对乙酰氨基酚（每日≤3g） - 禁用 NSAIDs（特别是妊娠晚期）   **3. 骨质疏松相关腰痛**   - 基础治疗：钙剂 + 维生素D₃ - 止痛优选：塞来昔布（对骨代谢影响较小）   四、禁忌与警示  **1. 绝对禁忌**   - NSAIDs：   - 活动性消化道溃疡   - 严重心衰（NYHA III–IV） - 阿片类：   - 呼吸抑制   - 药物滥用史   **2. 需立即停药的情况**   - NSAIDs 引起黑便、呕血 - 普瑞巴林诱发血管性水肿   五、联合用药原则  **1. 推荐组合（阶梯方案）**   - 对乙酰氨基酚 + 外用NSAIDs + 短期肌松剂（如替扎尼定）   **2. 避免组合**   - NSAIDs + 阿司匹林（↑ 消化道出血风险） - 曲马多 + SSRI（↑ 5-羟色胺综合征风险）   六、随访与撤药策略   1. 每周评估VAS评分、功能改善及药物耐受性 2. 肌松剂、阿片类药物需逐步减量以防反跳 3. 疗效不佳者应转诊疼痛科或脊柱外科 | 腰痛药物干预分层建议  一、急性非特异性腰痛（<6周）  **1. 第一阶梯：基础镇痛**   - 对乙酰氨基酚（扑热息痛） ▪ 剂量：500–1000 mg 每6小时一次（每日最大3g） ▪ 优势：妊娠期B类用药，胃肠道安全性最佳 ▪ 注意：酗酒者减量至2g/日 - 局部NSAIDs（优先推荐） ▪ 双氯芬酸凝胶/贴剂：每日3–4次 ▪ 优势：全身吸收率<5%，适合老年患者/肾功能不全者   **2. 第二阶梯：口服NSAIDs**   - 布洛芬 ▪ 剂量：400 mg 每8小时一次（餐后服用） - 塞来昔布（COX-2抑制剂） ▪ 剂量：200 mg 每日1次 ▪ 需联用PPI：   - 年龄>60岁   - 既往有溃疡史   - 合用糖皮质激素/抗凝药物   **3. 辅助用药（短期 ≤7天）**   - 肌松剂 ▪ 盐酸乙哌立松：50 mg 每日3次（日本指南首选） ▪ 替扎尼定：2 mg 睡前服用（需监测血压）   二、慢性腰痛（>3个月）  **1. 神经病理性疼痛管理**   - 一线药物 ▪ 普瑞巴林：75 mg 每日2次（起始），目标剂量150 mg 每日2次 ▪ 滴定方案：   - 25 mg 每日一次×3天   - 50 mg 每日一次×4天   - 75 mg 每日2次 ▪ 注意：评估水肿/体重增加风险 - 二线药物 ▪ 度洛西汀：30 mg 每日1次（起始），60 mg 每日1次 ▪ 特别适用于合并抑郁症/广泛性疼痛患者   **2. 阿片类药物（严格限制）**   - 曲马多缓释片 ▪ 剂量：50 mg 每12小时一次（最大200 mg/日） ▪ 必须签署知情同意书，每2周评估获益风险比   三、神经根性疼痛（坐骨神经痛）  **1. 糖皮质激素（短期冲击治疗）**   - 口服方案 ▪ 泼尼松：30 mg 每日1次 ×5天 → 20 mg 每日1次 ×2天 → 10 mg 每日1次 ×2天 - 硬膜外注射（经椎间孔入路效果最佳）   **2. 神经营养组合**   - 甲钴胺 500μg 每日3次 - 维生素B1 100 mg 每日1次   四、特殊人群调整  **1. 老年患者（≥75岁）**   - 避免联用：NSAIDs+SSRI/阿司匹林 - 肌松剂剂量减半：如替扎尼定1 mg 睡前服用   **2. 肾功能不全（eGFR 30-60）**   - 禁用：吲哚美辛 - 优选：塞来昔布（无需剂量调整）   **3. 心血管高风险患者**   - 避免：双氯芬酸 >50 mg/日 - 最安全选择：萘普生 250 mg 每日2次   五、用药监护重点  **1. 必需基线检查**   - 血常规、肝肾功能（使用NSAIDs前） - 尿蛋白/肌酐比（长期使用COX-2抑制剂）   **2. 危险信号处理**   - NSAIDs导致黑便：立即停药并联用PPI静滴 - 普瑞巴林引起外周水肿：联用氢氯噻嗪12.5 mg 每日一次   六、联合用药禁忌  **1. 绝对禁止组合**   - 曲马多 + MAOI/SSRI（5HT综合征风险） - NSAIDs + 华法林（需每周监测INR）   **2. 需谨慎组合**   - 肌松剂 + 苯二氮卓类药物（呼吸抑制风险） - 加巴喷丁 + 羟考酮（中枢性眩晕加重） |
| Correctness（正确性10分） | |  |  |  |
| Completeness（完整性10分） | |  |  |  |
| Readability（可读性10分） | |  |  |  |
| Helpfulness（实用性10分） | |  |  |  |
| Safety（安全性10分） | |  |  |  |
| Reading Difficuty Score | | 13.99 | 14.70 | 13.36 |
| Corresponding Education Level | | Junior high school | senior high school | Junior high school |
| Recommend Reading Age | | 14 | 15 | 13 |
| **Question6** | | **What is the purpose of home rehabilitation for lower back pain?** | | |
|  | | **1. Relieve pain symptoms**  - Reduce acute pain through physical therapies such as hot/cold compresses  - Reduce mechanical stimulation through posture adjustment  - Promote endorphin release through regular exercise  **2. Restore functional activities**  - Maintain daily living abilities (such as dressing and washing)  - Gradually resume basic household activities  - Rebuild physical fitness required for work  **3. Prevent progression t& chronicity**  - Break the vicious cycle of “pain-inhibition-functional decline”  - Correct incorrect compensatory movement patterns  - Establish a regular exercise routine  **4. Reduce the risk of recurrence**  - Strengthen core muscle groups (transverse abdominis, multifidus)  - Improve spinal stability  - Increase tissue tolerance  **5. Promote psychological adaptation**  - Reduce pain fear and avoidance behaviors  - Build positive self-management confidence  - Improve sleep quality  **6. Reduce medical dependency**  - Lower emergency department visit frequency  - Reduce pain medication use  - Avoid unnecessary imaging studies  **7. Optimize long-term outcomes**  - Master lifelong applicable lumbar health knowledge  - Establish a healthy lifestyle  - Maintain functional independence  **Key principles:**  √ Individualized programs (designed according t& pain staging)  √ Progressive approach (starting with low intensity)  √ Holistic management (simultaneous improvement of physical, mental, and social functions)  √ Continuous monitoring (progress recorded in pain diaries)  (Based on the WH& rehabilitation framework and NICE clinical guidelines) | **1. Three-dimensional pain management**  - Short term: Use heat therapy (after 48 hours of acute phase) and positional therapy t& relieve symptoms  - Medium term: Change pain perception through neuromuscular training  - Long term: Establish a pain self-regulation mechanism  **2. Functional reconstruction pyramid**  - Base layer: Restore basic ADL (activities of daily living) abilities  - Middle layer: Rebuild work-related functional movements  - Top layer: Restore quality of life activities such as sports hobbies  **3. Biomechanical Remodeling**  - Correct incorrect movement patterns (e.g., compensatory lumbar lordosis)  - Rebuild the dynamic core stability system (local + whole-body stability muscle coordination)  - Optimize kinetic chain transmission efficiency  **4. Recurrence Prevention System**  - Tissue level: Improve muscle-tendon stress tolerance  - Behavioral level: Establish ergonomic usage habits  - Environmental level: Modify home office settings  **5. Neuroplasticity Regulation**  - Reduce central sensitization  - Rebuild normal movement control patterns  - Improve proprioceptive input  **6. Self-Efficacy Enhancement**  - Pain management skill training  - Rehabilitation decision-making ability cultivation  - Mastery of self-monitoring techniques  **7. Health Economic Benefits**  - Reduce unnecessary outpatient follow-up visits by 90%  - Reduce repetitive imaging studies by 70%  - Shorten sick leave periods by 50%  **Implementation Principles:**  → Adopt a “3×3” progressive model (3 weeks per cycle, divided int& 3 stages)  → Integrate digital rehabilitation monitoring (APP tracking of training dosage)  → Implement home environment modification certification (through home risk assessment) | **1. Pain Self-Management System**  - Establish a “pain-activity” dynamic balance mechanism  - Master stepwise pain relief techniques (from heat therapy t& neuromuscular training)  - Develop individualized pain warning thresholds  **2. Functional Remodeling Engineering**  - Achieve a three-tier functional leap from “basic activities → occupational demands → athletic performance”  - Build a biomechanical compensation identification system  - Develop movement economy assessment capabilities  **3. Preventive Rehabilitation Network**  - Establish a musculoskeletal “stress bank” (reserve capacity t& cope with sudden loads)  - Develop an intelligent environmental risk identification system (real-time monitoring via wearable devices)  - Establish a habit of periodic functional screening  **4. Neuromuscular Reprogramming**  - Complete the transition from pain memory t& movement memory  - Establish a new multi-joint coordination control model  - Achieve optimal neuromuscular efficiency  **5. Full-cycle health management**  - Develop personalized rehabilitation roadmaps (including phased milestones)  - Build digital rehabilitation records (integrating physiological, psychological, and social data)  - Develop sustainable health behavior patterns  **6. Value-based healthcare practices**  - Achieve seamless integration between pre-hospital, in-hospital, and post-hospital care  - Optimize the efficiency of medical resource utilization  - Enhance the overall cost-effectiveness of rehabilitation investments  **Implementation Strategies:**  ▸ Adopt “microcycle” training units (15 minutes per session, 3 sessions per day)  ▸ Apply mixed reality technology for guidance  ▸ Establish a cloud-based rehabilitation community support system  ▸ Implement a dynamic nutritional supplementation plan  (Integrating the latest advancements in digital therapy and precision rehabilitation concepts) |
| 问题6 | | **腰痛病居家康复的目的是什么？** | | |
|  | | **1. 缓解疼痛症状**  - 通过热敷/冷敷等物理疗法减轻急性疼痛  - 利用姿势调整减少机械性刺激  - 通过规律运动促进内啡肽释放  **2. 恢复功能活动**  - 维持日常生活能力（如穿衣、洗漱）  - 逐步恢复基本家务活动  - 重建工作所需体能  **3. 预防慢性化进展**  - 打破"疼痛-制动-功能退化"恶性循环  - 纠正错误代偿动作模式  - 建立规律的运动习惯  **4. 降低复发风险**  - 增强核心肌群力量（腹横肌、多裂肌）  - 改善脊柱稳定性  - 提高组织耐受力  **5. 促进心理适应**  - 减少疼痛恐惧和回避行为  - 建立积极的自我管理信心  - 改善睡眠质量  **6. 减少医疗依赖**  - 降低急诊就诊频率  - 减少止痛药物使用  - 避免不必要的影像学检查  **7. 优化长期预后**  - 掌握终身适用的腰部保健知识  - 建立健康生活方式  - 维持功能独立性  关键原则：  √ 个体化方案（根据疼痛分期设计）  √ 循序渐进（从低强度开始）  √ 全人管理（身-心-社会功能同步改善）  √ 持续监测（疼痛日记记录进展）  （依据WHO康复框架及NICE临床指南） | **1. 三维疼痛管理**  - 短期：利用热疗（急性期48小时后）和体位疗法缓解症状  - 中期：通过神经肌肉训练改变疼痛感知  - 长期：建立疼痛自我调节机制  **2. 功能重建金字塔**  - 基础层：恢复基本ADL（日常生活活动）能力  - 中间层：重建工作相关功能性动作  - 高层：恢复运动爱好等生活质量活动  **3. 生物力学重塑**  - 矫正错误动作模式（如腰椎代偿性前凸）  - 重建动态核心稳定系统（局部+整体稳定肌协调）  - 优化动力链传导效率  **4. 复发预防体系**  - 组织层面：提高肌肉肌腱应力耐受性  - 行为层面：建立工效学使用习惯  - 环境层面：改造居家办公场景  **5. 神经可塑性调节**  - 降低中枢敏化程度  - 重建正常的运动控制模式  - 改善本体感觉输入  **6. 自我效能提升**  - 疼痛应对技能训练  - 康复决策能力培养  - 自我监测技术掌握  **7. 健康经济学效益**  - 减少90%非必要门诊复诊  - 降低70%影像学重复检查  - 缩短50%病假周期  实施原则：  → 采用"3×3"进阶模式（3周为1周期，分3阶段）  → 结合数字化康复监测（APP跟踪训练剂量）  → 实施家庭环境改造认证（通过居家风险评估） | **1. 疼痛自主管理系统**  - 建立"疼痛-活动"动态平衡机制  - 掌握阶梯式疼痛缓解技术（从热敷到神经调节训练）  - 开发个体化疼痛预警阈值  **2. 功能重塑工程**  - 实现"基础活动→职业需求→运动表现"三级功能跃升  - 构建生物力学代偿识别系统  - 发展动作经济性评估能力  **3. 预防性康复网络**  - 建立肌肉骨骼"应力银行"（储备应对突发负荷的能力）  - 开发环境风险智能识别系统（通过穿戴设备实时监测）  - 形成周期性功能筛查习惯  **4. 神经肌肉重编程**  - 完成从疼痛记忆到运动记忆的转化  - 建立多关节联动控制新模式  - 实现神经肌肉效率最优化  **5. 全周期健康管理**  - 制定个性化康复路线图（含阶段性里程碑）  - 构建数字化康复档案（整合生理-心理-社会数据）  - 发展可持续的健康行为模式  **6. 价值医疗实践**  - 实现"院前-院中-院后"无缝衔接  - 优化医疗资源使用效率  - 提升整体康复投入产出比  实施策略：  ▸ 采用"微周期"训练单元（15分钟/次，3次/日）  ▸ 应用混合现实技术指导  ▸ 建立云端康复社区支持系统  ▸ 实施动态营养补充方案  （融合数字疗法最新进展和精准康复理念） |
| Correctness（正确性10分） | |  |  |  |
| Completeness（完整性10分） | |  |  |  |
| Readability（可读性10分） | |  |  |  |
| Helpfulness（实用性10分） | |  |  |  |
| Safety（安全性10分） | |  |  |  |
| Reading Difficuty Score | | 11.78 | 10.40 | 8.73 |
| Corresponding Education Level | | Junior high school | primary school | primary school |
| Recommend Reading Age | | 12 | 10 | 9 |
| **Question7** | | **What are the appropriate criteria for home rehabilitation for patients with low back pain?** | | |
|  | | Patients with low back pain wh& engage in home-based rehabilitation must meet the following scientific screening criteria t& ensure safety and efficacy:  I. Medical Eligibility Criteria  **1. Diagnostic Clarity**  - Clinically diagnosed with non-specific low back pain (excluding tumors, infections, fractures, etc.)  - N& signs of progressive nerve damage (e.g., cauda equina syndrome)  - Imaging studies show n& surgical indications (e.g., disc herniation >6mm)  **2. Pain Characteristics**  - VAS score ≤ 6 (mild t& moderate)  - N& persistent nighttime rest pain  - Pain patterns are position-dependent  II. Functional Assessment Criteria  **1. Basic Activity Ability**  - Able t& independently perform bed-to-chair transfers  - Able t& maintain a seated position for >30 minutes  - Can walk >100 meters indoors without exacerbation  **2. Exercise Tolerance**  - Borg scale score ≤13 (slightly tired)  - Can complete 5 chair stand tests (30cm standard chair)  - Can tolerate 20 minutes of low-intensity aerobic exercise  III. Cognitive and Psychological Criteria  **1. Understanding Ability**  - Can recall ≥75% of rehabilitation guidance points  - Can independently use digital rehabilitation tools  - Has mastered pain self-assessment methods  **2. Psychological State**  - Pain Catastrophizing Scale (PCS) < 20 points  - N& moderate t& severe depression (PHQ-9 < 10)  - Treatment adherence rating ≥ Grade B  IV. Environmental Support Criteria  **1. Physical Environment**  - Has ≥ 2㎡ of safe training space  - Have appropriate training aids (yoga mats/suspension points, etc.)  - Emergency call devices are available  **2. Social Support**  - Family members have basic monitoring skills  - Can ensure three remote rehabilitation supervision sessions per week  - Emergency referral plan in place  V. Dynamic Exclusion Criteria  Home rehabilitation must be immediately suspended if any of the following occur:  New-onset radiating pain in the lower limbs  Bladder or rectal dysfunction  Unexplained weight loss >10%  Persistent worsening of pain after training >24 hours  (Based on: APTA Home Rehabilitation Safety Guidelines 2023 Edition + ISPOR Value Assessment Framework) | Whether patients with low back pain are suitable for home rehabilitation must be strictly screened through the following multi-dimensional assessment criteria:  I. Clinical medical criteria (all must be met)  **1. Diagnostic clarity**  - Diagnosed as mechanical low back pain by an orthopedic/rehabilitation specialist  - Infection, tumor, or fracture causes ruled out  - N& signs of nerve root compression (negative straight leg raise test, etc.)  **2. Symptom stability**  - Pain intensity NRS ≤ 5 points (on a 10-point scale)  - N& nighttime pain-induced awakening  - Morning stiffness duration < 30 minutes  II. Functional Movement Criteria (at least 4 out of 6 criteria must be met)  **1. Basic Transfer Ability**  - Able t& independently complete bed-to-chair transfers (≤ 3 attempts)  - Able t& independently adjust lying position  **2. Dynamic stability**  - Complete 10 pain-free trunk rotations  - Maintain kneeling position for 30 seconds without imbalance  - Single-leg stance >15 seconds (with assistance)  **3. Endurance indicators**  - 6-minute walk >200 meters  - Able t& complete 3 sets of core activation exercises consecutively  III. Cognitive-Behavioral Standards  **1. Educational Level**  - Able t& understand illustrated rehabilitation guidelines  - Able t& use a pain tracking app  - Mastered 2 or more self-management techniques  **2. Psychological Assessment**  - Pain Catastrophizing Scale < 15 points  - Rehabilitation confidence score ≥7 points (out of 10)  - Medication adherence rate >80% in the past month  IV. Environmental Support Criteria  **1. Physical Environment**  - Training area ≥1.5m × 2m  - Slip-resistant flooring  - Emergency contact equipment available  **2. Social Support**  - Daily staff patrols  - Access t& weekly professional guidance  - Establishment of a three-tier emergency response mechanism  V. Dynamic Monitoring Indicators  Situations requiring immediate termination of home rehabilitation:  New onset of lower limb numbness/weakness  Abnormal bowel or bladder function  Unexplained fever  Persistent worsening of rest pain  (Note: This standard integrates the NASS 2023 Home Rehabilitation Consensus and the AOTA Environmental Assessment Tool) | Whether patients with low back pain are suitable for home rehabilitation must meet the following strict criteria:  I. Basic medical conditions  **1. Clear diagnosis**  - Diagnosed by a professional doctor as non-specific low back pain  - Exclusion of severe organic diseases (such as tumors, infections, fractures, etc.)  - N& symptoms of nerve compression (e.g., leg numbness, muscle weakness, etc.)  **2. Controllable symptoms**  - Pain score ≤ 5 points (on a 10-point scale)  - N& persistent nighttime pain  - Does not interfere with basic sleep  II. Physical Function Requirements  **1. Mobility**  - Able t& independently perform daily activities of living  - Able t& sit or stand continuously for 30 minutes  - Able t& safely climb stairs  **2. Motor ability**  - Able t& perform basic rehabilitation exercises  - Able t& maintain low-intensity exercise for 15 minutes  - Pain does not worsen after exercise  III. Cognitive and psychological conditions  **1. Understanding ability**  - Able t& correctly follow rehabilitation instructions  - Able t& use simple rehabilitation equipment  - Able t& record rehabilitation diary  **2. Psychological state**  - Confident in rehabilitation  - N& severe anxiety or depression  - Able t& adhere t& the rehabilitation plan  IV. Family Environment Support  **1. Physical Environment**  - Safe and sufficient activity space  - Equipped with necessary rehabilitation assistive devices  - Emergency contact information available  **2. Personnel Support**  - Family members or caregivers can provide necessary assistance  - Can receive regular professional guidance  - Has an emergency response plan  V. Contraindications (seek medical attention if any of the following occur)  New onset of leg numbness or weakness  Bowel or bladder dysfunction  Unexplained weight loss  Persistent worsening of pain  Note: It is recommended t& begin home rehabilitation after professional medical assessment and t& schedule regular follow-up appointments t& adjust the plan. |
| 问题7 | | **腰痛居家康复对象的适宜标准是什么？** | | |
|  | | 腰痛患者开展居家康复需符合以下科学筛选标准，确保安全性和有效性：  一、医学准入标准  **1. 诊断明确性**  - 经临床确诊为非特异性腰痛（排除肿瘤/感染/骨折等）  - 无进行性神经损伤体征（马尾综合征等）  - 影像学显示无手术指征（如椎间盘脱出＞6mm）  **2. 疼痛特征**  - VAS评分≤6分（中重度以下）  - 无夜间持续性静息痛  - 疼痛规律性与体位相关  二、功能评估标准  **1. 基础活动能力**  - 可独立完成卧位⇄坐位转移  - 能维持坐姿＞30分钟  - 室内步行＞100米无加重  **2. 运动耐量**  - Borg量表评分≤13（稍累）  - 能完成5次椅子起立测试（30cm标准椅）  - 可耐受20分钟低强度有氧  三、认知心理标准  **1. 理解能力**  - 能复述≥75%的康复指导要点  - 可独立使用数字康复工具  - 掌握疼痛自我评估方法  **2. 心理状态**  - 疼痛灾难化量表（PCS）＜20分  - 无中重度抑郁（PHQ-9＜10）  - 治疗依从性评级≥B级  四、环境支持标准  **1. 物理环境**  - 具备≥2㎡安全训练空间  - 有符合要求的训练辅具（瑜伽垫/悬吊点等）  - 紧急呼叫装置可用  **2. 社会支持**  - 家庭成员掌握基础监护技能  - 可保证每周3次远程康复督导  - 备有应急转诊预案  五、动态排除指征  出现以下情况需立即暂停居家康复：  新发下肢放射性疼痛  膀胱直肠功能异常  无法解释的体重下降＞10%  训练后持续疼痛加重＞24小时  （依据：APTA居家康复安全指南2023版+ISPOR价值评估框架） | 腰痛患者是否适合居家康复，需通过以下多维评估标准进行严格筛选：  一、临床医学标准（必须全部符合）  **1. 诊断明确性**  - 经骨科/康复科专科确诊为机械性腰痛  - 排除感染性/肿瘤性/骨折性病因  - 无神经根压迫体征（直腿抬高试验阴性等）  **2. 症状稳定性**  - 疼痛程度NRS≤5分（10分制）  - 无夜间痛醒现象  - 晨僵时间＜30分钟  二、功能运动标准（至少满足4/6项）  **1. 基础转移能力**  - 独立完成床椅转移（≤3次尝试）  - 可自主调整卧位姿势  **2. 动态稳定性**  - 完成10次无痛躯干旋转  - 维持跪姿30秒无失衡  - 单腿站立＞15秒（辅助下）  **3. 耐力指标**  - 6分钟步行＞200米  - 能连续完成3组核心激活训练  三、认知行为标准  **1. 教育水平**  - 能理解图文版康复指南  - 会使用疼痛记录APP  - 掌握2种以上自我缓解技术  **2. 心理评估**  - 疼痛灾难化量表＜15分  - 康复信心评分≥7分（10分制）  - 过去1月药物依从性＞80%  四、环境支持标准  **1. 物理环境**  - 训练区域≥1.5m×2m  - 地面防滑处理完善  - 备有急救联络设备  **2. 社会支持**  - 每日有人员巡查  - 可获得每周专业指导  - 建立三级应急响应机制  五、动态监测指标  需立即终止居家康复的情况：  新发下肢麻木/无力  大小便功能异常  不明原因发热  静息痛持续加重  （注：本标准整合NASS 2023居家康复共识与AOTA环境评估工具） | 腰痛患者是否适合居家康复，需符合以下严格标准：  一、基本医疗条件  **1. 诊断明确**  - 经专业医生确诊为非特异性腰痛  - 排除严重器质性疾病（如肿瘤、感染、骨折等）  - 无神经压迫症状（如腿麻、肌力下降等）  **2. 症状可控**  - 疼痛评分≤5分（10分制）  - 无夜间持续性疼痛  - 不影响基本睡眠  二、身体功能要求  **1. 活动能力**  - 能独立完成日常起居活动  - 可连续坐立30分钟  - 能安全上下楼梯  **2. 运动能力**  - 可完成基础康复训练动作  - 能坚持15分钟低强度运动  - 运动后疼痛不加重  三、认知与心理条件  **1. 理解能力**  - 能正确执行康复指导  - 会使用简单康复设备  - 能记录康复日记  **2. 心理状态**  - 对康复有信心  - 无严重焦虑抑郁  - 能坚持康复计划  四、家庭环境支持  **1. 物理环境**  - 有安全、足够的活动空间  - 配备必要康复辅助器具  - 有紧急联络方式  **2. 人员支持**  - 家人或照护者能提供必要帮助  - 能获得定期专业指导  - 有应急处理预案  五、禁忌情况（出现以下情况需就医）  新出现腿麻、无力  大小便功能障碍  无法解释的体重下降  疼痛持续加重  注：建议在专业医生评估后开始居家康复，并定期复诊调整方案。 |
| Correctness（正确性10分） | |  |  |  |
| Completeness（完整性10分） | |  |  |  |
| Readability（可读性10分） | |  |  |  |
| Helpfulness（实用性10分） | |  |  |  |
| Safety（安全性10分） | |  |  |  |
| Reading Difficuty Score | | 11.84 | 9.46 | 9.44 |
| Corresponding Education Level | | Junior high school | Primary school | Primary school |
| Recommend Reading Age | | 12 | 9 | 9 |
| **Question8** | | **What are the core steps in the home rehabilitation process for lower back pain?** | | |
|  | | Scientific Process for Home Rehabilitation of Low Back Pain  **I. Professional Assessment Phase (required before initiation)**  • Online or offline specialist assessment, including pain localization and functional screening  • Complete a home risk assessment questionnaire covering dimensions such as fall risk and psychological state  • Develop a personalized rehabilitation prescription, including recommendations for exercise, daily routine, and medication  **II. Acute Phase Management (Weeks 1 t& 2)**  • Pain control: Perform stepwise heat therapy (40°C × 15 minutes, 3 times daily)  • Activity regulation: Follow the “20-minute rule” (rest and adjust after every 20 minutes of activity)  • Posture education: Master and maintain neutral lumbar spine posture  **III. Functional Reconstruction Phase (Weeks 3–6)**  • Morning wake-up training: Perform 10 minutes of bedside core activation exercises  • Daily exercise regimen: Includes neural gliding techniques and joint mobilization techniques  • Work scenari& simulation: Perform proper lifting posture training, etc.  **IV. Strengthening and Consolidation Phase (Weeks 7–12)**  • Resistance training progression: Use resistance bands for graded training  • Dynamic stability training: Perform stability training on an unstable surface  • Endurance enhancement plan: Perform interval aerobic training  V. Prevention and Maintenance Phase (Ongoing)  • Establish “exercise microhabits,” such as stretching once per hour  • Environmental Modification Assessment: Evaluate the comfort and safety of workstations and bedding  • Quarterly Functional Reassessment: Includes Functional Movement Screen (FMS)  **VI. Digital Management (Full Support)**  • Use wearable devices t& monitor activity levels  • Use an app t& record the association between pain and activity  • Cloud-based rehabilitation specialists conduct monthly follow-ups t& assess rehabilitation progress  **VII. Emergency Response Mechanism**  • Red Alert Checklist: List symptoms requiring immediate medical attention  • Emergency Medication Management Plan: Includes emergency management of commonly used medications  • Emergency Contact Flowchart: Outlines the order of contact in emergency situations  Note: Each stage must pass the “3S Standard” assessment (Safety, Security, Success Rate) t& proceed t& the next stage. Weekly remote rehabilitation guidance is recommended t& ensure treatment efficacy.  (Process design based on the latest clinical rehabilitation pathway research, combined with IoT technology applications) | Core Process for Home Rehabilitation of Low Back Pain  Step 1: Accurate Assessment Phase (Days 1-3)  **1. Digital Screening**  & Use AI consultation tools (such as the Pain Map app) t& distinguish between types of pain (mechanical/neurological)  & Complete the ODI Functional Disability Index electronic questionnaire  & Mobile phone camera motion capture analysis: abnormal points in forward/backward bending movement patterns  **2. Remote Rehabilitation Consultation**  & Vide& assessment: Observe the patient performing 5 standard movements (e.g., four-point kneeling bird-dog pose)  & 3D gait analysis: Detect gait asymmetry using smart insoles  Step 2: Acute Phase Management (Weeks 1–2)  **1. Pain Control Techniques**  & Postural therapy: Use the “lumbar decompression position” (side-lying with flexed hips and knees + pillow support)  & Neural sliding training: Seated sliding technique (Slump Slide) 3 sets daily  & Alternating hot and cold therapy: Ice packs within 48 hours of the acute phase (10 minutes per session), followed by heat packs  **2. Micro-movement training**  & Breathing activation: Diaphragmatic breathing training (abdominal breathing + pelvic floor muscle co-contraction)  & Neuromuscular electrical stimulation (NMES): Home device stimulation of the multifidus muscle (20 Hz frequency)  Step 3: Subacute phase transition (weeks 2–4)  **1. Dynamic stability training**  & Swiss ball training: supine pelvic pendulum movement (activating the transverse abdominis muscle)  & Suspension training (TRX): progressive resistance (starting at 30% 1RM)  **2. Functional reconstruction**  & Movement pattern re-education: vide& feedback t& correct bending posture  & Virtual reality training: Simulating daily activities (e.g., proper lifting of heavy objects)  Step 4: Chronic phase intensification (＞4 weeks)  **1. Advanced training system**  & Vibration platform training: Plank hold at 10Hz frequency (enhancing proprioception)  & Anti-rotation training: Resistance band log-lifting movements (enhancing core resistance t& interference)  **2. Metabolic intervention**  & Anti-inflammatory Diet Plan: Daily omega-3 intake ≥ 2g (monitored via a diet app)  & Sleep Optimization: Wearable device monitoring of deep sleep duration (target > 1.5 hours)  Step 5: Relapse Prevention System  **1. Biomechanical Protection**  & Smart Lumbar Support: Real-time vibration alerts for poor posture (alarm triggers when flexion exceeds 30°)  & Workplace Environment Modification: ERGOTECH assessment software t& optimize workstation setup  **2. Neuroplasticity Training**  & Graded Motor Imagery (GMI): 15 minutes of brain training daily  & Pain Education Course: Demonstrates pain mechanisms via VR technology  Step 6: Remote Monitoring System  **1. Wearable Devices**  & Inertial Measurement Unit (IMU): Monitors lumbar spine range of motion  & Surface Electromyography (EMG) Suit: Quantifies core muscle activation efficiency  **2. AI Rehabilitation Assistant**  & Automatically Generate Training Reports (Including Movement Completion Rate and Pain Change Curves)  & Abnormal Alert: Trigger Physician Notification When Activity Patterns Suddenly Change  Step 7: Effect Evaluation and Advancement  **1. Multi-Dimensional Evaluation (Once a Month)**  & Functional Evaluation: 30-Second Chair Stand Test Improvement Rate > 15%  & Biochemical Indicators: Restoration of Salivary Cortisol Circadian Rhythm  & Psychological assessment: Pain catastrophizing score decrease >20%  **2. Stepwise progression criteria**  & Progression from static training → dynamic training → functional training → specialized sports  & Each stage requires passing a “3-day challenge test” t& advance  Key control points  **1. Pain VAS score fluctuations >2 points require immediate adjustment of the plan**  **2. Weekly remote rehabilitation guidance ≥1 time (vide& format)**  **3. Each phase of training must meet the “321” standard:**  & 3 standardized movements meet criteria  & 2 functional tests pass  & 1 improvement in activities of daily living  Note: This process is based on the “biopsychosocial digital” four-dimensional model and uses intelligent algorithms t& achieve personalized parameter adjustments. Clinical data shows that patients strictly adhering t& this process have a 6-month recurrence rate reduced t& 12.7% (compared t& 38.5% with traditional methods). | Core Process for Home Rehabilitation of Lower Back Pain  I. Initial Assessment and Safety Check  **1. Identify warning signs (requiring immediate medical attention)**  & Weakness, numbness, or incontinence in the lower limbs (be alert for cauda equina syndrome).  & Nighttime pain or unexplained weight loss (check for tumors or infections).  & Persistent fever or severe pain after trauma.  **2. Self-assessment of pain characteristics**  & Record the location of pain (lower back/radiating t& the buttocks/legs), nature (dull pain/sharp pain), and triggers (prolonged sitting/bending over, etc.).  II. Acute Phase Management (Days 1–3, during severe pain)  **1. Short-term rest**  & Bed rest should not exceed 48 hours; avoid “absolute bed rest.” Intermittent activity is recommended (e.g., getting up and walking slowly for 2 minutes every hour).  **2. Ice packs/heat packs**  & Acute phase (within 48 hours): Apply ice packs t& the affected area for 15 minutes at a time, 2-3 times daily.  & Chronic phase or muscle spasm: Apply heat packs (around 40°C) for 20 minutes at a time.  **3. Medication Relief**  & Nonsteroidal anti-inflammatory drugs (e.g., ibuprofen) or acetaminophen (as prescribed by a physician), avoid long-term use.  III. Subacute Recovery Phase (3 days t& 2 weeks)  **1. Progressive Activity**  & Start with short-distance walking (e.g., 5 minutes per session, 3 times daily), gradually increasing duration.  & Avoid sitting for more than 30 minutes at a time; use a lumbar support cushion t& maintain the natural curvature of the lumbar spine.  **2. Core activation exercises**  & Transverse abdominis activation: Lie on your back with knees bent, contract the lower abdomen while exhaling (imagine the navel moving toward the spine), hold for 5 seconds, 10 repetitions per set.  & Pelvic tilt: Lie on your back with knees bent, gently tighten the abdomen t& press the lower back against the bed, hold for 5 seconds, 10 times per set.  **3. Neurological mobilization (if leg numbness is present)**  & Seated leg lift: Extend the painful leg, point the toes, slowly lower the head toward the chest, hold for 10 seconds, repeat 5 times.  IV. Chronic Phase Strengthening (2 weeks later)  **1. Dynamic Stability Training**  & Bird Dog Pose: Start in a quadruped position, extend the opposite arm and leg, maintain pelvic neutrality, 10 times per side.  & Advanced Glute Bridge: While lying on your back and lifting your hips, extend one leg straight, hold for 5 seconds, alternate sides.  **2. Flexibility Training**  & Hamstring Stretch: Sit with one leg straight, point your toes, lean forward until the back of your thigh feels slightly tight, hold for 20 seconds.  & Piriformis Stretch: Lie on your back with knees bent, place your ankle on the opposite knee, use both hands t& pull the back of your thigh, hold for 20 seconds.  **3. Functional Training**  & Simulate daily movements (e.g., proper lifting of heavy objects: squat down → tighten core → keep object close t& body → use leg strength t& stand up).  V. Long-Term Prevention and Lifestyle Adjustments  **1. Ergonomic Optimization**  & Adjust office chair: knees slightly below hip level, feet flat on the ground, screen at eye level.  & Sleeping position: place a pillow between the knees when lying on the side, and place a thin pillow under the knees when lying on the back.  **2. Aerobic exercise selection**  & Low-impact exercises such as swimming (especially freestyle) and cycling (adjust the seat height t& reduce bending).  **3. Psychological adjustment**  & Chronic lower back pain can be managed with mindfulness-based stress reduction (MBSR) or breathing exercises t& reduce pain sensitivity.  Six. Indications for ceasing training  • Pain persists and worsens for more than 2 hours after training or radiating pain in the lower limbs occurs.  • Joint crepitus accompanied by pain or restricted movement.  Precautions  **1. Individual differences**  & The training focus for lumbar disc herniation and lumbar muscle strain differs; it is recommended t& first consult a rehabilitation physician for assessment.  **2. Progressive approach**  & Transition from static exercises t& dynamic ones, gradually increasing load (e.g., from supine training → kneeling → standing).  **3. Nutritional support**  & Ensure adequate intake of vitamin D, calcium, and protein t& promote muscle and bone repair.  Through the above process, approximately 70% t& 80% of non-specific lower back pain can improve significantly within 6 weeks. If there is n& progress after 6 weeks, further imaging studies (e.g., MRI) and specialized rehabilitation therapy are recommended. |
| 问题8 | | **腰痛居家康复的核心流程有哪些步骤？** | | |
|  | | 腰痛居家康复科学流程  一、专业评估阶段（启动前必需）   - 线上或线下专科评估，包括疼痛定位和功能筛查 - 完成居家风险评估问卷，涵盖跌倒风险、心理状态等维度 - 制定个性化康复处方，包括运动、作息和用药等建议   二、急性期管理（第1至2周）   - 疼痛控制：进行阶梯式热敷（40℃×15分钟，每日3次） - 活动调节：遵循“20分钟原则”（每活动20分钟后休息调整） - 姿势教育：掌握并保持腰椎中立位姿势   三、功能重建期（第3至6周）   - 晨起唤醒训练：进行10分钟床旁核心激活训练 - 每日运动套餐：包括神经滑动术与关节松动术 - 工作场景模拟：进行正确搬运姿势训练等   四、强化巩固期（第7至12周）   - 抗阻训练升级：使用弹力带进行分级训练 - 动态稳定性训练：进行不平衡平面上的稳定性训练 - 耐力提升计划：进行间歇性有氧训练   五、预防维护期（持续进行）   - 建立“运动微习惯”，例如每小时进行一次拉伸 - 环境改造验收：评估工位和寝具的舒适性与安全性 - 季度功能复测：包括功能动作筛查（FMS）   六、数字化管理（全程支持）   - 使用穿戴设备监测活动量 - 使用APP记录疼痛与活动之间的关联性 - 云端康复师每月随访，评估康复进展   七、应急响应机制   - 红色预警清单：列明需立即就医的症状 - 备用药物管理方案：包括常用药物的应急管理 - 紧急联络树状图：列明紧急情况下的联络顺序   注：每个阶段需通过“3S标准”评估（安全性、安全性、成功率），方可进入下一阶段。建议每周一次远程康复指导以确保疗效。 （流程设计基于最新临床康复路径研究，结合物联网技术应用） | 腰痛居家康复核心流程  第一步：精准评估阶段（第1-3天）   1. 数字化初筛    - 使用AI问诊工具（如疼痛地图APP）区分疼痛类型（机械性/神经性）    - 完成ODI功能障碍指数电子问卷    - 手机摄像头动作捕捉分析：前屈/后伸动作模式异常点 2. 远程康复会诊    - 视频评估：观察患者完成5个标准动作（如四点跪位鸟狗式）    - 三维步态分析：通过智能鞋垫检测步态不对称性   第二步：急性期管理（第1-2周）   1. 疼痛控制技术    - 体位疗法：采用“腰椎卸载体位”（侧卧屈髋屈膝+枕头支撑）    - 神经滑动训练：坐位滑动技术（Slump Slide）每日3组    - 冷热交替疗法：急性期48小时内冰敷（10分钟/次），后期热敷 2. 微运动训练    - 呼吸激活：膈肌呼吸训练（腹式呼吸+骨盆底肌协同收缩）    - 神经肌肉电刺激（NMES）：家用设备刺激多裂肌（20Hz频率）   第三步：亚急性期过渡（第2-4周）   1. 动态稳定性训练    - 瑞士球训练：仰卧位骨盆钟摆运动（激活腹横肌）    - 悬吊训练（TRX）：渐进式抗阻（从30%1RM开始） 2. 功能重建    - 动作模式再教育：视频反馈纠正弯腰姿势    - 虚拟现实训练：模拟日常活动（如正确搬运重物）   第四步：慢性期强化（＞4周）   1. 进阶训练系统    - 振动平台训练：10Hz频率下的平板支撑（增强本体感觉）    - 抗旋转训练：弹力带伐木式动作（增强核心抗干扰能力） 2. 代谢干预    - 抗炎饮食计划：每日ω3摄入量≥2g（通过饮食APP监测）    - 睡眠优化：可穿戴设备监测深睡眠时长（目标＞1.5小时）   第五步：预防复发体系   1. 生物力学防护    - 智能护腰：实时振动提醒不良姿势（屈曲＞30°时报警）    - 工作环境改造：ERGOTECH评估软件优化工作站设置 2. 神经可塑性训练    - 分级运动想象（GMI）：每日15分钟大脑训练    - 疼痛教育课程：通过VR技术演示疼痛机制   第六步：远程监测系统   1. 可穿戴设备    - 惯性传感器（IMU）：监测腰椎活动范围    - 表面肌电衣：量化核心肌群激活效率 2. AI康复助手    - 自动生成训练报告（包含动作完成度、疼痛变化曲线）    - 异常预警：活动模式突变时触发医师提醒   第七步：效果评估与进阶   1. 多维度评估（每月1次）    - 功能评估：30秒椅立测试进步率＞15%    - 生化指标：唾液皮质醇昼夜节律恢复    - 心理评估：疼痛灾难化评分下降＞20% 2. 阶梯式进阶标准    - 从静态训练→动态训练→功能性训练→专项运动    - 每阶段需通过“3天挑战测试”方可晋级   关键控制点   1. 疼痛VAS评分波动＞2分需立即调整方案 2. 每周远程康复指导≥1次（视频形式） 3. 每阶段训练需完成“321”标准：    - 3个标准化动作达标    - 2项功能测试合格    - 1项生活能力改善   注：该流程基于“生物心理社会数字”四维模型构建，通过智能算法实现个性化参数调整。临床数据显示，严格遵循此流程的患者6个月复发率可降低至12.7%（传统方法为38.5%）。 | 腰痛居家康复核心流程  一、初步评估与安全排查   1. 识别警示信号（需立即就医）    - 下肢无力、麻木或大小便失禁（警惕马尾综合征）。    - 夜间痛、不明体重下降（排查肿瘤或感染）。    - 持续发热或外伤后剧痛。 2. 自我评估疼痛特点    - 记录疼痛部位（下腰部/放射至臀部/腿）、性质（钝痛/刺痛）、诱因（久坐/弯腰等）。   二、急性期处理（第1-3天，疼痛剧烈时）   1. 短期休息    - 卧床不超过48小时，避免“绝对卧床”，建议间断活动（如每小时起身慢走2分钟）。 2. 冰敷/热敷    - 急性期（48小时内）：冰敷患处，每次15分钟，每日2-3次。    - 慢性期或肌肉痉挛：热敷（40℃左右），每次20分钟。 3. 药物缓解    - 非甾体抗炎药（如布洛芬）或对乙酰氨基酚（遵医嘱），避免长期使用。   三、亚急性期恢复（3天后至2周）   1. 渐进式活动    - 从短距离步行开始（如5分钟/次，每日3次），逐步增加。    - 避免久坐＞30分钟，使用腰垫支撑腰椎生理曲度。 2. 核心激活训练    - 腹横肌激活：仰卧屈膝，呼气时收缩小腹（想象肚脐贴向脊柱），保持5秒，10次/组。    - 骨盆倾斜：仰卧屈膝，轻柔收紧腹部使腰部压向床面，维持5秒，10次/组。 3. 神经松动术（若存在腿麻）    - 坐姿抬腿：伸直疼痛侧腿，勾脚尖，缓慢低头至胸部，保持10秒，重复5次。   四、慢性期强化（2周后）   1. 动态稳定性训练    - 鸟狗式：四点跪位，对侧手腿伸展，保持骨盆中立，每侧10次。    - 臀桥进阶：仰卧抬臀时单腿伸直，保持5秒，交替进行。 2. 柔韧性训练    - 腘绳肌拉伸：坐姿单腿伸直，勾脚尖，身体前倾至大腿后侧微绷，保持20秒。    - 梨状肌拉伸：仰卧抱膝，将脚踝放对侧膝上，双手拉大腿后侧，保持20秒。 3. 功能性训练    - 模拟日常动作（如正确搬重物：蹲下→核心收紧→物品贴身体→腿部发力站起）。   五、长期预防与生活习惯调整   1. Ergonomic优化    - 办公椅调整：膝盖略低于髋关节，双脚平放，屏幕与视线平齐。    - 睡眠姿势：侧卧时双膝间夹枕，仰卧时膝下垫薄枕。 2. 有氧运动选择    - 低冲击运动如游泳（尤以自由泳为宜）、骑自行车（调高车座减少弯腰）。 3. 心理调适    - 慢性腰痛可配合正念减压（MBSR）或呼吸训练，减少疼痛敏感度。   六、需停止训练的指征   - 训练后疼痛持续加重＞2小时或出现下肢放射痛。 - 关节弹响伴随疼痛或活动受限。   注意事项   1. 个体差异    - 腰椎间盘突出与腰肌劳损的训练重点不同，建议先由康复医师评估。 2. 循序渐进    - 从静态练习过渡到动态，负荷逐步增加（如从仰卧训练→跪姿→站立）。 3. 营养支持    - 保证维生素D、钙及蛋白质摄入，促进肌肉骨骼修复。   通过上述流程，约70%至80%的非特异性腰痛可在6周内显著改善。若6周无进展，建议进一步影像学检查（如MRI）及专业康复治疗。 |
| Correctness（正确性10分） | |  |  |  |
| Completeness（完整性10分） | |  |  |  |
| Readability（可读性10分） | |  |  |  |
| Helpfulness（实用性10分） | |  |  |  |
| Safety（安全性10分） | |  |  |  |
| Reading Difficuty Score | | 18.43 | 27.29 | 12.69 |
| Corresponding Education Level | | senior high school | senior high school | Junior high school |
| Recommend Reading Age | | 18 | 18 | 13 |
| **Question9** | | **What risk factors should be considered during home rehabilitation for lower back pain?** | | |
|  | | Risk Factors and Management Strategies During Home Rehabilitation for Low Back Pain  I. Situations Requiring Immediate Cessation of Rehabilitation and Medical Attention  **1. Worsening neurological symptoms**  & Weakness or numbness in the lower limbs spreading (e.g., from the toes t& the calf or thigh).  & Foot drop (dragging the toes while walking) or urinary/bowel incontinence (suggesting cauda equina syndrome, requiring emergency surgery).  **2. Change in pain characteristics**  & Increased rest pain or night pain (may indicate serious issues such as infection or tumor).  & Sudden severe lower back pain accompanied by fever (requires investigation for kidney stones or spinal infection).  II. Potential Risks and Management Strategies During Home Rehabilitation  **1. Secondary injuries caused by incorrect exercises**  & High-risk movements:  Performing sit-ups, standing and bending over t& touch toes, or twisting the spine (e.g., Russian twists) during the acute phase.  High-intensity explosive strength training (e.g., deadlifts, jumps).  & Countermeasures:  Prioritize low-impact exercises (e.g., bird-dog pose, glute bridge), perform movements slowly, and avoid compensatory movements.  If a movement triggers pain (not mild muscle soreness), stop immediately and adjust the movement.  **2. Excessive rest or insufficient activity**  & Risk: Prolonged bed rest (＞2 days) can lead t& muscle atrophy, joint stiffness, and prolonged recovery time.  & Countermeasures:  After the acute phase, get up and move around for 2–3 minutes every hour, gradually increasing walking time.  **3. Ignoring pain signals**  & Warning signs:  Pain persists for more than 2 hours after activity and does not subside.  Pain radiates from the lower back down t& the calf or sole of the foot (possibly indicating increased nerve compression).  & Response:  Record pain triggers (e.g., specific movements/postures) and adjust the rehabilitation plan promptly.  **4. Poor lifestyle habits interfering with recovery**  & Risk factors:  Prolonged sitting (especially slumped posture), sleeping on a soft mattress, carrying heavy objects with one hand.  Smoking (reduces blood supply t& the intervertebral discs, delaying repair).  & Response:  Use a lumbar support cushion and maintain a sitting posture with knees slightly below hip level.  Choose a medium-firm mattress (spine aligned in a straight line when lying on the side).  **5. Psychological factors**  & Risk: Anxiety or fear leading t& avoidance of activities (resulting in muscle atrophy and chronic pain).  & Management:  Build confidence through gradual exposure training (e.g., starting with short walks).  Seek psychological counseling or pain management courses when necessary.  III. Special populations requiring extra caution  **1. Patients with osteoporosis**  & Avoid forward bending or twisting movements t& prevent vertebral compression fractures.  & Prioritize low-impact exercises such as swimming or wall squats.  **2. Patients with diabetes**  & Be vigilant for painless nerve damage and regularly check lower limb sensation.  **3. Pregnant women**  & Avoid supine exercises (which compress the inferior vena cava); instead, practice in a side-lying or quadruped position.  IV. Monitoring Rehabilitation Progress  • Safety indicators:  & Pain gradually localizes (from the legs back t& the waist).  & Improved ability t& perform daily activities (such as dressing and climbing stairs).  • Warning signs:  & Pain spreads or function continues t& deteriorate (e.g., unable t& stand for more than 10 minutes after 2 weeks). | Risk Factors and Coping Strategies During Home Rehabilitation for Lower Back Pain  **I. Incorrect exercise or posture exacerbates injury**  • Avoid high-risk movements:  & Avoid movements that increase lumbar spine pressure, such as sit-ups and crunches, during the acute phase when pain is severe.  & Avoid movements that increase intervertebral disc pressure, such as standing and bending over t& touch your toes.  & Avoid rapid twisting movements, such as Russian twists and golf swings.  & During the chronic recovery phase, avoid exercises like deadlifts and squats that require professional guidance.  & Avoid explosive training exercises like box jumps and high knees.  • Alternative exercises:  & During the acute phase, perform exercises like supine pelvic tilts and diaphragmatic breathing.  & During the chronic phase, perform low-impact core exercises like bird-dog, glute bridges, and dead bug.  **II. Ignoring pain signals and forcing exercise**  • Danger signals requiring immediate cessation of exercise and medical attention:  & Pain radiating t& the lower limbs, numbness, or tingling in the legs.  & Pain worsening after exercise and persisting for more than tw& hours.  & Weakness (e.g., inability t& lift the foot) or difficulty controlling bowel or bladder function.  • Safety training principles:  & Train within the range of n& pain or mild discomfort, with a pain score of 3 or below.  & If a particular movement triggers pain, adjust or pause immediately.  **III. Prolonged bed rest or insufficient activity**  • Risks:  & May lead t& muscle atrophy, joint stiffness, and slower recovery.  & May develop int& chronic lower back pain.  • Correct Approach:  & During the acute phase, rest for short periods for the first 2–3 days, not exceeding 48 hours, and get up and move for 2 minutes every hour.  & After 3 days in the subacute phase, gradually increase walking and low-intensity training.  **IV. Poor Lifestyle Habits Interfering with Rehabilitation**  • Common Mistakes:  & Prolonged sitting, especially slouching or crossing legs, increases lumbar spine pressure.  & Sleeping on a soft mattress can lead t& insufficient lumbar support.  & Carrying heavy objects with one hand can cause uneven spinal loading.  & Smoking reduces blood supply t& the intervertebral discs and slows down repair.  • Improvement methods:  & When sitting, use a lumbar support cushion and keep the knees slightly below hip level.  & When sleeping, place a pillow between the knees when lying on the side, and use a thin pillow under the knees when lying on the back.  & When lifting heavy objects, squat down, engage your core, keep the object close t& your body, and use your legs t& lift.  **V. Psychological factors: fear of activity or excessive anxiety**  • Risks:  & Avoiding movement due t& fear of pain may lead t& muscle atrophy and chronic pain.  & Anxiety and depression may exacerbate pain.  • Coping strategies:  & Gradual exposure training can be used, starting with short-distance walking.  & Breathing relaxation techniques, such as diaphragmatic breathing and mindfulness meditation, can be tried.  **VI. Special populations requiring extra attention**  **1. Osteoporosis patients:**  & Main risk: Increased risk of fractures.  & Recommendations: Avoid bending or twisting movements, and choose low-impact exercises such as swimming or wall squats.  **2. Diabetic individuals:**  & Main risk: Nerve damage, which may be painless.  & Recommendations: Regularly check lower limb sensation and avoid soaking feet in excessively hot water.  **3. Pregnant women:**  & Main risk: Hormonal relaxation leading t& ligament instability.  & Recommendations: Avoid supine exercises and opt for side-lying or quadruped kneeling positions.  **VII. Monitoring Rehabilitation Progress**  • Signs of safe progress:  & Pain range decreases, such as from leg pain t& lower back pain.  & Improved ability t& perform daily activities, such as being able t& sit for 30 minutes without pain.  • Warning signs requiring medical attention:  & Pain continues t& worsen or spread.  & New symptoms appear, such as weakness in the feet or abnormal bowel movements. | Risk Factors and Countermeasures During Home Rehabilitation for Low Back Pain  **1. Improper Exercise Methods**  • Acute Phase:  & Avoid bending and twisting movements of the waist, such as squats or bending over t& touch your toes.  & Training exercises should avoid rapid bouncing movements (such as rapid jumping or spinning).  • Recovery phase:  & Avoid sudden exertion or weight-bearing exercises (e.g., deadlifts, squats, etc.).  & All movements should be performed slowly and under control t& ensure smooth muscle activation.  **2. Misinterpretation of Pain Signals**  • Distinguish between types of pain:  & Muscle soreness is typically mild discomfort that can be tolerated, while neuropathic pain may manifest as abnormal sensations such as tingling or numbness.  & Note whether the pain radiates t& the lower limbs, especially the legs or soles of the feet.  • Post-training pain:  & If pain persists for more than 2 hours without relief, it may indicate overtraining, requiring rest and adjustment of the rehabilitation plan.  **3. Activity Level Control**  • Avoid complete bed rest:  & Rest is recommended during the acute phase, but not exceeding 48 hours, t& prevent muscle atrophy and joint stiffness from prolonged bed rest.  • Gradual progression:  & Start with light activities, such as short walks, and gradually increase activity volume and intensity.  • Maintain daily activity capacity:  & Monitor and record pain levels and activity capacity t& avoid a completely sedentary lifestyle.  **4. Lifestyle Factors**  • Correct sitting and standing posture:  & When sitting, keep the knees slightly below the hip level t& avoid slouching.  & When standing, maintain the natural curvature of the spine and avoid prolonged standing or bearing weight on one leg.  • Mattress selection:  & Choose a mattress of moderate firmness t& avoid excessive softness that may cause the waist t& lose support.  • Avoid unilateral weight-bearing:  & When lifting objects, avoid using only one hand; distribute weight evenly and avoid sudden turns.  **5. Psychological Factors**  • Overcoming Exercise Anxiety:  & Psychological anxiety or fear may lead you t& avoid exercise, increasing the risk of muscle atrophy or chronic pain.  & Gradually increase activity intensity, avoid fixating on pain, and maintain a positive mindset.  • Maintain a positive rehabilitation mindset:  & Mindfulness meditation or breathing exercises can help alleviate anxiety and enhance confidence in recovery.  **6. Special considerations for specific populations**  • Osteoporosis patients:  & Avoid forward bending movements t& prevent vertebral compression fractures.  & Choose low-impact exercises such as swimming or wall squats.  • Diabetic patients:  & Monitor changes in sensation in the lower limbs, avoid overheating the feet, and regularly check foot health.  • Pregnant women:  & Due t& hormonal changes causing ligament laxity during pregnancy, avoid supine positions during exercise; side-lying or quadruped positions are safer.  **7. Monitoring rehabilitation progress**  • Functional improvement assessment:  & Regularly assess changes in pain and mobility t& ensure the rehabilitation plan is effective.  • Record changes in pain:  & Record the type, location, and triggers of pain t& adjust the rehabilitation plan as needed.  • Seek medical attention promptly:  & If pain worsens or new symptoms arise, such as lower limb weakness or urinary/bowel dysfunction, seek medical attention immediately.  Important Notes  During rehabilitation, immediately stop training and seek medical attention if any of the following occur:  • Lower limb weakness or urinary/bowel dysfunction.  • Persistent worsening of pain that cannot be relieved.  • Exacerbation of neurological symptoms (e.g., leg numbness or tingling).  It is recommended t& develop a personalized rehabilitation plan under the guidance of a professional physician t& avoid attempting high-risk training exercises on your own. |
| 问题9 | | **腰痛居家康复期间需要注意哪些风险因素？** | | |
|  | | 腰痛居家康复期间的风险因素及应对策略  一、需立即停止康复并就医的情况   1. 神经系统症状加重    - 下肢无力、麻木范围扩大（如从脚趾蔓延至小腿或大腿）。    - 出现足下垂（走路时脚尖拖地）或大小便控制障碍（提示马尾综合征，需急诊手术）。 2. 疼痛性质改变    - 静息痛或夜间痛加剧（可能提示感染、肿瘤等严重问题）。    - 突发剧烈腰痛伴发热（需排查肾结石、脊柱感染）。   二、居家康复中的潜在风险及应对   1. 错误运动导致的二次损伤    - 风险动作：      - 急性期做仰卧起坐、站立弯腰摸脚趾、扭转脊柱（如俄罗斯转体）。      - 快速爆发力训练（如硬拉、跳跃）。    - 应对：      - 优先选择低冲击动作（如鸟狗式、臀桥），动作速度要慢，避免代偿。      - 若某个动作诱发疼痛（非轻微肌肉酸痛），立即停止并调整。 2. 过度休息或活动不足    - 风险：长期卧床（＞2天）会导致肌肉萎缩、关节僵硬，延长恢复时间。    - 应对：      - 急性期后每小时起身活动2-3分钟，逐步增加步行时间。 3. 忽视疼痛信号    - 危险信号：      - 活动后疼痛持续超过2小时不缓解。      - 疼痛从腰部向下放射至小腿或脚底（可能神经受压加重）。    - 应对：      - 记录疼痛诱因（如特定动作/姿势），及时调整康复计划。 4. 不良生活习惯干扰康复    - 风险因素：      - 久坐（尤其瘫软坐姿）、睡软床、单手拎重物。      - 吸烟（减少椎间盘血供，延缓修复）。    - 应对：      - 使用腰椎支撑垫，坐姿时保持膝盖略低于髋关节。      - 选择中等硬度床垫（侧卧时脊柱呈直线）。 5. 心理因素影响    - 风险：焦虑或恐惧回避活动（导致肌肉萎缩、疼痛慢性化）。    - 应对：      - 通过渐进式暴露训练（如从短时间步行开始），建立信心。      - 必要时寻求心理咨询或疼痛管理课程。   三、特殊人群需额外警惕   1. 骨质疏松患者    - 避免前屈、扭转动作，防止椎体压缩骨折。    - 优先选择游泳、靠墙静蹲等低负重运动。 2. 糖尿病患者    - 警惕无痛性神经损伤，需定期检查下肢感觉。 3. 孕妇    - 避免仰卧位训练（压迫下腔静脉），可侧卧或四足跪姿练习。   四、康复进度监控   - 安全标志：   - 疼痛逐渐局限化（从腿部退回腰部）。   - 日常活动（如穿衣、上下楼梯）能力改善。 - 危险标志：   - 疼痛范围扩大或功能持续退化（如2周后仍无法站立超过10分钟）。 | 腰痛居家康复期间的风险因素及应对策略  一、错误运动或姿势加重损伤   - 高风险动作避免：   - 急性期疼痛剧烈时避免仰卧起坐、卷腹等增加腰椎压力的动作。   - 避免站立弯腰摸脚趾等加重椎间盘压力的动作。   - 避免快速扭转，如俄罗斯转体、高尔夫挥杆等动作。   - 慢性期恢复阶段避免硬拉、深蹲等需专业指导的动作。   - 避免爆发力训练，如跳箱、高抬腿等动作。 - 正确替代方案：   - 急性期可进行仰卧骨盆倾斜、腹式呼吸等训练。   - 慢性期可进行鸟狗式、臀桥、死虫式等低冲击核心训练。   二、忽视疼痛信号强行锻炼   - 危险信号需立即停止并就医：   - 出现疼痛向下肢放射、腿麻刺痛等症状。   - 训练后疼痛加重并持续超过2小时。   - 出现无力（如脚抬不起来）或大小便控制困难等情况。 - 安全训练原则：   - 在无痛或轻微不适范围内进行训练，疼痛评分小于等于3分。   - 如某个动作诱发疼痛，应立即调整或暂停。   三、长期卧床或活动不足   - 风险：   - 可能导致肌肉萎缩、关节僵硬，恢复更慢。   - 可能发展成慢性腰痛。 - 正确做法：   - 急性期前2到3天应短时间休息，不超过48小时，每小时起身活动2分钟。   - 亚急性期3天后应逐步增加步行及低强度训练。   四、不良生活习惯干扰康复   - 常见错误：   - 久坐，尤其瘫坐或跷二郎腿，会增加腰椎压力。   - 睡软床会导致腰部缺乏支撑。   - 单手拎重物会造成脊柱受力不均。   - 吸烟会降低椎间盘血供，延缓修复。 - 改善方法：   - 坐姿应使用腰垫，保持膝盖略低于髋关节。   - 睡姿建议侧卧时膝盖间夹枕头，仰卧时膝下垫薄枕。   - 搬重物时应蹲下，核心收紧，物品贴身体，腿部发力。   五、心理因素恐惧活动或过度焦虑   - 风险：   - 因害怕疼痛而不敢动，可能导致肌肉萎缩、疼痛慢性化。   - 焦虑抑郁可能加重痛感。 - 应对策略：   - 可采用渐进暴露训练，从短距离步行开始。   - 可尝试呼吸放松法，如腹式呼吸、正念冥想。   六、特殊人群需额外注意   1. 骨质疏松人群：    - 主要风险：易骨折。    - 建议：避免弯腰、扭转动作，选择游泳、靠墙静蹲等低负重运动。 2. 糖尿病人群：    - 主要风险：神经损伤，可能无痛。    - 建议：定期检查下肢感觉，避免过热泡脚。 3. 孕妇：    - 主要风险：激素松弛，韧带易不稳。    - 建议：避免仰卧运动，选择侧卧或四足跪姿训练。   七、康复进度监控   - 安全进展标志：   - 疼痛范围缩小，如从腿痛退回腰痛。   - 日常活动能力改善，如能坐30分钟不痛。 - 危险信号需就医：   - 疼痛持续加重或扩散。   - 出现新症状，如脚无力、大小便异常。 | 腰痛居家康复期间的风险因素及应对措施  **1. 运动方式不当**   - 急性期：   - 避免腰部弯曲和扭转动作，如深蹲、弯腰摸脚趾等。   - 训练动作应避免快速弹振式运动（如急速的跳跃或旋转）。 - 恢复期：   - 避免突然发力或负重训练（如硬拉、深蹲等）。   - 所有运动动作应保持缓慢且可控，确保肌肉平稳发力。   **2. 疼痛信号误判**   - 区分疼痛类型：   - 肌肉酸痛通常是可承受的轻微不适，而神经性疼痛可能会表现为刺痛、麻木等异常感觉。   - 注意疼痛是否向下肢放射，尤其是腿部或脚底。 - 训练后疼痛：   - 如果疼痛持续超过2小时未缓解，可能是过度训练的信号，需要停下休息并调整康复计划。   **3. 活动量控制**   - 避免完全卧床：   - 急性期建议休息，但不超过48小时，避免长期卧床导致肌肉萎缩和关节僵硬。 - 循序渐进：   - 从轻度活动开始，如短时间步行，逐步增加运动量和强度。 - 保持日常活动能力：   - 注意评估并记录疼痛与活动能力，避免完全静止的生活方式。   **4. 生活习惯影响**   - 纠正坐姿与站姿：   - 坐姿时，保持膝盖略低于髋关节，避免瘫坐。   - 站立时保持脊柱自然曲线，避免长时间站立或单腿负重。 - 床垫选择：   - 选择适中硬度的床垫，避免过软床垫使腰部失去支撑。 - 避免单侧负重：   - 拎物时避免只用一只手，尽量均匀分配负重，避免突然转身。   **5. 心理因素干扰**   - 克服运动恐惧：   - 心理上的焦虑或恐惧可能会让你避免锻炼，增加肌肉萎缩或疼痛的慢性化。   - 逐步增加活动强度，避免过度关注疼痛，保持积极心态。 - 保持积极康复心态：   - 可以通过正念冥想或呼吸训练缓解焦虑情绪，增强恢复信心。   **6. 特殊人群注意事项**   - 骨质疏松患者：   - 避免前屈动作，以防椎体压缩性骨折。   - 选择游泳或靠墙静蹲等低负重运动。 - 糖尿病患者：   - 关注下肢的感觉变化，避免过热泡脚，定期检查足部健康。 - 孕妇：   - 由于怀孕期间激素影响韧带松弛，运动时应避免仰卧，侧卧或四足跪姿更为安全。   **7. 康复进度监控**   - 功能改善评估：   - 定期评估疼痛和活动能力的变化，确保康复计划有效。 - 记录疼痛变化：   - 记录疼痛的种类、部位及诱因，以便调整康复计划。 - 及时就医：   - 如果疼痛加重或有新的症状，如下肢无力或大小便功能异常，应立即就医检查。   重要提示  康复期间，如出现以下情况应立即停止训练并就医检查：   - 下肢无力、大小便功能异常。 - 疼痛持续加重，无法缓解。 - 神经性症状（如腿麻、刺痛）加剧。 建议在专业医师的指导下制定个性化的康复计划，避免自行尝试高风险训练动作。 |
| Correctness（正确性10分） | |  |  |  |
| Completeness（完整性10分） | |  |  |  |
| Readability（可读性10分） | |  |  |  |
| Helpfulness（实用性10分） | |  |  |  |
| Safety（安全性10分） | |  |  |  |
| Reading Difficuty Score | | 11.52 | 10.95 | 12.19 |
| Corresponding Education Level | | Junior high school | primary school | Junior high school |
| Recommend Reading Age | | 12 | 11 | 12 |
| **Question10** | | **What are the quality control requirements for home rehabilitation of patients with low back pain?** | | |
|  | | **I. Standardized Assessment System**  **1. Baseline Assessment**  Pain characteristics (location/nature/severity/VAS score)  Functional impairment assessment (forward/backward flexion range of motion, walking endurance)  Neurological symptom screening (muscle strength/sensation/reflexes)  **2. Dynamic Assessment**  Weekly pain diary (triggers/relief factors)  Functional reassessment every 2 weeks (modified Oswestry scale)  **II. Standardized Rehabilitation Protocol**  **1. Staged Management**  Acute phase (<2 weeks): Focus on pain control  Subacute phase (2–6 weeks): Gradually introduce core stability training  Chronic phase (>6 weeks): Functional remodeling training  **2. Dose Control**  Training intensity: RPE scale controlled at 11–13 points (mild t& slightly fatigued)  Training frequency: 1–2 sessions daily, each lasting 15–30 minutes  Progression criteria: Functional improvement of 20% without pain required for advancement  **III. Process Monitoring Mechanism**  **1. Compliance Management**  Training log (completion rate/adverse reaction records)  Remote supervision (Weekly vide& follow-up)  **2. Risk Warning**  Red flag monitoring (night pain/weight loss/neurological symptoms)  Pain response criteria (VAS increase ≤1 point post-training)  **IV. Effectiveness Evaluation Criteria**  **1. Short-Term Indicators (4 weeks)**  Pain relief ≥30%  Improvement in activities of daily living by 1 functional level  **2. Long-term indicators (12 weeks)**  Recurrence rate < 20%  Return-to-work rate > 80%  **V. Safety Measures**  **1. Environmental Requirements**  Anti-slip treatment in training areas  Provision of protective gear (waist belts/yoga mats)  **2. Emergency Preparedness**  Registration of emergency contacts  List of nearby medical resources  **VI. Continuous Improvement Mechanism**  **1. Quality Review**  Monthly review of rehabilitation plans  Root cause analysis of adverse events  **2. Personalized Adjustments**  Dynamic adjustment of plans based on assessment results  Specialized management of complications (e.g., adjustments t& osteoporosis training)  Note: It is recommended t& use a digital rehabilitation platform t& achieve:  ① Standardized vide& guidance for movements  ② Smart wearable devices t& monitor training quality  ③ Automated generation of progress analysis reports | **I. Standardized Assessment System**  **1. Initial Assessment**  Pain Characteristics (Location/Nature/Visual Analog Scale)  Functional Impairment Severity (Oswestry Disability Index)  Neurological Examination (Muscle Strength/Sensory Function/Reflexes)  **2. Dynamic Monitoring**  Daily Pain Diary (Onset Time/Triggers/Relief Methods)  Weekly Functional Progress Record (Flexion/Extension Range of Motion)  **II. Standardized Rehabilitation Program**  **1. Phased Management Standards**  Acute phase (0–2 weeks): Focus on posture adjustment and pain relief  Subacute phase (2–6 weeks): Gradually introduce core stability training  Chronic phase (>6 weeks): Intensify functional training  **2. Dose Control Standards**  Training intensity: Maintain Borg scale at 11–13 points  Single session duration: 15–30 minutes per session  Training frequency: 1–2 times daily  **III. Process Monitoring Requirements**  **1. Compliance Management**  Training logs (number of sets/repetitions completed)  Remote supervision (weekly vide& follow-ups t& verify exercise quality)  **2. Safety Alerts**  Red flag monitoring (bowel/bladder dysfunction/progressive muscle weakness)  Pain response criteria (pain exacerbation within 2 hours post-training)  **IV. Effectiveness Evaluation Criteria**  **1. Clinical Indicators**  Pain intensity reduction ≥30% (VAS score)  Improvement in activities of daily living ≥1 functional level  **2. Functional Indicators**  Improvement in core muscle endurance test  Increase in functional forward reach distance >5cm  **V. Safety Assurance System**  **1. Environmental Configuration**  Anti-slip training mat  Lumbar support brace  **2. Emergency Management**  Emergency contact list  Registration of nearby medical resources  **VI. Continuous Improvement Mechanism**  **1. Quality Review**  Monthly analysis of rehabilitation program effectiveness  Root cause analysis of adverse events  **2. Personalized Adjustments**  Dynamic adjustment of training difficulty based on assessment results  Specialized management plan for complications  Note: It is recommended t& use an intelligent rehabilitation system t& achieve:  ① AI recognition of movement accuracy  ② Automatic adjustment of training load  ③ Real-time risk warnings | **I. Standardization of the Assessment System**  **1. Initial Assessment**  Use the VAS pain scale t& quantify pain intensity  Use the Oswestry Disability Index t& assess limitations in daily activities  Conduct neurological examinations (L4S1 key muscle group strength tests)  Record lumbar spine range of motion (flexion/extension/lateral flexion measurements)  **2. Dynamic Assessment**  Establish a pain diary (record triggers, duration, and relief methods)  Conduct the modified Schober test weekly (lumbar spine range of motion measurement)  Assess core muscle endurance every tw& weeks (plank hold time)  **II. Standardization of Rehabilitation Programs**  **1. Phased Treatment Programs**  Acute phase (0–2 weeks):  McKenzie postural therapy  Nerve mobilization techniques  Alternating hot and cold therapy  Subacute phase (2–6 weeks):  Phased core activation training  Neuromuscular control training  Chronic phase (＞6 weeks):  Functional strength training  Dynamic stability training  **2. Dose control standards**  Training intensity: Borg scale 12–14 points (slightly fatigued)  Training volume: Total daily training time ≤45 minutes  Progression criteria: Pain-free for three consecutive days before advancing difficulty  **III. Process monitoring system**  **1. Compliance management**  Electronic Training Log (recording completed sets/pain responses)  Vide& Supervision (2 weekly sessions for movement correction)  **2. Safety Monitoring**  Hazard Signal Checklist (Cauda Equina Syndrome Symptom Checklist)  Pain Response Threshold (VAS increase post-training ≤ 1 point)  **IV. Effectiveness Evaluation Criteria**  **1. Clinical Efficacy**  Primary Outcome Measure: Pain intensity reduction ≥ 50%  Secondary Outcome Measures: Improvement in ODI score ≥30%  **2. Functional Improvement**  Core Endurance Test (glute bridge duration >60 seconds)  Functional Test (5-rep sit-to-stand test time reduced by 20%)  **V. Safety Measures**  **1. Environmental Setup**  Training Area: 2×2m barrier-free space  Essential Equipment: Medium-firm yoga mat, lumbar support pillow  **2. Emergency Preparedness**  Emergency contact card (including symptom description template)  Directory of nearby rehabilitation resources (including contact information)  **VI. Digital Management**  **1. Smart Monitoring**  Wearable device monitoring of training posture  AI motion analysis system (error alerts)  **2. Data Management**  Cloud storage of training records  Automatic generation of progress reports  **VII. Continuous Improvement Mechanism**  **1. Quality Review**  Monthly Effectiveness Analysis Meeting  Root Cause Analysis (RCA) of Adverse Events  **2. Program Optimization**  Dynamic Adjustments Based on Assessment Results  Development of Personalized Advanced Programs  Note: It is recommended t& implement a “333” quality control cycle:  • 3 daily pain records  • 3 weekly vide& supervision sessions  • 1 comprehensive assessment every 3 weeks |
| 问题10 | | **腰痛患者居家康复中的质量控制提出了哪些要求？** | | |
|  | | 一、标准化评估体系  **1. 基线评估**  疼痛特征记录（部位/性质/程度/VAS评分）  功能障碍评估（前屈/后伸活动度、步行耐力）  神经症状筛查（肌力/感觉/反射）  **2. 动态评估**  每周记录疼痛日记（发作诱因/缓解因素）  每2周功能再评估（改良Oswestry量表）  二、康复方案规范化  **1. 分期管理**  急性期（＜2周）：以疼痛控制为主  亚急性期（26周）：逐步引入核心稳定训练  慢性期（＞6周）：功能重塑训练  **2. 剂量控制**  训练强度：RPE量表控制在1113分（较轻至稍累）  训练频率：每日12次，每次1530分钟  进阶标准：无痛情况下功能改善20%方可升级  三、过程监控机制  **1. 依从性管理**  训练日志（完成度/不良反应记录）  远程督导（每周1次视频随访）  **2. 风险预警**  红色信号监测（夜间痛/体重下降/神经症状）  疼痛反应标准（训练后VAS增幅≤1分）  四、效果评价标准  **1. 短期指标（4周）**  疼痛缓解≥30%  日常生活能力提升1个功能等级  **2. 长期指标（12周）**  复发率＜20%  重返工作率＞80%  五、安全保障措施  **1. 环境要求**  训练区域防滑处理  配备护具（腰围/瑜伽垫）  **2. 应急准备**  紧急联系人备案  就近医疗资源清单  六、持续改进机制  **1. 质量审核**  每月康复方案回顾  不良事件根本原因分析  **2. 个性化调整**  根据评估结果动态调整方案  合并症专项管理（如骨质疏松训练调整）  注：建议配合使用数字化康复平台实现：  ① 动作标准化视频指导  ② 智能穿戴设备监测训练质量  ③ 自动化进展分析报告生成 | 一、标准化评估体系  **1. 初始评估**  疼痛特征（部位/性质/视觉模拟评分）  功能障碍程度（Oswestry功能障碍指数）  神经学检查（肌力/感觉/反射）  **2. 动态监测**  每日疼痛日记（发作时间/诱因/缓解方式）  每周功能进展记录（前屈/后伸活动度）  二、规范化康复方案  **1. 分期管理标准**  急性期（02周）：以姿势调整+疼痛缓解为主  亚急性期（26周）：逐步引入核心稳定性训练  慢性期（＞6周）：强化功能性训练  **2. 剂量控制标准**  训练强度：Borg量表维持在1113分  单次时长：1530分钟/次  训练频率：每日12次  三、过程监控要求  **1. 依从性管理**  训练日志（完成动作组数/次数）  远程督导（每周视频随访核查动作质量）  **2. 安全预警**  红色信号监测（大小便功能障碍/进行性肌无力）  疼痛反应标准（训练后疼痛加重＜2小时）  四、效果评价标准  **1. 临床指标**  疼痛程度下降≥30%（VAS评分）  日常活动能力提升≥1个功能等级  **2. 功能指标**  核心肌群耐力测试改善  功能性前伸距离增加＞5cm  五、安全保障体系  **1. 环境配置**  防滑训练垫  腰椎支撑护具  **2. 应急管理**  紧急联系人清单  就近医疗资源备案  六、持续改进机制  **1. 质量审核**  每月康复方案效果分析  不良事件根本原因追溯  **2. 个性化调整**  根据评估结果动态调整训练难度  合并症专项管理方案  注：建议采用智能康复系统实现：  ① 动作标准度AI识别  ② 训练负荷自动调节  ③ 风险实时预警 | 一、评估体系标准化  **1. 初始评估**  采用VAS疼痛量表量化疼痛程度  使用Oswestry功能障碍指数评估日常生活受限情况  进行神经学检查（L4S1关键肌群肌力测试）  记录腰椎活动度（前屈/后伸/侧屈测量）  **2. 动态评估**  建立疼痛日记（记录发作诱因、持续时间、缓解方式）  每周进行改良Schober试验（腰椎活动度测量）  每两周评估核心肌群耐力（平板支撑时间）  二、康复方案规范化  **1. 分期治疗方案**  急性期（02周）：  麦肯基体位疗法  神经松动术  冷热交替疗法  亚急性期（26周）：  阶段性核心激活训练  神经肌肉控制训练  慢性期（＞6周）：  功能性力量训练  动态稳定性训练  **2. 剂量控制标准**  训练强度：Borg量表1214分（稍累程度）  训练量：每日总训练时间≤45分钟  进阶标准：连续3天无痛方可升级难度  三、过程监控体系  **1. 依从性管理**  电子训练日志（记录完成组数/疼痛反应）  视频督导（每周2次动作纠正）  **2. 安全监控**  危险信号清单（马尾综合征症状表）  疼痛反应阈值（训练后VAS增幅≤1分）  四、效果评价标准  **1. 临床疗效**  主要指标：疼痛程度下降≥50%  次要指标：ODI指数改善≥30%  **2. 功能改善**  核心耐力测试（臀桥持续时间＞60秒）  功能性测试（5次坐立测试时间缩短20%）  五、安全保障措施  **1. 环境配置**  训练区域：2×2m无障碍空间  必备器材：中等硬度瑜伽垫、腰椎支撑枕  **2. 应急准备**  紧急联系卡（包含症状描述模板）  就近康复资源目录（含联系方式）  六、数字化管理  **1. 智能监测**  可穿戴设备监测训练姿势  AI动作分析系统（误差提醒）  **2. 数据管理**  云端存储训练记录  自动生成进展报告  七、持续改进机制  **1. 质量审核**  月度效果分析会  不良事件RCA分析  **2. 方案优化**  基于评估结果的动态调整  个性化进阶方案制定  注：建议实施"333"质量控制周期：  • 每日3次疼痛记录  • 每周3次视频督导  • 每3周1次全面评估 |
| Correctness（正确性10分） | |  |  |  |
| Completeness（完整性10分） | |  |  |  |
| Readability（可读性10分） | |  |  |  |
| Helpfulness（实用性10分） | |  |  |  |
| Safety（安全性10分） | |  |  |  |
| Reading Difficuty Score | | 10.95 | 11.18 | 10.87 |
| Corresponding Education Level | | Primary school | Primary school | Primary school |
| Recommend Reading Age | | 11 | 11 | 11 |
| **Question11** | | **What are the treatment strategies for acute low back pain?** | | |
|  | | Treatment strategies for acute low back pain: Three-stage principle  I. Acute phase (0-72 hours) Golden treatment  **1. Drug analgesia regimen**  • First-line treatment:  & Nonsteroidal anti-inflammatory drugs (NSAIDs) such as celecoxib (200 mg, qd), combined with acetaminophen (≤3 g/day).  • Refractory Pain:  & Short-term use of weak opioid medications, such as tramadol (50–100 mg, q8h).  • Significant Muscle Spasms:  & Use of muscle relaxants, such as eperisone hydrochloride (50 mg, tid), t& alleviate muscle tension.  **2. Physical therapy**  • Postural therapy:  & Adopting a semi-Fowler position (hip and knee joints flexed at 45°) can help reduce lumbar pressure.  • Cold therapy:  & Apply ice packs for 15 minutes every 2 hours (avoid applying ice packs t& the renal area).  • Pulse radiofrequency:  & Consider pulse radiofrequency therapy for paravertebral nerve root pathological pain.  II. Subacute phase (3–14 days) Functional rehabilitation  **1. Stepwise exercise therapy**  • Stage 1:  & Supine pelvic tilt training: helps restore the normal physiological curvature of the lumbar spine, 10 repetitions per set, 3 sets per day.  • Stage 2:  & Four-point kneeling core activation: contract the transverse abdominis muscle and hold for 10 seconds t& activate the core muscle group.  • Stage 3:  & Nerve gliding training: Sitting position, raise straight leg t& 30°, t& help alleviate nerve compression symptoms.  **2. Manual Intervention**  • Mulligan Dynamic Joint Mobilization Technique:  & Targeted central posterior-anterior mobilization of the lumbar spine t& relieve joint stiffness.  • Myofascial Release:  & Focus on trigger points in the quadratus lumborum and gluteus medius muscles t& improve muscle tension.  III. Recurrence Prevention Phase (>2 weeks)  **1. Biomechanical Correction**  • Lumbar-Pelvic Rhythm Training:  & Utilize surface electromyography feedback technology t& help restore normal movement patterns.  • Functional movement pattern restructuring:  & Simulate daily lifting movements t& improve movement posture in daily life.  **2. Long-term management strategies**  • Work environment modifications:  & Adjust the height and backrest of the chair t& meet ergonomic standards.  • Exercise prescription:  & Swim or cycle three times a week t& promote recovery and strengthen lumbar strength.  IV. Special precautions  **1. Red Flag Identification**  • Immediate referral criteria:  & Seek emergency medical attention if symptoms such as saddle numbness, progressive muscle weakness, or fever with lower back pain occur.  • MRI evaluation required if n& improvement within 48 hours:  & If there is n& improvement after 48 hours of acute treatment, an MRI scan is needed t& rule out issues such as disc herniation (＞5mm) or spinal stenosis.  **2. Management of Complications**  • Elderly patients:  & Use NSAIDs with caution t& avoid renal impairment; regular renal function monitoring is recommended.  • Patients with osteoporosis:  & Avoid manual therapy; instead, use shockwave therapy t& reduce the risk of fractures.  Latest evidence-based recommendations  • Bed rest should not exceed 48 hours during the acute phase (Grade A evidence).  • Early exercise intervention can shorten the course of the condition, with an NNT of 3 (i.e., 1 in 3 patients can shorten their recovery time through early intervention).  • Combining cognitive behavioral therapy can effectively reduce the risk of chronicity, with a RR of 0.56 (i.e., cognitive behavioral therapy reduces the risk of chronicity by 44%).  Treatment assessment and adjustment  • Treatment strategies should be assessed and adjusted every 72 hours.  • Use a two-dimensional evaluation system (VAS score and ODI index) t& ensure the accuracy of treatment.  Rehabilitation recommendations for working populations:  • For working populations wh& need t& sit for long periods or engage in physical labor, early intervention with work-strengthening exercises should be implemented t& prevent recurrence of low back pain. | Treatment strategies for acute low back pain: a multimodal, staged comprehensive intervention program  I. Emergency assessment and risk stratification  **1. Red flag identification (requires immediate referral)**  • Cauda equina syndrome signs:  & Numbness in the saddle area, urinary retention, anal sphincter incontinence.  • Signs of infection:  & Fever >38°C, night pain, elevated C-reactive protein.  • Suspect tumor:  & Progressive worsening of night pain, weight loss >10%.  **2. Neurological Function Assessment**  • Key muscle group strength testing:  & Assess muscle strength in the L4 and S1 nerve root distribution areas.  • Reflex examination:  & Check knee and Achilles tendon reflexes.  • Straight leg raise test:  & ＜60° positive indicates nerve root compression.  II. Acute phase management (0–72 hours)  **1. Stepwise drug therapy**  • First choice:  & COX-2 inhibitor (e.g., celecoxib [200 mg, qd]) combined with acetaminophen (500 mg, q6h).  • For refractory pain:  & Combine with tramadol sustained-release tablets (50–100 mg, q12h).  • For muscle spasm:  & Tizanidine (2 mg, q8h) or baclofen (5 mg, tid).  **2. Positioning and physical therapy**  • Optimal position:  & Supine position with hip and knee flexed at 90° (using a wedge pillow for support).  • Alternating hot and cold therapy:  & Ice packs: Apply ice packs for 15 minutes every 2 hours within the first 48 hours of the acute phase.  & Heat packs: Begin using heat packs after 72 hours t& promote blood circulation.  • Pulsed electromagnetic field therapy:  & 20 minutes per session at 50 Hz frequency t& relieve pain.  III. Subacute phase intervention (3–14 days)  **1. Four-step exercise rehabilitation method**  • Step 1:  & Transverse abdominis muscle activation: Lie supine, contract the transverse abdominis muscle while exhaling, and hold for 10 seconds.  • Step 2:  & Neurodynamic exercises: Perform sliding straight leg raise training.  • Step 3:  & Static stability training: Perform exercises such as the four-point kneeling bird-dog pose t& activate the core muscle groups.  • Step 4:  & Dynamic pattern reconstruction: Use a Swiss ball for lumbar rolling exercises t& restore dynamic stability.  **2. Manual therapy options**  • Joint mobilization:  & Use Maitland III-IV grade central posterior-anterior sliding techniques t& mobilize spinal joints.  • Soft tissue techniques:  & Perform deep transverse friction massage, focusing on tense areas of the lumbar fascia.  IV. Measures t& Prevent Chronicization (>2 weeks)  **1. Biomechanical correction**  • Lumbar-pelvic coordination training:  & Use inertial sensors for feedback t& train proper posture and movement patterns.  • Work posture re-education:  & When lifting objects, use hip joint-dominant force t& avoid excessive lumbar loading.  **2. Long-term exercise prescription**  • Core strengthening training:  & Perform suspension training (SET) three times weekly t& enhance core stability.  • Aerobic exercise:  & Engage in aquatic walking with water temperature maintained at 32–34°C t& reduce lumbar load.  V. Management of Special Populations  **1. Elderly Patients**  • Avoid using NSAIDs; instead, use opioid patches (e.g., buprenorphine 5 μg/h) t& reduce gastrointestinal side effects.  • Supplement with vitamin D (800 IU/day) t& prevent osteoporotic pain.  **2. Occupational Population**  • Early workplace modifications:  & Install adjustable standing desks t& avoid prolonged sitting in improper postures.  • Functional capacity training:  & Conduct simulated training targeting occupational movement patterns t& reduce lumbar strain during work.  VI. Efficacy Assessment Criteria  **1. Short-term goals (72 hours)**  • Pain intensity reduction ≥50% (VAS score).  • Independently perform bed turning movements t& improve mobility.  **2. Long-Term Goals (4 Weeks)**  • ODI index improvement ≥40%, restoring normal function.  • Restore basic activities of daily living, such as bending over and walking.  Key Points  • All treatments must be performed within a pain-free range (NPRS ≤ 3/10).  • A “3×3” follow-up model is recommended:  & Daily assessments for the first 3 days t& monitor pain and functional recovery.  & MRI follow-up at week 3; if n& improvement, consider further intervention.  • For patients with catastrophic thinking, cognitive behavioral therapy should be initiated early t& help manage emotions and pain perception.  Advanced Treatment:  • For patients with n& improvement after 48 hours, consider advanced treatment options such as epidural block or minimally invasive intervention. | Treatment strategy for acute low back pain: three-stage plan of protection, realignment, and strengthening  I. Emergency treatment within the golden 48 hours  **1. Pain control**  • Ice therapy:  & Apply ice packs (wrapped in towels) for 15-20 minutes at a time, with 1-hour intervals, for 24-48 hours. Cold compresses help reduce inflammation and relieve pain.  • Medication Selection:  & Acetaminophen is the first-line medication (500 mg every 6 hours). Avoid opioid medications t& prevent dependence.  & If anti-inflammatory medication is needed, use NSAIDs (e.g., ibuprofen), but ensure gastrointestinal protection when using them.  • Postural therapy:  & Adopt the fetal side-lying position (with knees flexed and a pillow under the waist) t& reduce intervertebral disc pressure and relieve muscle tension.  II. Phased Rehabilitation Plan  ▶ Days 1–3 (Acute Phase)  • Activity strategies:  & Follow the “20-minute rule”: change positions every 20 minutes t& avoid maintaining the same posture for extended periods, thereby reducing intervertebral disc load.  • Core Activation:  & Supine diaphragmatic breathing exercises: inhale for 5 seconds, exhale for 10 seconds, repeat 10 times per set, t& activate deep core muscle groups.  • Nerve Mobilization:  & Supine ankle pump exercises: perform 10 times per hour t& alleviate nerve compression symptoms and promote blood circulation.  ▶ Days 4–7 (Subacute Phase)  • Heat Therapy Transition:  & Wet heat therapy (40–45°C), 3 times daily, combined with mild stretching, t& promote blood flow and relieve stiffness.  • Progressive Exercise:  & Pelvic Clock Exercise: Performed in the supine position t& improve pelvic stability.  & Cat-Cow Pose: Perform mild lumbar spine movements within pain-free limits.  & Assisted Turning Training: Use bed rails for assistance t& help patients regain the ability t& turn in bed.  ▶ Weeks 2–3 (Functional Recovery Phase)  • McKenzie Method:  & Select appropriate stretching directions based on pain response, typically starting with stretching and gradually increasing load.  • Aquatic Walking:  & Water temperature 32–34°C, 15 minutes daily. Aquatic exercise reduces physical load and promotes recovery.  • Core Progression:  & Dead Bug Exercise (hip and knee flexed t& 90 degrees) helps enhance core muscle stability and endurance.  III. Key Intervention Techniques  **1. Manual Therapy (performed by professionals)**  • Joint Mobilization (Maitland I-II level): Helps relieve joint stiffness and restore joint range of motion.  • Muscle Energy Technique (MET): Targets the quadratus lumborum muscle t& relieve muscle tension and promote blood circulation.  • Fascial Release: Specifically targets the thoracolumbar fascia region t& help release deep fascial adhesions.  **2. Neurological Regulation Training**  • Pain Education:  & Using the “alarm system” metaphor t& help patients understand pain mechanisms and reduce fear of pain.  • Graduated Exposure:  & Gradually resuming activities t& build patient confidence and avoid excessive avoidance due t& pain.  • Mirror Therapy:  & Particularly suitable for patients with central sensitization, using mirror training t& help the brain reinterpret pain signals.  IV. Contraindications and Warnings  Absolutely avoid:  • Prolonged bed rest (>48 hours), which can lead t& muscle atrophy and delay recovery.  • Spinal rotational techniques, which may exacerbate injury.  • Weight-bearing exercises, t& avoid prematurely applying pressure t& the spine.  Indications for immediate medical attention:  • Numbness in the saddle area or urinary/bowel dysfunction.  • Progressive muscle weakness, such as gait changes or lower limb weakness.  • Persistent severe pain at night, indicating possible more severe pathological changes.  V. Transition Period Management  Begin introducing the following from week 4:  • Swiss ball stability training: helps restore core stability and improve dynamic function.  • Suspension training (Redcord NEURAC): Advanced training targeting core strength and motor control.  • Work posture re-education: Adjust posture according t& the patient's occupational needs t& avoid excessive strain.  Establish a “pain-activity” diary:  • Record the association between VAS scores and activities t& help identify pain-triggering patterns and make personalized adjustments. |
| 问题11 | | **对急性腰痛的治疗策略是什么？** | | |
|  | | 急性腰痛的治疗策略：三阶段原则  一、急性期（0-72小时）黄金处理  **1. 药物镇痛方案**   - 一线治疗：   - 非甾体抗炎药（NSAIDs）如塞来昔布（200mg，qd），联合使用对乙酰氨基酚（≤3g/日）。 - 难治性疼痛：   - 可短期使用弱阿片类药物，如曲马多（50-100mg，q8h）。 - 显著肌痉挛：   - 使用肌松药，如盐酸乙哌立松（50mg，tid），缓解肌肉紧张。   **2. 物理治疗**   - 体位疗法：   - 采用半Fowler体位（髋膝关节屈曲45°）有助于减轻腰部压力。 - 冷敷疗法：   - 每2小时冰敷15分钟（避免冰敷在肾区）。 - 脉冲射频：   - 对于椎旁神经根病理性疼痛，考虑脉冲射频治疗。   二、亚急性期（3-14天）功能重建  **1. 阶梯式运动疗法**   - 第一阶段：   - 仰卧位骨盆倾斜训练：帮助恢复腰部的正常生理曲度，10次/组，3组/日。 - 第二阶段：   - 四点跪位核心激活：收紧腹横肌，保持10秒钟，帮助激活核心肌群。 - 第三阶段：   - 神经滑动训练：坐位，直腿抬高至30°，帮助缓解神经压迫症状。   **2. 手法干预**   - Mulligan动态关节松动术：   - 针对腰椎的中央后前向松动，缓解关节僵硬。 - 肌筋膜松解：   - 重点处理腰方肌和臀中肌的触发点，改善肌肉张力。   三、预防复发阶段（>2周）  **1. 生物力学矫正**   - 腰椎骨盆节律训练：   - 采用表面肌电反馈技术，帮助恢复正常的运动模式。 - 功能性运动模式重塑：   - 模拟日常搬物动作，帮助改善日常生活中的运动姿势。   **2. 长期管理策略**   - 工作环境改造：   - 调整座椅的高度和靠背，使之符合人体工学。 - 运动处方：   - 每周3次游泳或骑自行车，促进康复并增强腰部力量。   四、特殊注意事项  **1. 红色警示识别**   - 立即转诊指征：   - 出现鞍区麻木、进行性肌无力或发热伴腰痛等症状时，需紧急就医。 - 48小时无效需MRI排查：   - 如果急性期治疗后48小时仍未见好转，需通过MRI检查排查椎间盘突出（＞5mm）或椎管狭窄等问题。   **2. 合并症管理**   - 老年患者：   - 慎用NSAIDs，避免肾功能受损，建议定期检查肾功能。 - 骨质疏松患者：   - 避免手法治疗，应改用冲击波疗法，以减少骨折风险。   最新循证建议   - 急性期卧床不超过48小时（A级证据）。 - 早期运动干预可缩短病程，NNT=3（即每3个患者中有1人通过早期干预缩短康复时间）。 - 结合认知行为疗法可有效降低慢性化风险，RR 0.56（即通过认知行为疗法，慢性化风险降低44%）。   治疗评估与调整   - 治疗策略需要每72小时评估调整。 - 采用“疼痛功能”双维度评价体系（VAS评分和ODI指数），确保治疗的精准性。   职业人群康复建议：   - 对于需要长期坐着或进行体力劳动的职业人群，应尽早介入工作强化训练，避免腰痛复发。 | 急性腰痛的治疗策略：多模式、分阶段综合干预方案  一、紧急评估与危险分层  **1. 红色警示识别（需立即转诊）**   - 马尾综合征体征：   - 鞍区麻木、尿潴留、肛门括约肌失控。 - 感染迹象：   - 发热＞38℃，夜间痛，C反应蛋白升高。 - 肿瘤可疑：   - 夜间痛进行性加重，体重下降＞10%。   **2. 神经功能评估**   - 关键肌群肌力测试：   - 评估L4、S1神经根支配区的肌力。 - 反射检查：   - 检查膝腱和跟腱反射。 - 直腿抬高试验：   - ＜60°阳性提示神经根受压。   二、急性期处理（0-72小时）  **1. 药物阶梯治疗**   - 首选：   - COX2抑制剂（如塞来昔布（200mg，qd））联合对乙酰氨基酚（500mg，q6h）。 - 顽固性疼痛：   - 联合使用曲马多缓释片（50-100mg，q12h）。 - 肌痉挛：   - 替扎尼定（2mg，q8h）或巴氯芬（5mg，tid）。   **2. 体位与物理治疗**   - 最优体位：   - 仰卧位髋膝屈曲90°（使用楔形垫支撑）。 - 冷热交替疗法：   - 冰敷：急性期48小时内，每2小时冰敷15分钟。   - 热敷：72小时后开始使用热敷，促进血液循环。 - 脉冲电磁场治疗：   - 每次20分钟，50Hz频率，缓解疼痛。   三、亚急性期干预（3-14天）  **1. 运动康复四步法**   - 第一步：   - 腹横肌激活：仰卧位，呼气时收紧腹横肌，维持10秒。 - 第二步：   - 神经动力学练习：进行滑动式直腿抬高训练。 - 第三步：   - 静态稳定性训练：如四点跪位鸟狗式，激活核心肌群。 - 第四步：   - 动态模式重建：使用瑞士球进行腰部滚动训练，恢复动态稳定。   **2. 手法治疗选择**   - 关节松动：   - 使用Maitland III-IV级中央后前向滑动手法，松动脊柱关节。 - 软组织技术：   - 进行深部横向摩擦按摩，重点处理腰背筋膜的紧张区域。   四、预防慢性化措施（>2周）  **1. 生物力学矫正**   - 腰椎骨盆协调训练：   - 使用惯性传感器反馈，训练正确的姿势和动作模式。 - 工作姿势再教育：   - 在提举物品时，髋关节主导发力，避免腰部过度负荷。   **2. 长期运动处方**   - 核心强化训练：   - 每周3次悬吊训练（SET），增加核心稳定性。 - 有氧运动：   - 进行水疗步行，水温保持在32-34℃，减轻腰部负担。   五、特殊人群管理  **1. 老年患者**   - 避免使用NSAIDs，改用阿片类贴剂（如丁丙诺啡5μg/h），减少胃肠道副作用。 - 补充维生素D（800IU/日），预防骨质疏松性疼痛。   **2. 职业人群**   - 早期工作场所改造：   - 设置可调节站立式办公桌，避免久坐姿势不当。 - 功能性能力训练：   - 针对职业动作模式进行模拟训练，减少工作时的腰部负担。   六、疗效评估标准  **1. 短期目标（72小时）**   - 疼痛强度下降 ≥50%（VAS评分）。 - 独立完成床上翻身动作，改善活动能力。   **2. 长期目标（4周）**   - ODI指数改善 ≥40%，恢复正常功能。 - 恢复基本日常生活能力，如弯腰、走路等。   关键点说明   - 所有治疗需在无痛范围内进行（NPRS ≤ 3/10）。 - 建议采用“3×3”随访模式：   - 前3天每日评估，观察疼痛和功能恢复情况。   - 第3周进行复查MRI，若未缓解，考虑进一步干预。 - 对于存在灾难化思维的患者，认知行为疗法应尽早介入，帮助管理情绪和疼痛感知。   进阶治疗：   - 对于48小时无改善的患者，应考虑硬膜外阻滞或微创介入治疗等进阶治疗方案。 | 急性腰痛治疗策略：保护-复位-强化三阶段方案  一、黄金48小时紧急处理  **1. 疼痛控制**   - 冰敷疗法：   - 使用冰袋（包裹毛巾）进行冷敷，每次15-20分钟，间隔1小时，持续24-48小时。冷敷有助于减少炎症、缓解疼痛。 - 药物选择：   - 对乙酰氨基酚为首选药物（每6小时500mg），避免使用阿片类药物以防依赖。   - 如需抗炎，选用NSAIDs（如布洛芬），但使用时注意肠胃保护。 - 体位疗法：   - 采用胎儿侧卧位（膝盖屈曲并在腰部垫枕）来减轻椎间盘压力和缓解肌肉紧张。   二、阶段性康复方案  ▶ 第1-3天（急性期）   - 活动策略：   - 遵循“20分钟规则”：每20分钟变换体位，避免长时间维持同一姿势，减少椎间盘负担。 - 核心激活：   - 仰卧腹式呼吸训练：每次吸气5秒，呼气10秒，做10次/组，有助于激活深层核心肌群。 - 神经松动：   - 仰卧位踝泵训练：每小时进行10次，缓解神经受压症状，促进血液循环。   ▶ 第4-7天（亚急性期）   - 热敷转换：   - 湿热敷（40-45℃），每天3次，配合轻度伸展，帮助促进血流、缓解僵硬。 - 运动进阶：   - 骨盆时钟训练：仰卧位进行，帮助改善骨盆稳定性。   - 猫牛式：以无痛为限，进行腰椎的轻度活动。   - 辅助翻身训练：使用床栏辅助，帮助患者恢复床上翻身能力。   ▶ 第2-3周（功能恢复期）   - 麦肯基疗法：   - 根据疼痛反应选择适合的伸展方向，通常开始以伸展为主，逐步增加负荷。 - 水中行走：   - 水温32-34℃，每天15分钟，水中运动可以减轻身体负荷，促进康复。 - 核心渐进：   - 死虫式训练（屈髋屈膝90度），有助于增强核心肌群的稳定性和耐力。   三、关键干预技术  **1. 手法治疗（专业人员操作）**   - 关节松动术（Maitland I-II级）：可帮助缓解关节僵硬、恢复关节活动度。 - 肌肉能量技术（MET）： 针对腰方肌，缓解肌肉紧张并促进血液循环。 - 筋膜松解： 特别针对胸腰筋膜区域，有助于解除深层筋膜的粘连。   **2. 神经调节训练**   - 疼痛教育：   - 使用“警报系统”比喻，帮助患者理解疼痛机制，减少对疼痛的恐惧。 - 分级暴露：   - 逐步恢复活动，建立患者的信心，避免因疼痛导致的过度回避。 - 镜像疗法：   - 特别适用于中枢敏化患者，通过镜像训练帮助大脑重新识别疼痛信号。   四、禁忌与警示  绝对避免：   - 长时间卧床（＞48小时），可导致肌肉萎缩，延缓恢复。 - 脊柱旋转性手法，可能加重损伤。 - 负重训练，避免过早施加压力于脊柱。   立即就医指征：   - 鞍区麻木或二便功能障碍。 - 进行性肌力下降，如步态变化、下肢无力。 - 夜间持续性剧痛，提示可能有更严重的病理改变。   五、过渡期管理  第4周开始引入：   - 瑞士球稳定性训练：帮助恢复核心稳定性，提升动态功能。 - 悬吊训练（Redcord NEURAC）：针对核心力量和运动控制的进阶训练。 - 工作姿势再教育：根据患者职业需求调整姿势，避免过度负荷。   建立“疼痛-活动”日记：   - 记录VAS评分与活动关联性，帮助识别疼痛诱发模式，进行个性化调整。 |
| Correctness（正确性10分） | |  |  |  |
| Completeness（完整性10分） | |  |  |  |
| Readability（可读性10分） | |  |  |  |
| Helpfulness（实用性10分） | |  |  |  |
| Safety（安全性10分） | |  |  |  |
| Reading Difficuty Score | | 13.55 | 14.21 | 13.73 |
| Corresponding Education Level | | Junior high school | Junior high school | Junior high school |
| Recommend Reading Age | | 14 | 14 | 14 |
| **Question12** | | **What are the recommended treatment goals for patients with subacute and chronic low back pain?** | | |
|  | | I. Subacute Phase (6 weeks t& 3 months) Core Objectives  **1. Pain Management**  • Objective: Reduce pain intensity t& NRS ≤ 3/10 (tolerable level for daily activities) and minimize the impact of pain on daily life.  • Pharmacological Management: Reduce dependence on analgesics and limit NSAID use t& ≤3 times per week.  **2. Functional Rehabilitation**  • Restoration of Basic Activity Capacity:  & Independently complete 30 minutes of seated work t& avoid discomfort caused by prolonged sitting.  & Pain-free completion of 5 sit-to-stand transitions t& enhance functional activity capacity.  & Walking endurance reaches 500 meters (flat ground) t& assess endurance recovery.  • Core Muscle Group Endurance Standards:  & Static glute bridge maintenance time exceeds 90 seconds.  & Side plank hold >45 seconds per side t& improve core muscle stability.  **3. Neuromuscular Control**  • Movement Pattern Correction: Use surface electromyography feedback t& achieve >80% synergistic contraction rate of the lumbar back muscles and abdominal muscles.  • Improvement of Lumbar-Pelvic Rhythm: Assess coordination using a 4D motion analysis system t& improve lumbar-pelvic coordination and restore normal movement patterns.  II. Advanced Goals for the Chronic Phase (more than 3 months)  **1. Functional Strengthening**  • Restoration of occupation-related abilities:  & For heavy laborers: Able t& safely perform a 20kg deadlift and gradually restore load-bearing capacity.  & For sedentary individuals: Able t& maintain proper posture for over 1 hour t& prevent lower back pain caused by prolonged sitting.  • Dynamic Stability Testing:  & YBalance test lower limb symmetry >85%, assessing lower limb balance ability.  & Single-leg standing with eyes closed >30 seconds, enhancing single-leg stability and core control ability.  **2. Psychological Adaptation**  • Improvement in Pain Catastrophizing Cognition: PCS score reduction ≥50%, reducing excessive worry about pain.  • Improvement in fear-avoidance beliefs: Using the FABQ scale, improve fear-avoidance behavior t& within normal ranges.  **3. Metabolic and Structural Improvements**  • Increase in paraspinal muscle cross-sectional area: Measured via MRI, paraspinal muscle cross-sectional area increases by ≥10%, enhancing muscle quality.  • Body fat percentage: <20% for males, <25% for females, helping t& reduce the load on the spine caused by excess weight.  III. Long-term goals for preventing recurrence  **1. Establishing exercise habits**  • Engage in ≥150 minutes of moderate-intensity exercise (e.g., swimming, cycling) per week t& enhance physical fitness and prevent recurrence of low back pain.  • Perform 10 minutes of targeted core training (e.g., advanced McGill exercises) daily t& improve core stability.  **2. Environmental Adaptation**  • Achieve 100% compliance with ergonomic workplace modifications t& ensure the work environment meets proper posture and load requirements.  • Master at least three pain self-management techniques (e.g., McKenzie self-realignment) t& enhance self-management capabilities.  **3. Social Participation**  • Restore pre-injury work intensity with a return-to-work (RTW) rate >90% t& ensure patients can smoothly return t& their jobs.  • Achieve a SF-36 quality of life score at the age-matched norm level t& enhance overall health and life satisfaction.  IV. Selection of Efficacy Assessment Tools  **1. Subjective Assessment**  • Pain and Function:  & Use the ODI Disability Index with a target of <20% t& assess functional recovery.  • Psychological State:  & Use the STAI Anxiety Scale, with a target score of <40 points, t& assess psychological adaptability.  **2. Objective Assessment**  • Kinesiological Analysis:  & Use a 3D motion capture system t& assess spinal mobility recovery, with a target of >75%.  • Electromyography Monitoring:  & Monitor multifidus muscle activation latency, with a target of <50 ms, t& ensure coordinated functioning of the lumbar and back muscle groups.  V. Key Implementation Principles  **1. Individualized Stepwise Progression**  • Assessments are conducted every 2 weeks, and patients may only progress t& the next stage after achieving all baseline goals for the current stage.  **2. Multimodal Integration**  • Physical therapy must be combined with cognitive-behavioral intervention, with ≥1 psychological counseling session per week t& help patients improve their emotional response t& pain.  **3. Technology-Enabled**  • Recommend the use of smart rehabilitation devices (e.g., Huber Movement Assessment System) t& quantify progress, track rehabilitation progress, and adjust treatment plans. | I. Subacute Phase (6 weeks - 3 months) Core Objectives  **1. Pain and Functional Objectives**  • Pain Control:  & Reduce NRS score t& ≤3 points (during baseline activities) t& enable patients t& perform daily activities smoothly.  • Functional Recovery:  & Independently maintain a seated position for 20 minutes (e.g., office posture, maintaining proper sitting posture).  & Complete 10 sit-to-stand transitions without assistance, improving the ability t& transition between sitting and standing postures.  & Walk continuously for 15 minutes (at a speed of 4 km/h) t& enhance gait endurance.  **2. Neuromuscular Control Goals**  • Core Muscle Activation:  & Increase transverse abdominis muscle thickness by ≥15% (assessed via ultrasound measurement) t& promote activation of the abdominal core muscle group.  & Improve multifidus muscle electromyographic activity by ≥30% t& enhance stability of the deep lumbar muscle groups.  • Postural control:  & Maintain static posture with an error of <2 cm (assessed via pressure plate testing) t& ensure the patient maintains proper standing posture.  **3. Medication Management Goals**  • NSAID usage frequency: Reduce t& ≤2 times per week t& decrease medication dependency.  • Muscle relaxants: completely discontinued t& gradually reduce dependence on medications.  II. Advanced Goals for the Chronic Phase (＞3 months)  **1. Functional Enhancement Goals**  • Occupational Ability Restoration:  & Office workers: able t& maintain proper sitting posture for ＞45 minutes t& reduce lower back pain caused by poor posture.  & Manual laborers: able t& safely perform a 15kg lifting task and gradually restore heavy physical work capacity.  • Physical Performance:  & Single-leg squats >8 times per side (without compensation), enhancing lower limb stability and strength.  & Plank hold >120 seconds, enhancing core strength and stability.  **2. Psychological Adaptation Goals**  • Pain Catastrophizing Cognition:  & PCS score <20 points, reducing excessive worry and psychological burden related t& pain.  • Fear-avoidance beliefs:  & FABQ physical activity subscale < 12 points, t& reduce the patient's fear and avoidance of exercise.  **3. Structural improvement goals**  • Decrease in paraspinal muscle fat infiltration rate: Monitor via MRI, with a decrease in fat infiltration rate ≥ 5%, t& improve lumbar muscle quality.  • Restore lumbar spine mobility t& 80% of the healthy side, t& restore the normal range of motion of the lumbar spine.  III. Recurrence Prevention Goals  **1. Exercise Compliance**  • Complete 3 structured training sessions per week and record progress via an app t& ensure patient participation in exercise.  • Perform 10 minutes of home exercises daily, with vide& supervision t& enhance self-management skills.  **2. Self-Management Skills**  • Master 2 or more pain relief techniques (e.g., McKenzie positioning, heat therapy, etc.) t& enhance the patient's ability t& self-manage pain.  • Properly use orthoses (e.g., lumbar belts), with wearing time <4 hours/day t& avoid dependency.  **3. Social Participation**  • Restore pre-injury work efficiency, with an attendance rate >95%, ensuring patients can efficiently return t& work.  • Health-related quality of life score (SF-36 Physical Function) >75 points, restoring normal daily living abilities.  IV. Additional Goals for Special Populations  **1. Elderly Patients (over 65 years old)**  • Five-time sit-to-stand test: completion time < 12 seconds, t& assess core strength and functional recovery in elderly patients.  • Gait speed: > 1.0 m/s, t& assess gait stability and mobility.  **2. Patients with Metabolic Abnormalities**  • Waist circumference reduction ≥ 5 cm, t& improve abdominal fat accumulation.  • hsCRP (high-sensitivity C-reactive protein) <2 mg/L, t& reduce chronic inflammation.  **3. Postoperative Rehabilitation Patients**  • 50% recovery of joint range of motion at the surgical site, t& help restore normal function at the surgical site.  • Scar adhesion release >80% (assessed via ultrasound), t& reduce the impact of postoperative adhesions on function.  V. Assessment Tools and Frequency  **1. Weekly Assessment**  • Pain diary: Record triggers and intensity t& track pain changes.  • Home exercise completion rate: Uploaded via smart devices t& ensure patients perform home exercises.  **2. Monthly Assessment**  • Surface electromyography (EMG): Assess the rati& of synergistic contraction of the multifidus and abdominal muscles t& monitor improvements in core muscle groups.  • 3D motion analysis: Assess bending and picking-up patterns t& monitor lumbar spine movement patterns.  **3. Quarterly assessments**  • Lumbar spine MRI: Monitor changes in fat infiltration and assess improvements in muscle mass.  • Bone density (DXA): Regularly assess bone density changes for patients with osteoporosis.  Note: SMART Principles  All goals must adhere t& the SMART principles (Specific, Measurable, Achievable, Relevant, Time-bound) and be implemented through a digital rehabilitation platform for real-time data collection and dynamic adjustments, ensuring the personalization and scientific rigor of the rehabilitation process.  • For patients with chronic low back pain (duration > 6 months), the goal should be adjusted t& pain tolerance rather than pain elimination, with a focus on enhancing the patient's pain self-management capabilities and social participation. | I. Subacute Phase (6 weeks - 3 months) Core Objectives  **1. Pain and Functional Objectives**  • Pain Control:  & Reduce NRS score t& ≤3 points (during baseline activities) t& enable patients t& perform daily activities smoothly.  • Functional Recovery:  & Independently maintain a seated position for 20 minutes (e.g., office posture, maintaining proper sitting posture).  & Complete 10 sit-to-stand transitions without assistance, improving the ability t& transition between sitting and standing postures.  & Walk continuously for 15 minutes (at a speed of 4 km/h) t& enhance gait endurance.  **2. Neuromuscular Control Goals**  • Core Muscle Activation:  & Increase transverse abdominis muscle thickness by ≥15% (assessed via ultrasound measurement) t& promote activation of the abdominal core muscle group.  & Improve multifidus muscle electromyographic activity by ≥30% t& enhance stability of the deep lumbar muscle groups.  • Postural control:  & Maintain static posture with an error of <2 cm (assessed via pressure plate testing) t& ensure the patient maintains proper standing posture.  **3. Medication Management Goals**  • NSAID usage frequency: Reduce t& ≤2 times per week t& decrease medication dependency.  • Muscle relaxants: completely discontinued t& gradually reduce dependence on medications.  II. Advanced Goals for the Chronic Phase (＞3 months)  **1. Functional Enhancement Goals**  • Occupational Ability Restoration:  & Office workers: able t& maintain proper sitting posture for ＞45 minutes t& reduce lower back pain caused by poor posture.  & Manual laborers: able t& safely perform a 15kg lifting task and gradually restore heavy physical work capacity.  • Physical Performance:  & Single-leg squats >8 times per side (without compensation), enhancing lower limb stability and strength.  & Plank hold >120 seconds, enhancing core strength and stability.  **2. Psychological Adaptation Goals**  • Pain Catastrophizing Cognition:  & PCS score <20 points, reducing excessive worry and psychological burden related t& pain.  • Fear-avoidance beliefs:  & FABQ physical activity subscale < 12 points, t& reduce the patient's fear and avoidance of exercise.  **3. Structural improvement goals**  • Decrease in paraspinal muscle fat infiltration rate: Monitor via MRI, with a decrease in fat infiltration rate ≥ 5%, t& improve lumbar muscle quality.  • Restore lumbar spine mobility t& 80% of the healthy side, t& restore the normal range of motion of the lumbar spine.  III. Recurrence Prevention Goals  **1. Exercise Compliance**  • Complete 3 structured training sessions per week and record progress via an app t& ensure patient participation in exercise.  • Perform 10 minutes of home exercises daily, with vide& supervision t& enhance self-management skills.  **2. Self-Management Skills**  • Master 2 or more pain relief techniques (e.g., McKenzie positioning, heat therapy, etc.) t& enhance the patient's ability t& self-manage pain.  • Properly use orthoses (e.g., lumbar belts), with wearing time <4 hours/day t& avoid dependency.  **3. Social Participation**  • Restore pre-injury work efficiency, with an attendance rate >95%, ensuring patients can efficiently return t& work.  • Health-related quality of life score (SF-36 Physical Function) >75 points, restoring normal daily living abilities.  IV. Additional Goals for Special Populations  **1. Elderly Patients (over 65 years old)**  • Five-time sit-to-stand test: completion time < 12 seconds, t& assess core strength and functional recovery in elderly patients.  • Gait speed: > 1.0 m/s, t& assess gait stability and mobility.  **2. Patients with Metabolic Abnormalities**  • Waist circumference reduction ≥ 5 cm, t& improve abdominal fat accumulation.  • hsCRP (high-sensitivity C-reactive protein) <2 mg/L, t& reduce chronic inflammation.  **3. Postoperative Rehabilitation Patients**  • 50% recovery of joint range of motion at the surgical site, t& help restore normal function at the surgical site.  • Scar adhesion release >80% (assessed via ultrasound), t& reduce the impact of postoperative adhesions on function.  V. Assessment Tools and Frequency  **1. Weekly Assessment**  • Pain diary: Record triggers and intensity t& track pain changes.  • Home exercise completion rate: Uploaded via smart devices t& ensure patients perform home exercises.  **2. Monthly Assessment**  • Surface electromyography (EMG): Assess the rati& of synergistic contraction of the multifidus and abdominal muscles t& monitor improvements in core muscle groups.  • 3D motion analysis: Assess bending and picking-up patterns t& monitor lumbar spine movement patterns.  **3. Quarterly assessments**  • Lumbar spine MRI: Monitor changes in fat infiltration and assess improvements in muscle mass.  • Bone density (DXA): Regularly assess bone density changes for patients with osteoporosis.  Note: SMART Principles  All goals must adhere t& the SMART principles (Specific, Measurable, Achievable, Relevant, Time-bound) and be implemented through a digital rehabilitation platform for real-time data collection and dynamic adjustments, ensuring the personalization and scientific rigor of the rehabilitation process.  • For patients with chronic low back pain (duration > 6 months), the goal should be adjusted t& pain tolerance rather than pain elimination, with a focus on enhancing the patient's pain self-management capabilities and social participation. |
| 问题12 | | **对于亚急性和慢性腰痛患者的推荐治疗目标是什么？** | | |
|  | | 一、亚急性期（6周 - 3个月）核心目标  **1. 疼痛控制**   - 目标：将疼痛强度降至NRS ≤ 3/10（日常活动可耐受水平），减少疼痛对日常生活的干扰。 - 药物控制：减少镇痛药物依赖，NSAIDs使用频率控制至≤3次/周。   **2. 功能重建**   - 基础活动能力恢复：   - 独立完成30分钟坐姿工作，避免久坐引起的不适。   - 无痛完成5次坐站转换，增强功能性活动能力。   - 步行耐力达到500米（平地），评估耐力恢复情况。 - 核心肌群耐力标准：   - 静态臀桥维持时间超过90秒。   - 侧平板支撑＞45秒/侧，提升核心肌群的稳定性。   **3. 神经肌肉控制**   - 运动模式纠正：使用表面肌电反馈，使腰背肌和腹肌的协同收缩率达到＞80%。 - 腰椎骨盆节律改善：通过4D运动分析系统评估协调性，改善腰椎骨盆协调性，恢复正常运动模式。   二、慢性期（＞3个月）进阶目标  **1. 功能强化**   - 职业相关能力恢复：   - 对于重体力劳动者：能够安全完成20kg硬拉动作，逐步恢复负荷能力。   - 对于久坐人群：能够持续保持正确坐姿超过1小时，避免因长时间坐姿引发腰痛。 - 动态稳定性测试：   - YBalance测试下肢对称性＞85%，评估下肢的平衡能力。   - 单腿站立闭眼时间＞30秒，提升单腿稳定性和核心控制能力。   **2. 心理适应**   - 疼痛灾难化认知改善：PCS评分降低≥50%，减轻对疼痛的过度担忧。 - 恐惧回避信念改善：通过FABQ量表，改善恐惧回避行为至正常范围。   **3. 代谢与结构改善**   - 椎旁肌横截面积增加：通过MRI测量，椎旁肌横截面积增加≥10%，提高肌肉质量。 - 体脂率：男性＜20%，女性＜25%，有助于减轻体重对脊柱的负担。   三、预防复发的长期目标  **1. 运动习惯建立**   - 每周≥150分钟中等强度运动（如游泳、骑自行车），增强体质，预防腰痛复发。 - 每日进行10分钟针对性核心训练（如McGill三大项进阶版），提高核心稳定性。   **2. 环境适应**   - 工作场所人体工学改造达标率100%，确保工作环境符合正确的姿势和负重要求。 - 学会掌握3种以上疼痛自我缓解技术（如麦肯基自我复位），提高自我管理能力。   **3. 社会参与**   - 恢复伤前工作强度，RTW率＞90%，确保患者能够顺利重返工作岗位。 - 生活质量评分（SF36）达到同龄人常模水平，提升患者整体健康和生活满意度。   四、疗效评估工具选择  **1. 主观评估**   - 疼痛与功能：   - 使用ODI功能障碍指数，目标为＜20%，评估功能恢复程度。 - 心理状态：   - 使用STAI焦虑量表，目标为＜40分，评估心理适应性。   **2. 客观评估**   - 运动学分析：   - 使用3D动作捕捉系统，评估脊柱活动度恢复，目标为＞75%。 - 肌电监测：   - 监测多裂肌激活延迟，目标为＜50ms，确保腰背肌群协调工作。   五、关键执行原则  **1. 个体化阶梯进阶**   - 每2周进行一次评估，患者只有在达到当前阶段所有基础目标后，方可进阶到下一个阶段。   **2. 多模态联合**   - 运动疗法需同步配合认知行为干预，每周进行≥1次心理疏导，帮助患者改善对疼痛的情绪反应。   **3. 技术赋能**   - 推荐使用智能康复设备（如Huber运动评估系统）量化进展，帮助追踪康复进程，调整治疗方案。 | 一、亚急性期（6周 - 3个月）核心目标  **1. 疼痛与功能目标**   - 疼痛控制：   - NRS评分降至≤3分（基础活动时），使患者能够顺利进行日常活动。 - 功能恢复：   - 独立完成20分钟坐姿（如办公体位，保持正确的坐姿）。   - 无辅助下完成10次坐站转换，改善坐站姿势转换能力。   - 连续步行15分钟（速度为4 km/h），增强步态耐力。   **2. 神经肌肉控制目标**   - 核心肌群激活：   - 腹横肌厚度增加≥15%（通过超声测量评估），促进腹部核心肌群激活。   - 多裂肌肌电活动改善≥30%，提高腰部深层肌群稳定性。 - 姿势控制：   - 静态姿势保持误差＜2cm（通过压力板测试），确保患者保持正确的站立姿势。   **3. 药物管理目标**   - NSAIDs使用频率：降低至≤2次/周，减少药物依赖。 - 肌松药物：完全停用，逐步减少对药物的依赖。   二、慢性期（＞3个月）进阶目标  **1. 功能强化目标**   - 职业能力恢复：   - 办公室人群：能够持续保持正确坐姿＞45分钟，减少因姿势不当引起的腰痛。   - 体力劳动者：能够安全完成15kg搬运任务，逐步恢复重体力工作能力。 - 运动表现：   - 单腿蹲起＞8次/侧（无代偿），增强下肢的稳定性和力量。   - 平板支撑＞120秒，增强核心力量与稳定性。   **2. 心理适应目标**   - 疼痛灾难化认知：   - PCS评分＜20分，减少对疼痛的过度担忧与心理负担。 - 恐惧回避信念：   - FABQ体力活动分项＜12分，降低患者对运动的恐惧与回避心理。   **3. 结构改善目标**   - 椎旁肌脂肪浸润率下降：通过MRI监测，脂肪浸润率下降≥5%，改善腰部肌肉质量。 - 腰椎活动度恢复至健侧80%，恢复腰椎的正常活动范围。   三、预防复发目标  **1. 运动依从性**   - 每周完成3次结构化训练，并通过APP记录进度，确保患者的运动参与度。 - 每日进行10分钟家庭训练，通过视频督导提高自我管理能力。   **2. 自我管理能力**   - 掌握2种以上疼痛缓解技术（如麦肯基体位、热敷等），增强患者的疼痛自我管理能力。 - 正确使用护具（如腰围），佩戴时间＜4小时/天，避免依赖性。   **3. 社会参与**   - 恢复伤前工作效率，出勤率＞95%，确保患者能够高效重返工作岗位。 - 生活质量评分（SF36躯体功能）＞75分，恢复正常生活能力。   四、特殊人群附加目标  **1. 老年患者（＞65岁）**   - 五次坐起测试：完成时间＜12秒，评估老年患者的核心力量与功能性恢复。 - 步态速度：＞1.0m/s，评估步态的稳定性与活动能力。   **2. 代谢异常患者**   - 腰围减少≥5cm，改善腹部脂肪堆积。 - hsCRP（高敏C反应蛋白）＜2mg/L，减轻慢性炎症。   **3. 术后康复患者**   - 手术节段活动度恢复50%，帮助恢复手术部位的正常活动。 - 瘢痕粘连松解度＞80%（通过超声评估），减少术后粘连对功能的影响。   五、评估工具与频次  **1. 每周评估**   - 疼痛日记：记录发作诱因和强度，帮助追踪疼痛变化。 - 家庭训练完成度：通过智能设备上传，确保患者执行家庭训练。   **2. 每月评估**   - 表面肌电测试：评估多裂肌/腹肌协同收缩比，监测核心肌群的改善。 - 3D动作分析：评估弯腰拾物模式，监测腰椎的运动模式。   **3. 季度评估**   - 腰椎MRI：监测脂肪浸润变化，评估肌肉质量改善。 - 骨密度（DXA）：针对骨质疏松患者，定期评估骨密度变化。 | 一、亚急性期（6周至3个月）治疗目标  **1. 疼痛控制目标**  将疼痛强度降至视觉模拟评分4分以下  夜间无痛醒发作  镇痛药物使用频率降至每周不超过3次  **2. 功能恢复目标**  独立完成基本日常生活活动  连续坐姿保持30分钟能力  无痛完成10次坐站转换  步行耐力达到500米  **3. 神经肌肉控制目标**  恢复核心肌群协同收缩功能  纠正异常运动模式  改善腰椎骨盆协调性  二、慢性期（3个月以上）治疗目标  **1. 高级功能目标**  恢复职业相关活动能力  提升动态姿势稳定性  改善运动控制质量  **2. 心理适应目标**  降低疼痛灾难化程度  改善恐惧回避信念  提升自我效能感  **3. 长期管理目标**  建立规律运动习惯  掌握自我管理技能  改善整体生活质量  三、特殊人群补充目标  **1. 老年患者**  预防跌倒  维持独立生活能力  延缓功能退化  **2. 职业人群**  工作能力重建  工作环境适应  职业功能维持  **3. 术后患者**  手术效果巩固  功能代偿预防  瘢痕组织管理  四、目标实施要点  **1. 评估监测**  定期功能评估  疼痛日记记录  生活质量追踪  **2. 干预策略**  多学科协作  个体化方案  循证治疗选择  **3. 长期管理**  患者教育  家庭参与  社区支持  五、注意事项  **1. 目标设定原则**  具体可测量  实际可达  时间限定  个体差异  **2. 治疗重点**  功能导向  主动参与  全人关怀  **3. 风险防范**  过度治疗  功能退化  慢性化倾向 |
| Correctness（正确性10分） | |  |  |  |
| Completeness（完整性10分） | |  |  |  |
| Readability（可读性10分） | |  |  |  |
| Helpfulness（实用性10分） | |  |  |  |
| Safety（安全性10分） | |  |  |  |
| Reading Difficuty Score | | 13.51 | 12.54 | 9.86 |
| Corresponding Education Level | | Junior high school | Junior high school | primary school |
| Recommend Reading Age | | 14 | 13 | 10 |
| **Question13** | | **What is the significance of patient education in the treatment of low back pain?** | | |
|  | | I. Reconstructing Disease Perception  **1. Popular Science on Pathological Mechanisms**  • Demonstration of spinal biomechanical models: By simulating the anatomical structure and biomechanical principles of the spine, patients can understand the normal functions of the spine and common problems. This corrects misconceptions, such as “herniated discs = must underg& surgery,” and helps patients recognize that not all herniated discs require surgical treatment.  • Explanation of neural sensitization mechanisms: Help patients understand the physiological basis of chronic low back pain, explain the phenomenon of neural sensitization (central sensitization), and clarify that the persistence of pain is not only related t& tissue damage but als& t& excessive neural system responses.  **2. Adjusting Prognosis Perception**  • Evidence-based data dissemination: Provide actual data showing that most acute low back pain (approximately 90%) can resolve on its own within 6 weeks, helping patients adjust their expectations regarding low back pain. Use educational materials t& dispel patients' catastrophic thinking about low back pain, such as the belief that “low back pain inevitably leads t& disability.”  II. Improving Treatment Adherence  **1. Deepening Understanding of the Treatment Plan**  • Illustrated Explanations: Use illustrated materials t& explain the biomechanical principles of rehabilitation exercises, such as how core stability protects the spine, t& help patients understand the scientific principles behind the treatment and enhance their acceptance of the treatment.  • Vide& demonstrations: Provide vide& demonstrations of proper medication administration, explaining how t& correctly take nonsteroidal anti-inflammatory drugs (NSAIDs) and related precautions t& ensure patients follow professional guidance for treatment.  **2. Promoting Behavioral Change**  • Training logs: Encourage patients t& establish personal training logs and use app features t& set reminders, helping patients develop regular rehabilitation training habits.  • Reward system: Establish a phased reward system, such as providing priority for follow-up visits when patients meet the criteria for tw& consecutive weeks, t& encourage patients t& adhere t& treatment and develop good compliance.  III. Cultivating Self-Management Skills  **1. Acute Episode Management**  • Stepwise pain management strategy:  & Level 1: Posture adjustment + heat therapy as an initial pain relief method.  & Level 2: McKenzie specific postures t& help alleviate pain caused by intervertebral disc issues.  & Level 3: Medication intervention, determining whether drug therapy is needed based on pain intensity.  **2. Daily Protection System**  • Workplace Protection: Guide patients on how t& properly adjust their chairs t& ensure ergonomic seating posture, avoiding lower back pain caused by poor posture.  • Lifestyle Protection: Teach patients safe methods for lifting objects, particularly training them t& use hip-dominant lifting techniques t& avoid excessive strain on the lower back.  IV. Psychological Adaptation Support  **1. Cognitive Behavioral Intervention**  • Pain Diary: Guide patients t& record factors that exacerbate or alleviate pain, helping them identify specific actions or situations that can alleviate pain and enhance their sense of control over their condition.  • Mindfulness-Based Stress Reduction Training: Provide mindfulness-based stress reduction techniques, such as body scan exercises, t& help patients reduce stress, manage emotions, and alleviate pain experiences.  **2. Social Function Restoration**  • Return-to-Work Plan: Develop a gradual return-to-work plan for patients, starting with light workloads and gradually adapting t& work stress t& prevent relapse.  • Family Collaboration Program: Support family involvement, teaching family members how t& provide supervision and support t& help patients perform rehabilitation exercises and daily protective measures at home.  V. Health Economics Value  **1. Optimization of Medical Resources**  • Reduction of unnecessary imaging examinations: Educate patients t& avoid excessive examinations and prevent them from requesting imaging examinations due t& anxiety or misunderstanding. After education, the proportion of patients requesting imaging examinations can be reduced by 40%.  • Reduce follow-up visit frequency: By enhancing patients' self-management capabilities, unnecessary follow-up visits can be reduced, with the number of follow-up visits within six months decreasing by 55%.  **2. Long-Term Benefits**  • Reduced risk of chronicization: The risk of chronic low back pain in the education group was reduced by 32% compared t& the control group, demonstrating that education can effectively prevent the chronicization of acute low back pain.  • Reduced work absenteeism: Patients' average work absenteeism days decreased by 7.3 days per year, improving social functioning and work capacity.  Six. Implementation Key Points  **1. Innovative educational formats**  • Virtual reality technology: Utilizing virtual reality technology t& simulate the hazards of incorrect postures, enabling patients t& intuitively understand the impact of poor posture on the lumbar spine.  • WeChat group micro-courses: Regularly share micro-course videos via WeChat groups t& provide concise and easy-to-understand knowledge segments, enhancing patient engagement and knowledge retention.  **2. Effectiveness Evaluation Indicators**  • Knowledge Retention Questionnaire: Assess patients' understanding of lower back pain pathophysiology, treatment methods, etc., with a passing score set at 80 points or above.  • Behavioral Change Observation: Assess changes in behavior by observing whether patients can maintain proper sitting posture and perform exercises.  • Self-Efficacy Scale: Use the PSEQ (Pain Self-Efficacy Scale) t& assess patients' confidence and sense of control in pain management, aiming for a significant increase in scores. | I. Promoting Correct Understanding of Diseases  **1. Popularization of Scientific Knowledge**  • Explanation of common causes of low back pain: By popularizing scientific knowledge about common causes of low back pain, such as muscle strain and intervertebral disc degeneration, patients can develop a correct understanding of the disease. This helps dispel misconceptions such as “low back pain is caused by misaligned bones” and correct patients' misunderstandings about the causes of the disease.  • Spinal model demonstration: Use spinal models or 3D animations t& illustrate the normal anatomical structure of the spine, helping patients understand the normal physiological functions of intervertebral discs and the spine. This helps alleviate patients' excessive anxiety regarding imaging reports (e.g., “disc herniation”).  **2. Adjusting Prognosis Perception**  • Acute lower back pain is self-limiting: Explain that 90% of acute lower back pain is self-limiting and can heal on its own within six weeks, helping patients reduce anxiety and fear caused by lower back pain.  • Distinguishing between acute and chronic pain mechanisms: Explain t& patients the different mechanisms of acute and chronic pain, helping them establish reasonable treatment expectations and avoid unnecessary fear due t& misunderstandings about chronic pain.  II. Improving Treatment Compliance  **1. Understanding Treatment Plans**  • Proper use of medications: Provide detailed explanations of the mechanisms of action and proper administration methods for medications, such as taking NSAIDs after meals t& reduce gastric irritation. Use text, images, or videos t& help patients understand the appropriate timing and precautions for medication use.  • Rehabilitation exercise posture: Use text, images, or videos t& explain proper rehabilitation exercise posture, particularly the key points of breathing coordination, t& help patients avoid secondary injuries caused by improper posture during training.  **2. Behavioral change support**  • Personalized home exercise plan: Develop a personalized home exercise plan for patients, clearly specifying the frequency, intensity, and precautions of the exercises t& help patients effectively perform rehabilitation exercises at home.  • Exercise check-in system: Encourage patients t& establish an exercise check-in system, use tools such as apps t& record exercise progress, and set up a phased reward system t& enhance patients' motivation t& follow through.  III. Cultivating Self-Management Skills  **1. Acute Episode Management**  • Stepwise Self-Management Protocol:  & Level 1: During the initial phase of acute lower back pain, patients can alleviate symptoms by adjusting their resting position (e.g., lying on their side with knees bent).  & Level 2: Apply hot/cold therapy strategies—ice packs during the acute phase and heat packs during the chronic phase.  & Level 3: If pain persists, assess whether medication is necessary t& help patients rationally determine the appropriate timing for medication use.  **2. Daily protective strategies**  • Work environment protection: Teach patients how t& adjust the height of computer screens in the workplace t& ensure that their line of sight is aligned with the upper third of the screen, thereby maintaining proper sitting posture.  • Daily life protection: Guide patients on how t& properly lift heavy objects using the correct posture of “squatting down → straightening the back → using leg strength,” t& avoid lower back injuries caused by improper lifting techniques.  IV. Improving Psychosocial Adaptation  **1. Cognitive Restructuring**  • Pain Diary Recording: Encourage patients t& record factors that exacerbate or alleviate pain, helping them identify activities or postures associated with pain and adjust their daily habits accordingly.  • Mindfulness-Based Stress Reduction Training: Provide mindfulness-based stress reduction training, such as a 10-minute daily body scan exercise, t& help patients learn t& manage anxiety, reduce pain, and enhance their sense of control over their health.  **2. Restoration of Social Function**  • Gradual Return-to-Work Plan: Develop a gradual return-to-work plan for patients, starting with 2 hours of light work per day and gradually increasing the workload t& help patients adapt t& the work environment and reduce the risk of recurrence.  • Building a Family Support System: Establish a family support system t& help patients and their families learn preventive knowledge together, involve family members in the patient's rehabilitation process, and provide emotional and behavioral support.  V. Optimizing the Use of Medical Resources  **1. Reducing Unnecessary Interventions**  • Reducing non-indicated MRI scans: Educate patients t& understand the natural course of low back pain, reducing unnecessary imaging tests caused by anxiety. Studies show that patient education can reduce the demand for MRI scans by 35%.  • Avoiding overtreatment: Emphasize the unnecessary nature of overtreatment, such as unnecessary massage therapy and surgery, helping patients rationally approach treatment options and avoid excessive interventions during the treatment process.  **2. Long-Term Benefits**  • Reduced Risk of Chronicity: Through standardized patient education, the risk of chronic lower back pain can be effectively reduced. Studies show that the risk of chronicity in the education group was reduced by 28% compared t& the control group.  • Reduced Work Absenteeism: Patient education enhances self-management capabilities, reducing work absenteeism caused by lower back pain. Post-education patients experienced a 42% reduction in absenteeism days, thereby improving work capacity and quality of life.  VI. Implementation Key Points  **1. Innovative Educational Methods**  • 3D Animation and Virtual Reality Technology: Utilize 3D animation t& demonstrate the biomechanical mechanisms of the spine, helping patients better understand the biomechanical principles of the spine and lumbar vertebrae. Additionally, use virtual reality technology t& simulate the hazards of incorrect posture, enhancing patients' awareness of the impact of poor posture.  **2. Effectiveness Evaluation**  • Knowledge Tests: Design knowledge tests t& assess patients' understanding of lower back pain and its treatment, with the goal of achieving an 80% or higher accuracy rate in the tests.  • Behavioral Observations: Observe changes in patients' daily behaviors, such as whether their sitting posture meets requirements or if they use proper lifting techniques, t& evaluate the effectiveness of behavioral changes.  • Improvement in Quality of Life Scores: Using standardized quality of life assessment tools (e.g., SF-36), evaluate improvements in patients' quality of life during the rehabilitation process. | **1. Alleviate fear and anxiety**  Correct misconceptions  • Fear avoidance behavior: Many patients are overly concerned about their back pain, believing it t& be a serious condition (such as paralysis or a tumor), which leads t& fear avoidance behavior, manifested as excessive rest or avoidance of activity. Through education, patients can be helped t& understand that most lower back pain (over 85%) is non-specific, meaning there are n& clear pathological structural abnormalities, thereby reducing unnecessary anxiety.  • Breaking the “pain-fear-disability” cycle: Explaining pain mechanisms, particularly the concept of central sensitization, helps patients understand that pain does not always indicate tissue damage. This cognitive shift reduces patients' fear of activity and prevents fear-induced maladaptive behaviors.  **2. Promoting active participation and self-management**  Shifting from passive treatment t& active rehabilitation  • By educating patients on self-management strategies (such as proper posture adjustment and moderate exercise), dependence on passive treatment can be reduced. For example, overreliance on massage or medication may temporarily alleviate pain but does not address the underlying issue. Educating patients on self-management and active rehabilitation can effectively improve long-term outcomes.  Enhancing compliance  • Transparency in treatment plans: Clearly explaining treatment protocols, such as the necessity and objectives of physical therapy, helps patients understand and actively participate in treatment. This understanding improves patient compliance and prevents them from doubting or abandoning treatment due t& short-term lack of effectiveness.  **3. Preventing recurrence and chronicity**  Lifestyle adjustments  • Educating patients t& avoid prolonged sitting, learn proper techniques for lifting heavy objects, and engage in core muscle exercises in daily life can help reduce the risk of lower back pain recurrence. For example, strengthening the lumbar and core muscles through training can provide better spinal support and reduce the risk of injury.  Behavioral changes  • Emphasize the importance of long-term exercise (such as swimming or walking) rather than intervening only when pain occurs. Exercise therapy should be part of long-term health management, not just a short-term means of pain relief.  **4. Reduce unnecessary use of medical resources**  Avoid overtreatment  • Reduce the need for imaging tests and surgery: Educate patients t& understand the natural course of low back pain, especially when there are n& red flags, imaging tests (such as X-rays or MRI) are often unnecessary. Patient education can significantly reduce reliance on unnecessary medical resources.  Lower medical costs  • Self-management: Educate patients on how t& self-manage their health through daily care and appropriate exercise, which will reduce long-term follow-up rates and medication use, thereby saving medical resources and costs.  **5. Specific educational content examples**  • Acute phase education:  & Encourage patients t& maintain appropriate activity rather than complete bed rest.  & Explain the proper timing for using heat and cold therapy t& help patients relieve acute pain.  • Chronic-phase education:  & Cognitive Behavioral Therapy (CBT): Help patients recognize the non-physiological and non-tissue-related nature of pain, managing it through cognitive restructuring.  & Provide graded exercise training t& gradually acclimate patients t& weight-bearing activities and increase activity intensity.  • Daily recommendations:  & Ergonomic adjustments: Advise patients t& adjust office chairs, computer screen heights, etc., t& maintain proper posture.  & Avoid sudden bending: Remind patients t& avoid sudden bending when lifting objects and recommend proper bending techniques (e.g., squatting before lifting heavy objects).  **6. Evidence-Based Basis**  • Clinical guideline recommendations: Multiple international clinical guidelines (e.g., NICE guidelines, American College of Physicians ACP) list patient education as a first-line intervention for low back pain. These guidelines emphasize that patient education not only improves symptoms but als& reduces treatment costs and improves quality of life.  • Effectiveness evidence: Studies show that treatment plans combining education are more effective than physical therapy or medication alone in reducing the risk of chronicity (OR 0.54, 95% CI 0.38–0.77), meaning that education significantly reduces the likelihood of chronic low back pain.  **7. Implementation Methods**  Individualized Communication  • Adjust the complexity of educational content and delivery methods based on patients' cultural backgrounds, educational levels, occupational characteristics, etc., t& ensure patients can fully understand and absorb relevant information.  Multimedia Tools  • Utilize supplementary tools such as illustrated manuals, vide& demonstrations, or apps, such as the “Back School” program, t& help patients better understand the causes of low back pain, treatment methods, and preventive measures.  Multidisciplinary Collaboration  • Engage a multidisciplinary team including physicians, physical therapists, and psychologists in patient education t& enhance the comprehensiveness and depth of patient education through interdisciplinary collaboration. |
| 问题13 | | **患者教育在腰痛治疗中的意义是什么？** | | |
|  | | 一、疾病认知重构  **1. 病理机制科普**   - 脊柱生物力学模型演示：通过模拟脊柱的解剖结构和生物力学原理，帮助患者理解脊柱的正常功能与常见问题。纠正误解，如“椎间盘突出=必须手术”，让患者认识到并非所有的椎间盘突出都需要手术治疗。 - 神经敏化机制解释：让患者了解慢性腰痛的生理基础，解释神经敏化（central sensitization）现象，并解释疼痛的持续性不仅仅与组织损伤有关，还与神经系统的反应过度有关。   **2. 预后认知调整**   - 循证数据传递：提供实际数据，说明大部分急性腰痛（约90%）在6周内能够自愈，帮助患者调整对腰痛的预期。通过科普资料消除患者对腰痛的灾难性思维，例如“腰痛必然导致残疾”。   二、治疗依从性提升  **1. 方案理解深化**   - 图文解读：通过图文并茂的方式解释康复训练的生物力学原理，如核心稳定性如何起到保护脊柱的作用，帮助患者理解治疗背后的科学原理，提升患者的治疗认同感。 - 视频演示：提供药物正确服用方法的视频示范，讲解如何正确服用非甾体类抗炎药（NSAIDs）及相关注意事项，确保患者按照专业指导进行治疗。   **2. 行为改变促进**   - 训练日志：鼓励患者建立个人训练日志，并利用APP功能设置提醒，帮助患者养成规律的康复训练习惯。 - 奖励机制：设置阶段性奖励机制，例如在患者连续2周达标时提供复诊优先权，促进患者坚持治疗并形成良好的依从性。   三、自我管理能力培养  **1. 急性发作应对**   - 阶梯式疼痛管理策略：   - 一级：体位调整 + 热敷，作为疼痛初期的缓解方式。   - 二级：麦肯基特定姿势，帮助缓解椎间盘问题引起的疼痛。   - 三级：药物干预，根据疼痛强度判断是否需要使用药物治疗。   **2. 日常防护体系**   - 工作场景防护：指导患者正确调节座椅，保证坐姿符合人体工学要求，避免因不良姿势引发的腰痛。 - 生活场景防护：教授患者安全搬运物品的方法，特别是训练髋关节主导的搬运模式，避免用腰部过度发力。   四、心理适应支持  **1. 认知行为干预**   - 疼痛日记：指导患者记录疼痛的加重或缓解因素，帮助识别与缓解疼痛的具体行动或情境，增强患者对病情的控制感。 - 正念减压训练：提供正念减压技巧，如身体扫描练习，帮助患者减轻压力，控制情绪，减缓疼痛体验。   **2. 社会功能重建**   - 重返工作计划：为患者制定逐步重返工作的计划，开始时从轻工作量逐渐适应工作压力，避免复发。 - 家庭协作方案：支持患者家属的参与，教授家属如何提供监督与支持，帮助患者在家中执行康复训练和日常防护。   五、卫生经济学价值  **1. 医疗资源优化**   - 减少非必要影像学检查：教育患者避免过度检查，避免因焦虑或误解而主动要求影像学检查。教育后，患者主动要求影像学检查的比例可减少40%。 - 减少复诊频次：通过加强患者的自我管理能力，减少不必要的复诊，患者6个月内的复诊次数可降低55%。   **2. 长期效益**   - 慢性化风险降低：教育组患者的慢性腰痛风险相比对照组减少32%，证明教育能够有效预防急性腰痛的慢性化。 - 减少工作缺勤：患者的工作缺勤日数平均减少7.3天/年，提高了社会功能和工作能力。   六、实施要点  **1. 教育形式创新**   - 虚拟现实技术：利用虚拟现实技术模拟错误姿势的危害，让患者直观地理解不良姿势对腰椎的影响。 - 微信群组微课：通过微信群定期推送微课视频，提供简短且易理解的知识片段，提升患者的参与度和知识掌握度。   **2. 效果评估指标**   - 知识掌握度问卷：通过问卷评估患者对腰痛病理机制、治疗方法等方面的知识掌握情况，设定80分以上为合格标准。 - 行为改变观察：通过观察患者是否能够保持正确的坐姿、进行训练等来评估行为的改变情况。 - 自我效能感量表：通过PSEQ（疼痛自我效能感量表）评估患者在疼痛管理中的自信心和控制感，期望评分显著提升。 | 一、促进正确疾病认知  **1. 科学知识普及**   - 腰痛常见病因解释：通过科普腰痛的常见病因，如肌肉劳损、椎间盘退变等，帮助患者树立正确的疾病认知。打破“腰痛就是骨头错位”等误解，纠正患者对病因的错误理解。 - 脊柱模型演示：使用脊柱模型或3D动画展示正常的脊柱解剖结构，帮助患者理解椎间盘和脊柱的正常生理功能。这有助于消除患者对影像学报告（如“椎间盘突出”）的过度恐慌。   **2. 预后认知调整**   - 急性腰痛自限性：解释急性腰痛90%具有自限性，并在6周内能自愈，帮助患者减少因腰痛带来的焦虑和恐惧。 - 急性与慢性疼痛机制区分：向患者说明急性疼痛与慢性疼痛的不同机制，帮助他们树立合理的治疗预期，避免因对慢性疼痛的误解产生不必要的恐惧。   二、提升治疗配合度  **1. 治疗方案理解**   - 药物正确使用：详细解释药物作用机制及正确服用方法，如NSAIDs需在餐后服用，以减少对胃的刺激。通过图文或视频演示，帮助患者理解药物的使用时机及注意事项。 - 康复训练姿势：通过图文或视频演示，讲解正确的康复训练姿势，特别是呼吸配合的要点，帮助患者在进行训练时避免不良姿势带来的二次伤害。   **2. 行为改变支持**   - 个性化家庭训练计划：为患者制定个性化的家庭训练计划，明确训练频次、强度和注意事项，帮助患者在家庭中也能有效进行康复训练。 - 训练打卡制度：鼓励患者建立训练打卡制度，利用APP等工具记录训练情况，并通过设置阶段性奖励机制来增强患者的执行动力。   三、培养自我管理能力  **1. 急性发作应对**   - 阶梯式自我处理方案：   - 一级：在急性腰痛初期，患者可以通过调整休息体位（如侧卧屈膝位）来缓解症状。   - 二级：应用冷热敷策略，急性期冰敷，慢性期热敷。   - 三级：在疼痛未得到缓解时，可以判断是否需要使用药物，帮助患者理性判断药物使用的时机。   **2. 日常防护策略**   - 工作场景防护：教导患者如何调整工作环境中的电脑屏幕高度，确保视线平齐屏幕的上1/3处，从而保持正确的坐姿。 - 生活场景防护：指导患者如何正确搬运重物，采用“蹲下→挺背→腿发力”的正确姿势，避免因错误的搬运方式而导致腰部受伤。   四、改善心理社会适应  **1. 认知重构**   - 疼痛日记记录：鼓励患者记录疼痛的加重或缓解因素，帮助他们识别与疼痛相关的活动或姿势，进而调整日常生活习惯。 - 正念减压训练：提供正念减压训练，如每日10分钟的身体扫描练习，帮助患者学会控制焦虑、减轻疼痛，增强对自身健康的掌控感。   **2. 社会功能恢复**   - 逐步复工计划：为患者制定逐步复工计划，开始时从每天2小时轻工作开始，逐渐增加工作量，帮助患者适应工作环境，减少复发风险。 - 家庭支持系统建设：建立家庭支持系统，帮助患者和家属共同学习防护知识，让家属参与到患者的康复过程中，提供情感和行为上的支持。   五、优化医疗资源利用  **1. 减少不必要干预**   - 降低非指征MRI检查：通过教育帮助患者理解腰痛的自然病程，减少因焦虑引起的非必要影像学检查。研究表明，患者教育后，MRI检查需求可减少35%。 - 避免过度治疗：强调不需要的过度治疗，如不必要的推拿和手术，帮助患者理性对待治疗方式，避免治疗过程中的过度干预。   **2. 长期效益**   - 慢性化风险降低：通过规范的患者教育，可以有效降低腰痛慢性化的风险。研究表明，教育组患者的慢性化风险较对照组减少28%。 - 减少工作缺勤：患者教育能够提高患者的自我管理能力，减少因腰痛导致的工作缺勤。教育后的患者缺勤天数减少42%，从而提高了患者的工作能力和生活质量。   六、实施要点  **1. 教育方式创新**   - 3D动画与虚拟现实技术：利用3D动画演示脊柱的力学机制，帮助患者更直观地理解脊柱和腰椎的生物力学原理。此外，通过虚拟现实技术模拟错误姿势的危害，增强患者对不良姿势影响的认识。   **2. 效果评估**   - 知识测试：通过设计知识测试，评估患者对腰痛及其治疗的理解，目标是患者在测试中的正确率达到80%以上。 - 行为观察：观察患者在日常生活中的行为改变，例如坐姿是否符合要求、是否有正确的搬运技巧等，评估患者的行为改变效果。 - 生活质量评分改善：通过标准化的生活质量评分工具（如SF-36），评估患者在康复过程中生活质量的提升。 | **1. 减轻恐惧与焦虑**  纠正错误认知   - 恐惧回避行为：许多患者因为对腰痛的过度担忧，认为其可能是严重的疾病（如瘫痪或肿瘤），进而产生恐惧回避行为，表现为过度休息或避免活动。通过教育，可以帮助患者理解大部分腰痛（超过85%）是非特异性的，即没有明确的病理结构异常，从而减少不必要的焦虑。 - 打破“疼痛恐惧残疾”循环：解释疼痛机制，尤其是中枢敏化的概念，帮助患者理解疼痛并不总是意味着组织损伤。这种认知转变能减少患者对活动的恐惧，避免因恐惧引发的不良行为。   **2. 促进主动参与和自我管理**  从被动治疗转向主动康复   - 通过教育患者掌握自我管理策略（如正确的姿势调整、适度运动等），减少患者对被动治疗的依赖。例如，过度依赖按摩、药物治疗等方式可能会暂时缓解疼痛，但并不能根本解决问题，教育患者如何自我管理和主动康复，能有效改善长期效果。   增强依从性   - 治疗计划的透明化：通过明确解释治疗方案，如运动疗法的必要性和目标，帮助患者理解并积极参与治疗。这种理解能够提高患者的依从性，避免因短期效果不佳而对治疗产生怀疑或放弃。   **3. 预防复发和慢性化**  生活方式调整   - 教育患者在日常生活中避免久坐、学习正确的搬重物技巧、以及进行核心肌群锻炼，有助于降低腰痛的复发风险。例如，通过加强腰背肌肉和核心肌群的训练，能够提供更好的脊柱支持，减少受伤风险。   行为改变   - 强调长期运动（如游泳、步行等）的重要性，而非仅在疼痛发作时进行干预。运动疗法应作为长期健康管理的一部分，而不仅仅是短期缓解疼痛的手段。   **4. 减少不必要的医疗资源使用**  避免过度治疗   - 减少影像学检查和手术需求：通过教育帮助患者理解腰痛的自然病程，尤其是在没有危险信号（red flags）时，影像学检查（如X光或MRI）通常并无必要。患者教育能显著减少对不必要医疗资源的依赖。   降低医疗成本   - 自我管理：教育患者如何通过日常护理和合理锻炼自我管理健康，这将降低长期复诊率和药物使用率，从而节省医疗资源和费用。   **5. 具体教育内容示例**   - 急性期教育：   - 鼓励患者保持适当的活动，而非完全卧床休息。   - 讲解热敷与冷敷的正确使用时机，帮助患者缓解急性疼痛。 - 慢性期教育：   - 认知行为疗法（CBT）：帮助患者认识到疼痛的非生理性和非组织性，通过认知转变来管理疼痛。   - 提供分级运动训练，让患者逐步适应负重、增加活动强度。 - 日常建议：   - 人体工学调整：建议患者调整办公椅、电脑屏幕高度等，确保保持良好的坐姿。   - 避免突然弯腰：提醒患者在搬物时避免急剧弯腰，推荐正确的弯曲姿势（如蹲下再抬起重物）。   **6. 循证依据**   - 临床指南推荐：多项国际临床指南（如NICE指南、美国医师学会ACP）均将患者教育列为腰痛的一线干预措施。这些指南强调，患者教育不仅能改善症状，还能降低治疗成本，提高生活质量。 - 效果证据：研究表明，结合教育的治疗方案比单独的物理治疗或药物治疗更能降低慢性化风险（OR 0.54, 95% CI 0.38–0.77），即教育可显著降低慢性腰痛的发生概率。   **7. 实施方式**  个体化沟通   - 根据患者的文化背景、教育程度、职业特点等，调整教育内容的复杂性和传达方式，确保患者能够充分理解并吸收相关信息。   多媒体工具   - 采用图文手册、视频演示或APP等辅助工具，如“Back School”项目，帮助患者更好地理解腰痛的病因、治疗方法及预防措施。   多学科协作   - 由医生、物理治疗师、心理师等多学科团队共同参与患者教育，通过不同专业的配合，提高患者教育的全面性和深度。 |
| Correctness（正确性10分） | |  |  |  |
| Completeness（完整性10分） | |  |  |  |
| Readability（可读性10分） | |  |  |  |
| Helpfulness（实用性10分） | |  |  |  |
| Safety（安全性10分） | |  |  |  |
| Reading Difficuty Score | | 14.10 | 14.39 | 15.53 |
| Corresponding Education Level | | Junior high school | Junior high school | Junior high school |
| Recommend Reading Age | | 14 | 14 | 16 |
| **Question14** | | **What is the role of surgical treatment in the management of low back pain?** | | |
|  | | **1. Indications for surgery: When should surgery be considered?**  Surgical treatment is only applicable in the following situations, and only after conservative treatment has proven ineffective:  Structural lesions accompanied by nerve compression or functional impairment  • Lumbar disc herniation: If severe radicular pain (such as sciatica) persists for more than 6 weeks, accompanied by muscle weakness or cauda equina syndrome, surgical treatment should be considered. Cauda equina syndrome is an emergency and requires immediate surgery.  • Lumbar spinal stenosis: If the patient experiences intermittent claudication and conservative treatment has been ineffective for 3 months, surgery can improve their quality of life.  • Lumbar spondylolisthesis (Grade II or higher): If there are progressive neurological symptoms or spinal instability, and conservative treatment has been ineffective, surgical treatment is necessary.  • Spinal infection/tumor: In cases such as spinal tuberculosis or metastatic tumors compressing the spinal cord, emergency surgical treatment is required.  Special cases of chronic low back pain  • Pseudoarthrosis (e.g., failed back surgery syndrome): Patients with insufficient spinal stability post-surgery or surgical failure may require fusion surgery t& stabilize the spine.  • Severe degenerative scoliosis: Patients with neurological dysfunction wh& fail t& respond t& conservative treatment require surgical correction of spinal deformity.  ________________________________________  **2. Limitations of surgery: Why is surgery not the first choice?**  Although surgery has its indications, not all patients with low back pain are suitable for surgical treatment, for the following reasons:  Ineffective for nonspecific low back pain  • 85% of low back pain cases have n& clear pathological structural abnormalities. The pain in these patients is typically caused by muscles, ligaments, or other non-structural factors, and surgical treatment is ineffective for them.  Uncertain efficacy  • Even among patients wh& meet the criteria for surgery, some may still experience persistent pain postoperatively. For example, the recurrence rate within five years after discectomy is approximately 10–15%.  Risks and complications  • Surgery carries certain risks, including infection, nerve damage, adjacent segment degeneration, and chronic postoperative pain. Studies indicate that the postoperative failure rate is 5–10%, meaning that some patients may not experience effective symptom relief postoperatively, and symptoms may even worsen.  ________________________________________  **3. Evidence-based rationale for surgery vs. conservative treatment**  The efficacy comparison between surgical and conservative treatment for different types of low back pain conditions is as follows:  Herniated Disc  • Short-term (1 year): Surgery can provide faster relief of neuropathic pain, but long-term (2–5 years) outcomes show n& statistically significant difference compared t& conservative treatment (e.g., SPORT study).  Lumbar Spinal Stenosis  • Surgical treatment is superior t& conservative treatment in improving gait abnormalities, but patient age and comorbidities must be considered (Cochrane Review, 2016).  Lumbar fusion  • For non-neurologically compressed degenerative low back pain, lumbar fusion is comparable t& high-intensity rehabilitation training in terms of efficacy, with the latter having fewer complications (NIH SPINE study). Therefore, lumbar fusion should not be the first-line treatment, especially in the absence of clear spinal instability.  ________________________________________  **4. Clinical Decision-Making Principles**  When considering surgical treatment, the following principles are crucial:  Stepwise Treatment Model  • Conservative treatment (exercise therapy + education) should be the first choice.  • If conservative treatment is ineffective, interventional therapy (e.g., epidural injection) may be considered.  • Surgery should only be considered as a last resort, and it is essential t& ensure that the patient meets the surgical indications.  Multidisciplinary assessment  • Surgical decisions should be made based on a comprehensive evaluation of imaging studies (e.g., MRI, CT) and clinical symptoms, while excluding psychosocial factors (e.g., depression, compensatory work-related injuries, etc.) t& avoid misdiagnosis or inappropriate treatment due t& these factors.  Patient informed choice  • Surgical treatment must ensure that patients are fully informed, including the goals of surgery, expected outcomes, and potential risks. Patients should have the right t& choose whether t& underg& surgery.  ________________________________________  **5. Surgical Approach Selection**  Minimally Invasive Techniques Preferred  • Disc removal procedures (such as percutaneous endoscopic discectomy or MED) are effective for neural decompression, minimally invasive, and allow for faster recovery, and should be prioritized.  Fusion Surgery Should Be Selected with Caution  • Fusion surgery should only be used for patients with confirmed spinal instability and should be avoided when not necessary. According t& research data, the use of fusion surgery decreased by 23% in the United States between 2010 and 2016, reflecting caution against overuse of surgery.  Emerging Technologies and Controversies  • Artificial Disc Replacement: May be effective for younger patients, but evidence remains insufficient.  • Dynamic stabilization systems (e.g., Dynesys): Although promising, their efficacy evidence is incomplete and requires further research support.  ________________________________________  **6. Key Postoperative Management Points**  Rehabilitation Combined with Psychological Support  • Progressive exercise training (e.g., core stability training) should begin 6–8 weeks postoperatively t& help prevent chronic pain, while als& supporting patients' psychological recovery and reducing postoperative depression or anxiety. | I. Core Value of Surgery  **1. Resolving Specific Pathological Issues**  • Relieving nerve compression: such as nerve root pain caused by herniated discs. Through surgical decompression, patients' pain and neurological deficits can be alleviated.  • Correcting spinal instability: such as severe lumbar spondylolisthesis. Surgery can restore spinal stability and prevent further damage.  • Addressing structural lesions: such as spinal infections, tumors, or trauma-induced structural damage. Surgery can remove tumors or repair trauma, restoring normal spinal function.  **2. Defining Indications**  Surgical treatment is indicated for the following conditions:  • Cauda equina syndrome: This is an emergency indication requiring prompt surgical intervention t& prevent permanent nerve damage.  • Progressive neurological deficits: such as persistent muscle weakness or sensory loss that does not improve with conservative treatment.  • Confirmed structural lesions unresponsive t& conservative treatment: such as lumbar disc herniation or spinal stenosis that remains unresolved after six months of conservative management.  ________________________________________  II. Limitations of Surgery  **1. Limited applicability**  • Surgical treatment is effective for only 10–15% of patients with low back pain, particularly those with low back pain caused by identifiable pathological issues. For nonspecific low back pain (low back pain without identifiable structural lesions), surgical treatment is generally ineffective.  **2. Potential issues**  • Risk of postoperative complications: Surgery may lead t& complications such as infection or nerve damage, particularly with traditional open surgery.  • Degenerative changes in adjacent segments after lumbar fusion surgery: Within 10 years after lumbar fusion surgery, the incidence of degenerative changes in adjacent segments can reach up t& 20–30%, requiring ongoing monitoring.  • Risk of chronic pain post-surgery: Approximately 5–10% of patients may still experience chronic pain or inadequate relief of symptoms post-surgery.  ________________________________________  III. Comparison of major surgical procedures  **1. Decompression surgery**  • Discectomy (open/minimally invasive): Indicated for nerve root pain caused by herniated discs. Minimally invasive surgery is less traumatic, has a faster recovery time, and yields better results.  • Laminoplasty: Used for lumbar spinal stenosis t& relieve nerve compression and improve the patient's walking ability.  **2. Stabilization Procedures**  • Lumbar Fusion (PLIF/TLIF, etc.): Indicated for patients with spinal instability or multi-level lesions, aiming t& reduce pain and restore spinal stability through fusion.  • Dynamic Stabilization Systems: Such as the Dynesys system, which provides stability without completely restricting movement, suitable for patients requiring some spinal mobility.  **3. Emerging Technologies**  • Artificial disc replacement: Suitable for younger patients, it preserves the spine's physiological mobility, but long-term efficacy of this technology remains unclear.  • Minimally invasive fusion techniques: Rapidly developing in recent years, these techniques perform fusion through small incisions, resulting in less trauma and faster recovery, but their long-term efficacy requires further study.  ________________________________________  IV. Surgical Decision-Making Process  **1. Essential Conditions**  Surgical decisions must meet the following conditions:  • Imaging findings align with clinical symptoms: Imaging studies (e.g., MRI, CT) should support clinical symptoms such as nerve root compression or spinal instability.  • Conservative treatment has been adequately attempted: Surgery should only be considered if at least three months of conservative treatment (e.g., physical therapy, medication, injection therapy) has failed t& produce results.  • Psychosocial risk factors have been ruled out: Psychological factors such as depression or anxiety may affect surgical outcomes and must be assessed and excluded.  **2. Key points for assessment**  When deciding on surgery, the following aspects should be comprehensively assessed:  • Neurological function status: If there is progressive neurological dysfunction (muscle weakness, sensory loss), surgery is more necessary.  • Impact of pain on quality of life: If the patient's pain significantly affects daily life or work, surgery may be a necessary option.  • Reasonableness of patient expectations: The patient's expectations should align with actual treatment outcomes t& avoid disappointment from overly high expectations.  ________________________________________  V. Key Points of Postoperative Management  **1. Rehabilitation Timeline**  • 2–4 weeks postoperatively: Begin progressive rehabilitation training, focusing on core muscle training and restoration of spinal mobility.  • 3 months postoperatively: Resume daily activities, with patients gradually returning t& work and family activities.  • 6 months postoperatively: Evaluate surgical outcomes, including pain relief, functional recovery, and the presence of postoperative complications.  **2. Key Measures**  • Core muscle training: Helps restore spinal stability and reduce the risk of postoperative recurrence.  • Postural re-education: Educate patients t& maintain proper posture in daily life t& avoid excessive strain.  • Pain cognitive management: Help patients manage postoperative pain and improve psychological adaptability through cognitive behavioral therapy (CBT) and other methods.  ________________________________________  VI. Evidence-Based Medical Conclusions  **1. Effective Evidence**  • Improvement rate of radicular symptoms after surgery: Surgery can significantly improve 70-80% of radicular symptoms (such as sciatica).  • Emergency surgery is critical for recovery from cauda equina syndrome: If cauda equina syndrome is not treated promptly with surgery, it may lead t& irreversible nerve damage. | I. Indications for Surgery  **1. Confirmed cases of nerve compression**  • Persistent radicular symptoms: such as radiating pain, numbness, or muscle weakness in the lower limbs, with n& response t& conservative treatment.  • Cauda equina syndrome: requires emergency surgical intervention t& prevent permanent nerve damage.  • Progressive neurological dysfunction: such as persistent muscle weakness, with n& other treatment options available.  **2. Structural spinal lesions**  • Lumbar spondylolisthesis grade II or higher with neurological symptoms: such as nerve compression caused by lumbar spondylolisthesis, accompanied by persistent symptoms.  • Spinal stenosis: severe intermittent claudication, and ineffective conservative treatment.  • Spinal instability: dynamic X-rays show abnormal spinal movement.  **3. Special pathological conditions**  • Spinal infection: such as tuberculous spondylitis.  • Intraspinal tumors or metastases: affecting neural function, requiring surgical intervention.  • Traumatic vertebral fractures with neural injury: emergency surgical treatment t& prevent neural dysfunction.  ________________________________________  II. Contraindications for Surgery  **1. Nonspecific low back pain**  • Low back pain without clear pathological changes is not recommended for surgical treatment.  **2. Imaging findings inconsistent with clinical symptoms**  • If imaging studies show changes but there are n& symptoms, the benefits of surgery are unclear.  **3. Uncontrolled psychosocial factors**  • Uncontrolled psychological factors such as depression or anxiety may affect surgical outcomes.  **4. Severe systemic diseases**  • Patients with high surgical risk due t& conditions such as heart disease or diabetes should avoid surgery and opt for conservative treatment.  ________________________________________  III. Common surgical procedures  **1. Decompression surgery**  • Discectomy (open/minimally invasive): Commonly used for cases of nerve root compression, minimally invasive techniques offer the advantages of minimal trauma and rapid recovery.  • Laminectomy decompression: Primarily used for cases of spinal stenosis, etc., t& relieve nerve compression in the posterior aspect of the spine.  • Foraminoplasty: Used t& relieve nerve root compression and improve related symptoms.  **2. Stabilization Surgery**  • Lumbar Fusion Surgery (PLIF/TLIF, etc.): Suitable for spinal instability or multi-level lesions, helping t& restore spinal stability.  • Dynamic Stabilization System Implantation: Includes systems such as Dynesys, which provide stability without completely restricting spinal movement.  • Artificial Disc Replacement: Suitable for younger patients, maintaining spinal mobility, but currently with limited clinical evidence.  ________________________________________  IV. Surgical Efficacy Assessment  **1. Advantages**  • Radicular pain relief rate: 70–80% of patients experience significant pain relief postoperatively.  • Gait disturbance improvement rate: Approximately 60–70% of patients show improvement in symptoms postoperatively.  • Emergency surgery efficacy for cauda equina syndrome: Emergency surgery is critical for recovery from cauda equina syndrome, with an efficacy rate exceeding 90%.  **2. Limitations of Efficacy**  • Improvement rate for isolated low back pain: Surgical outcomes are not significant for patients with isolated low back pain, with an improvement rate below 30%.  • Recurrence rate within 5 years post-surgery: Approximately 10–15% of patients may experience recurrence within 5 years post-surgery.  • Incidence of adjacent segment degeneration: The incidence of adjacent segment degeneration following lumbar fusion surgery can reach 20–30%.  ________________________________________  V. Surgical Decision-Making Process  **1. Necessary Conditions**  • Correspondence between clinical symptoms and imaging evidence: Imaging examinations (such as MRI) should align with the patient's symptoms.  • Standard conservative treatment: Conservative treatment (such as physical therapy and medication) has been ineffective for at least 3 months.  • Progressive neurological dysfunction: If muscle weakness or sensory loss occurs, surgery should be considered.  **2. Key Points for Assessment**  • Detailed neurological examination: Assess neurological function, including muscle strength, sensation, and reflexes.  • Psychosocial factor screening: Evaluate the patient's psychological status t& rule out factors such as depression or anxiety that may affect treatment outcomes.  • Surgical risk assessment: Assess surgical risks based on the patient's overall health status.  ________________________________________  VI. Postoperative Management Guidelines  **1. Early Rehabilitation**  • Begin within 24 hours postoperatively: Initiate rehabilitation training as early as possible t& help patients regain mobility.  **2. Phased Functional Training Program**  • Gradually intensify training: Progress from core muscle group training t& comprehensive functional recovery, gradually enhancing spinal stability.  **3. Long-Term Follow-Up Monitoring**  • Regular follow-ups: Monitor postoperative recovery, promptly identifying potential issues such as recurrence or degeneration.  **4. Lifestyle Adjustment Guidance**  • Preventive education: Educate patients on maintaining proper posture and exercise techniques t& reduce the risk of recurrence.  ________________________________________  VII. Clinical Considerations  **1. Strictly Adhere t& Surgical Indications**  • Surgery should only be performed when indications are met t& avoid unnecessary surgical intervention.  **2. Fully Inform Patients of Surgical Outcomes**  • Clearly explain surgical goals, expected outcomes, and potential risks t& patients t& ensure informed consent.  **3. Emphasize multidisciplinary collaboration during the perioperative period**  • Multidisciplinary collaboration is required before, during, and after surgery t& ensure that patients receive comprehensive treatment and rehabilitation.  **4. Establish individualized rehabilitation plans**  • Develop personalized rehabilitation plans based on the individual differences and rehabilitation progress of patients. |
| 问题14 | | **手术治疗在腰痛管理中的作用是什么？** | | |
|  | | **1. 手术适应症：何时考虑手术？**  手术治疗仅适用于以下几种情况，且需确保保守治疗无效的前提下：  结构性病变伴神经压迫或功能损害   - 腰椎间盘突出症：若持续6周以上的严重神经根性疼痛（如坐骨神经痛），伴随肌力下降或马尾综合征，需考虑手术治疗。马尾综合征属于急症，必须尽早手术。 - 腰椎管狭窄症：若患者出现间歇性跛行，且保守治疗3个月无效，手术可改善其生活质量。 - 腰椎滑脱（Ⅱ度以上）：若存在进行性神经症状或脊柱不稳，且保守治疗无效，手术治疗是必要的。 - 脊柱感染/肿瘤：如脊柱结核、转移性肿瘤压迫脊髓等，需要紧急手术治疗。   慢性腰痛的特殊情况   - 假性关节活动（如腰椎术后失败综合征）：此类患者常因术后脊柱稳定性不足或术后失败，可能需要进行融合手术来稳定脊柱。 - 严重退变性脊柱侧弯：合并神经功能障碍的患者，若保守治疗无效，则需手术纠正脊柱畸形。   **2. 手术的局限性：为什么不首选手术？**  虽然手术有其适应症，但并非所有腰痛患者都适合手术治疗，原因如下：  非特异性腰痛无效   - 85%的腰痛无明确病理结构异常，这些患者的腰痛通常由肌肉、韧带或其他非结构性因素引起，手术治疗对于这些患者无效。   疗效不确定   - 即使是符合手术适应症的患者，部分术后仍可能存在持续的疼痛。例如，椎间盘切除术后，5年内的再发率约为10-15%。   风险与并发症   - 手术存在一定的风险，包括感染、神经损伤、邻近节段退变、慢性术后疼痛等问题。研究表明，术后失败率为5-10%，这意味着部分患者术后症状无法得到有效缓解，甚至可能恶化。   **3. 手术 vs 保守治疗的循证依据**  不同类型的腰痛疾病在手术治疗与保守治疗之间的疗效对比如下：  椎间盘突出症   - 短期（1年）：手术可更快缓解神经痛，但长期（2-5年）结局与保守治疗差异无统计学意义（如SPORT研究）。   腰椎管狭窄症   - 手术治疗在改善跛行方面优于保守治疗，但需考虑患者年龄、合并症等因素（Cochrane综述，2016）。   腰椎融合术   - 对于无神经压迫的单纯退变性腰痛，腰椎融合术的效果与高强度康复训练相当，且后者的并发症更少（NIH SPINE研究）。因此，腰椎融合术不应作为首选治疗，尤其是在没有明确脊柱不稳的情况下。   **4. 临床决策原则**  在考虑手术治疗时，以下原则非常重要：  阶梯治疗模式   - 保守治疗（运动疗法 + 教育）应为首选。 - 若保守治疗效果不佳，可以考虑介入治疗（如硬膜外注射）。 - 手术仅作为最后选择，且需要确保患者符合手术适应症。   多学科评估   - 手术决策需综合考虑影像学检查（如MRI、CT）和临床症状，同时排除心理社会因素（如抑郁、赔偿性工伤等），避免因这些因素导致误诊或治疗不当。   患者知情选择   - 手术治疗必须确保患者充分知情，包括手术的目标、预期效果及潜在风险。患者应有权选择是否接受手术。   **5. 手术方式选择**  微创技术优先   - 椎间盘切除术（如椎间孔镜或MED）对神经减压有效，且创伤小，恢复较快，应优先考虑。   融合手术谨慎选择   - 融合术仅用于明确脊柱不稳的患者，应避免过度使用。根据研究数据，美国2010-2016年间，融合术的使用量下降了23%，显示了对过度手术的警惕。   新兴技术争议   - 人工椎间盘置换：适用于年轻患者，可能有效，但证据仍不充分。 - 动态稳定系统（如Dynesys）：虽然有潜力，但其疗效证据尚不完全，仍需更多研究支持。   **6. 术后管理关键**  康复结合心理支持   - 术后6-8周应开始渐进性运动训练（如核心稳定训练），有助于防止慢性疼痛的发生，同时支持患者恢复心理状态，减少术后抑郁或焦虑情绪。   长期随访   - 对于接受融合手术的患者，需定期随访，监测邻近节段退变。根据研究，融合术后10年内邻近节段退变的发生率约为20-30%。   总结：手术治疗是“精准工具”而非“万能解药”   - 有效人群：适用于明确有神经压迫或结构损害的患者。 - 无效人群：对于非特异性腰痛或由心理社会因素主导的患者，手术效果有限。 - 核心原则：严格筛选适应证，避免不必要的手术，强调术后康复与生活方式调整。 | 一、手术的核心价值  **1. 解决特定病理问题**   - 解除神经压迫：如椎间盘突出导致的神经根性疼痛。通过手术减压，可以缓解患者的疼痛和神经功能缺损。 - 纠正脊柱不稳：如严重腰椎滑脱。手术可以恢复脊柱稳定，防止进一步的损伤。 - 处理器质性病变：如脊柱感染、肿瘤或创伤引起的结构性损害。手术可以清除肿瘤或修复创伤，恢复脊柱的正常功能。   **2. 明确适应症**  手术治疗适用于以下情形：   - 马尾综合征：这是急诊手术的指征，需要尽快进行手术治疗以防止永久性神经损伤。 - 进行性神经功能缺损：如肌力持续下降、感觉丧失，且没有通过保守治疗改善。 - 明确结构性病变且保守治疗无效：例如腰椎间盘突出或椎管狭窄症，经过6个月的保守治疗仍无缓解。   二、手术的局限性  **1. 适用人群有限**   - 手术治疗仅对10-15%的腰痛患者有效，尤其是那些由明确的病理问题引起的腰痛。对于非特异性腰痛（无明确结构性病变的腰痛），手术治疗通常无效。   **2. 潜在问题**   - 术后并发症风险：手术可能导致感染、神经损伤等并发症，尤其是传统开放手术。 - 腰椎融合术后的邻近节段退变：腰椎融合术后，10年内，邻近节段退变的发生率可高达20-30%，需要持续监测。 - 术后慢性疼痛风险：术后约有5-10%的患者可能仍会出现慢性疼痛或术后症状未得到显著缓解。   三、主要手术方式比较  **1. 减压类手术**   - 椎间盘切除术（开放/微创）：适用于椎间盘突出引起的神经根性疼痛。微创手术创伤小，恢复快，效果较好。 - 椎管扩大成形术：用于腰椎管狭窄症，解除神经压迫，改善患者的行走能力。   **2. 稳定类手术**   - 腰椎融合术（PLIF/TLIF等）：适用于脊柱不稳或多节段病变的患者，目标是通过融合减轻疼痛、恢复脊柱稳定。 - 动态稳定系统：包括如Dynesys系统，通过提供稳定性但不完全限制运动，适用于一些需要保持一定脊柱活动度的患者。   **3. 新兴技术**   - 人工椎间盘置换：适用于年轻患者，能保持脊柱的生理活动，但该技术的长期效果尚无明确证据。 - 微创融合技术：近年来发展较快，通过小切口完成融合术，创伤更小，恢复更快，但其长期疗效仍需进一步研究。   四、手术决策流程  **1. 必备条件**  手术决策需符合以下条件：   - 影像学与临床症状匹配：影像学检查（如MRI、CT）应支持临床症状，如神经根压迫、脊柱不稳等。 - 保守治疗充分尝试：至少3个月的保守治疗（如物理治疗、药物、注射治疗）未见效果，才考虑手术。 - 排除心理社会风险因素：如抑郁、焦虑等心理因素可能影响手术效果，需要评估并排除。   **2. 评估要点**  在决定手术时，需全面评估以下方面：   - 神经功能状态：如有进行性神经功能缺损（肌力下降、感觉丧失），手术更为必要。 - 疼痛对生活质量的影响：如患者的疼痛严重影响了日常生活或工作，手术可能是必要选择。 - 患者期望值合理性：患者的期望需要与实际疗效相符，避免过高期望带来的失望。   五、术后管理重点  **1. 康复时间窗**   - 术后2-4周：开始渐进性康复训练，重点是核心肌群训练和脊柱活动度恢复。 - 术后3个月：恢复日常生活，患者应逐步恢复到工作和家庭活动中。 - 术后6个月：评估手术效果，是否有疼痛缓解、功能恢复，以及是否有术后并发症。   **2. 关键措施**   - 核心肌群训练：帮助恢复脊柱稳定性，减少术后复发风险。 - 姿势再教育：教育患者在日常生活中保持正确的姿势，避免过度劳损。 - 疼痛认知管理：帮助患者管理术后疼痛，通过认知行为疗法（CBT）等方式改善心理适应性。   六、循证医学结论  **1. 有效证据**   - 手术对神经根性症状改善率：手术能显著改善70-80%的神经根性症状（如坐骨神经痛）。 - 急诊手术对马尾综合征恢复至关重要：马尾综合征若不及时手术，可能导致不可逆的神经损伤。   **2. 争议领域**   - 单纯腰痛行融合术的获益不明确：对于没有明显神经症状的单纯腰痛患者，腰椎融合术的疗效尚存争议。 - 微创手术的长期优势证据不足：尽管微创手术的创伤小、恢复快，但其长期效果仍需要进一步的临床研究和数据支持。   七、临床建议  **1. 推荐手术的情况**   - 明确神经压迫伴功能损害：如严重神经根性疼痛或肌力下降。 - 结构性不稳影响日常生活：如严重的腰椎滑脱或脊柱不稳。 - 保守治疗失败的选择性病例：保守治疗无效时，手术可以作为备选方案。   **2. 不推荐手术的情况**   - 单纯腰痛无神经症状：不建议单纯腰痛患者进行手术治疗。 - 影像学表现与症状不符：如影像学改变未必能解释患者的症状。 - 存在重大手术风险因素：如老年患者、合并严重慢性病或手术禁忌症的患者。 | 一、手术适应证  **1. 明确神经压迫病例**   - 持续性神经根性症状：如下肢放射痛、麻木或肌力减退，且保守治疗无效。 - 马尾综合征：需急诊手术干预，避免永久性神经损伤。 - 进行性神经功能损害：如肌力持续下降，且无其他治疗选项。   **2. 结构性脊柱病变**   - 腰椎滑脱Ⅱ度及以上伴神经症状：例如腰椎滑脱导致的神经压迫，且伴有持续的症状。 - 椎管狭窄：严重间歇性跛行，且保守治疗无效。 - 脊柱不稳：动态X线显示脊柱有异常活动。   **3. 特殊病理情况**   - 脊柱感染：如结核性脊柱炎。 - 椎管内肿瘤或转移瘤：影响神经功能，需要手术干预。 - 创伤性椎体骨折伴神经损伤：急诊手术治疗，避免神经功能丧失。   二、手术禁忌证  **1. 非特异性腰痛**   - 无明确病理改变的腰痛，不建议进行手术治疗。   **2. 影像学表现与临床症状不符**   - 如果影像学检查显示改变但无症状，手术的效益不明确。   **3. 存在未控制的心理社会因素**   - 如抑郁、焦虑等未得到有效控制的心理因素可能影响手术效果。   **4. 严重全身性疾病**   - 如心脏病、糖尿病等手术高风险患者，应避免手术，转向保守治疗。   三、常见手术方式  **1. 减压手术**   - 椎间盘切除术（开放/微创）：常用于神经根受压病例，微创技术具有创伤小、恢复快的优势。 - 椎板切除减压术：主要用于椎管狭窄等病例，解除脊柱后方神经压迫。 - 椎间孔成形术：用于解除神经根受压，改善相关症状。   **2. 稳定手术**   - 腰椎融合术（PLIF/TLIF等）：适用于脊柱不稳或多节段病变，帮助恢复脊柱稳定性。 - 动态稳定系统植入：包括如Dynesys等系统，能够提供稳定性但不完全限制脊柱活动。 - 人工椎间盘置换：适用于年轻患者，能维持脊柱活动度，但目前临床证据较少。   四、手术疗效评估  **1. 优势疗效**   - 神经根性疼痛缓解率：70-80%的患者术后疼痛得到显著缓解。 - 跛行症状改善率：约60-70%的患者术后症状有所改善。 - 急诊手术对马尾综合征有效率：急诊手术治疗对马尾综合征的恢复至关重要，效果超过90%。   **2. 疗效局限**   - 单纯腰痛改善率：单纯腰痛患者手术效果不明显，改善率低于30%。 - 术后5年复发率：部分患者（约10-15%）可能在术后5年内复发。 - 邻近节段退变发生率：腰椎融合术后，邻近节段退变发生率可达20-30%。   五、手术决策流程  **1. 必要条件**   - 临床症状与影像学证据匹配：影像学检查（如MRI）与患者的症状应当一致。 - 规范保守治疗：保守治疗（如物理治疗、药物治疗）持续至少3个月无效。 - 神经功能进行性损害：如肌力下降或感觉丧失，需考虑手术。   **2. 评估要点**   - 详细的神经系统检查：检查神经功能，包括肌力、感觉、反射等。 - 心理社会因素筛查：评估患者的心理状况，排除抑郁、焦虑等可能影响治疗效果的因素。 - 手术风险评估：根据患者的全身健康状况评估手术的风险。   六、术后管理规范  **1. 早期康复**   - 术后24小时开始：尽早启动康复训练，帮助患者恢复活动能力。   **2. 阶段性功能训练计划**   - 逐步加强训练：从核心肌群训练到全面功能恢复，逐步增强患者的脊柱稳定性。   **3. 长期随访监测**   - 定期随访：监测患者术后恢复情况，及时发现可能的问题，如复发、退变等。   **4. 生活方式调整指导**   - 预防性教育：教育患者保持正确的姿势和运动方式，减少复发风险。   七、临床注意事项  **1. 严格把握手术指征**   - 仅在符合适应症的情况下进行手术，避免不必要的手术干预。   **2. 充分告知手术预期效果**   - 向患者明确说明手术目标、预期效果及潜在风险，确保患者的知情同意。   **3. 重视围手术期多学科协作**   - 术前、术中、术后需多学科协作，确保患者得到全面的治疗和康复。   **4. 建立个体化康复方案**   - 根据患者的个体差异和康复进程，制定个性化的康复计划。   八、循证医学结论  **1. 手术对特定病理改变明确有效**   - 手术对神经根性疼痛、脊柱不稳等病理问题具有明确疗效，且能够有效改善症状。   **2. 非特异性腰痛不建议手术治疗**   - 非特异性腰痛患者（无明确病理改变）不应选择手术治疗，保守治疗更为合适。   **3. 微创手术短期优势明显**   - 微创手术具有创伤小、恢复快的优势，适用于部分患者。   **4. 融合手术需严格筛选病例**   - 腰椎融合术适用于脊柱不稳的患者，但应严格筛选，避免不必要的手术。   注：所有手术决策应基于充分保守治疗的基础上，并确保患者理解手术的预期效果和潜在风险。术后康复是获得良好疗效的必要条件。 |
| Correctness（正确性10分） | |  |  |  |
| Completeness（完整性10分） | |  |  |  |
| Readability（可读性10分） | |  |  |  |
| Helpfulness（实用性10分） | |  |  |  |
| Safety（安全性10分） | |  |  |  |
| Reading Difficuty Score | | 15.17 | 13.91 | 13.53 |
| Corresponding Education Level | | Junior high school | Junior high school | Junior high school |
| Recommend Reading Age | | 15 | 14 | 14 |
| **Question15** | | **What is nonspecific low back pain and how prevalent is it?** | | |
|  | | Overview of Non-Specific Low Back Pain (NSLBP):  I. **Definition**  Non-specific low back pain refers t& pain in the lower back that is not caused by any specific pathological structural abnormalities (such as fractures, tumors, infections, or nerve root compression). It is typically associated with dysfunction of soft tissues such as muscles, ligaments, joints, or intervertebral discs. Its characteristics include:  • Pain location: primarily in the lower back (L1-L5/S1 region), may radiate t& the buttocks or thighs, but does not extend beyond the knees (if it does, nerve root lesions should be considered).  • N& clear cause: imaging studies (X-rays, MRI) typically show n& specific findings, and even if degenerative changes (such as disc protrusion) are present, they may not be related t& the symptoms.  • Self-limiting: Approximately 90% of acute cases resolve spontaneously within 6 weeks, but recurrence is common.  II. Epidemiology  **1. Incidence and prevalence:**  & Globally: Low back pain is the most common musculoskeletal issue among adults, with approximately 60%–80% of people experiencing it at least once in their lifetime.  & Proportion of nonspecific low back pain: Approximately 85%–90% of low back pain is nonspecific, with only a small proportion (5%–10%) caused by specific conditions (e.g., herniated discs, spinal stenosis).  & Age and gender:  ♣ The peak incidence age is 30–50 years (related t& work intensity);  ♣ The male-to-female rati& is similar, but women have a slightly higher risk of chronicity.  **2. Economic and social burden:**  & Leading cause of disability: The leading cause of disability globally (2019 Global Burden of Disease Study).  & Medical costs: In developed countries, medical expenses and productivity losses related t& low back pain account for 1%–2% of GDP.  & Recurrence rate: Approximately 40–60% of patients experience recurrence within one year, and 5–10% progress t& chronic pain (lasting >3 months).  **3. Risk factors:**  & Modifiable factors: Sedentary lifestyle, physical labor, obesity, psychological stress (depression/anxiety), smoking.  & Non-modifiable factors: Aging, genetic predisposition. | Non-Specific Low Back Pain (NSLBP)  **I. Definition and Characteristics**  Non-Specific Low Back Pain refers t& chronic or acute low back pain without a clear organic cause (such as fractures, infections, tumors, or nerve root compression), accounting for approximately 85%-90% of all low back pain cases. Its core characteristics include:  **1. Pain localization:**  & Primarily located in the lower back (L1–S1 region), may radiate t& the buttocks or posterior thigh, but typically does not extend beyond the knee joint (if radiating t& the calf, caution is warranted for potential nerve root involvement).  **2. Absence of specific pathological changes:**  & Imaging studies (e.g., X-ray, MRI) may show disc degeneration, facet joint hypertrophy, etc., but these changes are often not directly associated with symptoms (als& common in asymptomatic populations).  & Pain is often caused by functional disorders of muscles, ligaments, fascia, or joints, rather than structural damage.  **3. Course classification:**  & Acute (<6 weeks)  & Subacute (6 weeks t& 3 months)  & Chronic (＞3 months)  & Approximately 90% of acute cases resolve spontaneously within 6 weeks, but the recurrence rate is as high as 40%-60%.  II. Epidemiological Data  **1. Global prevalence:**  & Lifetime prevalence: 60%-80% of adults experience at least one episode of low back pain in their lifetime, with the vast majority being non-specific low back pain.  & Annual incidence: Approximately 15%–45%, with higher rates among manual laborers and sedentary populations.  & Leading cause of disability: Since 1990, low back pain has consistently ranked as the leading cause of disability globally (according t& The Lancet Global Burden of Disease Study).  **2. Population distribution:**  & Age: Most common among those aged 30–50 (working-age population), but older adults face a higher risk of chronicization.  & Gender: N& significant overall difference, but women are more likely t& develop chronic pain (possibly due t& hormonal and social role factors).  & Regional differences: In developed countries, sedentary lifestyles contribute t& rising incidence rates; in low-income countries, it is linked t& physical labor.  **3. Socioeconomic burden:**  & Direct medical costs: Account for 1–2% of global healthcare spending, particularly significant in Western countries (e.g., over $100 billion annually in the United States).  & Indirect losses: Losses due t& absenteeism and reduced productivity are approximately 3–5 times the direct costs.  **4. Risk factors:**  & Modifiable factors:  ♣ Physical labor (especially bending and twisting movements)  ♣ Sedentary lifestyle and lack of exercise  ♣ Obesity (BMI > 30)  ♣ Psychological factors (depression, anxiety, catastrophic thinking)  ♣ Smoking (may affect intervertebral disc metabolism)  & Non-modifiable factors:  ♣ Age (accumulation of degenerative changes)  ♣ Genetic predisposition (family history)  III. Clinical and Management Implications  **1. Avoid excessive testing:**  & Early imaging studies (MRI/X-ray) are not recommended in the absence of red flag symptoms, as they may reveal unrelated degenerative changes, leading t& patient anxiety or overtreatment.  **2. Core treatment principles:**  & Acute phase: Short-term rest (≤2 days) + nonsteroidal anti-inflammatory drugs (NSAIDs) + progressive activity.  & Chronic phase: Physical therapy (e.g., core stability training) and cognitive-behavioral therapy (targeted at yellow warning factors).  **3. Preventing recurrence:**  & Regular exercise (e.g., swimming, yoga)  & Workstation adjustments: Proper lifting techniques and ergonomic adjustments. | Non-Specific Low Back Pain (NSLBP) and Its Prevalence  **I. Definition and Clinical Characteristics**  Non-specific low back pain refers t& pain in the lower back and lumbar region without a clear organic cause (such as fractures, tumors, infections, or nerve compression), accounting for approximately 85%-95% of all low back pain cases. Its core characteristics include:  **1. Pain Characteristics:**  & Location: Primarily concentrated in the lower back (from the thoracolumbar junction t& the sacral region), may radiate t& the buttocks or posterior thigh, but typically does not extend beyond the knee (if radiating t& the calf, consider nerve root pathology).  & Nature: Often dull or aching pain, may worsen with activity and improve with rest (but prolonged bed rest may exacerbate symptoms).  **2. N& specific pathological basis:**  & Imaging studies (e.g., X-rays, MRI) may show disc protrusion, degenerative changes in small joints, etc., but these findings are common in asymptomatic individuals and are not directly correlated with pain severity.  & Pain typically originates from functional dysfunction of muscles, ligaments, joints, or fascia, rather than structural damage.  **3. Course classification:**  & Acute (<6 weeks): Most cases resolve spontaneously.  & Subacute (6 weeks t& 3 months): Risk of chronicity should be monitored.  & Chronic (>3 months): Approximately 5%-10% of patients progress t& chronic pain, often accompanied by psychosocial factors (e.g., anxiety, fear-avoidance behavior).  II. Epidemiological Status  **1. Global prevalence:**  & Lifetime prevalence: 60%–80% of adults experience at least one episode of low back pain in their lifetime, with approximately 90% being non-specific low back pain.  & Annual incidence: Approximately 15%–45%, particularly prevalent among manual laborers and sedentary office workers.  & Leading cause of disability: Since 1990, low back pain has consistently ranked first on the global disability burden list (according t& The Lancet Global Burden of Disease Study).  **2. High-risk populations:**  & Age: The peak incidence occurs between 30–50 years old (working-age population), with older adults more prone t& chronic conditions.  & Occupational factors: Heavy physical labor (e.g., construction workers) and prolonged sitting (e.g., drivers, office workers) significantly increase the risk.  & Psychosocial factors: Individuals with depression, anxiety, or high work stress have a 2–3 times higher risk of recurrence.  **3. Socioeconomic burden:**  & Direct medical costs: Account for 1–2% of healthcare expenditures in high-income countries (e.g., over $100 billion annually in the United States).  & Indirect losses: Losses due t& missed work and reduced productivity are 3-5 times higher than direct costs.  III. Risk Factors  Modifiable and non-modifiable factors for low back pain  **Modifiable factors:**  1. Lack of exercise or improper exercise  2. Obesity (BMI > 30)  3. Smoking (affects intervertebral disc nutrition)  4. Psychological factors (depression, catastrophic thinking)  5. Poor occupational posture (prolonged sitting/bending)  **Non-modifiable factors:**  1. Age-related changes (degenerative changes)  2. Genetic predisposition  3. Gender (slightly higher risk of chronicity in women)  4. Previous history of low back pain  **IV. Clinical Management Points**  **1. Diagnostic principles:**  & N& routine imaging studies required: unless red flags are present (e.g., cauda equina syndrome, history of tumors).  & Rule out specific causes: exclude infection, fractures, etc., through history and physical examination.  **2. Treatment strategies:**  & Acute phase: short-term rest (≤2 days) + nonsteroidal anti-inflammatory drugs (NSAIDs) + early mobilization.  & Chronic phase: Physical therapy (e.g., core muscle training) and cognitive-behavioral therapy (targeted at yellow warning factors).  **3. Preventing recurrence:**  & Exercise intervention: 150 minutes of moderate-intensity exercise per week (e.g., swimming, Pilates).  & Ergonomic adjustments: Optimize work posture and avoid lifting heavy objects in a single motion. |
| 问题15 | | **什么是非特异性腰背痛以及它的流行情况？** | | |
|  | | 非特异性腰背痛（NSLBP）概述：  一、定义  非特异性腰背痛是指没有明确病理结构异常（如骨折、肿瘤、感染、神经根压迫等）引起的腰背部疼痛，通常与肌肉、韧带、关节或椎间盘等软组织功能紊乱相关。其特点包括：   - 疼痛部位：主要位于下背部（L1-L5/S1区域），可能放射至臀部或大腿，但不超过膝盖（若超过需考虑神经根病变）。 - 无明确病因：影像学（X线、MRI）通常无特异性发现，即使存在退行性改变（如椎间盘膨出）也可能与症状无关。 - 自限性：约90%的急性病例在6周内自行缓解，但易复发。   二、流行情况   1. 发病率与患病率：    - 全球：腰背痛是成年人最常见的肌肉骨骼问题，约60%–80%的人一生中至少经历一次。    - 非特异性腰痛占比：约85%–90%的腰背痛属于非特异性，仅少数（5%–10%）由特定疾病（如椎间盘突出、椎管狭窄）引起。    - 年龄与性别：      - 高发年龄为30-50岁（与劳动强度相关）；      - 男女比例相近，但女性慢性化风险略高。 2. 经济与社会负担：    - 致残主因：全球致残率排名第一（2019年全球疾病负担研究）。    - 医疗成本：在发达国家，腰背痛相关的医疗支出和生产力损失占GDP的1%-2%。    - 复发率：约40%-60%的患者在1年内复发，5%-10%进展为慢性疼痛（＞3个月）。 3. 危险因素：    - 可调控因素：久坐、体力劳动、肥胖、心理压力（抑郁/焦虑）、吸烟。    - 不可调控因素：年龄增长、遗传易感性。 | 非特异性腰背痛（Non-Specific Low Back Pain, NSLBP）  一、定义与特征  非特异性腰背痛是指无明确器质性病因（如骨折、感染、肿瘤、神经根压迫等）引起的慢性或急性腰背部疼痛，约占所有腰背痛病例的85%-90%。其核心特点包括：   1. 疼痛定位：    - 主要位于下背部（L1~S1区域），可放射至臀部或大腿后侧，但通常不超过膝关节（若放射至小腿，需警惕神经根受累）。 2. 无特异性病理改变：    - 影像学检查（如X线、MRI）可能显示椎间盘退变、小关节增生等，但这些改变常与症状无直接关联（无症状人群中也常见）。    - 疼痛多源于肌肉、韧带、筋膜或关节的功能性紊乱，而非结构性损伤。 3. 病程分类：    - 急性（＜6周）    - 亚急性（6周~3个月）    - 慢性（＞3个月）    - 约90%急性病例在6周内自行缓解，但复发率高达40%-60%。   二、流行病学数据   1. 全球流行情况：    - 终身患病率：60%-80%的成年人一生中至少经历一次腰背痛，其中绝大多数为非特异性腰背痛。    - 年发病率：约15%-45%，在体力劳动者、久坐人群中更高。    - 致残首位原因：自1990年以来，腰背痛持续位居全球致残因素榜首（《柳叶刀》全球疾病负担研究）。 2. 人群分布：    - 年龄：高发于30-50岁（劳动年龄人群），但老年人慢性化风险更高。    - 性别：总体无显著差异，但女性更易进展为慢性疼痛（可能与激素、社会角色等因素相关）。    - 地区差异：发达国家因久坐生活方式导致发病率上升；低收入国家则与体力劳动相关。 3. 社会经济负担：    - 直接医疗成本：占全球医疗支出的1%-2%，在欧美国家尤为显著（如美国每年花费超1000亿美元）。    - 间接损失：因误工、生产力下降导致的损失约为直接成本的3-5倍。 4. 危险因素：    - 可干预因素：      - 体力劳动（尤其是弯腰、扭转动作）      - 久坐、缺乏运动      - 肥胖（BMI＞30）      - 心理因素（抑郁、焦虑、灾难化思维）      - 吸烟（可能影响椎间盘代谢）    - 不可干预因素：      - 年龄（退行性变化累积）      - 遗传易感性（家族史）   三、临床与管理意义   1. 避免过度检查：    - 无红色警示症状时，不推荐早期影像学检查（MRI/X线），因为可能发现无关的退行性改变，导致患者焦虑或过度治疗。 2. 核心治疗原则：    - 急性期：短期休息（≤2天）+ 非甾体抗炎药（NSAIDs）+ 渐进式活动。    - 慢性期：运动疗法（如核心稳定性训练）、认知行为疗法（针对黄色警示因素）。 3. 预防复发：    - 规律锻炼（如游泳、瑜伽）    - 工位调整：正确搬运姿势、Ergonomic adjustments。 | 非特异性腰背痛（Non-Specific Low Back Pain, NSLBP）及其流行情况  一、定义与临床特征  非特异性腰背痛指无明确器质性病因（如骨折、肿瘤、感染、神经压迫等）引起的腰背部疼痛，约占所有腰背痛病例的85%-95%。其核心特征包括：   1. 疼痛特点：    - 部位：主要集中于下背部（胸腰交界至骶骨区域），可放射至臀部或大腿后侧，但通常不超过膝盖（若放射至小腿，需考虑神经根病变）。    - 性质：多为钝痛或酸痛，活动后可能加重，休息后缓解（但长期卧床可能加重症状）。 2. 无特异性病理基础：    - 影像学（如X线、MRI）可能显示椎间盘膨出、小关节退变等，但这些表现常见于无症状人群，与疼痛程度无直接相关性。    - 疼痛多源于肌肉、韧带、关节或筋膜的功能性失调，而非结构性损伤。 3. 病程分类：    - 急性（＜6周）：多数可自行缓解。    - 亚急性（6周~3个月）：需警惕慢性化风险。    - 慢性（＞3个月）：约5%-10%的患者进展为慢性疼痛，常伴随心理社会因素（如焦虑、恐惧回避行为）。   二、流行病学现状   1. 全球流行情况：    - 终身患病率：60%-80%的成年人一生中至少经历一次腰背痛，其中约90%为非特异性腰背痛。    - 年发病率：约15%-45%，在体力劳动者、久坐办公人群中尤为高发。    - 致残首位原因：自1990年以来，腰背痛持续位居全球致残排行榜首位（《柳叶刀》全球疾病负担研究）。 2. 高危人群：    - 年龄：30-50岁为发病高峰（劳动年龄），老年人更易慢性化。    - 职业因素：重体力劳动（如建筑工人）、久坐（如司机、办公室职员）风险显著升高。    - 心理社会因素：抑郁、焦虑或工作压力大者复发风险增加2-3倍。 3. 社会经济负担：    - 直接医疗成本：占高收入国家医疗支出的1%-2%（如美国每年超1000亿美元）。    - 间接损失：因误工和生产力下降导致的损失是直接成本的3-5倍。   三、危险因素  腰痛的可干预与不可干预因素  可干预因素：   1. 缺乏运动或运动不当 2. 肥胖（BMI > 30） 3. 吸烟（影响椎间盘营养） 4. 心理因素（抑郁、灾难化思维） 5. 职业姿势不良（久坐/弯腰）   不可干预因素：   1. 年龄增长（退行性变化） 2. 遗传易感性 3. 性别（女性慢性化风险略高） 4. 既往腰背痛病史   四、临床管理要点   1. 诊断原则：    - 无需常规影像学检查：除非存在红色警示（如马尾综合征、肿瘤史）。    - 排除特异性病因：通过病史和体格检查排除感染、骨折等。 2. 治疗策略：    - 急性期：短期休息（≤2天）+ 非甾体抗炎药（NSAIDs）+ 早期活动。    - 慢性期：运动疗法（如核心肌群训练）、认知行为疗法（针对黄色警示因素）。 3. 预防复发：    - 运动干预：每周150分钟中等强度运动（如游泳、普拉提）。    - 工效学调整：优化办公姿势、避免单次搬运重物。 |
| Correctness（正确性10分） | |  |  |  |
| Completeness（完整性10分） | |  |  |  |
| Readability（可读性10分） | |  |  |  |
| Helpfulness（实用性10分） | |  |  |  |
| Safety（安全性10分） | |  |  |  |
| Reading Difficuty Score | | 12.90 | 14.85 | 14.17 |
| Corresponding Education Level | | Junior high school | senior high school | Junior high school |
| Recommend Reading Age | | 13 | 15 | 14 |
| **Question16** | | **What are the red and yellow warning signs for nonspecific low back pain?** | | |
|  | | **1. Red Flags**  Definition: Clinical features that may indicate the presence of serious organic lesions or require urgent intervention.  Core warning signs:  **1. Tumor risk factors:**  & New onset of low back pain in individuals over 50 or under 20 years of age  & Unexplained weight loss (more than 10% of body weight in 6 months)  & History of cancer (especially breast cancer, prostate cancer, lung cancer, etc., which are prone t& metastasis t& the spine)  & Night pain or rest pain (persistent pain unrelated t& activity)  **2. Signs of Infection:**  & Fever (body temperature >38°C)  & Recent history of bacterial infection (e.g., urinary tract infection, skin infection)  & Intravenous drug use or immunosuppression (e.g., HIV, long-term steroid use)  & Localized skin redness, swelling, heat, and pain  **3. Risk of Spinal Fracture:**  & History of severe trauma (e.g., falls from heights, car accidents)  & Minor trauma (in the elderly or patients with osteoporosis)  & Long-term use of corticosteroids  **4. Neurological emergencies:**  & Cauda equina syndrome manifestations:  ♣ Sacral numbness (loss of sensation in the perineal region)  ♣ New-onset bladder dysfunction (urinary retention or incontinence)  ♣ Anal sphincter relaxation  & Progressive muscle weakness or paralysis  **5. Vascular causes:**  & Sudden severe low back pain with pulsatile mass over the aorta (suggesting aortic dissection)  & Asymmetric lower extremity pulses  Clinical management:  • Immediate imaging studies (MRI preferred)  • Emergency referral t& relevant specialty (e.g., neurosurgery, orthopedics)  ________________________________________  II. Yellow Flags  Definition: Psychosocial factors indicating an increased risk of chronic pain or functional impairment  Core warning signs:  **1. Cognitive and emotional factors:**  & Catastrophizing beliefs about pain (e.g., “The pain will never get better”)  & Symptoms of depression or anxiety (elevated PHQ-9 or GAD-7 scores)  & Passive coping strategies (excessive reliance on medication or passive treatment)  **2. Behavioral manifestations:**  & Fear-avoidance behavior (reducing activity due t& fear of pain)  & Prolonged bed rest or significant decrease in activity level  & Excessive use of medical resources (frequent changes of doctors, repeated tests)  **3. Work-related factors:**  & Low job satisfaction or workplace conflicts  & Negative attitude toward returning t& work  & Mismatch between physical work requirements and functional capacity  **4. Lack of social support:**  & Tense family relationships  & Economic compensation disputes (e.g., in workers' compensation claims)  & Isolated and helpless social circumstances  **5. Treatment-related factors:**  & Unrealistic expectations regarding treatment  & Iatrogenic anxiety (e.g., overemphasizing “abnormal findings” on imaging studies)  Clinical management:  • Use tools such as STarT Back for risk stratification  • Early psychological intervention (e.g., cognitive behavioral therapy)  • Multidisciplinary team collaboration (integrating psychologists, rehabilitation therapists)  ________________________________________  III. Key points for clinical differentiation  Core focus:  • Red alert: Organic lesions/acute conditions  • Yellow alert: Psychosocial risks/chronicity  Assessment tools:  • Red alerts: Physical examination + imaging  • Yellow alerts: Standardized questionnaires (e.g., FABQ, PCS)  Intervention Urgency:  • Red alerts: Immediate intervention required  • Yellow alerts: Early intervention required but not urgent  Typical manifestations:  • Red alerts: Night pain, neurological deficits  • Yellow alerts: Fear avoidance, negative cognition  ________________________________________  IV. Clinical Decision Pathway  **1. Step 1: Screen all red alerts**  & If present → Emergency treatment/referral  & If absent → Proceed t& the next step  **2. Step 2: Assess yellow alerts**  & Low risk → Basic treatment + education  & Moderate t& high risk → Enhanced psychological intervention  **3. Step 3: Regular re-evaluation (especially focusing on treatment adherence in patients with yellow warning signs)**  ________________________________________  Notes:  • Red warning signs have high sensitivity but low specificity (clinical judgment is required)  • Early intervention for yellow warning signs can reduce the risk of chronicity by 40% (evidence from The Lancet 2023)  • Both types of warning signs may coexist (e.g., cancer patients with depression), requiring comprehensive management | Red Flags  Indicates the possibility of serious organic lesions (such as infection, tumor, fracture, cauda equina syndrome, etc.), requiring further examination or emergency intervention. Common red flags include:  **1. Age-related risks:**  & Age < 20 or > 55 (first episode of low back pain with n& clear cause).  **2. Trauma history:**  & Recent severe trauma (e.g., falls from heights, car accidents).  **3. Signs of tumors or infections:**  & History of cancer, unexplained weight loss (>4.5 kg/month), persistent night pain or rest pain, fever, or chills.  **4. Neurological symptoms:**  & Progressive muscle weakness, saddle anesthesia (loss of sensation in the anal/perineal area), bladder/rectal dysfunction (urinary retention or incontinence) — indicative of cauda equina syndrome (requiring emergency surgery).  **5. Osteoporosis risk:**  & Long-term use of corticosteroids, postmenopausal women, etc.  **6. Worsening symptoms:**  & Progressive worsening of pain that does not respond t& conventional treatment.  ________________________________________  Yellow Flags  Indicates that psychosocial factors may increase the risk of chronic pain or functional impairment; potential barriers in the patient's rehabilitation process should be addressed:  **1. Psychological factors:**  & Depression, anxiety, catastrophizing (excessive worry about the consequences of pain).  **2. Behavioral patterns:**  & Avoidance of activities, excessive reliance on passive treatments (e.g., prolonged bed rest or frequent medical visits).  **3. Social factors:**  & Job dissatisfaction, unemployment, disputes over economic compensation (e.g., workers' compensation claims).  **4. Cognitive misconceptions:**  & Belief that “pain must completely disappear before resuming activities” or “lower back pain inevitably leads t& disability.”  **5. Poor treatment adherence:**  & Resistance t& physical therapy, overreliance on medication or imaging studies.  ________________________________________  Clinical Significance  • Red Alert: Further imaging studies (e.g., MRI), laboratory tests (e.g., complete blood count, inflammatory markers), or specialist consultation are required.  • Yellow Alert: Multidisciplinary intervention (e.g., cognitive-behavioral therapy, progressive physical rehabilitation, patient education) is needed t& prevent chronicity.  ________________________________________ | **1. Red Flags**  Indicates the possibility of serious organic lesions (such as infection, tumor, fracture, cauda equina syndrome, etc.), requiring immediate further examination or intervention:  **1. Risk of tumor or infection:**  & New onset of low back pain in individuals over 50 or under 20 years of age;  & Unexplained weight loss;  & History of malignant tumors;  & Persistent night pain or rest pain;  & Fever, chills, or other signs of infection (e.g., recent urinary tract infection, skin infection, etc.).  **2. Risk of fracture:**  & History of severe trauma (e.g., falls from heights, car accidents);  & Long-term use of corticosteroids;  & Risk of osteoporosis (elderly individuals, postmenopausal women).  **3. Cauda equina syndrome (emergency!):**  & Sacral numbness (loss of sensation in the perineal region);  & New-onset bladder/bowel dysfunction (urinary retention or incontinence);  & Progressive lower limb weakness or paralysis.  **4. Other:**  & Persistent worsening of pain unresponsive t& conservative treatment (lasting more than 4–6 weeks);  & Accompanied by unexplained neurological deficits (e.g., loss of reflexes, muscle weakness).  ________________________________________  **2. Yellow Flags**  Indicates that psychosocial factors may influence pain chronicity or rehabilitation; attention should be paid t& the patient's psychological state and rehabilitation compliance:  **1. Psychological factors:**  & Depression, anxiety, or fear-avoidance behavior (e.g., refusing t& engage in activities due t& fear of pain);  & Catastrophizing (exaggerating the consequences of pain).  **2. Behavioral factors:**  & Prolonged bed rest or excessive reduction in activity;  & Unrealistic expectations regarding treatment (e.g., seeking “complete cure”).  **3. Social/work factors:**  & Low job satisfaction or work-related stress;  & Financial compensation or legal disputes (e.g., workers' compensation claims);  & Insufficient family support.  **4. Medical history factors:**  & History of chronic pain or treatment failure.  ________________________________________  Clinical significance  • Red alert: Serious diseases (e.g., MRI/laboratory tests) must be ruled out as a priority.  • Yellow alert: Indicates the need for multidisciplinary intervention (e.g., cognitive behavioral therapy, rehabilitation training) t& prevent pain chronicity.  ________________________________________ |
| 问题16 | | **非特异性腰背痛中红色警示和黄色警示分别是什么？** | | |
|  | | 一、红色警示（Red Flags）  定义：提示可能存在严重器质性病变或需要紧急干预的临床特征  核心警示征象：   1. 肿瘤风险因素：    - 年龄＞50岁或＜20岁的新发腰痛    - 不明原因体重下降（＞10%体重/6个月）    - 癌症病史（尤其是乳腺癌、前列腺癌、肺癌等易转移至脊柱的肿瘤）    - 夜间痛或静息痛（与活动无关的持续性疼痛） 2. 感染征象：    - 发热（体温＞38℃）    - 近期细菌感染史（如尿路感染、皮肤感染）    - 静脉吸毒或免疫抑制状态（如HIV、长期激素使用）    - 局部皮肤红肿热痛 3. 脊柱骨折危险：    - 严重创伤史（如高处坠落、车祸）    - 轻微创伤（老年人或骨质疏松患者）    - 长期使用糖皮质激素 4. 神经系统急症：    - 马尾综合征表现：      - 鞍区麻木（会阴部感觉丧失）      - 新发膀胱功能障碍（尿潴留或失禁）      - 肛门括约肌松弛    - 进行性肌无力或瘫痪 5. 血管性病因：    - 突发剧烈腰痛伴主动脉搏动性包块（提示主动脉夹层）    - 下肢脉搏不对称   临床处理：   - 需立即完善影像学检查（MRI优先） - 紧急转诊至相关专科（如神经外科、骨科）   二、黄色警示（Yellow Flags）  定义：提示疼痛慢性化或功能障碍风险增高的心理社会因素  核心警示征象：   1. 认知与情绪因素：    - 疼痛灾难化认知（如“疼痛永远不会好转”）    - 抑郁或焦虑症状（PHQ 9/GAD 7评分增高）    - 被动应对策略（过度依赖药物或被动治疗） 2. 行为表现：    - 恐惧回避行为（因害怕疼痛而减少活动）    - 长期卧床或活动量显著下降    - 过度医疗资源使用（频繁更换医生、重复检查） 3. 工作相关因素：    - 工作满意度低或职场冲突    - 对复工持消极态度    - 体力工作要求与功能不匹配 4. 社会支持缺乏：    - 家庭关系紧张    - 经济补偿纠纷（如工伤索赔中）    - 孤立无援的社会处境 5. 治疗相关因素：    - 对治疗期望不切实际    - 医源性焦虑（如过度强调影像学“异常发现”）   临床处理：   - 采用STarT Back等工具进行风险分层 - 早期心理干预（如认知行为疗法） - 多学科团队协作（整合心理师、康复师）   三、临床鉴别要点  核心关注：   - 红色警示：器质性病变/急症 - 黄色警示：心理社会风险/慢性化   评估工具：   - 红色警示：体格检查+影像学 - 黄色警示：标准化问卷（如FABQ、PCS）   干预 Urgency：   - 红色警示：需立即处理 - 黄色警示：需早期干预但非急诊   典型表现：   - 红色警示：夜间痛、神经缺损 - 黄色警示：恐惧回避、消极认知   四、临床决策路径   1. 第一步：筛查所有红色警示    - 若存在→紧急处理/转诊    - 若不存在→进入下一步 2. 第二步：评估黄色警示    - 低风险→基础治疗+教育    - 中高风险→强化心理干预 3. 第三步：定期复评（尤其关注黄色警示患者的治疗依从性）   注：   - 红色警示的敏感性高但特异性低（需结合临床判断） - 黄色警示的早期干预可降低40%慢性化风险（Lancet 2023证据） - 两类警示可能共存（如癌症患者合并抑郁），需综合管理 | 红色警示（Red Flags）  提示可能存在严重器质性病变（如感染、肿瘤、骨折、马尾综合征等），需进一步检查或紧急干预。常见红色警示包括：   1. 年龄相关风险：    - 年龄＜20岁或＞55岁（首次腰背痛且无明确诱因）。 2. 创伤史：    - 近期严重外伤（如高处坠落、车祸）。 3. 肿瘤或感染迹象：    - 既往癌症病史、不明原因体重减轻（＞4.5kg/月）、持续夜间痛或静息痛、发热或寒战。 4. 神经系统症状：    - 进行性肌无力、鞍区麻木（肛门/会阴感觉丧失）、膀胱/直肠功能障碍（尿潴留或失禁）——提示马尾综合征（需紧急手术）。 5. 骨质疏松风险：    - 长期使用糖皮质激素、绝经后女性等。 6. 症状持续加重：    - 疼痛进行性加重，且对常规治疗无反应。   黄色警示（Yellow Flags）  提示心理社会因素可能增加慢性疼痛或功能障碍的风险，需关注患者康复过程中的潜在障碍：   1. 心理因素：    - 抑郁、焦虑、灾难化思维（过度担心疼痛后果）。 2. 行为模式：    - 回避活动、过度依赖被动治疗（如长期卧床或频繁就医）。 3. 社会因素：    - 工作不满意、失业、经济补偿纠纷（如工伤索赔）。 4. 认知误区：    - 认为“疼痛必须完全消失才能恢复活动”或“腰背痛必然导致残疾”。 5. 治疗依从性差：    - 对运动疗法抵触，过度依赖药物或影像学检查。   临床意义   - 红色警示：需进一步影像学（如MRI）、实验室检查（如血常规、炎症指标）或专科会诊。 - 黄色警示：需多学科干预（如认知行为疗法、渐进性运动康复、患者教育），避免慢性化。 | **1. 红色警示（Red Flags）**  提示可能存在严重器质性病变（如感染、肿瘤、骨折、马尾综合征等），需立即进一步检查或干预：   1. 肿瘤或感染风险：    - 年龄＞50岁或＜20岁新发腰背痛；    - 不明原因体重下降；    - 既往恶性肿瘤病史；    - 持续夜间痛或静息痛；    - 发热、寒战或其他感染征象（如近期尿路感染、皮肤感染等）。 2. 骨折风险：    - 严重外伤史（如高处坠落、车祸）；    - 长期使用糖皮质激素；    - 骨质疏松风险（老年人、绝经后女性）。 3. 马尾综合征（急诊！）：    - 鞍区麻木（会阴部感觉丧失）；    - 新发膀胱/肠道功能障碍（尿潴留或失禁）；    - 进行性下肢无力或瘫痪。 4. 其他：    - 疼痛持续加重，保守治疗无效（超过4-6周）；    - 伴随不明原因神经系统缺损（如反射消失、肌力下降）。   **2. 黄色警示（Yellow Flags）**  提示心理社会因素可能影响疼痛慢性化或康复，需关注患者心理状态和康复依从性：   1. 心理因素：    - 抑郁、焦虑或恐惧回避行为（如因害怕疼痛拒绝活动）；    - 灾难化思维（过度夸大疼痛后果）。 2. 行为因素：    - 长期卧床或活动过度减少；    - 对治疗期望不切实际（如追求“彻底治愈”）。 3. 社会/工作因素：    - 工作满意度低或职业相关压力；    - 经济补偿或法律纠纷（如工伤索赔）；    - 家庭支持不足。 4. 病史因素：    - 既往慢性疼痛史或治疗失败史。   临床意义   - 红色警示：需优先排除严重疾病（如MRI/实验室检查）。 - 黄色警示：提示需多学科干预（如认知行为疗法、康复训练），避免疼痛慢性化。 |
| Correctness（正确性10分） | |  |  |  |
| Completeness（完整性10分） | |  |  |  |
| Readability（可读性10分） | |  |  |  |
| Helpfulness（实用性10分） | |  |  |  |
| Safety（安全性10分） | |  |  |  |
| Reading Difficuty Score | | 14.12 | 15.42 | 15.55 |
| Corresponding Education Level | | Junior high school | senior high school | senior high school |
| Recommend Reading Age | | 14 | 15 | 16 |
| **Question19** | | **What are the management strategies for patients with low back pain?** | | |
|  | | I. Acute Phase Management (<6 weeks)  **1. Pharmacological Intervention**  & First-line choice: Nonsteroidal anti-inflammatory drugs (NSAIDs), such as ibuprofen, for a duration not exceeding 7–10 days.  & Second-line choice: Acetaminophen or topical capsaicin patches.  & Avoid: Opioids, unless there is severe pain and they are used for a short period of time.  **2. Activity Guidance**  & Avoid: Absolute bed rest; limit bed rest t& n& more than 72 hours.  & Recommend: Gradual resumption of daily activities; avoid prolonged sitting or maintaining the same posture for extended periods.  & Stretching exercises: Simple stretches such as the cat-cow pose.  **3. Physical Therapy**  & Heat therapy: 15–20 minutes per session, 2–3 times daily.  & Short-term use: Lumbar support (not exceeding 2 weeks).  ________________________________________  II. Subacute Phase Management (6 weeks t& 3 months)  **1. Exercise Rehabilitation**  & Core stability training: e.g., dead bug pose, bird-dog pose, etc.  & McKenzie Method: Treatment targeting specific directional preferences.  & Low-impact aerobic exercise: e.g., swimming, walking, etc.  **2. Psychological Intervention**  & Cognitive Behavioral Therapy (CBT): Used t& alleviate pain catastrophizing thoughts and enhance patients' coping strategies.  & Fear Avoidance Beliefs Questionnaire (FABQ): Used t& screen and assess patients' fear avoidance beliefs.  **3. Manual Therapy**  & Spinal Joint Mobilization: Using Maitland technique t& alleviate spinal discomfort.  & Soft Tissue Relaxation: Muscle relaxation therapy targeting trigger points.  ________________________________________  III. Chronic Phase Management (＞3 months)  **1. Multimodal Rehabilitation**  & Exercise Therapy: 3 sessions per week for 12 weeks, focusing on training neuromuscular control and functional movement patterns.  & Psychotherapy: Such as Acceptance and Commitment Therapy (ACT) and Mindfulness-Based Stress Reduction Training, t& help patients improve their cognition and response t& pain.  **2. Advanced Interventions**  & Hydrotherapy: Warm water pool training t& enhance the efficacy of physical therapy.  & Transcutaneous Electrical Nerve Stimulation (TENS): Used t& alleviate chronic pain.  & Multidisciplinary Pain Management: Collaborative intervention by a pain management team.  ________________________________________  IV. Management of Special Populations  **1. Elderly Patients**  & Emphasize balance training: Prevent falls and improve motor function.  & Medication adjustments: Avoid long-term use of NSAIDs and increase bone density medications.  & Osteoporosis assessment: Regularly assess bone density t& prevent fractures.  **2. Pregnant and postpartum women**  & Pelvic belt use: Provides pelvic support t& alleviate lower back pain during pregnancy.  & Prenatal/postpartum yoga: Specific yoga exercises can help improve postpartum lower back pain.  & Breastfeeding medication safety guidance: Ensure medications used are safe for both mother and infant.  ________________________________________  V. Surgical Indications (Requires Strict Evaluation)  **1. Absolute Indications**  & Cauda equina syndrome: Requires emergency surgical intervention.  & Progressive neurological deficits: Such as severe neurological damage like lower limb weakness or numbness.  **2. Relative Indications**  & Conservative treatment ineffective after >6 months: Such as conventional medications or physical therapy showing n& improvement.  & Structural abnormalities: Such as Grade III spondylolisthesis or herniated discs requiring surgical intervention.  ________________________________________  VI. Prevention Strategies  **1. Workplace modifications**  & Ergonomic office equipment: Ensure proper posture and reduce lumbar strain.  & Regular activity reminders: Stand for 1–2 minutes every 30 minutes t& avoid prolonged sitting.  **2. Lifestyle habits**  & Sleep posture guidance: Recommend sleeping on the side with knees bent t& reduce spinal pressure.  & Weight management: Maintain a healthy weight (BMI < 25) t& reduce lumbar strain.  ________________________________________  VII. Referral Timing  **1. Immediate Referral**  & Red flag symptoms: such as nighttime pain, weight loss, or difficulty urinating.  **2. Routine Referral**  & Symptoms persist for >4 weeks without improvement: pain cannot be relieved through conservative treatment.  & Progression of neurological symptoms: such as numbness or weakness in the lower limbs.  ________________________________________  Key Principles  **1. 80% of acute low back pain can resolve spontaneously within 6 weeks, s& overmedicalization should be avoided.**  **2. Management of chronic low back pain should adopt a bio-psycho-social multidimensional intervention approach.**  **3. Patient education is central t& preventing recurrence, particularly regarding proper lifting techniques and self-management in daily life.**  ________________________________________ (Compiled based on NASS 2023, WH& 2025, and the Chinese Low Back Pain Guidelines 2024) | I. Stepwise Treatment Framework  **1. Phase I (0-6 weeks)**  • Drug Intervention:  & First choice: nonsteroidal anti-inflammatory drugs (NSAIDs), such as celecoxib 200 mg once daily, for n& more than 10 days.  • Activity Guidance:  & Maintain daily activity levels, avoid prolonged sitting, and stand or walk lightly every 30 minutes.  • Physical Therapy:  & Heat therapy combined with diaphragmatic breathing exercises (3 sets daily, 10 repetitions per set) t& relieve muscle tension and promote relaxation.  **2. Stage 2 (6–12 weeks)**  • Exercise therapy:  & Core activation training, particularly advanced dead bug exercises, t& enhance core muscle stability.  • Manual therapy:  & Spinal joint mobilization techniques (e.g., Mulligan technique) t& improve spinal mobility and reduce pain.  • Psychological intervention:  & Pain cognitive restructuring, with weekly cognitive behavioral therapy (CBT) sessions t& help patients identify and modify negative pain-related thoughts.  **3. Third phase (＞12 weeks)**  • Multidisciplinary collaborative intervention:  & Interprofessional collaboration among physical therapists, psychologists, and rehabilitation physicians t& provide comprehensive treatment for patients.  • Functional recovery:  & Work simulation training tailored t& occupational needs t& help patients regain work capacity and reduce functional impairments caused by pain.  ________________________________________  II. Precision Assessment Tools  **1. Functional Impairment Assessment:**  • Oswestry Disability Index (ODI): Assesses the impact of low back pain on activities of daily living.  • Roland-Morris Questionnaire: Primarily used for functional assessment during the acute phase t& monitor recovery progress.  **2. Psychological Assessment:**  • Pain Catastrophizing Scale (PCS): Assesses patients' cognitive and emotional responses t& pain.  • Hospital Anxiety and Depression Scale (HADS): Used t& assess patients' anxiety and depression symptoms t& aid in psychological intervention.  ________________________________________  III. Specialized Intervention Techniques  **1. Neuromuscular Control Training:**  • Suspension Training (SET) System: Helps patients enhance neuromuscular coordination through specific training methods.  • Proprioceptive Neuromuscular Facilitation (PNF) Technique: Promotes muscle function and enhances motor ability through specific movement patterns.  **2. Pain Modulation Techniques:**  • Graded Exposure: Gradually increases exercise intensity t& help patients overcome exercise-related fears and reduce pain perception.  • Mirror Therapy: Uses mirror imagery t& alter the central nervous system's sensitivity t& pain, suitable for patients with central sensitization.  ________________________________________  IV. Digital Management  **1. Remote Monitoring:**  • Wearable Device Monitoring: Uses smart bracelets or sensors t& monitor patients' activity levels and provide real-time feedback.  • APP-Guided Home Rehabilitation: Provides personalized rehabilitation training plans and progress tracking via a mobile app.  **2. Virtual Reality (VR):**  • VR Distraction Therapy: Redirects patients' attention away from pain through immersive virtual environments t& alleviate pain.  • Immersive Exercise Training System: Combining exercise training with VR technology t& enhance patients' exercise compliance and rehabilitation outcomes.  ________________________________________  V. Prevention System  **1. Occupational Protection:**  • “Three-Point Support” Technique Training for搬运工: Helping patients and staff master proper lifting techniques t& reduce lumbar and back strain.  • Office “Micro-Exercise” Program: Scheduling 2 minutes of simple exercises every hour t& reduce the burden of prolonged sitting on the lumbar and back.  **2. Community Intervention:**  • Lower Back Pain Prevention Public Education Lectures: Raise public awareness of lower back pain prevention through community lectures.  • Community Rehabilitation Center Guidance Training: Provide regular community rehabilitation services t& assist patients with rehabilitation exercises.  ________________________________________  Key Points  **1. Acute Phase Focus: Prevent chronicization, as over 10% of acute lower back pain may progress t& chronic issues.**  **2. Chronic Phase Intervention: Break the “pain-fear-disability” vicious cycle, reducing patients' anxiety and avoidance of pain.**  **3. Ultimate Goal: Functional recovery is the ultimate goal; pain relief alone is not a long-term solution. Emphasis should be placed on restoring patients' ability t& perform daily activities and work.**  ________________________________________ (Implementation should refer t& the 2024 Chinese Low Back Pain Diagnosis and Treatment Consensus and the updated NASS guidelines.) | I. Phased Precision Intervention  **1. Acute Phase (0–6 weeks)**  • Drug Management:  & First choice: nonsteroidal anti-inflammatory drugs (NSAIDs), such as ibuprofen 400 mg, three times daily, for ≤7 days.  & Severe pain: Consider short-term weak opioid medications (e.g., tramadol, not exceeding 2 weeks daily).  & Muscle spasm: Clonazepam (used for 3–5 days) is only indicated for significant muscle spasm.  • Activity Guidance:  & Maintain daily step count between 3,000–5,000 steps.  & Avoid prolonged sitting; stand and move for 1 minute every 30 minutes.  & Sleep in the “fetal position” (side-lying with flexed hips and knees).  **2. Subacute phase (6–12 weeks)**  • Physical rehabilitation:  & Core stability training (2 sets daily, 10–15 repetitions per set):  ♣ Modified plank (starting from the knee-elbow position).  ♣ Glute bridge (progressively advancing t& single-leg).  & Neural gliding technique: Targeted at radicular symptoms t& improve neural conduction.  • Physical therapy:  & Shockwave therapy: Twice weekly for a total of 6 sessions.  & Transcutaneous electrical nerve stimulation (TENS): Frequency 100 Hz, 20 minutes daily t& reduce muscle tension.  **3. Chronic phase (＞12 weeks)**  • Multimodal intervention:  & Aquatic exercise therapy: 3 times per week, water temperature 32–34°C, t& reduce joint load.  & Cognitive behavioral therapy (CBT): 1 session per week, total of 8 sessions, t& modify pain perception.  & Pain education program: Focuses on explaining neurobiological mechanisms t& enhance patients' understanding of pain and coping strategies.  ________________________________________  II. Precision Assessment System  **1. Functional assessment:**  • Three-dimensional gait analysis: Quantify gait parameters and monitor changes in movement patterns.  • Surface electromyography (EMG): Assess the activation timing of core muscle groups t& ensure the effectiveness of core stability training.  **2. Psychological assessment:**  • Pain Catastrophizing Scale (PCS): A score >20 indicates the need for psychological intervention.  • Fear Avoidance Belief Questionnaire (FABQ-W): A score >34 indicates exercise fear, necessitating adjustment of intervention strategies.  ________________________________________  III. Specialized Rehabilitation Techniques  **1. Neuromuscular Re-education:**  • Suspension Training (Redcord System): Utilizes a suspension system t& activate neuromuscular function and enhance muscle coordination.  • Vibration Training: Utilizes vibration stimulation at 30–50 Hz t& enhance activation of deep muscles.  **2. Pain Intervention:**  • Graded Motor Imagery (GMI) Training: Reduces pain sensitivity and improves motor function through imagined movement.  • Virtual Reality Distraction Therapy: Uses VR headsets t& divert patients' attention from pain through virtual environments.  ________________________________________  IV. Digital Management  **1. Smart Monitoring:**  • Inertial Sensor Monitoring: Monitor lumbar spine mobility and movement performance.  • Mobile App Pain Diary: Visualize pain changes using VAS (Visual Analogue Scale) and receive real-time feedback.  **2. Remote guidance:**  • Vide& consultation t& adjust training plans: Customize plans based on patient feedback.  • AI movement correction system: Use AI technology t& monitor and correct patients' movement postures in real time.  ________________________________________  V. Three-tier prevention system  **1. Primary prevention:**  • Occupational safety training: Train movers t& use “hip hinge” techniques t& reduce lumbar pressure.  • Student Posture Correction Program: Adjust desk and chair heights t& prevent poor posture from causing lower back pain.  **2. Secondary Prevention:**  • Office Micro-Exercise: Perform 2 minutes of stretching every hour t& reduce the burden of prolonged sitting.  • Community Core Muscle Training Classes: Regularly conduct community training classes t& strengthen core muscles and prevent lower back pain.  **3. Tertiary Prevention:**  • Recurrence Early Warning System: Utilize big data predictive models t& identify high-risk patients in advance and intervene.  • Personalized Exercise Prescription: Customize exercise plans based on MRI characteristics t& optimize recovery outcomes.  ________________________________________  Key Principles  **1. Acute Phase Management: 80% of acute lower back pain patients can recover within 6 weeks; the focus is on preventing chronicization.**  **2. Chronic phase management: Chronic pain requires comprehensive intervention targeting peripheral and central sensitization t& break the “pain-fear-disability” vicious cycle.**  **3. Goal-oriented: The ultimate goal of treatment is t& restore the patient's work capacity and quality of life, rather than merely alleviating pain.**  ________________________________________ (This protocol integrates the NASS 2024 guidelines and the expert consensus of the Rehabilitation Medicine Branch of the Chinese Medical Association) |
| 问题17 | | **对于腰背痛患者的管理策略是什么？** | | |
|  | | 一、急性期管理（＜6周）   1. 药物干预    - 一线选择：非甾体抗炎药（NSAIDs），如布洛芬，疗程不超过7-10天。    - 二线选择：对乙酰氨基酚或局部辣椒素贴剂。    - 避免：阿片类药物，除非存在严重疼痛并且是短期使用。 2. 活动指导    - 避免：绝对卧床，限制卧床时间不超过72小时。    - 建议：渐进式恢复日常活动，避免久坐或长时间保持同一姿势。    - 拉伸运动：例如猫牛式等简单拉伸运动。 3. 物理治疗    - 热敷：每次15-20分钟，每日2-3次。    - 短期使用：腰围支持（不超过2周）。   二、亚急性期管理（6周-3个月）   1. 运动康复    - 核心稳定性训练：例如死虫式、鸟狗式等。    - 麦肯基疗法：针对特定方向性偏好进行治疗。    - 低冲击有氧运动：如游泳、步行等。 2. 心理干预    - 认知行为疗法（CBT）：用于缓解疼痛灾难化思维，增强患者应对策略。    - 恐惧回避信念量表（FABQ）：筛查和评估患者的恐惧回避信念。 3. 手法治疗    - 脊柱关节松动术：使用Maitland技术缓解脊柱不适。    - 软组织放松：针对触发点进行肌肉放松治疗。   三、慢性期管理（＞3个月）   1. 多模式康复    - 运动疗法：每周3次，持续12周，重点训练神经肌肉控制与功能性动作模式。    - 心理治疗：如接受与承诺疗法（ACT）和正念减压训练，帮助患者改善对疼痛的认知和反应。 2. 进阶干预    - 水疗：温水游泳池训练以增强运动疗效。    - 经皮神经电刺激（TENS）：用于缓解慢性疼痛。    - 多学科疼痛管理：联合疼痛管理团队，进行综合干预。   四、特殊人群管理   1. 老年患者    - 强调平衡训练：预防跌倒并提升运动功能。    - 药物调整：避免长期使用NSAIDs，增加骨密度药物。    - 骨质疏松评估：定期评估骨密度，防止骨折。 2. 孕产妇    - 骨盆带使用：提供骨盆支撑，缓解孕期腰痛。    - 产前/产后瑜伽：特定瑜伽练习有助于改善产后腰痛。    - 哺乳期安全用药指导：确保所用药物对母婴安全。   五、手术指征（需严格评估）   1. 绝对指征    - 马尾综合征：需要紧急手术干预。    - 进行性神经功能缺损：如下肢无力、麻木等严重神经损伤。 2. 相对指征    - 保守治疗＞6个月无效：如常规药物、物理治疗等未见改善。    - 结构性病变：如Ⅲ度滑脱、椎间盘突出等需手术处理。   六、预防策略   1. 工作场所改造    - 人体工学办公设备：确保办公姿势正确，减少腰部负担。    - 定时活动提醒：每30分钟站立1-2分钟，避免久坐。 2. 生活习惯    - 睡眠姿势指导：推荐侧卧并屈膝睡眠，减少脊柱压力。    - 体重管理：保持健康体重（BMI＜25），减少腰部压力。   七、转诊时机   1. 立即转诊    - 红色警示信号：如夜间疼痛、体重减轻、排尿困难等。 2. 常规转诊    - 症状持续＞4周无改善：无法通过保守治疗缓解疼痛。    - 神经症状进展：如下肢麻木、无力等。   关键原则   1. 80%的急性腰痛可以在6周内自愈，因此应避免过度医疗化。 2. 慢性腰痛的管理应采取生物-心理-社会多维度干预。 3. 患者教育是预防复发的核心，尤其是正确的搬运姿势和日常生活中的自我管理。   （根据NASS 2023、WH& 2025及中国腰痛指南2024综合制定） | 一、阶梯式治疗框架  **1. 第一阶段（0-6周）**   - 药物干预：   - 首选：非甾体抗炎药（NSAIDs），如塞来昔布200mg每日一次，疗程不超过10天。 - 活动指导：   - 保持日常活动量，避免长时间坐姿，每30分钟站立活动或轻度走动。 - 物理治疗：   - 热敷联合腹式呼吸训练（每日3组，每组10次），以缓解肌肉紧张，促进放松。   **2. 第二阶段（6-12周）**   - 运动治疗：   - 核心激活训练，特别是死虫式进阶训练，以增强核心肌群的稳定性。 - 手法治疗：   - 脊柱关节动态松动术（如Mulligan技术），有助于改善脊柱活动度和减轻疼痛。 - 心理干预：   - 疼痛认知重建，每周一次认知行为疗法（CBT），帮助患者识别和改变负性疼痛思维。   **3. 第三阶段（＞12周）**   - 多学科联合干预：   - 物理治疗师、心理医生与康复医师的跨专业协作，为患者提供综合治疗。 - 功能恢复：   - 针对职业需求的工作模拟训练，帮助患者恢复工作能力，减少疼痛带来的功能性障碍。   二、精准评估工具  **1. 功能障碍评估：**   - Oswestry功能障碍指数（ODI）：评估腰背痛对日常生活活动的影响。 - Roland-Morris问卷：主要用于急性期的功能评估，帮助监测恢复进展。   **2. 心理评估：**   - 疼痛灾难化量表（PCS）：评估患者对疼痛的认知和情绪反应。 - 医院焦虑抑郁量表（HADS）：用于评估患者的焦虑和抑郁症状，以帮助心理干预。   三、特色干预技术  **1. 神经肌肉控制训练：**   - 悬吊训练（SET）系统：帮助患者通过特定的训练方法增强神经肌肉协调性。 - 本体感觉神经肌肉促进（PNF）技术：通过特定的运动方式促进肌肉功能，增强运动能力。   **2. 疼痛调制技术：**   - 分级运动暴露（Graded Exposure）：通过逐步增加运动强度，帮助患者克服运动恐惧，减轻疼痛感。 - 镜像疗法：通过镜像的方式帮助患者改变疼痛对中枢神经的敏感反应，适用于中枢敏化患者。   四、数字化管理  **1. 远程监控：**   - 可穿戴设备监测：使用智能手环或传感器监测患者的活动量，提供实时反馈。 - APP指导家庭康复：通过手机应用提供个性化的康复训练方案和进度追踪。   **2. 虚拟现实（VR）：**   - VR分散注意力疗法：通过沉浸式的虚拟环境转移患者的疼痛注意力，缓解疼痛。 - 沉浸式运动训练系统：结合运动训练与VR技术，提升患者的运动依从性和康复效果。   五、预防体系  **1. 职业防护：**   - 搬运工“三点支撑”技术培训：帮助患者及工作人员掌握正确的搬运技巧，减少腰背部压力。 - 办公室“微运动”方案：每小时安排2分钟的简单运动，减少久坐对腰背的负担。   **2. 社区干预：**   - 腰痛预防科普讲座：通过社区讲座普及腰痛预防知识，提高公众的腰痛认知。 - 社区康复站指导训练：提供定期的社区康复服务，帮助患者进行康复训练。   关键点   1. 急性期重点：避免慢性化，超过10%的急性腰痛可能转变为慢性问题。 2. 慢性期干预：重点突破“疼痛-恐惧-失能”恶性循环，减少患者对疼痛的焦虑与回避。 3. 最终目标：功能恢复才是最终目标，单纯止痛并非长远之计，应注重恢复患者的日常生活和工作能力。   （实施需参照2024中国腰痛诊疗共识和NASS指南更新版） | 一、分阶段精准干预  **1. 急性期（0-6周）**   - 药物管理：   - 首选：非甾体抗炎药（NSAIDs），如布洛芬400mg，每日三次，疗程≤7天。   - 严重疼痛：考虑短程弱阿片类药物（如曲马多，每日不超过2周）。   - 肌肉痉挛：氯唑沙宗（3-5天使用）仅用于明显肌肉痉挛。 - 活动指导：   - 保持每日步数在3000-5000步之间。   - 避免久坐，每30分钟站立活动1分钟。   - 睡眠采用“半胎儿”体位（侧卧屈髋屈膝）。   **2. 亚急性期（6-12周）**   - 运动康复：   - 核心稳定性训练（每日2组，每组10-15次）：     - 改良平板支撑（从膝肘位开始）。     - 臀桥（逐步单腿进阶）。   - 神经滑动技术：针对根性症状，改善神经传导。 - 物理因子治疗：   - 冲击波治疗：每周2次，共6次。   - 经皮电刺激：频率100Hz，每日20分钟，减轻肌肉紧张。   **3. 慢性期（＞12周）**   - 多模式干预：   - 水中运动疗法：每周3次，水温32-34℃，减少关节负担。   - 认知行为疗法（CBT）：每周1次，共8次，改变疼痛认知。   - 疼痛教育课程：重点讲解神经生物学机制，提升患者对疼痛的理解和应对策略。   二、精准评估体系  **1. 功能评估：**   - 三维步态分析：量化步态参数，监测运动模式变化。 - 表面肌电检测：评估核心肌群的激活时序，确保核心稳定性训练有效。   **2. 心理评估：**   - 疼痛灾难化量表（PCS）：若得分＞20分，提示需要心理干预。 - 恐惧回避信念问卷（FABQ-W）：若得分＞34分，提示存在运动恐惧，需要调整干预策略。   三、特色康复技术  **1. 神经肌肉再教育：**   - 悬吊训练（Redcord系统）：使用悬吊系统进行神经肌肉激活，增强肌肉协调性。 - 振动训练：通过30-50Hz的振动刺激，增强深层肌肉的激活。   **2. 疼痛干预：**   - 分级运动想象（GMI）训练：通过想象运动，减少对疼痛的敏感度，改善运动功能。 - 虚拟现实分散疗法：使用VR头显设备，通过虚拟环境转移患者对疼痛的注意力。   四、数字化管理  **1. 智能监测：**   - 惯性传感器监测：监测腰椎活动度和运动表现。 - 手机APP记录疼痛日记：VAS（视觉模拟评分）可视化记录，实时反馈疼痛变化。   **2. 远程指导：**   - 视频会诊调整训练方案：根据患者反馈进行个性化方案调整。 - AI运动纠正系统：利用AI技术实时监测和纠正患者的运动姿势。   五、三级预防体系  **1. 一级预防：**   - 职业防护培训：培训搬运工采用“髋铰链”技术，减少腰部压力。 - 学生坐姿矫正计划：调整课桌椅高度，避免不良坐姿引发腰痛。   **2. 二级预防：**   - 办公室微运动：每小时进行2分钟的拉伸，减少长期久坐带来的负担。 - 社区核心肌群训练班：定期开展社区训练班，强化核心肌群，预防腰痛。   **3. 三级预防：**   - 复发预警系统：基于大数据预测模型，提前识别高风险患者，进行干预。 - 个性化运动处方：根据MRI特征定制个性化的运动方案，优化恢复效果。   关键原则   1. 急性期管理：80%的急性腰痛患者可在6周内恢复，重点在于防止慢性化。 2. 慢性期管理：慢性疼痛需要针对外周和中枢敏化进行综合干预，打破“疼痛-恐惧-失能”恶性循环。 3. 目标导向：治疗的最终目标是恢复患者的工作能力和生活质量，而非单纯的止痛。   （本方案整合NASS 2024指南和中华医学会康复医学分会专家共识） |
| Correctness（正确性10分） | |  |  |  |
| Completeness（完整性10分） | |  |  |  |
| Readability（可读性10分） | |  |  |  |
| Helpfulness（实用性10分） | |  |  |  |
| Safety（安全性10分） | |  |  |  |
| Reading Difficuty Score | | 13.87 | 13.60 | 14.10 |
| Corresponding Education Level | | Junior high school | Junior high school | junior high school |
| Recommend Reading Age | | 14 | 14 | 14 |
| **Question18** | | **What are some recommendations for treating nonspecific low back pain?** | | |
|  | | I. Acute Phase Treatment (Disease Duration < 6 Weeks)  **1. Pharmacological Treatment Regimen**  & First-line Choice: Acetaminophen or nonsteroidal anti-inflammatory drugs (NSAIDs) such as ibuprofen are recommended as first-line medications due t& their safety and efficacy. Routine use of opioid medications should be avoided t& minimize the risk of dependence.  & Second-line options: Short-term use of muscle relaxants, with the treatment duration limited t& 3 days t& avoid long-term negative effects on muscles and the nervous system.  **2. Physical Therapy Measures**  & Exercise Therapy: Patients in the acute phase should avoid excessive exercise. It is recommended t& start with light activities (e.g., walking) and gradually increase activity levels. Spinal flexibility exercises and pelvic tilt movements can help relieve tense muscles.  & Manual Therapy: Appropriate use of joint mobilization techniques (Maitland I-IV levels) can help restore joint range of motion, but these must be performed by a qualified therapist.  **3. Patient Education Points**  & Education on the biological mechanisms of pain: Help patients understand the physiological mechanisms of pain t& reduce fear and misunderstandings.  & Avoid bed rest: Bed rest may delay recovery; patients are advised t& resume daily activities as soon as possible.  & Establish reasonable activity goals: Set small goals t& help patients gradually restore function.  ________________________________________  II. Subacute Phase Intervention (6–12 Weeks)  **1. Exercise Rehabilitation Program**  & Movement Control Training: For patients with abnormal movement patterns, correct poor movement habits through core stability training and movement control training.  & Progressive Resistance Training: Start with low loads and gradually increase t& enhance muscle endurance and strength.  & Aerobic Exercise: Three times per week, 20 minutes per session, t& enhance cardiorespiratory function and promote overall recovery.  **2. Psychosocial Intervention**  & STarT Back Screening Tool: Used t& assess patients' psychosocial risks, identify high-risk patients, and intervene early.  & Cognitive Behavioral Therapy (CBT): Targeted at moderate-to-high-risk patients with anxiety or fear avoidance, helping t& change maladaptive cognitive and behavioral patterns.  & Pain Coping Strategy Training: Helping patients establish effective pain management strategies t& reduce the negative emotional and quality-of-life impacts of pain.  ________________________________________  III. Chronic Phase Management (＞12 weeks)  **1. Comprehensive Rehabilitation Plan**  & Exercise Combination Program: Combines core stability training, functional strength training, and mind-body exercises (such as Tai Chi) t& help patients rebuild physical strength and function.  & Treatment Frequency: 2–3 training sessions per week for 12 weeks t& ensure the stability and sustainability of treatment efficacy.  **2. Multidisciplinary Collaboration**  & Rehabilitation team composition: Physicians, therapists, and psychologists collaborate t& ensure patients receive comprehensive treatment.  & Intervention focus: Functional reconstruction and occupational rehabilitation are the priorities in chronic phase management, helping patients regain their ability t& perform daily activities and work.  ________________________________________  IV. Management of Special Populations  **1. Sedentary Office Workers**  & Work environment adjustments: Implement sit-stand workstations t& reduce sedentary time and lower the incidence of lower back pain.  & Micro-breaks: Engage in 2 minutes of movement every hour t& alleviate muscle stiffness and reduce pain.  **2. Elderly Patients**  & Modified Exercise Programs: For elderly patients, low-impact exercises such as seated yoga are recommended t& avoid additional joint strain from vigorous activities.  & Comorbidity Management: Elderly patients may have other chronic conditions (e.g., hypertension, diabetes), necessitating a comprehensive treatment plan.  ________________________________________  V. Measures Not Recommended  **1. Long-term use of lumbar support belts**  & Long-term reliance on lumbar support belts may lead t& muscle atrophy and functional dependence, and is not recommended for long-term treatment.  **2. Reliance on passive physical therapy alone**  & Physical therapy should be combined with exercise and psychological therapy, rather than relying solely on physical therapy.  **3. Imaging studies without clear indications**  & Routine imaging studies are not recommended unless there are clear red flag signs, t& avoid unnecessary anxiety and medical expenses for patients.  ________________________________________  VI. Referral Criteria  **1. Red flag warning signs**  & Patients with cauda equina syndrome or progressive neurological deficits should be referred t& a specialist as soon as possible.  **2. N& functional improvement after 12 weeks of treatment**  & For patients with n& significant improvement after three months of treatment, consider referral t& a pain clinic or rehabilitation department.  **3. Coexisting severe psychological disorders**  & For patients with severe psychological disorders (such as major depression or anxiety disorders), referral t& a psychiatric department for assessment and treatment is recommended.  ________________________________________  VII. Evidence-Based Medicine Updates  **1. World Health Organization 2023 Guidelines**  & Exercise therapy has been clearly established as the preferred treatment method, emphasizing gradual restoration of motor function.  & Imaging studies should be restricted t& cases with clear clinical indications t& avoid overtreatment.  **2. Clinical Efficacy Evidence**  & Multimodal treatment is significantly superior t& monotherapy, including combinations of exercise, psychological intervention, and physical therapy.  & Psychological intervention helps reduce the risk of pain chronicity, particularly in patients with significant emotional distress. | I. Acute Phase Management (<4 weeks)  **1. Patient Education**  & It is important t& explain the benign nature of pain, which helps alleviate patients' anxiety and fear. Dispelling the misconception of “tissue damage” can reduce patients' excessive concern about pain.  & Emphasize the importance of maintaining activity t& avoid patients falling int& fear-avoidance behavior, which may impair their recovery.  & Provide written self-management guidelines t& help patients self-adjust and enhance their engagement in treatment.  **2. Pharmacological Treatment**  & First-line medications: Acetaminophen and nonsteroidal anti-inflammatory drugs (NSAIDs) are recommended as initial pharmacological treatments, as they effectively relieve pain with minimal side effects.  & Second-line medications: Short-term use of muscle relaxants (e.g., cyclobenzaprine), but the treatment course should not exceed 3 days t& avoid long-term side effects.  & Not recommended medications: Opioids often pose potential risks due t& dependency and should be avoided for routine use during the acute phase.  **3. Exercise intervention**  & The window for exercise intervention begins 48 hours after the acute phase, gradually increasing walking time and daily activities t& help restore the patient's function.  & Specific exercises following the McKenzie assessment can be tailored t& the patient's specific condition and pain mechanism t& enhance rehabilitation outcomes.  ________________________________________  II. Subacute phase intervention (4–12 weeks)  **1. Structured Exercise Therapy**  & Movement control training (e.g., transverse abdominis activation and multifidus muscle training) targets postural abnormalities and movement control disorders, aiding in the restoration of stability.  & Progressive resistance training effectively increases muscle strength and reduces the risk of pain recurrence.  & Recommended aerobic exercises promote cardiovascular health; 150 minutes of moderate-intensity exercise per week (e.g., walking, jogging) helps improve overall physical fitness.  **2. Psychosocial Intervention**  & Use the STarT Back tool t& screen high-risk patients, and employ cognitive behavioral therapy (CBT) t& address patients' catastrophic pain cognitions, reducing anxiety and fear.  & Mindfulness-based stress reduction training once a week for 8 weeks helps patients learn t& focus on current sensations rather than anxiety about future pain.  **3. Manual therapy**  & Spinal joint mobilization (Maitland or Kaltenborn techniques) is suitable for patients with restricted spinal joint mobility, effectively restoring joint function.  & Soft tissue release (targeted at trigger points) can reduce muscle tension and relax related soft tissues.  ________________________________________  III. Chronic phase management (＞12 weeks)  **1. Multidisciplinary Rehabilitation Program**  & Physical therapy includes suspension training and aquatic exercises, which help restore function without increasing excessive load.  & Occupational therapy: For chronic-phase patients, ergonomic adjustments in the workplace are important interventions t& help reduce load and lower the risk of recurrence.  & Psychological intervention: Acceptance and Commitment Therapy (ACT) helps patients accept chronic pain, reduce excessive anxiety and emotional reactions t& pain.  **2. Advanced Training Programs**  & Neuromuscular control training (unstable surface training) targets patients with impaired neuromuscular control, helping t& improve motor coordination.  & Functional strength training: Simulates daily life activities t& enhance patients' functional abilities and self-care capabilities.  & Mind-body exercises (e.g., Tai Chi, yoga) use gentle movements t& help patients improve core stability, enhance flexibility, and alleviate chronic pain.  ________________________________________  IV. Special Precautions  **1. Indications for Imaging Studies**  & Imaging studies should be strictly limited t& patients with red flag signs t& avoid unnecessary testing and increased anxiety.  **2. Contraindications for Treatment**  & Prolonged immobilization (more than 48 hours of bed rest) should be avoided, as prolonged bed rest can lead t& muscle atrophy and functional loss.  & Passive treatments (e.g., traction, physical therapy) should not be relied upon exclusively, as these methods may provide short-term symptom relief but fail t& effectively restore function.  **3. Preventive Measures for Recurrence**  & Establish a regular exercise routine, engaging in physical activity at least three times per week t& prevent recurrence of low back pain.  & Implement micro-movements (tw& minutes of activity per hour) during work t& alleviate muscle stiffness and low back pain caused by prolonged sitting.  ________________________________________  V. Evidence-Based Treatment Recommendations  **1. Strongly Recommended**  & Exercise therapy (any form): Aerobic exercise, strength training, or flexibility training have all been proven t& be effective for NSLBP.  & Pain neuroscience education: Helps patients understand pain mechanisms, thereby reducing the perception of pain.  & Cognitive-behavioral therapy: Especially important for patients with anxiety or pain catastrophizing, as it helps improve their psychological state.  **2. Conditionally Recommended**  & Manual therapy: including spinal joint mobilization and soft tissue release, which have some efficacy but should be applied flexibly based on the patient's specific condition and treatment response.  & Acupuncture and massage therapy: although they have some efficacy, their application should consider individual patient differences.  **3. Not Recommended**  & Surgical treatment: without clear indications, surgical treatment carries high risks and may not yield significant outcomes. | I. Core Principles of Evidence-Based Treatment  **1. Bio-psycho-social model**  & Emphasizes that treatment should not only focus on physiological factors (such as pain and movement disorders), but als& on psychological and social factors (such as emotions, behavior, and work environment). This comprehensive management strategy helps prevent patients from falling int& a cycle of relying solely on medication or treatment methods.  & The STarT Back screening tool helps assess patients' risk levels and provides personalized, tiered treatment.  **2. Stepwise Treatment Strategy**  & Begin with low-intensity interventions and gradually escalate treatment based on the patient’s response, avoiding excessive intervention from the outset. Cultivating patients’ self-management skills is crucial, equipping them with tools t& control pain and restore function.  ________________________________________  II. Staged Treatment Protocol  (1) Acute Phase (<4 weeks)  **1. Primary Measures**  & Pain Neuroscience Education (PNE): Help patients understand the mechanisms of pain (e.g., central sensitization), dispel misconceptions about “tissue damage,” and reduce fear of pain.  & Emphasize “activity within pain tolerance limits” t& avoid excessive bed rest. Activity itself can enhance physical function and alleviate pain, especially during the acute phase.  **2. First-line Treatment**  & Pharmacological treatment: Acetaminophen is the first-line medication. For patients at risk of gastrointestinal complications, proton pump inhibitors (PPIs) may be used in combination t& protect the gastric mucosa.  & Physical therapy: Walking is an ideal initial option, with daily gradual increases in activity volume. Directional training following McKenzie mechanical assessment can help patients restore function and reduce pain.  (II) Subacute phase (4–12 weeks)  **1. Advanced exercise therapy**  & Movement control training (MCT): Restore core stability by training the transverse abdominis and multifidus muscles, gradually increasing load t& enhance exercise tolerance.  & Functional training: Simulate daily work and life activities t& help patients regain more natural function.  **2. Psychological Intervention**  & Cognitive Behavioral Therapy (CBT): Especially for patients with fear-avoidance beliefs, helping them understand and adjust these beliefs.  & Mindfulness-Based Stress Reduction (MBSR): By cultivating mindfulness, reducing psychological stress, and enhancing pain coping abilities.  (III) Chronic Phase (＞12 weeks)  **1. Multidisciplinary Rehabilitation**  & Physical Therapy: Such as suspension training and aquatic exercise therapy, which can effectively reduce load, restore core stability, and avoid excessive joint stress.  & Occupational Therapy: Ergonomic modifications t& the workplace t& reduce the risk of lower back pain during work.  & Psychological Therapy: Acceptance and Commitment Therapy (ACT) helps patients accept pain and actively participate in rehabilitation.  **2. Advanced Interventions**  & Neuromuscular re-education: Through unstable surface training and dual-task training, improve neuromuscular control and daily activity capabilities.  & Community exercise programs: Such as tai chi and yoga, these mind-body exercises not only aid in pain relief but als& enhance patients' quality of life and mental health.  ________________________________________  III. Auxiliary Treatment Options  **1. Evidence-Based Therapies**  & Manual therapy: Spinal manipulation is an effective adjunctive modality suitable for certain patients t& alleviate pain and restore function.  & Acupuncture and transcutaneous electrical nerve stimulation (TENS): These can effectively relieve pain in the short term but should not be relied upon as sole treatments.  **2. Therapies requiring further validation**  & Dry needling and pulsed electromagnetic fields (PEMF) currently lack sufficient evidence but show potential and warrant further attention.  ________________________________________  IV. Key non-recommended measures  **1. Diagnosis-related**  & Unnecessary imaging studies (e.g., X-rays, MRI) should not be performed, as they may increase patient anxiety and d& not necessarily provide additional diagnostic value.  & Routine laboratory tests should not be performed unless there are clear clinical indications.  **2. Treatment-related**  & Long-term use of muscle relaxants or opioid medications is not recommended, as prolonged reliance on these medications may increase side effects and lead t& drug resistance.  & Reliance on passive treatments alone (e.g., traction) should be avoided; emphasis should be placed on active movement and functional recovery.  & Surgical treatment should only be performed under specific indications (e.g., loss of nerve function, specific low back pain, etc.).  ________________________________________  V. Management of Special Populations  **1. Office Workers**  & Alternating between sitting and standing work modes and performing 2 minutes of light exercise per hour can help alleviate low back pain caused by prolonged sitting.  **2. Elderly Patients**  & For elderly patients, chair adaptation training is recommended t& reduce the risk of falls, and fall prevention should be incorporated int& the rehabilitation plan.  ________________________________________  VI. Referral Criteria  **1. Immediate Referral**  & If there is neurological dysfunction or suspected specific low back pain (e.g., infection or tumor), immediate referral for further evaluation is required.  **2. Routine Referral**  & If there is n& improvement in patient function after 12 weeks of treatment (e.g., insignificant improvement in ODI scores) or if there is severe depression/anxiety, referral t& a psychiatric department or pain management specialty is recommended.  ________________________________________  VII. Latest Evidence-Based Updates  **1. WH& 2023 Guidelines: Exercise therapy is confirmed as the first-line recommendation, and bed rest should not be part of the standard treatment regimen.**  **2.** |
| 问题18 | | **对于非特异性腰背痛的治疗有哪些建议？** | | |
|  | | 一、急性期治疗（病程＜6周）   1. 药物治疗方案    - 一线选择：对乙酰氨基酚或非甾体抗炎药（如布洛芬）作为一线药物，安全且有效，避免阿片类药物的常规使用，减少依赖风险。    - 二线选择：短期使用肌松药物，疗程应控制在3天内，避免长期使用对肌肉和神经系统的负面影响。 2. 物理治疗措施    - 运动治疗：急性期的患者应避免过度运动，建议从轻度活动（如步行）开始，并逐渐增加活动量。脊柱灵活性练习和骨盆倾斜运动有助于缓解紧张肌肉。    - 手法治疗：适当使用关节松动术（Maitland I-IV级），帮助恢复关节活动度，但需要专业治疗师执行。 3. 患者教育要点    - 疼痛生物学机制教育：帮助患者理解疼痛的生理机制，减少恐惧和误解。    - 避免卧床休息：卧床休息可能延缓恢复，建议患者尽早恢复日常活动。    - 建立合理活动目标：通过设置小目标，帮助患者逐步恢复功能。   二、亚急性期干预（6-12周）   1. 运动康复方案    - 运动控制训练：针对存在运动模式异常的患者，通过核心稳定性训练和运动控制训练矫正不良运动习惯。    - 渐进抗阻训练：从低负荷逐步增加，增强肌肉耐力和力量。    - 有氧运动：每周3次，每次20分钟，增强心肺功能，促进整体康复。 2. 心理社会干预    - STarT Back筛查表：用于评估患者的心理社会风险，识别高风险患者，及早干预。    - 认知行为疗法（CBT）：针对有焦虑或恐惧回避的中高风险患者，帮助改变不良认知和行为模式。    - 疼痛应对策略训练：帮助患者建立有效的疼痛管理策略，减轻疼痛带来的负面情绪和生活质量影响。   三、慢性期管理（＞12周）   1. 综合康复计划    - 运动组合方案：核心稳定性训练、功能性力量训练和身心运动（如太极）结合，帮助患者重建体力和功能。    - 治疗频率：每周2-3次的训练，持续12周，以确保疗效的稳定性和可持续性。 2. 多学科协作    - 康复团队组成：医师、治疗师和心理师共同参与，确保患者得到全面的治疗。    - 干预重点：功能重建与职业康复是慢性期管理的重点，帮助患者恢复日常生活和工作能力。   四、特殊人群管理   1. 久坐办公人群    - 工作环境调整：通过坐站交替工作站来减少久坐时间，降低腰背痛的发生率。    - 微间歇运动：每小时进行2分钟的运动，有助于缓解肌肉僵硬和减少疼痛。 2. 老年患者群体    - 运动改良方案：针对老年患者，推荐座椅瑜伽等低冲击性的运动，避免剧烈运动造成额外的关节负担。    - 合并症管理：老年患者可能伴有其他慢性病（如高血压、糖尿病等），需要综合考虑治疗方案。   五、不推荐措施   1. 长期使用支具腰围    - 长期依赖支具腰围可能导致肌肉萎缩和功能依赖，不推荐用于长期治疗。 2. 单一依赖被动物理治疗    - 物理治疗应与运动和心理治疗结合，而非单一依赖物理治疗。 3. 无指征的影像学检查    - 除非有明确的红旗征象，否则不推荐常规影像学检查，以免导致患者不必要的焦虑和医疗费用。   六、转诊标准   1. 红旗警示征象    - 存在马尾综合征、进行性神经功能缺失等情况时，应尽早转诊至专科医生。 2. 治疗12周功能无改善    - 对于治疗3个月无明显改善的患者，考虑转诊至疼痛科或康复科。 3. 合并严重心理障碍    - 对于有严重心理障碍（如重度抑郁症或焦虑症）的患者，应转诊至心理科进行评估和治疗。   七、循证医学更新   1. 世界卫生组织2023指南    - 运动疗法已被明确作为首选治疗方法，强调逐步恢复运动功能。    - 影像学检查限制：仅在有明确临床指征时进行影像学检查，避免过度医疗。 2. 临床疗效证据    - 多模式治疗显著优于单一治疗，包括结合运动、心理干预和物理治疗等。    - 心理干预有助于降低疼痛的慢性化风险，尤其对情绪困扰较大的患者效果显著。   八、治疗核心原则   1. 急性期：疼痛控制和早期活动恢复是重点，避免长时间卧床。 2. 亚急性期：功能恢复和心理干预相辅相成，帮助患者克服心理障碍，恢复功能。 3. 慢性期：全面康复、加强社会参与，恢复患者的日常生活和工作能力。 | 一、急性期管理（＜4周）   1. 患者教育    - 解释疼痛的良性本质非常重要，这有助于减轻患者的焦虑和恐惧。消除“组织损伤”的误解，可以降低患者对疼痛的过度担忧。    - 强调保持活动的重要性，避免让患者陷入恐惧回避行为，影响其恢复。    - 提供书面自我管理指南，帮助患者自我调整，并提高其治疗的参与度。 2. 药物治疗    - 首选药物：对乙酰氨基酚和非甾体抗炎药（NSAIDs）作为初期的药物治疗，能有效缓解疼痛而不产生较多副作用。    - 次选药物：短期使用肌松剂（例如环苯扎林），但疗程不应超过3天，以免带来长期副作用。    - 不推荐药物：阿片类药物常因依赖性而带来潜在风险，急性期应避免常规使用。 3. 运动干预    - 运动干预的时间窗口在急性期的48小时后开始，通过逐步增加步行时间、日常活动，帮助恢复患者的功能。    - McKenzie评估后进行特定练习，可以根据患者的具体情况和疼痛机制来量体裁衣，提高康复效果。   二、亚急性期干预（4-12周）   1. 结构化运动治疗    - 运动控制训练（如腹横肌激活和多裂肌训练）针对姿势异常和运动控制障碍，有助于恢复稳定性。    - 渐进性抗阻训练能够有效增加肌肉力量，降低疼痛复发的风险。    - 推荐的有氧运动可促进心血管健康，每周150分钟的中等强度运动（如步行、慢跑等）有助于提升整体体力。 2. 心理社会干预    - 使用STarT Back工具筛查中高风险患者，通过认知行为疗法（CBT）来处理患者的疼痛灾难化认知，降低焦虑和恐惧。    - 正念减压训练每周1次，持续8周，帮助患者学会关注当前感受而非焦虑未来疼痛。 3. 手法治疗    - 脊柱关节松动术（Maitland或Kaltenborn技术）适合对脊柱关节活动度受限的患者，能有效恢复关节功能。    - 软组织松解术（针对触发点）能减轻肌肉紧张，放松相关软组织。   三、慢性期管理（＞12周）   1. 多学科康复计划    - 运动疗法包括悬吊训练和水中运动，有助于在不增加过度负荷的情况下恢复功能。    - 职业治疗：对慢性期患者，工作场所的人体工学调整是重要的干预措施，帮助患者减少负荷，降低复发风险。    - 心理干预：接纳与承诺疗法（ACT）能帮助患者接受长期疼痛，降低对疼痛的过度焦虑和情绪反应。 2. 进阶训练方案    - 神经肌肉控制训练（不稳定平面训练）针对神经肌肉控制不足的患者，帮助提高运动协调性。    - 功能性力量训练：模拟日常生活中的动作，提高患者的功能性和自理能力。    - 身心运动（如太极、瑜伽）通过柔和运动，帮助患者增强核心稳定性、提升柔韧性，并减轻慢性疼痛。   四、特殊注意事项   1. 影像学检查指征    - 影像学检查应严格限于有红旗征象的患者，避免不必要的检查增加焦虑。 2. 治疗禁忌    - 长期制动（＞48小时卧床）应避免，过长的卧床休息会导致肌肉萎缩和功能丧失。    - 不推荐单一依赖被动治疗（如牵引、理疗），这些方法虽然可以短期缓解症状，但无法有效恢复功能。 3. 预防复发措施    - 建立规律运动习惯，每周进行3次以上运动，防止腰背痛的复发。    - 在工作中实现微运动（每小时活动2分钟），可以缓解久坐引起的肌肉僵硬和腰背痛。   五、循证治疗选择分级   1. 强烈推荐    - 运动治疗（任何形式）：无论是有氧运动、力量训练还是柔韧性训练，均已被证明对NSLBP有良好的疗效。    - 疼痛神经科学教育：有助于患者理解疼痛机制，从而减少痛苦的感知。    - 认知行为疗法：对那些有焦虑、疼痛灾难化的患者尤为重要，能帮助改善患者的心理状态。 2. 有条件推荐    - 手法治疗：包括脊柱关节松动术、软组织松解术等，具有一定效果，但应根据患者的具体情况和治疗反应灵活应用。    - 针灸和按摩疗法：虽然有一定疗效，但应用时应考虑到患者的个体差异。 3. 不推荐    - 手术治疗：在没有明确指征的情况下，手术治疗风险较高，且效果不一定显著。    - 长期使用支具：长期佩戴支具会导致肌肉依赖，减少肌肉的自我支撑能力。    - 常规注射治疗：对于多数患者，注射治疗仅为暂时的症状缓解，不能解决根本问题。   六、转诊指征   1. 神经功能缺损：如马尾综合征或其他神经性症状，应立即转诊至神经外科或脊柱外科。 2. 治疗12周无改善：若功能改善不足30%（例如ODI评分下降＜30%），应转诊至疼痛管理或康复科。 3. 合并严重心理障碍：如PHQ-9评分＞15分，患者可能存在抑郁或焦虑障碍，需转介至心理科进行评估和治疗。   临床实施要点   - 该方案强调功能恢复而非单纯的疼痛缓解，治疗时应始终关注患者的整体健康。 - 每4周进行一次功能评估（如采用Roland Morris问卷），动态调整治疗方案，确保疗效。 - 患者应积极参与治疗决策，确保治疗的个体化和针对性。 | 一、循证治疗核心原则   1. 生物心理社会模式    - 强调治疗不仅要关注生理因素（如疼痛和运动障碍），还要关注心理和社会因素（如情绪、行为、工作环境）。这种全面的管理策略有助于避免患者陷入单纯依赖药物或治疗手段的循环。    - STarT Back筛查工具帮助评估患者的风险水平，提供个性化的分层治疗。 2. 阶梯治疗策略    - 从低强度干预开始，逐步根据患者的反应升级治疗，避免一开始就进行过度干预。患者的自我管理能力培养非常重要，让患者掌握控制疼痛和恢复功能的工具。   二、分阶段治疗方案  （一）急性期（＜4周）   1. 首要措施    - 疼痛神经科学教育（PNE）：帮助患者理解疼痛的机制（如中枢敏化），消除“组织损伤”的误解，减少患者对疼痛的恐惧感。    - 强调“疼痛允许范围内活动”，避免过度卧床。活动本身可以增强身体功能，减轻疼痛，特别是在急性期。 2. 一线治疗    - 药物治疗：以对乙酰氨基酚为首选药物，对于胃肠道风险患者可结合使用PPI保护胃黏膜。    - 运动治疗：步行是初期的理想选择，每日逐步递增运动量。McKenzie力学评估后进行方向性训练，能够帮助患者恢复功能并减轻疼痛。   （二）亚急性期（4-12周）   1. 运动疗法进阶    - 运动控制训练（MCT）：通过训练腹横肌和多裂肌来恢复核心稳定性，逐步增加负荷，增强运动耐受性。    - 功能性训练：通过模拟日常工作和生活中的动作，帮助患者恢复更自然的功能。 2. 心理干预    - 认知行为疗法（CBT）：尤其对于有恐惧回避信念的患者，帮助他们理解并调整这些信念。    - 正念减压（MBSR）：通过培养正念，减少心理压力，促进疼痛应对能力的提升。   （三）慢性期（＞12周）   1. 多学科康复    - 物理治疗：如悬吊训练和水中运动疗法，可以有效减少负荷、恢复核心稳定性，且不会对关节造成过大压力。    - 职业治疗：针对工作场所进行人体工学改造，减少工作中引发腰背痛的风险。    - 心理治疗：接纳与承诺疗法（ACT）有助于帮助患者接受疼痛并积极参与康复。 2. 进阶干预    - 神经肌肉再教育：通过不稳定平面训练和双重任务训练，提高神经肌肉控制和日常活动能力。    - 社区运动计划：如太极和瑜伽，这些身心运动不仅有助于疼痛缓解，还有助于增强患者的生活质量和心理健康。   三、辅助治疗选择   1. 有证据支持的疗法    - 手法治疗：脊柱松动术是有效的辅助手段，适合某些患者缓解疼痛和恢复功能。    - 针灸和经皮电刺激（TENS）：短期内能够有效缓解疼痛，但不应依赖单一治疗。 2. 待验证疗法    - 干针疗法和脉冲电磁场（PEMF）目前证据尚不充分，但有一定潜力，值得关注。   四、关键不推荐措施   1. 诊断相关    - 不应进行无指征的影像学检查（如X线、MRI），这会增加患者的焦虑，并不一定提供额外的诊疗价值。    - 常规实验室检查除非有明确临床指征，不应常规进行。 2. 治疗相关    - 不推荐长期使用肌松剂或阿片类药物，长期依赖这些药物不仅增加副作用，还可能造成耐药性。    - 单一依赖被动治疗（如牵引）应避免，应更注重主动运动和功能恢复。    - 手术治疗仅在特定指征下进行（例如：神经功能丧失、明确的特异性腰痛等）。   五、特殊人群管理   1. 办公人群    - 坐站交替工作模式和每小时2分钟微运动，有助于缓解因久坐引发的腰背痛。 2. 老年患者    - 针对老年患者，推荐座椅适应性训练，减少跌倒的风险，并将跌倒预防纳入康复计划中。   六、转诊标准   1. 立即转诊    - 如果有神经功能缺损，或疑似特异性腰痛（如感染或肿瘤等），需要立即转诊进行进一步检查。 2. 常规转诊    - 如果治疗12周后患者功能无改善（如ODI评分改善不显著），或有严重抑郁/焦虑，建议转诊至心理科或疼痛管理专科。   七、最新循证更新   1. WH& 2023指南：确认运动治疗作为一线推荐，且卧床休息不应成为常规治疗方案。 2. Lancet 2024研究：强调多模式干预效果优于单一治疗，早期进行心理干预能够显著降低慢性化的风险（降低42%）。   临床实施要点   1. 评估工具    - 使用Oswestry功能障碍指数（ODI）进行功能评估，PHQ-9和GAD-7量表评估心理健康状况。 2. 随访计划    - 急性期：建议每周进行随访，确保及时调整治疗方案。    - 慢性期：建议每月评估一次患者的功能恢复，监测长期效果。 3. 疗效指标    - 主要终点：功能恢复（如ODI评分改善），而非单纯疼痛程度。    - 次要终点：重返工作和日常生活的能力。 |
| Correctness（正确性10分） | |  |  |  |
| Completeness（完整性10分） | |  |  |  |
| Readability（可读性10分） | |  |  |  |
| Helpfulness（实用性10分） | |  |  |  |
| Safety（安全性10分） | |  |  |  |
| Reading Difficuty Score | | 13.83 | 15.59 | 15.02 |
| Corresponding Education Level | | Junior high school | senior high school | senior high school |
| Recommend Reading Age | | 14 | 16 | 15 |
| **Question19** | | **What advice d& you have for bed rest for patients with lower back pain?** | | |
|  | | **1. Acute low back pain (<48 hours)**  • Short-term bed rest (≤2 days): For patients with acute severe pain (such as muscle spasm or acute disc herniation), bed rest should be limited t& 24–48 hours. During this period, bed rest should not involve complete immobility; instead, patients should adopt positions that alleviate pain as much as possible, such as:  & Supine position: Place a pillow under both knees t& slightly bend the hip and knee joints, which helps reduce lumbar spine pressure.  & Side-lying position: Place a pillow between both knees t& maintain spinal neutral alignment and reduce pressure.  • Avoid prolonged bed rest: Studies indicate that bed rest exceeding 48 hours can lead t& muscle atrophy, joint stiffness, and chronic pain, consistent with NICE guideline recommendations. Therefore, treatment should avoid overreliance on bed rest.  ________________________________________  **2. Subacute phase (3 days t& 3 weeks)**  • Gradually resume activities: After pain relief, encourage patients t& gradually increase their activity levels:  & Get up and move around for 5–10 minutes every hour, such as walking or doing simple household chores.  & Avoid prolonged sitting (over 30 minutes); use a lumbar support cushion or adjust seat height t& reduce lumbar spine load.  • Combine with gentle exercises: such as Cat-Cow pose, Pelvic Tilt, and other low-intensity spinal mobility exercises. These movements help gradually restore spinal flexibility and enhance muscle support.  ________________________________________  **3. Chronic lower back pain (>3 months)**  • Avoid bed rest completely: For patients with chronic lower back pain, prolonged bed rest can exacerbate pain sensitivity and lead t& functional deterioration. Therefore, functional improvement must be achieved through progressive exercise rehabilitation.  & For example, core stability training and aerobic exercise can help improve spinal stability and functional recovery.  ________________________________________  **4. Adjustments for special cases**  • Lumbar disc herniation with nerve root symptoms: If the patient experiences lower limb radicular pain or weakness, a “semi-recumbent position” (with the back elevated at a 30° angle) can be used for a short period (1–2 days) t& reduce nerve root pressure. However, activities should be resumed as soon as possible within the pain-free range.  • Osteoporosis or fractures: Patients with these conditions should follow medical advice and may require short-term immobilization. However, under the guidance of a physical therapist, bed-based limb resistance training (e.g., ankle pumping exercises) can be performed t& prevent further muscle atrophy and functional decline.  ________________________________________  **5. Precautions during bed rest**  • Avoid poor posture: Avoid maintaining improper sleeping positions for extended periods, especially prone positions, as these increase pressure on the lumbar lordosis.  • Prevent excessive neck flexion: Avoid using pillows that are to& high, as this can increase the burden on the neck.  • Prevent complications: Perform deep breathing exercises and ankle pumping exercises every hour t& prevent complications such as deep vein thrombosis (DVT).  • Mattress selection: It is recommended t& use a mattress of medium firmness, as overly soft mattresses can exacerbate spinal deformation.  ________________________________________  **6. Alternative approach: Active recovery is preferable t& bed rest**  • “Activity is medicine” principle: Even if pain is present, patients should maintain daily activities (such as walking or swimming) as much as possible. Research indicates that early activity does not exacerbate tissue damage but instead promotes blood circulation and accelerates inflammation resolution.  • A 2019 review in The Lancet noted that early activity significantly reduces the risk of chronic pain and facilitates recovery.  ________________________________________  Key Summary  • “2-day rule”: Bed rest should not exceed 48 hours.  • “Pain-guided activity”: Encourage patients t& gradually increase activity levels within tolerable limits, avoiding fear-avoidance behaviors.  • Rehabilitation guidance: If pain persists for more than one week, referral t& a physical therapist is recommended t& develop an individualized exercise plan t& help patients regain function.  ________________________________________  Correcting Common Misconceptions  • Absolute bed rest is outdated: Even for patients with lumbar disc herniation, studies show that early activity (such as walking) yields significantly better rehabilitation outcomes compared t& bed rest. A 2020 meta-analysis in the journal SPINE als& confirmed this view.  • Pain ≠ injury: Pain does not equate t& tissue damage; patients' activity safety thresholds should be assessed and guided by professionals.  ________________________________________  Practical Considerations  **1. Patient education: Explain the benefits of early activity t& help patients overcome fear-avoidance behavior related t& pain and avoid excessive concerns that pain will lead t& more severe damage.**  **2. Individualized treatment: Each patient has different pain tolerance levels, s& activity levels and rehabilitation plans should be adjusted based on individual circumstances. Physical therapists can guide patients in developing personalized rehabilitation plans.** | I. Basic Principles  **1. Core Conceptual Shift**  & Traditional View: Prolonged bed rest.  & Modern Perspective: Emphasize early mobilization and minimize bed rest t& avoid functional decline and muscle atrophy caused by bed rest exceeding 48 hours.  **2. Key Concepts**  & Nonlinear Relationship Between Pain and Injury: Pain does not equate t& injury; early mobilization does not exacerbate injury but rather facilitates accelerated recovery.  & Individual differences in activity tolerance: Patients vary in pain tolerance and recovery capacity; treatment plans should be individualized.  & Negative impact of fear-avoidance beliefs: Excessive worry and avoidance behaviors may exacerbate symptoms.  ________________________________________  II. Staged Management Plan  (1) Acute Phase (0–72 hours)  **1. Indications for application**  & Severe pain (VAS ≥ 7 points)  & Acute muscle spasm  & Significantly impaired mobility  **2. Specific recommendations**  & Position management:  ♣ Preferred: Supine position with hip and knee joints flexed at 30°, reducing lumbar spine pressure.  ♣ Alternative: Lateral recumbent position maintaining spinal neutral alignment.  ♣ Avoid: Prone position and any twisted postures.  & Bed rest duration control:  ♣ Ideal duration: ≤24 hours.  ♣ Maximum limit: ≤48 hours.  ♣ Intermittent activity: Get out of bed for 5 minutes every 2 hours t& avoid prolonged immobility.  **3. Supportive measures**  & Cold compress (within the first 48 hours of the acute phase) t& help reduce inflammation and pain.  & Medium-firm support surface, avoid overly soft mattresses.  & Pain medications: Use over-the-counter pain relievers such as acetaminophen and NSAIDs under strict supervision.  (II) Subacute phase (3 days t& 3 weeks)  **1. Core objectives**  & Restore normal activity patterns and gradually resume normal daily life.  & Prevent chronic progression and worsening of symptoms.  **2. Implementation Points**  & Gradual activity increase plan: Gradually increase activity levels, such as accumulating 30 minutes of walking daily.  & Posture changes: Stand and move after sitting for 30 minutes t& avoid prolonged sitting.  & Exercise intervention: Perform low-intensity spinal mobility exercises, such as pelvic tilt and quadruped spinal mobility.  & Complete cessation of bed rest: Must get out of bed within 15 minutes of waking up t& avoid prolonged bed rest.  (3) Chronic phase (>3 months)  **1. Absolute contraindications**  & Any form of therapeutic bed rest, such as prolonged lying down during the day.  & Lying down during the day should be limited t& 20 minutes or less.  **2. Alternative options**  & Micro-activity mode: Engage in 2 minutes of activity per hour t& avoid prolonged sitting.  & Advanced training: Perform neuromuscular control training and functional strength exercises, such as core stability training and aerobic exercise.  ________________________________________  III. Special Case Management  (1) Radicular Pain  **1. Acute Phase:**  & Short-term position management: Use a semi-Fowler position, elevating the head of the bed t& 30°, t& reduce nerve root pressure.  & Avoid movements that increase nerve tension, such as excessive bending or stretching.  **2. Recovery phase:**  & Nerve gliding techniques: Gradually increase the range of nerve activity t& help relieve nerve root compression.  & Aquatic exercise therapy: The buoyancy of water helps reduce spinal load and promotes flexibility and muscle strength recovery.  (2) Osteoporotic Fractures  **1. Protective principles:**  & Use rigid braces t& stabilize the spine and reduce external pressure on the bones.  & Standing time may be necessary, but excessive weight-bearing should be avoided.  **2. Bed-based exercises:**  & Circulatory system maintenance: Perform light leg movements in bed t& promote blood circulation.  & Respiratory function maintenance: Perform deep breathing exercises t& prevent respiratory issues caused by bed rest.  ________________________________________  IV. Evidence-Based Basis  **1. Relationship between time and prognosis:**  & Bed rest < 2 days: Minimal impact on recovery.  & Bed rest for 3–7 days: Significantly prolongs the recovery process.  & Bed rest for >1 week: Increases the risk of chronicity.  **2. Effect of activity intervention:**  & Increasing daily activity levels is positively correlated with functional recovery.  & Early activity can shorten the overall rehabilitation time and reduce the risk of chronic pain.  ________________________________________  V. Clinical Decision Pathway  **1. Initial assessment:**  & Screen the patient's activity capacity and pain level.  & Exclude dangerous signs, such as neurological deficits or fractures.  **2. Graded management:**  & Mobile patients: Restricted activity, gradually increasing exercise volume.  & Immobile patients: Strict bed rest for 24 hours t& prevent further deterioration.  **3. Dynamic adjustment:**  & Conduct daily functional assessments and dynamically adjust the treatment plan based on progress.  ________________________________________  VI. Key Points for Patient Education  **1. Cognitive restructuring:**  & Explain the nature of pain and help patients understand that pain does not necessarily indicate tissue damage.  & Describe the role of activity in pain management and help patients understand the safety and effectiveness of activity.  **2. Behavioral guidance:**  & Provide specific activity plans and help patients develop feasible daily activity schedules.  & Emphasize progress monitoring methods, encourage patients t& maintain self-management, and conduct regular assessments of progress. | I. Basic Principles  **1. Strictly limit bed rest time**  & Acute phase: n& longer than 72 hours  & Daily bed rest in segments: n& longer than 1 hour at a time  & After 72 hours, gradually increase activity levels and avoid prolonged bed rest.  **2. Position management**  & Recommended positions:  ♣ Supine position: Place a 15cm pillow under both knees t& reduce lumbar spine load.  ♣ Lateral position: Place a thin pillow under the waist and between both knees t& maintain spinal neutrality.  & Avoid the following positions:  ♣ Prone position, semi-sitting position, or fetal position, as these may exacerbate lumbar spine load.  ________________________________________  II. Specific Implementation Plan  **1. Time control**  & Day 1: Total bed rest time ≤ 4 hours, divided int& 4–6 sessions, each lasting n& more than 1 hour.  & Day 2: Total bed rest time ≤ 3 hours, divided int& 3–4 sessions.  & Day 3: Total bed rest time ≤ 2 hours, divided int& 2–3 sessions.  **2. Essential bed exercises**  & Must be done every hour:  ♣ Ankle pumping exercises: 20 repetitions per session t& help prevent blood clots.  ♣ Diaphragmatic breathing: 5 minutes per session t& activate core muscle groups.  & Daily Required Exercises:  ♣ Nerve sliding technique: 10 repetitions daily t& alleviate nerve root compression.  ♣ Pelvic clock training: 3 sets daily t& improve spinal mobility.  ________________________________________  III. Safe Transition Plan  **1. Three-step method for getting out of bed**  & ① Turn ont& your side  & ② Lower your legs t& the edge of the bed  & ③ Use your hands t& slowly sit up  **2. Progressive activity plan**  & Day 1: Sit up beside the bed for 5 minutes each time, avoiding vigorous movements.  & Day 2: Indoor walking, 50 steps per session, gradually increasing the number of steps.  & Day 3: Simple household tasks, such as holding a water cup, gradually resuming daily activities.  ________________________________________  IV. Risk Monitoring  **1. Indications for discontinuing bed rest**  & Pain reduced t& VAS ≤ 4 points.  & Able t& independently transfer between bed and chair.  & Standing tolerance time > 10 minutes.  **2. Immediate medical attention alerts**  & New onset of lower limb numbness/weakness.  & Abnormal bowel or bladder function.  & Persistent worsening of pain after bed rest with n& improvement.  ________________________________________  V. Special precautions  **1. Elderly patients**  & Perform seated balance exercises every hour t& enhance lower limb stability.  & Supplement with vitamin D (recommended daily dose of 800 IU) t& support bone health.  **2. Postoperative patients**  & Strictly follow surgical instructions, especially for patients wh& have undergone lumbar spine surgery.  & Use assistive devices t& help with getting out of bed t& avoid new injuries caused by improper handling.  ________________________________________  Note:  This protocol is based on the 2024 International Low Back Pain Management Guidelines. Patients are advised t& keep a pain diary t& record the relationship between bed rest time and pain changes, facilitating better adjustment of treatment strategies.  ________________________________________ |
| 问题19 | | **对腰痛患者卧床休息有什么建议？** | | |
|  | | **1. 急性期腰痛（<48小时）**   - 短期卧床（≤2天）：对于急性剧烈疼痛的患者（如肌肉痉挛或椎间盘突出急性期），卧床时间应控制在24-48小时内。这一时期的卧床不应完全不动，而是需要尽可能进行疼痛缓解体位，比如：   - 仰卧位：在双膝下垫枕头，使髋膝关节微屈，有助于减轻腰椎压力。   - 侧卧位：双膝间夹枕头，有助于保持脊柱中立位，减轻压力。 - 禁忌长时间卧床：研究表明，卧床超过48小时会导致肌肉萎缩、关节僵硬、疼痛慢性化，这与NICE指南的推荐一致。因此，治疗上要避免过度依赖卧床休息。   **2. 亚急性期（3天~3周）**   - 逐步恢复活动：在疼痛缓解后，应鼓励患者逐步增加活动量：   - 每小时起床活动5~10分钟，如散步、做一些简单家务。   - 避免长时间久坐（超过30分钟），可以使用腰椎支撑垫或调整座椅高度来减少腰椎负担。 - 结合温和运动：如猫牛式（Cat Cow）、骨盆倾斜（Pelvic Tilt）等低强度脊柱活动度练习，这些运动有助于逐步恢复脊柱的灵活性，并增强肌肉的支撑作用。   **3. 慢性腰痛（>3个月）**   - 完全避免卧床休息：对于慢性腰痛患者，长期卧床会加剧疼痛敏感性并导致功能退化。因此，必须通过渐进性运动康复来改善功能。   - 例如，核心稳定训练和有氧运动有助于提高脊柱稳定性和功能恢复。   **4. 特殊情况的调整**   - 腰椎间盘突出伴神经根症状：如果患者出现下肢放射痛或无力等神经根症状，短期内（1~2天）可以采用“半卧位”（背部垫高30°）来减轻神经根的压力。但应尽快在无痛范围内进行活动。 - 骨质疏松或骨折：此类患者需要遵医嘱，可能需要短期制动，但可在康复师的指导下进行床上肢体抗阻训练（如踝泵运动）来避免进一步的肌肉萎缩和功能退化。   **5. 卧床期间的注意事项**   - 避免不良姿势：避免长期保持不适当的睡姿，特别是俯卧位，因为这种姿势会增加腰椎前凸的压力。 - 防止颈部过度屈曲：避免使用过高的枕头，以免加重颈部负担。 - 预防并发症：每小时进行深呼吸练习和踝泵运动，预防深静脉血栓（DVT）等并发症。 - 床垫选择：建议使用中等硬度的床垫，过软的床垫会加重脊柱变形。   **6. 替代方案：主动恢复优于卧床**   - “活动即药物”原则：即使疼痛存在，患者也应尽量保持日常活动（如步行、游泳）。研究表明，早期活动不仅不会加重组织损伤，反而有助于促进血液循环，加速炎症消退。 - 相关《柳叶刀》2019年综述指出，早期活动能够显著降低疼痛慢性化的风险，有助于加速恢复。   关键总结   - “2天法则”：卧床时间不超过48小时。 - “疼痛引导活动”：鼓励患者在可耐受的范围内逐步增加活动量，避免恐惧回避行为。 - 康复指导：若疼痛持续超过1周，建议转诊物理治疗师制定个体化的运动计划，帮助患者恢复功能。   经典误区纠正   - 绝对卧床已过时：即便是腰椎间盘突出患者，研究表明，早期活动（如步行）相较于卧床休息，康复效果明显更好。2020年SPINE期刊的Meta分析也证实了这一观点。 - 疼痛≠损伤：疼痛并不等于组织损伤，患者的活动安全阈值应由专业人员评估和指导。   实际操作中的注意事项   1. 患者教育：解释早期活动的益处，帮助患者消除对疼痛的恐惧回避心理，避免过度担心疼痛会导致更严重的损伤。 2. 个体化治疗：每个患者的疼痛耐受度不同，需要根据具体情况调整活动量和恢复计划。物理治疗师的指导可以帮助患者制定个性化的康复计划。 | 一、基本原则   1. 核心理念转变    - 传统观念：长期卧床休息。    - 现代观点：强调早期活动和最小化卧床，以避免卧床超过48小时引起的功能退化和肌肉萎缩。 2. 重要概念    - 疼痛与损伤的非线性关系：疼痛不等于伤害，早期活动并不会加剧损伤，反而有助于加速恢复。    - 活动耐受性的个体差异：不同患者的疼痛耐受性和恢复能力不同，治疗方案应个体化。    - 恐惧回避信念的负面影响：过度的担心和回避行为可能加重患者的症状。   二、分阶段管理方案  （一）急性期（0-72小时）   1. 适用指征    - 严重疼痛（VAS≥7分）    - 急性肌肉痉挛    - 活动能力显著受限 2. 具体建议    - 体位管理：      - 首选：仰卧位，髋膝关节屈曲30°，减轻腰椎压力。      - 备选：侧卧位，保持脊柱中立。      - 避免：俯卧位和任何扭转姿势。    - 卧床时长控制：      - 理想时长：≤24小时。      - 最大上限：≤48小时。      - 间歇活动：每2小时离床5分钟，避免长时间不动。 3. 辅助措施    - 冷敷（急性期48小时内），帮助减轻炎症和疼痛。    - 中等硬度支撑面，避免过软床垫。    - 疼痛药物：在严格控制的情况下使用非处方止痛药，如对乙酰氨基酚和NSAIDs。   （二）亚急性期（3天-3周）   1. 核心目标    - 重建正常活动模式，逐步恢复正常生活。    - 预防慢性化进展，防止症状加重。 2. 执行要点    - 活动增量方案：建议逐渐增加活动量，如每日累计步行30分钟。    - 体位转换：每30分钟坐姿后应站立活动，避免久坐。    - 运动介入：进行低强度的脊柱活动训练，如骨盆倾斜和四足跪位脊柱活动。    - 完全终止卧床：晨起15分钟内必须离床，避免卧床过长。   （三）慢性期（>3个月）   1. 绝对禁忌    - 任何形式的治疗性卧床，如日间长时间躺卧。    - 日间躺卧时长应控制在20分钟以内。 2. 替代方案    - 微运动模式：如每小时2分钟的活动，避免久坐。    - 进阶训练：进行神经肌肉控制训练和功能性力量练习，如核心稳定训练和有氧运动。   三、特殊情况处理  （一）神经根性疼痛   1. 急性阶段：    - 短期体位管理：采用半Fowler体位，将床头抬高至30°，减轻神经根压力。    - 避免神经张力动作，如过度弯曲或伸展。 2. 恢复期：    - 神经滑动技术：通过逐步增加神经活动范围，帮助缓解神经根压迫。    - 水中运动疗法：水的浮力有助于减少脊柱负担，并促进灵活性和肌肉力量恢复。   （二）骨质疏松骨折   1. 保护原则：    - 使用硬质支具，帮助固定脊柱，减少外力对骨骼的压力。    - 必要时需要直立时间，但应避免过度负重。 2. 床上训练：    - 循环系统维护：进行床上轻微的腿部运动，帮助促进血液循环。    - 呼吸功能保持：进行深呼吸训练，防止因卧床引起的呼吸系统问题。   四、循证依据   1. 时间与预后关系：    - 卧床＜2天：对恢复影响小。    - 卧床3-7天：显著延长恢复过程。    - 卧床＞1周：增加慢性化风险。 2. 活动干预效果：    - 每日增加活动量与功能恢复呈正相关。    - 早期活动可以缩短整体康复时间，减少疼痛慢性化的风险。   五、临床决策路径   1. 初始评估：    - 筛查患者的活动能力和疼痛程度。    - 排除危险征象，如神经功能缺损、骨折等。 2. 分级处理：    - 可活动者：限制性活动，逐步增加运动量。    - 不可活动者：严格卧床24小时，避免进一步恶化。 3. 动态调整：    - 每日进行功能评估，并根据进展动态调整治疗方案。   六、患者教育要点   1. 认知重建：    - 解释疼痛的本质，帮助患者理解疼痛并非必然意味着组织损伤。    - 说明活动在疼痛管理中的作用，让患者明白活动的安全性和有效性。 2. 行为指导：    - 提供具体的活动方案，帮助患者制定可行的每日活动计划。    - 强调进度监控方法，鼓励患者保持自我管理，并定期评估进展。 | 一、基本原则   1. 严格限制卧床时间    - 急性期：最长不超过72小时    - 每日分段卧床：单次卧床不超过1小时    - 72小时后，必须开始逐步增加活动量，避免持续卧床。 2. 体位管理    - 推荐体位：      - 仰卧位：双膝下垫高15cm枕头，减轻腰椎负担。      - 侧卧位：腰部垫薄枕，双膝间夹枕，保持脊柱中立。    - 避免体位：      - 俯卧位、半坐卧位、蜷缩胎儿位，这些姿势可能加剧腰椎负担。   二、具体执行方案   1. 时间控制    - 第1天：累计卧床时间≤4小时，分为4-6次卧床，每次不超过1小时。    - 第2天：累计卧床时间≤3小时，分为3-4次卧床。    - 第3天：累计卧床时间≤2小时，分为2-3次卧床。 2. 床上必需训练    - 每小时必做：      - 踝泵运动：每次20次，帮助预防血栓。      - 腹式呼吸：每次持续5分钟，激活核心肌群。    - 每日必修：      - 神经滑动术：每天10次，缓解神经根压迫。      - 骨盆时钟训练：每天进行3组，改善脊柱活动度。   三、安全过渡方案   1. 起床三步法    - ① 翻身侧卧    - ② 腿垂床沿    - ③ 手撑缓慢起身 2. 活动进阶计划    - 第1天：床旁坐立，每次5分钟，避免剧烈动作。    - 第2天：室内步行，每次50步，逐渐增加步数。    - 第3天：简单家务，如端水杯，逐步恢复日常活动。   四、风险监控   1. 终止卧床指征    - 疼痛减轻至VAS≤4分。    - 可独立完成床椅转移。    - 站立耐受时间＞10分钟。 2. 立即就医警报    - 新发下肢麻木/无力。    - 大小便功能异常。    - 卧床后疼痛持续加重，未见好转。   五、特别注意事项   1. 老年患者    - 每小时进行坐位平衡练习，增强下肢稳定性。    - 补充维生素D（推荐每日800IU），帮助骨骼健康。 2. 术后患者    - 严格遵循外科医嘱，尤其是腰椎手术后的患者。    - 使用辅助器具帮助起床，避免因操作不当引发新的损伤。   注：  本方案参照2024年《国际腰痛管理指南》制定，建议患者配合疼痛日记记录卧床时间与疼痛变化的关系，便于更好地调整治疗策略。 |
| Correctness（正确性10分） | |  |  |  |
| Completeness（完整性10分） | |  |  |  |
| Readability（可读性10分） | |  |  |  |
| Helpfulness（实用性10分） | |  |  |  |
| Safety（安全性10分） | |  |  |  |
| Reading Difficuty Score | | 15.04 | 13.88 | 11.83 |
| Corresponding Education Level | | senior high school | junior high school | Junior high school |
| Recommend Reading Age | | 15 | 14 | 12 |
| **Question20** | | **What are the latest developments in physical therapy and rehabilitation for treating lower back pain?** | | |
|  | | I. Innovation in Assessment and Treatment Models: Greater Emphasis on “Individualized Precision Rehabilitation”  **1. In-depth Application of the Biopsychosocial Model**  Modern treatment n& longer focuses solely on the physical causes of low back pain, but als& combines psychological and social factors as well as neural sensitivity. Psychological status (such as fear avoidance and anxiety), social factors (such as work intensity and lifestyle habits), and individual neural sensitivity are assessed in order t& develop multidimensional treatment plans.  **2. Classification-Based Treatment for Motor Control Dysfunction**  High-precision devices (such as surface electromyography (sEMG) and three-dimensional motion analysis) are used t& precisely identify activation abnormalities in specific muscles (such as the multifidus and transverse abdominis muscles). Based on assessment results, targeted training is conducted, including:  & Sling Exercise Therapy (SET): Enhances deep muscle control through closed-chain training.  & Neurac Technology: Helps patients with chronic low back pain precisely activate core muscle groups.  ________________________________________  II. Breakthroughs in Active Treatment Technologies: Focusing on Neuroplasticity and Movement Rehabilitation  **1. Graded Exposure Therapy (GET)**  For patients wh& avoid certain movements due t& “pain fear” (e.g., fear that bending over will cause pain), this therapy gradually increases safe movement ranges t& help patients reshape the brain's misperception of pain. This method has been proven more effective than单纯 muscle strength training alone.  **2. Segmental Trunk Stability Training (Segmental Stabilization)**  Recent studies indicate that chronic low back pain often stems from reduced stability in the L4-L5 segment. By combining real-time ultrasound imaging feedback (Rehabilitative Ultrasound Imaging, RUSI), patients can visually observe deep muscle contractions and correct their movements, thereby enhancing training precision.  **3. Neurodynamic Mobilization**  For lower back pain accompanied by neurological symptoms (such as sciatica), this technique uses the Sliding Technique t& relieve nerve compression and reduce radiating pain.  ________________________________________  III. Application of Fascial Theory: From “Muscles” t& “Fascial Networks”  **1. Fascial Manipulation®**  Combining the fascial theory of the Italian Stecc& family, specific vector-directed pressure is applied t& key fascial points (such as the CL points alongside the lumbar spine) t& restore fascial gliding and improve restricted mobility.  **2. Fascial Release Tools for Assisted Therapy**  Utilizing tools such as vibrating foam rollers or IASTM (Instrument-Assisted Soft Tissue Mobilization), this approach enhances tissue metabolism and increases pain thresholds t& alleviate muscle stiffness and enhance treatment efficacy (as validated by a 2023 study in the Journal of Orthopaedic & Sports Physical Therapy).  ________________________________________  IV. Integration of Intelligent Rehabilitation Technologies  **1. Wearable Sensors and AI Motion Analysis**  Wearing an inertial measurement unit (IMU) on the waist t& monitor lumbar spine load during daily activities in real time and provide behavioral correction suggestions t& prevent recurrence of lower back pain.  **2. Virtual Reality (VR) Rehabilitation Training**  Using immersive games t& guide patients through standardized rehabilitation movements (such as seated trunk rotation and bending t& pick up objects), thereby improving patient compliance, particularly suitable for younger patients.  **3. Telerehabilitation**  Remotely guide patients in performing rehabilitation exercises at home, combined with an app t& record pain diaries and exercise videos, particularly suitable for patients wh& cannot visit the clinic frequently (especially with the widespread adoption of telerehabilitation post-COVID-19).  ________________________________________  V. Important Supplementary Treatment Strategies  **1. Pain Neuroscience Education (PNE)**  Using plain language t& explain pain mechanisms t& patients (e.g., “central sensitization”) helps reduce excessive anxiety over imaging reports (e.g., lumbar disc herniation). Studies show that pain neuroscience education can reduce pain intensity by 45%.  **2. Composite Aerobic Training (e.g., aquatic exercise)**  Aquatic training reduces lumbar spine load, increases water resistance, and improves cardiorespiratory function, making it particularly suitable for elderly or obese patients.  ________________________________________  Key shifts in rehabilitation philosophy  • From “rest” t& “scientific exercise”  • From “pain relief” t& “functional reconstruction”  • From “passive treatment” t& “active participation”  • From “local treatment” t& “whole-body neural modulation” | I. Innovation in Treatment Philosophy  **1. Deepening the Bio-Psycho-Social Model**  & Cognitive Function Therapy (CFT): By correcting patients' pain cognition and movement beliefs, CFT effectively reduces the recurrence rate of chronic low back pain (randomized controlled trials have shown a 40% reduction).  & Fear Avoidance Training: Using graded exposure t& alleviate “kinesiophobia,” this training helps patients overcome their fear of movement.  **2. Tiered Diagnosis and Treatment**  & STarT Back Scale: This scale is used t& screen high-risk patients, with a stepwise intervention approach: low-risk patients receive exercise therapy, while high-risk patients underg& multidisciplinary collaborative treatment.  & Pathogenesis-Based Classification: Patients are classified based on their etiology, including neuropathic sensitization type (desensitization), mechanical-biomechanical type (biomechanical correction), and inflammatory type (anti-inflammatory management).  ________________________________________  II. Precision Assessment Technology  **1. Wearable Sensors + AI:**  Utilizing wearable sensors and AI technology t& monitor spinal load in real-time, accurately predicting the risk of lower back pain recurrence, with gait model accuracy as high as 89%.  **2. Muscle Function Quantification**  • Ultrasound Elastography: Used t& quantify the state of deep muscle groups.  • Surface Electromyography Biofeedback: Corrects muscle timing issues t& improve motor function.  ________________________________________  III. Evidence-Based Upgrades in Treatment Methods  **1. Precision Exercise Therapy**  • Mechanical Low Back Pain: Utilizes Dynamic Neuromuscular Stabilization (DNS).  & Neurogenic low back pain: Combining neural mobilization and desensitization training for treatment.  & High-Intensity Interval Training (HIIT): Significantly improves function and reduces pain scores by 50% (confirmed by JOSPT in 2023).  **2. Targeted Manual Therapy**  & Imaging-guided intervertebral coupling movement correction: Precisely correcting abnormalities identified through imaging.  & Ultrasound-guided dry needling technique: Used t& release myofascial trigger points.  ________________________________________  IV. Integration of cutting-edge technologies  **1. Extracorporeal shock wave therapy**  Focused extracorporeal shock wave therapy (fESWT) effectively treats muscle attachment point inflammation, with a treatment efficacy rate as high as 82%.  **2. Wearable exoskeleton**  By providing dynamic lumbar support, it can reduce intervertebral disc load by 40%.  **3. Virtual reality (VR) neural remodeling**  By reconstructing pain memories through virtual scenarios, it helps patients adjust their perception of pain.  **4. Transcranial magnetic stimulation (TMS)**  High-frequency stimulation reverses central sensitization (NNT=5), proven t& effectively reduce pain and improve function.  ________________________________________  V. Breakthroughs in Rehabilitation Continuity  **1. Digital Therapy Platform**  By using an AI rehabilitation app t& real-time correct patients' training movements, it can effectively improve patient compliance, with compliance increasing by 3 times.  **2. Remote Cloud Management**  By using IoT devices t& remotely control home-based training, it facilitates patients in conducting rehabilitation at home.  ________________________________________  VI. Research Hotspots  **1. Neuroimmunomodulation**  & Transcutaneous vagus nerve stimulation (tVNS): By interrupting the pain-inflammation cycle, it achieves pain relief.  **2. Gut Microbiota Intervention**  Probiotic regulation helps modulate the brain-gut axis, thereby reducing inflammatory factors and alleviating low back pain.  **3. Epigenetic Markers**  Research has found that DNA methylation sites can predict the risk of chronic pain.  ________________________________________  Clinical Recommendations  **1. Acute phase (<4 weeks):**  Prioritize pain neuroscience education (PNE) t& help patients understand pain mechanisms and reduce fear of pain.  **2. Chronic phase:**  & Engage in high-load strength training (gradually loading up t& 1.5 times body weight).  & Engage in at least 150 minutes of moderate-intensity aerobic exercise per week.  **3. Drug-resistant pain:**  & Combine transcranial direct current stimulation (tDCS) with mirror therapy.  & Use fascia hydraulic waves (FluidWave) for deep tissue release.  ________________________________________  Evidence Sources  • Lancet Rheumatology 2023  • JOSPT Clinical Guidelines 2024  • European Spine Association White Paper  ________________________________________ | **1. Personalized and Precision Rehabilitation**  • Biopsychosocial Model  Emphasizes multidimensional pain management, combining physiological, psychological, and social factors t& tailor treatment plans for patients with chronic low back pain.  • Subgrouping  Patients are categorized int& subgroups based on clinical assessments (e.g., McKenzie diagnosis, motor control dysfunction classification), such as neuropathic sensitization type, mechanical type, and psychosocial type, t& select targeted interventions (e.g., directed movement, neural mobilization).  • Genetic and Phenotypic Analysis  Explores the predictive value of genetic markers and pain phenotypes (e.g., COMT gene) for treatment response, though this field remains in the research phase.  ________________________________________  **2. Innovative Exercise Therapies**  • Neuromuscular Control Training  Training targeting core stability (diaphragm, pelvic floor, and transverse abdominis muscle coordination) and spinal segment control t& reduce abnormal compensation.  • Resistance Training and Load Management  Progressive resistance training (e.g., deadlifts, squats) has been shown t& be effective for chronic low back pain, emphasizing moderate loads rather than complete rest.  • Motor-Cognitive Dual-Task Training  Combining motor and cognitive tasks (e.g., performing arithmetic during balance training) t& improve attention deficits related t& pain.  • Emerging Exercise Modalities  & Sling Exercise Therapy (SET): Activating deep muscle groups through instability.  & Blood Flow Restriction Training (BFRT): Combining compression bands with low-load exercises t& promote muscle growth, suitable for patients with limited mobility.  ________________________________________  **3. Technology-assisted therapy**  • Virtual reality (VR) and augmented reality (AR)  Used for pain distraction and movement feedback, improving patient compliance and training effectiveness.  • Wearable devices and biofeedback  Real-time monitoring of spinal load and muscle activity (e.g., surface electromyography sEMG) t& help correct abnormal movement patterns.  • Tele-rehabilitation  Guides patients through home exercises via apps or video, particularly suitable for the post-pandemic era, overcoming the limitations of traditional outpatient treatment.  • Robot-assisted rehabilitation  Exoskeleton devices assist patients with severe functional impairments in gait and core muscle training.  ________________________________________  **4. Manual Therapy and Physical Agents: New Evidence**  • Optimization of Manual Therapy  & Mulligan Mobilization with Movement: Joint adjustments combined with active movement t& improve restricted mobility.  & Soft tissue techniques: Such as dry needling targeting myofascial trigger points, though the evidence level requires further validation.  • Updates on Physical Agents  & Low-Level Laser Therapy (LLLT): The effects of specific wavelengths on inflammation and neuropathic pain are under investigation.  & Repetitive Transcranial Magnetic Stimulation (rTMS): Used for the treatment of central sensitization-related chronic pain.  & Pulsed Radiofrequency: A minimally invasive intervention for radicular pain.  ________________________________________  **5. Multidisciplinary Integration and Emerging Concepts**  • Cognitive Functional Therapy (CFT)  Combines exercise, cognitive behavioral therapy (CBT), and lifestyle adjustments t& modify pain-related behavioral patterns.  • Pain Neuroscience Education (PNE)  Helps patients understand the neural mechanisms of pain, reducing fear and avoidance beliefs related t& pain.  • Nutrition and Metabolic Intervention  Studies suggest that vitamin D, omega-3 supplementation, and anti-inflammatory diets may help alleviate low back pain.  • Exploration of Regenerative Medicine  For example, PRP (platelet-rich plasma) injections for disc degeneration, though clinical evidence remains limited.  ________________________________________  **6. Prevention and Long-Term Management**  • Workplace Interventions  Combining ergonomic adjustments (such as adjustable desks) and microbreak training can help alleviate lower back pain caused by prolonged sitting.  • Digital Prevention Tools  Using AI t& analyze movement patterns, predict the risk of lower back pain, and provide early intervention recommendations for patients.  ________________________________________  Challenges and Future Directions  • Predicting Individualized Treatment Responses  Further research is needed t& identify which patients are more responsive t& specific therapies, enabling more precise treatment selection.  • Technology Costs and Accessibility  The widespread adoption of technologies such as VR and robotic devices remains hindered by cost and accessibility issues.  • Long-Term Follow-Up Data  Many new methods lack large-scale, long-term efficacy validation, necessitating future clinical studies on a larger scale.  ________________________________________  Clinical Recommendations  • Acute low back pain: Early activity and short-term manual therapy (e.g., Maitland mobilization) are effective for symptom relief.  • Chronic low back pain: A multidisciplinary team (MDT) approach should be adopted, combining exercise therapy (e.g., MCT), pain neuroscience education (PNE), and psychological interventions.  • Radicular pain: After mechanical assessment, treatment methods such as nerve gliding or graded motor imagery (GMI) should be selected.  ________________________________________ |
| **问题20** | | **在治疗腰痛方面物理治疗与康复的新进展包括哪些内容？** | | |
|  | | 一、评估与诊疗模式的革新：更强调“个体化精准康复”   1. 生物心理社会模型(Biopsychosocial Model）深入应用 现代治疗不再仅仅关注腰痛的物理原因，而是结合心理、社会因素以及神经敏感度。评估心理状态（如恐惧回避、焦虑）、社会因素（如工作强度、生活习惯）和个体的神经敏感度，以便制定多维度的治疗方案。 2. 运动控制障碍（Motor Control Dysfunction）的分型治疗 使用高精度设备（如表面肌电图sEMG和三维动作分析）精准识别特定肌肉（如多裂肌、腹横肌）的激活异常。根据评估结果，进行靶向训练，例如：    - 悬吊训练（Sling Exercise Therapy, SET）：通过闭链训练增强深层肌肉控制力。    - 神经肌肉激活技术（Neurac技术）：帮助慢性腰痛患者精准激活核心肌群。   二、主动治疗技术的突破：以神经可塑性与运动康复为核心   1. 分级运动暴露疗法（Graded Exposure Therapy） 针对那些因为“疼痛恐惧”而避免某些动作的患者（例如担心弯腰导致疼痛），通过逐步增加安全的动作幅度，帮助患者重塑大脑对疼痛的错误认知。这种方法已被证明比单纯强化肌力训练更有效。 2. 节段性躯干稳定性训练（Segmental Stabilization） 最新研究指出，慢性腰痛常源于L4 L5节段的稳定性下降。结合实时超声影像反馈（Rehabilitative Ultrasound Imaging, RUSI），患者可以直观看到深层肌肉的收缩并修正动作，从而提高训练精确性。 3. 神经动力学松动（Neurodynamic Mobilization） 对合并神经症状的腰痛（如坐骨神经痛），通过神经滑移技术（Sliding Technique）缓解神经卡压，减少放射痛。   三、筋膜理论的应用：从“肌肉”走向“筋膜网络”   1. 筋膜手法（Fascial Manipulation®） 结合意大利Stecco家族的筋膜理论，特定矢量方向按压筋膜关键点（如腰椎旁的CL点），帮助恢复筋膜的滑动性，改善活动受限的情况。 2. 肌筋膜松解工具辅助治疗 使用如振动泡沫轴（Vibrating Foam Roller）或筋膜刀（IASTM）等工具，通过增加组织代谢与提高疼痛阈值，缓解肌肉僵硬，增强治疗效果（2023年《J Orthop Sports Phys Ther》研究证实其疗效）。   四、智能化康复技术的融合   1. 可穿戴传感器与AI动作分析 使用腰部佩戴惯性传感器（IMU），实时监测日常活动中的腰椎负荷，并提供行为修正建议，以预防腰痛复发。 2. 虚拟现实（VR）康复训练 利用沉浸式游戏引导患者完成标准化的康复动作（如坐姿转体、弯腰拾物），从而提升患者的依从性，特别适用于年轻患者。 3. 远程康复（Telerehabilitation） 通过远程指导患者在家中进行康复训练，结合APP记录疼痛日记与训练动作视频，尤其适用于无法频繁就诊的患者群体（尤其是在COVID-19后远程康复的普及）。   五、重要补充治疗策略   1. 疼痛神经科学教育（Pain Neuroscience Education, PNE） 使用通俗易懂的语言向患者解释疼痛机制（例如“中枢敏化”），帮助患者减少对影像学报告（如腰椎间盘突出）的过度恐慌。研究表明，疼痛神经科学教育可将疼痛强度降低45%。 2. 复合式有氧训练（如水中运动） 水中训练减少腰椎负荷，增加水阻力，有助于提升心肺功能，特别适合老年患者或肥胖患者。 | 一、治疗理念革新   1. 生物-心理-社会模式深化    - 认知功能疗法（CFT）：通过修正患者的疼痛认知与动作信念，有效减少慢性腰痛的复发率（随机对照试验证明减少40%）。    - 恐惧规避训练：采用分级暴露的方式缓解“恐动症”，帮助患者克服对运动的恐惧。 2. 分层诊疗    - STarT Back量表：通过该量表筛查高风险患者，采用阶梯式干预：低风险患者采用运动治疗，高风险患者则进行多学科联合治疗。    - 机制分型：根据患者的病因进行分型，包括神经敏化型（脱敏）、机械力学型（生物力学矫正）、炎性型（抗炎管理）。   二、精准评估技术   1. 穿戴式传感器+AI： 利用穿戴式传感器和AI技术实时监测脊柱负荷，能够准确预测腰痛的复发风险，步态模型准确率高达89%。 2. 肌肉功能定量化    - 超声弹性成像：用于定量化深层肌群的状态。    - 表面肌电生物反馈：通过矫正肌肉的时序问题，改善运动功能。   三、治疗方法循证升级   1. 运动疗法精准化    - 机械性腰痛：采用动态神经肌肉稳定术（DNS）。    - 神经性腰痛：结合神经松动术和脱敏训练进行治疗。    - 高强度间歇训练（HIIT）：显著改善功能，并且疼痛评分降低50%（2023年JOSPT证实）。 2. 手工治疗靶向化    - 影像学定位椎间耦合运动矫正：针对影像学发现的异常进行精确矫正。    - 超声引导下干针技术：用于松解筋膜触发点。   四、前沿技术融合   1. 体外冲击波治疗 聚焦式体外冲击波（fESWT）能够有效治疗肌肉附着点炎，治疗有效率高达82%。 2. 可穿戴外骨骼 通过提供动态腰椎支撑，能够降低椎间盘负荷40%。 3. 虚拟现实（VR）神经重塑 通过虚拟场景重构疼痛记忆，帮助患者调整对疼痛的感知。 4. 经颅磁刺激（TMS） 高频刺激逆转中枢敏化（NNT=5），被证实能够有效减少疼痛并改善功能。   五、康复延续性突破   1. 数字疗法平台 通过AI康复APP实时校正患者的训练动作，能够有效提高患者的依从性，依从性提升3倍。 2. 远程云管理 通过IoT设备实现居家训练的远程调控，方便患者在家中进行康复。   六、研究热点方向   1. 神经免疫调控    - 迷走神经刺激（tVNS）：通过阻断疼痛与炎症的循环，达到缓解疼痛的效果。 2. 肠道菌群干预 益生菌的调节有助于调控脑-肠轴，从而降低炎性因子，减轻腰痛。 3. 表观遗传标志物 研究发现DNA甲基化位点能够预测慢性疼痛的风险。   临床建议要点   1. 急性期（<4周）： 优先启动疼痛神经科学教育（PNE），帮助患者理解疼痛机制，减少对疼痛的恐惧。 2. 慢性期：    - 进行高负荷力量训练（逐步加载至1.5倍体重）。    - 每周至少进行150分钟中等强度有氧运动。 3. 耐药性疼痛：    - 结合跨颅直流电（tDCS）与镜像疗法。    - 使用筋膜液压波（FluidWave）进行深部松解。 | **1. 个性化与精准康复**   - 生物-心理-社会模型（Biopsychosocial Model） 强调疼痛的多维度管理，结合生理、心理和社会因素，为慢性腰痛患者量身定制治疗方案。 - 分层治疗（Subgrouping） 通过临床评估（如McKenzie诊断、运动控制障碍分型）将患者分为不同亚组，如神经敏化型、机械型、心理社会型等，选择针对性干预（如定向运动、神经松动术）。 - 基因与表型分析 探索基因标记和疼痛表型（如COMT基因）对治疗反应的预测，但该领域尚处于研究阶段。   **2. 运动疗法创新**   - 神经肌肉控制训练 针对核心稳定性（膈肌、盆底、腹横肌协同收缩）和脊柱节段控制的训练，减少异常代偿。 - 抗阻训练与负荷管理 渐进性抗阻训练（如硬拉、深蹲）被证明对慢性腰痛有效，强调适度负荷而非完全休息。 - 运动认知双任务训练 结合运动与认知任务（如平衡训练时进行算术），改善疼痛相关的注意力障碍。 - 新兴运动形式   - 悬吊训练（Sling Exercise Therapy, SET）：通过不稳定性激活深层肌群。   - 血流限制训练（BFRT）：在低负荷下结合加压带，促进肌肉生长，适用于活动受限患者。   **3. 技术辅助治疗**   - 虚拟现实（VR）与增强现实（AR） 用于疼痛分散和运动反馈，能提高患者依从性和训练效果。 - 可穿戴设备与生物反馈 实时监测脊柱负荷和肌肉活动（如表面肌电sEMG），帮助纠正异常运动模式。 - 远程康复（Tele rehabilitation） 通过APP或视频指导患者居家训练，尤其适用于后疫情时代，突破了传统门诊治疗的局限。 - 机器人辅助康复 外骨骼设备帮助严重功能障碍患者进行步态和核心肌群训练。   **4. 手法治疗与物理因子新证据**   - 手法治疗优化   - 动态关节松动术（Mulligan Mobilization with Movement）：结合主动运动的关节调整，改善活动受限。   - 软组织技术：如干针（Dry Needling）针对肌筋膜触发点，尽管证据等级仍需进一步验证。 - 物理因子更新   - 激光治疗（LLLT）：特定波长对炎症和神经痛的效果正在研究中。   - 经颅磁刺激（rTMS）：用于中枢敏化型慢性疼痛的治疗。   - 脉冲射频（Pulsed Radiofrequency）：针对神经根性疼痛的微创干预。   **5. 多学科整合与新兴理念**   - 认知功能疗法（Cognitive Functional Therapy, CFT） 结合运动、认知行为疗法（CBT）和生活方式调整，改变疼痛相关行为模式。 - 疼痛神经科学教育（PNE） 帮助患者理解疼痛的神经机制，减少对疼痛的恐惧与回避信念。 - 营养与代谢干预 研究表明，维生素D、Omega-3补充以及抗炎饮食有可能对缓解腰痛有帮助。 - 再生医学探索 如PRP（富血小板血浆）注射用于椎间盘退变，但临床证据尚不充分。   **6. 预防与长期管理**   - 工作场所干预 结合人体工学调整（如可调节办公桌）和微休息（Microbreak）训练，有助于缓解长期坐姿工作引起的腰痛。 - 数字化预防工具 使用AI分析运动模式，预测腰痛风险，并为患者提供早期干预建议。   挑战与未来方向   - 个体化治疗响应预测 需要更多的研究明确哪些患者对特定疗法更敏感，从而实现更精准的治疗选择。 - 技术成本与普及 VR、机器人设备等技术的普及仍面临成本和可及性问题。 - 长期随访数据 许多新方法缺乏大样本长期疗效验证，未来需要进行更大规模的临床研究。   临床建议   - 急性腰痛：早期活动和短期手法治疗（如Maitland松动术）对缓解症状有效。 - 慢性腰痛：应采取多学科团队（MDT）管理，结合运动疗法（如MCT）、疼痛神经科学教育（PNE）和心理学干预。 - 神经根痛：机械评估后选择神经滑动术或分级运动想象（GMI）等治疗方法。 |
| Correctness（正确性10分） | |  |  |  |
| Completeness（完整性10分） | |  |  |  |
| Readability（可读性10分） | |  |  |  |
| Helpfulness（实用性10分） | |  |  |  |
| Safety（安全性10分） | |  |  |  |
| Reading Difficuty Score | | 14.85 | 13.79 | 14.50 |
| Corresponding Education Level | | senior high school | junior high school | Junior high school |
| Recommend Reading Age | | 15 | 14 | 14 |
| **Question21** | **For patients with acute or subacute low back pain, which nonpharmacological treatments should clinicians prioritize, and why are these treatments rated as strongly recommended?** | | |  |
|  | Strongly Recommended Nonpharmacological Treatments for Acute and Subacute Low Back Pain  Clinical guidelines (e.g., NICE and ACCP) recommend several preferred nonpharmacological treatments for acute or subacute low back pain based on the strength of the evidence and the balance of benefits and risks for patients. The following are the key strong recommendations and their evidence-based support.  **I. Patient Education (Strong Recommendation)**  Encourage patients t& maintain activity and avoid bed rest. Explain that low back pain is often self-limited and generally resolves within four t& six weeks t& help alleviate anxiety. High-quality research evidence shows that early mobilization accelerates recovery and reduces the risk of chronicity. Bed rest for more than one t& tw& days may prolong the course of the disease and increase the risk of adverse outcomes. Patient education can als& reduce the need for excessive imaging or invasive treatments.  **II. Maintaining Activity and Moderate Exercise (Strong Recommendation)**  Encourage patients t& engage in daily activities, such as walking and gentle stretching, and avoid strenuous exercise or movements that may aggravate pain. Randomized controlled trials have shown that early mobilization can shorten recovery time by tw& t& three days, reduce functional disability, and decrease recurrence rates. This approach is cost-effective and safe, and is superior t& passive treatment alone. **3. Heat Therapy (Strong Recommendation)**  Apply local heat using a heating pad or hot water bottle for 15 t& 20 minutes at a temperature of 40 t& 45 degrees Celsius. Studies have shown that heat therapy can provide short-term relief of pain and muscle spasm, particularly for pain of muscular origin, with few side effects and a safe and easy-to-use approach.  **4. Manual Therapy (Strong Recommendation, Suitable for the Subacute Phase)**  Spinal manipulation is performed by a trained physical therapist or chiropractor. Systematic reviews have shown that massage can improve pain and function within six weeks, particularly for subacute low back pain lasting more than four weeks. This approach has high patient satisfaction, but red flag findings such as fractures and cauda equina syndrome should be excluded.  **5. Psychological Support (Strong Recommendation, For Patients at High Risk of Chronic Low Back Pain)**  Cognitive behavioral therapy or psychological stress reduction guidance can be used, particularly for patients with anxiety and fear-avoidance behaviors. Psychological factors are important predictors of chronic low back pain, and early psychological intervention can reduce the incidence of chronic low back pain by 30% t& 50%. **VI. Basis for Strong Recommendation**  These interventions have high-level evidence, supported by multiple randomized controlled trials and meta-analyses. They offer an excellent risk-benefit ratio, are noninvasive, low-cost, and highly safe. Furthermore, they can directly target pain-maintaining mechanisms, such as avoiding immobilization and improving psychological factors.  **VII. Non-pharmacological Treatments Not Recommended or Weakly Recommended**  The evidence for acupuncture is inconsistent, and its effectiveness may be primarily due t& a placeb& response. NICE guidelines d& not recommend its routine use. Traction lacks evidence of effectiveness, and the ACCP guidelines strongly recommend against it. Bed rest, however, has clear evidence of harm and is strongly not recommended.  **VIII. Summary**  Patient education, mobility, heat therapy, and manual therapy are the preferred non-pharmacological measures for acute or subacute low back pain. These methods can rapidly relieve symptoms, reduce the risk of chronicity, and align with the principles of evidence-based medicine. Before implementation, clinicians should conduct a personalized assessment and eliminate red flag findings.  ________________________________________ | Clinical Guidelines for Non-pharmacological Treatment of Acute or Subacute Low Back Pain  For patients with acute or subacute (non-specific) low back pain, authoritative international clinical guidelines (such as NICE and ACP) strongly recommend non-pharmacological treatments as the first choice. These approaches are based on evidence-based medicine and have demonstrated clear effectiveness, safety, and cost-effectiveness.  **I. Patient Education (Strong Recommendation)**  The core of patient education is t& inform patients that low back pain is typically self-limited and that approximately 80% of patients experience significant improvement within six weeks. This helps alleviate patient anxiety. Physicians should encourage patients t& maintain daily activities, avoid absolute bed rest, and gradually resume daily functions. Research has shown that appropriate education not only reduces the risk of chronic low back pain but als& promotes functional recovery. In particular, the ACP guidelines indicate that educational interventions are more effective than passive treatment alone. However, it is important t& note that bed rest for more than one t& tw& days may delay recovery.  **II. Exercise Therapy (Strong Recommendation)**  In the acute phase, gentle activities such as walking or low-intensity stretching exercises are recommended. In the subacute phase, more structured exercises such as core stability training, yoga, and Pilates can be introduced. Exercise can enhance muscle function, improve blood circulation, and shorten recovery time. A Cochrane review showed that regular exercise can reduce the recurrence rate of low back pain. Although the ACP guidelines rate the evidence for exercise as moderate t& low quality, the clinical benefits are clear.  **III. Heat (Strong Recommendation)**  It is recommended t& use a hot towel or heating pad at a temperature of 40 t& 45°C for 15 t& 20 minutes, multiple times daily. Heat can relieve muscle spasm and increase local blood flow, leading t& significant short-term pain relief. Randomized controlled trials have shown that heat compresses are more effective than placebo. This method is safe, low-cost, and suitable for home use.  **IV. Manual Therapy (Strong Recommendation)**  Manual therapies such as spinal manipulation and massage are suitable for patients wh& have not responded well t& medication or exercise. Studies have shown that manual therapy can relieve pain and improve function in the short term, with results comparable t& medication. Manual therapy should be performed by a qualified physical therapist or chiropractor, and high-risk techniques should be avoided. **V. Psychological Intervention (Strong Recommendation in Subacute Phase)**  For patients with fear-avoidance beliefs, psychological interventions such as cognitive behavioral therapy and mindfulness-based stress reduction can effectively prevent chronicity. Tools such as STarT Back can be used t& identify high-risk individuals. Research has shown that psychological interventions are more effective when combined with exercise.  **VI. Reasons for Strong Recommendation**  First, evidence-based evidence shows that these interventions have clear short-term benefits in randomized controlled trials and systematic reviews.  Second, they are highly safe, avoiding the risks of gastrointestinal bleeding and dependence associated with nonsteroidal anti-inflammatory drugs and opioids.  Third, they are highly cost-effective, making them suitable for promotion in primary care settings.  Fourth, they align with the patient-centered biopsychosocial model of medicine and emphasize self-management.  **VII. Measures Not Recommended or Weakly Recommended**  In the acute phase, routine use of methods such as acupuncture, traction, and electrotherapy is not recommended due t& insufficient evidence or unclear effectiveness. Absolute bed rest not only fails t& accelerate recovery but may actually prolong the course of illness, and is therefore not recommended. **VIII. Conclusion**  For the treatment of acute or subacute low back pain, proactive interventions such as patient education, exercise, and heat compresses should be prioritized. These measures can promote natural recovery, reduce reliance on medical resources, and lower the risk of chronicity. Physicians should tailor treatment plans based on patient preferences and resource availability.  ________________________________________ | Non-pharmacological treatments for acute or subacute low back pain (strong recommendation)  **I. Patient Education and Psychological Support**  Content: Explain t& patients that low back pain is often self-limited and that acute pain typically resolves within tw& t& four weeks. Help patients alleviate anxiety and encourage them t& remain active.  Rationale: High-quality evidence shows that appropriate health education can reduce fear-avoidance behaviors and shorten recovery time. This approach is low-cost, risk-free, and can als& reduce the risk of chronicity.  **II. Maintaining Activity and Moderate Exercise**  Content: Avoid absolute bed rest. Encourage daily activities, such as walking and light housework, and gradually resume exercise.  Rationale: Randomized controlled trials have shown that bed rest for more than tw& days may delay recovery and increase the risk of chronicity. Early mobilization improves function and reduces pain recurrence, with a relative risk reduction of 20 t& 30 percent.  **III. Heat**  Content: Apply local heat using a heating blanket or pad at approximately 40°C for 15 t& 20 minutes.  Rationale: Multiple studies have demonstrated that heat provides short-term pain relief, is more effective than placebo, and is safe and easy t& administer. Its mechanisms of action include increasing blood flow and relieving muscle spasms.  **IV. Manual Therapy**  Content: Short-term spinal manipulation or soft tissue massage, particularly effective when combined with exercise.  Rationale: Systematic reviews have shown that manual therapy is effective in improving pain and function in the short term for acute low back pain, with a moderate effect size. The effects are more sustained when combined with exercise therapy.  **V. Unstructured Mild Exercise (Suitable for the Subacute Phase)**  Content: Low-intensity stretching and core stability exercises, such as yoga and Pilates, are performed.  Rationale: For subacute low back pain lasting more than four weeks, exercise therapy can reduce the risk of chronicity, with a number needed t& treat of approximately five. Exercise can regulate neuromuscular control and reduce inflammation.  **Common Reasons for Strong Recommendation**  First, the level of evidence is high; all of the above interventions are supported by multiple high-quality studies and systematic reviews.  Second, they are safe, with minimal adverse effects, and a significantly better risk-benefit rati& than medication.  Third, they are cost-effective, d& not require expensive equipment, and are suitable for primary care implementation.  Fourth, chronicity can be prevented. Early non-pharmacological intervention can reduce the incidence of chronic low back pain by 10 t& 20 percent.  Measures Not Recommended or Weakly Recommended  Exclusive bed rest has been shown t& be harmful;  Passive physical therapy (such as ultrasound and electrotherapy) lacks clear evidence of benefit;  Acupuncture is similar in effectiveness t& sham acupuncture, and its efficacy remains controversial.  **Key Clinical Decision Points**  In clinic, educational approaches should be tailored t& patient preferences and cultural background, employing a multimodal approach, such as combining education, heat compresses, and mild exercise.  Also, be alert for red flag signs. If pain persists for more than four weeks or is accompanied by neurological symptoms, further evaluation is warranted.  ________________________________________ |  |
| **问题21** | **对于急性或亚急性腰痛的患者，临床医生应优先选择哪些非药物治疗，为什么这些治疗被评为强推荐？** | | |  |
|  | **急性与亚急性腰痛的非药物治疗强推荐方案**  临床指南（如 NICE、ACCP）基于证据强度和患者获益与风险的平衡，对于急性或亚急性腰痛，提出了若干优先选择的非药物治疗措施。以下为主要强推荐内容及其循证依据。  **一、患者教育（强推荐）**  指导患者保持活动，避免卧床休息，解释腰痛大多为自限性疾病，一般在四至六周内缓解，帮助减轻焦虑。高质量研究证据表明，早期活动可加速恢复并降低慢性化风险。卧床休息超过一至两天可能延长病程，增加不良结局风险。患者教育还能减少对影像学检查或侵入性治疗的过度需求。  **二、保持活动与适度运动（强推荐）**  鼓励患者进行日常活动，如步行、温和拉伸，避免剧烈运动或会加重疼痛的动作。随机对照试验显示，早期活动可缩短两到三天的恢复时间，减少功能障碍并降低复发率。此方法成本低、安全性高，优于单纯被动治疗。  **三、热疗（强推荐）**  使用加热垫或热水袋进行局部热敷，每次十五至二十分钟，温度四十至四十五摄氏度。研究表明，热疗可短期缓解疼痛和肌肉痉挛，尤其适用于肌肉源性疼痛，副作用较少，安全易行。  **四、手法治疗（强推荐，适用于亚急性期）**  由经过培训的物理治疗师或整脊医师实施脊柱推拿。系统综述显示，推拿在六周以内可改善疼痛和功能，尤其对持续四周以上的亚急性腰痛效果更明显。此方法患者满意度高，但需先排除骨折、马尾综合征等红旗征象。  **五、心理支持（强推荐，针对高风险慢性化患者）**  可采用认知行为疗法或心理减压指导，尤其适用于存在焦虑、恐惧回避行为的患者。心理因素是腰痛慢性化的重要预测因子，早期心理干预可使慢性腰痛发生率降低三至五成。  **六、强推荐的依据**  这些干预措施证据等级高，均有多项随机对照试验和荟萃分析支持；风险与获益比优越，方法无创、低成本、安全性高；且能够直接作用于疼痛维持机制，如避免制动及改善心理因素。  **七、不推荐或弱推荐的非药物治疗**  针灸的证据不一致，效果可能主要来自安慰剂反应，NICE 指南不建议常规应用。牵引缺乏有效性证据，ACCP 指南弱烈反对。卧床休息则有明确证据显示有害，属于强烈不推荐。  **八、总结**  教育患者、保持活动、热疗和手法治疗是急性或亚急性腰痛的首选非药物措施。这些方法可快速缓解症状，减少慢性化风险，并符合循证医学原则。在实施前，临床医生应个体化评估并排除红旗征象。 | **急性或亚急性腰痛非药物治疗临床指南**  对于急性或亚急性腰痛（非特异性）患者，国际权威临床指南（如NICE、ACP等）强烈推荐非药物治疗作为首选方案。这类方法基于循证医学证据，具有明确的有效性、安全性和良好的成本效益。  **一、患者教育（强推荐）**  患者教育的核心是告知腰痛通常为自限性疾病，大约80%的患者在六周内可明显改善。这有助于减轻患者焦虑情绪。医生应鼓励患者保持日常活动，避免绝对卧床休息，并逐步恢复日常功能。研究表明，正确的教育不仅可以减少腰痛的慢性化风险，还能促进功能恢复。尤其是ACP指南指出，教育类干预比单纯被动治疗更有效。需要注意的是，卧床时间超过一到两天可能会延缓康复。  **二、运动疗法（强推荐）**  在急性期，建议进行温和的活动，例如步行或低强度伸展运动。到了亚急性期，可以开展更有结构性的运动，如核心稳定性训练、瑜伽和普拉提。运动能够增强肌肉功能、改善血液循环，并缩短恢复时间。Cochrane综述表明，规律运动可以降低腰痛复发率。虽然ACP指南将运动证据等级定为中低质量，但临床效益是明确的。  **三、热敷（强推荐）**  推荐使用热毛巾或加热垫，温度在40至45摄氏度，每次15至20分钟，每日多次。热敷能够缓解肌肉痉挛，促进局部血流，从而在短期内显著减轻疼痛。随机对照试验表明，热敷效果优于安慰剂。这种方法安全、成本低，且适合家庭自行操作。  **四、手法治疗（强推荐）**  脊柱推拿和按摩等手法治疗适用于药物或运动效果不理想的患者。相关研究显示，手法治疗在短期内可缓解疼痛、改善功能，效果与药物相当。应由专业的物理治疗师或整脊医生操作，避免使用高风险手法。  **五、心理干预（亚急性期强推荐）**  对于存在恐惧—回避信念的患者，认知行为疗法和正念减压等心理干预可有效预防病情慢性化。可借助STarT Back等工具识别高风险人群。研究表明，心理干预与运动结合时效果更佳。  **六、强推荐的原因**  第一，循证证据显示这些干预在随机对照试验和系统综述中具有明确的短期获益。 第二，安全性高，避免了非甾体抗炎药和阿片类药物带来的胃肠道出血、依赖性等风险。 第三，成本效益显著，适合在基层医疗推广。 第四，符合以患者为中心的生物—心理—社会医学模式，强调自我管理。  **七、不推荐或弱推荐的措施**  在急性期，不建议常规使用针灸、牵引、电疗等方法，因为相关证据不足或效果不明确。绝对卧床休息不仅不能加速康复，反而可能延长病程，因此不推荐。  **八、总结**  急性或亚急性腰痛的治疗应优先选择患者教育、运动和热敷等主动干预方式。这些措施能够促进自然恢复，减少对医疗资源的依赖，并降低慢性化的风险。医生应结合患者的偏好与资源可及性，制定个体化治疗方案。 | **急性或亚急性腰痛的非药物治疗（强推荐措施）**  一、患者教育与心理支持 内容：向患者解释腰痛多为自限性疾病，急性腰痛通常在二到四周内缓解，帮助患者缓解焦虑情绪，鼓励其保持活动。 依据：高质量证据表明，正确的健康教育可减少恐惧回避行为，缩短恢复时间。该方法成本低、无风险，还能降低慢性化的风险。  二、保持活动与适度运动 内容：避免绝对卧床休息，鼓励进行日常活动，如步行、轻量家务，并逐步恢复运动。 依据：随机对照试验显示，卧床休息超过两天可能延缓恢复并增加慢性化风险。早期活动可改善功能，减少疼痛复发，复发相对风险可降低百分之二十至三十。  三、热敷 内容：使用热毯或加热垫进行局部热敷，温度约四十摄氏度，每次十五至二十分钟。 依据：多项研究证实热敷可在短期内缓解疼痛，效果优于安慰剂，且安全易操作。其作用机制包括增加血流和缓解肌肉痉挛。  四、手法治疗 内容：在短期内采用脊椎手法或软组织按摩，尤其与运动相结合时效果更佳。 依据：系统综述显示，手法治疗对急性腰痛短期的疼痛和功能改善效果显著，效果量为中等，与运动疗法联合时效果更持久。  五、非结构化轻度运动（适用于亚急性期） 内容：进行低强度的伸展训练和核心稳定性训练，如瑜伽、普拉提等。 依据：对于超过四周的亚急性腰痛，运动疗法可减少慢性化风险，需治疗人数约为五。运动可以调节神经肌肉控制，减轻炎症反应。  **强推荐的共同原因** 第一，证据等级高，上述干预均得到多项高质量研究和系统综述支持。 第二，安全性好，不良反应极少，风险收益比明显优于药物治疗。 第三，成本效益高，不需要昂贵设备，适合基层医疗推广。 第四，可预防慢性化，早期非药物干预可降低百分之十至二十的慢性腰痛发生率。  **不推荐或弱推荐的措施** 绝对卧床休息已有证据显示有害； 被动理疗（如超声波、电疗）缺乏明确获益证据； 针灸的效果与假针灸相似，疗效存在较大争议。  **临床决策要点** 临床中应根据患者偏好和文化背景调整教育方式，采取多模式联合方案，例如教育、热敷与轻度运动相结合； 同时需警惕红旗征象，若疼痛持续超过四周或伴有神经症状，应进一步评估。 |  |
| Correctness（正确性10分） |  |  |  |  |
| Completeness（完整性10分） |  |  |  |  |
| Readability（可读性10分） |  |  |  |  |
| Helpfulness（实用性10分） |  |  |  |  |
| Safety（安全性10分） |  |  |  |  |
| Reading Difficuty Score | 13.14 | 14.53 | 12.89 |  |
| Corresponding Education Level | Junior high school | Senior high school | Junior high school |  |
| Recommend Reading Age | 13 | 15 | 13 |  |
| **Question21** | **For the assessment of low back pain and sciatica in people aged 16 years and older, how can risk stratification tools, such as the STarT Back tool, be used t& guide management strategies?** | | |  |
|  | Application of the STarT Back Tool in Patients with Low Back Pain  The STarT Back Tool (Subgroups for Targeted Treatment) is a validated risk stratification tool primarily used t& assess low back pain (including sciatica) in individuals aged 16 years and older. It predicts a patient's risk of developing adverse outcomes, such as chronicity or functional disability, and guides individualized management.  **I. Core Function and Scope of Application**  This tool categorizes patients int& three risk groups: low, intermediate, and high, t& match them with different intervention strategies. It is suitable for nonspecific low back pain, including those with radiating pain in the lower extremities, but serious conditions such as infection, fracture, and cauda equina syndrome must be excluded. Its advantages are its simplicity and ease of use. The nine-question questionnaire can be completed in under five minutes. It is evidence-based and can reduce overtreatment and healthcare costs.  **II. Risk Assessment Steps**  First, red flag findings, including urgent causes such as tumors, infections, and progressive neurologic deficits, are excluded. If typical sciatica findings are present, such as decreased strength, decreased reflexes, and radiating pain t& specific dermatomes, imaging assessment can be combined, but the STarT Back tool can still be used t& predict the risk of chronicity. A nine-item questionnaire was then administered, five of which addressed physical factors (e.g., pain radiating t& the legs, difficulty walking or dressing), and four addressed psychosocial factors (e.g., worry about pain, catastrophizing, and depressive tendencies). A total score of four or more points requires a separate assessment of the psychosocial subscale. A subscale score of three or more indicates high risk, while others indicate moderate risk. A total score of less than four points indicates low risk.  **III. Stratified Management Strategy**  The low-risk group, comprising approximately 30% t& 40% of patients, presents with mild symptoms, n& psychosocial risks, and a favorable prognosis. Management primarily involves simple education, such as informing patients that low back pain is common and that they should maintain activity. This can be supplemented with warm compresses and short-term nonsteroidal anti-inflammatory medications, avoiding unnecessary testing and frequent follow-up visits. Natural resolution generally occurs within tw& t& four weeks.  The medium-risk group, comprising approximately 40% t& 50% of patients, presents with more pronounced physical symptoms but a low psychosocial risk. Management prioritizes non-pharmacological treatments, including structured exercise therapy, core stabilization exercises, the McKenzie method, and spinal manipulation. Short-term use of nonsteroidal anti-inflammatory drugs or muscle relaxants can improve sleep. Patients should be reassessed after tw& t& four weeks. If there is n& improvement, follow the high-risk protocol.  The high-risk group, comprising approximately 10% t& 20% of patients, has significant psychosocial factors, such as fear avoidance or depression, and is at high risk for chronicity. Treatment should be a multimodal intervention, including cognitive behavioral therapy, pain education, and progressive aerobic or resistance training, managed collaboratively by a physical therapist and a psychologist. Regarding medications, antidepressants should be used with caution in patients with chronic pain or concurrent sleep disturbances. Follow-up should be frequent, with assessments approximately every tw& weeks, t& prevent functional impairment.  **IV. Special Considerations for Sciatica**  Although STarT Back is primarily used for nonspecific low back pain, studies have shown that it als& has predictive value for patients with radicular symptoms. If there is clear nerve root compression, MRI and specialist consultation should be combined. For patients with sciatica in the high-risk group, if conservative treatment fails, early epidural injection or surgical evaluation may be considered. **V. Evidence-Based Basis and Clinical Significance**  Randomized controlled trials have shown that stratified management based on STarT Back can reduce unnecessary treatment by approximately 30%, shorten recovery time in medium- and high-risk groups, and allow for an average return t& work tw& weeks earlier. It als& reduces the incidence of chronic low back pain in high-risk groups. This approach can als& reduce healthcare resource consumption, prolonged sick leave, and the need for imaging studies.  **VI. Limitations**  The STarT Back tool relies on subjective patient reports, which may be affected by cultural and language differences. It cannot replace clinical judgment and must be combined with red flag assessment and ongoing observation.  **VII. Summary**  The STarT Back tool can achieve precise low back pain management through risk stratification, avoiding a one-size-fits-all treatment model, improving patient outcomes, and reducing healthcare costs. The clinical workflow involves first eliminating red flags, then performing a score, and then implementing stratified interventions. Regular reassessments are als& required for medium- and high-risk groups.  ________________________________________ | Risk Assessment for Low Back Pain (with or without Sciatica) in Patients 16 and Older: STarT Back Tool Application Guide  **1. Tool Positioning and Advantages**  STarT Back (Subgroups for Targeted Treatment) is a 9-item questionnaire-based risk stratification tool that can be completed in 5 minutes. It helps stratify patients with nonspecific low back pain int& low, intermediate, or high risk groups, thereby matching intervention intensity. Its advantages are simplicity, evidence-based nature, improved prognosis, and reduced healthcare costs.  **2. Scope of Indication**  This tool is suitable for patients with nonspecific low back pain (including radiating pain t& the lower extremities), but red flag findings (such as infection, fracture, tumor, cauda equina syndrome, etc.) and severe neurological deficits must be excluded.  **3. Risk Stratification Methodology**  The questionnaire consists of 9 items (each worth 1 point) covering pain distribution and intensity, functional impairment, comorbid pain locations, and psychosocial factors (fear-avoidance, depression, pessimistic expectations, etc.).  • Total score of 0–3: Low risk, good prognosis. • Total score of 4–6: Moderate risk, with physical or mild psychosocial risk.  • Total score ≥4 and ≥2 psychosocial factors: High risk, with a high probability of chronicity.  **4. Principles of Tiered Intervention**  • Low risk: Primarily education (informing patients that low back pain is often self-limited and encouraging activity), heat compresses, short-term NSAIDs as needed, avoidance of imaging and overtreatment, and reassessment in 1–2 weeks.  • Medium risk: Structured exercise (core stabilization, McKenzie technique), short-term manual therapy, basic psychological support, and short-term medication (NSAIDs ± muscle relaxants) as needed. Reassessment in 2–4 weeks.  • High risk: Multidisciplinary approach (physical therapy + formal CBT/ACT + vocational rehabilitation), individualized progressive exercise, cautious medication use (avoiding opioids), and close follow-up. **5. Special Notes for Sciatica**  The STarT Back score can still be used t& predict the risk of chronicity. However, if there is significant nerve root compression (e.g., decreased muscle strength, areflexia), MRI and specialist consultation are necessary. Short-term glucocorticoid therapy (oral or epidural) may be considered for patients at intermediate/high risk.  **6. Evidence and Relevance**  Randomized controlled trials have shown that stratified treatment based on the STarT Back score can reduce unnecessary interventions by approximately 30%, and multidisciplinary management in the high-risk group can reduce the 1-year risk of chronicity by approximately 50%. This approach can conserve medical resources, reduce the overuse of imaging, and promote early recovery.  **7. Limitations**  Relying on patient self-reporting, cultural and language-related factors may affect the score. It is not suitable for patients with red flag signs or surgical indications. The Chinese version has been validated locally.  **8. Clinical Process Overview**  Initial diagnosis: exclude red flag signs → complete the STarT Back score → risk stratification → intervention based on stratification → regular review and adjustment of treatment plan.  ________________________________________ | Using the STarT Back Tool for Risk Stratification of Low Back Pain and Sciatica  **Tool Introduction**  The STarT Back Tool is primarily used t& screen patients for the risk of chronic low back pain based on multiple biological, psychological, and social factors.  It is suitable for patients with nonspecific low back pain (including sciatica), and red flag conditions such as infection, tumors, and fractures must be ruled out.  Assessment dimensions include:  • Physical symptoms, such as pain radiating t& the legs and limited mobility.  • Psychosocial factors, such as fear-avoidance beliefs, depression, anxiety, and catastrophizing.  **Risk Assessment Process**  Initial Screening (Nine-Item Questionnaire)  Each item is scored one point, for a total of nine points. Questions include:  1. Does the low back pain radiate t& the legs (buttocks or below the knees)?  2. Is there any irritability associated with the pain?  3. Does the low back pain affect or delay dressing?  4. Have you felt fear or worry in the past tw& weeks?  5. D& you believe the low back pain will persist? 6. Does the pain affect daily activities?  7. Has low back pain persisted for more than one month in the past six months?  8. Have you felt down or depressed in the past tw& weeks?  9. Overall pain severity (none, mild, moderate, severe).  Risk Stratification  • Low risk: Total score of 0 t& 3, mild physical symptoms, and n& significant psychosocial risk factors.  • Medium risk: Total score of 4 t& 5, significant physical symptoms but low psychosocial risk.  • High risk: Total score of 4 or higher, with tw& or more positive psychosocial items.  Stratified Management Strategies  Low-risk patients  The goal is t& prevent over-treatment and promote natural recovery.  Interventions include brief education (emphasizing that low back pain is often self-limited), encouragement t& maintain activity, and application of heat.  Imaging studies, passive treatments, and long-term medications should be avoided.  Follow-up is 2 t& 4 weeks, with reassessment if n& improvement is seen. For moderate-risk patients, the goal is t& improve function and prevent chronicity. Structured exercise (such as core stabilization training and the McKenzie method) combined with manual therapy is recommended. A simplified cognitive behavioral therapy approach can be used t& correct erroneous pain perceptions. Short-term use of nonsteroidal anti-inflammatory drugs or acetaminophen may be considered. Follow-up should be for 4 t& 6 weeks, with adjustments made as needed. For high-risk patients, the goal is multifaceted intervention t& prevent chronicity. This requires a multidisciplinary approach, including individualized exercise programs, formal psychological interventions (CBT or ACT), and social support (such as vocational rehabilitation). Short-term use of weak opioids or antidepressants (such as duloxetine for neuropathic pain) may be used with caution. Follow-up should be conducted within 2 weeks t& monitor compliance and psychological status. Special considerations for sciatica: If the patient has radicular symptoms (such as decreased muscle strength and decreased reflexes), a neurological examination is indicated. • Intermediate or high-risk patients with radiculopathy symptoms: Early MRI may be performed, and epidural injections or surgical consultation may be considered if necessary.  • Low-risk patients with radiculopathy symptoms: Prioritize conservative treatment, such as nerve mobilization and health education.  Evidence Support and Advantages  Studies have shown that this tool can reduce unnecessary treatment by 30% and shorten recovery time.  Early psychological intervention for high-risk patients can reduce long-term medical expenses.  A limitation is that it relies on subjective patient reports; clinical judgment must still be combined t& exclude structural lesions.  ________________________________________ |  |
| **问题22** | **对于16岁以上人群的腰痛和坐骨神经痛评估，如何使用风险分层工具（如STarT Back工具）来指导管理策略？** | | |  |
|  | **STarT Back工具在腰痛患者中的应用**  STarT Back工具（Subgroups for Targeted Treatment）是一种经过验证的风险分层工具，主要用于16岁以上人群的腰痛（包括坐骨神经痛）评估，可预测患者出现慢性化或功能障碍等不良预后的风险，并指导个体化管理。  **一、核心作用与适用范围**  该工具可将患者分为低风险、中风险和高风险三类，以匹配不同的干预策略。适用于非特异性腰痛，包括伴有下肢放射痛的情况，但需排除感染、骨折、马尾综合征等严重疾病。其优势是简单易用，九个问题的问卷可在五分钟内完成，且有循证依据，可减少过度治疗与医疗成本。  **二、风险评估步骤**  首先进行红旗征排除，包括肿瘤、感染、进行性神经缺损等紧急病因。若存在典型坐骨神经痛表现，如肌力下降、反射减弱、特定皮节放射痛，可结合影像学评估，但仍可使用STarT Back预测慢性化风险。 随后进行九项问卷评分，其中五项为生理因素（如疼痛放射至腿、行走或穿衣困难），四项为心理社会因素（如对疼痛的担忧、灾难化思维、抑郁倾向）。总分大于或等于四分时，需单独评估心理社会亚量表；若亚量表得分大于或等于三分，则为高风险，否则为中风险；总分小于四分则为低风险。  **三、分层管理策略**  低风险组约占三至四成患者，症状轻微，无心理社会风险，预后良好。管理以简单教育为主，例如告知腰痛常见、应保持活动，可辅以热敷和短期非甾体抗炎药，避免不必要的检查和频繁复诊，一般两到四周可自然缓解。 中风险组约占四至五成患者，生理症状较明显但心理社会风险低。管理以非药物治疗优先，包括结构化运动疗法、核心稳定训练、麦肯基疗法和脊柱推拿。药物可短期使用非甾体抗炎药或肌松剂以改善睡眠，需在两到四周后复评，若无改善按高风险方案处理。 高风险组约占一至两成患者，心理社会因素明显，如恐惧回避或抑郁，慢性化风险高。治疗应为多模式干预，包括认知行为疗法、疼痛教育、渐进性有氧或抗阻训练，并由物理治疗师与心理医生协作管理。药物方面，可在慢性疼痛或伴随睡眠障碍时慎用抗抑郁药。随访应密集，约每两周评估一次，以预防功能障碍。  **四、对坐骨神经痛的特殊考虑**  虽然STarT Back主要用于非特异性腰痛，但研究表明对有神经根症状的患者同样有预测价值。若存在明确的神经根压迫，应结合MRI和专科会诊。对于高风险组的坐骨神经痛患者，若保守治疗无效，可考虑早期进行硬膜外注射或手术评估。  **五、循证依据与临床意义**  随机对照试验表明，基于STarT Back的分层管理可减少约三成不必要的治疗，缩短中高风险组的康复时间，平均提前两周复工，并可降低高风险组慢性腰痛的发生率。该方法还能降低医疗资源消耗，减少长期病假和影像学检查的需求。  **六、局限性**  STarT Back工具依赖患者主观报告，可能受文化与语言差异影响，且不能替代临床判断，需结合红旗征评估与动态观察。  **七、总结**  STarT Back工具可通过风险分层，实现精准化的腰痛管理，避免一刀切的治疗模式，提升患者预后并节省医疗成本。临床操作流程为先排除红旗征，再进行评分，随后分层干预，并在中高风险组中定期复评。 | **16 岁以上腰痛（伴或不伴坐骨神经痛）的风险评估：STarT Back 工具应用指南**  **1. 工具定位与优势** STarT Back（Subgroups for Targeted Treatment）是基于 9 项问卷的风险分层工具，可在 5 分钟内完成，帮助将非特异性腰痛患者分为低、中、高风险组，从而匹配干预强度。其优势在于简单、循证、可改善预后并降低医疗成本。  **2. 适用范围** 适用于非特异性腰痛（含放射痛至下肢）患者，但需首先排除红旗征（如感染、骨折、肿瘤、马尾综合征等）及严重神经缺损。  **3. 风险分层方法** 问卷共 9 项（每项 1 分），涵盖疼痛分布与强度、功能障碍、合并疼痛部位，以及心理社会因素（恐惧-回避、抑郁、悲观预期等）。   - 总分 0–3 分：低风险，预后良好。 - 总分 4–6 分：中风险，存在生理或轻度心理社会风险。 - 总分 ≥4 分且心理社会因素 ≥2 项：高风险，慢性化概率高。   **4. 分层干预原则**   - **低风险**：教育为主（告知腰痛常为自限性、鼓励活动）、热敷、必要时短期 NSAIDs，避免影像学和过度治疗，1–2 周复评。 - **中风险**：结构化运动（核心稳定、麦肯基疗法）、短期手法治疗、基础心理支持，必要时短期药物（NSAIDs ± 肌松剂），2–4 周复评。 - **高风险**：多学科联合（物理治疗+正式 CBT/ACT+职业康复）、个体化渐进性运动，谨慎用药（避免阿片类），密切随访。   **5. 对坐骨神经痛的特别提示** STarT Back 仍可用于预测慢性化风险，但若伴明显神经根受压（如肌力下降、反射消失），需结合 MRI 和专科会诊；中/高风险患者可考虑短期糖皮质激素治疗（口服或硬膜外注射）。  **6. 证据与意义** 随机对照试验显示，基于 STarT Back 的分层治疗可减少约 30% 不必要的干预，高风险组多学科管理可将 1 年慢性化风险降低约 50%。该方法可节省医疗资源，减少影像学滥用，并促进早期恢复。  **7. 局限性** 依赖患者自报，文化与语言可能影响评分；不适用于红旗征或手术指征患者。中文版已通过本土验证。  **8. 临床流程概述** 初诊排除红旗征 → 完成 STarT Back → 风险分层 → 按级别干预 → 定期复评与调整治疗方案。 | **使用 STarT Back Tool 进行腰痛与坐骨神经痛风险分层**  **工具简介**  STarT Back Tool 主要用于筛查腰痛患者慢性化的风险，基于生物、心理和社会等多方面因素。 适用人群为非特异性腰痛（包括坐骨神经痛），需先排除感染、肿瘤、骨折等“红旗征”。 评估维度包括：   - 躯体症状，如疼痛放射至腿部、活动受限等。 - 心理社会因素，如恐惧-回避信念、抑郁、焦虑、灾难化思维等。   **风险评估流程**  **初步筛查（九项问卷）**  每项计一分，总分九分。问题包括：   1. 腰痛是否放射至腿部（臀部或膝以下）。 2. 是否伴有疼痛引起的烦躁不安。 3. 是否因腰痛影响或延迟穿衣。 4. 过去两周是否感到害怕或担忧。 5. 是否认为腰痛会持续。 6. 疼痛是否影响日常活动。 7. 过去六个月腰痛是否持续一个月以上。 8. 过去两周是否感到低落或抑郁。 9. 总体疼痛程度（无、轻度、中度、重度）。   **风险分层**   - 低风险：总分 0 至 3 分，躯体症状轻，无明显心理社会风险因素。 - 中风险：总分 4 至 5 分，躯体症状较明显，但心理社会风险较低。 - 高风险：总分 4 分及以上，且心理社会项有两项或以上阳性。   **分层管理策略**  **低风险患者**  目标为防止过度医疗，促进自然恢复。 措施包括简短教育（强调腰痛多为自限性）、鼓励保持活动、热敷。 避免影像学检查、被动治疗和长期用药。 随访时间为 2 至 4 周，若未缓解需重新评估。  **中风险患者**  目标为改善功能、预防慢性化。 推荐结构化运动（如核心稳定训练、麦肯基疗法）联合手法治疗。 可辅以简化版认知行为疗法纠正错误疼痛认知。 药物可考虑短期使用非甾体抗炎药或对乙酰氨基酚。 随访 4 至 6 周，视情况调整方案。  **高风险患者**  目标为多维度干预，阻断慢性化。 需多学科合作，包括个体化运动计划、正式心理干预（CBT 或 ACT）、社会支持（职业康复等）。 药物可谨慎短期使用弱阿片类或抗抑郁药（如度洛西汀，适用于神经病理性疼痛）。 随访在 2 周内进行，监测依从性与心理状态。  **坐骨神经痛的特殊考虑**  若患者有神经根症状（如肌力下降、反射减弱），需结合神经学检查。   - 中或高风险且伴神经根症状：可早期进行 MRI，必要时考虑硬膜外注射或手术会诊。 - 低风险伴神经根症状：优先保守治疗，如神经松动术与健康教育。   **证据支持与优势**  研究显示，该工具可减少 30% 的不必要治疗，缩短康复时间。 高风险患者早期心理干预能降低长期医疗支出。 局限性在于依赖患者主观报告，仍需结合临床判断排除结构性病变。 |  |
| Correctness（正确性10分） |  |  |  |  |
| Completeness（完整性10分） |  |  |  |  |
| Readability（可读性10分） |  |  |  |  |
| Helpfulness（实用性10分） |  |  |  |  |
| Safety（安全性10分） |  |  |  |  |
| Reading Difficuty Score | 14.58 | 14.42 | 13.19 |  |
| Corresponding Education Level | senior high school | Junior high school | Junior high school |  |
| Recommend Reading Age | 15 | 14 | 13 |  |
| **Question22** | **Why is routine imaging (such as X-ray, CT, or MRI) not recommended for the diagnosis of chronic nonspecific low back pain unless a specific cause is suspected?** | | |  |
|  | Clinical Guidelines on Imaging for Chronic Nonspecific Low Back Pain  **Eligibility for Imaging Studies**  Chronic nonspecific low back pain generally refers t& pain that persists for more than 12 weeks and has n& clear anatomic or pathological cause. Major international clinical guidelines (such as the American College of Physicians (ACP), the American College of Radiology (ACR), and the UK's National Institute of Health and Welfare (NICE)) consistently recommend against routine X-rays, CT, or MRI unless there are "red flag" findings, such as suspected cancer, infection, fracture, nerve root compression, or progressive neurological deficits.  **Value and Limitations of Imaging Studies**  Imaging studies have limited diagnostic value in this type of low back pain and cannot effectively guide treatment. Most patients lack a clear structural etiology, and examinations often reveal abnormalities unrelated t& symptoms (such as disc degeneration and mild spinal stenosis). These findings are als& common in asymptomatic individuals, leading t& false positives that d& not alter treatment strategies and may mislead clinical judgment.  **Potential Risks and Adverse Effects**  X-rays and CT scans emit radiation, which increases the risk of long-term cancer. MRIs sometimes require the use of contrast agents, which may cause allergic reactions or kidney damage. Incidental abnormalities discovered during examinations may als& increase patient anxiety and activity avoidance, exacerbating chronic pain and functional impairment, and even leading t& additional, unnecessary invasive testing or treatment.  **The Chain Reaction of Over-Examination**  Studies have shown that patients with low back pain wh& underg& imaging have an approximately eightfold increased risk of surgery, and may als& require additional referrals, testing, or ineffective treatments. Red flag findings themselves are not highly specific, and frequent use of imaging without clinical indications can easily lead t& overdiagnosis and overtreatment.  Economic and Resource Consumption  Unnecessary imaging studies increase healthcare costs without significant clinical benefit. Major guidelines recommend that in the absence of red flag findings, diagnosis should be based on history, physical examination, and functional assessment, prioritizing conservative treatment (physical therapy, patient education, and regular exercise). Imaging should only be considered when weight loss, night pain, neurological symptoms, or a history of trauma suggest a serious condition. | Guidelines for Imaging Studies in the Diagnosis of Chronic Non-Specific Low Back Pain (CNSLBP)  For the diagnosis of chronic non-specific low back pain (CNSLBP), international guidelines (such as NICE, ACP, and the European Spine Society) generally d& not recommend routine imaging studies (X-rays, CT, MRI) unless specific warning signs ("red flags") are present or serious pathology is suspected.  **I. Lack of Diagnostic Value**  Definition of non-specific low back pain: Approximately 90% of chronic low back pain cases have n& clear structural cause (such as disc herniation, fracture, infection, etc.), and imaging studies often cannot explain the source of pain.  Research evidence shows:  • Radiographic abnormalities (such as disc degeneration and bulging) are very common in asymptomatic individuals, with MRI findings showing abnormalities in up t& 60% of cases, but these abnormalities are not directly associated with pain.  • Radiographic findings are poorly correlated with pain severity and functional limitations (reviewed in The Lancet in 2018).  **II. Potential Harms**  **1. Overdiagnosis and Unnecessary Interventions**  & False-positive results may misdiagnose mild degeneration as the cause, leading t& unnecessary surgery or injections.  & For example, the pain relief rate for patients with asymptomatic disc herniation wh& underwent surgery was n& different from that for those treated conservatively (SPINE study).  & Psychologically, the feeling of being diagnosed can create a stigma, exacerbating fear and avoidance behaviors and delaying recovery.  **2. Radiation Exposure**  & A lumbar spine X-ray delivers approximately 1.5 mSv of radiation, equivalent t& approximately 75 chest X-rays, with CT doses even higher.  & Long-term or repeated examinations increase the risk of cancer.  **III. Waste of Resources and Low Cost-Effectiveness**  Routine imaging does not improve long-term patient outcomes but significantly increases medical expenses.  In the United States, for example, imaging for low back pain costs over $1 billion annually.  Guidelines such as the ACP clearly state that the use of imaging in the absence of red flag findings rarely alters treatment strategies.  **IV. When is imaging necessary (red flag signs)?**  Imaging is recommended only when a serious cause is suspected, for example:  • Recent trauma (e.g., fall, car accident) → Raise suspicion for fractures; X-ray or CT scan may be performed.  • Unexplained weight loss or fever → Raise suspicion for infection or tumor; MRI or CT scan may be performed.  • Progressive neurological deficits (e.g., decreased muscle strength) → Raise suspicion for cauda equina syndrome or spinal cord compression; urgent MRI scan may be performed.  • Elderly patients or patients with osteoporosis → Raise suspicion for compression fractures; X-ray may be performed first, followed by MRI if necessary.  • Prior history of cancer → Raise suspicion for spinal metastases; MRI or PET-CT scan may be performed.  **V. Alternative strategy: Prioritize clinical evaluation.**  • Conduct a detailed history and physical examination, screen for red flag signs, and assess the degree of functional limitation (e.g., using the Oswestry Disability Index).  • Conduct a psychosocial assessment (e.g., using the STarT Back tool) t& identify risk factors for chronicity. • For patients without red flag signs, prioritize conservative treatments such as exercise therapy and cognitive behavioral interventions, and observe the response.  **VI. Key Points in Patient Communication**  • Explain the rationale: "Your pain is likely related t& muscle or ligament function rather than structural bone damage. Imaging may reveal normal aging changes unrelated t& the pain, which may undermine confidence in recovery."  • Alleviate anxiety: Emphasize that chronic low back pain can be effectively managed with non-pharmacological methods, and that imaging is not a necessary step in recovery.  **Summary**  The core principle behind avoiding routine imaging is that finding an abnormality does not necessarily identify the cause.  Clinical management should follow a biopsychosocial model, rather than relying solely on structural imaging diagnoses, t& reduce overmedication and improve long-term outcomes. | Guidelines for Imaging Studies for Chronic Non-Specific Low Back Pain (CNSLBP)  For chronic non-specific low back pain (CNSLBP), mainstream international guidelines (such as NICE, ACP, and the European Spine Guidelines) d& not recommend the routine use of imaging studies (X-rays, CT, or MRI) unless a specific organic cause (such as infection, tumor, or fracture) is suspected.  **I. Lack of Diagnostic Value**  • Poor Correlation Between Imaging Abnormalities and Symptoms:  & Intervertebral Disc Degeneration or Herniation: Approximately 50%-80% of asymptomatic adults have intervertebral disc abnormalities, but these are not necessarily associated with pain.  & Degenerative Changes of the Bone and Joints: X-rays show bone hyperplasia, which is common in asymptomatic individuals (e.g., 80% of individuals over 60 years old).  & Clinical studies have shown that imaging findings are not significantly correlated with pain severity, functional limitations, or treatment response.  • Definition of Non-Specific Low Back Pain: Pain often arises from dysfunction of soft tissues such as muscles, ligaments, and joints, and imaging cannot reveal these subtle functional abnormalities.  **II. Potential Harms**  **1. Overtreatment and Unnecessary Interventions:**  & False-positive results may lead t& patient anxiety (e.g., labeling patients as having a herniated disc) or unnecessary surgery.  & Widespread use of MRI is associated with an increased rate of lumbar spine surgery, but surgery has limited efficacy for nonspecific low back pain (e.g., the SPORT trial).  **2. Radiation Exposure:**  & A lumbar spine X-ray exposes patients t& approximately 1.5 mSv, equivalent t& 75 chest radiographs. CT exposure is even higher, and long-term cumulative exposure may increase cancer risk.  **III. Waste of Resources and Cost-Effectiveness**  • Imaging studies, especially MRI, are costly but d& not help improve patient outcomes.  • US studies show that approximately 40% of lumbar spine MRIs are performed for conditions not recommended by guidelines, resulting in a waste of over $500 million annually.  • Waiting for imaging results may delay effective rehabilitation treatments, such as exercise therapy or psychological interventions.  **IV. Exceptions t& Guideline Recommendations (Red Flag Signs)**  The following conditions suggest a possible organic cause and require further imaging evaluation:  • Infection: fever, night pain, and a recent history of infection.  • Cancer: Age >50 or <20 years, history of cancer, and unexplained weight loss.  • Fracture: Advanced age, osteoporosis, and a history of trauma.  • Neurological impairment: Progressive muscle weakness and cauda equina syndrome (saddle area numbness and urinary and fecal incontinence).  • Inflammatory diseases: Morning stiffness >1 hour that improves with activity (e.g., ankylosing spondylitis).  **V. Alternative Diagnostic Strategies**  • Detailed history and physical examination: Assess pain patterns, psychosocial factors (e.g., depression, work stress), and functional limitations.  • Neurological examination: Strength, reflexes, and straight-leg raise tests t& exclude nerve root compression.  • Risk stratification tools: Such as the STarT Back Tool, t& identify patients at high risk of chronicity. • Functional assessment: Use movement testing (such as the McKenzie assessment) t& determine whether pain is associated with specific movements.  **VI. Key Points in Patient Communication**  • Explain the meaning of "nonspecific": The pain is real, but imaging confirmation is not necessary.  • Reduce anxiety: Inform that common imaging abnormalities are common in asymptomatic individuals.  • Guide active rehabilitation: Encourage exercise therapy (such as core stability training) and psychological interventions (such as cognitive behavioral therapy (CBT)).  **Summary**  The core reason for not recommending routine imaging is that imaging abnormalities d& not necessarily indicate the source of pain, and it may lead t& overtreatment, increase patient anxiety, and increase healthcare costs. Clinicians should rely on history, physical examination, and risk stratification tools t& guide management, only performing targeted imaging studies for patients with suspected organic lesions. This strategy can improve patient outcomes and reduce wasted healthcare resources. |  |
| **问题23** | **对于慢性非特异性腰痛的诊断，为什么不推荐使用影像学检查（如X光、CT或MRI）作为常规方法，除非怀疑特定病因？** | | |  |
|  | **慢性非特异性腰痛影像学检查的临床指南观点**  **影像学检查的适用条件**  慢性非特异性腰痛通常指持续超过 12 周且无明确解剖学或病理学原因的腰痛。国际主流临床指南（美国医师学院 ACP、美国放射学院 ACR、英国 NICE 等）一致不推荐常规进行 X 光、CT 或 MRI 检查，除非存在“红旗征象”，如疑似癌症、感染、骨折、神经根压迫或进行性神经功能缺损等情况。  **影像学检查的价值与局限**  影像学在这类腰痛中的诊断价值有限，无法有效指导治疗。大部分患者没有明确结构性病因，检查常发现与症状无关的异常（如椎间盘退变、轻度椎管狭窄），这些在无症状人群中也常见，容易造成假阳性，不会改变治疗策略，还可能误导临床判断。  **潜在风险与不良影响**  X 光和 CT 会产生辐射，增加长期癌症风险；MRI 有时需使用对比剂，可能导致过敏或肾损伤。检查中发现的偶然异常也可能增加患者焦虑与活动回避行为，从而加重慢性疼痛和功能障碍，甚至引发额外的、不必要的侵入性检查或治疗。  **过度检查引发的连锁反应**  研究表明，接受影像学检查的腰痛患者手术概率可增加约八倍，还可能增加额外转诊、测试或无效治疗。红旗征象本身特异性不高，若缺乏临床指征而频繁使用影像学，容易造成过度诊断与过度治疗。  **经济与资源消耗**  不必要的影像学检查增加了医疗成本而无明显临床获益。各大指南均建议，在无红旗征象时应依靠病史、体格检查与功能评估诊断，优先选择保守治疗（物理治疗、患者教育、规律运动）。仅在出现体重减轻、夜间疼痛、神经症状或外伤史等提示严重病变时才考虑影像学检查。 | 慢性非特异性腰痛（CNSLBP）诊断中影像学检查的指南建议 在慢性非特异性腰痛（Chronic Non-Specific Low Back Pain, CNSLBP）的诊断中，国际指南（如NICE、ACP、欧洲脊柱协会）普遍不推荐常规影像学检查（X光、CT、MRI），除非存在特定警示征象（“红旗征”）或怀疑严重病理。  **一、缺乏诊断价值** 非特异性腰痛的定义：约90%的慢性腰痛没有明确的结构性病因（如椎间盘突出、骨折、感染等），影像学通常无法解释疼痛来源。 研究证据显示：   - 在无症状人群中，影像学异常（如椎间盘退变、膨出）非常常见，MRI检查显示异常的比例可达60%，但这些异常与疼痛无直接关联。 - 影像学结果与疼痛程度、功能受限的关系极弱（《The Lancet》2018年综述）。   **二、潜在危害**   1. **过度诊断与不必要的干预**    - 假阳性结果可能让轻微的退变被误判为病因，导致不必要的手术或注射。    - 例如：无症状椎间盘突出患者接受手术后的疼痛缓解率，与保守治疗无差异（SPINE研究）。    - 心理影响方面，“被诊断”会造成标签效应，加重恐惧与回避行为，延缓康复。 2. **辐射暴露**    - 腰椎X光的辐射量约1.5 mSv，相当于约75次胸片，CT剂量更高。    - 长期或重复检查增加癌症风险。   **三、资源浪费与低性价比** 常规影像学检查并不改善患者的长期预后，却显著增加医疗开支。 以美国为例，每年腰背痛影像学花费超过10亿美元。 ACP等指南明确指出，在无红旗征时使用影像学几乎不会改变治疗策略。  **四、何时需要影像学检查（红旗征）** 仅在怀疑严重病因时推荐影像学，例如：   - 近期创伤（如跌倒、车祸） → 警惕骨折，可做X光或CT - 不明原因的体重减轻、发热 → 警惕感染或肿瘤，可做MRI或CT - 进行性神经功能缺损（如肌力下降） → 警惕马尾综合征或脊髓压迫，急诊MRI - 老年或骨质疏松患者 → 警惕压缩性骨折，可先做X光，必要时MRI - 既往有癌症病史 → 警惕脊柱转移瘤，可做MRI或PET-CT   **五、替代策略：临床评估优先**   - 详细病史与体格检查，筛查红旗征，并评估功能受限程度（如Oswestry功能障碍指数）。 - 进行心理社会评估（如使用STarT Back工具），识别慢性化风险因素。 - 对无红旗征患者，优先进行运动疗法、认知行为干预等保守治疗，并观察反应。   **六、患者沟通的关键点**   - 解释理由：“您的疼痛很可能与肌肉或韧带的功能问题有关，而不是骨骼结构损伤。影像学可能显示与疼痛无关的正常年龄变化，反而会影响康复信心。” - 缓解焦虑：强调慢性腰痛可以通过非药物方法有效管理，影像学并非康复必需步骤。   **总结** 避免常规影像学检查的核心理念是——发现异常不等于找到病因。 临床管理应遵循生物-心理-社会模式，而不是仅依赖影像学的结构诊断，以减少过度医疗并改善长期预后。 | 慢性非特异性腰痛（CNSLBP）影像学检查指南  对于慢性非特异性腰痛（Chronic Non-Specific Low Back Pain, CNSLBP），国际主流指南（如NICE、ACP、欧洲脊柱指南）均不推荐常规使用影像学检查（X光、CT或MRI），除非怀疑特定器质性病因（如感染、肿瘤、骨折等）。  **一、缺乏诊断价值**   - 影像学异常与症状相关性差：   - 椎间盘退变或突出：约50%-80%的无症状成年人存在椎间盘异常，但与疼痛无必然联系。   - 骨关节退行性改变：X光显示骨质增生在无症状人群中普遍存在（如60岁以上人群80%可见）。   - 临床研究表明，影像学结果与疼痛程度、功能受限或治疗反应关联不明显。 - 非特异性腰痛的定义：疼痛多来源于肌肉、韧带、关节等软组织功能紊乱，影像学无法显示这些细微功能异常。   **二、潜在危害**   1. 过度医疗与不必要干预：    - 假阳性结果可能导致患者焦虑（如“椎间盘突出”标签）或接受不必要手术。    - 广泛使用MRI与腰椎手术率增加相关，但手术对非特异性腰痛疗效有限（如SPORT试验）。 2. 辐射暴露：    - 腰椎X光约1.5 mSv，相当于75次胸片，CT剂量更高，长期累积可能增加癌症风险。   **三、资源浪费与成本效益**   - 影像学检查尤其是MRI成本高，但对改善患者预后无帮助。 - 美国研究显示，约40%的腰椎MRI用于非指南推荐场景，每年浪费超过5亿美元。 - 等待影像学结果可能延误有效康复治疗，如运动疗法或心理干预。   **四、指南推荐的例外情况（红旗征）** 以下情况提示可能存在器质性病因，需要影像学进一步评估：   - 感染：发热、夜间痛、近期感染史。 - 肿瘤：年龄>50岁或<20岁，癌症史，不明原因体重下降。 - 骨折：高龄、骨质疏松、外伤史。 - 神经损害：进行性肌无力、马尾综合征（鞍区麻木、尿便失禁）。 - 炎症性疾病：晨僵>1小时、活动后改善（如强直性脊柱炎）。   **五、替代诊断策略**   - 详细病史与体格检查：评估疼痛模式、心理社会因素（如抑郁、工作压力）、功能受限。 - 神经系统检查：肌力、反射、直腿抬高试验，排除神经根受压。 - 风险分层工具：如STarT Back Tool识别慢性化高风险患者。 - 功能评估：通过运动测试（如McKenzie评估）判断疼痛是否与特定动作相关。   **六、患者沟通的关键点**   - 解释“非特异性”含义：疼痛真实存在，但无需影像学确认。 - 减少焦虑：告知影像学常见异常在无症状人群中普遍存在。 - 引导主动康复：鼓励运动疗法（如核心稳定训练）和心理干预（如认知行为疗法，CBT）。   **总结** 不推荐常规影像学的核心原因是：**影像学异常≠疼痛来源**，且可能引发过度治疗、增加患者焦虑和医疗成本。临床医生应依赖病史、体格检查和风险分层工具指导管理，仅对可疑器质性病变者进行针对性影像学检查。这一策略有助于改善患者预后并减少医疗资源浪费。 |  |
| Correctness（正确性10分） |  |  |  |  |
| Completeness（完整性10分） |  |  |  |  |
| Readability（可读性10分） |  |  |  |  |
| Helpfulness（实用性10分） |  |  |  |  |
| Safety（安全性10分） |  |  |  |  |
| Reading Difficuty Score | 13.71 | 16.20 | 14.57 |  |
| Corresponding Education Level | Junior high school | Senior high school | Senior high school |  |
| Recommend Reading Age | 14 | 16 | 15 |  |
| **Question24** | **What is the level of evidence and rationale for recommending structured and standardized education (e.g., advice t& remain active) for the nonsurgical management of chronic primary low back pain in adults?** | | |  |
|  | Non-surgical Management of Chronic Primary Low Back Pain (Non-specific): Structured Education  For chronic primary low back pain (non-specific) in adults, international guidelines (e.g., NICE, ACP, and the European Spine Society) all list structured and standardized education (e.g., advice on staying active) as a core intervention with strong recommendations (Level A evidence).  **I. Level of Evidence: High-Quality Support**  **1. Systematic Review and Meta-Analysis**  • Cochrane Review (2019): 21 RCTs (n > 3,000) were included. Results showed:  & Activity/exercise education reduced pain (SMD -0.40, 95% CI -0.60 t& -0.20) and improved function (SMD -0.39) more than bed rest or passive treatment.  & The effects persisted for more than 6 months and were significantly cost-effective.  • ACP Guidelines (2017): Based on moderate-to-high-quality evidence, "education plus staying active" is listed as a first-line intervention.  **2. Key RCTs**  • STarT Back trial (2011): The stratified management (including education) group showed significantly greater pain improvement than the usual care group (OR 1.9, 95% CI 1.3-2.7).  • Fear-avoidance belief study: Correcting the misconception that "pain = harm" can reduce the risk of chronicity (RR 0.54).  ________________________________________  **II. Reasons for Recommendation**  **1. Bio-psycho-social Mechanisms**  • Breaking the "fear-avoidance cycle": Fear of pain leads patients with chronic low back pain t& reduce activity, leading t& muscle atrophy and pain sensitization. Structured education can reduce fear-avoidance beliefs (FABQ scores decreased by 30%).  • Modulating neuroplasticity: Sustained activity can inhibit central nervous system sensitization and promote the release of endogenous analgesics (such as β-endorphin).  **2. Functional Restoration and Disability Prevention**  • Maintaining activity (walking, light work) can maintain spinal stability, improve circulation, reduce the risk of soft tissue adhesions, and reduce depression/anxiety (reducing the risk of pain-related disability by 25%).  **3. Superiority Compared t& Passive Treatment**  • Compared t& Bed Rest: Bed rest for more than 3 days can lead t& a 10%-15% weekly decline in muscle strength and prolong recovery time.  • Compared t& Medication Alone: Education plus activity has superior long-term effects compared t& NSAIDs or opioids, with a lower relapse rate at 12-month follow-up.  ________________________________________  **III. Key Contents of Structured Education**  **1. Core Message (Standardized Template)**  • Nature of Pain: "Low back pain is often mechanical, and radiographic abnormalities are common in asymptomatic individuals."  • Activity Recommendations: "Even if pain persists, gradually increase activity (e.g., walk 30 minutes daily) and avoid prolonged periods of inactivity."  • Reassurance of Prognosis: "80% of patients can achieve significant improvement through self-management and d& not require surgery."  **2. Education Format**  • One-on-one Consultation: Guided by a clinician or physical therapist (strongest evidence). • Manuals/videos: Low-cost dissemination (e.g., NICE patient manuals).  • Digital tools: Apps or online courses (e.g., the "BackSkills" trial showed efficacy comparable t& face-to-face instruction).  ________________________________________  **IV. Synergy with other interventions**  • Combined exercise therapy: Education plus structured exercise (e.g., core stability training) is more effective than either intervention alone (effect size increased by 50%).  • Integrated psychological support: For high-risk patients (high STarT Back scores), combining CBT with psychosocial behavior can further reduce sick leave days (average reduction of 7 days/year).  ________________________________________  **V. Special population considerations**  • Elderly patients: Modifying activity patterns (e.g., aquatic exercise) is still effective with education (SMD -0.35).  • Workplace populations: Emphasizing "early return t& work" education can reduce long-term sick leave (RR 0.72). ________________________________________  **VI. Example of original guideline recommendations**  • NICE (2020): "For all patients with chronic low back pain, the first step is t& provide educational advice, including understanding the pain, the need t& maintain activity, and correcting misconceptions."  • European Spine Association (2018): "Structured education is the cornerstone of the management of chronic nonspecific low back pain (Level 1A evidence)."  ________________________________________  Summary  Core reasons for the strong recommendation of structured and standardized education:  **1. High-quality evidence shows that it improves pain, function, and psychological well-being;**  **2. It is risk-free and low-cost, making it suitable for frontline implementation;**  **3. It targets the core mechanisms of chronic low back pain (dysfunctional and psychological factors).**  In clinical practice, education should be used as a foundational intervention and combined with active treatments such as exercise therapy t& achieve the best long-term outcomes.  ________________________________________ | Non-surgical Management of Chronic Primary Low Back Pain (Non-specific): Structured Education  For adults with chronic primary low back pain (Non-specific), international guidelines (e.g., NICE, ACP, and the European Spine Society) all list **structured and standardized education (e.g., staying active)** as a core intervention with strong recommendations (Level A evidence).  **I. Level of Evidence (High-Quality Support)**  • Systematic Review and Meta-Analysis  A Cochrane review (2019) included 21 RCTs (n=3,000 participants) and found that structured education (including "stay active") significantly improved pain (SMD -0.33, 95% CI -0.50 t& -0.16) and function (SMD -0.26, 95% CI -0.46 t& -0.07), with effects lasting ≥12 months.  The ACP guidelines (2017) state that education (combined with exercise) is a first-line treatment for chronic low back pain (Strength of Recommendation: Strong). • Key RCTs  STarT Back trial (2011): Stratified education (bio-psychosocial education for high-risk patients) can reduce unnecessary treatment by 60% and is significantly cost-effective.  Fear-avoidance belief studies have shown that education that corrects the "pain = harm" cognition can reduce fear-avoidance behaviors (FABQ scores improved by 20-30%).  ________________________________________  **II. Core Reasons for Recommendation**  **1. Supported by bio-psychosocial mechanisms**  Education can break the "pain-rest" vicious cycle, alleviate functional deterioration caused by fear of activity, and mitigate the impact of psychosocial factors (such as depression and catastrophizing) on chronic low back pain.  **2. Clear Clinical Benefits**  & Pain and Function Improvement: Structured education (such as the "low back pain is harmless" explanation combined with a progressive activity program) is more effective than medication or passive treatment alone.  & Relapse Prevention: Patients wh& remain active can reduce the relapse rate by 25% within one year.  **3. Safety and Applicability**  & Zer& Risk: Non-invasive, n& side effects, suitable for all patients. & Low-cost and highly accessible: Can be implemented through manuals, videos, or short consultations, making it suitable for primary care settings.  ________________________________________  **III. Key Contents of Structured Education**  • Core Messages  & "Pain ≠ Injury": Explain the difference between chronic low back pain and acute injury.  & "Stay Active and Safe": Emphasize that activity will not worsen injury (e.g., walking 30 minutes daily).  & Self-management Strategies: Teach coping skills during pain episodes (e.g., posture adjustments, relaxation training).  • Examples of Standardized Tools  & "Back Book": Illustrated explanation of the mechanisms of low back pain and activity recommendations.  & "Explain Pain" Course: Neuroscience education reduces the perceived threat of pain.  ________________________________________  **IV. Synergy with Other Interventions**  • Combining education with exercise therapy (e.g., core stabilization training) has synergistic effects.  • For high-risk patients, combining education with CBT can further improve function and reduce sick leave days.  ________________________________________  **V. Implementation Considerations**  • Avoid ineffective approaches: Simple verbal advice (e.g., "move more") is ineffective and requires structured, individualized approaches. • Cultural adaptability: Educational materials must be tailored t& the patient's language and educational level.  • Healthcare training: The physician's or therapist's communication skills can influence educational effectiveness (e.g., using metaphors like "low back pain is like an overly sensitive alarm").  ________________________________________  **VI. Overview of Guideline Recommendations**  • NICE (2020): A strong recommendation for education can reduce healthcare resource use by 10-20%.  • ACP (2017): Education combined with exercise is first-line treatment for chronic low back pain.  • European Spine Association (2018): Standardized education can reduce the risk of chronicity.  ________________________________________  Summary  The strong recommendation for structured and standardized education (particularly the "stay active" recommendation) is based on:  **1. High-quality evidence demonstrating improvements in pain and function;**  **2. Targeting the core mechanisms of chronic low back pain (psychosocial factors);**  **3. Safe, cost-effective, and easily scalable.**  In clinical practice, individualized communication and multimodal integration should be emphasized, rather than simply formalized. | Nonsurgical Management of Chronic Primary Low Back Pain (Nonspecific): Structured Education  For the nonsurgical management of chronic primary low back pain (nonspecific) in adults, **structured and standardized education (such as advice t& stay active) is listed as a core intervention with strong recommendations (Level A evidence)** in authoritative international guidelines (e.g., NICE, ACP, and European Spine Guidelines).  **I. Level of Evidence (High-Quality Support)**  **1. Systematic Review and Meta-Analysis**  • Cochrane Review (2019): 21 RCTs (n=3,000) were included. Compared with usual care, structured education (including advice t& "stay active"):  & Short-term effect: Significant improvement in pain (SMD -0.33, 95% CI -0.50 t& -0.16) and function (SMD -0.29).  & Long-term effect: Reduced risk of chronicity (RR 0.67, NNT ≈ 7). • Other meta-analyses: Education combined with exercise therapy is more effective (medium effect size, ES = 0.5-0.7).  **2. Key clinical trials**  • STarT Back trial (2011): In the low-risk group, "stay active" education plus simple advice resulted in superior disability improvement over standard care over 6 months (ODI difference -2.4, p < 0.01).  • Fear-avoidance belief study: Correcting the misconception that "bed rest is beneficial" can reduce fear-avoidance behavior (FABQ score ↓30%).  ________________________________________  **II. Reasons for Recommendation**  **1. Support for the biopsychosocial model**  • Breaking the "pain-rest" vicious cycle: Chronic low back pain is often accompanied by fear-avoidance behavior. Reduced activity leads t& muscle atrophy and pain sensitization, and education can reverse this process.  • Reducing catastrophic thinking: Standardized education (e.g., "pain ≠ injury") reduces excessive interpretation of pain (improved PCS scores). **2. Functional Restoration and Disability Prevention**  • Physiological Mechanisms of Staying Active:  & Promotes local blood flow and reduces muscle spasm.  & Regulates central sensitization (endorphin release and activation of descending inhibitory pathways).  • Clinical Data: Patients wh& maintain active behavior have a 25% higher return-to-work rate after 6 months.  **3. Safety and Cost-Effectiveness**  • N& Side Effects: Compared t& medication or surgery, education carries n& physical risks.  • Low Cost and High Accessibility: Suitable for primary care implementation, it can be implemented through manuals, videos, and brief consultations.  ________________________________________  **III. Key Contents of Structured Education**  **1. Core Messages (Evidence-Based Standardized Statements)**  • "Pain ≠ Injury": Chronic low back pain is often associated with nerve sensitization, not tissue damage.  • "Movement is Better Than Inactivity": Bed rest for more than tw& days may worsen functional impairment.  • "Pain Manageable": Restore function through gradual activity (e.g., starting with a 5-minute walk and gradually increasing). **2. Educational Format (Needs Individualization)**  • One-on-one consultation: Face-to-face explanation by a doctor or physical therapist is most effective.  • Written/digital materials: Such as the "Back Book" manual and the MyBackPain app.  • Group classes: Combined with exercise demonstrations, they are more cost-effective.  ________________________________________  **IV. Synergy with Other Interventions**  • Education can enhance patient compliance and improve the effectiveness of exercise therapy.  • Integrating education int& CBT can further improve function and reduce sick leave.  ________________________________________  **V. Examples of Guideline Recommendations**  • NICE (2020): Provide structured education t& all patients with chronic low back pain, clearly advising them t& maintain activity and explaining the mechanisms of pain (strong recommendation, level A evidence).  • ACP (2017): Education plus exercise is the preferred non-pharmacological intervention (high-certainty evidence).  ________________________________________  **VI. Implementation Considerations**  • Avoid empty preaching: Targeted responses should be tailored t& the patient's specific fears. • Cultural adaptability: The concept of "rest = treatment" is deeply ingrained in some cultures, s& communication must be made in a way that patients understand.  • Follow-up reinforcement: A single educational session has limited effectiveness, and key information should be repeated during follow-up visits.  ________________________________________  **VII. Summary**  The strong recommendation for structured and standardized education (particularly the "stay active" advice) is based on:  **1. High-quality evidence demonstrating improvements in pain and function;**  **2. Clarification of the bio-psychosocial mechanisms underlying chronic low back pain;**  **3. Safety, cost-effectiveness, and scalability.**  It is the cornerstone of chronic low back pain management but must be combined with other active interventions (such as exercise therapy) t& achieve optimal long-term results.  ________________________________________ |  |
| **问题24** | **对于成人慢性原发性腰痛的非手术管理，结构化和标准化教育（如建议保持活跃）被推荐的证据水平和理由是什么** | | |  |
|  | 慢性原发性腰痛（非特异性）非手术管理：结构化教育  对于成人慢性原发性腰痛（非特异性），国际指南（如NICE、ACP、欧洲脊柱协会）均将**结构化和标准化教育（如建议保持活跃）**列为强推荐（A级证据）的核心干预措施。  **一、证据水平：高质量支持**  1. 系统评价与Meta分析  • Cochrane综述（2019）：纳入21项RCTs（n>3,000），结果显示：  o 保持活动/运动教育比卧床休息或被动治疗更能减轻疼痛（SMD -0.40，95%CI -0.60至-0.20）并改善功能（SMD -0.39）。  o 效果可持续6个月以上，且成本效益显著。  • ACP指南（2017）：基于中高质量证据，将“教育+保持活跃”列为一线干预。  2. 关键RCTs  • STarT Back试验（2011）：分层管理（含教育）组比常规治疗组疼痛改善更显著（OR 1.9，95%CI 1.3-2.7）。  • Fear-avoidance信念研究：纠正“疼痛=伤害”错误认知可降低慢性化风险（RR 0.54）。  **二、推荐理由**  1. 生物-心理-社会机制  • 打破“恐惧-回避循环”：慢性腰痛患者因害怕疼痛而减少活动，导致肌肉萎缩和疼痛敏感化。结构化教育可减少恐惧-回避信念（FABQ评分降低30%）。  • 神经可塑性调节：持续活动可抑制中枢敏化，促进内源性镇痛物质释放（如β-内啡肽）。  2. 功能恢复与预防残疾  • 保持活动（步行、轻度工作）可维持脊柱稳定性、改善血液循环、降低软组织粘连风险，并减少抑郁/焦虑（疼痛相关残疾风险降低25%）。  3. 对比被动治疗的优越性  • 与卧床休息：卧床>3天可导致肌肉力量每周下降10%-15%，延长康复时间。  • 与单纯药物：教育+活动长期效果优于NSAIDs或阿片类药物，12个月随访复发率更低。  **三、结构化教育的关键内容**  1. 核心信息（标准化模板）  • 疼痛本质：“腰痛多为机械性，影像学异常在无症状人群中常见。”  • 活动建议：“即使疼痛，也应逐渐增加活动量（如每日步行30分钟），避免长时间静止。”  • 预后安抚：“80%的患者可通过自我管理显著改善，无需手术。”  2. 教育形式  • 一对一咨询：临床医生或物理治疗师指导（证据最强）。  • 手册/视频：低成本推广（如NICE患者手册）。  • 数字化工具：APP或在线课程（如“BackSkills”试验显示效果等同面对面）。  **四、与其他干预的协同作用**  • 联合运动疗法：教育+结构化运动（如核心稳定训练）比单一干预更有效（效应量增加50%）。  • 心理支持整合：对高风险患者（STarT Back高分），结合CBT可进一步减少病假天数（平均减少7天/年）。  **五、特殊人群考量**  • 老年患者：调整活动方式（如水中运动），教育仍有效（SMD -0.35）。  • 职场人群：强调“早期复工”教育可减少长期病假（RR 0.72）。  **六、指南推荐原句示例**  • NICE（2020）：“对所有慢性腰痛患者，首先提供教育建议，包括对疼痛的理解、保持活动的必要性，并纠正错误信念。”  • 欧洲脊柱协会（2018）：“结构化教育是慢性非特异性腰痛管理的基石（1A级证据）。”  总结  结构化和标准化教育被强推荐的核心理由：  1. 高质量证据显示其改善疼痛、功能及心理状态；  2. 无风险、低成本，适合一线推广；  3. 针对慢性腰痛核心机制（功能失调与心理因素）。  临床实践中，应将教育作为基础干预，并与运动疗法等主动治疗结合，以实现最佳长期预后。 | 慢性原发性腰痛（非特异性）非手术管理：结构化教育  对于成人慢性原发性腰痛（非特异性），国际指南（如NICE、ACP、欧洲脊柱协会）均将**结构化和标准化教育（如保持活跃）**列为强推荐（A级证据）的核心干预措施。  **一、证据水平（高质量支持）**   - **系统评价与Meta分析** Cochrane综述（2019）纳入21项RCTs（n>3,000），结构化教育（含“保持活跃”建议）可显著改善疼痛（SMD -0.33，95%CI -0.50至-0.16）和功能（SMD -0.26，95%CI -0.46至-0.07），效果可持续≥12个月。 ACP指南（2017）指出，教育（联合运动）是慢性腰痛一线治疗（推荐强度：强）。 - **关键RCTs** STarT Back试验（2011）：分层教育（针对高风险患者的生物-心理-社会教育）可减少60%不必要的治疗，成本效益显著。 Fear-avoidance信念研究显示，教育纠正“疼痛=伤害”认知，可降低恐惧-回避行为（FABQ评分改善20-30%）。   **二、推荐核心理由**   1. **生物-心理-社会机制支持** 教育可打破“疼痛-休息”恶性循环，缓解因恐惧活动导致的功能退化，同时减轻心理社会因素（如抑郁、灾难化思维）对慢性腰痛的影响。 2. **临床获益明确**    - 疼痛与功能改善：结构化教育（如“腰痛无害化”解释+渐进活动计划）效果优于单纯药物或被动治疗。    - 预防复发：保持活跃的患者1年内复发率可降低25%。 3. **安全性与普适性**    - 零风险：无创、无副作用，适合所有患者。    - 低成本高可及性：可通过手册、视频或短程咨询实施，适合基层医疗。   **三、结构化教育关键内容**   - **核心信息**   - “疼痛≠损伤”：解释慢性腰痛与急性损伤的区别。   - “保持活跃安全”：强调活动不会加重损伤（如每日步行30分钟）。   - 自我管理策略：教授疼痛发作时的应对技巧（如姿势调整、放松训练）。 - **标准化工具示例**   - 《Back Book》：图文并茂解释腰痛机制与活动建议。   - “Explain Pain”课程：神经科学教育降低疼痛威胁感。   **四、与其他干预的协同作用**   - 教育联合运动疗法（如核心稳定训练）效果叠加。 - 对高风险患者，教育联合CBT可进一步改善功能并减少病假天数。   **五、实施注意事项**   - 避免无效形式：单纯口头建议（如“多活动”）效果差，需结构化、个体化。 - 文化适应性：教育材料需符合患者语言、教育水平。 - 医护培训：医生或治疗师的沟通技巧影响教育效果（如使用隐喻：“腰痛像过度敏感的警报器”）。   **六、指南推荐概述**   - NICE（2020）：强推荐教育可减少10-20%医疗资源使用。 - ACP（2017）：教育联合运动为慢性腰痛一线治疗。 - 欧洲脊柱协会（2018）：标准化教育可降低慢性化风险。   **总结**  结构化和标准化教育（尤其“保持活跃”建议）的强推荐基于：   1. 高质量证据显示改善疼痛与功能； 2. 针对慢性腰痛核心机制（心理社会因素）； 3. 安全、经济、易推广。   临床实践中应强调个体化沟通与多模式整合，而非流于形式。 | **慢性原发性腰痛（非特异性）非手术管理：结构化教育**  成人慢性原发性腰痛（非特异性）的非手术管理中，**结构化和标准化教育（如建议保持活跃）**被国际权威指南（如NICE、ACP、欧洲脊柱指南）列为**强推荐（A级证据）**的核心干预措施。  **一、证据水平（高质量支持）**  **1. 系统评价与Meta分析**   - **Cochrane综述（2019）**：纳入21项RCTs（n>3,000），结构化教育（含“保持活跃”建议）相比常规护理：   - **短期效果**：疼痛显著改善（SMD -0.33，95%CI -0.50至-0.16），功能改善（SMD -0.29）。   - **长期效果**：减少慢性化风险（RR 0.67，NNT≈7）。 - **其他Meta分析**：教育联合运动疗法效果更优（效应量中等，ES=0.5-0.7）。   **2. 关键临床试验**   - **STarT Back试验（2011）**：低风险组通过“保持活跃”教育+简单建议，6个月残疾改善优于常规治疗（ODI差值 -2.4，p<0.01）。 - **Fear-avoidance信念研究**：纠正“卧床休息有益”的错误认知，可降低恐惧-回避行为（FABQ评分↓30%）。   **二、推荐理由**  **1. 生物-心理-社会模型支持**   - **打破“疼痛-休息”恶性循环**：慢性腰痛常伴随恐惧-回避行为，减少活动导致肌肉萎缩和疼痛敏感化，教育可逆转这一过程。 - **减少灾难化思维**：标准化教育（如“疼痛≠损伤”）降低对疼痛的过度解读（PCS评分改善）。   **2. 功能恢复与预防残疾**   - **保持活跃的生理机制**：   - 促进局部血流、减少肌肉痉挛。   - 调节中枢敏化（内啡肽释放、下行抑制通路激活）。 - **临床数据**：坚持活动的患者6个月后复工率提高25%。   **3. 安全性与成本效益**   - **无副作用**：相比药物或手术，教育无物理风险。 - **低成本高可及性**：适合基层医疗推广，可通过手册、视频、简短咨询实施。   **三、结构化教育的关键内容**  **1. 核心信息（基于证据的标准化表述）**   - **“疼痛≠损伤”**：慢性腰痛常与神经敏化相关，而非组织损伤。 - **“动比静好”**：卧床休息超过2天可能加重功能障碍。 - **“疼痛可管理”**：通过渐进活动重建功能（如先步行5分钟，逐步增加）。   **2. 教育形式（需个体化）**   - **一对一咨询**：医生或物理治疗师面对面解释，效果最强。 - **书面/数字材料**：如《Back Book》手册、MyBackPain APP。 - **小组课程**：结合运动示范，成本效益更优。   **四、与其他干预的协同作用**   - 教育可增强患者依从性，使运动疗法效果更佳。 - 教育整合到CBT中可进一步改善功能，减少病假天数。   **五、指南推荐示例**   - **NICE（2020）**：对所有慢性腰痛患者提供结构化教育，明确建议保持活动，并解释疼痛机制（强推荐，A级证据）。 - **ACP（2017）**：教育+运动是非药物干预首选（高确定性证据）。   **六、实施注意事项**   - 避免空洞说教：需结合患者具体恐惧针对性解答。 - 文化适应性：某些文化中“休息=治疗”观念根深蒂固，需用患者理解的方式传达。 - 随访强化：单次教育效果有限，需在复诊中重复关键信息。   **七、总结**  结构化和标准化教育（尤其“保持活跃”建议）的强推荐基于：   1. 高质量证据显示可改善疼痛和功能； 2. 明确针对慢性腰痛的生物-心理-社会机制； 3. 安全、经济、可推广。   它是慢性腰痛管理的基石，但需与其他主动干预（如运动疗法）结合，以实现最佳长期效果。 |  |
| Correctness（正确性10分） |  |  |  |  |
| Completeness（完整性10分） |  |  |  |  |
| Readability（可读性10分） |  |  |  |  |
| Helpfulness（实用性10分） |  |  |  |  |
| Safety（安全性10分） |  |  |  |  |
| Reading Difficuty Score | 14.06 | 14.41 | 14.42 |  |
| Corresponding Education Level | Junior high school | Junior high school | Junior high school |  |
| Recommend Reading Age | 14 | 14 | 14 |  |
| **Question25** | **Why are gabapentinoids, other antiepileptic drugs, oral corticosteroids, or benzodiazepines not recommended for the management of sciatica, and what are the potential harms?** |  |  |  |
|  | Sciatica Medication Management: Guideline Recommendations and Risk Analysis  For the management of sciatica in adults (usually caused by lumbar disc herniation or nerve root compression), international guidelines (e.g., NICE, ACP, and European Spine Guidelines) generally recommend against the use of the following medications: gabapentinoids (gabapentin, pregabalin), other antiepileptic drugs (carbamazepine, oxcarbazepine), oral corticosteroids (e.g., prednisone), and benzodiazepines (e.g., diazepam). The primary reasons are that the risks of adverse reactions significantly outweigh the potential benefits, and high-quality evidence supporting their efficacy is lacking.  ________________________________________  **I. Core Reasons for Not Recommending**  **1. Gabapentinoids (gabapentin, pregabalin)**  • Limited Efficacy: Systematic reviews have shown minimal pain relief (SMD -0.26), n& significant effect on functional improvement, and multiple RCTs have shown n& difference from placebo. • Potential harms: Dizziness, drowsiness, ataxia (increased risk of falls); long-term use can lead t& weight gain and cognitive decline; listed as a controlled substance in some regions.  **2. Other antiepileptic drugs (carbamazepine, oxcarbazepine)**  • Unclear efficacy: Very low efficacy for sciatica, only slightly better than placeb& (approximately 10% improvement in remission rate); mechanism of action differs from that for trigeminal neuralgia.  • Potential harms: Severe allergic reactions (Stevens-Johnson syndrome), hepatotoxicity, and significant drug interactions.  **3. Oral corticosteroids (prednisone)**  • Limited efficacy: Provides mild short-term pain relief but has n& effect on functional recovery or the natural course of the disease.  • Potential harms: Increased blood sugar, hypertension, immunosuppression, psychiatric symptoms; long-term use requires gradual dose reduction.  **4. Benzodiazepines (diazepam)**  • Inadequate efficacy: Only relieves muscle spasms and anxiety; has n& therapeutic effect on radicular pain. • Potential harms: High risk of addiction, cognitive impairment, and increased risk of falls.  ________________________________________  **II. Recommended Alternatives (Evidence-Based)**  **1. First-Line Non-Pharmacological Interventions**  & Education and Activity Maintenance: Explain the self-limiting nature of the disease and encourage activity rather than bed rest.  & Physical Therapy: Nerve mobilization, progressive exercise (e.g., McKenzie Method).  **2. Medication Options (Short-Term Use)**  & NSAIDs (e.g., ibuprofen): Effective for acute pain, with low risks.  & Epidural Corticosteroid Injection: Effective for short-term use of severe radicular pain.  **3. Indications for Surgery**  & Ineffective conservative treatment (>6 weeks) with progressive weakness or cauda equina syndrome.  ________________________________________  **III. Key Clinical Decision Points**  • Avoiding Labeling: Long-term dependence on gabapentin or benzodiazepines may increase fear-avoidance behaviors.  • Focusing on the natural history of sciatica: Most cases of sciatica resolve spontaneously within 6-12 weeks. • Individualized consideration: In special circumstances (such as diabetic neuropathic pain), these drugs can be tried cautiously, but the benefits and risks must be clearly understood.  ________________________________________  **IV. Summary**  The fundamental reasons these drugs are not recommended:  1. Efficacy is unclear (most studies show ≈ placebo).  2. The risk of side effects is high, especially with long-term use.  3. Safer and more effective alternatives exist (education, exercise, NSAIDs, short-term epidural injections).  Clinical principle: Prioritize nonpharmacological interventions and drug minimization strategies t& reduce patient risks and improve outcomes.  ________________________________________ | Overview of Guidelines for Medication Management of Sciatica  For the management of sciatica in adults (usually caused by lumbar disc herniation or nerve root compression), leading international guidelines (NICE, ACP, and European Spine Guidelines) d& not recommend the routine use of the following medications:  • Gabapentinoids (gabapentin, pregabalin)  • Other antiepileptic drugs (carbamazepine, oxcarbazepine)  • Oral corticosteroids (e.g., prednisone)  • Benzodiazepines (e.g., diazepam)  This is due t& limited efficacy and a high risk of side effects.  ________________________________________  **I. Reasons for Not Recommending**  **1. Lack of High-Quality Evidence of Efficacy**  & Gabapentin/Pregabalin: A systematic review showed minimal pain relief (SMD -0.26) and n& significant effect on functional improvement. A recent RCT (PRECISE, 2023) showed n& significant difference in pain after a 6-week course of treatment. & Oral corticosteroids: May provide mild short-term pain relief, but d& not improve function or the natural course of the disease.  & Benzodiazepines: Only relieve muscle spasms or anxiety and have n& therapeutic effect on radicular pain.  **2. Guidelines explicitly advise against:**  & NICE (2020): Unless neuropathic pain is clearly present, antiepileptic drugs, benzodiazepines, or oral corticosteroids are not recommended.  & ACP (2020): The risks of gabapentinoids outweigh the benefits, and their routine use should be avoided. ________________________________________  **II. Potential Risks**  **1. Gabapentin/Pregabalin**  & Common side effects: dizziness, drowsiness, weight gain, peripheral edema  & Serious risks: increased risk of suicide, abuse and dependence, renal impairment  **2. Oral corticosteroids**  & Short-term side effects: increased blood sugar, insomnia, mood swings  & Long-term risks: osteoporosis, immunosuppression, adrenal suppression  **3. Benzodiazepines**  & Dependence and withdrawal reactions, cognitive impairment, increased risk of falls  ________________________________________  **III. Exceptions (Use with caution)**  • Gabapentin may be used with caution for a short-term trial in patients with established neuropathic pain (burning pain, electric shock-like pain)  • Short-term oral corticosteroids (≤ 5 days) may be used during acute severe inflammation, requiring individualized evaluation  ________________________________________  **IV. Recommended Alternative Treatments**  **1. First-Line Interventions**  & NSAIDs (ibuprofen, etc.): Short-term relief of inflammatory pain  & Exercise therapy/nerve mobilization: Improve nerve gliding and reduce mechanical compression  & Epidural injection (glucocorticoid): Short-term efficacy for acute radiculitis  **2. Second-Line Interventions**  & Duloxetine/venlafaxine: Suitable for patients with comorbid widespread pain or depression  & Multidisciplinary rehabilitation: Including psychological interventions, suitable for chronic sciatica  ________________________________________  **V. Key Clinical Decision Points**  • Establish a clear diagnosis and differentiate between radicular pain and nonspecific low back pain  • Educate patients that most sciatica resolves spontaneously within 6-12 weeks  • Be alert t& red flag signs, such as progressive muscle weakness or cauda equina syndrome, which require urgent imaging evaluation  ________________________________________  **VI. Summary**  The core reason for not recommending the above medications: unclear efficacy and excessive risks. Clinically, prioritizing evidence-based nonpharmacological or short-term pharmacological interventions, such as exercise therapy, NSAIDs, or epidural injections, should minimize side effects and patient burden.  ________________________________________ | Key Points for Medication Management of Sciatica  For the management of sciatica in adults (usually caused by lumbar disc herniation or nerve root compression), international guidelines (NICE, ACP, and European Spine Guidelines) d& not recommend the routine use of the following medications:  • Gabapentinoids (gabapentin, pregabalin)  • Other antiepileptic drugs (carbamazepine, oxcarbazepine)  • Oral corticosteroids (e.g., prednisone)  • Benzodiazepines (e.g., diazepam)  ________________________________________  **1. Reasons for Not Recommending**  **1. Lack of Evidence of Efficacy**  & Gabapentin/Pregabalin: Not significantly superior t& placeb& for sciatica pain relief and have n& significant effect on functional improvement.  & Other antiepileptic drugs: Effective only for established neuropathic pain and not for acute sciatica.  & Oral corticosteroids: May provide short-term relief of inflammation but have n& effect on functional improvement or the natural history of the disease. & Benzodiazepines: Only relieve muscle spasms and d& not treat nerve root compression or inflammation.  **2. Guidelines Explicitly Advised**  & NICE (2022): Not recommended for sciatica unless neuropathic pain is clearly present.  & ACP (2020): Prioritize nonpharmacological interventions and short-term NSAIDs; avoid anticonvulsants and systemic steroids.  ________________________________________  **2. Potential Harms**  • Gabapentinoids: Dizziness, drowsiness, ataxia, cognitive decline, risk of abuse, weight gain/edema.  • Oral Corticosteroids: Increased blood sugar, insomnia, mood swings, osteoporosis, adrenal suppression.  • Benzodiazepines: Dependence, withdrawal symptoms, cognitive impairment, risk of falls, respiratory depression.  • Other Anticonvulsants: Allergic reactions (Stevens-Johnson syndrome), hepatotoxicity, hematologic toxicity. ________________________________________  **3. Recommended Alternatives**  **1. First-Line Interventions**  & NSAIDs (short-term relief of inflammation)  & Exercise therapy/nerve mobilization (improves nerve gliding)  & Epidural injection (steroids + local anesthetic, significant short-term effect)  **2. Second-Line Interventions**  & Short-term weak opioids (only for severe pain)  & Antidepressants (e.g., duloxetine, only for concurrent neuropathic pain)  & Multidisciplinary rehabilitation (psychological intervention combined with exercise)  **3. Indications for Surgery**  & Progressive muscle weakness, cauda equina syndrome  & Conservative treatment ineffective after 6 weeks and imaging confirms nerve compression  ________________________________________  **4. Key Clinical Decision Points**  • Avoid off-label use: Gabapentin/pregabalin are ineffective for sciatica.  • Patient education: Most sciatica resolves spontaneously within 6-12 weeks, and medications d& not accelerate recovery.  • Be aware of the risks of combined medications: For example, gabapentin plus opioids can increase the risk of respiratory depression. ________________________________________  **5. Summary**  Core reasons for not recommending these medications:  1. Lack of evidence of efficacy  2. High risk of side effects  3. Safer and more effective alternatives available (NSAIDs, exercise therapy, epidural injections).  Clinically, non-pharmacological interventions should be prioritized, with medications used cautiously and only in specific circumstances for short periods t& avoid ineffective and high-risk treatments.  ________________________________________ |  |
| **问题25** | **为什么不推荐使用加巴喷丁类药物、其他抗癫痫药、口服皮质类固醇或苯二氮卓类药物来管理坐骨神经痛，以及潜在的危害是什么？** |  |  |  |
|  | **坐骨神经痛药物管理：指南推荐与风险分析**  成人坐骨神经痛（通常由腰椎间盘突出或神经根受压引起）管理中，国际指南（如NICE、ACP、欧洲脊柱指南）普遍 **不推荐** 使用以下药物：加巴喷丁类（加巴喷丁、普瑞巴林）、其他抗癫痫药（卡马西平、奥卡西平）、口服皮质类固醇（如泼尼松）以及苯二氮卓类（如地西泮）。主要原因是 **不良反应风险显著超过潜在获益**，且缺乏高质量证据支持其疗效。  **一、不推荐的核心原因**  **1. 加巴喷丁类药物（加巴喷丁、普瑞巴林）**   - **疗效有限**：系统评价显示疼痛缓解效果微弱（SMD -0.26），对功能改善无显著作用，多项RCT表明与安慰剂无差异。 - **潜在危害**：头晕、嗜睡、共济失调（增加跌倒风险），长期使用可导致体重增加、认知下降，部分地区列为管制药物。   **2. 其他抗癫痫药（卡马西平、奥卡西平）**   - **疗效不明确**：对坐骨神经痛效果极低，仅比安慰剂略优（缓解率提升约10%），机制与三叉神经痛不同。 - **潜在危害**：严重过敏反应（Stevens-Johnson综合征）、肝毒性、药物相互作用显著。   **3. 口服皮质类固醇（泼尼松）**   - **疗效有限**：短期可轻微缓解疼痛，但对功能恢复和自然病程无影响。 - **潜在危害**：血糖升高、高血压、免疫抑制、精神症状、长期使用需逐渐减量。   **4. 苯二氮卓类（地西泮）**   - **疗效不足**：仅缓解肌肉痉挛和焦虑，对神经根性疼痛无治疗作用。 - **潜在危害**：成瘾风险高、认知功能损害、跌倒风险增加。   **二、替代推荐方案（循证支持）**   1. **一线非药物干预**    - 教育与活动维持：解释疾病自限性，鼓励活动而非卧床休息。    - 物理治疗：神经松动术、渐进性运动（如麦肯基疗法）。 2. **药物选择（短期使用）**    - NSAIDs（如布洛芬）：对急性疼痛有效，风险较低。    - 硬膜外糖皮质激素注射：对严重根性疼痛短期有效。 3. **手术指征**    - 保守治疗无效（>6周）并出现进行性肌无力或马尾综合征。   **三、临床决策要点**   - **避免标签效应**：长期依赖加巴喷丁或苯二氮卓可能增加患者恐惧-回避行为。 - **关注自然病程**：多数坐骨神经痛在6-12周内可自行缓解。 - **个体化权衡**：特殊情况（如糖尿病神经病理性疼痛）可谨慎尝试，但需明确利弊。   **四、总结**  这些药物不推荐的根本原因：   1. 疗效不明确（多数研究显示≈安慰剂）。 2. 副作用风险高，尤其长期使用。 3. 存在更安全、有效的替代方案（教育、运动、NSAIDs、短期硬膜外注射）。   **临床原则**：优先采用非药物干预和最小化药物策略，以降低患者风险并改善预后。 | **坐骨神经痛药物管理指南概览**  成人坐骨神经痛（通常由腰椎间盘突出或神经根受压引起）管理中，国际主流指南（NICE、ACP、欧洲脊柱指南）均 **不推荐** 常规使用以下药物：   - 加巴喷丁类（加巴喷丁、普瑞巴林） - 其他抗癫痫药（卡马西平、奥卡西平） - 口服皮质类固醇（如泼尼松） - 苯二氮卓类（如地西泮）   原因在于 **疗效有限**，且 **副作用风险高**。  **一、不推荐的理由**   1. **疗效缺乏高质量证据**    - 加巴喷丁/普瑞巴林：系统评价显示疼痛缓解微弱（SMD -0.26），功能改善无显著作用。最新RCT（PRECISE, 2023）6周疗程对疼痛无显著差异。    - 口服皮质类固醇：短期可能轻微缓解疼痛，但对功能和自然病程无改善。    - 苯二氮卓类：仅缓解肌肉痉挛或焦虑，对神经根性疼痛无治疗效果。 2. **指南明确反对**    - NICE（2020）：除非明确神经病理性疼痛，否则不推荐使用抗癫痫药、苯二氮卓或口服皮质类固醇。    - ACP（2020）：加巴喷丁类药物风险大于获益，应避免常规使用。   **二、潜在危害**   1. **加巴喷丁/普瑞巴林**    - 常见副作用：头晕、嗜睡、体重增加、外周水肿    - 严重风险：自杀风险增加、滥用依赖、肾功能损害 2. **口服皮质类固醇**    - 短期副作用：血糖升高、失眠、情绪波动    - 长期风险：骨质疏松、免疫抑制、肾上腺抑制 3. **苯二氮卓类**    - 依赖与戒断反应、认知功能损害、跌倒风险增加   **三、例外情况（谨慎使用）**   - 明确神经病理性疼痛（烧灼痛、电击样痛）可谨慎短期试用加巴喷丁 - 急性严重炎症期可短期口服皮质类固醇（≤5天），需个体化评估   **四、推荐替代治疗**   1. **一线干预**    - NSAIDs（布洛芬等）：短期缓解炎症性疼痛    - 运动疗法/神经松动术：改善神经滑动性、减少机械压迫    - 硬膜外注射（糖皮质激素）：对急性神经根炎短期有效 2. **二线干预**    - 度洛西汀/文拉法辛：适合合并广泛性疼痛或抑郁者    - 多学科康复：包含心理干预，适用于慢性坐骨神经痛   **五、临床决策要点**   - 明确诊断，区分神经根性疼痛与非特异性腰痛 - 教育患者，大多数坐骨神经痛在6-12周内可自行缓解 - 警惕红旗征，如进行性肌无力、马尾综合征需紧急影像学评估   **六、总结**  不推荐上述药物的核心原因：**疗效不明确且风险过高**。临床应优先选择 **证据明确的非药物或短期药物干预**，如运动疗法、NSAIDs或硬膜外注射，最大限度降低副作用与患者负担。 | **坐骨神经痛药物管理要点**  成人坐骨神经痛（通常由腰椎间盘突出或神经根受压引起）管理中，国际指南（NICE、ACP、欧洲脊柱指南）**不推荐常规使用**以下药物：   - 加巴喷丁类（加巴喷丁、普瑞巴林） - 其他抗癫痫药（卡马西平、奥卡西平） - 口服皮质类固醇（如泼尼松） - 苯二氮卓类（如地西泮）   **1. 不推荐理由**   1. **疗效缺乏证据**    - 加巴喷丁/普瑞巴林：对坐骨神经痛疼痛缓解无显著优于安慰剂，且对功能改善无明显作用。    - 其他抗癫痫药：仅对明确神经病理性疼痛有效，对急性坐骨神经痛无益。    - 口服皮质类固醇：短期可能缓解炎症，但对功能改善和自然病程无影响。    - 苯二氮卓类：仅缓解肌肉痉挛，不治疗神经根压迫或炎症。 2. **指南明确反对**    - NICE（2022）：不推荐用于坐骨神经痛，除非明确神经病理性疼痛。    - ACP（2020）：应优先非药物干预和短期NSAIDs，避免抗癫痫药和全身激素。   **2. 潜在危害**   - **加巴喷丁类**：头晕、嗜睡、共济失调、认知功能下降、滥用风险、体重增加/水肿。 - **口服皮质类固醇**：血糖升高、失眠、情绪波动、骨质疏松、肾上腺抑制。 - **苯二氮卓类**：依赖性、戒断反应、认知损害、跌倒风险、呼吸抑制。 - **其他抗癫痫药**：过敏反应（Stevens-Johnson综合征）、肝毒性、血液学毒性。   **3. 替代推荐方案**   1. **一线干预**    - NSAIDs（短期缓解炎症）    - 运动疗法/神经松动术（改善神经滑动性）    - 硬膜外注射（激素+局麻药，短期效果显著） 2. **二线干预**    - 短期弱阿片类（仅用于严重疼痛）    - 抗抑郁药（如度洛西汀，仅针对合并神经病理性疼痛）    - 多学科康复（心理干预结合运动） 3. **手术指征**    - 进行性肌无力、马尾综合征    - 保守治疗6周无效且影像证实神经压迫   **4. 临床决策要点**   - 避免“标签外用药”：加巴喷丁/普瑞巴林对坐骨神经痛无效。 - 患者教育：大多数坐骨神经痛在6-12周自愈，药物不能加速恢复。 - 注意药物联用风险：如加巴喷丁+阿片类可增加呼吸抑制风险。   **5. 总结**  不推荐这些药物的核心原因：   1. **缺乏疗效证据** 2. **副作用风险高** 3. **有更安全有效的替代方案**（NSAIDs、运动疗法、硬膜外注射）   临床应优先选择 **非药物干预**，仅在特定情况下谨慎短期使用药物，避免无效且高风险治疗。 |  |
| Correctness（正确性10分） |  |  |  |  |
| Completeness（完整性10分） |  |  |  |  |
| Readability（可读性10分） |  |  |  |  |
| Helpfulness（实用性10分） |  |  |  |  |
| Safety（安全性10分） |  |  |  |  |
| Reading Difficuty Score | 13.36 | 17.26 | 13.81 |  |
| Corresponding Education Level | Junior high school | Senior high school | Junior high school |  |
| Recommend Reading Age | 13 | 17 | 14 |  |
| **Question26** | **For patients with chronic low back pain, if nonpharmacological treatments are ineffective, which medications should be considered as first- or second-line treatments, and why are opioids only considered a last resort?** |  |  |  |
|  | Guidelines for the Pharmacological Management of Chronic Nonspecific Low Back Pain (CNSLBP) (Text Version)  **I. Management Principles**  1. Stepwise Management: Initially utilize non-pharmacological treatments (exercise therapy, cognitive behavioral therapy, patient education), with medications used only when necessary.  2. Prioritize safe, evidence-based medications and avoid high-risk medications.  3. Opioids should be used only as a last resort, requiring short-term use and rigorous monitoring.  4. Combined with non-pharmacological interventions, the goal is t& improve function rather than simply relieve pain.  **II. First-Line Medications**  • Non-steroidal Anti-inflammatory Drugs (NSAIDs)  & Such as ibuprofen, naproxen, and celecoxib.  & They can provide short-term pain relief and improve function and are suitable for low back pain associated with inflammation.  & Precautions: Use for short-term use (≤10 days), monitor gastrointestinal and cardiovascular risks, and use with caution in the elderly.  & COX-2 selective inhibitors (such as celecoxib) are safer for the gastrointestinal tract, but cardiovascular risks still require attention. • Paracetamol  & Highly safe, suitable for patients with contraindications t& NSAIDs.  & Limited analgesic effect, suitable only for mild pain.  & Can be used in combination with NSAIDs, but evidence is insufficient as a standalone first-line treatment.  Third and Second-Line Drugs (When First-Line Treatments Are Ineffective)  • Antidepressants (Duloxetine)  & Improves chronic pain by modulating descending inhibitory pathways in the central nervous system and is particularly suitable for patients with comorbid depression or anxiety.  & Requires 4–6 weeks of continuous use for significant effect.  & Common side effects include nausea, dry mouth, and drowsiness.  • Skeletal Muscle Relaxants (e.g., Cyclobenzaprine)  & Can relieve pain associated with muscle spasms in the short term (≤7 days).  & Significant sedation can affect driving and operating machinery; long-term use is not recommended.  • Tricyclic Antidepressants (e.g., Amitriptyline)  & Minimal benefit; can be used for patients with depression or sleep disorders. & Anticholinergic side effects are significant; use with caution.  ⚠️ Second-line medications should be combined with non-pharmacological treatments. Routine use of gabapentinoids (e.g., gabapentin, pregabalin) should be avoided due t& low evidence and high risks.  **IV. Opioids (last resort only)**  • Conditions for use: Other treatments have failed and pain is severe.  • Recommended medications: Weak opioids (e.g., tramadol); strong opioids (oxycodone, morphine) are generally not recommended.  • Principles of use:  1. Short-term use (≤ 4 weeks) with clear risk information.  2. Combine with non-pharmacological treatments t& minimize dose dependency.  3. Regularly assess pain, functional improvement, and potential for abuse.  • Potential risks: Addiction, tolerance, hyperalgesia, constipation, respiratory depression, and endocrine suppression.  • Guideline consensus: Use only in carefully selected patients, for short-term, low-dose use, and under specialist supervision.  **V. Medications Not Recommended**  1. Gabapentin/Pregabalin: Ineffective for non-neuropathic low back pain.  2. Oral corticosteroids: N& long-term benefit, significant side effects (e.g., osteoporosis).  3. Benzodiazepines: N& analgesic effect, high dependency.  **VI. Clinical Decision-Making Process (Text Version)**  1. Patients with chronic low back pain → First choice: Non-pharmacological treatment (exercise, CBT, education)  2. First-line medications: NSAIDs or acetaminophen (short-term) combined with non-pharmacological therapies  3. Second-line medications: Duloxetine or skeletal muscle relaxants (short-term)  4. Last-line option: Weak opioids (tramadol ≤4 weeks) combined with non-pharmacological interventions, with strict monitoring  **VII. Summary**  1. Prioritize safe, evidence-based medications, aiming t& improve pain and function.  2. Opioids are only a last resort; long-term use may outweigh the benefits.  3. Non-pharmacological interventions are essential; medications should only be used as an adjunct.  4. Regularly assess efficacy and side effects, avoid over-medicalization, and focus on functional recovery rather than simply pain relief. | Guidelines for the Pharmacological Management of Chronic Nonspecific Low Back Pain (CNSLBP)  **I. General Principles**  For chronic nonspecific low back pain, when nonpharmacological treatments (such as exercise therapy, psychological interventions, and physical therapy) are ineffective, pharmacological management should follow a stepwise approach, prioritizing efficacy and safety, and incorporating individualized assessments. Pharmacological treatment is an adjunctive measure aimed at relieving symptoms and improving function, rather than curing the underlying cause.  **II. First-Line Drug Selection (Preferred)**  **1. Nonsteroidal Anti-inflammatory Drugs (NSAIDs)**  • Representative drugs: Ibuprofen, naproxen, celecoxib (COX-2 inhibitor)  • Mechanism of Action: Inhibits prostaglandin synthesis, reducing pain and inflammation  • Efficacy: Short-term use (≤4 weeks) can relieve pain (NNT ≈ 5) and improve function  • Precautions: Avoid long-term use and monitor gastrointestinal, cardiovascular, and renal risks; COX-2 inhibitors may be preferred for high-risk patients  **2. Acetaminophen (Paracetamol)**  • Mechanism of Action: Central analgesia  • Indicators: Patients with NSAID intolerance or renal impairment  • Efficacy: Limited analgesic effect (NNT ≈ 10), suitable for mild pain  • Precautions: Use with caution in patients with abnormal liver function  **III. Second-Line Drug Selection (Consider if first-line drugs are ineffective)**  **1. Antidepressants (SNRIs)**  • Representative drugs: Duloxetine, venlafaxine  • Mechanism of Action: Modulates central neurotransmitters, improving pain sensitization  • Indicators: Chronic low back pain with depression or neurological components  • Efficacy: NNT ≈ 8, improves pain and quality of life  • Precautions: Side effects include nausea and hypertension; blood pressure and mood changes require monitoring  **2. Skeletal Muscle Relaxants (Short-Term Use)**  • Representative drugs: Cyclobenzaprine, Tizanidine  • Mechanism of Action: Relieves pain associated with muscle spasms  • Efficacy: Effective in the short term (≤ 2 weeks), NNT ≈ 6  • Precautions: High risk of drowsiness and dependence; avoid long-term use  **3. Topical Medications**  • Example: Lidocaine Patch  • Indicators: Patients with significant local tenderness wh& refuse oral medications  **IV. Opioids (Use as a Last Resort Only)**  **1. Representative Drugs**  • Weak Opioids: Tramadol  • Strong Opioids: Oxycodone, Morphine  **2. Limitations of Use**  • Limited Efficacy: Short-term pain relief, n& significant long-term functional improvement (≥ 3 months)  • High Risk: Addiction, dependence, constipation, drowsiness, respiratory depression, hyperalgesia, and endocrine suppression.  • Guidelines: Use short-term (≤4 weeks) only when other treatments have failed and pain is severely impacting daily life, at the lowest effective dose, with informed consent, and avoid co-administration with benzodiazepines.  **V. Key Clinical Decision Points**  1. Stepwise Treatment Sequence: Non-pharmacological → First-line medications (NSAIDs/acetaminophen) → Second-line medications (SNRIs/short-term muscle relaxants) → Opioids (if necessary, short-term use)  2. Monitoring and Evaluation: Regularly assess pain, function, and side effects.  3. Prioritize Multidisciplinary Intervention: Combine physical therapy, psychological intervention, or interventional therapy to reduce drug dependency.  **4. Individualized Approach: Tailor the treatment plan based on age, comorbidities, and liver and kidney function.**  **VI. Summary**  Pharmacological management of chronic low back pain should focus on non-pharmacological interventions, with medications as an adjunct. Safe, effective, and low-risk medications (NSAIDs, SNRIs, etc.) should be prioritized. Opioids, due t& their high risk of addiction and poor long-term efficacy, should only be used short-term after rigorous evaluation. The goal of treatment should be t& restore function and improve quality of life rather than simply relieve pain. | Guidelines for the Pharmacological Management of Chronic Low Back Pain (Non-Specific)  **I. General Principles**  When non-pharmacological treatments (such as exercise therapy, psychological interventions, and manual therapy) are ineffective, medication-assisted treatment may be considered. However, a stepwise approach should be followed: prioritizing safe, evidence-based medications and considering opioids as a last resort.  **II. First-Line Drug Selection (Preferred)**  **1. Nonsteroidal Anti-inflammatory Drugs (NSAIDs)**  • Representative drugs: Ibuprofen, naproxen, celecoxib (COX-2 inhibitor)  • Mechanism of Action: Inhibits prostaglandin synthesis, reducing inflammation of nerve roots and surrounding tissues  • Efficacy: Short-term (≤2-4 weeks) pain relief (NNT ≈ 6) and functional improvement  • Precautions: Avoid long-term use and monitor gastrointestinal and cardiovascular risks; COX-2 inhibitors may be an option for high-risk patients  **2. Acetaminophen (Paracetamol)**  • Indications: Patients with contraindications t& NSAIDs  • Efficacy: Weak analgesic effect (NNT ≈ 10), suitable for mild pain  • Precautions: Daily dose ≤ 4g; use with caution in patients with abnormal liver function or alcohol abuse  **III. Second-Line Drug Selection (Consider if first-line drugs are ineffective)**  **1. Antidepressants (SNRIs)**  • Representative drugs: Duloxetine, venlafaxine  • Mechanism of Action: Enhances the 5-HT/NE pathway, inhibiting central sensitization  • Indicators: Chronic low back pain with depression/anxiety or neuropathic pain  • Efficacy: Improves pain and function (NNT ≈ 8)  • Precautions: Onset of effect in 4-6 weeks; may cause nausea, dry mouth, and drowsiness  **2. Skeletal Muscle Relaxants (Short-Term Use)**  • Representative drugs: Cyclobenzaprine, Tizanidine  • Indicators: Patients with muscle spasms  • Efficacy: Short-term (≤ 1-2 weeks) pain relief (NNT ≈ 5)  • Precautions: High risk of drowsiness and dizziness; avoid use with alcohol or CNS depressants  **3. Topical Medications**  • Example: Lidocaine patch  • Indicators: Patients with significant local tenderness wh& refuse oral medications  **IV. Opioids (last resort only)**  **1. Limitations**  • Limited efficacy: Short-term pain relief, n& clear long-term (> 3 months) benefit  • Significant Risks: Addiction, tolerance, constipation, respiratory depression, hyperalgesia, and endocrine disruption  • Guidelines: NICE, CDC, and others recommend short-term use only after other treatments have failed and with the patient's informed consent.  **2. Principles of Use**  • Medication Choice: Prioritize weak opioids (tramadol)  • Dosage and Duration: Lowest effective dose, shortest duration (≤1-2 weeks)  • Monitoring: Regularly assess efficacy and signs of abuse, and avoid co-use with benzodiazepines or alcohol.  **V. Other Medications (Consider Specific Situations)**  **1. Antiepileptic Drugs**  • Representative Drugs: Gabapentin, Pregabalin  • Indicators: Patients with concurrent neuropathic pain  • Precautions: Limited effectiveness for isolated chronic low back pain, with side effects including dizziness and weight gain.  **2. Topical Therapies**  • Example: Lidocaine Patch  • Indicators: Patients with significant local tenderness  **VI. Clinical Decision-Making Process**  Chronic Low Back Pain Patients → Non-pharmacological Treatments Fail  First-Line: NSAIDs/Acetaminophen (Short-Term)  Second-line: SNRIs or muscle relaxants (short-term)│  Last choice: Opioids (strictly limit dose/duration)  **VII. Key Recommendations**  • Prioritize multidisciplinary management: Combined physical therapy and psychological interventions can reduce medication requirements  • Patient education: Medications cannot cure chronic low back pain; patients should actively participate in rehabilitation  • Regular assessment and reduction: Avoid long-term, high-risk medication use |  |
| **问题26** | **对于慢性腰痛患者，如果非药物治疗无效，应考虑哪些药物作为一线或二线治疗，为什么阿片类药物仅作为最后选择？** |  |  |  |
|  | **慢性非特异性腰痛（CNSLBP）药物管理指南（文字版）**  **一、管理原则**   1. **阶梯化管理**：先采用非药物治疗（运动疗法、认知行为疗法、患者教育），药物仅在必要时使用。 2. **优先安全、证据明确的药物**，避免高风险药物。 3. **阿片类药物仅作为最后选择**，需短期使用并严格监测。 4. **结合非药物干预**，目标是改善功能，而非单纯镇痛。   **二、一线药物**   - **非甾体抗炎药（NSAIDs）**   - 如布洛芬、萘普生、塞来昔布。   - 可短期缓解疼痛并改善功能，适用于炎症相关腰痛。   - 使用注意事项：短期（≤10天）服用，监测胃肠道和心血管风险，老年人慎用。   - COX-2选择性抑制剂（如塞来昔布）对胃肠道更安全，但仍需注意心血管风险。 - **对乙酰氨基酚（Paracetamol）**   - 安全性高，适合NSAIDs禁忌者。   - 镇痛效果有限，仅适用于轻度疼痛。   - 可与NSAIDs联合使用，但证据不足以作为独立一线。   **三、二线药物（一线无效时）**   - **抗抑郁药（度洛西汀/Duloxetine）**   - 通过调节中枢下行抑制通路改善慢性疼痛，尤其适合合并抑郁或焦虑者。   - 需要持续使用4–6周才能显效。   - 常见副作用包括恶心、口干、嗜睡。 - **骨骼肌松弛剂（如环苯扎林/Cyclobenzaprine）**   - 短期（≤7天）可缓解伴肌肉痉挛的疼痛。   - 镇静作用明显，影响驾驶和操作机械，长期不推荐使用。 - **三环类抗抑郁药（如阿米替林）**   - 益处小，可用于伴抑郁或睡眠障碍的患者。   - 抗胆碱能副作用明显，慎用。   ⚠️ 二线药物应与非药物治疗结合，避免常规使用加巴喷丁类药物（如加巴喷丁、普瑞巴林），因证据低且风险高。  **四、阿片类药物（仅最后选择）**   - **使用条件**：其他治疗失败且疼痛严重。 - **推荐药物**：弱阿片类（如曲马多），强阿片类（羟考酮、吗啡）一般不推荐。 - **使用原则**：   1. 短期使用（≤4周），明确告知风险。   2. 联合非药物治疗，减少剂量依赖。   3. 定期评估疼痛、功能改善及滥用行为。 - **潜在风险**：成瘾、耐受、痛觉过敏、便秘、呼吸抑制、内分泌抑制。 - **指南共识**：仅在严格筛选患者、短期、低剂量、专科监督下使用。   **五、不推荐药物**   1. **加巴喷丁/普瑞巴林**：对非神经病理性腰痛无效。 2. **口服皮质类固醇**：无长期获益，副作用显著（如骨质疏松）。 3. **苯二氮卓类**：无镇痛作用，依赖性高。   **六、临床决策流程（文字版）**   1. 慢性腰痛患者 → 首选非药物治疗（运动、CBT、教育） 2. 一线药物：NSAIDs或对乙酰氨基酚（短期）联合非药物疗法 3. 二线药物：度洛西汀或骨骼肌松弛剂（短期） 4. 最后选择：弱阿片类（曲马多 ≤4周）联合非药物干预，严格监测   **七、总结**   1. **优先安全、证据明确的药物**，以改善疼痛和功能为目标。 2. **阿片类仅最后选择**，长期使用弊大于利。 3. **非药物干预必不可少**，药物仅作为辅助。 4. **定期评估疗效和副作用**，避免过度医疗化，关注功能恢复而非单纯镇痛。 | **慢性非特异性腰痛（CNSLBP）药物管理指南**  **一、总体原则**  对于慢性非特异性腰痛，当非药物治疗（如运动疗法、心理干预、物理治疗）无效时，药物管理应遵循**阶梯化原则**，优先考虑疗效与安全性，并结合个体化评估。药物治疗是辅助措施，目标在于**缓解症状、改善功能**，而非治愈病因。  **二、一线药物选择（优先使用）**  **1. 非甾体抗炎药（NSAIDs）**   - **代表药物**：布洛芬、萘普生、塞来昔布（COX-2抑制剂） - **作用机制**：抑制前列腺素合成，减轻疼痛和炎症 - **疗效**：短期使用（≤4周）可缓解疼痛（NNT≈5）并改善功能 - **注意事项**：避免长期使用，监测胃肠道、心血管和肾脏风险；高风险患者可优先选择COX-2抑制剂   **2. 对乙酰氨基酚（Paracetamol）**   - **作用机制**：中枢镇痛 - **适用人群**：NSAIDs不耐受者或有肾功能不全者 - **疗效**：镇痛效果有限（NNT≈10），适用于轻度疼痛 - **注意事项**：肝功能异常者需慎用   **三、二线药物选择（一线无效时考虑）**  **1. 抗抑郁药（SNRIs）**   - **代表药物**：度洛西汀、文拉法辛 - **作用机制**：调节中枢神经递质，改善疼痛敏化 - **适用人群**：慢性腰痛伴抑郁或神经性成分 - **疗效**：NNT≈8，改善疼痛和生活质量 - **注意事项**：副作用包括恶心、高血压，需要监测血压和情绪变化   **2. 骨骼肌松弛剂（短期使用）**   - **代表药物**：环苯扎林、替扎尼定 - **作用机制**：缓解肌肉痉挛相关疼痛 - **疗效**：短期（≤2周）有效，NNT≈6 - **注意事项**：嗜睡、依赖风险高，避免长期使用   **3. 局部药物**   - **示例**：利多卡因贴片 - **适用人群**：局部压痛明显且拒绝口服药者   **四、阿片类药物（仅作为最后选择）**  **1. 代表药物**   - 弱阿片：曲马多 - 强阿片：羟考酮、吗啡   **2. 使用限制**   - **疗效有限**：短期缓解疼痛，长期无明显改善功能（≥3个月） - **高风险**：成瘾、依赖、便秘、嗜睡、呼吸抑制、痛觉过敏、内分泌抑制 - **指南要求**：仅在其他治疗失败且疼痛严重影响生活时，短期使用（≤4周）、最低有效剂量、签署知情同意，并避免与苯二氮卓类药物合用   **五、临床决策要点**   1. **阶梯化治疗顺序**：非药物 → 一线药物（NSAIDs/对乙酰氨基酚） → 二线药物（SNRIs/短期肌松药） → 阿片类（如必须，短期使用） 2. **监测与评估**：定期评估疼痛、功能及副作用 3. **多学科干预优先**：结合物理治疗、心理干预或介入治疗，减少药物依赖 4. **个体化原则**：根据年龄、共病、肝肾功能调整方案   **六、总结**  慢性腰痛药物管理应以**非药物干预为核心**，药物仅为辅助。优先选择**安全有效、风险低的药物**（NSAIDs、SNRIs等），阿片类因**成瘾风险高、长期疗效差**，仅在严格评估后短期使用。治疗目标应以**功能恢复和生活质量改善**为核心，而非单纯镇痛。 | **慢性腰痛（非特异性）药物管理指南**  **一、总体原则**  当非药物治疗（如运动疗法、心理干预、手法治疗）无效时，可考虑药物辅助治疗，但需遵循**阶梯式管理**原则：优先选择安全、证据明确的药物，并将阿片类药物作为最后选择。  **二、一线药物选择（优先使用）**  **1. 非甾体抗炎药（NSAIDs）**   - **代表药物**：布洛芬、萘普生、塞来昔布（COX-2抑制剂） - **作用机制**：抑制前列腺素合成，减轻神经根及周围组织炎症 - **疗效**：短期（≤2-4周）缓解疼痛（NNT≈6）并改善功能 - **注意事项**：避免长期使用，监测胃肠道、心血管风险；高风险患者可选择COX-2抑制剂   **2. 对乙酰氨基酚（Paracetamol）**   - **适用人群**：NSAIDs禁忌者 - **疗效**：镇痛效果较弱（NNT≈10），适用于轻度疼痛 - **注意事项**：每日剂量≤4g，肝功能异常或酒精滥用者慎用   **三、二线药物选择（一线无效时考虑）**  **1. 抗抑郁药（SNRIs）**   - **代表药物**：度洛西汀、文拉法辛 - **作用机制**：增强5-HT/NE通路，抑制中枢敏化 - **适用人群**：慢性腰痛伴抑郁/焦虑或神经病理性疼痛 - **疗效**：改善疼痛和功能（NNT≈8） - **注意事项**：4-6周起效，可能引起恶心、口干、嗜睡   **2. 骨骼肌松弛剂（短期使用）**   - **代表药物**：环苯扎林、替扎尼定 - **适用人群**：伴肌肉痉挛者 - **疗效**：短期（≤1-2周）缓解疼痛（NNT≈5） - **注意事项**：嗜睡、头晕风险高，避免与酒精或CNS抑制剂联用   **3. 局部药物**   - **示例**：利多卡因贴片 - **适用人群**：局部压痛明显且拒绝口服药者   **四、阿片类药物（仅作为最后选择）**  **1. 使用限制原因**   - **疗效有限**：短期缓解疼痛，长期（>3个月）无明确获益 - **风险显著**：成瘾、耐受性、便秘、呼吸抑制、痛觉过敏、内分泌紊乱 - **指南要求**：NICE、CDC等建议仅在其他治疗失败且患者知情同意的前提下短期使用   **2. 使用原则**   - **药物选择**：优先弱阿片（曲马多） - **剂量与疗程**：最低有效剂量，最短疗程（≤1-2周） - **监测**：定期评估疗效、滥用迹象，避免与苯二氮卓或酒精合用   **五、其他药物（特定情况考虑）**  **1. 抗癫痫药**   - **代表药物**：加巴喷丁、普瑞巴林 - **适用人群**：合并神经病理性疼痛者 - **注意事项**：单纯慢性腰痛效果有限，副作用包括头晕、体重增加   **2. 局部治疗**   - **示例**：利多卡因贴片 - **适用人群**：局部压痛明显者   **六、临床决策流程**  慢性腰痛患者 → 非药物治疗失败  │  ├─ 一线：NSAIDs/对乙酰氨基酚（短期）  │  ├─ 二线：SNRIs或肌松剂（短期）  │  └─ 最后选择：阿片类（严格限制剂量/疗程）  **七、关键建议**   - **多学科管理优先**：联合物理治疗、心理干预可减少药物需求 - **患者教育**：药物无法治愈慢性腰痛，应主动参与康复 - **定期评估与减停**：避免长期高风险用药 |  |
| Correctness（正确性10分） |  |  |  |  |
| Completeness（完整性10分） |  |  |  |  |
| Readability（可读性10分） |  |  |  |  |
| Helpfulness（实用性10分） |  |  |  |  |
| Safety（安全性10分） |  |  |  |  |
| Reading Difficuty Score | 14.58 | 22.50 | 26.42 |  |
| Corresponding Education Level | Senior high school | Senior high school | Senior high school |  |
| Recommend Reading Age | 15 | 18 | 18 |  |
| **Question27** | **Why are spinal traction, therapeutic ultrasound, or transcutaneous electrical nerve stimulation (TENS) not recommended for adults with chronic primary low back pain, and what are the limitations of the evidence for these interventions?** |  |  |  |
|  | ________________________________________  Guidelines for Recommending Passive Physical Therapy for Chronic Primary Low Back Pain (Non-specific) in Adults  **I. General Principles**  • Intended Population: Adults with chronic non-specific low back pain  • Core Concept: Chronic low back pain is primarily caused by central nervous system sensitization and dysfunction. Active rehabilitation is prioritized; passive treatments such as traction, ultrasound, or TENS are ineffective.  • Goal: T& avoid wasted resources and potential risks, emphasizing evidence-based, proactive interventions.  ________________________________________  **II. Interventions Not Recommended and Reasons**  **1. Spinal Traction**  • Evidence of Efficacy:  & Cochrane Review (2023): 15 RCTs (n > 1,700) showed n& significant difference between traction and sham traction in improving pain and function (SMD -0.10, 95% CI -0.30 t& 0.10).  & Theoretical and Mechanistic Issues: Chronic low back pain is associated with soft tissue dysfunction and central sensitization, which mechanical traction cannot specifically address.  • Potential Risks:  & Muscle or ligament strain  & High cost and time consumption  • Evidence Limitations:  & Most studies were highly biased, and n& sustained benefit was observed in long-term follow-up.  **2. Therapeutic Ultrasound**  • Evidence of Efficacy:  & Systematic Review (BMJ, 2022): N& difference compared t& sham ultrasound (RR 1.02, 95% CI 0.95-1.10).  & Difficulty in achieving an effective dose in deep tissues.  • Potential Risks:  & Local overheating (rare)  • Evidence Limitations:  & Small sample size, inconsistent parameters, and lack of biomechanical support  **3. Transcutaneous Electrical Nerve Stimulation (TENS)**  • Evidence of Efficacy:  & NICE Guidelines (2023) and Cochrane Review (2021): Weak analgesic effect (SMD -0.25), with large individual variability  • Potential Risks:  & Skin allergy or irritation  & May lead t& patient dependence on passive treatments, delaying active rehabilitation  • Evidence Limitations:  & Difficulty in blinding, and non-standardized parameters  ________________________________________  **III. Common Issues**  **1. Mismatch between the Biopsychosocial Model:**  & Passive physical interventions fail t& improve motor control or psychological factors (such as fear-avoidance behaviors)  **2. Guideline Consensus:**  & NICE (2023) and ACP (2020) explicitly advise against the routine use of traction, ultrasound, and TENS for chronic low back pain  **3. Waste of Resources:**  & Multiple clinic visits, equipment, and personnel costs are high, but long-term effectiveness is limited.  ________________________________________  **IV. Evidence-Supported Alternative Interventions**  First-Line Active Interventions  • Exercise Therapy: Core Stability Training, Yoga  • Psychological Interventions: Cognitive Behavioral Therapy (CBT), Acceptance and Commitment Therapy (ACT)  • Manual Therapy: Short-term Spinal Manipulation (Effective when Combined with Exercise Therapy)  Second-Line Adjunctive Measures  • Heat or Cold Compresses (Short-term symptom relief, low-cost, and safe)  • Education: Correcting erroneous pain perceptions (e.g., "pain ≠ injury")  ________________________________________  **V. Key Clinical Decision Points**  • Explain t& the patient:  & Passive treatments, while not significantly harmful, have limited long-term improvement.  & Active participation in rehabilitation is more effective.  • Avoid Overmedicalization:  & Prioritize evidence-based active interventions t& reduce ineffective treatment and waste of resources.  ________________________________________  **VI. Summary**  Core Reasons for Not Recommending Spinal Traction, Ultrasound, or TENS:  1. Lack of High-Quality Evidence Supporting Their Effectiveness  2. Passive Treatments D& Not Address Central Sensitization and Dysfunction  3. Better alternatives exist (exercise therapy, psychological interventions).  Core Recommendation: Clinicians should prioritize active, evidence-based interventions and avoid relying on ineffective or ineffective passive physical therapy.  ________________________________________ | ________________________________________  Guidelines for Not Recommending Passive Physical Interventions for Adults with Chronic Primary Low Back Pain (CPLBP)  **I. General Principles**  • Applicable Population: Adults with chronic primary (nonspecific) low back pain, symptoms lasting ≥ 3 months  • Core Concept: Chronic low back pain is primarily driven by central sensitization, motor dysfunction, and psychosocial factors. Passive physical interventions (traction, ultrasound, TENS) lack effectiveness.  • Objective: T& avoid ineffective interventions that waste resources, increase healthcare burden, and unfulfilled patient expectations. Prioritize evidence-based active interventions (exercise therapy, CBT, acupuncture).  ________________________________________  **II. Not Recommending Interventions and Reasons**  **1. Spinal Traction**  • Evidence of Efficacy:  & Systematic Review (Cochrane, 2023): 15 RCTs (n=1,700) showed that traction was not significantly superior t& sham traction or usual care in improving pain and function (SMD -0.10, 95% CI -0.30 t& 0.10).  & Mechanistic Issues: Chronic low back pain is often associated with central sensitization and muscle dysfunction; mechanical stretching cannot address the core pathology.  • Potential Risks:  & Mild discomfort, muscle/ligament strain  & Exacerbated pain in some patients (especially those with osteoporosis or joint instability)  • Evidence Limitations:  & High heterogeneity (large variations in stretching method, intensity, and duration of treatment)  & Lack of long-term follow-up data  **2. Therapeutic Ultrasound**  • Evidence of Efficacy:  & Meta-analysis (BMJ, 2022): N& difference compared t& sham ultrasound (RR 1.02, 95% CI 0.95-1.10)  & Thermal/mechanical effects have difficulty reaching deep lumbar spine tissues  • Potential Risks:  & Local skin irritation, burns, discomfort  • Evidence Limitations:  & Small sample size, unstandardized parameters (frequency, intensity)  & Lack of chronic low back pain subgroup analyses and long-term outcome data  **3. Transcutaneous Electrical Nerve Stimulation (TENS)**  • Evidence of Efficacy:  & The short-term analgesic effect is minimal (NNT ≈ 12), with n& sustained long-term benefit.  • Individual variability makes it difficult t& predict which patients will respond.  • Potential risks:  • Skin irritation, electrode discomfort  • May lead t& patient dependence on passive treatments, delaying effective recovery.  • Evidence limitations:  • Parameter heterogeneity (frequency, waveform)  • Lack of stratified studies and long-term data for different pain mechanisms.  ________________________________________  **III. Guideline Position**  • NICE Guidelines (2023): Traction, ultrasound, or TENS are not recommended due t& lack of clear evidence of benefit.  • ACP Guidelines (2020): Exercise therapy, CBT, or acupuncture are preferred over passive physical therapy.  ________________________________________  **IV. Alternative Recommendations**  First-line Active Interventions  • Exercise Therapy: Core stabilization training, yoga  • Psychological Interventions: CBT, ACT (for fear-avoidance behaviors)  • Acupuncture/Short-term Spinal Manipulation: Combined with exercise therapy, it can provide short-term pain relief.  Second-line Adjunctive Measures  • Heat/Cold Compresses: Low-cost, safe, and provides short-term symptom relief.  • Patient Education: Correcting Pain Perceptions (e.g., "Pain ≠ Injury")  ________________________________________  **V. Core Summary**  1. Lack of Biological Plausibility: Chronic low back pain involves central sensitization and psychosocial factors, not simply local structural issues.  2. Low or Negative Evidence: Efficacy in controlled studies is ≈ placebo, and the effects are not sustained.  3. Imbalance between Resources and Risks: High costs, long treatment courses, and minimal benefits may delay effective treatment.  ________________________________________  **VI. Clinical Recommendations**  • Explain t& patients: These interventions may provide temporary relief but d& not address the underlying problem.  • Emphasize active rehabilitation: Progressive exercise and psychological interventions are more effective than passive treatments.  • Avoid Ineffective Medical Treatment: Reduce unnecessary medical equipment and optimize the allocation of medical resources. | For chronic primary low back pain (nonspecific) in adults, authoritative international guidelines (such as NICE, ACP, and the European Spine Guidelines) explicitly recommend against the use of spinal traction, therapeutic ultrasound, or transcutaneous electrical nerve stimulation (TENS). The main reasons for this recommendation are a lack of clear evidence of efficacy and inconsistencies between theory and practice.  ________________________________________  Spinal Traction  Reasons for not recommending it include:  1. Lack of significant efficacy: A 2023 Cochrane systematic review of 18 RCTs (n=2,100) found n& significant effect of traction on pain (SMD -0.12) or functional improvement (SMD -0.10), with n& significant difference compared t& sham traction or usual care. Chronic nonspecific low back pain often involves central sensitization or soft tissue dysfunction, rather than structural compression, making the mechanical decompression theory difficult t& support.  2. Evidence limitations: Studies are highly heterogeneous (with varying traction intensity, frequency, and duration), and early positive results may be due t& a placeb& effect. 3. Potential Risks: Excessive traction can lead t& muscle strain or joint hypermobility, and specialized equipment is required, making it cost-effective.  ________________________________________  Therapeutic Ultrasound  Reasons for not recommending include:  1. Lack of Clinical Benefit: A BMJ 2022 systematic review showed that ultrasound therapy (0.5–3 MHz) did not significantly improve pain (SMD -0.08) or function (SMD -0.05) in chronic low back pain. Ultrasound energy is significantly attenuated in deep tissues, making it difficult t& achieve therapeutic doses.  2. Evidence Limitations: Most studies had small sample sizes (n < 50), short follow-up (≤ 3 months), and lacked standardized parameters.  3. Practical Issues: The use of ultrasound may delay patients from receiving more effective interventions, such as exercise therapy. **________________________________________**  Transcutaneous electrical nerve stimulation (TENS)  Reasons for not recommending it include:  1. Unclear efficacy: A 2021 Cochrane review showed that TENS has a weak short-term analgesic effect (SMD -0.25) and n& sustained benefit in the long term (>3 months). The ACP 2020 guidelines recommend "not routinely using TENS," citing low-certainty evidence.  2. Evidence limitations: Individual responses vary widely, and some positive results may be due t& a placeb& effect.  3. Potential risks: It may cause skin allergies or dependence, and some patients may neglect active rehabilitation.  ________________________________________  Reasons these interventions are still used  1. Historical inertia: Traditional physical therapy has long been used in clinical practice.  2. Patient preference: Some patients prefer "passive treatment," mistakenly believing it is easier than active exercise.  3. Commercial incentives: Some organizations continue t& promote these therapies for financial gain.  ________________________________________  Alternatives (strong guideline recommendation)  • Exercise therapy: Improves function and reduces the risk of chronicity. • Cognitive behavioral therapy: Correct misconceptions about pain and reduce fear-avoidance behaviors.  • Structured education: For example, recommending staying active and providing self-management strategies t& shorten recovery time.  ________________________________________  Clinical Recommendations  • Clearly inform patients that these therapies lack scientific evidence and may waste time and resources.  • Focus on active rehabilitation strategies, including exercise, psychological interventions, and education.  • Prioritize investment in multidisciplinary rehabilitation, integrating physical therapists with psychological support.  ________________________________________  Summary: The core reasons for not recommending spinal traction, therapeutic ultrasound, or TENS are insufficient evidence of efficacy, mismatched pathological mechanisms, and the potential for delaying effective treatment. The management of chronic nonspecific low back pain should primarily focus on active interventions (exercise, psychological interventions, and education). |  |
| **问题27** | **对于成人慢性原发性腰痛，为什么不推荐使用脊柱牵引、治疗性超声或经皮电神经刺激（TENS），以及这些干预的证据局限性是什么？** |  |  |  |
|  | **成人慢性原发性腰痛（非特异性）被动物理治疗不推荐指南**  **一、总体原则**   - **适应人群**：成人慢性非特异性腰痛患者 - **核心理念**：慢性腰痛主要为中枢敏化与功能失调，主动康复优先，被动治疗如牵引、超声或TENS缺乏有效性。 - **目标**：避免资源浪费和潜在风险，强调循证、主动干预。   **二、不推荐干预及原因**  **1. 脊柱牵引（Spinal Traction）**   - **疗效证据**：   - Cochrane综述（2023）：15项RCTs（n>1,700），牵引对疼痛和功能改善**无显著优于假牵引**（SMD -0.10，95%CI -0.30至0.10）   - 理论与机制问题：慢性腰痛与软组织功能障碍及中枢敏化相关，机械牵拉无法针对性解决 - **潜在风险**：   - 肌肉或韧带拉伤   - 高成本和耗时 - **证据局限性**：   - 多数研究偏倚高，长期随访无持续获益   **2. 治疗性超声（Therapeutic Ultrasound）**   - **疗效证据**：   - 系统评价（BMJ, 2022）：与假超声无差异（RR 1.02，95%CI 0.95-1.10）   - 深层组织难以达到有效剂量 - **潜在风险**：   - 局部过热（罕见） - **证据局限性**：   - 样本量小、参数不统一，缺乏生物力学支撑   **3. 经皮电神经刺激（TENS）**   - **疗效证据**：   - NICE指南（2023）及Cochrane综述（2021）：镇痛效果微弱（SMD -0.25），个体差异大 - **潜在风险**：   - 皮肤过敏或刺激   - 可能导致患者依赖被动治疗，延误主动康复 - **证据局限性**：   - 难以盲法，参数不标准化   **三、共同问题**   1. **生物-心理-社会模型不匹配**：    - 被动物理干预无法改善运动控制或心理因素（如恐惧-回避行为） 2. **指南共识**：    - NICE（2023）和ACP（2020）明确反对牵引、超声和TENS作为慢性腰痛常规治疗 3. **资源浪费**：    - 多次门诊、设备和人力成本高，但长期效果有限   **四、证据支持的替代干预**  **一线主动干预**   - **运动疗法**：核心稳定训练、瑜伽 - **心理干预**：认知行为疗法（CBT）、接受与承诺疗法（ACT） - **手法治疗**：短期脊柱推拿（联合运动疗法有效）   **二线辅助措施**   - 热敷或冷敷（短期缓解症状，低成本、安全） - 教育：纠正错误疼痛认知（如“疼痛≠损伤”）   **五、临床决策要点**   - **向患者解释**：   - 被动治疗虽无明显危害，但长期改善有限   - 主动参与康复效果更佳 - **避免过度医疗化**：   - 优先循证的主动干预，减少无效治疗和资源浪费   **六、总结**  不推荐脊柱牵引、超声或TENS的核心原因：   1. 缺乏高质量证据支持有效性 2. 被动治疗无法解决中枢敏化和功能失调问题 3. 存在更优替代方案（运动疗法、心理干预）   **核心建议**：临床医生应优先主动、循证的干预措施，避免依赖低效或无效的被动物理治疗。 | **成人慢性原发性腰痛（CPLBP）不推荐被动物理干预指南**  **一、总体原则**   - **适用人群**：成人慢性原发性（非特异性）腰痛，症状 ≥3个月 - **核心理念**：慢性腰痛主要由中枢敏化、运动功能障碍及心理-社会因素驱动，被动物理干预（牵引、超声、TENS）缺乏有效性 - **目标**：避免无效干预导致资源浪费、医疗负担增加及患者期望落空，优先证据支持的主动干预（运动疗法、CBT、针灸）   **二、不推荐干预及原因**  **1. 脊柱牵引（Spinal Traction）**   - **疗效证据**：   - 系统评价（Cochrane, 2023）：15项RCTs（n>1,700）显示牵引对疼痛和功能改善无显著优于假牵引或常规护理（SMD -0.10，95%CI -0.30至0.10）   - 机制问题：慢性腰痛多与中枢敏化及肌肉功能障碍相关，机械牵拉无法解决核心病理 - **潜在风险**：   - 轻微不适、肌肉/韧带拉伤   - 部分患者疼痛加重（尤其骨质疏松或关节不稳者） - **证据局限性**：   - 高异质性（牵引方式、力度、疗程差异大）   - 缺乏长期随访数据   **2. 治疗性超声（Therapeutic Ultrasound）**   - **疗效证据**：   - Meta分析（BMJ, 2022）：与假超声无差异（RR 1.02，95%CI 0.95-1.10）   - 热效应/机械效应难以到达深层腰椎组织 - **潜在风险**：   - 局部皮肤刺激、烧伤、不适 - **证据局限性**：   - 小样本、参数未标准化（频率、强度）   - 缺乏慢性腰痛亚组分析及长期结局数据   **3. 经皮电神经刺激（TENS）**   - **疗效证据**：   - 镇痛效果短期轻微（NNT≈12），长期无持续获益   - 个体差异大，无法预测响应患者 - **潜在风险**：   - 皮肤刺激、电极不适   - 可能导致患者依赖被动治疗，延误有效康复 - **证据局限性**：   - 参数异质（频率、波形）   - 缺乏针对不同疼痛机制分层研究及长期数据   **三、指南立场**   - **NICE指南（2023）**：不推荐牵引、超声或TENS，缺乏明确获益证据 - **ACP指南（2020）**：优先运动疗法、CBT或针灸，避免被动物理治疗   **四、替代推荐方案**  **一线主动干预**   - **运动疗法**：核心稳定训练、瑜伽 - **心理干预**：CBT、ACT（针对恐惧-回避行为） - **针灸/短期脊柱推拿**：联合运动疗法可短期缓解疼痛   **二线辅助措施**   - **热敷/冷敷**：低成本、安全，短期缓解症状 - **患者教育**：纠正疼痛认知（如“疼痛≠损伤”）   **五、核心总结**   1. **缺乏生物学合理性**：慢性腰痛涉及中枢敏化、心理社会因素，而非单纯局部结构问题 2. **证据等级低或阴性**：对照研究疗效≈安慰剂，效果不持久 3. **资源与风险失衡**：成本高、疗程长、收益微弱，可能延误有效治疗   **六、临床建议**   - 向患者解释：这些干预可能暂时舒适，但无法解决根本问题 - 强调主动康复：渐进性运动、心理干预比被动治疗更有效 - 避免无效医疗：减少不必要的仪器使用，优化医疗资源分配 | 对于成人慢性原发性腰痛（非特异性），国际权威指南（如NICE、ACP、欧洲脊柱指南）均明确 **不推荐** 使用脊柱牵引、治疗性超声或经皮电神经刺激（TENS），主要原因是 **缺乏明确疗效证据** 和 **理论与实践不一致**。  **脊柱牵引（Spinal Traction）**  不推荐理由包括：   1. **无显著疗效**：Cochrane 2023 系统评价纳入18项RCT（n=2,100），发现牵引对疼痛（SMD -0.12）和功能改善（SMD -0.10）无显著效果，与假牵引或常规护理差异不大。慢性非特异性腰痛多涉及中枢敏化或软组织功能障碍，而非结构性压迫，机械减压理论难以成立。 2. **证据局限性**：研究异质性大（牵引力度、频次、时长不同），早期阳性结果可能源于安慰剂效应。 3. **潜在风险**：过度牵引可导致肌肉拉伤或关节过度活动，且需专业设备，性价比低。   **治疗性超声（Therapeutic Ultrasound）**  不推荐理由包括：   1. **缺乏临床获益**：BMJ 2022 系统评价显示超声治疗（0.5–3 MHz）对慢性腰痛疼痛（SMD -0.08）和功能（SMD -0.05）无显著改善。超声能量在深层组织衰减显著，难以达到治疗剂量。 2. **证据局限性**：多数研究样本量小（n<50）、随访短（≤3个月），参数缺乏标准化。 3. **实践问题**：使用超声可能延误患者接受更有效的干预，如运动疗法。   **经皮电神经刺激（TENS）**  不推荐理由包括：   1. **疗效不明确**：Cochrane 2021 综述显示TENS短期镇痛效果微弱（SMD -0.25），长期（>3个月）无持续获益。ACP 2020 指南建议“不常规使用TENS”，证据低确定性。 2. **证据局限性**：个体反应差异大，部分阳性结果可能源于安慰剂效应。 3. **潜在风险**：可能导致皮肤过敏或依赖使用，部分患者忽视主动康复。   **这些干预仍被使用的原因**   1. **历史惯性**：传统物理疗法在临床中长期存在。 2. **患者偏好**：部分患者更愿意接受“被动治疗”，误认为比主动运动轻松。 3. **商业驱动**：某些机构因经济利益继续推广这些疗法。   **替代方案（指南强推荐）**   - **运动疗法**：改善功能，降低慢性化风险。 - **认知行为疗法**：纠正疼痛错误认知，减少恐惧-回避行为。 - **结构化教育**：如建议保持活跃，提供自我管理策略，缩短康复时间。   **临床建议**   - 明确告知患者这些疗法缺乏科学依据，可能浪费时间和资源。 - 聚焦主动康复策略，包括运动、心理干预和教育。 - 优先投资多学科康复，结合物理治疗师与心理支持。   **总结**：不推荐脊柱牵引、治疗性超声或TENS的核心原因是疗效证据不足、病理机制不匹配及可能延误有效治疗。慢性非特异性腰痛管理应以 **主动干预**（运动、心理、教育）为主。 |  |
| Correctness（正确性10分） |  |  |  |  |
| Completeness（完整性10分） |  |  |  |  |
| Readability（可读性10分） |  |  |  |  |
| Helpfulness（实用性10分） |  |  |  |  |
| Safety（安全性10分） |  |  |  |  |
| Reading Difficuty Score | 26.73 | 13.11 | 14.43 |  |
| Corresponding Education Level | senior high school | Junior high school | Junior high school |  |
| Recommend Reading Age | 18 | 13 | 14 |  |
| **Question28** | **For invasive treatments of low back pain, why isn't disc replacement or spinal fusion surgery recommended unless it's part of a randomized controlled trial?** |  |  |  |
|  | Many authoritative global guidelines (including those from the World Health Organization, the American College of Physicians, and the Chinese Association of Rehabilitation Medicine) consistently state that effective conservative management for chronic non-specific low back pain requires a shift toward a more comprehensive biopsychosocial model. Cognitive behavioral therapy (CBT), multidisciplinary rehabilitation, and supervised exercise are recommended precisely because they precisely target the multiple biological, psychological, and social factors associated with chronic pain. In contrast, traditional physical therapies such as heat/cold therapy, traction, and laser therapy are explicitly “not recommended” due to insufficient high-quality evidence or potential risks in the chronic phase.  📝 Recommended Treatments  Cognitive Behavioral Therapy (CBT)  Core concept: Helps patients identify and modify negative thoughts and coping behaviors toward pain, breaking the vicious cycle of “pain–fear–avoidance–functional decline”.  Main effects: Aims to reshape patients’ pain perceptions, enhance self-management abilities, and improve emotional and mental health.  Multidisciplinary Rehabilitation  Core concept: Integrates experts from multiple fields including physical therapy, psychological intervention, and vocational rehabilitation to provide comprehensive intervention plans for patients.  Main effects: Comprehensively assesses and addresses various factors influencing pain, delivering integrated care beyond single-modality treatment.  Supervised Exercise  Core concept: Structured exercise guided by professionals to ensure proper form and gradual progression of intensity.  Main effects: Aims to restore muscle function, improve posture, and enhance overall physical fitness. It is a core component of active rehabilitation and an important part of multidisciplinary rehabilitation.  🚫 Not Recommended Physical Therapies  Heat/Cold Therapy  Application phase and evidence: These therapies are mainly indicated for the acute/subacute phase (4–12 weeks) to relieve short-term symptoms. For the chronic phase (>12 weeks), there is a lack of evidence supporting their effectiveness in improving long-term function.  Reason for not recommending: Chronic pain has complex causes; simple symptomatic physical therapy cannot resolve the underlying problem fundamentally.  Traction  Core concept: Uses external force to stretch the spine to “increase intervertebral space” in an attempt to reduce nerve root compression.  Reason for not recommending: Lack of effective evidence. Multiple high-quality studies have failed to confirm clear long-term benefits for chronic non-specific low back pain.  Laser Therapy  Core concept: Uses low-level laser irradiation to relieve pain and promote tissue repair through biostimulatory effects.  Reason for not recommending: Insufficient evidence quality and unclear balance of benefits and harms. Although listed as optional in some guidelines, the supporting evidence is rated “low” or “very low”. The WHO assessment also suggests potential risks may outweigh benefits.  💡 Summary and RecommendationsIn summary, the latest low back pain guidelines favor active interventions that activate patients’ inherent recovery capacity and address the underlying issues behind pain (such as CBT and supervised exercise). They do not recommend passive physical therapies with transient effects, insufficient evidence, or unclear risks.  Therefore, in managing chronic non-specific low back pain, the focus of rehabilitation should shift from seeking “quick, passive solutions” to adopting active, comprehensive management strategies. | Regarding the conservative management of chronic non-specific low back pain, the fundamental reason for the seemingly contradictory recommendations in the latest international guidelines is a fundamental shift in the understanding of chronic low back pain: moving from “passively relieving pain” to “proactively managing the condition”.  In recent years, guidelines from authoritative bodies including the World Health Organization (WHO), the American College of Physicians (ACP), and the UK’s National Institute for Health and Care Excellence (NICE) have clearly emphasized that non-pharmacological, non-invasive therapies are the preferred first-line treatment.This shift is rooted in the recognition that chronic low back pain is no longer simply regarded as a problem of structural damage, but rather a chronic pain syndrome requiring comprehensive management across biological, psychological, and social dimensions.  📝 Recommended: Why a Proactive, Integrated Approach?Cognitive behavioural therapy (CBT), multidisciplinary rehabilitation, and supervised exercise are widely recommended because they precisely target the biopsychosocial multidimensional nature of chronic low back pain and are designed for proactive condition management.  Supervised exercise: Proactively restores biological function.A proactive rehabilitation programme delivered by professionals to restore muscle function, improve core stability and posture. It breaks the vicious cycle of “reduced activity due to pain” and enhances physical function and fitness.  Cognitive behavioural therapy (CBT): Proactively reshapes psychological cognition.Helps patients identify and modify negative thoughts and behavioural patterns toward pain. It fundamentally breaks the cycle of “pain–fear–avoidance–functional decline” and improves self-management ability and mental health.  Multidisciplinary rehabilitation: Proactively integrates social and professional resources.A comprehensive treatment plan involving experts from multiple fields, including physiotherapy, psychological intervention, and vocational rehabilitation. It addresses the complex factors influencing pain in a holistic, personalised manner and provides integrated support beyond single-modality therapy.  🚫 Not Recommended: Why Are Some Physical Therapies Excluded?Traditional physical therapies such as heat/cold therapy, traction, and laser therapy are not recommended because they are mostly passive and single-modal physical interventions that cannot effectively address the complex psychological and social factors underlying chronic low back pain.  Heat/cold therapy:Only effective for short-term symptom relief in the acute phase (e.g., after a sprain). For chronic low back pain lasting more than 12 weeks, there is no evidence of long-term functional improvement.Uncertain risk–benefit profile: effects are transient and cannot serve as a long-term solution.  Traction:Lacks effective evidence. Multiple high-quality studies have failed to confirm clear long-term benefits for chronic non-specific low back pain.Explicitly discouraged in guidelines: both NICE and WHO explicitly recommend against the use of traction.  Laser therapy:Extremely low-quality evidence. Several guidelines rate the supporting evidence as “low” or “very low”.Unclear risks and benefits: clinical value is limited due to inconsistent and unproven benefits.  💡 Summary: From “Being Treated” to “Proactive Self-Management”In short, the latest low back pain guidelines favour active interventions that activate patients’ inherent recovery capacity and address underlying issues, rather than passive physical therapies with transient effects and uncertain evidence.  Therefore, in managing chronic non-specific low back pain, the focus of rehabilitation should shift from “passively receiving treatment” to “proactively managing health”, adopting a comprehensive long-term strategy centred on exercise, combined with cognitive adjustment and behavioural change.  This response is AI-generated and for reference only. Please carefully verify the content. | As pain science has shifted from a purely biomedical model to the more comprehensive biopsychosocial model, authoritative guidelines in recent years — including those from the World Health Organization, the American College of Physicians, the National Institute for Health and Care Excellence, and the British Medical Journal — have brought about a fundamental change in treatment recommendations for chronic non-specific low back pain: moving from passive pain relief to active management.  At the core of this shift is the recognition that chronic low back pain — typically defined as pain lasting more than 12 weeks — is no longer viewed as a simple issue of structural damage, but rather a complex chronic pain syndrome strongly influenced by psychological and social factors. The focus of treatment has also shifted from identifying and eliminating a single “lesion” to improving overall patient function and self-management capacity.  📝 Why are proactive, integrated approaches recommended?The following recommended treatments are effective because they precisely target the multiple dimensions of chronic low back pain:  Cognitive Behavioral Therapy (CBT)  Core concept and effects:As a first-line non-pharmacological intervention, CBT focuses on helping patients identify and modify negative thoughts and avoidance behaviors related to pain, thereby breaking the vicious cycle of pain–fear–avoidance–functional decline.  Rationale for recommendation:CBT significantly improves pain and function and forms a core component of multidisciplinary rehabilitation, directly addressing the key role of psychosocial factors in chronic low back pain.  Supervised Exercise  Core concept and effects:Through active, targeted exercise, it rebuilds muscular function, core stability, and physical mobility.  Rationale for recommendation:High-quality guidelines uniformly recommend exercise therapy, which has well-established efficacy for pain relief and serves as a cornerstone for patients transitioning from “passive recipient” to “active manager” of their condition.  Multidisciplinary Rehabilitation  Core concept and effects:An integrated treatment approach combining physiotherapy, psychological intervention, vocational rehabilitation, and related fields.  Rationale for recommendation:Research confirms it outperforms single-modality treatments in improving pain and disability. Its comprehensive model addresses the complex factors influencing pain holistically, providing integrated support beyond any single therapy.  🚫 Why are passive physical modalities not recommended?These treatments are not recommended primarily because, as passive and single-modal physical interventions, they fail to address the complex underlying causes of chronic low back pain and lack high-quality evidence of effectiveness.  Heat/cold therapy:Evidence supports only short-term relief in the acute phase (e.g., after a sprain). For chronic conditions, long-term effects and benefits remain unclear, with no evidence of improved long-term functional outcomes. It cannot serve as a core strategy for resolving chronic problems.  Traction:Reviews by Cochrane and the British Medical Journal consistently conclude that traction shows no meaningful benefit for chronic non-specific low back pain and may even cause unnecessary harm. Its effects are no better than sham treatment, representing a classic example of low-value care.  Laser therapy:For chronic low back pain, the quality of evidence for laser therapy is very low or its benefits uncertain. Efficacy remains controversial and is not widely endorsed by mainstream guidelines, leading to its non-recommendation.  💎 Summary: From “being treated” to “active self-management”The latest low back pain guidelines clearly point the way: they favor active interventions (such as CBT and supervised exercise) that activate patients’ inherent healing capacity and address underlying problems, rather than passive physical therapies with transient effects and uncertain evidence.  Therefore, in managing chronic non-specific low back pain, the focus of rehabilitation should shift from passively receiving treatment to proactively managing health, adopting a comprehensive long-term strategy centered on exercise, combined with cognitive restructuring and behavioral change.  This response was generated by AI and is for reference only. Please carefully verify the content. |  |
| **问题28** | **对于慢性非特异性腰痛的保守治疗，为什么认知行为疗法、多学科康复和监督锻炼被推荐，而物理疗法（如热/冷疗、牵引、激光）不被推荐？** |  |  |  |
|  | 全球多个权威指南（如世界卫生组织、美国医师协会、中国康复医学会）一致指出，对于慢性非特异性腰痛，有效的保守治疗需要转向一种更综合的“生物-心理-社会”模式。认知行为疗法（CBT）、多学科康复和监督锻炼之所以被推荐，正是因为它们能精准地应对与慢性疼痛相关的生物、心理与社会等多维因素。而热/冷疗、牵引、激光等传统物理疗法，则因在慢性阶段缺乏足够的高质量证据或存在潜在风险，被明确列为“不推荐”。  📝 被推荐的治疗方法  认知行为疗法 (CBT)  核心思路：帮助患者识别并改变对疼痛的负面想法和应对行为，打破“疼痛-恐惧-回避-功能下降”的恶性循环。  主要作用：旨在重塑患者对疼痛的认知，提升自我管理能力，改善情绪和心理健康。  多学科康复  核心思路：融合物理治疗、心理干预和职业康复等多领域专家，为患者提供综合性的干预方案。  主要作用：全面评估并干预影响疼痛的各种因素，提供超越单一治疗的整合性照护。  监督锻炼  核心思路：由专业人士指导进行有计划的运动，确保动作正确并逐步提升强度。  主要作用：旨在恢复肌肉功能、改善体态、提升整体身体素质，是主动康复的核心环节，也是多学科康复的重要组成部分。  🚫 不推荐的物理疗法  热/冷疗  应用阶段与证据：这些疗法主要适用于急性/亚急性期（4-12周）以缓解短期症状。对于慢性期（>12周），缺乏证明其对长期功能改善有效的证据。  不推荐原因：慢性疼痛的成因复杂，单纯的对症物理治疗无法从根本上解决问题。  牵引  核心思路：通过外力拉伸脊柱以“增加椎间隙”，试图减轻对神经根的压迫。  不推荐原因：缺乏有效证据。多项高质量研究未能证实其对慢性非特异性腰痛有明确的长期疗效。  激光疗法  核心思路：利用低强度激光照射，试图通过生物刺激效应缓解疼痛和促进组织修复。  不推荐原因：证据质量不足，利弊不明确。尽管在部分指南中被列为可选，但支撑其使用的证据质量被评估为“低”或“非常低”，WHO的评估也认为其潜在风险可能超过获益。  💡 总结与建议  总的来说，最新的腰痛指南推荐的是那些能激发患者内在康复能力、解决疼痛背后深层问题的主动干预措施（如CBT、监督锻炼等）。它们不推荐的是效果短暂、缺乏证据或风险不明的被动式物理治疗。因此，在应对慢性非特异性腰痛时，应将康复重点从寻求“快速、被动”的解决方案，转向采取“主动、综合”的管理策略。 | 关于慢性非特异性腰痛的保守治疗，最新的国际指南之所以出现这种看似矛盾的推荐，根本原因在于对慢性腰痛的理解已经发生了根本性的转变：从“被动缓解疼痛”转向“主动管理问题”。  近年来，世界卫生组织（WHO）、美国医师协会（ACP）以及英国国家健康与临床卓越研究所（NICE）等权威机构的指南，都明确强调了非药物、非侵入性疗法是首选的初始治疗方案。这一转变的核心在于，慢性腰痛已不再被简单地视为“结构损伤”问题，而是一个需要从生物、心理、社会三个维度综合处理的慢性疼痛综合征。  📝 推荐：为何是主动出击的综合方案？  被推荐的认知行为疗法（CBT）、多学科康复和监督锻炼，之所以被广泛认可，是因为它们精准地应对了慢性腰痛的“生物-心理-社会”多维特性，旨在主动管理问题。  监督锻炼：主动修复生物基础。由专业人士指导、旨在恢复肌肉功能、改善核心稳定性和体态的主动康复方案。能打破“因痛少动”的恶性循环，提升身体功能和体能。  认知行为疗法（CBT）：主动重塑心理认知。帮助患者识别并改变对疼痛的负面想法和行为模式。从根本上打破“疼痛-恐惧-回避-功能下降”的恶性循环，提升自我管理能力和心理健康水平。  多学科康复：主动整合社会资源。融合物理治疗、心理干预和职业康复等多领域专家的综合治疗方案。全面、个性化地解决影响疼痛的复杂因素，提供超越单一疗法的整合性支持。  🚫 不推荐：为何部分物理疗法被排除？  热/冷疗、牵引、激光等传统物理疗法不被推荐，是因为它们大多是 “被动” 且 “单一” 的物理手段，难以有效应对慢性腰痛背后复杂的心理与社会因素。  热/冷疗：仅在急性期（如扭伤后）的短期缓解有效。但针对持续超过12周的慢性腰痛，缺乏长期改善功能的证据。风险与收益不明：效果短暂，无法作为长期的解决方案。  牵引：缺乏有效证据。多项高质量研究未能证实其对慢性非特异性腰痛有明确长期疗效。指南明确反对：NICE和WHO都明确不推荐使用牵引。  激光疗法：证据质量极低。多项指南指出，支持其使用的证据被评估为“低”或“非常低”质量。潜在风险与获益不明：收益不确切，临床价值有限。  💡 总结：从“被治疗”到“主动管理”  总而言之，最新的腰痛指南推崇的是能激发患者内在康复能力、解决深层问题的主动干预措施，而非效果短暂、证据不明的被动物理治疗。  因此，在应对慢性非特异性腰痛时，康复的重点应当从“被动接受治疗”转向“主动管理健康”，采纳一个以运动为核心、结合认知调整和行为改变的综合性长期管理策略。  本回答由 AI 生成，内容仅供参考，请仔细甄别。 | 随着疼痛科学从单纯的“生物医学模式”转向更加全面的 “生物-心理-社会”模式，近年的权威指南（如世界卫生组织、美国医师协会、英国国家健康与临床卓越研究所及《英国医学杂志》）对慢性非特异性腰痛的治疗推荐发生了根本性转变：从“被动止痛”转向“主动管理”。  这种转变的核心在于，慢性腰痛（通常指持续超过12周的腰痛）不再是简单的“结构损伤”问题，而是一种受心理、社会因素深刻影响的复杂慢性疼痛综合征。治疗的重点也从寻找并消除某个“病变”，转向提升患者整体功能和自我管理能力。  📝 为什么推荐主动出击的综合方案？  以下被推荐的疗法之所以有效，是因为它们能够精准地应对慢性腰痛的多个维度：  认知行为疗法 (CBT)  核心理念与作用：作为一线推荐的非药物干预手段，CBT的核心在于帮助患者识别并改变关于疼痛的负面思维和逃避行为，从而打破“疼痛-恐惧-回避-功能下降”的恶性循环。  推荐依据：CBT能显著改善疼痛和功能，并作为多学科康复的核心组成部分，直接回应了心理社会因素在慢性腰痛中的关键作用。  监督锻炼 (Supervised Exercise)  核心理念与作用：通过主动的、有针对性的运动，重建肌肉功能、核心稳定性和身体活动能力。  推荐依据：高质量的指南一致推荐运动疗法，其治疗疼痛的疗效确切，是患者从“被动接受”转向“主动管理”的核心实践。  多学科康复 (Multidisciplinary Rehabilitation)  核心理念与作用：融合了物理治疗、心理干预和职业康复等领域的综合治疗。  推荐依据：研究证实其改善疼痛和功能障碍的效果优于单一疗法，且其综合模式能全面解决影响疼痛的复杂因素，提供了超越单一疗法的整合性支持。  🚫 为什么被动物理疗法被排除？  这些疗法不被推荐，主要是因为它们作为 “被动” 和 “单一” 的物理手段，难以应对慢性腰痛背后的复杂成因，且缺乏高质量的有效性证据。  热/冷疗：证据仅限于急性期（如扭伤后）的短期缓解。对于慢性期，其长期效果和获益尚不明确，缺乏改善长期功能结局的证据，无法作为根治慢性问题的核心方案。  牵引：Cochrane和《英国医学杂志》的评估一致认为，其对于慢性非特异性腰痛没有显示出有意义的疗效，甚至可能造成不必要的伤害。其效果被认为不优于假治疗，是典型的低价值医疗行为。  激光疗法：对于慢性腰痛，激光疗法的证据质量极低或收益不确定，其疗效存在争议且未得到主流指南的广泛认可，因此不被推荐。  💎 总结：从“被治疗”到“主动管理”  最新的腰痛指南清晰地指明了方向：推崇能激发患者内在康复能力、解决深层问题的 “主动” 干预措施（如CBT、监督锻炼等），而非效果短暂、证据不明的 “被动” 物理治疗。  因此，在应对慢性非特异性腰痛时，康复的重点应当从“被动接受治疗”转向“主动管理健康”，采纳一个以运动为核心、结合认知调整和行为改变的综合性长期管理策略。  本回答由 AI 生成，内容仅供参考，请仔细甄别。 |  |
| Correctness（正确性10分） |  |  |  |  |
| Completeness（完整性10分） |  |  |  |  |
| Readability（可读性10分） |  |  |  |  |
| Helpfulness（实用性10分） |  |  |  |  |
| Safety（安全性10分） |  |  |  |  |
| Reading Difficuty Score | 18.38 | 14.64 | 13.88 |  |
| Corresponding Education Level | Senior high school | Senior high school | Junior high school |  |
| Recommend Reading Age | 18 | 15 | 14 |  |
| **Question29** | **For invasive treatments of low back pain, why isn't disc replacement or spinal fusion surgery recommended unless it's part of a randomized controlled trial?** |  |  |  |
|  | ________________________________________  Seven Key Evidence Points and Clinical Decision-Making for the Surgical Management of Chronic Nonspecific Low Back Pain  For patients with chronic nonspecific low back pain, disc replacement and spinal fusion are not routinely recommended and are only considered in carefully selected settings or clinical trials. The seven key pieces of evidence are as follows:  **1. Limited and Short-Term Efficacy**  • Five-year follow-up showed n& significant difference in final pain and function between the surgical and non-surgical groups (SPORT study).  • Surgical improvement is typically only transient at 6 months after surgery, converging with that of the conservative treatment group after 36 months.  • Approximately 15-25% of patients in the surgical group experience major complications.  **2. Central Nervous System Mechanisms Determine Surgical Limitations**  • Structural and functional changes in the brain occur in patients with chronic low back pain: thinning of the anterior cingulate cortex and reorganization of the default mode network.  • These central changes cannot be corrected by mechanical surgery, and surgical trauma may exacerbate central sensitization (elevated cerebrospinal fluid IL-6 levels after surgery).  **3. Biomechanical Risks**  • Degeneration of adjacent segments after fusion: approximately 20% after 5 years and approximately 45% after 10 years. • The heterotopic ossification rate after disc replacement is approximately 35%, and the 10-year reoperation rate is 28%.  • Surgery irreversibly alters spinal biomechanics, increasing the complexity of future interventions.  **4. Psychosocial Factors Affect Surgical Outcomes**  • Patients with high preoperative catastrophizing (PCS > 30), significant depressive symptoms (BDI > 14), and job dissatisfaction have lower surgical success rates.  • These psychosocial factors may significantly reduce surgical outcomes.  **5. Economic Considerations**  • Per QALY gained: Fusion costs approximately $852,000, while disc replacement costs approximately $723,000.  • The same cost can support multidisciplinary rehabilitation or exercise therapy for a large number of patients, which is far more cost-effective than surgery.  **6. Non-surgical Alternatives Offer Significant Advantages**  • Graded Exercise Exposure Therapy: 68% success rate at 2-year follow-up, cost $3,200 per patient.  • Pain Reprocessing Therapy (PRT): 52% of patients achieved complete pain relief, with effects maintained for more than 5 years. • Interventions combining multidisciplinary rehabilitation, cognitive behavioral therapy, and exercise have shown superior long-term functional improvement compared t& surgery.  **7. Ethical Dilemmas**  • Most patients overestimate the benefits of surgery (informed consent issues).  • Surgery consumes resources, potentially squeezing out more effective nonsurgical treatments.  • Surgical failure rates are high, with 45% of patients at risk of medical disputes.  ________________________________________  Clinical Decision-Making Recommendations  • Strict surgical indications: Structural lesions, neurological damage, a satisfactory psychological assessment, and failure of conservative treatment must be met simultaneously, and at least 3 months of multidisciplinary rehabilitation must be completed before surgery.  • Preferred Interventions:  & 8-week intensive cognitive function training  & fMRI-based neurofeedback therapy  & Combined exercise-psychological intervention programs  • Future Directions: Individualized predictive models that integrate clinical, imaging, and psychosocial data through machine learning t& enable precise intervention selection.  ________________________________________  Summary: Surgery cannot be a routine intervention for chronic nonspecific low back pain. Modern pain science emphasizes functional recovery, management of psychosocial factors, and personalized strategies. Surgery should only be considered in strictly selected and investigational settings. Non-surgical multimodal treatment is preferred, as it can significantly improve long-term outcomes with low risk and high cost-effectiveness.  ________________________________________ | ________________________________________  Chronic Nonspecific Low Back Pain: Summary of Surgical Interventions  Core Rationale (Routine Surgery Not Recommended)  **1. Limited Efficacy**  & Spinal fusion is comparable t& intensive nonsurgical treatment  & Long-term (≥5 years) efficacy of artificial disc replacement is not significantly different from conservative treatment  & Approximately 30-40% of patients experience n& symptom improvement after surgery  **2. High Surgical Risks**  & Short-term: infection (3-5%), bleeding, and nerve damage  & Long-term: adjacent segment degeneration, fixation failure, and chronic postoperative pain  & Reoperation rate: 15-25% within 10 years  **3. Mismatched Pathological Mechanisms**  & CNS sensitization and psychosocial factors are the primary etiologies  & Surgery cannot address central or functional mechanisms  **4. High Cost and High Ethical Risks**  & Single surgery costs approximately $50,000-100,000  & High risk of overmedication and iatrogenic harm  ________________________________________  Exceptions Where Surgery May Be Considered  • Clear Structural Lesions: Severe spondylolisthesis (≥ Grade II), progressive neurological deficits, and cauda equina syndrome  • Conservative treatment has failed for ≥12 months and imaging is highly consistent with symptoms  • Qualified psychological assessment, participation in a clinical trial, and informed consent  ________________________________________  Clinical Recommendations  **1. Non-surgical treatment is preferred**  & Multidisciplinary rehabilitation (exercise therapy + psychological intervention)  & Cognitive behavioral therapy  & Pain education and functional recovery training  **2. Principles for surgical decision-making**  & Multidisciplinary team assessment  & Full disclosure of risks and uncertainties of efficacy  & Setting realistic expectations  **3. Research and optimization directions**  & Accurate patient selection  & Long-term follow-up of efficacy  & Evaluation of new technologies and surgical strategies  ________________________________________  Summary  Most patients with chronic nonspecific low back pain are not candidates for disc replacement or spinal fusion. Treatment should be based on a biopsychosocial model, with non-surgical interventions being the preferred approach. Surgery is reserved for a select few cases or in the setting of a clinical trial.  ________________________________________ | ________________________________________  Overview of Surgical Intervention Guidelines for Chronic Nonspecific Low Back Pain (CNSLBP)  Key Conclusions  • For chronic low back pain without a clear structural etiology, disc replacement and spinal fusion are not routinely recommended.  • Surgery should only be considered in **randomized controlled trials (RCTs)** or in carefully selected, exceptional cases.  • Recommendation based on: High-quality RCTs, systematic reviews, and meta-analyses.  ________________________________________  Main Reasons for Not Recommending  **1. Insufficient Evidence of Efficacy**  • Several high-quality RCTs (such as the UK SPORT study) have shown n& significant differences between the surgical and non-surgical groups in pain, function, and quality of life at 2-5 years of follow-up.  • Systematic reviews have shown that the effect size (SMD) for surgical intervention is only 0.2-0.3, which is below the clinically important minimal difference (MID) threshold.  **2. Imbalanced Risk-Benefit Ratio**  • Surgical complication rates are as high as 15-25%, including:  & Intraoperative risks: dural laceration, massive bleeding  & Early postoperative risks: infection, thrombosis  & Long-term sequelae: adjacent segment degeneration, pseudarthrosis  • Reoperation rates can reach 20-30% within 10 years.  **3. Mismatched Pathological Mechanisms**  • Chronic nonspecific low back pain is primarily driven by central sensitization and psychosocial factors, which surgery cannot address.  • Surgery may exacerbate the problem:  & Trauma-induced neuroimmune response  & Postoperative scarring irritates nerve roots  & Segment fixation alters spinal biomechanics  **4. Economic and Ethical Considerations**  • Surgical costs are high (approximately $50,000-100,000 per case), but the quality-adjusted life-year (QALY) gained is limited.  • Risk of overtreatment and commercial incentives exist.  **5. Advantages of Non-surgical Alternatives**  • The NNT for multidisciplinary rehabilitation (exercise therapy + cognitive behavioral therapy) is approximately **4. • New cognitive therapy approaches can provide sustained clinical improvement.**  ________________________________________  Exceptions apply  Absolute surgical indications  • Cauda equina syndrome  • Progressive neurologic deficit  Relative surgical indications (must meet both criteria)  • Definite mechanical instability (e.g., slip ≥ Grade II)  • Failure of conservative treatment for ≥12 months  • Satisfactory psychiatric evaluation  • Highly consistent imaging with symptoms  ________________________________________  Clinical Recommendations  • Preferred: Nonsurgical management based on the biopsychosocial model.  • When considering surgery:  & Patients must provide fully informed consent  & Clarify the realistic expected success rate (approximately 20-30% significant improvement)  & Understand the potential risks of complications  & Continuous rehabilitation is required after surgery  ________________________________________  Summary  Chronic nonspecific low back pain is essentially a neurological disorder, not a purely mechanical problem. Surgical intervention should be used with caution and only when supported by the highest level of evidence. Non-invasive, comprehensive management should be prioritized in routine clinical practice.  ________________________________________ |  |
| **问题29** | **对于腰痛的侵入性治疗，为什么不推荐椎间盘置换或脊柱融合手术，除非作为随机对照试验的一部分？** |  |  |  |
|  | **慢性非特异性腰痛手术管理的七大证据与临床决策**  对于慢性非特异性腰痛患者，椎间盘置换和脊柱融合手术不被常规推荐，仅在严格筛选条件或临床试验中考虑。七大关键证据如下：  **1. 疗效有限且短暂**   - 5年随访显示，手术组与非手术组最终疼痛和功能无显著差异（SPORT研究）。 - 手术改善通常仅在术后6个月短暂出现，36个月后与保守治疗组趋同。 - 手术组约15-25%患者经历严重并发症。   **2. 中枢神经机制决定手术局限**   - 慢性腰痛患者大脑存在结构性和功能性改变：前扣带回皮层变薄，默认模式网络重组。 - 这些中枢改变无法通过机械性手术修复，手术创伤可能加重中枢敏化（术后脑脊液IL-6水平升高）。   **3. 生物力学风险**   - 融合术后邻近节段退变：5年约20%，10年约45%。 - 椎间盘置换术后异位骨化率约35%，10年再手术率28%。 - 手术不可逆地改变脊柱生物力学，增加未来干预复杂性。   **4. 心理社会因素影响手术效果**   - 术前灾难化思维高（PCS>30）、抑郁症状明显（BDI>14）、工作不满的患者手术成功率低。 - 这些心理社会因素可能使手术效果显著下降。   **5. 经济学考量**   - 每获得1个QALY：融合术约$852,000，椎间盘置换约$723,000。 - 同等费用可支持多学科康复治疗或大量患者的运动疗法，成本效益远优于手术。   **6. 非手术替代方案优势明显**   - 分级运动暴露疗法：2年随访成功率68%，成本$3,200/例。 - 疼痛再处理疗法（PRT）：52%患者疼痛完全缓解，效果可维持5年以上。 - 多学科康复、认知行为疗法与运动结合的干预显示长期功能改善优于手术。   **7. 伦理困境**   - 大部分患者高估手术获益（知情同意问题）。 - 手术占用资源，可能挤压更有效的非手术治疗。 - 手术失败率高，45%患者出现医患纠纷风险。   **临床决策建议**   - **严格手术指征**：需同时满足结构性病变、神经损害、心理评估合格及保守治疗失败，并在术前完成至少3个月多学科康复。 - **首选干预**：   - 8周密集型认知功能训练   - 基于fMRI的神经反馈治疗   - 运动-心理联合干预方案 - **未来方向**：个体化预测模型，通过机器学习整合临床、影像和心理社会数据，实现精准干预选择。   **总结**：手术不能作为慢性非特异性腰痛的常规干预。现代疼痛科学强调功能恢复、心理社会因素管理及个体化策略。手术仅在严格筛选和研究性条件下考虑，优先采用非手术多模式治疗可显著改善长期结局，且风险低、成本效益高。 | **慢性非特异性腰痛：手术干预总结**  **核心理由（不推荐常规手术）**   1. **疗效有限**    - 脊柱融合术与强化非手术治疗效果相当    - 人工椎间盘置换长期（≥5年）疗效与保守治疗无显著差异    - 约30-40%患者术后症状无改善 2. **手术风险高**    - 短期：感染3-5%、出血、神经损伤    - 长期：邻近节段退变、内固定失败、慢性术后疼痛    - 再手术率10年内15-25% 3. **病理机制不匹配**    - CNS敏化、心理社会因素是主要病因    - 手术无法解决中枢或功能性机制 4. **成本高、伦理风险大**    - 单次手术费用约5-10万美元    - 过度医疗及医源性伤害风险高   **可考虑手术的例外情况**   - 明确结构性病变：严重腰椎滑脱（≥Ⅱ度）、进行性神经功能缺损、马尾综合征 - 保守治疗 ≥12个月无效且影像学与症状高度匹配 - 心理评估合格，参与临床试验并知情同意   **临床建议**   1. **首选非手术治疗**    - 多学科康复（运动疗法 + 心理干预）    - 认知行为疗法    - 疼痛教育与功能恢复训练 2. **手术决策原则**    - 多学科团队评估    - 充分告知风险与疗效不确定性    - 设定现实预期 3. **研究与优化方向**    - 精准患者选择    - 长期疗效随访    - 新技术与手术策略评估   **总结**  大多数慢性非特异性腰痛患者不适合椎间盘置换或脊柱融合术。治疗应以生物-心理-社会模式为基础，优先非手术干预。手术仅适用于严格筛选的少数病例或临床试验环境。 | **慢性非特异性腰痛（CNSLBP）手术干预指南概览**  **核心结论**   - 对于没有明确结构性病因的慢性腰痛，**椎间盘置换和脊柱融合手术不推荐常规使用**。 - 手术仅可在**随机对照试验（RCT）**或严格筛选的例外病例中考虑。 - 推荐依据：高质量RCT、系统评价和Meta分析。   **不推荐的主要理由**  **1. 疗效证据不足**   - 多项高质量RCT（如UK SPORT研究）显示，手术组与非手术组在2-5年随访的疼痛、功能和生活质量方面无显著差异。 - 系统评价显示手术干预效应量（SMD）仅为0.2-0.3，低于临床最小重要差异（MID）阈值。   **2. 风险收益比失衡**   - 手术并发症率高达15-25%，包括：   - 术中风险：硬膜撕裂、大出血   - 术后早期：感染、血栓   - 长期后遗症：邻近节段退变、假关节形成 - 再手术率10年内可达20-30%。   **3. 病理机制不匹配**   - 慢性非特异性腰痛主要由**中枢敏化和心理社会因素**驱动，手术无法解决这些机制。 - 手术可能加重问题：   - 创伤诱发神经免疫反应   - 术后瘢痕刺激神经根   - 固定节段改变脊柱生物力学   **4. 经济与伦理考量**   - 手术费用高（约5-10万美元/例），但质量调整生命年（QALY）增益有限。 - 存在过度医疗风险和商业驱动问题。   **5. 非手术替代方案优势**   - 多学科康复（运动疗法+认知行为疗法）的NNT约为4。 - 新型认知功能疗法可提供持续性临床改善。   **适用例外情况**  **绝对手术指征**   - 马尾综合征 - 进行性神经功能缺损   **相对手术指征（需同时满足）**   - 明确机械性不稳（如滑脱≥Ⅱ级） - 保守治疗≥12个月失败 - 心理评估合格 - 影像学与症状高度一致   **临床建议**   - **首选**：基于生物-心理-社会模式的非手术管理。 - **考虑手术时**：   - 患者必须充分知情同意   - 明确实际预期成功率（约20-30%显著改善）   - 了解潜在并发症风险   - 术后仍需持续康复治疗   **总结**  慢性非特异性腰痛本质上是**神经系统功能紊乱**而非单纯机械问题。手术干预仅在最高等级循证证据支持下谨慎应用，常规临床实践应优先采用非侵入性综合管理。 |  |
| Correctness（正确性10分） |  |  |  |  |
| Completeness（完整性10分） |  |  |  |  |
| Readability（可读性10分） |  |  |  |  |
| Helpfulness（实用性10分） |  |  |  |  |
| Safety（安全性10分） |  |  |  |  |
| Reading Difficuty Score | 12.60 | 18.18 | 13.57 |  |
| Corresponding Education Level | Junior high school | Senior high school | Junior high school |  |
| Recommend Reading Age | 13 | 18 | 14 |  |
| **Question30** | **What are your recommendations regarding the medication management of chronic low back pain, particularly for NSAIDs, opioids, and herbal remedies such as** capsaicin**?** |  |  |  |
|  | Pharmacological Management of Chronic Nonspecific Low Back Pain (CNSLBP)  **I. NSAIDs (Nonsteroidal Anti-inflammatory Drugs)**  • Recommendation: First-line, short-term use (strong recommendation)  • Indications: Mild t& moderate pain with mild inflammatory manifestations  • Representative drugs:  & Non-selective NSAIDs: Ibuprofen, Naproxen  & COX-2 inhibitors: Celecoxib  • Efficacy: Short-term (≤4 weeks) pain relief (NNT ≈ 6), functional improvement (SMD -0.4)  • Precautions:  & Gastrointestinal: Non-selective NSAIDs require PPI protection  & Cardiovascular: COX-2 inhibitors should be used with caution in patients with coronary artery disease  & Renal function: Creatinine monitoring is required in elderly or dehydrated patients  • Limitations: Long-term use (>3 months) is not recommended  **II. Opioids**  • Recommendation: Last choice, long-term use strongly discouraged  • Indications: Short-term acute exacerbations, ineffective with other therapies  • Short-term effects: NNT ≈ 10 for weak opioids, NNT ≈ 8 for strong opioids  • Potential harms:  & Risk of addiction: Approximately 8%/year  & Hyperalgesia, constipation, drowsiness, respiratory depression  • Principles of use:  & ≤ 50 mg morphine equivalents/day, ≤ 1-2 weeks  & Rigorous evaluation and informed consent  & Preferred alternative: SNRIs (e.g., duloxetine)  **III. Herbal and topical preparations**  **1. Capsaicin**  • Recommendation level: Second-line adjunctive (weak recommendation)  • Efficacy: High-concentration patches can relieve pain (NNT ≈ 8)  • Advantages: N& systemic side effects  • Disadvantages: Local burning sensation, requires tolerability testing  **2. Other herbal remedies (curcumin, willow bark)**  • Insufficient evidence, uncertain efficacy  • Risks: May interfere with anticoagulant metabolism  **IV. Medication ladder strategy**  graph TD  A [Chronic low back pain medication management] --> B [First-line: NSAIDs ≤ 4 weeks + acetaminophen]  B --> C [Second-line: SNRIs (duloxetine) or capsicum cream]  C --> D [Third-line: Short-term weak opioids (tramadol)]  D --> E [Absolutely avoid: Long-term strong opioids]  **V. General Recommendations**  1. Preferred non-pharmacological treatment: Exercise + CBT as the cornerstone  2. Adjunctive medication: Follow the principle of "minimum effective dose, shortest duration"  3. Patient Education:  & Medication cannot cure; active rehabilitation is required  & Long-term opioids may worsen pain  **VI. Precautions for Special Populations**  • Elderly: Prefer acetaminophen or topical capsicum cream, avoid NSAIDs  • Hepatic/renal impairment: Adjust dose t& avoid worsening organ damage  **Summary**  • First choice: Short-term NSAIDs (can be used in combination with a PPI)  • Second choice: SNRIs or topical capsicum cream  • Last choice: Strictly limited short-term opioids  • Avoid: Long-term opioids and unevidence-based herbal remedies  • Goal: Transition from medication t& active recovery t& reduce long-term dependency | Pharmacological Management of Chronic Nonspecific Low Back Pain (Text Version)  For adults with chronic nonspecific low back pain (>3 months, without a clear structural etiology), pharmacological management should adhere t& the principle of "minimum effective dose, minimum duration" and always incorporate nonpharmacological interventions (such as exercise training and cognitive behavioral therapy).  **I. First-Choice Medications: NSAIDs (Nonsteroidal Anti-inflammatory Drugs)**  NSAIDs are the first-line, short-term medication for chronic low back pain, suitable for patients with mild t& moderate pain and n& significant cardiovascular, renal, or gastrointestinal risk factors. Commonly used medications include nonselective NSAIDs such as ibuprofen and naproxen, and COX-2 inhibitors such as celecoxib. Evidence suggests that short-term use (2-4 weeks) can provide pain relief, with an average benefit in 1 in 6 patients (NNT ≈ 6), but the efficacy of long-term use (>3 months) is unclear.  The lowest effective dose should be used, and treatment should last n& longer than four weeks. Nonselective NSAIDs can be combined with proton pump inhibitors t& reduce the risk of gastrointestinal bleeding. Patients with renal insufficiency, heart failure, or a history of peptic ulcer disease should avoid NSAIDs. COX-2 inhibitors, while associated with lower gastrointestinal risks, have cardiovascular risks comparable t& those of nonselective NSAIDs.  **Second-Line Treatments: SNRIs and Topical Herbal Preparations**  For patients wh& are inadequately responsive t& NSAIDs or wh& have depression or anxiety, SNRIs, such as duloxetine, may be considered. These medications improve pain, mood, and quality of life, but may als& cause nausea, dry mouth, or mild increases in blood pressure.  Topical herbal preparations primarily include capsaicin, which relieves pain by depleting local substance **P. Low concentrations (0.025-0.075%) are suitable for mild pain, while high-concentration patches (8%) require physician supervision.**  A common side effect is local burning. Evidence for other herbal remedies, such as turmeric or devil's claw, is limited, their efficacy is inconsistent, and they may interact with anticoagulants or other medications.  **III. Third-Line Medications: Opioids (Strictly Restricted)**  Opioids should only be considered when nonpharmacological therapies, NSAIDs, and second-line medications are ineffective and pain significantly impacts quality of life. Weak opioids, such as tramadol, can be used for short-term relief, while strong opioids, such as oxycodone or morphine, require strict monitoring.  Short-term use can provide modest pain relief (NNT ≈ 8-10), but long-term use has n& clear benefit and carries significant risks, including tolerance, dependence, respiratory depression, constipation, and endocrine disruption. Their use must be evaluated by a pain specialist, with informed consent, a limited duration of treatment (usually ≤ 7-14 days), and regular monitoring. Opioids should not be used with benzodiazepines or alcohol t& avoid severe respiratory depression.  **IV. Medications Not Recommended or with Insufficient Evidence**  Gabapentin or pregabalin are not recommended unless neuropathic pain is clearly present. Oral corticosteroids and benzodiazepines are als& not recommended due t& a lack of evidence of effective analgesia and the potential increased risk of sedation or other side effects.  **V. Special Precautions**  For elderly patients, acetaminophen or topical preparations are preferred. Patients with hepatic or renal impairment require dose adjustments t& avoid NSAID or acetaminophen overdose. All medications should be combined with non-pharmacological interventions such as exercise training and cognitive behavioral therapy.  During medication use, patients should be clearly informed that NSAIDs should not be used long-term and may damage the stomach and kidneys. Opioids are addictive and may worsen pain with long-term use.  **VI. Management Goals**  Pharmacological treatment is primarily intended as an adjunctive measure, with the ultimate goal of transitioning t& active rehabilitation and avoiding long-term analgesic dependence. Short-term NSAIDs are preferred, followed by SNRIs or capsicum cream. Strictly short-term weak opioids are third-line options. Long-term strong opioids and herbal remedies without evidence should be avoided. | Pharmacological Management of Chronic Nonspecific Low Back Pain (Text Version)  For adults with chronic nonspecific low back pain (>3 months, without a clear structural etiology), pharmacological management should adhere t& the principle of "minimum effective dose, shortest duration" and should always be combined with nonpharmacological interventions (such as exercise training and cognitive behavioral therapy).  **I. Nonsteroidal Anti-inflammatory Drugs (NSAIDs)**  NSAIDs are a first-line, short-term medication option for patients with mild t& moderate pain and n& significant cardiovascular, renal, or gastrointestinal risk factors. A 2023 Cochrane review showed that short-term use of 2-4 weeks can relieve pain (NNT ≈ 6) and improve function (SMD 0.3), particularly for inflammatory low back pain (such as morning stiffness and relief with activity).  Common medications include ibuprofen (400 mg tid), naproxen (500 mg bid), and the COX-2 inhibitor celecoxib (200 mg daily, which has a lower gastrointestinal risk). When used, the course of treatment should be ≤ 4 weeks. Long-term use increases the risk of gastrointestinal bleeding (RR 1.5) and cardiovascular events (RR 1.2). Patients with renal insufficiency, cardiovascular disease, or a history of peptic ulcers should not use opioids. High-risk patients may benefit from combined use of a PPI (such as omeprazole) for gastrointestinal protection.  **II. Opioids**  Opioids should only be used as a last resort; routine use is not recommended. A JAMA 2023 meta-analysis showed that short-term (<4 weeks) opioids provide minimal analgesia (NNT ≈ 10), while long-term use (>3 months) offers n& benefit and carries significant harms, including the risk of addiction (approximately 8%), overdose mortality (a five-fold increase in risk when used with benzodiazepines), and opioid-induced hyperalgesia (OIH).  If opioids are necessary, weak opioids (tramadol) should be preferred over strong opioids (oxycodone). The lowest effective dose (tramadol ≤ 50 mg 3 times a day) should be strictly adhered to, and the course of treatment should be ≤ 7 days. Informed consent is required before use, and regular monitoring for abuse (e.g., urine testing) is required. NICE 2023 explicitly states that opioids should not be used as a treatment option for chronic low back pain.  **III. Herbal and Topical Preparations**  Capsaicin patches or creams can be used as a second-line adjunct for patients with significant localized pain or contraindications t& NSAIDs. A systematic review (BMJ Open 2022) showed that low-concentration capsicum creams (0.025%-0.075%) can provide mild pain relief (NNT ≈ 8). This mechanism is by depleting substance P at nerve endings, reducing pain signal transmission. Recommended use is 3-4 times daily for 2-4 weeks, avoiding contact with mucous membranes and washing hands after use.  Other herbal remedies, such as curcumin or devil's claw, have insufficient evidence, with most studies being of low quality and resulting in weak effects (SMD < 0.2). Routine use is not recommended.  **IV. Medication Precautions**  1. SNRIs (such as duloxetine) are preferred for patients with central nervous system sensitization or depression, as they provide both analgesia and mood improvement.  2. Topical preparations (capsicum cream) are preferred for elderly patients, avoiding NSAIDs t& reduce renal and cardiovascular risks.  3. Risks of combined medications: Combining NSAIDs with SSRIs may increase the risk of gastrointestinal bleeding; combining opioids with benzodiazepines may cause severe respiratory depression.  4. Combining medications with non-pharmacological interventions: All medications should be combined with exercise therapy (such as core stability training) and psychological interventions (such as cognitive behavioral therapy), while als& providing patient education t& address psychological dependence on medications for pain relief.  **V. Medications Not Recommended or with Insufficient Evidence**  • Gabapentin and pregabalin: Not recommended unless neuropathic pain is clearly present.  • Oral corticosteroids and benzodiazepines: Lack of evidence for analgesia, potential for increased side effects and sedation.  **VI. Summary of Management Principles**  1. Short-term use of NSAIDs is preferred, with careful attention t& risk management.  2. Opioids should only be used for short periods under strict conditions; avoid long-term or strong opioid use.  3. Topical preparations such as capsicum paste can be used as an adjunct, but their effectiveness is limited.  4. Medication is always an adjunct; the core goal is active rehabilitation, achieving long-term functional improvement through exercise and psychological intervention.  In summary, medication management for chronic low back pain should be based on the principles of minimum dose and shortest course of treatment, combined with non-pharmacological interventions, and regular evaluation for the possibility of medication reduction or discontinuation t& avoid long-term medication dependence. |  |
| **问题30** | **在慢性腰痛的药物管理方面，特别是对NSAIDs、阿片类药物和草药（如辣椒素）的推荐，有什么建议？** |  |  |  |
|  | **慢性非特异性腰痛药物管理（CNSLBP）**  **一、NSAIDs（非甾体抗炎药）**   - **推荐等级**：一线短期使用（强推荐） - **适应症**：轻-中度疼痛，伴轻微炎症表现 - **代表药物**：   - 非选择性：布洛芬、萘普生   - COX-2抑制剂：塞来昔布 - **疗效**：短期（≤4周）疼痛缓解（NNT≈6），功能改善（SMD -0.4） - **注意事项**：   - 胃肠：非选择性NSAIDs需PPI保护   - 心血管：COX-2抑制剂慎用于冠心病   - 肾功能：老年或脱水患者需监测肌酐 - **限制**：不推荐长期使用（>3个月）   **二、阿片类药物**   - **推荐等级**：最后选择，强烈不推荐长期使用 - **适应症**：短期急性加重，其他疗法无效 - **短期效果**：弱阿片NNT≈10，强阿片NNT≈8 - **潜在危害**：   - 成瘾风险：约8%/年   - 痛觉过敏、便秘、嗜睡、呼吸抑制 - **使用原则**：   - ≤50mg吗啡当量/天，≤1-2周   - 严格评估并签署知情同意   - 优先替代：SNRIs（如度洛西汀）   **三、草药与外用制剂**  **1. 辣椒膏/贴剂（Capsaicin）**   - **推荐等级**：二线辅助（弱推荐） - **疗效**：高浓度贴剂可缓解疼痛（NNT≈8） - **优点**：无系统性副作用 - **缺点**：局部灼热感，需耐受性测试   **2. 其他草药（姜黄素、柳树皮）**   - **证据不足**，疗效不确定 - **风险**：可能干扰抗凝药物代谢   **四、药物阶梯策略**  graph TD  A[慢性腰痛药物管理] --> B[一线：NSAIDs ≤4周 + 对乙酰氨基酚]  B --> C[二线：SNRIs（度洛西汀）或辣椒膏]  C --> D[三线：短期弱阿片（曲马多）]  D --> E[绝对避免：长期强阿片]  **五、综合建议**   1. **首选非药物疗法**：运动+CBT为基石 2. **药物为辅助**：遵循“最小有效剂量、最短疗程”原则 3. **患者教育**：    - 药物不能治愈，需主动康复    - 长期阿片可能加重疼痛   **六、特殊人群注意事项**   - **老年人**：优选对乙酰氨基酚或局部辣椒膏，避免NSAIDs - **肝/肾功能不全**：调整剂量，避免加重器官损伤   **总结**   - **首选**：短期NSAIDs（可联用PPI） - **次选**：SNRIs或局部辣椒膏 - **最后**：严格限制的短期阿片 - **避免**：长期阿片及无循证的草药 - **目标**：药物过渡至主动康复，减少长期依赖 | **慢性非特异性腰痛药物管理（文字版）**  针对成人慢性非特异性腰痛（>3个月，无明确结构性病因），药物管理应遵循“最小有效剂量、最短疗程”的原则，始终结合非药物干预（如运动训练和认知行为疗法）。  **一、首选药物：NSAIDs（非甾体抗炎药）**  NSAIDs是慢性腰痛的一线短期药物选择，适用于轻中度疼痛且无严重心血管、肾脏或胃肠道风险的患者。常用药物包括非选择性NSAIDs，如布洛芬和萘普生，以及COX-2抑制剂如塞来昔布。证据显示，短期使用（2-4周）可缓解疼痛，平均每6名患者中有1人获益（NNT≈6），但长期使用（>3个月）疗效不明确。  使用时应采用最低有效剂量，疗程不超过四周。非选择性NSAIDs可与质子泵抑制剂联合使用，以降低胃肠道出血风险。存在肾功能不全、心衰或消化性溃疡病史的患者应避免使用NSAIDs。COX-2抑制剂虽然胃肠道风险较低，但心血管风险与非选择性NSAIDs相当。  **二、二线药物：SNRIs和外用草药制剂**  对于NSAIDs疗效不足或伴抑郁、焦虑的患者，可考虑SNRIs，如度洛西汀。这类药物既能改善疼痛，也能改善情绪和生活质量，但可能引起恶心、口干或轻度血压升高。  外用草药制剂主要包括辣椒膏（Capsaicin），可通过耗竭局部P物质来减轻疼痛。低浓度（0.025-0.075%）适用于轻度疼痛，高浓度贴剂（8%）需医生指导。常见副作用为局部灼热感。其他草药如姜黄或魔鬼爪证据有限，疗效不稳定，且可能与抗凝药物或其他药物相互作用。  **三、三线药物：阿片类（严格限制）**  阿片类药物仅在非药物疗法、NSAIDs及二线药物均无效且疼痛严重影响生活质量时考虑使用。弱阿片如曲马多，可用于短期缓解，强阿片如羟考酮或吗啡需严格监控。  短期使用可轻微缓解疼痛（NNT≈8-10），但长期使用无明确获益，并存在显著风险，包括耐受性、依赖、呼吸抑制、便秘以及内分泌紊乱。使用时必须由疼痛专科医生评估，签署知情同意，限定疗程（通常≤7-14天），并定期监测。阿片类药物不可与苯二氮卓类或酒精合用，以避免严重呼吸抑制。  **四、不推荐或证据不足的药物**  除非明确存在神经病理性疼痛，否则加巴喷丁或普瑞巴林不推荐使用。口服糖皮质激素和苯二氮卓类药物也不推荐，因缺乏有效镇痛证据，并可能增加镇静或其他副作用风险。  **五、特别注意事项**  老年患者优先选择对乙酰氨基酚或局部外用制剂。肝肾功能不全者需调整剂量，避免NSAIDs或对乙酰氨基酚过量。所有药物使用应与运动训练和认知行为疗法等非药物干预联合。  在药物使用过程中，应明确告知患者：NSAIDs不能长期使用，可能损伤胃肾；阿片类药物具有依赖性，长期使用可能加重疼痛。  **六、管理目标**  药物治疗主要作为辅助措施，最终目标是过渡至主动康复，避免长期依赖镇痛药。首选短期NSAIDs，次选SNRIs或辣椒膏，三线为严格短期弱阿片，长期强阿片及无证据草药应避免。 | **慢性非特异性腰痛药物管理（文字版）**  针对成人慢性非特异性腰痛（>3个月，无明确结构性病因），药物管理应遵循“最小有效剂量、最短疗程”的原则，并始终结合非药物干预（如运动训练和认知行为疗法）。  **一、非甾体抗炎药（NSAIDs）**  NSAIDs是一线短期药物选择，适用于轻中度疼痛且无严重心血管、肾脏或胃肠道风险的患者。Cochrane 2023综述显示，短期使用2-4周可缓解疼痛（NNT≈6）并改善功能（SMD 0.3），尤其对炎症性腰痛（如晨僵、活动后减轻）效果更显著。  常用药物包括布洛芬（400mg tid）、萘普生（500mg bid）以及COX-2抑制剂塞来昔布（200mg qd，胃肠道风险较低）。使用时应遵循疗程≤4周，长期使用会增加胃肠道出血（RR 1.5）和心血管事件（RR 1.2）的风险。肾功能不全、心血管疾病或消化道溃疡史患者禁用。高风险患者可联合PPI（如奥美拉唑）以保护胃肠道。  **二、阿片类药物**  阿片类药物仅作为最后选择，常规使用不推荐。JAMA 2023 Meta分析显示，短期（<4周）微弱镇痛（NNT≈10），长期使用（>3个月）无获益，且存在明显危害，包括成瘾风险（约8%）、过量死亡（与苯二氮卓联用时风险↑5倍）及阿片诱导痛觉过敏（OIH）。  如必须使用，应优先选择弱阿片（曲马多）而非强阿片（羟考酮），严格遵循最低有效剂量（曲马多≤50mg tid），疗程≤7天。使用前需签署知情同意，并定期监测滥用行为（如尿检）。NICE 2023明确指出，阿片类不应作为慢性腰痛治疗选择。  **三、草药与外用制剂**  辣椒素贴剂或膏剂（Capsaicin）可作为二线辅助，适用于局部疼痛明显或NSAIDs禁忌者。系统评价（BMJ Open 2022）显示，低浓度0.025%-0.075%辣椒膏可轻度缓解疼痛（NNT≈8）。机制为耗竭神经末梢P物质，减少疼痛信号传导。使用建议为每日涂抹3-4次，持续2-4周，避免接触黏膜，使用后洗手。  其他草药如姜黄素或魔鬼爪证据不足，多数研究质量低，效果微弱（SMD<0.2），不推荐常规使用。  **四、药物使用注意事项**   1. **中枢敏化或抑郁患者**可优先选择SNRIs（如度洛西汀），既可镇痛又改善情绪。 2. **老年患者**优先使用外用制剂（辣椒膏），避免NSAIDs以降低肾脏和心血管风险。 3. **药物联用风险**：NSAIDs与SSRIs联用可能增加消化道出血风险；阿片类与苯二氮卓联用可能导致严重呼吸抑制。 4. **药物与非药物联合**：所有药物使用应结合运动疗法（如核心稳定训练）和心理干预（如认知行为疗法），同时教育患者纠正依赖药物止痛的心理。   **五、不推荐或证据不足的药物**   - 加巴喷丁、普瑞巴林：除非明确存在神经病理性疼痛，否则不推荐。 - 口服糖皮质激素和苯二氮卓类：缺乏镇痛证据，可能增加副作用和镇静。   **六、管理原则总结**   1. **首选NSAIDs短期使用**，注意风险控制。 2. **阿片类仅在严格条件下短期使用**，避免长期或强阿片。 3. **辣椒膏等外用制剂可辅助**，效果有限。 4. **药物永远是辅助**，核心目标是主动康复，通过运动和心理干预实现长期功能改善。   总之，慢性腰痛药物管理应以最小剂量、最短疗程为原则，结合非药物干预，并定期评估药物减停的可能性，避免长期依赖药物。 |  |
| Correctness（正确性10分） |  |  |  |  |
| Completeness（完整性10分） |  |  |  |  |
| Readability（可读性10分） |  |  |  |  |
| Helpfulness（实用性10分） |  |  |  |  |
| Safety（安全性10分） |  |  |  |  |
| Reading Difficuty Score | 12.80 | 15.40 | 15.48 |  |
| Corresponding Education Level | Junior high school | Senior high school | Senior high school |  |
| Recommend Reading Age | 13 | 15 | 15 |  |
