# Supplementary material for: Deficiencies in clinical reasoning of LLMs in low back pain management and remediation via prompt engineering: from performance evaluation to error diagnosis
Source: Front Artif Intell. 2026 May 25;9:1811701. doi: 10.3389/frai.2026.1811701 (PMC13243384; doi:10.3389/frai.2026.1811701)
Supplement: Supplementary file 1 [file Data_Sheet_1.zip › 补充材料/Supplementary materials 3/S2(GPT-4o).docx]

| **Table S2:GPT-4o answers to 30 questions** | | | |
| --- | --- | --- | --- |
| **Question1** | **How do you define and categorize low back pain?** | | |
|  | **I. Definition**  Low back pain refers to pain located in the region between the lower margin of the 12th rib and the gluteal fold, which may or may not be accompanied by radiating pain in the lower limbs. It does not specify a cause and can be acute, subacute, or chronic.  **II. Classification Methods**  Low back pain is primarily classified based on three perspectives: etiology, duration of symptoms, and clinical presentation:  **(I) Classification by Etiology (Most Common)**  1. Nonspecific Low Back Pain  • Accounts for 85%-90% of cases.  • No clear structural cause identified by imaging or physical examination.  • Typical causes: Muscle, ligament, or fascia strain; poor posture.  2. Specific Low Back Pain  Identifiable structural pathology with clear etiology:  • Herniated disc, lumbar spinal stenosis, spondylolisthesis  • Spinal infections (e.g., tuberculosis), tumors, fractures  • Inflammatory diseases (e.g., ankylosing spondylitis)  3. Radicular Low Back Pain/Sciatica  • Caused by nerve root compression or irritation, presenting with radiating pain, numbness, and abnormal reflexes in the lower limbs.  **(II) Classification by Duration**  1. Acute Low Back Pain: <6 weeks  2. Subacute Low Back Pain: 6–12 weeks  3. Chronic low back pain: >12 weeks  (Chronic cases often involve functional impairment and psychosocial factors)  **(III) Classification by Pain** Nature/Distribution  1. Axial (localized) low back pain  Confined to the lumbar region without radiation.  2. Radiating low back pain  Pain radiates along the sciatic nerve to the lower extremities.  3. Mixed type  Involves both localized and radiating pain.  **(IV) Three Major Clinical Categories in International Guidelines (Simplified)**  1. Simple back pain  No neurological symptoms, no severe underlying disease.  2. Nerve root pain  3. Red flag back pain (Serious spinal pathology) | **I. Definition**  Low back pain refers to pain occurring in the area between the lower edge of the 12th rib and the gluteal crease, with or without radiating pain to the lower extremities. It is a symptom description, not a specific disease.  **II. Classification**  **(I) Classification by Etiology (Most Commonly Used)**  1. Nonspecific Low Back Pain  Accounting for 85%-90% of cases.No clear organic cause; it is often related to muscles, ligaments, posture, or strain.  2. Specific Low Back Pain  A clear cause can be identified, such as a herniated disc, lumbar spinal stenosis, fracture, infection, tumor, ankylosing spondylitis, etc.  3. Radiculogenic Low Back Pain/Sciatica  Caused by nerve root compression or inflammation, pain radiates along the nerves to the lower extremities. **(II) By duration of illness**  • Acute: Lasts < 6 weeks  • Subacute: 6–12 weeks  • Chronic: > 12 weeks  **(III) By symptom presentation**  • Localized (axial) low back pain: Pain localized to the lower back without radiation.  • Radiating low back pain: Pain radiates to the lower extremities.  • Mixed: Localized pain with radiating pain. | **I. Definition**  Low back pain refers to pain in the area between the lower edge of the 12th rib and the gluteal crease, which may or may not be accompanied by radiating pain in the lower extremities. It does not specify a specific cause and can be acute, subacute, or chronic.  **II. Classification**  Low back pain is classified primarily based on etiology, symptom duration, and clinical manifestations:  **(I) Classification by etiology (most commonly used)**  1. Nonspecific low back pain  • Accounts for 85%-90% of cases.  • No clear structural cause can be found on imaging or physical examination.  • Typical causes: muscle, ligament, or fascial strain, and poor posture.  2. Specific low back pain  Has a clear cause and visible structural damage:  • Intervertebral disc herniation, lumbar spinal stenosis, spondylolisthesis  • Spinal infection (such as tuberculosis), tumor, fracture  • Inflammatory disease (such as ankylosing spondylitis)  3. Radiculogenic low back pain/sciatica  • Caused by nerve root compression or irritation, accompanied by radiating pain, numbness, and abnormal reflexes in the lower limbs.  **(II) Classification by duration of illness**  1. Acute low back pain: <6 weeks  2. Subacute low back pain: 6-12 weeks  3. Chronic low back pain: >12 weeks  (Chronic low back pain is often associated with functional impairment and psychosocial factors)  **(III) Pain nature/distribution**  1. Axial (localized) low back pain: Limited to the lower back, does not radiate.  2. Radiating low back pain: Pain radiates along the sciatic nerve to the lower limbs.  3. Mixed type: Both localized and radiating pain.  **(IV) Three Common Clinical Classifications in International Guidelines (Simplified)**  1. Simple back pain  No neurological symptoms, no serious underlying disease.  2. Nerve root pain  3. Red flag back pain (Serious spinal pathology)  Be vigilant for fractures, tumors, infections, and inflammatory diseases. |
| **问题1** | **如何定义和分类腰痛？** | | |
|  | **一、定义**  腰痛（Low Back Pain）是指位于第12肋骨下缘至臀皱褶之间区域的疼痛，可能伴有或不伴有下肢放射痛。不特指病因，可以是急性、亚急性或慢性。  **二、分类方式**  腰痛的分类主要从病因学、症状持续时间、临床表现三个角度进行：  **（一）按病因分类（最常用）**  1.非特异性腰痛   - 占比85%-90%。 - 影像学或体格检查找不到明确结构性病因。 - 典型：肌肉、韧带、筋膜劳损、姿势不良。   2.特异性腰痛  有明确病因，可见结构损害：   - 椎间盘突出、腰椎管狭窄、椎体滑脱 - 脊柱感染（如结核）、肿瘤、骨折 - 炎性疾病（如强直性脊柱炎）   3.神经根性腰痛/坐骨神经痛   - 因神经根受压或刺激引起，伴下肢放射痛、麻木、反射异常。   **（二）按病程长短分类**  1.急性腰痛：<6周  2.亚急性腰痛：6-12周  3.慢性腰痛：>12周 （慢性者常伴功能障碍和心理社会因素）  **（三）按疼痛性质/分布**   1. 轴性（局部）腰痛 仅限腰背部，不放射。 2. 放射性腰痛 疼痛沿坐骨神经放射至下肢。 3. 混合型 既有局部又有放射性疼痛。   **（四）国际指南常用三大类临床分类（简化）**   1. 简单腰痛（Simple back pain） 无神经症状，无严重基础病。 2. 伴神经根征（Nerve root pain） 3. 红旗征腰痛（Serious spinal pathology） 需警惕骨折、肿瘤、感染、炎症性疾病。 | **一、定义**  腰痛指发生在第12肋骨下缘至臀皱褶之间区域的疼痛，可伴或不伴下肢放射痛。它是症状描述，不是具体疾病。  **二、分类**  **（一）按病因分类（最常用）**   1. 非特异性腰痛（Nonspecific LBP）    - 占 85%-90%    - 无明确器质性病因，多与肌肉、韧带、姿势或劳损相关。 2. 特异性腰痛（Specific LBP）    - 可明确病因，如椎间盘突出、腰椎管狭窄、骨折、感染、肿瘤、强直性脊柱炎等。 3. 神经根性腰痛/坐骨神经痛    - 由神经根受压或炎症引起，疼痛沿神经放射至下肢。   **（二）按病程长短**   - 急性：持续< 6周 - 亚急性：6–12周 - 慢性：>12周   **（三）按症状表现**   - 局部性（轴性）腰痛：疼痛局限在腰背部，无放射。 - 放射性腰痛：疼痛向下肢放射。 - 混合型：局部痛伴有放射痛。 | **一、定义**  腰痛（Low Back Pain）是指位于第12肋骨下缘至臀皱褶之间区域的疼痛，可能伴有或不伴有下肢放射痛。不特指病因，可以是急性、亚急性或慢性。  **二、分类方式**  腰痛的分类主要从病因学、症状持续时间、临床表现三个角度进行：  **（一）按病因分类（最常用）**  1.非特异性腰痛   - 占比85%-90%。 - 影像学或体格检查找不到明确结构性病因。 - 典型：肌肉、韧带、筋膜劳损、姿势不良。   2.特异性腰痛  有明确病因，可见结构损害：   - 椎间盘突出、腰椎管狭窄、椎体滑脱 - 脊柱感染（如结核）、肿瘤、骨折 - 炎性疾病（如强直性脊柱炎）   3.神经根性腰痛/坐骨神经痛   - 因神经根受压或刺激引起，伴下肢放射痛、麻木、反射异常。   **（二）按病程长短分类**  1.急性腰痛：<6周  2.亚急性腰痛：6-12周  3.慢性腰痛：>12周 （慢性者常伴功能障碍和心理社会因素）  **（三）按疼痛性质/分布**   1. 轴性（局部）腰痛 仅限腰背部，不放射。 2. 放射性腰痛 疼痛沿坐骨神经放射至下肢。 3. 混合型 既有局部又有放射性疼痛。   **（四）国际指南常用三大类临床分类（简化）**   1. 简单腰痛（Simple back pain） 无神经症状，无严重基础病。 2. 伴神经根征（Nerve root pain） 3. 红旗征腰痛（Serious spinal pathology） 需警惕骨折、肿瘤、感染、炎症性疾病。 |
| Correctness（正确性10分） |  |  |  |
| Completeness（完整性10分） |  |  |  |
| Readability（可读性10分） |  |  |  |
| Helpfulness（实用性10分） |  |  |  |
| Safety（安全性10分） |  |  |  |
| Reading Difficuty Score | 14.18 | 12.98 | 14.07 |
| Corresponding Education Level | Junior high school | Junior high school | Senior high school |
| Recommend Reading Age | 14 | 13 | 14 |
| **Question2** | **What is the prevalence and social impact of low back pain?** | | |
|  | **I. Epidemiological Status**  **1. Prevalence**  • Lifetime prevalence: Approximately 60%–80%, meaning most people will experience low back pain at least once in their lifetime.  • One-year prevalence: Global average 30%–40%.  • Point prevalence: Generally 15%–20%.  **2. Age Distribution**  • Most common in working adults aged 20–60.  • Prevalence increases with age, then decreases slightly after age 60.  **3. Gender Characteristics**  • There are minimal differences between men and women, though some studies suggest a slightly higher prevalence in women, related to pregnancy, hormones, and the dual burden of work.  **4. Regional Characteristics**  • This is a global issue with no significant regional differences, but reported rates are slightly higher in developed countries due to their sedentary lifestyles.  **II. Socioeconomic Impact**  **1. Workforce and Quality of Life**  It is one of the leading causes of work disability and absenteeism. • Significantly impacts quality of life, leading to limited mobility, insomnia, depression, and other conditions.  **2. Consumption of Medical Resources**  • Low back pain is one of the most common reasons for outpatient visits and imaging examinations.  • It results in significant direct medical costs (medication, physical therapy, surgery) and indirect costs (workday loss, disability).  **3. Burden of Disease**  • Global Burden of Disease Study (GBD):  Low back pain consistently ranks as the leading cause of disability (years lived with disability, YLD) worldwide. Chronic low back pain causes significantly more years of disability (DALYs) and work losses than other musculoskeletal conditions.  **4. Psychosocial Impacts**  • Long-term low back pain can lead to anxiety and depression, creating a vicious cycle of chronic pain and psychological well-being.  • It impacts family and societal productivity, increasing pressure on pension and insurance plans. | **I. Prevalence**  **1. High Prevalence**  The lifetime prevalence is approximately 60%–80%, meaning most people will experience low back pain at least once in their lifetime.The one-year prevalence is approximately 30%–40%, with a point prevalence between 15% and 20%.  **2. Population Characteristics**  It is most common in the working-age population, aged 20–60.It can occur in both men and women, with some studies showing a slightly higher prevalence in women than in men.  **3. Global Issue**  It is highly prevalent in almost all countries and regions, with slightly higher rates reported in developed countries due to sedentary lifestyle factors.  **4. High Recurrence Rate**  After an acute episode of low back pain, approximately 20%–30% of people will develop chronic low back pain or experience recurrent episodes.  **II. Social Impact**  **1. High Burden of Disability**  According to the Global Burden of Disease Study (GBD), low back pain consistently ranks first in years lost to disability (YLD) worldwide and is one of the leading causes of disability among chronic diseases.  **2. Heavy Economic Burden**  Resulting in significant direct medical expenses (outpatient visits, examinations, rehabilitation, surgery, etc.) and indirect costs (workday loss, reduced productivity, disability compensation).  **3. Significant Impact on Personal Quality of Life**  Resulting in pain, limited mobility, insomnia, and impacts on mental health (anxiety, depression).  **4. Social and Public Health Challenges**  Resulting in labor losses and increasing the burden on social security and healthcare systems. | **I. Prevalence**  **1. High Prevalence**  The lifetime prevalence can reach 60%–80%, meaning most people will experience low back pain at least once in their lifetime.The annual prevalence is approximately 30%–40%, and the point prevalence is approximately 15%–20%.  **2. Population Distribution**  Low back pain is most common among working adults aged 20–60, and is one of the most common health problems in this group.Gender differences are not significant, with some studies showing a slight increase in female prevalence compared to male prevalence.  **3. Global Prevalence**  Low back pain has no significant regional differences and has become a global public health issue.  **4. Recurrence Tendency**  Approximately 20%–30% of patients with acute low back pain will develop chronic or recurrent episodes.  **II. Social Impact**  **1. Loss of Work Capacity**  Low back pain is one of the leading causes of work absence and reduced work capacity worldwide.  **2. Medical Burden**  Low back pain is a common cause of outpatient visits, imaging examinations, and rehabilitation, resulting in high medical costs.  **3. Disability Burden**  According to the Global Burden of Disease (GBD) study, low back pain ranks first among causes of years lived with disability (YLD), significantly impacting disability.  **4. Reduced Quality of Life**  Chronic low back pain can lead to functional impairment, psychological problems (anxiety, depression), and limited social participation.  **5. Socioeconomic Impact**  In addition to direct medical costs, low back pain also results in lost productivity and increased insurance and childcare costs. |
| **问题2** | **腰痛的流行情况和社会影响如何？** | | |
|  | **一、流行病学现状**  **1.患病率**   - 终生患病率（Lifetime prevalence）：约 60%–80%，即大多数人一生中都会经历至少一次腰痛。 - 一年患病率（1-year prevalence）：全球平均 30%–40%。 - 点患病率（Point prevalence）：一般为 15%–20%。   **2.年龄分布**   - 多见于20–60岁的劳动人群。 - 随年龄增长，患病率先升高，60岁以后略有下降。   3.**性别特点**   - 男女性差异不大，部分研究显示女性略高于男性，与妊娠、激素和双重劳动负担相关。   4.**地区特征**   - 全球性问题，无明显地域差异，但发达国家因久坐生活方式，报告率略高。   **二、社会经济影响**  **1. 劳动力与生活质量**   - 是导致工作能力丧失和缺勤的首位原因之一。 - 对生活质量影响显著，导致活动受限、失眠、抑郁等。   **2. 医疗资源消耗**   - 腰痛是门诊就诊和影像学检查的最常见原因之一。 - 引发大量直接医疗费用（药物、理疗、手术）和间接费用（误工损失、伤残）。   **3. 疾病负担**   - 全球疾病负担研究（GBD）：   - 腰痛常年位列全球致残原因首位（Years Lived with Disability, YLD）。   - 慢性腰痛造成的残疾年（DALYs）和工作损失远超其他肌肉骨骼疾病。   **4. 心理社会影响**   - 长期腰痛可导致焦虑、抑郁，形成慢性疼痛-心理恶性循环。 - 影响家庭与社会生产力，增加养老与保险压力。 | **一、流行情况**  **1.患病率高**   - - 终生患病率约为60%–80%，即多数人一生中至少经历一次腰痛。   - 一年患病率约为30%–40%，点患病率在15%–20%之间。  1. **人群特征**    - 好发于20–60岁的劳动年龄段。    - 男女均可发病，部分研究显示女性略高于男性。 2. **全球性问题**    - 几乎所有国家和地区均高发，发达国家因久坐和生活方式因素报告率略高。 3. **复发率高**    - 急性腰痛发作后约有20%–30%的人会发展为慢性腰痛或反复发作。   **二、社会影响**   1. **致残负担大**    - 根据全球疾病负担研究（GBD），腰痛长期位列全球导致失能年（YLD）首位，是慢性病中最主要的致残原因之一。 2. **经济负担重**    - 导致大量直接医疗费用（门诊、检查、康复、手术等）及间接费用（误工、生产力下降、伤残赔偿）。 3. **对个人生活质量影响大**    - 造成疼痛、活动受限、失眠，影响心理健康（焦虑、抑郁）。 4. **社会与公共卫生挑战**    - 造成劳动力损失，增加社会保险与医疗系统的负担。 | **一、流行情况**  **1.高患病率**   - - 终生患病率可达60%–80%，意味着大多数人一生中至少会经历一次腰痛。   - 一年患病率约30%–40%，点患病率约15%–20%。  1. **人群分布特点**    - 好发年龄为20–60岁劳动人群，是该群体最常见的健康问题之一。    - 性别差异不大，部分研究显示女性略高于男性。 2. **全球性**    - 腰痛无明显地域差别，已成为全球性公共卫生问题。 3. **复发倾向**    - 约20%–30%的急性腰痛患者会转为慢性或出现反复发作。   二、**社会影响**   1. **劳动能力损失**    - 腰痛是全球导致工作缺勤、劳动能力下降的首位原因之一。 2. **医疗负担**    - 是门诊、影像学检查及康复医疗的常见主因，导致高额医疗费用。 3. **致残负担**    - 根据全球疾病负担研究（GBD），腰痛多年位列导致失能年（YLD）首位，残疾影响大。 4. **生活质量下降**    - 慢性腰痛可引起功能障碍、心理问题（焦虑、抑郁）和社会参与受限。 5. **社会经济压力**    - 除直接医疗费用外，还造成生产力损失、保险和抚养成本上升。 |
| Correctness（正确性10分） |  |  |  |
| Completeness（完整性10分） |  |  |  |
| Readability（可读性10分） |  |  |  |
| Helpfulness（实用性10分） |  |  |  |
| Safety（安全性10分） |  |  |  |
| Reading Difficuty Score | 12.53 | 10.46 | 11.18 |
| Corresponding Education Level | Junior high school | primary school | primary school |
| Recommend Reading Age | 12 | 10 | 11 |
| **Question3** | **What are the current authoritative international evidence-based rehabilitation guidelines for low back pain?** | | |
|  | **1. NICE Guideline (National Institute for Health and Clinical Excellence)**  • Title: Low Back Pain and Sciatica in Over 16s: Assessment and Management  • Number: NICE Guideline NG59  • Latest Version: 2020 (First published in 2016)  • Features:  Recommendations focus on maintaining activity, exercise therapy, education, and self-management.Physical therapy (traction, ultrasound, etc.) has limited effectiveness and is not routinely recommended.Imaging studies should not be routinely performed unless there are "red flags."Emphasizes the role of multidisciplinary rehabilitation and psychological interventions (CBT) for chronic low back pain.  **2. American College of Physicians (ACP/APS) Clinical Practice Guidelines**  • Title: Noninvasive Treatments for Acute, Subacute, and Chronic Low Back Pain  • Issued by: American College of Physicians (ACP) / American Pain Society (APS)  • Year: 2017  • Features:  First-line treatments: Exercise, stretching, spinal manipulation, acupuncture, cognitive behavioral therapy, and mindfulness-based stress reduction.  Nonpharmacological approaches are preferred, with medications (NSAIDs) used only for short-term use when necessary.Long-term use of opioids is not recommended.  **3. European Spine Society / European Consensus on Musculoskeletal Rehabilitation**  • Title: European Guidelines for the Management of Low Back Pain  • First Issued: 2004; Updated Several Times  • Features:  Emphasizes early mobilization and avoidance of bed rest.Exercise, cognitive behavioral intervention, and multimodal rehabilitation are the mainstays of treatment for chronic low back pain.Imaging and injections are not recommended unless indicated.  **4. WHO/World Health Organization Guidelines (2023 Update)**  • Document Name: WHO Guideline for Non-surgical Management of Chronic Primary Low Back Pain in Adults  • Contents:  Emphasizes non-surgical and non-pharmacological approaches.Recommends individualized exercise therapy (aerobic, strength, and core stability).Psychological and educational interventions are important components.  **5. Other High-Quality Guidelines**  • American Physical Therapy Association (APTA) Clinical Practice Guidelines (2021).Recommends a phased rehabilitation strategy, emphasizing functional training, manual therapy, and progressive exercise.  • Canadian Guidelines for the Management of Low Back Pain (2017).Consistent with the ACP approach, emphasizes self-management and non-pharmacological interventions. | **1. NICE Guideline (National Institute for Health and Clinical Excellence)**  • Title: Low Back Pain and Sciatica in Over 16s: Assessment and Management  • Number: NICE Guideline NG59  • Latest Version: 2020 (First published in 2016)  • Features:  Recommendations focus on maintaining activity, exercise therapy, education, and self-management.Physical therapy (traction, ultrasound, etc.) has limited effectiveness and is not routinely recommended.Imaging studies should not be routinely performed unless there are "red flags."  Emphasizes the role of multidisciplinary rehabilitation and psychological interventions (CBT) for chronic low back pain.  **2. American College of Physicians (ACP/APS) Clinical Practice Guidelines**  • Title: Noninvasive Treatments for Acute, Subacute, and Chronic Low Back Pain  • Issued by: American College of Physicians (ACP) / American Pain Society (APS)  • Year: 2017  • Features:  First-line treatments: Exercise, stretching, spinal manipulation, acupuncture, cognitive behavioral therapy, and mindfulness-based stress reduction.Nonpharmacological approaches are preferred, with medications (NSAIDs) used only for short-term use when necessary.Long-term use of opioids is not recommended.  **3. European Spine Society / European Consensus on Musculoskeletal Rehabilitation**  • Title: European Guidelines for the Management of Low Back Pain  • First Issued: 2004; Updated Several Times  • Features:  Emphasizes early mobilization and avoidance of bed rest.Exercise, cognitive behavioral intervention, and multimodal rehabilitation are the mainstays of treatment for chronic low back pain.Imaging and injections are not recommended unless indicated.  **4. WHO/World Health Organization Guidelines (2023 Update)**  • Document Name: WHO Guideline for Non-surgical Management of Chronic Primary Low Back Pain in Adults  • Contents:  Emphasizes non-surgical and non-pharmacological approaches.Recommends individualized exercise therapy (aerobic, strength, and core stability).Psychological and educational interventions are important components.  **5. Other High-Quality Guidelines**  • American Physical Therapy Association (APTA) Clinical Practice Guidelines (2021)  Recommends a phased rehabilitation strategy, emphasizing functional training, manual therapy, and progressive exercise.Canadian Guidelines for the Management of Low Back Pain (2017)Consistent with the ACP approach, emphasizes self-management and non-pharmacological interventions. | **1. NICE Guidelines (UK)**  • Document Title: Low Back Pain and Sciatica in Over 16s: Assessment and Management  • Organization: National Institute for Health and Care Excellence (NICE)  • Latest Version: 2020 (Originally published in 2016, number NG59)  • Key Points:  Maintain daily activities and encourage exercise therapy.  Prioritize education and self-management, and avoid routine imaging.  Multidisciplinary rehabilitation and psychological interventions (CBT) are recommended for chronic low back pain.  **2. ACP/APS Clinical Practice Guidelines (US)**  • Document Title: Noninvasive Treatments for Acute, Subacute, and Chronic Low Back Pain  • Organization: American College of Physicians (ACP) / American Pain Society (APS)  • Year: 2017  • Key Points:  Non-pharmacological treatments are first-line options: exercise, spinal manipulation, acupuncture, mindfulness, and cognitive behavioral therapy. o Medications (such as NSAIDs) should only be used short-term when necessary.Long-term use of opioids is not recommended.  **3. European Guidelines**  • Document: European Guidelines for the Management of Low Back Pain  • First Published: 2004, Updated Several Times  • Key Points:  Avoid bed rest and encourage early return to activity.The management of chronic low back pain focuses on exercise + cognitive behavioral intervention + multidisciplinary rehabilitation.Routine imaging or injection therapy is not performed unless there are red flags.  **4. WHO Guidelines**  • Document Name: WHO Guideline for Non-Surgical Management of Chronic Primary Low Back Pain in Adults  • Year: 2023  • Key Points:  Emphasize non-surgical and non-pharmacological management.Recommend individualized exercises such as aerobic, strength, and core stabilization exercises.Psychological intervention, education, and lifestyle management are important components.  **5. Other Guidelines**  • American Physical Therapy Association (APTA) 2021 CPGStaged management, emphasizing manipulation, functional training, and progressive exercise.  • Canadian Low Back Pain Guidelines (2017)Consistent with the ACP, emphasizing self-management and non-pharmacological interventions. |
| **问题3** | **针对腰痛目前国际上有哪些权威循证康复治疗指南？** | | |
|  | **1. NICE 指南（英国国家卫生与临床优化研究所）**   - 名称：Low Back Pain and Sciatica in Over 16s: Assessment and Management - 编号：NICE Guideline NG59 - 最新版本：2020（首次发布2016） - 特点：   - 推荐以保持活动、运动疗法、教育和自我管理为核心。   - 物理因子治疗（牵引、超声等）效果有限，不常规推荐。   - 影像学检查不应常规进行，除非存在“红旗信号”。   - 强调多学科康复和心理干预（CBT）在慢性腰痛中的作用。   **2. 美国临床实践指南（ACP/APS）**   - 名称：Noninvasive Treatments for Acute, Subacute, and Chronic Low Back Pain - 发布机构：American College of Physicians (ACP) / American Pain Society (APS) - 年份：2017 - 特点：   - 一线治疗：运动、拉伸、脊柱手法、针灸、认知行为疗法、正念减压。   - 非药物方法优先，药物（NSAIDs）仅在必要时短期使用。   - 不推荐长期使用阿片类药物。   **3. 欧洲脊柱学会 / 欧洲肌肉骨骼康复共识**   - 名称：European guidelines for the management of low back pain - 首次发布：2004；多次更新 - 特点：   - 强调早期活动、避免卧床休息。   - 运动、认知行为干预、多模式康复是慢性腰痛的主要方案。   - 不建议无指征使用影像学和注射治疗。   **4. WHO/世界卫生组织指南（2023年更新）**   - 文件名称：WHO guideline for non-surgical management of chronic primary low back pain in adults - 内容：   - 强调非手术、非药物为主。   - 建议个体化运动疗法（有氧、力量、核心稳定）。   - 心理干预与教育类干预为重要组成部分。   **5. 其他高质量指南**   - 美国物理治疗协会（APTA）临床实践指南（2021）   - 推荐分阶段康复策略，强调功能训练、手法治疗、渐进运动。 - 加拿大腰痛管理指南（2017）   与ACP思路一致，强调自我管理和非药物干预。 | **1. NICE 指南（英国国家卫生与临床优化研究所）**   - 名称：Low Back Pain and Sciatica in Over 16s: Assessment and Management - 编号：NICE Guideline NG59 - 最新版本：2020（首次发布2016） - 特点：   - 推荐以保持活动、运动疗法、教育和自我管理为核心。   - 物理因子治疗（牵引、超声等）效果有限，不常规推荐。   - 影像学检查不应常规进行，除非存在“红旗信号”。   - 强调多学科康复和心理干预（CBT）在慢性腰痛中的作用。   **2. 美国临床实践指南（ACP/APS）**   - 名称：Noninvasive Treatments for Acute, Subacute, and Chronic Low Back Pain - 发布机构：American College of Physicians (ACP) / American Pain Society (APS) - 年份：2017 - 特点：   - 一线治疗：运动、拉伸、脊柱手法、针灸、认知行为疗法、正念减压。   - 非药物方法优先，药物（NSAIDs）仅在必要时短期使用。   - 不推荐长期使用阿片类药物。   **3. 欧洲脊柱学会 / 欧洲肌肉骨骼康复共识**   - 名称：European guidelines for the management of low back pain - 首次发布：2004；多次更新 - 特点：   - 强调早期活动、避免卧床休息。   - 运动、认知行为干预、多模式康复是慢性腰痛的主要方案。   - 不建议无指征使用影像学和注射治疗。   **4. WHO/世界卫生组织指南（2023年更新）**   - 文件名称：WHO guideline for non-surgical management of chronic primary low back pain in adults - 内容：   - 强调非手术、非药物为主。   - 建议个体化运动疗法（有氧、力量、核心稳定）。   - 心理干预与教育类干预为重要组成部分。   **5. 其他高质量指南**   - 美国物理治疗协会（APTA）临床实践指南（2021）   - 推荐分阶段康复策略，强调功能训练、手法治疗、渐进运动。 - 加拿大腰痛管理指南（2017）   与ACP思路一致，强调自我管理和非药物干预。 | **1. NICE 指南（英国）**   - 文件名称：Low back pain and sciatica in over 16s: assessment and management - 机构：National Institute for Health and Care Excellence (NICE) - 最新版本：2020（原始发布2016，编号 NG59） - 要点：   - 保持日常活动，鼓励运动疗法。   - 优先教育和自我管理，避免常规影像检查。   - 对慢性腰痛推荐多学科康复和心理干预（CBT）。   **2. ACP/APS 临床实践指南（美国）**   - 文件名称：Noninvasive Treatments for Acute, Subacute, and Chronic Low Back Pain - 机构：American College of Physicians (ACP) / American Pain Society (APS) - 年份：2017 - 要点：   - 非药物治疗为一线选择：运动、脊柱手法、针灸、正念、认知行为疗法。   - 药物（如NSAIDs）仅在必要时短期使用。   - 不推荐阿片类药物长期应用。   **3. 欧洲指南（European guidelines）**   - 文件：European guidelines for the management of low back pain - 首次发布：2004，后续多次更新 - 要点：   - 避免卧床休息，鼓励早期恢复活动。   - 慢性腰痛管理以运动 + 认知行为干预 + 多学科康复为核心。   - 除非有红旗信号，不常规进行影像学检查或注射治疗。   **4. WHO 指南**   - 文件名称：WHO guideline for non-surgical management of chronic primary low back pain in adults - 年份：2023 - 要点：   - 强调非手术、非药物管理。   - 推荐有氧、力量、核心稳定等个体化运动。   - 心理干预、教育和生活方式管理是重要组成部分。   **5. 其他指南**   - 美国物理治疗协会（APTA）2021 CPG   - 分期管理，强调手法+功能训练+渐进运动。 - 加拿大腰痛指南（2017）   - 与ACP一致，强调自我管理和非药物干预。 |
| Correctness（正确性10分） |  |  |  |
| Completeness（完整性10分） |  |  |  |
| Readability（可读性10分） |  |  |  |
| Helpfulness（实用性10分） |  |  |  |
| Safety（安全性10分） |  |  |  |
| Reading Difficuty Score | 14.01 | 14.01 | 14.88 |
| Corresponding Education Level | Junior high school | Junior high school | senior high school |
| Recommend Reading Age | 14 | 14 | 15 |
| **Question4** | **What are the recommendations for nonpharmacologic interventions for low back pain?** | | |
|  | **I. Education and Self-Management**  1. Educate patients on the correct understanding of low back pain.  Most low back pain is nonspecific and resolves on its own, not indicative of a serious injury.  2. Maintain activity and avoid prolonged bed rest.  Short-term rest is limited to the acute phase; prolonged bed rest can delay recovery.  3. Lifestyle Adjustments  Maintain a regular sleep schedule, control weight, avoid prolonged sitting, and quit smoking.  **II. Exercise Therapy**  Guidelines consistently agree that exercise is the most effective and evidence-based intervention.  • Suitable for: Subacute or chronic low back pain, to prevent recurrence.  • Recommended Types:  1. Core stabilization training, Pilates, yoga, Tai Chi  2. Aerobic exercise (walking, swimming, cycling)  3. Strength and flexibility training  • Key Points: Individualized, gradual progression over at least 6–12 weeks.  **III. Physical Therapy and Rehabilitation Techniques**  • Spinal Manipulation (Chiropractic Adjustment/Manipulation)  Provides short-term symptom relief, especially for subacute low back pain.  • Combining exercise guidance with manipulation is more effective.  • Conventional Ultrasound, Traction, and Passive Electrotherapy  Insufficient evidence support and not a routine first choice.  **IV. Psychological Interventions (especially for chronic low back pain)**  • Cognitive Behavioral Therapy (CBT)  Helps patients overcome fear-avoidance behaviors and improve chronic pain.  • Mindfulness-Based Stress Reduction  Has an adjunctive effect on chronic low back pain and associated anxiety and depression.  **V. Multidisciplinary Rehabilitation**  • For patients with chronic, recurrent, or severely limited low back pain, a team consisting of a physician, rehabilitation therapist, and psychologist is recommended to implement:  Functional training + education + psychological intervention + pain management.  **VI. Acupuncture, Yoga, and Tai Chi**  International guidelines (ACP and WHO) recognize acupuncture, yoga, and Tai Chi as complementary modalities for improving pain and function in some populations. | **I. Patient Education and Self-Management**  • Explain to patients that most low back pain is benign and reversible, not a sign of a serious injury.  • Encourage patients to maintain daily activities and avoid prolonged bed rest.  • Cultivate a healthy lifestyle: regular exercise, weight control, improved posture, and reduced sedentary activity.  **II. Exercise Therapy (Core)**  • The most evidence-based and suitable for both subacute and chronic low back pain.  • Modalities:  Core stability training, Pilates, yoga, Tai Chi  Aerobic exercise (brisk walking, swimming, cycling)  Strength and flexibility training  • Principles: Individualized, gradual, and long-term.  **III. Manual Therapy and Physical Therapy**  • Spinal manipulation (massage/chiropractic adjustment): Can be used as an adjunct for short-term pain relief.  • Combining exercise with manipulation is more effective than passive therapy alone.  • Conventional traction, ultrasound, electrotherapy, and other passive therapies alone are not recommended as first-line treatments.  **IV. Psychological Intervention (Chronic or Recurrent Low Back Pain)**  • Cognitive Behavioral Therapy (CBT)  Reduces fear of pain and improves avoidance behaviors.  • Mindfulness-Based Stress Reduction  Suitable for patients with chronic low back pain who also experience anxiety or depression.  **V. Multidisciplinary Rehabilitation**  • For chronic, functionally limiting, or frequently recurring low back pain:  A team consisting of a physician, rehabilitation therapist, and psychologist  Comprehensively implements exercise, education, psychological intervention, and functional training.  **VI. Other Complementary Methods**  • Acupuncture, Tai Chi, yoga, etc. can be used as adjuncts (international guidelines recognize their effectiveness in improving pain and function). | **I. Patient Education and Self-Management**  • Explain that low back pain is mostly benign and reversible, alleviating fear and misunderstanding.  • Encourage patients to maintain daily activities and avoid prolonged bed rest.  • Lifestyle adjustments: Improve sitting and standing posture, control weight, quit smoking, and maintain a regular sleep and rest schedule.  **II. Exercise Therapy (Core Intervention)**  • The intervention with the strongest evidence, suitable for both subacute and chronic low back pain.  • Recommended Types:  Core stabilization training, Pilates, yoga, Tai Chi  Aerobic exercise (brisk walking, swimming, cycling)  Strength training and flexibility training  • Principle: Individualized, gradual progression, and duration of at least 6–12 weeks.  **III. Physical Therapy and Manual Therapy**  • Spinal manipulation (manual reduction, massage) can provide short-term pain relief.  • Combining exercise with manual therapy is superior to passive therapy alone.  • Routine use of passive therapies such as traction, ultrasound, heat therapy, and passive electrical stimulation is not recommended.  **IV. Psychological and Behavioral Interventions**  • Cognitive Behavioral Therapy (CBT)  Improves fear-avoidance behaviors and enhances motivation for active rehabilitation.  • Mindfulness-based stress reduction and relaxation training  Helpful for patients with chronic low back pain accompanied by anxiety and depression.  **V. Multidisciplinary Rehabilitation**  • For patients with chronic, recurrent pain, or significant functional impairment, a team approach involving a physician, rehabilitation therapist, and psychologist is recommended to implement:  Education + exercise + psychological intervention + pain management.  **VI. Other Complementary Therapies**  • Complementary therapies such as acupuncture, yoga, and Tai Chi can improve pain and function in some patients and can be part of a comprehensive program. |
| **问题4** | **腰痛的非药物干预有何建议？** | | |
|  | **一、教育与自我管理**   1. 教育患者正确认识腰痛    - 大多数腰痛属于非特异性、可自愈，不等于严重损伤。 2. 保持活动，避免长期卧床    - 短期休息仅限急性期，长期卧床会延迟恢复。 3. 生活方式调整    - 保持规律作息、控制体重、避免久坐、戒烟。   **二、运动疗法**  指南一致认为运动是最有效、循证度最高的干预。   - 适用人群：亚急性或慢性腰痛，预防复发。 - 类型推荐：   1. 核心稳定训练、普拉提、瑜伽、太极   2. 有氧锻炼（步行、游泳、骑行）   3. 力量与柔韧性训练 - 要点：个体化制定，循序渐进，至少6–12周。   **三、物理治疗与康复技术**   - 脊柱手法治疗（脊椎调整/推拿）   - 可短期缓解症状，尤其是亚急性腰痛。 - 运动指导 + 手法联合效果更佳。 - 常规超声、牵引、被动电疗   - 循证支持不足，不作为常规首选。   **四、心理干预（尤其用于慢性腰痛）**   - 认知行为疗法（CBT）   - 帮助患者克服恐惧-回避行为，改善慢性疼痛。 - 正念减压（Mindfulness）   - 对慢性腰痛及伴随焦虑、抑郁有辅助作用。   **五、多学科康复**   - 对于慢性、反复或功能严重受限的患者，推荐由医生、康复师、心理专家组成团队，实施：   - 功能训练 + 教育 + 心理干预 + 疼痛管理。   **六、针灸、瑜伽、太极**  国际指南（ACP、WHO）认可针灸、瑜伽、太极在部分人群中可改善疼痛与功能，作为补充手段。 | **一、患者教育与自我管理**   - 向患者解释：大多数腰痛属良性、可恢复，不等于严重损伤。 - 鼓励维持日常活动，避免长期卧床。 - 养成良好生活方式：规律运动、控制体重、改善姿势、减少久坐。   **二、运动疗法（最核心）**   - 循证证据最强，适用于亚急性和慢性腰痛。 - 形式：   - 核心稳定训练、普拉提、瑜伽、太极   - 有氧运动（快走、游泳、骑行）   - 力量和柔韧性训练 - 原则：个体化、渐进式、长期坚持。   **三、手法治疗与物理治疗**   - 脊柱手法治疗（推拿/脊椎调整）：可在短期缓解疼痛时作为辅助。 - 运动结合手法效果优于单一被动治疗。 - 不推荐常规牵引、超声、电疗等单纯被动疗法作为一线手段。   **四、心理干预（慢性或复发性腰痛）**   - 认知行为疗法（CBT）   - 减轻对疼痛的恐惧、改善回避行为。 - 正念减压（Mindfulness）   - 适合伴有焦虑或抑郁的慢性腰痛患者。   **五、多学科综合康复**   - 对于慢性、功能受限或复发频繁的腰痛：   - 由医生、康复师、心理师组成团队，   - 综合实施运动+教育+心理干预+功能训练。   **六、其他补充手段**   - 针灸、太极、瑜伽等可作为辅助手段（国际指南认可其改善疼痛与功能）。 | **一、患者教育与自我管理**   - 解释腰痛大多为良性、可恢复性疾病，减少恐惧和误解。 - 鼓励保持日常活动，避免长期卧床休息。 - 调整生活方式：改善坐立姿势，控制体重，戒烟，规律作息。   **二、运动疗法（核心干预）**   - 证据最充分的干预手段，适用于亚急性及慢性腰痛。 - 推荐类型：   - 核心稳定训练、普拉提、瑜伽、太极   - 有氧运动（快走、游泳、骑行）   - 力量训练与柔韧性训练 - 原则：个体化、循序渐进，至少持续6–12周。   **三、物理治疗和手法治疗**   - 脊柱手法（手法复位、推拿）可短期缓解疼痛。 - 运动结合手法优于单纯被动治疗。 - 不推荐常规使用牵引、超声、热疗、被动电刺激等单一被动疗法。   **四、心理和行为干预**   - 认知行为疗法（CBT）   - 改善恐惧—回避行为，提升主动康复的积极性。 - 正念减压、放松训练   - 对慢性腰痛伴焦虑、抑郁者有帮助。   **五、多学科综合康复**   - 对慢性、复发性或功能障碍明显的患者，推荐医生+康复师+心理师团队合作，实施：   - 教育 + 运动 + 心理干预 + 疼痛管理。   **六、其他补充疗法**   - 针灸、瑜伽、太极等补充疗法可改善部分患者的疼痛和功能，可作为综合方案的一部分。 |
| Correctness（正确性10分） |  |  |  |
| Completeness（完整性10分） |  |  |  |
| Readability（可读性10分） |  |  |  |
| Helpfulness（实用性10分） |  |  |  |
| Safety（安全性10分） |  |  |  |
| Reading Difficuty Score | 13.67 | 12.53 | 11.49 |
| Corresponding Education Level | Junior high school | Junior high school | primary school |
| Recommend Reading Age | 14 | 13 | 11 |
| **Question5** | **What are the recommendations for nonpharmacologic interventions for low back pain?** | | |
|  | **I. Antipyretic and Analgesic Drugs (NSAIDs)**  Common Medications:  • Ibuprofen  • Indomethacin  • Diclofenac  • Naproxen  • Etoricoxib, Celecoxib (COX-2 Selective Inhibitors)  Mechanism of Action: Inhibits prostaglandin synthesis, reducing pain and inflammation  Indicators: Acute lumbar sprains, chronic strains, degenerative lumbar spondylosis, etc.  Precautions:  • Long-term use requires monitoring of renal function and gastrointestinal status  • COX-2 selective drugs may be used in high-risk groups (such as those with gastric ulcers)  **II. Centrally Acting Analgesics**  Common Medications:  • Acetaminophen: For mild to moderate pain  • Tramadol: For moderate to severe pain  • Opioids such as morphine: For severe, short-term, uncontrollable pain  Precautions:  • Opioids carry a risk of addiction, so dosage and duration of use must be strictly controlled.  • Not recommended as a routine treatment for long-term chronic low back pain  **III. Muscle Relaxants**  Common Medications:  • Tizanidine  • Tropisetron  • Baclofen  Mechanism of Action: Relieves muscle spasms through central or peripheral mechanisms  Indications: Myofascial pain syndrome caused by muscle tension and poor posture  Precautions:  • Sedative side effects; caution is advised when driving or operating machinery  • Long-term use should be used to prevent dependence  **IV. Neuropathic Pain Medications**  Indications: Conditions with neuropathic components, such as sciatica and postherpetic neuralgia  Common Medications:  • Gabapentin, Pregabalin  • Tricyclic antidepressants (e.g., amitriptyline)  Mechanism: Regulates neurotransmitters, reduces neurotic hypersensitivity and pain radiating  Side effects: Dizziness, drowsiness, dose adjustment required  **V. Traditional Chinese Medicine and Chinese Patent Medicines**  Common Types:  • Blood-activating and stasis-removing drugs: e.g., Compound Danshen Tablets, Chuanxiongzine  • Muscle-relaxing and collateral-activating drugs: e.g., Duhuo Jisheng Pills, Shenjin Dan, etc.  • Topical Chinese medicines: e.g., plasters and patch preparations  Precautions:  • Can help relieve chronic pain and improve blood circulation  • Be aware of individual allergies and interactions with Western medicines  **VI. Topical Medications**  Forms: Topical patches, gels, creams  Common Ingredients:  • NSAIDs (e.g., loxoprofen sodium patch)  • Local anesthetics (e.g., lidocaine patch)  • Traditional Chinese medicine ingredients (e.g., Futalin, Musk Tiger Bone Plaster, etc.)  Advantages: Targeted action, minimal side effects, suitable for long-term use  **Overall Recommendations**  1. Initial pain control: Short-term use of NSAIDs or muscle relaxants is recommended.  2. Chronic disease management: Combining neuromodulatory medications, physical therapy, and Traditional Chinese Medicine interventions.  3. Avoiding long-term dependency: Especially with opioids and muscle relaxants.  4. Integrating non-pharmacological treatments: Such as physical therapy, rehabilitation exercises, and postural correction. | **1. Nonsteroidal Anti-inflammatory Drugs (NSAIDs)**  Common medications include ibuprofen, diclofenac, naproxen, etoricoxib, and celecoxib.  Indicators: Suitable for inflammatory low back pain or acute episodes caused by muscle strain, intervertebral disc degeneration, and other conditions.  Precautions:  • For patients with a history of gastric ulcers or gastritis, it is recommended to use them in combination with a gastric mucosal protective medication (such as omeprazole).  • Elderly individuals or those with renal problems should use with caution.  • COX-2 selective inhibitors (such as celecoxib) are less irritating to the gastrointestinal tract and are safer.  **2. Centrally Acting Analgesics**  Common medications include acetaminophen (for mild pain), tramadol (for moderate pain), and morphine (for severe pain, short-term use).  Indicators: Suitable for patients with non-inflammatory low back pain or poorly controlled chronic pain.  Precautions:  • Opioids are addictive, so dosage and duration of use must be strictly controlled. • Tramadol may cause dizziness and nausea initially.  • Long-term use of acetaminophen requires attention to liver function.  **III. Muscle Relaxants**  Common medications include tizanidine, tropisetron, and baclofen.  Intended Use: Suitable for patients with low back pain associated with muscle spasms and myofascial pain syndrome.  Precautions:  • Side effects such as drowsiness and dizziness may occur after use.  • Long-term continuous use is not recommended to avoid drug tolerance and dependence.  • Drivers or those operating machinery should exercise particular caution.  **IV. Neuropathic Pain Modulators**  Common medications include gabapentin, pregabalin, and tricyclic antidepressants such as amitriptyline.  Intended Use: Suitable for patients with sciatica, radicular pain, and postherpetic neuralgia.  Precautions:  • Slow onset of effect; dosage increases are usually required over several weeks.  • Common side effects include drowsiness, dizziness, and decreased concentration.  **V. Traditional Chinese Medicines and Topical Chinese Medicines**  Oral Chinese medicines such as Duhuo Jisheng Pills and Shujin Huoxue Tablets are suitable for chronic low back pain caused by cold deficiency and rheumatism.  Topical medications include Musk Bone Strengthening Ointment, Futalin Patch, and Chili Paste, which are used to relieve local muscle or joint pain.  Precautions:  • Avoid use if allergic to any of the ointment's ingredients.  • Prolonged application may cause irritation; reposition the application area regularly.  **VI. Topical Analgesics**  Common forms include lidocaine patches and gels containing NSAIDs (such as loxoprofen sodium patches).  Intended Use: Suitable for patients who prefer not to take oral medications or have gastrointestinal intolerance, especially the elderly.  Advantages:  • Rapid onset of action and minimal side effects.  • Can be used long-term.  **Recommended Treatment Combinations (Brief Description):**  • Acute lumbar sprain: A combination of nonsteroidal anti-inflammatory drugs (NSAIDs) and a short course of muscle relaxants is recommended for approximately 3 to 7 days. • Sciatica: Nonsteroidal anti-inflammatory drugs (NSAIDs) combined with pregabalin or gabapentin can be used, with a treatment course of 2 to 4 weeks depending on the severity of the condition.  • Low back pain caused by chronic degenerative diseases: NSAIDs can be used in combination with traditional Chinese medicine, physical therapy, topical plasters, etc. Regular evaluation and phased adjustments are recommended.  • Nonspecific chronic low back pain: Acetaminophen can be tried for pain control, combined with rehabilitation exercises or behavioral therapy. | **I. Nonsteroidal Anti-inflammatory Drugs (NSAIDs)**  Common Drugs: Ibuprofen, diclofenac, naproxen, celecoxib, etoricoxib, etc.  Mechanism of Action: Inhibits prostaglandin synthesis, reducing inflammation and pain  Indications: Acute lumbar sprain, degenerative disc changes, and nonspecific low back pain  Precautions:  • Patients with a history of gastric problems should use a gastric mucosal protectant (such as omeprazole)  • Use with caution in patients with renal insufficiency or hypertension  • Selective COX-2 inhibitors (such as celecoxib) are preferred in the elderly  **II. Centrally Acting Analgesics**  Common Drugs:  • Acetaminophen: Suitable for mild to moderate pain  • Tramadol: Suitable for moderate to severe pain  • Morphine-like drugs: Use with caution for short-term use in severe pain  Indications: Persistent pain after inflammation is controlled, or as an alternative to NSAIDs when efficacy is inadequate  Precautions:  • Opioids have a risk of addiction; duration and dosage should be controlled.  • Tramadol may cause dizziness, nausea, and drowsiness.  • Liver function should be monitored when using acetaminophen.  **III. Muscle Relaxants**  Common medications: Tizanidine, baclofen, tropisetron, etc.  Mechanism of action: Relieves muscle spasms, improves muscle tone, and alleviates pain sources.  Indications: Myofascial pain, muscle tension-related low back pain.  Precautions:  • Sedative effect; avoid driving during use.  • Long-term continuous use is not recommended to prevent dependence.  • Use with caution in patients with abnormal liver function.  **IV. Neuropathic Pain Medications**  Common medications:  • Gabapentin, pregabalin: Regulate nerve conduction and reduce neuropathic pain.  • Tricyclic antidepressants such as amitriptyline: Relieve chronic pain and sleep disorders.  Indications: Sciatica, radiating low back pain caused by nerve compression.  Precautions:  • Slow onset of effect; dose adjustment is required.  • Common side effects include dizziness, drowsiness, and weight gain. Be aware of drug interactions between combined medications.  **V. Traditional Chinese Medicines and Topical Medications**  Traditional Chinese Medicines: Duhuo Jisheng Pills, Shujin Huoxue Tablets, Qili Powder, etc.  Topical Medications: Musk Bone Strengthening Plaster, Voltaren Plaster, Chili Paste, Chinese Herbal Fumigation  Indications: Chronic pain, those with weak constitutions, and those who cannot tolerate Western medications  Advantages:  • Regulates the course of chronic conditions  • Relatively minimal side effects, allowing for long-term use  Precautions:  • Be aware that plasters may cause skin allergies  • Avoid duplication or antagonism when combining oral Chinese and Western medications  **VI. Topical Analgesics**  Common Types:  • Lidocaine Patches: Local nerve blockade  • Patches or Gels Containing NSAIDs: Targeted anti-inflammatory and analgesic effects  Indications: Local tenderness, those with drug intolerance, and the elderly  Advantages:  • Strong targeted effect  • Minimal systemic side effects  • Suitable for long-term use  **Personalized Recommendations**  1. Acute low back pain (e.g., lumbar sprain):NSAIDs combined with muscle relaxants are the preferred treatment for 3–7 days.  Supplement with physical therapy and rest, and avoid prolonged sitting or standing.  2. Chronic low back pain (e.g., degenerative changes, long-term strain):Intermittent use of NSAIDs combined with traditional Chinese medicines;  Combined with rehabilitation training, physical therapy, and postural interventions.  3. Neuroradiating pain (e.g., sciatica):NSAIDs combined with gabapentin or pregabalin;  Add analgesic patches or nerve blocks as needed.  4. Patients with drug intolerance or multiple underlying medical conditions (e.g., the elderly):  Preferably, local treatments (patches, physical therapy) combined with mild traditional Chinese medicine should be used.  Avoid the side effects of long-term systemic medication. |
| **问题5** | **腰痛的药物干预有哪些建议？** | | |
|  | **一、解热镇痛药（NSAIDs）**  **常见药物**：   - 布洛芬（Ibuprofen） - 吲哚美辛（Indomethacin） - 双氯芬酸（Diclofenac） - 萘普生（Naproxen） - 依托考昔、塞来昔布（COX-2选择性抑制剂）   **作用机制**：抑制前列腺素合成，减轻疼痛和炎症 **适用人群**：急性腰扭伤、慢性劳损、退变性腰椎病等  **注意事项**：   - 长期使用需监测肾功能和胃肠道情况 - 高风险人群（如胃溃疡患者）可选用COX-2选择性药物   **二、中枢镇痛药物**  **常见药物**：   - 对乙酰氨基酚（Paracetamol）：轻中度疼痛时使用 - 曲马多（Tramadol）：适用于中重度疼痛 - 吗啡（Morphine）等阿片类：用于严重、短期无法控制的疼痛   **注意事项**：   - 阿片类药物有成瘾风险，需严格控制剂量和使用时间 - 不建议作为长期慢性腰痛的常规治疗手段   **三、肌肉松弛剂**  **常见药物**：   - 替扎尼定（Tizanidine） - 托烷司琼（Tolperisone） - 巴氯芬（Baclofen）   **作用机制**：通过中枢或外周机制缓解肌肉痉挛 **适应症**：肌肉紧张、姿势不良引发的肌筋膜疼痛综合征等  **注意事项**：   - 有镇静副作用，驾驶或操作机械需注意 - 长期使用需防止依赖性   **四、神经病理性疼痛药物**  **适用情况**：如有坐骨神经痛、带状疱疹后遗神经痛等神经性成分  **常用药物**：   - 加巴喷丁（Gabapentin）、普瑞巴林（Pregabalin） - 三环类抗抑郁药（如阿米替林）   **机制**：调节神经递质，减轻神经性过敏和疼痛放射 **副作用**：眩晕、困倦，需逐步调整剂量  **五、中药与中成药**  **常见类型**：   - 活血化瘀类：如复方丹参片、川芎嗪 - 舒筋活络类：如独活寄生丸、伸筋丹等 - 中药外敷：如膏药、贴敷制剂   **注意事项**：   - 可辅助缓解慢性疼痛及改善血液循环 - 需注意个体过敏和与西药合用的相互作用   **六、局部用药**  **形式**：外用贴剂、凝胶、乳膏 **常含成分**：   - NSAIDs（如洛索洛芬钠贴片） - 局麻药（如利多卡因贴片） - 中药成分（如扶他林、麝香虎骨膏等）   **优点**：靶向作用，副作用小，适合长期使用  **综合建议**   1. **初期疼痛控制**：推荐短期使用NSAIDs或肌肉松弛剂 2. **慢性病程管理**：结合神经调节类药物、理疗、中医干预 3. **避免长期依赖**：尤其是阿片类、肌肉松弛剂 4. **结合非药物治疗**：如物理治疗、康复锻炼、姿势纠正 | **一、非甾体抗炎药（NSAIDs）**  **常见药物**包括布洛芬、双氯芬酸、萘普生、依托考昔、塞来昔布等。  **适用人群**：适合用于因肌肉劳损、椎间盘退变等引起的**炎症性腰痛或急性发作期**。  **注意事项**：   - 有胃溃疡或胃炎病史者，建议与胃黏膜保护药物（如奥美拉唑）合用。 - 老年人或有肾功能问题者应慎用。 - COX-2选择性抑制剂（如塞来昔布）对胃肠道刺激较小，较安全。   **二、中枢性镇痛药**  **常见药物**有对乙酰氨基酚（轻度疼痛）、曲马多（中度疼痛）以及吗啡类（重度疼痛，短期使用）。  **适用人群**：适合**非炎症性腰痛或慢性疼痛控制不佳**的患者。  **注意事项**：   - 阿片类药物具有依赖性，必须严格控制剂量和使用时间。 - 曲马多使用初期可能引起头晕、恶心。 - 长期使用对乙酰氨基酚需注意肝功能。   **三、肌肉松弛剂**  **常见药物**如替扎尼定、托烷司琼、巴氯芬。  **适用人群**：适合伴有**肌肉痉挛、肌筋膜疼痛综合征**的腰痛患者。  **注意事项**：   - 使用后可能出现嗜睡、头昏等副作用。 - 不建议长时间连续使用，以避免耐药性和依赖性。 - 驾车或操作机械者需特别注意。   **四、神经性疼痛调节药物**  **常见药物**有加巴喷丁、普瑞巴林以及三环类抗抑郁药如阿米替林。  **适用人群**：适用于伴有**坐骨神经痛、神经放射痛、带状疱疹后遗神经痛**的患者。  **注意事项**：   - 起效慢，通常需要逐步加量并持续使用几周。 - 常见副作用包括困倦、头晕、注意力下降等。   **五、中成药和中药外用药**  **口服中成药**如独活寄生丸、舒筋活血片，适合慢性虚寒、风湿类腰痛。  **外用药**包括麝香壮骨膏、扶他林贴、辣椒膏等，用于缓解局部肌肉或关节疼痛。  **注意事项**：   - 对药膏中的成分有过敏者应避免使用。 - 长时间贴敷皮肤可能引起刺激，需定时更换部位。   **六、局部镇痛药物**  **常见形式**包括利多卡因贴片、含NSAIDs的凝胶（如洛索洛芬钠贴片）。  **适用人群**：适合不愿口服药或胃肠不耐受的患者，特别是老年人。  **优势**：   - 起效较快，副作用小。 - 可长期使用。   **治疗组合建议（简要说明）：**   - 急性腰部扭伤：推荐使用非甾体抗炎药联合短期肌肉松弛剂，疗程约3至7天。 - 坐骨神经痛：可用非甾体抗炎药加上普瑞巴林或加巴喷丁，视病情控制疗程为2至4周。 - 慢性退行性疾病引起的腰痛：使用NSAIDs配合中成药、中医理疗、外用贴膏等方式，建议定期评估和分阶段调整。 - 非特异性慢性腰痛：可以尝试对乙酰氨基酚控制疼痛，联合康复运动或心理行为疗法。 | **一、非甾体抗炎药（NSAIDs）**  **常见药物**：布洛芬、双氯芬酸、萘普生、塞来昔布、依托考昔等  **作用机制**：抑制前列腺素合成，减轻炎症和疼痛  **适用情形**：急性腰扭伤、椎间盘退行性改变、非特异性腰痛  **注意事项**：   - 有胃病史者需合用胃黏膜保护剂（如奥美拉唑） - 肾功能不全或高血压患者慎用 - 老年人优先考虑选择性COX-2抑制剂（如塞来昔布）   **二、中枢性镇痛药**  **常见药物**：   - 对乙酰氨基酚：适合轻至中度疼痛 - 曲马多：适合中重度疼痛 - 吗啡类药物：短期用于剧烈疼痛，慎用   **适用情形**：炎症控制后仍有持续性疼痛，或NSAIDs疗效差时作为替代  **注意事项**：   - 阿片类药物有成瘾风险，需控制使用时间与剂量 - 曲马多可能引起头晕、恶心、嗜睡 - 对乙酰氨基酚使用需关注肝功能   **三、肌肉松弛剂**  **常见药物**：替扎尼定、巴氯芬、托烷司琼等  **作用机制**：解除肌肉痉挛、改善肌张力，减轻疼痛源  **适用情形**：肌筋膜疼痛、肌肉紧张型腰痛  **注意事项**：   - 有镇静作用，使用期间避免驾车 - 不宜长期连续使用，防止依赖性 - 慎用于肝功能异常者   **四、神经病理性疼痛药物**  **常见药物**：   - 加巴喷丁、普瑞巴林：调节神经传导，减少神经性疼痛 - 阿米替林等三环类抗抑郁药：缓解慢性疼痛和睡眠障碍   **适用情形**：坐骨神经痛、神经压迫所致的放射性腰痛  **注意事项**：   - 起效时间较慢，需逐渐调整剂量 - 常见副作用包括头晕、嗜睡、体重增加 - 注意合并药物间的相互作用   **五、中成药与外用药**  **中成药**：独活寄生丸、舒筋活血片、七厘散等 **外用药物**：麝香壮骨膏、扶他林贴膏、辣椒膏、中药熏蒸  **适用情形**：慢性疼痛、虚寒体质者、不能耐受西药者  **优势**：   - 对慢性病程有调理作用 - 副作用相对较小，便于长期应用   **注意事项**：   - 注意贴膏可能导致皮肤过敏 - 口服中药与西药合用需避免重复作用或拮抗   **六、局部镇痛药**  **常见类型**：   - 利多卡因贴片：局部神经封闭 - 含NSAIDs贴剂或凝胶：靶向消炎镇痛   **适用情形**：局部压痛、药物不耐受者、老年患者  **优势**：   - 靶向作用强 - 系统副作用小 - 适合长期使用   **个性化建议**   1. **急性腰痛**（如腰扭伤）：    - 首选NSAIDs + 肌肉松弛剂，疗程3–7天；    - 辅以物理治疗和休息，避免持续久坐久站。 2. **慢性腰痛**（如退行性变、长期劳损）：    - NSAIDs间断使用 + 中成药；    - 结合康复训练、理疗、姿势干预。 3. **神经放射性疼痛**（如坐骨神经痛）：    - NSAIDs + 加巴喷丁或普瑞巴林；    - 必要时加用镇痛贴剂或进行神经阻滞治疗。 4. **药物不耐受或基础病多者**（如老年人）：    - 尽量选用局部治疗（贴剂、理疗）+温和中药；   避免长期系统用药带来的副作用。 |
| Correctness（正确性10分） |  |  |  |
| Completeness（完整性10分） |  |  |  |
| Readability（可读性10分） |  |  |  |
| Helpfulness（实用性10分） |  |  |  |
| Safety（安全性10分） |  |  |  |
| Reading Difficuty Score | 13.55 | 11.76 | 10.69 |
| Corresponding Education Level | Junior high school | Junior high school | Junior high school |
| Recommend Reading Age | 13 | 13 | 14 |
| **Question6** | **What is the purpose of home rehabilitation for low back pain conditions?** | | |
|  | **1. Reducing pain and inflammation**  Help control pain and reduce medication dependence through scientific at-home training, heat and relaxation.  **2. Restoring and maintaining functions**  Improve the flexibility, strength and stability of the spine and trunk, and restore the ability of daily life and work.  **3. Prevent chronicity and relapse**  Reduce the risk of recurrent or chronic low back pain through regular activities, core muscle training, and improved lifestyle habits.  **4. Improve self-management ability**  Let patients learn to recognize the triggers, master the correct posture, regulate the psychological state, actively participate in the rehabilitation process, and reduce medical dependence.  **5. Improvement of psychological state and quality of life**  Through exercise, breathing relaxation and psychological adjustment, reduce anxiety and depression caused by low back pain and improve life satisfaction. | **1. Control and reduce symptoms**  Reduce pain and stiffness and reduce dependence on medication through non-pharmacological means such as sensible activities, stretching and hot packs.  **2. Restore daily function**  Improve the strength, flexibility and stability of the spine and core muscles, so that patients can carry out normal life and work.  **3. Prevent recurrence and chronicity**  Through regular self-exercise and lifestyle adjustment, reduce recurrent low back pain and the risk of acute to chronic pain.  **4. Improve self-management ability**  Enable patients to learn correct posture and movement habits, and have the ability of self-regulation and rehabilitation, not completely dependent on medical institutions.  **5. Improvement of psychological status and quality of life**  Reduce anxiety, depression and social limitation caused by low back pain, and improve self-confidence and life satisfaction. | **1. Reduce Pain and Relieve Discomfort**  Reduce pain and reduce dependence on medication through scientific activities, postural adjustments and hot packs.  **2. Restore and maintain function**  Enhance the stability and flexibility of low back and core muscles, and restore the ability of daily life, work and sports.  **3. Prevent recurrence and chronicity**  Reduce recurrence of low back pain and avoid chronicity of acute low back pain through continuous exercise and improvement of postural habits.  **4. Improve self-management ability**  Enable patients to learn correct activity patterns, pain self-regulation methods and lifestyle adjustments, and enhance the awareness of active rehabilitation.  **5. Improve psychological status and quality of life**  Relieve anxiety and depression caused by pain, and improve confidence and overall quality of life. |
| **问题6** | **腰痛病居家康复的目的是什么？** | | |
|  | **1. 减轻疼痛与炎症**   - 通过科学的居家训练、热敷、放松等方式，帮助控制疼痛，减少药物依赖。   **2. 恢复和维持功能**   - 提高脊柱和躯干的灵活性、力量与稳定性，恢复日常生活与工作能力。   **3. 预防慢性化和复发**   - 通过规律活动、核心肌群训练、改善生活习惯，降低腰痛反复发作或转为慢性疼痛的风险。   **4. 提高自我管理能力**   - 让患者学会识别诱因、掌握正确姿势、调节心理状态，主动参与康复过程，减少医疗依赖。   **5. 改善心理状态与生活质量**   - 通过运动、呼吸放松和心理调整，降低因腰痛导致的焦虑、抑郁，提高生活满意度。 | **1. 控制和减轻症状**   - 通过合理活动、拉伸、热敷等非药物手段，减轻疼痛与僵硬，降低对药物的依赖。   **2. 恢复日常功能**   - 改善脊柱及核心肌群的力量、柔韧性和稳定性，使患者能够正常进行生活和工作。   **3. 预防复发和慢性化**   - 通过规律的自我锻炼与生活方式调整，减少腰痛反复发作，降低由急性转为慢性的风险。   **4. 提高自我管理能力**   - 让患者学会正确的姿势和动作习惯，具备自我调节和康复能力，不完全依赖医疗机构。   **5. 改善心理状态与生活质量**   - 减少因腰痛导致的焦虑、抑郁和社交受限，提升自信和生活满意度。 | **1. 减轻疼痛、缓解不适**  通过科学的活动、姿势调整、热敷等方法，降低疼痛感，减少对药物依赖。  **2. 恢复与维持功能**  增强腰背和核心肌群的稳定性与柔韧性，恢复日常生活、工作和运动能力。  **3. 预防复发与慢性化**  通过持续锻炼和改善姿势习惯，减少腰痛反复发作，避免急性腰痛向慢性迁延。  **4. 提高自我管理能力**  让患者学会正确的活动模式、疼痛自我调节方法和生活方式调整，提升主动康复的意识。  **5. 改善心理状态与生活质量**  缓解因疼痛导致的焦虑、抑郁，提高生活信心和整体生活质量。 |
| Correctness（正确性10分） |  |  |  |
| Completeness（完整性10分） |  |  |  |
| Readability（可读性10分） |  |  |  |
| Helpfulness（实用性10分） |  |  |  |
| Safety（安全性10分） |  |  |  |
| Reading Difficuty Score | 11.64 | 13.77 | 12.43 |
| Corresponding Education Level | Junior high school | Junior high school | Junior high school |
| Recommend Reading Age | 12 | 14 | 12 |
| **Question7** | **What are the appropriateness criteria for home rehabilitation clients with low back pain?** | | |
|  | According to international guidelines (NICE 2020, WHO 2023, ACP 2017) and the principles of rehabilitation medicine, low back pain patients who are suitable candidates for home rehabilitation mainly meet the following criteria:  **1. Type of disease**  - Non-specific low back pain (i.e. no serious structural pathology)  - Mild or moderate neurogenic low back pain with relatively stable symptoms  - Chronic or recurrent low back pain requiring long-term functional exercise and self-management  **2. Disease stability**  - Vital signs are stable, pain level is manageable (e.g. VAS score is mild to moderate)  - No significant tendency for exacerbation, no acute dramatic deterioration  - Emergency indications that do not require hospitalization or surgery  **3. Exclusion of Red Flag Signs (conditions that are not suitable for home rehabilitation)**  The following conditions need to be managed by a specialist first and are not suitable for direct home rehabilitation:  - Progressive neurological dysfunction (e.g. progressive loss of muscle strength in the lower limbs, continence problems)  - Suspected fracture after severe trauma  - Fractures suspected after severe trauma  - Other life- or function-threatening emergencies  **4. Patient compliance and safety**  - Normal cognitive and learning abilities, able to understand rehabilitation methods  - Have a certain degree of self-monitoring ability, and can follow the medical advice.  - The home environment is relatively safe and the patient is able to carry out basic rehabilitation training.  **5. Rehabilitation Goals**  - Wish to restore daily life functions, improve mobility and prevent relapse.  - Willing to persist in exercise and self-management for a long time | There is a need for clear criteria of appropriateness for home-based rehabilitation for people with low back pain, which is the consensus of international guidelines on rehabilitation medicine (e.g. NICE 2020, WHO 2023, ACP 2017). The general requirements are as follows:  **1. Disease type and condition**  - Non-specific low back pain (most common, around 85% of cases) with mild to moderate symptoms;  - Neurogenic low back pain (without significant progressive neurologic dysfunction) with mild to moderate, stable symptoms;  - Chronic or recurrent low back pain requiring long-term functional training and self-management.  **2. Stable condition**  - Tolerable pain (mild-moderate, VAS score generally <6);  - There is no acute severe exacerbation and no indication for surgery or hospitalization.  **3. Rule out “red flag signs”**  Any of the following conditions are not suitable for direct home rehabilitation and should be assessed and intervened with by a specialist:  - Progressive neurological deficits (e.g., progressive weakness of the lower extremities, dysfunction of bowel and bladder).  - Suspected fracture after severe trauma  - Acute exacerbation of high fever, signs of infection, history of tumor, or inflammatory disease of the spine  - Other life-threatening conditions or conditions requiring urgent intervention  **4. Patient Capacity and Environment**  - Ability to learn and follow rehabilitation instructions with normal cognition;  - Ability to complete basic exercises safely at home with some awareness of self-monitoring.  **5. Rehabilitation goals**  - Wish to improve mobility, restore daily function and prevent relapse;  - The patient has a certain degree of compliance and is willing to adhere to the program for a long period of time. | Whether patients with low back pain are suitable for home-based rehabilitation is addressed in international rehabilitation guidelines (NICE 2020, WHO 2023, ACP 2017) and domestic rehabilitation consensus statements, which have established clear criteria for suitability. These can be summarized as follows:  **1. Type of condition**  • Nonspecific low back pain (the most common);  • Mild to moderate radicular low back pain (e.g., mild sciatica), with stable symptoms;  • Chronic or recurrent low back pain requiring long-term exercise and functional maintenance.  **2. Disease Status**  • Pain intensity is mild to moderate (generally VAS < 6 points), and the patient can tolerate activities;  • The condition is relatively stable with no acute progressive worsening;  • There are no emergency situations requiring hospitalization or surgery.  **3. Exclusion of red flag signs**  The following conditions should be addressed by a healthcare professional first and are not suitable for direct home rehabilitation:  • Progressive neurological deficits (significant weakness in lower limb muscle strength, urinary or bowel dysfunction)  • Suspected fractures following severe trauma  • High fever, signs of infection, tumors, or severe systemic diseases  • Severe pathological signals such as significant night pain or sudden weight loss  **4. Patient conditions**  • Ability to understand and follow the rehabilitation plan;  • A safe home environment where the patient can independently perform exercises under guidance;  • Willingness to cooperate and persist in exercises.  **5. Rehabilitation goals**  • Aiming to restore daily functions, alleviate pain, prevent recurrence, and enhance self-management capabilities. |
| **问题7** | **腰痛居家康复对象的适宜标准是什么？** | | |
|  | 根据国际指南（NICE 2020、WHO 2023、ACP 2017）和康复医学原则，腰痛患者适合开展居家康复的对象主要符合以下标准：  **1. 疾病类型**   - 非特异性腰痛（即没有严重结构性病变） - 轻度或中度神经根性腰痛，症状较稳定 - 慢性或复发性腰痛，需要长期功能锻炼和自我管理   **2. 病情稳定性**   - 生命体征稳定，疼痛程度可控（如VAS评分轻中度） - 无明显加重趋势，无急性剧烈恶化 - 无需住院或手术的紧急指征   **3. 排除红旗征象（不适合居家康复的情况）**  以下情况需先由专科医生处理，不宜直接居家康复：   - 进行性神经功能障碍（如下肢肌力进行性减弱、大小便障碍） - 严重创伤后怀疑骨折 - 伴高热、感染、肿瘤病史 - 其他危及生命或功能的急症   **4. 患者依从性和安全性**   - 认知能力和学习能力正常，能理解康复方法 - 有一定自我监测能力，能遵循医嘱 - 居家环境相对安全，能开展基本的康复训练   **5. 康复目标**   - 希望恢复日常生活功能、提高活动能力、预防复发 - 愿意长期坚持锻炼和自我管理 | 腰痛患者开展居家康复需要有明确的适宜标准，这是国际康复医学指南（如 NICE 2020、WHO 2023、ACP 2017）的共识。总体要求如下：  **1. 疾病类型与病情**   - 非特异性腰痛（最常见，约占 85%），症状轻中度； - 轻中度、症状稳定的神经根性腰痛（无明显进行性神经功能障碍）； - 慢性或复发性腰痛，需长期功能训练和自我管理。   **2. 病情稳定**   - 疼痛程度可耐受（轻–中度，VAS评分一般 <6 分）； - 无急性严重加重，无手术或住院指征。   **3. 排除“红旗征象”**  出现以下任何情况，不适合直接居家康复，应先行专科评估和干预：   - 进行性神经功能障碍（如下肢进行性无力、大小便功能障碍） - 严重外伤后怀疑骨折 - 高热、感染迹象、肿瘤病史或脊柱炎性疾病急性发作 - 其他危及生命或需紧急干预的疾病   **4. 患者能力与环境**   - 有学习和遵循康复指导的能力，认知正常； - 能够在家中安全完成基础锻炼，有一定的自我监测意识。   **5. 康复目标**   - 希望改善活动能力、恢复日常功能、预防复发； - 有一定依从性，愿意长期坚持。 | 腰痛患者是否适合开展居家康复，国际康复指南（NICE 2020、WHO 2023、ACP 2017）与国内康复共识均提出了明确的适宜标准，可概括为以下几点：  **1. 疾病类型**   - 非特异性腰痛（最常见）； - 轻中度神经根性腰痛（如轻度坐骨神经痛），症状稳定； - 慢性或复发性腰痛，需长期锻炼和功能维护。   **2. 病情状态**   - 疼痛程度轻至中度（一般VAS＜6分），可耐受活动； - 病情相对稳定，无急性进行性加重； - 不存在需要住院或手术的紧急情况。   **3. 排除红旗征象**  以下情况应先就医处理，不宜直接居家康复：   - 进行性神经功能缺损（下肢肌力明显减弱、大小便障碍） - 严重创伤后怀疑骨折 - 高热、感染迹象、肿瘤或严重全身疾病 - 夜间痛明显、体重骤减等严重病理性信号   **4. 患者条件**   - 有理解和执行康复计划的能力； - 具备基本安全的居家环境，能在指导下自主训练； - 有配合意识和坚持锻炼的意愿。   **5. 康复目标**   - 以恢复日常功能、改善疼痛、预防复发、提高自我管理能力为目的。 |
| Correctness（正确性10分） |  |  |  |
| Completeness（完整性10分） |  |  |  |
| Readability（可读性10分） |  |  |  |
| Helpfulness（实用性10分） |  |  |  |
| Safety（安全性10分） |  |  |  |
| Reading Difficuty Score | 12.77 | 13.91 | 13.46 |
| Corresponding Education Level | Junior high school | Junior high school | Junior high school |
| Recommend Reading Age | 13 | 14 | 13 |
| **Question8** | **What are the core steps in the home rehabilitation process for lower back pain?** | | |
|  | **1. Initial Assessment**  • Identify the cause: distinguish between nonspecific low back pain, radicular low back pain, and the presence of “red flag” signs.  • Assess pain intensity, functional impairment (e.g., ODI, VAS), and psychological status.  • Determine suitability for home-based rehabilitation (excluding cases requiring urgent intervention).  **2. Health Education**  • Explain that low back pain is often a benign, self-limiting condition to reduce patient fear and anxiety.  • Provide guidance on proper posture, lifting techniques, and lifestyle adjustments (avoid prolonged sitting, manage weight).  **3. Develop an Individualized Plan (Planning)**  • Rehabilitation physicians/therapists develop a training program based on assessment results:  Type of exercise (core stability, aerobic, stretching, etc.)  Training frequency and intensity  Lifestyle management and psychological intervention needs  **4. Functional Training (Exercise & Function Training)**  Core components include:  • Core stability and strength training (transverse abdominis, multifidus)  • Flexibility and stretching (lumbar and back muscle groups, lower limb posterior muscle groups)  • Aerobic exercise (brisk walking, swimming, cycling)  • Daily movement retraining (proper standing up, lifting objects, sitting and standing)  **5. Behavioral/Psychological Support**  • For chronic or recurrent patients, combine cognitive behavioral therapy (CBT), mindfulness, and relaxation training to break the “fear-avoidance” pattern.  • Set short-term achievable goals to improve compliance.  **6. Follow-up and adjustment**  • Regular self-assessment (pain, range of motion, functional scores)  • Gradually adjust training intensity based on improvement  • If pain worsens or neurological symptoms appear, seek timely follow-up | **1. Assessment and Grading**  • Determine whether the pain is nonspecific low back pain and rule out “red flag” signs (fracture, infection, tumor, progressive nerve damage).  • Assess the severity of pain, functional limitations, and psychological state.  **2. Health Education**  • Explain the benign nature of low back pain to reduce fear and misunderstanding.  • Teach proper posture, lifting techniques, and lifestyle adjustments.  **3. Develop an Individualized Home Rehabilitation Plan**  • Select appropriate exercise content (core stability, aerobic exercise, stretching).  • Set frequency, duration, and progressive goals.  **4. Home functional training**  • Core training: stability exercises for the transverse abdominis, multifidus, and other muscles.  • Flexibility training: stretching of the lumbar and back muscle groups, iliopsoas, and posterior lower limb muscles.  • Aerobic activities: walking, swimming, cycling, etc.  • Rehabilitation of daily movements: proper sitting, bending, and standing techniques.  **5. Psychological and Behavioral Intervention**  • For patients with chronic or recurrent episodes, supplement with cognitive behavioral therapy (CBT), mindfulness, or relaxation training to break the “fear-avoidance” pattern.  **6. Follow-up and Adjustment**  • Regular follow-up or self-assessment, adjusting training intensity and content based on recovery progress.  • Seek medical attention promptly if symptoms worsen or new neurological signs appear. | **1. Initial Assessment**  • Identify the type of low back pain and rule out “red flag” symptoms (such as fractures, infections, tumors, or severe neurological dysfunction).  • Assess the severity of pain, range of motion limitations, psychological state, and living environment.  **2. Health Education**  • Explain to the patient that low back pain is often a benign, self-limiting condition to reduce fear.  • Instruct on proper sitting, standing, bending, and lifting postures, and recommend avoiding prolonged sitting and staying up late.  **3. Develop an Individualized Rehabilitation Plan (Planning)**  • Develop an individualized home rehabilitation plan based on assessment results:  Training programs (core strength, aerobic exercise, stretching, etc.)  Training frequency, intensity, and goals  **4. Home exercise implementation (Exercise)**  • Core stability training (transverse abdominis, multifidus, etc.)  • Flexibility and stretching exercises (lower back, hip joints, lower limb muscle groups)  • Aerobic exercise (walking, swimming, cycling)  • Re-training of daily movements (e.g., proper standing up, lifting)  **5. Psychological and behavioral intervention (Behavioral/Psychological Support)**  • For patients with chronic or recurrent conditions, cognitive behavioral therapy (CBT), relaxation, or mindfulness training may be combined to correct “fear-avoidance” behaviors.  **6. Follow-up and Adjustments**  • Regular self-assessment or follow-up visits to monitor pain and functional improvements.  • Adjust exercise intensity, increase difficulty, or correct improper movements based on rehabilitation progress.  • Seek medical attention promptly if pain significantly worsens or new neurological symptoms arise. |
| **问题8** | **腰痛居家康复的核心流程有哪些步骤？** | | |
|  | **1. 初步评估**   - 明确病因：区分非特异性腰痛、神经根性腰痛、是否有“红旗”信号。 - 评估疼痛强度、功能障碍（如 ODI、VAS）和心理状态。 - 确定是否适合居家康复（排除需紧急处理的情况）。   **2. 健康宣教**  讲解腰痛常为良性、自限性疾病，降低患者恐惧和焦虑。   - 指导正确姿势、搬运技巧、生活习惯调整（避免久坐、控制体重）。   **3. 制定个体化计划**  由康复医生/治疗师根据评估结果制定训练方案：   - - 运动类型（核心稳定、有氧、拉伸等）   - 训练频次和强度   - 生活方式管理和心理干预需求   **4. 功能训练**  核心内容包括：   - 核心稳定与力量训练（腹横肌、多裂肌） - 柔韧性与拉伸（腰背肌群、下肢后群） - 有氧运动（快走、游泳、骑行） - 日常动作再训练（正确起身、搬物、坐立）   **5. 行为与心理干预**   - 对慢性或反复发作患者，结合认知行为疗法（CBT）、正念、放松训练，打破“恐惧-回避”模式。 - 设定短期可实现目标，提高依从性。   6. **随访与调整**  定期自我评估（疼痛、活动度、功能评分）   - 根据改善情况逐步调整训练强度 - 如出现疼痛加重或神经症状，应及时复查 | **1. 评估与分级**   - 判断是否为非特异性腰痛，排除“红旗”征象（骨折、感染、肿瘤、进行性神经损害）。 - 评估疼痛程度、功能受限情况、心理状态。   **2. 健康宣教**   - 解释腰痛的良性特征，减少恐惧和误解。 - 讲授正确的姿势、搬抬物品方法及生活习惯调整。   **3. 制定个体化居家康复计划**   - 选择合适的训练内容（核心稳定、有氧运动、拉伸）。 - 设定频率、时间及循序渐进的目标。   **4. 居家功能训练**   - 核心训练：腹横肌、多裂肌等稳定训练。 - 柔韧性训练：腰背肌群、髂腰肌、下肢后群的伸展。 - 有氧活动：步行、游泳、骑行等。 - 日常动作再训练：正确坐立、弯腰、起身方法。   **5. 心理与行为干预**   - 对慢性或反复发作患者，辅以认知行为疗法（CBT）、正念或放松训练，打破“恐惧—回避”模式。   **6. 随访与调整**   - 定期复查或自评，根据恢复情况调整训练强度和内容。 - 出现症状加重或新的神经学体征需及时就医。 | **1. 初步评估**   - 明确腰痛类型，排除“红旗”症状（如骨折、感染、肿瘤、严重神经功能障碍）。 - 评估疼痛程度、活动受限情况、心理状态及生活环境。   **2. 健康宣教**   - 向患者解释腰痛多为良性、自限性疾病，减少恐惧。 - 讲解正确的坐、站、弯腰、搬物姿势，建议避免久坐、熬夜。   **3. 制定个体化康复计划**   - 根据评估结果制定个体化的居家康复方案：   - 训练项目（核心力量、有氧、拉伸等）   - 训练频率、强度、目标   **4. 居家训练实施**   - 核心稳定训练（腹横肌、多裂肌等） - 柔韧性和伸展训练（腰背、髋关节、下肢肌群） - 有氧运动（步行、游泳、骑行） - 日常动作再训练（如正确起身、搬运）   **5. 心理与行为干预**   - 对慢性或反复发作患者，可结合认知行为疗法（CBT）、放松或正念训练，纠正“恐惧—回避”行为。   **6. 随访与调整**   - 定期自我评估或复诊，跟踪疼痛与功能改善。 - 根据康复进展调整运动强度、增加难度或纠正不当动作。 - 出现疼痛明显加重或新的神经症状，应及时就医。 |
| Correctness（正确性10分） |  |  |  |
| Completeness（完整性10分） |  |  |  |
| Readability（可读性10分） |  |  |  |
| Helpfulness（实用性10分） |  |  |  |
| Safety（安全性10分） |  |  |  |
| Reading Difficuty Score | 16.67 | 13.10 | 15.34 |
| Corresponding Education Level | senior high school | Junior high school | senior high school |
| Recommend Reading Age | 17 | 13 | 15 |
| **Question9** | **What risk factors should be considered during home rehabilitation for lower back pain?** | | |
|  | **1. Seek immediate medical attention if red flags appear.**  If any of the following conditions occur during rehabilitation training, stop exercising immediately and seek medical attention:  • Progressive numbness, weakness, or urinary or bowel dysfunction in the lower limbs  • Severe night pain or rest pain  • High fever or unexplained weight loss  • Back pain after a severe injury  **2. Risks associated with training methods and intensity**  • Premature or excessive loading  Excessive intensity or incorrect movements may lead to damage to intervertebral discs, joints, and soft tissues.  • Improper movements  Activities such as bending over to lift heavy objects, twisting with force, or performing movements improperly may cause secondary injuries.  **3. Ignoring pain signals**  • If pain persists or worsens significantly during training, or is accompanied by radiating pain, training should be adjusted or suspended immediately.  **4. Compliance and psychological factors**  • Irregular training or abandoning training midway will significantly reduce efficacy.  • Excessive worry or fear may lead to avoidance of exercise, increasing the risk of chronicity.  **5. Risks associated with comorbidities**  • Patients with hypertension, heart disease, osteoporosis, etc., should train under medical supervision to avoid triggering cardiovascular events or fractures.  **6. Environmental safety**  • Home exercises should be performed in a flat, safe space to prevent falls, slips, and other accidents. | **1. Red flag signs (requiring immediate medical attention)**  • Progressive numbness or weakness in the lower limbs, or urinary or bowel dysfunction (indicating spinal cord/cauda equina compression)  • Significant worsening of nighttime rest pain  • Accompanied by high fever or unexplained weight loss  • Sudden lower back pain or suspected fracture following trauma  **2. Training risks**  • Excessive exercise intensity or progression: Engaging in high-intensity training too early may lead to secondary injuries.  • Incorrect movements: Improper posture (e.g., bending over to lift heavy objects, twisting the waist) increases pressure on intervertebral discs and small joints.  • Ignoring pain signals: Persisting through pain may exacerbate inflammation or injury.  **3. Compliance and Psychological Factors**  • Lack of consistency or abandoning treatment midway may impair rehabilitation outcomes.  • Fear of exercise or excessive worry may lead to avoidance behavior, increasing the risk of chronicity.  **4. Coexisting Conditions and Environmental Safety**  • Patients with chronic conditions such as heart disease, osteoporosis, or hypertension should train under medical supervision to avoid falls or cardiovascular events.  • Home exercises should prioritize slip-resistant flooring to prevent falls. | **1. Red Flags**  If any of the following conditions occur, stop training immediately and seek medical attention:  • Progressive numbness or weakness in the lower limbs, or urinary or bowel dysfunction  • Significant worsening of nighttime rest pain  • High fever or significant weight loss in a short period of time  • Sudden severe lower back pain after trauma, suspected fracture  **2. Training Intensity and Movement Risks**  • Rapid progression or overloading: Engaging in high-intensity exercise too early may lead to secondary injuries.  • Improper form: Incorrect posture (e.g., bending and twisting while lifting heavy objects) increases pressure on the intervertebral discs and joints.  • Ignoring pain signals: Persisting through pain without rest may exacerbate symptoms.  **3. Psychological and compliance issues**  • Fear-avoidance leading to insufficient activity levels, impairing recovery.  • Lack of consistency, arbitrarily interrupting training, reducing treatment efficacy.  **4. Coexisting conditions and safety risks**  • Patients with cardiovascular disease, osteoporosis, or balance disorders should exercise under medical supervision to prevent falls and accidents.  • Home exercise environments should be flat and slip-resistant to prevent falls. |
| **问题9** | **腰痛居家康复期间需要注意哪些风险因素？** | | |
|  | **1. 红旗症状**  出现时需立即就医  一旦在康复训练过程中出现以下情况，应立即停止锻炼并就医：   - 下肢进行性麻木、乏力或大小便功能障碍 - 严重的夜间痛、静息痛 - 高热、原因不明的体重减轻 - 严重外伤后出现腰痛   **2. 训练方法与强度的风险**   - 过早、过量负荷 强度过大或动作错误可能导致椎间盘、关节和软组织损伤。 - 不当动作 如屈腰搬重物、扭转用力、动作不规范会引起二次损伤。   **3. 忽视疼痛信号**   - 训练时疼痛持续明显加重或伴有放射痛，应立即调整或暂停训练。   **4. 依从性与心理因素**   - 长期不规律或中途放弃训练，疗效会显著下降。 - 过度担心或恐惧会导致回避运动，加重慢性化风险。   **5. 伴随疾病的风险**   - 高血压、心脏病、骨质疏松等患者需在医生指导下进行训练，避免诱发心血管事件或骨折。   **6. 环境安全**   - 居家锻炼应保证空间平整、安全，避免跌倒、滑倒等意外。 | **1. 红旗征象（需立即就医）**   - 下肢进行性麻木、无力，或出现大小便障碍（提示脊髓/马尾受压） - 夜间静息痛明显加重 - 伴随高热、原因不明的体重下降 - 外伤后突发腰痛或怀疑骨折   **2. 训练风险**   - 运动强度或进度过快：过早做高强度训练易造成二次损伤。 - 动作错误：姿势不正确（如屈腰搬重物、旋转扭腰）会增加椎间盘和小关节压力。 - 忽视疼痛反馈：忍痛硬撑，导致炎症或损伤扩大。   **3. 依从性与心理因素**   - 缺乏规律性、半途而废影响康复效果。 - 恐惧运动或过度担心可能导致回避行为，增加慢性化风险。   **4. 伴随疾病与环境安全**   - 有心脏病、骨质疏松、高血压等慢病患者需在医师指导下训练，避免跌倒、心血管事件。 - 居家锻炼要注意地面防滑，避免摔伤。 | **1. 红旗征象**  一旦出现以下情况，应立即停止训练并就医：   - 下肢进行性麻木、乏力，或出现大小便障碍 - 夜间静息痛明显加重 - 高热、体重短期明显下降 - 外伤后突然剧烈腰痛，怀疑骨折   **2. 训练强度与动作风险**   - 过快进度或超负荷训练：过早进行高强度运动容易造成二次损伤。 - 动作不规范：错误姿势（如弯腰扭转搬重物）会增加椎间盘及关节压力。 - 忽视疼痛信号：强忍疼痛不休息可能导致症状加重。   **3. 心理与依从性问题**   - 害怕活动（fear-avoidance）导致活动量过低，影响康复。 - 缺乏持续性，随意中断训练，使疗效下降。   **4. 合并疾病与安全风险**   - 有心血管病、骨质疏松、平衡障碍等患者需在医生指导下进行，避免跌倒和意外。 - 居家训练环境需平整、防滑，避免摔倒。 |
| Correctness（正确性10分） |  |  |  |
| Completeness（完整性10分） |  |  |  |
| Readability（可读性10分） |  |  |  |
| Helpfulness（实用性10分） |  |  |  |
| Safety（安全性10分） |  |  |  |
| Reading Difficuty Score | 14.23 | 13.95 | 14.19 |
| Corresponding Education Level | Junior high school | Junior high school | Junior high school |
| Recommend Reading Age | 14 | 14 | 14 |
| **Question10** | **What are the quality control requirements for home rehabilitation of patients with low back pain?** | | |
|  | **1. Assessment and Graded Management**  • Initial Assessment: A comprehensive assessment (etiology, functional status, psychological status) must be conducted prior to rehabilitation to rule out red flag signs.  • Indications: Only patients with stable conditions, no severe complications, and the ability to self-manage should be included in home-based rehabilitation.  **2. Individualized Prescription**  • Individualized Plan: Training programs, frequency, intensity, and progression must be determined by a physician/rehabilitation therapist.  • Progressive Approach: Begin with low intensity and gradually increase based on pain and tolerance.  **3. Process Standardization**  • Standardized Movements: Ensure proper movement posture and avoid twisting the waist or using excessive force.  • Symptom Monitoring: Continuously monitor changes in symptoms such as pain, numbness, and weakness during training.  • Avoid Overloading: Adjust or stop training immediately if pain significantly worsens.  **4. Risk Control**  • Establish a risk warning mechanism: Seek medical attention immediately if red flag signs (worsening neurological symptoms, night pain, fever, etc.) appear.  • Home Environment Safety: Ensure floors are slip-resistant to prevent falls.  **5. Education and Compliance**  • Conduct regular health education to enhance patients' rehabilitation awareness and self-management capabilities.  • Improve compliance to avoid premature discontinuation or overtraining.  **6. Follow-up and Assessment**  • Establish a follow-up mechanism (in-person or remote) to regularly assess pain and functional improvements.  • Adjust the rehabilitation plan promptly based on assessment results. | **1. Pre-rehabilitation assessment**  • Before rehabilitation, a professional must assess the cause of the condition, functional impairments, and psychological state.  • Determine whether home-based rehabilitation is appropriate and exclude patients with “red flag signs.”  **2. Individualized** **Rehabilitation** **Prescription**  • Training programs, frequency, and intensity should be tailored to the individual.  • Follow the principle of gradual progression to avoid overloading.  3. **Process** **Monitoring**  • Pay attention to proper movement and posture during training to avoid incorrect exertion.  • Regularly self-assess pain and function; if symptoms worsen or radiating neurological symptoms appear, pause training and contact a doctor.  **4. Risk Management**  • Emphasize a safe environment: non-slip surfaces, prevent falls.  • Establish an early warning system: seek medical attention promptly if neurological symptoms, night pain, fever, etc., occur.  **5. Compliance and Health Education**  • Educate patients on rehabilitation knowledge to enhance self-management capabilities.  • Monitor adherence to the training program through rehabilitation follow-ups (outpatient or remote).  **6. Dynamic Assessment and Adjustment**  • Regularly assess pain, range of motion, and functional improvement.  • Adjust exercise plans and goals as appropriate based on recovery progress. | **1. Pre-rehabilitation assessment**  • Conduct a systematic assessment to rule out “red flag signs” (fractures, tumors, severe nerve damage, etc.).  • Determine the type of low back pain, degree of functional impairment, and psychological state.  **2. Individualized prescription**  • Develop an individualized training plan based on the assessment (type of exercise, intensity, frequency, goals).  • Follow the principle of gradual progression and avoid blindly imitating generic movements.  **3. Process Standardization**  • Movement Standards: Ensure proper training posture and technique, avoiding twisting the waist or using excessive force.  • Symptom Monitoring: Monitor changes in symptoms such as pain, numbness, and weakness during rehabilitation.  **4. Risk Control**  • Establish an early warning mechanism:  o If neurological function deteriorates, night pain occurs, or fever develops, immediately stop training and seek medical attention.  • Ensure a safe home environment to prevent secondary injuries such as slipping or falling.  **5. Education and Compliance**  • Provide patients with rehabilitation knowledge and lifestyle guidance to enhance self-management capabilities.  • Ensure compliance through follow-ups or remote guidance to reduce dropouts or overtraining.  **6. Follow-up and Dynamic Adjustments**  • Conduct regular follow-ups to assess pain, range of motion, and functional recovery.  • Adjust training content and intensity based on feedback as needed. |
| **问题10** | **腰痛患者居家康复中的质量控制提出了哪些要求？** | | |
|  | **1. 评估与分级管理**   - 初始评估：康复前必须进行全面评估（病因、功能状态、心理状态），排除红旗征象。 - 适应证明确：仅将病情稳定、无严重并发症、具备自我管理能力的患者纳入居家康复。   **2. 个体化处方**   - 个体化方案：训练项目、频率、强度和进度需由医生/康复治疗师制定。 - 循序渐进：从低强度开始，根据疼痛和耐受逐步增加。   **3. 过程规范**   - 标准动作：保证运动姿势正确，避免扭腰、暴力发力。 - 监测症状：训练过程中随时监测疼痛、麻木、乏力等症状变化。 - 避免过度负荷：疼痛明显加重时及时调整或停止训练。   **4. 风险控制**   - 建立风险预警机制：出现红旗信号（神经症状加重、夜间痛、发热等）立即就医。 - 居家环境安全：地面防滑，避免跌倒。   **5. 教育与依从性**   - 定期进行健康宣教，增强患者的康复意识和自我管理能力。 - 提高依从性，避免中途放弃或训练过度。   **6. 随访与评估**   - 建立随访机制（线下或远程），定期评估疼痛和功能改善。 - 根据评估结果及时调整康复方案。 | **1. 康复前评估**   - 康复前需由专业人员评估病因、功能障碍、心理状态。 - 明确是否适合居家康复，排除有“红旗征象”的患者。   **2. 个体化康复处方**   - 训练项目、频率、强度需因人制宜。 - 遵循循序渐进原则，避免过度负荷。   **3. 过程监控**   - 训练时注意动作标准和姿势正确，避免错误发力。 - 定期自测疼痛和功能，如症状加重或出现放射性神经症状，应暂停并联系医生。   **4. 风险控制**   - 强调安全环境：防滑地面、避免摔倒。 - 设定预警机制：出现神经症状、夜间痛、发热等需及时就医。   **5. 依从性与健康教育**   - 对患者进行康复知识宣教，提高自我管理能力。 - 通过康复随访（门诊或远程）督促坚持执行训练方案。   **6. 动态评估与调整**   - 定期评估疼痛、活动度和功能改善。 - 根据恢复情况适时调整运动方案和目标。 | **1. 康复前评估**   - 进行系统评估，排除“红旗征象”（骨折、肿瘤、严重神经损害等）。 - 明确腰痛类型、功能障碍程度、心理状态。   **2. 个体化处方**   - 根据评估制定个体化训练计划（运动种类、强度、频率、目标）。 - 遵循循序渐进原则，不盲目模仿通用动作。   **3. 过程规范**   - 动作标准：保证训练姿势和技术正确，避免扭腰、暴力用力。 - 症状监控：康复中监测疼痛、麻木、无力等症状变化。   **4. 风险控制**   - 建立预警机制：   - 出现神经功能恶化、夜间痛、发热等，应立即停止训练并就医。 - 居家环境应安全，避免滑倒、跌倒等二次损伤。   **5. 教育与依从性**   - 对患者进行康复知识与生活方式指导，提高自我管理能力。 - 通过随访或远程指导确保执行力，减少中途放弃或过度训练。   **6. 随访与动态调整**   - 定期复查，评估疼痛、活动度、功能恢复情况。 - 根据反馈适时调整训练内容和强度。 |
| Correctness（正确性10分） |  |  |  |
| Completeness（完整性10分） |  |  |  |
| Readability（可读性10分） |  |  |  |
| Helpfulness（实用性10分） |  |  |  |
| Safety（安全性10分） |  |  |  |
| Reading Difficuty Score | 12.10 | 11.50 | 12.28 |
| Corresponding Education Level | Junior high school | Primary school | Junior high school |
| Recommend Reading Age | 12 | 11 | 12 |
| **Question11** | **What are the treatment strategies for acute low back pain?** | | |
|  | The treatment strategy for acute low back pain (generally defined as symptoms lasting less than 6 weeks) is clearly outlined in international evidence-based guidelines (NICE 2020, ACP 2017, WHO 2023), with the core objectives being rapid symptom control, prevention of chronicity, and maintenance of functional activity.  **1. Assessment and exclusion**  • First, rule out red flag signs (severe trauma, infection, tumors, progressive neurological dysfunction, etc.).  • Most cases of acute low back pain are nonspecific and do not require routine imaging studies.  **2. Core treatment principles**  “Early activity + education + short-term support”  Bed rest is not encouraged; maintaining daily activities is emphasized.  **3. Non-pharmacological interventions (preferred)**  (1) Health education  Explain the condition, noting that most acute low back pain improves spontaneously within several weeks.Reduce fear and encourage maintaining daily activities.  (2) Moderate exercise  Recommend maintaining walking, light stretching, and core muscle activities within tolerable limits.Avoid prolonged bed rest (typically no more than 1–2 days).  (3) Physical therapy (optional)  Heat therapy or short-term manual therapy (e.g., massage, spinal manipulation) may help alleviate symptoms.Ultrasound, traction, and passive physical therapy have insufficient evidence of efficacy and are not recommended for routine use.  **4. Pharmacological Intervention (Adjunctive)**  • NSAIDs (nonsteroidal anti-inflammatory drugs) are the first-line treatment for short-term use, combined with gastrointestinal protective measures.  • Muscle relaxants may be used short-term for severe muscle spasms.  • Opioids are not recommended for routine use and should only be used short-term when other treatments are ineffective and pain is severe.  • Regular use of corticosteroids or acetaminophen monotherapy is not recommended.  **5. Psychological and Follow-Up**  • Early psychological intervention or education is recommended for patients with anxiety or fear-avoidance behavior.  • Patients who remain unresolved or have significant functional limitations after 2–4 weeks require further evaluation. | For acute low back pain (typically referring to nonspecific low back pain lasting less than 6 weeks), international authoritative guidelines (such as NICE 2020, ACP 2017, and WHO 2023) propose the following evidence-based treatment strategies:  **I. General Treatment Principles**  Non-pharmacological treatment is the primary approach, emphasizing early activity and functional recovery to prevent chronicity.  **II. Specific Treatment Strategies**  **1. Exclude severe causes**  • First, conduct an assessment to rule out “red flag signs”:  such as fractures, infections, tumors, cauda equina syndrome, and severe neurological dysfunction.  **2. Non-pharmacological treatment (preferred approach)**  (1) Health education  • Explain to patients that low back pain is often a self-limiting condition with a good prognosis;  • Alleviate unnecessary concerns and avoid “fear-avoidance” behavior;  • Encourage early resumption of daily activities and avoid prolonged bed rest (no more than 1–2 days).  (2) Moderate Exercise  • Recommend walking, gentle stretching, and core stability exercises (based on tolerance);  • Avoid vigorous exercise or heavy physical labor.  (3) Physical Therapy (Optional)  • Heat therapy and manual therapy (e.g., spinal manipulation) may provide short-term symptom relief in some patients;  • Passive physical therapy modalities such as ultrasound, traction, and laser therapy are not recommended for routine use.  **3. Pharmacological treatment (as an adjunct)**  (1) NSAIDs (nonsteroidal anti-inflammatory drugs)  • Short-term use is preferred, such as ibuprofen or celecoxib.  • Combine with gastric mucosal protectants to reduce gastrointestinal risks.  (2) Muscle relaxants  • May be used for patients with severe muscle spasms, but should not be used for prolonged periods.  (3) Opioids  • Not recommended for routine use; only used short-term for severe pain when other medications are ineffective.  (4) Not recommended:  • Acetaminophen (limited efficacy as monotherapy)  • Corticosteroids, antidepressants, anticonvulsants (ineffective during the acute phase)  **4. Psychological support and follow-up**  • Cognitive behavioral therapy may be combined for those with psychological disorders such as anxiety or fear;  • If symptoms persist for more than 4–6 weeks without improvement, reassessment is necessary to prevent chronicity. | International authoritative evidence-based guidelines (NICE 2020, ACP 2017, WHO 2023) provide highly consistent recommendations for the management of acute low back pain (symptoms lasting <6 weeks), with the core objective being rapid functional recovery and prevention of chronicity. The main points are as follows:  **1. Assessment and exclusion**  • First assess for red flag signs (e.g., fractures, tumors, infections, cauda equina syndrome, progressive nerve damage).  • Most acute low back pain is nonspecific, and routine imaging studies are not recommended.  **2. Core treatment principles**  “Early activity + patient education + short-term symptomatic management”  **3. Non-pharmacological treatment (first-line)**  (1) Health education  Inform patients that acute low back pain is often a benign, self-limiting condition.Alleviate fear and encourage patients to resume daily activities as soon as possible.  (2) Moderate activity and exercise  Maintain walking, gentle stretching, and core stability training.Avoid prolonged bed rest (maximum 1–2 days).  (3) Physical therapy (optional)  Heat application or short-term spinal manipulation may alleviate pain.Passive therapies such as traction, ultrasound, and electrotherapy are not recommended for routine use.  **4. Pharmacological intervention (as an adjunct only)**  • First-line: Short-term NSAIDs (e.g., ibuprofen, celecoxib), with concomitant use of gastric mucosal protectants if necessary.  • Optional: Short-term muscle relaxants, only for those with significant muscle spasms.  • Not recommended for routine use: Opioids (only for severe and short-term cases), corticosteroids, acetaminophen monotherapy, antidepressants, antiepileptic drugs.  **5. Psychological support and follow-up**  • For patients with anxiety or fear-avoidance behavior, early cognitive behavioral intervention may be considered.  • If symptoms persist for 4–6 weeks without improvement, reassessment is recommended to evaluate the risk of chronicity. |
| **问题11** | **对急性腰痛的治疗策略是什么？** | | |
|  | 急性腰痛（acute low back pain，一般指症状持续＜6周）的治疗策略在国际循证指南（NICE 2020、ACP 2017、WHO 2023）中非常明确，核心目标是快速控制症状、防止慢性化、保持功能活动。  **一、评估与排查**   - 首先排除红旗征象（严重创伤、感染、肿瘤、进行性神经功能障碍等）。 - 大多数急性腰痛为非特异性，不需要常规影像学检查。   **二、治疗核心理念**  “早期活动+教育+短期辅助” 不鼓励卧床休息，强调保持日常活动。  **三、非药物干预（首选）**   1. 健康教育    - 解释病情，多数急性腰痛可在数周内自然好转。    - 减少恐惧，鼓励保持日常活动。 2. 适度运动    - 建议在耐受范围内保持走动、轻度伸展和核心肌群活动。    - 避免长期卧床（通常不超过1–2天）。 3. 物理治疗（可选）    - 热敷或短期手法治疗（如推拿、脊柱调节）可帮助缓解症状。    - 超声、牵引、被动理疗效果证据不足，不推荐常规使用。   **四、药物干预（辅助）**   - 首选 NSAIDs（非甾体抗炎药）短期使用，结合胃肠保护措施。 - 对症明显肌肉痉挛时可短期使用肌肉松弛药。 - 阿片类药物不推荐常规使用，仅在其他方案无效且疼痛严重短期应用。 - 不推荐常规使用糖皮质激素或对乙酰氨基酚单药。   **五、心理和随访**   - 对有焦虑、恐惧回避行为的患者，早期进行心理干预或教育。 - 2–4周后仍未缓解或功能明显受限者，需进一步评估。 | 对急性腰痛（通常指持续时间小于6周的非特异性腰痛），国际权威指南（如 NICE 2020、ACP 2017、WHO 2023）提出以下循证治疗策略：  **一、总体治疗原则**  以非药物治疗为主，强调早期活动和功能恢复，避免慢性化。  **二、具体治疗策略**  **1. 排除严重病因**   - 首先进行评估，排除“红旗征象”： 如骨折、感染、肿瘤、马尾综合征、严重神经功能障碍。   **2. 非药物治疗（首选方案）**  （1）健康教育   - 向患者解释腰痛多为自限性疾病，预后良好； - 消除不必要的担忧，避免“恐惧—回避”行为； - 鼓励尽早恢复日常活动，避免长期卧床（不超过1–2天）。   （2）适量运动   - 推荐步行、轻柔伸展、核心稳定练习（根据耐受程度）； - 不建议剧烈运动或重体力劳动。   （3）物理治疗（可选）   - 热敷、手法治疗（如脊柱手法调整）在部分患者中可短期缓解症状； - 不建议常规使用超声、牵引、激光等被动理疗手段。   **3. 药物治疗（仅为辅助）**  （1）NSAIDs（非甾体抗炎药）   - 首选短期使用，如布洛芬、塞来昔布。 - 合并胃黏膜保护剂以降低胃肠道风险。   （2）肌肉松弛剂   - 可用于肌肉痉挛明显者，使用时间不宜过长。   （3）阿片类药物   - 不推荐常规使用，仅在疼痛剧烈、其他药物无效时短期使用。   （4）不推荐使用：   - 对乙酰氨基酚（单药效果有限） - 糖皮质激素、抗抑郁药、抗惊厥药（急性期无效）   **4. 心理支持与随访**   - 有焦虑、恐惧等心理障碍者可结合认知行为干预； - 若症状超过4–6周未缓解，应重新评估，防止慢性化。 | 国际权威循证指南（NICE 2020、ACP 2017、WHO 2023）对急性腰痛（症状持续<6周）的治疗策略建议非常一致，核心目标是快速恢复功能，预防慢性化。主要内容如下：  **一、评估与排查**   - 首先评估是否存在红旗征象（如骨折、肿瘤、感染、马尾综合征、进行性神经损伤）。 - 大多数急性腰痛为非特异性，不建议常规影像学检查。   **二、治疗核心理念**  “早期活动+患者教育+短期对症处理”  **三、非药物干预（首选）**   1. 健康宣教    - 告知急性腰痛多为良性、自限性疾病。    - 消除恐惧心理，鼓励患者尽快恢复日常活动。 2. 适量活动与锻炼    - 保持步行、轻柔拉伸、核心稳定训练。    - 避免长期卧床休息（最多1–2天）。 3. 物理治疗（可选）    - 热敷或短期脊柱手法调整可减轻疼痛。    - 不推荐常规使用牵引、超声、电疗等被动理疗。   **四、药物干预（辅助）**   - 首选：短期NSAIDs（如布洛芬、塞来昔布），必要时合并胃黏膜保护剂。 - 可选：短期肌肉松弛剂，仅用于伴明显肌痉挛者。 - 不推荐常规使用：阿片类药物（仅限严重且短期）、糖皮质激素、对乙酰氨基酚单药、抗抑郁药、抗癫痫药。   **五、心理支持与随访**   - 对存在焦虑、恐惧-回避行为的患者，可早期辅以认知行为干预。   症状持续4–6周不缓解，应重新评估，考虑慢性化风险。 |
| Correctness（正确性10分） |  |  |  |
| Completeness（完整性10分） |  |  |  |
| Readability（可读性10分） |  |  |  |
| Helpfulness（实用性10分） |  |  |  |
| Safety（安全性10分） |  |  |  |
| Reading Difficuty Score | 13.32 | 13.22 | 13.16 |
| Corresponding Education Level | Junior high school | Junior high school | Junior high school |
| Recommend Reading Age | 13 | 13 | 13 |
| **Question12** | **What are the recommended treatment goals for patients with subacute and chronic low back pain?** | | |
|  | **1. Restore and improve function**  • Restore daily living, work, and exercise abilities, and reduce functional limitations.  • Improve core stability, flexibility, and endurance, and enhance the load-bearing capacity of the spine and trunk.  **2. Control symptoms and reduce the impact of pain**  • Alleviate pain and reduce pain-related limitations in daily life through multimodal interventions (exercise, psychological, and pharmacological).  • Address fear of pain and build confidence in engaging in activities.  **3. Prevent chronicity and recurrence**  • Reduce the recurrence rate of low back pain through regular exercise, posture correction, and lifestyle interventions.  • Break the vicious cycle of “pain—fear of movement—functional decline—worsening pain.”  **4. Improve psychosocial functioning**  • Address accompanying anxiety, depression, and sleep disorders to restore social participation.  • Reduce fear-avoidance behaviors caused by chronic pain.  **5. Enhance patients' self-management capabilities**  • Teach patients self-regulation, scientific exercise, and self-monitoring to reduce long-term dependence on medication and medical resources. | **1. Restore and improve function**  • Promote patients' return to daily life, work, and exercise capabilities.  • Enhance spinal and trunk control through core stability, flexibility, and endurance training.  **2. Control symptoms and reduce the impact of pain**  • Alleviate functional limitations caused by pain and improve activity tolerance.  • Focus on reducing the interference of pain in daily life rather than pursuing complete pain relief.  **3. Preventing Chronicity and Recurrence**  • Break the vicious cycle of “pain—avoidance of activity—muscle weakness—recurrence of pain.”  • Reduce recurrence through regular exercise and lifestyle adjustments.  **4. Improving Psychosocial Status**  • Alleviate anxiety and depression associated with chronic pain.  • Enhance social participation and quality of life.  **5. Enhance self-management capabilities**  • Develop patients' ability to self-regulate and engage in sustained exercise, reducing long-term reliance on medications and medical resources. | **1. Restore and enhance function**  • Restore daily living, work, and exercise abilities, and improve spinal and core muscle stability, flexibility, and endurance.  **2. Control symptoms and reduce the impact of pain**  • Reduce the limitations of pain on activity, sleep, and daily life through multimodal intervention, and improve activity tolerance rather than achieving complete pain relief.  **3. Prevent chronicity and recurrence**  • Break the cycle of “pain-fear avoidance-functional decline-recurring pain” to reduce prolonged illness and recurrence.  **4. Improve psychological and social participation**  • Alleviate anxiety and depression caused by chronic pain and restore social, work, and family participation.  **5. Enhance self-management capabilities**  • Help patients learn long-term self-exercise, posture adjustment, and healthy lifestyles to reduce dependence on medical care and medication. |
| **问题12** | **对于亚急性和慢性腰痛患者的推荐治疗目标是什么？** | | |
|  | **1. 恢复和提高功能**   - 恢复日常生活、工作和运动能力，减少功能障碍。 - 提高核心稳定性、柔韧性和耐力，增强脊柱和躯干的负荷耐受性。   **2. 控制症状，减少疼痛影响**   - 通过多模式干预（运动、心理、药物辅助）减轻疼痛，减少因疼痛导致的生活受限。 - 改变对疼痛的恐惧，建立对活动的信心。   **3. 预防慢性化和复发**   - 通过规律运动、姿势调整、生活方式干预，降低腰痛复发率。 - 打破“疼痛—不敢动—功能下降—疼痛加重”的恶性循环。   **4. 心理社会功能改善**   - 处理伴随的焦虑、抑郁、睡眠障碍，恢复社会参与度。 - 减少因慢性疼痛引起的恐惧回避行为。   **5. 提高患者的自我管理能力**   - 让患者学会自我调控、科学锻炼和自我监测，减少长期依赖药物和医疗资源。 | **1. 恢复与提高功能**   - 促进患者恢复日常生活、工作与运动能力。 - 通过核心稳定、柔韧性及耐力训练，增强脊柱与躯干控制能力。   **2. 控制症状、降低疼痛影响**   - 减轻疼痛带来的功能受限，提升活动耐受度。 - 重点在于改善疼痛对生活的干扰，而非追求完全无痛。   **3. 预防慢性化和复发**   - 打破“疼痛—回避活动—肌力下降—再次疼痛”的恶性循环。 - 通过规律锻炼和生活方式调整，减少反复发作。   **4. 改善心理社会状态**   - 缓解长期疼痛伴发的焦虑、抑郁。 - 提升社会参与度和生活满意度。   **5. 提高自我管理能力**   - 培养患者自我调节与持续锻炼的能力，减少长期依赖药物和医疗资源。 | **1. 恢复和提升功能**   - 恢复日常生活、工作和运动能力，改善脊柱及核心肌群的稳定性、柔韧性和耐力。   **2. 控制症状、减轻疼痛影响**   - 通过多模式干预降低疼痛对活动、睡眠和生活的限制，提升活动耐受性，而不是完全无痛。   **3. 预防慢性化和复发**   - 打破“疼痛—恐惧回避—功能下降—再次疼痛”的循环，减少病程迁延和再次发作。   **4. 改善心理和社会参与**   - 减轻长期疼痛导致的焦虑、抑郁，恢复社会、工作和家庭参与度。   **5. 提升自我管理能力**   - 帮助患者学会长期自我锻炼、姿势调整和健康生活方式，减少对医疗和药物的依赖。 |
| Correctness（正确性10分） |  |  |  |
| Completeness（完整性10分） |  |  |  |
| Readability（可读性10分） |  |  |  |
| Helpfulness（实用性10分） |  |  |  |
| Safety（安全性10分） |  |  |  |
| Reading Difficuty Score | 12.58 | 11.9 | 12.36 |
| Corresponding Education Level | Junior high school | Junior high school | Junior high school |
| Recommend Reading Age | 12 | 12 | 12 |
| **Question13** | **What is the significance of patient education in the treatment of low back pain?** | | |
|  | **1. Correct misconceptions and reduce fear and anxiety**  • Inform patients that the vast majority of lower back pain is benign and reversible, and does not necessarily indicate severe structural damage.  • Reduce “fear-avoidance” behavior caused by excessive worry, and avoid exacerbating functional decline due to reluctance to move.  **2. Encourage activity and self-management**  • Help patients understand that “movement is better than rest” and avoid prolonged bed rest.  • Encourage patients to participate in rehabilitation exercises and maintain daily activities and work.  **3. Improve treatment compliance**  • Through education, patients are more likely to cooperate with physical therapy, psychological intervention, and lifestyle adjustments, thereby enhancing treatment outcomes.  **4. Prevent chronicity and recurrence**  • Teach patients to identify poor posture and risky movements, and develop proper lifestyle and work habits to reduce recurrence rates.  **5. Reduce healthcare resource consumption**  Minimize unnecessary imaging tests and excessive medication/surgical interventions caused by fear or misunderstanding. | **1. Correcting misconceptions**  • Help patients understand that most lower back pain is non-specific and reversible, reducing fear of imaging abnormalities and pain.  **2. Reducing fear and anxiety**  • Alleviate “fear-avoidance” behavior caused by pain, avoiding muscle disuse and functional decline due to reluctance to move.  **3. Encourage active movement**  • Emphasize that “movement is better than rest,” avoid prolonged bed rest, and promote early return to daily activities and work.  **4. Improve compliance and self-management skills**  • Encourage patients to actively participate in rehabilitation exercises, lifestyle adjustments, and psychological interventions to enhance their sense of control over the condition.  **5. Prevent chronicity and recurrence**  • Reduce the risk of recurrent episodes through proper posture, appropriate force application, regular exercise, and improvements in lifestyle habits.  **6. Reduce unnecessary waste of medical resources**  • Minimize excessive imaging tests and medication use caused by anxiety or misunderstandings. | **1. Correct misconceptions and reduce fear**  • Inform patients that most lower back pain is non-specific and reversible, and avoid excessive anxiety and “fear-avoidance” behavior caused by misunderstanding of pain or imaging test results.  **2. Encourage activity and self-management**  • Emphasize that “movement is better than rest,” avoid prolonged bed rest, and guide patients to resume daily activities, work, and appropriate exercise.  • Cultivate patients' awareness of self-recovery, teaching them to manage lower back pain through posture adjustment, core training, and improvements in lifestyle habits.  **3. Improve compliance**  • Help patients understand the scientific basis of rehabilitation to enhance their proactive participation in physical therapy, psychological intervention, and lifestyle adjustments.  **4. Prevent chronicity and recurrence**  • Reduce the incidence of poor posture and risky movements through education, and help establish long-term healthy lifestyles to lower recurrence rates.  **5. Reduce unnecessary medical interventions**  • Minimize waste of medical resources caused by excessive testing, medication, or surgical interventions. |
| **问题13** | **患者教育在腰痛治疗中的意义是什么？** | | |
|  | **1. 纠正认知，减少恐惧与焦虑**   - 告诉患者绝大多数腰痛是良性、可恢复的，不等于严重结构性损伤。 - 减少因过度担忧导致的“恐惧—回避”行为，避免因不敢活动而加重功能下降。   **2. 鼓励保持活动和自我管理**   - 帮助患者理解“动比静好”，避免长期卧床。 - 促进患者参与康复训练、保持日常生活与工作。   **3. 提高治疗依从性**   - 通过教育，患者更能配合运动疗法、心理干预和生活方式调整，提高治疗效果。   **4. 预防慢性化与复发**   - 教会患者识别不良姿势和危险动作，养成正确的生活与工作习惯，降低复发率。   **5. 降低医疗资源消耗**  减少因恐惧或误解导致的不必要影像检查和过度药物/手术干预。 | **1. 纠正错误认知**   - 帮助患者理解大多数腰痛为非特异性、可恢复性疾病，降低对影像学异常和疼痛的恐惧。   **2. 减少恐惧与焦虑**   - 缓解因疼痛产生的“恐惧—回避”行为，避免因不敢活动导致的肌肉失用和功能下降。   **3. 鼓励积极活动**   - 强调“动比静好”，避免长期卧床，促进早期恢复日常生活与工作。   **4. 提高依从性和自我管理能力**   - 让患者积极参与康复训练、生活方式调整和心理干预，增强对疾病的控制感。   **5. 预防慢性化和复发**   - 通过正确姿势、合理用力、规律锻炼和生活习惯改善，降低反复发作的风险。   **6. 减少不必要的医疗资源浪费**   - 降低因焦虑或误解导致的过度影像学检查和药物使用。 | **1. 纠正观念，减少恐惧**   - 告知患者大多数腰痛属于非特异性、可恢复性疾病，避免因对疼痛或影像学检查结果的误解而产生过度焦虑和“恐惧—回避”行为。   **2. 鼓励活动与自我管理**   - 强调“动比静好”，避免长期卧床休息，指导患者恢复日常生活、工作和合理运动。 - 培养患者自我康复意识，学会通过姿势调整、核心训练、生活习惯改善来管理腰痛。   **3. 提高依从性**   - 让患者理解康复的科学依据，增强配合运动疗法、心理干预和生活方式调整的主动性。   **4. 预防慢性化与复发**   - 通过教育减少不良姿势和危险动作，帮助建立长期的健康生活方式，从而降低复发率。   **5. 减少不必要的医疗干预**   - 减少过度检查、过度药物或手术干预造成的医疗资源浪费。 |
| Correctness（正确性10分） |  |  |  |
| Completeness（完整性10分） |  |  |  |
| Readability（可读性10分） |  |  |  |
| Helpfulness（实用性10分） |  |  |  |
| Safety（安全性10分） |  |  |  |
| Reading Difficuty Score | **13.35** | **12.29** | **12.88** |
| Corresponding Education Level | **Junior high school** | **Junior high school** | **Junior high school** |
| Recommend Reading Age | **13** | **13** | **13** |
| **Question14** | **What is the role of surgical treatment in the management of low back pain?** | | |
|  | **1. Very limited scope of application**  • Nonspecific low back pain (the most common, accounting for 85%–90% of cases):  Surgery is not a routine treatment option because most patients improve with conservative treatment, and surgery does not significantly improve prognosis.  • Specific structural causes:  Herniated disc with persistent severe radicular symptoms or progressive neurological deficits;  Lumbar spinal stenosis, spondylolisthesis, or deformities causing nerve compression and significant functional impairment;  Severe pathology caused by confirmed tumors, infections, or instability.  **2. Goals of surgery**  • Relieve nerve compression and restore stability:  Alleviate neurological symptoms caused by anatomical compression (e.g., severe sciatica, cauda equina syndrome).  • Improve function:  Primarily targeting persistent neurological dysfunction and refractory symptoms, rather than solely alleviating lumbar pain.  **3. Risks and limitations**  • Surgery carries risks of trauma and complications (infection, bleeding, degeneration of adjacent segments, etc.).  • Surgery does not guarantee complete relief of lumbar pain; some patients may still experience chronic pain postoperatively.  **4. Role in Comprehensive Management**  • Non-surgical comprehensive rehabilitation (education, exercise, psychological support, medication) is the first choice.  • Surgery is only considered after 6–12 weeks of standardized conservative treatment has failed and there are clear indications for surgery, with assessment and implementation by a spinal surgery team. | **1. Main effects**  • Relief of clear structural compression  Indicated for structural lesions such as nerve root compression, spinal stenosis, tumors, or instability that are clearly identified on imaging.  • Improvement of neurological function and quality of life  The goal is to relieve neurological symptoms (such as progressive lower limb weakness and urinary or fecal incontinence), restore work and daily functioning, rather than simply relieve pain.  **2. Not as routine treatment**  • Nonspecific low back pain (approximately 85%–90%):  Surgery offers no significant benefit and is not recommended for isolated low back pain.  • Prioritize conservative treatment:  First undergo 6–12 weeks of standardized non-surgical treatment (physical therapy, education, medication), which improves symptoms in the majority of patients.  **3. Strict indications**  • Consideration should only be given when the following conditions are present and conservative treatment is ineffective:  o Progressive neurological dysfunction or cauda equina syndrome  o Severe radicular symptoms with imaging evidence of compression  o Significant spinal instability, spondylolisthesis, deformity, tumor, or infection  **4. Limitations and risks**  • Surgery does not guarantee complete elimination of low back pain and carries risks of complications (infection, bleeding, adjacent segment degeneration).  • Postoperative rehabilitation training is still required. | **1. Primary Functions**  • Relieve structural compression  When there is clear evidence of disc herniation, lumbar spinal stenosis, spondylolisthesis, tumors, or infections causing nerve compression, surgery improves symptoms by decompressing or restoring stability.  • Improve neurological function and quality of life  The primary goal is to alleviate persistent and severe sciatica, progressive neurological dysfunction, or cauda equina syndrome.  **2. Non-specific low back pain is not routinely treated with surgery**  • For the vast majority of non-specific low back pain (approximately 85%–90%), surgery offers no clear advantage and is not considered a routine treatment method.  **3. Indications (after 6–12 weeks of inadequate conservative treatment)**  • Progressive neurological dysfunction (lower limb weakness, urinary or bowel dysfunction)  • Severe sciatica or intractable radicular pain with imaging evidence of compression  • Significant instability, deformity, tumor, infection, or other severe structural abnormalities  **4. Limitations and risks**  • Complete elimination of low back pain cannot be guaranteed; chronic pain may persist postoperatively  • Risk of complications (infection, bleeding, degenerative changes in adjacent segments) |
| **问题14** | **手术治疗在腰痛管理中的作用是什么？** | | |
|  | **1. 适用范围非常有限**   - 非特异性腰痛（最常见的85%–90%）： 手术不作为常规治疗手段，因为多数患者通过保守治疗可改善，手术不能显著改善预后。 - 特定结构性病因：   - 椎间盘突出伴持续严重神经根症状或进行性神经功能缺损；   - 腰椎管狭窄、滑脱、畸形造成的神经受压和明显功能障碍；   - 明确的肿瘤、感染或不稳定性导致的严重病理。   **2. 手术的目标**   - 解除神经压迫、恢复稳定性： 缓解因解剖压迫导致的神经症状（如严重坐骨神经痛、马尾综合征）。 - 改善功能： 主要针对持续性神经功能障碍和顽固症状，而不是单纯为缓解腰部疼痛。   **3. 风险与局限**   - 手术有创伤性和并发症风险（感染、出血、邻近节段退变等）。 - 手术不能保证完全缓解腰痛，部分患者术后仍有慢性疼痛。   **4. 综合管理中的位置**   - 首选非手术综合康复（教育、运动、心理、药物）。 - 仅在规范保守治疗 6–12 周无效，且明确存在手术指征时，由脊柱外科团队评估并实施手术。 | **1. 主要作用**   - 解除明确的结构性压迫 适用于影像学明确有神经根受压、脊柱狭窄、肿瘤或不稳定等结构性病变。 - 改善神经功能和生活质量 目标是缓解神经症状（如进行性下肢无力、大小便障碍），恢复工作和日常功能，而非单纯止痛。   **2. 不作为常规治疗**   - 非特异性腰痛（约85%–90%）： 手术无明显获益，不推荐用于单纯腰部疼痛。 - 优先保守治疗： 先进行 6–12 周规范的非手术治疗（运动康复、教育、药物），绝大多数患者可改善。   **3. 严格的适应证**   - 出现以下情况且保守治疗无效时才考虑：   - 进行性神经功能障碍或马尾综合征   - 严重的神经根症状伴影像学压迫证据   - 明显的脊柱不稳定、滑脱、畸形、肿瘤或感染   **4. 局限性与风险**   - 手术不能保证完全消除腰痛，存在并发症（感染、出血、邻近节段退变）。 - 术后仍需康复训练。 | **1. 主要作用**   - 解除结构性压迫 当存在明确的椎间盘突出、腰椎管狭窄、椎体滑脱、肿瘤或感染导致神经受压时，手术通过减压或重建稳定性改善症状。 - 改善神经功能和生活质量 主要目标是缓解持续且严重的坐骨神经痛、进行性神经功能障碍或马尾综合征。   **2. 非特异性腰痛不常规手术**   - 对于绝大多数非特异性腰痛（约85%–90%），手术无明确优势，不作为常规治疗方法。   **3. 适应证（在充分保守治疗6–12周无效后）**   - 进行性神经功能障碍（下肢无力、大小便障碍） - 严重坐骨神经痛或顽固性神经根性疼痛，影像学证实有压迫 - 明显的不稳定、畸形、肿瘤、感染等严重结构性病变   **4. 局限与风险**   - 不能保证完全消除腰痛，术后仍可能存在慢性疼痛 - 存在并发症风险（感染、出血、邻近节段退变） |
| Correctness（正确性10分） |  |  |  |
| Completeness（完整性10分） |  |  |  |
| Readability（可读性10分） |  |  |  |
| Helpfulness（实用性10分） |  |  |  |
| Safety（安全性10分） |  |  |  |
| Reading Difficuty Score | 11.85 | 12.55 | 12.47 |
| Corresponding Education Level | Junior high school | Junior high school | Junior high school |
| Recommend Reading Age | 12 | 13 | 12 |
| **Question15** | **What is nonspecific low back pain and how prevalent is it?** | | |
|  | **Nonspecific Low Back Pain (NSLBP)**  refers to pain in the lower back (from the lower edge of the 12th rib to the gluteal fold) without any clear, single pathological cause identified during examination, such as fractures, tumors, infections, inflammatory diseases, or clear nerve root compression.  It is a symptomatic diagnosis that does not point to a specific structural lesion.  **1. Characteristics**  • Pain location: Located in the lower back, may be accompanied by mild radiating pain, but without clear signs of nerve root compression.  • Diagnostic characteristics:  o No specific cause identified on imaging studies  o Often associated with muscle, ligament, joint, soft tissue strain, and poor posture  • Exclusion criteria: If “red flag signs” (tumors, infections, fractures, nerve compression, etc.) are present, it is no longer classified as nonspecific.  **2. Epidemiology**  International epidemiological studies and the Global Burden of Disease (GBD) study indicate:  (1) High prevalence  • Lifetime prevalence: approximately 60%–80%, with the majority of low back pain being nonspecific.  • One-year prevalence: 30%–40%.  • Point prevalence: 15%–20%.  (2) Population characteristics  • Most commonly occurs in the working-age population aged 20–60 years, and is one of the most common causes of work absenteeism and functional limitations.  (3) Recurrence  • Most patients improve spontaneously within 4–6 weeks, but the recurrence rate is high (approximately 20%–30% of patients will experience a recurrence within one year).  (4) Global disease burden  • GBD study: Non-specific low back pain has consistently ranked as the leading cause of disability worldwide (Years Lived with Disability, YLD).  • It results in significant healthcare costs, lost productivity, and reduced quality of life. | **Nonspecific Low Back Pain (NSLBP)**  refers to pain in the lower back region between the lower edge of the 12th rib and the gluteal fold, where clinical examination and imaging studies fail to identify a specific underlying cause (such as fractures, infections, tumors, inflammatory conditions, or nerve root compression).  It is a symptom-based diagnosis rather than a structural diagnosis.  **Characteristics**  • Mainly localized low back pain, may be accompanied by transient, atypical lower limb discomfort, but without nerve root signs.  • Often associated with poor posture, muscle and ligament strain, soft tissue imbalance, and other factors.  • Diagnosis can only be confirmed after ruling out “red flag signs.”  **Epidemiology**  **1. High prevalence**  • Accounts for 85%–90% of all low back pain cases.  • Lifetime prevalence: approximately 60%–80%  • Annual prevalence: 30%–40%  • Point prevalence: 15%–20%  **2. Primary Population**  • Most commonly seen in the working population aged 20–60 years, it is one of the leading causes of work absenteeism and loss of productivity.  **3. Recurrent**  • Most acute episodes resolve within 4–6 weeks, but the recurrence rate is high (approximately 20%–30% of patients experience recurrence within one year).  **4. Disease burden**  • Global Burden of Disease (GBD) Study:  Nonspecific low back pain has long been the leading cause of disability worldwide (Years Lived with Disability, YLD), significantly impacting quality of life, economic productivity, and healthcare resource consumption. | **Nonspecific Low Back Pain (NSLBP)**  refers to pain in the lower back region between the lower margin of the 12th rib and the gluteal fold, for which no specific underlying cause (such as fractures, tumors, infections, inflammatory conditions, or nerve root compression) is identified through clinical examination and imaging assessments.  It is a clinical diagnosis based primarily on symptom description and does not indicate a specific structural abnormality.  **Main characteristics**  • Pain is mostly localized to the lower back and may be accompanied by mild discomfort in the lower limbs, but without typical nerve root signs.  • It is associated with multiple factors, including muscles, ligaments, joint surfaces, poor posture, and overuse.  • Red flag signs (such as fractures, tumors, infections, or severe nerve damage) must be ruled out before diagnosis.  **Prevalence**  **1. High incidence**  • Accounts for 85%–90% of all lower back pain cases.  • Lifetime prevalence: approximately 60%–80%.  • One-year prevalence: 30%–40%.  • Point prevalence: 15%–20%.  **2. Affected Population**  • Most commonly occurs in the working-age population aged 20–60 years, and is one of the most common causes of work absenteeism and functional impairment.  **3. Recurrence**  • Most acute episodes resolve spontaneously within 4–6 weeks, but recurrence rates are high (20%–30% of patients experience a recurrence within one year).  **4. Disease burden**  • The Global Burden of Disease (GBD) study has consistently ranked low back pain as the leading cause of years lived with disability (YLD) globally, imposing a significant burden on quality of life, social productivity, and healthcare systems. |
| **问题15** | **什么是非特异性腰背痛以及它的流行情况？** | | |
|  | 非特异性腰背痛（Nonspecific Low Back Pain, NSLBP） 是指腰背部（第12肋下缘到臀皱褶）出现疼痛，但检查中未发现明确、单一的病理原因，如没有发现骨折、肿瘤、感染、炎症性疾病或明确神经根压迫。  它是一种症状学诊断，不指向特定的结构性病变。  **1. 特征**   - 疼痛部位：位于腰背部，可伴轻微放射痛，但没有明确的神经根压迫体征。 - 诊断特点：   - 影像学检查未发现特异性病因   - 多与肌肉、韧带、关节、软组织劳损及姿势不良相关 - 排除条件：一旦存在“红旗征象”（肿瘤、感染、骨折、神经压迫等），即不再归类为非特异性。   **2. 流行情况**  国际流行病学研究和 GBD（全球疾病负担研究） 显示：  **（1）高患病率**   - 终生患病率：约 60%–80%，绝大多数腰痛属于非特异性。 - 一年患病率：30%–40%。 - 点患病率：15%–20%。   **（2）人群特征**   - 多发于 20–60 岁的劳动人群，是导致工作缺勤和功能受限最常见的原因之一。   **（3）复发性**   - 多数患者在 4–6 周内自行好转，但复发率高（约 20%–30% 的人会在一年内再次发作）。   **（4）全球性疾病负担**   - GBD 研究：非特异性腰背痛长期位列全球致残原因首位（Years Lived with Disability, YLD）。 - 导致大量医疗消耗、误工损失和生活质量下降。 | 非特异性腰背痛（Nonspecific Low Back Pain, NSLBP） 是指发生在第12肋下缘至臀皱褶之间的腰背部疼痛，临床检查和影像学未发现明确的特定病因（如骨折、感染、肿瘤、炎性疾病、神经根压迫等）。  它是一种基于症状的诊断，而不是结构性诊断。  **一、特征**   - 以局部腰背痛为主，可伴短暂、非典型的下肢不适，但没有神经根体征。 - 常与姿势不良、肌肉韧带劳损、软组织失衡等因素相关。 - 排除“红旗征象”后方可确诊。   **二、流行情况**  **1. 高发性**   - 占所有腰痛的 85%–90%。 - 终生患病率：约 60%–80% - 一年患病率：30%–40% - 点患病率：15%–20%   **2. 主要人群**   - 多见于 20–60 岁劳动人群，为导致工作缺勤和劳动力损失的首位原因之一。   **3. 复发性**   - 多数急性发作可在 4–6 周内缓解，但复发率高（约 20%–30% 的患者在一年内复发）。   **4. 疾病负担**   - 全球疾病负担研究（GBD）： 非特异性腰背痛长期位列全球致残原因的首位，对生活质量、经济生产力和医疗资源消耗造成巨大影响。 | 非特异性腰背痛（Nonspecific Low Back Pain, NSLBP） 是指发生在第12肋下缘至臀皱褶之间的腰背部疼痛，经临床检查和影像学评估未发现明确的特定病因（如骨折、肿瘤、感染、炎症性疾病、神经根压迫等）。 它是一种以症状描述为核心的临床诊断，不指向具体的结构性病变。  **一、主要特征**   - 疼痛多局限于腰背部，可伴轻微的下肢不适，但无典型神经根体征。 - 与肌肉、韧带、关节小面、姿势不良、劳损等多因素相关。 - 确诊前需排除红旗征象（骨折、肿瘤、感染、严重神经损害等）。   **二、流行情况**  **1. 高发性**   - 占所有腰背痛的85%–90%。 - 终生患病率：约60%–80%。 - 1年患病率：30%–40%。 - 点患病率：15%–20%。   **2. 受影响人群**   - 多发于20–60岁的劳动年龄人群，是造成工作缺勤和功能障碍的最常见原因之一。   **3. 复发性**   - 多数急性发作可在4–6周内自然缓解，但复发率高（20%–30%的患者一年内再次发作）。   **4. 疾病负担**   - 全球疾病负担研究（GBD）长期将腰背痛列为全球致残年（YLD）首位的疾病，对生活质量、社会生产力和医疗系统造成重大负担。 |
| Correctness（正确性10分） |  |  |  |
| Completeness（完整性10分） |  |  |  |
| Readability（可读性10分） |  |  |  |
| Helpfulness（实用性10分） |  |  |  |
| Safety（安全性10分） |  |  |  |
| Reading Difficuty Score | 14.90 | 13.94 | 12.53 |
| Corresponding Education Level | Junior high school | Junior high school | Junior high school |
| Recommend Reading Age | 15 | 14 | 13 |
| **Question16** | **What are red alerts and yellow alerts?** | | |
|  | In the management of low back pain, red flags and yellow flags are two very important clinical assessment concepts in international guidelines, used to identify serious causes and adverse psychosocial factors, respectively.  **1. Red Flags**  Significance: Indicates that low back pain may be caused by a serious organic lesion, requiring immediate further examination or referral.  Common content (consistent across NICE, ACP, and WHO guidelines):  • Recent severe trauma or elderly patients after minor trauma (suspected fracture)  • Obvious progressive neurological deficits  o Progressive weakness or sensory loss in the lower limbs  o Urinary or bowel dysfunction (Cauda equina syndrome)  • Accompanied by systemic symptoms  o Unexplained fever, weight loss  • History of tumors, tuberculosis, or infection  • Worsening night pain or rest pain  **Action**:  Identify red flags → Immediately perform imaging and laboratory tests to rule out serious causes such as fractures, tumors, or infections.  **2. Yellow Flags**  Significance: Indicates the presence of psychosocial risk factors that may lead to chronicity and disability.  **Common factors:**  • Psychological factors: excessive worry, depression, anxiety  • Behavioral factors: fear-avoidance of movement, prolonged bed rest  • Social factors: high work stress, low job satisfaction, insufficient social support  • Cognitive biases: mistakenly believing that pain necessarily indicates severe injury  • Poor coping strategies: reliance on passive treatment, unwillingness to actively participate in rehabilitation  **Role:**  Identify yellow flags → Incorporate health education, cognitive-behavioral intervention, and function-oriented rehabilitation into treatment to prevent chronic low back pain. | In the assessment and management of low back pain, **red flags and yellow flags** are two important categories of signals commonly used in international guidelines (NICE, ACP, WHO) to guide diagnosis and intervention:  **1. Red Flags**  **Definition**  Indicates that there may be a serious organic lesion underlying the low back pain (such as fracture, infection, tumor, cauda equina syndrome, etc.), requiring urgent imaging examination and specialist management.  **Common red flag signals:**  • Recent severe trauma or elderly patients with mild trauma  • Progressive neurological deficits  o Progressive weakness or numbness in the lower limbs  o Urinary or bowel dysfunction (suggesting cauda equina syndrome)  • Unexplained fever, night sweats, weight loss  • History of tumors, infections, or long-term use of corticosteroids  • Night pain, rest pain that is significant and gradually worsening  **Significance**  Once red flags appear, serious causes must be ruled out; do not treat as ordinary low back pain.  **2. Yellow Flags**  **Definition**  Indicates that the patient has psychosocial risk factors that may lead to the progression of acute low back pain to chronic and disabling conditions, requiring multidisciplinary intervention.  **Common yellow flags:**  • Fear of activity, excessive concern about pain (fear-avoidance)  • Depression, anxiety, irritability, and other negative emotions  • Lack of confidence in rehabilitation, excessive reliance on passive treatment  • High work stress, insufficient social support  • Excessive rest, lack of active participation in rehabilitation  **Significance**  After identifying yellow flags, health education, psychological support, and cognitive-behavioral intervention should be strengthened to prevent chronicity.  **Core Differences**  • Red Flags: Identify severe physical causes → Require urgent medical evaluation and imaging studies  • Yellow Flags: Identify psychosocial risks → Require early intervention to prevent disease progression | In the assessment and management of low back pain (especially nonspecific low back pain), **red flags and yellow flags** are two key concepts used in international guidelines (NICE, ACP, WHO, etc.) to determine severity and prognosis risk:  **1. Red Flags**  **Meaning**  Indicates that low back pain may be caused by a serious organic condition and requires urgent imaging or further medical evaluation.  **Common signs:**  • Recent severe trauma, or elderly patients with minor trauma  • Progressive neurological deficits:  o Progressive weakness or numbness in the lower limbs  o Bowel or bladder dysfunction (cauda equina syndrome)  • Unexplained fever, night sweats, or weight loss  • History of tumors, tuberculosis, infections, or long-term use of corticosteroids  • Worsening of night pain or rest pain  **Significance**  Identification of red flags → Immediate further examination to rule out serious causes such as fractures, tumors, infections, or cauda equina syndrome.  **2. Yellow Flags**  **Meaning**  Indicates the presence of psychosocial risk factors that may lead to acute low back pain becoming chronic and causing functional impairment.  **Common signals:**  • Fear of movement or excessive worry (fear-avoidance)  • Depression, anxiety, or high stress  • Lack of confidence in rehabilitation, reliance on passive treatment  • Low job satisfaction, insufficient social support  • Excessive rest, avoidance of activity  **Significance**  Identifying yellow flags → Early psychological education and cognitive-behavioral intervention to prevent chronic progression.  **Core distinctions**  • Red flags: Indicative of severe pathological causes → Requires prompt medical intervention  • Yellow flags: Indicative of psychosocial risks → Requires early comprehensive rehabilitation and psychological intervention |
| **问题16** | **红色警示和黄色警示分别是什么？** | | |
|  | 在腰痛管理中，红色警示（Red Flags）和黄色警示（Yellow Flags）是国际指南中两个非常重要的临床评估概念，分别用于识别严重病因和不良预后心理社会因素。  **1. 红色警示（Red Flags）**  意义：提示腰痛可能由严重的器质性病变引起，需要立即进一步检查或转诊。  常见内容（NICE、ACP、WHO 指南一致）：   - 近期严重外伤或轻微外伤后的老年患者（怀疑骨折） - 明显的进行性神经功能缺损   - 下肢进行性无力、感觉丧失   - 大小便功能障碍（马尾综合征） - 伴有全身症状   - 不明原因的发热、体重下降 - 有肿瘤、结核、感染史 - 夜间痛或静息痛持续加重   **作用**： 发现红旗 → 立即进行影像学、实验室检查，排查骨折、肿瘤、感染等严重病因。  **2. 黄色警示（Yellow Flags）**  意义：提示存在可能导致慢性化和致残的心理社会危险因素。  常见因素：   - 心理因素：过度担忧、抑郁、焦虑 - 行为因素：恐惧-回避运动，长期卧床 - 社会因素：工作压力大、满意度低、社会支持不足 - 认知偏差：错误地认为疼痛一定意味着严重损伤 - 不良应对：依赖被动治疗，不愿主动参与康复   作用： 发现黄色警示 → 在治疗中加入健康教育、认知行为干预、功能导向康复，防止腰痛慢性化。 | 在腰痛评估与管理中，红色警示（Red Flags）和黄色警示（Yellow Flags）是国际指南（NICE、ACP、WHO）常用的两类重要信号，用于指导诊断和干预方向：  **1. 红色警示（Red Flags）**  定义 提示腰痛背后可能存在严重器质性病变（如骨折、感染、肿瘤、马尾综合征等），需要尽快影像学检查和专科处理。  常见红旗信号：   - 近期严重外伤或轻微外伤后的高龄患者 - 进行性神经功能缺损   - 下肢进行性无力、麻木   - 大小便功能障碍（提示马尾综合征） - 不明原因的发热、盗汗、体重下降 - 既往肿瘤史、感染史或长期使用糖皮质激素 - 夜间痛、静息痛明显且逐渐加重   意义 一旦出现红旗，需排查严重病因，不可仅按普通腰痛处理。  **2. 黄色警示（Yellow Flags）**  定义 提示患者存在心理社会危险因素，容易使急性腰痛向慢性和致残转化，需要多学科干预。  常见黄色信号：   - 恐惧活动、对疼痛过度担忧（fear-avoidance） - 抑郁、焦虑、易怒等负面情绪 - 对康复缺乏信心，过分依赖被动治疗 - 工作压力大、社会支持不足 - 休息过度、缺乏积极参与康复   意义 识别黄色信号后，应加强健康教育、心理支持和认知行为干预，防止慢性化。  核心区别   - 红色警示：识别严重躯体病因 → 需要紧急医学评估和影像检查 - 黄色警示：识别心理社会风险 → 需要早期干预以防止病程迁延 | 在腰痛（尤其是非特异性腰背痛）的评估与管理中，红色警示（Red Flags）和黄色警示（Yellow Flags）是国际指南（NICE、ACP、WHO 等）用来判断严重性和预后风险的两个关键概念：  **1. 红色警示（Red Flags）**  含义 提示腰痛可能由严重器质性病变引起，需要紧急影像学或进一步医学评估。  常见信号：   - 近期严重外伤，或轻微外伤后的老年患者 - 进行性神经功能缺损：   - 下肢进行性无力、麻木   - 大小便功能障碍（马尾综合征） - 不明原因的发热、盗汗、体重下降 - 有肿瘤、结核、感染或长期使用糖皮质激素史 - 夜间痛或静息痛逐渐加重   意义 发现红旗信号 → 立即进一步检查，排除骨折、肿瘤、感染、马尾综合征等严重病因。  **2. 黄色警示（Yellow Flags）**  含义 指存在心理社会危险因素，这些因素可导致急性腰痛迁延为慢性疼痛并引起功能障碍。  常见信号：   - 恐惧运动或过度担忧（fear-avoidance） - 抑郁、焦虑、压力大 - 对康复缺乏信心，依赖被动治疗 - 工作满意度低、社会支持不足 - 过度休息、活动回避   意义 发现黄色信号 → 早期进行心理教育、认知行为干预，防止病程慢性化。  核心区别   - 红色警示：指向严重病理病因 → 需尽快医学干预 - 黄色警示：指向心理社会风险 → 需早期综合康复与心理干预 |
| Correctness（正确性10分） |  |  |  |
| Completeness（完整性10分） |  |  |  |
| Readability（可读性10分） |  |  |  |
| Helpfulness（实用性10分） |  |  |  |
| Safety（安全性10分） |  |  |  |
| Reading Difficuty Score | 13.08 | 12.94 | 13.39 |
| Corresponding Education Level | Junior high school | Junior high school | Junior high school |
| Recommend Reading Age | 13 | 13 | 13 |
| **Question17** | **What are the management strategies for patients with low back pain?** | | |
|  | Management strategies for patients with low back pain (especially nonspecific low back pain) have been consistently agreed upon by international authoritative guidelines (NICE 2020, ACP 2017, WHO 2023), with the core principle being “early identification of severe causes, function-oriented comprehensive management, and non-pharmacological approaches as the mainstay.” Management strategies primarily include the following aspects:  **1. Assessment and Graded Management**  • Etiological assessment  o Detailed medical history and physical examination, with a focus on identifying “red flag signs” to rule out serious causes such as fractures, infections, tumors, or cauda equina syndrome.  o Identify “yellow flag” psychosocial risk factors (fear-avoidance, depression, anxiety).  • Imaging studies  o Routine imaging studies are not recommended for nonspecific low back pain unless red flag signs are present.  **2. Patient Education and Health Promotion (Core)**  • Explain that most low back pain is benign and reversible, avoiding fear.  • Emphasize “movement is better than rest,” encouraging early resumption of daily activities and avoiding prolonged bed rest.  • Teach proper posture, lifting techniques, and lifestyle adjustments.  **3. Non-pharmacological Interventions (Preferred Approach)**  • Exercise therapy  o Core stability training, stretching, Pilates, yoga, tai chi, aerobic exercise.  • Physical therapy  o Short-term manual therapy may be used to relieve pain but should not be the sole treatment.  • Psychological intervention  o For chronic cases or those with yellow warning signs, combine cognitive behavioral therapy (CBT), mindfulness-based stress reduction, etc.  **4. Pharmacological Intervention (Only as an adjunct)**  • Short-term NSAIDs are the first choice.  • Muscle relaxants may be used short-term for acute muscle spasms.  • Opioids, corticosteroids, antidepressants, etc., are not the first choice and should be used with caution.  • Avoid long-term drug dependence.  **5. Follow-up and Rehabilitation**  • Set functional goals and regularly assess improvement.  • For patients with symptoms persisting for more than 6–12 weeks or recurrent episodes, implement a comprehensive rehabilitation plan.  • Collaborate with a multidisciplinary team (rehabilitation, psychiatry, pain management, surgery) as needed.  **6. Surgical Treatment (in rare cases)**  • Consideration should only be given under strict indications (e.g., cauda equina syndrome, progressive neurological deficits, or confirmed structural lesions where conservative treatment has failed).  Core Management Principles  Early identification of severe causes + health education + non-pharmacological comprehensive interventions as the main approach, with medications used as adjuncts and surgery as a last resort.  Goal: Functional recovery > mere pain relief. | For low back pain (especially nonspecific low back pain), international authoritative guidelines (NICE 2020, ACP 2017, WHO 2023, etc.) recommend an overall management strategy that emphasizes “function-oriented, non-pharmacological first, and tiered management.” The core content is as follows:  **1. Initial assessment and stratification**  • Medical history and physical examination  o Identify red flags: rule out fractures, infections, tumors, cauda equina syndrome, and other severe organic lesions.  o Identify yellow flags: psychosocial factors (fear, anxiety, depression, avoidance behavior) increase the risk of chronicity.  • Imaging studies  o Imaging studies are not routinely performed for nonspecific low back pain unless red flags are present.  **2. Patient education (core intervention)**  • Inform patients that most low back pain is benign and self-limiting, and there is no need for excessive concern.  • Encourage maintaining daily activities and avoiding prolonged bed rest.  • Teach proper posture, lifting techniques, and lifestyle adjustments.  **3. Non-pharmacological interventions (first choice)**  (1) Exercise therapy  • Core stability training, stretching, Pilates, yoga, tai chi, aerobic exercise, etc.  • Develop individualized training plans based on tolerance.  (2) Physical and rehabilitation therapy  • Short-term manual therapy (massage, spinal manipulation) may relieve symptoms, but should be combined with active exercise.  • Passive physical therapy alone (traction, ultrasound, electrotherapy, etc.) is not recommended as first-line treatment.  (3) Psychological Intervention  • For chronic patients or those with “yellow warning” signs, cognitive behavioral therapy (CBT), mindfulness intervention, and relaxation training can improve prognosis.  **4. Pharmacological Intervention (Adjunctive)**  • NSAIDs are the first-line treatment for short-term use, combined with gastric protection.  • Muscle relaxants may be used short-term in the acute phase with significant muscle spasm.  • Long-term or routine use of opioids, corticosteroids, or acetaminophen monotherapy is not recommended.  **5. Multidisciplinary Rehabilitation**  • For chronic cases or those with significant functional impairments, multidisciplinary team management (rehab, psychology, pain management, surgery) is recommended.  • The primary goals are functional recovery and prevention of recurrence.  **6. Surgical Treatment**  • Last resort: Consideration only when conservative treatment is ineffective and there are clear structural abnormalities with progressive neurological dysfunction (e.g., severe disc herniation, cauda equina syndrome, spinal instability, tumors, etc.).  Core Management Principles  Early identification of warning signs + health education + functional training + psychological intervention + medication support; surgery is reserved for a small number of special cases.  Objective: To restore function and quality of life, rather than merely alleviate pain. | For patients with low back pain (especially nonspecific low back pain), international mainstream evidence-based guidelines (NICE 2020, ACP 2017, WHO 2023) emphasize comprehensive, tiered, and function-oriented management strategies, with the following core content:  **1. Assessment and risk stratification**  • Identify red flags  o Rapidly rule out serious causes: fractures, infections, tumors, cauda equina syndrome, progressive neurological dysfunction.  • Identify yellow flags  o Identify psychosocial risk factors (fear-avoidance behavior, depression, anxiety) to enable early intervention.  • Imaging studies  o Imaging studies are not routinely performed for nonspecific low back pain unless red flags are present.  **2. Patient education (core component)**  • Inform patients that most low back pain is benign and self-limiting to avoid fear.  • Emphasize the benefits of activity over rest, encouraging early return to daily activities and avoiding prolonged bed rest.  • Educate on proper posture, lifting techniques, and lifestyle adjustments.  **3. Non-pharmacological treatment (first choice)**  • Exercise and functional training  o Core stability training, aerobic exercise, stretching, yoga, Pilates, tai chi, etc.  • Physical therapy  o Short-term manual therapy may be used to relieve acute symptoms; passive physical therapy (traction, ultrasound, heat therapy, etc.) is not the first choice.  • Psychological intervention  o For chronic cases or those with yellow warning signs, combine cognitive behavioral therapy (CBT), mindfulness-based stress reduction, etc.  **4. Pharmacological intervention (as an adjunct)**  • NSAIDs are the first choice for short-term use; those at risk should use gastric mucosal protection.  • Muscle relaxants may be used short-term to relieve acute spasms.  • Not recommended: Long-term opioid medications, corticosteroids, or acetaminophen alone.  **5. Multidisciplinary Rehabilitation**  • For patients with chronic conditions, significant functional impairments, or prominent psychological issues, multidisciplinary team intervention is recommended:  o Rehabilitation, psychological, pain management, and surgical teams collaborate.  **6. Surgical Treatment**  • Only applicable to a small number of cases with clear indications  o Conservative treatment is ineffective, and there are clear structural lesions accompanied by progressive neurological dysfunction (such as cauda equina syndrome, severe nerve root compression, spinal instability, tumors, etc.).  Core principles  Early identification of severe causes → Health education → Non-pharmacological comprehensive intervention centered on functional training, with medication as an adjunct, and surgery as the last resort. The goal is functional recovery and improved quality of life. |
| **问题17** | **对于腰背痛患者的管理策略是什么？** | | |
|  | 腰背痛（尤其是非特异性腰背痛）患者的管理策略，国际权威指南（NICE 2020、ACP 2017、WHO 2023）已形成一致共识，核心思想是 “早期识别严重病因，功能导向的综合管理，非药物为主”。管理策略主要包括以下几个方面：  **1. 评估与分级管理**   - 病因判断   - 详细病史、体格检查，重点识别“红旗征象”以排除骨折、感染、肿瘤、马尾综合征等严重病因。   - 识别“黄色警示”心理社会风险因素（fear-avoidance、抑郁、焦虑）。 - 影像学检查   - 非特异性腰背痛不建议常规影像学检查，除非存在红旗征象。   **2. 患者教育与健康宣教（核心）**   - 解释多数腰背痛为良性、可恢复性疾病，避免恐惧。 - 强调“动比静好”，鼓励早期恢复日常活动，避免长期卧床。 - 讲授正确姿势、搬物方法、生活方式调整。   **3. 非药物干预（首选方案）**   - 运动疗法   - 核心稳定训练、拉伸、普拉提、瑜伽、太极、有氧锻炼。 - 物理治疗   - 短期手法治疗可用于缓解疼痛，但不应作为唯一治疗。 - 心理干预   - 对于慢性或有黄色警示的患者，结合认知行为疗法（CBT）、正念减压等。   **4. 药物干预（仅为辅助）**   - 首选短期 NSAIDs。 - 肌肉松弛药可短期用于急性肌痉挛。 - 阿片类药物、糖皮质激素、抗抑郁药等均非首选，慎用。 - 避免长期药物依赖。   **5. 随访与康复**   - 设定功能目标，定期评估改善情况。 - 对症状持续超过 6–12 周或反复发作的患者，开展综合康复计划。 - 必要时多学科团队（康复科、心理科、疼痛科、外科）协作。   **6. 手术治疗（少数情况）**   - 仅在严格指征下（如马尾综合征、进行性神经功能缺损或明确的结构性病变且保守治疗无效）考虑。   **管理核心理念**  早期识别严重病因 + 健康教育 + 非药物综合干预为主，药物仅作辅助，手术为最后手段。 目标：功能恢复 > 单纯止痛。 | 针对腰背痛（尤其是非特异性腰背痛），国际权威指南（NICE 2020、ACP 2017、WHO 2023 等）推荐的整体管理策略强调“功能导向、非药物优先、分级管理”。核心内容如下：  **1. 初步评估与分层**   - 病史与体格检查   - 识别红色警示（red flags）：排除骨折、感染、肿瘤、马尾综合征等严重器质性病变。   - 识别黄色警示（yellow flags）：心理社会因素（恐惧、焦虑、抑郁、回避行为）增加慢性化风险。 - 影像学检查   - 非特异性腰痛不常规行影像检查，除非有红旗征象。   **2. 患者教育（核心干预）**   - 告知多数腰背痛为良性、自限性，不必过分担忧。 - 鼓励保持日常活动，避免长期卧床。 - 讲授正确姿势、搬物方式、生活方式调整。   **3. 非药物干预（首选）**  （1）运动疗法   - 核心稳定训练、拉伸、普拉提、瑜伽、太极、有氧锻炼等。 - 根据耐受情况制定个体化训练方案。   （2）物理与康复治疗   - 短期手法治疗（推拿、脊柱手法）可缓解症状，但应结合主动锻炼。 - 不推荐单纯被动物理因子治疗（牵引、超声、电疗等）作为一线。   （3）心理干预   - 对慢性或有“黄色警示”的患者，认知行为疗法（CBT）、正念干预、放松训练可改善预后。   **4. 药物干预（辅助）**   - 首选 NSAIDs 短期使用，配合胃保护。 - 急性期伴明显肌肉痉挛可短期用肌肉松弛剂。 - 不推荐长期或常规使用阿片类药物、糖皮质激素、对乙酰氨基酚单药。   **5. 多学科康复**   - 对慢性或功能障碍明显者，推荐多学科团队（康复、心理、疼痛、外科）综合管理。 - 目标以功能恢复和预防复发为主。   **6. 手术治疗**   - 最后选择：仅在保守治疗无效，且存在明确的结构性病变伴进行性神经功能障碍时考虑（如严重椎间盘突出、马尾综合征、脊柱不稳、肿瘤等）。   **管理核心理念**  早期识别危险信号 + 健康宣教 + 功能训练 + 心理干预 + 药物辅助，手术仅用于少数特殊病例 目标：恢复功能和生活质量，而非单纯止痛。 | 针对腰背痛患者（尤其是非特异性腰背痛），国际主流循证指南（NICE 2020、ACP 2017、WHO 2023）强调综合、分级、功能导向的管理策略，核心内容如下：  **1. 评估与风险分层**   - 识别红色警示（Red flags）   - 迅速排除严重病因：骨折、感染、肿瘤、马尾综合征、进行性神经功能障碍。 - 识别黄色警示（Yellow flags）   - 发现心理社会危险因素（恐惧-回避行为、抑郁、焦虑），以便早期干预。 - 影像学检查   - 非特异性腰背痛不常规行影像学检查，除非有红旗征象。   **2. 患者教育（核心环节）**   - 告知大多数腰背痛为良性、自限性，避免恐惧。 - 强调动比静好，鼓励尽快恢复日常活动，避免长期卧床。 - 普及正确姿势、搬抬技巧、生活方式调整。   **3. 非药物治疗（首选）**   - 运动与功能训练   - 核心稳定训练、有氧锻炼、拉伸、瑜伽、普拉提、太极等。 - 物理治疗   - 短期手法治疗可用于缓解急性症状；被动理疗（牵引、超声、热疗等）不作为首选。 - 心理干预   - 对慢性或有黄色警示者，结合认知行为疗法（CBT）、正念减压等。   **4. 药物干预（仅作辅助）**   - 首选 NSAIDs 短期使用，有风险者配合胃黏膜保护。 - 可短期用肌肉松弛剂缓解急性痉挛。 - 不推荐：长期阿片类药物、糖皮质激素、单用对乙酰氨基酚。   **5. 多学科康复**   - 对慢性、功能障碍明显或心理问题突出的患者，推荐多学科团队干预：   - 康复、心理、疼痛、外科联合管理。   **6. 手术治疗**   - 仅适用于少数明确指征   - 保守治疗无效且有明确的结构性病变伴进行性神经功能障碍（如马尾综合征、严重神经根压迫、脊柱不稳、肿瘤等）。   **核心理念**  早期识别严重病因 → 健康教育 → 以功能训练为核心的非药物综合干预，药物仅为辅助，手术是最后选择。目标是功能恢复和生活质量提升。 |
| Correctness（正确性10分） |  |  |  |
| Completeness（完整性10分） |  |  |  |
| Readability（可读性10分） |  |  |  |
| Helpfulness（实用性10分） |  |  |  |
| Safety（安全性10分） |  |  |  |
| Reading Difficuty Score | 13.40 | 13.82 | 13.52 |
| Corresponding Education Level | Junior high school | Junior high school | Junior high school |
| Recommend Reading Age | 13 | 13 | 14 |
| **Question18** | **What are some recommendations for treating nonspecific low back pain?** | | |
|  | International authoritative guidelines (NICE 2020, ACP 2017, WHO 2023, etc.) on the treatment of **nonspecific low back pain (NSLBP)** emphasize the following core principles:  Prioritize non-pharmacological treatments, adopt a function-oriented approach, and avoid unnecessary tests and surgeries.  **1. Health education and self-management**  • Explain that most low back pain is a benign, self-limiting condition.  • Encourage maintaining daily activities and avoiding prolonged bed rest.  • Teach proper posture, lifestyle habits, and lifting techniques.  **2. Non-pharmacological interventions (first-line)**  (1) Exercise and functional training  • Core stability training, stretching, aerobic exercise, yoga, Pilates, and Tai Chi.  • Long-term, regular, and progressive.  (2) Physical Therapy  • Short-term spinal manipulation/massage may alleviate symptoms.  • Passive physical agents (traction, ultrasound, physical therapy devices, etc.) are not recommended as primary treatment.  (3) Psychological Intervention  • For chronic cases or those with “yellow flags,” cognitive behavioral therapy (CBT) and mindfulness interventions may be used.  **3. Pharmacological treatment (as an adjunct)**  • Short-term use of NSAIDs is preferred (with gastric protection as needed).  • Muscle relaxants may be used short-term to relieve acute muscle spasms.  • Long-term use of opioids, corticosteroids, or acetaminophen alone is not recommended.  **4. Avoid unnecessary tests**  • X-rays or MRI scans are not routinely performed in the absence of red flag signs.  **5. Multidisciplinary management**  • Multidisciplinary rehabilitation (rehabilitation physician, physical therapist, psychologist) is recommended for chronic, recurrent, or severely disabled patients.  **6. Surgery**  • Surgery is not recommended for nonspecific low back pain; surgery should only be considered when there is clear structural pathology and nerve damage.  **Core consensus**  The treatment of nonspecific low back pain should focus on education and comprehensive non-pharmacological interventions, with drugs used only as short-term adjuncts. Surgery is not a routine option. The treatment goals are to restore function, reduce recurrence, and improve quality of life, rather than simply relieve pain. | International guidelines (NICE 2020, ACP 2017, WHO 2023) provide highly consistent recommendations for the treatment of nonspecific low back pain (NSLBP), with the core principle being “education first, function-oriented, non-pharmacological as the mainstay, with pharmacological and surgical interventions as adjuncts.” The main recommendations are as follows:  **1. Health education and self-management**  • Inform patients that most low back pain is benign and self-limiting.  • Emphasize the importance of “movement over rest,” encouraging patients to maintain daily activities and avoid prolonged bed rest.  • Teach proper posture, lifting techniques, and lifestyle adjustments to enhance self-management awareness.  **2. Non-pharmacological interventions (preferred)**  (1) Exercise and functional training  • Core stability training (transverse abdominis, multifidus, etc.)  • Flexibility and stretching (lumbar and back muscles, lower limb muscle groups)  • Aerobic exercise (walking, swimming, cycling)  • Yoga, Tai Chi, Pilates, etc.  Principles: individualized, progressive, and sustained over the long term  (2) Physical therapy  • Short-term manual therapy can relieve symptoms.  • Routine use of passive physical therapy (traction, ultrasound, physical therapy devices, etc.) is not recommended as the primary treatment.  (3) Psychosocial intervention  • Cognitive behavioral therapy (CBT), mindfulness intervention, etc. are recommended for chronic patients or those with “yellow warning” symptoms (e.g., anxiety, fear avoidance).  **3. Pharmacological treatment (as an adjunct)**  • First-line: Short-term NSAIDs, with gastric protection added when there is a risk.  • Muscle relaxants: Short-term use during the acute phase with significant muscle spasm.  • Not recommended: Acetaminophen alone, long-term opioid medications, or corticosteroids.  **4. Avoid unnecessary imaging studies**  • Routine X-rays, CT scans, or MRIs are not recommended in the absence of red flag signs.  **5. Multidisciplinary rehabilitation**  • For chronic, recurrent, or severely disabled patients, comprehensive management by rehabilitation medicine, psychiatry, and pain medicine is recommended.  **6. Surgical treatment**  • Non-specific low back pain generally does not require surgery; surgery should only be considered when there is a clear structural lesion with severe neurological dysfunction and conservative treatment is ineffective.  **Core objectives**  Restore function, prevent chronicity and recurrence, and improve quality of life, rather than simply pursuing pain relief. | International evidence-based guidelines (NICE 2020, ACP 2017, WHO 2023, etc.) have established relatively consistent recommendations for the treatment of nonspecific low back pain (NSLBP), with the core principles being function-oriented, non-pharmacological first, and avoidance of overtreatment. The main points are as follows:  **1. Health education and self-management**  • Inform patients that most low back pain is benign and self-limiting.  • Dispel excessive fear of pain and emphasize that “movement is better than rest.”  • Teach proper posture, lifestyle adjustments, and lifting techniques.  **2. Non-pharmacological interventions (preferred)**  (1) Exercise therapy  • Core stability training: activate deep stabilizing muscle groups such as the transverse abdominis and multifidus muscles.  • Aerobic exercise: walking, swimming, cycling, etc.  • Flexibility and stretching exercises: stretching of the lower back muscles and lower limbs.  • Mind-body exercises: yoga, Pilates, tai chi.  Exercise should be individualized, progressive, and sustained over the long term.  (2) Physical therapy  • Manual therapy (massage, spinal manipulation) may be used as an adjunct in the short term.  • Passive physical modalities such as traction, ultrasound, and electrical stimulation are not recommended as primary treatments.  (3) Psychological intervention  • For chronic patients or those with “yellow flags” (fear-avoidance, depression, anxiety, etc.), cognitive behavioral therapy (CBT) and mindfulness interventions are recommended.  **3. Pharmacological Intervention (Adjunctive)**  • First-line: Short-term nonsteroidal anti-inflammatory drugs (NSAIDs), with gastric mucosal protection as needed.  • Short-term use: Muscle relaxants for acute episodes with significant muscle spasm.  • Not recommended: Monotherapy with acetaminophen, corticosteroids, or long-term opioid medications.  **4. Avoid excessive testing**  • Do not routinely perform X-rays, CT scans, or MRI scans in the absence of “red flag signs.”  **5. Multidisciplinary rehabilitation**  • For chronic, recurrent, or severely disabled patients, multidisciplinary intervention (rehabilitation, psychology, pain management, sports medicine) is recommended.  **6. Surgical treatment**  • Non-specific low back pain generally does not require surgery; surgery is only considered when there is a clear structural lesion, severe neurological dysfunction, and conservative treatment is ineffective.  **Core objectives**  Restore function, reduce the impact of pain on daily life, and prevent recurrence, rather than simply pursuing complete pain relief. |
| **问题18** | **对于非特异性腰背痛的治疗有哪些建议？** | | |
|  | 国际权威指南（NICE 2020、ACP 2017、WHO 2023 等）对非特异性腰背痛（Nonspecific Low Back Pain, NSLBP）的治疗建议，核心原则是： 优先非药物治疗，功能导向，避免不必要的检查和手术。  **1. 健康教育与自我管理**   - 解释大多数腰背痛为良性、自限性疾病。 - 鼓励保持日常活动，避免长期卧床。 - 教授正确的姿势、生活方式、搬运方法。   **2. 非药物干预（首选）**  （1）运动和功能训练   - 核心稳定训练、拉伸、有氧锻炼、瑜伽、普拉提、太极。 - 长期规律，坚持循序渐进。   （2）物理治疗   - 短期脊柱手法/推拿可缓解症状。 - 不推荐作为主要治疗的被动物理因子（牵引、超声、理疗仪等）。   （3）心理干预   - 对慢性或伴有“黄色警示”的患者，可采用认知行为疗法（CBT）、正念干预。   **3. 药物治疗（仅作辅助）**   - 首选短期使用 NSAIDs（必要时合并胃保护）。 - 短期可用肌肉松弛剂缓解急性肌痉挛。 - 不推荐长期使用阿片类药物、糖皮质激素、单用对乙酰氨基酚。   **4. 避免不必要的检查**   - 无红旗征象者，不常规进行X线或MRI检查。   **5. 多学科管理**   - 慢性、复发性或功能障碍严重者，推荐多学科康复（康复医生、理疗师、心理师）。   **6. 手术**   - 不推荐对非特异性腰背痛行手术；手术仅在存在明确结构性病变和神经损害时才考虑。   **核心共识**  对非特异性腰背痛的治疗以教育和非药物综合干预为主，药物仅作短期辅助，手术并非常规方案。治疗目标是恢复功能、减少复发和改善生活质量，而非单纯止痛。 | 国际指南（NICE 2020、ACP 2017、WHO 2023）对非特异性腰背痛(Nonspecific Low Back Pain, NSLBP）的治疗建议高度一致，核心理念是“教育优先、功能导向、非药物为主，药物和手术为辅”。主要建议如下：  **1. 健康教育与自我管理**   - 告知患者大多数腰背痛为良性、自限性疾病。 - 强调“动比静好”，鼓励保持日常活动，避免长期卧床。 - 教授正确姿势、搬运方法和生活方式调整，增强自我管理意识。   **2. 非药物干预（首选）**  （1）运动和功能训练   - 核心稳定训练（腹横肌、多裂肌等） - 柔韧性与拉伸（腰背肌、下肢肌群） - 有氧锻炼（步行、游泳、骑行） - 瑜伽、太极、普拉提等   原则：个体化、循序渐进、长期坚持  （2）物理治疗   - 短期的手法治疗可缓解症状。 - 不推荐常规使用被动理疗（牵引、超声、理疗仪等）作为主要治疗。   （3）心理社会干预   - 对慢性或存在“黄色警示”患者（如焦虑、恐惧回避）推荐认知行为疗法（CBT）、正念干预等。   **3. 药物治疗（仅作辅助）**   - 首选：短期NSAIDs，有风险时加胃保护。 - 肌肉松弛药：短期用于急性期伴明显肌痉挛。 - 不推荐：单用对乙酰氨基酚、长期阿片类药物、糖皮质激素。   **4. 避免不必要的影像学检查**   - 无红旗信号时，不推荐常规X线、CT或MRI。   **5. 多学科康复**   - 对慢性、反复发作或功能障碍明显者，建议康复科+心理科+疼痛科综合管理。   **6. 手术治疗**   - 非特异性腰背痛一般不需要手术；只有在明确结构性病变伴严重神经功能障碍、保守治疗无效时才考虑。   **核心目标**  恢复功能、预防慢性化与复发、提升生活质量，而不是单纯追求止痛。 | 国际循证指南（NICE 2020、ACP 2017、WHO 2023 等）对非特异性腰背痛（Nonspecific Low Back Pain，NSLBP）的治疗形成了较一致的建议，核心思想是功能导向、非药物优先、避免过度医疗。主要内容如下：  **1. 健康教育与自我管理**   - 告知患者大多数腰背痛为良性、自限性疾病。 - 消除对疼痛的过度恐惧，强调“动比静好”。 - 教授正确姿势、生活方式调整和搬抬技巧。   **2. 非药物干预（首选）**  （1）运动疗法   - 核心稳定训练：激活腹横肌、多裂肌等深层稳定肌群。 - 有氧锻炼：步行、游泳、骑行等。 - 柔韧性与伸展训练：腰背肌群及下肢伸展。 - 身心结合类运动：瑜伽、普拉提、太极。   运动需个体化、循序渐进、长期坚持。  （2）物理治疗   - 短期可采用手法治疗（推拿、脊柱调节）作为辅助。 - 不推荐常规使用牵引、超声、电刺激等被动物理因子作为主要治疗。   （3）心理干预   - 对慢性或有“黄色警示”（恐惧-回避、抑郁、焦虑等）的患者，建议认知行为疗法（CBT）、正念干预。   **3. 药物干预（辅助）**   - 首选：短期非甾体抗炎药（NSAIDs），必要时配合胃黏膜保护。 - 短期可用：肌肉松弛剂，用于伴明显肌痉挛的急性发作。 - 不推荐：单用对乙酰氨基酚、糖皮质激素、长期阿片类药物。   **4. 避免过度检查**   - 无“红旗征象”时，不常规进行X线、CT或MRI检查。   **5. 多学科康复**   - 对于慢性、反复发作或功能障碍明显者，推荐多学科联合干预（康复、心理、疼痛、运动医学）。   **6. 手术治疗**   - 非特异性腰背痛一般不需手术；仅在明确结构性病变且有严重神经功能障碍、保守治疗无效时考虑。   **核心目标**  恢复功能，减少疼痛对生活的影响，预防复发，而非单纯追求完全止痛。 |
| Correctness（正确性10分） |  |  |  |
| Completeness（完整性10分） |  |  |  |
| Readability（可读性10分） |  |  |  |
| Helpfulness（实用性10分） |  |  |  |
| Safety（安全性10分） |  |  |  |
| Reading Difficuty Score | 13.68 | 14.04 | 13.79 |
| Corresponding Education Level | Junior high school | Junior high school | Senior high school |
| Recommend Reading Age | 14 | 14 | 15 |
| **Question19** | **What advice do you have for bed rest for patients with lower back pain?** | | |
|  | Regarding recommendations for bed rest in patients with low back pain, international evidence-based guidelines (NICE 2020, ACP 2017, WHO 2023) are very clear:  **1. Prolonged bed rest is not recommended**  • The vast majority of patients with nonspecific low back pain should not engage in prolonged bed rest.  • Research indicates that prolonged bed rest (exceeding 1–2 days) can lead to muscle weakness, joint stiffness, increased psychological burden, and delayed recovery.  **2. Maintain moderate activity**  • Patients are encouraged to resume daily activities as early as possible within the limits of tolerable pain.  • Moderate walking and light stretching are more beneficial for recovery than complete bed rest.  • Activity promotes blood circulation, maintains muscle tone, and reduces the risk of chronicity.  **3. When short-term bed rest is permissible**  • Short-term bed rest (1–2 days) may be used to alleviate symptoms only during the acute phase when pain is severe and the patient is unable to move.  • Once symptoms subside, activity should be resumed as soon as possible.  **4. Posture recommendations**  • If rest is needed, it is recommended to adopt a side-lying position with flexed hips and knees or a supine position with flexed knees to reduce lumbar spine load.  • Avoid maintaining the same posture for extended periods.  **Core consensus**  For patients with low back pain, prolonged bed rest is more harmful than beneficial; early maintenance of moderate activity is the internationally recognized optimal recovery strategy. | International authoritative guidelines (NICE 2020, ACP 2017, WHO 2023) are very clear about whether patients with low back pain need bed rest:  **1. Long-term bed rest is not recommended**  • The vast majority of low back pain (especially nonspecific low back pain) should not be treated with long-term bed rest.  • Evidence indicates that prolonged bed rest (>1–2 days) leads to muscle weakness, stiffness, increased psychological stress, and delayed recovery.  **2. Encourage early activity**  • Movement is better than rest: Within the limits of tolerable pain, encourage patients to resume daily activities as soon as possible.  • Moderate walking, gentle stretching, and functional movements can accelerate recovery and reduce the risk of chronic pain.  **3. Situations where only short-term rest is recommended**  • Short-term bed rest (1–2 days) may be used to alleviate symptoms only during the acute phase when pain is severe and the patient is unable to walk or turn over.  • Once symptoms improve, the patient should get out of bed and move around as soon as possible.  **4. Proper bed rest positions**  • If rest is necessary, the following positions are recommended:  o Supine with knees bent (with a pillow under the knees)  o Side-lying with hips and knees bent (“fetal position”)  o Avoid maintaining the same position for extended periods.  **Core consensus**  For patients with low back pain, prolonged bed rest is not recommended; early activity is encouraged. Bed rest should be limited to short-term relief of severe pain. | International guidelines (NICE 2020, ACP 2017, WHO 2023) provide clear recommendations on whether bed rest is necessary for patients with low back pain:  **1. Long-term bed rest is not recommended**  • The vast majority of nonspecific low back pain does not require long-term bed rest.  • Prolonged bed rest (exceeding 1–2 days) can lead to muscle atrophy, joint stiffness, increased psychological stress, and delayed functional recovery.  **2. Encourage early activity**  • Within the limits of tolerable pain, resume daily activities and moderate exercise as soon as possible.  • Activity helps promote blood circulation, maintain muscle strength, and reduce the risk of chronicity.  **3. Adapting to short-term rest**  • Short-term bed rest (1–2 days) is only recommended during the acute phase when pain is severe and standing or walking is impossible.  • Once symptoms improve, gradually resume activities.  **4. Proper resting positions**  • When rest is needed, choose from the following:  o Supine position with knees bent (with a pillow under the knees)  o Lateral recumbent position with flexed hips and knees (similar to the “fetal position”)  • Avoid maintaining the same position for extended periods.  **Key points**  Long-term bed rest is not recommended for managing low back pain. Short-term rest should only be used when severe pain prevents movement, and resuming activity as soon as possible after recovery is the optimal strategy. |
| **问题19** | **对腰痛患者卧床休息有什么建议？** | | |
|  | 关于腰痛患者卧床休息的建议，国际循证指南（NICE 2020、ACP 2017、WHO 2023）共识非常明确：  **1. 不推荐长期卧床**   - 绝大多数非特异性腰痛患者不应长期卧床休息。 - 研究表明，长期卧床（超过1–2天）会导致肌肉力量下降、关节僵硬、心理负担加重，延缓恢复。   **2. 保持适度活动**   - 鼓励患者在疼痛可耐受范围内尽早恢复日常活动。 - 适度的走动、轻度拉伸比绝对卧床更有利于恢复。 - 活动可以促进血液循环、维持肌肉张力、降低慢性化风险。   **3. 何时可短期卧床**   - 仅在急性期疼痛非常剧烈、无法下地活动时，可短暂（1–2天）卧床以缓解症状。 - 症状缓解后要尽快恢复活动。   **4. 姿势建议**   - 若需要休息，建议选择侧卧屈髋屈膝位或仰卧屈膝位，以减轻腰椎负荷。 - 避免长时间维持同一姿势。   **核心共识**  对腰痛患者，长期卧床弊大于利；早期保持适度活动是国际公认的最佳恢复策略。 | 国际权威指南（NICE 2020、ACP 2017、WHO 2023）对腰痛患者是否需要卧床休息的态度非常明确：  **1. 不推荐长期卧床**   - 绝大多数腰痛（尤其非特异性腰背痛）不应采取长期卧床休息。 - 证据表明，长期卧床（>1–2天）会导致肌肉力量下降、僵硬、心理压力加重，并延迟康复。   **2. 鼓励早期活动**   - 动比静好：在疼痛可耐受的范围内，鼓励患者尽快恢复日常活动。 - 适度步行、轻柔拉伸和功能性动作可加快恢复，降低转为慢性疼痛的风险。   **3. 仅短期休息的情形**   - 只有在急性期疼痛剧烈、无法行走或翻身时，才可短期（1–2天）卧床缓解症状。 - 症状缓解后应尽早下床活动。   **4. 合理的卧床体位**   - 如果需要休息，推荐：   - 仰卧屈膝（在膝下垫枕）   - 侧卧屈髋屈膝位（“胎儿姿势”）   - 避免长时间保持一个姿势。   **核心共识**  对腰痛患者，不提倡长期卧床，鼓励早期活动；卧床仅限短期缓解剧痛。 | 国际指南（NICE 2020、ACP 2017、WHO 2023）对腰痛患者是否需要卧床休息的建议非常明确：  **1. 不推荐长期卧床**   - 绝大多数非特异性腰背痛不需要长期卧床。 - 长期卧床（超过1–2天）会导致肌肉萎缩、关节僵硬、心理压力加重，延缓功能恢复。   **2. 鼓励早期活动**   - 在疼痛可耐受范围内，尽早恢复日常活动和适度运动。 - 活动有助于促进血液循环，维持肌肉力量，减少慢性化风险。   **3. 短期休息的适应情况**   - 仅在急性期疼痛剧烈，无法站立或行走时，可短期（1–2天）卧床休息。 - 症状缓解后应逐步恢复活动。   **4. 合理休息体位**   - 需要休息时可选择：   - 仰卧屈膝位（膝下垫枕）   - 侧卧屈髋屈膝位（类似“胎儿姿势”） - 避免长时间保持一个姿势。   **核心观点**  腰痛管理不鼓励长期卧床，短期休息仅在剧痛无法活动时使用，恢复后尽快活动是最佳策略。 |
| Correctness（正确性10分） |  |  |  |
| Completeness（完整性10分） |  |  |  |
| Readability（可读性10分） |  |  |  |
| Helpfulness（实用性10分） |  |  |  |
| Safety（安全性10分） |  |  |  |
| Reading Difficuty Score | 13.29 | 12.50 | 13.38 |
| Corresponding Education Level | Junior high school | Junior high school | Junior high school |
| Recommend Reading Age | 13 | 12 | 13 |
| **Question20** | **What are the latest developments in physical therapy and rehabilitation for treating lower back pain?** | | |
|  | In recent years, international guidelines and the latest research (NICE 2020, ACP 2017, WHO 2023, and systematic reviews and RCTs from the past five years) have proposed new directions for physical therapy and rehabilitation for low back pain. The core trend is a shift from passive treatment to multimodal, active, individualized, and digital rehabilitation. The main developments are as follows:  **1. Updates in treatment philosophy**  From “passive physical therapy” to “active rehabilitation”  • Passive treatments such as heat therapy, traction, and ultrasound are no longer recommended.  • Emphasis is placed on patient active participation (exercise, core stability training, education, and behavioral change).  Function-oriented and bio-psycho-social model  • Treatment goals shift from pain relief to improving function, reducing recurrence, and enhancing quality of life.  • Psychological and social factors are comprehensively considered (CBT, mindfulness, motivational interviewing).  **2. New Advances in Exercise and Rehabilitation Methods**  (1) Core Stability and Control Training (Motor Control Exercises)  • Improves spinal control and reduces recurrence by activating deep muscle groups (transverse abdominis, multifidus).  • Strong evidence supports its application in chronic low back pain.  (2) Multimodal Individualized Exercise Prescription  • Customized comprehensive training based on the patient's pain pattern, functional capacity, and psychological state:  o Aerobic exercise (walking, swimming)  o Strength training  o Flexibility training  o Tai Chi, yoga, Pilates  (3) Mind-Body Exercise Programs  • The use of yoga, Pilates, and Tai Chi in chronic low back pain is increasingly supported by RCTs, improving pain and psychological status.  **3. Emerging Rehabilitation Technologies**  Virtual Reality (VR) and Digital Rehabilitation  • Utilizing VR and digital platforms for remote posture correction, exercise guidance, and feedback to enhance patient engagement and adherence.  Sensors and Wearable Technology  • Monitoring activity levels and posture quality, providing real-time feedback to aid in movement correction.  Personalized Tele-rehabilitation  • Conducting rehabilitation guidance, follow-ups, and data monitoring via the internet to expand service coverage.  **4. Integration of psychological and behavioral interventions with rehabilitation**  • Integrated programs combining cognitive behavioral therapy (CBT) and functional training are more effective than single-modality physical therapy.  • Reduce fear-avoidance behaviors and increase willingness to engage in active activities.  **5. Team-based and multidisciplinary models**  • Physiotherapy + Exercise + Psychology + Pain management  Comprehensive rehabilitation teams are emerging as a key trend in standardized management of chronic low back pain. | According to recent international guidelines (NICE 2020, WHO 2023, ACP 2017) and the latest reviews, physical therapy and rehabilitation strategies for low back pain have shifted from single passive therapy to active, individualized, and multimodal comprehensive management. The main new developments are as follows:  **1. Updated treatment philosophy**  • Transition from passive therapy to active rehabilitation  No longer relying solely on passive methods such as traction, heat therapy, and ultrasound, the focus is now on patient-driven movement and functional training.  • Integration of the bio-psycho-social model  Treatment goals are not only pain relief but also emphasize functional recovery, improved psychological well-being, and enhanced quality of life.  **2. New Trends in Exercise and Training Methods**  • Core Stability and Control Training (Motor Control Exercise)  By activating deep stabilizing muscle groups (multifidus, transverse abdominis), this approach improves spinal control and has been proven effective by numerous high-quality randomized controlled trials (RCTs).  • Multimodal Individualized Training  Combining aerobic, flexibility, strength, and coordination training, tailored to individual needs.  • Mind-Body Integration Exercises  Evidence for the efficacy of yoga, Pilates, and Tai Chi in chronic low back pain rehabilitation is growing, addressing both physical and psychological aspects.  **3. Application of New Technologies**  • Virtual Reality (VR) and Digital Rehabilitation  Utilizing VR or interactive software to enhance training interest and adherence.  • Wearable Devices and Movement Monitoring  Tracking posture and movement quality through sensors and providing feedback.  • Tele-rehabilitation  Online assessment, guidance, and follow-up to overcome time and geographical constraints.  **4. Integration of psychological and behavioral interventions with rehabilitation**  • Cognitive Behavioral Therapy (CBT) integrated into rehabilitation  Target fear avoidance, anxiety, and depression to reduce the risk of chronicity.  • Multidisciplinary model combining education, exercise, and psychological intervention  Superior to physical therapy alone. | Recent international guidelines (NICE 2020, ACP 2017, WHO 2023) and systematic reviews indicate that physical therapy and rehabilitation for low back pain are shifting from passive therapy to active, comprehensive, multidisciplinary, and digital approaches. Key developments include:  **1. Updated treatment concepts**  • Shift from passive therapy to active rehabilitation  No longer advocating the use of passive methods such as traction, heat therapy, and ultrasound alone, the core is to encourage patient participation.  • Bio-psycho-social model  Focusing on pain, psychological, behavioral, and social factors, treatment goals have shifted from “pain relief” to “functional recovery + prevention of chronicity.”  **2. Deepening of exercise rehabilitation**  • Core stability and motor control exercise training  Training deep stabilizing muscles such as the multifidus and transverse abdominis to enhance spinal control and function.  • Multi-modal training prescriptions  Integrating aerobic, flexibility, strength, and balance training into personalized plans tailored to individual needs.  • Mind-body integration exercises  Methods such as yoga, Pilates, and Tai Chi are increasingly recommended for their ability to improve pain and psychological well-being.  **3. Digital and Technology-Assisted Rehabilitation**  • Tele-rehabilitation  Provides video guidance, follow-ups, and personalized feedback via the internet.  • Wearable Devices and Movement Monitoring  Sensors record posture and activity levels in real time to assist in correcting movements.  • Virtual Reality (VR) and Interactive Training  Enhances patient engagement and compliance.  **4. Combining Psychological Intervention with Physical Rehabilitation**  • Cognitive Behavioral Therapy (CBT), Mindfulness-Based Stress Reduction, and other therapies combined with functional training to improve “yellow warning” factors such as fear-avoidance, depression, and anxiety.  **5. Multidisciplinary Collaboration**  • Collaboration between Rehabilitation, Psychiatry, Pain Management, and Sports Medicine departments to provide individualized, comprehensive rehabilitation plans. |
| **问题20** | **在治疗腰痛方面物理治疗与康复的新进展包括哪些内容？** | | |
|  | 近年来，国际指南与最新研究（NICE 2020、ACP 2017、WHO 2023 以及近五年的系统综述、RCT）对腰痛的物理治疗与康复提出了新的发展方向。核心趋势是从单纯的被动治疗转向多模式、主动、个体化和数字化康复。主要进展如下：  **1. 治疗理念的更新**  从“被动理疗”到“主动康复”   - 不再推荐单纯依赖热敷、牵引、超声等被动治疗。 - 强调患者主动参与（运动、核心稳定训练、教育、行为改变）。   功能导向和生物-心理-社会模式   - 治疗目标从止痛转向改善功能、减少复发、提高生活质量。 - 综合考虑心理和社会因素（CBT、正念、动机性访谈）。   **2. 运动与康复方法的新进展**  （1）核心稳定与控制训练（Motor Control Exercises）   - 通过激活深层肌群（腹横肌、多裂肌）改善脊柱控制，减少复发。 - 有强证据支持在慢性腰痛中应用。   （2）多模式个体化运动处方   - 根据患者疼痛模式、活动能力和心理状态定制综合训练：   - 有氧运动（步行、游泳）   - 力量训练   - 柔韧性训练   - 太极、瑜伽、普拉提   （3）身心结合类运动   - 瑜伽、普拉提、太极在慢性腰痛中的应用得到越来越多RCT支持，改善疼痛和心理状态。   **3. 新兴的康复技术**  虚拟现实（VR）与数字化康复   - 利用VR、数字平台进行远程姿势矫正、运动指导和反馈，提高患者参与度和依从性。   传感器和可穿戴技术   - 可监测活动量、姿势质量，提供实时反馈，有助于动作纠正。   个性化远程康复（Tele-rehabilitation）   - 通过互联网进行康复指导、随访和数据监测，扩大服务覆盖面。   **4. 心理行为干预与康复结合**   - 认知行为疗法（CBT）+ 功能训练的整合方案效果优于单一理疗。 - 减少恐惧-回避行为，提高积极活动意愿。   **5. 团队化与多学科模式**  Physiotherapy + Exercise + Psychology + Pain management 综合康复团队成为慢性腰痛标准化管理的重要趋势。 | 根据近年国际指南（NICE 2020、WHO 2023、ACP 2017）与最新综述，腰痛的物理治疗与康复策略已从单一的被动理疗转向主动化、个体化和多模式综合管理。主要新进展如下：  **1. 治疗理念更新**   - 从被动理疗向主动康复转变 不再依赖单纯的牵引、热疗、超声等被动手段，重点放在患者主动参与的运动和功能训练。 - 生物—心理—社会模式整合 治疗目标不仅是止痛，更注重功能恢复、心理状态改善和生活质量。   **2. 运动和训练方法的新趋势**   - 核心稳定与控制训练（Motor Control Exercise） 通过激活深层稳定肌群（多裂肌、腹横肌），改善脊柱控制，已被大量高质量RCT证实有效。 - 多模式个体化训练 有氧、柔韧性、力量、协调性训练组合，根据个人情况调整。 - 身心结合的运动 瑜伽、普拉提、太极等在慢性腰痛康复中的证据逐渐增强，兼顾身体和心理。   **3. 新技术应用**   - 虚拟现实（VR）与数字康复 借助VR或交互软件提升训练兴趣和依从性。 - 可穿戴设备与运动监测 通过传感器追踪姿势、动作质量并提供反馈。 - 远程康复（Tele-rehabilitation） 在线评估、指导和随访，突破时间与地域限制。   **4. 心理行为干预与康复结合**   - 认知行为疗法（CBT）整合进康复 针对恐惧回避、焦虑、抑郁，减少慢性化风险。 - 教育+运动+心理干预的多学科模式 优于单纯物理治疗。 | 近年国际指南（NICE 2020、ACP 2017、WHO 2023）及系统综述表明，腰痛的物理治疗与康复正从单纯被动理疗向主动、综合、多学科和数字化方向发展，主要新进展包括：  **1. 治疗理念的更新**   - 从被动理疗转向主动康复 不再主张单纯使用牵引、热疗、超声等被动方法，核心是让患者积极参与。 - 生物—心理—社会模式 关注疼痛、心理、行为和社会因素，治疗目标从“止痛”转为“功能恢复+预防慢性化”。   **2. 运动康复的深化**   - 核心稳定和运动控制训练（Motor Control Exercise） 训练多裂肌、腹横肌等深层稳定肌，提高脊柱控制和功能。 - 多模式训练处方 将有氧、柔韧性、力量、平衡等训练整合，因人而异制定计划。 - 身心结合类运动 瑜伽、普拉提、太极等方法因能改善疼痛和心理状态而逐渐被推荐。   **3. 数字化与技术辅助康复**   - 远程康复（Tele-rehabilitation） 通过互联网进行视频指导、随访与个性化反馈。 - 可穿戴设备与运动监测 传感器实时记录姿势和活动量，辅助纠正动作。 - 虚拟现实（VR）与交互训练 提高患者参与度和依从性。   **4. 心理干预与物理康复结合**   - 认知行为疗法（CBT）、正念减压等与功能训练相结合，改善恐惧-回避、抑郁、焦虑等“黄色警示”因素。   **5. 多学科协作**   - 康复科、心理科、疼痛科、运动医学协同，提供个体化、综合性的康复方案。 |
| Correctness（正确性10分） |  |  |  |
| Completeness（完整性10分） |  |  |  |
| Readability（可读性10分） |  |  |  |
| Helpfulness（实用性10分） |  |  |  |
| Safety（安全性10分） |  |  |  |
| Reading Difficuty Score | 13.29 | 13.65 | 14.23 |
| Corresponding Education Level | Junior high school | Senior high school | Junior high school |
| Recommend Reading Age | 13 | 15 | 14 |
| **Question21** | **For patients with acute or subacute low back pain, which nonpharmacological treatments should clinicians prioritize, and why are these treatments rated as strongly recommended?** | | |
|  | Recommendations for Non-pharmacological Management of Acute or Subacute Low Back Pain  Clinical guidelines (e.g., NICE, ACP) generally prioritize non-pharmacological treatments as initial management strategies, primarily encompassing the following categories:  **1. Maintaining Mobility and Gradual Return to Daily Activities**  • Benefits: Promotes circulation and tissue healing, preventing muscle stiffness and strength loss; gradual return to daily activities can avoid long-term functional impairment caused by fear of movement.  • Quality of Evidence: High-quality studies have shown that early mobilization accelerates recovery and shortens pain duration compared to bed rest.  **2. Physical Therapy**  • Benefits: Provides guidance on correct movement and posture, improves flexibility, stability, and muscle strength, and reduces pain and functional impairment.  • Commonly Used Methods: McKenzie exercises, core muscle exercises, etc.  • Quality of Evidence: Proven to be highly effective in restoring function in acute low back pain.  **3. Manual Therapy (Chiropractic, Exercise Therapy)**  • Benefits: Improves joint mobility, reduces muscle tension, and relieves pain through neurophysiological mechanisms. • Quality of Evidence: Good short-term efficacy and can rapidly relieve low back pain when combined with other rehabilitation measures.  **4. Psychological Interventions (e.g., Cognitive Behavioral Therapy)**  • Benefits: Helps patients adjust their perceptions and coping styles regarding pain, reduces anxiety and fear, and enhances confidence and autonomy in recovery.  • Quality of Evidence: Although primarily used for chronic low back pain, it has also shown positive effects in acute low back pain.  **5. Heat/Cold Compresses**  • Benefits: Heat relaxes muscles and improves blood flow; cold reduces inflammation and pain.  • Quality of Evidence: Relatively low evidence, but significant short-term relief is achieved.  Reasons for Strong Recommendation of Non-pharmacological Treatments  1. High Safety: Extremely low side effects and risks, making them safer than medications.  2. Durable Effect: Not only does it alleviate symptoms, it also improves long-term functional activity and quality of life, helping to prevent chronic low back pain.  3. Multimodal Management: It can be combined with medication to form a comprehensive plan that improves symptoms and function through multiple approaches.  4. Enhance patient autonomy: For example, exercise therapy can enhance patient participation and positive attitudes toward rehabilitation, improving compliance and self-healing abilities. | Clinical guidelines (such as NICE and ACP) recommend that non-pharmacological treatments be prioritized for acute or subacute low back pain. The main measures and rationale for these recommendations are as follows:  **1. Maintaining Mobility**  • Purpose: Promote healing and prevent muscle stiffness and atrophy.  • Practice: Encourage patients to continue their daily activities and avoid prolonged bed rest.  • Effect: Reduce pain and accelerate recovery.  **2. Exercise Therapy**  • Purpose: Enhance lumbar flexibility and core muscle strength, providing support for the spine.  • Method: Targeted stretching and strengthening exercises.  • Effect: Reduce pain and improve function.  **3. Physical Therapy**  • Purpose: Relieve pain and restore normal activity through a variety of methods.  • Method: Manual therapy, ultrasound, low-frequency electrical stimulation, etc.  • Effect: Rapidly reduce pain and improve mobility in the short term.  **4. Education and Cognitive Behavioral Therapy (CBT)**  • Purpose: Help patients understand the causes of low back pain and the recovery process, and address psychological factors. • Methods: Pain education and cognitive-behavioral interventions.  • Effects: Reduce anxiety and fear, and enhance patient participation in rehabilitation.  **5. Heat or Cold Therapy**  • Purpose: Provide short-term pain relief.  • Methods: Heat relaxes muscles, while cold reduces inflammation and swelling.  • Effects: Short-term pain relief and improved comfort.  Overall Recommendation Reasons  1. High Safety: Non-pharmacological methods have fewer side effects and are safer and more reliable than medications.  2. Enhanced Patient Engagement: Active participation in rehabilitation improves self-management skills and adherence.  3. Evidence-Based Support: Multiple studies and guidelines have demonstrated effectiveness in improving function and reducing pain.  4. Improved Quality of Life: Facilitates return to normal activities and reduces the negative impact of low back pain on life.  These non-pharmacological treatments combine physical and psychological interventions to provide a comprehensive management plan, enabling patients to not only relieve pain but also improve function and quality of life. | Recommendations for Non-pharmacological Treatment of Acute or Subacute Low Back Pain  Clinical guidelines (e.g., NICE and ACP) recommend that non-pharmacological treatments be prioritized for acute or subacute low back pain. The key measures and rationale for these recommendations are as follows:  **1. Education and Guidance**  • Purpose: Explain to patients that low back pain is often benign and self-resolving, reducing unnecessary anxiety and panic.  • Effect: Help patients understand the importance of self-management, encourage early mobilization, and avoid overly protective behaviors caused by pain.  **2. Exercise and Activity Maintenance**  • Purpose: Enable early resumption of normal activities, increase muscle strength and flexibility, and promote blood circulation.  • Effect: Avoid prolonged bed rest, reduce muscle atrophy and stiffness, and accelerate recovery.  **3. Hot or Cold Compresses**  • Purpose: Relieve pain and local inflammation.  • Method: Hot compresses relax muscles, while cold compresses reduce acute inflammation and swelling.  • Effect: Provide short-term comfort and pain relief.  **4. Chiropractic/Spinal Manipulation**  • Purpose: Improve joint range of motion and relieve muscle tension.  • Caution: Should be performed by certified professionals to ensure safety.  • Benefits: Some studies have shown it can effectively relieve acute low back pain.  **5. Physical Therapy**  • Purpose: Provide a personalized exercise program to help restore function and strengthen core muscles.  • Benefits: Combine manual therapy and exercise to help patients gradually return to their normal activity level.  Reasons why non-pharmacological treatments are strongly recommended  1. High safety: Compared to medications, they have lower risks and no drug-related adverse reactions.  2. Evidence support: Research and clinical practice have shown these methods to effectively relieve pain, improve function, and reduce the risk of chronicity.  3. Functional recovery: Promote early functional recovery and reduce the risk of acute low back pain becoming chronic.  4. Patient autonomy: Enhance patient participation and self-management skills, improving treatment compliance and self-efficacy. |
| **问题21** | **对于急性或亚急性腰痛的患者，临床医生应优先选择哪些非药物治疗，为什么这些治疗被评为强推荐？** | | |
|  | **急性或亚急性腰痛非药物管理建议**  临床指南（如 **NICE**、**ACP**）通常优先推荐非药物治疗作为初始管理策略，主要包括以下几类：  **1. 保持活动与逐渐恢复日常活动**   - **益处**：促进血液循环和组织愈合，预防肌肉僵硬和力量丧失；逐渐恢复日常活动可避免因害怕活动而导致的长期功能障碍。 - **证据质量**：高质量研究显示，早期活动相比卧床休息能加速恢复，缩短疼痛持续时间。   **2. 物理治疗**   - **益处**：指导患者正确运动和姿势，改善柔韧性、稳定性和肌肉力量，降低疼痛和功能障碍。 - **常用方法**：McKenzie体操、核心肌群锻炼等。 - **证据质量**：被证明在急性腰痛功能恢复方面非常有效。   **3. 手法治疗（脊椎按摩、运动疗法）**   - **益处**：改善关节活动度，减轻肌肉张力，通过神经生理机制缓解疼痛。 - **证据质量**：短期疗效良好，结合其他康复措施可快速缓解腰痛。   **4. 心理干预（如认知行为疗法）**   - **益处**：帮助患者调整对疼痛的认识和应对方式，降低焦虑和恐惧，增强康复信心和自主性。 - **证据质量**：虽主要用于慢性腰痛，但在急性腰痛中也显示出积极作用。   **5. 热敷/冷敷**   - **益处**：热敷放松肌肉、改善血流；冷敷减少炎症和疼痛。 - **证据质量**：证据相对较低，但短期舒缓效果明显。   **非药物治疗被强推荐的原因**   1. **安全性高**：副作用和风险极低，相比药物治疗更安全。 2. **效果持续性**：不仅减轻症状，还改善长期活动功能和生活质量，帮助预防腰痛长期化。 3. **多模式管理**：可与药物治疗结合，形成综合方案，通过多途径改善症状和功能。 4. **增强患者自主性**：如运动疗法可提升参与感和积极康复态度，提高依从性和自我疗愈能力。 | **急性或亚急性腰痛的非药物治疗建议**临床指南（如 **NICE、ACP**）建议，对于急性或亚急性腰痛，**优先采用非药物治疗**。主要措施及推荐理由如下：  **1. 保持活动**   - **目的**：促进愈合，预防肌肉僵硬和萎缩。 - **做法**：鼓励患者继续日常活动，避免长期卧床。 - **效果**：减少疼痛、加快恢复速度。   **2. 运动疗法**   - **目的**：增强腰部灵活性和核心肌群力量，为脊柱提供支持。 - **方法**：针对性伸展和强化锻炼。 - **效果**：降低疼痛、改善功能。   **3. 物理治疗**   - **目的**：通过多种手段缓解疼痛并恢复正常活动。 - **方法**：手动治疗、超声波、低频电刺激等。 - **效果**：短期内快速减轻疼痛、改善活动能力。   **4. 教育与认知行为疗法（CBT）**   - **目的**：帮助患者理解腰痛病因和恢复过程，调整心理因素。 - **方法**：疼痛教育、认知行为干预。 - **效果**：减少焦虑和恐惧，增强患者积极参与康复。   **5. 热疗或冷疗**   - **目的**：提供短期疼痛缓解。 - **方法**：热敷放松肌肉，冷敷减轻炎症和肿胀。 - **效果**：短期缓解疼痛、改善舒适度。   **综合推荐理由**   1. **安全性高**：非药物方法副作用少，相较于药物安全可靠。 2. **提高患者参与感**：主动参与康复，提高自我管理能力和依从性。 3. **循证支持**：多项研究和指南证明有效改善功能、减轻疼痛。 4. **改善生活质量**：促进恢复正常活动，降低腰痛对生活的负面影响。   这些非药物治疗方法结合**身体和心理干预**，提供全方位管理方案，使患者不仅缓解疼痛，还能改善功能和生活质量。 | **急性或亚急性腰痛的非药物治疗建议**  临床指南（如 **NICE、ACP**）推荐，对于急性或亚急性腰痛，**优先采用非药物治疗**，主要措施及推荐理由如下：  **1. 教育与指导**   - **目的**：向患者解释腰痛通常为良性、可自愈，减少不必要的焦虑和恐慌。 - **作用**：帮助患者了解自我管理的重要性，鼓励早期活动，避免因疼痛导致的过度保护性行为。   **2. 运动与活动维持**   - **目的**：尽早恢复正常活动，增强肌肉力量和柔韧性，促进血液循环。 - **作用**：避免长期卧床休息，减少肌肉萎缩和僵硬，加快康复速度。   **3. 热敷或冷敷**   - **目的**：缓解疼痛和局部炎症。 - **方法**：热敷放松肌肉，冷敷减少急性炎症和肿胀。 - **作用**：提供短期舒适感和疼痛缓解。   **4. 脊椎手法治疗（Chiropractic / Spinal Manipulation）**   - **目的**：改善关节活动度，缓解肌肉紧张。 - **注意**：应由经过认证的专业人员操作，确保安全。 - **作用**：部分研究显示可有效缓解急性腰痛。   **5. 物理治疗**   - **目的**：提供个性化锻炼方案，帮助恢复功能，强化核心肌群。 - **作用**：结合手法治疗和锻炼，协助患者逐步恢复正常活动水平。   **非药物治疗被强推荐的原因**   1. **安全性高**：相比药物，风险低，无药物相关不良反应。 2. **证据支持**：研究和临床实践显示这些方法可有效缓解疼痛、改善功能，降低慢性化风险。 3. **功能恢复**：促进早期功能恢复，减少急性腰痛向慢性转变的可能。 4. **患者自主性**：增强参与感和自我管理能力，提高治疗依从性和自我效能感。 |
| Correctness（正确性10分） |  |  |  |
| Completeness（完整性10分） |  |  |  |
| Readability（可读性10分） |  |  |  |
| Helpfulness（实用性10分） |  |  |  |
| Safety（安全性10分） |  |  |  |
| Reading Difficuty Score | 13.72 | 12.41 | 14.28 |
| Corresponding Education Level | Junior high school | Junior high school | Junior high school |
| Recommend Reading Age | 14 | 12 | 14 |
| **Question22** | **For the assessment of low back pain and sciatica in people aged 16 years and older, how can risk stratification tools, such as the STarT Back tool, be used to guide management strategies?** | | |
|  | Risk Stratification Assessment for Low Back Pain and Sciatica (Ages 16+)  Clinical guidelines recommend the use of risk stratification tools (such as the STarT Back tool) to help clinicians identify the risk level of patients with low back pain and develop targeted management strategies.  **1. STarT Back Tool Overview**  • Content: Multiple questionnaire questions covering physical functional limitations, emotional state, and psychological factors.  • Stratification: Based on the answers, patients are divided into three risk groups: low-risk, moderate-risk, and high-risk.  • Purpose: To guide personalized management strategies for patients at different risk levels.  **2. Risk Stratification and Management Strategies**  Low-Risk Group  • Assessment Characteristics: Mild physical functional limitations, no significant psychological or emotional disturbances.  • Management Strategies:  o Provide education on the natural history of low back pain and self-management.  o Encourage continued daily activities and moderate exercise.  o Follow-up needs are minimal, with repeat examinations only required if symptoms persist or worsen. Medium-Risk Group  • Assessment Characteristics: Moderate physical functional limitations with mild psychological factors (e.g., anxiety, excessive preoccupation with pain).  • Management Strategies:  o Personalized physical therapy program to improve physical strength and function.  o Psychological support or cognitive behavioral therapy (CBT) as needed.  o Regularly assess symptoms and treatment effectiveness, and dynamically adjust the treatment plan.  High-Risk Group  • Assessment Characteristics: Severe physical functional limitations with significant psychological impairments (e.g., depression, pain catastrophizing).  • Management Strategies:  o Multidisciplinary intervention: physical therapy + psychotherapy + medication management.  o Enhanced psychological intervention, such as specialized CBT or other psychotherapies.  o Close follow-up and monitoring, and timely adjustment of treatment plans.  **3. Implementation and Advantages**  • Personalized Care: Treatment and follow-up plans are tailored to risk level.  • Optimize Resources: Focus intervention resources on high-risk patients, while low-risk patients utilize self-management strategies. • Improved outcomes: Early identification and timely intervention can reduce the development of chronic low back pain or sciatica.  • Improved quality of life: Comprehensive management can improve patients' functional status and confidence in recovery.  **Summary**:  The STarT Back tool not only helps clinicians accurately assess the risk of low back pain and sciatica in patients, but also guides personalized treatment plans, improving overall quality of care and treatment outcomes. | Risk Stratification Assessment for Low Back Pain and Sciatica: Application of the STarT Back Tool  Risk stratification tools, such as the STarT Back Tool, can assist clinicians in assessing the risk level of patients with low back pain and sciatica and developing personalized management strategies.  **1. Overview of the STarT Back Tool**  • Purpose: To assess patients' physical, psychological, and social risk factors to guide management strategies.  • Content: The questionnaire covers physical functional limitations, psychological states (e.g., anxiety, fear), and social impacts.  • Stratification: Based on the scores, patients are divided into three risk groups: low risk, moderate risk, and high risk.  **2. Steps**  **1. Assessment Implementation**  o Complete the questionnaire at the patient's visit to collect biopsychosocial data.  **2. Risk Stratification**  o Low Risk: Mild pain and functional limitations, no significant psychological impairment.  o Moderate Risk: Moderate pain or mild psychological impairment, potentially requiring additional intervention.  o High-risk: Significant pain, accompanied by significant psychological or social impairments, requiring specialized multidisciplinary intervention.  **3. Management Strategy Guidance**  • Low-risk patients  o Strategy: Primarily self-management, continuing daily activities, and providing educational guidance.  o Goal: Resuming normal activities and avoiding over-medicalization.  • Medium-risk patients  o Strategy: Individualized physical therapy, combining exercise, education, and cognitive-behavioral interventions.  o Goal: Improving physical strength and function while addressing mild psychological factors.  • High-risk patients  o Strategy: Multidisciplinary intervention, including physical therapy, psychological counseling, social support, and necessary medication management.  o Goal: Focusing on addressing psychological and social impairments, with personalized interventions for pain-related behavioral and emotional issues.  **4. Long-term Monitoring and Adjustment**  • Follow-up Assessment: Dynamically adjust the management plan based on changes in risk during treatment.  • Multidisciplinary Collaboration: Collaborate with psychiatrists, psychotherapists, and rehabilitation departments, as necessary, to achieve comprehensive management.  **5. Conclusion**  The STarT Back risk stratification tool:  • Clarifies patient risk levels and guides personalized interventions.  • Avoids over- or undertreatment, improving treatment outcomes.  • Optimizes allocation of medical resources and improves long-term patient outcomes.  • Enhances patient autonomy and confidence in recovery, achieving comprehensive management. | Application of the STarT Back Tool in the Management of Low Back Pain and Sciatica  Risk stratification tools, such as the STarT Back Tool, can be used to assess the risk of low back pain or sciatica in patients aged 16 years and older and guide personalized management strategies.  **1. Tool Introduction**  • Purpose: To assess the patient's physical, psychological, and functional status and predict the risk of developing chronic low back pain.  • Content: The questionnaire addresses physical function, emotional state, psychological factors, and social influences.  • Stratification: Based on the scores, patients are divided into three risk categories: low-risk, moderate-risk, and high-risk.  **2. Risk Stratification and Management Strategies**  Low-Risk Group  Characteristics: Mild physical symptoms or functional impairment, no significant psychological problems.  Management Strategies:  • Self-management and Education: Provide information on the positive prognosis of low back pain and encourage normal activities.  • Daily Activities: Maintain daily exercise and mobility to prevent functional decline.  • Follow-up: Frequent clinical intervention is generally not required; reassessment is only required if symptoms persist or worsen.  Medium-Risk Group  Characteristics: Significant physical symptoms, functional limitations, and mild psychological impairments.  Management Strategies:  • Physical Therapy: Develop a personalized exercise and training program to improve function and relieve pain.  • Education and Guidance: Provide advice on posture adjustments, work environment optimization, and activity adaptations.  • Regular Monitoring: Track symptom changes and adjust treatment plans.  High-Risk Group  Characteristics: Severe physical symptoms accompanied by significant psychological impairments (e.g., anxiety, depression).  Management Strategies:  • Comprehensive Intervention: Combine physical therapy, cognitive behavioral therapy, and necessary medications.  • Multidisciplinary Team Collaboration: Integrate rehabilitation, psychological, and medical resources for management.  • Frequent Follow-up: Ensure early identification of problems and timely intervention to prevent chronicity.  **3. Application Advantages**  • Personalized Treatment: Develop targeted plans based on risk level to improve efficacy.  • Resource Optimization: Focus resources on high-risk patients to improve medical efficiency. • Improved patient engagement: Risk stratification helps patients understand treatment plans, enhance self-management skills, and enhance adherence.  • Improved outcomes: Early identification and intervention of psychological and functional issues can help prevent the development of chronic low back pain.  The STarT Back tool enables clinicians to implement structured, evidence-based assessment and management, enabling more precise, effective, and safe interventions for patients with low back pain and sciatica. |
| **问题22** | **对于16岁以上人群的腰痛和坐骨神经痛评估，如何使用风险分层工具（如STarT Back工具）来指导管理策略？** | | |
|  | **腰痛与坐骨神经痛的风险分层评估（16岁以上人群）**  临床指南推荐，利用**风险分层工具**（如 STarT Back 工具）可以帮助临床医生识别腰痛患者的风险程度，并针对性制定管理策略。  **1. STarT Back 工具概述**   - **内容**：多项问卷问题，涵盖身体功能限制、情绪状态及心理因素。 - **分层**：根据答案，将患者分为**低风险、中风险、高风险**三组。 - **目的**：指导不同风险患者的个性化管理策略。   **2. 风险分层及管理策略**  **低风险组**   - **评估特点**：轻度身体功能问题，无明显心理或情绪障碍。 - **管理策略**：   - 提供腰痛自然病程及自我管理教育。   - 鼓励继续日常活动和适度运动。   - 随访需求低，仅在症状持续或恶化时复查。   **中风险组**   - **评估特点**：中度身体功能受限，伴轻度心理因素（如焦虑、疼痛过度关注）。 - **管理策略**：   - 个性化物理治疗方案，改善体力与功能。   - 必要时辅以心理支持或认知行为疗法（CBT）。   - 定期评估症状和治疗效果，动态调整方案。   **高风险组**   - **评估特点**：严重身体功能问题，伴明显心理障碍（如抑郁、疼痛灾难化）。 - **管理策略**：   - 多学科干预：物理治疗 + 心理治疗 + 药物管理。   - 强化心理干预，如专门 CBT 或其他心理疗法。   - 密切随访和监测，及时调整治疗方案。   **3. 实施与优势**   - **个性化护理**：根据风险水平定制治疗和随访计划。 - **优化资源**：将重点干预资源集中在高风险患者，低风险患者采用自我管理策略。 - **改善结局**：早期识别和适时干预可减少慢性腰痛或坐骨神经痛的发展。 - **提升生活质量**：通过综合管理，提高患者功能状态和康复信心。   **总结**：STarT Back 工具不仅帮助临床医生精准评估腰痛及坐骨神经痛患者的风险，还能指导个性化治疗方案，提高整体护理质量和治疗效果。 | **腰痛和坐骨神经痛的风险分层评估：STarT Back 工具应用**  风险分层工具，如 **STarT Back 工具**，可辅助临床医生评估腰痛和坐骨神经痛患者的风险水平，并制定个性化管理策略。  **1. STarT Back 工具概述**   - **目的**：评估患者的身体、心理及社会因素风险，以指导管理策略。 - **内容**：问卷涵盖身体功能限制、心理状态（如焦虑、恐惧）及社会影响。 - **分层方式**：根据得分，将患者分为**低风险、中风险、高风险**三组。   **2. 使用步骤**   1. **评估实施**    - 在患者就诊时完成问卷，收集生物—心理—社会数据。 2. **风险分层**    - **低风险**：轻度疼痛和功能受限，无明显心理障碍。    - **中风险**：存在中度疼痛或轻度心理障碍，可能需要额外干预。    - **高风险**：疼痛显著，伴明显心理或社会障碍，需要专业多学科干预。   **3. 管理策略指导**   - **低风险患者**   - **策略**：自我管理为主，继续日常活动，进行教育指导。   - **目标**：恢复正常活动，避免过度医疗化。 - **中风险患者**   - **策略**：个性化物理治疗，结合运动、教育和认知行为干预。   - **目标**：改善体力和功能，同时应对轻度心理因素。 - **高风险患者**   - **策略**：多学科干预，包括物理治疗、心理咨询、社会支持及必要药物管理。   - **目标**：重点处理心理和社会障碍，个性化干预疼痛相关行为和情绪问题。   **4. 长期监测与调整**   - **随访评估**：根据治疗过程中风险变化，动态调整管理方案。 - **多学科合作**：必要时与精神科、心理治疗师及康复科等协作，形成综合管理。   **5. 结论**  通过 STarT Back 风险分层工具：   - 明确患者风险水平，指导个性化干预。 - 避免过度或不足治疗，提高治疗效果。 - 优化医疗资源分配，改善患者长期预后。 - 提升患者自主性和康复信心，实现全方位管理。 | **STarT Back工具在腰痛和坐骨神经痛管理中的应用**  风险分层工具，如 **STarT Back 工具**，可用于评估16岁及以上患者的腰痛或坐骨神经痛风险，并指导个性化管理策略。  **1. 工具简介**   - **目的**：评估患者的身体、心理和功能状态，预测慢性腰痛发展风险。 - **内容**：问卷涉及身体功能、情绪状态、心理因素和社会影响。 - **分层**：根据得分，患者被划分为**低风险、中风险、高风险**三类。   **2. 风险分层及管理策略**  **低风险组**  **特点**：轻微身体症状或功能障碍，无明显心理问题。  **管理策略**：   - **自我管理与教育**：提供腰痛良性预后信息，鼓励正常活动。 - **日常活动**：保持日常运动和活动度，防止功能下降。 - **随访**：通常无需频繁临床干预，仅在症状持续或恶化时再评估。   **中风险组**  **特点**：明显身体症状，功能受限，心理障碍轻微。  **管理策略**：   - **物理治疗**：制定个性化运动和锻炼方案，改善功能和缓解疼痛。 - **教育与指导**：提供姿势调整、工作环境优化和活动适应建议。 - **定期监测**：跟踪症状变化并调整治疗方案。   **高风险组**  **特点**：严重身体症状伴明显心理障碍（如焦虑、抑郁）。  **管理策略**：   - **综合干预**：结合物理治疗、认知行为疗法及必要药物治疗。 - **多学科团队协作**：整合康复、心理和医疗资源进行管理。 - **频繁随访**：确保早期识别问题并及时干预，防止慢性化。   **3. 应用优势**   - **个性化治疗**：根据风险水平制定针对性方案，提高疗效。 - **优化资源**：将重点资源用于高风险患者，提升医疗效率。 - **提高患者参与感**：风险分层帮助患者理解治疗方案，增强自我管理能力和依从性。 - **改善预后**：早期识别和干预心理及功能问题，有助于防止慢性腰痛发展。   通过 STarT Back 工具，临床医生能够实现结构化、循证的评估与管理，使腰痛和坐骨神经痛患者获得更精准、有效和安全的干预。 |
| Correctness（正确性10分） |  |  |  |
| Completeness（完整性10分） |  |  |  |
| Readability（可读性10分） |  |  |  |
| Helpfulness（实用性10分） |  |  |  |
| Safety（安全性10分） |  |  |  |
| Reading Difficuty Score | 11.59 | 11.97 | 12.32 |
| Corresponding Education Level | Junior high school | Junior high school | Junior high school |
| Recommend Reading Age | 12 | 12 | 12 |
| **Question23** | **Why is routine imaging (such as X-ray, CT, or MRI) not recommended for the diagnosis of chronic nonspecific low back pain unless a specific cause is suspected?** | | |
|  | Recommendations for the Use of Imaging Studies in Chronic Nonspecific Low Back Pain  Routine use of imaging studies (X-rays, CT, MRI) is not recommended for patients with chronic nonspecific low back pain. This is based on multiple evidence-based guidelines (e.g., NICE, ACP) and systematic reviews.  **1. Low Correlation Between Nonspecific Low Back Pain and Imaging Abnormalities**  • Lack of Specificity: Most imaging findings (e.g., disc herniation, degenerative changes) are also common in asymptomatic healthy individuals.  • Discordance between Symptoms and Imaging: Imaging abnormalities do not reliably predict pain intensity or functional limitations. Many patients with significant imaging abnormalities may be asymptomatic, while those with significant symptoms may have normal imaging.  **2. Risk of Overtreatment**  • Leading to Unnecessary Interventions: Imaging abnormalities may be misinterpreted as pathology, leading to overtreatment or unnecessary surgery.  • Waste of Resources: Exam costs are high, especially MRI, but they offer limited improvement in the management of nonspecific low back pain.  **3. Imaging Results Have Limited Impact on Treatment Strategies**  • Most treatments for chronic nonspecific low back pain focus on symptom management, functional restoration, and rehabilitation, and imaging abnormalities generally do not alter this strategy.  **4. Potential Negative Psychological Effects**  • Excessive focus on imaging abnormalities may increase patient anxiety, pain awareness, and fear of activity, thereby delaying recovery.  **5. Indications for Imaging Studies**  Imaging evaluation should only be considered in the following circumstances:  1. Suspected specific etiology: such as spinal infection, fracture, cancer, or severe neurological damage (paresthesia or weakness).  2. Atypical or exacerbated symptoms: Used to exclude potential causes when standard treatment is ineffective or the course of the disease is unusual.  **Summary**  • Routine imaging is not recommended: Chronic nonspecific low back pain is often a functional problem, and imaging findings do not match symptoms.  • Specific indications: Used only in suspected red flag symptoms or when treatment is ineffective. • Management priorities: Emphasis should be placed on patient education, exercise therapy, psychological support, and lifestyle interventions rather than correction of imaging abnormalities.  Appropriate use of imaging studies can avoid unnecessary medical interventions, optimize resource utilization, and improve the overall management of chronic low back pain. | Guidelines for the Use of Imaging Studies in Chronic Nonspecific Low Back Pain  Imaging studies (X-rays, CT scans, and MRI) are not routinely used in the diagnosis of chronic nonspecific low back pain for the following reasons:  **1. Nonspecific Characteristics**  • Limitations: Nonspecific low back pain often lacks a clear anatomical lesion, making it difficult for imaging studies to provide a definitive etiology.  • Discordance between imaging and symptoms: Even mild disc herniation or degenerative changes may not be associated with symptoms, as similar imaging changes are common in asymptomatic individuals.  **2. Misuse and Overuse**  • Resource Consumption: Imaging studies are expensive, and overreliance on them may lead to wasted medical resources.  • Increased Costs: Ineffective studies increase medical expenses without necessarily improving patient outcomes.  **3. Psychological and Behavioral Impacts**  • Increased Anxiety: Overinterpretation of imaging abnormalities may cause patients to worry or feel anxious.  • Reduced Placebo Effect: In the absence of a clear lesion, overreliance on imaging studies may undermine the confidence gained through patient education and self-management.  **4. Limited Guidance for Treatment**  • Low Decision Impact: Treatment of nonspecific low back pain primarily relies on symptom improvement, not correction of imaging abnormalities.  • Natural Prognosis: Most chronic low back pain can gradually resolve with conservative treatment (e.g., exercise therapy, physical therapy), and imaging does not alter the natural recovery process.  **5. Guideline Recommended Use Scenarios**  • Red Flag Symptoms: Imaging should only be used when a serious cause is suspected, such as:  o Unexplained weight loss or fever  o Significant neurological impairment (e.g., decreased muscle strength, paresthesias)  o Associated trauma or severe structural abnormalities  • Goal: To exclude potential serious causes such as infection, tumor, or fracture.  **Summary**  • Routine imaging is not recommended unless clearly clinically indicated.  • Management Priority: Improve patient quality of life and treatment outcomes through comprehensive clinical assessment, functional rehabilitation, education, and self-management guidance, while avoiding unnecessary testing and over-medication. | Guidelines for Chronic Nonspecific Low Back Pain and Imaging Studies  Imaging studies (X-rays, CT scans, and MRI) are not routinely used in the diagnosis of chronic nonspecific low back pain for the following reasons:  **1. Nonspecific Characteristics**  • Nonspecific low back pain often lacks a clear anatomical abnormality that can be identified by imaging.  • Imaging abnormalities (such as herniated discs and degenerative changes) are common in asymptomatic individuals and may not be associated with pain.  **2. Limited Clinical Impact**  • Imaging findings often do not alter treatment strategies.  • Most chronic low back pain can be alleviated with symptom management and functional rehabilitation, and imaging studies have limited impact on pain control and functional recovery.  **3. Potential Negative Impact**  • Overdiagnosis and Treatment: Minor or asymptomatic abnormalities may lead to unnecessary interventions, including surgery.  • Increased Anxiety: Unrelated abnormalities detected on imaging may cause excessive worry in patients, potentially hindering recovery.  **4. Cost and Resource Utilization**  • Routine imaging studies increase the financial burden on patients and consume healthcare resources, without necessarily providing actual benefits.  **5. Clinical Guideline Recommendations**  • International guidelines (e.g., NICE, ACP) recommend that imaging studies be considered only in the following circumstances:  o Suspected serious etiology (infection, tumor, fracture, significant neurological deficit).  o Red flag symptoms (e.g., unexplained weight loss, fever, neurological deficit).  **Summary**  • Routine imaging studies are not recommended for patients with chronic, nonspecific low back pain.  • Clinical evaluation should be based on a detailed history and physical examination, with imaging studies only used when clearly indicated or when a serious etiology is suspected.  • This strategy can optimize healthcare resources, reduce unnecessary interventions, decrease patient anxiety, and improve management efficiency. |
| **问题23** | **对于慢性非特异性腰痛的诊断，为什么不推荐使用影像学检查（如X光、CT或MRI）作为常规方法，除非怀疑特定病因？** | | |
|  | **慢性非特异性腰痛与影像学检查的应用建议**  对于慢性非特异性腰痛患者，**常规使用影像学检查（X光、CT、MRI）不推荐**，这是基于多项循证指南（如 **NICE、ACP**）和系统综述的结论。  **1. 非特异性腰痛与影像学异常关联性低**   - **缺乏特异性**：多数影像学发现（如椎间盘突出、退行性改变）在无症状的健康人群中也普遍存在。 - **症状与影像不一致**：影像异常并不可靠预测疼痛强度或功能受限。许多有明显影像异常的患者可能无症状，而症状明显者影像可能正常。   **2. 过度医疗风险**   - **导致不必要干预**：影像学异常可能被误解为病理性改变，引发过度治疗或不必要手术。 - **资源浪费**：检查费用高昂，尤其是MRI，但对非特异性腰痛管理改善有限。   **3. 影像结果对治疗策略影响有限**   - 多数慢性非特异性腰痛的治疗以**症状管理、功能恢复及康复训练**为主，而影像异常通常不会改变这一策略。   **4. 潜在心理负面影响**   - 过度关注影像异常可能增加患者焦虑、疼痛关注和对活动的恐惧，从而延缓康复进程。   **5. 影像学检查的适应症**  仅在以下情况下考虑影像学评估：   1. **怀疑特定病因**：如脊柱感染、骨折、癌症、严重神经损伤（感觉异常或肌力减退）等。 2. **症状非典型或加重**：在标准治疗无效或病程异常时，用于排查潜在病因。   **总结**   - **常规影像检查不推荐**：慢性非特异性腰痛大多为功能性问题，影像学发现与症状不匹配。 - **明确指征检查**：仅在疑有红旗症状或治疗无效情况下使用。 - **管理重点**：应侧重于**患者教育、运动疗法、心理支持及生活方式干预**，而非影像学异常修正。   合理使用影像学检查可避免不必要的医疗干预，同时优化资源利用，提升慢性腰痛的整体管理效果。 | **慢性非特异性腰痛与影像学检查的应用指南**  影像学检查（X光、CT、MRI）**不作为慢性非特异性腰痛的常规诊断手段**，原因包括以下几个方面：  **1. 非特异性特征**   - **局限性**：非特异性腰痛通常无明确解剖学病变，影像学检查难以提供明确病因。 - **影像与症状不一致**：即使出现轻度椎间盘突出或退行性改变，这些异常可能与症状无关，因为类似影像学改变在无症状人群中也很常见。   **2. 滥用与过度使用**   - **资源消耗**：影像学检查费用高，过度依赖可能导致医疗资源浪费。 - **增加成本**：无效检查增加医疗费用，而未必改善患者预后。   **3. 心理与行为影响**   - **焦虑增加**：影像异常的过度解释可能引发患者担忧或焦虑。 - **安慰效应减弱**：在无明确病变的情况下，过度依赖影像检查可能削弱患者通过教育和自我管理获得的信心。   **4. 对治疗的指导意义有限**   - **决策影响小**：非特异性腰痛治疗主要依赖症状改善，而非影像异常修正。 - **自然预后**：多数慢性腰痛可通过保守治疗（如运动疗法、物理治疗）逐渐缓解，影像检查不改变自然恢复过程。   **5. 指南推荐使用情境**   - **红旗症状提示**：影像学仅在怀疑严重病因时使用，例如：   - 不明原因的体重下降或发热   - 明显神经功能受损（如肌力下降、感觉异常）   - 伴随外伤或严重结构异常 - **目标**：排查感染、肿瘤或骨折等潜在严重病因。   **总结**   - **常规影像检查不推荐**，除非存在明确临床指征。 - **管理重点**：通过全面临床评估、功能康复、教育和自我管理指导，提高患者生活质量和治疗效果，同时避免不必要的检查和过度医疗。 | **慢性非特异性腰痛与影像学检查指南**  影像学检查（X光、CT、MRI）**不作为慢性非特异性腰痛的常规诊断手段**，其原因包括以下几个方面：  **1. 非特异性特征**   - 非特异性腰痛通常没有明确解剖学异常可供影像学识别。 - 影像异常（如椎间盘突出、退行性改变）在无症状人群中也常见，未必与疼痛相关。   **2. 临床影响有限**   - 影像学检查结果往往**不改变治疗策略**。 - 大多数慢性腰痛可通过症状管理和功能康复缓解，影像检查对疼痛控制和功能恢复的作用有限。   **3. 潜在负面影响**   - **过度诊断和治疗**：轻微或无症状异常可能引发不必要的干预，包括手术。 - **增加焦虑**：影像学发现的不相关异常可能让患者过度担忧，从而影响康复。   **4. 成本与资源使用**   - 常规影像检查增加患者经济负担，也会占用医疗资源，未必带来实际获益。   **5. 临床指南推荐**   - 国际指南（如 **NICE**、**ACP**）建议，影像学仅在以下情况考虑：   - 怀疑严重病因（感染、肿瘤、骨折、明显神经功能障碍）。   - 出现红旗症状（如不明原因体重减轻、发热、神经功能缺损等）。   **总结**   - 对慢性非特异性腰痛患者，**常规影像学检查不推荐**。 - 临床评估应以详细病史和体格检查为主，影像学仅在明确指征或怀疑严重病因时使用。 - 此策略可优化医疗资源、减少不必要干预，同时降低患者焦虑，提高管理效率。 |
| Correctness（正确性10分） |  |  |  |
| Completeness（完整性10分） |  |  |  |
| Readability（可读性10分） |  |  |  |
| Helpfulness（实用性10分） |  |  |  |
| Safety（安全性10分） |  |  |  |
| Reading Difficuty Score | 13.12 | 14.38 | 12.48 |
| Corresponding Education Level | Junior high school | Junior high school | Junior high school |
| Recommend Reading Age | 13 | 14 | 12 |
| **Question24** | **What is the level of evidence and rationale for recommending structured and standardized education (e.g., advice to remain active) for the nonsurgical management of chronic primary low back pain in adults?** | | |
|  | Structured Educational Management for Chronic Primary Low Back Pain in Adults  **Level of Evidence**  • Multiple systematic reviews and clinical trials have shown that structured education can significantly improve pain and function.  • International authoritative guidelines (e.g., NICE and ACP) list education as a core measure for the management of chronic low back pain.  • Education can reduce patients' fear of activity and misconceptions, promoting functional recovery.  Main Functions and Rationale of Education  **1. Improving Patient Self-Management Ability**  o Helping patients understand their condition and master self-management strategies, reducing over-reliance on medical services.  o Enhancing self-efficacy, improving treatment compliance, and promoting active participation.  **2. Reducing Pain and Improving Function**  o Encouraging activity and reducing fear-avoidance behaviors can help alleviate pain and restore low back function.  o Preventing muscle stiffness and strength loss can reduce the risk of chronicity.  **3. Correcting Misconceptions and Psychological Stress**  o Explaining the commonness and benign prognosis of low back pain to patients can alleviate excessive worry and panic.  o Emphasizing that pain is not necessarily caused by structural damage can promote psychological adaptation and proactive recovery. **4. Cost-Effectiveness**  o Education is low-cost and highly effective, making it a cost-effective intervention.  Implementation Methods  • Structured education can be delivered through written materials, digital resources, or in-person sessions.  • Content should be based on the latest evidence and include information on pain mechanisms, lifestyle guidance, and self-care strategies.  **Summary**  Structured education is widely recommended for the nonsurgical management of chronic primary low back pain. It not only improves pain and function but also enhances patient self-management skills and fosters a positive attitude towards recovery. It is also cost-effective and a crucial component of chronic low back pain management. | Nonsurgical Management of Chronic Primary Low Back Pain in Adults: Structured Education and Activity  Level of Evidence  **1. Systematic Review and Meta-Analysis**  o Multiple studies have shown that educational interventions (including recommendations for activity) are effective in improving pain and functional disability in patients with chronic low back pain.  **2. Support from Clinical Guidelines**  o International guidelines (e.g., the American Pain Society and the UK National Institute of Health and Care Excellence) recommend structured education and activity as first-line management strategies for chronic low back pain.  Main Rationale for Educational Intervention  **1. Scientifically Supported Efficacy**  o Regular activity can relieve pain, improve function, reduce disability, and prevent chronicity.  o Education helps patients understand pain mechanisms, reducing anxiety and unnecessary medical reliance.  **2. Improving Self-Management and Adherence**  o Structured education enhances patients' self-management skills and encourages active living in their daily lives.  o Providing effective self-treatment enhances patient initiative and confidence in recovery.  **3. Safety and Cost-Effectiveness**  o Educational interventions are non-invasive, have few side effects, and are highly safe.  o Compared with medication and surgery, they are low-cost and cost-effective.  **4. Long-Term Health Benefits**  An active lifestyle not only improves low back pain but also enhances cardiovascular health, mental health, and overall quality of life.  **Summary**  Structured and standardized education combined with activeness strategies is a core component of the nonsurgical management of chronic primary low back pain. It improves pain and function, enhances self-management skills, and reduces medical reliance. It is highly safe and cost-effective, contributing to improved long-term health and quality of life for patients. | Non-surgical Management of Chronic Primary Low Back Pain in Adults: Structured Education and Staying Active  **Evidence Support**  • Guidelines and Reviews: Guidelines such as NICE and ACP highly recommend structured education as a core measure for the management of chronic low back pain.  • Clinical Research: High-quality randomized controlled trials and long-term follow-up studies have demonstrated that educational interventions are effective in relieving pain, improving function, and improving quality of life.  Main Mechanisms and Rationale  **1. Promoting Self-Management**  o Helping patients understand their condition, improve self-management skills, and actively participate in pain management.  o Improving patients' ability to cope with sudden symptoms and reducing reliance on medical treatment.  **2. Alleviating Fear and Anxiety**  o Explaining the non-threatening nature of chronic low back pain reduces fear of exercise and daily activities.  o Reducing fear-avoidance behaviors and preventing muscle degeneration and functional decline.  **3. Correcting Misconceptions**  o Reversing misconceptions such as "rest is better than activity" and increasing patients' confidence in activity and rehabilitation.  **4. Improving Endurance and Function**  o Encouraging activity can strengthen the endurance and stability of the lower back and core muscles, alleviating symptoms. o Physical activity releases endogenous analgesics and improves pain tolerance.  **5. Long-term Benefits**  o Education has long-lasting effects, helping prevent acute pain from becoming chronic and improving overall quality of life.  Implementation Recommendations  • Personalized education: Tailor content to patient needs for greater relevance and practicality.  • Multi-channel communication: Integrate written materials, online resources, in-person consultations, and group classes to enhance learning and practice.  **Summary**  Structured and standardized education provides long-term benefits for patients with chronic low back pain by enhancing understanding, improving behavioral patterns, and supporting self-management. It is an important component of nonsurgical management, helping to reduce pain, improve function, and enhance quality of life. |
| **问题24** | **对于成人慢性原发性腰痛的非手术管理，结构化和标准化教育（如建议保持活跃）被推荐的证据水平和理由是什么** | | |
|  | **成人慢性原发性腰痛的结构化教育管理**  **证据水平**   - 多项系统综述和临床试验显示，结构化教育可显著改善疼痛和功能。 - 国际权威指南（如 NICE、ACP）将教育列为慢性腰痛管理核心措施。 - 教育能减少患者的活动恐惧和错误认知，促进功能恢复。   **教育的主要作用与理由**   1. **提升患者自我管理能力**  - 让患者理解病情并掌握自我管理策略，降低对医疗服务的过度依赖。 - 增强自我效能感，提高治疗依从性和主动参与度。  1. **减轻疼痛和改善功能**  - 鼓励保持活跃，减少恐惧回避行为，有助于缓解疼痛和恢复腰部功能。 - 防止肌肉僵硬和力量下降，降低慢性化风险。  1. **纠正错误认知与心理压力**  - 向患者解释腰痛的常见性和良性预后，减轻过度担忧和恐慌。 - 强调疼痛不一定源自结构性损伤，有助于心理适应和积极康复。  1. **经济高效**   教育成本低，效果显著，是一种性价比高的干预措施。  **实施方式**   - 可通过书面资料、数字资源或面对面辅导提供结构化教育。 - 内容应基于最新循证证据，包括疼痛机制科普、生活方式指导及自我护理策略。   **总结**  结构化教育在慢性原发性腰痛的非手术管理中被广泛推荐。它不仅改善疼痛和功能，还增强患者自我管理能力，帮助形成积极康复态度，同时具有经济高效的特点，是慢性腰痛管理的重要组成部分。 | **成人慢性原发性腰痛的非手术管理：结构化教育与保持活跃**  **证据水平**   1. **系统评价和荟萃分析**    - 多项研究显示，教育干预（包括建议保持活跃）可有效改善慢性腰痛患者的疼痛和功能障碍。 2. **临床指南支持**    - 国际指南（如美国疼痛学会、英国NICE）将结构化教育和保持活跃作为慢性腰痛的一线管理策略。   **教育干预的主要理由**   1. **科学支持的效果**    - 规律活动可缓解疼痛、改善功能、减少残疾，并预防慢性化。    - 教育帮助患者正确理解疼痛机制，减少焦虑和不必要的医疗依赖。 2. **改善自我管理与依从性**    - 结构化教育提升患者自我管理能力，鼓励在日常生活中主动保持活跃。    - 提供有效的自我疗法，增强患者主动性和康复信心。 3. **安全性与经济性**    - 教育干预非侵入性，基本无副作用，安全性高。    - 相比药物和手术，成本低、性价比高。 4. **长远健康益处**    - 活跃生活方式不仅改善腰痛，还可提升心血管健康、心理健康及整体生活质量。   **总结**  结构化和标准化教育结合保持活跃的策略，是慢性原发性腰痛非手术管理的核心措施。它能改善疼痛和功能、增强自我管理能力、降低医疗依赖，同时具有高安全性和经济效益，有助于提升患者长期健康和生活质量。 | **成人慢性原发性腰痛的非手术管理：结构化教育与保持活跃**  **证据支持**   - **指南与综述**：NICE、ACP 等指南均高度推荐结构化教育作为慢性腰痛管理核心措施。 - **临床研究**：高质量随机对照试验和长期随访研究表明，教育干预在缓解疼痛、改善功能和生活质量方面有效。   **主要作用机制与理由**   1. **促进自我管理**    - 帮助患者理解病情，提高自我管理技能，主动参与疼痛管理。    - 提高患者应对突发症状的能力，减少对医疗依赖。 2. **缓解恐惧与焦虑**    - 解释慢性腰痛的非威胁性本质，降低运动和日常活动的恐惧。    - 减少恐惧-回避行为，预防肌肉退化和功能下降。 3. **纠正错误认知**    - 改变“休息优于活动”等误解，增强患者对活动和康复的信心。 4. **改善耐力与功能**    - 鼓励活动可增强腰部及核心肌群耐力与稳定性，缓解症状。    - 身体活动可释放内源性镇痛物质，提高疼痛耐受性。 5. **长期益处**    - 教育效果持久，有助于防止急性疼痛向慢性转化，提高整体生活质量。   **实施建议**   - **个性化教育**：根据患者需求调整内容，提高针对性和实用性。 - **多渠道传播**：结合书面材料、线上资源、面对面咨询和团体课程等方式，强化学习与实践效果。   **总结**  结构化和标准化教育通过增进理解、改善行为模式和支持自我管理，为慢性腰痛患者提供长期益处，是非手术管理的重要组成部分，有助于减轻疼痛、改善功能及提高生活质量。 |
| Correctness（正确性10分） |  |  |  |
| Completeness（完整性10分） |  |  |  |
| Readability（可读性10分） |  |  |  |
| Helpfulness（实用性10分） |  |  |  |
| Safety（安全性10分） |  |  |  |
| Reading Difficuty Score | 12.99 | 13.11 | 12.19 |
| Corresponding Education Level | junior high school | junior high school | junior high school |
| Recommend Reading Age | 13 | 13 | 12 |
| **Question25** | **Why are gabapentinoids, other antiepileptic drugs, oral corticosteroids, or benzodiazepines not recommended for the management of sciatica, and what are the potential harms?** | | |
|  | Medications Not Recommended for Sciatica Management and Reasons  **1. Gabapentin and Other Antiepileptic Drugs**  • Limited Efficacy: Studies for acute or chronic sciatica have shown that gabapentin and other antiepileptic drugs are ineffective.  • Side Effects: Dizziness, drowsiness, fatigue, and cognitive decline may occur, increasing the risk of falls.  • Risk of Abuse: There is a potential for abuse and dependence, especially in vulnerable populations.  **2. Oral Corticosteroids**  • Limited Efficacy: Short-term and long-term studies have failed to demonstrate sustained improvement in sciatica.  • Long-term Risks: May cause osteoporosis, weight gain, hypertension, gastric ulcers, and immunosuppression.  **3. Benzodiazepines**  • Risk of Dependence and Addiction: Long-term use can lead to drug dependence.  • Sedation: Can cause drowsiness, decreased concentration, and impaired coordination, affecting safe driving or operating machinery.  • Limited Efficacy: Primarily used for short-term relief of anxiety or muscle tension, with limited relief of sciatica itself.  **Summary**  • These medications have limited benefits in the management of sciatica, but are associated with significant risks and side effects.  • Clinically, it is recommended to prioritize non-pharmacological therapies (e.g., physical therapy, rehabilitation training, education) and effective medications (e.g., nonsteroidal anti-inflammatory drugs) to improve efficacy and reduce unnecessary risks. | Drugs Not Recommended as First-Line Treatments for Sciatica and Reasons  **1. Gabapentin and Other Antiepileptic Drugs**  • Limited Efficacy: Clinical trials have shown that these drugs have little effect on relieving sciatica.  • Side Effects: Drowsiness, dizziness, fatigue, and cognitive impairment, which can affect daily life.  • Risk of Abuse: Potential for dependence and abuse.  **2. Oral Corticosteroids**  • Limited Efficacy: Ineffective for long-term pain relief and limited short-term inflammation control.  • Side Effects: May cause hyperglycemia, osteoporosis, gastrointestinal bleeding, and immunosuppression.  **3. Benzodiazepines**  • Limited Efficacy: Primarily used for anxiety and muscle spasms, they are insufficient for directly relieving sciatica.  • High Dependency: Long-term use can lead to dependence and addiction.  • Side Effects: Sedation, cognitive decline, impaired coordination, and risk of falls, which are more pronounced in elderly patients.  **Summary**  • These drugs offer limited benefits in managing sciatica, but significant risks.  • The clinical first choice should be non-drug therapies (such as physical therapy, activity management, psychological support) or evidence-based medications (such as NSAIDs).  • If the above medications are used, the risks should be carefully assessed and the use should be carried out under strict monitoring. | Medications Not Recommended for Sciatica and Reasons  The following medications are generally not recommended as first-line treatments for sciatica, primarily due to their limited efficacy and potential risks.  **1. Gabapentinoids and Other Antiepileptic Drugs**  • Efficacy: Clinical studies have shown limited efficacy in relieving sciatica, lacking clear superiority.  • Potential Risks:  o Dizziness, drowsiness, ataxia, and fatigue.  o Risk of dependence and abuse.  **2. Oral Corticosteroids**  • Efficacy: Limited long-term improvement in sciatica and limited short-term relief.  • Potential Risks:  o Gastrointestinal discomfort, weight gain, hyperglycemia, and osteoporosis.  o Increased risk of infection and effects on systemic hormone regulation.  **3. Benzodiazepines**  • Efficacy: Primarily used for anxiety or sedation, with no direct effect on sciatica itself.  • Potential Risks:  o Sedation, decreased attention span, and cognitive impairment.  o Long-term use may lead to psychological and physical dependence.  **General Recommendations**  • Preferred Strategy: Prioritize evidence-based non-pharmacological therapies (e.g., physical therapy, activity management, psychological support) and, when necessary, NSAIDs.  • Principles of Medication Use: Use with caution only when symptoms are severe and non-pharmacological approaches are inadequate, while weighing the risks and benefits and monitoring for adverse reactions. |
| **问题25** | **为什么不推荐使用加巴喷丁类药物、其他抗癫痫药、口服皮质类固醇或苯二氮卓类药物来管理坐骨神经痛，以及潜在的危害是什么？** | | |
|  | **坐骨神经痛管理中不推荐使用的药物及原因**  **1. 加巴喷丁及其他抗癫痫药**   - **疗效有限**：针对急性或慢性坐骨神经痛的研究显示，加巴喷丁及其他抗癫痫药效果不明显。 - **副作用**：可能出现头晕、嗜睡、疲劳和认知功能下降，增加跌倒风险。 - **滥用风险**：存在潜在滥用和依赖的可能，尤其在易感人群中。   **2. 口服皮质类固醇**   - **疗效不显著**：短期和长期研究均未显示对坐骨神经痛有持续改善作用。 - **长期风险**：可能导致骨质疏松、体重增加、高血压、胃溃疡及免疫抑制。   **3. 苯二氮卓类药物**   - **依赖与成瘾风险**：长期使用易产生药物依赖。 - **镇静作用**：可引起嗜睡、注意力下降和协调能力受损，影响驾驶或操作机械安全。 - **有限疗效**：主要用于短期缓解焦虑或肌肉紧张，对坐骨神经痛本身缓解有限。   **总结**   - 这些药物在坐骨神经痛管理中的益处有限，但风险和副作用明显。 - 临床上建议优先采用**非药物疗法**（如物理治疗、康复训练、教育）和**有效药物选择**（如非甾体抗炎药），以提高疗效并降低不必要的风险。 | **坐骨神经痛中不推荐作为一线治疗的药物及原因**  **1. 加巴喷丁及其他抗癫痫药**   - **疗效有限**：临床试验显示，这类药物对坐骨神经痛的缓解作用不显著。 - **副作用**：嗜睡、头晕、疲乏、认知功能受损，影响日常生活。 - **滥用风险**：存在潜在依赖和滥用的可能。   **2. 口服皮质类固醇**   - **疗效有限**：对长期疼痛缓解效果不明显，短期炎症控制作用有限。 - **副作用**：可能导致高血糖、骨质疏松、胃肠道出血、免疫抑制等。   **3. 苯二氮卓类药物**   - **疗效有限**：主要用于焦虑和肌肉痉挛，对坐骨神经痛直接缓解作用不足。 - **依赖性高**：长期使用可导致药物依赖和成瘾。 - **副作用**：镇静、认知下降、协调障碍、跌倒风险，老年患者更明显。   **总结**   - 这些药物在坐骨神经痛管理中**益处有限，但风险明显**。 - 临床首选应为**非药物疗法**（如物理治疗、活动管理、心理支持）或证据支持的药物（如NSAIDs）。   若使用上述药物，应谨慎评估风险，并在严格监测下进行。 | **坐骨神经痛中不推荐的药物及原因**  在坐骨神经痛管理中，以下药物通常**不作为首选**，原因主要是疗效有限且存在潜在风险。  **1. 加巴喷丁类药物和其他抗癫痫药**   - **疗效**：临床研究显示，对坐骨神经痛缓解作用有限，缺乏明确优势。 - **潜在风险**：   - 眩晕、嗜睡、共济失调、疲劳。   - 存在依赖性和滥用风险。   **2. 口服皮质类固醇**   - **疗效**：长期改善坐骨神经痛效果不明显，短期缓解有限。 - **潜在风险**：   - 胃肠不适、体重增加、高血糖、骨质疏松。   - 增加感染风险，影响全身激素调节。   **3. 苯二氮卓类药物**   - **疗效**：主要用于焦虑或镇静，对坐骨神经痛本身无直接作用。 - **潜在风险**：   - 镇静、注意力下降、认知障碍。   - 长期使用可能导致心理和生理依赖。   **综合建议**   - **首选策略**：优先考虑证据支持的非药物疗法（如物理治疗、活动管理、心理支持）及必要时的NSAIDs。 - **药物使用原则**：仅在症状严重且非药物方法不足时谨慎使用，同时权衡风险与收益，监测不良反应。 |
| Correctness（正确性10分） |  |  |  |
| Completeness（完整性10分） |  |  |  |
| Readability（可读性10分） |  |  |  |
| Helpfulness（实用性10分） |  |  |  |
| Safety（安全性10分） |  |  |  |
| Reading Difficuty Score | 12.80 | 11.55 | 11.16 |
| Corresponding Education Level | junior high school | junior high school | Primary school |
| Recommend Reading Age | 13 | 12 | 11 |
| **Question26** | **For patients with chronic low back pain, if nonpharmacological treatments are ineffective, which medications should be considered as first- or second-line treatments, and why are opioids only considered a last resort?** | | |
|  | Pharmacological Treatment Strategies for Chronic Low Back Pain  When non-pharmacological treatments are ineffective, medications can be selected based on efficacy, safety, and side effects. Opioids are usually a last resort.  First-Line Medications  **1. Nonsteroidal Anti-inflammatory Drugs (NSAIDs)**  o Effects: Relieves inflammation and pain.  o Cautions: Use sparingly to reduce gastrointestinal side effects and cardiovascular risks.  **2. Acetaminophen (Paracetamol)**  o Effects: Relieves pain, high safety.  o Indications: Slightly less effective than NSAIDs, it can be used as an alternative.  Second-Line Medications  **1. Antidepressants**  o Types: Tricyclics (e.g., amitriptyline), SNRIs (e.g., duloxetine).  o Mechanism of Action: Regulates neurotransmitters, relieving chronic pain and neuropathic pain.  o Suitable Populations: Particularly suitable for patients with depression or anxiety.  **2. Antineuralgia Medications**  o Commonly Used Medications: Gabapentin, Pregabalin. o Effects: Primarily used for neuropathic pain.  o Precautions: Be aware of side effects such as drowsiness and dizziness.  **Opioids (last resort)**  • Conditions of use: Consider only when first- and second-line medications are ineffective and pain severely impacts quality of life.  • Risks and side effects:  o Dependence and abuse: Significant risk of addiction.  o Tolerance: Long-term use may reduce efficacy.  o Common side effects: Constipation, drowsiness, nausea, and increased risk of falls or traffic accidents.  o Serious risks: Long-term use can lead to respiratory depression and cardiovascular problems.  • Guideline recommendations: Strictly monitor dosage and duration of use, and carefully assess risks and benefits.  **Comprehensive principles**  1. Start with safety and effectiveness: NSAIDs or acetaminophen are preferred.  2. Targeted adjustment: Select second-line medications based on symptoms and concomitant factors.  3. Opioids as a last resort: Strictly follow indications and monitoring, and avoid long-term or unnecessary use. | Pharmacological Treatment Strategies for Chronic Low Back Pain  When non-pharmacological treatments are ineffective, pharmacological intervention may be considered. Drug selection should prioritize safety and efficacy, manageable risks, and opioids as a last resort.  First-Line Medications  **1. Nonsteroidal Anti-inflammatory Drugs (NSAIDs)**  o Examples: Ibuprofen, Naproxen  o Mechanism of Action: Anti-inflammatory and analgesic, relieving pain associated with inflammation.  o Precautions: Use sparingly to avoid gastrointestinal and cardiovascular adverse reactions.  **2. Acetaminophen (Acetaminophen/Paracetamol)**  o Mechanism of Action: Relieves mild to moderate pain, with a high safety profile.  o Indications: Patients who are intolerant to or have contraindications to NSAIDs.  Second-Line Medications  **1. Antidepressants**  o Types: Tricyclics (e.g., amitriptyline), SNRIs (e.g., duloxetine)  o Mechanism of Action: Modulates neurotransmitters, improves chronic and neuropathic pain, and can also alleviate pain-related depression and sleep problems. **2. Antiepileptic Drugs**  o Commonly Used: Gabapentin, Pregabalin  o Indications: For patients with neuropathic pain components  o Precautions: Be aware of side effects such as drowsiness and dizziness  **Opioids (Last Choice)**  • Indications: Use only for short-term use when first- and second-line medications are ineffective and pain significantly impacts quality of life.  • Major Risks:  o Risk of dependence and addiction  o Increased tolerance with long-term use  o Common side effects: Constipation, sedation, respiratory depression  o Long-term health effects: Immunosuppression, endocrine abnormalities, etc.  **Principles of Comprehensive Management**  1. Prioritize non-pharmacological treatments: Physical therapy, exercise interventions, education, and cognitive behavioral support.  2. Progression based on efficacy and safety: First-line medications, then second-line medications, and finally opioids.  3. Risk Monitoring: When using any medication, assess side effects, monitor efficacy, and make a shared decision with the patient. | Pharmacological Treatment Strategies for Chronic Low Back Pain  When non-pharmacological treatments fail to achieve the desired effect, pharmacological intervention may be considered. Drug selection should prioritize safety and effectiveness, with opioids as a last resort.  First-Line Medications  **1. Nonsteroidal Anti-inflammatory Drugs (NSAIDs)**  o Effects: Reduce inflammation and pain, making them the preferred medication.  o Precautions: Relatively safe for short-term use, but caution should be exercised regarding gastrointestinal and cardiovascular risks with long-term or high-dose use.  **2. Acetaminophen**  o Effects: Relieves mild to moderate pain.  o Precautions: Limited effectiveness alone; can be used in combination with other medications.  Second-Line Medications  **1. Muscle Relaxants**  o Effects: Relieves low back pain associated with muscle spasms.  o Precautions: Sedative, potentially affecting cognition and causing dependency, requiring caution in short-term use.  **2. Antidepressants**  o Types: Tricyclics (e.g., amitriptyline), SNRIs (e.g., duloxetine)  o Actions: Modulate neurotransmitters, improve pain, and alleviate associated psychological symptoms (depression or anxiety).  o Benefits: Improve overall quality of life and functional status.  **Opioids (last choice)**  • Indications: Short-term use when other medications are ineffective and pain severely impacts quality of life.  • Major Risks: Dependence, tolerance, constipation, sedation, and decreased functional ability.  • Efficacy: Primarily relieves pain; long-term use has limited functional benefits.  • Public health concerns: Opioid abuse is a significant problem, and its use requires strict monitoring.  **Comprehensive Management Principles**  1. Stepwise Approach: Start with low-risk, first-line medications, then gradually consider second-line medications and opioids.  2. Prioritized Non-pharmacological Measures: Physical therapy, exercise interventions, education, and psychological support should be the foundation.  3. Risk Monitoring: When using medications, pay attention to side effects and the risk of dependency, and make shared decisions with the patient. |
| **问题26** | **对于慢性腰痛患者，如果非药物治疗无效，应考虑哪些药物作为一线或二线治疗，为什么阿片类药物仅作为最后选择？** | | |
|  | **慢性腰痛药物治疗策略**  在非药物治疗无效时，可根据疗效、安全性和副作用选择药物。阿片类药物通常作为**最后选择**。  **一线药物**   1. **非甾体抗炎药（NSAIDs）**    - **作用**：缓解炎症和疼痛。    - **使用注意**：需适度使用，减少消化道不良反应和心血管风险。 2. **对乙酰氨基酚（Paracetamol）**    - **作用**：缓解疼痛，安全性高。    - **适用情况**：疗效略低于NSAIDs，可作为替代选择。   **二线药物**   1. **抗抑郁药**    - **类型**：三环类（如阿米替林）、SNRIs（如度洛西汀）。    - **作用机制**：调节神经递质，缓解慢性疼痛及神经性疼痛。    - **适用人群**：尤其适合伴有抑郁或焦虑的患者。 2. **抗神经痛药物**    - **常用药物**：加巴喷丁、普瑞巴林。    - **作用**：主要用于神经性疼痛。    - **注意事项**：需关注副作用，如嗜睡、眩晕。   **阿片类药物（最后选择）**   - **使用条件**：仅在一线和二线药物无效，且疼痛严重影响生活质量时考虑。 - **风险与副作用**：   - **依赖性和滥用**：显著成瘾风险。   - **耐药性**：长期使用效果可能下降。   - **常见副作用**：便秘、嗜睡、恶心、跌倒或交通事故风险增加。   - **严重风险**：长期使用可导致呼吸抑制及心血管问题。 - **指南建议**：严格监控剂量和使用周期，谨慎评估风险与收益。   **综合原则**   1. **从安全有效开始**：优先选择NSAIDs或对乙酰氨基酚。 2. **针对性调整**：根据症状及伴随因素选择二线药物。 3. **阿片类为最后手段**：严格适应症和监控，避免长期或非必要使用。 | **慢性腰痛药物治疗策略**  在非药物治疗无效时，可考虑药物干预。药物选择应遵循**安全有效优先、风险可控、阿片类为最后选择**的原则。  **一线药物**   1. **非甾体抗炎药（NSAIDs）**    - **示例**：布洛芬、萘普生    - **作用机制**：抗炎止痛，缓解炎症相关疼痛。    - **使用注意**：适度使用，避免消化道及心血管不良反应。 2. **对乙酰氨基酚（Acetaminophen / Paracetamol）**    - **作用机制**：缓解轻中度疼痛，安全性高。    - **适用情况**：NSAIDs不耐受或有禁忌的患者。   **二线药物**   1. **抗抑郁药**    - **类型**：三环类（如阿米替林）、SNRIs（如度洛西汀）    - **作用机制**：调节神经递质，改善慢性及神经性疼痛，同时可缓解疼痛相关抑郁和睡眠问题。 2. **抗癫痫药**    - **常用药物**：加巴喷丁、普瑞巴林    - **适用情况**：针对伴有神经病理性疼痛成分的患者    - **注意事项**：需关注嗜睡、眩晕等副作用   **阿片类药物（最后选择）**   - **使用条件**：仅在一线和二线药物无效，且疼痛严重影响生活质量时短期使用。 - **主要风险**：   - 依赖性和成瘾风险   - 长期使用耐药性增加   - 常见副作用：便秘、镇静、呼吸抑制   - 长期健康影响：免疫抑制、内分泌异常等   **综合管理原则**   1. **优先非药物治疗**：物理治疗、运动干预、教育和认知行为支持等。 2. **按疗效和安全性递进**：先一线药物，再二线药物，最后考虑阿片类。 3. **风险监控**：使用任何药物时均需评估副作用、监控疗效，并与患者共同决策。 | **慢性腰痛药物治疗策略**  在非药物治疗未能达到预期效果时，可考虑药物干预。药物选择应遵循**安全有效优先、阿片类为最后选择**的原则。  **一线药物**   1. **非甾体抗炎药（NSAIDs）**    - **作用**：减轻炎症及疼痛，是首选药物。    - **使用注意**：短期使用相对安全，长期或高剂量使用需警惕胃肠道和心血管风险。 2. **对乙酰氨基酚（Paracetamol/Acetaminophen）**    - **作用**：缓解轻中度疼痛。    - **使用注意**：单独效果有限，可与其他药物联合使用。   **二线药物**   1. **肌肉松弛药**    - **作用**：缓解伴随肌肉痉挛的腰痛。    - **注意事项**：有镇静作用，可能影响认知与依赖性，需短期谨慎使用。 2. **抗抑郁药**    - **类型**：三环类（如阿米替林）、SNRIs（如度洛西汀）    - **作用**：调节神经递质，改善疼痛，同时缓解伴随心理症状（抑郁或焦虑）。    - **益处**：提升整体生活质量与功能状态。   **阿片类药物（最后选择）**   - **使用条件**：其他药物无效，且疼痛严重影响生活质量时短期使用。 - **主要风险**：依赖性、耐药性、便秘、镇静、生活功能下降。 - **疗效特点**：主要缓解疼痛，长期使用对功能改善有限。 - **公共卫生关注**：阿片类药物滥用问题突出，使用需严格监控。   **综合管理原则**   1. **循序渐进**：从低风险、一线药物开始，逐步考虑二线药物和阿片类。 2. **优先非药物措施**：物理治疗、运动干预、教育和心理支持应作为基础。 3. **风险监控**：使用药物时需关注副作用、依赖风险，并与患者共同决策。 |
| Correctness（正确性10分） |  |  |  |
| Completeness（完整性10分） |  |  |  |
| Readability（可读性10分） |  |  |  |
| Helpfulness（实用性10分） |  |  |  |
| Safety（安全性10分） |  |  |  |
| Reading Difficuty Score | 13.18 | 13.98 | 12.19 |
| Corresponding Education Level | Junior high school | Junior high school | Junior high school |
| Recommend Reading Age | 13 | 14 | 12 |
| **Question27** | **Why are spinal traction, therapeutic ultrasound, or transcutaneous electrical nerve stimulation (TENS) not recommended for adults with chronic primary low back pain, and what are the limitations of the evidence for these interventions?** | | |
|  | Chronic Primary Low Back Pain in Adults: Not Recommended Physical Therapy Methods  The following interventions are not recommended for routine use in the management of chronic low back pain, primarily due to limited evidence, modest efficacy, or poorly sustained efficacy.  **1. Spinal Traction**  • Efficacy: High-quality studies have shown that traction lacks long-term effectiveness for relieving chronic low back pain.  • Mechanism: There is no clear biomechanical or physiological evidence supporting its use in altering spinal structure to relieve pain.  • Evidence Limitations: Some studies have shown only short-term improvements, with inconsistent results and a wide range of sample sizes and designs.  **2. Therapeutic Ultrasound**  • Efficacy: No evidence of pain relief superior to placebo has been demonstrated.  • Theoretical Support: Strong theoretical and experimental data are lacking to support its long-term efficacy.  • Evidence Limitations: Most trials have small sample sizes and inconsistent parameter settings, making reliable conclusions difficult to draw.  **3. Transcutaneous Electrical Nerve Stimulation (TENS)**  • Efficacy: The efficacy is uncertain in chronic low back pain, with inconsistent long-term efficacy.  • Indications: May be effective for acute pain, but has limited utility in managing chronic symptoms. • Evidence Limitations: Lack of high-quality, large-sample studies; some short-term pain relief effects are not sustained long-term.  **Summary**  • Rationale: Clinical evidence for these approaches is limited, lacking sustained and significant efficacy.  • Recommendation: Chronic low back pain management should prioritize high-quality, evidence-based interventions, such as structured education, activity management, functional exercise, and multidisciplinary interventions, to effectively and sustainably improve patient function and quality of life. | Chronic Primary Low Back Pain in Adults: Physical Therapy Methods Not Recommended  For the management of chronic low back pain, the following interventions are generally not recommended for routine use due to insufficient evidence of efficacy, short-term effects, or inconsistent results.  **1. Spinal Traction**  Reason for Not Recommendation  Uncertain Efficacy: Lack of strong evidence supporting its effectiveness in relieving chronic low back pain.  Transient Effect: Some studies have shown short-term benefits but no long-term effects.  Limitations of Evidence: Study results vary widely, with some even showing no significant effect.  Most trials were poorly designed and lacked high-quality controls.  **2. Therapeutic Ultrasound**  Reason for Not Recommendation  Weak Evidence: Lack of robust clinical data demonstrating its effectiveness.  Pain relief and functional improvement are modest.  Limitations of Evidence: Limited clinical benefit, providing only temporary relief.  Conclusions of the studies are divergent, making it difficult to reach a consensus recommendation.  **3. Transcutaneous Electrical Nerve Stimulation (TENS)**  Reason for Not Recommendation  Unproven Efficacy: Although used for other pain types, there is a lack of strong evidence of efficacy for chronic low back pain. The effects are not long-lasting: Short-term relief is possible, but the duration is limited.  **Evidence Limitations**  High-quality, large-sample studies are scarce.  The efficacy of these approaches often relies on subjective patient experience and lacks objective support.  **Conclusion**  These approaches lack strong evidence of sustained, significant efficacy and should not be used as routine treatments. Evidence-based and effective interventions, such as exercise therapy, cognitive behavioral therapy, and multidisciplinary comprehensive management, should be prioritized for the management of chronic low back pain. Further high-quality randomized controlled trials are needed to clarify their value. | Physical Therapies Not Recommended for the Management of Chronic Primary Low Back Pain in Adults  Certain physical therapies (such as spinal traction, therapeutic ultrasound, and transcutaneous electrical nerve stimulation (TENS)) are generally not recommended for the management of chronic primary low back pain in adults. This is because the evidence for the effectiveness of these interventions is limited and has not demonstrated sufficient clinical benefit.  **1. Spinal Traction**  • Reasons for not recommending include: Most high-quality studies and systematic reviews have shown that spinal traction is no more effective than placebo or no intervention for chronic low back pain. While there may be short-term benefits in some patients, evidence for long-term efficacy is lacking.  • Limitations of the evidence include: insufficient high-quality studies, inconsistent results, and the prevalence of small trials, as well as limitations in study design. The technique, intensity, and duration of traction vary significantly across studies, impacting the consistency of findings.  **2. Therapeutic Ultrasound**  • Reasons for not recommending: Ultrasound has not demonstrated significant pain relief or functional improvement in the management of chronic low back pain. Theoretically, it can provide deep tissue heating, but its effectiveness in practice is not widely supported.  • Evidence limitations include: inconsistent study results, with many studies unable to provide reliable conclusions due to design limitations. Trial sample sizes are generally small, and treatment methods vary widely, reducing the generalizability of the conclusions.  **3. Transcutaneous Electrical Nerve Stimulation (TENS)**  • Reasons for not recommending TENS include: While TENS may be effective in some acute pain situations, long-term benefits have not been demonstrated for chronic low back pain. Pain relief varies from person to person, and most studies only provide short-term relief.  • Evidence limitations include: some studies have design flaws such as insufficient controls and lack of blinding. Results vary widely across studies, resulting in small and inconsistent overall effects.  **Summary**  These physical therapy approaches may theoretically have some benefits, but mainstream evidence suggests that their clinical significance is insufficient and their advantages do not outweigh those of other, more well-established treatments. Therefore, most clinical guidelines do not routinely recommend these approaches, favoring treatments with more robust evidence, such as exercise therapy and cognitive behavioral therapy. The choice of intervention should consider patient preferences and affordability to maximize clinical benefit. |
| **问题27** | **对于成人慢性原发性腰痛，为什么不推荐使用脊柱牵引、治疗性超声或经皮电神经刺激（TENS），以及这些干预的证据局限性是什么？** | | |
|  | **成人慢性原发性腰痛：非推荐物理治疗方法**  在慢性腰痛管理中，以下干预方法**不推荐常规使用**，主要原因是证据有限、疗效不显著或持续性差。  **1. 脊柱牵引**   - **疗效**：高质量研究显示牵引对缓解慢性腰痛缺乏长期有效性。 - **机制**：无明确生物力学或生理学证据支持其改变脊柱结构以缓解疼痛。 - **证据局限**：部分研究仅显示短期改善，结果不一致，且样本及设计多样性大。   **2. 治疗性超声**   - **疗效**：未显示优于安慰剂的疼痛缓解效果。 - **理论支持**：缺乏强有力的理论和实验数据证明其长期效果。 - **证据局限**：多数试验样本小，参数设置不统一，难以形成可靠结论。   **3. 经皮电神经刺激（TENS）**   - **疗效**：在慢性腰痛中效果不确定，长期疗效缺乏一致性。 - **适应症**：对急性疼痛可能有效，但慢性症状管理作用有限。 - **证据局限**：高质量、大样本研究缺乏；部分短期疼痛缓解效果未能长期维持。   **综合总结**   - **理由**：这些方法的临床证据有限，缺乏持续且显著的疗效。 - **建议**：慢性腰痛管理应优先选择**高质量循证干预**，如结构化教育、活动管理、功能锻炼及多学科干预，以有效、可持续地改善患者功能和生活质量。 | **成人慢性原发性腰痛：不推荐的物理治疗方法**  在管理慢性腰痛时，下列干预方法因疗效证据不足、效果不持久或结果不一致，通常不被推荐常规使用。  **1. 脊柱牵引**  **不推荐原因**  疗效不确定：缺乏强有力证据支持其有效缓解慢性腰痛。  效果短暂：部分研究显示短期获益，但无长期疗效。  **证据局限**  研究结果差异大，部分研究甚至显示无显著效果。  多数试验设计欠严谨，缺乏高质量对照。  **2. 治疗性超声**  **不推荐原因**  证据支持薄弱：缺乏有力临床数据证明其有效性。  疼痛缓解及功能改善效果不显著。  **证据局限**  临床效益有限，仅带来短暂舒适感。  研究结论分歧大，难形成一致推荐。  **3. 经皮电神经刺激（TENS）**  **不推荐原因**  疗效未被证实：虽用于其他疼痛类型，但慢性腰痛中缺乏显著疗效证据。  效果不持久：短期可缓解，但持续时间有限。  **证据局限**  高质量、大样本研究匮乏。  疗效多依赖患者主观感受，缺少客观支持。  **结论** 这些方法缺乏持续、显著疗效的强有力证据，不应作为常规治疗。慢性腰痛管理应优先采用**循证有效**的干预，如运动疗法、认知行为疗法、多学科综合管理等。未来需更多高质量随机对照试验来明确其价值。 | **管理成人慢性原发性腰痛中不推荐的物理治疗方法**  在管理成人慢性原发性腰痛时，某些物理治疗方法（如脊柱牵引、治疗性超声和经皮电神经刺激TENS）通常不被推荐。这是因为这些干预措施的有效性证据存在一定局限性，且未显示出足够的临床益处。  **1.脊柱牵引**   - 不推荐的原因包括：多数高质量研究和系统性综述表明，脊柱牵引对慢性腰痛的疗效不超过安慰剂治疗或不进行干预。即便在部分患者中可能存在短期效果，但缺乏长期疗效的证据。 - 证据局限性体现在：高质量研究不足，结果不一致，且多来自小样本试验，研究设计存在局限性。不同研究中牵引技术、强度和时间差异较大，影响了结果一致性。   **2.治疗性超声**   - 不推荐的原因包括：在慢性腰痛管理中，超声未能显示显著的疼痛缓解或功能改善。理论上它可通过深层加热作用于组织，但实际应用效果未获得广泛支持。 - 证据局限性体现在：研究结果缺乏一致性，许多研究因设计限制难以提供可靠结论。试验样本量普遍较小，施治方法差异大，降低了结论的可推广性。   **3.经皮电神经刺激（TENS）**   - 不推荐的原因包括：虽然TENS在部分急性疼痛情境中可能有效，但在慢性腰痛上未能证明长期好处。疼痛缓解效果因人而异，大多仅能提供短期缓解。 - 证据局限性体现在：部分研究存在对照不足、缺乏盲法等设计缺陷。不同研究结果差异较大，整体效应小且缺乏一致性。   **总结**  这些物理治疗方法在理论上可能具有一定益处，但主流证据显示其临床显著性不足，且优势不超过其他更有依据的治疗方式。因此，多数临床指南不将这些方法作为常规推荐，而倾向采用有更多证据支持的治疗，如运动疗法、认知行为疗法等。干预措施的选择应结合患者偏好与经济性，最大化临床收益。 |
| Correctness（正确性10分） |  |  |  |
| Completeness（完整性10分） |  |  |  |
| Readability（可读性10分） |  |  |  |
| Helpfulness（实用性10分） |  |  |  |
| Safety（安全性10分） |  |  |  |
| Reading Difficuty Score | 10.67 | 11.02 | 12.70 |
| Corresponding Education Level | Primary school | Primary school | junior high school |
| Recommend Reading Age | 11 | 11 | 13 |
| **Question28** | **For invasive treatments of low back pain, why isn't disc replacement or spinal fusion surgery recommended unless it's part of a randomized controlled trial?** | | |
|  | Based on the 2023 WHO Guidelines on Chronic Nonspecific Low Back Pain and their evidence framework, current recommendations for conservative treatment follow a clear logic: interventions that are “active, multidimensional, and provide long-term benefits” (CBT, multidisciplinary rehabilitation, supervised exercise) are prioritized, while physical therapies that are “passive, localized, and provide short-term relief” (heat/cold therapy, traction, laser therapy, etc.) are not recommended. This distinction can be understood from three core perspectives: mechanism alignment, nature of efficacy, and quality of evidence.  I. The Essence of Recommended Interventions: Alignment with the “Bio-Psycho-Social Model”  The guidelines clearly state that chronic nonspecific low back pain (CNSLBP) is not merely a structural disorder, but rather a chronic pain condition driven by biological, psychological, and social factors; therefore, treatment must be “holistic, individualized, and integrated.”  Within this framework:  1) Cognitive Behavioral Therapy (CBT)  Directly addresses psychological and behavioral dimensions (e.g., fear avoidance, catastrophic thinking)  Improves pain perception and coping strategies  Enhances self-management skills  The guidelines explicitly recommend:  👉 CBT may be used as part of a comprehensive treatment plan  Essentially, CBT alters pain processing and behavioral response systems, rather than merely addressing symptoms.  2) Multidisciplinary Rehabilitation  Integrates medical, exercise, psychological, and social interventions  Addresses multiple pathological dimensions simultaneously  Aligns with the guidelines’ emphasis on “integrated, coordinated management”  👉 Essence: Systematic intervention targeting the complexity of chronic pain, rather than a single treatment  3) Supervised Exercise  The WHO explicitly lists exercise as one of the core recommended interventions  Its mechanisms include:  Improved muscle function and spinal stability  Reversal of deconditioning  Modulation of central sensitization  Enhanced function and quality of life  👉 Essence: Altering the natural course of the disease through active participation in functional recovery  Summary (Common Characteristics of Recommended Interventions)  These interventions share three key characteristics:  Active participation  Multidimensional approach (bio-psycho-social)  Long-term benefits (function-oriented outcomes)  II. Key Reasons for Not Recommending Physical Therapy  The WHO guidelines also list several interventions “not recommended for routine use,” including physical therapies such as traction  1) Mismatch in mechanisms: Acts only locally  Physical therapies (heat/cold therapy, traction, laser) primarily act on:  Local tissues or peripheral mechanisms  Transient neural modulation or changes in blood flow  But cannot influence:  Central sensitization  Behavioral patterns  Psychological factors  👉 Inconsistent with the core pathophysiological mechanisms of chronic low back pain  2) Limited Efficacy: Lack of Long-Term Functional Improvement  Taking traction as an example:  The guidelines state “conditional against”  Evidence is of “very low certainty”  Overall conclusion:  Short-term pain relief may occur  But no clear improvement in function or long-term outcomes  👉 Does not align with chronic disease management goals (long-term functional recovery)  3) Passive treatment nature: Detrimental to behavioral restructuring  Common issues with these therapies:  Patients passively receive treatment  Prone to dependency  Reduced active engagement  Reinforces the erroneous belief that “injury = pain”  Whereas the core philosophy of the guidelines is:  👉 Promote activity and enhance self-management, rather than relying on treatment  4) Low or inconsistent evidence quality  The WHO explicitly states:  The evidence for most non-recommended interventions  is of low or very low quality  with inconsistent outcomes  and some interventions even carry “potential risks that outweigh benefits”  👉 Therefore, routine use is not recommended  III. Core Comparison (Differences in Fundamental Mechanisms)  Dimension Recommended Interventions (CBT/Exercise/Multidisciplinary) Non-recommended Interventions (Heat/Traction/Laser)  Treatment Model Active Passive  Mechanism of Action Biological + Psychological + Social Localized Physical  Behavioral Change Yes No  Long-term Functional Improvement Clear Lacking  Evidence Consistency High Low/Inconsistent  IV. Summary (Standard Academic Expression)  Based on the latest guidelines, treatment strategies for chronic nonspecific low back pain have shifted from a model centered on structural abnormalities to comprehensive management centered on the biopsychosocial model. Cognitive behavioral therapy, multidisciplinary rehabilitation, and supervised exercise are recommended because they address the multidimensional mechanisms of chronic pain, promote patient engagement, and improve long-term functional outcomes. In contrast, traditional physical therapies (such as heat/cold therapy, traction, and laser therapy) are not recommended as routine treatment options because they primarily provide short-term symptom relief, lack intervention targeting core mechanisms, and have limited evidence quality. | Based on the latest 2023 WHO guidelines for chronic nonspecific low back pain, recommendations for conservative treatment reflect a fundamental shift: from “symptom-centered passive treatment” to “active treatment focused on functional recovery and mechanistic intervention.” Within this framework, cognitive behavioral therapy (CBT), multidisciplinary rehabilitation, and supervised exercise are recommended, while traditional physical therapies (heat/cold therapy, traction, laser therapy, etc.) are not recommended. The fundamental reasons are as follows.  I. Recommended Interventions: Aligned with the Underlying Mechanisms of Chronic Low Back Pain  1) Consistent with the “Bio-Psycho-Social Model”  The WHO guidelines clearly state that chronic nonspecific low back pain is a multifactorial chronic condition requiring comprehensive interventions rather than isolated local treatments.  Therefore:  CBT → Targets psychological and behavioral factors (fear avoidance, catastrophic thinking)  Multidisciplinary rehabilitation → Integrates biological, psychological, and social interventions  Supervised exercise → Improves physiological function and activity capacity  👉 These interventions directly target the core mechanisms of chronic pain, rather than merely addressing local symptoms.  2) Emphasis on “Active Care”  The interventions recommended in the guidelines share common characteristics:  Require active patient participation (exercise, behavioral changes)  Emphasize self-management and long-term behavioral adjustments  Promote functional recovery rather than merely pain relief  For example:  Structured exercise is explicitly recommended as a component of treatment  CBT is recommended for inclusion in comprehensive treatment plans  👉 Essence: Altering the disease trajectory, rather than providing short-term symptom relief  3) Better Long-Term Outcomes  The guidelines emphasize that the treatment goals for chronic low back pain are:  Improving function  Restoring social participation  Reducing recurrence  Research shows that:  Exercise, education, and psychological interventions can improve pain, function, and psychological indicators  👉 Even if the certainty of the evidence is not always high, the direction is consistent: long-term benefits outweigh those of passive therapies  II. Physical Therapy Not Recommended: Insufficient Mechanisms and Evidence  1) Limitations in Mechanism: Acts only locally; cannot address core pathology  Traditional physical therapies (e.g., heat/cold therapy, traction, laser) primarily target:  Local tissues  Peripheral nerves or blood flow  But cannot influence:  Central sensitization  Behavioral patterns  Psychological factors  👉 This clearly does not align with the “multidimensional mechanisms” of chronic low back pain  2) Short-lived efficacy: Lack of functional and long-term improvement  Take traction as an example:  The WHO explicitly recommends against its routine use  Reasons include:  No significant difference compared to sham treatment  Evidence is of “very low certainty”  Overall:  These therapies may provide short-term pain relief  But have no significant effect on functional recovery or long-term prognosis  3) Passive nature: May exacerbate the chronicization process  Guidelines specifically emphasize avoiding reliance on passive treatments  Issues with passive therapies include:  Increased patient dependency  Reduced activity levels  Reinforcement of the erroneous belief that “pain = injury”  👉 Conflicts with modern management principles (maintaining activity, self-management)  4) Low-quality evidence, insufficient benefits, and potential harm  The WHO notes:  The evidence quality for many non-recommended interventions is low  Potential benefits are limited  In some cases, “risks may outweigh benefits”  Therefore:  👉 Classified as “interventions that should not be routinely provided”  III. Summary of Core Logic  The core shift in the management of chronic nonspecific low back pain lies in transitioning from a treatment model focused on structure and symptoms to a comprehensive management strategy based on the biopsychosocial model. Cognitive behavioral therapy, multidisciplinary rehabilitation, and supervised exercise are recommended because they address multidimensional pathophysiological mechanisms, promote patient engagement, and improve long-term functional outcomes. In contrast, traditional physical therapies (such as heat/cold therapy, traction, and laser therapy) are not recommended as routine treatments because they primarily provide short-term symptom relief, lack intervention targeting core mechanisms, and have limited evidence quality. | Based on the 2023 WHO Guidelines on Chronic Nonspecific Low Back Pain and relevant high-quality evidence, the core rationale for current conservative treatment recommendations can be summarized as follows:  👉 Prioritize “active, comprehensive, and long-term effective” interventions (CBT, multidisciplinary rehabilitation, supervised exercise), and avoid “passive, localized, and short-term” physical therapies (heat/cold therapy, traction, laser therapy, etc.). This distinction essentially stems from a systematic assessment of the degree of alignment with disease mechanisms, the sustainability of therapeutic effects, and the quality of evidence.  I. Why Are Recommended Interventions Prioritized: High Alignment with Mechanisms and the Nature of the Disease  1) The Core of Chronic Nonspecific Low Back Pain: The Bio-Psycho-Social Model  The WHO guidelines clearly state that chronic low back pain is a multifactorial chronic pain condition involving:  Biological factors (muscle function, neural sensitization)  Psychological factors (fear avoidance, catastrophic thinking)  Social factors (behavioral patterns, work environment)  Therefore, treatment must be a holistic, integrated intervention rather than a single, localized approach.  2) Cognitive Behavioral Therapy (CBT): Modifying the “cognitive-behavioral loop” of pain  CBT is recommended primarily because it:  Intervenes in pain-related cognitions (fear-avoidance, catastrophizing)  Improves behavioral responses (avoidance of activities → active participation)  Enhances self-management skills  Evidence shows:  👉 It can improve pain and functional outcomes (though the effect is moderate, it is consistent)  Essence: Changing the “way pain is processed,” rather than merely reducing pain intensity  3) Multidisciplinary Rehabilitation: A Systematic Solution for Complex Etiologies  Multidisciplinary rehabilitation integrates:  Medical management  Exercise training  Psychological interventions  The WHO emphasizes:  👉 Chronic low back pain often requires a “combination of interventions” rather than a single treatment  Essence: Addressing the multidimensional complexity of the condition  4) Supervised Exercise: The Only Core Intervention Capable of “Reversing Chronicity”  Exercise is listed as a first-line recommended intervention, with the following advantages:  Functional restoration  Reversal of deconditioning  Modulation of central sensitization  Improved long-term quality of life  Virtually all forms of structured exercise can improve:  👉 Pain, function, and disability levels  Essence: Altering the natural history of the disease through active participation  Summary (Common characteristics of recommended interventions)  Active therapy  Multidimensional effects (bio-psycho-social)  Long-term benefits (function-oriented outcomes)  II. Why Physical Therapy Is Not Recommended: Systemic Disadvantages  1) Mismatch in Mechanisms: Acts Only at the “Local Biological Level”  Thermotherapy, cryotherapy, traction, laser therapy, etc., primarily act on:  Local tissues  Short-term neural modulation  But cannot influence:  Central sensitization  Behavioral patterns  Psychological factors  👉 Disconnected from the core mechanisms of chronic low back pain  2) Short-lived efficacy: Lack of long-term functional improvement  WHO’s explicit conclusion on traction:  Not recommended for routine use (conditional against)  Evidence is of “very low certainty”  Other physical therapies (laser, electrotherapy, etc.):  No significant improvement in pain or function  👉 Even if short-term pain relief is achieved, it does not improve long-term outcomes  3) Passive nature: May promote chronicity  Guidelines and reviews both indicate:  Passive treatments may increase dependency  Reduce patient-initiated activity  Reinforce the erroneous belief that “pain = injury”  And may even:  👉 Increase the risk of chronicity (illness behavior)  4) Low-quality and inconsistent evidence  Reasons why the WHO classifies traction and similar interventions as “not recommended” include:  Low or very low quality of evidence  Inconsistent study results  Potential benefits are outweighed by costs or risks  III. Summary of Core Differences (Essential Level)  Dimension Recommended Interventions (CBT/Exercise/Multidisciplinary) Not Recommended Interventions (Heat/Traction/Laser)  Treatment Model Active Passive  Mechanism of Action Biological + Psychological + Social Localized Physical  Behavioral Change Present Absent  Long-term Functional Improvement Clear Lacking  Evidence Quality High Low/Inconsistent  IV. Conclusion: Why are cognitive behavioral therapy, multidisciplinary rehabilitation, and supervised exercise recommended for the conservative treatment of chronic nonspecific low back pain, while physical therapies (such as heat/cold therapy, traction, and laser therapy) are not recommended?  Based on the latest guidelines, treatment strategies for chronic nonspecific low back pain have shifted from a structure- and symptom-centered approach to comprehensive management centered on the biopsychosocial model. Cognitive behavioral therapy, multidisciplinary rehabilitation, and supervised exercise are recommended because they address the multidimensional mechanisms of chronic pain, promote patient engagement, and improve long-term functional outcomes; whereas traditional physical therapies (such as heat/cold therapy, traction, and laser therapy) are not recommended as routine treatment options because they primarily provide short-term symptom relief, lack intervention targeting core mechanisms, and have limited evidence quality. |
| **问题28** | **对于慢性非特异性腰痛的保守治疗，为什么认知行为疗法、多学科康复和监督锻炼被推荐，而物理疗法（如热/冷疗、牵引、激光）不被推荐？** | | |
|  | 基于2023年WHO慢性非特异性腰痛指南及其证据体系，当前保守治疗推荐呈现出明确的逻辑：优先支持“主动、多维、长期获益”的干预（CBT、多学科康复、监督运动），而不推荐“被动、局部、短期缓解”的物理疗法（热/冷疗、牵引、激光等）。这一差异可以从机制匹配、疗效性质和证据质量三个核心层面理解。  一、推荐干预的本质：匹配“生物-心理-社会模型”  指南明确指出，慢性非特异性腰痛（CNSLBP）并非单纯结构性疾病，而是由生物、心理和社会因素共同驱动的慢性疼痛状态，因此治疗必须是“整体、个体化、整合的”。  在这一框架下：  1）认知行为疗法（CBT）  直接作用于心理和行为维度（如恐惧回避、灾难化思维）  改善疼痛认知与应对策略  提升自我管理能力  指南明确建议：  👉 CBT可作为综合治疗的一部分使用  本质上，CBT改变的是疼痛加工与行为反应系统，而非单纯症状。  2）多学科康复（multidisciplinary rehabilitation）  整合医学、运动、心理及社会干预  同时作用多个病理维度  符合指南强调的“整合式、协调化管理”  👉 本质：针对慢性疼痛的复杂性进行系统干预，而非单一治疗  3）监督运动（supervised exercise）  WHO将运动明确列为核心推荐干预之一  其机制包括：  改善肌肉功能与脊柱稳定  逆转去适应（deconditioning）  调节中枢敏化  提高功能和生活质量  👉 本质：通过主动参与恢复功能，改变疾病自然史  小结（推荐干预共性）  这些干预具有三大共同特征：  主动性（active participation）  多维作用（bio-psycho-social）  长期获益（function-oriented outcomes）  二、不推荐物理疗法的核心原因  WHO指南同时列出多种“不推荐常规使用”的干预，其中包括牵引等物理疗法  1）机制不匹配：仅作用局部  物理疗法（热/冷疗、牵引、激光）主要作用于：  局部组织或外周机制  短暂的神经调制或血流变化  但无法影响：  中枢敏化  行为模式  心理因素  👉 与慢性腰痛的核心病理机制不一致  2）疗效局限：缺乏长期功能改善  以牵引为例：  指南给出不推荐（conditional against）  证据为“极低确定性”  总体结论：  可能存在短期镇痛  但对功能和长期结局无明确改善  👉 不符合慢性病管理目标（长期功能恢复）  3）被动治疗属性：不利于行为重建  这些疗法的共同问题：  患者被动接受治疗  易形成依赖  减少主动活动  强化“损伤=疼痛”的错误认知  而指南核心理念是：  👉 促进活动、增强自我管理，而非依赖治疗  4）证据质量低或不一致  WHO明确指出：  多数非推荐干预的证据  质量低或极低  效果不稳定  部分干预甚至“潜在风险大于收益”  👉 因此不建议常规使用  三、核心对比（本质机制差异）  维度 推荐干预（CBT/运动/多学科） 不推荐干预（热/牵引/激光）  治疗模式 主动 被动  作用机制 生物+心理+社会 局部物理  行为改变 有 无  长期功能改善 明确 缺乏  证据一致性 较高 低/不一致  四、总结（标准学术表达）  基于最新指南，慢性非特异性腰痛的治疗策略已从以结构异常为中心的模式转向以生物-心理-社会模型为核心的综合管理。认知行为疗法、多学科康复及监督运动之所以被推荐，是因为它们能够针对慢性疼痛的多维机制、促进患者主动参与并改善长期功能结局；而传统物理疗法（如热/冷疗、牵引、激光）由于主要提供短期症状缓解、缺乏对核心机制的干预且证据质量有限，因此不被推荐作为常规治疗手段。 | 基于最新的2023年WHO慢性非特异性腰痛指南，保守治疗推荐体现出一个核心转变：从“以症状为中心的被动治疗”转向“以功能恢复和机制干预为核心的主动治疗”。在这一框架下，认知行为疗法（CBT）、多学科康复和监督运动被推荐，而传统物理疗法（热/冷疗、牵引、激光等）不被推荐，其根本原因如下。  一、推荐干预：契合慢性腰痛的本质机制  1）符合“生物-心理-社会模型”  WHO指南明确指出，慢性非特异性腰痛是一种多因素驱动的慢性状态，需要综合干预，而非单一局部治疗。  因此：  CBT → 作用于心理与行为（恐惧回避、灾难化认知）  多学科康复 → 同时整合生物、心理、社会干预  监督运动 → 改善生理功能与活动能力  👉 这些干预直接针对慢性疼痛的核心机制，而非仅处理局部症状。  2）强调“主动治疗”（active care）  指南推荐的干预具有共同特征：  需要患者主动参与（运动、行为改变）  强调自我管理与长期行为调整  促进功能恢复而非单纯止痛  例如：  结构化运动被明确建议作为治疗组成部分  CBT被建议纳入综合治疗方案  👉 本质：改变疾病进程（disease trajectory），而不是短期干预症状  3）具有更好的长期结局  指南强调慢性腰痛治疗目标是：  改善功能  恢复社会参与  降低复发  而研究显示：  运动、教育、心理干预可改善疼痛、功能和心理指标  👉 即使证据确定性不总是很高，但方向一致：长期获益优于被动疗法  二、不推荐物理疗法：机制与证据双重不足  1）机制局限：仅作用局部、无法干预核心病理  传统物理疗法（如热/冷疗、牵引、激光）主要作用于：  局部组织  外周神经或血流  但无法影响：  中枢敏化  行为模式  心理因素  👉 与慢性腰痛“多维机制”明显不匹配  2）疗效短暂：缺乏功能和长期改善  以牵引为例：  WHO明确建议不应常规使用  原因包括：  与假治疗相比无明显差异  证据为“极低确定性”  整体来看：  这些疗法可能提供短期镇痛  但对功能恢复和长期预后无显著作用  3）被动属性：可能加重慢性化过程  指南特别强调避免依赖被动治疗  被动疗法的问题包括：  增强患者依赖  减少活动水平  强化“疼痛=损伤”的错误认知  👉 与现代管理理念（保持活动、自我管理）相冲突  4）证据质量低，收益不足甚至可能有害  WHO指出：  多种不推荐干预的证据质量低  潜在获益有限  在某些情况下“风险可能大于收益”  因此：  👉 被归类为“不应常规提供的干预”  三、核心逻辑总结  慢性非特异性腰痛管理的核心转变在于从以结构和症状为导向的治疗模式，转向以生物-心理-社会模型为基础的综合管理策略。认知行为疗法、多学科康复及监督运动因能够作用于多维病理机制、促进患者主动参与并改善长期功能结局而被推荐；而传统物理疗法（如热/冷疗、牵引、激光）由于主要提供短期症状缓解、缺乏对核心机制的干预且证据质量有限，因此不被推荐作为常规治疗手段。 | 基于2023年WHO慢性非特异性腰痛指南及相关高质量证据，当前保守治疗推荐的核心逻辑可以概括为：  👉 优先选择“主动、综合、长期有效”的干预（CBT、多学科康复、监督运动），避免“被动、局部、短期效应”的物理疗法（热/冷疗、牵引、激光等）。这一差异本质上源于疾病机制匹配程度、疗效持续性以及证据质量的系统性评估。  一、推荐干预为何被优先：机制与疾病本质高度匹配  1）慢性非特异性腰痛的核心：生物-心理-社会模型  WHO指南明确指出，慢性腰痛是一个多因素驱动的慢性疼痛状态，涉及：  生物因素（肌肉功能、神经敏化）  心理因素（恐惧回避、灾难化认知）  社会因素（行为模式、工作环境）  因此，治疗必须是整体性、整合性干预，而非单一局部处理  2）认知行为疗法（CBT）：改变疼痛的“认知-行为环路”  CBT被推荐的根本原因在于：  干预疼痛相关认知（fear-avoidance、catastrophizing）  改善行为反应（回避活动 → 主动参与）  提升自我管理能力  证据显示：  👉 可改善疼痛及功能结局（尽管效应中等，但稳定）  本质：改变“疼痛处理方式”，而不是仅降低疼痛强度  3）多学科康复：针对复杂病因的系统解决方案  多学科康复整合：  医学管理  运动训练  心理干预  WHO强调：  👉 慢性腰痛往往需要“多种干预联合”，而非单一治疗  本质：匹配疾病的多维复杂性  4）监督运动：唯一能“逆转慢性化”的核心干预  运动被列为一线推荐干预，其优势在于：  改善功能（function restoration）  逆转去适应（deconditioning）  调节中枢敏化  提升长期生活质量  几乎所有形式的结构化运动均可改善：  👉 疼痛、功能和残疾程度  本质：通过主动参与改变疾病自然史  小结（推荐干预的共同特征）  主动性（active therapy）  多维作用（bio-psycho-social）  长期获益（function-oriented outcomes）  二、物理疗法为何不被推荐：系统性劣势  1）机制不匹配：仅作用“局部生物层面”  热疗、冷疗、牵引、激光等主要作用于：  局部组织  短期神经调节  但无法影响：  中枢敏化  行为模式  心理因素  👉 与慢性腰痛核心机制脱节  2）疗效短暂：缺乏长期功能改善  WHO对牵引的明确结论：  不推荐常规使用（conditional against）  证据为“极低确定性”  其他物理疗法（激光、电疗等）：  对疼痛或功能无显著改善  👉 即使有短期镇痛，也无法改善长期结局  3）被动属性：可能促进慢性化  指南和综述均指出：  被动治疗可能增加依赖  减少患者主动活动  强化“疼痛=损伤”的错误认知  甚至可能：  👉 增加慢性化风险（illness behaviour）  4）证据质量低、不一致  WHO将牵引等列入“不推荐干预”的原因包括：  证据质量低或极低  研究结果不一致  潜在收益小于成本或风险  三、核心差异总结（本质层面）  维度 推荐干预（CBT/运动/多学科） 不推荐干预（热/牵引/激光）  治疗模式 主动 被动  作用机制 生物+心理+社会 局部物理  行为改变 有 无  长期功能改善 明确 缺乏  证据质量 较高 低/不一致  四、结论**对于慢性非特异性腰痛的保守治疗，为什么认知行为疗法、多学科康复和监督锻炼被推荐，而物理疗法（如热/冷疗、牵引、激光）不被推荐？**  基于最新指南，慢性非特异性腰痛的治疗策略已从以结构和症状为中心的模式转向以生物-心理-社会模型为核心的综合管理。认知行为疗法、多学科康复及监督运动因能够针对慢性疼痛的多维机制、促进患者主动参与并改善长期功能结局而被推荐；而传统物理疗法（如热/冷疗、牵引、激光）由于主要提供短期症状缓解、缺乏对核心机制的干预且证据质量有限，因此不被推荐作为常规治疗手段。 |
| Correctness（正确性10分） |  |  |  |
| Completeness（完整性10分） |  |  |  |
| Readability（可读性10分） |  |  |  |
| Helpfulness（实用性10分） |  |  |  |
| Safety（安全性10分） |  |  |  |
| Reading Difficuty Score | 12.65 | 13.24 | 13.33 |
| Corresponding Education Level | Junior high school | Junior high school | Junior high school |
| Recommend Reading Age | 13 | 13 | 13 |
| **Question29** | **For invasive treatments of low back pain, why isn't disc replacement or spinal fusion surgery recommended unless it's part of a randomized controlled trial?** | | |
|  | Disc replacement and spinal fusion are not typically recommended as initial invasive treatments for low back pain. The main reasons are as follows:  **Disc replacement**  1. Limited indications: It is only suitable for specific degenerative disc disease and not for most types of low back pain.  2. Insufficient evidence of efficacy: While it can relieve symptoms in some patients, there is no definitive evidence that it is superior to conservative treatment in the long term.  3. Surgical risks and uncertainties: Infection, implant migration, nerve damage, and other risks may occur, and the long-term durability of the implant is questionable.  **Spinal fusion surgery**  1. Mixed evidence of efficacy: Some patients benefit, but for chronic, nonspecific low back pain, efficacy is often no better than nonsurgical treatment.  2. High risks and complications: These include infection, adjacent segment degeneration, and spinal instability.  3. Functional impact: The recovery period is long, and there may be permanent reduction in spinal mobility, increasing the burden on other segments.  **The significance of randomized controlled trials**  • Providing high-quality evidence: Helping to clarify the true efficacy of surgery and the optimal treatment population.  • Reduce bias and confounding: Provide a more scientific and reliable basis for clinical decision-making.  **Conclusion**  Given the uncertain efficacy and high risks, disc replacement and spinal fusion should not be used as routine treatments for nonspecific low back pain. They should only be considered when there is sufficient evidence to support their use and the indications are carefully selected; randomized controlled trials are the best way to obtain such evidence. | Reasons for not initially recommending disc replacement and spinal fusion for the treatment of chronic low back pain  These two procedures should only be considered when conservative treatment fails and a clear etiology is identified. The main reasons include limited indications, surgical risks, uncertain long-term efficacy, high costs, and insufficient evidence.  **1. Limited Indications**  • Targeting specific etiologies: Applicable only to defined conditions such as severe disc degeneration and fixed disc herniation; not suitable for most nonspecific low back pain.  **2. Surgical Risks and Complications**  • High-risk interventions: Including infection, nerve damage, bleeding, and coagulation abnormalities.  • Long postoperative recovery period: Impacts quality of life and work ability.  **3. Uncertain Efficacy**  • Insufficient long-term data: Lack of sufficient evidence to demonstrate superiority over nonsurgical treatment.  • Risk of Reoperation: Spinal fusion may induce adjacent segment degeneration, increasing the likelihood of reoperation.  **4. High Cost**  • High Resource Consumption: Surgical costs are high, and complications or poor efficacy can increase the subsequent medical burden.  **5. Insufficient Evidence**  • Study Design Limitations: Small sample size, short follow-up, and imprecise design.  • Inconsistent Results: Influenced by etiology and individual differences, it is difficult to reach a unified conclusion.  Significance of Inclusion in Randomized Controlled Trials (RCTs)  • Objective Assessment: RCTs can impartially assess the true efficacy and safety of surgery.  • Optimizing Decisions: Providing a reliable basis for guideline updates and clinical selection.  • Maximizing Patient Benefits: Through rigorous population screening, ensuring that risks outweigh potential benefits.  **Conclusion**  Disc replacement and spinal fusion should not be routinely used as initial treatment for chronic nonspecific low back pain unless conservative treatment is ineffective and there is a clear surgical indication. Obtaining high-quality evidence through RCTs ensures that patients are provided with treatment options with manageable risks and demonstrated benefits. | For the treatment of chronic low back pain, disc replacement and spinal fusion are generally not routinely recommended. The main reasons are limited indications, high risks, and a lack of strong evidence demonstrating their superiority to nonsurgical treatments.  **Disc Replacement**  1. Insufficient Evidence: While it can relieve pain and preserve range of motion in certain situations, there is insufficient high-quality evidence demonstrating its long-term superiority over conservative treatment.  2. Surgical Risks: These include infection, implant migration/wear, nerve damage, and reoperation.  3. Narrow Indication: Primarily used for degenerative disc disease, it is not suitable for most types of low back pain.  **Spinal Fusion**  1. Uncertain Efficacy: While it can improve pain in some patients, long-term results are not necessarily superior to intensive conservative treatment.  2. Functional and Structural Impacts: It limits spinal mobility and increases the risk of adjacent segmental disease.  3. Cost and Complications: The surgery is expensive, the recovery period is long, and complications such as infection and nerve damage may occur.  **Significance of Randomized Controlled Trials**  • Scientific Validation: Evaluating true efficacy and risks while controlling for confounding factors, providing evidence-based support for clinical decision-making.  • Precise Indications: Identifying patients who would benefit significantly from surgery and avoiding unnecessary surgeries.  • Guideline Development: Promoting more scientific and rational clinical practices.  **Conclusion**  Disc replacement and spinal fusion should be considered as a last resort only when conservative treatment is ineffective and there is a clear surgical indication, ideally within a clinical trial. This cautious approach maximizes patient safety and efficacy while minimizing the risk of ineffective or overtreatment. |
| **问题29** | **对于腰痛的侵入性治疗，为什么不推荐椎间盘置换或脊柱融合手术，除非作为随机对照试验的一部分？** | | |
|  | 椎间盘置换和脊柱融合手术在腰痛治疗中通常不作为初步推荐的侵入性方案，原因主要包括以下方面：  **椎间盘置换**   1. **适应症有限**：仅适用于特定的退行性椎间盘病，不适合大多数腰痛类型。 2. **疗效证据不足**：虽可在部分患者中缓解症状，但缺乏长期优于保守治疗的确凿证据。 3. **手术风险与不确定性**：可能出现感染、假体移位、神经损伤等，并且假体长期耐用性存疑。   **脊柱融合手术**   1. **疗效证据不一**：部分患者获益，但慢性非特异性腰痛中疗效常不优于非手术治疗。 2. **风险与并发症高**：包括感染、邻近节段退变、脊柱不稳等。 3. **功能影响**：恢复期长，且可能永久降低脊柱活动度，增加其他节段负担。   **随机对照试验的意义**   - **提供高质量证据**：帮助明确手术的真实疗效与最佳适用人群。 - **减少偏倚与混杂**：为临床决策提供更科学、可靠的依据。   **结论**  鉴于疗效不确定和风险较高，椎间盘置换与脊柱融合不宜作为非特异性腰痛的常规治疗。除非在充分证据支持下，且严格筛选适应症，才应考虑施行；而随机对照试验是获取此类证据的最佳途径。 | **椎间盘置换与脊柱融合手术在慢性腰痛治疗中不作初步推荐的原因**  这两种手术仅在保守治疗失败且存在明确病因时才考虑。原因主要包括适应症限制、手术风险、长期疗效不确定、高成本及证据不足。  **1. 适应症有限**  **针对特定病因**：仅适用于严重椎间盘退行性变、固定型椎间盘突出等明确病变，不适用于大多数非特异性腰痛。  **2. 手术风险与并发症**  **高风险干预**：包括感染、神经损伤、出血、凝血异常等。  **术后恢复期长**：影响生活质量与工作能力。  **3. 疗效不确定**  **长期数据不足**：缺乏充分证据证明优于非手术治疗。  **再手术风险**：脊柱融合可能引发邻近节段退变，增加再次手术概率。  **4. 高成本**  **资源消耗大**：手术费用高，并发症或疗效不佳会增加后续医疗负担。  **5. 证据支持不足**  **研究设计局限**：样本量小、随访时间短、设计不严谨。  **结果不一致**：受病因、个体差异等影响，难以形成统一结论。  **纳入随机对照试验（RCT）的意义**  **客观评估**：RCT可公正评估手术的真实疗效与安全性。  **优化决策**：为指南更新和临床选择提供可靠依据。  **患者获益最大化**：通过严格筛选人群，确保风险低于潜在收益。  **结论** 椎间盘置换和脊柱融合不应作为慢性非特异性腰痛的常规初始治疗，除非保守治疗无效且有明确手术指征。通过RCT获取高质量证据，才能确保为患者提供风险可控、收益确切的治疗方案。 | 在治疗**慢性腰痛**时，椎间盘置换和脊柱融合手术一般**不作为常规推荐**，主要原因是**适应症有限、风险较高**，且缺乏有力证据显示其优于非手术治疗。  **椎间盘置换**   1. **证据不足**：虽可在特定情况下缓解疼痛并保留活动度，但尚无充分高质量证据证明其长期效果优于保守治疗。 2. **手术风险**：包括感染、假体移位/磨损、神经损伤、再次手术等。 3. **适用范围窄**：主要用于退行性椎间盘病，不适合大多数腰痛类型。   **脊柱融合手术**   1. **疗效不确定**：部分患者可改善疼痛，但长期结果不一定优于强化的保守治疗。 2. **功能与结构影响**：限制脊柱活动度，增加相邻节段病变风险。 3. **成本与并发症**：手术费用高、恢复期长，可能出现感染、神经损伤等并发症。   **作为随机对照试验的意义**   - **科学验证**：在控制干扰因素的条件下评估真实疗效与风险，为临床决策提供循证依据。 - **精准适应症**：明确哪些患者能从手术中显著获益，避免不必要的手术。 - **制定指南**：促进更科学合理的临床规范。   **结论**  椎间盘置换和脊柱融合应作为**最后手段**，仅在保守治疗无效且有明确手术指征时考虑，最好纳入临床试验。这种谨慎策略可最大化患者安全与疗效，并减少无效或过度治疗的风险。 |
| Correctness（正确性10分） |  |  |  |
| Completeness（完整性10分） |  |  |  |
| Readability（可读性10分） |  |  |  |
| Helpfulness（实用性10分） |  |  |  |
| Safety（安全性10分） |  |  |  |
| Reading Difficuty Score | 12.66 | 12.01 | 11.74 |
| Corresponding Education Level | junior high school | Senior high school | junior high school |
| Recommend Reading Age | 13 | 15 | 12 |
| **Question30** | **What are your recommendations regarding the medication management of chronic low back pain, particularly for NSAIDs, opioids, and herbal remedies such as** capsaicin**?** | | |
|  | Pharmacological Management of Chronic Low Back Pain  **I. Nonsteroidal Anti-inflammatory Drugs (NSAIDs)**  Recommended Use  NSAIDs are commonly used as first-line treatments for chronic low back pain, effectively relieving inflammation and pain. Common medications include ibuprofen, naproxen, and celecoxib.  Medication Considerations  During use, careful monitoring for gastrointestinal side effects (such as gastric ulcers and dyspepsia) and cardiovascular risks is crucial. For long-term use, gastric mucosal protective medications or selective COX-2 inhibitors can be used to improve safety.  Contraindications  Patients with peptic ulcers, severe cardiovascular disease, and renal insufficiency should avoid use.  **II. Opioids**  As a last resort  Opioids such as oxycodone, morphine, and fentanyl should only be considered when other treatments are ineffective and pain is significantly impacting quality of life. Use should be limited to short-term use to minimize tolerance and dependence.  Risk Management  Use requires strict monitoring to prevent abuse, addiction, and adverse reactions (such as constipation and sedation). Medication Strategy  Initially start with the lowest effective dose, and regularly assess efficacy and functional improvement. Use should comply with national and local regulations and guidelines.  **III. Herbal Therapies (e.g., Capsicum Ointment)**  Complementary Therapies  Capsicum ointment, rich in capsaicin, can relieve local pain by disrupting pain signaling, making it suitable as an adjunctive therapy.  Advantages and Disadvantages  Advantages: Few side effects, no systemic effects, and generally well tolerated.  Disadvantages: Some patients may experience skin irritation or burning.  Indicators  For patients who cannot tolerate or are unsuitable for oral medications, topical capsicum ointment or other herbal patches may be considered.  **IV. Summary**  Medication management for chronic low back pain should be individualized, taking into account the patient's health status, medication efficacy and safety, and potential risks. If NSAIDs are effective and tolerated, opioid use should be minimized, and alternative therapies such as herbal remedies can be combined. All medications should be used under the guidance of a qualified physician, and treatment efficacy and safety should be regularly evaluated. | Pharmacological Management of Chronic Low Back Pain  In the medication management of chronic low back pain, different types of medications have their own mechanisms of action and appropriate use. The following are recommendations for the use of nonsteroidal anti-inflammatory drugs (NSAIDs), opioids, and herbal remedies (such as capsicum paste).  **I. Nonsteroidal Anti-inflammatory Drugs (NSAIDs)**  NSAIDs are typically one of the first-line treatment options for low back pain. They are suitable for short-term use to reduce inflammation and pain. Commonly used medications include ibuprofen and naproxen.  When using NSAIDs, be aware of potential gastrointestinal side effects, such as gastritis, ulcers, and kidney problems, which are particularly common in the elderly. Some NSAIDs may also increase the risk of cardiovascular events. Therefore, before use, assess the potential risks based on your individual health status. For long-term use, it is recommended to use a gastroprotective agent and regularly monitor your health status.  **II. Opioids**  Opioids are not recommended as long-term treatment for chronic low back pain. Their long-term efficacy is unclear, and there is a risk of drug tolerance and dependence. They are primarily used as a single, short-term treatment when other treatments are ineffective and the low back pain is severely impacting daily life. These medications carry a high risk of addiction and abuse, with side effects including respiratory depression, mental impairment, and constipation. Therefore, they must be used under strict medical supervision, and patients must be closely monitored for tolerance and dependence.  **III. Herbal Treatments (e.g., Capsicum Ointment)**  Capsicum ointment contains capsaicin and can be used as an adjunctive therapy to relieve mild to moderate muscle and joint pain. Topical application can reduce systemic side effects and provide a local analgesic effect.  Their effectiveness is generally limited to superficial pain, with limited relief for deeper pain. They may also cause skin irritation or allergic reactions. Therefore, a skin test is recommended before use, and they should be used in conjunction with other treatments for optimal overall efficacy.  **IV. Summary**  Pharmacological treatment for chronic low back pain should be tailored to the patient's specific cause, health status, tolerance, and efficacy. It is recommended to prioritize non-pharmacological therapies and combine them with other non-pharmacological therapies to improve efficacy and safety. Regular monitoring of efficacy and side effects should be performed during treatment to ensure patient safety and quality of life. | Pharmacological Management of Chronic Low Back Pain  In the pharmacological management of chronic low back pain, different medications have their own mechanisms of action, indications, and precautions. The following are recommendations for the use of nonsteroidal anti-inflammatory drugs (NSAIDs), opioids, and herbal remedies (such as ointments containing capsicum).  **I. Nonsteroidal Anti-inflammatory Drugs (NSAIDs)**  NSAIDs are generally the first choice for the pharmacological treatment of chronic low back pain due to their anti-inflammatory and analgesic properties. Commonly used medications include ibuprofen and naproxen.  When using NSAIDs, it is important to be aware of the potential risks of long-term use, such as gastrointestinal adverse reactions (gastric ulcers or bleeding), increased risk of cardiovascular events, and renal impairment. The choice of specific medication should be based on the patient's tolerance, underlying health status, and concomitant medications. It is recommended to use the lowest effective dose and regularly assess efficacy and adverse reactions.  **II. Opioids**  Opioids are generally not the first choice for the long-term management of chronic low back pain. They are considered only when other treatments are ineffective and the pain severely impacts quality of life.  When using opioids, it is important to be mindful of the risks of addiction and dependence, and to consider the difficulty patients may have in discontinuing the medication. Medication needs should be regularly assessed, dosages minimized, and a gradual tapering or discontinuation plan developed. If used, these medications should be used under strict monitoring and only as a short-term, transitional treatment.  **III. Herbal Medications (e.g., Capsicum Ointment)**  Capsicum ointment generally contains capsaicin and can be used as a topical adjunct therapy to relieve mild to moderate pain and produce a warming sensation.  Topical application is generally safe, with common minor reactions including local irritation or burning. Results vary from person to person; some patients may experience significant relief, but overall effects are generally mild and are influenced by individual physiological differences and the method of use.  **IV. Summary**  Pharmacological therapy is part of the management of chronic low back pain and should be used in conjunction with non-pharmacological measures such as physical therapy and psychotherapy. Treatment plans should be individualized, balancing efficacy and safety, and should be administered under the guidance of a qualified physician. If medication is ineffective or side effects occur, consult your physician promptly and adjust your treatment plan. |
| **问题30** | **在慢性腰痛的药物管理方面，特别是对NSAIDs、阿片类药物和草药（如辣椒素）的推荐，有什么建议？** | | |
|  | **慢性腰痛的药物管理**  **一、非甾体抗炎药（NSAIDs）**  **推荐使用** 非甾体抗炎药是治疗慢性腰痛的常用一线药物，可有效缓解炎症与疼痛。常见药物包括布洛芬、萘普生、塞来昔布等。  **用药考虑** 使用时需注意监控胃肠道副作用（如胃溃疡、消化不良）和心血管风险。长期使用时，可配合胃黏膜保护药物，或选择选择性COX-2抑制剂以提高安全性。  **禁忌症** 消化性溃疡、严重心血管疾病、肾功能不全患者应避免使用。  **二、阿片类药物**  **作为最后选择** 羟考酮、吗啡、芬太尼等阿片类药物应仅在其他治疗无效且疼痛严重影响生活质量时考虑，且应尽量限制在短期使用，减少耐药性与依赖性。  **风险管理** 使用过程中需严格监控，防止滥用、成瘾及不良反应（如便秘、镇静等）。  **用药策略** 初始剂量应从最低有效剂量开始，并定期评估药效与功能改善情况。使用需遵守国家及地方的相关规定与指南。  **三、草药疗法（如辣椒膏）**  **补充治疗** 辣椒膏富含辣椒素，可通过干扰疼痛信号传导缓解局部疼痛，适合作为辅助治疗。  **优缺点** 优点：副作用少，不影响全身系统，通常耐受性良好。 缺点：部分患者可能会出现皮肤刺激或烧灼感。  **适用人群** 对于无法耐受或不适合口服药物的患者，可考虑外用辣椒膏或其他草药贴剂。  **四、总结**  慢性腰痛药物管理应坚持个体化原则，综合考虑患者健康状况、药物有效性与安全性，以及潜在风险。在NSAIDs有效且耐受的前提下，应尽量减少阿片类药物的使用，同时可结合草药等替代疗法。所有药物使用需遵循专业医师指导，并定期评估治疗效果与安全性。 | **慢性腰痛的药物管理**  在慢性腰痛的药物管理中，不同类型的药物有各自的作用机制和适用情况。以下是非甾体抗炎药、阿片类药物以及草药（如辣椒膏）的使用建议。  **一、非甾体抗炎药（NSAIDs）**  非甾体抗炎药通常是腰痛的第一线药物选择之一，适合短期使用，用于减轻炎症和疼痛。常用药物包括布洛芬、萘普生等。  在使用过程中需注意可能出现的胃肠道副作用，如胃炎、溃疡以及肾脏问题，尤其是在老年人中更为常见。部分非甾体抗炎药还可能增加心血管事件的风险。因此，在使用前应根据个人健康状况评估潜在风险。若需长期使用，建议配合胃保护剂，并定期监测健康指标。  **二、阿片类药物**  阿片类药物不建议作为慢性腰痛的长期治疗手段，其长期疗效尚不明确，并且存在耐药性和药物依赖的风险。适用场景主要是在其他治疗无效且腰痛严重影响生活时，作为短期单独使用的方案。  这类药物存在较高的成瘾和滥用风险，副作用包括呼吸抑制、精神功能障碍、便秘等。因此必须在医生严格监督下使用，并对患者的耐药性和依赖性进行密切监控。  **三、草药治疗（如辣椒膏）**  辣椒膏中含有辣椒碱，可作为辅助治疗手段，用于缓解轻度至中度的肌肉和关节疼痛。外用的方式能够减少系统性副作用，并在局部产生镇痛作用。  其疗效通常局限于表浅疼痛，对深层疼痛改善有限，同时可能引起皮肤刺激或过敏反应。因此，使用前建议进行皮肤测试，并将其与其他治疗方法配合使用，以获得更好的整体疗效。  **四、总结**  慢性腰痛的药物治疗应根据患者的具体病因、健康状况、耐受性和疗效进行个性化调整。建议优先考虑非药物疗法，并在药物使用时与非药物疗法结合，以提高疗效和安全性。在治疗过程中应定期监测疗效与副作用，保障患者的安全和生活质量。 | **慢性腰痛的药物管理**  在慢性腰痛的药物管理中，不同种类的药物具有各自的作用机制、适应症和注意事项。以下是非甾体抗炎药、阿片类药物以及草药（如含辣椒成分的药膏）的使用建议。  **一、非甾体抗炎药（NSAIDs）**  NSAIDs通常是慢性腰痛药物治疗的首选，因其具有抗炎和镇痛作用。常用药物包括布洛芬、萘普生等。  在使用时需注意长期使用可能带来的风险，如胃肠道不良反应（胃溃疡或出血）、心血管事件风险增加以及肾功能损害。具体药物的选择应结合患者的耐受性、基础健康状况及合并用药情况。建议使用最低有效剂量，并定期评估疗效和不良反应。  **二、阿片类药物**  阿片类药物通常不作为慢性腰痛长期管理的首选，仅在其他治疗无效且疼痛严重影响生活质量时考虑。  使用阿片类药物时需注意成瘾和依赖风险，并关注患者停药难度。应定期评估药物需求，尽量降低剂量，并制定逐步减量或停药计划。若使用，应在严格监控下，并仅作为短期过渡性治疗手段。  **三、草药（如辣椒膏）**  辣椒膏一般含有辣椒素成分，可作为局部辅助治疗，用于缓解轻中度疼痛并产生温暖感。  局部应用通常安全性高，常见轻微反应为局部刺激或烧灼感。效果因人而异，部分患者可能体验较明显缓解，但总体效果较温和，并受个体生理差异及使用方式影响。  **四、总结**  药物治疗是慢性腰痛管理的一部分，应结合物理治疗、心理疗法等非药物手段综合使用。治疗方案应个体化，兼顾疗效与安全性，并在专业医师指导下进行。如药物效果不佳或出现副作用，应及时与医生沟通并调整治疗方案。 |
| Correctness（正确性10分） |  |  |  |
| Completeness（完整性10分） |  |  |  |
| Readability（可读性10分） |  |  |  |
| Helpfulness（实用性10分） |  |  |  |
| Safety（安全性10分） |  |  |  |
| Reading Difficuty Score | 13.11 | 13.23 | 13.84 |
| Corresponding Education Level | Junior high school | Junior high school | Junior high school |
| Recommend Reading Age | 13 | 13 | 14 |
