# Supplementary material for: Deficiencies in clinical reasoning of LLMs in low back pain management and remediation via prompt engineering: from performance evaluation to error diagnosis
Source: Front Artif Intell. 2026 May 25;9:1811701. doi: 10.3389/frai.2026.1811701 (PMC13243384; doi:10.3389/frai.2026.1811701)
Supplement: Supplementary file 1 [file Data_Sheet_1.zip › 补充材料/Supplementary materials 3/S2(GPT-5).docx]

| **Table S2 GPT-5 answers t30 questions** | | | | |
| --- | --- | --- | --- | --- |
| **Question1** | | **How do you define and categorize low back pain?** | | |
|  | | Low back pain (LBP) has a relatively clear medical definition and can be classified in various ways based on its causes, course, and pathological characteristics. Below, I have compiled a systematic overview of its definition and classification for academic or clinical use.  ________________________________________  I. Definition of Low Back Pain  Low back pain refers to pain **located in the lumbar or lumbosacral region (between the lower margin of the 12th rib and the gluteal cleft)**, often accompanied by pain radiating to the lower limbs, numbness, or other discomfort.  This is a symptom rather than a disease in itself and can be caused by a variety of factors.  Common accompanying manifestations:  • Localized aching, dull pain, sharp pain, or a burning sensation  • Limited mobility, stiffness  • Radiating pain in the lower limbs (e.g., sciatica)  • Sometimes accompanied by numbness or weakness  ________________________________________  II. Classification of Low Back Pain  1. Classification by Duration (Commonly Used Clinically)  • Acute low back pain: Duration <6 weeks  • Subacute low back pain: Duration 6–12 weeks  • Chronic low back pain: Duration >12 weeks  Acute low back pain is often caused by strain, sprain, acute disc herniation, etc.; chronic low back pain is often associated with degenerative changes, chronic inflammation, or structural abnormalities.  ________________________________________  2. Classification by Etiology (Most Important Clinical Classification)  (1) Non-specific Low Back Pain (Non-specific LBP)  • Accounts for 80%–90% of all low back pain  • No clear single structural cause; often related to muscle and ligament strain, poor posture, or weak core muscles.  • Usually resolves with conservative treatment.  (2) Specific LBP  Has a clear anatomical or pathological cause, including:  1. Degenerative diseases  o Disc degeneration, disc herniation  o Degenerative arthritis of the facet joints  o Spinal spondylolisthesis  2. Spinal deformities  o Scoliosis, kyphosis, etc.  3. Bone disorders  o Osteoporotic compression fractures  o Spinal tumors, metastases  4. Inflammatory diseases  o Ankylosing spondylitis, spinal infections (tuberculosis, suppurative inflammation)  5. Traumatic Lesions  o Vertebral fractures, ligament ruptures  (3) Neurogenic Low Back Pain  • Caused by compression or irritation of spinal nerve roots, such as nerve compression from a herniated disc or spinal stenosis.  • Often accompanied by radiating pain and numbness in the lower limbs.  ________________________________________  3. Classification by Nature of Pain (Pathophysiological Perspective)  • Mechanical low back pain (most common): Related to posture, weight-bearing, and movement; worsens with activity and improves with rest.  • Inflammatory low back pain: Pronounced morning stiffness; improves with activity but persists at night or at rest; commonly seen in ankylosing spondylitis.  • Referred low back pain: Low back pain caused by disease in adjacent organs (e.g., kidney stones, pelvic diseases).  ________________________________________  4. Classification by warning signs (Red Flag/Yellow Flag classification)  • Red Flags: Indicate the possible presence of serious underlying conditions (tumors, infections, fractures, neurological dysfunction) requiring immediate evaluation and management.  • Yellow Flags: Indicate that psychosocial factors (anxiety, depression, fear of movement) may affect recovery, requiring comprehensive intervention.  ________________________________________ | Low back pain (LBP) has a clear medical definition and is classified in various ways; it is typically defined based on three factors: clinical presentation, duration, and underlying mechanisms.  ________________________________________  I. Definition of Low Back Pain  Low back pain refers to pain in the region between the lower costal margin and the gluteal cleft, which may or may not be accompanied by symptoms radiating to the lower limbs (such as sciatica).  • It can be acute (such as sudden onset following a sprain) or chronic (long-term, recurrent episodes).  • It may present as simple localized pain or be accompanied by symptoms of nerve root compression (numbness, tingling, weakness).  • Clinically, low back pain is both a symptom and a manifestation of various diseases.  ________________________________________  II. Classification of Low Back Pain  1. Classification by Duration  • Acute low back pain: Symptoms lasting <6 weeks, often caused by injury or overuse.  • Subacute low back pain: Symptoms lasting 6–12 weeks.  • Chronic low back pain: Symptoms lasting >12 weeks, often accompanied by structural changes or central sensitization.  ________________________________________  2. Classification by Etiology and Pathogenesis  (1) Specific Low Back Pain (specific LBP)  • Has a clear structural or pathological cause, accounting for **<5%** of all low back pain.  • Common causes:  o Herniated disc, spinal stenosis  o Vertebral fractures (e.g., osteoporotic fractures)  o Spinal tumors or infections (e.g., spinal tuberculosis)  o Inflammatory spinal disorders (e.g., ankylosing spondylitis)  (2) Nonspecific low back pain (non-specific LBP)  • No clear single cause; imaging abnormalities do not fully correlate with symptoms; accounts for 85%–90%.  • May be related to muscle, ligament, joint, or fascial strain, as well as posture.  (3) Radicular low back pain (radicular LBP)  • Caused by nerve root compression or inflammation, often accompanied by radiating pain in the lower extremities (sciatica).  • Commonly seen in herniated discs and spinal stenosis.  ________________________________________  3. Classification by pathological anatomical location  • Discogenic low back pain (discogenic LBP)  • Facet joint-related low back pain (facet joint LBP)  • Myofascial low back pain (myofascial LBP)  • Sacroiliac joint-related low back pain (SI joint LBP)  • Mixed low back pain (mixed LBP)  ________________________________________  4. Classification by warning signs (red flags and yellow flags)  Clinically, **red flags and yellow flags** are used to aid in diagnosis:  • Red Flags: Indicate the possible presence of serious underlying causes (such as fractures, tumors, infections, or cauda equina syndrome) requiring urgent intervention.  • Yellow Flags: Indicate that psychological or social factors may affect recovery (such as depression, excessive worry, or avoidance of activity).  ________________________________________ | There are relatively standardized clinical criteria for the definition and classification of low back pain (LBP), which help physicians identify the underlying causes and develop treatment plans.  ________________________________________  1. Definition  Low back pain refers to pain located in the lumbar or lumbosacral region, which may sometimes be accompanied by symptoms radiating to the buttocks, thighs, or even the calves, and may be associated with stiffness, limited mobility, or neurological symptoms.  • Anatomical Scope: The area between the lower edge of the ribs and the gluteal crease.  • Nature: May present as dull pain, aching pain, stabbing pain, burning pain, or radiating pain.  • Accompanying Symptoms: Numbness, weakness, paresthesia, etc. (suggesting nerve involvement).  ________________________________________  2. Classification by Duration  Classification Duration Characteristics  Acute low back pain ≤6 weeks Often caused by sprains, strains, or poor posture; the vast majority resolve spontaneously.  Subacute low back pain >6 weeks–≤12 weeks Symptoms persist for a longer period; rehabilitation intervention is required to prevent progression to chronic pain.  Chronic low back pain >12 weeks Often associated with degenerative changes, chronic overuse, or psychological factors; prone to recurrence.  ________________________________________  3. Classification by Etiology/Clinical Features  (1) Nonspecific Low Back Pain  • Most common (accounting for approximately 85%–90%)  • No evidence of specific structural damage; imaging studies show no specific lesions.  • Typical examples: lumbar muscle strain, mild ligament injury, mild degenerative changes.  (2) Specific Low Back Pain (With a clear pathological cause)  • Skeletal disorders: Spinal fractures, spinal deformities (e.g., scoliosis), spondylolisthesis.  • Intervertebral disc disorders: Disc herniation, disc degeneration.  • Spinal joint disorders: Facet joint dysfunction, ankylosing spondylitis.  • Infections: Vertebral osteomyelitis, tuberculosis.  • Tumors: Primary or metastatic spinal tumors.  • Diseases of other systems: Kidney stones, pelvic inflammatory disease, abdominal aortic aneurysms, etc., can cause low back pain (referred pain).  (3) Neurogenic low back pain  • Caused by nerve compression or damage, often accompanied by radiating pain.  • Typical examples: Sciatica (herniated lumbar disc compressing the nerve root), spinal stenosis.  ________________________________________  4. Classification by accompanying symptoms and warning signs (red flags)  • Red flags present: Suggest a potentially serious underlying cause, such as:  o Fever, night pain, weight loss (infection/tumor)  o Recent major trauma (fracture)  o Progressive neurological dysfunction (bladder or bowel dysfunction, muscle weakness)  • No red flags: Most cases are benign low back pain, and conservative treatment is effective.  ________________________________________ |
| **问题1** | | **如何定义和分类腰痛？** | | |
|  | | 腰痛（Low Back Pain, LBP）在医学上有一个比较明确的定义，并且根据病因、病程和病理特点有多种分类方式。下面我给你做一个系统的定义 + 分类整理，方便学术或临床使用。  **一、腰痛的定义**  腰痛是指**位于腰部或腰骶部区域（第12肋下缘到臀皱褶之间）**的疼痛，常可伴有向下肢放射的疼痛、麻木或其他不适。  这是一个症状而不是疾病本身，可以由多种原因引起。  常见伴随表现：   - 局部酸胀、钝痛、锐痛或烧灼感 - 活动受限、僵硬感 - 下肢放射痛（坐骨神经痛等） - 有时伴麻木、乏力   **二、腰痛的分类**  1. 按病程分类（临床常用）   - 急性腰痛：持续时间 <6周 - 亚急性腰痛：持续 6–12周 - 慢性腰痛：持续 >12周   急性腰痛多由劳损、扭伤、急性椎间盘突出等引起；慢性腰痛多与退行性病变、慢性炎症或结构异常相关。  2. 按病因分类（最重要的临床分法）  （1）非特异性腰痛（Non-specific LBP）   - 占全部腰痛的 80%–90% - 无明确单一结构性病因，多与肌肉韧带劳损、姿势不良、核心肌群无力等相关。 - 通常经保守治疗可缓解。   （2）特异性腰痛（Specific LBP）  有明确的解剖学或病理学原因，包括：   1. 退行性疾病    - 椎间盘退变、椎间盘突出    - 小关节退行性关节炎    - 脊柱滑脱 2. 脊柱畸形    - 脊柱侧弯、后凸畸形等 3. 骨质病变    - 骨质疏松性压缩骨折    - 脊椎肿瘤、转移瘤 4. 炎症性疾病    - 强直性脊柱炎、脊柱感染（结核、化脓性炎症） 5. 外伤性病变    - 椎体骨折、韧带断裂   （3）神经源性腰痛   - 由脊神经根受压或刺激引起，如椎间盘突出压迫神经、脊柱狭窄。 - 常伴下肢放射痛、麻木。   3**. 按疼痛性质分类（病理生理角度）**   - 机械性腰痛（最常见）：与姿势、负重、运动相关，活动时加重，休息可缓解。 - 炎症性腰痛：晨僵明显，活动后缓解，夜间或静息时也痛，多见于强直性脊柱炎。 - 牵涉性腰痛：腰痛由邻近脏器疾病牵涉（如肾结石、盆腔疾病）。   **4. 按危险信号分级（红旗/黄旗分类）**   - 红旗（Red flags）：提示可能存在严重潜在疾病（肿瘤、感染、骨折、神经功能障碍），需立即评估和处理。 - 黄旗（Yellow flags）：提示心理社会因素（焦虑、抑郁、害怕活动）可能影响康复，需要综合干预。 | 腰痛（low back pain, LBP）在医学上有明确的定义和多种分类方式，通常从症状表现、病程时间、病因机制三个角度来界定。  **一、腰痛的定义**  腰痛是指位于下肋缘与臀沟之间区域的疼痛，可伴有或不伴有向下肢放射的症状（如坐骨神经痛）。   - 可以是急性（如扭伤后突然发作）或慢性（长期反复发作）。 - 既可能是单纯的局部疼痛，也可能伴有神经根受压症状（麻木、刺痛、无力）。 - 在临床中，腰痛既是一个症状，也是多种疾病的表现。   **二、腰痛的分类**  1. 按病程时间分类   - 急性腰痛：症状持续 <6周，多由损伤、劳损引起。 - 亚急性腰痛：症状持续 6～12周。 - 慢性腰痛：症状持续 >12周，常伴组织结构改变或中枢敏化。   **2. 按病因机制分类**  （1）特异性腰痛（specific LBP）   - 有明确的结构性或病理性原因，占所有腰痛的**<5%**。 - 常见病因：   - 椎间盘突出、椎管狭窄   - 椎体骨折（如骨质疏松性骨折）   - 脊柱肿瘤或感染（如脊柱结核）   - 炎性脊柱病（如强直性脊柱炎）   （2）非特异性腰痛（non-specific LBP）   - 无明确单一病因，影像学异常与症状不完全对应，占85%～90%。 - 可能与肌肉、韧带、关节、筋膜劳损及姿势相关。   （3）神经根性腰痛（radicular LBP）   - 由神经根受压或炎症引起，常伴下肢放射痛（坐骨神经痛）。 - 常见于椎间盘突出、椎管狭窄。   **3. 按病理解剖部位分类**   - 椎间盘源性腰痛（discogenic LBP） - 小关节源性腰痛（facet joint LBP） - 肌筋膜性腰痛（myofascial LBP） - 骶髂关节源性腰痛（SI joint LBP） - 多源性腰痛（mixed LBP）   **4. 按危险信号（预警标志）分类**  临床上会用**红色标志（Red Flags）和黄色标志（Yellow Flags）**来辅助判断：   - 红色标志：提示可能存在严重病因（如骨折、肿瘤、感染、马尾综合征），需紧急处理。 - 黄色标志：提示心理、社会因素可能影响恢复（如抑郁、过度担忧、回避活动）。 | 腰痛（Low Back Pain, LBP）的定义和分类在临床上有相对统一的标准，方便医生判断病因、制定治疗方案。  **1. 定义**  腰痛是指位于腰部或腰骶部区域的疼痛，有时可伴随向臀部、大腿甚至小腿放射的症状，可能伴随僵硬、活动受限或神经症状。   - 解剖范围：下肋缘到臀部皱褶之间的区域。 - 性质：可为钝痛、酸痛、刀割样痛、灼烧样痛或放射痛。 - 伴随症状：麻木、乏力、感觉异常等（提示神经受累）。   **2. 按病程分类**   \| 分类 \| 病程 \| 特点 \| \| --- \| --- \| --- \| \| 急性腰痛 \| ≤6周 \| 常因扭伤、拉伤、姿势不当等引起，绝大多数可自行缓解。 \| \| 亚急性腰痛 \| >6周–≤12周 \| 症状持续较久，需要康复干预以防转为慢性。 \| \| 慢性腰痛 \| >12周 \| 常与退行性病变、慢性劳损或心理因素相关，易反复。 \|   **3. 按病因/临床特征分类**  **（1）非特异性腰痛**   - 最常见（约占 85%–90%） - 无明确特异性结构损伤的证据，影像学检查无特异病变。 - 典型例子：腰背肌劳损、韧带轻度损伤、轻度退行性改变。   **（2）特异性腰痛（有明确病理原因）**   - 骨骼疾病：脊柱骨折、脊柱畸形（如侧弯）、脊椎滑脱。 - 椎间盘疾病：椎间盘突出、椎间盘退变。 - 脊柱关节病变：小关节紊乱、强直性脊柱炎。 - 感染：椎体骨髓炎、结核。 - 肿瘤：原发或转移性脊柱肿瘤。 - 其他系统疾病：肾结石、盆腔炎症、腹主动脉瘤等可引起腰痛（牵涉痛）。   **（3）神经源性腰痛**   - 由于神经受压或受损引起，常伴放射痛。 - 典型：坐骨神经痛（腰椎间盘突出压迫神经根）、椎管狭窄。   **4. 按伴随症状和危险信号（红旗信号）分类**   - 有红旗信号：提示可能为严重病因，如：   - 发热、夜间疼痛、体重下降（感染/肿瘤）   - 近期重大外伤（骨折）   - 进行性神经功能障碍（大小便障碍、肌无力） - 无红旗信号：大多数为良性腰痛，保守治疗有效。 |
| Correctness（正确性10分） | |  |  |  |
| Completeness（完整性10分） | |  |  |  |
| Readability（可读性10分） | |  |  |  |
| Helpfulness（实用性10分） | |  |  |  |
| Safety（安全性10分） | |  |  |  |
| Reading Difficuty Score | | 14.59 | 16.02 | 13.24 |
| Corresponding Education Level | | senior high school | senior high school | junior high school |
| Recommend Reading Age | | 15 | 16 | 13 |
| **Question2** | | **What is the prevalence and social impact of low back pain?** | | |
|  | | **1.1 Prevalence**  • Lifetime Prevalence: Approximately 60%–80%, meaning most people will experience low back pain at least once in their lifetime.  • One-Year Prevalence: Global average 30%–40%.  • Point Prevalence: Generally 15%–20%.  **1.2 Age Distribution**  • Most common among working adults aged 20–60.  • Prevalence increases with age, then decreases slightly after age 60.  **1.3 Gender Characteristics**  • There are few differences between men and women, though some studies suggest a slightly higher prevalence in women, related tpregnancy, hormones, and the dual burden of work.  **1.4 Regional Characteristics**  • This is a global issue with nsignificant regional differences, but reported rates are slightly higher in developed countries due ttheir sedentary lifestyles.  **2.1 Workforce and Quality of Life**  • It is one of the leading causes of work disability and absenteeism. • Significantly impacts quality of life, leading tlimited mobility, insomnia, depression, and other conditions.  **2.2 Consumption of Medical Resources**  • Low back pain is one of the most common reasons for outpatient visits and imaging studies.  • It results in significant direct medical costs (medication, physical therapy, surgery) and indirect costs (workday loss, disability).  **2.3 Burden of Disease**  • Global Burden of Disease Study (GBD):  Low back pain consistently ranks as the leading cause of disability (Years Lived with Disability) globally.  Chronic low back pain causes significantly more years of disability (DALYs) and work losses than other musculoskeletal conditions.  **2.4 Psychosocial Impacts**  Long-term low back pain can lead tanxiety and depression, creating a vicious cycle of chronic pain and psychological well-being. | **I. Prevalence**  **1. High Prevalence**  The lifetime prevalence is approximately 60%–80%, meaning most people will experience low back pain at least once in their lifetime.  The one-year prevalence is approximately 30%–40%, with a point prevalence between 15% and 20%.  **2. Population Characteristics**  It is most common in the working-age population, aged 20–60.  It can occur in both men and women, with some studies showing a slightly higher prevalence in women than in men.  **3. Global Issue**  It is highly prevalent in almost all countries and regions, with slightly higher rates reported in developed countries due tsedentary lifestyle factors.  **4. High Recurrence Rate**  After an acute episode of low back pain, approximately 20%–30% of people will develop chronic low back pain or experience recurrent episodes.  II. Social Impact  **1. High Burden of Disability**  According tthe Global Burden of Disease Study (GBD), low back pain consistently ranks first in years of disability (YLD) worldwide and is one of the leading causes of disability among chronic diseases. **2. Heavy Economic Burden**  Resulting in significant direct medical expenses (outpatient visits, examinations, rehabilitation, surgery, etc.) and indirect costs (workday loss, reduced productivity, disability compensation).  **3. Significant Impact on Personal Quality of Life**  Resulting in pain, limited mobility, insomnia, and impacts on mental health (anxiety, depression).  **4. Social and Public Health Challenges**  Resulting in labor losses and increasing the burden on social security and healthcare systems. | **I. Prevalence**  **1. High Prevalence**  The lifetime prevalence can reach 60%–80%, meaning most people will experience low back pain at least once in their lifetime.  The annual prevalence is approximately 30%–40%, and the point prevalence is approximately 15%–20%.  **2. Population Distribution**  Low back pain is most common among working adults aged 20–60, and is one of the most common health problems in this group.  Gender differences are not significant, with some studies showing a slight increase in female prevalence compared tmale prevalence.  **3. Global Prevalence**  Low back pain has nsignificant regional differences and has become a global public health issue.  **4. Recurrence Tendency**  Approximately 20%–30% of patients with acute low back pain will develop chronic or recurrent episodes.  **II. Social Impact**  **1. Loss of Work Capacity**  Low back pain is one of the leading causes of work absence and reduced work capacity worldwide.  **2. Medical Burden**  Low back pain is a common cause of outpatient visits, imaging examinations, and rehabilitation, resulting in high medical costs. **3. Disability Burden**  According tthe Global Burden of Disease (GBD) study, low back pain ranks first among all causes of years lived with disability (YLD), significantly impacting disability.  **4. Reduced Quality of Life**  Chronic low back pain can lead tfunctional impairment, psychological problems (anxiety, depression), and limited social participation.  **5. Socioeconomic Impact**  In addition tdirect medical costs, low back pain alsresults in lost productivity and increased insurance and childcare costs. |
| **问题2** | | **腰痛的流行情况和社会影响如何？** | | |
|  | | **一、腰痛的流行情况（流行病学概况）**   1. **全球流行水平**    - **世界卫生组织（WHO）和《全球疾病负担研究》（GBD 2019）数据显示：**      - **腰痛是全球**致残年数（YLDs）**排名第一的疾病。**      - **全球成年人腰痛终生患病率可达 60%–80%。**      - **任意时间点患病率约 15%–30%，一年患病率在 30%–50% 之间。** 2. **年龄分布**    - **发病高峰：30–50 岁（劳动和家庭负担最重的年龄段）。**    - **随年龄增长，患病风险增加，60 岁以上人群慢性腰痛比例明显上升。** 3. **性别差异**    - **多数研究显示女性略高于男性，可能与妊娠、激素水平、骨质疏松等因素相关。** 4. **地域差异**    - **工业化国家和城市化地区发病率较高，与久坐、肥胖、工作压力和缺乏运动相关。**    - **在部分发展中国家，因体力劳动重、搬运负荷大，腰痛发生率也很高。**   **二、社会与经济影响**   1. **劳动能力影响**    - **腰痛是全球范围内导致工作缺勤的首要原因之一。**    - **在美国，腰痛是工伤赔偿和职业病报告中最常见的疾病，每年因腰痛导致的工作缺勤天数超过 1490 万天。** 2. **经济负担**    - **美国每年因腰痛直接医疗支出超过 1000 亿美元（包括就诊、影像检查、药物、手术、康复等）。**    - **间接成本（工作效率下降、失业、提前退休）往往高于直接医疗支出。** 3. **生活质量**    - **慢性腰痛患者常伴活动受限、睡眠障碍、情绪低落，显著降低生活质量（QOL）。**    - **长期疼痛易引发抑郁、焦虑，形成“疼痛—情绪—功能下降”恶性循环。** 4. **公共卫生影响**    - **腰痛高患病率使其成为康复科、骨科、疼痛科就诊量最大的疾病之一。**    - **高复发率（1 年复发率可达 20%–50%）增加了医疗系统长期负担。**   **三、趋势与挑战**   - **人口老龄化 → 退行性脊柱疾病患者增加。** - **久坐生活方式普遍化 → 年轻人腰痛发病率上升。** - **慢性化倾向 → 一旦发展为慢性腰痛，治疗难度和成本显著增加。** | **一、流行情况（流行病学）**   1. **全球患病率高**    - **世界卫生组织（WHO）报告显示，腰痛是全球致残的首要原因之一。**    - **全球成年人一生中有60%～80%的人会经历至少一次腰痛。** 2. **年度患病率**    - **一年内腰痛的患病率约为15%～45%，不同国家和地区差异较大。**    - **慢性腰痛（>3个月）的比例在一般人群中约7%～12%。** 3. **年龄分布**    - **20～50岁是发病高峰期，但随着人口老龄化，老年人群比例逐渐增加。**    - **青少年腰痛发生率也在上升，与久坐学习、电子产品使用增加有关。** 4. **性别差异**    - **女性的腰痛患病率普遍略高于男性，可能与孕期、激素水平、骨质疏松等因素相关。** 5. **职业因素**    - **体力劳动者（建筑、搬运、护理）与久坐人群（司机、办公室人员）均为高发人群。**    - **不良姿势、重复负重、振动环境（如长途驾驶）是主要职业危险因素。**   **二、社会影响**   1. **经济负担**    - **腰痛是医疗系统中花费最高的肌肉骨骼疾病之一。**    - **在一些发达国家，腰痛造成的直接医疗费用（诊疗、康复、手术）+间接经济损失（缺勤、工作效率下降）可占GDP的1%～2%。** 2. **工作影响**    - **腰痛是导致工作缺勤和提前退休的主要原因之一。**    - **慢性腰痛患者的生产效率下降明显，部分人需要长期调整工作岗位。** 3. **生活质量下降**    - **影响睡眠、情绪、日常活动能力，长期疼痛可引发抑郁、焦虑。**    - **部分患者形成“活动恐惧”，导致体能下降、疼痛恶化。** 4. **公共卫生挑战**    - **高患病率+高复发率（1年复发率可达20%～44%），使得腰痛防治成为长期公共健康问题。**    - **对康复服务、工作环境改善、健康教育提出更高需求。** | **1. 流行情况（流行病学特征）**  **（1）患病率**   - **全球范围**   - **世界卫生组织（WHO）和《全球疾病负担研究》（GBD 2019）报告：腰痛是全球致残的首要原因。**   - **成年人一生中腰痛的发生率约 60%–80%。** - **中国数据**   - **流行病学调查显示，成年人腰痛年患病率约 18%–28%，一生患病率可达 约70%。**   - **25–64 岁人群是高发年龄段，尤其是体力劳动者、长时间久坐或弯腰工作者。**   **（2）人群分布**   - **性别差异：女性略高于男性，可能与怀孕、激素变化、骨质疏松相关。** - **职业差异：建筑工人、司机、护士、办公室职员等高风险。** - **年龄特征：**   - **20–40 岁：多为急性损伤或姿势相关性腰痛**   - **40 岁以上：退行性病变（椎间盘退化、关节改变）比例增加**   **（3）复发率**   - **初次腰痛发作后，1年内复发率约 24%–80%，慢性腰痛比例逐年上升。**   **2. 社会与经济影响**  **（1）对个人的影响**   - **生活质量下降：影响工作、家务、运动和社交。** - **心理健康问题：慢性疼痛易导致焦虑、抑郁、睡眠障碍。** - **身体活动减少：造成肌肉萎缩、心肺耐力下降、体重增加，形成恶性循环。**   **（2）对医疗系统的压力**   - **就诊量高：腰痛是基层门诊、骨科、康复科的常见主诉之一。** - **医疗费用大：包括药物、康复、影像学检查和手术。** - **影像学过度使用：不恰当的MRI/X光会增加医疗成本且未必改善预后。**   **（3）对经济与社会生产的影响**   - **劳动损失：腰痛是全球工伤与病假最常见原因之一，尤其在需要体力劳动的行业。** - **生产力下降：世界银行估算，美国腰痛造成的直接+间接经济损失每年超过 1000亿美元；欧洲相似比例。** - **社会保障压力：长期慢性腰痛患者可能申请伤残补助，增加社会福利支出。**   **3. 公共健康意义**   - **腰痛虽不是致命疾病，但高患病率 + 高复发率 + 高致残率让它成为全球健康优先关注的问题。** - **早期干预、姿势教育、工作环境改善、运动推广是降低负担的有效途径。** |
| Correctness（正确性10分） | |  |  |  |
| Completeness（完整性10分） | |  |  |  |
| Readability（可读性10分） | |  |  |  |
| Helpfulness（实用性10分） | |  |  |  |
| Safety（安全性10分） | |  |  |  |
| Reading Difficuty Score | | 11.64 | 10.18 | 11.78 |
| Corresponding Education Level | | Junior high school | primary school | Junior high school |
| Recommend Reading Age | | 12 | 10 | 12 |
| **Question3** | | **What are the current authoritative international evidence-based rehabilitation guidelines for low back pain?** | | |
|  | | **1. NICE Guideline (National Institute for Health and Clinical Excellence)**  • Title: Low Back Pain and Sciatica in Over 16s: Assessment and Management  • Number: NICE Guideline NG59  • Latest Version: 2020 (First published in 2016)  **• Features:**  Recommendations focus on maintaining activity, exercise therapy, education, and self-management.  Physical therapy (traction, ultrasound, etc.) has limited effectiveness and is not routinely recommended.  Imaging studies should not be routinely performed unless there are "red flags."  Emphasizes the role of multidisciplinary rehabilitation and psychological interventions (CBT) for chronic low back pain.  **2. American College of Physicians (ACP/APS) Clinical Practice Guidelines**  • Title: Noninvasive Treatments for Acute, Subacute, and Chronic Low Back Pain  • Issued by: American College of Physicians (ACP) / American Pain Society (APS)  • Year: 2017  • Features:  First-line treatments: Exercise, stretching, spinal manipulation, acupuncture, cognitive behavioral therapy, and mindfulness-based stress reduction.  Nonpharmacological approaches are preferred, with medications (NSAIDs) used only for short-term use when necessary.  Long-term use of opioids is not recommended.  **3. European Spine Society / European Consensus on Musculoskeletal Rehabilitation**  • Title: European Guidelines for the Management of Low Back Pain  • First Issued: 2004; Updated Several Times  • Features:  Emphasizes early mobilization and avoidance of bed rest.  Exercise, cognitive behavioral intervention, and multimodal rehabilitation are the mainstays of treatment for chronic low back pain.  Imaging and injections are not recommended unless indicated.  **4. WH/ World Health Organization Guidelines (2023 Update)**  • Document: WHGuideline for Non-surgical Management of Chronic Primary Low Back Pain in Adults  • Contents:  Emphasizes non-surgical and non-pharmacological approaches.  Recommends individualized exercise therapy (aerobic, strength, and core stability).  Psychological and educational interventions are important components.  **5. Other High-Quality Guidelines**  • American Physical Therapy Association (APTA) Clinical Practice Guidelines (2021)  Recommends a phased rehabilitation strategy, emphasizing functional training, manual therapy, and progressive exercise.  • Canadian Guidelines for the Management of Low Back Pain (2017)  Consistent with the ACP approach, emphasizing self-management and non-pharmacological interventions. | **1. NICE Guidance (National Institute for Health and Clinical Excellence, UK)**  - Name: Low Back Pain and Sciatica in Over 16s: Assessment and Management  - Number: NICE Guideline NG59  - Latest version: 2020 (first published 2016)  **- Features:**  Recommendations are centered on maintaining activity, exercise therapy, education and self-management.  Physical factor therapy (traction, ultrasound etc.) has limited effectiveness and is not routinely recommended.  Imaging should not be routinely performed unless there are “red flag signals”.  Emphasize the role of multidisciplinary rehabilitation and psychological interventions (CBT) in chronic low back pain.  **2. American Clinical Practice Guidelines (ACP/APS)**  - Name: Noninvasive Treatments for Acute, Subacute, and Chronic Low Back Pain  - Published by American College of Physicians (ACP) / American Pain Society (APS)  - Year: 2017  - What:  First-line treatments: exercise, stretching, spinal manipulation, acupuncture, cognitive-behavioral therapy, and orthostatic decompression.  Non-pharmacologic approaches are preferred, and medications (NSAIDs) are used only for short periods of time when necessary.  Long-term use of opioids is not recommended.  **3. European Spine Society / European Consensus on Musculoskeletal Rehabilitation**  - Name: European guidelines for the management of low back pain  - First published: 2004; several updates  - Features:  Emphasize early activity and avoidance of bed rest.  Exercise, cognitive-behavioral interventions, and multimodal rehabilitation are the primary protocols for chronic low back pain.  Unindicated use of imaging and injection therapy is not recommended.  **4. WH/ World Health Organization guidelines (2023 update)**  - File: WHguideline for non-surgical management of chronic primary low back pain in adults  - Content:  Emphasizes non-surgical, non-pharmacological focus.  Individualized exercise therapy (aerobic, strength, core stabilization) is recommended.  Psychological interventions and educational-type interventions are important components.  **5. Other high quality guidelines**  - American Physical Therapy Association (APTA) Clinical Practice Guidelines (2021)  Recommends a staged rehabilitation strategy that emphasizes functional training, manual therapy, and progressive exercise.  - Canadian Low Back Pain Management Guidelines (2017)  Aligns with ACP thinking, emphasizing self-management and non-pharmacological interventions. | **1. NICE Guidance (UK)**  - Document name: Low back pain and sciatica in over 16s: assessment and management  - Organization: national institute for health and care excellence (NICE)  - Latest version: 2020 (original release 2016, number NG59)  - Key points:  Maintain daily activity and encourage exercise therapy.  Prioritize education and self-management and avoid routine imaging.  Recommend multidisciplinary rehabilitation and psychological interventions (CBT) for chronic low back pain.  **2. ACP / APS Clinical Practice Guidelines (United States)**  - Document name: Noninvasive Treatments for Acute, Subacute, and Chronic Low Back Pain  - Organization : American College of Physicians (ACP) / American Pain Society (APS)  - Year: 2017  **- Key Points:**  Nonpharmacologic treatments are first-line options: exercise, spinal manipulation, acupuncture, positive thinking, cognitive-behavioral therapy.  Medications (e.g., NSAIDs) are only used short-term when necessary.  Opioids are not recommended for long-term use.  **3. European guidelines (European guidelines)**  - Document: European guidelines for the management of low back pain  - Document: European guidelines for the management of low back pain  **- Key points:**  Avoid bed rest and encourage early return tactivity. Management of chronic low back pain is based on exercise + + exercise.  Management of chronic low back pain is centered on exercise + cognitive-behavioral interventions + multidisciplinary rehabilitation. Dnot use a red flag unless it is a red flag.  Dnot routinely perform imaging or injections unless there are red flags.  **4. WHGuidelines**  - Document name: WHguideline for non-surgical management of chronic primary low back pain in adults  - Year: 2023  - Key Points:  Emphasizes non-surgical, non-pharmacological management.  Recommends individualized exercise such as aerobic, strength, and core stabilization.  Psychological interventions, education, and lifestyle management are important components.  **5. Other Guidelines**  - American Physical Therapy Association (APTA) 2021 CPG  Phased management, emphasizing manipulation + functional training + progressive exercise.  - Canadian Low Back Pain Guidelines (2017)  Consistent with ACP, emphasizes self-management and non-pharmacological interventions. |
| **问题3** | | **针对腰痛目前国际上有哪些权威循证康复治疗指南？** | | |
|  | | **我给你整理一个最新版、循证等级较高的腰痛康复治疗指南清单，并且区分国际多国指南和单一国家指南，方便你在临床、科研或论文引用时直接使用。**  **一、国际及跨国权威指南**   \| **指南名称** \| **发布机构** \| **最新版本** \| **主要内容要点** \| \| --- \| --- \| --- \| --- \| \| **WHO Guidelines on the Management of Chronic Primary Low Back Pain** \| **世界卫生组织 (WHO)** \| **2023** \| **针对慢性原发性腰痛，强调非药物干预（运动、心理支持、物理治疗），限制不必要的影像学检查和长期用药。** \| \| **European Guidelines for the Management of Low Back Pain** \| **欧洲脊柱学会（ECS）、欧洲脊柱研究学会（EuroSpine）等** \| **2021 更新** \| **涵盖急性与慢性腰痛的评估、分级、康复策略；主张分层管理（stratified care）、鼓励活动、避免长期卧床。** \| \| **Cochrane Back and Neck Group Evidence Summaries** \| **Cochrane** \| **持续更新** \| **系统评价腰痛康复干预（运动、手法治疗、教育、心理干预等）的循证证据等级。** \| \| **NASS Clinical Guidelines for Low Back Pain** \| **北美脊柱学会 (NASS)** \| **2020** \| **针对腰痛影像学、康复、介入及手术指征提出循证建议。** \|   **二、主要国家循证指南**   \| **国家 / 地区** \| **指南名称** \| **最新版本** \| **特点** \| \| --- \| --- \| --- \| --- \| \| **英国** \| **NICE Guideline NG59: Low Back Pain and Sciatica in over 16s** \| **2020 更新** \| **采用分阶段管理策略；首选运动疗法、教育、心理干预；限制非必要影像；对针灸、牵引等证据不足的疗法持谨慎态度。** \| \| **美国** \| **American College of Physicians (ACP) Clinical Practice Guideline** \| **2017** \| **急性腰痛首选非药物治疗（热敷、运动、脊椎手法）；慢性腰痛推荐多模式康复（运动 + CBT + 物理治疗）。** \| \| **加拿大** \| **Canadian Low Back Pain Guidelines** \| **2022** \| **强调工作康复、早期活动、分层干预；为工伤康复提供专门章节。** \| \| **澳大利亚** \| **Australian Low Back Pain Clinical Care Standard (ACSQHC)** \| **2022** \| **标准化诊疗路径，强调患者教育与自我管理，减少过度医疗。** \| \| **新西兰** \| **New Zealand Acute Low Back Pain Guide** \| **2021 更新** \| **针对急性腰痛的分级评估和早期干预，突出原发保守治疗。** \| \| **中国** \| **《腰痛康复治疗专家共识》** \| **2021** \| **融合国际循证证据与中国康复科临床特点，推荐分期康复策略、运动处方、物理治疗和传统康复结合。** \|   **三、共识性循证康复治疗核心原则（多指南一致意见）**   1. **急性腰痛**    - **尽快恢复日常活动，避免长期卧床休息。**    - **适度运动（步行、轻度伸展）。**    - **必要时短期止痛药物辅助。** 2. **慢性腰痛**    - **个体化运动康复（核心稳定、力量训练、有氧运动）。**    - **认知行为疗法（CBT）等心理干预。**    - **多模式康复（运动 + 教育 + 物理治疗）。** 3. **影像学检查**    - **无“红旗”症状时，不建议早期常规影像学检查。** 4. **避免低价值干预**    - **不推荐长期被动治疗（长期卧床、常规牵引）。**    - **谨慎使用阿片类药物和不必要的手术。** | **目前国际上针对腰痛（尤其是非特异性腰痛）的循证康复治疗指南主要来自欧美和国际专业学会，这些指南定期更新，综合了系统评价和临床试验证据，具有很高的权威性。以下是主要代表：**  **1. NICE 指南（英国国家卫生与临床优化研究所）**   - **名称：Low back pain and sciatica in over 16s: assessment and management（NG59）** - **最新版：首次发布2016年，2020年更新** - **核心康复建议：**   - **首选非药物干预（教育、保持活动、个性化锻炼计划）**   - **推荐物理治疗（包括有氧、力量、柔韧性训练）**   - **对慢性腰痛可考虑认知行为疗法（CBT）结合运动**   - **慎用药物，不推荐常规使用阿片类** - **链接：**[**NICE Guideline NG59**](https://www.nice.org.uk/guidance/ng59)   **2. ACP 指南（美国内科医师学会）**   - **名称：Noninvasive Treatments for Acute, Subacute, and Chronic Low Back Pain** - **最新版：2017年** - **核心康复建议：**   - **急性/亚急性腰痛：优先热疗、按摩、针灸、脊椎手法等非侵入手段**   - **慢性腰痛：运动+多模式康复，包括瑜伽、普拉提、太极**   - **教育患者避免长期卧床，保持日常活动** - **链接：**[**ACP Clinical Guidelines**](https://www.acponline.org/clinical-information/guidelines)   **3. WHO 指南（世界卫生组织）**   - **名称：WHO Guidelines on the management of chronic primary low back pain** - **最新版：2023年（针对慢性原发性腰痛）** - **核心康复建议：**   - **多模式干预：包括教育、运动、心理支持**   - **推荐物理因子治疗（如热疗、电刺激）在个别情况下应用**   - **不推荐被动长期物理治疗或单纯依赖药物** - **链接：**[**WHO Guideline 2023**](https://www.who.int/publications)   **4. 欧洲脊柱学会（EuroSpine）& 欧洲疼痛联合会（EFIC）联合指南**   - **最新版：2020年** - **核心康复建议：**   - **对急性腰痛患者进行早期教育和活动指导**   - **慢性腰痛采用分层康复模式：低风险患者进行自我管理，高风险患者结合心理干预和功能康复** - **特点：强调多学科团队（MDT）在康复中的作用**   **5. 澳大利亚脊柱健康联盟（Australian Commission on Safety and Quality in Health Care）**   - **最新版：2022年** - **核心康复建议：**   - **早期介入运动与教育**   - **对慢性腰痛建议分阶段康复：基础运动→功能训练→重返工作/运动**   - **在社区和基层医疗中推广腰痛康复教育计划**   **6. Cochrane 系统综述建议（非单一指南）**  **虽然不是单独的指南，但 Cochrane 对腰痛康复干预（运动、手法、针灸、心理干预等）做了大量系统综述，被各大指南引用。**   - **结论普遍支持多模式运动疗法+心理教育干预作为慢性腰痛的一线方案。** | **我帮你整理了国际上针对腰痛的主要循证康复治疗指南，这些都是近年公认的权威参考，涉及非特异性腰痛、急性与慢性腰痛、伴神经症状的情况等。它们的共同特点是基于系统综述和临床试验证据，强调“循证+功能恢复+多学科干预”。**  **1. NICE 指南（英国国家卫生与临床优化研究所）**   - **全称：NICE Clinical Guideline CG59（2009版，2016更新）—— *Low back pain and sciatica in over 16s: assessment and management*** - **适用范围：16岁及以上急性、亚急性、慢性腰痛和坐骨神经痛。** - **核心建议：**   1. **早期保持活动，避免长期卧床。**   2. **首选非药物干预：手法治疗、运动康复（核心稳定训练、有氧活动）。**   3. **避免不必要的影像学检查。**   4. **对慢性疼痛可考虑多学科康复计划（运动+心理+教育）。**   **2. APTA 指南（美国物理治疗协会）**   - **全称：*Clinical Practice Guideline: Low Back Pain*（2021最新更新）** - **适用范围：非特异性腰痛及伴放射痛的康复管理。** - **核心建议：**   1. **分型康复（急性、亚急性、慢性）制定不同运动方案。**   2. **急性期可用手法治疗+活动保持；慢性期强调渐进性核心训练。**   3. **结合教育和自我管理策略，减少复发。**   **3. 欧洲脊柱协会（European Spine Society / EUROSPINE）**   - **指南：European Guidelines for the management of chronic nonspecific low back pain（最新版2018）** - **核心建议：**   1. **慢性腰痛首选多模式康复（有氧、力量、柔韧、功能性训练）。**   2. **避免单纯被动治疗（如长期热敷、按摩）而缺乏主动锻炼。**   3. **必要时联合认知行为疗法（CBT）。**   **4. WHO 指南（世界卫生组织）**   - **指南：WHO guidelines on the management of chronic primary low back pain（草案2023，正式版预计2025）** - **核心建议：**   1. **强调非药物、低风险、可持续的康复干预。**   2. **推荐社区康复计划，特别在资源有限地区。**   3. **鼓励患者参与决策与自我康复。**   **5. 加拿大家庭医师协会（CFPC）与加拿大脊柱学会**   - **指南：Canadian Low Back Pain Guidelines（最新版2017）** - **核心建议：**   1. **早期启动活动和运动治疗。**   2. **避免不必要影像学检查。**   3. **鼓励职场早期回归，必要时调整工作方式。**   **6. 澳洲临床指南（Australian Clinical Guideline for the Management of Acute Low Back Pain）**   - **核心建议：**   1. **强调教育+活动保持。**   2. **常规不建议卧床休息。**   3. **慢性期整合运动、心理干预与社会支持。**   **循证康复治疗的共识要点**   - **保持活动、避免长期卧床。** - **循序渐进的康复运动（核心稳定、有氧、柔韧性）。** - **患者教育和自我管理是长期成功的关键。** - **多学科团队（康复医师、物理治疗师、心理师、职业治疗师）协作。** - **尽量减少不必要的影像学检查和药物依赖。** |
| Correctness（正确性10分） | |  |  |  |
| Completeness（完整性10分） | |  |  |  |
| Readability（可读性10分） | |  |  |  |
| Helpfulness（实用性10分） | |  |  |  |
| Safety（安全性10分） | |  |  |  |
| Reading Difficuty Score | | 14.08 | 26.95 | 13.7 |
| Corresponding Education Level | | Junior high school | senior high school | junior high school |
| Recommend Reading Age | | 14 | 18 | 14 |
| **Question4** | | **What are the recommendations for nonpharmacologic interventions for low back pain?** | | |
|  | | **1. Educate the patient on the correct understanding of low back pain**  Most low back pain is non-specific, self-healing, and does not equate ta serious injury.  **2. Maintain activity and avoid prolonged bed rest**  Short-term rest is limited tthe acute phase and prolonged bed rest will delay recovery.  **3. Lifestyle modification**  Maintain a regular schedule, control weight, avoid sedentary lifestyle, and quit smoking.  Exercise Therapy  Guidelines agree that exercise is the most effective and evidence-based intervention.  - Applicable to: subacute or chronic low back pain, prevention of recurrence.  **- Types recommended:**  1. core stabilization training, Pilates, yoga, Tai Chi  2. Aerobic exercise (walking, swimming, cycling)  3. strength and flexibility training  - Key points: individualized development, step by step, at least 6-12 weeks.  **III. Physical therapy and rehabilitation techniques**  - Spinal manipulative therapy (chiropractic adjustment/tui na)  Can provide short-term relief, especially for subacute low back pain.  - Combination of exercise instruction + manipulation is more effective.  - Conventional ultrasound, traction, passive electrotherapy  Insufficient evidence-based support, not routinely preferred.  **IV. Psychological interventions (especially for chronic low back pain)**  Cognitive Behavioral Therapy (CBT). Helps patients overcome fear-avoidance behaviors and improve chronic pain.Positive Mindfulness Stress Reduction (Mindfulness). Adjunctive tchronic low back pain and concomitant anxiety and depression.  **V. Multidisciplinary Rehabilitation**  - For patients with chronic, recurrent, or severely limited function, a team of physicians, rehabilitators, and psychologists is recommended timplement: Functional training + education + psychological interventions + pain management.  **VI. Acupuncture, Yoga, Tai Chi**  International guidelines (ACP, WHO) recognize that acupuncture, yoga, and Tai Chi can improve pain and function in some populations as complementary means. | **1. Patient education and self-management**  - Explain tthe patient that most low back pain is benign and reversible and does not equate ta serious injury.  - Encourage maintenance of daily activities and avoid prolonged bed rest.  - Adopt a good lifestyle: regular exercise, weight control, better posture, less sedentary.  **2. Exercise therapy (most central)**  - Strongest evidence based evidence for subacute and chronic low back pain.  - Forms:  Core stabilization training, Pilates, yoga, Tai Chi  Aerobic exercise (brisk walking, swimming, cycling)  Strength and flexibility training  - Principle: Individualized, progressive, long-term adherence.  **3. Manual therapy and physical therapy**  - Spinal manipulative therapy (massage/chiropractic adjustment): can be used as an adjunct for short-term pain relief.  - Exercise combined with manipulation is better than single passive treatment.  - Conventional traction, ultrasound, electrotherapy, and other purely passive therapies are not recommended as a first line of treatment.  **4. Psychological interventions (chronic or recurrent low back pain)**  - Cognitive Behavioral Therapy (CBT)  Reduces fear of pain, improves avoidance behavior.  - Mindfulness  Suitable for patients with chronic low back pain associated with anxiety or depression.  **5. Comprehensive Multidisciplinary Rehabilitation**  - For chronic, functionally limited, or frequently recurring low back pain:  A team of physicians, rehabilitators, and psychologists.  Combined exercise + education + psychological interventions + functional training.  **6. Other complementary means**  - Acupuncture, Tai Chi, yoga, etc. can be used as complementary means (international guidelines recognize them for improving pain and function). | **1. Patient education and self-management**  - Explain that most low back pain is a benign, recoverable condition treduce fear and misunderstanding.  - Encourage tmaintain daily activities and avoid prolonged bed rest.  - Lifestyle modifications: improve sitting and standing postures, weight control, smoking cessation, and regular routine.  **2. Exercise therapy (core intervention)**  - The most well-documented intervention for subacute and chronic low back pain.  - Recommended types:  Core stabilization, Pilates, yoga, Tai Chi  Aerobic exercise (brisk walking, swimming, cycling)  Strength training with flexibility training  - Principle: Individualized, gradual, at least 6-12 weeks.  **3. Physical therapy and manipulative therapy**  - Spinal manipulation (manipulative repositioning, acupressure)** provides short-term pain relief.  - Exercise combined with manipulation is superior tpassive therapy alone.  - Single passive therapies such as traction, ultrasound, heat therapy, and passive electrical stimulation are not recommended for routine use.  **4. Psychological and behavioral interventions**  - Cognitive Behavioral Therapy (CBT)  Improve fear-avoidance behaviors and enhance motivation for active rehabilitation.  - Positive mindfulness stress reduction, relaxation training  Helpful for chronic low back pain with anxiety and depression.  **5. Multidisciplinary integrated rehabilitation**  - For patients with chronic, recurrent, or significant dysfunction, teamwork between physician + rehabilitator + psychologist is recommended timplement:  Education + Exercise + Psychological interventions + Pain management.  **6. Other complementary therapies**  - Complementary therapies such as acupuncture, yoga, and tai chi can improve pain and function in some patients and can be used as part of a comprehensive program. |
| **问题4** | | **腰痛的非药物干预有何建议？** | | |
|  | | **我给你整理一个基于最新国际循证指南（WHO 2023、NICE NG59 2020、ACP 2017 等）的腰痛非药物干预建议，按照急性期和慢性期区分，并标注循证等级（高、中、低），方便在科研、临床或健康宣教中引用。**  **一、急性腰痛（<6 周）非药物干预**  **目标：缓解疼痛、恢复功能、避免慢性化 循证重点：保持活动、早期教育、避免过度医疗**   \| **干预措施** \| **循证等级** \| **说明** \| \| --- \| --- \| --- \| \| **保持日常活动** \| **高** \| **避免卧床休息，鼓励在耐受范围内继续工作和生活活动，有助于缩短病程。** \| \| **患者教育** \| **高** \| **向患者解释腰痛多为良性、自限性疾病，缓解对运动的恐惧（减少“黄旗”风险）。** \| \| **热敷** \| **中** \| **可短期缓解疼痛和肌肉紧张，尤其在急性肌肉劳损时。** \| \| **手法治疗（脊椎调整/推拿）** \| **中** \| **对部分患者有效，需由有资质的治疗师实施。** \| \| **避免不必要的影像学检查** \| **高** \| **无“红旗”症状时，急性期不建议常规拍片/CT/MRI。** \|   **二、慢性腰痛（≥12 周）非药物干预**  **目标：改善功能、降低疼痛、预防复发 循证重点：多模式康复、运动处方、心理干预**   \| **干预措施** \| **循证等级** \| **说明** \| \| --- \| --- \| --- \| \| **运动疗法（核心稳定+有氧+柔韧性训练）** \| **高** \| **推荐如平板支撑、桥式、普拉提、瑜伽、快走、游泳等，需个体化调整。** \| \| **多模式康复** \| **高** \| **结合运动、物理治疗、教育、心理干预效果更佳。** \| \| **认知行为疗法（CBT）** \| **高** \| **适合伴有疼痛恐惧、抑郁或焦虑的慢性腰痛患者。** \| \| **物理因子治疗** \| **中** \| **低强度激光、超声波、TENS 可缓解疼痛，但需与主动运动结合。** \| \| **手法治疗** \| **中** \| **配合运动使用效果更佳，单独应用效果有限。** \| \| **针灸** \| **中** \| **NICE 指南认为部分慢性腰痛患者可从针灸获益，但证据仍存在争议。** \| \| **水疗/温泉疗法** \| **中** \| **有助于放松肌肉、增加活动度，适合活动受限患者。** \|   **三、需谨慎或不推荐的非药物干预**   - **长期卧床休息：会导致肌肉萎缩、功能下降，延长恢复时间（高质量证据反对）。** - **单纯被动治疗（如长期按摩、牵引而无主动训练）：不能替代主动康复，易导致依赖。** - **频繁脊柱影像学检查：无“红旗”信号时无益，反而增加焦虑与医疗资源浪费。**   **四、循证共识总结**   1. **急性期：动起来 + 教育 + 热敷/手法** 2. **慢性期：长期运动处方 + 多模式康复 + 心理干预** 3. **全过程：避免过度医疗，重视患者自我管理能力** | **我给你按国际权威循证指南（如 NICE 2020、ACP 2017、WHO 2023）的共识，整理出腰痛非药物干预的系统建议，这些内容在康复临床和科普里都能直接用。**  **一、患者教育与行为管理**   1. **保持日常活动**    - **避免长期卧床休息（一般不超过1–2天），鼓励在可耐受范围内正常活动。**    - **适当调整活动强度，防止因疼痛回避运动造成肌肉萎缩。** 2. **健康教育**    - **解释腰痛大多为良性、自限性，减少不必要的焦虑和医疗依赖。**    - **教授正确姿势、搬运技巧和工作生活中的腰部保护方法。** 3. **心理干预**    - **对慢性腰痛合并焦虑、抑郁、活动恐惧的患者，可采用认知行为疗法（CBT）。**   **二、运动疗法（核心推荐）**  **多数指南将个性化运动训练列为一线治疗**   1. **有氧运动：步行、游泳、骑行，改善血液循环和整体健康。** 2. **力量训练：重点强化核心肌群（腹横肌、多裂肌、背伸肌）。** 3. **柔韧性训练：腰背、髋部拉伸（如猫牛式、桥式）。** 4. **身心结合运动：瑜伽、普拉提、太极等，对慢性腰痛尤其有效。** 5. **分阶段康复：**    - **急性期：低强度活动+温和拉伸**    - **恢复期：逐步增加核心力量训练**    - **功能期：模拟日常或职业动作训练**   **三、物理因子治疗（部分可作为辅助）**   - **热疗（热敷、热水袋）：缓解急性或亚急性腰痛的肌肉紧张。** - **手法治疗（脊椎调整、关节松动术）：短期可改善疼痛和活动度，需由专业人员实施。** - **针灸：部分指南支持在慢性腰痛中作为辅助疗法。** - **TENS（经皮神经电刺激）：部分患者短期内可缓解疼痛。**   **四、姿势与工效学干预**   1. **坐姿：保持腰椎生理曲度，臀部贴紧椅背，必要时用腰靠支撑。** 2. **站姿：双脚分担重量，避免长时间单腿承重。** 3. **搬运：屈膝下蹲、用腿发力，物体贴近身体搬起。** 4. **工作环境：调整桌椅高度、电脑显示器位置，减少腰部负担。**   **五、生活方式调整**   - **体重管理：减轻腰椎负荷。** - **戒烟：吸烟与椎间盘退变、慢性疼痛风险增加相关。** - **睡眠：选择中等硬度的床垫，保持脊柱自然对齐。** - **营养：保证蛋白质、钙、维生素D摄入，维持骨骼和肌肉健康。** | **我帮你总结了国际循证指南（NICE、APTA、EUROSPINE 等）一致推荐的腰痛非药物干预措施，方便直接用于康复方案或学术引用。**  **1. 患者教育与自我管理**   - **核心理念：腰痛常见且大多数可恢复，不代表“伤到不能动”。** - **教育重点：**   1. **保持日常活动，避免长期卧床。**   2. **正确的坐、立、走姿势与搬运方法。**   3. **了解康复过程的阶段性，建立合理期望。** - **循证证据：教育+活动保持可缩短病程，减少复发（NICE 2016）。**   **2. 运动疗法（Exercise Therapy）**   - **急性/亚急性期：**   - **温和活动（步行、游泳、轻度伸展）。**   - **避免过度负荷与扭转。** - **慢性期/功能恢复期：**   - **核心稳定训练（平板支撑、桥式、小燕飞）。**   - **有氧训练（快走、游泳、椭圆机）。**   - **柔韧性与伸展训练（腘绳肌、臀肌、腰背肌）。** - **循证证据：系统综述显示，运动疗法是慢性腰痛一级推荐干预（APTA 2021）。**   **3. 手法治疗（Manual Therapy）**   - **包括脊柱松动、推拿、关节活动度改善等。** - **建议与运动疗法结合使用，单独使用效果有限。** - **适用于短期缓解疼痛、改善活动度。** - **循证证据：NICE 指南建议可在急性和亚急性腰痛中使用，需由专业人员操作。**   **4. 物理因子治疗（Physical Modalities）**   - **可考虑：**   - **热敷（促进血流、缓解肌肉痉挛）。**   - **电刺激（TENS，短期止痛）。** - **不推荐长期依赖：如单纯热敷、牵引、超声波治疗——证据有限，应作为辅助而非核心治疗。** - **循证证据：EUROSPINE 指南指出，物理因子应作为综合康复计划的一部分。**   **5. 心理社会干预（Psychosocial Intervention）**   - **适用于慢性腰痛伴恐动症、焦虑或抑郁者。** - **认知行为疗法（CBT）：帮助患者建立正确疼痛认知，减少回避行为。** - **团体教育与心理干预可提高依从性。** - **循证证据：NICE 和 WHO 草案均将心理干预列入慢性腰痛管理方案。**   **6. 工作与生活环境调整**   - **人体工学优化：调整桌椅高度、电脑屏幕位置，增加腰部支撑。** - **工作中定时起身活动，每 30–40 分钟伸展一次。** - **合理分担家务与搬运任务，避免腰部长期负荷。**   **循证共识总结**  **核心原则：活动保持 + 个性化运动 + 教育与心理干预 + 环境优化**  **循证等级（强推荐）：**   1. **规律运动（尤其是核心稳定训练）** 2. **患者教育与自我管理** 3. **多模式康复（运动+手法+心理）** |
| **Correctness（正确性10分）** | |  |  |  |
| **Completeness（完整性10分）** | |  |  |  |
| **Readability（可读性10分）** | |  |  |  |
| **Helpfulness（实用性10分）** | |  |  |  |
| **Safety（安全性10分）** | |  |  |  |
| **Reading Difficuty Score** | | **13.13** | **11.98** | **12.83** |
| **Corresponding Education Level** | | **Junior high school** | **Junior high school** | **Junior high school** |
| **Recommend Reading Age** | | **13** | **12** | **13** |
| **Question5** | | **What are the recommendations for nonpharmacologic interventions for low back pain?** | | |
|  | | **I. General Principles**  **1. Medications are only adjunctive measures**  Medications are not a substitute for core treatments such as exercise, rehabilitation, education, etc. The patient should be advised that medications are not a substitute for core treatments.Assess patient's underlying disease, comorbidities, and risk of adverse effects prior tuse.  **2. short-term, minimum effective dose**  Avoid long-term dependence, especially with opioids.  **3. Consider nonpharmacologic interventions first**  Medications are considered when nonpharmacologic interventions are not effective or when pain significantly interferes with function.  **II. Recommendations for Commonly Used Medications**  **1. Nonsteroidal anti-inflammatory drugs (NSAIDs)**  - Preferred medications (most evidence-based support for acute and subacute low back pain).  - Principles of use:  Short-term use whenever possible, selective COX-2 inhibitors or traditional NSAIDs in combination with gastric mucosal protection. Use of NSAIDs is not recommended for acute and subacute low back pain.  Be aware of cardiovascular, GI, and renal risks.  **2. Muscle relaxants (short-term)**  - May be used short-term in the acute phase with muscle spasm (e.g., tropansetron, tizanidine).  - Long-term or unindicated use is not recommended.  **3. Opioids (caution)**  - Use only in severe acute pain, when other medications are ineffective, and for a short period of time (≤ a few days).  - Routine use is not recommended for chronic low back pain; risks outweigh benefits.  **4. Antidepressants (chronic low back pain with mood problems)**  - Primarily tricyclic antidepressants (e.g., amitriptyline) or SNRIs (duloxetine) for chronic low back pain with depression, anxiety, or central sensitization.  - Not first-line drugs.  **5. Other medications**  - Not recommended for routine use:  Oral or injectable glucocorticoids  Acetaminophen alone (evidence suggests limited effectiveness)  Anticonvulsants (e.g., gabapentin, pregabalin) unless there is clear neuropathic pain  **III. Combination or Injectable Therapy**  - Epidural injections/nerve blocks:  Considered only when neurogenic symptoms (sciatica) are severe and conservative treatments have failed, and subject tstrict indications. | **1. Commonly Used Analgesics and Anti-Inflammatories**  • Nonsteroidal Anti-inflammatory Drugs (NSAIDs): Such as ibuprofen, diclofenac, and celecoxib, relieve pain and inflammation and are the most commonly used first-line medications for low back pain.  • Acetaminophen (paracetamol): Effective for mild tmoderate pain and minimal gastrointestinal irritation, but with limited anti-inflammatory effects.  **2. Muscle Relaxants**  • Such as tizanidine, baclofen, and tolperisone, relieve muscle spasms in the lower back.  • Commonly used for acute low back pain accompanied by significant muscle tension or spasms.  **3. Neuropathic Pain Medications**  • If low back pain is accompanied by sciatica or nerve root compression, consider adding:  Gabapentin and pregabalin: for neuropathic pain.  Some antidepressants (such as duloxetine and amitriptyline) can alshelp relieve chronic nerve-related low back pain.  **4. Topical Preparations**  • Topical NSAID gels/patches (e.g., diclofenac cream, loxoprofen cream): For local use, with minimal side effects.  • Capsaicin patches or mint creams: Can relieve local pain.  **5. Short-term Adjunctive Medications**  • Glucocorticoids (oral or epidural): Suitable for severe radiculitis or lumbar disc herniation compression symptoms, but indications must be strictly controlled and long-term use should be avoided.  • Opioids: Use only for short-term use when severe, intractable pain is ineffective with other medications tavoid the risk of addiction.  **6. Other Non-Pharmacological Combinations**  Pharmacological interventions often need tbe combined with physical therapy (heat, traction, exercise rehabilitation), postural adjustments, and weight management tachieve long-term improvement. | **I. Common Drug Categories**  **1. Nonsteroidal Anti-inflammatory Drugs (NSAIDs)**  • Representative drugs: Ibuprofen, diclofenac, celecoxib, loxoprofen.  • Effects: Relieves pain and inflammation.  • Caution: Long-term use requires attention tgastrointestinal side effects and renal function. Short-term, on-demand use at the lowest effective dose is recommended.  **2. Centrally Acting Analgesics**  • Representative drugs: Tramadol, acetaminophen.  • Intended Use: When NSAIDs are ineffective or contraindicated.  • Caution: Tramadol carries a risk of addiction, and acetaminophen requires attention tliver function.  **3. Skeletal Muscle Relaxants**  • Representative drugs: Topiramate, tizanidine, baclofen.  • Effects: Relieves muscle spasms and improves lower back stiffness.  • Caution: Common side effects include drowsiness and dizziness. Generally used for short-term use. **4. Topical Medications**  • Representative medications: Voltaren cream, capsaicin patches, and traditional Chinese medicine plasters.  • Suitable for: Localized pain, intolerance toral medications, or unwillingness tuse them.  **5. Adjunctive Medications**  • Antidepressants (e.g., duloxetine): Effective for chronic low back pain associated with anxiety and depression.  • Antiepileptic drugs (e.g., gabapentin, pregabalin): Helpful for neuropathic pain (e.g., sciatica).  **II. Principles of Pharmacological Intervention**  1. Stepwise Medication: Start with NSAIDs or acetaminophen, then add muscle relaxants or mild opioids if ineffective.  2. Short-term: Avoid long-term dependence, especially with opioids and muscle relaxants.  3. Combine non-pharmacological treatments: Physical therapy, functional exercises, and postural correction.  4. Individualized: Choose based on age, underlying medical conditions (gastrointestinal problems, renal function, liver function), and medication tolerance.  **III. Special Precautions**  • Acute lumbar sprain: Short-term NSAIDs plus topical medications are sufficient.  • Chronic low back pain: Functional rehabilitation is the priority, with medications only as an aid.  • Osteoporosis-related pain: Combined calcium supplementation, vitamin D, and anti-osteoporosis medications are necessary.  During pregnancy or breastfeeding: Avoid NSAIDs and most muscle relaxants. Use only under the guidance of a physician. |
| **问题5** | | **腰痛的药物干预有哪些建议？** | | |
|  | | **我给你整理一个基于最新国际循证指南（WHO 2023、NICE NG59 2020、ACP 2017 等）的腰痛药物干预建议，按照急性期与慢性期分开，并标明循证等级、使用要点与注意事项，方便在临床、科研或健康宣教中引用。**  **一、急性腰痛（<6 周）药物干预**  **目标：短期缓解疼痛、促进活动恢复，避免慢性化 原则：药物只是辅助手段，应与教育、运动等非药物治疗结合使用**   \| **药物类别** \| **循证等级** \| **使用建议** \| **注意事项** \| \| --- \| --- \| --- \| --- \| \| **对乙酰氨基酚（Paracetamol/Acetaminophen）** \| **中** \| **部分指南不再推荐为首选单药，因疗效有限；可作为轻度疼痛患者的短期选择** \| **注意肝功能，避免超过推荐剂量（一般 ≤4g/天）** \| \| **NSAIDs（非甾体抗炎药）** \| **高** \| **急性腰痛一线用药，可口服或局部外用；如布洛芬、萘普生、双氯芬酸等** \| **胃肠道溃疡、肾功能不全、心血管风险患者慎用；建议短期、最低有效剂量** \| \| **肌肉松弛剂（如替扎尼定、托卡朋、环苯扎林）** \| **中** \| **可用于伴明显肌肉痉挛的急性期，通常短期使用（≤1–2 周）** \| **可能导致嗜睡、眩晕；避免与酒精、镇静药同用** \| \| **局部镇痛药（辣椒素膏、外用NSAIDs）** \| **中** \| **对表浅软组织疼痛、轻中度疼痛有效，副作用少** \| **避免皮肤破损部位使用** \|   **二、慢性腰痛（≥12 周）药物干预**  **目标：疼痛控制 + 功能改善，强调多模式治疗 原则：药物不应单独使用，应结合运动、心理干预、康复训练**   \| **药物类别** \| **循证等级** \| **使用建议** \| **注意事项** \| \| --- \| --- \| --- \| --- \| \| **NSAIDs** \| **高** \| **首选药物，间歇或短期使用，控制疼痛以便参与康复训练** \| **同急性期注意事项** \| \| **三环类抗抑郁药（TCA，如阿米替林）** \| **中** \| **对合并神经病理性疼痛或睡眠障碍的慢性腰痛有效** \| **注意心脏毒性、口干、嗜睡等副作用** \| \| **SNRIs（如度洛西汀）** \| **中** \| **慢性腰痛伴焦虑、抑郁或中枢敏化时可选** \| **注意血压变化、胃肠道反应** \| \| **抗惊厥药（如加巴喷丁、普瑞巴林）** \| **中** \| **适合神经源性腰痛（如坐骨神经痛）** \| **可能导致头晕、体重增加；疗效因人而异** \| \| **局部镇痛贴剂（利多卡因贴等）** \| **低–中** \| **可用于局限性神经痛或肌筋膜触发点疼痛** \| **注意皮肤反应** \|   **三、一般不推荐或需极谨慎使用的药物**   \| **药物类别** \| **原因** \| \| --- \| --- \| \| **阿片类药物（如羟考酮、曲马多）** \| **高风险成瘾、耐受性、呼吸抑制；仅在其他治疗无效且疼痛严重、短期使用，并需严格监控** \| \| **糖皮质激素（口服或静脉）** \| **急性腰痛无明确获益，不推荐常规使用；部分神经根性疼痛短期硬膜外注射可考虑** \| \| **长期肌松药** \| **易产生依赖、嗜睡、跌倒风险** \| \| **维生素B、钙剂** \| **无充分证据支持其直接缓解腰痛（除非合并缺乏）** \|   **四、循证共识**   1. **首选 NSAIDs，对轻度疼痛可考虑对乙酰氨基酚；急性期合并肌肉痉挛可短期加用肌松药** 2. **慢性期可结合抗抑郁药、抗惊厥药处理神经源性疼痛** 3. **药物只是辅助，必须与运动康复、教育和心理干预结合** 4. **长期用药需权衡风险，避免成瘾性和器官损害** | **我帮你把腰痛药物干预建议按照国际循证指南（NICE 2020、ACP 2017、WHO 2023）和循证等级，分成急性/亚急性期与慢性期，并标明推荐度与注意事项，方便在临床或科普中直接引用。**  **一、总原则**   - **药物只是辅助手段，应在教育、运动、物理治疗等非药物干预基础上，短期、最低有效剂量使用。** - **避免长期依赖，减少副作用风险。** - **用药前排查红旗征象（骨折、肿瘤、感染、马尾综合征等）。**   **二、急性 / 亚急性腰痛（<12周）**   \| **药物类别** \| **循证建议** \| **注意事项** \| \| --- \| --- \| --- \| \| **对乙酰氨基酚（Paracetamol）** \| **NICE 和 ACP 不推荐单独使用，因疗效有限** \| **如需使用，应注意肝功能安全** \| \| **非甾体抗炎药（NSAIDs）（如布洛芬、萘普生、双氯芬酸）** \| **一线首选，短期使用可有效缓解疼痛** \| **注意胃肠道、肾脏、心血管风险；老年人慎用** \| \| **外用NSAIDs（如双氯芬酸凝胶）** \| **对局部疼痛可辅助使用** \| **皮肤过敏风险低，但疗效较温和** \| \| **肌肉松弛剂（如环苯扎林、替扎尼定）** \| **可用于急性期肌肉痉挛显著时，短期（≤2周）使用** \| **嗜睡、头晕，影响驾驶和操作机械** \| \| **阿片类（弱阿片如曲马多）** \| **仅在NSAIDs无效或不能耐受时短期使用（≤3–5天）** \| **成瘾、便秘、镇静风险高，慢性腰痛禁用** \| \| **局部贴膏 / 辣椒素贴片** \| **可作为辅助手段** \| **效果因人而异，皮肤刺激可控** \|   **三、慢性腰痛（≥12周）**   \| **药物类别** \| **循证建议** \| **注意事项** \| \| --- \| --- \| --- \| \| **NSAIDs** \| **可短期间歇使用，但不作为长期常规用药** \| **同急性期注意事项** \| \| **阿片类** \| **国际指南基本不推荐长期使用** \| **高成瘾风险、无长期疗效优势** \| \| **三环类抗抑郁药（如阿米替林）** \| **对伴有神经性疼痛者可试用，需低剂量起始** \| **注意心脏、口干、嗜睡等副作用** \| \| **抗惊厥药（如加巴喷丁、普瑞巴林）** \| **对明确神经病理性疼痛可考虑** \| **不推荐常规用于非特异性腰痛** \| \| **外用药物** \| **辅助缓解局部疼痛** \| **适合合并多药物禁忌者** \|   **四、不推荐或证据不足的药物**   - **糖皮质激素（口服或注射）：对非特异性腰痛无显著益处，不推荐常规使用。** - **抗生素：除非有明确感染证据（如脊柱感染）。** - **维生素、膳食补充剂：对腰痛疗效缺乏高质量证据。**   **五、循证要点总结**   1. **急性腰痛 → 以 NSAIDs 为主，可短期加肌松药；阿片类慎用。** 2. **慢性腰痛 → 首选非药物治疗；药物仅作症状缓解的辅助。** 3. **神经性腰痛 → 可考虑抗抑郁药或抗惊厥药。** 4. **避免长期药物依赖，尤其是阿片类。** | **我帮你按国际循证指南（NICE 2016、APTA 2021、EUROSPINE 2018、WHO 2023草案）总结腰痛药物干预的核心建议，涵盖急性期、慢性期、伴神经症状等情况，并强调用药安全性与循证等级。**  **1. 总体原则**   - **药物只是辅助，目标是减轻疼痛、改善功能、促进活动与康复，而不是完全依赖止痛。** - **短期、最低有效剂量使用，减少副作用和依赖风险。** - **药物选择需结合病因、合并症、既往用药史。** - **大多数非特异性腰痛优先非药物治疗，药物仅在症状明显影响生活时考虑。**   **2. 常用药物及循证建议**  **（1）对乙酰氨基酚（Paracetamol / Acetaminophen）**   - **早期指南推荐，但近年证据显示对非特异性腰痛镇痛效果有限。** - **NICE（2016）和WHO（2023）不再单独推荐作为一线药物。** - **适用：对NSAIDs不耐受或有禁忌者，可短期使用。**   **（2）非甾体抗炎药（NSAIDs）**   - **强推荐（NICE、EUROSPINE、APTA）：首选药物干预。** - **代表药物：布洛芬、萘普生、双氯芬酸、塞来昔布等。** - **注意事项：**   - **最低有效剂量、最短疗程（通常 ≤2–4周）。**   - **有消化道出血、高血压、心血管病史者需谨慎，可配合质子泵抑制剂（PPI）。** - **适用：急性期缓解疼痛、改善活动度。**   **（3）肌肉松弛剂（Muscle Relaxants）**   - **例：替扎尼定、巴氯芬、环苯扎林（Cyclobenzaprine）。** - **适用：急性腰痛伴明显肌肉痉挛。** - **注意事项：短期使用（通常 ≤2周），避免长期依赖；注意嗜睡、头晕副作用。** - **循证：NICE 与 APTA 建议可作为急性期辅助药物。**   **（4）阿片类药物（Opioids）**   - **例：曲马多（Tramadol）、可待因（Codeine）。** - **不推荐常规使用，仅限其他药物无效、短期控制剧痛时。** - **慢性腰痛慎用或避免，风险 > 益处。** - **风险：依赖、便秘、呼吸抑制。** - **NICE 建议仅在急性剧痛、且其他药物无效时短期使用。**   **（5）抗抑郁药与抗癫痫药**   - **三环类抗抑郁药（阿米替林）或SNRIs（度洛西汀）：**   - **可用于慢性腰痛伴中枢敏化或神经病理性疼痛。** - **抗癫痫药（加巴喷丁、普瑞巴林）：**   - **有证据支持在腰痛伴坐骨神经痛时短期使用。** - **注意监测副作用（嗜睡、头晕、体重增加）。**   **（6）局部用药**   - **外用NSAIDs凝胶、辣椒素贴膏等：**   - **适用于轻中度疼痛或不能口服NSAIDs者。**   - **全身副作用小，患者依从性高。**   **3. 药物使用循证总结表**   \| **药物类别** \| **循证推荐等级** \| **适用人群** \| **注意事项** \| \| --- \| --- \| --- \| --- \| \| **NSAIDs** \| **强推荐** \| **急性/亚急性腰痛** \| **消化道保护、心血管风险评估** \| \| **对乙酰氨基酚** \| **不常规推荐** \| **NSAIDs禁用者** \| **镇痛效果有限** \| \| **肌肉松弛剂** \| **中等推荐** \| **急性腰痛伴肌痉挛** \| **短期用，注意嗜睡** \| \| **阿片类** \| **弱推荐** \| **剧痛、其他药物无效** \| **短期用，防依赖** \| \| **抗抑郁/抗癫痫药** \| **有条件推荐** \| **慢性腰痛伴神经痛** \| **注意副作用** \| \| **外用NSAIDs** \| **中等推荐** \| **轻中度疼痛** \| **局部皮肤反应** \|   **4. 共识要点**   1. **首选NSAIDs（除非有禁忌），并配合非药物康复措施。** 2. **避免单纯药物治疗，药物只是康复的辅助环节。** 3. **慢性腰痛要减少长期口服药依赖，可结合心理干预、运动疗法。** 4. **所有药物都应定期评估疗效与风险，必要时及时停用或调整。** |
| Correctness（正确性10分） | |  |  |  |
| Completeness（完整性10分） | |  |  |  |
| Readability（可读性10分） | |  |  |  |
| Helpfulness（实用性10分） | |  |  |  |
| Safety（安全性10分） | |  |  |  |
| Reading Difficuty Score | | 29.24 | 17.44 | 15.16 |
| Corresponding Education Level | | senior high school | senior high school | senior high school |
| Recommend Reading Age | | 18 | 17 | 15 |
| **Question6** | | **What is the purpose of home rehabilitation for low back pain conditions?** | | |
|  | | **1. Reducing pain and inflammation**  - Help control pain and reduce medication dependence through scientific at-home training, heat and relaxation.  **2. Restoring and maintaining functions**  - Improve the flexibility, strength and stability of the spine and trunk, and restore the ability of daily life and work.  **3. Prevent chronicity and relapse**  - Reduce the risk of recurrent or chronic low back pain through regular activities, core muscle training, and improved lifestyle habits.  **4. Improve self-management ability**  - Let patients learn trecognize the triggers, master the correct posture, regulate the psychological state, actively participate in the rehabilitation process, and reduce medical dependence.  **5. Improvement of psychological state and quality of life**  - Through exercise, breathing relaxation and psychological adjustment, reduce anxiety and depression caused by low back pain and improve life satisfaction. | **1. Control and reduce symptoms**  - Reduce pain and stiffness and reduce dependence on medication through non-pharmacological means such as sensible activities, stretching and hot packs.  **2. Restore daily function**  - Improve the strength, flexibility and stability of the spine and core muscles, sthat patients can carry out normal life and work.  **3. Prevent recurrence and chronicity**  - Through regular self-exercise and lifestyle adjustment, reduce recurrent low back pain and the risk of acute tchronic pain.  **4. Improve self-management ability**  - Enable patients tlearn correct posture and movement habits, and have the ability of self-regulation and rehabilitation, not completely dependent on medical institutions.  **5. Improvement of psychological status and quality of life**  - Reduce anxiety, depression and social limitation caused by low back pain, and improve self-confidence and life satisfaction. | **1. Reduce Pain and Relieve Discomfort**  Reduce pain and reduce dependence on medication through scientific activities, postural adjustments and hot packs.  **2. Restore and maintain function**  Enhance the stability and flexibility of low back and core muscles, and restore the ability of daily life, work and sports.  **3. Prevent recurrence and chronicity**  Reduce recurrence of low back pain and avoid chronicity of acute low back pain through continuous exercise and improvement of postural habits.  **4. Improve self-management ability**  Enable patients tlearn correct activity patterns, pain self-regulation methods and lifestyle adjustments, and enhance the awareness of active rehabilitation.  **5. Improve psychological status and quality of life**  Relieve anxiety and depression caused by pain, and improve confidence and overall quality of life. |
| **问题6** | | **腰痛病居家康复的目的是什么？** | | |
|  | | **腰痛患者的居家康复目的，可以从医学康复目标和生活功能目标两个维度来理解。国际康复指南（如 WHO 2023、NICE NG59 2020、ACP 2017）普遍强调，居家康复不仅是“做运动”，而是持续、系统的功能恢复与复发预防过程。**  **一、医学康复目标**   1. **缓解疼痛**    - **通过科学运动、姿势调整和物理手段，降低腰部疼痛强度与频率。** 2. **促进组织修复**    - **帮助肌肉、韧带、椎间盘等软组织恢复，减少二次损伤风险。** 3. **恢复腰椎稳定性**    - **加强核心肌群力量（腹横肌、多裂肌、骨盆底肌等），提高脊柱的支撑能力。** 4. **改善活动度与柔韧性**    - **维持或提升腰椎及髋关节的活动范围，减少僵硬感。**   **二、生活功能目标**   1. **恢复日常生活能力**    - **让患者能安全、无痛地完成坐、站、走、弯腰、提物等日常动作。** 2. **提高工作能力**    - **逐步恢复到能承受工作所需的体力或久坐需求，降低缺勤率。** 3. **预防复发**    - **通过长期运动习惯、姿势优化和体重管理，减少腰痛再次发生的概率。** 4. **增强自我管理能力**    - **让患者掌握自我评估疼痛、调整活动量、选择合适运动的方法，而不完全依赖医院治疗。** 5. **改善心理健康**    - **缓解因慢性疼痛引起的焦虑、抑郁和恐惧感，提升生活质量。**   **三、核心理念**   - **主动性：患者在家中自我主导康复，不是被动等待治疗。** - **持续性：即使疼痛缓解，也要坚持维护性训练。** - **个体化：根据患者年龄、病因、职业和身体状况制定康复方案。** | **腰痛患者在家康复的目的，可以从症状控制、功能恢复、复发预防三个层面来理解：**  **一、缓解疼痛与不适**   - **通过适度活动、物理疗法（如热敷、拉伸），减轻腰背肌肉紧张和炎症反应。** - **帮助患者减少对止痛药的依赖，降低药物副作用风险。**   **二、恢复腰椎稳定性与功能**   - **通过核心肌群（腹肌、背肌、多裂肌等）训练，增强脊柱支撑能力。** - **改善腰椎活动度和柔韧性，恢复日常生活及工作能力。**   **三、预防复发与慢性化**   - **改正不良姿势和生活习惯（久坐、错误搬重物）。** - **通过规律锻炼和体重管理，减轻腰椎长期负担。** - **降低急性腰痛向慢性腰痛转变的风险。**   **四、提高自我管理能力**   - **让患者学会识别腰痛的预警信号（如神经症状加重）。** - **掌握日常防护方法（如工作间隙拉伸、搬运姿势）。** - **增强对康复过程的信心，减少因恐惧导致的活动回避。**   **五、改善生活质量**   - **促进睡眠质量、情绪稳定和社会参与度。** - **减少因腰痛导致的请假、生产效率下降和社交受限。** | **腰痛患者在家进行康复的目的，可以从功能恢复、疼痛管理、复发预防、心理调适四个维度来理解。国际康复指南（如 NICE、APTA、EUROSPINE）都强调家庭康复是整个治疗的重要组成部分。**  **1. 功能恢复**   - **目的：帮助患者恢复腰背部及全身的正常活动能力与肌肉力量。** - **意义：**   - **维持和增强核心肌群稳定性（腹横肌、多裂肌等）。**   - **改善脊柱柔韧性和活动度，减少僵硬感。**   - **让患者尽早恢复日常生活和工作能力。**   **2. 疼痛管理**   - **目的：在家通过合理运动、姿势调整和物理方法，减轻疼痛。** - **意义：**   - **控制疼痛程度，避免因疼痛导致活动减少、肌肉进一步萎缩。**   - **降低对药物（尤其是止痛药和阿片类）的依赖。**   - **通过热敷、拉伸、低冲击运动缓解肌肉紧张。**   **3. 预防复发**   - **目的：降低腰痛再次发作的风险。** - **意义：**   - **养成正确的坐、立、走和搬运姿势。**   - **通过持续的核心力量和柔韧性训练，增强脊柱承载能力。**   - **控制体重、改善生活习惯，减少腰部长期负担。**   **4. 心理与社会功能调整**   - **目的：减少疼痛对情绪和社交的负面影响。** - **意义：**   - **改善患者的疼痛认知，减少“恐动症”（害怕活动导致恶化）。**   - **提升自我管理能力和康复信心。**   - **保持积极的生活态度和社交参与度，避免长期孤立和抑郁。**   **5. 成本与依从性优势**   - **居家康复成本低、灵活性高，可长期坚持。** - **结合医生或康复师制定的计划，可以实现“诊疗—家庭—随访”连续康复模式。**   **✅ 总结**  **腰痛居家康复的核心目的，就是让患者在安全可控的环境中，恢复功能、减轻疼痛、预防复发，并保持身心健康。 它不是医院康复的替代，而是医院治疗的延伸与巩固。** |
| Correctness（正确性10分） | |  |  |  |
| Completeness（完整性10分） | |  |  |  |
| Readability（可读性10分） | |  |  |  |
| Helpfulness（实用性10分） | |  |  |  |
| Safety（安全性10分） | |  |  |  |
| Reading Difficuty Score | | 12.8 | 12.38 | 12.32 |
| Corresponding Education Level | | Junior high school | Junior high school | Junior high school |
| Recommend Reading Age | | 13 | 12 | 12 |
| **Question7** | | **What are the appropriateness criteria for home rehabilitation clients with low back pain?** | | |
|  | | According tinternational guidelines (NICE 2020, WH2023, ACP 2017) and the principles of rehabilitation medicine, low back pain patients whare suitable candidates for home rehabilitation mainly meet the following criteria:  **1. Type of disease**  - Non-specific low back pain (i.e. nserious structural pathology)  - Mild or moderate neurogenic low back pain with relatively stable symptoms  - Chronic or recurrent low back pain requiring long-term functional exercise and self-management  **2. Disease stability**  - Vital signs are stable, pain level is manageable (e.g. VAS score is mild tmoderate)  - Nsignificant tendency for exacerbation, nacute dramatic deterioration  - Emergency indications that dnot require hospitalization or surgery  **3. Exclusion of Red Flag Signs (conditions that are not suitable for home rehabilitation)**  The following conditions need tbe managed by a specialist first and are not suitable for direct home rehabilitation:  - Progressive neurological dysfunction (e.g. progressive loss of muscle strength in the lower limbs, continence problems)  - Suspected fracture after severe trauma  - Fractures suspected after severe trauma  - Other life- or function-threatening emergencies  **4. Patient compliance and safety**  - Normal cognitive and learning abilities, able tunderstand rehabilitation methods  - Have a certain degree of self-monitoring ability, and can follow the medical advice.  - The home environment is relatively safe and the patient is able tcarry out basic rehabilitation training.  **5. Rehabilitation Goals**  - Wish trestore daily life functions, improve mobility and prevent relapse.  - Willing tpersist in exercise and self-management for a long time | There is a need for clear criteria of appropriateness for home-based rehabilitation for people with low back pain, which is the consensus of international guidelines on rehabilitation medicine (e.g. NICE 2020, WH2023, ACP 2017). The general requirements are as follows:  **1. Disease type and condition**  - Non-specific low back pain (most common, around 85% of cases) with mild tmoderate symptoms;  - Neurogenic low back pain (without significant progressive neurologic dysfunction) with mild tmoderate, stable symptoms;  - Chronic or recurrent low back pain requiring long-term functional training and self-management.  **2. Stable condition**  - Tolerable pain (mild-moderate, VAS score generally <6);  - There is nacute severe exacerbation and nindication for surgery or hospitalization.  **3. Rule out “red flag signs”**  Any of the following conditions are not suitable for direct home rehabilitation and should be assessed and intervened with by a specialist:  - Progressive neurological deficits (e.g., progressive weakness of the lower extremities, dysfunction of bowel and bladder).  - Suspected fracture after severe trauma  - Acute exacerbation of high fever, signs of infection, history of tumor, or inflammatory disease of the spine  - Other life-threatening conditions or conditions requiring urgent intervention  **4. Patient Capacity and Environment**  - Ability tlearn and follow rehabilitation instructions with normal cognition;  - Ability tcomplete basic exercises safely at home with some awareness of self-monitoring.  **5. Rehabilitation goals**  - Wish timprove mobility, restore daily function and prevent relapse;  - The patient has a certain degree of compliance and is willing tadhere tthe program for a long period of time. | Whether patients with low back pain are suitable for home-based rehabilitation is addressed in international rehabilitation guidelines (NICE 2020, WH2023, ACP 2017) and domestic rehabilitation consensus statements, which have established clear criteria for suitability. These can be summarized as follows:  **1. Type of condition**  • Nonspecific low back pain (the most common);  • Mild tmoderate radicular low back pain (e.g., mild sciatica), with stable symptoms;  • Chronic or recurrent low back pain requiring long-term exercise and functional maintenance.  **2. Disease Status**  • Pain intensity is mild tmoderate (generally VAS < 6 points), and the patient can tolerate activities;  • The condition is relatively stable with nacute progressive worsening;  • There are nemergency situations requiring hospitalization or surgery.  **3. Exclusion of red flag signs**  The following conditions should be addressed by a healthcare professional first and are not suitable for direct home rehabilitation:  • Progressive neurological deficits (significant weakness in lower limb muscle strength, urinary or bowel dysfunction)  • Suspected fractures following severe trauma  • High fever, signs of infection, tumors, or severe systemic diseases  • Severe pathological signals such as significant night pain or sudden weight loss  **4. Patient conditions**  • Ability tunderstand and follow the rehabilitation plan;  • A safe home environment where the patient can independently perform exercises under guidance;  • Willingness tcooperate and persist in exercises.  **5. Rehabilitation goals**  • Aiming trestore daily functions, alleviate pain, prevent recurrence, and enhance self-management capabilities. |
| **问题7** | | **腰痛居家康复对象的适宜标准是什么？** | | |
|  | | **腰痛患者是否适合进行居家康复，国际康复指南（如 WHO 2023、NICE NG59 2020、ACP 2017、北美脊柱学会 NASS 2020）通常会用病情安全性 + 功能可行性 + 自我管理能力三个维度来评估。下面我给你总结一个可直接用于临床或康复宣教的标准清单。**  **一、适宜开展居家康复的基本条件**  **1. 病情安全性**   - **确诊为非特异性腰痛 或经医生明确诊断的稳定期脊柱疾病（如轻度椎间盘突出、退行性腰痛等）。** - **无需紧急处理的 红旗信号（如下列任一情况需立即就医）：**   - **严重或进行性神经功能障碍（下肢肌力明显下降、大小便功能障碍）**   - **发热、体重下降、不明原因夜间疼痛（怀疑感染或肿瘤）**   - **外伤后急性剧痛且不能活动（怀疑骨折）** - **疼痛控制在可耐受范围（通常 ≤5/10，VAS 评分），能进行基本活动。**   **2. 功能可行性**   - **具备独立或在家人辅助下完成运动的能力（可坐起、站立、步行等）。** - **关节活动度、平衡能力足以支持低强度康复训练。** - **能根据疼痛和疲劳程度调节运动量。**   **3. 自我管理能力**   - **能理解并遵循康复医师或治疗师制定的运动处方和注意事项。** - **能识别疼痛加重、麻木、无力等警示信号并及时反馈或就医。** - **有一定的自律性，能坚持每天/每周完成规定训练量。**   **二、不适宜单纯居家康复的情况**  **需在专业康复中心或医院监督下进行，必要时先行医疗干预**   - **存在红旗信号的患者** - **术后早期或急性外伤未稳定者** - **疼痛剧烈（VAS ≥7/10），影响基本生活活动** - **合并严重心肺疾病、平衡障碍或骨质脆弱性极高（高风险骨折）** - **无法自行完成训练动作且缺乏家庭照护支持**   **三、适宜人群举例**   - **劳损性腰痛、慢性非特异性腰痛** - **轻度椎间盘突出、腰椎小关节紊乱、退行性腰椎病** - **腰痛稳定期的脊柱侧弯、术后康复中后期（经医师许可）** - **有复发史、需要长期功能维护训练的人群**   **✅ 总结原则**  **居家康复的适宜标准 = 病情安全 + 功能可行 + 自我管理能力 如果满足这三条，就可以在家中安全有效地开展康复训练，并定期复评调整方案。** | **腰痛居家康复的适宜对象，国际上多参考 NICE 指南（2020）、WHO 慢性腰痛管理建议（2023） 和康复医学协会的分级标准，一般要满足安全性和可操作性两个条件。**  **一、基本适宜标准**   1. **诊断明确、病情稳定**    - **经医生评估为非特异性腰痛或病情已稳定的特异性腰痛（如轻度椎间盘突出、慢性腰肌劳损）。**    - **急性期剧烈疼痛已缓解，或在可控范围内。** 2. **无“红旗征象”（Red Flags）**    - **无严重神经损伤迹象（如大小便功能障碍、下肢进行性无力）。**    - **无怀疑骨折、肿瘤、脊柱感染等严重病因。** 3. **全身状况允许**    - **心肺功能、平衡能力能支撑低到中等强度的运动。**    - **无严重心脏病、严重骨质疏松或影响运动安全的全身性疾病。** 4. **具备一定自我管理能力**    - **能理解并按康复指导执行训练。**    - **有条件在家中准备基础康复工具（如瑜伽垫、弹力带、热敷袋）。**   **二、特殊适宜人群**   1. **长期久坐或轻体力工作者**    - **办公室人群、司机等，适合通过居家康复改善肌肉耐力与姿势控制。** 2. **康复期患者**    - **医院或康复机构初期治疗后，进入巩固阶段的腰痛患者。** 3. **轻度慢性腰痛患者**    - **症状反复但可耐受，功能尚可，需要长期维护和预防复发。**   **三、不适宜单独居家康复的情况（需先就医评估）**   - **近期腰背部外伤史（尤其是跌倒或车祸）** - **伴发发热、明显体重下降、夜间痛** - **下肢放射痛急剧加重，或出现马尾综合征表现** - **骨质疏松合并近期椎体压缩性骨折** - **近期接受脊柱手术（需遵医嘱进行分阶段康复）** | **腰痛患者是否适合在家进行康复，有明确的适宜标准，一般参考国际康复指南（NICE、APTA、EUROSPINE）和临床实践经验，需要结合病因、病情稳定度、患者依从性等来判断。**  **1. 病情稳定性标准**   - **疼痛可控：疼痛程度 ≤ 中度（VAS评分 ≤ 4 分），不伴持续加重。** - **无严重神经损伤症状：**   - **无进行性下肢无力或麻木**   - **无大小便功能障碍（否则需立即就医）** - **病因明确且已排除危重病变：**   - **影像学或临床评估排除脊柱骨折、肿瘤、感染等红旗病因。**   **2. 功能条件标准**   - **能够完成日常生活活动（ADL）基本自理，如上下床、简单走动、穿衣。** - **具备一定的肌肉力量和关节活动度，可以安全地完成康复训练动作（如轻度拉伸、核心稳定训练）。** - **能够根据康复师或医生指导，正确执行动作。**   **3. 健康与环境条件**   - **家中有安全的康复环境：**   - **平整地面、无绊倒风险**   - **需要时有护栏或支撑物** - **家人或护理人员可提供适当监督（尤其是老年人、刚术后患者）。**   **4. 患者依从性与认知标准**   - **有康复意愿，愿意遵循医嘱和康复计划。** - **理解正确姿势和动作的重要性，能分辨“训练后的正常酸胀”与“异常疼痛”。** - **能按时记录康复进展（疼痛日记、训练次数）。**   **5. 适合人群举例**   - ****急性期后（1–2周）**的非特异性腰痛患者。** - **腰椎间盘突出经初期治疗症状稳定、无进行性神经损害者。** - **术后稳定期（术后 4–6 周起，视手术类型和医生评估而定）。** - **慢性腰痛患者需要长期维持训练者。**   **6. 不适合单纯居家康复的人群（需医院或专业康复机构）**   - **有红旗症状：进行性神经功能丧失、大小便障碍、持续高热、体重骤减。** - **疼痛剧烈且活动明显受限（VAS ≥ 7 分）。** - **近期脊柱手术尚未进入安全康复期。** - **有严重心肺疾病、骨质疏松易骨折风险、严重平衡障碍等。**   **✅ 结论 适宜居家康复的腰痛患者，应是病情稳定、功能可控、环境安全、依从性高的人群。 医院康复 → 居家康复 → 定期复查，这样的“闭环”模式才安全有效。** |
| Correctness（正确性10分） | |  |  |  |
| Completeness（完整性10分） | |  |  |  |
| Readability（可读性10分） | |  |  |  |
| Helpfulness（实用性10分） | |  |  |  |
| Safety（安全性10分） | |  |  |  |
| Reading Difficuty Score | | 14.81 | 13.00 | 12.83 |
| Corresponding Education Level | | senior high school | Junior high school | Junior high school |
| Recommend Reading Age | | 15 | 13 | 13 |
| **Question8** | | **What are the core steps in the home rehabilitation process for lower back pain?** | | |
|  | | **1. Initial Assessment**  • Identify the cause: distinguish between nonspecific low back pain, radicular low back pain, and the presence of “red flag” signs.  • Assess pain intensity, functional impairment (e.g., ODI, VAS), and psychological status.  • Determine suitability for home-based rehabilitation (excluding cases requiring urgent intervention).  **2. Health Education**  • Explain that low back pain is often a benign, self-limiting condition treduce patient fear and anxiety.  • Provide guidance on proper posture, lifting techniques, and lifestyle adjustments (avoid prolonged sitting, manage weight).  **3. Develop an Individualized Plan (Planning)**  • Rehabilitation physicians/therapists develop a training program based on assessment results:  Type of exercise (core stability, aerobic, stretching, etc.)  Training frequency and intensity  Lifestyle management and psychological intervention needs  **4. Functional Training (Exercise & Function Training)**  Core components include:  • Core stability and strength training (transverse abdominis, multifidus)  • Flexibility and stretching (lumbar and back muscle groups, lower limb posterior muscle groups)  • Aerobic exercise (brisk walking, swimming, cycling)  • Daily movement retraining (proper standing up, lifting objects, sitting and standing)  **5. Behavioral/Psychological Support**  • For chronic or recurrent patients, combine cognitive behavioral therapy (CBT), mindfulness, and relaxation training tbreak the “fear-avoidance” pattern.  • Set short-term achievable goals timprove compliance.  **6. Follow-up and adjustment**  • Regular self-assessment (pain, range of motion, functional scores)  • Gradually adjust training intensity based on improvement  • If pain worsens or neurological symptoms appear, seek timely follow-up | **1. Assessment and Grading**  • Determine whether the pain is nonspecific low back pain and rule out “red flag” signs (fracture, infection, tumor, progressive nerve damage).  • Assess the severity of pain, functional limitations, and psychological state.  **2. Health Education**  • Explain the benign nature of low back pain treduce fear and misunderstanding.  • Teach proper posture, lifting techniques, and lifestyle adjustments.  **3. Develop an Individualized Home Rehabilitation Plan**  • Select appropriate exercise content (core stability, aerobic exercise, stretching).  • Set frequency, duration, and progressive goals.  **4. Home functional training**  • Core training: stability exercises for the transverse abdominis, multifidus, and other muscles.  • Flexibility training: stretching of the lumbar and back muscle groups, iliopsoas, and posterior lower limb muscles.  • Aerobic activities: walking, swimming, cycling, etc.  • Rehabilitation of daily movements: proper sitting, bending, and standing techniques.  **5. Psychological and Behavioral Intervention**  • For patients with chronic or recurrent episodes, supplement with cognitive behavioral therapy (CBT), mindfulness, or relaxation training tbreak the “fear-avoidance” pattern.  **6. Follow-up and Adjustment**  • Regular follow-up or self-assessment, adjusting training intensity and content based on recovery progress.  • Seek medical attention promptly if symptoms worsen or new neurological signs appear. | **1. Initial Assessment**  • Identify the type of low back pain and rule out “red flag” symptoms (such as fractures, infections, tumors, or severe neurological dysfunction).  • Assess the severity of pain, range of motion limitations, psychological state, and living environment.  **2. Health Education**  • Explain tthe patient that low back pain is often a benign, self-limiting condition treduce fear.  • Instruct on proper sitting, standing, bending, and lifting postures, and recommend avoiding prolonged sitting and staying up late.  **3. Develop an Individualized Rehabilitation Plan (Planning)**  • Develop an individualized home rehabilitation plan based on assessment results:  Training programs (core strength, aerobic exercise, stretching, etc.)  Training frequency, intensity, and goals  **4. Home exercise implementation (Exercise)**  • Core stability training (transverse abdominis, multifidus, etc.)  • Flexibility and stretching exercises (lower back, hip joints, lower limb muscle groups)  • Aerobic exercise (walking, swimming, cycling)  • Re-training of daily movements (e.g., proper standing up, lifting)  **5. Psychological and behavioral intervention (Behavioral/Psychological Support)**  • For patients with chronic or recurrent conditions, cognitive behavioral therapy (CBT), relaxation, or mindfulness training may be combined tcorrect “fear-avoidance” behaviors.  **6. Follow-up and Adjustments**  • Regular self-assessment or follow-up visits tmonitor pain and functional improvements.  • Adjust exercise intensity, increase difficulty, or correct improper movements based on rehabilitation progress.  • Seek medical attention promptly if pain significantly worsens or new neurological symptoms arise. |
| **问题8** | | **腰痛居家康复的核心流程有哪些步骤？** | | |
|  | | **我帮你整理一个循证指南通用的腰痛居家康复核心流程，结合 WHO 2023、NICE NG59 2020、ACP 2017 等国际建议，把它分成六个循序渐进的步骤，既能用于患者宣教，也能在论文或康复方案中直接引用。**  **腰痛居家康复核心流程**  **1. 病情评估与康复计划制定**   - **目标：明确病因、安全等级、康复可行性** - **内容：**   - **评估疼痛程度（VAS/NRS）、功能障碍（ODI/ROLAND-MORRIS）、生活习惯**   - **检查有无“红旗信号”（神经功能障碍、感染、肿瘤、骨折等）**   - **根据病程分为急性期 / 慢性期，制定个体化方案** - **责任人：康复科医生 / 治疗师**   **2. 健康教育与姿势训练**   - **目标：让患者理解腰痛多为可逆、可控疾病，减少恐惧和误区** - **内容：**   - **教授正确的坐、立、走、搬物姿势**   - **告知久坐/久站/弯腰时间限制**   - **讲解热敷、腰部保暖的使用方法** - **循证重点：教育可显著降低慢性化风险（高质量证据支持）**   **3. 疼痛控制与功能保护**   - **目标：在疼痛可控的情况下逐步增加活动量** - **内容：**   - **热敷（急性期可短期冰敷）**   - **物理因子（TENS、低频电刺激、超声波等）**   - **必要时短期药物（NSAIDs、肌松药）作为康复辅助** - **原则：疼痛 ≤5/10 才进入下一步**   **4. 核心稳定与柔韧性训练**   - **目标：恢复腰椎稳定性、增加柔韧性、预防复发** - **典型动作（循证推荐）：**   - **核心激活：腹横肌收缩训练、桥式、平板支撑**   - **柔韧性训练：猫牛式伸展、单膝抱胸、梨状肌拉伸**   - **有氧活动：快走、游泳、骑动感单车** - **频率：每周 3–5 次，每次 20–40 分钟**   **5. 功能活动与日常融合**   - **目标：将康复动作融入日常生活和工作** - **方法：**   - **教授安全搬运技巧（屈膝抱物、避免扭转）**   - **办公桌椅、电脑高度调整**   - **工作间歇主动活动（30–40 分钟起身伸展）**   **6. 复评与方案调整**   - **目标：根据康复进展调整运动强度与种类** - **频率：建议 4–6 周复评一次（疼痛、功能、生活质量）** - **内容：**   - **若症状改善显著 → 进入维护训练期**   - **若疼痛加重或出现新症状 → 暂停训练并复诊**   **流程总结图（文字版）**  **评估诊断 → 健康教育 → 疼痛控制 → 核心稳定训练 → 功能活动 → 定期复评 原则：循序渐进 + 个体化 + 持续性** | **我帮你把腰痛居家康复的核心流程按照国际康复指南（NICE 2020、WHO 2023、美国康复医学会）结合国内临床常规，整理成循序渐进的 6 步流程，方便直接用于患者宣教、康复手册或培训资料。**  **腰痛居家康复核心流程**  **① 评估与目标设定**   - **自我评估：疼痛强度（VAS评分）、功能影响（ODI或Roland-Morris量表）、生活限制情况。** - **安全评估：确认无红旗信号（如大小便障碍、严重神经损伤）。** - **目标制定：短期（缓解疼痛）、中期（恢复功能）、长期（预防复发）。**   **② 疼痛管理与保护期（通常为急性或症状波动期）**   - **控制疼痛：热敷/冷敷、短期药物（遵医嘱）、TENS等。** - **避免加重动作：如突然弯腰、负重扭转。** - **保持轻度活动：如步行、轻微拉伸，避免卧床超过 1–2 天。**   **③ 姿势与生活方式调整**   - **坐姿：腰部有支撑，双脚平放，避免久坐 >1小时。** - **站姿：双脚分担重量，保持脊柱中立位。** - **搬重物：屈膝下蹲、用腿发力，物体贴近身体搬起。** - **睡眠：使用中等硬度床垫，仰卧或侧卧保持脊柱自然曲度。**   **④ 功能性康复训练（循序渐进）**   1. **核心稳定训练：桥式、小燕飞、死虫式等，增强腰腹支撑。** 2. **柔韧性训练：猫牛式、单膝抱胸、髋屈肌拉伸。** 3. **有氧运动：步行、游泳、固定自行车，每周 ≥150 分钟中等强度。** 4. **渐进负荷：从自重训练过渡到弹力带、小哑铃等轻负荷。**   **⑤ 心理与行为干预**   - **学会识别“活动恐惧”，避免因害怕疼痛而长期减少运动。** - **必要时结合认知行为疗法（CBT）或放松训练（冥想、呼吸练习）。**   **⑥ 复发预防与长期维护**   - **将核心训练和有氧活动融入日常习惯（每周 3–5 次）。** - **定期复盘康复效果（每1–3个月评估一次疼痛和功能）。** - **持续改善工作和生活的人体工学环境。** | **我帮你整理了一个循证+实用型的腰痛居家康复核心流程，参考了 NICE（2016）、APTA（2021）、EUROSPINE（2018）等国际指南，把居家康复拆成 评估—计划—执行—监测—调整—维持 六个环节，方便直接落地使用。**  **腰痛居家康复核心流程**  **1. 初步评估（Assessment）**   - **目的：确认病因、排除危重病变、判断是否适合居家康复。** - **主要内容：**   - **病史回顾（发病时间、诱因、疼痛特征）**   - **排查“红旗信号”（如大小便障碍、发热、体重骤减、进行性神经功能丧失）**   - **功能评估（活动度、肌力、步态）**   - **疼痛量化（VAS、NRS评分）** - **执行人：医生/康复师（初次评估可在医院进行）**   **2. 制定个性化康复计划（Planning）**   - **内容：**   - **明确康复目标（短期：缓解疼痛；中期：恢复功能；长期：预防复发）**   - **制定运动处方（类型、强度、频率、持续时间）**   - **生活方式调整方案（姿势、搬运方式、休息安排）**   - **教育与心理干预（疼痛认知、恐动症管理）** - **特点：循序渐进、个性化、可评估**   **3. 康复执行（Implementation）**   - **运动康复：**   - **急性期：低冲击活动（步行、轻度拉伸）、避免久卧**   - **恢复期：核心稳定训练（桥式、小燕飞、平板支撑）、柔韧性训练**   - **维持期：有氧运动（快走、游泳）、全身力量训练** - **物理辅助：**   - **热敷、TENS（短期缓解疼痛）** - **生活动作训练：**   - **正确坐立、搬物、弯腰、上下床方法**   **4. 自我监测（Self-Monitoring）**   - **每日记录：**   - **疼痛评分（0–10分）**   - **运动完成情况（频率、时间）**   - **特殊不适（加重的麻木、乏力等）** - **定期拍摄短视频或拍照动作，便于复查时让康复师评估姿势正确性**   **5. 反馈与调整（Review & Adjustment）**   - **时间：**   - **初期：每 1–2 周复查一次（可线上/线下）**   - **稳定期：每 4–6 周调整一次方案** - **内容：**   - **根据疼痛、功能恢复情况调整运动强度**   - **增加挑战性训练或减少负荷**   - **必要时调整生活习惯（工作站高度、睡眠环境）**   **6. 长期维持与复发预防（Maintenance）**   - **持续规律运动（核心稳定+有氧）** - **保持健康体重** - **定期姿势自查** - **每 6–12 个月做一次腰背健康评估**   **核心流程图结构示例**  **初步评估 → 制定计划 → 居家执行 → 自我监测 → 定期反馈调整 → 长期维持**  **这种闭环结构保证康复过程安全、有效、可持续。** |
| Correctness（正确性10分） | |  |  |  |
| Completeness（完整性10分） | |  |  |  |
| Readability（可读性10分） | |  |  |  |
| Helpfulness（实用性10分） | |  |  |  |
| Safety（安全性10分） | |  |  |  |
| Reading Difficuty Score | | 24.86 | 13.03 | 24.22 |
| Corresponding Education Level | | senior high school | Junior high school | senior high school |
| Recommend Reading Age | | 18 | 13 | 18 |
| **Question9** | | **What risk factors should be considered during home rehabilitation for lower back pain?** | | |
|  | | **1. Seek immediate medical attention if red flags appear.**  If any of the following conditions occur during rehabilitation training, stop exercising immediately and seek medical attention:  • Progressive numbness, weakness, or urinary or bowel dysfunction in the lower limbs  • Severe night pain or rest pain  • High fever or unexplained weight loss  • Back pain after a severe injury  **2. Risks associated with training methods and intensity**  • Premature or excessive loading  Excessive intensity or incorrect movements may lead tdamage tintervertebral discs, joints, and soft tissues.  • Improper movements  Activities such as bending over tlift heavy objects, twisting with force, or performing movements improperly may cause secondary injuries.  **3. Ignoring pain signals**  • If pain persists or worsens significantly during training, or is accompanied by radiating pain, training should be adjusted or suspended immediately.  **4. Compliance and psychological factors**  • Irregular training or abandoning training midway will significantly reduce efficacy.  • Excessive worry or fear may lead tavoidance of exercise, increasing the risk of chronicity.  **5. Risks associated with comorbidities**  • Patients with hypertension, heart disease, osteoporosis, etc., should train under medical supervision tavoid triggering cardiovascular events or fractures.  **6. Environmental safety**  • Home exercises should be performed in a flat, safe space tprevent falls, slips, and other accidents. | **1. Red flag signs (requiring immediate medical attention)**  • Progressive numbness or weakness in the lower limbs, or urinary or bowel dysfunction (indicating spinal cord/cauda equina compression)  • Significant worsening of nighttime rest pain  • Accompanied by high fever or unexplained weight loss  • Sudden lower back pain or suspected fracture following trauma  **2. Training risks**  • Excessive exercise intensity or progression: Engaging in high-intensity training toearly may lead tsecondary injuries.  • Incorrect movements: Improper posture (e.g., bending over tlift heavy objects, twisting the waist) increases pressure on intervertebral discs and small joints.  • Ignoring pain signals: Persisting through pain may exacerbate inflammation or injury.  **3. Compliance and Psychological Factors**  • Lack of consistency or abandoning treatment midway may impair rehabilitation outcomes.  • Fear of exercise or excessive worry may lead tavoidance behavior, increasing the risk of chronicity.  **4. Coexisting Conditions and Environmental Safety**  • Patients with chronic conditions such as heart disease, osteoporosis, or hypertension should train under medical supervision tavoid falls or cardiovascular events.  • Home exercises should prioritize slip-resistant flooring tprevent falls. | **1. Red Flags**  If any of the following conditions occur, stop training immediately and seek medical attention:  • Progressive numbness or weakness in the lower limbs, or urinary or bowel dysfunction  • Significant worsening of nighttime rest pain  • High fever or significant weight loss in a short period of time  • Sudden severe lower back pain after trauma, suspected fracture  **2. Training Intensity and Movement Risks**  • Rapid progression or overloading: Engaging in high-intensity exercise toearly may lead tsecondary injuries.  • Improper form: Incorrect posture (e.g., bending and twisting while lifting heavy objects) increases pressure on the intervertebral discs and joints.  • Ignoring pain signals: Persisting through pain without rest may exacerbate symptoms.  **3. Psychological and compliance issues**  • Fear-avoidance leading tinsufficient activity levels, impairing recovery.  • Lack of consistency, arbitrarily interrupting training, reducing treatment efficacy.  **4. Coexisting conditions and safety risks**  • Patients with cardiovascular disease, osteoporosis, or balance disorders should exercise under medical supervision tprevent falls and accidents.  • Home exercise environments should be flat and slip-resistant tprevent falls. |
| **问题9** | | **腰痛居家康复期间需要注意哪些风险因素？** | | |
|  | | **我帮你把腰痛居家康复期间的风险因素整理成一个循证+实用的清单，分成医学风险和行为风险两大类，方便你在康复指导、科普宣教或论文中引用。**  **一、医学风险因素（身体状况相关）**   1. **红旗信号被忽视**    - **下肢肌力明显下降、大小便障碍、进行性麻木**    - **发热、体重减轻、不明原因夜间痛**    - **外伤后不能下地或疼痛剧烈**   **出现以上任何一种，应立即停止康复并就医。**   1. **急性期负荷过大**    - **急性损伤 1–2 周内做大幅度扭转、弯腰或负重动作**    - **会加重炎症和组织损伤，延长康复时间。** 2. **疼痛管理不足**    - **疼痛 >5/10 时继续训练**    - **易导致代偿性动作、肌肉痉挛，甚至形成慢性疼痛模式。** 3. **合并其他疾病**    - **心肺功能不良、骨质疏松严重、平衡障碍**    - **需调整运动强度，避免跌倒或骨折风险。**   **二、行为风险因素（康复习惯与生活方式相关）**   1. **姿势错误**    - **运动或日常活动时腰椎长时间过度前屈、扭转**    - **例如：弯腰搬物、久坐驼背、沙发瘫等。** 2. **缺乏循序渐进原则**    - **运动量突然增加或强度过大**    - **会引发延迟性肌肉酸痛（DOMS）或再次受伤。** 3. **只做被动治疗**    - **长期依赖按摩、牵引、热敷，而不进行主动训练**    - **容易导致肌肉萎缩、核心力量下降。** 4. **忽略日常环境改造**    - **桌椅高度不当、床垫过软或过硬、工作中无活动间隙**    - **这些因素会持续增加腰椎负担。** 5. **缺乏长期坚持**    - **疼痛缓解后停止训练，未进入维护期**    - **易导致功能下降和复发（复发率可达 20%–50%/年）。**   **三、降低风险的核心策略**   - **训练前 → 进行疼痛与功能评估** - **训练中 → 遵循“无痛或轻痛”原则，避免高负荷扭转、冲击动作** - **训练后 → 适当伸展与放松，关注延迟性疼痛** - **全程 → 保持正确姿势，定期复评，必要时调整康复计划** | **：腰痛居家康复时，最重要的是在恢复功能的同时避免二次损伤。结合国际康复指南（NICE 2020、WHO 2023、美国康复医学会）和临床经验，可以把风险因素分成动作类、环境类、健康状况类三大类：**  **一、动作相关风险因素**   1. **过早进行高强度或大幅度运动**    - **在疼痛未稳定时做负重深蹲、硬拉、快速扭转等，可能加重损伤。** 2. **动作姿势错误**    - **拉伸或核心训练时腰椎过度前屈/后伸，造成椎间盘或小关节压力增加。** 3. **突然加大运动量**    - **没有循序渐进，直接从低负荷跳到高强度，容易导致肌肉拉伤。** 4. **忽视疼痛信号**    - **出现持续或加重的疼痛、下肢麻木无力还继续训练，会延误治疗。**   **二、环境相关风险因素**   1. **地面湿滑或不平整**    - **居家训练时容易导致摔倒或闪腰。** 2. **缺乏合适康复工具**    - **没有瑜伽垫、腰部支撑等，导致姿势不稳或腰部悬空受力不均。** 3. **训练空间狭小**    - **容易碰撞家具、造成意外伤害。**   **三、健康状况相关风险因素**   1. **存在红旗征象却未就医**    - **如大小便障碍、进行性下肢无力、夜间痛明显，应立即停止居家康复并就医。** 2. **合并严重基础疾病**    - **心脏病、严重骨质疏松、近期骨折或手术未愈合，应由医生评估康复强度。** 3. **慢性炎症或急性发作期**    - **腰痛急性加重时强行训练，会延长恢复时间。** 4. **过度依赖护腰**    - **长期佩戴护腰而不进行核心训练，会削弱腰部稳定性。**   **四、安全管理建议**   - **康复前热身 5–10 分钟，结束后做放松拉伸。** - **训练强度遵循“无明显加重疼痛”的原则（疼痛 ≤ 5/10 分）。** - **保持康复日志，记录训练内容、疼痛变化。** - **出现新症状（如下肢麻木加重、发热、体重骤降）立即就医。** | **我帮你整理了腰痛居家康复期间的主要风险因素，结合了 NICE（2016）、APTA（2021）、EUROSPINE（2018）等国际指南，以及临床康复师的安全建议，方便你在执行康复时规避潜在问题。**  **1. 运动相关风险**   - **动作不规范**   - **错误的核心训练姿势（如平板支撑塌腰、小燕飞颈部过伸）会加重腰部压力。**   - **缺乏专业示范或复查，导致长期错误发力模式。** - **强度/进度过快**   - **急性期运动负荷过大易引发二次损伤。**   - **恢复期突然增加重量或次数可能刺激神经根或关节。** - **忽视疼痛信号**   - **勉强坚持导致疼痛持续加重或出现放射痛。**   - **“越疼越练”是错误观念，急性炎症期应以低强度活动为主。**   **2. 病情变化风险**   - **出现红旗症状却未就医**   - **大小便功能障碍**   - **进行性下肢麻木或无力**   - **不明原因的发热、体重下降** - **症状性质改变**   - **原来局限的疼痛变为广泛放射痛**   - **休息后反而疼痛加重或夜间痛明显**   **3. 环境与器材风险**   - **训练环境不安全**   - **地面湿滑或有障碍物，增加跌倒风险**   - **缺乏支撑物，导致平衡训练时摔倒** - **不当使用器械**   - **家用健身器械负荷设置过重**   - **弹力带、哑铃等使用姿势不当造成拉伤**   **4. 生活习惯风险**   - **康复训练外的腰部负荷**   - **长时间久坐、弯腰搬重物、错误睡姿**   - **工作中未进行姿势调整和休息** - **体重未控制**   - **超重增加腰椎承重负担，延缓康复** - **睡眠不足/压力大**   - **睡眠缺乏和精神紧张会增加疼痛敏感性**   **5. 心理与依从性风险**   - **恐动症（Fear-avoidance）**   - **过度害怕活动导致肌肉萎缩、功能下降** - **依从性差**   - **康复计划中断或执行不规律，效果不佳**   **安全提醒**  **居家康复的原则是 “不加重疼痛、不引发新伤”。**  **如果康复中出现剧烈疼痛、神经症状加重、红旗信号，应立即停止训练并就医。** |
| Correctness（正确性10分） | |  |  |  |
| Completeness（完整性10分） | |  |  |  |
| Readability（可读性10分） | |  |  |  |
| Helpfulness（实用性10分） | |  |  |  |
| Safety（安全性10分） | |  |  |  |
| Reading Difficuty Score | | 12.87 | 13.00 | 16.50 |
| Corresponding Education Level | | Junior high school | Junior high school | senior high school |
| Recommend Reading Age | | 13 | 13 | 17 |
| **Question10** | | **What are the quality control requirements for home rehabilitation of patients with low back pain?** | | |
|  | | **1. Assessment and Graded Management**  • Initial Assessment: A comprehensive assessment (etiology, functional status, psychological status) must be conducted prior trehabilitation trule out red flag signs.  • Indications: Only patients with stable conditions, nsevere complications, and the ability tself-manage should be included in home-based rehabilitation.  **2. Individualized Prescription**  • Individualized Plan: Training programs, frequency, intensity, and progression must be determined by a physician/rehabilitation therapist.  • Progressive Approach: Begin with low intensity and gradually increase based on pain and tolerance.  **3. Process Standardization**  • Standardized Movements: Ensure proper movement posture and avoid twisting the waist or using excessive force.  • Symptom Monitoring: Continuously monitor changes in symptoms such as pain, numbness, and weakness during training.  • Avoid Overloading: Adjust or stop training immediately if pain significantly worsens.  **4. Risk Control**  • Establish a risk warning mechanism: Seek medical attention immediately if red flag signs (worsening neurological symptoms, night pain, fever, etc.) appear.  • Home Environment Safety: Ensure floors are slip-resistant tprevent falls.  **5. Education and Compliance**  • Conduct regular health education tenhance patients' rehabilitation awareness and self-management capabilities.  • Improve compliance tavoid premature discontinuation or overtraining.  **6. Follow-up and Assessment**  • Establish a follow-up mechanism (in-person or remote) tregularly assess pain and functional improvements.  • Adjust the rehabilitation plan promptly based on assessment results. | **1. Pre-rehabilitation assessment**  • Before rehabilitation, a professional must assess the cause of the condition, functional impairments, and psychological state.  • Determine whether home-based rehabilitation is appropriate and exclude patients with “red flag signs.”  **2. Individualized Rehabilitation Prescription**  • Training programs, frequency, and intensity should be tailored tthe individual.  • Follow the principle of gradual progression tavoid overloading.  **3. Process Monitoring**  • Pay attention tproper movement and posture during training tavoid incorrect exertion.  • Regularly self-assess pain and function; if symptoms worsen or radiating neurological symptoms appear, pause training and contact a doctor.  **4. Risk Management**  • Emphasize a safe environment: non-slip surfaces, prevent falls.  • Establish an early warning system: seek medical attention promptly if neurological symptoms, night pain, fever, etc., occur.  **5. Compliance and Health Education**  • Educate patients on rehabilitation knowledge tenhance self-management capabilities.  • Monitor adherence tthe training program through rehabilitation follow-ups (outpatient or remote).  **6. Dynamic Assessment and Adjustment**  • Regularly assess pain, range of motion, and functional improvement.  • Adjust exercise plans and goals as appropriate based on recovery progress. | **1. Pre-rehabilitation assessment**  • Conduct a systematic assessment trule out “red flag signs” (fractures, tumors, severe nerve damage, etc.).  • Determine the type of low back pain, degree of functional impairment, and psychological state.  **2. Individualized prescription**  • Develop an individualized training plan based on the assessment (type of exercise, intensity, frequency, goals).  • Follow the principle of gradual progression and avoid blindly imitating generic movements.  **3. Process Standardization**  • Movement Standards: Ensure proper training posture and technique, avoiding twisting the waist or using excessive force.  • Symptom Monitoring: Monitor changes in symptoms such as pain, numbness, and weakness during rehabilitation.  **4. Risk Control**  • Establish an early warning mechanism:  If neurological function deteriorates, night pain occurs, or fever develops, immediately stop training and seek medical attention.  • Ensure a safe home environment tprevent secondary injuries such as slipping or falling.  **5. Education and Compliance**  • Provide patients with rehabilitation knowledge and lifestyle guidance tenhance self-management capabilities.  • Ensure compliance through follow-ups or remote guidance treduce dropouts or overtraining.  **6. Follow-up and Dynamic Adjustments**  • Conduct regular follow-ups tassess pain, range of motion, and functional recovery.  • Adjust training content and intensity based on feedback as needed. |
| **问题10** | | **腰痛患者居家康复中的质量控制提出了哪些要求？** | | |
|  | | **我帮你整理一个循证指南与康复实践结合的版本，把腰痛患者居家康复的质量控制要求，按国际指南（WHO 2023、NICE NG59 2020、NASS 2020）常用的四个核心维度拆解，这样既可用于论文，也可直接放到康复培训或患者管理手册中。**  **一、康复目标的质量控制**   1. **目标清晰、可量化**    - **明确短期目标（疼痛缓解、活动度增加）与长期目标（功能恢复、预防复发）。**    - **设定可测指标，如疼痛评分（VAS）、功能量表（ODI、Roland-Morris）。** 2. **阶段性目标**    - **急性期：疼痛 ≤3/10，基本生活自理。**    - **亚急性期：核心稳定性提升，耐力增加。**    - **慢性期/维护期：维持功能，减少复发。**   **二、康复过程的质量控制**   1. **个体化方案**    - **训练内容、强度、频率必须根据病情、体能、职业特点调整。** 2. **循序渐进原则**    - **强度从低到高，避免一次性大幅增加负荷。** 3. **正确动作执行**    - **保证核心收紧、中立位脊柱，避免代偿动作。** 4. **过程监测**    - **每次训练记录疼痛变化、疲劳程度、运动完成情况。** 5. **安全监控**    - **出现红旗信号（神经症状、剧痛、发热等）立即停训并就医。**   **三、康复效果的质量控制**   1. **定期评估**    - **建议 4–6 周进行一次全面复评，包括疼痛、功能、生活质量。** 2. **客观量化**    - **使用标准化量表（ODI、SF-36、PSFS 等）追踪改善程度。** 3. **疗效反馈机制**    - **根据评估结果调整训练计划，优化动作与强度。**   **四、患者自我管理质量控制**   1. **健康教育到位**    - **让患者理解康复意义、正确姿势和生活习惯的重要性。** 2. **依从性管理**    - **通过康复打卡、线上指导、家庭支持提高训练坚持率。** 3. **生活方式控制**    - **工作姿势、睡眠环境、体重管理等与康复同步优化。**   **质量控制的核心要求总结**  **明确目标 → 个体化计划 → 过程监控 → 定期复评 → 动态调整 → 长期维护** | **：腰痛患者在居家康复中的质量控制，核心是确保康复训练安全、有效、可持续，并且能够被患者长期坚持。结合国际康复指南（NICE 2020、WHO 2023、ISPRM 康复质量标准）和国内康复实践，主要有以下要求：**  **一、康复前质量控制**   1. **医学评估到位**    - **确认腰痛类型（非特异性/特异性）和阶段（急性、亚急性、慢性）。**    - **排除“红旗征象”（骨折、肿瘤、感染、马尾综合征等）。** 2. **制定个性化康复方案**    - **结合年龄、职业、体能、疼痛程度设定训练内容和强度。** 3. **患者教育**    - **明确训练目标、方法和注意事项，让患者理解康复的重要性和风险控制。**   **二、康复过程中的质量控制**   1. **动作规范**    - **按标准示范和分解动作进行训练，避免代偿动作（如用腰代替腿发力）。** 2. **强度控制**    - **采用“疼痛可控原则”：训练时疼痛评分不超过 5/10，并在24小时内恢复到训练前水平。** 3. **循序渐进**    - **每1–2周评估一次，根据耐受度逐步增加次数、时间或阻力。** 4. **过程记录**    - **建立康复日志，记录日期、训练内容、疼痛变化、身体反应，便于追踪和调整。**   **三、康复环境与设备要求**   1. **安全环境**    - **地面防滑、空间足够、无绊倒风险。** 2. **适当工具**    - **配备瑜伽垫、弹力带、护腰带、热敷袋等辅助器具。** 3. **防护与监测**    - **必要时使用心率监测、计时器，确保训练负荷合理。**   **四、康复评估与反馈**   1. **定期评估**    - **使用 ODI（Oswestry 功能障碍指数）、Roland-Morris 问卷、VAS 评分等工具，每月或每阶段评估一次。** 2. **及时调整**    - **出现疼痛加重、活动受限、下肢症状恶化，应立即减量或暂停训练并复查。** 3. **长期跟踪**    - **建立 3–6 个月的随访制度，预防复发并优化方案。**   **五、患者依从性与心理支持**   - **采用分阶段目标，让患者持续获得成就感，减少中途放弃。** - **必要时结合心理干预（CBT、放松训练）改善活动恐惧和疼痛焦虑。** | **我帮你整理了腰痛患者居家康复的质量控制要求，结合了 NICE（2016）、APTA（2021）、EUROSPINE（2018）等国际循证指南，以及国内康复医学会的建议，把它分成目标、过程、结果三大维度，方便直接用于方案设计或康复管理。**  **1. 质量控制的总体目标**   - **安全性：保证居家康复过程不引发新损伤或加重病情。** - **有效性：康复训练能够科学促进功能恢复、疼痛缓解和生活质量提升。** - **依从性：患者能够长期按计划执行并形成生活习惯。** - **可评估性：康复效果可通过量化指标客观追踪。**   **2. 过程质量控制要求**  **（1）康复计划的制定**   - **个性化：根据患者病因、病程、功能状态制定运动处方。** - **循序渐进：从低强度到中高强度，逐步增加难度与时间。** - **多元组合：包括核心稳定、有氧、柔韧性训练及生活方式指导。** - **风险评估：制定前排除红旗症状和不适合居家的情况。**   **（2）训练过程监控**   - **动作标准化：**   - **按康复师示范或视频指导执行，保持正确姿势。**   - **初期可通过家人或线上康复师进行监督。** - **疼痛监测：**   - **使用 VAS（视觉模拟评分）或 NRS 记录疼痛变化。**   - **原则：训练中疼痛不超过 5 分（0–10 分制），且结束后 24 小时内恢复到基线水平。** - **频率与强度控制：**   - **急性期：每天 1–2 次，低负荷**   - **恢复期：每周 ≥3 次，中等强度**   - **慢性期/维持期：每周 ≥150 分钟有氧+核心训练** - **环境安全：**   - **地面防滑、空间宽敞，有必要时配备支撑物。**   **（3）生活习惯配合**   - **保持健康坐立走姿** - **避免搬重物或突然扭转** - **保证充足睡眠与营养摄入**   **3. 结果质量控制要求**   - **短期目标（2–4 周）：**   - **疼痛评分下降 ≥30%**   - **腰部活动度提升 ≥10%（屈伸、侧弯、旋转）** - **中期目标（1–3 个月）：**   - **核心肌群力量提升（桥式、平板支撑时间延长 ≥20%）**   - **日常生活活动（ADL）能力恢复至接近发病前水平** - **长期目标（≥6 个月）：**   - **保持无或轻微疼痛（VAS ≤ 2）**   - **无功能受限，正常参与工作和运动**   - **复发率显著下降**   **4. 数据与反馈机制**   - **建立康复日记：记录训练内容、疼痛评分、功能改善情况。** - **每 2–4 周进行一次阶段评估：可通过线上视频或线下复查进行动作和功能复评。** - **根据评估结果及时调整康复处方，避免长期停留在低效或高风险阶段。**   **✅ 总结**  **腰痛居家康复的质量控制要实现 “安全执行—科学监控—动态调整—量化评估” 四个闭环，才能确保康复不仅做了，而且做得对、做得好、坚持得住。** |
| Correctness（正确性10分） | |  |  |  |
| Completeness（完整性10分） | |  |  |  |
| Readability（可读性10分） | |  |  |  |
| Helpfulness（实用性10分） | |  |  |  |
| Safety（安全性10分） | |  |  |  |
| Reading Difficuty Score | | 12.72 | 14.21 | 13.83 |
| Corresponding Education Level | | Junior high school | Junior high school | Junior high school |
| Recommend Reading Age | | 13 | 14 | 14 |
| **Question11** | | **What are the treatment strategies for acute low back pain?** | | |
|  | | The treatment strategy for acute low back pain (generally defined as symptoms lasting less than 6 weeks) is clearly outlined in international evidence-based guidelines (NICE 2020, ACP 2017, WH2023), with the core objectives being rapid symptom control, prevention of chronicity, and maintenance of functional activity.  **1. Assessment and exclusion**  • First, rule out red flag signs (severe trauma, infection, tumors, progressive neurological dysfunction, etc.).  • Most cases of acute low back pain are nonspecific and dnot require routine imaging studies.  **2. Core treatment principles**  “Early activity + education + short-term support”  Bed rest is not encouraged; maintaining daily activities is emphasized.  **3. Non-pharmacological interventions (preferred)**  **1. Health education**  Explain the condition, noting that most acute low back pain improves spontaneously within several weeks.  Reduce fear and encourage maintaining daily activities.  **2. Moderate exercise**  Recommend maintaining walking, light stretching, and core muscle activities within tolerable limits.  Avoid prolonged bed rest (typically nmore than 1–2 days).  **3. Physical therapy (optional)**  Heat therapy or short-term manual therapy (e.g., massage, spinal manipulation) may help alleviate symptoms.  Ultrasound, traction, and passive physical therapy have insufficient evidence of efficacy and are not recommended for routine use.  **4. Pharmacological Intervention (Adjunctive)**  • NSAIDs (nonsteroidal anti-inflammatory drugs) are the first-line treatment for short-term use, combined with gastrointestinal protective measures.  • Muscle relaxants may be used short-term for severe muscle spasms.  • Opioids are not recommended for routine use and should only be used short-term when other treatments are ineffective and pain is severe.  • Regular use of corticosteroids or acetaminophen monotherapy is not recommended.  **5. Psychological and Follow-Up**  • Early psychological intervention or education is recommended for patients with anxiety or fear-avoidance behavior.  • Patients whremain unresolved or have significant functional limitations after 2–4 weeks require further evaluation. | For acute low back pain (typically referring tnonspecific low back pain lasting less than 6 weeks), international authoritative guidelines (such as NICE 2020, ACP 2017, and WH2023) propose the following evidence-based treatment strategies:  **I. General Treatment Principles**  Non-pharmacological treatment is the primary approach, emphasizing early activity and functional recovery tprevent chronicity.  **II. Specific Treatment Strategies**  **1. Exclude severe causes**  • First, conduct an assessment trule out “red flag signs”:  such as fractures, infections, tumors, cauda equina syndrome, and severe neurological dysfunction.  **2. Non-pharmacological treatment (preferred approach)**  (1) Health education  • Explain tpatients that low back pain is often a self-limiting condition with a good prognosis;  • Alleviate unnecessary concerns and avoid “fear-avoidance” behavior;  • Encourage early resumption of daily activities and avoid prolonged bed rest (nmore than 1–2 days).  (2) Moderate Exercise  • Recommend walking, gentle stretching, and core stability exercises (based on tolerance);  • Avoid vigorous exercise or heavy physical labor.  (3) Physical Therapy (Optional)  • Heat therapy and manual therapy (e.g., spinal manipulation) may provide short-term symptom relief in some patients;  • Passive physical therapy modalities such as ultrasound, traction, and laser therapy are not recommended for routine use.  **3. Pharmacological treatment (as an adjunct)**  (1) NSAIDs (nonsteroidal anti-inflammatory drugs)  • Short-term use is preferred, such as ibuprofen or celecoxib.  • Combine with gastric mucosal protectants treduce gastrointestinal risks.  (2) Muscle relaxants  • May be used for patients with severe muscle spasms, but should not be used for prolonged periods.  (3) Opioids  • Not recommended for routine use; only used short-term for severe pain when other medications are ineffective.  (4) Not recommended:  • Acetaminophen (limited efficacy as monotherapy)  • Corticosteroids, antidepressants, anticonvulsants (ineffective during the acute phase)  **4. Psychological support and follow-up**  • Cognitive behavioral therapy may be combined for those with psychological disorders such as anxiety or fear;  • If symptoms persist for more than 4–6 weeks without improvement, reassessment is necessary tprevent chronicity. | International authoritative evidence-based guidelines (NICE 2020, ACP 2017, WH2023) provide highly consistent recommendations for the management of acute low back pain (symptoms lasting <6 weeks), with the core objective being rapid functional recovery and prevention of chronicity. The main points are as follows:  **1. Assessment and exclusion**  • First assess for red flag signs (e.g., fractures, tumors, infections, cauda equina syndrome, progressive nerve damage).  • Most acute low back pain is nonspecific, and routine imaging studies are not recommended.  **2. Core treatment principles**  “Early activity + patient education + short-term symptomatic management”  **3. Non-pharmacological treatment (first-line)**  **1. Health education**  Inform patients that acute low back pain is often a benign, self-limiting condition.  Alleviate fear and encourage patients tresume daily activities as soon as possible.  **2. Moderate activity and exercise**  Maintain walking, gentle stretching, and core stability training.  Avoid prolonged bed rest (maximum 1–2 days).  **3. Physical therapy (optional)**  Heat application or short-term spinal manipulation may alleviate pain.  Passive therapies such as traction, ultrasound, and electrotherapy are not recommended for routine use.  **4. Pharmacological intervention (as an adjunct only)**  • First-line: Short-term NSAIDs (e.g., ibuprofen, celecoxib), with concomitant use of gastric mucosal protectants if necessary.  • Optional: Short-term muscle relaxants, only for those with significant muscle spasms.  • Not recommended for routine use: Opioids (only for severe and short-term cases), corticosteroids, acetaminophen monotherapy, antidepressants, antiepileptic drugs.  **5. Psychological support and follow-up**  • For patients with anxiety or fear-avoidance behavior, early cognitive behavioral intervention may be considered.  • If symptoms persist for 4–6 weeks without improvement, reassessment is recommended tevaluate the risk of chronicity. |
| **问题11** | | **对急性腰痛的治疗策略是什么？** | | |
|  | | **我给你整理一个基于最新国际循证指南（WHO 2023、NICE NG59 2020、ACP 2017、NASS 2020）的急性腰痛治疗策略，分为核心原则—非药物治疗—药物治疗—避免措施四个部分，这样你可以直接用于临床培训、康复指导或论文写作。**  **一、核心治疗原则**   1. **以非药物治疗为主，药物为辅**    - **大多数急性腰痛是非特异性的，90%以上 4–6 周内可自行好转。** 2. **鼓励活动，避免长期卧床**    - **适度活动有助于防止肌肉萎缩和功能退化。** 3. **早期教育**    - **告诉患者急性腰痛通常是可逆的、良性的，减少焦虑与“怕动”心理。** 4. **个体化干预**    - **根据疼痛程度、职业需求、生活习惯制定方案。**   **二、非药物治疗（循证推荐）**  **国际指南一致建议：急性腰痛首选非药物干预**   \| **干预方式** \| **循证等级** \| **使用建议** \| \| --- \| --- \| --- \| \| **保持日常活动** \| **高** \| **鼓励继续日常生活和工作（在可耐受范围内），避免绝对卧床** \| \| **患者教育** \| **高** \| **解释病情、自限性过程、康复要点** \| \| **热敷** \| **中** \| **缓解肌肉痉挛与疼痛，可在家使用热水袋或热贴** \| \| **手法治疗（推拿/脊柱调整）** \| **中** \| **对部分患者有帮助，需专业人员实施** \| \| **早期功能训练** \| **中** \| **在疼痛可耐受的前提下进行轻柔核心激活和拉伸** \|   **三、药物治疗（短期辅助）**  **药物只在疼痛影响日常活动时使用，且应短期、低剂量**   \| **药物类别** \| **循证等级** \| **建议** \| \| --- \| --- \| --- \| \| **NSAIDs（非甾体抗炎药）** \| **高** \| **一线首选，如布洛芬、萘普生；注意胃肠道、肾脏风险** \| \| **对乙酰氨基酚** \| **中** \| **有争议，可用于不能用NSAIDs者** \| \| **肌肉松弛剂** \| **中** \| **适用于伴有明显肌肉痉挛者，疗程不超过 1–2 周** \| \| **局部用药** \| **中** \| **外用NSAIDs、辣椒素膏等作为辅助** \|   **四、避免或不推荐的措施**   - **长期卧床休息：会延缓恢复并增加慢性化风险。** - **过早影像学检查：无“红旗”症状时，不建议急性期常规拍片/MRI。** - **阿片类药物：除非疼痛极重且其他治疗无效，否则不推荐。** - **常规牵引：证据不足，不推荐作为常规治疗。**   **五、典型治疗路径（临床示例）**   1. **第 1–3 天**    - **活动调整 + 热敷 + NSAIDs 短期使用**    - **避免重体力劳动** 2. **第 3–7 天**    - **增加轻度活动（步行、伸展）**    - **教授正确姿势和搬运技巧** 3. **1–4 周**    - **开始核心稳定训练**    - **渐进恢复工作和运动** 4. **4 周后未好转或出现红旗症状**    - **进一步检查与专科评估** | **：我帮你按国际权威循证指南（NICE 2020、ACP 2017、WHO 2023）和临床常规，把急性腰痛（症状 <6 周）的一线到辅助治疗策略整理成分阶段、循序渐进的方案，方便直接用于康复方案、科普或学术材料。**  **一、治疗总原则**   - **绝大多数急性腰痛为非特异性，多在 2–6 周内自然缓解。** - **核心策略是缓解疼痛 + 保持活动 + 预防慢性化。** - **药物只是辅助，避免不必要的影像学检查和长期卧床。**   **二、治疗策略分层**  **① 患者教育与自我管理（所有患者必做）**   - **告知疾病通常为良性、自限性。** - **鼓励在可耐受范围内保持日常活动，避免卧床 >1–2 天。** - **教授正确姿势、搬重物技巧、工作与生活的人体工学调整。**   **② 疼痛管理**   1. **非药物为主**    - **热敷：缓解肌肉紧张和疼痛（NICE、WHO 推荐）。**    - **手法治疗（脊椎松动、软组织松解）：短期可改善症状，需专业人员操作。**    - **针灸、按摩：部分患者有效，可作为辅助。** 2. **药物（短期、最低有效剂量）**    - **首选 NSAIDs（如布洛芬、萘普生）—短期使用，注意胃肠道和肾脏风险。**    - **对乙酰氨基酚单药疗效有限，不推荐作为唯一选择。**    - **肌肉松弛剂：仅用于伴明显肌痉挛且 NSAIDs 效果不佳者，限 ≤2 周。**    - **阿片类：仅在其他方法无效且疼痛严重影响功能时短期使用（≤3–5天）。**   **③ 功能活动与康复训练**   - **急性期可做低强度活动：步行、温和伸展（如猫牛式、单膝抱胸）。** - **避免剧烈弯腰、扭转、负重。** - **症状缓解后 1–2 周可逐渐加入核心肌群稳定训练（桥式、小燕飞）。**   **④ 监测与复评**   - **症状一般在 2–4 周内明显改善。** - **若出现红旗征象（大小便障碍、进行性下肢无力、发热、夜间痛等）立即复查。** - **4–6 周仍无改善或反复发作 → 考虑影像学检查及进一步诊疗。**   **⑤ 预防慢性化**   - **鼓励早期重返工作（可调整负荷）。** - **教授生活方式管理（体重、姿势、规律运动）。** - **对存在活动恐惧或焦虑的患者，早期引入心理干预（CBT）。** | **我帮你整理了腰痛患者居家康复的质量控制要求，参考了 NICE（2016）、APTA（2021）、EUROSPINE（2018）等国际循证指南，并结合临床康复管理的质量控制思路，把它分成 过程控制—结果评估—安全保障 三大类，方便直接应用到康复方案或患者管理手册中。**  **一、过程控制（Process Control）**  **确保康复全过程科学、有序、可追踪**   1. **康复方案个性化**    - **由医生或康复师制定，明确目标（疼痛缓解、功能恢复、预防复发）**    - **根据病情阶段（急性期/恢复期/维持期）动态调整** 2. **动作标准化**    - **使用视频、图片或面对面指导确保动作正确**    - **建议初次训练由康复师监督，后续定期复查** 3. **训练频率与负荷控制**    - **按“循序渐进”原则，每次运动强度不超过最大耐受的 70%**    - **每日/每周训练次数、时间应记录在案** 4. **生活方式配合**    - **结合坐、站、搬运姿势训练**    - **定期提醒调整工作环境和生活习惯**   **二、结果评估（Outcome Evaluation）**  **通过量化指标来判断康复效果**   1. **疼痛评估**    - **采用 VAS（0–10分）、NRS 或 McGill 疼痛问卷，每周一次** 2. **功能评估**    - **Oswestry功能障碍指数（ODI）、Roland-Morris功能问卷（RMDQ）**    - **日常活动能力（穿衣、上下楼、久坐耐受度）** 3. **体能与柔韧性测量**    - **核心力量测试（如平板支撑时间）**    - **腰椎活动度（前屈、后伸、侧弯范围）** 4. **依从性与满意度**    - **记录完成训练的比例**    - **定期收集患者的主观康复感受**   **三、安全保障（Safety Assurance）**  **确保康复过程无严重并发症、无可避免的二次损伤**   1. **红旗信号监测**    - **出现大小便功能障碍、进行性麻木、持续夜间痛等立即停训就医** 2. **疼痛加重控制**    - **若训练后疼痛持续 >48 小时或评分增加 ≥2 分，应降低负荷** 3. **训练环境安全**    - **防滑地面、无绊倒物、必要时有支撑物** 4. **并发症防控**    - **避免急性肌肉拉伤、关节扭伤等二次损伤**   **质量控制核心目标**   - **有效性：康复后疼痛明显下降、功能改善** - **安全性：康复期间无严重不良事件** - **持续性：康复方案可长期执行，依从性高** |
| Correctness（正确性10分） | |  |  |  |
| Completeness（完整性10分） | |  |  |  |
| Readability（可读性10分） | |  |  |  |
| Helpfulness（实用性10分） | |  |  |  |
| Safety（安全性10分） | |  |  |  |
| Reading Difficuty Score | | 17.02 | 12.51 | 23.57 |
| Corresponding Education Level | | senior high school | Junior high school | senior high school |
| Recommend Reading Age | | 17 | 13 | 18 |
| **Question12** | | **What are the recommended treatment goals for patients with subacute and chronic low back pain?** | | |
|  | | **1. Restore and improve function**  • Restore daily living, work, and exercise abilities, and reduce functional limitations.  • Improve core stability, flexibility, and endurance, and enhance the load-bearing capacity of the spine and trunk.  **2. Control symptoms and reduce the impact of pain**  • Alleviate pain and reduce pain-related limitations in daily life through multimodal interventions (exercise, psychological, and pharmacological).  • Address fear of pain and build confidence in engaging in activities.  **3. Prevent chronicity and recurrence**  • Reduce the recurrence rate of low back pain through regular exercise, posture correction, and lifestyle interventions.  • Break the vicious cycle of “pain—fear of movement—functional decline—worsening pain.”  **4. Improve psychosocial functioning**  • Address accompanying anxiety, depression, and sleep disorders trestore social participation.  • Reduce fear-avoidance behaviors caused by chronic pain.  **5. Enhance patients' self-management capabilities**  • Teach patients self-regulation, scientific exercise, and self-monitoring treduce long-term dependence on medication and medical resources. | **1. Restore and improve function**  • Promote patients' return tdaily life, work, and exercise capabilities.  • Enhance spinal and trunk control through core stability, flexibility, and endurance training.  **2. Control symptoms and reduce the impact of pain**  • Alleviate functional limitations caused by pain and improve activity tolerance.  • Focus on reducing the interference of pain in daily life rather than pursuing complete pain relief.  **3. Preventing Chronicity and Recurrence**  • Break the vicious cycle of “pain—avoidance of activity—muscle weakness—recurrence of pain.”  • Reduce recurrence through regular exercise and lifestyle adjustments.  **4. Improving Psychosocial Status**  • Alleviate anxiety and depression associated with chronic pain.  • Enhance social participation and quality of life.  **5. Enhance self-management capabilities**  • Develop patients' ability tself-regulate and engage in sustained exercise, reducing long-term reliance on medications and medical resources. | **1. Restore and enhance function**  • Restore daily living, work, and exercise abilities, and improve spinal and core muscle stability, flexibility, and endurance.  **2. Control symptoms and reduce the impact of pain**  • Reduce the limitations of pain on activity, sleep, and daily life through multimodal intervention, and improve activity tolerance rather than achieving complete pain relief.  **3. Prevent chronicity and recurrence**  • Break the cycle of “pain-fear avoidance-functional decline-recurring pain” treduce prolonged illness and recurrence.  **4. Improve psychological and social participation**  • Alleviate anxiety and depression caused by chronic pain and restore social, work, and family participation.  **5. Enhance self-management capabilities**  • Help patients learn long-term self-exercise, posture adjustment, and healthy lifestyles treduce dependence on medical care and medication. |
| **问题12** | | **对于亚急性和慢性腰痛患者的推荐治疗目标是什么？** | | |
|  | | **下面给出亚急性（6–12周）与慢性（≥12周）腰痛的推荐治疗目标，按“可量化（SMART）”原则列出，便于随访与质量控制（常用量表：NRS/VAS、ODI、Roland-Morris、PSFS、FABQ、SF-12/36）。**  **亚急性腰痛（6–12周）目标**   1. **疼痛控制**  - **NRS/VAS 较基线下降≥30%或≥2分；静息痛≤3/10，活动痛≤4/10。** - **夜间痛明显减少，睡眠中断≤1次/夜。**  1. **功能恢复**  - **ODI 改善≥10分（或相对改善≥30%）；RMDQ 改善≥5分。** - **PSFS（患者自定功能）每项提高≥2分。**  1. **活动耐力与动作质量**  - **日行走/站立累计≥30–45分钟/日、坐位耐受≥45–60分钟且无症状反跳。** - **核心与髋稳定：桥式/前板支撑各30–45秒可保持中立位、无代偿。** - **关键动作（弯腰拾物、屈膝提物）技术正确率≥90%（居家打卡/视频随访评估）。**  1. **重返工作与生活**  - **带保护与分级负荷的部分或全部复工；日常家务基本不受限。**  1. **药物与被动治疗最小化**  - **NSAIDs 等短期按需；停用或显著减少肌松药/镇静药；不依赖被动理疗。**  1. **风险管理与教育**  - **能识别红旗与运动加重阈值；建立每周≥3次、20–30分钟的家庭运动处方。**   **慢性腰痛（≥12周）目标**   1. **疼痛与复发控制（以“可持续”为核心）**  - **NRS/VAS 较基线下降≥30–50%并连续≥3个月稳定；急性发作**≤1次/6个月**，且强度≤中度、48小时内可自我控制。**  1. **功能与参与度**  - **ODI 改善≥10–12分或相对≥30%；RMDQ 持续改善并趋稳。** - **关键生活/工作任务在PSFS上每项≥7/10；SF-12/36 体能维度提升。** - **连续坐/站/走60–90分钟不出现显著症状反跳；可完成个体化工作全部或≥80%岗位需求。**  1. **体能与运动处方（长期维护）**  - **每周≥150分钟中等强度有氧 + 2–3次力量/核心训练 + 2–3次柔韧与神经肌控。** - **动作质量：**   - **前板支撑/侧桥各60秒无代偿；**   - **髋铰链/深蹲/提物技术稳定，日常场景可自动化应用。**  1. **心理社会与自我管理**  - **FABQ/PCS（恐惧回避/灾难化）下降，恢复无畏运动；必要时完成CBT等干预。** - **形成复发应对方案：加重阈值、减量周、热/冷敷、主动放松与睡眠策略。**  1. **药物与医疗资源优化**  - **避免或停用阿片类/长期肌松药；NSAIDs 间歇低剂量、明确停用点。** - **无红旗且病情稳定时不做常规影像学复查。**  1. **质量控制与随访**  - **每4–6周复评疼痛/功能/心理指标并动态调强度；** - **达标后进入维护期：每周≥2次维护训练，季度复评一次。**   **进阶与调整触发点（两类人群通用）**   - **上调强度：2周内疼痛稳定≤3/10，运动后24小时内无症状反跳。** - **下调/转诊：出现红旗；NRS上升≥2分且持续>1周；ODI/RMDQ无改善≥6–8周；出现进行性神经体征或功能倒退。** | **下面把**亚急性（6–12周）与慢性（≥12周）**腰痛的推荐治疗目标，按指南共识做成“目标—内涵—衡量指标”的方式，便于落地执行。**  **总体目标（两类人群共同适用）**   1. **以功能和参与为核心：围绕日常活动、工作与社会参与设定目标，采用以患者为中心、身心社会整合的管理路径（运动+教育+心理干预为主）。(**[**世界卫生组织**](https://www.who.int/news/item/07-12-2023-who-releases-guidelines-on-chronic-low-back-pain?utm_source=chatgpt.com)**,** [**acponline.org**](https://www.acponline.org/acp-newsroom/american-college-of-physicians-issues-guideline-for-treating-nonradicular-low-back-pain?utm_source=chatgpt.com)**)** 2. **优先非药物干预、减少低价值医疗：尽量用运动/自我管理/心理干预等非药物手段，避免不必要影像学与长期阿片类依赖；仅当结果会改变处理时再考虑影像。(**[**acponline.org**](https://www.acponline.org/acp-newsroom/american-college-of-physicians-issues-guideline-for-treating-nonradicular-low-back-pain?utm_source=chatgpt.com)**,** [**NICE**](https://www.nice.org.uk/guidance/ng59/chapter/recommendations?utm_source=chatgpt.com)**)**   **亚急性腰痛（6–12周）目标**   - **阻断慢性化：在4–8周内把“疼痛-回避-失能”循环扭转为“活动-适应-恢复”。以结构化运动与自我管理教育为主，必要时短期药物辅助。(**[**acponline.org**](https://www.acponline.org/acp-newsroom/american-college-of-physicians-issues-guideline-for-treating-nonradicular-low-back-pain?utm_source=chatgpt.com)**)** - **恢复关键功能：逐步恢复坐/站/行与弯腰搬举等功能任务，促成尽早、分阶段重返工作/学习。(**[**NICE**](https://www.nice.org.uk/guidance/ng59/chapter/recommendations?utm_source=chatgpt.com)**)** - **建立自我管理框架：教会识别诱因与加重因素，配置家中工效学与“加重—缓解”策略卡。(**[**NICE**](https://www.nice.org.uk/guidance/ng59/chapter/recommendations?utm_source=chatgpt.com)**)**   **可量化指标（示例）**   - **功能量表达成MCID：Roland-Morris（RMDQ）较基线改善≥30%；或Oswestry（ODI）下降≥12分（约10–13分）。(**[**PubMed**](https://pubmed.ncbi.nlm.nih.gov/16360560/?utm_source=chatgpt.com)**,** [**Shirley Ryan AbilityLab**](https://www.sralab.org/rehabilitation-measures/oswestry-disability-index?utm_source=chatgpt.com)**)** - **活动耐受度：无显著加重疼痛前提下，连续步行时间/距离或久坐耐受↑（以个人基线为参照）。**   **慢性腰痛（≥12周）目标**   - **降低疼痛负担、提升生活质量：通过“运动处方+心理干预（如CBT/正念）+教育”多模式方案，减少疼痛干扰度与波动幅度。(**[**acponline.org**](https://www.acponline.org/acp-newsroom/american-college-of-physicians-issues-guideline-for-treating-nonradicular-low-back-pain?utm_source=chatgpt.com)**,** [**世界卫生组织**](https://www.who.int/news/item/07-12-2023-who-releases-guidelines-on-chronic-low-back-pain?utm_source=chatgpt.com)**)** - **持续功能提升与复发管理：巩固核心稳定与全身耐力，制定“加重期自我调节计划”（负荷下调、睡眠与热疗、短期药物按医嘱等）。(**[**世界卫生组织**](https://www.who.int/news/item/07-12-2023-who-releases-guidelines-on-chronic-low-back-pain?utm_source=chatgpt.com)**)** - **减少医疗依赖：用可视化目标与自评量表驱动自我管理，避免长期被动治疗与不必要检查/侵袭操作。(**[**NICE**](https://www.nice.org.uk/guidance/ng59/chapter/recommendations?utm_source=chatgpt.com)**)**   **可量化指标（示例）**   - **功能与残疾：ODI 下降≥10–13分 或 RMDQ 较基线下降≥30%（达临床重要改善）；定期（每4–8周）复测并记录趋势。(**[**Shirley Ryan AbilityLab**](https://www.sralab.org/rehabilitation-measures/oswestry-disability-index?utm_source=chatgpt.com)**,** [**PubMed**](https://pubmed.ncbi.nlm.nih.gov/16360560/?utm_source=chatgpt.com)**)** - **参与与目标任务：达成个性化“关键活动KPI”（如：无加重地完成≥30分钟家务/班前步行、全班次工作、每周≥150分钟中等强度活动等）。(**[**acponline.org**](https://www.acponline.org/acp-newsroom/american-college-of-physicians-issues-guideline-for-treating-nonradicular-low-back-pain?utm_source=chatgpt.com)**)**   **过程质量与随访**   - **SMART 目标：具体（Specific）、可测量（Measurable）、可达成（Achievable）、相关（Relevant）、有时限（Time-bound），并以功能为主、疼痛为辅。(**[**世界卫生组织**](https://www.who.int/news/item/07-12-2023-who-releases-guidelines-on-chronic-low-back-pain?utm_source=chatgpt.com)**)** - **随访与调整：每4–8周用 ODI/RMDQ + 简易疼痛/睡眠评分复盘；若功能/疼痛无改善或出现红旗征象，及时升级评估与转诊。(**[**NICE**](https://www.nice.org.uk/guidance/ng59/chapter/recommendations?utm_source=chatgpt.com)**)** | **：下面给你一份**可量化（SMART）的目标清单，分别适用于亚急性（6–12周）与慢性（＞12周）**腰痛患者。你可以直接据此设定计划与随访节点。**  **亚急性与慢性腰痛的推荐治疗目标（SMART）**   \| **目标维度** \| **量化指标** \| **亚急性阶段目标（6–12周内）** \| **慢性阶段目标（滚动8–12周周期）** \| \| --- \| --- \| --- \| --- \| \| **疼痛控制** \| **VAS/NRS（0–10）** \| **下降≥2分；多数日平均≤3–4/10；发作频次↓≥50%** \| **维持多数日≤3/10；急性加重天数≤3天/月** \| \| **功能残疾** \| **ODI（0–100）或 RMDQ** \| **ODI↓≥10分 *或* RMDQ↓≥4–5分（达到MCID）** \| **连续一个周期保持ODI≤20 *或* RMDQ≤6；功能稳定无倒退** \| \| **患者自定功能** \| **PSFS（0–10，挑3项活动）** \| **每项≥+2分（如“久坐/弯腰/上楼”）** \| **目标活动≥8/10，可在无明显加重下完成** \| \| **日常活动量** \| **步数/有氧时长** \| **从基线增加**+2000–3000步/日** *或* 每周**≥90–120分钟**中等强度有氧** \| **每周**≥150分钟**中等强度（可分3–5次）** \| \| **核心耐力** \| **平板/侧桥/背伸** \| **平板45–60秒；侧桥30–45秒/侧；Sorensen背伸60–90秒** \| **McGill耐力比值接近1:1:1；可加入负重抗阻训练** \| \| **活动度与柔韧** \| **腰髋联合活动** \| **达到无明显疼痛完成穿袜、系鞋、取物等ADL** \| **ROM对称、功能性深蹲/髋铰链动作规范无代偿** \| \| **睡眠与心理** \| **睡眠干扰评分/恐动** \| **夜间痛导致醒来≤1次/夜；恐动行为明显下降** \| **维持良好睡眠；必要时CBT等心理策略巩固** \| \| **自我管理与依从** \| **训练打卡率** \| **家庭训练**≥80%**按处方完成，能识别“正常酸胀 vs 异常疼痛”** \| **长期坚持，能自我加减量并记录** \| \| **药物使用** \| **镇痛依赖** \| **逐步降至按需使用（≤2–3天/周）** \| **尽量停用或最低剂量，以非药物策略为主** \| \| **复发预防/重返工作** \| **复发与出勤** \| **4–8周内不出现失能性发作；可带方案返岗/改良工位** \| **维持全职责；3个月内无严重复发，掌握搬运与工位微休息** \|   **过程里程碑（用于随访时判定是否“进阶”）**   - **2–4周：疼痛下降趋势明确（≥1–2分），动作规范；否则检查负荷与姿势。** - **6–8周：达到ODI/RMDQ的MCID；进入力量与耐力进阶期。** - **8–12周：能完成目标有氧量与核心耐力时间；开始运动多样化与情境化训练（提物、久坐间歇等）。**   **何时“降阶/就医”**   - **训练后疼痛增加≥2分并持续＞48小时。** - **出现红旗：大小便功能障碍、进行性下肢无力/麻木、发热或夜间痛进行性加重。** - **影像或临床提示结构性进展而功能倒退。**   **小提示：把上表做成你的随访打勾清单（每2–4周评一次），既能证明疗效，也能及时微调处方。如果需要，我可以把它整理成可打印的一页表格给你用。** |
| Correctness（正确性10分） | |  |  |  |
| Completeness（完整性10分） | |  |  |  |
| Readability（可读性10分） | |  |  |  |
| Helpfulness（实用性10分） | |  |  |  |
| Safety（安全性10分） | |  |  |  |
| Reading Difficuty Score | | 13.06 | 14.2 | 19.52 |
| Corresponding Education Level | | Junior high school | Junior high school | senior high school |
| Recommend Reading Age | | 13 | 14 | 18 |
| **Question13** | | **What is the significance of patient education in the treatment of low back pain?** | | |
|  | | **1. Correct misconceptions and reduce fear and anxiety**  • Inform patients that the vast majority of lower back pain is benign and reversible, and does not necessarily indicate severe structural damage.  • Reduce “fear-avoidance” behavior caused by excessive worry, and avoid exacerbating functional decline due treluctance tmove.  **2. Encourage activity and self-management**  • Help patients understand that “movement is better than rest” and avoid prolonged bed rest.  • Encourage patients tparticipate in rehabilitation exercises and maintain daily activities and work.  **3. Improve treatment compliance**  • Through education, patients are more likely tcooperate with physical therapy, psychological intervention, and lifestyle adjustments, thereby enhancing treatment outcomes.  **4. Prevent chronicity and recurrence**  • Teach patients tidentify poor posture and risky movements, and develop proper lifestyle and work habits treduce recurrence rates.  **5. Reduce healthcare resource consumption**  Minimize unnecessary imaging tests and excessive medication/surgical interventions caused by fear or misunderstanding. | **1. Correcting misconceptions**  • Help patients understand that most lower back pain is non-specific and reversible, reducing fear of imaging abnormalities and pain.  **2. Reducing fear and anxiety**  • Alleviate “fear-avoidance” behavior caused by pain, avoiding muscle disuse and functional decline due treluctance tmove.  **3. Encourage active movement**  • Emphasize that “movement is better than rest,” avoid prolonged bed rest, and promote early return tdaily activities and work.  **4. Improve compliance and self-management skills**  • Encourage patients tactively participate in rehabilitation exercises, lifestyle adjustments, and psychological interventions tenhance their sense of control over the condition.  **5. Prevent chronicity and recurrence**  • Reduce the risk of recurrent episodes through proper posture, appropriate force application, regular exercise, and improvements in lifestyle habits.  **6. Reduce unnecessary waste of medical resources**  • Minimize excessive imaging tests and medication use caused by anxiety or misunderstandings. | **1. Correct misconceptions and reduce fear**  • Inform patients that most lower back pain is non-specific and reversible, and avoid excessive anxiety and “fear-avoidance” behavior caused by misunderstanding of pain or imaging test results.  **2. Encourage activity and self-management**  • Emphasize that “movement is better than rest,” avoid prolonged bed rest, and guide patients tresume daily activities, work, and appropriate exercise.  • Cultivate patients' awareness of self-recovery, teaching them tmanage lower back pain through posture adjustment, core training, and improvements in lifestyle habits.  **3. Improve compliance**  • Help patients understand the scientific basis of rehabilitation tenhance their proactive participation in physical therapy, psychological intervention, and lifestyle adjustments.  **4. Prevent chronicity and recurrence**  • Reduce the incidence of poor posture and risky movements through education, and help establish long-term healthy lifestyles tlower recurrence rates.  **5. Reduce unnecessary medical interventions**  • Minimize waste of medical resources caused by excessive testing, medication, or surgical interventions. |
| **问题13** | | **患者教育在腰痛治疗中的意义是什么？** | | |
|  | | **下面按临床视角，说明患者教育在腰痛治疗中的意义与落地做法（适用于门诊、康复和随访管理）。**  **1) 定位：患者教育=治疗本身**   - **腰痛多为良性、可逆，教育能直接降低疼痛与功能障碍，并减少慢性化。** - **与运动/康复合用时，提高依从性与长期坚持，效果更稳、更持久。** - **减少**不必要影像学检查、低价值干预与药物（尤其阿片）**的使用，降低成本与风险。**   **2) 作用机制（为什么有效）**   - **纠正错误认知：减少“结构性恐惧”“一动就坏”的观念→降低恐惧回避（FABQ）、灾难化（PCS）。** - **提升自我效能：教会“如何安全活动与自我调节负荷”，促成早期活动与自我管理。** - **生物-心理-社会整合：把疼痛从“只靠影像/药物”转向“多维度可控”，缓解焦虑、抑郁对疼痛的放大。** - **预防复发：通过姿势、动作策略与维护性训练，建立长期保护机制。**   **3) 临床价值（你能立刻看到的变化）**   - **更快恢复日常/工作功能、更少复诊与失能天数。** - **更高治疗依从性：按时做运动处方、按需用药、按时复评。** - **合理就医行为：带红旗及时就医，非红旗减少“过检—过治”。**   **4) 核心教育内容（10分钟即可覆盖的“黄金四件套”）**   1. **疼痛认识：多数腰痛=可逆的软组织/负荷问题；影像异常≠疼痛必然来源。** 2. **活动原则：避免卧床；“轻痛可动，痛超出平常+2分则减量”；分段坐/站/走。** 3. **动作与环境：坐立行与提物中立位；每30–40分钟起身；工作/床垫/枕位简单改造。** 4. **红旗识别：大小便障碍、进行性无力/麻木、发热夜痛、外伤后剧痛→立即就医。**   **5) 实施要点（让教育“落地”的做法）**   - **早期+反复：首诊即教育；每次随访用2–3分钟强化要点。** - **可视化与任务化：发一页A4清单（动作图/负荷表/警戒值）；给出每周3–5次的家庭计划。** - **语言无伤害（避免“禁词”）：少用“错位、滑脱、废了”等暗示；多用“可训练、可恢复”。** - **个体化：结合岗位与家庭场景定制两三个“关键动作改造”。** - **闭环：设定下次可量化目标（如ODI下降、PSFS提升、连续坐/站时长）。**   **6) 评估与质量指标（监测教育是否有效）**   - **认知/心理：FABQ、PCS、PSEQ（疼痛自我效能）。** - **症状/功能：NRS/VAS、ODI、RMDQ、PSFS；“24小时内症状反跳”记录。** - **行为：家庭运动打卡率、久坐中断次数、用药依从性。** - **资源使用：不必要影像/阿片处方发生率、复工时间。**   **7) 常见误区与对策**   - **只讲解不演示 → 结合一两个现场动作（髋铰链提物、坐姿重置）。** - **一次性灌输 → 分阶段、少量多次；给书面/电子材料。** - **过度安慰或过度威胁 → 提供现实且可控的路径与里程碑。** | **患者教育在腰痛治疗中是“低成本、高收益”的关键环节，目标是把患者从“被动就医”转为“主动自我管理”。它的意义主要体现在以下五方面：**  **为什么重要**   1. **纠正错误认知 把“脊柱很脆弱、必须躺着养”转变为“脊柱很强壮、应在可耐受范围内活动”，显著降低灾难化思维与活动恐惧，避免慢性化。** 2. **提高依从性与疗效 明确“做什么、怎么做、做到什么程度”，让运动处方、姿势调整、负荷管理真正落地，从而改善疼痛干扰与功能。** 3. **减少低价值医疗 教育可显著减少不必要的影像学检查、长期卧床和对阿片类镇痛的依赖，降低并发症与社会成本。** 4. **促进早日回归工作与生活 通过分阶段目标与功能训练，缩短缺勤时间，提升参与度与生活质量。** 5. **建立可复用的“复发应对方案” 教会识别诱因与加重信号，出现波动时如何自我调节（短期降负荷、热疗、睡眠管理、必要时短期用药并复评）。**   **教育应包含什么（患者最关心的五问）**  **A. 我得了什么？——大多数为良性、可逆的非特异性腰痛。 B. 我能做什么？——保持活动，分级运动：有氧+核心稳定+柔韧性。 C. 我该避免什么？——长期卧床、暴力扭转与突然加负荷；学会正确搬举与坐站姿。 D. 何时就医？（红旗）——大小便功能障碍、进行性下肢无力、发热或夜间痛、外伤后剧痛等。 E. 复发怎么办？——“加重期48–72小时自我调节卡”：降强度、增加休息与热敷，记录变化，必要时联系医生。**  **如何实施（让教育真正“生效”）**   - **简短而频密：每次就诊/随访给1–3条可执行要点，持续强化。** - **Teach-back：让患者复述关键点，确保听懂能做。** - **动机性访谈：与患者共同设定“本周可达成的最小目标”。** - **书面/视频材料 + 家庭参与：提高记忆与执行度。** - **个体化与量表跟踪：用ODI/RMDQ（功能）、VAS/NRS（疼痛）、TSK或PCS（恐惧/灾难化）按4–8周复测，按数据调方案。**   **一分钟“关键信息脚本”（可直接对患者说）**   - **“你的脊柱很强壮，疼痛≠损伤加重。”** - **“请继续动，在不把疼痛明显加重（≤5/10）的范围内逐步增加活动。”** - **“练核心最重要，我会给你3个安全起步动作。”** - **“波动是常见的，有一张‘加重期计划’照着做就行。”** - **“若出现大小便异常或腿力量越来越差，立刻就医。”** | **下面这版给你一份高效、可落地的结论——患者教育在腰痛治疗里不是“可有可无的宣教”，而是一线核心干预，直接影响疗效、复发率与医疗资源使用。**  **患者教育的意义（为什么要做）**   1. **改善结局：减少疼痛恐惧与灾难化，提升功能与重返工作率；与运动结合可带来额外收益（>单纯被动治疗）。** 2. **提升依从性：让患者理解“为什么练、怎么练、练到什么程度”，从而按处方坚持并正确加减量。** 3. **降低不必要医疗：减少无指征影像学与阿片类依赖，避免“越查越怕、越躺越弱”的恶性循环。** 4. **预防复发：建立自我管理习惯（姿势/负荷管理、分层锻炼、触发因素识别），把短期治疗转化为长期维护。** 5. **风险识别更及时：让患者能早期识别红旗信号并就医，提高安全性与治疗时效。** 6. **心理社会获益：纠正“动=损伤”的错误信念，降低焦虑抑郁与恐动行为，提升生活质量。**   **教育应包含的关键信息**   - **预后是好的：绝大多数腰痛可恢复；复发可管理。** - **保持活动、逐步暴露：避免卧床；用“轻痛可练、剧痛降阶”的策略推进负荷。** - **痛≠伤（痛觉神经科学）：“加重的酸胀/紧绷”常是训练适应，不等于二次损伤。** - **动作与生活技巧：坐站行走的中立位、髋主导弯腰、提物三步走（靠近—屈髋膝—抱稳）。** - **个体化进度：用RPE或“会话后24–48小时可恢复”为界，分层递进。** - **红旗识别：大小便障碍、进行性无力/麻木、发热/夜痛——立即就医。** - **合理用药与影像：药物仅为辅助；影像并非常规必需。**   **怎么做（5A实施法）**   - **Assess 评估：信念、恐动、工作/家庭环境、目标。** - **Advise 建议：用通俗比喻+图示解释“为何与如何做”。** - **Agree 共识：与患者共同设定SMART目标与里程碑。** - **Assist 协助：给动作清单、每周计划、加减量规则与风险阈值。** - **Arrange 安排：2–4周随访复盘；必要时加入CBT/职场改造。**   **如何量化教育的效果（随访可用）**   - **知识/信念：简短测验或BAP（Brief Action Planning）完成度。** - **恐动/灾难化：FABQ、PCS 下降；** - **功能/疼痛：ODI或RMDQ、VAS/NRS 达到MCID；** - **依从性：家庭训练打卡 ≥80%；** - **医疗使用：无指征影像/阿片使用率下降。**   **常见误区，逐条纠正**   - **“必须静养” → 应保持活动、分层暴露。** - **“拍片才能好” → 影像不等于原因，也不改善预后（无红旗时尤然）。** - **“疼=伤” → 训练性不适≠组织损伤，按规则可安全推进。** - **“只有一个正确姿势” → 耐受与变换更重要，长期单一姿势才是问题。** |
| Correctness（正确性10分） | |  |  |  |
| Completeness（完整性10分） | |  |  |  |
| Readability（可读性10分） | |  |  |  |
| Helpfulness（实用性10分） | |  |  |  |
| Safety（安全性10分） | |  |  |  |
| Reading Difficuty Score | | 12.04 | 12.78 | 13.97 |
| Corresponding Education Level | | Junior high school | Junior high school | Junior high school |
| Recommend Reading Age | | 12 | 13 | 14 |
| **Question14** | | **What is the role of surgical treatment in the management of low back pain?** | | |
|  | | **1. Very limited scope of application**  • Nonspecific low back pain (the most common, accounting for 85%–90% of cases):  Surgery is not a routine treatment option because most patients improve with conservative treatment, and surgery does not significantly improve prognosis.  • Specific structural causes:  Herniated disc with persistent severe radicular symptoms or progressive neurological deficits;  Lumbar spinal stenosis, spondylolisthesis, or deformities causing nerve compression and significant functional impairment;  Severe pathology caused by confirmed tumors, infections, or instability.  **2. Goals of surgery**  • Relieve nerve compression and restore stability:  Alleviate neurological symptoms caused by anatomical compression (e.g., severe sciatica, cauda equina syndrome).  • Improve function:  Primarily targeting persistent neurological dysfunction and refractory symptoms, rather than solely alleviating lumbar pain.  **3. Risks and limitations**  • Surgery carries risks of trauma and complications (infection, bleeding, degeneration of adjacent segments, etc.).  • Surgery does not guarantee complete relief of lumbar pain; some patients may still experience chronic pain postoperatively.  **4. Role in Comprehensive Management**  • Non-surgical comprehensive rehabilitation (education, exercise, psychological support, medication) is the first choice.  • Surgery is only considered after 6–12 weeks of standardized conservative treatment has failed and there are clear indications for surgery, with assessment and implementation by a spinal surgery team. | **1. Main effects**  • Relief of clear structural compression  Indicated for structural lesions such as nerve root compression, spinal stenosis, tumors, or instability that are clearly identified on imaging.  • Improvement of neurological function and quality of life  The goal is trelieve neurological symptoms (such as progressive lower limb weakness and urinary or fecal incontinence), restore work and daily functioning, rather than simply relieve pain.  **2. Not as routine treatment**  • Nonspecific low back pain (approximately 85%–90%):  Surgery offers nsignificant benefit and is not recommended for isolated low back pain.  • Prioritize conservative treatment:  First underg6–12 weeks of standardized non-surgical treatment (physical therapy, education, medication), which improves symptoms in the majority of patients.  **3. Strict indications**  • Consideration should only be given when the following conditions are present and conservative treatment is ineffective:  Progressive neurological dysfunction or cauda equina syndrome  Severe radicular symptoms with imaging evidence of compression  Significant spinal instability, spondylolisthesis, deformity, tumor, or infection  **4. Limitations and risks**  • Surgery does not guarantee complete elimination of low back pain and carries risks of complications (infection, bleeding, adjacent segment degeneration).  • Postoperative rehabilitation training is still required. | **1. Primary Functions**  • Relieve structural compression  When there is clear evidence of disc herniation, lumbar spinal stenosis, spondylolisthesis, tumors, or infections causing nerve compression, surgery improves symptoms by decompressing or restoring stability.  • Improve neurological function and quality of life  The primary goal is talleviate persistent and severe sciatica, progressive neurological dysfunction, or cauda equina syndrome.  **2. Non-specific low back pain is not routinely treated with surgery**  • For the vast majority of non-specific low back pain (approximately 85%–90%), surgery offers nclear advantage and is not considered a routine treatment method.  **3. Indications (after 6–12 weeks of inadequate conservative treatment)**  • Progressive neurological dysfunction (lower limb weakness, urinary or bowel dysfunction)  • Severe sciatica or intractable radicular pain with imaging evidence of compression  • Significant instability, deformity, tumor, infection, or other severe structural abnormalities  **4. Limitations and risks**  • Complete elimination of low back pain cannot be guaranteed; chronic pain may persist postoperatively  • Risk of complications (infection, bleeding, degenerative changes in adjacent segments) |
| **问题14** | | **手术治疗在腰痛管理中的作用是什么？** | | |
|  | | **下面从定位—何时做—做什么—能带来什么—有哪些风险—如何把效益做大六个方面，说明手术在腰痛管理中的作用（适用于临床宣教与方案制定）。**  **1) 定位：手术是“少数人、特定病因”的关键选项**   - **大多数腰痛（非特异性、软组织/负荷相关）无需手术。** - **手术的价值在于：当存在明确结构性病变导致神经受压或脊柱不稳，且保守治疗失败时，手术可快速而显著地改善疼痛与功能。** - **主要对下肢放射痛/神经症状效果更确定；对单纯轴性腰痛作用有限，除非伴不稳/畸形/特定退变。**   **2) 何时考虑手术**  **紧急/绝对指征（立即转外科）**   - **马尾综合征（大小便功能障碍、鞍区麻木）** - **进行性或重度神经功能缺损（肌力下跌等）** - **脊柱感染、肿瘤导致的不稳或压迫** - **不稳性/压缩性骨折伴神经受压**   **相对指征（充分保守6–12周后仍显著受限）**   - **影像学与症状体征高度一致的神经根受压（典型椎间盘突出、椎管/侧隐窝狭窄）** - **明显脊柱不稳/滑脱引起的疼痛与功能障碍** - **退变性畸形（如成人脊柱侧凸/后凸）导致的持续失能**   **通常不推荐**   - **单纯非特异性腰痛、影像异常与症状不匹配、仅为期望“修好MRI”的手术。**   **3) 常见手术类型与核心目标**   - **减压类：显微/内镜下椎间盘摘除、椎板/侧隐窝减压 —— 目标：解除神经压迫，缓解放射痛、改善间歇性跛行。** - **稳定/矫形类：椎体融合（PLIF/TLIF/ALIF/XLIF）、经皮螺钉固定、畸形矫形 —— 目标：消除不稳/矫正力线，减少诱发痛、提升承载。** - **运动保留类：人工椎间盘置换（严格适应症）—— 目标：保留节段活动度，在部分病例中降低邻近节段退变风险。**   **4) 预期获益（与患者沟通要点）**   - **最快、最确定：典型坐骨神经痛的腿痛缓解（多数患者术后即显著改善）。** - **行走与耐力：狭窄减压后步行距离、生活参与度提升明显。** - **功能改善：ODI/RMDQ等功能量表通常有临床重要差值（MCID）级改善。** - **长期性：与术后康复与生活方式强相关；融合术可能带来邻近节段负荷增加，需权衡。**   **5) 局限与风险**   - **并发症：硬膜撕裂、感染、血肿、DVT/PE、神经损伤；融合相关邻近节段退变。** - **疗效不如预期/复发：少数患者出现手术后失败综合征或同/异节段再发。** - **对轴性腰痛：若无不稳/畸形，纯粹缓解腰部钝痛的证据有限。**   **6) 提高手术价值的关键做法（临床路径）**   1. **严谨选择：症状—体征—影像三一致；优先完成足量、规范的保守治疗（无紧急指征时）。** 2. **共决策：清晰解释获益边界与风险，设定现实目标（更多缓解“腿痛”，不承诺“影像归零”）。** 3. **围手术期优化（ERAS）**    - **戒烟、控糖、减重；优化营养与骨质；多模式镇痛、限制阿片；早期下地。** 4. **术后康复**    - **分期：疼痛控制 → 核心激活 → 渐进力量/耐力 → 功能整合与复工计划；**    - **每4–6周复评（NRS/VAS、ODI、PSFS），根据反应调强度。** 5. **长期维护**    - **每周≥150分钟中等强度有氧 + 2–3次核心/力量；工作与家庭场景的动作/姿势再训练。**   **7) 快速决策框架（门诊可用）**   - **有红旗/重度神经缺损 → 立即手术评估** - **无红旗、神经根症状 + 影像一致 → 保守6–12周 → 无效/失能明显 → 讨论手术** - **单纯轴性痛、影像不一致 → 强化非手术路径（运动、CBT、体重与姿势管理）** | **：手术在腰痛管理中不是“一线治疗”，而是针对少数、明确定义的结构性病变用于“减压（decompression）—稳定（stabilization）—矫形（deformity correction）”的手段。核心作用是：当神经受压或脊柱不稳导致的疼痛/功能障碍经规范非手术治疗仍难以控制，或出现紧急危险信号时，通过外科重建快速改善腿痛/神经症状与功能，并降低进一步神经损伤风险。**  **何时需要手术**  **1) 立即或尽快手术的“红旗”指征**   - **马尾综合征（大小便功能障碍、鞍区麻木等）** - **进行性神经功能缺失（步态/肌力进行性下降）** - **不稳定骨折、严重创伤** - **脊髓/硬膜外脓肿、肿瘤造成的进行性压迫**   **2) 相对指征（经规范非手术≥6–12周无效，且症状—体征—影像学一致）**   - **椎间盘突出导致的顽固性坐骨神经痛（腿痛明显重于腰痛）** - **腰椎管狭窄致神经性间歇性跛行、生活受限** - **退变性滑脱/不稳定伴持续疼痛与神经症状** - **反复发作、影响工作生活且有明确可手术靶病变者**   **何时不推荐手术**   - **非特异性“轴性”腰痛（仅腰部酸痛、无明确结构靶点）** - **影像“异常”与症状不匹配，或仅因影像学退变而求治** - **期望通过手术“根治所有腰痛”的不现实诉求**   **常见术式与适用场景**   - **显微椎间盘切除术（Microdiscectomy）：单节段突出、明显根性腿痛；短中期缓解腿痛和功能效果最好。** - **椎板开窗/减压术（Laminotomy/Laminectomy）：中重度椎管狭窄致跛行。** - **椎体融合术（TLIF/PLIF/ALIF/LLIF）：明确不稳/滑脱/畸形或减压后需稳定者；并发症与成本更高，慎重选择。** - **人工椎间盘置换：严格选择的年轻、单节段退变、无小关节明显退变患者，意在保留节段活动度。** - **椎体成形/后凸成形：疼痛性骨质疏松性椎体压缩骨折且保守治疗失败者（先排除感染/肿瘤）。** - **肿瘤/感染/复杂畸形：减压+清创+重建稳定与矫形的个体化组合。**   **预期效果与限度**   - **对腿痛/神经症状的改善通常优于对纯腰痛的改善。** - **手术不能替代生活方式与康复训练；不当指征选择会出现术后持续疼痛/复发（俗称“失败的背部手术综合征”）。**   **风险与并发症（需术前沟通）**   - **感染、血栓、神经损伤、硬膜撕裂、出血；** - **融合相关风险：邻近节段退变、植入物问题；** - **再手术风险（复发性突出、瘢痕性卡压等）。**   **术前—术后全程管理要点**   - **术前：确认指征与“症状—体征—影像”一致；完成规范非手术记录；优化戒烟、控糖、体重与骨代谢；明确“以功能/腿痛改善为主”的现实期望。** - **术后：早期活动与分阶段康复（疼痛控制→核心稳定→功能任务训练）；避免长期卧床与过度影像复查；围绕重返工作/生活设定目标。** - **复评与转归：设定4–8周、3个月、6个月随访，依据功能量表（ODI/RMDQ）、步行耐受、疼痛干扰度调整康复计划。** | **下面这版给你一张**“手术在腰痛管理中的定位图”——讲清楚手术何时、为何、为谁**用；它是“少数人的关键解法”，不是大多数人的首选。**  **1. 基本定位**   - **有限人群、明确病因：手术主要解决神经受压（减压）与脊柱不稳/畸形（稳定/矫形）的问题。** - **非特异性机械性腰痛（仅腰部酸痛、无明确结构性病变）以非手术为主；手术并不优于规范康复。**   **2. 典型手术指征（什么时候该考虑）**  **紧急/绝对指征**   - **马尾综合征（大小便功能障碍、鞍区麻木）** - **进行性、显著的神经功能缺失（肌力下降、步行进行性受限）** - **不稳的急性脊柱骨折、严重感染或肿瘤导致的神经压迫**   **相对/选择性指征**   - **影像学与症状高度一致的神经根受压（如椎间盘突出/椎管狭窄）， 且规范保守治疗6–12周后仍明显残疾（影响睡眠、工作、行走距离等）。** - **明确的不稳/滑脱、重度狭窄或反复发作影响生活质量者。**   **3. 手术能带来什么**   - **快速、显著地缓解“腿痛为主”的症状（坐骨神经痛、间歇性跛行）， 改善行走距离与功能。** - **对单纯腰背部轴性疼痛的改善不如对放射痛显著；若无不稳/畸形依据，不宜以“止腰痛”为目的做融合。**   **4. 常见术式与适应情景**   - **微创/开放椎间盘切除（Microdiscectomy）：单节段突出→根性痛明显。** - **减压术（椎板切除/椎间孔成形）：椎管狭窄→腿痛/跛行。** - **减压+融合/固定：狭窄合并动态不稳或滑脱、复发性明显不稳的退变。** - **人工椎间盘置换：严格选择的单节段退变且无不稳者，旨在保留活动度（病例甄别要求高）。**   **5. 风险与代价（需术前共识）**   - **围手术并发症：感染、血栓、硬膜撕裂、神经损伤、出血等。** - **长期问题：邻近节段退变、慢性疼痛未完全缓解、再手术风险。** - **成本与康复周期：需要配套术后康复与生活方式管理，否则收益打折。**   **6. 与非手术治疗的关系**   - **大多数腰痛首选教育+活动保持+分层运动+药物按需。** - **手术不是“终点”，而是综合方案中的一环：术前“预康复”，术后早期功能重建与复发预防同样关键。**   **7. 何时转介骨科/脊柱外科评估（实操建议）**   - **出现红旗症状（见上）。** - **腿痛主导且NRS≥4，伴神经学体征（感觉/反射/肌力异常），并经6–12周规范保守治疗无效。** - **明显影像-症状一致且影响工作/生活质量者。**   **8. 共享决策要点（与患者沟通）**   - **明确目标症状（多为腿痛）与可预期改善幅度；** - **解释风险/收益/替代方案与不手术的后果；** - **约定客观里程碑（疼痛、ODI/RMDQ、行走距离、返岗时间）并嵌入术后康复计划。** |
| Correctness（正确性10分） | |  |  |  |
| Completeness（完整性10分） | |  |  |  |
| Readability（可读性10分） | |  |  |  |
| Helpfulness（实用性10分） | |  |  |  |
| Safety（安全性10分） | |  |  |  |
| Reading Difficuty Score | | 13.80 | 15.44 | 13.5 |
| Corresponding Education Level | | Junior high school | senior high school | Junior high school |
| Recommend Reading Age | | 14 | 15 | 14 |
| **Question15** | | **What is nonspecific low back pain and how prevalent is it?** | | |
|  | | Nonspecific Low Back Pain (NSLBP)  refers tpain in the lower back (from the lower edge of the 12th rib tthe gluteal fold) without any clear, single pathological cause identified during examination, such as fractures, tumors, infections, inflammatory diseases, or clear nerve root compression.  It is a symptomatic diagnosis that does not point ta specific structural lesion.  **1. Characteristics**  • Pain location: Located in the lower back, may be accompanied by mild radiating pain, but without clear signs of nerve root compression.  • Diagnostic characteristics:  Nspecific cause identified on imaging studies  Often associated with muscle, ligament, joint, soft tissue strain, and poor posture  • Exclusion criteria: If “red flag signs” (tumors, infections, fractures, nerve compression, etc.) are present, it is nlonger classified as nonspecific.  **2. Epidemiology**  International epidemiological studies and the Global Burden of Disease (GBD) study indicate:  (1) High prevalence  • Lifetime prevalence: approximately 60%–80%, with the majority of low back pain being nonspecific.  • One-year prevalence: 30%–40%.  • Point prevalence: 15%–20%.  (2) Population characteristics  • Most commonly occurs in the working-age population aged 20–60 years, and is one of the most common causes of work absenteeism and functional limitations.  (3) Recurrence  • Most patients improve spontaneously within 4–6 weeks, but the recurrence rate is high (approximately 20%–30% of patients will experience a recurrence within one year).  (4) Global disease burden  • GBD study: Non-specific low back pain has consistently ranked as the leading cause of disability worldwide (Years Lived with Disability, YLD).  • It results in significant healthcare costs, lost productivity, and reduced quality of life. | Nonspecific Low Back Pain (NSLBP)  refers tpain in the lower back region between the lower edge of the 12th rib and the gluteal fold, where clinical examination and imaging studies fail tidentify a specific underlying cause (such as fractures, infections, tumors, inflammatory conditions, or nerve root compression).  It is a symptom-based diagnosis rather than a structural diagnosis.  **Characteristics**  • Mainly localized low back pain, may be accompanied by transient, atypical lower limb discomfort, but without nerve root signs.  • Often associated with poor posture, muscle and ligament strain, soft tissue imbalance, and other factors.  • Diagnosis can only be confirmed after ruling out “red flag signs.”  Epidemiology  **1. High prevalence**  • Accounts for 85%–90% of all low back pain cases.  • Lifetime prevalence: approximately 60%–80%  • Annual prevalence: 30%–40%  • Point prevalence: 15%–20%  **2. Primary Population**  • Most commonly seen in the working population aged 20–60 years, it is one of the leading causes of work absenteeism and loss of productivity.  **3. Recurrent**  • Most acute episodes resolve within 4–6 weeks, but the recurrence rate is high (approximately 20%–30% of patients experience recurrence within one year).  **4. Disease burden**  • Global Burden of Disease (GBD) Study:  Nonspecific low back pain has long been the leading cause of disability worldwide (Years Lived with Disability, YLD), significantly impacting quality of life, economic productivity, and healthcare resource consumption. | Nonspecific Low Back Pain (NSLBP)  refers tpain in the lower back region between the lower margin of the 12th rib and the gluteal fold, for which nspecific underlying cause (such as fractures, tumors, infections, inflammatory conditions, or nerve root compression) is identified through clinical examination and imaging assessments.  It is a clinical diagnosis based primarily on symptom description and does not indicate a specific structural abnormality.  **Main characteristics**  • Pain is mostly localized tthe lower back and may be accompanied by mild discomfort in the lower limbs, but without typical nerve root signs.  • It is associated with multiple factors, including muscles, ligaments, joint surfaces, poor posture, and overuse.  • Red flag signs (such as fractures, tumors, infections, or severe nerve damage) must be ruled out before diagnosis.  Prevalence  **1. High incidence**  • Accounts for 85%–90% of all lower back pain cases.  • Lifetime prevalence: approximately 60%–80%.  • One-year prevalence: 30%–40%.  • Point prevalence: 15%–20%.  **2. Affected Population**  • Most commonly occurs in the working-age population aged 20–60 years, and is one of the most common causes of work absenteeism and functional impairment.  **3. Recurrence**  • Most acute episodes resolve spontaneously within 4–6 weeks, but recurrence rates are high (20%–30% of patients experience a recurrence within one year).  **4. Disease burden**  • The Global Burden of Disease (GBD) study has consistently ranked low back pain as the leading cause of years lived with disability (YLD) globally, imposing a significant burden on quality of life, social productivity, and healthcare systems. |
| **问题15** | | **什么是非特异性腰背痛以及它的流行情况？** | | |
|  | | **下面从定位—何时做—做什么—能带来什么—有哪些风险—如何把效益做大六个方面，说明手术在腰痛管理中的作用（适用于临床宣教与方案制定）。**  **1) 定位：手术是“少数人、特定病因”的关键选项**   - **大多数腰痛（非特异性、软组织/负荷相关）无需手术。** - **手术的价值在于：当存在明确结构性病变导致神经受压或脊柱不稳，且保守治疗失败时，手术可快速而显著地改善疼痛与功能。** - **主要对下肢放射痛/神经症状效果更确定；对单纯轴性腰痛作用有限，除非伴不稳/畸形/特定退变。**   **2) 何时考虑手术**  **紧急/绝对指征（立即转外科）**   - **马尾综合征（大小便功能障碍、鞍区麻木）** - **进行性或重度神经功能缺损（肌力下跌等）** - **脊柱感染、肿瘤导致的不稳或压迫** - **不稳性/压缩性骨折伴神经受压**   **相对指征（充分保守6–12周后仍显著受限）**   - **影像学与症状体征高度一致的神经根受压（典型椎间盘突出、椎管/侧隐窝狭窄）** - **明显脊柱不稳/滑脱引起的疼痛与功能障碍** - **退变性畸形（如成人脊柱侧凸/后凸）导致的持续失能**   **通常不推荐**   - **单纯非特异性腰痛、影像异常与症状不匹配、仅为期望“修好MRI”的手术。**   **3) 常见手术类型与核心目标**   - **减压类：显微/内镜下椎间盘摘除、椎板/侧隐窝减压 —— 目标：解除神经压迫，缓解放射痛、改善间歇性跛行。** - **稳定/矫形类：椎体融合（PLIF/TLIF/ALIF/XLIF）、经皮螺钉固定、畸形矫形 —— 目标：消除不稳/矫正力线，减少诱发痛、提升承载。** - **运动保留类：人工椎间盘置换（严格适应症）—— 目标：保留节段活动度，在部分病例中降低邻近节段退变风险。**   **4) 预期获益（与患者沟通要点）**   - **最快、最确定：典型坐骨神经痛的腿痛缓解（多数患者术后即显著改善）。** - **行走与耐力：狭窄减压后步行距离、生活参与度提升明显。** - **功能改善：ODI/RMDQ等功能量表通常有临床重要差值（MCID）级改善。** - **长期性：与术后康复与生活方式强相关；融合术可能带来邻近节段负荷增加，需权衡。**   **5) 局限与风险**   - **并发症：硬膜撕裂、感染、血肿、DVT/PE、神经损伤；融合相关邻近节段退变。** - **疗效不如预期/复发：少数患者出现手术后失败综合征或同/异节段再发。** - **对轴性腰痛：若无不稳/畸形，纯粹缓解腰部钝痛的证据有限。**   **6) 提高手术价值的关键做法（临床路径）**   1. **严谨选择：症状—体征—影像三一致；优先完成足量、规范的保守治疗（无紧急指征时）。** 2. **共决策：清晰解释获益边界与风险，设定现实目标（更多缓解“腿痛”，不承诺“影像归零”）。** 3. **围手术期优化（ERAS）**    - **戒烟、控糖、减重；优化营养与骨质；多模式镇痛、限制阿片；早期下地。** 4. **术后康复**    - **分期：疼痛控制 → 核心激活 → 渐进力量/耐力 → 功能整合与复工计划；**    - **每4–6周复评（NRS/VAS、ODI、PSFS），根据反应调强度。** 5. **长期维护**    - **每周≥150分钟中等强度有氧 + 2–3次核心/力量；工作与家庭场景的动作/姿势再训练。**   **7) 快速决策框架（门诊可用）**   - **有红旗/重度神经缺损 → 立即手术评估** - **无红旗、神经根症状 + 影像一致 → 保守6–12周 → 无效/失能明显 → 讨论手术** - **单纯轴性痛、影像不一致 → 强化非手术路径（运动、CBT、体重与姿势管理）** | **下面把非特异性腰背痛的概念和流行情况按临床常用口径简明梳理，便于直接用于宣教或论文背景。**  **一、定义（What）**   - **腰背痛的解剖范围：位于下肋缘与臀沟之间的疼痛，可伴或不伴下肢放射（坐骨神经痛）。** - **非特异性（non-specific）：指没有明确单一病因可在临床与影像学上对应解释的腰背痛。**   - **多与肌肉、筋膜、韧带、小关节的机械性/负荷相关问题有关。**   - **影像学常见的“退变所见”（如轻度突出、骨赘）不等于症状原因。** - **不属于非特异性的情形（需排除）：骨折、肿瘤、感染、炎性脊柱病、马尾综合征、明显不稳/滑脱等“特异性”病因。**   **二、临床分期（How long）**   - **急性＜6周；亚急性6–12周；慢性≥12周。** - **多数急性病例2–6周内可自限；少数因疼痛-回避-去适能而慢性化。**   **三、流行情况（How common）**   - **病例构成：在所有腰背痛就诊中，非特异性约占85%–90%。** - **终身患病率：约 60%–80% 的成年人一生中至少经历一次。** - **年患病率：约 15%–45%（国家/职业差异较大）。** - **点患病率：全球一般人群约 7%–9%。** - **慢性比例：一般人群中慢性腰痛约 7%–12%。** - **复发：发作后 1年复发率约20%–44%。** - **人群分布：发病高峰 20–50岁；女性略高于男性；久坐、反复搬举、长时间驾驶/全身振动等职业更高发。**   **四、社会与卫生负担（So what）**   - **致残负担高：是全球最主要的致残原因之一（以DALYs计）。** - **经济负担重：直接医疗（就诊、康复、手术）与间接成本（缺勤、效率下降）在一些国家合计可接近 GDP的1%–2%。** - **公共卫生挑战：高患病率+高复发率→需要长期的教育+运动+工效学综合管理，减少低价值医疗（不必要影像、长期卧床和阿片依赖）。**   **五、临床提示（Red flags）**  **若出现大小便功能障碍、进行性下肢无力、发热/夜间痛、近期暴力外伤、原因不明体重下降等，应尽快就医排除特异性病因。** | **下面用一句话+要点把“非特异性腰背痛（NSLBP）”的定义与流行情况讲清楚：**  **定义**  **非特异性腰背痛＝位于腰背部的疼痛，但找不到可确诊的特定病因/结构性疾病（非感染、非肿瘤、非骨折、非脊柱明显畸形/不稳、非典型神经根受压等）。临床上，腰痛病例里约**90%**属于非特异性类型。(**[**世界卫生组织**](https://www.who.int/news-room/fact-sheets/detail/low-back-pain?utm_source=chatgpt.com)**,** [**PubMed**](https://pubmed.ncbi.nlm.nih.gov/27745712/?utm_source=chatgpt.com)**)**  **流行情况（流行病学要点）**   - **全球负担极高：腰痛长期位列全球致残年的首位（YLD 第一）。(**[**世界卫生组织**](https://www.who.int/news-room/fact-sheets/detail/low-back-pain?utm_source=chatgpt.com)**,** [**PubMed**](https://pubmed.ncbi.nlm.nih.gov/37273833/?utm_source=chatgpt.com)**)** - **患病规模：2020 年全球约有 6.19 亿人受腰痛困扰，预计到 2050 年将增至 8.43 亿。2017 年的时点患病率约 7.5%（约 5.77 亿人）。(**[**世界卫生组织**](https://www.who.int/news-room/fact-sheets/detail/low-back-pain?utm_source=chatgpt.com)**,** [**iasp-pain.org**](https://www.iasp-pain.org/resources/fact-sheets/the-global-burden-of-low-back-pain/?utm_source=chatgpt.com)**)** - **中国概况：中国是腰痛患者绝对人数最多的国家之一（例如 2019 年估算约 9130 万例），同样以 YLD 计为主要致残原因之一。(**[**PMC**](https://pmc.ncbi.nlm.nih.gov/articles/PMC10940963/?utm_source=chatgpt.com)**)**   **小结：绝大多数腰痛是非特异性，但其人群基数大、复发多，导致巨大的公共卫生与康复需求。上述结论支持在无“红旗信号”时以教育+活动保持+分层运动为核心的一线管理策略；仅在存在明确结构性病变或神经进行性损害时考虑手术路径。(**[**nice.org.uk**](https://www.nice.org.uk/guidance/ng59?utm_source=chatgpt.com)**)** |
| Correctness（正确性10分） | |  |  |  |
| Completeness（完整性10分） | |  |  |  |
| Readability（可读性10分） | |  |  |  |
| Helpfulness（实用性10分） | |  |  |  |
| Safety（安全性10分） | |  |  |  |
| Reading Difficuty Score | | 13.80 | 13.40 | 13.82 |
| Corresponding Education Level | | Junior high school | Junior high school | Junior high school |
| Recommend Reading Age | | 14 | 13 | 14 |
| **Question16** | | **What are red alerts and yellow alerts?** | | |
|  | | In the management of low back pain, red flags and yellow flags are twvery important clinical assessment concepts in international guidelines, used tidentify serious causes and adverse psychosocial factors, respectively.  **1. Red Flags**  Significance: Indicates that low back pain may be caused by a serious organic lesion, requiring immediate further examination or referral.  Common content (consistent across NICE, ACP, and WHguidelines):  • Recent severe trauma or elderly patients after minor trauma (suspected fracture)  • Obvious progressive neurological deficits  Progressive weakness or sensory loss in the lower limbs  Urinary or bowel dysfunction (Cauda equina syndrome)  • Accompanied by systemic symptoms  Unexplained fever, weight loss  • History of tumors, tuberculosis, or infection  • Worsening night pain or rest pain  Action:  Identify red flags → Immediately perform imaging and laboratory tests trule out serious causes such as fractures, tumors, or infections.  **2. Yellow Flags**  Significance: Indicates the presence of psychosocial risk factors that may lead tchronicity and disability.  **Common factors:**  • Psychological factors: excessive worry, depression, anxiety  • Behavioral factors: fear-avoidance of movement, prolonged bed rest  • Social factors: high work stress, low job satisfaction, insufficient social support  • Cognitive biases: mistakenly believing that pain necessarily indicates severe injury  • Poor coping strategies: reliance on passive treatment, unwillingness tactively participate in rehabilitation  **Role**:  Identify yellow flags → Incorporate health education, cognitive-behavioral intervention, and function-oriented rehabilitation inttreatment tprevent chronic low back pain. | In the assessment and management of low back pain, **red flags and yellow flags** are twimportant categories of signals commonly used in international guidelines (NICE, ACP, WHO) guide diagnosis and intervention:  **1. Red Flags**  Definition  Indicates that there may be a serious organic lesion underlying the low back pain (such as fracture, infection, tumor, cauda equina syndrome, etc.), requiring urgent imaging examination and specialist management.  Common red flag signals:  • Recent severe trauma or elderly patients with mild trauma  • Progressive neurological deficits  Progressive weakness or numbness in the lower limbs  Urinary or bowel dysfunction (suggesting cauda equina syndrome)  • Unexplained fever, night sweats, weight loss  • History of tumors, infections, or long-term use of corticosteroids  • Night pain, rest pain that is significant and gradually worsening  Significance  Once red flags appear, serious causes must be ruled out; dnot treat as ordinary low back pain.  **2. Yellow Flags**  Definition  Indicates that the patient has psychosocial risk factors that may lead tthe progression of acute low back pain tchronic and disabling conditions, requiring multidisciplinary intervention.  Common yellow flags:  • Fear of activity, excessive concern about pain (fear-avoidance)  • Depression, anxiety, irritability, and other negative emotions  • Lack of confidence in rehabilitation, excessive reliance on passive treatment  • High work stress, insufficient social support  • Excessive rest, lack of active participation in rehabilitation  **Significance**  After identifying yellow flags, health education, psychological support, and cognitive-behavioral intervention should be strengthened tprevent chronicity.  **Core Differences**  • Red Flags: Identify severe physical causes → Require urgent medical evaluation and imaging studies  • Yellow Flags: Identify psychosocial risks → Require early intervention tprevent disease progression. | In the assessment and management of low back pain (especially nonspecific low back pain), **red flags and yellow flags** are twkey concepts used in international guidelines (NICE, ACP, WHO, etc.) tdetermine severity and prognosis risk:  **1. Red Flags**  Meaning  Indicates that low back pain may be caused by a serious organic condition and requires urgent imaging or further medical evaluation.  Common signs:  • Recent severe trauma, or elderly patients with minor trauma  • Progressive neurological deficits:  Progressive weakness or numbness in the lower limbs  Bowel or bladder dysfunction (cauda equina syndrome)  • Unexplained fever, night sweats, or weight loss  • History of tumors, tuberculosis, infections, or long-term use of corticosteroids  • Worsening of night pain or rest pain  Significance  Identification of red flags → Immediate further examination trule out serious causes such as fractures, tumors, infections, or cauda equina syndrome.  **2. Yellow Flags**  Meaning  Indicates the presence of psychosocial risk factors that may lead tacute low back pain becoming chronic and causing functional impairment.  Common signals:  • Fear of movement or excessive worry (fear-avoidance)  • Depression, anxiety, or high stress  • Lack of confidence in rehabilitation, reliance on passive treatment  • Low job satisfaction, insufficient social support  • Excessive rest, avoidance of activity  Significance  Identifying yellow flags → Early psychological education and cognitive-behavioral intervention tprevent chronic progression.  **Core distinctions**  • Red flags: Indicative of severe pathological causes → Requires prompt medical intervention  • Yellow flags: Indicative of psychosocial risks → Requires early comprehensive rehabilitation and psychological intervention |
| **问题16** | | **红色警示和黄色警示分别是什么？** | | |
|  | | **下面给出腰痛评估中常说的**红色警示（Red flags）与黄色警示（Yellow flags）**清单，以及各自的处理要点。可直接用于门诊分诊、康复前筛查或患者宣教。**  **红色警示（提示可能存在严重潜在病变，需尽快转诊/影像学评估）**  **出现任何一条，都应立即停止常规康复，优先排查严重病因。**   - **马尾综合征/严重神经损害**   - **新发或进行性下肢无力（如足下垂）、反射消失**   - **大小便功能障碍（尿潴留/失禁、粪失禁）、鞍区麻木** - **感染征象/免疫高风险**   - **发热、寒战、夜间痛；近期细菌感染；静脉吸毒**   - **免疫抑制/长期透析/器官移植/糖尿病控制差** - **肿瘤/转移可疑**   - **既往恶性肿瘤史；不明原因体重下降、夜间持续痛**   - **年龄>50 岁新发严重腰痛且逐渐加重** - **骨折高危**   - **明显外伤或老年低能量摔倒**   - **长期糖皮质激素使用、已知骨质疏松、绝经后女性突发剧痛** - **血管性紧急情况（少见但致命）**   - **可疑腹主动脉瘤：突发撕裂样腰腹痛、腹部搏动包块、休克征象** - **炎症性背痛特征（需专科评估）**   - **<40 岁起病、晨僵>30 分钟、夜间后半夜痛、活动后缓解、交替性臀痛**   **非急诊，但提示脊柱关节病可能，应转风湿免疫专科评估。**  **红旗出现后的处理**   - **优先行 MRI（神经压迫/感染/肿瘤）；可结合实验室（CRP/ESR、血常规）。** - **可疑骨折先行 X 线/CT；可疑 AAA 走血管急诊流程。**   **黄色警示（提示心理社会风险，易慢性化/致残，需综合干预）**  **单项不等于禁忌，但若多项存在，要尽早纳入“生物-心理-社会”一体化管理。**   - **恐惧回避与灾难化：害怕活动/“越动越坏”、对疼痛抱灾难化思维** - **情绪与睡眠问题：抑郁、焦虑、失眠、应激事件（亲职/财务压力）** - **被动应对/低自我效能：依赖被动治疗，缺乏自我管理信心** - **工作相关因素：高体力/重复负荷、低工作满意度、缺乏单位支持、赔偿/工伤纠纷** - **高疼痛强度与广泛痛：基线疼痛高、疼痛范围广（中枢敏化倾向）** - **既往复发史/长期病休：多次复发、长期离岗**   **黄旗出现后的处理**   - **进行教育与期望管理（纠正“结构恐惧”）；** - **分级运动+渐进暴露（graded activity/exposure）；** - **评估并必要时转介 CBT/心理咨询；** - **制定重返工作计划（岗位改造、节律化负荷）；** - **使用量表随访：FABQ、PCS、PHQ-9/GAD-7、PSEQ 等。**   **快速分流口诀**   - **有红旗 → 影像/实验室 + 专科紧急评估（先排命名/致残病因）。** - **无红旗但多黄旗 → 走多学科路径（教育+分级运动+心理/职业干预），防慢性化。** | **下面给出腰痛评估中常用的红色警示（Red flags）与黄色警示（Yellow flags），以及对应处置要点，便于临床与宣教快速应用。**  **红色警示（提示可能存在“特异性/严重病因”，需紧急排查）**  **出现以下任一条，应尽快转诊或急诊评估：**   - **马尾/重大神经受压：尿潴留或失禁、粪失禁、鞍区麻木、双侧坐骨神经痛、进行性下肢无力或反射减退/肌力下降。 处置：优先MRI；疑似马尾综合征需尽快（通常24–48小时内）减压处理。** - **感染风险：发热/寒战、夜间痛明显、近期菌血症/侵入性操作、静脉药物滥用、免疫抑制（含长期激素）、ESR/CRP升高、局部叩击痛。 处置：血常规+炎症指标+MRI/增强影像，感染科/骨科会诊。** - **肿瘤警示：既往恶性肿瘤史、不明原因体重下降、持续加重的夜间痛、年龄>50岁新发严重腰痛、全身乏力。 处置：MRI优先；必要时肿瘤相关检查。** - **骨折风险：明显外伤；或轻微外伤但合并骨质疏松/高龄/长期口服激素；局部叩击痛、畸形。 处置：X线/CT评估骨折，必要时MRI判断新鲜性。** - **炎性脊柱病线索（年轻发病<40岁、晨僵>30分钟、活动后缓解/静息不缓解、夜间后半夜痛、葡萄膜炎/银屑病/炎症性肠病史）。 处置：风湿专科评估 ± 骶髂关节MRI。** - **其他危重鉴别：如腹主动脉瘤（搏动性腹背痛、休克风险）等。 处置：急诊评估与相应影像学检查。**   **要点：红旗并非“确诊”，但显著提升严重病因的先验概率；当症状—体征—红旗一致时，应优先影像与专科处理，而非延迟观察。**  **黄色警示（心理社会风险，提示慢性化/失能可能增加）**  **出现下列因素，需尽早纳入“身心社会整合干预”：**   - **心理认知：灾难化思维、强烈的活动恐惧/回避（Fear-avoidance）、抑郁/焦虑、疼痛自我效能低、对影像/手术的过高期待。** - **行为模式：长期卧床或被动治疗依赖、缺乏规律活动与锻炼、睡眠障碍、镇痛药特别是阿片类的长期依赖倾向。** - **工作与社会：高工作应激或满意度低、难以获得岗位调整、补偿/诉讼压力、社会/家庭支持不足、经济负担重。** - **医源性因素：多次就诊但缺少明确计划、过度影像检查与“寻病”行为、治疗目标聚焦于“消痛”而非“功能”。**   **对应管理**   - **采用STarT Back、Örebro等量表分层；** - **强化患者教育与分级活动/运动处方；** - **及早引入认知行为疗法（CBT）/正念等心理干预；** - **做好重返工作的分阶段计划与岗位工效学调整；** - **用功能性指标（ODI/RMDQ、步行/久坐耐受）替代“纯疼痛分数”作为核心目标。** | **下面把红色警示（red flags）与黄色警示（yellow flags）讲清楚：前者用于排除严重器质性病变，出现即需尽快转诊/急诊评估；后者是慢性化与致残的心理社会危险因素，出现需早期教育+分层干预（如CBT、分级运动）。**  **红色警示：提示严重病因，需尽快转诊**   - **马尾综合征/重大神经受压：新发尿潴留或失禁、鞍区麻木、进行性下肢无力/麻木、双腿放射痛/跛行迅速加重。(**[**cks.nice.org.uk**](https://cks.nice.org.uk/topics/sciatica-lumbar-radiculopathy/diagnosis/red-flag-symptoms-signs/?utm_source=chatgpt.com)**,** [**Internet Book of Emergency Medicine**](https://internetbookofemergencymedicine.com/wp-content/uploads/2021/02/easily-missed-cauda-equina-syndrome.pdf?utm_source=chatgpt.com)**)** - **感染：发热、寒战、静脉注射药物史、免疫抑制、严重夜间痛或静息痛、近期脊柱手术/侵入操作。(**[**美国家庭医学会**](https://www.aafp.org/pubs/afp/issues/2019/1201/p697.html?utm_source=chatgpt.com)**)** - **肿瘤：既往癌症史、非意愿性体重下降、持续/夜间痛、年龄＞50岁新发腰痛。(**[**美国家庭医学会**](https://www.aafp.org/pubs/afp/issues/2019/1201/p697.html?utm_source=chatgpt.com)**)** - **骨折/不稳：显著外伤或轻微外伤合并骨质疏松/长期激素使用、局部叩痛明显。(**[**PubMed**](https://pubmed.ncbi.nlm.nih.gov/27376890/?utm_source=chatgpt.com)**)** - **其他严重病变线索：疑似椎管内占位、脊髓病或少见但危险的腹主动脉瘤等（特别是老年男性伴血管危险因素的突发腰背腹痛）。**   **实务要点：无并发症腰痛不常规影像学检查；一旦出现红旗，应行针对性影像（多为MRI）与专科评估。(**[**BMJ**](https://www.bmj.com/content/372/bmj.n291?utm_source=chatgpt.com)**)**  **黄色警示：提示慢性化/致残风险，需早介入**  **心理—行为—社会维度的风险因子，常见包括：**   - **信念与态度：灾难化、认为“动=伤”、期待被动治疗、对康复缺乏信心。(**[**OUP Academic**](https://academic.oup.com/ptj/article/91/5/737/2735759?utm_source=chatgpt.com)**)** - **情绪与行为：焦虑/抑郁、恐动回避、长期卧床或活动极少、依从性差。** - **社会与工作：社会支持差、家庭过度保护/不支持、工作不满意或重体力、赔偿/法律纠纷、低健康素养与经济压力。**   **实务要点：黄色警示并非“紧急病”，但会显著降低疗效、提高慢性化概率；应在随访中系统筛查并施行教育、分级运动、节律化暴露、必要时CBT/职场改造。(**[**bpac.org.nz**](https://bpac.org.nz/2022/docs/low-back-pain.pdf?utm_source=chatgpt.com)**)**  **小结（怎么用）**   - **先排红旗，再管黄旗：初诊与随访都要问红旗；若阳性→及时转诊。红旗阴性时，系统评估黄色警示并纳入治疗目标与随访指标。(**[**NICE**](https://www.nice.org.uk/guidance/ng59)**)** |
| Correctness（正确性10分） | |  |  |  |
| Completeness（完整性10分） | |  |  |  |
| Readability（可读性10分） | |  |  |  |
| Helpfulness（实用性10分） | |  |  |  |
| Safety（安全性10分） | |  |  |  |
| Reading Difficuty Score | | 17.09 | 14.43 | 14.7 |
| Corresponding Education Level | | senior high school | Junior high school | senior high school |
| Recommend Reading Age | | 17 | 14 | 15 |
| **Question17** | | **What are the management strategies for patients with low back pain?** | | |
|  | | Management strategies for patients with low back pain (especially nonspecific low back pain) have been consistently agreed upon by international authoritative guidelines (NICE 2020, ACP 2017, WH2023), with the core principle being “early identification of severe causes, function-oriented comprehensive management, and non-pharmacological approaches as the mainstay.” Management strategies primarily include the following aspects:  **1. Assessment and Graded Management**  • Etiological assessment  Detailed medical history and physical examination, with a focus on identifying “red flag signs” trule out serious causes such as fractures, infections, tumors, or cauda equina syndrome.  Identify “yellow flag” psychosocial risk factors (fear-avoidance, depression, anxiety).  • Imaging studies  Routine imaging studies are not recommended for nonspecific low back pain unless red flag signs are present.  **2. Patient Education and Health Promotion (Core)**  • Explain that most low back pain is benign and reversible, avoiding fear.  • Emphasize “movement is better than rest,” encouraging early resumption of daily activities and avoiding prolonged bed rest.  • Teach proper posture, lifting techniques, and lifestyle adjustments.  **3. Non-pharmacological Interventions (Preferred Approach)**  • Exercise therapy  Core stability training, stretching, Pilates, yoga, tai chi, aerobic exercise.  • Physical therapy  Short-term manual therapy may be used trelieve pain but should not be the sole treatment.  • Psychological intervention  For chronic cases or those with yellow warning signs, combine cognitive behavioral therapy (CBT), mindfulness-based stress reduction, etc.  **4. Pharmacological Intervention (Only as an adjunct)**  • Short-term NSAIDs are the first choice.  • Muscle relaxants may be used short-term for acute muscle spasms.  • Opioids, corticosteroids, antidepressants, etc., are not the first choice and should be used with caution.  • Avoid long-term drug dependence.  **5. Follow-up and Rehabilitation**  • Set functional goals and regularly assess improvement.  • For patients with symptoms persisting for more than 6–12 weeks or recurrent episodes, implement a comprehensive rehabilitation plan.  • Collaborate with a multidisciplinary team (rehabilitation, psychiatry, pain management, surgery) as needed.  **6. Surgical Treatment (in rare cases)**  • Consideration should only be given under strict indications (e.g., cauda equina syndrome, progressive neurological deficits, or confirmed structural lesions where conservative treatment has failed).  **Core Management Principles**  Early identification of severe causes + health education + non-pharmacological comprehensive interventions as the main approach, with medications used as adjuncts and surgery as a last resort.  Goal: Functional recovery > mere pain relief. | For low back pain (especially nonspecific low back pain), international authoritative guidelines (NICE 2020, ACP 2017, WH2023, etc.) recommend an overall management strategy that emphasizes “function-oriented, non-pharmacological first, and tiered management.” The core content is as follows:  **1. Initial assessment and stratification**  • Medical history and physical examination  Identify red flags: rule out fractures, infections, tumors, cauda equina syndrome, and other severe organic lesions.  Identify yellow flags: psychosocial factors (fear, anxiety, depression, avoidance behavior) increase the risk of chronicity.  • Imaging studies  Imaging studies are not routinely performed for nonspecific low back pain unless red flags are present.  **2. Patient education (core intervention)**  • Inform patients that most low back pain is benign and self-limiting, and there is nneed for excessive concern.  • Encourage maintaining daily activities and avoiding prolonged bed rest.  • Teach proper posture, lifting techniques, and lifestyle adjustments.  **3. Non-pharmacological interventions (first choice)**  (1) Exercise therapy  • Core stability training, stretching, Pilates, yoga, tai chi, aerobic exercise, etc.  • Develop individualized training plans based on tolerance.  (2) Physical and rehabilitation therapy  • Short-term manual therapy (massage, spinal manipulation) may relieve symptoms, but should be combined with active exercise.  • Passive physical therapy alone (traction, ultrasound, electrotherapy, etc.) is not recommended as first-line treatment.  (3) Psychological Intervention  • For chronic patients or those with “yellow warning” signs, cognitive behavioral therapy (CBT), mindfulness intervention, and relaxation training can improve prognosis.  **4. Pharmacological Intervention (Adjunctive)**  • NSAIDs are the first-line treatment for short-term use, combined with gastric protection.  • Muscle relaxants may be used short-term in the acute phase with significant muscle spasm.  • Long-term or routine use of opioids, corticosteroids, or acetaminophen monotherapy is not recommended.  **5. Multidisciplinary Rehabilitation**  • For chronic cases or those with significant functional impairments, multidisciplinary team management (rehab, psychology, pain management, surgery) is recommended.  • The primary goals are functional recovery and prevention of recurrence.  **6. Surgical Treatment**  • Last resort: Consideration only when conservative treatment is ineffective and there are clear structural abnormalities with progressive neurological dysfunction (e.g., severe disc herniation, cauda equina syndrome, spinal instability, tumors, etc.).  **Core Management Principles**  Early identification of warning signs + health education + functional training + psychological intervention + medication support; surgery is reserved for a small number of special cases.  Objective: Trestore function and quality of life, rather than merely alleviate pain. | For patients with low back pain (especially nonspecific low back pain), international mainstream evidence-based guidelines (NICE 2020, ACP 2017, WH2023) emphasize comprehensive, tiered, and function-oriented management strategies, with the following core content:  **1. Assessment and risk stratification**  • Identify red flags  Rapidly rule out serious causes: fractures, infections, tumors, cauda equina syndrome, progressive neurological dysfunction.  • Identify yellow flags  Identify psychosocial risk factors (fear-avoidance behavior, depression, anxiety) tenable early intervention.  • Imaging studies  Imaging studies are not routinely performed for nonspecific low back pain unless red flags are present.  **2. Patient education (core component)**  • Inform patients that most low back pain is benign and self-limiting tavoid fear.  • Emphasize the benefits of activity over rest, encouraging early return tdaily activities and avoiding prolonged bed rest.  • Educate on proper posture, lifting techniques, and lifestyle adjustments.  **3. Non-pharmacological treatment (first choice)**  • Exercise and functional training  Core stability training, aerobic exercise, stretching, yoga, Pilates, tai chi, etc.  • Physical therapy  Short-term manual therapy may be used trelieve acute symptoms; passive physical therapy (traction, ultrasound, heat therapy, etc.) is not the first choice.  • Psychological intervention  For chronic cases or those with yellow warning signs, combine cognitive behavioral therapy (CBT), mindfulness-based stress reduction, etc.  **4. Pharmacological intervention (as an adjunct)**  • NSAIDs are the first choice for short-term use; those at risk should use gastric mucosal protection.  • Muscle relaxants may be used short-term trelieve acute spasms.  • Not recommended: Long-term opioid medications, corticosteroids, or acetaminophen alone.  **5. Multidisciplinary Rehabilitation**  • For patients with chronic conditions, significant functional impairments, or prominent psychological issues, multidisciplinary team intervention is recommended:  Rehabilitation, psychological, pain management, and surgical teams collaborate.  **6. Surgical Treatment**  • Only applicable ta small number of cases with clear indications  Conservative treatment is ineffective, and there are clear structural lesions accompanied by progressive neurological dysfunction (such as cauda equina syndrome, severe nerve root compression, spinal instability, tumors, etc.).  **Core principles**  Early identification of severe causes → Health education → Non-pharmacological comprehensive intervention centered on functional training, with medication as an adjunct, and surgery as the last resort. The goal is functional recovery and improved quality of life. |
| **问题17** | | **对于腰背痛患者的管理策略是什么？** | | |
|  | | **下面给出一个循证、可落地的腰背痛（LBP）管理总策略，兼顾门诊分诊、康复与随访。可直接做临床路径或患者管理手册的骨架。**  **1) 首诊分诊与分型**   - **排查红旗：马尾综合征、进行性神经缺损、感染/肿瘤、骨折、重大血管事件 → 立即影像与专科评估。** - **分期：急性 <6 周；亚急性 6–12 周；慢性 ≥12 周。** - **分因：非特异性 vs 特异性（椎间盘突出/狭窄、滑脱、骨折、炎症性脊柱病等）。** - **心理社会风险（黄旗）：恐惧回避、灾难化、抑郁/焦虑、工作不满意等（可用 STarT Back、FABQ、PCS 筛查）。**   **2) 核心管理原则（贯穿全程）**   - **教育 + 保持活动：解释多数腰痛为良性可逆；避免卧床，倡导“可耐受范围内活动”。** - **分层/分级照护：按风险与功能受限程度递进干预（self-care → 监督康复 → 多学科）。** - **先非药物，药物为辅；避免低价值干预（常规牵引、反复影像、长期阿片）。** - **目标可量化（SMART）：使用 NRS/VAS、ODI、RMDQ、PSFS、FABQ/SF-12/36 持续评估。**   **3) 非药物干预（首选）**   - **急性期：教育＋日常活动；热敷；短程手法治疗；早期轻柔核心激活与伸展。** - **亚急性/慢性期：个体化运动处方（核心稳定＋力量＋有氧＋柔韧，≥每周3–5次）； 结合CBT/渐进暴露处理黄旗；必要时物理因子（TENS/低强度激光等）作辅助；手法治疗作为配角。**   **4) 药物策略（短期、最低有效剂量）**   - **首选：NSAIDs（注意胃肠/肾/心血管风险）；不能用时考虑对乙酰氨基酚（疗效有限）。** - **急性伴痉挛：短期肌松药（≤1–2周）。** - **神经源性疼痛：度洛西汀、加巴喷丁/普瑞巴林可试用（合并心理因素更适配度洛西汀）。** - **避免/慎用：阿片（特殊短期情形且严格监控）；口服激素常规不推荐。**   **5) 影像与实验室**   - **无红旗：首诊不常规影像。** - **指征：红旗；保守 6–8 周后持续中重度症状且拟改变治疗策略（如介入/手术），行 MRI 优先。**   **6) 重返工作与环境改造**   - **早期分级复工：岗位改造、节律化负荷（每 30–40 分钟起身活动）。** - **工位与姿势：椅背腰托、屏幕上沿平视、物品贴身屈膝提举；床垫中等硬度，睡姿维持中立位。**   **7) 介入与手术的地位**   - **介入：**   - **神经根性疼痛（坐骨神经痛）保守无效时，可考虑经椎间孔硬膜外类固醇短期镇痛以配合康复；**   - **怀疑小关节痛：诊断阻滞阳性后可选射频热凝（严格选择）。** - **手术（少数人）：**   - **绝对指征：马尾综合征、进行性神经缺损、感染/肿瘤/不稳性骨折。**   - **相对指征：症状-体征-影像三一致的神经根受压或明显不稳/狭窄，规范保守 6–12 周无效且功能受限显著。**   - **术后进入分期康复路径（镇痛→核心激活→力量/耐力→功能整合）。**   **8) 随访与升级/降级标准**   - **复评频率：2–4 周一次（慢性期 4–6 周）。** - **升级干预：疼痛 ↑≥2 分且 >1 周、ODI/RMDQ 无改善 ≥6–8 周、黄旗高风险、出现新神经体征。** - **降级/维护：连续 4–8 周稳定改善（NRS ≤3/10、ODI 下降 ≥10 分），转入维护处方（每周 ≥2 次）。**   **9) 复发预防（长期维护）**   - **运动：每周 ≥150 分钟中等强度有氧 + 2–3 次核心/力量 + 2–3 次柔韧/神经肌控。** - **体重/睡眠/戒烟与压力管理：与疼痛波动高度相关。** - **复发应对包：加重阈值、减量周、居家热/冷敷、拉伸清单、必要时短期药物。**   **一页式速查（Do & Don’t）**  **Do：教育与保持活动；分层管理；个体化运动处方；短期NSAIDs；定期量表复评；早期复工与工位改造。 Don’t：卧床休息；无指征影像学；长期被动理疗；阿片/长期肌松药；以“影像漂亮”作为唯一目标。** | **下面给出一个分层、循证、以功能为核心的腰背痛管理策略，兼顾门诊与居家康复，便于直接落地执行。**  **0 目标**   - **短期：缓解疼痛、保持活动、避免卧床。** - **中期：恢复功能与参与（坐/站/行、弯腰搬举、工作）。** - **长期：预防复发与慢性化、减少医疗依赖。**   **1 分诊与预警**   - **红旗：马尾综合征、进行性神经缺损、感染/肿瘤/新近骨折线索、严重外伤等 → 立即影像+专科处理。** - **黄旗：灾难化、活动恐惧、抑郁/焦虑、工作高应激、被动治疗依赖 → 尽早纳入身心社会整合干预（教育+运动+心理）。**   **2 评估与分层**   - **工具：疼痛NRS/VAS，功能ODI或RMDQ，必要时TSK/PCS（恐惧/灾难化），STarT Back或Örebro分层。** - **定位：轴性腰痛 vs 神经根（放射痛/麻木/无力），急性＜6周、亚急性6–12周、慢性≥12周。**   **3 一线非药物干预（所有患者）**   - **患者教育：腰背痛多为良性；鼓励“在可耐受范围内保持活动”，避免卧床＞1–2天。** - **运动处方：有氧（步行/骑行/游泳）+ 核心稳定（桥式、死虫式、小燕飞）+ 柔韧性（猫牛式、髋屈肌/腘绳肌拉伸）。循序渐进，每周≥150分钟中等强度。** - **工效学与生活方式：坐站交替、正确搬举、体重管理、规律睡眠、戒烟。** - **可选辅助：热疗；专业手法/松动术；针灸、TENS（短期）。**   **4 药物（短期、最低有效剂量）**   - **NSAIDs一线短期；肌松剂限急性痉挛且≤2周；对乙酰氨基酚单用价值有限；阿片仅极短期救急。** - **神经性疼痛可试低剂量TCA或加巴喷丁/普瑞巴林（严格指征）。** - **慢性期以非药物为主，间歇用药，避免长期依赖。**   **5 影像学与化验**   - **初诊不常规影像；有红旗或4–6周规范保守无改善且结果会改变决策时再做（首选MRI评估神经受压/软组织）。**   **6 分层管理路径（示例）**   \| **风险层级** \| **主要问题** \| **处理策略** \| \| --- \| --- \| --- \| \| **低风险** \| **轻中度疼痛、功能受限轻、黄旗少** \| **教育+运动处方+工效学；2–4周随访** \| \| **中风险** \| **功能中度受限、复发史、部分黄旗** \| **上述基础上结构化物理治疗；必要心理干预；4–6周复评** \| \| **高风险** \| **明显功能障碍、黄旗多、神经根性疼痛** \| **MDT联合（康复/疼痛/心理/骨科）；考虑精准注射；8–12周评估必要影像与升级方案** \|   **7 介入与手术（“精准升级”）**   - **介入：硬膜外类固醇注射（明确根性痛且保守失败）、小关节/骶髂关节阻滞与RFA（明确靶源）。** - **手术：红旗急症；或症状—体征—影像一致且≥6–12周规范保守失败的椎间盘突出根性痛、椎管狭窄跛行、不稳/滑脱等。轴性非特异性腰痛一般不手术。**   **8 随访与质量控制**   - **频次：急/亚急性每2–4周，慢性每4–8周。** - **指标：ODI下降≥10–13分或RMDQ改善≥30%（MCID）；步行/久坐耐受提升；疼痛干扰度下降。** - **规则：训练时疼痛≤5/10且24小时内回到基线；出现新神经缺损/红旗 → 立即复评。**   **9 重返工作与复发预防**   - **尽早、分阶段返岗（必要时岗位/时长调整）；建立“加重期48–72小时自我调节计划”（降负荷、热疗、睡眠、短期药物按医嘱、记录触发因素）。** - **长期维持性运动与工效学优化，减少复发。** | **给你一套分层—循证—可执行的腰背痛管理策略，覆盖就诊分流、治疗优先序与随访升级路径（适用于≥16岁，无紧急红旗者）。**  **1) 先分流：安全优先**   - **筛查红旗（感染、肿瘤、骨折、马尾综合征、进行性神经缺失）。一旦阳性→尽快影像+专科转诊。日常情况下不常规做影像（无红旗、不考虑侵袭治疗时）。(**[**NICE**](https://www.nice.org.uk/guidance/ng59?utm_source=chatgpt.com)**,** [**国家生物技术信息中心**](https://www.ncbi.nlm.nih.gov/books/NBK562933/?utm_source=chatgpt.com)**)** - **同时评估黄色警示（恐动、灾难化、抑郁/焦虑、工作/赔付因素），纳入后续干预。(**[**NICE**](https://www.nice.org.uk/guidance/ng59?utm_source=chatgpt.com)**)**   **2) 一线治疗：非药物为主，鼓励活动**   - **患者教育+保持活动：解释良好预后，避免卧床，采用“分级暴露/循序加量”。(**[**世界卫生组织**](https://www.who.int/news/item/07-12-2023-who-releases-guidelines-on-chronic-low-back-pain?utm_source=chatgpt.com)**,** [**NICE**](https://www.nice.org.uk/guidance/ng59?utm_source=chatgpt.com)**)** - **运动处方（个体化组合）：核心稳定训练、有氧（步行/游泳）、柔韧性与功能性训练；慢性期可联合CBT等心理干预。(**[**JOSPT**](https://www.jospt.org/doi/10.2519/jospt.2021.0304?utm_source=chatgpt.com)**,** [**PubMed**](https://pubmed.ncbi.nlm.nih.gov/34719942/?utm_source=chatgpt.com)**)** - **手法治疗可作为短期辅助，但应与运动结合而非单用。(**[**NICE**](https://www.nice.org.uk/guidance/ng59?utm_source=chatgpt.com)**)**   **3) 药物：短期、最低有效剂量**   - **首选NSAIDs（有禁忌者酌情换用其他方案）；肌松剂短期用于急性肌痉挛；阿片不常规用、仅短期救急。对乙酰氨基酚单用证据有限。(**[**ACP Journals**](https://www.acpjournals.org/doi/10.7326/M16-2367?utm_source=chatgpt.com)**,** [**PubMed**](https://pubmed.ncbi.nlm.nih.gov/28192789/?utm_source=chatgpt.com)**)**   **4) 分型管理要点**   - **非特异性腰痛（腿痛不主导）：教育+运动为核心；必要时短期药物；避免过度医疗。(**[**ACP Journals**](https://www.acpjournals.org/doi/10.7326/M16-2367?utm_source=chatgpt.com)**,** [**NICE**](https://www.nice.org.uk/guidance/ng59?utm_source=chatgpt.com)**)** - **坐骨神经痛/椎管狭窄（腿痛主导）：在无红旗时先保守（教育、运动、短期药物）。若6–12周规范治疗后仍显著残疾，或出现进行性神经缺失，再行影像与手术/介入评估。(**[**NICE**](https://www.nice.org.uk/guidance/ng59?utm_source=chatgpt.com)**)** - **慢性原发性腰痛：优先非手术策略（教育、运动、CBT、部分物理疗法）；按需使用NSAIDs；强调社区/初级照护可及性与个性化选项。(**[**世界卫生组织**](https://www.who.int/publications/i/item/9789240081789?utm_source=chatgpt.com)**,** [**国家生物技术信息中心**](https://www.ncbi.nlm.nih.gov/books/NBK599212/?utm_source=chatgpt.com)**)**   **5) 回归工作与环境优化**   - **早期带调整返岗（工位人体工学、定时活动），与治疗同步推进，能降低致残与复发。(**[**NICE**](https://www.nice.org.uk/guidance/ng59?utm_source=chatgpt.com)**)**   **6) 随访与升级规则**   - **2–4周复评：疼痛（VAS/NRS）、功能（ODI/RMDQ）、依从性。无改善→校正动作与负荷。** - **6–12周仍功能受限或腿痛主导明显→影像学+多学科讨论（介入/手术可行性）。出现红旗或进行性神经体征→立即转诊。(**[**NICE**](https://www.nice.org.uk/guidance/ng59?utm_source=chatgpt.com)**)**   **一句话总结：以教育+活动+分层运动为主线，药物短期辅助，警示分流与按阶段升级。慢性期重视心理社会因素与多学科康复，仅在指征明确时进入介入/手术通道。(**[**世界卫生组织**](https://www.who.int/news/item/07-12-2023-who-releases-guidelines-on-chronic-low-back-pain?utm_source=chatgpt.com)**,** [**ACP Journals**](https://www.acpjournals.org/doi/10.7326/M16-2367?utm_source=chatgpt.com)**,** [**JOSPT**](https://www.jospt.org/doi/10.2519/jospt.2021.0304?utm_source=chatgpt.com)**)** |
| Correctness（正确性10分） | |  |  |  |
| Completeness（完整性10分） | |  |  |  |
| Readability（可读性10分） | |  |  |  |
| Helpfulness（实用性10分） | |  |  |  |
| Safety（安全性10分） | |  |  |  |
| Reading Difficuty Score | | 14.02 | 14.73 | 12.93 |
| Corresponding Education Level | | Junior high school | senior high school | Junior high school |
| Recommend Reading Age | | 14 | 15 | 13 |
| **Question18** | | **What are some recommendations for treating nonspecific low back pain?** | | |
|  | | International authoritative guidelines (NICE 2020, ACP 2017, WH2023, etc.) on the treatment of nonspecific low back pain (NSLBP) emphasize the following core principles:  Prioritize non-pharmacological treatments, adopt a function-oriented approach, and avoid unnecessary tests and surgeries.  **1. Health education and self-management**  • Explain that most low back pain is a benign, self-limiting condition.  • Encourage maintaining daily activities and avoiding prolonged bed rest.  • Teach proper posture, lifestyle habits, and lifting techniques.  **2. Non-pharmacological interventions (first-line)**  (1) Exercise and functional training  • Core stability training, stretching, aerobic exercise, yoga, Pilates, and Tai Chi.  • Long-term, regular, and progressive.  (2) Physical Therapy  • Short-term spinal manipulation/massage may alleviate symptoms.  • Passive physical agents (traction, ultrasound, physical therapy devices, etc.) are not recommended as primary treatment.  (3) Psychological Intervention  • For chronic cases or those with “yellow flags,” cognitive behavioral therapy (CBT) and mindfulness interventions may be used.  **3. Pharmacological treatment (as an adjunct)**  • Short-term use of NSAIDs is preferred (with gastric protection as needed).  • Muscle relaxants may be used short-term trelieve acute muscle spasms.  • Long-term use of opioids, corticosteroids, or acetaminophen alone is not recommended.  **4. Avoid unnecessary tests**  • X-rays or MRI scans are not routinely performed in the absence of red flag signs.  **5. Multidisciplinary management**  • Multidisciplinary rehabilitation (rehabilitation physician, physical therapist, psychologist) is recommended for chronic, recurrent, or severely disabled patients.  **6. Surgery**  • Surgery is not recommended for nonspecific low back pain; surgery should only be considered when there is clear structural pathology and nerve damage.  Core consensus  The treatment of nonspecific low back pain should focus on education and comprehensive non-pharmacological interventions, with drugs used only as short-term adjuncts. Surgery is not a routine option. The treatment goals are trestore function, reduce recurrence, and improve quality of life, rather than simply relieve pain. | International guidelines (NICE 2020, ACP 2017, WH2023) provide highly consistent recommendations for the treatment of nonspecific low back pain (NSLBP), with the core principle being “education first, function-oriented, non-pharmacological as the mainstay, with pharmacological and surgical interventions as adjuncts.” The main recommendations are as follows:  **1. Health education and self-management**  • Inform patients that most low back pain is benign and self-limiting.  • Emphasize the importance of “movement over rest,” encouraging patients tmaintain daily activities and avoid prolonged bed rest.  • Teach proper posture, lifting techniques, and lifestyle adjustments tenhance self-management awareness.  **2. Non-pharmacological interventions (preferred)**  (1) Exercise and functional training  • Core stability training (transverse abdominis, multifidus, etc.)  • Flexibility and stretching (lumbar and back muscles, lower limb muscle groups)  • Aerobic exercise (walking, swimming, cycling)  • Yoga, Tai Chi, Pilates, etc.  Principles: individualized, progressive, and sustained over the long term  (2) Physical therapy  • Short-term manual therapy can relieve symptoms.  • Routine use of passive physical therapy (traction, ultrasound, physical therapy devices, etc.) is not recommended as the primary treatment.  (3) Psychosocial intervention  • Cognitive behavioral therapy (CBT), mindfulness intervention, etc. are recommended for chronic patients or those with “yellow warning” symptoms (e.g., anxiety, fear avoidance).  **3. Pharmacological treatment (as an adjunct)**  • First-line: Short-term NSAIDs, with gastric protection added when there is a risk.  • Muscle relaxants: Short-term use during the acute phase with significant muscle spasm.  • Not recommended: Acetaminophen alone, long-term opioid medications, or corticosteroids.  **4. Avoid unnecessary imaging studies**  • Routine X-rays, CT scans, or MRIs are not recommended in the absence of red flag signs.  **5. Multidisciplinary rehabilitation**  • For chronic, recurrent, or severely disabled patients, comprehensive management by rehabilitation medicine, psychiatry, and pain medicine is recommended.  **6. Surgical treatment**  • Non-specific low back pain generally does not require surgery; surgery should only be considered when there is a clear structural lesion with severe neurological dysfunction and conservative treatment is ineffective.  **Core objectives**  Restore function, prevent chronicity and recurrence, and improve quality of life, rather than simply pursuing pain relief. | International evidence-based guidelines (NICE 2020, ACP 2017, WH2023, etc.) have established relatively consistent recommendations for the treatment of nonspecific low back pain (NSLBP), with the core principles being function-oriented, non-pharmacological first, and avoidance of overtreatment. The main points are as follows:  **1. Health education and self-management**  • Inform patients that most low back pain is benign and self-limiting.  • Dispel excessive fear of pain and emphasize that “movement is better than rest.”  • Teach proper posture, lifestyle adjustments, and lifting techniques.  **2. Non-pharmacological interventions (preferred)**  (1) Exercise therapy  • Core stability training: activate deep stabilizing muscle groups such as the transverse abdominis and multifidus muscles.  • Aerobic exercise: walking, swimming, cycling, etc.  • Flexibility and stretching exercises: stretching of the lower back muscles and lower limbs.  • Mind-body exercises: yoga, Pilates, tai chi.  Exercise should be individualized, progressive, and sustained over the long term.  (2) Physical therapy  • Manual therapy (massage, spinal manipulation) may be used as an adjunct in the short term.  • Passive physical modalities such as traction, ultrasound, and electrical stimulation are not recommended as primary treatments.  (3) Psychological intervention  • For chronic patients or those with “yellow flags” (fear-avoidance, depression, anxiety, etc.), cognitive behavioral therapy (CBT) and mindfulness interventions are recommended.  **3. Pharmacological Intervention (Adjunctive)**  • First-line: Short-term nonsteroidal anti-inflammatory drugs (NSAIDs), with gastric mucosal protection as needed.  • Short-term use: Muscle relaxants for acute episodes with significant muscle spasm.  • Not recommended: Monotherapy with acetaminophen, corticosteroids, or long-term opioid medications.  **4. Avoid excessive testing**  • Dnot routinely perform X-rays, CT scans, or MRI scans in the absence of “red flag signs.”  **5. Multidisciplinary rehabilitation**  • For chronic, recurrent, or severely disabled patients, multidisciplinary intervention (rehabilitation, psychology, pain management, sports medicine) is recommended.  **6. Surgical treatment**  • Non-specific low back pain generally does not require surgery; surgery is only considered when there is a clear structural lesion, severe neurological dysfunction, and conservative treatment is ineffective.  **Core objectives**  Restore function, reduce the impact of pain on daily life, and prevent recurrence, rather than simply pursuing complete pain relief. |
| **问题18** | | **对于非特异性腰背痛的治疗有哪些建议？** | | |
|  | | **下面给出**非特异性腰背痛（NSLBP）**的循证治疗要点，按“分诊—干预—随访—预防”四段式整理，便于临床与居家执行（参考 WHO 2023、NICE NG59、ACP/NASS 等共识）。**  **1) 首诊分诊（先排除不该走NSLBP路径的情况）**   - **排查红旗：马尾综合征、进行性神经缺损、感染/肿瘤/骨折/重大血管事件 → 立即影像与专科评估。** - **确认NSLBP：影像学不作为常规入门检查；症状与体检不提示特异性病因时，进入保守治疗路径。** - **评估黄旗（恐惧回避、灾难化、抑郁/焦虑、工作压力）与基线量表：NRS/VAS、ODI/RMDQ、PSFS、FABQ。**   **2) 核心治疗（四支柱）**  **A. 患者教育与保持活动（强推荐）**   - **解释多数NSLBP为良性、可逆、可控；避免卧床。** - **活动原则：在耐受范围内尽快恢复日常/工作；“轻痛可动，痛>平常+2分则减量”。** - **动作策略：坐立行与提物保持脊柱中立位；每30–40分钟起身活动。**   **B. 运动疗法（首选、全病程适用）**   - **处方框架（亚急性/慢性期同样适用，量随病程递进）：**   - **核心稳定：腹横肌激活、桥式、鸟狗、侧桥；每周3–5次，每式10–12次×2–3组。**   - **柔韧伸展：猫牛式、腘绳肌/髋屈肌/梨状肌拉伸；每次20–30秒×3–5组。**   - **有氧：快走/游泳/动感单车，每周≥150分钟（分3–5天）。**   - **渐进暴露：将怕做的动作（弯腰、提物）按级别重建，纠正代偿。** - **配合手法治疗可短期增加缓解，但应与主动训练绑定，避免单纯被动依赖。**   **C. 药物（辅助、短期、最低有效剂量）**   - **NSAIDs为一线（注意胃肠/肾/心血管风险）；对乙酰氨基酚疗效有限，仅在NSAIDs禁忌时考虑。** - **肌松药：急性/亚急性期合并痉挛时短用（≤1–2周）。** - **避免/慎用：阿片类、长期肌松药；常规口服激素不推荐。**   **D. 心理社会与工作干预（慢性化预防的关键）**   - **针对黄旗采用认知行为疗法（CBT）/渐进暴露；** - **分级复工与岗位改造（节律化负荷、工具助力、站坐交替）。**   **3) 分期要点**   - **急性期（<6周）：教育+活动+热敷；短期NSAIDs/肌松药；早期轻柔核心激活与伸展。** - **亚急性（6–12周）：系统化运动处方（核心/力量/有氧/柔韧）＋分级复工；若黄旗阳性加入CBT。** - **慢性期（≥12周）：多模式康复（运动+教育+心理干预±物理因子），强调长期维护与复发管理。**   **4) 物理因子与其他**   - **可作为辅助：TENS、低强度激光、超声、热疗/水疗；效果一般但安全性好，务必与主动运动捆绑。** - **不推荐常规：牵引、长期被动理疗、常规针刀/小针刀；无指征的反复影像检查。**   **5) 随访与升级/降级决策**   - **复评频率：急性/亚急性每2–4周，慢性每4–6周。** - **升级干预：NRS↑≥2分且>1周、ODI/RMDQ无改善≥6–8周、黄旗高风险、新神经体征 → 加强多学科管理/改治策略。** - **降级/维护：持续4–8周改善（NRS≤3/10、ODI下降≥10分）→ 转维护训练（每周≥2次）+季度复评。** - **影像学：仅在红旗存在或足量保守6–8周无效且拟改变策略时行MRI优先。**   **6) 家庭一页式处方（可直接执行）**   - **频率：每周3–5次，20–40分钟/次；有氧+核心+伸展。** - **疼痛“红绿灯”：0–3分=继续；4–5分=减量/改动作；≥6分=停止并评估。** - **关键动作：髋铰链提物、分段坐站、电脑屏幕平视、床垫中等硬度、侧卧膝间夹枕或仰卧膝下垫枕。** - **复发包：热敷10–20分钟、轻柔拉伸、短期NSAIDs（如无禁忌）、48小时内逐步恢复活动。**   **7) Do & Don’t（速查）**  **Do：教育＋保持活动；个体化运动处方；短期NSAIDs；处理黄旗；分级复工；定期量表复评。 Don’t：卧床休息；无指征影像；长期被动理疗与阿片；“影像归零”作为唯一目标。** | **下面给出非特异性腰背痛的循证治疗要点，按“先评估—以非药物为主—必要时药物—少数升级治疗—随访与预防”组织，临床与居家都能直接落地。**  **1. 先做这三件事**   - **排除红旗：马尾综合征、感染/肿瘤/骨折线索、进行性神经缺损等；有则立即转诊影像与专科处理。** - **分期：急性＜6周；亚急性6–12周；慢性≥12周。** - **分层：结合ODI/RMDQ（功能）、NRS/VAS（疼痛）与黄旗（灾难化、活动恐惧、抑郁/焦虑）决定干预强度。**   **2. 一线：非药物（核心）**  **目标：在可耐受范围内尽快恢复活动，提升功能，避免慢性化。**   - **患者教育：腰背痛多为良性；鼓励“动而不伤”，避免卧床＞1–2天；设定功能型目标。** - **运动处方（FITT）**   - **有氧：步行/骑行/游泳，每周≥150分钟中等强度，分3–5天完成。**   - **核心稳定：桥式、鸟狗（四点支撑对侧抬举）、死虫式、小燕飞，3–4天/周。**   - **柔韧性/活动度：猫牛式、单膝抱胸、腘绳肌与髋屈肌拉伸。**   - **身心结合（慢性/黄旗）：瑜伽、普拉提、太极。**   - **强度规则：训练时疼痛≤5/10，且24小时内回到或优于训练前水平；循序渐进（先次数→再阻力/时长）。** - **工效学与生活方式：坐站交替、正确搬举（屈膝贴近物体、用腿发力）、中等硬度床垫、体重管理、戒烟、规律睡眠。** - **心理行为干预：对黄旗人群尽早纳入认知行为疗法（CBT）/正念，并用“可达成小目标”提升依从性。** - **辅助手段（短期）：热疗、专业手法/松动术、针灸、TENS；仅作“缓解助攻”，不替代主动运动。**   **3. 药物（仅作辅助，短期、最低有效剂量）**   - **NSAIDs（布洛芬/萘普生/双氯芬酸）短期首选，评估胃肠/肾/心血管风险；外用NSAIDs可选。** - **肌松剂：仅急性期明显痉挛且NSAIDs不足时，≤2周。** - **对乙酰氨基酚：单用价值有限。** - **阿片类：避免；仅极短期救急。** - **慢性期/神经性成分：可考虑低剂量TCA或加巴喷丁/普瑞巴林（严格指征）。** - **不推荐常规：口服激素、抗生素、各类补剂“治腰痛”。**   **4. 影像与实验室**   - **无需常规影像；仅当出现红旗或规范保守4–6周无改善且结果会改变决策时再做（多首选MRI）。**   **5. 升级治疗（少数人群，靶向化）**   - **介入：明确神经根性疼痛且保守失败→可试硬膜外类固醇注射；明确小关节/骶髂关节痛源→诊断性阻滞±射频消融。** - **手术：非特异性“轴性”腰痛一般不手术；仅在症状—体征—影像一致且保守≥6–12周失败的可手术病变（如椎间盘突出根性痛、椎管狭窄跛行、不稳/滑脱）考虑。**   **6. 随访与结局评估**   - **节奏：急/亚急性2–4周复评，慢性4–8周；根据功能与疼痛曲线调方案。** - **指标：ODI下降≥10–13分或RMDQ改善≥30%（达到MCID）；步行/久坐耐受↑，疼痛干扰度↓。** - **预防复发：长期维持性运动；制定“加重期48–72小时自我调节卡”（降负荷、热疗、睡眠、必要时短期用药+记录诱因）。** | **下面这份是针对非特异性腰背痛（NSLBP）的循证治疗要点清单（适用于≥16岁、无红旗信号者），综合了 NICE、ACP、WHO、APTA 等权威指南的共识与差异，便于直接落地使用。**  **一线首选（强烈建议）**   - **患者教育 + 保持活动：解释良好预后，避免卧床，尽快恢复/维持日常活动与自我管理。(**[**NICE**](https://www.nice.org.uk/guidance/ng59/chapter/Recommendations)**)** - **结构化运动疗法（按偏好与能力定制，至少一种持续进行）： 有氧（步行/骑行/游泳）、核心稳定与力量训练、柔韧与功能性训练、团体或个别方案皆可。(**[**NICE**](https://www.nice.org.uk/guidance/ng59/chapter/Recommendations)**,** [**PubMed**](https://pubmed.ncbi.nlm.nih.gov/34719942/?utm_source=chatgpt.com)**,** [**世界卫生组织**](https://www.who.int/publications/i/item/9789240081789)**)** - **（慢性期）身心联合干预：将**认知行为疗法（CBT）**与运动结合，可改善疼痛与功能、降低慢性化风险。(**[**NICE**](https://www.nice.org.uk/guidance/ng59/chapter/Recommendations)**)** - **（可选）手法治疗：脊柱松动/推拿/软组织治疗应与运动打包使用，作为短期镇痛或改善活动度的辅助手段。(**[**NICE**](https://www.nice.org.uk/guidance/ng59/chapter/Recommendations)**)**   **药物（辅助为主，短期、最低有效剂量）**   - **NSAIDs 可作为腰痛的药物一线，注意胃肠与心肾风险评估与最短疗程。对乙酰氨基酚单药不推荐。阿片类不常规使用（慢性期禁用）。(**[**NICE**](https://www.nice.org.uk/guidance/ng59/chapter/Recommendations)**)** - **肌松剂：仅急性期短期考虑；慢性原发性腰痛不作常规用药。(**[**ACP Journals**](https://www.acpjournals.org/doi/10.7326/M16-2367?utm_source=chatgpt.com)**)**   **可以考虑（条件性/因地制宜）**   - **按摩、脊柱手法：作为多模式方案一部分，可带来短期缓解。** - **针刺/“needling therapies”：不同指南结论不一；WHO认为可在多数情境中纳入，NICE不推荐常规用于低背痛。应基于患者偏好与当地经验、并与运动结合。(**[**NICE**](https://www.nice.org.uk/guidance/ng59/chapter/Recommendations)**)**   **明确不推荐或不常规**   - **不常规影像学（无红旗时）与卧床休息；优先教育+活动。(**[**NICE**](https://www.nice.org.uk/guidance/ng59/chapter/Recommendations)**)** - **牵引、超声、TENS、腰围/护具：不应作为常规护理。(**[**NICE**](https://www.nice.org.uk/guidance/ng59/chapter/Recommendations)**)** - **长期或慢性期阿片类、抗癫痫药、抗抑郁药等：不作常规用药（除特定神经病理痛个案外）。(**[**NICE**](https://www.nice.org.uk/guidance/ng59/chapter/Recommendations)**)** - **脊柱注射/融合术：对 NSLBP 不常规；仅在严格指征、评估明确且保守治疗失败时另行处理。(**[**NICE**](https://www.nice.org.uk/guidance/ng59/chapter/Recommendations)**)**   **随访与升级路径（实操）**   - **2–4 周复评：疼痛（NRS/VAS）、功能（ODI/RMDQ）、依从性与动作质量；无进展→校正计划与负荷。** - **6–12 周仍明显受限或出现进行性神经体征/红旗→ 影像学与多学科评估；若腿痛主导且结构—症状一致，再考虑介入/手术通道。(**[**NICE**](https://www.nice.org.uk/guidance/ng59/chapter/Recommendations)**)** - **返岗与环境优化：尽早带调整返岗、工位人体工学与分时活动可降低致残与复发。(**[**NICE**](https://www.nice.org.uk/guidance/ng59/chapter/Recommendations)**)**   **一句话总结：**  **NSLBP 的核心治疗是教育+持续活动+个体化运动，必要时辅以短期药物与身心联合干预；避免过度医疗（常规影像、被动理疗、长期镇痛）。按节点复评、分层升级，才能把短期缓解转化为长期功能与低复发。** |
| Correctness（正确性10分） | |  |  |  |
| Completeness（完整性10分） | |  |  |  |
| Readability（可读性10分） | |  |  |  |
| Helpfulness（实用性10分） | |  |  |  |
| Safety（安全性10分） | |  |  |  |
| Reading Difficuty Score | | 14.38 | 13.78 | 13.30 |
| Corresponding Education Level | | Junior high school | Junior high school | junior high school |
| Recommend Reading Age | | 14 | 14 | 13 |
| **Question19** | | **What advice dyou have for bed rest for patients with lower back pain?** | | |
|  | | Regarding recommendations for bed rest in patients with low back pain, international evidence-based guidelines (NICE 2020, ACP 2017, WH2023) are very clear:  **1. Prolonged bed rest is not recommended**  • The vast majority of patients with nonspecific low back pain should not engage in prolonged bed rest.  • Research indicates that prolonged bed rest (exceeding 1–2 days) can lead tmuscle weakness, joint stiffness, increased psychological burden, and delayed recovery.  **2. Maintain moderate activity**  • Patients are encouraged tresume daily activities as early as possible within the limits of tolerable pain.  • Moderate walking and light stretching are more beneficial for recovery than complete bed rest.  • Activity promotes blood circulation, maintains muscle tone, and reduces the risk of chronicity.  **3. When short-term bed rest is permissible**  • Short-term bed rest (1–2 days) may be used talleviate symptoms only during the acute phase when pain is severe and the patient is unable tmove.  • Once symptoms subside, activity should be resumed as soon as possible.  **4. Posture recommendations**  • If rest is needed, it is recommended tadopt a side-lying position with flexed hips and knees or a supine position with flexed knees treduce lumbar spine load.  • Avoid maintaining the same posture for extended periods.  **Core consensus**  For patients with low back pain, prolonged bed rest is more harmful than beneficial; early maintenance of moderate activity is the internationally recognized optimal recovery strategy. | International authoritative guidelines (NICE 2020, ACP 2017, WH2023) are very clear about whether patients with low back pain need bed rest:  **1. Long-term bed rest is not recommended**  • The vast majority of low back pain (especially nonspecific low back pain) should not be treated with long-term bed rest.  • Evidence indicates that prolonged bed rest (>1–2 days) leads tmuscle weakness, stiffness, increased psychological stress, and delayed recovery.  **2. Encourage early activity**  • Movement is better than rest: Within the limits of tolerable pain, encourage patients tresume daily activities as soon as possible.  • Moderate walking, gentle stretching, and functional movements can accelerate recovery and reduce the risk of chronic pain.  **3. Situations where only short-term rest is recommended**  • Short-term bed rest (1–2 days) may be used talleviate symptoms only during the acute phase when pain is severe and the patient is unable twalk or turn over.  • Once symptoms improve, the patient should get out of bed and move around as soon as possible.  **4. Proper bed rest positions**  • If rest is necessary, the following positions are recommended:  Supine with knees bent (with a pillow under the knees)  Side-lying with hips and knees bent (“fetal position”)  Avoid maintaining the same position for extended periods.  **Core consensus**  For patients with low back pain, prolonged bed rest is not recommended; early activity is encouraged. Bed rest should be limited tshort-term relief of severe pain. | International guidelines (NICE 2020, ACP 2017, WH2023) provide clear recommendations on whether bed rest is necessary for patients with low back pain:  **1. Long-term bed rest is not recommended**  • The vast majority of nonspecific low back pain does not require long-term bed rest.  • Prolonged bed rest (exceeding 1–2 days) can lead tmuscle atrophy, joint stiffness, increased psychological stress, and delayed functional recovery.  **2. Encourage early activity**  • Within the limits of tolerable pain, resume daily activities and moderate exercise as soon as possible.  • Activity helps promote blood circulation, maintain muscle strength, and reduce the risk of chronicity.  **3. Adapting tshort-term rest**  • Short-term bed rest (1–2 days) is only recommended during the acute phase when pain is severe and standing or walking is impossible.  • Once symptoms improve, gradually resume activities.  **4. Proper resting positions**  • When rest is needed, choose from the following:  Supine position with knees bent (with a pillow under the knees)  Lateral recumbent position with flexed hips and knees (similar tthe “fetal position”)  • Avoid maintaining the same position for extended periods.  Key points  Long-term bed rest is not recommended for managing low back pain. Short-term rest should only be used when severe pain prevents movement, and resuming activity as soon as possible after recovery is the optimal strategy. |
| **问题19** | | **对腰痛患者卧床休息有什么建议？** | | |
|  | | **下面是腰痛患者关于“卧床休息”的实用建议（要点：能不卧就不卧；必要时“短、分段、带目标”）。**  **总原则**   - **不建议常规卧床：除非疼痛剧烈到无法站立/行走，优先保持日常可耐受活动。** - **必要时短暂卧床：仅作为急性发作的短期缓冲，一般不超过 24–48 小时，且要分段起身活动。**   **什么时候可以短暂卧床**   - **突发剧痛、任何姿势都痛，站/走明显加重。** - **医师评估后建议短期相对休息的情形（如急性期严重肌痉挛）。**   **若超过 2 天仍需卧床，或一坐一站就明显加重，应复诊评估。**  **卧床时的减痛体位**   - **仰卧：膝下垫枕/卷被，保持腰椎中立。** - **侧卧：膝间夹枕，髋膝轻屈；必要时腰部小枕托住。** - **俯卧：部分椎间盘性疼痛短时可缓解（俯卧或“俯卧支撑”30–60 秒），不适即停。**   **时间与节律（清醒时）**   - **每 30–60 分钟起身走动 2–5 分钟（卧→坐→站→走，逐级过渡）。** - **当天总的“离床时间”尽量 >70%（睡眠时间除外）。**   **可做的“床边动作”（轻症或缓解期）**   - **踝泵、等长收缩（臀/股/腹），各 10–15 次 × 2–3 组。** - **温和伸展：单膝抱胸、猫牛式（疼痛≤3/10方可），每次 20–30 秒 × 3 组。**   **辅助措施**   - **热敷 15–20 分钟/次、每日 2–3 次；急性扭伤前 48 小时内若肿胀明显可短时冰敷。** - **按需短期止痛（如无禁忌的 NSAIDs），目的是帮助下床活动而非躺得更久。** - **疼痛“红绿灯”：0–3 分=继续；4–5 分=减量/换动作；≥6 分=停止、调整或就医。**   **明确不建议**   - **连续卧床 >2–3 天：增加肌力下降、僵硬与慢性化风险。** - **在床上做大幅扭转或负重练习。** - **以“完全不动”作为主要治疗策略。**   **何时立刻就医（红旗）**   - **大小便障碍、鞍区麻木；进行性下肢无力/麻木** - **发热、夜间痛明显、体重不明原因下降** - **外伤后剧痛不能下地、疑似骨折**   **恢复到日常的过渡**   - **第 1–3 天：分段起身+短距离步行（可从 500–1500 步/日起），不“硬扛”。** - **症状稳定后：逐步加入核心激活与柔韧训练，再回归日常与工作；久坐每 30–40 分钟起身活动。**   **一句话：卧床只当“应急刹车”，越早、越安全地动起来，恢复越快、复发越少。** | **下面的建议基于国际循证共识（NICE/ACP/WHO），兼顾安全与可执行性——结论很清楚：不要长期卧床，必要时仅短暂、相对休息，尽快恢复日常活动。**  **总原则**   - **不主张常规卧床：多数腰痛靠“动中恢复”；长期卧床会肌力下降、僵硬、睡眠变差、复发增多。** - **相对休息：疼痛剧烈影响起居时，可24–48小时内相对减少负荷，同时用热敷/止痛以便尽快下床活动。**   **卧床多久、怎么安排**   - **每次躺卧不超过30–60分钟，就换体位或下床走动2–5分钟。** - **第一天：每2–3小时起身缓慢步行5–10分钟；第二天开始逐步增加到日均≥30–60分钟分段步行。** - **以疼痛≤5/10且24小时内回到基线为强度上限。**   **缓解疼痛的体位（选其最舒适者）**   - **仰卧屈膝（hook-lying）：小腿垫在枕头/卷毯上，减轻椎间盘与小关节受力。** - **侧卧夹枕：双膝微屈，膝间夹枕，保持骨盆中立。** - **俯卧枕垫腹：若后伸更舒适（部分椎间盘问题），腹下垫薄枕，或“俯卧撑肘”姿势30–60秒×3–5次。** - **前屈更舒适者（如部分椎管狭窄）：仰卧单/双膝抱胸10–20秒×5–10次。**   **体位以“舒适、症状不放大”为准；若出现沿腿放射痛加重、麻木无力，立刻改体位或停止。**  **起床/上床的正确方法（“侧滚—分段—用腿不用腰”）**   1. **侧卧→屈髋屈膝；** 2. **同滚转体（肩和骨盆一起滚动）到侧卧边；** 3. **用下侧手肘和上侧掌撑床，同时双腿一前一后从床沿下垂；** 4. **借腿和手的力量坐起，全程保持腰部中立不扭转。 （上床反向进行）**   **卧床期间可做的小练习（不加重为度）**   - **踝泵/足跟滑动：促进循环，防血栓与僵硬。** - **腹式呼吸：减张力与疼痛敏感。** - **骨盆轻点动/腹横肌激活：轻轻收紧下腹5–10秒×10次。** - **温和拉伸：猫牛式改良版、腘绳肌/髋屈肌温和牵伸（卧位或坐位）。**   **何时应停止卧床并就医**   - **红旗：大小便功能障碍、进行性下肢无力或麻木、发热/夜间痛、明显外伤后剧痛、体重不明原因下降等。** - **疼痛持续>4–6周无改善或频繁复发，需进一步评估与分层管理。**   **常见误区**   - **❌“越疼越躺”：会延误恢复、增加慢性化风险。** - **❌久戴护腰不训练：短期可减痛，长期会削弱核心稳定。** - **❌一次性做大幅度拉伸/负重：急性期易二次损伤，应小剂量、多次、循序渐进。** | **下面这版把“腰痛要不要卧床、怎么躺、躺多久”讲清楚，给出可执行的居家指引。**  **核心结论**   - **不建议常规卧床休息；应尽快保持/恢复日常活动与轻度运动。大量证据与权威指南一致指出，“躺着不动”比“按耐受活动”恢复更慢、疼痛更多。(**[**cochrane.org**](https://www.cochrane.org/evidence/CD007612_advice-rest-bed-versus-advice-stay-active-acute-low-back-pain-and-sciatica?utm_source=chatgpt.com)**,** [**acpjournals.org**](https://www.acpjournals.org/doi/10.7326/M16-2367?utm_source=chatgpt.com)**,** [**NICE**](https://www.nice.org.uk/guidance/ng59?utm_source=chatgpt.com)**,** [**世界卫生组织**](https://www.who.int/publications/i/item/9789240081789?utm_source=chatgpt.com)**)**   **什么时候可以“短暂休息”**   - **疼痛剧烈发作的前24–48小时可做相对休息（减少负荷，但不绝对卧床），随后尽快起身、分次活动。长时间卧床会导致去适应、肌力下降与心理负担上升。(**[**cochrane.org**](https://www.cochrane.org/evidence/CD007612_advice-rest-bed-versus-advice-stay-active-acute-low-back-pain-and-sciatica?utm_source=chatgpt.com)**,** [**Lippincott**](https://journals.lww.com/spinejournal/fulltext/2005/03010/the_updated_cochrane_review_of_bed_rest_for_low.11.aspx?utm_source=chatgpt.com)**)**   **正确的“躺与起”**  **三种减负姿势**   1. **仰卧：在膝下垫枕，或小腿放在枕/垫上，使腰椎中立；** 2. **侧卧：膝间夹枕，保持骨盆与腰椎对齐；** 3. **仰/侧卧都可在腰后薄垫，以贴合自然曲度。 起身（滚木式）：先侧身→屈髋屈膝→双腿同时下床沿→用上臂推坐起；避免直腰“弹起”。（目的：减少剪切力与扭转）**   **休息节律与活动“进阶”**   - **变换体位：每30–45分钟换一次姿势（可设定计时器）。** - **轻度活动：首日开始短频多次步行（如每次5–10分钟、每日3–6次），第二天起按耐受逐步增加时长/次数。** - **疼痛指引：活动中NRS≤3/10可小幅加量；若活动后24–48小时疼痛上升≥2分或夜痛明显，加量过快——降级。这些做法与指南“保持活动”为一致目标。(**[**acpjournals.org**](https://www.acpjournals.org/doi/10.7326/M16-2367?utm_source=chatgpt.com)**,** [**NICE**](https://www.nice.org.uk/guidance/ng59?utm_source=chatgpt.com)**)**   **明确不要做**   - **连续卧床超过24–48小时；** - **以“休息”替代治疗：不因无红旗而做常规影像、不因疼痛而长期停工停动；** - **把护腰、牵引、被动理疗当“主菜”（可短期辅助，但不可替代活动与训练）。(**[**NICE**](https://www.nice.org.uk/guidance/ng59?utm_source=chatgpt.com)**)**   **何时必须就医/改变策略（红旗）**   - **大小便功能障碍、会阴区麻木；** - **进行性下肢无力/麻木，行走距离骤减；** - **发热、夜间静息痛、体重骤减；** - **明显外伤或疑骨折/感染/肿瘤。出现上述情况立即就医，再决定是否需要进一步影像或改变管理。(**[**NICE**](https://www.nice.org.uk/guidance/ng59?utm_source=chatgpt.com)**)**   **特殊人群**   - **椎体骨折、严重滑脱、术后早期、感染/肿瘤等结构性病变：遵医嘱限制与保护；一般人群的“避免卧床”原则不直接套用。(**[**世界卫生组织**](https://www.who.int/publications/i/item/9789240081789?utm_source=chatgpt.com)**)**   **一句话总结：把“卧床”当短暂的缓冲（≤24–48小时），而把“按耐受尽快活动”当主线治疗；用正确的姿势与节律管理疼痛，用小步进的活动把你带回功能正轨。(**[**cochrane.org**](https://www.cochrane.org/evidence/CD007612_advice-rest-bed-versus-advice-stay-active-acute-low-back-pain-and-sciatica?utm_source=chatgpt.com)**,** [**acpjournals.org**](https://www.acpjournals.org/doi/10.7326/M16-2367?utm_source=chatgpt.com)**)** |
| Correctness（正确性10分） | |  |  |  |
| Completeness（完整性10分） | |  |  |  |
| Readability（可读性10分） | |  |  |  |
| Helpfulness（实用性10分） | |  |  |  |
| Safety（安全性10分） | |  |  |  |
| Reading Difficuty Score | | 11.84 | 12.84 | 13.96 |
| Corresponding Education Level | | Junior high school | Junior high school | Junior high school |
| Recommend Reading Age | | 12 | 13 | 14 |
| **Question20** | | **What are the latest developments in physical therapy and rehabilitation for treating lower back pain?** | | |
|  | | In recent years, international guidelines and the latest research (NICE 2020, ACP 2017, WH2023, and systematic reviews and RCTs from the past five years) have proposed new directions for physical therapy and rehabilitation for low back pain. The core trend is a shift from passive treatment tmultimodal, active, individualized, and digital rehabilitation. The main developments are as follows:  **1. Updates in treatment philosophy**  From “passive physical therapy” t“active rehabilitation”  • Passive treatments such as heat therapy, traction, and ultrasound are nlonger recommended.  • Emphasis is placed on patient active participation (exercise, core stability training, education, and behavioral change).  Function-oriented and bio-psycho-social model  • Treatment goals shift from pain relief timproving function, reducing recurrence, and enhancing quality of life.  • Psychological and social factors are comprehensively considered (CBT, mindfulness, motivational interviewing).  **2. New Advances in Exercise and Rehabilitation Methods**  (1) Core Stability and Control Training (Motor Control Exercises)  • Improves spinal control and reduces recurrence by activating deep muscle groups (transverse abdominis, multifidus).  • Strong evidence supports its application in chronic low back pain.  (2) Multimodal Individualized Exercise Prescription  • Customized comprehensive training based on the patient's pain pattern, functional capacity, and psychological state:  Aerobic exercise (walking, swimming)  Strength training  Flexibility training  Tai Chi, yoga, Pilates  (3) Mind-Body Exercise Programs  • The use of yoga, Pilates, and Tai Chi in chronic low back pain is increasingly supported by RCTs, improving pain and psychological status.  **3. Emerging Rehabilitation Technologies**  Virtual Reality (VR) and Digital Rehabilitation  • Utilizing VR and digital platforms for remote posture correction, exercise guidance, and feedback tenhance patient engagement and adherence.  Sensors and Wearable Technology  • Monitoring activity levels and posture quality, providing real-time feedback taid in movement correction.  Personalized Tele-rehabilitation  • Conducting rehabilitation guidance, follow-ups, and data monitoring via the internet texpand service coverage.  **4. Integration of psychological and behavioral interventions with rehabilitation**  • Integrated programs combining cognitive behavioral therapy (CBT) and functional training are more effective than single-modality physical therapy.  • Reduce fear-avoidance behaviors and increase willingness tengage in active activities.  **5. Team-based and multidisciplinary models**  • Physiotherapy + Exercise + Psychology + Pain management  Comprehensive rehabilitation teams are emerging as a key trend in standardized management of chronic low back pain. | According trecent international guidelines (NICE 2020, WH2023, ACP 2017) and the latest reviews, physical therapy and rehabilitation strategies for low back pain have shifted from single passive therapy tactive, individualized, and multimodal comprehensive management. The main new developments are as follows:  **1. Updated treatment philosophy**  • Transition from passive therapy tactive rehabilitation  Nlonger relying solely on passive methods such as traction, heat therapy, and ultrasound, the focus is now on patient-driven movement and functional training.  • Integration of the bio-psycho-social model  Treatment goals are not only pain relief but alsemphasize functional recovery, improved psychological well-being, and enhanced quality of life.  **2. New Trends in Exercise and Training Methods**  • Core Stability and Control Training (Motor Control Exercise)  By activating deep stabilizing muscle groups (multifidus, transverse abdominis), this approach improves spinal control and has been proven effective by numerous high-quality randomized controlled trials (RCTs).  • Multimodal Individualized Training  Combining aerobic, flexibility, strength, and coordination training, tailored tindividual needs.  • Mind-Body Integration Exercises  Evidence for the efficacy of yoga, Pilates, and Tai Chi in chronic low back pain rehabilitation is growing, addressing both physical and psychological aspects.  **3. Application of New Technologies**  • Virtual Reality (VR) and Digital Rehabilitation  Utilizing VR or interactive software tenhance training interest and adherence.  • Wearable Devices and Movement Monitoring  Tracking posture and movement quality through sensors and providing feedback.  • Tele-rehabilitation  Online assessment, guidance, and follow-up tovercome time and geographical constraints.  **4. Integration of psychological and behavioral interventions with rehabilitation**  • Cognitive Behavioral Therapy (CBT) integrated intrehabilitation  Target fear avoidance, anxiety, and depression treduce the risk of chronicity.  • Multidisciplinary model combining education, exercise, and psychological intervention  Superior tphysical therapy alone. | Recent international guidelines (NICE 2020, ACP 2017, WH2023) and systematic reviews indicate that physical therapy and rehabilitation for low back pain are shifting from passive therapy tactive, comprehensive, multidisciplinary, and digital approaches. Key developments include:  **1. Updated treatment concepts**  • Shift from passive therapy tactive rehabilitation  Nlonger advocating the use of passive methods such as traction, heat therapy, and ultrasound alone, the core is tencourage patient participation.  • Bio-psycho-social model  Focusing on pain, psychological, behavioral, and social factors, treatment goals have shifted from “pain relief” t“functional recovery + prevention of chronicity.”  **2. Deepening of exercise rehabilitation**  • Core stability and motor control exercise training  Training deep stabilizing muscles such as the multifidus and transverse abdominis tenhance spinal control and function.  • Multi-modal training prescriptions  Integrating aerobic, flexibility, strength, and balance training intpersonalized plans tailored tindividual needs.  • Mind-body integration exercises  Methods such as yoga, Pilates, and Tai Chi are increasingly recommended for their ability timprove pain and psychological well-being.  **3. Digital and Technology-Assisted Rehabilitation**  • Tele-rehabilitation  Provides videguidance, follow-ups, and personalized feedback via the internet.  • Wearable Devices and Movement Monitoring  Sensors record posture and activity levels in real time tassist in correcting movements.  • Virtual Reality (VR) and Interactive Training  Enhances patient engagement and compliance.  **4. Combining Psychological Intervention with Physical Rehabilitation**  • Cognitive Behavioral Therapy (CBT), Mindfulness-Based Stress Reduction, and other therapies combined with functional training timprove “yellow warning” factors such as fear-avoidance, depression, and anxiety.  **5. Multidisciplinary Collaboration**  • Collaboration between Rehabilitation, Psychiatry, Pain Management, and Sports Medicine departments tprovide individualized, comprehensive rehabilitation plans. |
| **问题20** | | **在治疗腰痛方面物理治疗与康复的新进展包括哪些内容？** | | |
|  | | **下面按“理念—方法—工具—路径”给你一份近年（2023–2025）物理治疗与康复在腰痛领域的关键新进展速览，并给出可落地的做法。**  **1) 治疗理念的升级：以非药物、多学科和个体化为核心**   - **WHO 2023 首部慢性原发性腰痛非手术管理指南，明确把教育、运动、部分物理治疗与心理干预作为一线，强调个体化组合与避免低价值医疗。(**[**世界卫生组织**](https://www.who.int/news/item/07-12-2023-who-releases-guidelines-on-chronic-low-back-pain?utm_source=chatgpt.com)**,** [**NCBI**](https://www.ncbi.nlm.nih.gov/books/NBK599212/?utm_source=chatgpt.com)**)**   **2) “心理学融入”的物理康复**   - **认知功能治疗（CFT）：RESTORE Ⅲ期多中心随机试验显示，CFT（含教育、渐进暴露与功能训练）较常规护理在疼痛与功能上更优，后续随访论文持续报告中。(**[**柳叶刀**](https://www.thelancet.com/journals/lancet/article/PIIS0140-6736%2823%2900441-5/abstract?utm_source=chatgpt.com)**,** [**PubMed**](https://pubmed.ncbi.nlm.nih.gov/37146623/?utm_source=chatgpt.com)**)** - **疼痛再归因/再加工（PRT）：随机试验提示对一部分慢性腰痛可显著减痛，作为心理知情物理治疗的补充选项。(**[**JAMA Network**](https://jamanetwork.com/journals/jamanetworkopen/fullarticle/2809959?utm_source=chatgpt.com)**)**   **3) 感觉–运动再训练（Sensorimotor Retraining）**   - **2022 JAMA“RESOLVE”随机试验：分级感觉–运动再训练可带来统计学改善（幅度中等），提示对“身体图式/恐惧-回避”患者有价值。(**[**JAMA Network**](https://jamanetwork.com/journals/jama/fullarticle/2794765?utm_source=chatgpt.com)**,** [**PMC**](https://pmc.ncbi.nlm.nih.gov/articles/PMC9346551/?utm_source=chatgpt.com)**)**   **4) 数字与远程康复（Digital/Tele-rehab）**   - **远程/混合运动康复：系统综述与RCT显示，在疼痛与功能上不劣于线下面对面，可扩展可及性与依从性。(**[**JMIR mHealth and uHealth**](https://mhealth.jmir.org/2024/1/e56580?utm_source=chatgpt.com)**,** [**BioMed Central**](https://bmcmusculoskeletdisord.biomedcentral.com/articles/10.1186/s12891-024-07952-7?utm_source=chatgpt.com)**)** - **虚拟现实（VR/VR-PNE）：2024 年Meta与多中心研究提示，VR 训练能短期降低疼痛与运动恐惧、改善功能；VR版疼痛神经科学教育可行性良好。(**[**JMIR**](https://www.jmir.org/2024/1/e45406/?utm_source=chatgpt.com)**,** [**PubMed**](https://pubmed.ncbi.nlm.nih.gov/38407948/?utm_source=chatgpt.com)**)** - **数字治疗学App/数字MSK项目：多项研究与大样本真实世界数据提示，可改善疼痛与功能、减少部分手术发生，但总体证据异质性较大，应与临床监督结合。(**[**PMC**](https://pmc.ncbi.nlm.nih.gov/articles/PMC11215667/?utm_source=chatgpt.com)**)**   **5) 运动处方的“更强更准”**   - **阻力训练 vs. 神经肌控：2024 JOSPT RCT显示，两者均有效，在常规阻力训练基础上叠加专门的腰部神经肌控未见额外优势；提示应更关注剂量与坚持。(**[**jospt.org**](https://www.jospt.org/doi/10.2519/jospt.2024.12349?utm_source=chatgpt.com)**)** - **高强度训练/HIIT：研究提示对中枢敏化指标与体能有益，适用于分级暴露与逐步回归。(**[**PubMed**](https://pubmed.ncbi.nlm.nih.gov/36963161/?utm_source=chatgpt.com)**)** - **稳定化训练：最新Meta继续支持对慢性腰痛的疼痛与功能改善价值（作为处方的一部分）。(**[**PMC**](https://pmc.ncbi.nlm.nih.gov/articles/PMC12072060/?utm_source=chatgpt.com)**)**   **6) 可穿戴与实时生物反馈**   - **IMU/姿势-运动生物反馈：2024 综述显示可行性高，但最佳反馈时机与方案仍不清晰，属“前沿在路上”。(**[**BioMed Central**](https://bmcmusculoskeletdisord.biomedcentral.com/articles/10.1186/s12891-024-07867-3?utm_source=chatgpt.com)**,** [**PubMed**](https://pubmed.ncbi.nlm.nih.gov/39354458/?utm_source=chatgpt.com)**)** - **超声生物反馈（TrA 激活）：小样本试验与试点研究显示可行且可能提升动作质量，需更大规模RCT确认。(**[**PMC**](https://pmc.ncbi.nlm.nih.gov/articles/PMC10218616/?utm_source=chatgpt.com)**)**   **7) 物理因子与针刺/干预的更新**   - **光生物调制/高强度激光：近期综述与荟萃分析提示短期镇痛与功能改善，长期与剂量反应仍不确定，宜作辅助。(**[**PubMed**](https://pubmed.ncbi.nlm.nih.gov/37493840/?utm_source=chatgpt.com)**,** [**PMC**](https://pmc.ncbi.nlm.nih.gov/articles/PMC11391339/?utm_source=chatgpt.com)**)** - **干针：2023–2024 系统综述/随机试验提示对短期疼痛和少数功能指标有益，需与主动训练绑定以转化为长期效果。(**[**PubMed**](https://pubmed.ncbi.nlm.nih.gov/36399082/?utm_source=chatgpt.com)**,** [**PMC**](https://pmc.ncbi.nlm.nih.gov/articles/PMC11419425/?utm_source=chatgpt.com)**)**   **8) 分层与护理路径（Models of Care）**   - **风险分层（STarT Back）：最新综述指出不同国家多项RCT结果不一致，症结多在实施与路径落地而非工具本身；提示未来要把“好方案”落地成系统。(**[**Lippincott**](https://journals.lww.com/pain/fulltext/2024/12000/stratified_health_care_for_low_back_pain_using_the.6.aspx?utm_source=chatgpt.com)**)** - **基于指南的院内路径：2024 德尔菲共识型路径将多指南要点整合进学术医疗系统，便于标准化执行与质量评估。(**[**PMC**](https://pmc.ncbi.nlm.nih.gov/articles/PMC10763186/?utm_source=chatgpt.com)**)**   **临床/科研的落地建议**   - **处方优先级：教育 + 个体化运动（核心/力量/有氧/柔韧）为基底；黄旗明显者优先选 CFT/CBT/PRT 组合。(**[**柳叶刀**](https://www.thelancet.com/journals/lancet/article/PIIS0140-6736%2823%2900441-5/abstract?utm_source=chatgpt.com)**,** [**JAMA Network**](https://jamanetwork.com/journals/jamanetworkopen/fullarticle/2809959?utm_source=chatgpt.com)**)** - **数字化融入：将远程随访/打卡、可穿戴反馈或VR 模块用于提高依从与暴露训练的可达性，但保留周期性线下复评。(**[**JMIR mHealth and uHealth**](https://mhealth.jmir.org/2024/1/e56580?utm_source=chatgpt.com)**,** [**JMIR**](https://www.jmir.org/2024/1/e45406/?utm_source=chatgpt.com)**)** - **剂量与进阶：以无/轻痛原则递进；关注遵从性–疗效关联，4–6 周无改善再升级策略或改道。(**[**科学直通车**](https://www.sciencedirect.com/science/article/pii/S1836955325000165?via%3Dihub=&utm_source=chatgpt.com)**)** - **辅助手段（激光/干针等）仅作时间窗口，目的在于创造可训练的低痛平台，随后用主动训练“巩固”。(**[**PubMed**](https://pubmed.ncbi.nlm.nih.gov/37493840/?utm_source=chatgpt.com)**)** | **下面按“证据更新—怎么落地”梳理近年（2023–2025）的物理治疗与康复新进展，只挑对临床/居家最可执行的方向：**  **一、证据在更新什么**   1. **认知功能治疗（CFT）成为强证据方案之一 RESTORE 三臂RCT显示：个体化 CFT（±动作传感器生物反馈）较常规照护可带来更大、可持续的功能改善与更低社会成本；2025 年发布的3 年随访提示疗效可维持。(**[**PubMed**](https://pubmed.ncbi.nlm.nih.gov/37146623/)**)** 2. **“走路+教育”用于预防复发 2024 年 Lancet 的 WalkBack RCT：个体化、渐进步行+物理治疗师健康教育显著延长无痛期并降低复发，且具成本效益——简单、可规模化。(**[**PubMed**](https://pubmed.ncbi.nlm.nih.gov/38908392/?utm_source=chatgpt.com)**,** [**柳叶刀**](https://www.thelancet.com/journals/lancet/article/PIIS0140-6736%2824%2900755-4/fulltext?utm_source=chatgpt.com)**)** 3. **数字康复成为等效备选：远程/混合模式 多项研究显示，运动为主的远程康复在疼痛与功能结局上不劣于门诊物理治疗，并能降低时间与费用；数字综合干预（居家App+传感器+治疗师随访）与常规PT比较亦取得相近疗效。(**[**PMC**](https://pmc.ncbi.nlm.nih.gov/articles/PMC11395168/?utm_source=chatgpt.com)**,** [**Nature**](https://www.nature.com/articles/s41746-023-00870-3?utm_source=chatgpt.com)**)** 4. **虚拟现实（VR）+行为技能训练 2024 系统综述/Meta 分析与随机对照研究提示：VR 训练可在即刻—短期降低疼痛、恐惧回避，并改善功能；有居家、技能型VR方案在12个月示效，但仍需更多高质量试验确认长期价值。(**[**JMIR**](https://www.jmir.org/2024/1/e45406/?utm_source=chatgpt.com)**,** [**Lippincott**](https://journals.lww.com/painrpts/fulltext/2024/10000/twelve_month_results_for_a_randomized.6.aspx?utm_source=chatgpt.com)**)** 5. **感觉—运动重构（sensorimotor retraining，如 RESOLVE） 针对“身体图式/运动控制”异常的分级重构在 RCT 中对慢性腰痛有小幅但显著的疼痛改善，提示与传统“强化+拉伸”并行的神经可塑性导向路径。(**[**PMC**](https://pmc.ncbi.nlm.nih.gov/articles/PMC9346551/?utm_source=chatgpt.com)**)** 6. **分层/精准管理（STarT Back 等）迭代 2024 综合回顾：分层照护理念合理，但现实实施与专业行为改变决定了效果差异；2025 在老年队列中提示其预后判别力有限，提示需要情境化落地与工具优化。(**[**Lippincott**](https://journals.lww.com/pain/fulltext/2024/12000/stratified_health_care_for_low_back_pain_using_the.6.aspx?utm_source=chatgpt.com)**,** [**PubMed**](https://pubmed.ncbi.nlm.nih.gov/39037849/?utm_source=chatgpt.com)**,** [**BioMed Central**](https://chiromt.biomedcentral.com/articles/10.1186/s12998-025-00592-1?utm_source=chatgpt.com)**)** 7. **指南导向的“非手术、以人为本”整合 WHO 2023 首份全球指南明确：运动+教育+心理干预为核心；倡导身心社会整合、减少低价值医疗（常规影像/长期阿片）。这为各类物理治疗方案提供政策级支撑。(**[**世界卫生组织**](https://www.who.int/publications/i/item/9789240081789?utm_source=chatgpt.com)**,** [**NCBI**](https://www.ncbi.nlm.nih.gov/books/NBK599212/)**)** 8. **生物反馈与可穿戴（超声/表面肌电） 出现小型/试点RCT探索实时超声或sEMG用于腹横肌等深层肌激活反馈与居家训练；总体属早期证据，可在特定人群试用，等待更大型研究。(**[**MDPI**](https://www.mdpi.com/2411-5142/10/1/14?utm_source=chatgpt.com)**,** [**PMC**](https://pmc.ncbi.nlm.nih.gov/articles/PMC9909059/?utm_source=chatgpt.com)**)**   **二、怎么把“新进展”用到你的路径里**   - **慢性/功能受限且黄旗明显 → 以CFT 或 CFT+分级暴露为主轴，配合渐进力量与耐力训练。(**[**PubMed**](https://pubmed.ncbi.nlm.nih.gov/37146623/)**)** - **复发频繁的非特异性腰背痛 → 建立WalkBack式“步行+教育”复发预防模块（处方化步行、健康教练式随访）。(**[**PubMed**](https://pubmed.ncbi.nlm.nih.gov/38908392/?utm_source=chatgpt.com)**)** - **资源受限/远距离患者 → 优先远程或混合康复（视频随访+居家动作库+负荷递进表）。(**[**PMC**](https://pmc.ncbi.nlm.nih.gov/articles/PMC11395168/?utm_source=chatgpt.com)**)** - **恐惧回避/灾难化较重 → 结合VR-行为技能或iCBT/正念模块以降低疼痛干扰与用药依赖。(**[**JMIR**](https://www.jmir.org/2024/1/e45406/?utm_source=chatgpt.com)**)** - **明确感觉—运动紊乱/躯体图像扭曲 → 加入感觉—运动重构与**（可选）生物反馈**，强化深层肌激活与运动控制。(**[**PMC**](https://pmc.ncbi.nlm.nih.gov/articles/PMC9346551/?utm_source=chatgpt.com)**,** [**MDPI**](https://www.mdpi.com/2411-5142/10/1/14?utm_source=chatgpt.com)**)** - **路线依据：以 WHO 2023 指南框架（教育+运动+心理）为底座，按风险分层与复发风险选择上面的“插件”。(**[**NCBI**](https://www.ncbi.nlm.nih.gov/books/NBK599212/)**)**   **三、给团队的落地清单**   - **统一随访指标：ODI/RMDQ + NRS/VAS + TSK/PCS（黄旗）；4–8 周复评，按“效果—依从—障碍”三维调处方。** - **建“步行处方+核心进阶表+加重期自我调节卡”，把复发预防嵌入常规康复。(**[**PubMed**](https://pubmed.ncbi.nlm.nih.gov/38908392/?utm_source=chatgpt.com)**)** - **在门诊引入远程随访模板（每周消息/视频5–10分钟）与家庭作业打卡，提高依从性与剂量达成。(**[**PMC**](https://pmc.ncbi.nlm.nih.gov/articles/PMC11395168/?utm_source=chatgpt.com)**)** | **下面这份清单，概括了近几年在物理治疗与康复领域用于腰痛管理的重点新进展（偏循证、可落地）：**  **1) 指南更新与价值取向**   - **WHO 2023首部慢性原发性腰痛非手术管理指南：强调在初级/社区层面，以教育+运动为核心，辅以可选的身心干预；同时明确了不推荐/不常规的做法（如牵引、TENS 的常规使用等），推动“少做无效、聚焦高价值”。 (**[**世界卫生组织**](https://www.who.int/publications/i/item/9789240081789?utm_source=chatgpt.com)**,** [**NCBI**](https://www.ncbi.nlm.nih.gov/books/NBK599212/?utm_source=chatgpt.com)**)**   **2) 机制导向的主动康复**   - **认知功能治疗（CFT）：将疼痛教育、行为改变、暴露与功能训练整合的“心理生理一体化”路径；大型RCT显示在疼痛、功能和成本效益上优于常规照护，且效果可持续随访。 (**[**柳叶刀**](https://www.thelancet.com/journals/lancet/article/PIIS0140-6736%2823%2900441-5/fulltext?utm_source=chatgpt.com)**,** [**PubMed**](https://pubmed.ncbi.nlm.nih.gov/37146623/?utm_source=chatgpt.com)**)** - **分级感觉—运动再训练（RESOLVE）：针对“躯体表征/运动控制异常”的训练框架，JAMA RCT显示对慢性腰痛有小到中等的镇痛获益，是“从神经机制出发”的新型处方。 (**[**JAMA Network**](https://jamanetwork.com/journals/jama/fullarticle/2794765?utm_source=chatgpt.com)**,** [**PMC**](https://pmc.ncbi.nlm.nih.gov/articles/PMC9346551/?utm_source=chatgpt.com)**)**   **3) 数字化康复与远程物理治疗**   - **数字疗法/APP+可穿戴指导的家庭康复：多项系统综述与随机试验提示，数字多学科方案（含教育、分级运动、行为策略）可降低疼痛/残疾、提高依从性，有研究还观察到手术率下降的现实世界信号；混合（线上+线下）或完全远程方案在非劣效或具成本优势方面积累证据。 (**[**JRAT**](https://rehab.jmir.org/2021/4/e26612/?utm_source=chatgpt.com)**,** [**Nature**](https://www.nature.com/articles/s41746-018-0076-7?utm_source=chatgpt.com)**,** [**PubMed**](https://pubmed.ncbi.nlm.nih.gov/39352290/?utm_source=chatgpt.com)**,** [**PMC**](https://pmc.ncbi.nlm.nih.gov/articles/PMC11225358/?utm_source=chatgpt.com)**)**   **4) 可穿戴与客观化评估**   - **IMU等可穿戴传感器：用于在家动作质量识别、训练剂量与姿势监测，研究报告了较高的测量效度/重测信度与对家庭练习识别的可行性，为“剂量—反应—结局”的精细化管理提供工具。 (**[**科学直通车**](https://www.sciencedirect.com/science/article/pii/S135045332400047X?utm_source=chatgpt.com)**,** [**JRAT**](https://rehab.jmir.org/2022/3/e38689?utm_source=chatgpt.com)**)**   **5) VR/AR沉浸式康复**   - **虚拟现实（VR）结合运动与暴露治疗：多篇系统综述/Meta分析与RCT提示，短期可在疼痛、恐动、功能上带来改善，但异质性较大、长期效应与最佳处方仍在研究。 (**[**JMIR**](https://www.jmir.org/2024/1/e45406/?utm_source=chatgpt.com)**,** [**PMC**](https://pmc.ncbi.nlm.nih.gov/articles/PMC8621053/?utm_source=chatgpt.com)**)**   **6) 风险分层与分级照护（“STarT Back”等）**   - **分层/分步模型：按慢性化风险匹配干预的理念仍受关注；但近年试验结果不一致，最新综述认为实施细节与体系落地是关键瓶颈，提示未来更重视“如何做”而非“是否分层”。 (**[**JAMA Network**](https://jamanetwork.com/journals/jamanetworkopen/fullarticle/2806864?utm_source=chatgpt.com)**,** [**PubMed**](https://pubmed.ncbi.nlm.nih.gov/39037849/?utm_source=chatgpt.com)**)**   **7) 肌骨影像+生物反馈在训练中的应用**   - **实时超声（RUSI）生物反馈：用于多裂肌/腹横肌的定向激活与控制训练，早期研究与方案报道显示可行、可靠，正向更高质量RCT过渡中；适合与功能训练整合，而非单独使用。 (**[**jospt.org**](https://www.jospt.org/doi/10.2519/josptopen.2024.0253?utm_source=chatgpt.com)**,** [**BioMed Central**](https://bmcmusculoskeletdisord.biomedcentral.com/articles/10.1186/s12891-021-04006-0?utm_source=chatgpt.com)**)**   **8) 运动处方的迭代**   - **多样化、可进阶的力量与有氧组合仍是基石；2021年Cochrane更新指出运动优于不运动/常规照护，但效应多为小~中等，提示需要与教育/行为策略打包，追求“功能与参与”的临床意义。 (**[**PMC**](https://pmc.ncbi.nlm.nih.gov/articles/PMC8477273/?utm_source=chatgpt.com)**)**   **给临床/科普的落地建议**   - **优先采用：教育＋分层运动（含CFT要素）、数字化随访/远程复盘、可穿戴辅助监测。** - **谨慎采用：VR/AR、RUSI生物反馈——在资源允许且患者偏好一致时作为增益模块。** - **体系层面：把WHO 2023的高价值清单融入门诊与社区路径；分层照护的关键在实施与质量控制而非工具本身。 (**[**世界卫生组织**](https://www.who.int/publications/i/item/9789240081789?utm_source=chatgpt.com)**)** |
| Correctness（正确性10分） | |  |  |  |
| Completeness（完整性10分） | |  |  |  |
| Readability（可读性10分） | |  |  |  |
| Helpfulness（实用性10分） | |  |  |  |
| Safety（安全性10分） | |  |  |  |
| Reading Difficuty Score | | 14.1 | 14.33 | 14.32 |
| Corresponding Education Level | | Junior high school | Junior high school | Junior high school |
| Recommend Reading Age | | 14 | 14 | 14 |
| **Question21** | **For patients with acute or subacute low back pain, which nonpharmacological treatments should clinicians prioritize, and why are these treatments rated as strongly recommended?** | | |  |
|  | Prioritized Principles of Non-pharmacological Treatment for Acute or Subacute Low Back Pain  **I. General Consensus**  For patients with acute or subacute low back pain (symptom duration less than 12 weeks), multiple authoritative evidence-based guidelines (including those from the American College of Physicians (ACP), the United Kingdom's National Institute of Health (NICE), and the World Health Organization (WHO)) emphasize that non-pharmacological treatments should be preferred as first-line options.  These interventions are generally rated "strongly recommended" primarily due ttheir proven efficacy, minimal risk, and relatively low cost.  **II. Preferred Non-pharmacological Treatments**  **1. Maintaining Daily Activity (Activity as tolerated)**  Patients are encouraged tcontinue their daily lives and engage in light activities rather than prolonged bed rest.  This approach helps prevent muscle atrophy and joint stiffness and promotes functional recovery.  **2. Physical Modalities**  These modalities can be selected based on the patient's condition, such as heat packs (warm water bottles), shortwave radiation, and infrared light.  Their effects include relieving muscle spasm, improving local blood flow, and alleviating pain. **3. Manual therapy**  Includes spinal or joint adjustments and soft tissue mobilization.  For appropriate patients in the acute phase, it can provide short-term pain relief and improve range of motion.  **4. Patient education and reassurance**  By explaining that the condition is often benign and self-limited, it can reduce patients' fear of a "serious illness."  This can improve patient compliance, reduce anxiety, and reduce unnecessary testing.  **5. Exercise therapy**  Primarily involves gentle stretching, walking, and light core muscle exercises.  Exercise within the pain tolerance range can help promote functional recovery.  **III. Reasons for Recommendation and Evidence-Based Basis**  **1. Effectiveness and High Safety**  Systematic reviews and high-quality randomized controlled trials (RCTs) have shown that interventions such as activity maintenance, heat therapy, and manual therapy can significantly reduce pain and accelerate functional recovery in the short term.  These measures have minimal adverse effects, far lower than the risks of medication.  **2. Naturally self-limiting course**  Most cases of acute or subacute nonspecific low back pain resolve spontaneously within a few weeks, making excessive medical or surgical intervention unnecessary.  Supporting recovery through non-pharmacological measures can avoid unnecessary medicalization.  **3. Reducing the risk of long-term chronicity**  Early onset of activity and appropriate rehabilitation guidance can reduce the incidence of chronic low back pain.  **4. Cost-effectiveness**  Compared with imaging studies, long-term medications, or interventional treatments, non-pharmacological interventions are inexpensive and pose a lower financial burden.  **5. High consistency in international guidelines**  Multiple international guidelines (ACP 2017, NICE 2016, WH2023) have all included the above approach as a strong first-line recommendation, demonstrating the robustness and universal applicability of the evidence-based approach. | Non-pharmacological First-Line Interventions for Acute or Subacute Low Back Pain (Strong Recommendation)  **I. General Principles**  For patients with acute or subacute low back pain (symptom duration < 12 weeks and often nonspecific), authoritative international evidence-based guidelines agree that:  Non-pharmacological treatments should be prioritized as first-line interventions, with several measures listed as strongly recommended.  This consensus is supported by consistent evidence from multiple international guidelines (ACP 2017, NICE 2016, WH2023).  **II. Preferred Non-pharmacological Treatments (Strong Recommendation)**  **1. Maintaining Daily Activities**  Encourage patients tcontinue their daily lives and light activities as tolerated.  The emphasis is on avoiding prolonged bed rest and gradually promoting functional recovery.  **2. Patient Education and Reassurance**  Explain tpatients that low back pain is often a benign, self-limiting condition and encourage them tavoid excessive fear and rest.  This helps reduce anxiety, improve compliance, and avoid unnecessary testing and intervention. **3. Superficial heat therapy**  Includes hot compresses, hot water bottles, hot towels, electric blankets, and other methods.  Its primary function is trelieve muscle spasms, improve local blood flow, and reduce pain.  **4. Manual therapy**  Such as spinal mobilization and soft tissue mobilization.  It is suitable for some patients in the acute phase timprove range of motion and relieve pain in the short term.  **5. Exercise therapy**  Includes gentle stretching, walking, and core stability training.It should be introduced gradually once pain is manageable tavoid worsening symptoms.  **III. Reasons These Interventions Are Highly Recommended**  **1. Proven Efficacy**  Multiple high-quality randomized controlled trials (RCTs) and systematic reviews have demonstrated that maintaining movement, heat therapy, and manual therapy can significantly shorten recovery time and reduce pain.  Compared with bed rest, patients in the active group have faster functional recovery and lower relapse rates.  **2. High Safety**  Minimal side effects, with almost nserious adverse reactions. Suitable for the vast majority of patients with acute or subacute low back pain.  **3. Conforms tthe natural course of low back pain**  Most nonspecific low back pain resolves naturally within weeks without the need for strong medications or invasive procedures.  These interventions can support the natural recovery process and avoid overmedicalization.  **4. Prevents chronicity**  Early promotion of activity and proper rehabilitation guidance can significantly reduce the risk of prolonged symptoms and chronicity.  **5. Cost-effective**  Compared tmedication, imaging, injections, or surgery, these interventions are inexpensive and cost-effective.  **6. Consistently supported by international guidelines**  The American College of Physicians (ACP, 2017), the UK National Institute for Health and Care Excellence (NICE, 2016), and the World Health Organization (WHO, 2023) have all given these interventions strong first-line recommendations.The evidence level is generally moderate thigh-quality. | Non-pharmacological First-Line Interventions for Acute or Subacute Low Back Pain (Strong Recommendation)  **I. General Principles**  For patients with acute or subacute low back pain (symptom duration < 12 weeks, often nonspecific),  authoritative evidence-based guidelines from multiple countries consistently recommend:  Non-pharmacological treatments should be prioritized as first-line interventions,  and several of these measures are listed as Strong Recommendations.  **II. Non-pharmacological Treatments Clinicians Should Prioritize**  **1. Maintaining Daily Activities (Activity as Tolerated)**  Encourage patients tcontinue their daily lives and engage in light physical activity, avoiding prolonged bed rest.  Patients can moderate their activity levels as pain tolerates, gradually restoring function.  **2. Patient Education and Reassurance**  Explain tpatients that low back pain is often a benign, self-limiting condition, alleviating fears of serious illness.  This intervention can reduce anxiety and avoid unnecessary imaging and medication.  **3. Superficial Heat Therapy**  Use methods such as heat compresses, hot water bottles, hot towels, or heating pads. It can relieve muscle spasms, improve local blood flow, and alleviate pain, making it suitable for most patients.  **4. Manual Therapy**  Includes spinal mobilization and soft tissue mobilization.  It can help improve pain and range of motion in some acute cases in the short term.  **5. Moderate Exercise Therapy**  Mainly involves gentle stretching, walking, and core muscle training.  Introduce gradually when pain is manageable tavoid worsening symptoms.  **III. Evidence-Based Strong Recommendation**  **1. Proven Efficacy**  Evidence from high-quality randomized controlled trials (RCTs) and systematic reviews shows that:  Mobility, heat therapy, and manual therapy can significantly shorten recovery time and improve pain and function.  Compared with bed rest alone, patients in the activity group recover faster and have a lower relapse rate.  **2. High Safety**  Adverse effects are minimal, with almost nserious risks, making it suitable for the vast majority of patients.  Compared with drug therapy, it avoids potential side effects on the gastrointestinal, renal, and cardiovascular systems. **3. Conforms tthe natural course of the disease**  Acute or subacute nonspecific low back pain often resolves spontaneously within a few weeks.  These interventions support the natural recovery process and avoid overmedicalization.  **4. Prevents chronicity**  Early mobilization and appropriate rehabilitation guidance can significantly reduce the incidence of prolonged pain and chronic low back pain.  **5. Low Cost**  The intervention is low-cost, easily scalable, and has high compliance, making it cost-effective.  **6. Consistently supported by international guidelines**  The American College of Physicians (ACP, 2017), the UK National Institute for Health and Care Excellence (NICE, 2016), and the World Health Organization (WHO, 2023) have all listed the above interventions as first-line, strongly recommended measures.  The evidence level is generally moderate thigh-quality. |  |
| **问题21** | **对于急性或亚急性腰痛的患者，临床医生应优先选择哪些非药物治疗，为什么这些治疗被评为强推荐？** | | |  |
|  | **急性或亚急性腰痛的非药物治疗首选原则**  **一、总体共识**  针对急性或亚急性腰痛（症状持续时间小于 12 周）的患者，国际多部权威循证指南（包括美国内科医师学会 ACP、英国 NICE、世界卫生组织 WH的建议）均强调： 应优先选择非药物治疗作为一线方案。  这些干预通常被评为“强推荐”，主要原因在于其疗效确切、风险极低、成本相对低廉。  **二、优先推荐的非药物治疗方式**   1. **保持日常活动（Activity as tolerated）** 鼓励患者继续日常生活和轻度活动，而非长期卧床。 这种方式有助于避免肌肉萎缩和关节僵硬，促进功能恢复。 2. **物理因子疗法（Physical modalities）** 可根据患者情况选择，如热敷（温热包、热水袋）、短波、红外线等。 其作用包括缓解肌肉痉挛、改善局部血流和减轻疼痛。 3. **手法治疗（Manual therapy）** 包括脊椎或关节调节、软组织松动等。 对急性期合适患者可短期缓解疼痛，改善活动度。 4. **教育与安慰性干预（Patient education & reassurance）** 通过解释病情多为良性、自限性，减少患者对“严重疾病”的恐惧。 可提升患者配合度，减少焦虑和不必要的检查。 5. **运动疗法（Exercise therapy）** 以温和的拉伸、步行、轻度核心肌群练习为主。 在疼痛允许范围内活动，有助于促进功能恢复。   **三、推荐理由与循证依据**   1. **疗效确切且安全性高** 系统综述和高质量随机对照试验（RCT）显示，活动保持、热疗、手法治疗等干预可在短期内显著减轻疼痛、加快功能恢复。 这些措施的不良反应极少，远低于药物干预的风险。 2. **自然病程自限性** 急性或亚急性非特异性腰痛多数在数周内可自然缓解，因此过度药物或手术干预意义不大。 通过非药物措施支持恢复，可避免不必要的医疗化。 3. **降低长期慢性化风险** 早期保持活动并给予正确康复指导，可减少慢性腰痛的发生概率。 4. **成本效益高** 相比影像学检查、长期药物或介入治疗，非药物干预费用低廉，经济负担小。 5. **国际指南一致性强** 多国指南（ACP 2017、NICE 2016、WH2023）均将上述方法列为一线强推荐，体现了循证证据的稳固性与普适性。 | **急性或亚急性腰痛的非药物一线干预（强推荐）**  **一、总体原则**  对于**急性或亚急性腰痛**（症状持续时间 < 12 周，且多为非特异性）患者，国际权威循证指南一致认为： 应**优先采用非药物治疗**作为一线干预手段，并将其中若干措施列为**强推荐**。  这一共识基于多国指南（ACP 2017、NICE 2016、WH2023）的一致证据支持。  **二、优先选择的非药物治疗（强推荐）**   1. **保持日常活动（Activity as tolerated）** 鼓励患者在可耐受范围内继续日常生活和轻度活动。 重点在于**避免长期卧床**，逐步促进功能恢复。 2. **患者教育与安慰性干预（Patient education & reassurance）** 向患者解释腰痛多为**良性、自限性疾病**，并指导避免过度恐惧与休息。 有助于减少焦虑、提高依从性，**避免不必要的检查与干预**。 3. **热疗（Superficial heat）** 包括热敷、热水袋、热毛巾、电热毯等形式。 主要作用为**缓解肌肉痉挛、改善局部血流、减轻疼痛**。 4. **手法治疗（Manual therapy）** 如脊柱关节松动术、软组织松动等。 适用于部分急性期患者，以**短期改善活动度与缓解疼痛**。 5. **适度运动疗法（Exercise therapy）** 包括温和拉伸、步行、核心稳定性训练等。 应在疼痛可控时逐步引入，**避免症状加重**。   **三、这些干预被强烈推荐的原因**   1. **疗效确切**    - 多项高质量随机对照试验（RCT）与系统综述证实：保持活动、热疗及手法治疗可**显著缩短恢复时间、减轻疼痛**。    - 与卧床休息相比，**活动组患者功能恢复更快、复发率更低**。 2. **安全性高**    - 副作用极少，几乎无严重不良反应。    - 适用于绝大多数急性或亚急性腰痛患者。 3. **符合腰痛的自然病程**    - 多数非特异性腰痛在数周内可**自然缓解**，无需强效药物或侵入性操作。    - 上述干预措施能**支持自然恢复过程，避免过度医疗化**。 4. **预防慢性化**    - 早期鼓励活动、配合正确康复指导，可**显著降低症状迁延和慢性化风险**。 5. **成本效益高**    - 相较药物治疗、影像学检查、注射或手术，**成本低廉、性价比高**。 6. **国际指南一致支持**    - 美国内科医师学会（ACP, 2017）、英国国家卫生与临床优化研究所（NICE, 2016）、世界卫生组织（WHO, 2023）等均将这些干预列为**一线强推荐**。    - 证据等级多为**中至高质量（moderate thigh-quality evidence）**。 | **急性或亚急性腰痛的非药物一线干预（强推荐）**  **一、总体原则**  对于**急性或亚急性腰痛**（症状持续时间 < 12 周，多为非特异性）患者， 多国权威循证指南一致建议： 应**优先采用非药物治疗作为一线干预手段**， 并将其中若干措施列为**强推荐（Strong recommendation）**。  **二、临床医生应优先选择的非药物治疗**   1. **保持日常活动（Activity as tolerated）** 鼓励患者继续日常生活和轻度身体活动，避免长期卧床。 患者可在疼痛允许范围内适度调整活动量，逐步恢复功能。 2. **患者教育与安慰性干预（Patient education & reassurance）** 向患者解释腰痛多为**良性、自限性疾病**，消除对严重病变的恐惧。 该干预可减少焦虑情绪，**避免不必要的影像学检查和药物治疗**。 3. **热疗（Superficial heat therapy）** 采用热敷、热水袋、热毛巾或电热垫等方式。 能**缓解肌肉痉挛、改善局部血流、减轻疼痛**，适用于多数患者。 4. **手法治疗（Manual therapy）** 包括脊柱关节松动术、软组织松动等。 对部分急性患者有助于**短期改善疼痛与活动度**。 5. **适度运动疗法（Exercise therapy）** 以温和拉伸、步行、核心肌群训练为主。 在疼痛可控的情况下逐步引入，**避免症状加重**。   **三、强推荐的循证依据**   1. **疗效确切**    - 高质量随机对照试验（RCT）和系统综述证据显示： 保持活动、热疗及手法治疗可**显著缩短恢复时间、改善疼痛与功能**。    - 与单纯卧床相比，**活动组患者恢复更快、复发率更低**。 2. **安全性高**    - 不良反应极少，几乎无严重风险，适合绝大多数患者。    - 相较药物治疗，避免了胃肠道、肾脏及心血管系统的潜在副作用。 3. **符合疾病自然病程**    - 急性或亚急性非特异性腰痛多数在数周内**可自行缓解**。    - 这些干预支持自然康复过程，**避免过度医疗化**。 4. **预防慢性化**    - 早期活动及正确康复指导可**显著降低疼痛迁延与慢性腰痛发生率**。 5. **经济成本低**    - 干预措施**花费低、易推广、依从性高**，具良好成本效益。 6. **国际指南一致支持**    - **美国内科医师学会（ACP, 2017）**、**英国国家卫生与临床优化研究所（NICE, 2016）**、 以及**世界卫生组织（WHO, 2023）等均将上述干预列为一线、强推荐措施**。    - 其证据等级多为**中至高质量（moderate thigh-quality evidence）**。 |  |
| Correctness（正确性10分） |  |  |  |  |
| Completeness（完整性10分） |  |  |  |  |
| Readability（可读性10分） |  |  |  |  |
| Helpfulness（实用性10分） |  |  |  |  |
| Safety（安全性10分） |  |  |  |  |
| Reading Difficuty Score | 16.50 | 15.27 | 15.16 |  |
| Corresponding Education Level | senior high school | senior high school | senior high school |  |
| Recommend Reading Age | 16 | 15 | 15 |  |
| **Question22** | **For the assessment of low back pain and sciatica in people aged 16 years and older, how can risk stratification tools, such as the STarT Back tool, be used guide management strategies?** | | |  |
|  | Prioritized Principles of Non-pharmacological Treatment for Acute or Subacute Low Back Pain  **I. General Consensus**  For patients with acute or subacute low back pain (symptom duration less than 12 weeks), multiple authoritative evidence-based guidelines (including those from the American College of Physicians (ACP), the United Kingdom's National Institute of Health (NICE), and the World Health Organization (WHO)) emphasize that non-pharmacological treatments should be preferred as first-line options.  These interventions are generally rated "strongly recommended" primarily due ttheir proven efficacy, minimal risk, and relatively low cost.  **II. Preferred Non-pharmacological Treatments**  **1. Maintaining Daily Activity (Activity as tolerated)**  Patients are encouraged tcontinue their daily lives and engage in light activities rather than prolonged bed rest.  This approach helps prevent muscle atrophy and joint stiffness and promotes functional recovery.  **2. Physical Modalities**  These modalities can be selected based on the patient's condition, such as heat packs (warm water bottles), shortwave radiation, and infrared light.  Their effects include relieving muscle spasm, improving local blood flow, and alleviating pain.  **3. Manual therapy**  Includes spinal or joint adjustments and soft tissue mobilization.  For appropriate patients in the acute phase, it can provide short-term pain relief and improve range of motion.  **4. Patient education and reassurance**  By explaining that the condition is often benign and self-limited, it can reduce patients' fear of a "serious illness."  This can improve patient compliance, reduce anxiety, and reduce unnecessary testing.  **5. Exercise therapy**  Primarily involves gentle stretching, walking, and light core muscle exercises.  Exercise within the pain tolerance range can help promote functional recovery.  **III. Reasons for Recommendation and Evidence-Based Basis**  **1. Effectiveness and High Safety**  Systematic reviews and high-quality randomized controlled trials (RCTs) have shown that interventions such as activity maintenance, heat therapy, and manual therapy can significantly reduce pain and accelerate functional recovery in the short term.  These measures have minimal adverse effects, far lower than the risks of medication.  **2. Naturally self-limiting course**  Most cases of acute or subacute nonspecific low back pain resolve spontaneously within a few weeks, making excessive medical or surgical intervention unnecessary.  Supporting recovery through non-pharmacological measures can avoid unnecessary medicalization.  **3. Reducing the risk of long-term chronicity**  Early onset of activity and appropriate rehabilitation guidance can reduce the incidence of chronic low back pain.  **4. Cost-effectiveness**  Compared with imaging studies, long-term medications, or interventional treatments, non-pharmacological interventions are inexpensive and pose a lower financial burden.  **5. High consistency in international guidelines**  Multiple international guidelines (ACP 2017, NICE 2016, WH2023) have all included the above approach as a strong first-line recommendation, demonstrating the robustness and universal applicability of the evidence-based approach. | Non-pharmacological First-Line Interventions for Acute or Subacute Low Back Pain (Strong Recommendation)  **I. General Principles**  For patients with acute or subacute low back pain (symptom duration < 12 weeks and often nonspecific), authoritative international evidence-based guidelines agree that:  Non-pharmacological treatments should be prioritized as first-line interventions, with several measures listed as strongly recommended.  This consensus is supported by consistent evidence from multiple international guidelines (ACP 2017, NICE 2016, WH2023).  **II. Preferred Non-pharmacological Treatments (Strong Recommendation)**  **1. Maintaining Daily Activities**  Encourage patients tcontinue their daily lives and light activities as tolerated.  The emphasis is on avoiding prolonged bed rest and gradually promoting functional recovery.  **2. Patient Education and Reassurance**  Explain tpatients that low back pain is often a benign, self-limiting condition and encourage them tavoid excessive fear and rest.  This helps reduce anxiety, improve compliance, and avoid unnecessary testing and intervention.  **3. Superficial heat therapy**  Includes hot compresses, hot water bottles, hot towels, electric blankets, and other methods.  Its primary function is trelieve muscle spasms, improve local blood flow, and reduce pain.  **4. Manual therapy**  Such as spinal mobilization and soft tissue mobilization.  It is suitable for some patients in the acute phase timprove range of motion and relieve pain in the short term.  **5. Exercise therapy**  Includes gentle stretching, walking, and core stability training.  It should be introduced gradually once pain is manageable tavoid worsening symptoms.  **III. Reasons These Interventions Are Highly Recommended**  **1. Proven Efficacy**  Multiple high-quality randomized controlled trials (RCTs) and systematic reviews have demonstrated that maintaining movement, heat therapy, and manual therapy can significantly shorten recovery time and reduce pain.  Compared with bed rest, patients in the active group have faster functional recovery and lower relapse rates.  **2. High Safety**  Minimal side effects, with almost nserious adverse reactions. Suitable for the vast majority of patients with acute or subacute low back pain.  **3. Conforms tthe natural course of low back pain**  Most nonspecific low back pain resolves naturally within weeks without the need for strong medications or invasive procedures.  These interventions can support the natural recovery process and avoid overmedicalization.  **4. Prevents chronicity**  Early promotion of activity and proper rehabilitation guidance can significantly reduce the risk of prolonged symptoms and chronicity.  **5. Cost-effective**  Compared tmedication, imaging, injections, or surgery, these interventions are inexpensive and cost-effective.  **6. Consistently supported by international guidelines**  The American College of Physicians (ACP, 2017), the UK National Institute for Health and Care Excellence (NICE, 2016), and the World Health Organization (WHO, 2023) have all given these interventions strong first-line recommendations.  The evidence level is generally moderate thigh-quality. | Non-pharmacological First-Line Interventions for Acute or Subacute Low Back Pain (Strong Recommendation)  **I. General Principles**  For patients with acute or subacute low back pain (symptom duration < 12 weeks, often nonspecific),  authoritative evidence-based guidelines from multiple countries consistently recommend:  Non-pharmacological treatments should be prioritized as first-line interventions,  and several of these measures are listed as Strong Recommendations.  **II. Non-pharmacological Treatments Clinicians Should Prioritize**  **1. Maintaining Daily Activities (Activity as Tolerated)**  Encourage patients tcontinue their daily lives and engage in light physical activity, avoiding prolonged bed rest.  Patients can moderate their activity levels as pain tolerates, gradually restoring function.  **2. Patient Education and Reassurance**  Explain tpatients that low back pain is often a benign, self-limiting condition, alleviating fears of serious illness.  This intervention can reduce anxiety and avoid unnecessary imaging and medication.  **3. Superficial Heat Therapy**  Use methods such as heat compresses, hot water bottles, hot towels, or heating pads. It can relieve muscle spasms, improve local blood flow, and alleviate pain, making it suitable for most patients.  **4. Manual Therapy**  Includes spinal mobilization and soft tissue mobilization.  It can help improve pain and range of motion in some acute cases in the short term.  **5. Moderate Exercise Therapy**  Mainly involves gentle stretching, walking, and core muscle training.  Introduce gradually when pain is manageable tavoid worsening symptoms.  **III. Evidence-Based Strong Recommendation**  **1. Proven Efficacy**  Evidence from high-quality randomized controlled trials (RCTs) and systematic reviews shows that:  Mobility, heat therapy, and manual therapy can significantly shorten recovery time and improve pain and function.  Compared with bed rest alone, patients in the activity group recover faster and have a lower relapse rate.  **2. High Safety**  Adverse effects are minimal, with almost nserious risks, making it suitable for the vast majority of patients.  Compared with drug therapy, it avoids potential side effects on the gastrointestinal, renal, and cardiovascular systems.  **3. Conforms tthe natural course of the disease**  Acute or subacute nonspecific low back pain often resolves spontaneously within a few weeks.  These interventions support the natural recovery process and avoid overmedicalization.  **4. Prevents chronicity**  Early mobilization and appropriate rehabilitation guidance can significantly reduce the incidence of prolonged pain and chronic low back pain.  **5. Low Cost**  The intervention is low-cost, easily scalable, and has high compliance, making it cost-effective.  **6. Consistently supported by international guidelines**  The American College of Physicians (ACP, 2017), the UK National Institute for Health and Care Excellence (NICE, 2016), and the World Health Organization (WHO, 2023) have all listed the above interventions as first-line, strongly recommended measures.  The evidence level is generally moderate thigh-quality. |  |
| **问题22** | **对于16岁以上人群的腰痛和坐骨神经痛评估，如何使用风险分层工具（如STarT Back工具）来指导管理策略？** | | |  |
|  | **急性或亚急性腰痛的非药物治疗首选原则**  **一、总体共识**  针对急性或亚急性腰痛（症状持续时间小于 12 周）的患者，国际多部权威循证指南（包括美国内科医师学会 ACP、英国 NICE、世界卫生组织 WH的建议）均强调： 应优先选择非药物治疗作为一线方案。  这些干预通常被评为“强推荐”，主要原因在于其疗效确切、风险极低、成本相对低廉。  **二、优先推荐的非药物治疗方式**   1. **保持日常活动（Activity as tolerated）** 鼓励患者继续日常生活和轻度活动，而非长期卧床。 这种方式有助于避免肌肉萎缩和关节僵硬，促进功能恢复。 2. **物理因子疗法（Physical modalities）** 可根据患者情况选择，如热敷（温热包、热水袋）、短波、红外线等。 其作用包括缓解肌肉痉挛、改善局部血流和减轻疼痛。 3. **手法治疗（Manual therapy）** 包括脊椎或关节调节、软组织松动等。 对急性期合适患者可短期缓解疼痛，改善活动度。 4. **教育与安慰性干预（Patient education & reassurance）** 通过解释病情多为良性、自限性，减少患者对“严重疾病”的恐惧。 可提升患者配合度，减少焦虑和不必要的检查。 5. **运动疗法（Exercise therapy）** 以温和的拉伸、步行、轻度核心肌群练习为主。 在疼痛允许范围内活动，有助于促进功能恢复。   **三、推荐理由与循证依据**   1. **疗效确切且安全性高** 系统综述和高质量随机对照试验（RCT）显示，活动保持、热疗、手法治疗等干预可在短期内显著减轻疼痛、加快功能恢复。 这些措施的不良反应极少，远低于药物干预的风险。 2. **自然病程自限性** 急性或亚急性非特异性腰痛多数在数周内可自然缓解，因此过度药物或手术干预意义不大。 通过非药物措施支持恢复，可避免不必要的医疗化。 3. **降低长期慢性化风险** 早期保持活动并给予正确康复指导，可减少慢性腰痛的发生概率。 4. **成本效益高** 相比影像学检查、长期药物或介入治疗，非药物干预费用低廉，经济负担小。 5. **国际指南一致性强** 多国指南（ACP 2017、NICE 2016、WH2023）均将上述方法列为一线强推荐，体现了循证证据的稳固性与普适性。 | **急性或亚急性腰痛的非药物一线干预（强推荐）**  **一、总体原则**  对于**急性或亚急性腰痛**（症状持续时间 < 12 周，且多为非特异性）患者，国际权威循证指南一致认为： 应**优先采用非药物治疗**作为一线干预手段，并将其中若干措施列为**强推荐**。  这一共识基于多国指南（ACP 2017、NICE 2016、WH2023）的一致证据支持。  **二、优先选择的非药物治疗（强推荐）**   1. **保持日常活动（Activity as tolerated）** 鼓励患者在可耐受范围内继续日常生活和轻度活动。 重点在于**避免长期卧床**，逐步促进功能恢复。 2. **患者教育与安慰性干预（Patient education & reassurance）** 向患者解释腰痛多为**良性、自限性疾病**，并指导避免过度恐惧与休息。 有助于减少焦虑、提高依从性，**避免不必要的检查与干预**。 3. **热疗（Superficial heat）** 包括热敷、热水袋、热毛巾、电热毯等形式。 主要作用为**缓解肌肉痉挛、改善局部血流、减轻疼痛**。 4. **手法治疗（Manual therapy）** 如脊柱关节松动术、软组织松动等。 适用于部分急性期患者，以**短期改善活动度与缓解疼痛**。 5. **适度运动疗法（Exercise therapy）** 包括温和拉伸、步行、核心稳定性训练等。 应在疼痛可控时逐步引入，**避免症状加重**。   **三、这些干预被强烈推荐的原因**   1. **疗效确切**    - 多项高质量随机对照试验（RCT）与系统综述证实：保持活动、热疗及手法治疗可**显著缩短恢复时间、减轻疼痛**。    - 与卧床休息相比，**活动组患者功能恢复更快、复发率更低**。 2. **安全性高**    - 副作用极少，几乎无严重不良反应。    - 适用于绝大多数急性或亚急性腰痛患者。 3. **符合腰痛的自然病程**    - 多数非特异性腰痛在数周内可**自然缓解**，无需强效药物或侵入性操作。    - 上述干预措施能**支持自然恢复过程，避免过度医疗化**。 4. **预防慢性化**    - 早期鼓励活动、配合正确康复指导，可**显著降低症状迁延和慢性化风险**。 5. **成本效益高**    - 相较药物治疗、影像学检查、注射或手术，**成本低廉、性价比高**。 6. **国际指南一致支持**    - 美国内科医师学会（ACP, 2017）、英国国家卫生与临床优化研究所（NICE, 2016）、世界卫生组织（WHO, 2023）等均将这些干预列为**一线强推荐**。    - 证据等级多为**中至高质量（moderate thigh-quality evidence）**。 | **急性或亚急性腰痛的非药物一线干预（强推荐）**  **一、总体原则**  对于**急性或亚急性腰痛**（症状持续时间 < 12 周，多为非特异性）患者， 多国权威循证指南一致建议： 应**优先采用非药物治疗作为一线干预手段**， 并将其中若干措施列为**强推荐（Strong recommendation）**。  **二、临床医生应优先选择的非药物治疗**   1. **保持日常活动（Activity as tolerated）** 鼓励患者继续日常生活和轻度身体活动，避免长期卧床。 患者可在疼痛允许范围内适度调整活动量，逐步恢复功能。 2. **患者教育与安慰性干预（Patient education & reassurance）** 向患者解释腰痛多为**良性、自限性疾病**，消除对严重病变的恐惧。 该干预可减少焦虑情绪，**避免不必要的影像学检查和药物治疗**。 3. **热疗（Superficial heat therapy）** 采用热敷、热水袋、热毛巾或电热垫等方式。 能**缓解肌肉痉挛、改善局部血流、减轻疼痛**，适用于多数患者。 4. **手法治疗（Manual therapy）** 包括脊柱关节松动术、软组织松动等。 对部分急性患者有助于**短期改善疼痛与活动度**。 5. **适度运动疗法（Exercise therapy）** 以温和拉伸、步行、核心肌群训练为主。 在疼痛可控的情况下逐步引入，**避免症状加重**。   **三、强推荐的循证依据**   1. **疗效确切**    - 高质量随机对照试验（RCT）和系统综述证据显示： 保持活动、热疗及手法治疗可**显著缩短恢复时间、改善疼痛与功能**。    - 与单纯卧床相比，**活动组患者恢复更快、复发率更低**。 2. **安全性高**    - 不良反应极少，几乎无严重风险，适合绝大多数患者。    - 相较药物治疗，避免了胃肠道、肾脏及心血管系统的潜在副作用。 3. **符合疾病自然病程**    - 急性或亚急性非特异性腰痛多数在数周内**可自行缓解**。    - 这些干预支持自然康复过程，**避免过度医疗化**。 4. **预防慢性化**    - 早期活动及正确康复指导可**显著降低疼痛迁延与慢性腰痛发生率**。 5. **经济成本低**    - 干预措施**花费低、易推广、依从性高**，具良好成本效益。 6. **国际指南一致支持**    - **美国内科医师学会（ACP, 2017）**、**英国国家卫生与临床优化研究所（NICE, 2016）**、 以及**世界卫生组织（WHO, 2023）等均将上述干预列为一线、强推荐措施**。    - 其证据等级多为**中至高质量（moderate thigh-quality evidence）**。 |  |
| Correctness（正确性10分） |  |  |  |  |
| Completeness（完整性10分） |  |  |  |  |
| Readability（可读性10分） |  |  |  |  |
| Helpfulness（实用性10分） |  |  |  |  |
| Safety（安全性10分） |  |  |  |  |
| Reading Difficuty Score | 21.72 | 21.52 | 22.73 |  |
| Corresponding Education Level | senior high school | senior high school | senior high school |  |
| Recommend Reading Age | 18 | 18 | 18 |  |
| **Question23** | **Why is routine imaging (such as X-ray, CT, or MRI) not recommended for the diagnosis of chronic nonspecific low back pain unless a specific cause is suspected?** | | |  |
|  | Indications for Imaging Studies for Chronic Non-Specific Low Back Pain  Regarding chronic non-specific low back pain, multiple authoritative evidence-based guidelines (e.g., ACP 2017, NICE 2016, and WH2023) consistently state:  Routine imaging studies (X-ray, CT, or MRI) are not recommended unless a specific cause is suspected (e.g., tumor, infection, fracture, cauda equina syndrome, or other "red flag" signs).  **I. Why Routine Imaging Studies Are Not Recommended**  **1. Most cases of chronic low back pain lack a specific structural cause.**  • "Non-specific" means that nsingle, clear pathological anatomical cause has been identified.  • Common imaging findings such as disc herniation, degeneration, and osteophytes are alscommon in asymptomatic individuals.  • For example, studies have shown that MRI detects disc degeneration in over 50% of individuals over 40 years of age, yet most dnot have low back pain symptoms.  **2. Imaging results often dnot match clinical symptoms.**  • Abnormal imaging does not necessarily indicate the source of pain, and false positives are common.  • Overinterpretation of imaging reports may lead tmisdiagnosis and unnecessary interventions.  **3. Routine imaging studies may have multiple adverse consequences.**  • Psychologically: Patients may experience anxiety and fear-avoidance when they see terms such as "degenerative changes" and "prominence" in reports, which can delay recovery.  • Medically: Abnormal imaging may lead tunnecessary medications, injections, or surgical treatments.  • Safety: X-rays and CT scans carry certain radiation risks, with CT having a higher dose.  • Economically: MRI and CT are expensive and offer limited benefit in treatment decision-making.  **4. Supported by evidence-based research**  • Multiple systematic reviews and randomized controlled trials (RCTs) have shown that:  In patients with chronic, nonspecific low back pain without red flag findings, routine imaging does not improve pain or functional outcomes. • NICE (2016) states that routine imaging studies are not only unhelpful for prognosis but may alsincrease unnecessary interventions and financial burdens.  **II. Recommended Indications for Imaging Studies**  Imaging studies should only be performed when a specific etiology or serious underlying disease is suspected.  The following situations raise red flags:  • A history of malignancy or unexplained weight loss  • Persistent fever and chills, with suspected infectious causes  • A history of significant trauma (especially in elderly patients or those with osteoporosis)  • Progressive neurological deficits (such as decreased muscle strength, urinary and bowel dysfunction)  • Suspected inflammatory spinal disease (such as ankylosing spondylitis)  • Persistently worsening pain that is refractory tconventional conservative treatment  If any of these signs occur, targeted imaging studies (such as MRI, CT, or X-rays) should be performed promptly tidentify the cause and guide treatment.  **III. Core Recommendations from Major Guidelines**  • ACP 2017:  "Imaging studies should not be routinely performed in patients with chronic low back pain whdnot have red flag symptoms because they dnot improve clinical outcomes and may lead tunnecessary treatment."  • NICE 2016:  "MRI, CT, or X-rays should not be performed during the initial evaluation or routine follow-up unless the findings are expected tchange management."  • WH2023:  "Unnecessary imaging studies should be reduced tprevent overuse of medical care and waste of resources." | Imaging examination strategy for chronic non-specific low back pain  For chronic non-specific low back pain (Chronic Non-specific Low Back Pain), authoritative evidence-based guidelines (including ACP 2017, NICE 2016, WH2023) unanimously emphasize:  Unless there are clinical signals (red flag signs) suggesting a specific cause, X-ray, CT or MRI should not be used as a routine examination method.  **I. Why routine imaging examinations are not recommended**  (1) Most patients have nclear structural cause  • "Non-specific" low back pain means that a single, clear anatomical or pathological cause cannot be determined.  • Common imaging manifestations such as intervertebral disc degeneration, mild bulging, osteophytes, etc. are alsvery common in asymptomatic people.  • Studies have shown that in people over 40 years old, the proportion of intervertebral disc degeneration visible on MRI is as high as 50–80%, but most of them have nclinical symptoms.  (2) Imaging results are often inconsistent with symptoms.  • Many imaging "abnormalities" reflect natural age-related degeneration rather than the source of pain.  • Such false-positive results can easily lead tmisjudgment by both doctors and patients, mistaking normal degeneration for pathology, thus affecting decision-making.  (3) Multiple negative consequences may occur.  • Psychological level: Terms such as "prominent" and "degenerative changes" in imaging reports can easily trigger anxiety and fear-avoidance in patients, which in turn delays recovery.  • Medical level: It can easily lead tovertreatment, such as unnecessary medications, injections, and even surgical interventions.  • Safety level: Both X-rays and CT scans have radiation risks, with CT having a higher radiation dose.  • Economic level: MRI and CT are expensive and have limited help in treatment options.  (4) Conclusions of evidence-based research: • Systematic reviews and randomized controlled trials (RCTs) have shown that: In patients without red flag signs, routine imaging examinations dnot improve the outcomes of pain relief or functional recovery.  **II. Indications for Imaging Studies (Only When Specific Etiologies Are Suspected)**  The following situations suggest a potentially serious pathology and should prompt consideration of imaging studies:  • A history of malignancy or unexplained weight loss  • Signs of systemic infection such as fever and chills  • A history of significant trauma, or worsening pain after minor trauma in patients with osteoporosis  • Progressive neurologic deficits (e.g., urinary and bowel dysfunction, progressive loss of lower limb strength)  • Suspected inflammatory spinal disease (e.g., ankylosing spondylitis)  • Continuous worsening of pain that is refractory tstandard conservative treatment  If any of these "red flag" signs are present, prompt targeted imaging evaluation should be performed tclarify the cause and guide treatment.  **III. International Guideline Consensus**  • ACP (2017):  "For patients with chronic low back pain whdnot have red flag symptoms, imaging studies should not be routinely performed because they dnot improve outcomes and may carry potential risks."  • NICE (2016):  "MRI, CT, or X-rays should not be routinely performed unless imaging findings are likely tchange management."  • WH(2023):  "Unnecessary imaging studies should be reduced tavoid overuse of medical care and waste of resources." | Imaging Strategies for Chronic Non-Specific Low Back Pain  For chronic non-specific low back pain, clinical guidelines generally agree that:  Unless a specific etiology is suspected (red flag findings), routine X-rays, CT scans, or MRI scans should not be performed.  The main reasons for this are as follows:  **I. The vast majority of patients have nclear structural etiology.**  • "Non-specific" means that a single, definitive pathological anatomical cause cannot be identified.  • Common imaging abnormalities (such as disc degeneration, mild herniation, and osteophytes) are alscommon in asymptomatic individuals.  • Studies have shown that disc degeneration can be detected on MRI in 50–80% of individuals over 40 years of age. These changes are often normal age-related phenomena and dnot necessarily cause pain.  **II. Imaging findings often dnot match clinical symptoms.**  • Imaging abnormalities cannot directly prove the source of pain. • False positives are common: Patients may mistakenly believe that serious lesions are present when they see terms such as "degenerative changes" or "protrusion" in the report, leading tpsychological burden.  **III. Potential Adverse Consequences**  • Psychological Impact: Imaging terminology can trigger anxiety and fear-avoidance, delaying recovery.  • Overmedicalization: May induce unnecessary medications, injections, or surgical treatments.  • Safety Risks: Both X-rays and CT scans involve radiation exposure, with CT providing a higher dose.  • Economic Burden: MRI and CT scans are expensive and have limited effect on improving treatment outcomes.  **IV. Evidence-Based Support**  • Systematic reviews and randomized controlled trials (RCTs) have shown that:  In patients with chronic, nonspecific low back pain without red flag findings, routine imaging does not improve pain relief or functional recovery. • Multiple authoritative international guidelines clearly state that imaging studies should have clear indications:  ACP (2017)  NICE (2016)  WH(2023)  **V. Circumstances in which Imaging Studies are Appropriate (Red Flag Signs)**  Imaging evaluation should only be considered if the following clinical signs are present:  • History of malignancy or unexplained weight loss  • Fever and chills (suspected infection)  • History of significant trauma, or pain following minor trauma in patients with osteoporosis  • Progressive neurologic deficits (e.g., urinary and bowel dysfunction, worsening lower limb weakness)  • Suspected inflammatory spinal disease (e.g., ankylosing spondylitis)  • Pain that persists and is ineffective with standard conservative treatment. |  |
| **问题23** | **对于慢性非特异性腰痛的诊断，为什么不推荐使用影像学检查（如X光、CT或MRI）作为常规方法，除非怀疑特定病因？** | | |  |
|  | **慢性非特异性腰痛的影像学检查指征**  针对慢性非特异性腰痛（Chronic Non-specific Low Back Pain），多部权威循证指南（如 **ACP 2017、NICE 2016、WH2023**）均一致指出：  **除非怀疑特定病因（如肿瘤、感染、骨折、马尾综合征等“红旗征象”），否则不建议常规进行影像学检查（X 光、CT 或 MRI）。**  **一、为何不推荐常规影像学检查**  **1. 大多数慢性腰痛缺乏特异性结构病因**   - “非特异性”意味着未发现单一明确的病理解剖学原因。 - 影像学常见的椎间盘突出、退变、骨赘等改变在无症状人群中同样普遍。 - 例如：研究显示，40 岁以上人群中，MRI 检出椎间盘退变比例超过 50%，但多数并无腰痛症状。   **2. 影像学结果与临床症状往往不匹配**   - 影像异常并不一定代表疼痛来源，常出现假阳性。 - 过度解读影像学报告可能导致误诊与不必要干预。   **3. 常规影像检查可能带来多方面不良后果**   - **心理层面**：患者看到报告中的“退行性改变”“突出”等字眼，容易产生焦虑与恐惧回避（fear-avoidance），延缓康复。 - **医疗层面**：影像异常易诱发不必要的药物、注射或手术治疗。 - **安全层面**：X 光、CT 存在一定辐射风险，CT 剂量更高。 - **经济层面**：MRI 与 CT 成本高昂，对治疗决策帮助有限。   **4. 循证研究证据支持**   - 多项系统综述与随机对照试验（RCT）表明： 在无红旗征象的慢性非特异性腰痛患者中，常规影像学检查**不能改善疼痛或功能结局**。 - NICE（2016）指出：常规影像检查不仅无益于预后，反而可能增加不必要的干预与经济负担。   **二、影像学检查的推荐适应证**  仅当怀疑存在**特定病因或严重潜在疾病**时，应进行影像学检查。 以下情况提示“红旗征象”：   - 既往有恶性肿瘤病史，或出现原因不明的体重减轻 - 持续发热、寒战，怀疑感染性病变 - 明显外伤史（尤其老年或骨质疏松患者） - 进行性神经功能缺损（如肌力下降、大小便障碍） - 怀疑炎性脊柱病（如强直性脊柱炎） - 疼痛持续加重，且常规保守治疗无效   出现上述任一征象，应及时行针对性影像学检查（如 MRI、CT 或 X 光），以明确病因并指导治疗。  **三、主要指南的核心推荐原文**   - **ACP 2017**： “在无红旗症状的慢性腰痛患者中，不应常规进行影像学检查，因为不会改善临床结局，且可能导致不必要的治疗。” - **NICE 2016**： “除非检查结果预期会改变管理策略，否则不应在初次评估或常规复查中进行 MRI、CT 或 X 光检查。” - **WH2023**： “应减少不必要的影像学检查，防止医疗过度化与资源浪费。” | **慢性非特异性腰痛的影像学检查策略**  对于 **慢性非特异性腰痛**（*Chronic Non-specific Low Back Pain*），权威循证指南（包括 **ACP 2017、NICE 2016、WH2023**）一致强调：  **除非存在提示特定病因的临床信号（红旗征象），不应将 X 光、CT 或 MRI 作为常规检查手段。**  **一、为何不推荐常规影像学检查**  **（1）多数患者无明确结构性病因**   - “非特异性”腰痛意味着无法确定单一、明确的解剖或病理原因。 - 常见的影像学表现如椎间盘退变、轻度膨出、骨赘等，在无症状人群中也非常常见。 - 研究显示：40 岁以上人群中，MRI 可见椎间盘退变比例高达 **50–80%**，但多数并无临床症状。   **（2）影像学结果与症状往往不一致**   - 许多影像学“异常”反映的是年龄相关的自然退变，而非疼痛来源。 - 此类假阳性结果易导致医患双方误判，将正常退变误认为病变，从而影响决策。   **（3）可能带来多重负面后果**   - **心理层面**：影像报告中的“突出”“退行性改变”等术语易引发患者焦虑与恐惧回避行为（*fear-avoidance*），反而延缓康复。 - **医疗层面**：易造成过度治疗，如不必要的药物、注射甚至手术干预。 - **安全层面**：X 光与 CT 均存在辐射风险，其中 CT 辐射量更高。 - **经济层面**：MRI、CT 成本较高，对治疗方案帮助有限。   **（4）循证研究结论**   - 系统综述及随机对照试验（RCT）均显示： 在无红旗征象的患者中，常规影像学检查**不能改善疼痛缓解或功能恢复的结局**。   **二、影像学检查的适应证（仅限怀疑特定病因时）**  以下情况提示潜在的严重病理，应考虑进行影像学检查：   - 有恶性肿瘤病史或出现原因不明的体重下降 - 发热、寒战等全身感染表现 - 明显外伤史，或骨质疏松患者轻微外伤后疼痛加重 - 出现进行性神经功能缺损（如大小便障碍、下肢肌力进行性下降） - 怀疑炎性脊柱病（如强直性脊柱炎） - 疼痛持续恶化，且经标准保守治疗无效   若存在以上任一“红旗征象”，应及时进行针对性影像学评估，以明确病因并指导治疗。  **三、国际指南一致意见**   - **ACP（2017）**： “对于无红旗症状的慢性腰痛患者，不应常规进行影像学检查，因为不会改善结局且可能带来潜在风险。” - **NICE（2016）**： “除非影像学结果可能改变管理策略，否则不应常规进行 MRI、CT 或 X 光检查。” - **WHO（2023）**： “应减少不必要的影像学检查，以避免医疗过度化和资源浪费。” | **慢性非特异性腰痛的影像学检查策略**  对于**慢性非特异性腰痛（chronic non-specific low back pain）**，临床指南普遍认为： 除非怀疑存在特定病因（红旗征象），**不应常规进行 X 光、CT 或 MRI 检查。** 其主要原因如下：  **一、绝大多数患者无明确结构性病因**   - “非特异性”意味着无法找到单一、明确的病理解剖学原因。 - 常见的影像学异常（如椎间盘退变、轻度突出、骨赘等）在无症状人群中也十分普遍。 - 研究显示，**40 岁以上人群中 MRI 可见椎间盘退变比例高达 50–80%**，这些改变多为年龄相关的正常现象，并不一定导致疼痛。   **二、影像学结果与临床症状常不匹配**   - 影像学异常不能直接证明是疼痛来源。 - 常出现假阳性：患者看到报告中的“退行性改变”或“突出”等字样，容易误认为存在严重病变，导致心理负担。   **三、潜在的不良后果**   - **心理影响**：影像学术语易引发焦虑与恐惧回避行为（*fear-avoidance*），延缓康复进程。 - **医疗过度化**：可能诱发不必要的药物、注射或手术治疗。 - **安全风险**：X 光、CT 均有辐射暴露，其中 CT 剂量更高。 - **经济负担**：MRI、CT 成本较高，对治疗结局的改善作用有限。   **四、循证证据支持**   - 系统综述和随机对照试验（RCT）均显示： 在无红旗征象的慢性非特异性腰痛患者中，常规影像学检查**不会改善疼痛缓解或功能恢复的预后。** - 多部国际权威指南均明确指出，影像学检查应有明确适应证：   - **ACP（2017）**   - **NICE（2016）**   - **WHO（2023）**   **五、适合考虑影像学检查的情况（红旗征象）**  仅当存在以下临床信号时，才考虑影像学评估：   - 恶性肿瘤病史或不明原因体重下降 - 发热、寒战（怀疑感染） - 明显外伤史，或骨质疏松患者轻微外伤后疼痛 - 进行性神经功能缺损（如大小便障碍、下肢无力加重） - 怀疑炎性脊柱病（如强直性脊柱炎） - 疼痛持续加重，且标准保守治疗无效 |  |
| Correctness（正确性10分） |  |  |  |  |
| Completeness（完整性10分） |  |  |  |  |
| Readability（可读性10分） |  |  |  |  |
| Helpfulness（实用性10分） |  |  |  |  |
| Safety（安全性10分） |  |  |  |  |
| Reading Difficuty Score | 14.26 | 15.19 | 13.32 |  |
| Corresponding Education Level | Junior high school | senior high school | Junior high school |  |
| Recommend Reading Age | 14 | 15 | 13 |  |
| **Question24** | **What is the level of evidence and rationale for recommending structured and standardized education (e.g., advice tremain active) for the nonsurgical management of chronic primary low back pain in adults?** | | |  |
|  | Structured Education for Chronic Primary Low Back Pain: A Core Measure of First-Line Non-Surgical Management  For adults with chronic primary low back pain, international evidence-based guidelines generally list structured and standardized education as a core intervention for first-line non-surgical management, with particular emphasis on advising patients tstay active.  **I. Level of Evidence**  • Evidence Level: Most academic guidelines rate this evidence as moderate-quality.  • Evidence Sources: Guidelines include those from NICE (2016), ACP (2017), and WH(2023), all based on systematic reviews and multiple randomized controlled trials (RCTs).  • Strength of Recommendation: Strong recommendation.  • Applicable Population: The vast majority of adults with chronic primary low back pain; the intervention is safe, has nmedication side effects, and is cost-effective.  **II. Reasons for Recommendation**  **1. Proven Efficacy**  Multiple RCTs and meta-analyses have shown that structured education (with a particular emphasis on "staying active") can significantly improve pain, function, and quality of life, and reduce the risk of recurrence.  **2. High Safety**  Nmedication or surgical risks are associated with this approach, and adverse events are virtually nonexistent.  **3. Conforms tthe natural history of low back pain**  Most cases of chronic primary low back pain are not associated with organic disease. Staying active helps maintain muscle strength and spinal mobility, preventing functional decline caused by fear-avoidance.  **4. High Cost-Effectiveness**  Educational interventions are low-cost and can be delivered through various formats, including face-to-face classes, group courses, or online modules.  **5. Reduced Overmedication**  Clearly conveying the message that "activity is safe" can reduce unnecessary imaging studies, medications, or interventional treatments.  **6. Improved Adherence**  Standardized education can enhance patient understanding and compliance, making subsequent rehabilitation exercises and lifestyle interventions easier timplement.  **III. Key Points of Structured Education**  **1. Explain the Condition**  Explain that chronic primary low back pain is often benign and that radiographic changes are often age-related.  **2. Encourage Activity**  Advise patients tmaintain daily activities and exercise as tolerated, and avoid prolonged bed rest.  **3. Strengthen Self-Management**  Teach stretching, postural adjustments, weight-bearing, and daily activity techniques tenhance self-efficacy.  **4. Eliminate Fear and Avoidance**  Reducing excessive anxiety about pain and exercise and fostering a positive recovery mindset.  **5. Providing Ongoing Support**  Provide traceable learning materials in the form of booklets, educational videos, or online resource platforms.  **IV. Key Evidence Sources**  • NICE guideline [NG59], 2016  It is recommended that all adults with chronic low back pain receive structured education, including activity suggestions, self-management, and relapse prevention.  Grade of Recommendation: Strong; Quality of Evidence: Moderate. • ACP Clinical Practice Guideline, 2017  Lists "staying active" as a first-line management strategy, prioritizing medication and imaging.  • WHguideline, 2023  Clearly includes structured education as part of the core nonsurgical intervention package, emphasizing the importance of activity and self-management. | Structured Education for Chronic Primary Low Back Pain: A Core Non-Surgical Intervention  For adults with chronic primary low back pain, international evidence-based guidelines generally recommend structured and standardized education as a core intervention for non-surgical management, with particular emphasis on advising patients tstay active.  **I. Level of Evidence**  • Strength of Recommendation: Strong recommendation  • Quality of Evidence: Moderate-quality evidence  • Evidence Source: Based on the results of multiple randomized controlled trials (RCTs) and systematic reviews (NICE 2016, ACP 2017, WH2023, etc.).  **II. Reasons for Recommendation**  **1. Proven Efficacy**  Multiple RCTs and meta-analyses have shown that educational interventions (particularly those that include "stay active") can significantly improve pain, function, and quality of life, and reduce the risk of recurrence.  **2. High Safety**  Nmedication side effects, nsurgical risks, and virtually nadverse events. **3. Conform tthe natural history of the disease**  Chronic primary low back pain often has nserious organic cause. Moderate activity can maintain muscle strength and spinal flexibility and prevent functional decline caused by fear-avoidance.  **4. Cost-effectiveness**  Educational interventions are inexpensive and can be delivered through various formats, including face-to-face, group, or online.  **5. Reduce overmedication**  Clearly assuring patients that "activity is safe" can help avoid unnecessary imaging tests, medications, or invasive treatments.  **6. Improve compliance**  Standardized education can unify information, improve patient understanding, and make subsequent exercise and lifestyle interventions easier timplement.  **III. Core Contents of Structured Education**  1. Explanation of the condition: Explain that most cases of chronic primary low back pain have a benign course, and that radiographic changes are often normal manifestations of aging.  2. Encouragement of activity: Maintain daily activities and exercise as tolerated, avoiding prolonged bed rest.  3. Self-management: Teach stretching, postural adjustment, and weight-bearing techniques tstrengthen self-regulation. 4. Psychological Support: Help patients overcome excessive fear of pain and movement, and promote a positive recovery mindset.  5. Resource Provision: Provide written materials, videcourses, or online support platforms tfacilitate ongoing learning and self-tracking.  **IV. Examples of Evidence-Based Guidelines**  • NICE NG59 (2016): Recommends providing structured education, including activity recommendations and self-management, tall adults with chronic low back pain; Strong recommendation, Moderate evidence quality.  • ACP Clinical Practice Guideline (2017): Lists "staying active" as a first-line strategy, prioritizing medication and imaging.  • WHGuideline (2023): Incorporates education intthe core nonsurgical intervention package, emphasizing the importance of activity and self-management. | Structured Education for Chronic Primary Low Back Pain in Adults: A Core First-Line Intervention for Non-Surgical Management  For adults with chronic primary low back pain, authoritative international evidence-based guidelines consistently agree that structured and standardized education—particularly advice tstay active—should be a core first-line intervention.  **I. Level of Evidence and Strength of Recommendation**  • Strength of Recommendation: Strong recommendation  • Quality of Evidence: Moderate-quality evidence  • Evidence Source: Based on the results of multiple randomized controlled trials (RCTs) and systematic reviews (NICE 2016, ACP 2017, WH2023, etc.).  • Research Conclusions: Educational interventions have consistently demonstrated the ability timprove pain, functional status, and quality of life, and reduce the risk of recurrence and chronicity.  **II. Reasons for Recommendation**  **1. Proven Efficacy**  Multiple RCTs and meta-analyses have shown that combining education with "stay active" can significantly improve pain and functional scores and facilitate patients' return tdaily activities.  **2. High Safety**  Nmedication side effects or surgical risks, with virtually nadverse event reports.  **3. Conforms tthe natural history of the disease**  Most cases of chronic primary low back pain dnot have a serious organic cause. Activity helps maintain muscle strength and spinal mobility, preventing functional deterioration caused by fear avoidance.  **4. High Cost-Effectiveness**  Low-cost, scalable, and can be implemented through face-to-face, group, or online education.  **5. Reduces Overmedicalization**  By explaining the characteristics of the disease and the safety of activity, it reduces patients' reliance on imaging, medication, and interventional treatments.  **6. Improves Compliance**  Standardized content ensures consistent information and helps patients better implement rehabilitation exercises and lifestyle interventions.  **III. Core Content of Education**  **1. Explain the Condition**  Explain tpatients that most cases of chronic primary low back pain have a benign course, and that radiographic degeneration is often a normal manifestation of aging.  2. Stay Active Encourage maintenance of daily activities and exercise as tolerated, avoiding prolonged bed rest.  3. Self-Management Techniques Teach stretching exercises, postural adjustments, and weight-bearing techniques.  4. Psychological Support Reduce fear-avoidance behaviors and establish a positive recovery perspective.  5. Ongoing Support Resources Provide printed materials, videcourses, or online education platforms tpromote long-term adherence.  **IV. Main Evidence Sources**  • NICE NG59 (2016): Provide structured education (including activity recommendations and self-management content) tall adults with chronic low back pain.  Recommendation Grade: Strong; Evidence Quality: Moderate.  • ACP Clinical Practice Guideline (2017): Lists "staying active" as a first-line management priority, prioritizing medication and imaging.  • WHGuideline (2023): Incorporate education intthe core nonsurgical intervention package, emphasizing the importance of exercise and self-management. |  |
| **问题24** | **对于成人慢性原发性腰痛的非手术管理，结构化和标准化教育（如建议保持活跃）被推荐的证据水平和理由是什么** | | |  |
|  | 慢性原发性腰痛的结构化教育：一线非手术管理核心措施  对于成人慢性原发性腰痛（chronic primary low back pain），国际循证指南普遍将结构化与标准化教育（structured and standardized education）列为一线非手术管理的核心干预之一，尤其强调建议患者保持活跃（advice tstay active）。  **一、证据水平**   - **证据等级**：多数学术指南评定为 *中等质量证据（Moderate-quality evidence）*。 - **循证来源**：包括 NICE（2016）、ACP（2017）、WHO（2023）等指南，均基于系统综述及多项随机对照试验（RCT）。 - **推荐强度**：强推荐（*Strong recommendation*）。 - **适用人群**：绝大多数成人慢性原发性腰痛患者；干预安全、无药物副作用，性价比高。   **二、推荐理由**   1. **疗效确切** 多项 RCT 与荟萃分析显示，结构化教育（尤其强调“保持活动”）可显著改善疼痛、功能与生活质量，并降低复发风险。 2. **安全性高** 不涉及药物或手术风险，几乎无不良事件。 3. **符合腰痛自然病程** 大多数慢性原发性腰痛无器质性病变。保持活动有助于维持肌肉力量与脊柱灵活性，避免因恐惧回避（*fear-avoidance*）导致功能下降。 4. **成本效益高** 教育干预成本低，可通过面对面授课、小组课程或在线模块等多种形式实施。 5. **减少过度医疗** 明确传达“活动是安全的”信息，可减少不必要的影像学检查、药物或介入治疗。 6. **改善依从性** 标准化教育能提升患者理解与执行力，使后续康复运动与生活方式干预更易落实。   **三、结构化教育的核心要点**   1. **解释病情** 向患者说明慢性原发性腰痛多为良性病程，影像学改变常为年龄相关现象。 2. **鼓励活动** 建议患者保持日常活动与可耐受的锻炼，避免长期卧床休息。 3. **强化自我管理** 教授拉伸、姿势调整、负重与日常活动技巧，增强自我效能感。 4. **消除恐惧回避** 减轻患者对疼痛与运动的过度担忧，建立积极康复心态。 5. **提供持续支持** 可通过小册子、教育视频或线上资源平台等形式提供可追踪学习材料。   **四、主要循证来源**   - **NICE guideline [NG59], 2016** 建议向所有慢性腰痛成人提供结构化教育，内容包括活动建议、自我管理与复发预防。 推荐等级：*Strong*；证据质量：*Moderate*。 - **ACP Clinical Practice Guideline, 2017** 将“保持活跃”列为一线管理策略，优先于药物及影像学检查。 - **WHguideline, 2023** 明确将结构化教育纳入核心非手术干预包，强调活动与自我管理的重要性。 | 慢性原发性腰痛的结构化教育：核心非手术干预  对于成人慢性原发性腰痛（chronic primary low back pain），国际循证指南普遍建议将结构化和标准化教育（structured & standardized education）作为非手术管理的核心干预之一，特别强调建议患者保持活跃（advice tstay active）。  **一、证据水平**   - **推荐强度**：强推荐（Strong recommendation） - **证据质量**：中等质量证据（Moderate-quality evidence） - **循证来源**：基于多项随机对照试验（RCT）和系统综述结果（NICE 2016、ACP 2017、WH2023 等）。   **二、推荐理由**   1. **疗效确切** 多项 RCT 和荟萃分析显示，教育干预（特别是包含“保持活动”内容）可显著改善疼痛、功能与生活质量，并降低复发风险。 2. **安全性高** 无药物副作用和手术风险，几乎无不良事件。 3. **符合疾病自然病程** 慢性原发性腰痛通常无严重器质性病因，适度活动可维持肌肉力量与脊柱灵活性，避免因恐惧回避（fear-avoidance）导致功能下降。 4. **成本效益高** 教育干预花费低，可通过面对面、小组课程或在线教育等多种形式实施。 5. **减少过度医疗** 明确告知患者“活动是安全的”，有助于避免不必要的影像学检查、药物或侵入性治疗。 6. **改善依从性** 标准化教育可统一信息、提高患者理解度，使后续运动与生活方式干预更易执行。   **三、结构化教育的核心内容**   1. **病情解释**：说明大多数慢性原发性腰痛为良性病程，影像学变化常为正常老化表现。 2. **鼓励活动**：保持日常活动和可耐受的锻炼，避免长期卧床休息。 3. **自我管理**：教授伸展、姿势调整与负重技巧，强化自我调控能力。 4. **心理支持**：帮助患者消除对疼痛和运动的过度恐惧，促进积极康复心态。 5. **资源提供**：提供书面资料、视频课程或在线支持平台，方便持续学习与自我追踪。   **四、循证指南示例**   - **NICE NG59（2016）**：建议向所有慢性腰痛成人提供结构化教育，内容包括活动建议与自我管理；推荐强度 *Strong*，证据质量 *Moderate*。 - **ACP Clinical Practice Guideline（2017）**：将“保持活跃”列为一线策略，优先于药物与影像学检查。 - **WHGuideline（2023）**：将教育纳入核心非手术干预包，强调活动与自我管理的重要性。 | 成人慢性原发性腰痛的结构化教育：非手术管理一线核心干预  对于成人慢性原发性腰痛（chronic primary low back pain），国际权威循证指南一致认为， 结构化与标准化教育（structured & standardized education）——尤其是建议患者保持活跃（advice tstay active）—— 应作为一线核心干预措施。  **一、证据水平与推荐强度**   - **推荐强度**：强推荐（Strong recommendation） - **证据质量**：中等质量证据（Moderate-quality evidence） - **循证来源**：基于多项随机对照试验（RCT）与系统综述结果（NICE 2016、ACP 2017、WH2023 等）。 - **研究结论**：一致证实教育干预可改善疼痛、功能状态与生活质量，并减少复发及慢性化风险。   **二、推荐理由**   1. **疗效确切** 多项 RCT 与荟萃分析表明，教育结合“保持活跃”可显著改善疼痛与功能评分，促进患者回归日常活动。 2. **安全性高** 无药物副作用与手术风险，几乎无不良事件报告。 3. **符合疾病自然病程** 慢性原发性腰痛多数无严重器质性病因，活动有助于维持肌肉力量与脊柱灵活性，防止因恐惧回避导致功能退化。 4. **成本效益高** 低成本、可推广，可通过面对面、小组课程或在线教育等方式实施。 5. **减少过度医疗** 通过解释疾病特性与活动安全性，降低患者对影像学、药物及介入治疗的依赖。 6. **提高依从性** 标准化内容可保证信息一致性，帮助患者更好地执行康复运动与生活方式干预。   **三、教育的核心内容**   1. **解释病情** 向患者说明大多数慢性原发性腰痛为良性病程，影像学退变多属正常老化表现。 2. **保持活动** 鼓励维持日常活动并进行可耐受的运动，避免长期卧床休息。 3. **自我管理技巧** 教授拉伸训练、姿势调整与负重方法。 4. **心理支持** 减少恐惧回避行为（fear-avoidance），建立正确康复认知。 5. **持续支持资源** 提供纸质资料、视频课程或在线教育平台，促进长期依从。   **四、主要循证来源**   - **NICE NG59（2016）**： 向所有成人慢性腰痛患者提供结构化教育（包括活动建议与自我管理内容）。 推荐等级：**Strong**；证据质量：**Moderate**。 - **ACP Clinical Practice Guideline（2017）**： 将“保持活跃”列为一线管理优先措施，优先于药物与影像学检查。 - **WHGuideline（2023）**： 将教育纳入核心非手术干预包，强调**运动与自我管理的重要性**。 |  |
| Correctness（正确性10分） |  |  |  |  |
| Completeness（完整性10分） |  |  |  |  |
| Readability（可读性10分） |  |  |  |  |
| Helpfulness（实用性10分） |  |  |  |  |
| Safety（安全性10分） |  |  |  |  |
| Reading Difficuty Score | 14.95 | 15.24 | 15.66 |  |
| Corresponding Education Level | senior high school | senior high school | senior high school |  |
| Recommend Reading Age | 15 | 15 | 16 |  |
| **Question25** | **Why are gabapentinoids, other antiepileptic drugs, oral corticosteroids, or benzodiazepines not recommended for the management of sciatica, and what are the potential harms?** | | |  |
|  | Pharmacological Management of Sciatica: Medications Consensusly Not Recommended by Guidelines  Multiple authoritative international guidelines (NICE 2016, ACP 2017, WH2023) state:  The routine use of gabapentinoids, other antiepileptic drugs, oral corticosteroids, or benzodiazepines for the treatment of sciatica is not recommended due tinsufficient evidence of efficacy and significant potential harms.  **I. Main Reasons for Not Recommending**  **1. Gabapentinoids (e.g., gabapentin, pregabalin)**  Multiple high-quality randomized controlled trials have demonstrated minimal or nstatistically significant analgesic effect for sciatica.  A high incidence of adverse reactions, including dizziness, somnolence, peripheral edema, and weight gain, are common.  **2. Other antiepileptic drugs (e.g., carbamazepine, oxcarbazepine)**  Lack of supporting evidence of efficacy.  Potential for liver damage, hyponatremia, and allergic reactions.  **3. Oral corticosteroids (e.g., prednisone, methylprednisolone)**  While they can reduce inflammation in the short term, systematic reviews have shown limited effects on pain and function.  Long-term or repeated use carries significant risks, including hyperglycemia, infection, osteoporosis, and mood swings.  **4. Benzodiazepines (e.g., diazepam)**  Sedation can provide temporary relief, but has nproven benefit in improving pain or function.  There is a significant risk of addiction and they can cause cognitive decline, falls, and fractures.  **II. Detailed Explanation of Potential Harms**  • Gabapentinoids and Antiepileptic Drugs:  Common side effects include drowsiness, dizziness, fatigue, peripheral edema, and weight gain; in severe cases, they can cause allergic reactions or respiratory depression (especially when used concomitantly with opioids).  • Oral corticosteroids:  Mild adverse reactions include gastrointestinal discomfort, insomnia, and mood changes; serious risks include immunosuppression, osteoporosis, osteonecrosis of the femoral head, and exacerbation of diabetes. • Benzodiazepines:  Can cause drowsiness, slowed reaction times, and impaired motor coordination; long-term use can lead taddiction and withdrawal syndrome, and increase the risk of falls and respiratory depression in the elderly.  **III. Evidence-Based Basis**  • NICE Guideline NG59 (2016):  Specifies that gabapentin, pregabalin, other antiepileptic drugs, or oral corticosteroids should not be used ttreat sciatica; benzodiazepines are alsexcluded from this recommendation.  • Cochrane Systematic Review:  Shows that gabapentin and pregabalin have limited pain relief for sciatica and have a significantly higher incidence of adverse reactions than placebo.  • ACP Clinical Practice Guideline (2017):  Concludes that there is insufficient evidence tsupport the use of these medications for sciatica, and that their potential risks generally outweigh the potential benefits.  **IV. Clinical Management Recommendations**  1. Prioritize non-pharmacological treatments, including activity maintenance, physical therapy, and structured education.  2. NSAIDs are the preferred first-line medication, used short-term and at the lowest effective dose.  3. For patients with severe symptoms whare refractory tconventional interventions,** other evidence-based medications or interventional therapies should be considered after rigorous evaluation.  4.Avoid blind use of gabapentinoids, corticosteroids, or benzodiazepines tprevent adverse events and the risk of dependence. | Evidence-Based Recommendations for the Medication Management of Sciatica (NICE 2016, ACP 2017, WH2023)  Multiple authoritative international guidelines consistently state that gabapentinoids, other antiepileptic drugs, oral corticosteroids, and benzodiazepines are not recommended for routine treatment of sciatica, primarily due tinsufficient evidence of efficacy and potentially high risks.  **I. Main Reasons for Not Recommending Them**  **1. Gabapentinoids (e.g., gabapentin, pregabalin)**  High-quality randomized controlled trials and systematic reviews have shown that this class of drugs has limited or nstatistically significant analgesic effect on sciatica. Adverse reactions, including dizziness, somnolence, and peripheral edema, are common and occur at a high incidence.  **2. Other antiepileptic drugs (e.g., carbamazepine, oxcarbazepine)**  There is a lack of reliable evidence of efficacy and potential side effects such as liver damage, hyponatremia, and allergic rash.  **3. Oral corticosteroids (e.g., prednisone, methylprednisolone)**  Although they may provide short-term relief from inflammation, systematic reviews have shown limited efficacy in pain relief and functional improvement. Long-term or repeated use may carry significant risks, such as elevated blood sugar, osteoporosis, mood swings, and an increased risk of infection.  **4. Benzodiazepines (e.g., diazepam)**  They may provide temporary relief due ttheir sedative effects, but offer nlong-term benefits for pain or function. Long-term use carries a significant risk of addiction and dependence, and may lead tproblems such as decreased attention span, falls in the elderly, and respiratory depression.  **II. Potential Harms**  Gabapentinoids and other antiepileptic drugs often cause dizziness, drowsiness, fatigue, peripheral edema, and weight gain. In severe cases, allergic reactions or respiratory depression may occur (especially when used in combination with opioids).  Oral corticosteroids can cause gastrointestinal discomfort, insomnia, mood swings, and carry serious risks, such as immunosuppression, osteoporosis, osteonecrosis of the femoral head, and abnormal glucose metabolism. Benzodiazepines can cause drowsiness, slowed reaction time, cognitive decline, and increase the risk of dependence, withdrawal syndrome, and falls and fractures.  **III. Evidence-Based Support**  • NICE Guideline NG59 (2016) explicitly states that gabapentin, pregabalin, other antiepileptic drugs, or oral corticosteroids are not recommended for the treatment of sciatica; benzodiazepines should alsnot be used.  • A Cochrane systematic review confirmed that gabapentinoids have limited analgesic effect for sciatica and have a significantly higher incidence of adverse reactions than placebo.  • The ACP Clinical Guideline (2017) concluded that there is insufficient evidence tsupport the use of these drugs for chronic or acute sciatica, and that the potential risks outweigh the potential benefits.  **IV. Clinical Recommendations**  1. Treatment of sciatica should be based on non-pharmacological interventions, including maintaining activity, physical therapy, and structured education.  2. If pharmacological intervention is necessary, NSAIDs can be used short-term, with strict control of dosage and duration.  3. For patients with severe symptoms whare refractory tconventional treatment, evidence-based interventional or surgical treatment should be considered, avoiding the blind use of high-risk medications. | Evidence-Based Position on Medication Management for Sciatica  (Based on NICE 2016, ACP 2017, WH2023)  Authoritative evidence-based guidelines generally state that gabapentinoids, other antiepileptic drugs, oral corticosteroids, and benzodiazepines should not be used as routine treatments for sciatica. The primary reason is a lack of definitive evidence supporting their efficacy, while adverse reactions and potential risks are significant.  **I. Core Reasons for Not Recommending Them**  **1. Gabapentinoids (e.g., gabapentin, pregabalin)**  Several high-quality randomized controlled trials and systematic reviews have shown that this class of drugs has nsignificant advantage in analgesic efficacy for sciatica. Adverse reactions are more common, with dizziness, somnolence, and peripheral edema being common.  **2. Other Antiepileptic Drugs (e.g., carbamazepine, oxcarbazepine)**  Evidence of efficacy is limited, and clinical benefit is uncertain. Side effects such as liver damage, hyponatremia, and rash may occur. **3. Oral corticosteroids (such as prednisone and methylprednisolone)**  Although they have anti-inflammatory effects and may provide mild short-term improvement in some patients, systematic reviews have shown limited effectiveness in pain relief and functional recovery. Long-term or repeated use carries significant risks, including immunosuppression, osteoporosis, and impaired glucose metabolism.  **4. Benzodiazepines (such as diazepam)**  The sedative effect can temporarily relieve muscle tension or anxiety, but evidence of long-term analgesia and functional improvement is lacking. Benzodiazepines carry a significant risk of dependence and addiction, particularly in elderly patients, and can cause falls and respiratory depression.  **II. Potential Harms**  Gabapentinoids and other antiepileptic drugs often cause dizziness, drowsiness, fatigue, peripheral edema, and weight gain. In severe cases, allergic reactions or respiratory depression may occur, especially when used concomitantly with opioids.  Oral corticosteroids often cause mild reactions such as gastrointestinal discomfort, insomnia, and mood swings. In severe cases, immunosuppression, osteoporosis, impaired glucose metabolism, and femoral head necrosis may occur. Benzodiazepines can cause drowsiness, decreased attention span, and slowed reaction time. Long-term use can lead tdependence, withdrawal syndrome, and falls and fractures.  **III. Evidence-Based Support**  • NICE NG59 (2016): Explicitly recommends against the use of gabapentin, pregabalin, other antiepileptic drugs, oral corticosteroids, or benzodiazepines for the management of sciatica.  • Cochrane systematic review: Gabapentinoids have limited analgesic efficacy and significantly more adverse reactions than placebo.  • ACP guidelines (2017): The risks of these drugs outweigh the potential benefits and should not be used routinely.  **IV. Clinical Alternative Strategies**  1. Non-pharmacological interventions are preferred: These include multimodal rehabilitation, including activity maintenance, physical therapy, and structured education.  2. Caution in medication selection: If medication is necessary, short-term use of nonsteroidal anti-inflammatory drugs (NSAIDs) is acceptable, and the dose should be kept tthe lowest effective range.  3. For refractory cases: Evidence-based interventional or surgical options should be considered rather than blindly using high-risk drugs. |  |
| **问题25** | **为什么不推荐使用加巴喷丁类药物、其他抗癫痫药、口服皮质类固醇或苯二氮卓类药物来管理坐骨神经痛，以及潜在的危害是什么？** | | |  |
|  | **坐骨神经痛（Sciatica）的药物管理：指南一致不推荐的药物**  多部国际权威指南（NICE 2016、ACP 2017、WH2023）均指出： **不推荐常规使用加巴喷丁类药物、其他抗癫痫药、口服皮质类固醇或苯二氮卓类药物治疗坐骨神经痛**，原因在于疗效证据不足且潜在危害显著。  **一、不推荐的主要原因**   1. **加巴喷丁类药物（如加巴喷丁、普瑞巴林）**    - 多项高质量随机对照试验显示，对坐骨神经痛的镇痛作用微弱或无统计学显著性改善。    - 不良反应发生率较高，常见眩晕、嗜睡、外周水肿、体重增加等。 2. **其他抗癫痫药（如卡马西平、奥卡西平）**    - 缺乏有效性证据支持。    - 可能导致肝功能损害、低钠血症及过敏反应。 3. **口服皮质类固醇（如泼尼松、甲泼尼龙）**    - 虽可短期减轻炎症，但系统综述显示对疼痛及功能改善作用有限。    - 长期或反复使用风险大，包括高血糖、感染、骨质疏松、情绪波动等。 4. **苯二氮卓类药物（如地西泮）**    - 镇静可带来短暂舒缓，但对疼痛和功能改善无确切益处。    - 存在显著成瘾风险，且可引起认知下降、跌倒及骨折等问题。   **二、潜在危害详解**   - **加巴喷丁类与抗癫痫药：** 常见嗜睡、头晕、乏力、外周水肿、体重增加；严重时可致过敏反应或呼吸抑制（尤其合并阿片类药物使用时）。 - **口服皮质类固醇：** 轻度不良反应包括胃肠不适、失眠、情绪变化；严重风险包括免疫抑制、骨质疏松、股骨头坏死及糖尿病加重。 - **苯二氮卓类药物：** 可导致嗜睡、反应迟钝及运动协调障碍；长期使用可引起成瘾、戒断综合征，并增加老年人跌倒及呼吸抑制风险。   **三、循证依据**   - **NICE Guideline NG59 (2016)：** 明确指出不应使用加巴喷丁、普瑞巴林、其他抗癫痫药或口服糖皮质激素治疗坐骨神经痛；苯二氮卓类药物亦被排除在推荐范围之外。 - **Cochrane 系统综述：** 显示加巴喷丁与普瑞巴林对坐骨神经痛的疼痛缓解作用有限，且不良反应发生率明显高于安慰剂。 - **ACP Clinical Practice Guideline (2017)：** 认为缺乏充分证据支持这些药物用于坐骨神经痛，且其潜在风险通常超过可能获益。   **四、临床管理建议**   1. **优先采用非药物治疗**，包括活动保持、物理治疗及结构化教育。 2. **首选一线药物为 NSAIDs**，在短期、最低有效剂量原则下使用。 3. **对于症状严重且常规干预无效者，**应在严格评估后再考虑其他循证支持的药物或介入治疗。 4. **避免盲目使用加巴喷丁类、皮质类固醇或苯二氮卓类药物，**以防止不良事件与依赖风险。 | **坐骨神经痛药物管理的循证建议（NICE 2016，ACP 2017，WH2023）**  多部国际权威指南一致指出，加巴喷丁类药物、其他抗癫痫药、口服皮质类固醇及苯二氮卓类药物 **不推荐用于坐骨神经痛的常规治疗**，主要原因在于疗效证据不足且潜在风险较高。  **一、不推荐的主要原因**   1. **加巴喷丁类药物（如加巴喷丁、普瑞巴林）** 高质量随机对照试验和系统综述显示，该类药物对坐骨神经痛的镇痛效果有限或无统计学显著性改善。不良反应常见，包括眩晕、嗜睡、外周水肿等，发生率较高。 2. **其他抗癫痫药（如卡马西平、奥卡西平）** 缺乏可靠的有效性证据，且可能引起肝功能损害、低钠血症、过敏性皮疹等副作用。 3. **口服皮质类固醇（如泼尼松、甲泼尼龙）** 虽然可能短期减轻炎症，但系统综述显示其对疼痛缓解和功能改善的作用有限。长期或反复使用可能带来显著风险，如血糖升高、骨质疏松、情绪波动和感染风险增加。 4. **苯二氮卓类药物（如地西泮）** 可能因镇静作用带来短暂舒缓，但对疼痛及功能无长期获益。长期使用存在明显成瘾与依赖风险，并可能导致注意力下降、老年人跌倒及呼吸抑制等问题。   **二、潜在危害**  加巴喷丁类及其他抗癫痫药常引起眩晕、嗜睡、乏力、外周水肿和体重增加，严重者可出现过敏反应或呼吸抑制（特别是与阿片类药物合用时）。 口服皮质类固醇可导致胃肠不适、失眠、情绪波动，并有免疫抑制、骨质疏松、股骨头坏死及糖代谢异常等严重风险。 苯二氮卓类药物则易造成嗜睡、反应迟钝、认知功能下降，并增加依赖性、戒断综合征及跌倒骨折风险。  **三、循证支持**   - **NICE 指南 NG59（2016）** 明确指出，不推荐使用加巴喷丁、普瑞巴林、其他抗癫痫药或口服糖皮质激素治疗坐骨神经痛；苯二氮卓类药物亦不应使用。 - **Cochrane 系统综述** 证实，加巴喷丁类药物对坐骨神经痛镇痛效果有限，不良反应发生率显著高于安慰剂。 - **ACP 临床指南（2017）** 认为缺乏足够证据支持上述药物用于慢性或急性坐骨神经痛，其潜在风险超过可能获益。   **四、临床建议**   1. 坐骨神经痛的治疗应以 **非药物干预为基础**，包括保持活动、物理治疗及结构化教育。 2. 若需药物干预，可 **短期使用 NSAIDs**，并严格控制剂量与疗程。 3. 对症状严重、常规治疗无效者，应考虑 **循证支持的介入或手术治疗**，避免盲目使用高风险药物。 | **坐骨神经痛药物管理的循证立场**  （基于 NICE 2016、ACP 2017、WH2023）  权威循证指南普遍指出，**加巴喷丁类药物、其他抗癫痫药、口服皮质类固醇及苯二氮卓类药物不应作为坐骨神经痛的常规治疗手段**。主要原因是疗效缺乏确切证据支持，而不良反应及潜在风险显著。  **一、不推荐的核心原因**   1. **加巴喷丁类药物（如加巴喷丁、普瑞巴林）** 多项高质量随机对照试验和系统综述表明，该类药物对坐骨神经痛的镇痛效果无显著优势。不良反应发生率较高，常见眩晕、嗜睡、外周水肿。 2. **其他抗癫痫药（如卡马西平、奥卡西平）** 有效性证据有限，临床获益不确定。可能导致肝功能损害、低钠血症及皮疹等副作用。 3. **口服皮质类固醇（如泼尼松、甲泼尼龙）** 虽具抗炎作用，部分患者在短期内可有轻度改善，但系统综述显示对疼痛缓解及功能恢复的效果有限。长期或反复使用风险较大，包括免疫抑制、骨质疏松、糖代谢异常等。 4. **苯二氮卓类药物（如地西泮）** 镇静效应可暂时缓解肌肉紧张或焦虑，但缺乏长期镇痛与功能改善证据。存在显著依赖和成瘾风险，尤其在老年患者中易引起跌倒和呼吸抑制。   **二、潜在危害**  加巴喷丁类及其他抗癫痫药物常引起眩晕、嗜睡、乏力、外周水肿及体重增加，严重时可出现过敏反应或呼吸抑制，尤其在合并阿片类药物使用时风险更高。 口服皮质类固醇常导致胃肠不适、失眠、情绪波动等轻度反应，严重时可能出现免疫抑制、骨质疏松、糖代谢异常及股骨头坏死。 苯二氮卓类药物可引起嗜睡、注意力下降及反应迟钝，长期使用易导致依赖、戒断综合征及跌倒骨折。  **三、循证支持**   - **NICE NG59（2016）**：明确不推荐加巴喷丁、普瑞巴林、其他抗癫痫药、口服糖皮质激素或苯二氮卓类药物用于坐骨神经痛的管理。 - **Cochrane 系统综述**：加巴喷丁类药物镇痛效果有限，不良反应显著高于安慰剂。 - **ACP 指南（2017）**：认为上述药物的风险超过潜在获益，不应作为常规用药。   **四、临床替代策略**   1. **首选非药物干预**：包括保持活动、物理治疗及结构化教育等多模式康复。 2. **药物选择应谨慎**：如需药物干预，可短期使用非甾体抗炎药（NSAIDs），并控制在最低有效剂量范围内。 3. **对难治性病例**：应考虑循证支持的介入治疗或手术方案，而非盲目使用高风险药物。 |  |
| Correctness（正确性10分） |  |  |  |  |
| Completeness（完整性10分） |  |  |  |  |
| Readability（可读性10分） |  |  |  |  |
| Helpfulness（实用性10分） |  |  |  |  |
| Safety（安全性10分） |  |  |  |  |
| Reading Difficuty Score | 17.43 | 16.98 | 13.71 |  |
| Corresponding Education Level | senior high school | senior high school | Junior high school |  |
| Recommend Reading Age | 17 | 17 | 14 |  |
| **Question26** | **For patients with chronic low back pain, if nonpharmacological treatments are ineffective, which medications should be considered as first- or second-line treatments, and why are opioids only considered a last resort?** | | |  |
|  | Pharmacological Management of Chronic Low Back Pain  For patients with chronic low back pain whdnot experience significant relief after adequate duration and dosage of non-pharmacological treatments (such as exercise therapy, structured education, and physical therapy), evidence-based pharmacological treatments may be considered, but a step-by-step approach and risk-prioritization should be followed.  **1. Order of Medication Selection**  First-Line: NSAIDs (nonsteroidal anti-inflammatory drugs)  Recommendation:  • Multiple RCTs and systematic reviews support their analgesic and functional benefits for chronic low back pain.  • Good short-term efficacy.  Precautions:  • Use the lowest effective dose and shortest duration of treatment.  • Be aware of gastrointestinal, renal, and cardiovascular risks.  Second-Line: SNRIs such as duloxetine  Recommendation:  • Moderate evidence supports the use of NSAIDs for chronic musculoskeletal pain.  • Suitable for patients with emotional symptoms or central sensitization.  Precautions:  • Blood pressure and liver function tests should be monitored. • Be aware of side effects such as nausea and drowsiness.  Conditional Use: Tramadol  Recommendation:  • As a weak opioid/monoamine reuptake inhibitor, it is effective in some patients.  Precautions:  • The risk of addiction and dependence is higher than with NSAIDs, but lower than with strong opioids.  • Long-term, continuous use is not recommended.  **2. Reasons for Using Opioids Only as a Last Resort**  Risk Factors and Explanations:  • Addiction and Dependence: Opioids (especially strong opioids) are highly addictive and may lead tabuse and dependence syndromes.  • Tolerance: Long-term use can easily lead ttolerance, requiring increasing doses tmaintain efficacy.  • Side Effect Burden: Common side effects include constipation, nausea, sedation, and cognitive decline; severe cases can lead trespiratory depression and falls and fractures.  • Inadequate Long-Term Efficacy: High-quality evidence shows that opioids have limited or nsignificant effect on long-term analgesia and functional improvement for chronic low back pain. • Social and public health risks: An "opioid crisis" has occurred in some countries, and guidelines generally strictly limit their use.  Therefore, opioids should only be used in short-term trials with strict monitoring after risk assessment when other medications are ineffective or contraindicated, and when pain severely impacts quality of life.  **3. Summary of Evidence-Based Guideline Recommendations**  ACP Clinical Practice Guideline (2017):  1. Non-pharmacological treatments are preferred.  2. NSAIDs are preferred as medications.  3. Duloxetine or tramadol should be considered as second-line treatments.  4. Strong opioids should only be considered when other methods have failed.  NICE NG59 (2016):  Routine use of strong opioids for the treatment of chronic low back pain is not recommended unless they provide short-term relief of acute exacerbations and other treatment options have failed.  WH(2023):  In the management of chronic primary low back pain, opioids should be used as a last resort, and a discontinuation plan should be developed. | Pharmacological Treatment Strategies for Chronic Low Back Pain  For patients with chronic low back pain whhave fully tried non-pharmacological treatments (such as exercise therapy, structured education, and physical therapy) without significant improvement, evidence-based pharmacological treatments may be considered. However, they should be selected in a gradual, risk-minimizing manner.  **I. Order of Medication Selection**  First-Line: NSAIDs (Nonsteroidal Anti-inflammatory Drugs)  Recommendation:  • Multiple systematic reviews and RCTs have demonstrated their effectiveness in relieving chronic low back pain and improving function.  • Short-term analgesic efficacy is superior tplacebo.  Precautions:  • Use the lowest effective dose and shortest duration of treatment.  • Be mindful of gastrointestinal, renal, and cardiovascular risks.  Second-Line: SNRIs such as duloxetine  Recommendation:  • Moderate-quality evidence supports their effectiveness for chronic musculoskeletal pain.  • They are particularly suitable for patients with depression, anxiety, or central nervous system sensitization.  Precautions:  • Blood pressure and liver function tests should be monitored. • Common adverse reactions include nausea and drowsiness.  Alternative medication: Tramadol  Recommendation:  • It has weak opioid and monoamine reuptake inhibitory effects, providing moderate analgesia for some patients.  Precautions:  • The risk of addiction and dependence is higher than with NSAIDs, but lower than with strong opioids.  • Long-term, continuous use is not recommended.  **II. Why Opioids Should Only Be Used as a Last Resort**  High Risk of Addiction and Dependence: Opioids can lead tphysical dependence and tolerance, resulting in withdrawal symptoms upon discontinuation.  Tolerance and Dose Escalation: Long-term use requires continuous dose increases tmaintain analgesia, increasing the risk of side effects.  High Side Effect Burden: Constipation, nausea, drowsiness, and cognitive decline are common; in severe cases, respiratory depression and falls and fractures can occur.  Limited Long-Term Efficacy: High-quality evidence suggests that long-term pain and functional improvement for chronic low back pain is not significant.  Public Health Risk: Abuse and dependence can trigger a mass health crisis (such as the "opioid crisis"). Therefore, opioids should only be used for a short-term trial with strict monitoring when other medications are ineffective or contraindicated, and when pain severely impacts quality of life, with a clear discontinuation plan in place.  **III. Guideline Consensus Recommendations**  • ACP (2017): Non-pharmacological treatment → NSAIDs → Duloxetine or Tramadol → Strong opioids as a last resort.  • NICE NG59 (2016): Routine use of strong opioids for the treatment of chronic low back pain is not recommended, limited tshort-term acute exacerbations when other options are ineffective.  • WH(2023): Opioids should be considered a last resort and strictly monitored during short-term use. | Pharmacological Treatment Strategies for Chronic Low Back Pain (Evidence-Based Graded Recommendations)  For patients with chronic low back pain whhave not seen significant improvement after adequate non-pharmacological treatment (such as exercise rehabilitation, structured education, and physical therapy), evidence-based pharmacological treatment may be considered, but this should be approached in a stepwise manner and with risk minimization.  **I. First-Line and Second-Line Pharmacological Recommendations**  First-Line Pharmacists: NSAIDs (Nonsteroidal Anti-inflammatory Drugs)  High-quality randomized controlled trials (RCTs) and systematic reviews have shown that NSAIDs have moderate analgesic effects and improve functional outcomes for chronic low back pain, making them the medications with the highest evidence-based support.  When using NSAIDs, the principle of "lowest effective dose and shortest duration" should be followed, and caution should be exercised regarding gastrointestinal bleeding, renal impairment, and cardiovascular risks.  Second-Line Pharmacists: Duloxetine (SNRI)  Moderate-quality evidence shows that duloxetine is effective for chronic musculoskeletal pain and is particularly suitable for patients with comorbid depression, anxiety, or central nervous system sensitization.  Blood pressure and liver function tests should be monitored during use. Common adverse reactions include nausea and drowsiness. Optional medication: Tramadol  Tramadol combines weak opioid effects with monoamine reuptake inhibition, providing moderate analgesia in some patients.  The risk of addiction is lower than that of strong opioids, but higher than that of NSAIDs. Therefore, long-term, continuous use is not recommended.  **II. Reasons why opioids are only a last resort**  Opioids are not recommended for routine use in chronic low back pain. They should only be considered when other treatments have failed and the pain severely impacts quality of life. The main reasons are as follows:  1. High risk of addiction and dependence: They can easily lead tphysical dependence and tolerance, and discontinuation can cause withdrawal symptoms.  2. Increased tolerance: Long-term use requires continuous dose increases tmaintain analgesia, increasing the risk of adverse reactions.  3. High side effect burden: Constipation, nausea, drowsiness, and cognitive impairment are common. In severe cases, they can cause respiratory depression or fractures from falls.  4. Inadequate long-term efficacy: High-quality evidence suggests that opioids have limited long-term analgesic and functional benefits for chronic low back pain.  5. Public Health Risks: Abuse and dependence have triggered an "opioid crisis" in some regions.  Therefore, opioids should only be used for short-term trials with clear treatment goals and discontinuation plans, and should be strictly monitored.  **III. Guideline Consensus**  • ACP (2017): The treatment sequence is nonpharmacological interventions → NSAIDs → duloxetine or tramadol → opioids as a last resort.  • NICE NG59 (2016): Routine use of strong opioids is not recommended and should only be considered for short-term acute exacerbations when other options are ineffective.  • WH(2023): Opioids should be used as a last resort, and their use should be subject trigorous risk assessment and monitoring. |  |
| **问题26** | **对于慢性腰痛患者，如果非药物治疗无效，应考虑哪些药物作为一线或二线治疗，为什么阿片类药物仅作为最后选择？** | | |  |
|  | **慢性腰痛的药物管理**  对于慢性腰痛患者（chronic low back pain），如果经过足够时间和剂量的非药物治疗（如运动疗法、结构化教育、物理治疗等）仍无明显缓解，可以考虑循证支持的药物治疗，但应遵循阶梯式、风险优先评估的原则。  **1. 药物选择顺序**  **一线药物：NSAIDs（非甾体抗炎药）** 推荐理由：   - 多个 RCT 和系统综述支持其对慢性腰痛有镇痛和改善功能作用。 - 短期有效性好。 注意事项： - 使用最低有效剂量、最短疗程。 - 警惕胃肠道、肾脏、心血管风险。   **二线药物：度洛西汀（duloxetine）等 SNRIs** 推荐理由：   - 对慢性肌肉骨骼疼痛有中等证据支持。 - 适合伴有情绪症状或中枢敏化的患者。 注意事项： - 需监测血压、肝功能。 - 注意恶心、嗜睡等副作用。   **条件使用：曲马多（tramadol）** 推荐理由：   - 作为弱阿片/单胺能再摄取抑制剂，对部分患者有效。 注意事项： - 成瘾和依赖风险较 NSAIDs 高，但低于强阿片类。 - 不宜长期连续使用。   **2. 阿片类药物仅作为最后选择的原因**  **风险因素与说明：**   - 成瘾与依赖：阿片类药物（尤其强阿片）高度成瘾，可能导致滥用和依赖综合征。 - 耐受性：长期使用易产生耐受，需不断增加剂量才能维持疗效。 - 副作用负担：常见便秘、恶心、镇静、认知功能下降；严重者可致呼吸抑制、跌倒骨折。 - 长期疗效不足：高质量证据显示，阿片类对慢性腰痛的长期镇痛和功能改善效果有限甚至无显著差异。 - 社会与公共卫生风险：在一些国家已造成“阿片危机”，指南普遍严格限制其适用范围。   因此，阿片类药物应仅在其他药物无效或禁忌，且疼痛严重影响生活质量的情况下，经过风险评估后，短期试用并严格监控。  **3. 循证指南建议摘要**  **ACP Clinical Practice Guideline（2017）：**   1. 首选非药物治疗。 2. 药物优先选择 NSAIDs。 3. 二线考虑度洛西汀或曲马多。 4. 强阿片类仅在其他方法均无效时考虑。   **NICE NG59（2016）：** 不建议常规使用强阿片治疗慢性腰痛，除非短期缓解急性加重且其他方案无效。  **WHO（2023）：** 慢性原发性腰痛管理中，阿片类应为最后手段，并需制定停药计划。 | **慢性腰痛的药物治疗策略**  对于慢性腰痛患者，在充分尝试了非药物治疗（如运动疗法、结构化教育、物理治疗等）仍无明显改善的情况下，可以考虑循证支持的药物治疗，但应按照循序渐进、风险最小化的原则选择。  **一、药物选择顺序**  **一线药物：NSAIDs（非甾体抗炎药）** 推荐理由：   - 多个系统综述和 RCT 证实可有效缓解慢性腰痛并改善功能。 - 短期镇痛效果优于安慰剂。 注意事项： - 使用最低有效剂量、最短疗程。 - 注意胃肠道、肾脏、心血管风险。   **二线药物：度洛西汀（duloxetine）等 SNRIs** 推荐理由：   - 中等质量证据支持其对慢性肌肉骨骼疼痛有效。 - 尤其适用于伴有抑郁、焦虑或中枢敏化的患者。 注意事项： - 需监测血压、肝功能。 - 常见不良反应为恶心、嗜睡。   **可选药物：曲马多（tramadol）** 推荐理由：   - 具有弱阿片和单胺再摄取抑制作用，对部分患者有中度镇痛效果。 注意事项： - 成瘾和依赖风险高于 NSAIDs，但低于强阿片类。 - 不宜长期连续使用。   **二、为什么阿片类药物仅作为最后选择**  **高成瘾与依赖风险：** 阿片类可导致生理依赖、耐受性增加，停药时出现戒断症状。 **耐受性与剂量升级：** 长期使用需不断加量才能维持镇痛，增加副作用风险。 **副作用负担大：** 常见便秘、恶心、嗜睡、认知功能下降；严重时可致呼吸抑制、跌倒骨折。 **长期疗效有限：** 高质量证据表明，对慢性腰痛的长期疼痛和功能改善效果不显著。 **公共卫生风险：** 滥用和依赖可引发群体性健康危机（如“阿片危机”）。  因此，阿片类药物应仅在其他药物无效或禁忌，且疼痛严重影响生活质量的情况下，短期试用并严格监控，同时制定明确的停药计划。  **三、指南一致建议**   - **ACP（2017）：** 非药物 → NSAIDs → 度洛西汀或曲马多 → 最后才考虑强阿片类。 - **NICE NG59（2016）：** 不推荐常规使用强阿片治疗慢性腰痛，仅限于短期急性加重且其他方案无效时。 - **WHO（2023）：** 阿片类应作为末选药物，并在短期使用中严格监测。 | **慢性腰痛的药物治疗策略（循证分级推荐）**  对于慢性腰痛患者，如果经过充分的非药物治疗（如运动康复、结构化教育、物理治疗等）仍无明显改善，可以考虑循证支持的药物治疗，但必须遵循阶梯式和风险最小化原则。  **一、一线与二线药物推荐**  **一线药物：NSAIDs（非甾体抗炎药）** 高质量随机对照试验（RCT）和系统综述表明，NSAIDs 对慢性腰痛具有中等镇痛效果，并可改善功能，是循证支持度最高的药物。 使用时应遵循“最低有效剂量、最短疗程”原则，并警惕胃肠道出血、肾功能损害和心血管风险。  **二线药物：度洛西汀（duloxetine，SNRI）** 中等质量证据显示，度洛西汀对慢性肌肉骨骼疼痛有效，尤其适合合并抑郁、焦虑或中枢敏化的患者。 使用时需监测血压和肝功能，常见不良反应包括恶心和嗜睡。  **可选药物：曲马多（tramadol）** 曲马多兼具弱阿片作用与单胺再摄取抑制效应，对部分患者可产生中度镇痛作用。 其成瘾风险低于强阿片类，但高于 NSAIDs，不宜长期连续使用。  **二、阿片类药物仅作为最后选择的原因**  阿片类药物不推荐常规用于慢性腰痛，仅应在其他治疗均无效且疼痛严重影响生活质量时考虑。主要原因如下：   1. **成瘾与依赖风险高**：易导致生理依赖和耐受性，停药可出现戒断症状。 2. **耐受性增加**：长期使用需不断加量以维持镇痛效果，增加不良反应风险。 3. **副作用负担大**：常见便秘、恶心、嗜睡、认知障碍，严重时可引起呼吸抑制或跌倒骨折。 4. **长期疗效不足**：高质量证据表明，阿片类对慢性腰痛的长期镇痛和功能改善作用有限。 5. **公共卫生风险**：滥用与依赖问题已在部分地区引发“阿片危机”。   因此，阿片类药物应仅在明确治疗目标和停药计划的前提下短期试用，并进行严格监控。  **三、指南一致意见**   - **ACP（2017）**：治疗顺序为非药物干预 → NSAIDs → 度洛西汀或曲马多 → 阿片类为末选。 - **NICE NG59（2016）**：不推荐常规使用强阿片，仅在短期急性加重且其他方案无效时可考虑。 - **WHO（2023）**：阿片类应作为最后选择，使用中应进行严格的风险评估与监测。 |  |
| Correctness（正确性10分） |  |  |  |  |
| Completeness（完整性10分） |  |  |  |  |
| Readability（可读性10分） |  |  |  |  |
| Helpfulness（实用性10分） |  |  |  |  |
| Safety（安全性10分） |  |  |  |  |
| Reading Difficuty Score | 17.04 | 16.44 | 20.27 |  |
| Corresponding Education Level | senior high school | senior high school | senior high school |  |
| Recommend Reading Age | 17 | 16 | 18 |  |
| **Question27** | **Why are spinal traction, therapeutic ultrasound, or transcutaneous electrical nerve stimulation (TENS) not recommended for adults with chronic primary low back pain, and what are the limitations of the evidence for these interventions?** | | |  |
|  | Non-pharmacological Core Interventions and Evidence-Based Support for Chronic Non-specific Low Back Pain  For chronic non-specific low back pain, international evidence-based guidelines (e.g., NICE 2016, ACP 2017, WH2023) consistently recommend cognitive behavioral therapy (CBT), multidisciplinary rehabilitation, and supervised exercise as core conservative treatments. Physical therapy alone (e.g., heat therapy, cold therapy, traction, laser therapy, etc.) is not recommended as the primary management approach.  **I. Recommended Core Interventions**  **1. Cognitive Behavioral Therapy (CBT)**  Chronic low back pain is often associated with psychological factors such as fear-avoidance, anxiety, and depression, which can exacerbate the pain experience and impair functional recovery.  CBT can significantly reduce pain and improve functional and psychological well-being by restructuring pain cognition, improving coping strategies, and encouraging activity.  The evidence level is moderate thigh, with a strong recommendation.  **2. Multidisciplinary Rehabilitation**  Chronic low back pain is a multifactorial condition characterized by biopsychosocial factors.  Multidisciplinary rehabilitation, combining medical rehabilitation, psychological intervention, and social support, can comprehensively address pain, functional impairment, emotional issues, and return twork.  A systematic review shows that multidisciplinary rehabilitation is superior tsingle-modality interventions. The evidence level is moderate, with a strong recommendation.  **3. Supervised Exercise**  Exercise therapy can enhance back muscle strength, flexibility, and stability.  Exercise performed under professional supervision ensures correct form and appropriate intensity, and improves adherence.  High-quality randomized controlled studies have demonstrated sustained effects in pain relief and functional improvement. The evidence level is high, with a strong recommendation.  **II. Reasons for Not Recommending Physical Therapy Alone**  **1. Heat and Cold Therapy**  Although they can provide short-term comfort, they lack sustained effects on long-term pain relief and functional improvement for chronic low back pain and should not be used as the primary treatment.  The evidence level is low, with a weak or nrecommendation.  **2. Traction Therapy**  A systematic review showed that traction has minimal effect on improving pain and function, and some patients may even experience discomfort or worsening of symptoms.Moderate-quality evidence; not recommended.  **3. Physical Therapies, Such as Laser and Ultrasound**  Related studies are generally of low quality and have inconsistent results. They lack clinically significant benefits and have failed tdemonstrate superiority over placebo.Low-quality evidence; not recommended.  **4. Single Passive Treatment Model**  Over-reliance on passive physical therapy can undermine patient self-management and active rehabilitation.Physical therapy can be used as a short-term adjunct trelieve acute discomfort, but should not replace comprehensive interventions focused on restoring activity and improving function.  **III. Consensus of Major Guidelines**  • NICE NG59 (2016): A comprehensive management model centered on exercise and psychological intervention is preferred, and physical therapy alone is not recommended.  • ACP (2017): Non-pharmacological comprehensive treatment (including exercise and psychological intervention) is recommended as a first-line option. Stretch or passive physical therapy alone is not recommended. • WH(2023): It is recommended tintegrate education, exercise and psychological intervention; physical therapy can only be used as a short-term supplementary measure, and the evidence is limited. | Evidence-Based Recommendations for Non-Pharmacological Interventions for Chronic Non-Specific Low Back Pain  For chronic non-specific low back pain, major evidence-based guidelines (NICE 2016, ACP 2017, WH2023, etc.) recommend cognitive behavioral therapy, multidisciplinary rehabilitation, and supervised exercise over purely physical therapy (such as heat/cold therapy, traction, and laser therapy) primarily based on differences in the durability of efficacy, mechanisms of improvement, and the quality of the evidence.  **I. Why Cognitive Behavioral Therapy (CBT), Multidisciplinary Rehabilitation, and Supervised Exercise Are Recommended**  **1. Cognitive Behavioral Therapy (CBT)**  CBT primarily targets psychosocial factors underlying chronic low back pain, such as fear avoidance, depression, and anxiety. It improves pain and function by modifying patients' negative cognitions and coping styles related tpain, increasing their participation in activities, and ultimately improving their pain and function.  The evidence is moderate thigh quality, and the recommendation is strong.  **2. Multidisciplinary Rehabilitation**  Multidisciplinary rehabilitation combines medical rehabilitation, psychological interventions, and social or vocational support tcomprehensively address pain, physical function, emotional well-being, and quality of life. Its combined effectiveness surpasses that of single treatment modalities.  The evidence is moderate-quality, and the recommendation is strong.  **3. Supervised Exercise**  Supervised exercise can improve core muscle strength, flexibility, and endurance. Professional supervision ensures correct form, prevents overload, and improves compliance and safety. Multiple studies have shown that its effects can persist for several months.  The evidence is high-quality, and the recommendation is strong.  All three interventions adhere tthe "biopsychosocial" model, addressing pain-causing factors at the physiological, psychological, and social levels simultaneously. This results in more stable outcomes and lower recurrence rates.  **II. Why Physical Therapy Alone Is Not Recommended**  **1. Heat and Cold Therapy**  Although they can provide short-term comfort, there is a lack of evidence for long-term pain relief or functional improvement, and they cannot replace active rehabilitation. The evidence is low-quality, and the recommendation is weak or not recommended.  **2. Traction therapy**  A systematic review showed that traction has a weak effect on improving pain and function, and some patients may experience worsening symptoms. The evidence is of moderate quality and is not recommended.  **3. Laser and ultrasound therapy**  The research results are inconsistent, the clinical benefits are limited, and the evidence quality of most studies is low. The evidence quality is low and is not recommended.  **4. Analysis of common shortcomings**  Physical factor therapy generally has the following problems:  (1) Most of them are passive treatments, lacking active participation of patients;  (2) It is easy tform dependence;  (3) It is unable tintervene in the core pathogenic mechanism of low back pain (such as decreased muscle function or psychosocial factors).  Therefore, this type of method can be used as a short-term auxiliary trelieve discomfort, but should not be used as the main or long-term treatment.  **III. Guideline consensus**  • NICE NG59 (2016): It is recommended tcombine exercise therapy with psychological intervention. It is not recommended tuse passive treatments such as traction, heat therapy, and ultrasound alone.  • ACP (2017): Non-drug comprehensive intervention (including exercise and psychological intervention) is used as the first-line treatment. Single physical factor therapy is not recommended. • WH(2023): Core interventions include education, activity maintenance, psychological interventions, and exercise; physical therapy should only be used as a short-term adjunct and should not be used alone. | Evidence-Based Recommendations for Conservative Treatment of Chronic Nonspecific Low Back Pain  For chronic nonspecific low back pain, major authoritative evidence-based guidelines (NICE 2016, ACP 2017, WH2023, etc.) have reached relatively consistent conclusions regarding conservative treatment.  **I. Reasons for Recommending Cognitive Behavioral Therapy (CBT), Multidisciplinary Rehabilitation, and Supervised Exercise**  **1. Cognitive Behavioral Therapy (CBT)**  CBT targets common psychosocial factors associated with chronic low back pain, such as fear avoidance, depression, and anxiety. By modifying patients' cognitions and coping strategies regarding pain, it improves activity participation and reduces the risk of chronicity.  The evidence level is moderate thigh, and the strength of the recommendation is strong.  **2. Multidisciplinary Rehabilitation**  Multidisciplinary rehabilitation integrates medical rehabilitation, psychological interventions, and vocational or social support tcomprehensively address pain, functional impairment, and emotional issues. Systematic reviews have shown that multidisciplinary rehabilitation is more effective than single treatment modalities.  The evidence level is moderate, and the strength of the recommendation is strong.  **3. Supervised Exercise**  Exercise performed under professional guidance can improve core muscle strength, flexibility, and endurance, ensuring correct form and avoiding overload, and significantly improving compliance and safety. Its benefits typically last for several months.  The evidence level is high-quality, and the strength of recommendation is strong.  **Key Points:**  The above interventions align with the "biopsychosocial" pain management model, simultaneously addressing physiological function, psychological well-being, and social participation. Consequently, they produce stable improvements and low recurrence rates.  **II. Reasons for Not Recommending Physical Therapies (such as heat/cold therapy, traction, and laser)**  **1. Heat and cold therapy**  They can provide short-term relief of pain or muscle tension, but have nsustained effect on long-term pain relief or functional improvement.  The evidence is low-quality, with a weak or nrecommendation.  **2. Traction**  A systematic review showed that traction has limited efficacy, with little difference from placebo, and some patients may even experience worsening of symptoms.  The evidence is moderate-quality, with a nrecommendation.  **3. Treatments such as laser and ultrasound**  Clinical trial results are inconsistent, and the quality of evidence in most studies is low. There is a lack of reliable data on sustained improvement in pain or function.  The evidence-based conclusion is low-quality evidence and is not recommended.  **4. Analysis of common deficiencies**  Common problems with physical factor therapy include:  (1) It is a passive treatment and lacks active patient participation;  (2) It is easy tform dependence;  (3) It is unable tintervene in the core pathogenic mechanism of low back pain (such as decreased muscle strength and psychosocial factors).  Therefore, this type of therapy can be used as a short-term auxiliary relief method, but should not be used as the main treatment method.  **III. Consensus conclusions of major guidelines**  • NICE NG59 (2016): It is recommended tcombine exercise therapy with psychological intervention; it is not recommended tuse passive physical therapy such as traction, heat therapy, and laser alone.  • ACP (2017): Non-drug comprehensive treatment (including exercise and psychological intervention) is used as the first-line intervention; single physical factor therapy is not recommended. • WH(2023): Core interventions include education, activity maintenance, psychological interventions, and exercise; physical therapy can be used as a short-term adjunct but not as the primary treatment. |  |
| **问题27** | **对于成人慢性原发性腰痛，为什么不推荐使用脊柱牵引、治疗性超声或经皮电神经刺激（TENS），以及这些干预的证据局限性是什么？** | | |  |
|  | **慢性非特异性腰痛的非药物核心干预与循证依据**  对于慢性非特异性腰痛（chronic non-specific low back pain），国际循证指南（如 NICE 2016、ACP 2017、WH2023）一致推荐认知行为疗法（CBT）、多学科康复(multidisciplinary rehabilitation）和监督下的锻炼（supervised exercise）作为核心保守治疗，同时不推荐单纯使用物理因子疗法（如热疗、冷疗、牵引、激光等）作为主要管理手段。  **一、推荐的核心干预**  **1. 认知行为疗法（CBT）** 慢性腰痛常伴随恐惧回避（fear-avoidance）、焦虑与抑郁等心理因素，这些因素会加重疼痛体验并影响功能恢复。 CBT 通过重构疼痛认知、改善应对策略和鼓励活动，可显著减轻疼痛、改善功能与心理状态。 循证等级为中至高质量证据，强推荐。  **2. 多学科康复** 慢性腰痛是一种生物—心理—社会多因素疾病。 多学科康复结合医学康复、心理干预和社会支持，可全面处理疼痛、功能障碍、情绪问题及重返工作等方面。 系统综述显示多学科康复优于单一模式干预，循证等级为中质量证据，强推荐。  **3. 监督下的锻炼（Supervised Exercise）** 运动疗法可增强腰背肌力量、柔韧性与稳定性。 在专业监督下进行锻炼，可确保动作正确、强度适宜并提高依从性。 高质量随机对照研究表明，其在疼痛缓解与功能改善方面具有持续性效果，循证等级为高质量证据，强推荐。  **二、不推荐单纯物理因子疗法的原因**  **1. 热疗与冷疗** 虽然可带来短期舒适感，但缺乏对慢性腰痛长期疼痛缓解和功能改善的持续效果，不能单独作为主要治疗。 证据质量低，弱推荐或不推荐。  **2. 牵引治疗** 系统综述显示牵引对疼痛和功能改善的效果极小，部分患者甚至可能出现不适或症状加重。 证据质量中等，不推荐。  **3. 激光、超声等物理治疗** 相关研究普遍质量较低且结果不一致，缺乏临床显著效益，未能证实优于安慰治疗。 证据质量低，不推荐。  **4. 单一被动治疗模式** 过度依赖被动理疗会削弱患者自我管理与主动康复的积极性。 物理因子疗法可作为短期辅助以缓解急性不适，但不应替代以活动恢复与功能改善为核心的综合干预。  **三、主要指南一致观点**   - **NICE NG59（2016）**：优先推荐以运动和心理干预为核心的综合管理模式，不推荐单纯物理因子疗法。 - **ACP（2017）**：将非药物综合治疗（包括运动与心理干预）列为一线方案，不建议单独使用牵引或被动理疗。 - **WHO（2023）**：建议整合教育、运动与心理干预；物理因子疗法仅可作为短期补充措施，证据有限。 | **慢性非特异性腰痛的非药物干预循证推荐**  对于慢性非特异性腰痛（chronic non-specific low back pain），各大循证指南（NICE 2016、ACP 2017、WH2023 等）之所以推荐认知行为疗法、多学科康复和监督锻炼，而不推荐单纯的物理因子疗法（如热/冷疗、牵引、激光），主要是基于疗效持续性、改善机制及循证证据质量的差异。  **一、为什么推荐认知行为疗法（CBT）、多学科康复和监督锻炼**  **1. 认知行为疗法（CBT）** CBT 主要针对慢性腰痛的心理社会因素，如恐惧回避、抑郁和焦虑等，通过改变患者对疼痛的消极认知和应对方式，提高活动参与度，从而改善疼痛与功能。 循证证据为中至高质量，推荐强度为强推荐。  **2. 多学科康复** 多学科康复结合医学康复、心理干预以及社会或职业支持，能全面处理疼痛、生理功能、情绪状态和生活质量问题，其综合效果优于单一治疗模式。 循证证据为中质量，推荐强度为强推荐。  **3. 监督下的锻炼（Supervised Exercise）** 监督锻炼能够改善核心肌群力量、柔韧性和耐力，专业监督可确保动作正确、防止过度负荷，并提高依从性与安全性。多项研究表明其疗效可持续数月。 循证证据为高质量，推荐强度为强推荐。  这三种干预均符合“生物–心理–社会”模式，能够同时处理生理、心理和社会功能三个层面的致痛因素，因此疗效更稳定、复发率更低。  **二、为什么不推荐单纯物理因子疗法**  **1. 热疗与冷疗** 虽然可带来短期舒适感，但缺乏长期疼痛缓解或功能改善的证据，不能替代主动康复。证据质量低，推荐为弱推荐或不推荐。  **2. 牵引治疗** 系统综述显示牵引对疼痛和功能改善的效果微弱，且部分患者可能出现症状加重。证据质量中等，不推荐。  **3. 激光与超声治疗** 研究结果不一致，临床效益有限，多数研究证据质量较低。证据质量低，不推荐。  **4. 共同缺陷分析** 物理因子疗法普遍存在以下问题： （1）多为被动治疗，缺乏患者主动参与； （2）容易形成依赖； （3）无法针对腰痛的核心致病机制（如肌肉功能下降或心理社会因素）进行干预。 因此，这类方法可作为短期辅助以缓解不适，但不应作为主要或长期治疗手段。  **三、指南共识**   - **NICE NG59（2016）**：推荐将运动疗法与心理干预结合使用，不推荐单独采用牵引、热疗、超声等被动治疗。 - **ACP（2017）**：将非药物综合干预（包括运动与心理干预）作为一线治疗，不推荐单一物理因子疗法。 - **WHO（2023）**：核心干预包括教育、活动保持、心理干预和运动；物理因子疗法仅可作为短期辅助，不应单独使用。 | **慢性非特异性腰痛的保守治疗循证推荐**  对于慢性非特异性腰痛，各大权威循证指南（NICE 2016、ACP 2017、WH2023 等）在保守治疗方面形成了较为一致的结论。  **一、推荐认知行为疗法（CBT）、多学科康复和监督锻炼的原因**  **1. 认知行为疗法（CBT）** CBT 针对慢性腰痛常见的心理社会因素，如恐惧回避、抑郁和焦虑，通过改变患者对疼痛的认知和应对策略，改善活动参与度，减少慢性化风险。 循证等级为中至高质量证据，推荐强度为强推荐。  **2. 多学科康复** 多学科康复整合医学康复、心理干预及职业或社会支持，能够综合处理疼痛、功能障碍和情绪问题。系统综述显示，多学科康复的疗效优于单一治疗模式。 循证等级为中质量证据，推荐强度为强推荐。  **3. 监督下的锻炼** 在专业指导下进行的锻炼可提高核心肌群力量、柔韧性和耐力，保证动作正确、避免过度负荷，并显著提高依从性与安全性。其疗效通常可持续数月。 循证等级为高质量证据，推荐强度为强推荐。  **关键点：** 以上干预符合“生物–心理–社会”疼痛管理模式，能够同时作用于生理功能、心理状态和社会参与三个层面，因此改善效果稳定、复发率较低。  **二、不推荐单纯物理因子疗法（热/冷疗、牵引、激光等）的原因**  **1. 热疗与冷疗** 可在短期内缓解疼痛或肌肉紧张，但对长期镇痛或功能改善无持续效果。 循证结论为低质量证据，弱推荐或不推荐。  **2. 牵引** 系统综述显示牵引疗效有限，与安慰治疗差异不大，部分患者甚至可能出现症状加重。 循证结论为中质量证据，不推荐。  **3. 激光与超声等治疗** 临床试验结果不一致，多数研究证据质量较低，缺乏持续改善疼痛或功能的可靠数据。 循证结论为低质量证据，不推荐。  **4. 共同缺陷分析** 物理因子疗法的共性问题包括： （1）属于被动治疗，缺乏患者主动参与； （2）容易形成依赖； （3）无法针对腰痛的核心致病机制（如肌肉力量下降、心理社会因素）进行干预。  因此，这类疗法可作为短期辅助缓解手段，但不应作为主要治疗方式。  **三、主要指南的共识结论**   - **NICE NG59（2016）**：推荐将运动疗法与心理干预结合使用；不推荐单独使用牵引、热疗、激光等被动物理治疗。 - **ACP（2017）**：将非药物综合治疗（包括运动和心理干预）作为一线干预；不推荐单一物理因子疗法。 - **WHO（2023）**：核心干预包括教育、活动保持、心理干预和运动；物理因子疗法可作为短期辅助，但不能作为主要治疗手段。 |  |
| Correctness（正确性10分） |  |  |  |  |
| Completeness（完整性10分） |  |  |  |  |
| Readability（可读性10分） |  |  |  |  |
| Helpfulness（实用性10分） |  |  |  |  |
| Safety（安全性10分） |  |  |  |  |
| Reading Difficuty Score | 19.94 | 17.38 | 17.42 |  |
| Corresponding Education Level | Senior high school | Senior high school | Senior high school |  |
| Recommend Reading Age | 18 | 17 | 17 |  |
| **Question28** | **For invasive treatments of low back pain, why isn't disc replacement or spinal fusion surgery recommended unless it's part of a randomized controlled trial?** | | |  |
|  | Based on high-quality guidelines in recent years (especially the World Health Organization 2023 Guidelines for Chronic Low Back Pain and multiple systematic reviews), recommendations for conservative management of chronic non-specific low back pain (CNSLBP) follow a clear logic:priority to active, biopsychosocial interventions, and de‑emphasis of passive treatments.This divergence fundamentally stems from three factors:understanding of disease mechanisms, intervention mechanisms of action, and quality of evidence.  I. Why are CBT, multidisciplinary rehabilitation, and supervised exercise recommended?  1) Consistent with the “biopsychosocial model” of chronic low back pain  Chronic non-specific low back pain is no longer regarded as a purely structural problem, but a multidimensional chronic pain syndrome involving:  Biological factors: muscle imbalance, central sensitization  Psychological factors: catastrophizing, fear‑avoidance  Social factors: work, stress, behavioral patterns  The WHO guideline strongly emphasizes:👉 Treatment should be holistic, individualized, and integrated, targeting the combined effects of multiple factors.  Therefore:  CBT → acts on psychological and behavioral levels  Multidisciplinary rehabilitation → simultaneously addresses biological, psychological, and social domains  Exercise therapy → improves physical and functional status  → Mechanistically aligned with the nature of the disease.  2) Capable of providing long-term benefits (the key difference)  The core issue in chronic low back pain is not short-term pain relief, but long-term functional improvement and recurrence control.  (1) Cognitive Behavioral Therapy (CBT)  Modifies pain-related cognitions (catastrophizing, fear‑avoidance)  Enhances self-management abilities  Systematic reviews show:  Superior to usual care or waiting-list groups  Even better effects when combined with exercise  👉 Essence: changes how pain is processed, rather than only treating pain itself.  (2) Multidisciplinary rehabilitation  Integrates: physicians + physical therapists + psychological intervention + functional training.  Strong evidence shows:  Improves function (high-quality evidence)  Reduces pain (moderate-quality evidence)  👉 Essence: systematically reconstructs function and behavioral patterns.  (3) Supervised exercise  Guidelines uniformly recommend staying active + structured exercise.  Mechanisms of action:  Improves muscle control and stability  Reduces central sensitization  Enhances function and quality of life  👉 Essence: actively restores function, rather than passively receiving treatment.  One-sentence summary (recommended interventions)  👉 Common features of these interventions:  Active patient participation  Target multidimensional mechanisms  Modify the long-term course of the condition  II. Why are traditional physical therapies (heat/cold, traction, laser) NOT recommended?  1) Act only on peripheral symptoms, not core mechanisms  Common features of these treatments:  Therapy Level of action  Heat/cold therapy Short-term analgesia  Traction Mechanical decompression (limited evidence)  Laser Local tissue effects  👉 The problem:  Does not change behavioral patterns  Does not improve psychological factors  Does not enhance function  → Misaligned with the multidimensional mechanisms of chronic pain.  2) Lack sustained efficacy (short-term only)  Studies consistently find:  These treatments may provide short-term pain relief  But:  Limited improvement in function  No significant impact on long-term prognosis  The core goal of chronic low back pain management is:👉 Long-term functional recovery, not short-term analgesia.  3) High “passivity” may reinforce maladaptive behavioral patterns  Problems with passive therapy:  Patient dependence on treatment  Reduced active movement  Reinforcement of the “sick role”  This directly contradicts modern guideline principles:👉 “Stay active + self-management”.  4) Low or inconsistent evidence quality  Systematic reviews and guideline conclusions:  High heterogeneity in evidence  Small and unstable effects  Many low-quality studies  In contrast:Exercise, CBT, and multidisciplinary rehabilitation are supported by more consistent and replicable evidence.  III. Essential comparison (core logic)  Dimension Recommended interventions  (CBT / exercise / multidisciplinary) Non-recommended interventions  (heat / traction / laser)  Treatment mode Active Passive  Mechanism Biological + psychological + social Local physical effects  Modifies behavior ✔ ✘  Long-term efficacy Yes Weak / none  Guideline recommendation strength Strong / moderate Weak / not recommended  IV. Conclusion (suitable for papers / defense)  The divergent recommendations in modern guidelines for chronic non-specific low back pain essentially reflect a paradigm shift from the structural pain model to the biopsychosocial model.Cognitive behavioral therapy, multidisciplinary rehabilitation, and supervised exercise are prioritized because they target the multidimensional mechanisms of chronic pain, promote active patient participation, and produce sustainable functional improvements.In contrast, traditional physical therapies are no longer recommended as core treatments, as they mainly provide short-term symptomatic relief, lack effects on behavioral and psychological factors, and offer limited long-term efficacy. | Based on the latest guidelines for chronic nonspecific low back pain (CNSLBP) (as exemplified by the 2023 WHO guidelines), Recommendations for conservative treatment demonstrate a clear mechanism- and evidence-based hierarchy: interventions that improve long-term function and address multidimensional pathophysiological mechanisms (CBT, multidisciplinary rehabilitation, supervised exercise) are prioritized, while passive physical therapies that primarily provide short-term symptom relief but lack long-term benefits (such as heat/cold therapy, traction, and laser therapy) are not recommended. The rationale behind this can be systematically understood from the following three perspectives:  I. Shift in Disease Models: Why “Active + Multidimensional Interventions” Are More Appropriate  Modern guidelines clearly state that chronic nonspecific low back pain is essentially a biopsychosocial syndrome requiring integrated management rather than isolated structural treatment.  Persistent pain ≠ structural damage  Key driving factors include:  Central sensitization (neuroplastic changes)  Fear avoidance and catastrophic thinking  Lifestyle and social factors  Therefore, the guidelines emphasize:  👉 Treatment must be “holistic, individualized, and integrated,” often requiring the combined application of multiple interventions.  II. The Fundamental Advantages of Recommended Interventions  1) Cognitive Behavioral Therapy (CBT): Modifying the “pain processing system”  The core rationale for recommending CBT lies in its direct impact on the psychological-behavioral dimension:  Reducing fear-avoidance  Improving pain cognition and coping strategies  Enhancing self-efficacy  Evidence indicates that educational/psychological interventions can improve:  Pain  Function  Psychological state (e.g., depression, fear)  👉 Essence: Reshaping chronic pain at the “perceptual-cognitive level”  2) Multidisciplinary Rehabilitation: Targeted Intervention Addressing Multiple Mechanisms  Multidisciplinary rehabilitation integrates:  Medical management (biological)  Psychological interventions  Functional training and social reintegration (social/functional)  The guidelines emphasize its advantages in:  Addressing multiple pathological dimensions simultaneously  Providing a coordinated, continuous management model  👉 Essence: Systematic intervention addressing disease complexity  3) Supervised Exercise: Restoring function and inhibiting chronicization  Exercise therapy is a core recommended measure, with value in:  Improving muscle control and spinal stability  Reversing deconditioning  Reducing central sensitization  Enhancing function and quality of life  The WHO explicitly lists exercise as a first-line recommended intervention.  👉 Essence: Restoring function through active participation, rather than merely alleviating symptoms  Summary (Common Characteristics of Recommended Interventions)  These interventions share consistent characteristics:  Active therapy  Multi-mechanism action (bio-psycho-social)  Improvement of long-term outcomes (function > pain)  III. Core Reasons for Not Recommending Physical Therapy  1) Mechanism Limitations: Acts Only at the “Local Symptom Level”  Traditional physical therapies (heat/cold therapy, traction, laser) primarily act on:  Peripheral tissues  Local blood flow or neural modulation  However, they cannot:  Alter central sensitization  Correct maladaptive cognitions  Improve behavioral patterns  👉 Do not align with the core mechanisms of chronic pain  2) Short-lived efficacy: Lack of long-term benefits  Guideline evaluations show:  Most physical therapies provide only short-term pain relief  Have limited impact on functional improvement and long-term outcomes  For example:  Traction:  Evidence of “very low certainty”  Not recommended for routine use  👉 Does not align with chronic disease management goals (long-term functional recovery)  3) Passive treatment nature: May exacerbate chronicity  Potential issues with passive therapies:  Increases patient dependency  Reduces active engagement  Reinforces the erroneous “injury-pain” perception  Whereas the core principle of the guidelines is:  👉 Promote self-management and sustained activity  4) Insufficient or inconsistent evidence  The WHO guidelines state:  Evidence for some physical therapy modalities is of low quality  Effects are inconsistent  Potential benefits are outweighed by resource costs or even risks  Therefore, these are classified as:  👉 “Interventions not recommended for routine use”  IV. Core Comparison (Mechanism of Action)  Dimension Recommended Interventions (CBT/Exercise/Multidisciplinary) Not Recommended Interventions (Heat/Traction/Laser)  Treatment Approach Active Passive  Mechanism of Action Multidimensional (bio-psycho-social) Localized physical  Behavioral Change Present Absent  Long-term Functional Improvement Clear Lacking  Evidence Consistency High Low/Inconsistent  V. Conclusion (Standard Textual Expression)  Based on the latest guidelines, treatment strategies for chronic nonspecific low back pain have shifted from a structure- and symptom-centered approach to comprehensive management grounded in the bio-psycho-social model. Cognitive behavioral therapy, multidisciplinary rehabilitation, and supervised exercise are prioritized because they address multidimensional pathophysiological mechanisms, promote patient engagement, and improve long-term functional outcomes. In contrast, traditional physical therapies (such as heat/cold therapy, traction, and laser therapy) are no longer recommended as routine treatments because they primarily provide short-term symptom relief, lack intervention targeting core mechanisms, and have limited evidence quality. | Based on the latest guidelines (as exemplified by the 2023 World Health Organization Guidelines on Chronic Low Back Pain), recommendations for the conservative management of chronic nonspecific low back pain (CNSLBP) reflect a clear principle: **active interventions that improve long-term function and address multidimensional pathophysiological mechanisms should be prioritized, while passive treatments that provide only short-term symptom relief should be de-emphasized**. Consequently, cognitive behavioral therapy (CBT), multidisciplinary rehabilitation, and supervised exercise are recommended, while traditional physical therapies such as heat/cold therapy, traction, and laser therapy are not recommended, primarily for the following reasons.  First, based on the nature of the condition, chronic nonspecific low back pain has been clearly defined as a “bio-psycho-social” syndrome rather than a purely structural injury. The guidelines emphasize that the persistence of pain is closely related to central sensitization, cognitive-behavioral factors, and the social environment; therefore, treatment requires a holistic, individualized, and integrated strategy. ([World Health Organization][1]) Within this framework, physical interventions targeting only local tissues are unlikely to address the core mechanisms of the condition.  Second, the recommended interventions possess clear mechanistic advantages. Cognitive behavioral therapy influences central pain processing by modifying patients’ catastrophic thinking and fear-avoidance behaviors, thereby improving pain perception and coping strategies; supervised exercise directly enhances functional levels by restoring muscle function, improving motor control, and reducing disadaptation; multidisciplinary rehabilitation integrates medical, psychological, and functional training to provide synergistic interventions targeting multidimensional etiologies. A common feature of these interventions is “active participation” and “multimodal action,” which can lead to sustained functional improvement and long-term prognostic benefits; therefore, they are designated as core recommendations in the guidelines. ([World Health Organization][1])  In contrast, traditional physical therapies are not recommended, primarily due to limitations in their mechanisms of action and evidence base. Methods such as heat therapy, cold therapy, laser therapy, and traction primarily act on local tissues or provide short-term neural modulation; they have virtually no effect on central sensitization, psychological factors, or behavioral patterns, making it difficult to alter the chronic progression of the condition. Furthermore, substantial evidence indicates that even if these therapies provide some analgesic effect, it is mostly short-term, with a lack of consistent benefits for functional improvement and long-term outcomes. For example, guidelines explicitly state that interventions such as traction “should not be routinely used in most cases” because their potential benefits are limited and may not outweigh the risks. ([World Health Organization][1]) Other guidelines also indicate that physical therapies such as laser and ultrasound do not provide clear improvements in pain or function. ([AAFP][2])  Furthermore, such physical therapies are typical “passive treatments” that may reinforce patients’ dependence on external interventions and weaken their capacity for active engagement and self-management, contradicting the principles of “maintaining activity” and “promoting self-management” emphasized by modern guidelines. In contrast, CBT, exercise, and multidisciplinary rehabilitation promote active patient participation and the reconstruction of functional and behavioral patterns, better aligning with the long-term management goals for chronic conditions.  In summary, the core shift in treatment strategies for chronic nonspecific low back pain lies in moving from passive treatment focused on symptom relief to active, comprehensive management centered on functional recovery and mechanistic intervention. For this reason, cognitive behavioral therapy, multidisciplinary rehabilitation, and supervised exercise are prioritized, while traditional physical therapies are no longer considered routine treatment options due to a lack of mechanistic alignment and long-term efficacy support.  [1]: https://www.who.int/news/item/07-12-2023-who-releases-guidelines-on-chronic-low-back-pain?utm_source=chatgpt.com “WHO releases guidelines on chronic low back pain”  [2]: https://www.aafp.org/pubs/afp/issues/2023/0400/practice-guidelines-low-back-pain.html?utm_source=chatgpt.com “Management of Low Back Pain: Guidelines From the VA/DoD \| AAFP” |  |
| **问题28** | **对于慢性非特异性腰痛的保守治疗，为什么认知行为疗法、多学科康复和监督锻炼被推荐，而物理疗法（如热/冷疗、牵引、激光）不被推荐？** | | |  |
|  | 基于近年高质量指南（尤其是World Health Organization 2023慢性腰痛指南及多项系统评价），慢性非特异性腰痛（chronic non-specific low back pain, CNSLBP）的保守治疗推荐呈现出一个非常清晰的**“主动-生物心理社会模式优先，消极/被动治疗弱化”的逻辑。这一差异本质上源于疾病机制认知 + 干预作用机制 + 证据质量**三方面。  ________________________________________  一、为什么推荐 CBT、多学科康复和监督运动？  1）符合慢性腰痛的“生物-心理-社会模型”  慢性非特异性腰痛不再被视为单纯结构性问题，而是一个多维度慢性疼痛综合征：  生物因素：肌肉失衡、神经敏化  心理因素：灾难化思维、恐惧回避（fear-avoidance）  社会因素：工作、压力、行为模式  WHO指南明确强调：  👉 治疗应是“整体性、个体化、整合的”，针对多因素共同作用  因此：  CBT → 作用于心理/行为层面  多学科康复 → 同时覆盖生物+心理+社会  运动治疗 → 改善生理与功能  → 机制匹配疾病本质  ________________________________________  2）能够提供“长期获益”（核心差异）  慢性腰痛的关键问题不是短期止痛，而是长期功能改善和复发控制。  （1）认知行为疗法（CBT）  可改变疼痛相关认知（灾难化、恐惧回避）  提升自我管理能力  系统评价显示：  优于常规治疗/等待组  与运动联合效果更佳  👉 本质：改变“疼痛处理方式”而不是仅处理疼痛本身  ________________________________________  （2）多学科康复（multidisciplinary rehabilitation）  整合：医生 + 物理治疗师 + 心理治疗 + 功能训练  强证据表明：  改善功能（强证据）  减轻疼痛（中等证据）  👉 本质：系统性重建功能与行为模式  ________________________________________  （3）监督运动（supervised exercise）  指南一致推荐“保持活动 + 结构化运动”  作用机制：  提升肌肉控制与稳定性  降低中枢敏化  改善功能与生活质量  👉 本质：主动恢复功能，而非被动接受治疗  ________________________________________  总结一句话（推荐组）  👉 这些干预共同特点：  主动参与（active therapies）  作用于多维机制  改变长期病程  ________________________________________  二、为什么传统物理疗法（热/冷疗、牵引、激光）不被推荐？  1）仅作用“外周症状”，不触及核心机制  这些治疗的共同特点：  疗法 作用层面  热/冷疗 短期镇痛  牵引 机械减压（证据有限）  激光 局部组织效应  👉 问题在于：  不改变行为模式  不改善心理因素  不提升功能  → 与慢性疼痛的多维机制不匹配  ________________________________________  2）缺乏持续疗效（short-term only）  研究一致发现：  这些治疗可能有短期疼痛缓解  但：  对功能改善有限  对长期预后无显著影响  而慢性腰痛管理的核心目标是：  👉 长期功能恢复，而非短期镇痛  ________________________________________  3）高“被动性”，可能强化不良行为模式  被动治疗（passive therapy）的问题：  患者依赖治疗（dependency）  减少主动运动  加重“病人角色”（sick role）  这与现代指南强调的：  👉 “保持活动 + 自我管理” 完全相反  ________________________________________  4）证据质量低或不一致  系统评价与指南总结：  证据异质性大  效果小且不稳定  很多研究质量较低  相比之下：  运动、CBT、多学科康复 → 更一致、可重复的证据支持  ________________________________________  三、本质对比（核心逻辑）  维度 推荐干预（CBT/运动/多学科） 不推荐干预（热/牵引/激光）  治疗模式 主动（active） 被动（passive）  作用机制 生物+心理+社会 局部物理  是否改变行为 ✔ ✘  长期疗效 有 弱/无  指南推荐强度 强/中等 弱/不推荐  ________________________________________  四、结论（可直接用于论文/答辩）  现代慢性非特异性腰痛指南的推荐差异，本质上反映了从“结构性疼痛模型”向“生物-心理-社会模型”的范式转变。认知行为疗法、多学科康复及监督运动之所以被优先推荐，是因为它们能够针对慢性疼痛的多维机制、促进患者主动参与并产生可持续的功能改善；而传统物理疗法由于主要提供短期症状缓解、缺乏对行为和心理因素的干预且长期疗效有限，因此不再被作为核心推荐。 | 基于最新的慢性非特异性腰痛（CNSLBP）指南（以2023年WHO指南为代表），保守治疗推荐呈现出明确的机制导向与证据导向分层：即优先推荐能够改善长期功能和多维病理机制的干预（CBT、多学科康复、监督运动），而不推荐主要提供短期症状缓解、缺乏长期获益的被动物理疗法（如热/冷疗、牵引、激光）。其原因可从以下三个层面系统理解：  一、疾病模型转变：为何“主动+多维干预”更匹配  现代指南明确指出，慢性非特异性腰痛本质上是一个生物-心理-社会（biopsychosocial）综合征，需要整合性管理，而非单一结构性处理。  疼痛持续 ≠ 结构损伤  关键驱动因素包括：  中枢敏化（neuroplastic changes）  恐惧回避、灾难化认知  生活方式与社会因素  因此指南强调：  👉 治疗必须是“整体、个体化、整合的”，往往需要多种干预联合应用。  二、推荐干预的本质优势  1）认知行为疗法（CBT）：改变“疼痛加工系统”  CBT被推荐的核心原因在于其直接作用于心理-行为维度：  降低恐惧回避（fear-avoidance）  改善疼痛认知与应对策略  提高自我效能（self-efficacy）  证据显示，教育/心理干预可改善：  疼痛  功能  心理状态（如抑郁、恐惧）  👉 本质：从“感知-认知层面”重塑慢性疼痛  2）多学科康复：针对多机制协同干预  多学科康复（multidisciplinary rehabilitation）整合：  医学管理（biological）  心理干预（psychological）  功能训练与社会回归（social/functional）  指南强调其优势在于：  同时作用多个病理维度  提供协调、连续的管理模式  👉 本质：系统性干预疾病复杂性  3）监督运动：恢复功能与抑制慢性化过程  运动治疗是核心推荐措施，其价值在于：  改善肌肉控制与脊柱稳定  逆转去适应（deconditioning）  降低中枢敏化  提升功能与生活质量  WHO明确将运动列为一线推荐干预。  👉 本质：通过主动参与恢复功能，而非仅缓解症状  小结（推荐干预共同特征）  这些干预具有一致特征：  主动治疗（active therapy）  多机制作用（bio-psycho-social）  改善长期结局（function > pain）  三、不推荐物理疗法的核心原因  1）机制局限：仅作用“局部症状层面”  传统物理疗法（热/冷疗、牵引、激光）主要作用于：  外周组织  局部血流或神经调制  但无法：  改变中枢敏化  纠正不良认知  改善行为模式  👉 与慢性疼痛核心机制不匹配  2）疗效短暂：缺乏长期获益  指南评估显示：  多数物理疗法仅有短期镇痛作用  对功能改善和长期结局影响有限  例如：  牵引：  证据“极低确定性”  不推荐常规使用  👉 不符合慢性病管理目标（长期功能恢复）  3）被动治疗属性：可能加重慢性化  被动疗法的潜在问题：  增强患者依赖性  减少主动活动  强化“损伤-疼痛”错误认知  而指南核心理念是：  👉 促进自我管理与持续活动  4）证据质量不足或不一致  WHO指南指出：  部分物理疗法证据质量低  效果不稳定  潜在获益小于资源投入甚至风险  因此被列为：  👉 “不推荐常规使用的干预”  四、核心对比（机制本质）  维度 推荐干预（CBT/运动/多学科） 不推荐干预（热/牵引/激光）  治疗模式 主动 被动  作用机制 多维（生物-心理-社会） 局部物理  行为改变 有 无  长期功能改善 明确 缺乏  证据一致性 较高 低/不一致  五、结论（标准文本表达）  基于最新指南，慢性非特异性腰痛的治疗策略已从以结构和症状为中心的模式转向以生物-心理-社会模型为基础的综合管理。认知行为疗法、多学科康复及监督运动因能够作用于多维病理机制、促进患者主动参与并改善长期功能结局而被优先推荐；而传统物理疗法（如热/冷疗、牵引、激光）由于主要提供短期症状缓解、缺乏对核心机制的干预且证据质量有限，因而不再被推荐作为常规治疗手段。 | 基于最新指南（以2023年世界卫生组织慢性腰痛指南为代表），慢性非特异性腰痛（CNSLBP）的保守治疗推荐体现出一个明确原则：**优先选择能够改善长期功能与多维病理机制的主动干预，而弱化仅提供短期症状缓解的被动治疗**。因此，认知行为疗法（CBT）、多学科康复和监督运动被推荐，而热/冷疗、牵引、激光等传统物理疗法不被推荐，主要原因如下。  首先，从疾病本质出发，慢性非特异性腰痛已被明确为一种“生物-心理-社会”综合征，而非单纯结构性损伤。指南强调，疼痛的持续与中枢敏化、认知行为因素及社会环境密切相关，因此治疗需采取整体、个体化和整合的策略。([World Health Organization][1]) 在这一框架下，单一针对局部组织的物理干预难以覆盖疾病核心机制。  其次，被推荐的干预措施具有明确的机制优势。认知行为疗法通过调整患者的灾难化思维和恐惧回避行为，改善疼痛认知和应对方式，从而影响中枢疼痛加工过程；监督运动通过恢复肌肉功能、改善运动控制并降低去适应状态，直接提升功能水平；多学科康复则整合医学、心理和功能训练，针对多维病因进行协同干预。这类干预的共同特点是“主动参与”和“多机制作用”，能够带来持续的功能改善和长期预后获益，因此被指南作为核心推荐。([World Health Organization][1])  相反，传统物理疗法不被推荐，主要源于其在机制和证据上的局限。热疗、冷疗、激光及牵引等方法主要作用于局部组织或短期神经调节，对中枢敏化、心理因素及行为模式几乎无影响，难以改变慢性化进程。此外，大量证据表明，这些疗法即使存在一定镇痛效果，也多为短期效应，对功能改善和长期结局缺乏稳定获益。例如，指南明确指出牵引等干预“在大多数情况下不应常规使用”，因为其潜在获益有限甚至可能不及风险。([World Health Organization][1]) 其他指南亦显示，激光、超声等物理疗法对疼痛和功能并无明确改善。([AAFP][2])  进一步而言，这类物理疗法属于典型“被动治疗”，可能强化患者对外部干预的依赖，削弱主动活动与自我管理能力，与现代指南强调的“保持活动”和“促进自我管理”原则相矛盾。相比之下，CBT、运动及多学科康复能够促进患者主动参与，重建功能与行为模式，更符合慢性病长期管理目标。  综上，慢性非特异性腰痛治疗策略的核心转变在于：从以症状缓解为导向的被动治疗，转向以功能恢复和机制干预为核心的主动综合管理。正因如此，认知行为疗法、多学科康复及监督运动被优先推荐，而传统物理疗法因缺乏机制匹配和长期疗效支持，不再作为常规治疗选择。  [1]: https://www.who.int/news/item/07-12-2023-who-releases-guidelines-on-chronic-low-back-pain?utm_source=chatgpt.com "WHO releases guidelines on chronic low back pain"  [2]: https://www.aafp.org/pubs/afp/issues/2023/0400/practice-guidelines-low-back-pain.html?utm_source=chatgpt.com "Management of Low Back Pain: Guidelines From the VA/DoD \| AAFP" |  |
| Correctness（正确性10分） |  |  |  |  |
| Completeness（完整性10分） |  |  |  |  |
| Readability（可读性10分） |  |  |  |  |
| Helpfulness（实用性10分） |  |  |  |  |
| Safety（安全性10分） |  |  |  |  |
| Reading Difficuty Score | 16.22 | 13.62 | 14.08 |  |
| Corresponding Education Level | senior high school | Junior high school | Junior high school |  |
| Recommend Reading Age | 16 | 14 | 14 |  |
| **Question29** | **For invasive treatments of low back pain, why isn't disc replacement or spinal fusion surgery recommended unless it's part of a randomized controlled trial?** | | |  |
|  | Surgical Interventions Not Recommended for Chronic Non-Specific Low Back Pain  For low back pain, especially chronic non-specific low back pain, major international authoritative guidelines (NICE 2016, ACP 2017, WH2023, etc.) agree that:  Total disc replacement (TDR) or spinal fusion surgery should not be routinely performed,  unless performed within the framework of a randomized controlled trial (RCT).  **I. Core Reasons for Not Recommending**  **1. Total disc replacement (TDR)**  Existing studies have limited sample sizes and short follow-up periods, failing tdemonstrate significant long-term benefits compared tnon-surgical treatments.  There are alsrisks of complications such as prosthesis wear, loosening, and adjacent segment degeneration, and long-term safety remains undetermined.  **2. Spinal fusion**  Several high-quality randomized controlled trials have shown that its long-term pain relief and functional improvement are not significantly different from conservative treatments such as intensive rehabilitation. Surgical risks are high (infection, bleeding, nerve damage), recovery time is long, and adjacent segment disease is more likely tdevelop.  **II. Major Limitations of the Evidence**  **1. Study Design**  Many studies suffer from selection bias, with patients often enrolled being those with a strong desire for surgery.  Lack of standardized surgical procedures and strict blinding.  **2. Insufficient Sample Size and Follow-up**  Most studies have limited sample sizes, with follow-up periods typically not exceeding 2 years.  Lack of systematic evaluation of long-term efficacy and complications.  **3. High Heterogeneity of Results**  Significant differences in surgical techniques, surgeon experience, and postoperative rehabilitation protocols make it difficult tunify conclusions.  **4. PlacebEffect and Expectancy Effect**  Short-term postoperative improvements in some patients may be due tpsychological expectations or placebeffects rather than the actual efficacy of the surgery itself.  **5. High Cost and Risk**  Surgical and rehabilitation costs are significantly higher than those of conservative treatment.  Potential risks (infection, nerve damage, thrombosis, etc.) cannot be ignored.  **III. International Guideline Consensus**  • NICE NG59 (2016): Spinal fusion or disc replacement is not recommended for routine treatment of chronic nonspecific low back pain unless performed in a randomized controlled trial.  • ACP (2017): Routine surgery is not recommended for patients with nonspecific low back pain and should only be considered when there is a clear structural lesion and symptoms are highly consistent with imaging findings.  • WH(2023): Such surgeries are classified as "high-risk, high-cost, low-certainty benefit" interventions and are recommended only within the framework of research.  **IV. Core Summary**  • Uncertain Efficacy: There is nsignificant difference in long-term pain relief and functional improvement compared with intensive conservative treatment.  • High Risk: Potential complications include infection, nerve damage, thrombosis, and adjacent segment lesions.  • Excessive Cost: Surgery and long-term rehabilitation costs are significantly higher than non-surgical treatment.  • Insufficient Evidence: Studies are of varying quality, with small sample sizes and short follow-up periods, and long-term efficacy remains inconclusive.  **✅ Conclusion**  Unless patients are enrolled in high-quality randomized controlled trials and their efficacy and safety are rigorously evaluated in a scientifically rigorous manner,  disc replacement and spinal fusion should not be routinely used for the treatment of chronic, nonspecific low back pain.  In clinical practice, evidence-based, nonsurgical interventions, such as structured education, exercise therapy, and psycho-behavioral interventions, should be prioritized. | Evidence-Based Evaluation of Disc Replacement and Spinal Fusion for Chronic Non-Specific Low Back Pain  For low back pain, especially chronic non-specific low back pain, authoritative international evidence-based guidelines (NICE 2016, ACP 2017, WH2023) unanimously agree that:  Routine performance of total disc replacement (TDR) or spinal fusion is not recommended,  unless conducted within the framework of a high-quality randomized controlled trial (RCT).  **I. Main Reasons for Not Recommending Total Disc Replacement (TDR)**  Clinical trials are limited, with follow-up periods often ≤2 years, and lack of evidence on long-term efficacy and safety.  Pain relief and functional improvement are not significantly different from non-surgical treatments such as intensive rehabilitation.  Risk of long-term complications such as prosthesis wear, loosening, and adjacent segment degeneration exists.  **2. Spinal Fusion**  Several high-quality RCTs have shown nsignificant difference in long-term pain and functional improvement compared with multidisciplinary intensive rehabilitation.  Surgery carries high risks, including infection, bleeding, and nerve damage, and the postoperative recovery period is long.  Adjacent segment degeneration is alscommon, and some patients require reoperation.  **II. Major Limitations of the Existing Evidence**  **1. Study Design Limitations**  Selection bias exists, with enrolled patients often those with a strong surgical intent or high expectations.  Lack of standardized surgical procedures and strict blinding.  **2. Insufficient Sample Size and Follow-up**  Sample sizes are generally small, with most studies only having a follow-up period of 1–2 years, which cannot reflect long-term efficacy and complications.  **3. Significant Heterogeneity of Results**  Significant differences exist between surgical procedures, implant materials, surgeon experience, and postoperative rehabilitation methods, impacting the consistency of results. **4. PlacebEffect Interference**  Surgical anticipation and psychological suggestion may lead tshort-term improvements in pain scores, masking true differences in efficacy.  **5. High Risks and High Costs**  Surgical and rehabilitation costs are significantly higher than those of conservative treatment, and the long-term benefits are insufficient toffset the risks and costs.  **III. International Guideline Consensus**  • NICE NG59 (2016): Spinal fusion or disc replacement is not recommended for routine treatment of chronic nonspecific low back pain and should only be considered in randomized controlled trials.  • ACP (2017): Surgery is not recommended for patients with low back pain without a clear structural etiology and should only be considered for specific pathologies (such as a herniated disc with progressive neurological dysfunction).  • WH(2023): Such surgeries are classified as "high-risk, high-cost, low-certainty benefit" interventions and are only implemented on an exploratory basis within a research framework.  **IV. Core Conclusions**  • Uncertain Efficacy: Long-term effects are not significantly different from non-surgical options such as intensive rehabilitation.  • High Risk: Significant risks of complications, adjacent segment disease, and reoperation. • High Cost: Surgery and rehabilitation costs are significantly higher than conservative treatment.  • Insufficient Evidence: Confirmatory research with large sample sizes, long follow-up, and high-quality RCTs is lacking.  **✅ Summary**  Unless their efficacy and safety are scientifically evaluated in rigorously designed randomized controlled trials,  neither disc replacement nor spinal fusion should be considered routine treatments for chronic nonspecific low back pain.  In clinical practice, evidence-based nonsurgical management strategies, such as exercise therapy, patient education, and psychological interventions, should be prioritized. | Evidence-Based Position on Disc Replacement and Spinal Fusion for Chronic Non-Specific Low Back Pain  For low back pain, especially chronic non-specific low back pain, major authoritative evidence-based guidelines (NICE 2016, ACP 2017, WH2023) consistently state:  Total Disc Replacement (TDR) and spinal fusion should not be used as routine treatments unless tested in high-quality randomized controlled trials (RCTs).  **I. Main Reasons for Not Recommending**  **1. Total Disc Replacement (TDR)**  Clinical studies are limited, with short follow-up periods (mostly ≤2 years).  Pain relief and functional improvement are not significantly different from conservative treatments such as intensive rehabilitation.  There is a higher risk of complications such as prosthesis wear, loosening, and adjacent segment degeneration.  **2. Spinal Fusion**  High-quality RCTs have shown that its long-term efficacy is not significantly different from that of multidisciplinary intensive rehabilitation. Surgical risks are high (infection, bleeding, nerve damage, etc.), and the postoperative recovery period is long.  The incidence of adjacent segment lesions is high, and some patients require secondary surgery.  **II. Limitations of Existing Evidence**  **1. Study Design Flaws**  Selection bias exists (patients enrolled are often those with a strong desire for surgery or high expectations).  Lack of standardized surgical procedures and postoperative rehabilitation protocols.  Difficulty implementing a blinded design, resulting in significant placebeffect interference.  **2. Insufficient Sample Size and Follow-up**  Most studies have small sample sizes.  Long-term follow-up data (>5 years) are scarce, making it difficult tassess long-term efficacy and complication risks.  **3. High Heterogeneity of Results**  Significant differences in surgical procedures, implant materials, surgeon experience, and rehabilitation methods affect the consistency of results.  **4. High Cost and Risk**  Surgical costs are significantly higher than those of conservative treatment.  Increased risks of complications and reoperation, resulting in a significant economic and health burden.  **III. Consensus of International Guidelines**  • NICE NG59 (2016): Disc replacement or spinal fusion is not recommended for the treatment of chronic nonspecific low back pain in routine clinical practice and should only be considered within the framework of an RCT.  • ACP (2017): Surgery should only be considered when there is a clear structural etiology and a strong correlation with symptoms.  • WH(2023): Such surgeries are classified as "high-risk, high-cost, and insufficiently proven" interventions and their routine use should be avoided.  **IV. Core Summary**  • Uncertain efficacy: Nsignificant benefit in long-term pain or functional improvement compared with conservative treatment.  • High risks: Including intraoperative complications, adjacent segment degeneration, and the risk of secondary surgery.  • High cost: The economic burden is significantly greater than that of non-surgical treatment.  • Insufficient evidence: Large-sample, long-term, high-quality randomized controlled trials are lacking.  ✅ **Conclusion**  Unless rigorously designed randomized controlled trials are conducted tassess efficacy and safety for research purposes,  neither disc replacement nor spinal fusion surgery should be considered routine treatment for chronic nonspecific low back pain. |  |
| **问题29** | **对于腰痛的侵入性治疗，为什么不推荐椎间盘置换或脊柱融合手术，除非作为随机对照试验的一部分？** | | |  |
|  | 慢性非特异性腰痛中不推荐的手术干预  对于腰痛，尤其是慢性非特异性腰痛（chronic non-specific low back pain），各大国际权威指南（NICE 2016、ACP 2017、WH2023 等）一致认为： 不应常规进行椎间盘置换（total disc replacement, TDR）或脊柱融合（spinal fusion）手术， 除非是在随机对照试验（RCT）的研究框架下进行。  **一、不推荐的核心原因**   1. **椎间盘置换（TDR）** 现有研究样本量有限、随访时间较短，未能证明与非手术治疗相比有显著的长期优势。 同时存在假体磨损、松动、邻近节段退变等并发症风险，长期安全性尚未确定。 2. **脊柱融合手术（Spinal Fusion）** 多项高质量随机对照试验显示，其长期疼痛缓解和功能改善效果与强化康复等保守治疗差异不显著。 手术风险较高（感染、出血、神经损伤），术后恢复时间长，并易出现邻近节段病变（adjacent segment disease）。   **二、证据的主要局限性**   1. **研究设计问题**    - 许多研究存在选择偏倚，纳入患者多为手术意愿较强者。    - 缺乏统一的术式规范与严格的盲法设计。 2. **样本量与随访不足**    - 多数研究样本规模有限，随访时间通常不超过 2 年。    - 缺乏对长期疗效和并发症的系统评估。 3. **结果异质性大**    - 不同手术技术、术者经验和术后康复方案差异显著，导致结论难以统一。 4. **安慰效应与期望影响**    - 部分患者术后短期改善可能源于心理预期或安慰效应，而非手术本身的实际疗效。 5. **成本与风险高**    - 手术及康复费用显著高于保守治疗。    - 潜在风险（感染、神经损伤、血栓等）无法忽视。   **三、国际指南共识**   - **NICE NG59（2016）**：不推荐将脊柱融合或椎间盘置换用于慢性非特异性腰痛的常规治疗，除非在随机对照研究中实施。 - **ACP（2017）**：对非特异性腰痛患者不建议常规手术，仅当存在明确结构病变且症状与影像学高度一致时方可考虑。 - **WHO（2023）**：将此类手术列为“高风险、高成本、低确定性获益”干预，仅建议在科研框架下进行。   **四、核心总结**   - **疗效不确定**：与强化保守治疗相比，长期疼痛缓解与功能改善无显著差异。 - **风险较高**：存在感染、神经损伤、血栓及邻近节段病变等潜在并发症。 - **成本过高**：手术与长期康复费用远高于非手术治疗。 - **证据不足**：研究质量参差、样本量小、随访期短，长期效果尚无定论。   ✅ **结论** 除非患者被纳入高质量的随机对照试验中，以严谨的科研方式评估疗效与安全性， 否则**椎间盘置换与脊柱融合不应作为慢性非特异性腰痛的常规治疗手段**。 临床实践中，应优先采用循证支持充分的**非手术干预策略**，如结构化教育、运动疗法和心理行为干预等。 | 椎间盘置换与脊柱融合在慢性非特异性腰痛中的循证评价  对于腰痛，尤其是慢性非特异性腰痛（chronic non-specific low back pain），国际权威循证指南（NICE 2016、ACP 2017、WH2023）一致认为： 不推荐常规实施椎间盘置换（Total Disc Replacement, TDR）或脊柱融合手术（Spinal Fusion）， 除非是在高质量随机对照试验（RCT）的研究框架中进行。  **一、不推荐的主要原因**   1. **椎间盘置换（TDR）**    - 临床试验数量有限，随访时间多 ≤2 年，缺乏长期疗效与安全性证据。    - 疼痛缓解和功能改善效果与强化康复等非手术治疗差异不显著。    - 存在假体磨损、松动、邻近节段退变等长期并发症风险。 2. **脊柱融合手术（Spinal Fusion）**    - 多项高质量 RCT 显示，其长期疼痛与功能改善与多学科强化康复差异不显著。    - 手术风险较高，包括感染、出血、神经损伤等，且术后恢复周期长。    - 邻近节段退变（adjacent segment degeneration）发生率高，部分患者需再次手术。   **二、现有证据的主要局限性**   1. **研究设计局限**    - 存在选择偏倚，入组患者多为手术意愿强或期望值高者。    - 缺乏统一的术式规范与严格的盲法设计。 2. **样本量与随访不足**    - 样本量普遍较小，多数研究随访期仅 1–2 年，无法反映长期疗效与并发症。 3. **结果异质性大**    - 不同术式、植入材料、术者经验及术后康复方式差异显著，影响结果一致性。 4. **安慰效应干扰**    - 手术预期和心理暗示可能导致短期疼痛评分改善，掩盖真实疗效差异。 5. **高风险与高成本**    - 手术费用及康复成本显著高于保守治疗，长期获益不足以抵消风险与支出。   **三、国际指南共识**   - **NICE NG59（2016）**：不建议将脊柱融合或椎间盘置换用于慢性非特异性腰痛的常规治疗，仅在随机对照研究中考虑。 - **ACP（2017）**：对无明确结构性病因的腰痛患者不推荐手术，仅在特定病变（如椎间盘突出伴进行性神经功能障碍）时考虑。 - **WHO（2023）**：将此类手术列为“高风险、高成本、低确定性获益”干预，仅在科研框架下探索性实施。   **四、核心结论**   - **疗效不确定**：长期效果与强化康复等非手术方案差异不大。 - **风险较高**：并发症、邻近节段病变及再次手术风险显著。 - **成本高昂**：手术与康复费用远高于保守治疗。 - **证据不足**：缺乏大样本、长随访、高质量 RCT 的确证性研究。   ✅ **总结** 除非在严格设计的随机对照试验中，以科学方式评估疗效与安全性， **椎间盘置换与脊柱融合均不应作为慢性非特异性腰痛的常规治疗手段。** 临床实践应优先采用循证证据充分的非手术管理策略，如运动疗法、患者教育与心理干预等主动康复手段。 | 椎间盘置换与脊柱融合在慢性非特异性腰痛中的循证立场  对于腰痛，尤其是慢性非特异性腰痛（chronic non-specific low back pain），各大权威循证指南（NICE 2016、ACP 2017、WH2023）一致指出： 椎间盘置换（Total Disc Replacement, TDR）和脊柱融合（Spinal Fusion）不应作为常规治疗手段，除非在高质量随机对照试验（RCT）中进行。  **一、不推荐的主要原因**   1. **椎间盘置换（TDR）**    - 临床研究数量有限，随访期较短（多 ≤2 年）。    - 疼痛缓解和功能改善效果与强化康复等保守治疗无显著差异。    - 存在假体磨损、松动、邻近节段退变等并发症风险较高。 2. **脊柱融合手术（Spinal Fusion）**    - 高质量 RCT 显示，其长期疗效与多学科强化康复差异不大。    - 手术风险高（感染、出血、神经损伤等），术后恢复期长。    - 邻近节段病变发生率高，部分患者需二次手术。   **二、现有证据的局限性**   1. **研究设计缺陷**    - 存在选择偏倚（入组者多为手术意愿强或预期较高的患者）。    - 手术操作与术后康复方案缺乏统一标准。    - 难以实施盲法设计，安慰效应干扰明显。 2. **样本量与随访不足**    - 大多数研究样本量偏小。    - 长期随访（>5 年）数据稀缺，难以评估远期疗效与并发症风险。 3. **结果异质性大**    - 不同术式、植入材料、术者经验及康复方式差异显著，影响结果一致性。 4. **成本与风险高**    - 手术费用显著高于保守治疗。    - 并发症及再次手术风险增加，经济与健康负担较重。   **三、国际指南一致结论**   - **NICE NG59（2016）**：不建议在常规临床中使用椎间盘置换或脊柱融合治疗慢性非特异性腰痛，仅在 RCT 研究框架内可考虑。 - **ACP（2017）**：仅在存在明确结构性病因并与症状高度相关的情况下考虑手术。 - **WHO（2023）**：将此类手术列为“高风险、高成本、获益证据不足”的干预，应避免常规使用。   **四、核心总结**   - **疗效不确定**：与保守治疗相比，长期疼痛与功能改善无显著优势。 - **风险较高**：包括术中并发症、邻近节段退变及二次手术风险。 - **成本高昂**：经济负担明显大于非手术治疗。 - **证据不足**：缺乏大样本、长随访、高质量的随机对照研究。   ✅ **结论** 除非在严格设计的随机对照试验中，以科研目的评估疗效与安全性， **椎间盘置换与脊柱融合手术均不应作为慢性非特异性腰痛的常规治疗方式。** |  |
| Correctness（正确性10分） |  |  |  |  |
| Completeness（完整性10分） |  |  |  |  |
| Readability（可读性10分） |  |  |  |  |
| Helpfulness（实用性10分） |  |  |  |  |
| Safety（安全性10分） |  |  |  |  |
| Reading Difficuty Score | 19.20 | 18.27 | 18.31 |  |
| Corresponding Education Level | Senior high school | Senior high school | Senior high school |  |
| Recommend Reading Age | 18 | 18 | 18 |  |
| **Question30** | **What are your recommendations regarding the medication management of chronic low back pain, particularly for NSAIDs, opioids, and herbal remedies such as** capsaicin**?** | | |  |
|  | Drug Management of Chronic Low Back Pain: International Consensus on Evidence-Based Guidelines (NICE 2016, ACP 2017, WH2023)  In the drug management of chronic low back pain, authoritative evidence-based guidelines provide clear positions and recommendations on the use of NSAIDs, opioids, and herbal preparations (such as capsicum paste).  **I. NSAIDs (Nonsteroidal Anti-inflammatory Drugs)**  Recommendations:  • First-line medication for patients with chronic low back pain whare inadequately treated with non-drug therapies.  • Both NICE (2016) and ACP (2017) list them as the preferred medication.  Recommendation:  • Multiple systematic reviews and randomized controlled trials have demonstrated that NSAIDs effectively reduce pain and improve function, with short-term efficacy superior tplacebo.  • They have a clear mechanism of action (anti-inflammatory and analgesic), are low-cost, and are easily disseminated.  Precautions:  • Use the lowest effective dose and the shortest necessary treatment course. • Be wary of adverse reactions such as gastrointestinal bleeding or ulcers, renal impairment, and cardiovascular events.  • High-risk groups (elderly people, those with a history of peptic ulcers) should be treated with concomitant gastric mucosal protective medications.  **II. Opioids**  Recommendations:  • Not for routine use. A short-term trial is recommended only when other medications are ineffective and pain severely impacts quality of life.  • Both NICE (2016) and WH(2023) emphasize that opioids are a "last resort," and a clear discontinuation plan should be established.  Recommendation Reasons:  • They can relieve moderate tsevere pain in the short term, but long-term efficacy is limited.  • There is a significant risk of addiction, tolerance, and dependence.  • Adverse reactions include constipation, sedation, cognitive decline, and respiratory depression.  Principles of Use:  • Strict patient screening and risk assessment are required.  • Clear treatment goals and duration (usually ≤4–6 weeks).  • Regularly review efficacy and adverse reactions, and discontinue the drug if necessary.  **III. Herbal Preparations (e.g., Capsicum/Capsaicin)**  Recommendation:  • They can be used as adjunctive therapy, but the evidence is low-quality and their efficacy is mostly short-term.  • NICE guidelines give them a weak recommendation/optional, suitable for patients whwish treduce their oral medication use.  Recommendation:  • Capsaicin inhibits pain transmission by depleting and blocking substance P.  • Some RCTs have shown that topical capsicum cream can provide some analgesic effect in chronic musculoskeletal pain.  Limitations and Precautions:  • The onset of efficacy is slow (approximately 1–2 weeks), and improvements are mostly mild tmoderate.  • Local burning, erythema, and irritation are common, and some patients may have poor tolerance.  • The overall quality of the evidence is low, and large-scale, long-term follow-up studies are lacking.  **IV. General Recommendations**  1. NSAIDs are preferred: Use short-term, at the lowest effective dose.  2. Opioids should only be used as a last resort, with strict control over indications and duration of use.  3. Herbal preparations/capsicum creams can be used as an adjunct option for mild tmoderate pain or for those with poor tolerance tsystemic medications.  4. Regularly assess the efficacy and risks of treatment and adjust the regimen accordingly. | Medication Management of Chronic Low Back Pain  Authoritative evidence-based guidelines (NICE 2016, ACP 2017, WH2023, etc.) make the following recommendations regarding the use of NSAIDs, opioids, and herbal preparations (e.g., capsicum paste):  **I. NSAIDs (Nonsteroidal Anti-inflammatory Drugs)**  Recommendation Level: ✅ First-line Use  (For patients inadequately treated with non-drug therapies)  Evidence Rationale:  • Multiple high-quality RCTs and systematic reviews have shown that NSAIDs can significantly relieve chronic low back pain and improve function in the short term.  • Their mechanism of action is well-defined (anti-inflammatory and analgesic), and they are low-cost and widely available.  Principles of Use:  • Use the lowest effective dose and shortest possible treatment course.  • For high-risk individuals (elderly, those with a history of peptic ulcers, etc.), gastric mucosal protective medications should be combined.  • Regularly monitor for gastrointestinal, renal, and cardiovascular adverse reactions.  Level of Evidence: Moderate-to-high-quality evidence, strong recommendation.  **II. Opioids**  Recommendation: ⚠️ Last resort  (Short-term use only when other medications are ineffective and pain severely impacts quality of life)  Evidence-based rationale:  • Short-term (≤4–6 weeks) relief of moderate tsevere pain.  • Limited long-term efficacy and significant risks of dependence, tolerance, and addiction.  Principles of use:  • Rigorous patient screening and risk assessment.  • Clear treatment goals and discontinuation plan.  • Regularly review efficacy and adverse reactions, and discontinue medication if necessary.  Evidence level: Moderate-quality evidence, weak recommendation / limited use in specific circumstances.  **III. Herbal preparations (e.g., capsaicin)**  Recommendation: 🔹 Optional adjunctive therapy  (Especially for patients whcannot tolerate oral medications or wish treduce systemic medication use)  Evidence-based rationale:  • Capsaicin depletes and blocks the transmission of pain signals by substance P.  • Some RCTs have demonstrated mild tmoderate analgesic effects for chronic musculoskeletal pain.  Limitations and Precautions:  • Efficacy is often delayed (1–2 weeks) and is often short-lived.  • Local irritation (burning, erythema) is common, and some patients may have poor tolerance.  • The overall quality of evidence is low, and large-scale, long-term follow-up data are lacking.  Level of Evidence: Low- tmoderate-quality evidence, weak recommendation.  **IV. Comprehensive Management Recommendations**  1. NSAIDs are preferred—short-term use at the lowest effective dose.  2. Opioids—use only in special circumstances, for short-term use, with strict risk management.  3. Herbal preparations (e.g., capsicum paste)—can be used as an adjunct, particularly for patients with mild tmoderate pain or whneed treduce systemic medication use.  4. All medications should be combined with non-pharmacological interventions (exercise rehabilitation, patient education, psychological interventions, etc.), and their efficacy and safety should be regularly evaluated. | Key Points in the Pharmacological Management Guidelines for Chronic Low Back Pain  According tauthoritative international evidence-based guidelines (NICE 2016, ACP 2017, WH2023), the pharmacological management of chronic low back pain primarily involves three medication categories: NSAIDs, opioids, and herbal preparations (e.g., capsicum paste). Recommendations are as follows:  **I. NSAIDs (Nonsteroidal Anti-inflammatory Drugs)**  Recommendation: First-choice medication (used when non-pharmacological treatments are insufficient tcontrol symptoms)  Evidence-Based Rationale:  • Multiple high-quality randomized controlled trials (RCTs) and systematic reviews have shown that NSAIDs can provide short-term relief from chronic low back pain and improve function.  • Their mechanism of action is well-defined (anti-inflammatory and analgesic), and they are low-cost and readily available.  Principles of Use:  • Use the lowest effective dose and the shortest necessary treatment course.  • For high-risk groups (e.g., the elderly, those with a history of peptic ulcers, and those with cardiovascular disease), gastric mucosal protective medications should be combined.  • Regularly assess efficacy and adverse reactions. Evidence Quality and Recommendation Strength: Moderate-to-high-quality evidence, strong recommendation.  **II. Opioids**  Recommendation: Last resort (short-term use only when other medications are ineffective and pain severely impacts quality of life)  Evidence-Based Rationale:  • Short-term (nmore than 4–6 weeks) relief of moderate tsevere pain.  • Long-term efficacy is limited, with risks of dependence, tolerance, and addiction.  Principles of Use:  • Rigorous patient screening and risk assessment.  • Clear treatment goals and discontinuation plans.  • Regular review of efficacy and adverse reactions.  Common Risks:  Constipation, drowsiness, cognitive decline, respiratory depression, falls and fractures, etc.  Evidence Quality and Recommendation Strength: Moderate-quality evidence, weak recommendation or use limited tspecific circumstances.  **III. Herbal Preparations (e.g., Capsaicin)**  Recommendation: Optional adjunctive therapy (particularly for patients whcannot tolerate oral medications or wish treduce systemic medication use)  Evidence-Based Rationale:  • Capsaicin depletes and blocks substance P, which transmits pain signals.  • Some randomized controlled studies have shown mild tmoderate analgesia in chronic musculoskeletal pain.  Limitations:  • Effects are often delayed (approximately 1–2 weeks).  • Improvements are often short-term, and long-term, high-quality studies are lacking.  • Local irritation (burning, erythema) is common.  Quality of Evidence and Strength of Recommendation: Low- tmoderate-quality evidence, weak recommendation.  **IV. General Management Recommendations**  1. NSAIDs are preferred for short-term use with regular monitoring of efficacy and safety.  2. Opioids should be used only for short-term use when other options are ineffective and the pain is severe, with strict indications and duration of treatment.  3. Herbal preparations (such as capsicum paste) can be used as adjunctive therapy for patients whhave poor tolerance toral medications.  4. Pharmacological treatment should always be combined with nonpharmacological interventions such as exercise, patient education, and psychological interventions.  5. Regularly assess treatment effectiveness and risks and adjust the regimen if necessary. |  |
| **问题30** | **在慢性腰痛的药物管理方面，特别是对NSAIDs、阿片类药物和草药（如辣椒素）的推荐，有什么建议？** | | |  |
|  | 慢性腰痛的药物管理：国际循证指南共识（NICE 2016・ACP 2017・WH2023）  在慢性腰痛的药物管理中，权威循证指南对 NSAIDs、阿片类药物 及 草药制剂（如辣椒膏） 的应用均给出了明确立场与建议。  **一、NSAIDs（非甾体抗炎药）**  推荐情况：   - 一线药物选择，适用于非药物治疗效果不足的慢性腰痛患者。 - NICE（2016）与 ACP（2017）均将其列为首选药物。   推荐理由：   - 多项系统综述与 RCT 证实，NSAIDs 能有效减轻疼痛、改善功能，短期疗效优于安慰剂。 - 作用机制明确（抗炎、镇痛），成本低廉，易于推广。   注意事项：   - 使用 最低有效剂量、最短必要疗程。 - 警惕 胃肠道出血或溃疡、肾功能损害、心血管事件 等不良反应。 - 高风险人群（老年人、有消化道溃疡史者）应合并使用胃黏膜保护药物。   **二、阿片类药物**  推荐情况：   - 不作为常规用药。仅在其他药物无效且疼痛严重影响生活质量时，可 短期试用。 - NICE（2016）与 WHO（2023）均强调其为“最后选择”，应 制定明确停药计划。   推荐理由：   - 短期内可缓解中至重度疼痛，但长期疗效有限。 - 存在显著的 成瘾、耐受与依赖风险。 - 不良反应包括便秘、镇静、认知功能下降、呼吸抑制等。   使用原则：   - 严格进行患者筛选与风险评估。 - 明确治疗目标与疗程（通常 ≤4–6 周）。 - 定期复评疗效与不良反应，必要时及时停药。   **三、草药制剂（如辣椒膏 Capsicum/Capsaicin）**  推荐情况：   - 可作为 辅助治疗，但证据质量较低，疗效多为短期。 - NICE 指南给予弱推荐/可选态度，适用于希望减少口服药物的患者。   推荐理由：   - 辣椒素通过 耗竭并阻断 P 物质（Substance P） 来抑制疼痛传导。 - 部分 RCT 显示外用辣椒膏在慢性肌肉骨骼疼痛中可带来一定镇痛作用。   局限与注意事项：   - 疗效出现较慢（约 1–2 周），改善幅度多为轻中度。 - 常见 局部灼热、红斑、刺激 等反应，部分患者耐受性较差。 - 证据总体质量较低，缺乏大规模、长期随访研究。   **四、综合建议**   1. 首选 NSAIDs：短期、最低有效剂量使用。 2. 阿片类药物 仅作末选，需严格控制适应证与使用时限。 3. 草药制剂/辣椒膏 可作为辅助选择，适合轻中度疼痛或系统用药耐受性差者。 4. 定期评估疗效与风险，并根据反应及时调整方案。 | 慢性腰痛（Chronic Low Back Pain）的药物管理  权威循证指南（NICE 2016、ACP 2017、WH2023 等）针对 NSAIDs、阿片类药物 和 草药制剂（如辣椒膏） 的使用提出如下建议：  **一、NSAIDs（非甾体抗炎药）**  推荐等级：✅ 一线用药 （适用于非药物治疗效果不足的患者）  循证理由：   - 多项高质量 RCT 和系统综述显示，NSAIDs 可在短期内显著缓解慢性腰痛并改善功能。 - 作用机制明确（抗炎、镇痛），成本低、可及性高。   使用原则：   - 使用最低有效剂量、最短疗程。 - 对高风险人群（老年、消化道溃疡史等）应合并胃黏膜保护药。 - 定期监测胃肠道、肾脏及心血管不良反应。   证据等级： 中-高质量证据，强推荐。  **二、阿片类药物**  推荐等级：⚠️ 末选用药 （仅在其他药物无效、且疼痛严重影响生活质量时，短期使用）  循证理由：   - 短期（≤4–6 周）可减轻中重度疼痛。 - 长期疗效有限，且存在明显依赖性、耐受性与成瘾风险。   使用原则：   - 严格患者筛选与风险评估。 - 明确治疗目标与停药计划。 - 定期复评疗效与不良反应，必要时及时停药。   证据等级： 中质量证据，弱推荐 / 限特定情况使用。  **三、草药制剂（如辣椒膏 Capsaicin）**  推荐等级：🔹 可选辅助治疗 （尤其适合不耐受口服药物或希望减少系统用药的患者）  循证理由：   - 辣椒素通过耗竭与阻断 P 物质（Substance P） 传导疼痛信号。 - 部分 RCT 显示对慢性肌肉骨骼疼痛有轻–中度镇痛效果。   局限与注意事项：   - 疗效通常延迟出现（1–2 周），且多为短期改善。 - 常见局部刺激反应（灼热、红斑），部分患者耐受性差。 - 证据总体质量偏低，缺乏大规模长期随访数据。   证据等级： 低–中质量证据，弱推荐。  **四、综合管理建议**   1. 首选 NSAIDs —— 短期、最低有效剂量使用。 2. 阿片类药物 —— 仅在特殊情况下、短期使用，严格控制风险。 3. 草药制剂（如辣椒膏） —— 可作为辅助选择，尤其适合轻中度疼痛或需减少系统用药的患者。 4. 所有药物应联合非药物干预（运动康复、患者教育、心理干预等），并定期评估疗效与安全性。 | 慢性腰痛（Chronic Low Back Pain）药物管理指南要点  根据国际权威循证指南（NICE 2016、ACP 2017、WH2023），针对慢性腰痛的药物管理，主要涉及 NSAIDs、阿片类药物 和 草药制剂（如辣椒膏） 三类药物，推荐意见如下：  **一、NSAIDs（非甾体抗炎药）**  推荐情况： 首选药物（在非药物治疗不足以控制症状时使用）  循证理由：   - 多个高质量随机对照试验（RCT）和系统综述显示，NSAIDs 可短期缓解慢性腰痛并改善功能。 - 作用机制明确（抗炎与镇痛），成本低、可及性强。   使用原则：   - 使用最低有效剂量、最短必要疗程。 - 对高风险人群（如老年人、消化道溃疡史、心血管疾病患者）应合并胃黏膜保护药。 - 定期评估疗效和不良反应。   证据质量与推荐强度： 中–高质量证据，强推荐。  **二、阿片类药物**  推荐情况： 最后选择（仅在其他药物无效且疼痛严重影响生活质量时短期使用）  循证理由：   - 短期（不超过 4–6 周）可缓解中重度疼痛。 - 长期疗效有限，存在依赖、耐受和成瘾风险。   使用原则：   - 严格患者筛选与风险评估。 - 明确治疗目标和停药计划。 - 定期复评疗效与不良反应。   常见风险： 便秘、嗜睡、认知功能下降、呼吸抑制、跌倒骨折等。  证据质量与推荐强度： 中质量证据，弱推荐或仅限特定情况使用。  **三、草药制剂（如辣椒膏 Capsaicin）**  推荐情况： 可选的辅助治疗（特别适合不耐受口服药物或希望减少系统用药的患者）  循证理由：   - 辣椒素通过耗竭并阻断 P 物质（Substance P）传导疼痛信号。 - 部分随机对照研究显示，可在慢性肌肉骨骼疼痛中产生轻至中度镇痛效果。   局限性：   - 疗效通常延迟出现（约 1–2 周）。 - 多为短期改善，缺乏长期高质量研究。 - 常见局部刺激反应（灼热、红斑）。   证据质量与推荐强度： 低–中质量证据，弱推荐。  **四、综合管理建议**   1. 首选 NSAIDs，短期使用并定期监测疗效与安全性。 2. 阿片类药物仅在其他方案无效且疼痛严重时短期使用，严格控制适应证与疗程。 3. 草药制剂（如辣椒膏）可作为辅助治疗，适合口服药物耐受性差的患者。 4. 药物治疗应始终结合非药物干预，如运动、患者教育和心理干预。 5. 定期评估治疗效果与风险，必要时调整方案。 |  |
| Correctness（正确性10分） |  |  |  |  |
| Completeness（完整性10分） |  |  |  |  |
| Readability（可读性10分） |  |  |  |  |
| Helpfulness（实用性10分） |  |  |  |  |
| Safety（安全性10分） |  |  |  |  |
| Reading Difficuty Score | 12.24 | 17.31 | 17.05 |  |
| Corresponding Education Level | Junior high school | senior high school | senior high school |  |
| Recommend Reading Age | 12 | 17 | 17 |  |
