# Supplementary material for: Deficiencies in clinical reasoning of LLMs in low back pain management and remediation via prompt engineering: from performance evaluation to error diagnosis
Source: Front Artif Intell. 2026 May 25;9:1811701. doi: 10.3389/frai.2026.1811701 (PMC13243384; doi:10.3389/frai.2026.1811701)
Supplement: Supplementary file 1 [file Data_Sheet_1.zip › 补充材料/Supplementary materials 3/S2(GPT-o3).docx]

| **Table S2: GPT-o3 answers to 30 questions** | | | | |
| --- | --- | --- | --- | --- |
| **Question1** | | **How do you define and categorize low back pain?** | | |
|  | | **1. Anatomical definition**  • Low back pain refers to pain occurring between the lower edge of the 12th rib and the gluteal fold, which may or may not be accompanied by radiating pain in the lower limbs.  **2. Classification based on disease course (duration)**  • Acute: Duration of less than 6 weeks, with most patients experiencing spontaneous resolution within a few weeks.  • Subacute: Duration of 6 to 12 weeks, representing a critical transition period from acute to chronic.  • Chronic or persistent: Duration exceeding 12 weeks (i.e., more than 3 months), classified under the ICD-11 category of “chronic pain.”  **3. Classification by Etiology (Specificity)**  • Specific low back pain: Caused by identifiable structural or systemic etiologies, such as vertebral fractures, tumors, infections, inflammatory spondyloarthropathies, or nerve root compression, accounting for approximately 5–10%.  • Nonspecific low back pain: No specific structural abnormalities are identified to explain the pain, often associated with postural load, muscle control disorders, and psychosocial factors, accounting for approximately 90%.  **4. Classification based on pain mechanisms and clinical manifestations**  • Mechanical low back pain: Typically caused by degenerative changes, poor posture, or excessive load, with pain worsening during activity and improving with rest.  • Inflammatory low back pain: Common in ankylosing spondylitis, characterized by significant morning stiffness that improves with activity, often accompanied by nighttime pain awakening.  • Radicular low back pain (e.g., sciatica): Characterized by low back pain accompanied by radiating pain, numbness, or weakness along the nerve distribution, indicating nerve root compression.  • Referred pain: Pain originating from pelvic or abdominal organs that projects to the low back, such as in cases of kidney stones or aortic aneurysms.  **5. Application of the ICD-11 chronic pain classification in low back pain**  • MG30.02: Chronic primary musculoskeletal pain, applicable to chronic nonspecific low back pain.  • MG30.3X: Chronic secondary musculoskeletal pain, applicable to chronic low back pain caused by degenerative changes, fractures, etc.  • MG30.5: Chronic neuropathic pain, applicable to low back pain caused by postoperative or postherpetic neuralgia-related nerve damage.  **6. Red flag and yellow flag stratification (for prognosis assessment and referral criteria)**  • Red flags: indicate the possible presence of a serious specific cause and require immediate imaging or specialist evaluation. These include persistent nighttime pain, fever, sudden weight loss, recent severe trauma, immunosuppression, and a history of cancer.  • Yellow Flags: Indicating risk of chronicity or functional impairment, requiring multidisciplinary intervention. These include psychological and social factors such as low mood, catastrophic thinking, fear-avoidance behavior, and low job satisfaction.  **7. Key points for clinical use**  • During the initial visit, focus on identifying red flags to detect serious specific causes as early as possible.  • Develop individualized management strategies based on the stage of the disease and the mechanism of pain.  • For patients with chronic low back pain or high-risk patients, implement a multimodal treatment strategy that includes rehabilitation, patient education, and behavioral intervention, based on the ICD-11 classification and the biopsychosocial model. | **I. Definition**  • Anatomical scope: Pain ranging from the lower edge of the 12th rib to the gluteal fold, which may or may not be accompanied by lower limb pain, with radiation extending to the buttocks, thighs, or calves.  • Related Terms:  o Axial low back pain: Pain primarily localized to the lumbar region.  o Sciatica/radicular pain: Pain radiating along the nerve root segment to the lower extremities, often accompanied by numbness, pins-and-needles sensations, electric shock-like sensations, or changes in muscle strength or reflexes.  o Referred pain: Pain originating from structures outside the spine (e.g., kidneys, abdominal aorta, pelvis) and projected to the lumbar region.  **II. Classification by duration (Time-based)**  • Acute low back pain: lasting less than 6 weeks.  • Subacute low back pain: lasting 6 to 12 weeks.  • Chronic or persistent low back pain: lasting more than 12 weeks, including recurrent episodes.  **III. Classification by etiological specificity (Specificity)**  • Specific low back pain: Caused by a clear structural or systemic etiology, such as vertebral fractures, tumors or metastases, infections, inflammatory spondyloarthropathies, spinal cord or cauda equina syndrome, or significant nerve root compression.  • Nonspecific low back pain: No single clear etiology is identified, often associated with postural load, muscle control disorders, and psychosocial factors, and is the most common clinical type.  Practical tip: During the initial clinical examination, “specific” low back pain and related critical signs (see “red flags”) should be ruled out first. The majority of remaining patients should be managed according to the non-specific management process.  **IV. Classification based on pain mechanism (Mechanism-based)**  • Noxious/mechanical pain: Related to posture or load, worsening with activity and improving with rest. Common causes include disc-related issues, facet joint problems, sacroiliac joint issues, or myofascial problems.  • Neuropathic pain (radicular): Characterized by referred pain in dermatomal distribution, accompanied by numbness, electric shock-like sensations, a positive straight leg raise test, and may be associated with muscle weakness or abnormal reflexes. Commonly seen in nerve root compression caused by disc herniation or spinal canal/lateral recess stenosis.  • Inflammatory low back pain: characterized by morning stiffness lasting more than 30 minutes, nighttime pain awakening, and relief after activity, commonly seen in ankylosing spondylitis or axial spondyloarthritis.  • Visceral referred pain: typically unchanged with body position or activity, accompanied by related internal medical symptoms such as renal colic, abdominal aortic aneurysm, pancreatitis, or gynecological conditions.  **V. Classification by pain distribution (region-based)**  • Pure axial low back pain.  • Low back pain with referred pain in the buttocks or upper thigh.  • Radicular lower limb pain (sciatica) or spinal-related intermittent claudication.  **VI. Functional and severity grading (Impact/Severity)**  • Pain intensity assessment: Use a numerical rating scale (NRS 0–10).  • Functional impairment assessment: Use the Oswestry Disability Index (ODI, 0–100%) or the Roland-Morris Disability Questionnaire (RMDQ, 0–24).  • Social Function Assessment: Includes work capacity, sick leave days, and degree of limitation in daily activities.  Clinically, patients are often classified into low, moderate, or high-risk categories based on the above assessments to match intervention intensity and guide multi-level management.  **7. Red Flag and Yellow Flag Stratification (for triage and prognosis assessment)**  • Red flag factors: indicate potential specific etiologies or critical conditions, including recent major trauma, persistent nocturnal or resting pain, fever or signs of infection, unexplained weight loss, history of tumors, long-term steroid use or osteoporosis, severe or progressive neurological deficits, urinary or bowel dysfunction, etc. (may suggest cauda equina syndrome).  • Yellow flag factors: Psychosocial factors indicating chronicity and disability risk in low back pain, including catastrophic thinking, fear-avoidance behavior, anxiety or depression, low job satisfaction, excessive medical expectations, and social pressure.  **8. Correspondence with ICD-11 classification (optional)**  • Chronic primary musculoskeletal pain: Commonly seen in chronic nonspecific low back pain.  • Chronic secondary musculoskeletal pain: Seen in chronic low back pain caused by degenerative changes, fractures, tumors, or infections.  • Chronic neuropathic pain: Caused by definite nerve damage or persistent radicular pain. | **1. Definition (What counts as “low back pain”)**  Low back pain (LBP) refers to pain occurring between the lower edge of the 12th rib and the gluteal fold, which may or may not be accompanied by symptoms such as radiating pain, numbness, or weakness in the lower limbs.  **2. Classification by duration (Time-based)**  • Acute low back pain: lasts less than 6 weeks, with most cases resolving spontaneously within 4–6 weeks.  • Subacute low back pain: lasts 6–12 weeks, representing a transitional phase from acute to chronic, with early intervention recommended.  • Chronic or persistent low back pain: lasts more than 12 weeks or recurs, falling under the “chronic pain” category in ICD-11.  **3. Classification by etiological “specificity” (Specific vs Non-specific)**  • Specific low back pain: Structural or systemic causes can be clearly identified, such as fractures, tumors, infections, inflammatory spondyloarthropathies, or significant nerve root compression, accounting for approximately 5–10%.  • Non-specific low back pain: No single specific pain-causing structure can be identified through imaging or laboratory tests, often associated with postural load, muscle control disorders, and psychosocial factors, accounting for approximately 90%.  Clinical note: At the initial visit, specific causes (i.e., “red flag” signs) should be prioritized for exclusion; the majority of remaining patients can be managed according to the non-specific pathway.  **4. Classification based on pain mechanism**  • Mechanical/nociceptive pain: Worsens with activity or load, and improves with rest or posture changes. Commonly seen in discogenic lesions, vertebral or facet joint degeneration, and myofascial imbalance.  • Neuropathic (radicular) pain: Radiating along dermatomes, with electric shock-like pain, possibly accompanied by sensory loss or muscle weakness. Typical causes include disc herniation, spinal canal or lateral recess stenosis.  • Inflammatory pain: Morning stiffness lasting over 30 minutes, nighttime pain awakening, relieved by activity. Common in ankylosing spondylitis, psoriatic arthritis, etc.  • Referred/visceral pain: Weakly related to body position, often accompanied by internal medical symptoms. Common in conditions such as renal colic, aortic aneurysm, pancreatic diseases, or gynecological disorders.  **5. Correspondence between ICD-11 and low back pain**  • MG30.02 Chronic primary musculoskeletal pain: Typical of chronic nonspecific low back pain.  • MG30.3X Chronic secondary musculoskeletal pain: caused by secondary factors such as degeneration, fractures, tumors, or infections.  • MG30.5 Chronic neuropathic pain: characterized by confirmed nerve damage or persistent radicular pain.  **6. Red Flags and Yellow Flags**  • Red flags (indicating the need to rule out serious specific causes):  o Recent major trauma  o Persistent night pain  o Fever, significant weight loss  o History of malignant tumors  o Long-term hormone use or osteoporosis  o Severe or progressive neurological deficits  o Urinary or fecal incontinence (suggesting cauda equina syndrome)  → Patients with red flags should undergo imaging studies or specialist evaluation as soon as possible  • Yellow flags (suggesting risk of chronicity and functional disability):  o Catastrophizing  o Fear-avoidance behavior  o Depression, anxiety  o Low job satisfaction  o Pending compensation or litigation  o High psychosocial stress, etc.  → Early psychological-social assessment and multidisciplinary intervention should be conducted, such as cognitive-behavioral therapy or a tiered rehabilitation plan  **7. Rapid clinical application approach**  1. Initial screening for red flag signs: If red flags are present, imaging studies or referral to a specialist should be arranged immediately.  2. Assess disease course and pain mechanisms: Determine whether the condition is acute or chronic, and identify whether the pain is mechanical, radicular, inflammatory, or referred.  3. Assess yellow flag factors and functional impact: such as ODI, RMDQ scores, work absence status, etc.  4. Implement a tiered intervention strategy:  o Low-risk patients: Focus on health education, maintaining activity, and routine pain medication.  o Moderate-to-high-risk or chronic low back pain patients: Recommend adding physical therapy, personalized strengthening exercises, and cognitive-behavioral intervention; consider interventional therapy or surgical evaluation as needed. |
| **问题1** | | **如何定义和分类腰痛？** | | |
|  | | **1. 解剖学定义**   - 腰痛是指自第 12 肋骨下缘至臀下皱褶之间发生的疼痛，可伴随或不伴随下肢放射痛。   **2. 按病程（时间）分型**   - 急性：病程少于 6 周，多数患者可在数周内自然缓解。 - 亚急性：病程为 6 至 12 周，是急性未愈向慢性过渡的关键期。 - 慢性或持续性：病程超过 12 周，即超过 3 个月，归入 ICD-11 “慢性疼痛”范畴。   **3. 按病因（特异性）分型**   - 特异性腰痛：由明确结构或系统性病因引起，如椎体骨折、肿瘤、感染、炎性脊柱关节病、神经根受压等，约占 5–10%。 - 非特异性腰痛：未发现可解释疼痛的特定结构异常，常与姿势负荷、肌肉控制障碍及心理社会因素相关，约占 90%。   **4. 按疼痛机制与临床表现细分类别**   - 机械性腰痛：通常由退行性改变、姿势不良或负荷过度诱发，疼痛在活动时加重，休息后缓解。 - 炎性腰痛：常见于强直性脊柱炎，晨僵明显，活动后缓解，常有夜间痛醒表现。 - 神经根型腰痛（如坐骨神经痛）：表现为腰痛伴沿神经走向的放射痛、麻木或无力，提示存在神经根受压。 - 参照痛：源自盆腔、腹腔等器官的疼痛向腰部投射，例如肾结石、主动脉瘤等情况。   **5. ICD-11 慢性疼痛分类在腰痛中的应用**   - MG30.02：慢性原发性肌肉骨骼疼痛，适用于慢性非特异性腰痛。 - MG30.3X：慢性继发性肌肉骨骼疼痛，适用于退行性变、骨折等引起的慢性腰痛。 - MG30.5：慢性神经病理性疼痛，适用于术后或带状疱疹后神经损伤导致的腰痛。   **6. 红旗与黄旗分层提示（用于预后评估与转诊指征）**   - 红旗（Red Flags）：提示可能存在严重特异性病因，应立即进行影像学或专科评估。包括夜间持续性疼痛、发热、体重骤减、近期严重外伤、免疫抑制状态、既往癌症病史等。 - 黄旗（Yellow Flags）：提示慢性化或功能障碍风险，需多学科干预。包括情绪低落、灾难化思维、恐惧回避行为、工作满意度低等心理社会因素。   **7. 临床使用要点**   - 初次就诊时应重点排查红旗信号，尽早识别严重特异性病因。 - 根据病程阶段与疼痛机制制定个体化管理策略。 - 对慢性腰痛或高风险患者，结合 ICD-11 分类与生物–心理–社会模型评估，实施包括康复、患者教育和行为干预在内的多模态治疗策略。 | **一、定义（Definition）**   - **解剖范围**：指自第 12 肋骨下缘至臀下皱襞之间的疼痛，可伴随或不伴随下肢疼痛，放射范围可至臀部、大腿或小腿。 - **相关术语**：   - 轴性腰痛：疼痛主要局限于腰背部。   - 坐骨神经痛 / 根性痛：沿神经根节段向下肢放射，常伴麻木、针刺、电击感或肌力反射改变。   - 参照痛：源自脊柱外器官或结构（如肾脏、腹主动脉、盆腔）所投射至腰部的疼痛。   **二、按病程分型（Time-based）**   - 急性腰痛：持续时间少于 6 周。 - 亚急性腰痛：持续时间为 6 至 12 周。 - 慢性或持续性腰痛：持续时间超过 12 周，亦包括反复发作型。   **三、按病因“特异性”分型（Specificity）**   - 特异性腰痛：具有明确的结构性或系统性病因，如椎体骨折、肿瘤或转移、感染、炎性脊柱关节病、脊髓或马尾综合征、显著神经根受压等。 - 非特异性腰痛：未发现单一明确的致痛病因，常与姿势负荷、肌肉控制障碍及心理社会因素相关，是最常见的临床类型。   **实务提示**：临床首诊应优先排除“特异性”腰痛及相关危重征象（参见“红旗”），其余多数患者应按非特异性管理流程处理。  **四、按疼痛机制分型（Mechanism-based）**   - **伤害感受性 / 机械性疼痛**：与姿势或负荷相关，活动时加重、休息时缓解。常见病因包括椎间盘源性、关节突关节、骶髂关节或肌筋膜问题。 - **神经病理性疼痛（根性）**：表现为皮节分布的放射痛，伴麻木、电击感，直腿抬高试验阳性，可能合并肌力减退或反射异常。常见于椎间盘突出引起的神经根压迫或椎管、侧隐窝狭窄。 - **炎性腰痛**：以晨僵持续超过 30 分钟、夜间痛醒、活动后缓解为特征，常见于强直性脊柱炎或轴性脊柱关节炎。 - **内脏参照痛**：通常不随体位活动而改变，伴有相关内科症状，如肾绞痛、腹主动脉瘤、胰腺炎或妇科疾病等。   **五、按疼痛分布分型（Region-based）**   - 单纯轴性腰痛。 - 腰痛伴臀部或大腿上段参照痛。 - 根性下肢痛（坐骨神经痛）或脊柱源性间歇性跛行。   **六、功能与严重度分级（Impact / Severity）**   - **疼痛强度评估**：使用数字评分量表（NRS 0–10）。 - **功能障碍评估**：采用 Oswestry 功能障碍指数（ODI，0–100%）或 Roland-Morris 功能障碍问卷（RMDQ，0–24）。 - **社会功能评估**：包括工作能力、病假天数与日常活动受限程度。   临床上常据上述评估将患者分为低、中、高风险等级，以匹配干预强度并指导多层次管理。  **七、红旗与黄旗分层提示（用于分诊与预后评估）**   - **红旗因素**：提示可能存在特异性病因或危重情形，包括近期重大外伤、持续性夜间痛或静息痛、发热或感染迹象、体重不明原因下降、既往肿瘤史、长期激素使用或骨质疏松、严重或进行性神经功能缺失、排尿或排便障碍等（可能提示马尾综合征）。 - **黄旗因素**：提示腰痛慢性化和残疾风险的心理社会因素，包括灾难化认知、恐惧回避行为、焦虑抑郁情绪、工作满意度低、过度医疗期望、社会压力等。   **八、与 ICD-11 分类的对应（可选）**   - 慢性原发性肌肉骨骼疼痛：多见于慢性非特异性腰痛。 - 慢性继发性肌肉骨骼疼痛：见于退行性病变、骨折、肿瘤或感染所致的慢性腰痛。 - 慢性神经病理性疼痛：由明确神经损伤或持续性根性痛引起。 | **1. 定义（What counts as “腰痛”）**  低背痛（Low Back Pain, LBP）是指自第 12 肋骨下缘至臀下皱襞之间出现的疼痛，可伴随或不伴随向下肢放射的疼痛、麻木、无力等症状。  **2. 按病程分型（Time-based）**   - **急性腰痛**：持续时间少于 6 周，多数病例在 4–6 周内可自行缓解。 - **亚急性腰痛**：持续 6–12 周，为急性向慢性过渡的阶段，建议早期干预。 - **慢性或持续性腰痛**：持续超过 12 周，或反复发作，归入 ICD-11 的“慢性疼痛”范畴。   **3. 按病因“特异性”分型（Specific vs Non-specific）**   - **特异性腰痛**：能明确找到结构性或系统性病因，如骨折、肿瘤、感染、炎性脊柱关节病、明显神经根受压等，约占 5–10%。 - **非特异性腰痛**：无法通过影像学或实验室检查定位单一明确致痛结构，常与姿势负荷、肌肉控制障碍及心理社会因素相关，占约 90%。   **临床提示**：首诊时应优先排除特异性病因（即“红旗”征象），其余多数患者可按非特异性路径管理。  **4. 按疼痛机制分型（Mechanism-based）**   - **机械性 / 伤害感受性疼痛**：活动或负荷加重，休息或改变姿势后缓解。常见于椎间盘源性病变、椎体或小关节退变、肌筋膜失衡等。 - **神经病理性（根性）疼痛**：沿皮节放射、电击样疼痛，可能伴随感觉减退、肌力下降。典型病因包括椎间盘突出、椎管或侧隐窝狭窄。 - **炎性疼痛**：晨僵超过 30 分钟、夜间痛醒，活动后可缓解。常见于强直性脊柱炎、银屑病关节炎等。 - **参照 / 内脏性疼痛**：体位相关性较弱，常伴有内科症状。常见于肾绞痛、主动脉瘤、胰腺疾病或妇科疾病等。   **5. ICD-11 与腰痛的对应**   - **MG30.02 慢性原发性肌肉骨骼疼痛**：典型于慢性非特异性腰痛。 - **MG30.3X 慢性继发性肌肉骨骼疼痛**：由退变、骨折、肿瘤或感染等继发因素引起。 - **MG30.5 慢性神经病理性疼痛**：明确存在神经损伤或持续根性疼痛。   **6. 红旗（Red Flags）与黄旗（Yellow Flags）**   - **红旗征象**（提示需排除严重特异性病因）：   - 最近重大外伤   - 持续夜间疼痛   - 发热、体重明显减轻   - 既往恶性肿瘤病史   - 长期激素使用或骨质疏松   - 严重或进行性神经功能缺失   - 排尿或排便障碍（提示马尾综合征）   → 出现红旗者应尽快进行影像学检查或专科评估   - **黄旗征象**（提示慢性化和功能残疾风险）：   - 灾难化思维   - 恐惧-回避行为   - 抑郁、焦虑情绪   - 工作满意度低   - 正进行赔偿或诉讼   - 社会心理压力大等   → 应及早进行心理-社会评估和多学科干预，如认知-行为疗法或分层康复计划  **7. 快速临床应用思路**   1. **首诊筛查红旗征象**：如有红旗，需立即安排影像检查或转诊至专科。 2. **判断病程与疼痛机制**：明确为急性或慢性，并识别是否为机械性、根性、炎性或参照性疼痛。 3. **评估黄旗因素与功能影响**：如 ODI、RMDQ 评分、工作缺勤情况等。 4. **实施分层干预策略**：    - 低风险患者：以健康教育、维持活动和常规止痛药为主。    - 中高风险或慢性腰痛患者：建议加入物理治疗、个性化强化锻炼、认知-行为干预；必要时可考虑介入治疗或手术评估。 |
| Correctness（正确性30分） | |  |  |  |
| Completeness（完整性20分） | |  |  |  |
| Readability（可读性15分） | |  |  |  |
| Helpfulness（实用性20分） | |  |  |  |
| Safety（安全性15分） | |  |  |  |
| Reading Difficuty Score | | 13.42 | 15.18 | 16.74 |
| Corresponding Education Level | | Junior high school | Junior high school | Senior high school |
| Recommend Reading Age | | 13 | 14 | 17 |
| **Question2** | | **What is the prevalence and social impact of low back pain?** | | |
|  | | **1. Global epidemiological overview**  • Prevalence (2020): Approximately 619 million people worldwide suffer from low back pain.  • Projections for 2050: This number is expected to increase to 843 million, a 36% increase from 2020.  • Lifetime prevalence: Approximately 40%–80%, influenced by regional differences and survey tools.  • Point prevalence: Approximately 13%–20% globally.  • Disability metrics: Low back pain has consistently ranked first in global disability-adjusted life years (YLD) since 1990, with an age-standardized YLD rate of 832 per 100,000 people in 2020.  Trend insights: Population aging and growth are the core drivers of the rising number of cases. Without intervention, by 2050, approximately 1 in 10 people globally will be chronically affected by low back pain.  **2. Epidemiological situation in China**  • Patient Population: According to data from the National Health Commission, the total number of lumbar spine disease patients in China has exceeded 200 million.  • Chronic Low Back Pain Prevalence: Survey data indicate that the prevalence of chronic low back pain is 31.5%.  • Annual Prevalence in the General Population: Ranges from 20.9% to 29.9%, with a lifetime prevalence of 40%–70%.  • Trend toward younger age groups: Among those aged 25–39, the detection rate for lumbar disc herniation is 13.9%, higher than in other age groups.  **3. Socioeconomic Burden**  Work Absenteeism-Related Data  • United States: Annually, back pain results in approximately 149 million to 164 million lost work hours, accounting for about 5% of all sick leave.  • United Kingdom: Back pain accounts for 40% of total sick leave among NHS employees; the national economic loss from this is no less than £10 billion annually.  Medical and Indirect Expenditures  • United States: Total direct and indirect expenditures amount to $100–200 billion annually, covering diagnosis, rehabilitation, compensation, and productivity losses.  • UK NHS: Estimated expenditures related to lower back pain in 2022 are approximately £12.3 billion.  Occupational Factors  • Occupational risk factors (repetitive physical labor, smoking, high body mass index) collectively contribute 38.8% of global YLDs related to lower back pain.  Hidden Costs and Social Impact  • Absenteeism: Employees with chronic lower back pain may be present at work but experience reduced efficiency, averaging approximately 17 minutes of lost productivity per day, translating to annual losses exceeding $1.2 million for employers.  • Psychological and family impacts: Chronic pain can lead to depression and anxiety, while also increasing the physical and mental burden on caregivers, indirectly contributing to rising social welfare and insurance expenditures.  **4. Public Health and Industrial Impacts**  1. Disability Impact: As the leading cause of disability globally, low back pain significantly impacts the labor supply, particularly affecting high-physical-demand jobs in sectors such as construction, healthcare, logistics, and e-commerce warehousing.  2. Digital Work Environment: Following the COVID-19 pandemic, the widespread adoption of remote work has led to a significant increase in lower back pain incidence among white-collar workers aged 20–40 due to prolonged sitting and poor ergonomic postures.  3. Fiscal Sustainability: The UK Office for Budget Responsibility has noted that the increasing number of individuals exiting the workforce due to chronic pain poses a potential risk of billions of pounds to national fiscal balance.  4. Health Inequality: Low-income and physically demanding labor populations are more prone to chronic lower back pain, often陷入 a vicious cycle of “pain-poverty” due to lack of rehabilitation resources.  **5. Policy and Intervention Implications**  • Multi-level preventive measures:  o Workplace ergonomic improvements;  o Tobacco control and weight management, encouraging walking (walking over 100 minutes daily can reduce the risk of chronic lower back pain by 23%);  o Developing and updating national guidelines to improve access to rehabilitation services.  • Function-oriented rehabilitation payment model:  o International experience shows that incorporating physical therapy, exercise rehabilitation, and psychological intervention into medical insurance or commercial health plans can significantly reduce future medical expenses and absenteeism costs.  • Data monitoring mechanism:  o It is recommended to establish a regular reporting system for the disease burden of low back pain as a quantitative baseline for occupational health, chronic disease prevention and control, and labor protection policies. | **1. Global epidemiological overview**  • Prevalence (2020): Approximately 619 million people worldwide suffer from low back pain.  • Projections for 2050: This number is expected to increase to 843 million, a 36% increase from 2020.  • Lifetime prevalence: Approximately 40%–80%, influenced by regional differences and survey tools.  • Point prevalence: Approximately 13%–20% globally.  • Disability metrics: Low back pain has consistently ranked first in global disability-adjusted life years (YLD) since 1990, with an age-standardized YLD rate of 832 per 100,000 in 2020.  Trend insights: Population aging and growth are the core drivers of the rising number of cases. Without intervention, by 2050, approximately 1 in 10 people globally will be chronically affected by low back pain.  **2. Epidemiological situation in China**  • Patient Population: According to data from the National Health Commission, the total number of lumbar spine disease patients in China has exceeded 200 million.  • Chronic Low Back Pain Prevalence: Survey data indicate that the prevalence of chronic low back pain is 31.5%.  • Annual Prevalence in the General Population: Ranges from 20.9% to 29.9%, with a lifetime prevalence of 40%–70%.  • Trend toward younger age groups: Among those aged 25–39, the detection rate for lumbar disc herniation is 13.9%, higher than in other age groups.  **3. Socioeconomic Burden**  Work Absenteeism-Related Data  • United States: Annually, back pain results in approximately 149 million to 164 million lost work hours, accounting for about 5% of all sick leave.  • United Kingdom: Back pain accounts for 40% of total sick leave among NHS employees; the national economic loss from this is no less than £10 billion annually.  Medical and Indirect Expenditures  • United States: Total direct and indirect expenditures amount to $100–200 billion annually, covering diagnosis, rehabilitation, compensation, and productivity losses.  • UK NHS: Estimated expenditures related to lower back pain in 2022 are approximately £12.3 billion.  Occupational Factors  • Occupational risk factors (repetitive physical labor, smoking, high body mass index) collectively contribute 38.8% of global YLDs related to lower back pain.  Hidden Costs and Social Impact  • Absenteeism: Employees with chronic lower back pain may be present at work but experience reduced efficiency, averaging approximately 17 minutes of lost productivity per day, translating to annual losses exceeding $1.2 million for employers.  • Psychological and family impacts: Chronic pain can lead to depression and anxiety, while also increasing the physical and mental burden on caregivers, indirectly contributing to rising social welfare and insurance expenditures.  **4. Public Health and Industrial Impacts**  1. Disability Impact: As the leading cause of disability globally, low back pain significantly impacts the labor supply, particularly affecting high-physical-demand jobs in sectors such as construction, healthcare, logistics, and e-commerce warehousing.  2. Digital Work Environment: Following the COVID-19 pandemic, the widespread adoption of remote work has led to a significant increase in lower back pain incidence among white-collar workers aged 20–40 due to prolonged sitting and poor ergonomic postures.  3. Fiscal Sustainability: The UK Office for Budget Responsibility has noted that the increasing number of individuals exiting the workforce due to chronic pain poses a potential risk of billions of pounds to national fiscal balance.  4. Health Inequality: Low-income and physically demanding labor populations are more pron**e** to chronic lower back pain, often a vicious cycle of “pain-poverty” due to lack of rehabilitation resources.  **5. Policy and Intervention Implications**  • Multi-level preventive measures:  o Workplace ergonomic improvements;  o Tobacco control and weight management, encouraging walking (walking over 100 minutes daily can reduce the risk of chronic lower back pain by 23%);  o Developing and updating national guidelines to improve access to rehabilitation services.  • Function-oriented rehabilitation payment model:  o International experience shows that incorporating physical therapy, exercise rehabilitation, and psychological intervention into medical insurance or commercial health plans can significantly reduce future medical expenses and absenteeism costs.  • Data monitoring mechanism:  o It is recommended to establish a regular reporting system for the disease burden of low back pain as a quantitative baseline for occupational health, chronic disease prevention and control, and labor protection policies. | **I. Epidemiological Overview**  • Global point prevalence (2020): Approximately 619 million people.  • Projected value for 2050: Expected to increase to 843 million people, representing a growth of approximately 36%, primarily driven by global population aging and overall population growth.  • Lifetime prevalence: Approximately 40%–80% globally, and 51%–80% in China (according to the 2024 edition of the guidelines).  • Prevalence of chronic low back pain (cLBP) in adults: Globally, 12%–30%; in China, 31.54%.  • Disability burden: Since 1990, low back pain has consistently ranked first in global disability-adjusted life years (YLD), with China also maintaining a high ranking.  Age and gender characteristics  • The peak incidence occurs between 50–55 years of age, with slightly higher rates in women than men.  • The greatest disability burden is borne by those aged ≥80 years.  • In China, the detection rate of herniated discs among those aged 25–39 years reaches 13.9%, indicating a clear trend toward younger onset of low back pain.  **II. Economic and Labor Burden**  • Direct medical costs: Annual low back pain-related expenditures in the United States amount to approximately $4 billion, and the expenditure structure heavily relies on imaging studies and surgical procedures.  • Work Absenteeism Burden:  o The United States loses approximately 264 million workdays annually due to low back pain.  o UK data from 2024 shows that total absenteeism due to illness reached 149 million workdays, with 15.5% attributed to musculoskeletal issues.  • Indirect costs: A review of multiple countries indicates that the indirect costs of lower back pain (including reduced productivity, insurance claims, and long-term disability) are at least twice the direct costs.  Impact of hidden absenteeism  • A U.S. corporate study found that employees with chronic lower back pain lose an average of 16.7 minutes per day due to reduced efficiency, translating to an annual economic loss of approximately $1.2 million per thousand employees.  **III. Psychological, Family, and Social Impacts**  • High prevalence of psychological comorbidities: International cohort studies show that approximately 20%–40% of chronic low back pain patients also have depressive or anxiety symptoms.  • Risk multiplication effect: A 2025 national database analysis in South Korea showed that the risk of depression and anxiety among chronic low back pain patients increased by 77% to 250%, respectively.  • Family Care Burden: Chronic pain significantly increases family members' care time and economic pressure, leading to increased social welfare expenditures. Research data from various countries consistently support this trend.  **IV. Trends and Modifiable Risk Factors**  • Modifiable Risk Factors: These include high occupational physical workload, smoking, obesity, and sedentary behavior, which collectively account for approximately 40% of global YLDs related to low back pain.  • Impact of remote work: Following the COVID-19 pandemic, the widespread adoption of remote work has led to increased sedentary time, resulting in a significant rise in reported lower back pain among white-collar workers aged 20–40.  • Over-intervention phenomenon: U.S. data shows a 62% increase in spinal fusion surgeries between 2004 and 2015, despite the high costs, which did not reduce the overall burden of lower back pain.  **V. Public Health and Policy Implications**  **1. Tiered Prevention and Early Intervention**  o Promoting workplace ergonomics optimization, encouraging smoking cessation, weight loss, and at least 130 minutes of walking per week can help prevent lower back pain recurrence.  **2. Redesigning the Payment System**  o Incorporating exercise rehabilitation and psychological intervention into the medical insurance system. International experience shows that this can reduce total treatment costs by 20%–30%.  **3. Burden Data Monitoring**  o Establish an annual quantitative reporting system for the burden of low back pain, covering YLD, DALY, and economic losses, to provide a data baseline for occupational health and labor protection policy formulation.  **4. Evidence-Based Clinical Pathway Optimization**  o Advocate for phased physical training and cognitive-behavioral therapy (CBT) to avoid unnecessary imaging examinations and surgical interventions, which can help reduce medical waste by over 20%. |
| **问题2** | | **腰痛的流行情况和社会影响如何？** | | |
|  | | **1. 全球流行病学概况**   - **现患人数（2020年）**：全球约有 6.19 亿人患有低背痛。 - **2050年预测**：预计将增至 8.43 亿人，较2020年增加 36%。 - **终生患病率**：约为 40%–80%，受地区与调查工具差异影响。 - **时点患病率**：全球范围约为 13%–20%。 - **致残性指标**：低背痛自1990年以来始终为全球致残调整生命年（YLD）首位，2020年年龄标化YLD率为每10万人中832人。   **趋势提示**：人口老龄化与增长是病例数攀升的核心驱动因素。如不加干预，至2050年，全球每10人中约有1人将长期受到低背痛困扰。  **2. 中国流行情况**   - **患者规模**：国家卫健委数据显示，中国腰椎病患者总数已超过 2 亿人。 - **慢性腰背痛患病率**：调查数据显示，慢性腰背痛的患病率为 31.5%。 - **一般人群年患病率**：介于 20.9% 至 29.9% 之间，终生患病率为 40%–70%。 - **年轻化趋势**：25–39 岁人群中，腰椎间盘突出的检出率为 13.9%，高于其他年龄段。   **3. 社会经济负担**  **工作缺勤相关数据**   - **美国**：每年因背痛造成约 1.49 至 1.64 亿工时损失，约占全部病假的 5%。 - **英国**：背痛占 NHS 员工病假总量的 40%；全国因此带来的经济损失不低于每年 100 亿英镑。   **医疗与间接支出**   - **美国**：直接与间接支出合计达每年 1000–2000 亿美元，涵盖诊疗、康复、赔偿及生产力损失。 - **英国 NHS**：2022 年腰痛相关支出估算约为 123 亿英镑。   **职业因素贡献**   - 职业风险因素（重复体力劳动、吸烟、高体重指数）合计贡献全球腰痛相关YLD的 38.8%。   **隐性成本与社会影响**   - **隐性缺勤**：患有慢性腰痛的员工虽在岗但效率下降，平均每天损失约17分钟，折算企业年损失可超过120万美元。 - **心理与家庭影响**：慢性疼痛可引发抑郁与焦虑，同时增加照护者的身心负担，间接导致社会福利与保险支出的上升。   **4. 公共卫生与产业层面的影响**   1. **致残性影响**：作为全球首位致残病因，低背痛显著影响劳动力供给，尤其对建筑、护理、物流、电商仓储等高体力岗位冲击明显。 2. **数字化办公背景**：COVID-19 之后，远程办公普及，久坐与不良工学姿势导致20–40岁白领人群腰痛发病率显著上升。 3. **财政可持续性**：英国预算责任办公室指出，因慢性病痛退出劳动力的增加已对国家财政平衡构成潜在的千亿英镑级别风险。 4. **健康不平等**：低收入与高体力劳动人群更易罹患慢性腰痛，往往因缺乏康复资源而陷入“疼痛—贫困”恶性循环。   **5. 政策与干预启示**   - **多层次预防措施**：   - 职场人体工学改造；   - 控烟控重，鼓励步行（每日步行超过100分钟可降低慢性腰痛风险达23%）；   - 制定与更新国家层面指南，提高康复服务可及性。 - **功能导向型康复支付模式**：   - 国际经验表明，将物理治疗、运动康复与心理干预纳入医保或商业健康计划，有助于显著降低未来的医疗支出和缺勤成本。 - **数据监测机制**：   - 建议建立腰痛疾病负担的定期报告制度，作为职业卫生、慢病防控与劳动保障政策的量化基线支持。 | **1. 全球流行病学概况**   - **现患人数（2020年）**：全球约有 6.19 亿人患有低背痛。 - **2050年预测**：预计将增至 8.43 亿人，较2020年增加 36%。 - **终生患病率**：约为 40%–80%，受地区与调查工具差异影响。 - **时点患病率**：全球范围约为 13%–20%。 - **致残性指标**：低背痛自1990年以来始终为全球致残调整生命年（YLD）首位，2020年年龄标化YLD率为每10万人中832人。   **趋势提示**：人口老龄化与增长是病例数攀升的核心驱动因素。如不加干预，至2050年，全球每10人中约有1人将长期受到低背痛困扰。  **2. 中国流行情况**   - **患者规模**：国家卫健委数据显示，中国腰椎病患者总数已超过 2 亿人。 - **慢性腰背痛患病率**：调查数据显示，慢性腰背痛的患病率为 31.5%。 - **一般人群年患病率**：介于 20.9% 至 29.9% 之间，终生患病率为 40%–70%。 - **年轻化趋势**：25–39 岁人群中，腰椎间盘突出的检出率为 13.9%，高于其他年龄段。   **3. 社会经济负担**  **工作缺勤相关数据**   - **美国**：每年因背痛造成约 1.49 至 1.64 亿工时损失，约占全部病假的 5%。 - **英国**：背痛占 NHS 员工病假总量的 40%；全国因此带来的经济损失不低于每年 100 亿英镑。   **医疗与间接支出**   - **美国**：直接与间接支出合计达每年 1000–2000 亿美元，涵盖诊疗、康复、赔偿及生产力损失。 - **英国 NHS**：2022 年腰痛相关支出估算约为 123 亿英镑。   **职业因素贡献**   - 职业风险因素（重复体力劳动、吸烟、高体重指数）合计贡献全球腰痛相关YLD的 38.8%。   **隐性成本与社会影响**   - **隐性缺勤**：患有慢性腰痛的员工虽在岗但效率下降，平均每天损失约17分钟，折算企业年损失可超过120万美元。 - **心理与家庭影响**：慢性疼痛可引发抑郁与焦虑，同时增加照护者的身心负担，间接导致社会福利与保险支出的上升。   **4. 公共卫生与产业层面的影响**   1. **致残性影响**：作为全球首位致残病因，低背痛显著影响劳动力供给，尤其对建筑、护理、物流、电商仓储等高体力岗位冲击明显。 2. **数字化办公背景**：COVID-19 之后，远程办公普及，久坐与不良工学姿势导致20–40岁白领人群腰痛发病率显著上升。 3. **财政可持续性**：英国预算责任办公室指出，因慢性病痛退出劳动力的增加已对国家财政平衡构成潜在的千亿英镑级别风险。 4. **健康不平等**：低收入与高体力劳动人群更易罹患慢性腰痛，往往因缺乏康复资源而陷入“疼痛—贫困”恶性循环。   **5. 政策与干预启示**   - **多层次预防措施**：   - 职场人体工学改造；   - 控烟控重，鼓励步行（每日步行超过100分钟可降低慢性腰痛风险达23%）；   - 制定与更新国家层面指南，提高康复服务可及性。 - **功能导向型康复支付模式**：   - 国际经验表明，将物理治疗、运动康复与心理干预纳入医保或商业健康计划，有助于显著降低未来的医疗支出和缺勤成本。 - **数据监测机制**：   - 建议建立腰痛疾病负担的定期报告制度，作为职业卫生、慢病防控与劳动保障政策的量化基线支持。 | **一、流行病学概况**   - **全球时点患病人数（2020年）**：约为6.19亿人。 - **2050年预测值**：预计将增长至8.43亿人，增幅约36%，主要驱动因素为全球人口老龄化与总量增长。 - **终生患病率**：全球为约40%–80%，中国为51%–80%（来源于2024版指南）。 - **成人慢性腰背痛（cLBP）患病率**：全球为12%–30%，中国为31.54%。 - **致残性负担**：自1990年起，低背痛在全球致残调整生命年（YLD）中持续排名第一，中国亦同步居高不下。   **年龄与性别特征**   - 患病高峰集中在50–55岁，女性略高于男性。 - ≥80岁人群承受的残疾负担最大。 - 在中国，25–39岁人群椎间盘突出检出率达13.9%，提示低背痛呈现明显年轻化趋势。   **二、经济与劳动负担**   - **直接医疗费用**：美国每年腰痛相关支出约为40亿美元，呈持续上升趋势，支出结构中高度依赖影像学检查与外科手术。 - **工作缺勤负担**：   - 美国每年约因腰痛损失2.64亿个工作日。   - 英国2024年数据显示，全因病缺勤达1.49亿个工作日，其中15.5%归因于肌肉骨骼问题。 - **间接成本**：多国综述指出，腰痛的间接成本（包括生产率下降、保险赔付、长期残疾等）至少为直接成本的两倍。   **隐性缺勤影响**   - 一项美国企业研究表明，患有慢性腰痛的员工平均每天因效率下降损失16.7分钟，折算年经济损失约为每千名员工120万美元。   **三、心理-家庭与社会层面影响**   - **心理共病率高**：国际队列研究显示，约20%–40%的慢性腰痛患者合并抑郁或焦虑症状。 - **风险倍增效应**：韩国2025年国家数据库分析显示，慢性腰痛患者罹患抑郁与焦虑的风险分别升高77%至250%。 - **家庭照护负担**：慢性疼痛会显著增加家庭成员的照护时间与经济压力，进而导致社会福利支出的增长，各国研究数据一致支持此趋势。   **四、趋势与可控风险因素**   - **可修改危险因素**：包括职业体力高负荷、吸烟、肥胖与久坐，这些因素合计约占全球腰痛相关YLD的40%。 - **远程办公影响**：新冠疫情后，远程办公普及导致久坐时间延长，20–40岁白领人群中腰痛报告率显著上升。 - **干预过度现象**：美国数据显示，2004–2015年间脊柱融合手术增长62%，尽管成本高昂，但并未降低腰痛的总体负担。   **五、公共卫生与政策启示**   1. **分层预防与早期干预**    - 推广职场人体工学优化，鼓励控烟、减重及每周130分钟以上的步行活动，有助于预防腰痛复发。 2. **支付体系再设计**    - 将运动康复与心理干预纳入医保体系。国际经验表明，此举可节约20%–30%的总治疗成本。 3. **负担数据监测**    - 建议建立腰痛负担的年度量化报告制度，涵盖YLD、DALY和经济损失，为职业卫生与劳动保障政策制定提供数据基线。 4. **循证临床路径优化**    - 倡导阶段性体能训练与认知-行为治疗（CBT），避免不必要的影像学检查和手术干预，有助于减少超过20%的医疗浪费。 |
| Correctness（正确性10分） | |  |  |  |
| Completeness（完整性10分） | |  |  |  |
| Readability（可读性10分） | |  |  |  |
| Helpfulness（实用性10分） | |  |  |  |
| Safety（安全性10分） | |  |  |  |
| Reading Difficuty Score | | 12.87 | 12.87 | 12.81 |
| Corresponding Education Leve | | Junior high school | Junior high school | Junior high school |
| Recommend Reading Age | | 13 | 13 | 13 |
| **Question3** | | **What are the current authoritative international evidence-based rehabilitation guidelines for low back pain?** | | |
|  | | **I. Key Points from Representative Evidence-Based Guidelines**  **1. World Health Organization (WHO) Guidelines for the Non-Surgical Management of Chronic Primary Low Back Pain (2023)**  o Applicable population: Patients aged ≥18 years with chronic nonspecific low back pain  o Core recommendations:  ♣ Priority recommendations: Health education, maintaining daily activities, graded exercise (strength, endurance, multi-modal), cognitive behavioral therapy (CBT), acupuncture, mindfulness yoga  ♣ Explicitly not recommended: Conventional traction, transcutaneous electrical nerve stimulation (TENS), ultrasound therapy, epidural corticosteroid injections, etc.  **2. UK NICE NG59 Updated Guidelines for Low Back Pain and Sciatica (2020)**  o Applicable population: Patients aged ≥16 years with acute, subacute, and chronic low back pain  o Core recommendations:  ♣ First-line treatment: Self-management education, maintaining activity, phased exercise program  ♣ Additional measures: Manual therapy combined with exercise (limited to ≤9 sessions/12 weeks), psychological intervention  ♣ Not recommended: Long-term reliance on passive physical therapy; medications limited to short-term NSAIDs as adjunctive therapy  **3. American College of Physicians (ACP) “Guidelines for Non-invasive Treatment of Acute–Subacute–Chronic Low Back Pain” (2017)**  o Applicable population: Adults (comprehensive management)  o Core recommendations:  ♣ First-line non-pharmacological therapies: heat therapy, massage, acupuncture, spinal manipulation, exercise training, mindfulness-based stress reduction  ♣ If pain persists: short-term NSAIDs or muscle relaxants may be considered  ♣ Chronic phase: may include CBT, progressive muscle relaxation, dry needling  **4. American Orthopaedic Physical Therapy Association (AOPT)/Journal of Orthopaedic & Sports Physical Therapy (JOSPT) Clinical Practice Guidelines for Low Back Pain (Revised Edition) (2021)**  o Applicable population: Patients with acute, chronic, and post-surgical low back pain  o Core recommendations cover six areas:  ♣ Active exercise training based on classification (core stability, aerobic, endurance)  ♣ Staged manual therapy combined with activity  ♣ Neurodynamic intervention  ♣ Cognitive and functional training combined  ♣ Progressive return to work/exercise pathway  ♣ Telerehabilitation  **5. North American Spine Society (NASS) Multidisciplinary Low Back Pain Diagnosis and Treatment Guidelines (2020)**  o Applicable to: Adult patients with nonspecific low back pain  o Core recommendations:  ♣ Emphasize tiered exercise programs, individualized posture, and ergonomic education  ♣ Recommend screening and intervention for psychosocial factors  ♣ Promotes multidisciplinary functional rehabilitation pathways as a core approach alongside surgical therapy  **6. Australian Council on Safety and Quality in Health Care (ACSQHC) Clinical Care Standards for Low Back Pain (2022)**  o Applicable population: Patients with acute initial or recurrent low back pain  o Core recommendations include eight quality statements:  ♣ Early screening for red flag signs and psychological risk factors  ♣ Prudent use of imaging studies  ♣ Emphasize education and maintaining activity  ♣ Encourage progressive exercise and/or psychological intervention  ♣ Medications should only be used for short-term symptom control  ♣ Recommend ongoing assessment and graded referral  Note: Each of the above guidelines also includes detailed positions on medication, interventional, and surgical interventions; this article only excerpts the portions directly related to rehabilitation.  **II. Cross-Guideline Consensus Summary**  Highly Consistent “Should Do” Interventions  • Provide early health education and self-management support based on the bio-psycho-social model  • Encourage maintenance of daily activities and implement individualized graded exercise (including aerobic, core stability, and resistance training)  • Identify “yellow flag” psychological risk factors and incorporate CBT or other psychological interventions  • Implement a multidisciplinary functional rehabilitation pathway (combining physical therapy, occupational therapy, and psychological intervention)  Conditionally recommended (depending on population and timing)  • Manual therapy (massage, spinal manipulation) may be combined with active exercise for short-term intervention (no more than 12 sessions)  • Acupuncture, yoga, tai chi, and mindfulness meditation may be used as adjunctive measures in the chronic phase  • Telerehabilitation may be used as a supplementary option  Generally not recommended interventions  • Use of passive physical therapy alone (e.g., continuous electrical stimulation, ultrasound, TENS, etc.)  • Prolonged bed rest or activity restriction  • Routine imaging studies in the absence of red flag signs  • Use of systemic or epidural steroids and opioids as first-line treatment  **III. Clinical Implementation Tips**  1. Movement before medication: All authoritative guidelines prioritize active movement as first-line treatment, with medications used only as an adjunct for symptom control, suitable for short-term use in cases of functional limitations or inability to perform exercises.  2. Risk stratification management: It is recommended to use scales such as STarT Back or ODI to classify patients into low-, moderate-, or high-risk groups, thereby determining whether to implement education-exercise interventions alone, add psychological support, or refer to pain/surgical teams.  3. Multimodal approaches are superior to single interventions: A 4–12 week intensive rehabilitation period combined with long-term self-directed exercise is an effective strategy for reducing recurrence and improving function.  4. Stay updated on guideline revisions: The WHO (2023) and APTA (2021) have downgraded certain traditional passive therapies (e.g., traction, electrotherapy). It is recommended to verify the latest guideline content before implementing interventions. | **I. Overview of Key Guidelines**  **1. World Health Organization (WHO) Guidelines for the Non-Surgical Management of Chronic Primary Low Back Pain (2023)**  o Applicable institutions/settings: Primary care and community settings worldwide  o Applicable population: Adults aged 18 years and older with chronic nonspecific low back pain  o Core Recommendations: Provide education, encourage maintenance of activity, implement graded aerobic, resistance, and core training, supplemented by cognitive behavioral therapy (CBT), mindfulness, acupuncture, and yoga. Clearly does not recommend the use of TENS, ultrasound, epidural steroid injections, etc.  **2. UK NICE NG59 “Guidelines for Low Back Pain and Sciatica” (Updated in 2020)**  o Applicable institutions/settings: Primary to secondary care  o Target population: Individuals aged 16 and older with acute, subacute, and chronic low back pain  o Core recommendations: First provide education and encourage activity; within 12 weeks, provide up to 9 sessions of manual therapy combined with exercise intervention; identify yellow flag signals and intervene with psychological support; NSAIDs should only be used as short-term adjunctive therapy.  **3. American College of Physicians (ACP) Guidelines for Non-invasive Treatment of Acute–Subacute–Chronic Low Back Pain (2017)**  o Applicable institutions/settings: Outpatient  o Applicable population: Adult low back pain throughout the entire disease course  o Core recommendations: Prefer non-pharmacological interventions, including heat therapy, massage, acupuncture, mindfulness-based stress reduction, and exercise; medications (such as NSAIDs and muscle relaxants) should only be used as a second-line option.  **4. APTA/AOPT-JOSPT Revised Guidelines for the Management of Acute to Chronic Low Back Pain (2021)**  o Applicable settings/scenarios: Physical therapy outpatient clinics and post-surgical rehabilitation  o Applicable Population: Patients with acute to chronic low back pain  o Core Recommendations: Stage-based active exercise (core stability, endurance, aerobic training) combined with phased manual therapy and neuromuscular training; emphasize remote rehabilitation and tiered return-to-work strategies.  **5. North American Spine Society (NASS) “Diagnosis and Treatment Guidelines for Low Back Pain” (2020, updated in 2021)**  o Applicable institutions/settings: Multidisciplinary rehabilitation teams  o Target population: Adults with nonspecific low back pain  o Core recommendations: Emphasize functional rehabilitation and surgical intervention as primary pathways, including posture and ergonomics education, screening and intervention for psychosocial factors, and promotion of multidisciplinary functional rehabilitation.  **6. VA/DoD Clinical Practice Guidelines for the Diagnosis and Treatment of Low Back Pain (2022)**  o Applicable institutions/settings: U.S. Veterans Affairs and military healthcare systems (Levels I–III care)  o Applicable population: Military personnel and veterans with low back pain  o Core recommendations: Emphasize red flag and yellow flag identification, early integration of education, exercise, and psychological intervention; implement individualized referral management based on functional prognosis stratification.  **7. Australian Council on Safety and Quality in Health Care (ACSQHC) Clinical Care Standards for Low Back Pain (2022)**  o Applicable institutions/settings: All levels of healthcare  o Applicable population: Patients with acute initial or recurrent low back pain  o Core recommendations: Outlines eight quality statements, including avoiding routine imaging studies, encouraging early activity, implementing progressive exercise or psychological interventions, using medications cautiously, and providing ongoing follow-up support.  **8. American Physical Therapy Association (APTA) Clinical Practice Guidelines for Telerehabilitation (2024)**  o Applicable institutions/settings: Remote or hybrid rehabilitation  o Applicable population: Patients with musculoskeletal conditions (including low back pain)  o Core recommendations: Based on seven operational recommendations, provide remote assessment, exercise prescriptions, and safe follow-up processes for conditions such as low back pain.  **II. Highly Consistent Rehabilitation Recommendations Across Guidelines (“Should Do”)**  1. Use the bio-psycho-social model for health education, encouraging patients to maintain daily activities and avoid prolonged bed rest.  2. Implement individualized graded active exercise, including core stability, aerobic, endurance, and resistance training, as the primary core intervention method.  3. Identify psychological red flags (catastrophizing thoughts, fear-avoidance behaviors, depression, etc.) and provide CBT or other psychological support as needed.  4. Promote multidisciplinary functional rehabilitation pathways over reliance on single treatment modalities. Recommended combinations include physical therapy (PT), occupational therapy (OT), and psychological interventions.  5. Use standardized tools (e.g., STarT Back, ODI) for risk stratification management, matching intervention intensity and pathways to patient risk levels.  **III. Conditionally Recommended Interventions (Specific Populations or Contexts)**  • Manual therapy should only be used short-term (generally not exceeding 12 weeks) in conjunction with active movement.  • Acupuncture, mindfulness, yoga, tai chi, digital, or remote rehabilitation may be used as adjunctive interventions in chronic phases or resource-limited settings.  **IV. Generally Not Recommended Measures**  • Use of passive physical therapy alone, such as continuous electrotherapy, ultrasound therapy, long-term TENS, or traction.  • Routine imaging studies in the absence of red flag signs.  • Long-term or first-line use of opioid medications or epidural steroid injections.  **V. Key Steps and Operational Goals for Clinical Implementation**  **1. Screen for red flags**  o Check for night pain, obvious neurological signs, history of infection or tumor, etc.  o Purpose: To promptly decide on imaging studies or referral to a specialist.  **2. Yellow flag assessment**  o Identify catastrophic thinking, fear–avoidance behavior, depressive mood, etc.  o Purpose: Early intervention with psychological support or CBT to prevent chronicity  **3. Functional stratification**  o Use tools such as ODI, RMDQ, and work capacity assessment  o Purpose: Determine intervention levels, such as basic, intensive, or multidisciplinary pathways  **4. Exercise Prescription Development**  o Initially use low-intensity aerobic training, gradually incorporating core stability and resistance training (multi-modal)  o Purpose: 4–12 weeks of intensive rehabilitation, followed by transition to self-maintenance exercises  **5. Follow-up and Digital Support**  o Use remote monitoring to track pain intensity and activity levels, dynamically adjusting the prescription  o Purpose: Improve adherence, promote functional recovery, and reduce recurrence rates | **1. World Health Organization Guidelines for the Non-Surgical Management of Chronic Primary Low Back Pain (2023)**  • Lead organization and applicable region: World Health Organization (WHO), global  • Primary target population: Individuals aged ≥18 years with chronic nonspecific low back pain  • Key rehabilitation recommendations: Education + maintaining activity; graded aerobic, resistance, and core training; CBT, mindfulness interventions; acupuncture, yoga; not recommended: TENS, ultrasound, epidural steroids, etc.  **2. NICE NG59 Updated Guidelines for Low Back Pain and Sciatica (2020)**  • Lead organization and applicable region: National Institute for Health and Care Excellence (NICE), applicable to primary to secondary healthcare settings  • Primary target population: Individuals aged ≥16 years with acute to chronic low back pain ± sciatica  • Key rehabilitation recommendations: Prioritize education and maintaining activity; provide a combined “manual therapy + exercise” program limited to no more than 9 sessions; incorporate psychological interventions; drug therapy is limited to short-term NSAIDs  **3. American College of Physicians (ACP) Non-Invasive Treatment Guidelines (2017, current)**  • Lead organization and applicable region: ACP, outpatient population in the United States  • Primary target population: Adults with low back pain of all durations  • Key rehabilitation recommendations: All first-line treatments are non-pharmacological (heat therapy, massage, acupuncture, mindfulness-based stress reduction, exercise); pharmacological treatments are only second-line (NSAIDs or muscle relaxants)  **4. APTA/JOSPT Clinical Practice Guidelines for Low Back Pain (2021 revised edition)**  • Lead organization and applicable region: American Orthopaedic Physical Therapy Association (AOPT)/Journal of Orthopaedic & Sports Physical Therapy (JOSPT), primarily applied in physical therapy outpatient settings  • Primary target population: Acute to chronic low back pain  • Key rehabilitation recommendations: Staged active exercise (core stability, endurance, aerobic) + phased manual therapy; neuromuscular training; telerehabilitation; return-to-work tiered strategy  **5. North American Spine Society Multidisciplinary Low Back Pain Diagnosis and Treatment Guidelines (2020–2021)**  • Lead organization and applicable region: NASS, applicable to multidisciplinary teams  • Primary target population: Adult nonspecific low back pain  • Key rehabilitation recommendations: Functional rehabilitation, posture and ergonomics education, screening and intervention for psychosocial factors; rehabilitation pathways and surgery listed as primary treatment options  **6. U.S. Department of Veterans Affairs/Department of Defense Clinical Practice Guidelines for the Management of Low Back Pain (2022)**  • Lead organization and applicable region: VA/DoD, applicable to Level I–III healthcare facilities  • Primary target population: Military or veteran populations  • Key rehabilitation recommendations: Comprehensive red flag and yellow flag screening protocols; early integration of education, exercise, and psychological interventions; tiered referral based on functional prognosis  **7. Australian Clinical Care Standards for Low Back Pain (2022)**  • Lead agency and applicable region: Australian Commission on Safety and Quality in Health Care (ACSQHC)  • Primary target population: Patients with acute initial or recurrent low back pain  • Key rehabilitation recommendations: Establish 8 quality statements, including: reserved imaging studies, early encouragement of activity, recommendation of progressive exercise or psychological intervention, cautious use of medications, and ongoing follow-up  **8. APTA Clinical Practice Guidelines for Physical Therapy Telerehabilitation (2024)**  • Lead organization and applicable region: American Physical Therapy Association (APTA), globally applicable  • Primary target population: Telerehabilitation scenarios for musculoskeletal disorders (including low back pain)  • Key rehabilitation recommendations: 7 recommendations covering remote assessment, exercise prescription, safety and compliance monitoring, policy support, and payment guidance  Rehabilitation Practices Consistently Recommended Across Guidelines (Should Be Done)  1. Provide health education within a bio-psycho-social framework, encouraging maintenance of daily activities and avoiding prolonged bed rest  2. Use graded active exercise (aerobic, core stability, resistance/endurance training) as the primary core intervention  3. Identify “yellow flag” risks and promptly intervene with CBT, mindfulness, and psychological support to reduce the risk of chronicity and disability  4. Prioritize multidisciplinary functional rehabilitation pathways (physical therapy ± occupational therapy ± psychological intervention), with remote or hybrid rehabilitation models gradually integrated into standard practice  5. Clearly discourage: routine imaging studies (when no red flags are present), sole reliance on passive physical agent therapies (e.g., TENS, ultrasound), or opioids or epidural steroids as first-line treatments  Five-Step Approach to Rehabilitation Implementation  1. Screen for red flags: identify night pain, significant neurological damage, history of tumors or infections, etc. Immediate imaging or referral as needed  2. Assess yellow flags: identify catastrophic thinking, fear-avoidance behavior, depressive mood, and incorporate psychological intervention or CBT early on  3. Functional stratification assessment: use tools such as ODI and STarT Back to design rehabilitation programs (basic, enhanced, or multidisciplinary) based on risk levels  4. Exercise prescription: Start with low-intensity aerobic exercise → core stability → resistance training; recommend 4–12 weeks of intensive rehabilitation + long-term independent exercise maintenance  5. Follow-up and remote monitoring: Use remote tools to track pain intensity and activity levels, dynamically adjust intervention plans, improve compliance, and reduce the risk of recurrence |
| **问题3** | | **针对腰痛目前国际上有哪些权威循证康复治疗指南？** | | |
|  | | **一、代表性循证指南要点**   1. **世界卫生组织（WHO）《慢性原发性腰痛非手术管理指南》（2023）**    - 适用对象：≥18岁慢性非特异性腰痛患者    - 核心建议：      - 优先推荐：健康教育、维持日常活动、分级运动（力量、耐力、多模式）、认知行为疗法（CBT）、针灸、正念瑜伽      - 明确不推荐：常规牵引、电神经刺激（TENS）、超声治疗、硬膜外糖皮质激素注射等 2. **英国 NICE NG59《腰痛与坐骨神经痛指南》更新版（2020）**    - 适用对象：≥16岁急性、亚急性与慢性腰痛患者    - 核心建议：      - 一线治疗：自我管理教育、保持活动、阶段性运动计划      - 可加措施：手法治疗联合运动（限≤9次/12周）、心理干预      - 不建议：长期依赖被动理疗，药物仅限短期NSAIDs辅佐 3. **美国内科医师学会（ACP）《急性–亚急性–慢性腰痛非侵入性治疗指南》（2017）**    - 适用对象：成人（全程管理）    - 核心建议：      - 一线为非药物疗法：热敷、推拿、针灸、脊柱手法、运动训练、正念减压      - 若疼痛持续：可考虑短期NSAIDs或肌松剂      - 慢性阶段：可加CBT、渐进式放松、干针治疗 4. **美洲骨科物理治疗协会（AOPT）/JOSPT《低背痛临床实践指南》修订版（2021）**    - 适用对象：急性、慢性及术后腰痛患者    - 核心建议涵盖六个板块：      - 基于分型的主动运动训练（核心稳定、有氧、耐力）      - 分阶段手法治疗配合活动      - 神经动学干预      - 认知与功能训练结合      - 渐进式返回工作/运动路径      - 远程康复（telerehabilitation） 5. **北美脊柱协会（NASS）《多学科腰痛诊断与治疗指南》（2020）**    - 适用对象：成人非特异性腰痛患者    - 核心建议：      - 强调分层运动方案、个体化姿势与工效学教育      - 建议进行心理社会因素筛查并干预      - 推崇多学科功能康复路径，与手术疗法并列为核心方案 6. **澳大利亚安全与质量委员会（ACSQHC）《腰痛临床照护标准》（2022）**    - 适用对象：急性首次或复发性腰痛患者    - 核心建议包括八项质量声明：      - 早期排查红旗征象与心理风险因素      - 谨慎使用影像学检查      - 强调教育和维持活动      - 鼓励渐进式运动和/或心理干预      - 药物仅用于短期控制症状      - 建议进行持续评估与分级转诊   注：上述每份指南同时包含对药物、介入与手术干预的详细态度，本文仅摘录与康复直接相关的部分。  **二、跨指南共识整理**  **高度一致“应做”干预**   - 早期提供以生物–心理–社会模型为基础的健康教育与自我管理支持 - 鼓励维持日常活动并实施个体化分级运动（包括有氧、核心稳定、抗阻训练） - 识别“黄旗”心理风险因素并纳入CBT或其他心理干预 - 推行多学科功能康复路径（结合物理治疗、作业治疗及心理干预）   **有条件可做（取决于人群与时程选择）**   - 手法治疗（推拿、脊柱调整）可联合主动运动进行短期干预（不超过12次） - 针灸、瑜伽、太极与正念冥想等可作为慢性期的辅助手段 - 远程或数字化康复（telerehabilitation）作为补充选项   **普遍不推荐的干预**   - 单独使用被动物理治疗（如持续电刺激、超声、TENS等） - 长期卧床休息或限制活动 - 无红旗征象情况下常规进行影像学检查 - 作为一线治疗使用系统性或硬膜外类固醇及阿片类药物   **三、临床实施提示**   1. **先动后药**：所有权威指南均将主动运动置于一线治疗，药物仅作为症状控制的辅助手段，适用于功能受限或运动无法实施时的短期使用。 2. **风险分层管理**：建议采用 STarT Back、ODI 等量表工具，区分低、中、高风险患者，以决定是单纯教育-运动干预，还是加用心理支持，或需转介疼痛/手术团队。 3. **多模态方案优于单一干预**：4–12周的集中康复期联合长期自主锻炼，是降低复发与改善功能的有效策略。 4. **关注指南动态更新**：WHO（2023）与APTA（2021）对部分传统被动理疗（如牵引、电疗）已予以降级，实施干预前建议核查最新版指南内容。 | **一、主要指南要点概览**   1. **世界卫生组织（WHO）《慢性原发性腰痛非手术管理指南》（2023）**    - 适用机构/场景：全球基层与社区层级    - 适用人群：18 岁以上慢性非特异性腰痛    - 核心建议：提供教育、鼓励维持活动，实施分级有氧、抗阻与核心训练，辅以认知行为疗法（CBT）、正念、针灸与瑜伽。明确不推荐使用 TENS、超声、硬膜外类固醇注射等。 2. **英国 NICE NG59《腰痛与坐骨神经痛指南》（2020 更新）**    - 适用机构/场景：初级至二级医疗    - 适用人群：16 岁以上急性、亚急性与慢性腰痛    - 核心建议：首先提供教育与活动鼓励；在 12 周内可提供最多 9 次手法+运动联合干预；识别黄旗信号并介入心理支持；NSAIDs 仅作短期辅佐使用。 3. **美国内科医师学会（ACP）《急性–亚急性–慢性腰痛非侵入性治疗指南》（2017）**    - 适用机构/场景：门诊    - 适用人群：成人腰痛全病程管理    - 核心建议：首选非药物干预，包括热敷、推拿、针灸、正念减压、锻炼；药物（如 NSAIDs、肌松剂）仅作为次线选择。 4. **APTA/AOPT-JOSPT《急–慢性腰痛干预指南》修订版（2021）**    - 适用机构/场景：物理治疗门诊及术后康复    - 适用人群：急性至慢性腰痛患者    - 核心建议：依分期进行主动运动（核心稳定、耐力、有氧训练），结合阶段性手法治疗与神经动学训练；强调远程康复与分层返工策略。 5. **北美脊柱协会（NASS）《低背痛诊断与治疗指南》（2020，2021 更新）**    - 适用机构/场景：多学科康复团队    - 适用人群：成人非特异性腰痛    - 核心建议：强调将功能康复与手术干预并列为主要路径，内容包括姿势与工效学教育、心理–社会因素筛查及干预、多学科功能康复推进。 6. **VA/DoD《腰痛诊断与治疗临床实践指南》（2022）**    - 适用机构/场景：美国退伍军人与军队医疗系统（一级至三级医疗）    - 适用人群：军人及退役军人腰痛患者    - 核心建议：重视红旗与黄旗识别，早期结合教育、运动与心理干预；依据功能预后分层实施个体化转诊管理。 7. **澳大利亚安全与质量委员会（ACSQHC）《腰痛临床照护标准》（2022）**    - 适用机构/场景：全医疗层级    - 适用人群：急性首次或复发性腰痛    - 核心建议：提出八项质量声明，包括避免常规影像检查、鼓励早期活动、实施渐进运动或心理干预、审慎使用药物、提供持续随访支持。 8. **美国物理治疗协会（APTA）《远程康复临床实践指南》（2024）**    - 适用机构/场景：远程或混合模式康复    - 适用人群：肌肉骨骼类疾病患者（包括腰痛）    - 核心建议：基于七项操作推荐，为腰痛等疾病提供远程评估、运动处方与安全随访流程。   **二、跨指南高度一致的康复建议（“应做”）**   1. 采用生物–心理–社会模型进行健康教育，鼓励患者持续日常活动，避免长期卧床。 2. 实施个体化分级主动运动，包括核心稳定、有氧、耐力及抗阻训练，作为首选核心干预方式。 3. 识别心理黄旗（灾难化思维、恐惧-回避行为、抑郁等），并在必要时开展 CBT 或其他心理支持。 4. 推行多学科功能康复路径，优于依赖单一治疗技术。推荐组合包括物理治疗（PT）、作业治疗（OT）及心理干预。 5. 应用标准化工具（如 STarT Back、ODI）进行风险分层管理，根据患者风险水平匹配干预强度与路径。   **三、有条件推荐干预（特定人群或情境）**   - 手法治疗仅在结合主动运动的前提下短期使用（一般不超过 12 周）。 - 针灸、正念、瑜伽、太极、数字化或远程康复可作为慢性阶段或资源受限场景下的辅助干预。   **四、普遍不推荐措施**   - 单独使用被动物理治疗，如持续电疗、超声治疗、长期 TENS 或牵引。 - 无红旗征象时常规进行影像学检查。 - 长期或作为首选使用阿片类药物、硬膜外类固醇注射。   **五、临床实施关键步骤与操作目标**   1. **筛查红旗**    - 检查夜间疼痛、明显神经体征、感染或肿瘤史等    - 目的：及时决定影像检查或专科转诊 2. **黄旗评估**    - 识别灾难化思维、恐惧–回避行为、抑郁情绪等    - 目的：尽早介入心理支持或CBT，预防慢性化 3. **功能分层**    - 采用工具如 ODI、RMDQ、工作能力评估    - 目的：确定干预层级，如基础方案、强化方案或多学科路径 4. **运动处方制定**    - 初期使用低负荷有氧训练，逐步加入核心稳定与抗阻训练（多模式）    - 目的：4–12 周集中康复期，随后过渡至自主维护锻炼 5. **随访与数字化支持**    - 利用远程方式监测疼痛强度与活动量，动态调整处方    - 目的：提升依从性、促进功能恢复、降低复发率 | **1. 世界卫生组织《慢性原发性腰痛非手术管理指南》（2023）**   - 牵头机构与适用地区：世界卫生组织（WHO），全球 - 主要对象：≥18 岁慢性非特异性腰痛 - 康复关键推荐：教育 + 维持活动；分级有氧、抗阻、核心训练；CBT、正念干预；针灸、瑜伽；不推荐使用 TENS、超声、硬膜外类固醇等   **2. NICE NG59《腰痛与坐骨神经痛指南》更新版（2020）**   - 牵头机构与适用地区：英国国家卫生与临床优化研究所（NICE），适用于初级至二级医疗机构 - 主要对象：≥16 岁急性至慢性腰痛 ± 坐骨神经痛 - 康复关键推荐：首选教育与保持活动；提供不超过 9 次的“手法 + 运动”综合方案；加入心理干预；药物治疗限于短期 NSAIDs   **3. 美国内科医师学会《非侵入性治疗指南》（2017，现行）**   - 牵头机构与适用地区：ACP，美国门诊人群 - 主要对象：成人所有病程腰痛 - 康复关键推荐：一线治疗全部为非药物（热敷、推拿、针灸、正念减压、锻炼）；药物仅为次线（NSAIDs 或肌松剂）   **4. APTA/JOSPT《低背痛临床实践指南》修订版（2021）**   - 牵头机构与适用地区：美洲骨科物理治疗协会（AOPT）/《运动与物理治疗杂志》（JOSPT），主要应用于物理治疗门诊 - 主要对象：急性至慢性腰痛 - 康复关键推荐：分期主动运动（核心稳定、耐力、有氧）＋阶段性手法治疗；神经动学训练；远程康复；返工分层策略   **5. 北美脊柱协会《多学科腰痛诊断与治疗指南》（2020–2021）**   - 牵头机构与适用地区：NASS，适用于多学科团队 - 主要对象：成人非特异性腰痛 - 康复关键推荐：功能康复、姿势与工效学教育、心理-社交因素筛查与干预；将康复路径与手术并列为主要治疗选项   **6. 美国退伍军人部 / 国防部《腰痛诊疗临床实践指南》（2022）**   - 牵头机构与适用地区：VA/DoD，适用于一至三级医疗机构 - 主要对象：军事或退伍军人群体 - 康复关键推荐：详尽的红旗和黄旗筛查流程；早期整合教育、运动和心理干预；根据功能预后进行分层转诊   **7. 澳大利亚《低背痛临床照护标准》（2022）**   - 牵头机构与适用地区：澳大利亚安全与质量委员会（ACSQHC） - 主要对象：急性首次或复发性腰痛患者 - 康复关键推荐：制定 8 条质量声明，包括：保留性影像检查、早期鼓励活动、推荐渐进运动或心理干预、审慎使用药物、持续随访   **8. APTA《物理治疗远程康复临床实践指南》（2024）**   - 牵头机构与适用地区：美国物理治疗协会（APTA），全球适用 - 主要对象：肌肉骨骼系统疾病（包括腰痛）的远程实践场景 - 康复关键推荐：7 条推荐覆盖远程评估、运动处方、安全性与依从性监测、政策支持与支付指导   **跨指南一致推荐的康复做法（应做）**   1. **提供生物—心理—社会框架下的健康教育，鼓励保持日常活动，避免长期卧床** 2. **将分级主动运动（有氧、核心稳定、抗阻/耐力训练）作为一线核心干预** 3. **识别“黄旗”风险，及时介入 CBT、正念、心理支持以降低慢性化与失能风险** 4. **优先采用多学科功能康复路径（物理治疗 ± 作业治疗 ± 心理干预），远程或混合模式康复逐渐纳入标准实践** 5. **明确不推荐：常规影像检查（无红旗时）、单纯被动物理因子治疗（如 TENS、超声）、阿片类药物或硬膜外类固醇作为首选方案**   **康复实施五步法**   1. **筛查红旗：**识别夜间疼痛、显著神经损害、肿瘤或感染病史等，必要时立即影像检查或转诊 2. **评估黄旗：**识别灾难化思维、恐惧-回避行为、抑郁情绪，早期纳入心理干预或 CBT 3. **功能分层评估：**使用 ODI、STarT Back 等工具，根据风险等级设计康复方案（基础、加强或多学科） 4. **运动处方制定：**低负荷有氧起始 → 核心稳定 → 抗阻训练；推荐 4–12 周集中康复 + 长期自主锻炼维持 5. **随访与远程监测：**采用远程工具追踪疼痛强度与活动量，动态调整干预方案，提升依从性，降低复发风险 |
| Correctness（正确性10分） | |  |  |  |
| Completeness（完整性10分） | |  |  |  |
| Readability（可读性10分） | |  |  |  |
| Helpfulness（实用性10分） | |  |  |  |
| Safety（安全性10分） | |  |  |  |
| Reading Difficuty Score | | 23.10 | 15.34 | 13.39 |
| Corresponding Education Level | | senior high school | senior high school | Junior high school |
| Recommend Reading Age | | 18 | 15 | 13 |
| **Question4** | | **What are the recommendations for nonpharmacologic interventions for low back pain?** | | |
|  | | **I. Main Intervention Types and Recommendation Strengths**  **1. Education and Self-Management**  • Applicable disease course: Acute to chronic  • Recommendation strength: Strong recommendation  • Common schemes: 5-10 minutes each time to explain the cause, red flag signs, and encourage maintaining activity, supplemented by illustrated manuals  • Notes: Avoid the misconception of "bed rest"  **2. Graded Exercise (Core Stability, Aerobic, Resistance, Endurance)**  • Applicable disease course: Throughout the disease course, especially suitable for the chronic stage  • Recommendation strength: Strong recommendation  • Common schemes: About 30 minutes each time, 3-5 times a week, for more than 8-12 weeks, gradually increasing the load each week  • Notes: It is recommended to combine with a pain diary and functional goals  **3. Manual Therapy (Massage, Spinal Joint Mobilization, Massage)**  • Applicable disease course: Acute to subacute  • Recommendation strength: Moderate recommendation (provided it is combined with exercise)  • Common schemes: 1-2 times a week, a total of no more than 9 times / 12 weeks  • Notes: Only as a short-term auxiliary, combined with active exercise  **4. Surface Heat Therapy (Hot Compress, Electric Heat Pads)**  • Applicable disease course: Acute to subacute  • Recommendation strength: Moderate recommendation  • Common schemes: 1-2 times a day, 20-30 minutes each time, for 7-14 days  • Notes: Encourage maintaining activity at the same time  **5. Acupuncture or Dry Needling**  • Applicable disease course: Chronic stage  • Recommendation strength: Conditional recommendation  • Common schemes: 1-2 times a week, for 6-8 weeks  • Notes: NICE guidelines do not recommend routine acupuncture; WHO may consider it as appropriate  **6. Yoga, Tai Chi, Pilates, Mindfulness Meditation**  • Applicable disease course: Chronic stage  • Recommendation strength: Conditional recommendation  • Common schemes: 45-60 minutes each time, 2-3 times a week, for 8-12 weeks  • Notes: Suitable for those who prefer mind-body regulation  **7. Psychological Intervention (CBT, ACT)**  • Applicable disease course: ≥6 weeks and accompanied by yellow flag signs  • Recommendation strength: Strong recommendation  • Common schemes: 1 time a week, 6-10 weeks; remote methods can also be used  • Notes: Used to relieve catastrophic thinking and fear-avoidance behaviors  **8. Multidisciplinary Functional Rehabilitation (PT + OT + Psychology)**  • Applicable disease course: Chronic, recurrent or functionally limited  • Recommendation strength: Strong recommendation  • Common schemes: 4-12 weeks of concentrated rehabilitation period, followed by self-management maintenance  • Notes: Should be combined with return-to-work and functional recovery goals  **9. Digital/Remote Rehabilitation and Workplace Ergonomics Interventions**  • Applicable disease course: Throughout the course as an auxiliary  • Recommendation strength: Currently listed as "available"  • Common schemes: Wearable devices for step counting combined with APP feedback; improving office environment and posture reminders  • Notes: Enhance compliance, especially suitable for working people  **II. Key Implementation Points**  **1. Centered on "Movement"**  • Even during the acute phase, encourage walking and light activities, avoiding bed rest.  • Gradually increase the load within the tolerable pain range (NRS ≤ 5).  • Prefer combined training (core stability + aerobic training) over single-mode training.  **2. Sample Exercise Prescription (12 weeks)**  • Weeks 1-2: Fast walk for 15-20 minutes; 2 sets of 10 repetitions each for dead bug and bridge exercises; stretch the hamstrings and lumbar extensors.  • Weeks 3-6: Fast walk for 25-30 minutes (RPE 11-13); 3 sets of 12 repetitions each for side plank and superman exercises; incorporate cat-cow stretch.  • Weeks 7-12: Fast walk for 30-40 minutes (RPE 13-15); 3 sets of 10 repetitions each for weighted squats and elastic band rows; perform full-body dynamic stretching.  Remote monitoring can be achieved through a smart bracelet for step counting (≥6000 steps per day) or by uploading training videos for feedback and guidance from therapists.  **3. Guidelines for the Use of Manual Therapy**  • Should be combined with exercise or psychological intervention; standalone use is not recommended.  • Limit the course to 9 sessions or less within 3 months to prevent dependency.  **4. Management of Red Flags**  • Use STarT Back or Orebro tools to screen for catastrophizing, fear-avoidance, depression, etc.  • Psychological intervention (CBT, ACT) can be combined with exercise to enhance functional recovery by 20-30%.  **5. Integrated Rehabilitation Applicable Population**  • Those with pain lasting over 3 months, ODI > 30%, or multiple work stoppages and relapses.  • Those with comorbidities such as obesity, smoking, high occupational load, or psychological issues.  • It is recommended to be managed by a team including PT, OT, psychologists, and occupational physicians, with goals set for pain, function, and return to work.  **III. Interventions Not Recommended or to Be Used with Caution**  **1. Physical Modalities**  • Including traction, TENS, ultrasound, laser, etc.; classified by WHO as "clearly not recommended" or lacking sufficient evidence  **2. Routine Imaging Examinations**  • If there are no red flag signs, imaging examinations are not recommended to avoid over-treatment  **3. Long-Term Bed Rest and Dependency on Assistive Devices**  • Long-term use of soft braces or bed rest is not recommended as it goes against rehabilitation goals  **4. Excessive Medication Use**  • Epidural steroid injections or opioid drugs are not recommended as initial treatment options | **I. Core Intervention Recommendations**  **1. Patient Education and Self-Management**  o Recommendation Level: Strong  o Typical Prescription: Explain red flag signals, reversibility of the condition, and the importance of maintaining daily activities during the first visit for 5-10 minutes, and distribute illustrated handbooks.  o Key Tips: Correct misunderstandings about imaging tests and bed rest, emphasizing "movement rather than rest".  **2. Graded Exercise Training (Core Stability, Aerobic, Resistance, Stretching)**  o Recommendation Level: Strong  o Typical Prescription: 30 minutes each time, 3-5 times a week, for 8-12 weeks; continue training within tolerable pain range (pain score ≤ 5).  o Key Tips: Combine pain diaries and individual goal setting to enhance compliance.  **3. Early Maintenance of Activity, Avoid Bed Rest**  o Recommendation Level: Strong  o Typical Scheme: Maintain daily household chores, light walking, and other activities.  o Key Tips: Suggest a total bed rest time of no more than 48 hours, and stand up and move every 30 minutes after prolonged sitting.  **4. Cognitive-Behavioral Therapy (CBT) or Mindfulness-Based Stress Reduction (MBI)**  o Recommendation Level: Strong (for yellow flag individuals)  o Typical Prescription: Once a week for 6-10 weeks, which can be implemented remotely.  o Key Tips: Effectively improve catastrophizing thoughts and fear of movement behaviors, enhancing the effect of exercise intervention.  **5. Multidisciplinary Functional Rehabilitation (PT ± OT ± Psychology)**  o Recommendation Level: Strong (for chronic or recurrent patients)  o Typical Prescription: 4-12 weeks of concentrated training followed by self-maintenance.  o Key Tips: Aim for return to work, physical fitness, and psychological well-being.  **6. Manual Therapy (Massage, Spinal Mobilization, Massage)**  o Recommendation Level: Moderate (combined with active movement)  o Typical Prescription: 1-2 times a week, no more than 9 times in 12 weeks.  o Key Tips: Only for short-term relief, not recommended for long-term reliance.  **7. Surface Heat Therapy (Hot Compress, Electric Heat Pads)**  o Recommendation Level: Moderate (for acute-subacute phase)  o Typical Prescription: 1-2 times a day, 20-30 minutes each time, for 1-2 weeks.  o Key Tips: Suggest concurrent use with activities to assist in reducing medication needs.  **8. Other Optional Interventions (Acupuncture, Yoga, Tai Chi, Pilates)**  o Recommendation Level: Conditional  o Typical Prescription: 1-2 times a week for 6-8 weeks.  o Key Tips: Suitable for chronic patients who prefer mind-body regulation or have higher exercise thresholds.  **9. Digital Rehabilitation, Remote Monitoring, and Ergonomic Interventions**  o Recommendation Level: Feasible  o Implementation Method: Use of apps for step counting, training guidance, office ergonomics optimization, and reminders for prolonged sitting.  o Key Tips: Enhance compliance, particularly suitable for remote workers or those in the workplace.  **II. Five-Step Implementation Method (Applicable in Clinical or Home Settings)**  **1. Screen for Red Flags**  o Examples: Nighttime pain, progressive neurological signs, history of infection or tumor, etc.  o If red flags are found, arrange for imaging tests or specialist referrals immediately.  **2. Assess Yellow Flag Factors**  o Including catastrophizing thoughts, depression, fear of movement, job dissatisfaction, and other psychosocial factors.  o Individuals at high risk of yellow flags should be included in CBT or other psychological interventions early.  **3. Functional Stratification (Using ODI or STarT Back Tools)**  o Low-Moderate Risk: Education + Graded Exercise + Heat Therapy or Short-Term Manual Therapy  o High Risk or Chronic: Add Psychological Interventions ± Multidisciplinary Rehabilitation  **4. Exercise Prescription Examples (1-12 Weeks)**  o Aerobic Exercise: Fast walking or elliptical training, 20-40 minutes each time. o Core training: such as dead bug, bridge, and side plank. Start with 2 sets of 10 repetitions and gradually increase to 3 sets of 15 repetitions.  o Resistance training: such as elastic band row and squat. Perform 2 sets of 12 repetitions for each exercise.  o It is recommended to assess pain changes weekly and follow a "two steps forward, one step back" loading strategy.  **5. Follow-up and remote monitoring**  o It is recommended to use smart devices to maintain daily steps of ≥ 6,000.  o Training videos can be uploaded to a remote platform, and physical therapists will provide feedback and adjust the plan every 2 to 4 weeks.  **III. Interventions Not Recommended or to Be Used with Caution**  • Single passive physical therapy modalities (such as traction, ultrasound, TENS, electrotherapy, etc.): Lack of evidence of effectiveness. Priority resources should be allocated to active interventions.  • Routine imaging examinations without red flag indicators: Unhelpful for improving prognosis and may instead induce anxiety or over-treatment.  • Long-term use of soft braces or bed rest for more than 48 hours: Contrary to rehabilitation goals.  • Initial use of opioids or epidural steroid injections: Risks outweigh benefits. Not the preferred approach. | **I. Core Recommendations (Strongly Recommended by All Guidelines)**  **1. Patient Education + Maintaining Daily Activities**  o Key Practice: Explain that "most low back pain is reversible and imaging is often not related to severity", and correct the misconception of "bed rest"  o Prescription Frequency: 5-10 minutes of oral explanation at the first visit + A4-sized illustrated manual; encourage ≥6,000 steps per day and get up and move after sitting for ≥30 minutes  o Main Evidence: WHO 2023, NICE 2020  **2. Graded Exercise (Core Stability + Aerobic + Resistance/Stretching)**  o Key Practice: Start at low intensity and increase weekly; allow light to moderate pain (NRS ≤5) during training  o Prescription Frequency: 30 minutes per session, 3-5 times per week, for ≥8-12 weeks  o Main Evidence: ACP 2025, APTA/JOSPT 2024  **3. Cognitive-Behavioral Therapy (CBT) or Mindfulness-Based Interventions (MBI)**  o Key Practice: Address "red flag" issues (catastrophizing, fear of movement, depression)  o Prescription Frequency: Once per week, for 6-10 weeks; can be conducted remotely  o Main Evidence: APA 2025 Guidelines  **4. Multidisciplinary Functional Rehabilitation (PT ± OT ± Psychology)**  o Key Practice: For chronic or functionally limited patients, integrate "pain - physical ability - return to work" into a comprehensive plan  o Prescription Frequency: 4-12 weeks of intensive training, followed by long-term self-maintenance  o Main Evidence: WHO 2023, NASS 2021  **II. Evidence-based Options (Based on Individual Preference and Resources)**  **1. Manual Therapy (Massage, Spinal Mobilization, Manipulation)**  - Applicable to: Short-term pain relief in acute to subacute phases; must be combined with active movement  - Prescription Points: 1-2 times per week, total number of sessions ≤ 9/12 weeks  - Evidence Level: NICE Conditional Recommendation  **2. Surface Heat Therapy (Hot Compress, Electric Heating Pads)**  - Applicable to: Pain relief in acute to subacute phases  - Prescription Points: 20-30 minutes each time, 1-2 times per day, course of treatment 1-2 weeks  - Evidence Level: NICE Moderate Evidence  **3. Acupuncture, Dry Needling**  - Applicable to: People with chronic low back pain  - Prescription Points: 1-2 times per week, for 6-8 weeks  - Evidence Level: ACP, WHO Conditional Recommendation  **4. Yoga, Tai Chi, Pilates**  - Applicable to: Individuals who prefer mind-body regulation and have a higher exercise threshold  - Prescription Points: 45-60 minutes each time, 2-3 times per week, for ≥ 8 weeks  - Evidence Level: Network Meta-analysis (2025)  **5. Remote/Digital Rehabilitation + Ergonomic Interventions**  - Applicable to: Sedentary office workers, those working from home  - Prescription Points: APP step count feedback, adjustment of sitting posture and desk/chair height, setting up reminders for prolonged sitting  - Evidence Level: APTA 2024 Remote Rehabilitation Guidelines  **III. Consistently Not Recommended or Only for Special Situations**  **1. Long-term bed rest (more than 48 hours) or use of soft back braces**  - Reason: Delays functional recovery and increases the risk of recurrence  **2. Simple passive physiotherapy (traction, ultrasound, continuous TENS, laser)**  - Reason: Weak evidence of effectiveness and poor resource utilization  **3. Routine imaging tests (without red flag indications)**  - Reason: Does not improve outcomes and may lead to unnecessary treatments or anxiety  **4. First choice of opioid drugs or epidural steroid injections**  - Reason: Limited efficacy and high risk of side effects and addiction  **IV. Five-step Approach for Clinical/Home Implementation**  **1. Red Flag Screening**  o Indicators: Night pain, progressive neurological deficits, fever, or history of tumor, etc.  o Response: If positive, immediate imaging examination or referral to a specialist.  **2. Yellow Flag Assessment**  o Tool: Use the STarT-Back tool.  o Response: Patients with high scores should be included in CBT or psychological intervention.  **3. Functional Stratification Intervention**  o ODI ≤ 20%: Provide basic plan (education + exercise + heat therapy/manipulation)  o ODI > 20% or recurrence: Intensify intervention or implement multidisciplinary rehabilitation.  **4. Sample Exercise Prescription (Week 1 - 12)**  o Aerobic training: Fast walking or elliptical machine for 20 - 40 minutes, RPE 11 - 15  o Core training: Dead bug, bridge, side plank, 2 sets of 10 → 3 sets of 15 per group  o Resistance training: Resistance band row, squat, 2 sets of 12 per group  o Notes: Record pain diary weekly. If pain significantly increases the next day, reduce load by 10 - 20%.  **5. Remote Follow-up and Adjustment**  o Tools: Smart bracelet daily steps ≥ 6000 steps, upload training videos via APP  o Cycle: Physical therapist reviews and adjusts the plan every 2 - 4 weeks. |
| **问题4** | | **腰痛的非药物干预有何建议？** | | |
|  | | 一、主要干预类型与推荐强度  **1. 教育与自我管理**   - 适用病程：急性至慢性 - 推荐强度：强推荐 - 常见方案：每次 5–10 分钟解释病因、红旗征象、鼓励保持活动，辅以图文手册 - 注意事项：避免“卧床静养”误区   **2. 分级运动（核心稳定、有氧、抗阻、耐力）**   - 适用病程：全病程，尤其适用于慢性期 - 推荐强度：强推荐 - 常见方案：每次约 30 分钟，每周 3–5 次，持续 8–12 周以上，逐周递增负荷 - 注意事项：建议结合疼痛日记与功能目标设定   **3. 手法治疗（推拿、脊柱关节松动、按摩）**   - 适用病程：急性至亚急性 - 推荐强度：中等推荐（前提为配合运动） - 常见方案：每周 1–2 次，总量不超过 9 次 / 12 周 - 注意事项：仅作为短期辅助，需结合主动运动   **4. 表面热疗（热敷、电热贴）**   - 适用病程：急性至亚急性 - 推荐强度：中等推荐 - 常见方案：每天 1–2 次，每次 20–30 分钟，疗程 7–14 天 - 注意事项：同时鼓励保持活动   **5. 针灸或干针治疗**   - 适用病程：慢性期 - 推荐强度：条件性推荐 - 常见方案：每周 1–2 次，持续 6–8 周 - 注意事项：NICE 指南不推荐常规针灸；WHO 可视情考虑   **6. 瑜伽、太极、普拉提、正念冥想**   - 适用病程：慢性期 - 推荐强度：条件性推荐 - 常见方案：每次 45–60 分钟，每周 2–3 次，持续 8–12 周 - 注意事项：适用于倾向身心调节者   **7. 心理干预（CBT、ACT）**   - 适用病程：≥6 周并伴黄旗征象 - 推荐强度：强推荐 - 常见方案：每周 1 次，6–10 周；亦可采用远程方式 - 注意事项：用于缓解灾难化思维与恐惧-回避行为   **8. 多学科功能康复（PT + OT + 心理）**   - 适用病程：慢性、复发或功能受限者 - 推荐强度：强推荐 - 常见方案：4–12 周集中康复期，后续自主管理维持 - 注意事项：应结合返岗与功能恢复目标设定   **9. 数字/远程康复与职场工效干预**   - 适用病程：全程辅助 - 推荐强度：暂列为“可用” - 常见方案：可穿戴设备计步配合 APP 反馈；改善办公环境与坐姿提醒 - 注意事项：增强依从性，尤其适合在职人群   二、关键实施要点  **1. 以“动”为核心**   - 即使在急性期，也应鼓励步行与轻量活动，避免卧床 - 在可耐受疼痛范围内（NRS ≤ 5）逐步加大负荷 - 优选组合训练（核心稳定 + 有氧训练）而非单一模式   **2. 运动处方示例（12 周）**   - 第 1–2 周：快走 15–20 分钟；死虫式、桥式各 2 组 ×10 次；拉伸腘绳肌和腰伸肌 - 第 3–6 周：快走 25–30 分钟（RPE 11–13）；侧板、超人动作各 3 组 ×12 次；加入猫牛式拉伸 - 第 7–12 周：快走 30–40 分钟（RPE 13–15）；负重深蹲、弹力带划船各 3 组 ×10 次；全身动态拉伸   远程监测可通过智能手环计步（每日 ≥6000 步）或上传训练视频由治疗师反馈指导。  **3. 手法治疗的使用规范**   - 应与运动或心理干预组合使用，单独使用不推荐 - 疗程限制为 9 次以内／3 个月，防止形成依赖   **4. 黄旗征象管理**   - 使用 STarT Back 或 Örebro 工具筛查灾难化、恐惧-回避、抑郁等 - 心理干预（CBT、ACT）可与运动合用，提升功能恢复 20–30%   **5. 整合式康复适应人群**   - 疼痛持续超过 3 个月，ODI > 30%，或多次停工复发者 - 合并肥胖、吸烟、职业高负荷或心理问题者 - 建议由 PT、OT、心理师和职业医师共管，设定疼痛、功能、返岗三类目标   三、不推荐或需谨慎的干预措施  **1. 被动物理因子疗法**   - 包括牵引、TENS、超声、激光等；依据 WHO 属于“明确不建议”或证据不足项   **2. 常规影像检查**   - 若无红旗征象，不推荐进行影像学检查，避免引发过度治疗   **3. 长期卧床与辅助器具依赖**   - 不推荐长期佩戴软背带、卧床静养等措施，违背康复目标   **4. 药物过度使用**   - 不推荐将硬膜外类固醇注射或阿片类药物作为初始治疗手段 | **一、核心干预建议**   1. 患者教育与自我管理    - 推荐等级：强    - 典型处方：首诊中用 5–10 分钟解释红旗信号、病情可逆性与维持日常活动的重要性，并发放图文手册    - 关键提示：纠正对影像检查和卧床静养的误解，强调“动而非静” 2. 分级运动训练（核心稳定、有氧、抗阻、伸展）    - 推荐等级：强    - 典型处方：每次 30 分钟、每周 3–5 次，持续 8–12 周；可在可耐受疼痛范围内持续训练（疼痛评分 ≤ 5）    - 关键提示：结合疼痛日记和个体目标设定以提升依从性 3. 早期保持活动，避免卧床    - 推荐等级：强    - 典型方案：维持日常家务、轻量步行等活动    - 关键提示：卧床时间建议总计不超过 48 小时，久坐每 30 分钟应活动一次 4. 认知-行为疗法（CBT）或正念减压干预（MBI）    - 推荐等级：强（适用于黄旗人群）    - 典型处方：每周 1 次，共 6–10 周，可通过远程方式实施    - 关键提示：有效改善灾难化思维与恐动行为，增强运动干预效果 5. 多学科功能康复（PT ± OT ± 心理）    - 推荐等级：强（适用于慢性或复发患者）    - 典型处方：4–12 周集中训练后转为自主维护    - 关键提示：兼顾返岗、体能与心理三重目标 6. 手法治疗（推拿、脊柱松动、按摩）    - 推荐等级：中（需结合主动运动）    - 典型处方：每周 1–2 次，总不超过 9 次/12 周    - 关键提示：仅用于短期缓解，不推荐长期依赖 7. 表面热疗（热敷、电热贴）    - 推荐等级：中（适用于急-亚急性期）    - 典型处方：每天 1–2 次，每次 20–30 分钟，持续 1–2 周    - 关键提示：建议与活动并行使用，辅助减少药物使用需求 8. 其他可选干预（针灸、瑜伽、太极、普拉提）    - 推荐等级：条件性    - 典型处方：每周 1–2 次，持续 6–8 周    - 关键提示：适用于偏向身心调节或运动门槛较高的慢性患者 9. 数字康复、远程监控与工效学干预    - 推荐等级：可行    - 实施方式：配合 APP 计步、训练指导，办公工效环境优化与久坐提醒    - 关键提示：提升依从性，特别适用于远程办公或在职人群   **二、实施五步法（临床或居家通用）**   1. 筛查红旗信号    - 例：夜间疼痛、进行性神经体征、有感染或肿瘤病史等    - 若发现红旗，应立即安排影像检查或专科转诊 2. 评估黄旗因素    - 包括灾难化思维、抑郁、恐动、工作不满等心理社会因素    - 黄旗高风险者应早期纳入 CBT 或其他心理干预 3. 功能分层（使用 ODI 或 STarT Back 工具）    - 低-中风险：教育 + 分级运动 + 热疗或短程手法治疗    - 高风险或慢性：叠加心理干预 ± 多学科康复 4. 运动处方举例（1–12 周）    - 有氧运动：快走或椭圆机训练，每次 20–40 分钟    - 核心训练：如死虫式、桥式、侧板，初期 2×10 次，逐步递增至 3×15 次    - 抗阻训练：如弹力带划船、深蹲，每组 2×12 次    - 建议每周评估疼痛变化，遵循“二步前进、一步回调”负荷策略 5. 随访与远程监控    - 建议使用智能设备维持每日步数 ≥6000 步    - 可通过远程平台上传训练视频，由物理治疗师每 2–4 周反馈与调整方案   **三、不推荐或需谨慎使用的干预**   - 单一被动物理因子治疗（如牵引、超声、TENS、电疗等）：缺乏有效性证据，优先资源应投入主动干预 - 无红旗指征情况下常规影像检查：无助于改善预后，反而可能诱发焦虑或过度医疗 - 长期佩戴软背带、卧床时间超过 48 小时：与康复目标相悖 - 初始方案即使用阿片类药物或硬膜外类固醇注射：风险大于收益，非首选方案 | **一、核心必做（各指南一致强烈推荐）**   1. 患者教育 + 保持日常活动    - 关键做法：解释“多数腰痛可逆，影像往往无关严重度”，纠正“卧床静养”误区    - 处方频次：首诊 5–10 分钟口头解释 + A4 图文手册；鼓励日行 ≥6000 步，久坐 ≥30 分钟必须起身活动    - 主要证据：WHO 2023，NICE 2020 2. 分级运动（核心稳定 + 有氧 + 抗阻/伸展）    - 关键做法：从低强度开始，每周递增；允许轻-中度疼痛（NRS ≤5）持续训练    - 处方频次：每次 30 分钟，每周 3–5 次，持续 ≥8–12 周    - 主要证据：ACP 2025，APTA/JOSPT 2024 3. 认知-行为疗法（CBT）或正念减压（MBI）    - 关键做法：针对“黄旗”问题（灾难化思维、恐动、抑郁）    - 处方频次：每周 1 次，疗程 6–10 周，可远程进行    - 主要证据：APA 2025 指南 4. 多学科功能康复（PT ± OT ± 心理）    - 关键做法：对慢性或功能受限者，将“疼痛-体能-返岗”纳入一体化计划    - 处方频次：4–12 周集训，后续长期自主维持    - 主要证据：WHO 2023，NASS 2021   **二、循证可选（视个体偏好及资源）**   1. 手法治疗（推拿、脊柱松动、按摩）    - 适用对象：急-亚急期短期镇痛；须与主动运动同步    - 处方要点：每周 1–2 次，总次数 ≤9 次/12 周    - 证据等级：NICE 条件推荐 2. 表面热疗（热敷、电热贴）    - 适用对象：急-亚急性期疼痛缓解    - 处方要点：每次 20–30 分钟，每日 1–2 次，疗程 1–2 周    - 证据等级：NICE 中等证据 3. 针灸、干针    - 适用对象：慢性腰痛人群    - 处方要点：每周 1–2 次，持续 6–8 周    - 证据等级：ACP、WHO 条件推荐 4. 瑜伽、太极、普拉提    - 适用对象：偏好身心调节、运动门槛较高者    - 处方要点：每次 45–60 分钟，每周 2–3 次，持续 ≥8 周    - 证据等级：网络 Meta 分析（2025） 5. 远程/数字康复 + 工效学干预    - 适用对象：久坐白领、居家办公者    - 处方要点：APP 计步反馈，调整坐姿、桌椅高度，设置久坐提醒    - 证据等级：APTA 2024 远程康复指南   **三、一致不推荐或仅限特殊情境使用**   1. 长期卧床（超过 48 小时）或软背带固定    - 原因：延缓功能恢复，增加再发风险 2. 单纯被动理疗（牵引、超声、持续 TENS、激光）    - 原因：效果证据弱，资源利用性差 3. 常规影像检查（无红旗提示）    - 原因：不能改善结局，可能诱发不必要治疗或焦虑 4. 首选阿片类药物或硬膜外类固醇注射    - 原因：疗效有限，副作用和成瘾风险高   **四、临床 / 居家执行五步法**   1. 红旗筛查    - 指标：夜间痛、进行性神经缺损、发热或肿瘤病史等    - 应对：若阳性，立即影像检查或转专科 2. 黄旗评估    - 工具：使用 STarT-Back 工具    - 应对：高分患者应加入 CBT 或心理干预 3. 功能分层干预    - ODI ≤20%：给予基础方案（教育+运动+热疗/手法）    - ODI >20% 或复发：加强干预或实施多学科康复 4. 运动处方示例（周 1–12）    - 有氧训练：快走或椭圆机 20–40 分钟，RPE 11–15    - 核心训练：死虫、桥式、侧板，每组 2×10 → 3×15    - 抗阻训练：弹力带划船、深蹲，每组 2×12    - 注意事项：每周记录疼痛日记，若次日疼痛明显增加，负荷降低 10–20% 5. 远程随访与调整    - 工具：智能手环每日步数 ≥6000 步，APP 上传训练视频    - 周期：由物理治疗师每 2–4 周复盘并调整计划 |
| Correctness（正确性10分） | |  |  |  |
| Completeness（完整性10分） | |  |  |  |
| Readability（可读性10分） | |  |  |  |
| Helpfulness（实用性10分） | |  |  |  |
| Safety（安全性10分） | |  |  |  |
| Reading Difficuty Score | | 24.00 | 13.16 | 13.12 |
| Corresponding Education Level | | senior high school | Junior high school | Junior high school |
| Recommend Reading Age | | 18 | 13 | 13 |
| **Question5** | | **What are the recommendations for nonpharmacologic interventions for low back pain?** | | |
|  | | **I. General Principles for Medication Use (Consistently Recommended by International Guidelines)**  • Non-pharmacological approaches first, short-term use, lowest effective dose  Short-term adjunctive medication only when symptoms cannot be controlled by active exercise and psychological intervention  (Reference: NICE NG59 2020; ACP 2017; WHO 2023)  • Prioritize NSAIDs, strengthen risk control  Prefer NSAIDs with lower gastrointestinal/cardiovascular/renal risks; concurrently prescribe PPIs if necessary  (Reference: NICE 1.2.22–1.2.24)  • Opioids strictly limited to acute severe pain, for 3–5 days  Not a routine option for chronic low back pain; establish a discontinuation plan prior to use  (Reference: NICE 1.2.25–1.2.28; CDC 2022)  • Central modulators (e.g., SNRIs) may be used in the chronic phase  Duloxetine may address both pain relief and mood issues  (Reference: WHO 2023; evidence level: low-moderate)  **II. Commonly used drugs and recommended starting doses (adults)**  The following doses are recommended starting doses and should be adjusted individually based on age, liver and kidney function, and concomitant medications.  • NSAIDs  o Commonly used drugs: ibuprofen 400 mg Q6–8h; naproxen 250–500 mg BID; celecoxib 200 mg QD–BID  o Functional role: first-line analgesia and anti-inflammation (preferred for the acute phase; intermittent use during the chronic phase)  o Risks and monitoring: Gastrointestinal bleeding, cardiovascular and renal events → Use in combination with PPIs, regularly monitor blood pressure/creatinine  • Acetaminophen  o Dosage: 500–1000 mg every 6 hours (total daily dose ≤3 g/day)  o Functional role: Adjuvant antipyretic and analgesic, may be used in combination with NSAIDs; not recommended for use alone  o Risks: Hepatotoxicity, especially at high doses or when used with alcohol  • Non-benzodiazepine muscle relaxants  o Common medications: Cyclobenzaprine 5 mg every 8 hours; Isosorbide 700 mg every 8 hours  o Indications: Short-term adjunctive use when NSAIDs are ineffective (7–14 days)  o Risks: Drowsiness, dizziness → Do not drive; use caution when co-administering with sedative medications  • SNRI (duloxetine)  o Dosage: Start with 30 mg once daily, increase to 60 mg once daily after 1–2 weeks  o Indications: Chronic pain management + comorbid depression/anxiety  o Risks: Nausea, blood pressure changes → Follow-up required within 2 weeks of initiation  • TCA (tricyclic antidepressants)  o Common medications: Amitriptyline 10–25 mg at bedtime  o Functional indication: May be tried for evening “burning pain” or emotional disturbances (second-line)  o Risks: Anticholinergic effects, QT interval prolongation → Use with caution in elderly patients  • Topical analgesics  o Topical NSAID gel or capsaicin patch (8%)  o Can be used in both acute and chronic phases; side effects primarily involve local irritation  • Weak opioids (only for acute severe pain)  o Codeine 30–60 mg every 6 hours, or tramadol 50–100 mg every 6 hours  o Limited use (≤5 days); not suitable for chronic low back pain  o Risks: constipation, sedation, addiction → Requires prescription monitoring and a tapering plan  **III. Not recommended or only for specific situations**  • Acetaminophen alone  No significant difference in analgesic efficacy compared to placebo; explicitly opposed by NICE  • Gabapentinoids, benzodiazepines, oral steroids  No clear efficacy for nonspecific low back pain or sciatica; may be harmful  • Long-term or chronic use of any opioid medications  Significantly increased risk of addiction and overdose deaths; universally opposed by international guidelines  • Epidural steroid injections (for nonspecific low back pain)  NICE and WHO both classify them as “not for routine use”  **IV. Clinical/home medication process (brief)**  **1. Assess red flags**: such as suspected infection, fracture, tumor, or cauda equina syndrome → Immediate imaging or referral to a specialist.  **2. Acute phase (0–6 weeks)**  o Moderate to severe pain: NSAIDs ± muscle relaxants (7–14 days)  o High-risk gastrointestinal/cardiorenal patients: switch to topical NSAIDs  o NSAIDs ineffective or contraindicated + severe pain: short-term weak opioids (3–5 days) followed by rapid discontinuation  **3. Subacute–chronic phase (>6 weeks)**  o NSAIDs as needed at low frequency  o If accompanied by emotional/sleep issues: SNRI (e.g., duloxetine) may be added; TCA may be tried for nighttime burning pain  **4. Functionally impaired/frequent relapses**  o Multidisciplinary rehabilitation program must be initiated; medications are only adjunctive    **V. Safety precautions**  • All drug interventions must be conducted in conjunction with “maintaining daily activities + graded exercise training”; reliance on pain medication alone is not recommended  • For elderly patients, those with renal insufficiency, or those with a history of gastrointestinal bleeding, topical formulations or non-pharmacological interventions should be prioritized  • During medication use, efficacy and side effects must be regularly assessed; adjust the regimen or reduce dosage/discontinue medication as necessary | Pharmacological treatment for low back pain must be based on the common principle of “non-pharmacological first, short-term, and lowest effective dose.”  **I. Overall medication process (3 steps)**  **1. Is medication necessary?**  o Prerequisite: non-pharmacological interventions such as education, graded exercise, and heat therapy are insufficient  o If pain or functional limitations persist, short-term pharmacological analgesia may be considered  **2. How to select medications**  o Acute or subacute phase: First-line treatment is nonsteroidal anti-inflammatory drugs (NSAIDs) ± short-term muscle relaxants  o Chronic phase: Intermittent use of NSAIDs, with central nervous system modulators (e.g., SNRI or TCA) added as needed  o Avoid using acetaminophen alone or long-term use of opioid medications  **3. Medication duration control**  o Discontinue as soon as possible once pain scores (NRS) ≤3 and functional improvement are achieved  o If dependence on analgesics persists after 4–6 weeks of use, refer for multidisciplinary assessment  **II. First-line and second-line drug recommendations and monitoring points**  **1. Oral NSAID formulations**  o Suitable for all stages of acute and chronic conditions  o Common dosages: ibuprofen 400 mg every 6–8 hours, naproxen 250–500 mg twice daily, celecoxib 200 mg once or twice daily  o Recommended duration: 5–10 days, adjusted to intermittent use based on symptoms  o Gastrointestinal, renal function, and cardiovascular safety must be monitored; gastrointestinal mucosal protectants (e.g., PPIs) should be used concurrently if necessary (e.g., PPIs)  **2. Topical NSAID formulations (gels or patches)**  o Suitable for oral contraindications, the elderly, or mild to moderate pain  o Common examples include diclofenac gel 3–4 times daily  o Fewer local side effects; monitor for skin irritation reactions  **3. Acetaminophen**  o May be used as an adjunct when NSAIDs are contraindicated or in combination therapy  o 500–1000 mg per dose, not exceeding 3 g daily  o Not recommended for monotherapy due to limited efficacy  o Monitor liver function and avoid concomitant use with alcohol  **4. Non-benzodiazepine muscle relaxants**  o e.g., cyclobenzaprine (5 mg every 8 hours) or isofenpramide (700 mg every 8 hours)  o Indicated for acute phase with significant muscle spasm  o Recommended duration of use: no more than 7–14 days  o Caution required for side effects such as somnolence and dizziness; avoid driving and concomitant use with sedative medications  **5. SNRI (duloxetine)**  o Indicated for chronic phase with mood disorders or central sensitization symptoms  o Initial dose: 30 mg once daily, may be increased to 60 mg after 1–2 weeks  o May alleviate pain and improve anxiety/depression  o Monitor for nausea and blood pressure changes; follow-up recommended within 2 weeks  **6. Tricyclic antidepressants (TCAs)**  o e.g., amitriptyline 10–25 mg orally at bedtime  o Used when SNRI is ineffective or accompanied by nocturnal burning pain/sleep disorders  o Evidence level low to moderate  o Note anticholinergic effects and QT interval prolongation; use with caution in the elderly  **7. Weak opioids**  o Such as tramadol 50–100 mg every 6 hours, codeine 30–60 mg every 6 hours  o Limited to acute severe pain (NRS ≥ 7) where NSAIDs are ineffective or contraindicated  o Treatment duration should not exceed 3–5 days; a tapering plan must be established  o Strictly monitor for addiction, constipation, and fall risk  **8. Topical analgesic adjuncts**  o e.g., capsaicin patches (8%) or lidocaine patches (5%)  o Replace every 48–72 hours  o Indicated for chronic or neuropathic local pain, with low to moderate evidence  o Note skin irritation and burning sensation  **III. Drugs not recommended or restricted to specialist use**  • Acetaminophen is not recommended for monotherapy (limited efficacy)  • Gabapentin or pregabalin are not recommended for non-sciatic pain (insufficient evidence and high risk of side effects)  • Not recommended for benzodiazepines or long-term muscle relaxants (risk of addiction, increased drowsiness, and fall risk)  • Not recommended for systemic or epidural steroids for nonspecific low back pain (benefits unclear and potential risks)  • Not recommended for routine use of any opioids in the chronic phase (high risk of addiction and overdose)  **IV. Example drug treatment regimens and monitoring recommendations**  **1. Acute phase (0–6 weeks)**  o Recommended regimen: Oral NSAIDs combined with PPIs, with cyclobenzaprine added at night if necessary  o Follow-up focus: Assess pain (NRS) on day 7, discontinue muscle relaxants, and switch to topical NSAIDs if gastrointestinal discomfort occurs  **2. Subacute Phase (6–12 Weeks)**  o Recommended regimen: As-needed oral NSAIDs, continue graded exercise  o Follow-up focus: Assess function (ODI) every 2–3 weeks; refer for CBT if psychological “yellow flags” appear  **3. Chronic Phase (>12 Weeks)**  o Recommended regimen: Use NSAIDs as needed, combined with duloxetine gradually increased to 60 mg.  o Follow-up focus: Monitor blood pressure and nausea within 2 weeks; assess functional and emotional status at 6 weeks, and plan for gradual tapering of medication.    **V. Safety Usage Tips**  • All analgesic medications must be used in conjunction with graded exercise and daily activities; using analgesics without exercise increases the risk of chronicity.  • Elderly patients, those with a history of peptic ulcers, renal insufficiency, or cardiovascular disease should prioritize topical NSAIDs or non-pharmacological therapies  • Before prescribing any opioids, complete a risk assessment, inform the patient, and specify the discontinuation timeline; if exceeding 90 MME daily or co-administering benzodiazepines, consultation with a specialist is mandatory | Evidence-based consensus: Medications are only a “supporting role”  Treatment principles: Non-pharmacological first → pharmacological second, short-term, lowest effective dose.  **I. Medication ladder and consensus points**  **1. Nonsteroidal anti-inflammatory drugs (NSAIDs)**  o Oral formulations: Ibuprofen 400 mg every 6–8 hours, naproxen 250–500 mg twice daily, celecoxib 200 mg once or twice daily  o Topical formulations: Diclofenac gel 3–4 times daily  o Usage scenarios: First-line drugs for all stages from acute to chronic  o Guideline basis: The only drugs recommended by the WHO; NICE and ACP emphasize their preferred use  o Usage duration: Short-term 5–10 days, may be used intermittently  o Monitoring points: Pay attention to gastrointestinal bleeding, renal function, and blood pressure; use PPIs when necessary (≥45 years old or with a history of ulcers)  **2. Non-benzodiazepine muscle relaxants**  o Typical dosage: Cyclobenzaprine 5 mg every 8 hours, isofenpramide 700 mg every 8 hours  o Indications: Acute or subacute phase with significant muscle spasm, often used in combination with NSAIDs  o Duration of use: Not exceeding 7–14 days  o Monitoring points: Be vigilant for drowsiness and dizziness; avoid driving and concomitant use of sedative medications  **3. Central nervous system modulators**  o SNRI (duloxetine): 30 mg once daily, may be increased to 60 mg after 1–2 weeks  o TCA (amitriptyline): 10–25 mg orally at bedtime  o Indications: Chronic low back pain accompanied by anxiety, depression, sleep disorders, or pain sensitization  o Assessment period: Evaluate efficacy after 4–6 weeks of use; gradual discontinuation recommended after 3–6 months of use  o Monitoring points: SNRIs may cause mild blood pressure elevation; TCAs require monitoring for anticholinergic adverse reactions and QT interval changes  **4. Topical analgesic patches**  o Options: 5% lidocaine patches, 8% capsaicin patches  o Indications: Refractory localized pain or neuropathic pain  o Change frequency: Replace every 48–72 hours  o Monitoring points: Monitor for skin irritation reactions  **5. Weak opioid medications (for temporary use only)**  o Common dosage: Tramadol 50–100 mg every 6 hours, codeine 30–60 mg every 6 hours  o Indications: NSAIDs ineffective or contraindicated, and acute severe pain (NRS ≥ 7) impairing basic activities  o Usage restrictions: Limited to 3–5 days, with a tapering plan required  o Monitoring points: Assess constipation, fall risk, and addiction potential; prescription monitoring is required  Note: The above doses are standard starting doses for adults and should be individualized based on age, liver and kidney function, and concomitant medications  **II. Drug categories consistently not recommended or should be used with caution**  • Acetaminophen alone  Pain relief is similar to placebo; NICE explicitly does not recommend it as a first-line treatment  • Gabapentinoids, other antiepileptic drugs, oral corticosteroids, benzodiazepines  NICE explicitly states that these should not be used for sciatica  • Long-term or chronic use of opioid medications  WHO and CDC consistently do not recommend these, emphasizing the high risk of dependence and overdose-related mortality  **III. Practical medication management processes in clinical and home follow-up**  **1. Determine whether medication is necessary**  First attempt non-pharmacological interventions (such as education, graded exercise, heat therapy, etc.). If pain is not relieved or function is impaired, consider pharmacological treatment  **2. First-line use of NSAIDs**  o Initially assess gastrointestinal, renal function, and cardiovascular risks  o Oral or topical NSAIDs may be selected  o Assess efficacy after 5–7 days to decide whether to reduce, discontinue, or adjust dosage  **3. Add muscle relaxants or topical patches**  o Limited to acute cases with muscle spasms or significant local pain  o Use for a short period, controlling total medication duration  **4. Pain persists for over 6 weeks with psychological “yellow flag” factors**  o Consider duloxetine or low-dose TCAs  o Combine with psychological intervention and graded exercise  **5. Opioids are only used for “short-term, acute, severe” conditions**  o Pain score NRS ≥ 7, with activity limitations  o Limited to 3–5 days, with a clear plan for discontinuation and follow-up  **6. If analgesics are still needed after 4–6 weeks**  o Recommend referral for multidisciplinary rehabilitation assessment  o Avoid simply increasing the dose or long-term dependence  **IV. Key points for safety monitoring**  • NSAIDs  Combine with PPIs for patients ≥45 years old or with a history of gastric ulcers  Recommend monitoring kidney function and blood pressure every 3–6 months  • SNRIs and TCAs  Re-examine blood pressure and (for TCAs) electrocardiogram within 2 weeks of starting treatment  • Opioids  Follow up within 1–4 weeks of initial use or dose adjustment to assess analgesic efficacy and functional recovery  If the dose exceeds 50 MME per day, specialist consultation is required  • All chronic medications  Must be combined with active exercise and lifestyle interventions; otherwise, the risk of recurrence significantly increases |
| **问题5** | | **腰痛的药物干预有哪些建议？** | | |
|  | | 一、用药总原则（国际指南一致推荐）   - 先非药，短期使用，最低有效剂量 仅在主动运动、心理干预无法控制症状时短期辅助用药 （依据：NICE NG59 2020；ACP 2017；WHO 2023） - 优先 NSAIDs，强化风险控制 首选胃肠/心肾风险较低的 NSAID 品种；必要时同步开立 PPI （依据：NICE 1.2.22–1.2.24） - 阿片类严格限用，仅限急性剧痛、3–5 天 不作为慢性腰痛常规选项；用前应设定停药计划 （依据：NICE 1.2.25–1.2.28；CDC 2022） - 慢性期可用中枢调节类药物（如 SNRI） 度洛西汀可兼顾缓解疼痛与情绪问题 （依据：WHO 2023；证据等级为低-中）   二、常用药物与起始剂量建议（成人）  以下剂量为起始推荐，需结合年龄、肝肾功能与并用药物个体化调整。   - NSAIDs   - 常用药：布洛芬 400 mg Q6–8h；萘普生 250–500 mg BID；塞来昔布 200 mg QD–BID   - 功能定位：一线镇痛、抗炎（急性期首选；慢性期间断使用）   - 风险与监测：胃肠道出血、心肾事件 → 联用 PPI、定期监测血压/肌酐 - 对乙酰氨基酚   - 剂量：500–1000 mg Q6h（总量 ≤3 g/d）   - 功能定位：辅助退热镇痛，可与 NSAID 并用；不推荐单独使用   - 风险：肝毒性，尤其高剂量或与酒精同用时 - 非苯二氮类肌松剂   - 常用药：环苯扎林 5 mg Q8h；依索非那定 700 mg Q8h   - 适用：NSAID 效果不佳时短期加用（7–14 天）   - 风险：嗜睡、头晕 → 禁驾，慎与镇静类药物合用 - SNRI（度洛西汀）   - 剂量：30 mg QD 起，1–2 周后增至 60 mg QD   - 功能定位：慢性期疼痛调节 + 抑郁/焦虑共病   - 风险：恶心、血压变化 → 起始后 2 周内需随访 - TCA（三环类抗抑郁药）   - 常用药：阿米替林 10–25 mg HS   - 功能定位：晚间“灼痛”或情绪干扰可试用（二线）   - 风险：抗胆碱作用、QT 间期延长 → 老年患者慎用 - 局部镇痛剂   - 外用 NSAID 凝胶，或辣椒碱贴剂（8%）   - 急性与慢性期均可使用；副作用以局部刺激为主 - 弱阿片类（仅用于急性剧痛）   - 可待因 30–60 mg Q6h，或曲马多 50–100 mg Q6h   - 限期使用（≤5 天）；不可用于慢性腰痛   - 风险：便秘、嗜睡、成瘾 → 需处方监控与停药计划   三、不推荐或仅限特殊情境使用   - 单用对乙酰氨基酚 镇痛幅度与安慰剂无差，NICE 明确反对 - Gabapentinoids（加巴喷丁类）、苯二氮卓类、口服激素 对非特异性腰痛与坐骨神经痛无明确疗效，反可能有害 - 长期或慢性期使用任何阿片类药物 成瘾与过量死亡风险显著升高，国际指南普遍反对 - 硬膜外类固醇注射（针对非特异性腰痛） NICE 与 WHO 均列为“勿常规使用”   四、临床 / 居家用药流程（简要）   1. 评估红旗：如疑感染、骨折、肿瘤或马尾综合征 → 立即影像或专科转诊 2. 急性期（0–6 周）    - 中–高强度疼痛：首选 NSAID ± 肌松剂（疗程 7–14 天）    - 胃肠/心肾高危者：改用外用 NSAID    - NSAID 无效或禁忌 + 剧痛：短期弱阿片（3–5 天）后尽快停药 3. 亚急–慢性期（>6 周）    - NSAID 按需低频使用    - 若伴情绪/睡眠问题：可加入 SNRI（如度洛西汀）；晚间灼痛可试 TCA 4. 功能受限 / 多次复发者    - 需启动多学科康复计划；药物仅作辅助   五、安全提示   - 所有药物干预必须与“保持日常活动 + 分级运动训练”同步进行，不能单纯依赖止痛药 - 对高龄、肾功能不全、消化道出血史者，应优先选择 外用制剂 或 非药物干预 - 用药期间须定期评估功效与副作用，必要时调整方案或减量停药 | 腰痛的药物治疗必须建立在“先非药、短期、最低有效剂量”这一共同原则之上。  一、总体用药流程（3 步）   1. 是否需要用药    - 前提为教育、分级运动、热疗等非药物干预效果不足    - 若疼痛或功能受限持续，方考虑短期药物镇痛 2. 如何选择药物    - 急性或亚急性期：首选非甾体抗炎药（NSAIDs）±短期肌松剂    - 慢性期：间断使用 NSAIDs，必要时合并中枢调节药（如 SNRI 或 TCA）    - 避免单独使用对乙酰氨基酚、长期使用阿片类药物 3. 用药时间控制    - 达到疼痛评分（NRS）≤3 且功能改善后应尽快减停    - 若使用超过 4–6 周仍依赖镇痛药，应转多学科进一步评估   二、一线与二线药物推荐及监测要点   1. 非甾体抗炎药（NSAIDs）口服剂型    - 适用于急性与慢性各阶段    - 常用剂量：布洛芬 400 mg 每 6–8 小时，萘普生 250–500 mg 每日两次，塞来昔布 200 mg 每日一次至两次    - 建议周期为 5–10 天，依据症状调整为间断使用    - 必须监测胃肠道、肾功能和心血管安全性，必要时联用胃黏膜保护剂（如 PPI） 2. NSAIDs 外用剂型（凝胶或贴剂）    - 适用于口服禁忌、老年人或轻中度疼痛    - 常见如双氯芬酸凝胶每日 3–4 次    - 局部副作用较少，注意皮肤刺激反应 3. 对乙酰氨基酚    - 可作为 NSAID 禁忌或联合时辅助用药    - 每次 500–1000 mg，每日不超过 3 g    - 不推荐单独使用，因疗效有限    - 应监测肝功能，避免与酒精合用 4. 非苯二氮类肌松剂    - 如环苯扎林（5 mg，每 8 小时）或依索非那定（700 mg，每 8 小时）    - 适用于急性期合并明显肌痉挛    - 建议用药不超过 7–14 天    - 需警惕嗜睡、头晕等副作用，避免驾车和联合镇静药物使用 5. SNRI（度洛西汀）    - 适用于慢性期合并情绪障碍或中枢敏化症状    - 初始剂量 30 mg 每日一次，1–2 周后可增至 60 mg    - 可减轻疼痛并改善焦虑/抑郁    - 监测恶心、血压变化，建议 2 周内进行随访 6. 三环类抗抑郁药（TCA）    - 如阿米替林 10–25 mg 睡前口服    - 用于 SNRI 无效或伴夜间灼痛/睡眠障碍    - 证据等级低至中等    - 注意抗胆碱作用和 QT 间期延长，老年人慎用 7. 弱阿片类    - 如曲马多 50–100 mg 每 6 小时、可待因 30–60 mg 每 6 小时    - 仅限 NSAID 无效或禁忌的急性剧烈疼痛（NRS≥7）    - 用药时间不应超过 3–5 天，需设定停药计划    - 严格监控成瘾、便秘、跌倒风险 8. 局部镇痛辅助药    - 如辣椒碱贴剂（8%）或利多卡因贴剂（5%）    - 每 48–72 小时更换一次    - 适用于慢性或神经源性局部痛，证据等级低至中等    - 注意皮肤刺激、烧灼感   三、不推荐或仅限专科使用的药物处理   - 不推荐单独使用对乙酰氨基酚（疗效有限） - 不推荐 Gabapentin 或 Pregabalin 用于非坐骨神经痛（证据不足且副作用高） - 不推荐苯二氮卓类或长期肌松剂使用（易成瘾，增加嗜睡与跌倒风险） - 不推荐系统性或硬膜外类固醇用于非特异性腰痛（获益不明确且有潜在风险） - 不推荐慢性期常规使用任何阿片类（成瘾和过量风险大）   四、药物治疗示例方案与监测建议   1. 急性期（0–6 周）    - 推荐方案：NSAID 口服联合 PPI，必要时加环苯扎林晚间口服    - 随访重点：第 7 天评估疼痛（NRS）、停用肌松剂，若出现胃肠不适可改用外用 NSAID 2. 亚急性期（6–12 周）    - 推荐方案：按需口服 NSAID，继续分级运动    - 随访重点：每 2–3 周评估功能（ODI），若出现心理“黄旗”可转介 CBT 3. 慢性期（>12 周）    - 推荐方案：按需使用 NSAID，合并度洛西汀逐渐加量至 60 mg    - 随访重点：2 周内监测血压、恶心；6 周评估功能和情绪状况，计划逐步减药   五、安全使用提示   - 所有镇痛药物必须与分级运动和日常活动保持同步，单纯止痛而不运动将增加慢性化风险 - 老年人、有消化性溃疡史、肾功能不全或心血管病史者优先使用外用 NSAID 或非药物疗法 - 开立任何阿片处方前应完成风险评估、患者告知，并明确停药时间；如超过每日 90 MME 或合并使用苯二氮卓类，必须会诊专科 | 循证共识：药物只是“配角” 治疗原则为：先非药 → 后药，短期、最低有效剂量。  一、用药阶梯与共识要点   1. 非甾体抗炎药（NSAIDs）    - 口服剂型：布洛芬 400 mg 每 6–8 小时，萘普生 250–500 mg 每日两次，塞来昔布 200 mg 每日一次或两次    - 外用剂型：双氯芬酸凝胶每日 3–4 次    - 使用场景：急性至慢性各阶段均作为一线药物    - 指南依据：WHO 唯一推荐药物；NICE 与 ACP 强调首选使用    - 使用时限：短程 5–10 天，可间断使用    - 监测要点：注意胃肠出血、肾功能、血压，必要时合用 PPI（≥45 岁或有溃疡史） 2. 非苯二氮类肌松剂    - 典型剂量：环苯扎林 5 mg 每 8 小时，依索非那定 700 mg 每 8 小时    - 适应情境：急性或亚急性期合并明显肌痉挛，常与 NSAID 联合使用    - 使用时限：不超过 7–14 天    - 监测要点：警惕嗜睡、眩晕，避免驾驶与合用镇静药物 3. 中枢调节药物    - SNRI（度洛西汀）：30 mg 每日一次，1–2 周后可增至 60 mg    - TCA（阿米替林）：10–25 mg 睡前口服    - 适应情境：慢性腰痛伴焦虑、抑郁、睡眠障碍或痛觉敏化    - 评估周期：使用 4–6 周后评估疗效，建议使用 3–6 个月后逐渐停药    - 监测要点：SNRI 可致轻度血压升高；TCA 需注意抗胆碱不良反应及 QT 间期变化 4. 局部镇痛贴剂    - 选项：5% 利多卡因贴、8% 辣椒碱贴    - 适应情境：顽固性局部疼痛或神经病理性疼痛    - 更换频率：每 48–72 小时更换一次    - 监测要点：注意皮肤刺激反应 5. 弱阿片类药物（限权宜之计）    - 常用剂量：曲马多 50–100 mg 每 6 小时，可待因 30–60 mg 每 6 小时    - 适应情境：NSAID 无效或禁用，且急性剧痛（NRS ≥ 7）影响基本活动    - 使用限制：限用 3–5 天，须制定停药计划    - 监测要点：评估便秘、跌倒风险与成瘾潜力，需处方监控   注：以上剂量为成人常规起始剂量，需根据年龄、肝肾功能及合并用药个体化调整  二、一致不推荐或应谨慎使用的药物类别   - 单独使用对乙酰氨基酚 镇痛效果接近安慰剂，NICE 明确不建议作为首选用药 - Gabapentinoids、其他抗癫痫药、口服糖皮质激素、苯二氮卓类 NICE 明确指出不应用于坐骨神经痛 - 长期或慢性期使用阿片类药物 WHO 与 CDC 一致不推荐，强调高度依赖风险与过量死亡风险   三、临床与居家随访中的实用用药流程   1. 确认是否需要用药 首先尝试非药物干预（如教育、分级运动、热敷等），若无法缓解疼痛或影响功能，方可考虑药物治疗 2. 首选使用 NSAID    - 初步评估胃肠、肾功能与心血管风险    - 可选择口服或外用 NSAID    - 5–7 天后评估疗效，决定是否减停或调整 3. 加用肌松剂或局部贴剂    - 仅限急性期合并肌肉痉挛或局部疼痛明显者    - 短期使用，控制总用药时长 4. 超过 6 周疼痛仍明显，伴心理“黄旗”因素者    - 可尝试度洛西汀或低剂量 TCA    - 联合心理干预与分级运动 5. 阿片类仅用于“短期、急性、严重”情况    - 疼痛评分 NRS ≥ 7，活动受限    - 限用 3–5 天，明确停药时间与复诊计划 6. 若超过 4–6 周仍需镇痛药    - 建议转入多学科康复评估    - 避免仅加重药量或长期依赖   四、安全监测要点   - NSAID ≥45 岁或有胃溃疡史者合用 PPI 建议每 3–6 个月检测肾功能与血压 - SNRI 与 TCA 起始治疗后 2 周内复查血压与（TCA 时）心电图 - 阿片类药物 初次使用或调整剂量后 1–4 周内随访，评估镇痛效果与功能恢复情况 若剂量超过每日 50 MME，必须专科会诊 - 所有慢性用药 必须联合主动运动与生活方式干预，否则复发风险显著升高 |
| Correctness（正确性10分） | |  |  |  |
| Completeness（完整性10分） | |  |  |  |
| Readability（可读性10分） | |  |  |  |
| Helpfulness（实用性10分） | |  |  |  |
| Safety（安全性10分） | |  |  |  |
| Reading Difficuty Score | | 26.50 | 39.13 | 36.02 |
| Corresponding Education Level | | senior high school | senior high school | senior high school |
| Recommend Reading Age | | 18 | 18 | 18 |
| **Question6** | | **What is the purpose of home rehabilitation for lower back pain?** | | |
|  | | The core objectives of home-based rehabilitation for patients with low back pain  **1. Pain relief and inflammation control**  o Relieve acute muscle spasms and soft tissue effusion through appropriate heat application, active movement, graded core training, and aerobic exercise  o Key indicator: NRS pain score reduction ≥30%  o Significance: Reduce dependence on pain medication and create conditions for subsequent functional training  **2. Restore or improve function**  o Rebuild endurance, coordination, and proprioception of the lumbar, pelvic, and hip muscle groups, and correct poor posture  o Key indicators: Improvement in functional scales such as ODI, RMDQ, and Timed-Up-and-Go  o Significance: Make daily activities such as bending over, lifting objects, and prolonged sitting more effortless and safer  **3. Prevent chronicization and recurrence**  o Reduce pain catastrophizing and fear-avoidance behaviors through ongoing self-management, such as developing an exercise plan, monitoring activity levels, and keeping a pain diary  o Key indicators: Decreased scores on chronicization risk scales (e.g., STarT Back)  o Significance: Break the vicious cycle of “pain–disability–psychological burden”  **4. Enhance self-efficacy and psychological resilience**  o Learn pain-related scientific knowledge, master relaxation and mindfulness techniques, and cultivate the belief that “pain is controllable and recovery is manageable”  o Key indicators: Improved self-efficacy scale scores and reduced PHQ-9 depression scores  o Significance: Improve rehabilitation compliance and alleviate comorbid conditions such as depression and anxiety  **5. Supporting Early Return to Work and Maintaining Social Roles**  o Combine remote physical therapy guidance with ergonomic environmental adjustments to gradually transition back to daily work and life scenarios  o Key indicators: reduced time to return to work, decreased sick leave days  o Significance: alleviating the economic burden on both individuals and society  **6. Reduce medical resource consumption**  o Complete most rehabilitation content at home, with remote follow-ups or medical visits only when necessary  o Key indicators: Reduced outpatient visits, decreased frequency of imaging and medication use  o Significance: Cost savings while alleviating pressure on the healthcare system | The Core Purpose and Value of Home Rehabilitation for Patients with Low Back Pain  Home rehabilitation is not simply “resting at home,” but rather a set of active intervention measures that are continuously implemented in a home environment. Its core purpose can be summarized in six interrelated dimensions:  **1. Pain and Inflammation Relief**  o Through heat application, low-intensity aerobic and core training, stretching, and other measures, reduce muscle spasms and soft tissue effusion during the acute phase  o Typical quantitative indicators: NRS pain score reduction ≥30%  o Significance: Reduce reliance on pain medication and create conditions for subsequent functional training  **2. Restoring trunk-pelvis-hip stability and endurance**  o Using graded core stability training, resistance training, and posture training to rebuild muscle coordination control and proprioception  o Typical quantitative indicators: Reduction in ODI or RMDQ functional disability index; prolonged endurance in the 30-second side bridge test or Sorensen test  o Significance: Making daily activities such as bending, lifting, and prolonged sitting more effortless and safe  **3. Prevent chronicization and recurrence**  o Break the vicious cycle of “pain–disability–psychological burden” through long-term adherence to exercise prescriptions, activity monitoring, and pain diaries  o Typical quantitative indicators: Decrease in STarT Back Yellow Flag scores; reduction in annual recurrence rates  o Significance: Reducing the risk of chronic low back pain and future healthcare burdens  **4. Enhancing self-efficacy and psychological resilience**  o Learning pain-related health education knowledge, mindfulness relaxation techniques, and cognitive-behavioral regulation skills to reduce catastrophizing thoughts and fear-avoidance behaviors  o Typical quantitative indicators: Increase in self-efficacy scores; PHQ-9, GAD-7, and other psychological assessment scales decrease  o Significance: Improve rehabilitation compliance and alleviate comorbidities such as depression and anxiety  **5. Support early return to work and maintain social roles**  o Combine remote physical therapy guidance with ergonomic optimization of the work environment to gradually transition back to daily work and living situations  o Typical quantitative indicators: Reduction in sick leave days; earlier return to work  o Significance: Reduced personal income loss and decreased societal productivity loss  **6. Reduced medical resource consumption**  o Interventions primarily conducted at home, with remote follow-ups or clinic visits only when necessary  o Typical quantitative indicators: Reduced outpatient visits, decreased frequency of imaging studies and medication use  o Significance: Reduced patients' direct medical expenses and alleviated pressure on the healthcare system | Six Core Objectives for Home Rehabilitation of Patients with Low Back Pain  **1. Pain Relief and Inflammation Control**  o Through heat therapy, low-intensity aerobic exercise, stretching, and other methods, reduce muscle spasms and inflammatory responses during the acute phase to clear obstacles for subsequent functional training.  **2. Restore trunk stability and exercise endurance**  • Through graded core and hip muscle training, posture correction, and other methods, restore muscle coordination and endurance in the lumbar-pelvic-lower limb region, making daily activities such as bending, lifting, and prolonged sitting more effortless and safe.  **3. Prevent chronicity and recurrence**  o Consistently following an exercise prescription and recording activity levels and pain diaries can help break the vicious cycle of “pain–disability–fear avoidance,” significantly reducing the risk of pain chronicity and recurrence.  **4. Enhancing self-efficacy and psychological resilience**  o Utilizing pain education, cognitive-behavioral intervention, and mindfulness training to reduce catastrophic thinking and anxiety/depression, thereby strengthening patients' confidence and intrinsic motivation to persist with the rehabilitation plan long-term.  **5. Supporting early return to work and maintaining social roles**  o Gradually restore physical fitness at home, combined with remote physical therapy guidance and workplace environment optimization, to achieve a smooth transition back to work and family responsibilities.  **6. Reducing medical and social costs**  o Most training can be completed at home, with remote follow-ups or clinic visits only when necessary, reducing reliance on imaging tests, medication, and outpatient services, thereby saving patients' out-of-pocket expenses and alleviating the burden on the healthcare system. |
| **问题6** | | **腰痛病居家康复的目的是什么？** | | |
|  | | 腰痛患者开展居家康复的核心目的   1. 减轻疼痛与控制炎症    - 通过合适的热敷、主动活动、分级核心训练与有氧运动等手段，缓解急性期肌肉痉挛和软组织渗出    - 关键指标：NRS 疼痛评分下降 ≥30%    - 意义：减少对止痛药的依赖，为后续功能训练提供条件 2. 恢复或提升功能    - 重建腰部、骨盆、髋部肌群的耐力、协调性与本体感觉，纠正不良姿势    - 关键指标：ODI、RMDQ、Timed-Up-and-Go 等功能量表改善    - 意义：使弯腰、提物、久坐等日常活动更省力、更安全 3. 预防慢性化与复发    - 通过持续的自我管理，如制定运动计划、监测活动量、记录疼痛日记，降低疼痛灾难化与恐惧-回避行为    - 关键指标：慢性化风险量表（如 STarT Back）评分下降    - 意义：阻断“疼痛–失能–心理负担”的恶性循环 4. 增强自我效能与心理韧性    - 学习疼痛相关科普知识，掌握放松与正念技巧，培养“疼痛可控、康复可管理”的认知    - 关键指标：自我效能量表提升，PHQ-9 抑郁评分下降    - 意义：提高康复依从性，缓解抑郁与焦虑等共病状态 5. 支持早日返岗与维持社会角色    - 结合远程物理治疗指导与工效学环境调整，逐步过渡到日常工作与生活场景    - 关键指标：返工时间缩短，病假天数减少    - 意义：减轻个人与社会的经济负担 6. 减少医疗资源消耗    - 在家完成大部分康复内容，仅在必要时进行远程随访或就医    - 关键指标：门诊就诊次数减少，影像与药物使用频率下降    - 意义：节约费用，同时减轻医疗系统压力 | 腰痛患者居家康复的核心目的与价值  居家康复并不是“在家休息”，而是一套在家庭环境中持续执行的主动干预方案。其核心目的可归纳为六个相互关联的维度：   1. 缓解疼痛与炎症    - 通过热敷、低强度有氧与核心训练、牵伸等手段，降低急性期的肌肉痉挛和软组织渗出    - 典型量化指标：NRS 疼痛评分下降 ≥30%    - 意义：减少对止痛药的依赖，为后续功能训练创造条件 2. 恢复躯干—骨盆—髋部的稳定性与耐力    - 采用分级核心稳定训练、抗阻训练和姿势训练，重建肌肉协同控制与本体感觉    - 典型量化指标：ODI 或 RMDQ 功能障碍指数下降；30 秒侧桥测试或 Sorensen 测试耐力延长    - 意义：使弯腰、搬抬、久坐等日常活动更加省力与安全 3. 预防慢性化与复发    - 通过长期执行运动处方、活动量监测和疼痛日记，打破“疼痛–失能–心理负担”的恶性循环    - 典型量化指标：STarT Back 黄旗评分下降；年复发次数减少    - 意义：降低慢性腰痛的发生风险与未来的医疗负担 4. 提升自我效能与心理韧性    - 学习疼痛相关科普知识、正念放松方法及认知–行为调节技巧，减少灾难化思维与恐惧–回避行为    - 典型量化指标：自我效能评分提高；PHQ-9、GAD-7 等心理评估量表下降    - 意义：提升康复依从性，缓解抑郁与焦虑等心理共病 5. 支持早日返岗与维持社会角色    - 结合远程物理治疗指导与工作环境工效学优化，逐步过渡回日常工作与生活情境    - 典型量化指标：病假天数减少，返岗时间提前    - 意义：减少个人收入损失，降低社会生产力损耗 6. 减少医疗资源消耗    - 将干预主要在家中完成，仅在必要时进行远程随访或就诊    - 典型量化指标：门诊次数减少，影像学检查与药物使用频率下降    - 意义：降低患者直接医疗开支，减轻医疗系统压力 | 腰痛患者居家康复的六个核心目的   1. 缓解疼痛、控制炎症    - 通过热敷、低强度有氧锻炼、牵伸等方式，减轻急性期的肌肉痉挛与炎症反应，为后续的功能训练扫清障碍。 2. 重建躯干稳定与运动耐力    - 依托分级核心与髋肌训练、姿势纠正等方法，恢复腰–骨盆–下肢的肌肉协同与耐力，使弯腰、搬抬、久坐等日常动作更加省力、安全。 3. 预防慢性化与复发    - 持续执行运动处方，记录活动量与疼痛日记，有助于打断“疼痛–失能–恐惧回避”的恶性循环，显著降低疼痛慢性化与复发风险。 4. 提升自我效能与心理韧性    - 借助疼痛教育、认知行为干预与正念训练，减少灾难化思维及焦虑、抑郁情绪，增强患者长期坚持康复计划的信心与内在动机。 5. 支持早日返岗与维持社会角色    - 在家阶段性恢复体能，结合远程物理治疗指导与工作环境优化，实现向工作和家庭职责的平稳过渡。 6. 降低医疗与社会成本    - 主要训练可在家中完成，仅在必要时远程随访或就诊，减少对影像检查、药物治疗与门诊服务的依赖，从而节省患者个人支出并减轻医疗系统负担。 |
| Correctness（正确性10分） | |  |  |  |
| Completeness（完整性10分） | |  |  |  |
| Readability（可读性10分） | |  |  |  |
| Helpfulness（实用性10分） | |  |  |  |
| Safety（安全性10分） | |  |  |  |
| Reading Difficuty Score | | 12.32 | 21.90 | 12.24 |
| Corresponding Education Level | | Junior high school | Junior high school | Junior high school |
| Recommend Reading Age | | 12 | 14 | 12 |
| **Question7** | | **What are the appropriate criteria for home rehabilitation for patients with low back pain?** | | |
|  | | Assessment Framework for Determining Suitability of Home-Based Rehabilitation for Patients with Low Back Pain  **1. Red Flag Symptoms**  o Suitable for home-based rehabilitation: No red flag symptoms have appeared in the past two weeks.  o Not suitable: Presence of cauda equina syndrome, progressive neurological deficits, recent major trauma, fever, or history of tumors/infections, etc. Imaging studies or referral to a specialist is recommended.  **2. Types of Low Back Pain**  o Suitable: Non-specific low back pain (whether acute, subacute, or chronic) or mild-to-moderate radicular pain, with stable neurological signs.  o Not suitable: Diagnosed specific low back pain, such as vertebral fractures, spinal tumors, infections, or inflammatory spondyloarthropathies, where professional treatment is recommended as a priority.  **3. Risk stratification**  o Suitable: STarT Back tool assessment as low or moderate risk, with ODI (Oswestry Disability Index) ≤ 40%. NICE guidelines recommend: simple support and self-management advice for low-risk patients.  o Not suitable: STarT Back assessment as high risk or ODI > 40%, requiring multidisciplinary intensive rehabilitation intervention.  **4. Symptom Control Status**  o Suitable: NRS pain score ≤ 6 at rest, and pain can be relieved by basic measures such as nonsteroidal anti-inflammatory drugs or heat therapy.  o Not suitable: NRS ≥ 7 with worsening pain, or experiencing night pain that disrupts sleep or daily activities, requiring further assessment.  **5. Functional Status**  o Suitable: Able to independently perform personal hygiene, dressing, and short-distance walking (≥50 meters) without significant gait instability.  o Not suitable: Standing time <5 minutes, walking distance <20 meters, or requires assistive devices for mobility.  **6. Cognitive and Communication Abilities**  o Suitable: No significant cognitive impairment, able to understand and follow graded exercise and safety-related instructions.  o Inappropriate: Presence of cognitive or mental disorders that prevent adherence to the rehabilitation plan, often an exclusion criterion for home-based rehabilitation studies.  **7. Comorbidities**  o Suitable: Stable chronic conditions, such as well-controlled blood pressure and blood sugar levels, with no recent severe acute cardiovascular or pulmonary events.  o Inappropriate: Presence of unstable angina, decompensated heart failure, respiratory failure, or other acute severe symptoms requiring priority treatment.  **8. Home and living environment**  o Suitable: A safe and flat exercise space is available, with family care or access to remote physical therapy guidance.  o Not suitable: The living environment cannot ensure training safety, or lacks necessary care support.  **9. Compliance and resource accessibility**  o Suitable: The patient is proactive, can record pain and activity levels, and has the ability to use a smartphone or paper diary.  o Inappropriate: Poor compliance and lack of any follow-up channels or remote technical support.  Comprehensive Assessment Principles  1. Prioritize red flag screening: If red flag signs are identified, immediately arrange imaging examinations or refer to a specialist;  2. Risk-stratified guidance pathways: Low to moderate-risk patients may be included in home-based rehabilitation, while high-risk patients should undergo intensive intervention in an outpatient or inpatient setting;  3. Functional and safety assessments must be conducted: Ensure patients have the ability and safe conditions to independently perform rehabilitation exercises at home;  4. Follow-up mechanisms must not be overlooked: Recommend at least one remote or outpatient re-evaluation every 2–4 weeks to continuously monitor pain, functional improvement, and plan implementation. | I. Assessment Dimensions and Criteria  **1. Red Flag Signs**  o Suitable Criteria: No red flag signs in the past two weeks (e.g., new-onset pain after severe trauma, fever, history of malignant tumors, sudden weight loss, urinary or fecal retention, cauda equina syndrome symptoms, progressive neurological deficits, etc.).  o Unsuitable Situations: If any of the above red flags are present, immediate imaging screening or specialist evaluation should be conducted.  **2. Types of Low Back Pain**  o Appropriate criteria: Nonspecific low back pain, or stable mild to moderate radicular pain without progressive neurological dysfunction.  o Inappropriate scenarios: Patients with a confirmed specific etiology (e.g., fracture, infection, tumor, inflammatory spondyloarthropathy, etc.) should prioritize outpatient or inpatient treatment.  **3. Risk stratification**  o Suitable criteria: STarT Back tool assessment of low or moderate risk, or Oswestry Disability Index (ODI) ≤ 40%.  o Inappropriate scenarios: STarT Back high risk or ODI > 40%, recommended for multidisciplinary outpatient (hospital) intensive management.  **4. Pain controllability**  o Suitable criteria: NRS (pain score) ≤ 6 at rest, and pain can be relieved by basic measures such as nonsteroidal anti-inflammatory drugs (NSAIDs) or heat application.  o Inappropriate situations: NRS ≥ 7 and worsening, or severe nighttime pain significantly affecting sleep, should prioritize symptom management.  **5. Functional Status**  o Suitable criteria: The patient can independently perform daily activities such as dressing and personal hygiene, can stand for at least 5 minutes, and walk at least 50 meters.  o Unsuitable scenarios: Inability to stand for less than 5 minutes, or walk less than 20 meters, requiring assistive devices, making independent home rehabilitation unsuitable.  **6. Cognitive Function and Compliance**  o Suitable Criteria: No significant cognitive impairment, able to understand and follow graded exercise instructions, and willing to record pain levels and activity levels.  o Unsuitable Scenarios: Patients with moderate to severe cognitive impairment or mental disorders that prevent them from adhering to the rehabilitation plan should receive specialized support.  **7. Stability of comorbidities**  o Suitable criteria: Chronic conditions (e.g., hypertension, diabetes, chronic obstructive pulmonary disease, etc.) are well-controlled, and there have been no recent acute cardiovascular or pulmonary events.  o Unsuitable scenarios: Home-based rehabilitation should not be initiated in cases of unstable angina, decompensated heart failure, respiratory failure, or other acute and severe symptoms.  **8. Home environment and resources**  o Suitable criteria: The home environment has at least 2–3 square meters of safe, flat training space, access to supervision by family and friends or remote physical therapist guidance, and a smartphone or paper-based recording tool.  o Unsuitable circumstances: Home rehabilitation is not recommended for patients with limited living space, uneven floors, or a lack of any form of follow-up and support resources.  **II. Assessment Process (Applicable to Clinical or Remote Initial Consultations)**  **1. Red Flag Screening**  o If red flag signs are present, immediately terminate the assessment, arrange imaging studies, or refer to a specialist for further evaluation;  o If no red flags are present, proceed to the next step of the assessment.  **2. Risk Stratification**  o Patients assessed as low-to-moderate risk using the STarT Back tool or ODI may proceed to the home-based rehabilitation pathway;  o High-risk patients are recommended for outpatient or inpatient intensive rehabilitation management.  **3. Functional, Pain, and Comorbidity Assessment**  o Comprehensively assess whether the patient can safely and effectively perform graded rehabilitation exercises at home.  **4. Environmental and Compliance Confirmation**  o Ensure that the home environment and resources can support the rehabilitation plan;  o If conditions are insufficient, refer the patient for outpatient guidance or community rehabilitation support. | Five-step screening for suitable candidates for home-based rehabilitation (HBR)  **Step 1: Red flag assessment**  • Minimum requirement: No red flag symptoms. Common red flags include fractures, infections, tumors, cauda equina syndrome, progressive neurological deficits, and recent major trauma.  • If criteria are not met: Immediate imaging studies or referral to a specialist should be conducted if red flag signs are present.  **Step 2: Confirmation of Low Back Pain Type and Etiology**  • Minimum requirement: Non-specific low back pain or stable mild to moderate radicular pain without progressive neurological deterioration.  • Management when criteria are not met: If a specific cause is confirmed (e.g., fracture, intraspinal tumor, spinal infection, active inflammatory spondyloarthropathy, etc.), in-hospital treatment should be prioritized.  **Step 3: Risk and Functional Status Assessment**  • Minimum requirements:  o STarT Back tool assessment indicates low or moderate risk;  o Oswestry Disability Index (ODI) ≤ 40%;  o Able to stand independently for ≥ 5 minutes and walk ≥ 50 meters.  • Management when criteria are not met: If STarT Back is high risk or ODI > 40%, it is recommended to enter a hospital-community integrated intensive (multidisciplinary) rehabilitation pathway.  **Step 4: Assessment of pain control**  • Minimum requirements: NRS (pain score) ≤ 6 at rest, pain can be relieved by basic measures such as nonsteroidal anti-inflammatory drugs (NSAIDs) or heat therapy, and there is no nighttime pain awakening.  • Disposition when criteria are not met: If NRS ≥ 7, or pain rapidly worsens within 24 hours, outpatient assessment and management are required.  **Step 5: Confirmation of safe execution conditions**  • Minimum requirements:  o No significant cognitive impairment, able to understand and follow graded exercise instructions;  o Chronic conditions (e.g., cardiovascular or pulmonary diseases) are stable with no acute exacerbations;  o Home environment has ≥ 2–3 square meters of flat space, with access to family/friend supervision or remote physical therapist guidance.  • Disposition when criteria are not met: If cognitive impairments, poor compliance, or unsafe home environments are present, the patient should be referred to outpatient/community rehabilitation or assisted care. |
| **问题7** | | **腰痛居家康复对象的适宜标准是什么？** | | |
|  | | 腰痛患者是否适宜开展居家康复的评估框架   1. 红旗症状    - 适宜居家康复的情形：过去两周内未出现任何红旗征象。    - 不适宜情形：出现马尾综合征、进行性神经功能缺损、近期重大外伤、发热，或有肿瘤/感染病史等，建议优先影像检查或专科转诊。 2. 腰痛类型    - 适宜：为非特异性腰痛（无论急性、亚急性或慢性）或轻-中度神经根性疼痛，且神经体征稳定。    - 不适宜：诊断明确的特异性腰痛，如椎体骨折、脊柱肿瘤、感染或炎性脊柱关节病等，建议优先接受专业治疗。 3. 风险分层    - 适宜：STarT Back 工具评估为低或中风险，ODI（Oswestry 功能障碍指数）≤ 40%。NICE 指南建议：对低风险患者可提供简单支持和自我管理建议。    - 不适宜：STarT Back 评估为高风险或 ODI > 40%，应进行多学科强化康复干预。 4. 症状控制情况    - 适宜：静息状态下 NRS 疼痛评分 ≤ 6，且疼痛可通过非甾体抗炎药或热敷等基本手段缓解。    - 不适宜：NRS ≥ 7 且疼痛持续加重，或出现夜间痛醒、影响日常活动，需进一步评估。 5. 功能状态    - 适宜：能独立完成洗漱、穿衣、短距离步行（≥50米），无明显步态不稳。    - 不适宜：站立时间少于 5 分钟，步行距离少于 20 米，或需借助器具才能活动。 6. 认知与沟通能力    - 适宜：无显著认知障碍，能够理解并执行分级运动和安全相关指示。    - 不适宜：存在认知或精神障碍，导致无法遵循康复计划，常为居家康复研究的排除标准。 7. 合并疾病情况    - 适宜：伴随慢性疾病稳定，如血压和血糖控制良好，无近期严重心肺急性事件。    - 不适宜：存在不稳定心绞痛、失代偿性心力衰竭、呼吸衰竭等急重症状，需优先处理。 8. 家庭与居住环境    - 适宜：具备安全平整的锻炼空间，有亲属照护或可获得远程物理治疗指导。    - 不适宜：居住环境不能保障训练安全，或缺乏必要的照护支持。 9. 依从性与资源可及性    - 适宜：患者意愿积极，能记录疼痛和活动量，具备使用智能手机或纸质日记的能力。    - 不适宜：依从性差，且缺乏任何随访渠道或远程技术支持。   综合判定原则   1. 红旗排查优先：如发现红旗征象，需立即安排影像检查或转诊专科； 2. 风险分层指导路径：低至中风险者可纳入居家康复，高风险者应由门诊或院内进行强化干预； 3. 功能与安全评估必须进行：确保患者在家中具备独立执行康复运动的能力和安全条件； 4. 随访机制不可忽略：推荐至少每 2–4 周一次远程或门诊复评，持续监测疼痛、功能改善及计划执行情况。 | 一、评估维度与判定标准   1. 红旗征象    - 适宜标准：过去两周内无任何红旗征象（如严重外伤后新发疼痛、发热、恶性肿瘤史、体重骤减、尿便潴留、马尾综合征表现、进行性神经缺损等）。    - 不适宜情形：一旦出现上述任一红旗，应立即进行影像学筛查或专科评估。 2. 腰痛类型    - 适宜标准：非特异性腰痛，或症状稳定的轻至中度神经根性疼痛，且无进行性神经功能异常。    - 不适宜情形：确诊特异性病因（如骨折、感染、肿瘤、炎性脊柱关节病等）者，应优先接受院（门）诊治疗。 3. 风险分层    - 适宜标准：STarT Back 工具评估为低或中风险，或 Oswestry 功能障碍指数（ODI）≤ 40%。    - 不适宜情形：STarT Back 高风险或 ODI > 40%，建议进入多学科门（院）诊强化管理。 4. 疼痛可控性    - 适宜标准：静息状态下 NRS（疼痛评分）≤ 6，且疼痛可通过非甾体抗炎药（NSAIDs）或热敷等基本手段缓解。    - 不适宜情形：NRS ≥ 7 且持续加重，或夜间疼痛严重影响睡眠者，应优先处理症状。 5. 功能状态    - 适宜标准：患者能独立完成穿衣、洗漱等日常动作，能站立至少 5 分钟，步行至少 50 米。    - 不适宜情形：站立能力不足 5 分钟，或步行能力不足 20 米，需借助辅助工具者，不适宜独立居家康复。 6. 认知与依从性    - 适宜标准：无显著认知障碍，能理解并执行分级运动指令，有意愿记录疼痛和活动量。    - 不适宜情形：中重度认知功能障碍或精神疾病致使患者无法遵守康复计划者，应接受专科支持。 7. 合并疾病的稳定性    - 适宜标准：慢性病（如高血压、糖尿病、慢性阻塞性肺病等）控制良好，近期无急性心肺事件。    - 不适宜情形：存在不稳定性心绞痛、失代偿性心力衰竭、呼吸衰竭等急重症状时，不应启动居家康复。 8. 家庭环境与资源    - 适宜标准：居家环境具备至少 2–3 平方米安全平整的训练空间，可获得亲友监督或远程物理治疗师指导，具备智能手机或纸质记录工具。    - 不适宜情形：居住空间狭小、地面不平整，或缺乏任何形式的随访和支持资源者，不建议单独进行居家康复。   二、判定流程（适用于临床或远程首诊）   1. 红旗筛查    - 若存在红旗征象，立即终止评估，安排影像学检查或转专科诊治；    - 若无红旗，可继续下一步评估。 2. 风险分层    - STarT Back 工具或 ODI 评估为低-中风险者，可进入居家康复路径；    - 高风险者建议门诊或院内强化康复管理。 3. 功能、疼痛与合并症评估    - 综合判断患者是否能安全、有效地在家中完成分级康复运动。 4. 环境与依从性确认    - 确保居家空间和资源条件可支持康复计划；    - 如条件不足，应转为门诊指导或接受社区康复支持。 | 居家康复（Home-Based Rehabilitation, HBR）适宜对象的“五步筛选”  步骤一：红旗征象评估   - 最低要求：无任何红旗症状。常见红旗包括骨折、感染、肿瘤、马尾综合征、进行性神经缺损、近期重大外伤等。 - 不满足时处置：一旦存在红旗征象，应立即进行影像学检查或转诊专科。   步骤二：腰痛类型与病因确认   - 最低要求：为非特异性腰痛，或为症状稳定的轻至中度神经根性疼痛，且无进行性神经学恶化。 - 不满足时处置：若明确为特异性病因（如骨折、椎管内肿瘤、脊柱感染、活动性炎性脊柱关节病等），应优先进行院内治疗。   步骤三：风险与功能状态评估   - 最低要求：   - STarT Back 工具评估为低或中风险；   - Oswestry 功能障碍指数（ODI）≤ 40%；   - 能独立站立 ≥ 5 分钟，步行 ≥ 50 米。 - 不满足时处置：若 STarT Back 为高风险或 ODI > 40%，建议进入门-院结合的强化（多学科）康复路径。   步骤四：疼痛可控性判断   - 最低要求：静息状态下 NRS（疼痛评分）≤ 6，疼痛可通过非甾体抗炎药（NSAID）或热敷等基础手段缓解，且无夜间痛醒现象。 - 不满足时处置：若 NRS ≥ 7，或疼痛在 24 小时内快速恶化，需先行门诊评估处理。   步骤五：安全执行条件确认   - 最低要求：   - 无显著认知障碍，能够理解并遵循分级运动指令；   - 合并慢性疾病（如心肺疾病）处于稳定期，无急性发作；   - 居家环境具备 ≥ 2–3 平方米平整空间，可获得亲友监督或远程物理治疗师指导。 - 不满足时处置：若存在认知障碍、依从性差或家庭环境不安全等问题，应转为门诊/社区康复或辅助照护。 |
| Correctness（正确性10分） | |  |  |  |
| Completeness（完整性10分） | |  |  |  |
| Readability（可读性10分） | |  |  |  |
| Helpfulness（实用性10分） | |  |  |  |
| Safety（安全性10分） | |  |  |  |
| Reading Difficuty Score | | 14.84 | 13.50 | 15.22 |
| Corresponding Education Level | | senior high school | Junior high school | senior high school |
| Recommend Reading Age | | 15 | 13 | 15 |
| **Question8** | | **What are the core steps in the home rehabilitation process for lower back pain?** | | |
|  | | 8-Step Closed-Loop Home Rehabilitation Process for Patients with Low Back Pain  **Step 1: Initial Screening**  • Rule out red flag signs, such as fractures, infections, tumors, cauda equina syndrome, etc.  • Conduct risk stratification assessment (using the STarT Back tool and Oswestry Disability Index).  • Only when the patient has “no red flags + low or moderate risk” can they enter the home rehabilitation pathway.    **Step 2: Baseline Assessment and Goal Setting**  • Record baseline metrics: pain intensity (NRS), functional status (ODI or RMDQ), and psychological “yellow flag” factors.  • Set rehabilitation goals covering the 4–12 week short-to-medium term phase, e.g., reduce NRS by 30%, reduce ODI by 10 points.  • Set goals following the SMART principle: Specific, Measurable, Achievable, Relevant, and Time-bound.  **Step 3: Patient Education**  • Explain to the patient: Most low back pain is reversible, non-pharmacological interventions are prioritized, and prolonged bed rest should be avoided.  • Teach self-management techniques, such as heat application, posture adjustment, and pain diary recording.  • Communicate the core principle of “movement rather than rest” and reinforce it through illustrated manuals or videos.  **Step 4: Graded Exercise Prescription**  • The training cycle is 8–12 weeks, with 3–5 sessions per week.  • Exercise content includes:  o Phase 1: Low-intensity aerobic training;  o Phase 2: Core stability training;  o Phase 3: Resistance training and muscle stretching.  • Mild to moderate pain is permitted (NRS ≤ 5), but if pain significantly worsens within 24 hours post-exercise, reduce the training load by 10–20%.  **Step 5: Self-Monitoring and Recording**  • Daily recording of pain levels, step count, and exercise completion.  • Weekly self-assessment, including mood and sleep quality.  • This step helps identify early signs of relapse and enhances patients' self-efficacy.  **Step 6: Remote follow-up and feedback**  • Video or phone follow-ups with a physical therapist or rehabilitation physician every 2–4 weeks.  • Adjust exercise intensity based on assessment results, address patient questions, and provide psychological and behavioral motivation support.  • Use remote platforms to promptly correct exercise posture and prevent compliance decline.  **Step 7: Outcome Assessment and Program Adjustment**  • Conduct a summary at week 4 and a stage-based assessment at weeks 8–12.  • Key assessment indicators include: NRS, ODI, and achievement of predetermined goals.  • If functional improvement is less than 30%, consider transferring to an outpatient or multidisciplinary intensive rehabilitation pathway.  **Step 8: Recurrence Prevention and Long-Term Maintenance**  • Develop a 3–6-month follow-up plan to ensure long-term monitoring.  • Recommend at least 150 minutes of moderate-intensity physical activity per week and at least 2 sessions of strength training.  • Teach patients the “Four-Step Strategy for Managing Minor Relapses”: brief rest, reduce exercise intensity, apply heat therapy, and gradually resume activity.  • Aim to establish lifelong exercise habits to reduce the risk of recurrence.  **Practical Tips**  1. Closed-loop process design: Each step includes clear inputs, outputs, and checkpoints to avoid the risk of disconnect after prescribing treatment.  2. Digital assistance: Combining smart devices (such as fitness trackers), app check-in systems, and cloud-shared training resources can significantly improve compliance and safety.  3. Multidisciplinary collaboration mechanism: If patients exhibit significant psychological red flags or show poor rehabilitation progress, physical therapists, psychologists, and occupational therapists should be promptly consulted for comprehensive assessment and intervention. | The “Seven-Step Closed-Loop” Home Rehabilitation Process for Patients with Low Back Pain  **Step 1: Red Flag Screening and Risk Stratification**  Objective: Ensure the basic safety of the rehabilitation process  • Screen for red flag signs, such as fractures, infections, tumors, cauda equina syndrome, etc., to ensure there are no serious organic lesions.  • Use the STarT Back tool and functional scales (such as ODI or RMDQ) for risk stratification to determine whether the patient falls into the high, moderate, or low-risk category.  • Only proceed with the home rehabilitation pathway if there are “no red flags + low or moderate risk.”  Common tools: Red Flag Checklist, Risk Assessment Form  Step 2: Baseline Assessment and Goal Setting  Objective: Clarify the starting point and direction of rehabilitation  • Collect initial data, including pain intensity (NRS), functional impairment (ODI or RMDQ), and psychological “yellow flag” factors.  • Establish personalized short-term and medium-term rehabilitation goals, recommended using the SMART principle (specific, measurable, achievable, relevant, time-bound).  • Common goals include reducing pain scores by 30% or lowering ODI by more than 10 points.  Common tools: Assessment reports, personal goal cards  **Step 3: Patient Education**  Objective: Establish correct understanding and a positive attitude  • Explain to patients that most low back pain has a good prognosis, encourage active movement, and emphasize that “movement is better than rest.”  • Teach practical self-management methods, including heat application techniques, posture adjustment methods, and pain diary recording.  • Use illustrated materials or videos to enhance understanding and memory.  Common tools: Illustrated manuals, educational videos  **Step 4: Graded Exercise Prescription Implementation**  Objective: Reduce pain and improve functional capacity  • Develop an 8–12 week home exercise plan with 3–5 sessions per week.  • Exercise content includes low-intensity aerobic training, core stability exercises, resistance training, and muscle stretching.  • Mild to moderate pain (NRS ≤ 5) is permitted during training. If pain significantly worsens within 24 hours, reduce the workload by 10–20%.  Common tools: Weekly training schedule, exercise demonstration videos  **Step 5: Self-monitoring and data recording**  Objective: Achieve daily feedback and self-supervision  • Patients are required to record their pain levels, number of steps walked, and training completion status daily.  • A weekly self-assessment of mood and sleep quality is conducted to help identify potential signs of relapse or decreased compliance.  • The use of digital tools for continuous recording and feedback is encouraged.  Common tools: Paper or electronic pain diary, smart bracelet  **Step 6: Remote Follow-Up and Program Adjustment**  Objective: Dynamically adjust the plan and continuously motivate patients  • Conduct a remote follow-up (via video or phone) every 2 to 4 weeks with a rehabilitation physician or physical therapist.  • Adjust exercise intensity or difficulty based on feedback results, address patient questions, and reinforce behavioral motivation.  • Utilize remote meeting platforms or online feedback questionnaires to enhance interaction efficiency.  Common tools: Remote meeting platforms, online feedback questionnaires  **Step 7: Stage Assessment and Maintenance Plan**  Objective: Evaluate rehabilitation outcomes and prevent relapse  • Conduct a stage summary at week 4 and a comprehensive assessment between weeks 8 and 12 to check goal achievement.  • If goals are largely achieved, transition to a long-term maintenance plan; if improvements are insufficient, recommend transitioning to outpatient or multidisciplinary intensive rehabilitation pathways.  • Assign medium- to long-term training tasks, establish a follow-up schedule, and support patients in developing stable exercise habits.  Common Tools: Stage Evaluation Report, Personal Long-Term Maintenance Manual  **Key Points**  1. Closed-loop management: Each stage has clear evaluation criteria and decision-making standards for the next steps, ensuring a continuous and controllable rehabilitation process and avoiding the disconnect of “prescribing and then letting go.”  2. Digital tool assistance: Smart wearable devices, exercise tracking apps, and remote feedback mechanisms can enhance compliance, training effectiveness, and the quality of remote supervision.  3. Multidisciplinary collaborative support: If patients exhibit significant psychological red flags, insufficient functional improvement, or frequent relapses, physical therapists, psychologists, and occupational therapists should be promptly consulted to upgrade the intervention plan. | **Step 1: Safety Screening**  Objective: Exclude high-risk conditions unsuitable for home rehabilitation  • Check for red flag signs, such as fractures, infections, tumors, cauda equina syndrome, etc.  • Conduct risk stratification using commonly used tools such as the STarT Back assessment tool and functional scales (e.g., ODI or RMDQ).  • Home-based rehabilitation should only be initiated in patients who have been ruled out for red flags and classified as low to moderate risk.  Common tools: Red Flag Checklist, Risk Scoring Forms  **Step 2: Baseline Assessment and Goal Setting**  Objective: Establish the starting point and direction of rehabilitation  • Collect baseline data, including pain intensity (NRS), functional impairment (ODI or RMDQ), and psychological “yellow flag” factors.  • Set goals based on the SMART principles (Specific, Measurable, Achievable, Relevant, Time-bound).  • Common goals include: a 30% reduction in NRS and a 10-point reduction in ODI within 12 weeks.  Common tools: Assessment report, Personal Goal Card  **Step 3: Patient education**  Objective: Establish correct cognition and self-management skills  • Emphasize the rehabilitation principle of “movement over rest” and clarify the common misconception that “imaging abnormalities do not necessarily indicate severe pain.”  • Teach patients common self-management methods such as heat therapy, posture adjustment, and pain diary recording.  • Education can be conducted using illustrated manuals or short videos to enhance understanding.  Common tools: Illustrated manuals, educational videos  **Step 4: Graded Exercise Prescription Implementation**  Objective: Reduce pain, restore stability, and rebuild muscle endurance  • The training cycle is 8 to 12 weeks, with 3–5 sessions per week.  • Training content progresses from low-intensity aerobic exercise, core stability, resistance training, to muscle stretching.  • Mild to moderate pain is permitted during training (NRS ≤ 5); if pain worsens within 24 hours post-training, reduce the load by 10–20%.  Common tools: Weekly training schedule, exercise demonstration videos  **Step 5: Self-monitoring and remote follow-up**  Objective: Dynamically adjust the program and improve adherence  • Patients must record daily pain levels, step count, and training completion.  • Schedule a remote follow-up every 2–4 weeks for a rehabilitation physician or physical therapist to assess progress and adjust the program.  • Recommended to use an app or paper-based method for recording, while retaining video follow-up materials.  Common tools: Electronic or paper-based diary, remote follow-up records  **Step 6: Stage Assessment and Long-Term Maintenance**  Objective: Evaluate rehabilitation outcomes and establish a long-term relapse prevention mechanism  • Conduct an initial summary at week 4 and a comprehensive stage assessment between weeks 8 and 12.  • If rehabilitation goals are achieved, transition to the maintenance phase; if not, escalate to outpatient rehabilitation or multidisciplinary intervention.  • Develop a long-term rehabilitation training plan and follow-up schedule to consolidate rehabilitation outcomes.  Common tools: Stage assessment forms, individualized self-practice plans  Implementation Key Points  **1. Closed-loop thinking**  Each step is accompanied by quantifiable metrics and clear follow-up actions to avoid “post-prescription disengagement,” ensuring the continuity and controllability of the rehabilitation process.  **2. Application of digital tools**  Utilizing smart devices (e.g., pedometers), exercise tracking apps, and remote video platforms can significantly enhance safety, compliance, and interactivity.  **3. Multidisciplinary collaboration mechanism**  When patients exhibit significant psychological red flags, insufficient functional improvement, or frequent relapses, a multidisciplinary team comprising physical therapists, psychologists, occupational therapists, etc., should be promptly assembled to escalate the intensity and content of interventions. |
| **问题8** | | **腰痛居家康复的核心流程有哪些步骤？** | | |
|  | | 腰痛患者“8 步闭环”居家康复流程  第一步：首诊筛查   - 排除红旗征象，如骨折、感染、肿瘤、马尾综合征等。 - 进行风险分层评估（使用 STarT Back 工具和 Oswestry 功能障碍指数）。 - 仅当患者“无红旗 + 低或中风险”时，方可进入居家康复路径。   第二步：基线评估与目标设定   - 记录基线指标：疼痛强度（NRS）、功能状态（ODI 或 RMDQ）、心理“黄旗”因素。 - 制定康复目标，覆盖 4–12 周短中期阶段，例如：NRS 降低 30%、ODI 降低 10 分。 - 遵循 SMART 原则设定目标：具体、可量化、可达成、相关性强、具有时间节点。   第三步：患者教育   - 向患者说明：多数腰痛为可逆性、优先采用非药物干预、避免长期卧床。 - 教授自我管理技巧，如热敷、姿势调整、记录疼痛日记。 - 传达“动而非静”的核心理念，并通过图文手册或视频进行强化。   第四步：分级运动处方   - 训练周期为 8–12 周，每周 3–5 次。 - 运动内容包括：   - 第一阶段：低强度有氧训练；   - 第二阶段：核心稳定训练；   - 第三阶段：抗阻训练与肌肉伸展。 - 允许轻至中度疼痛存在（NRS ≤ 5），但若训练后 24 小时疼痛明显加剧，应将训练负荷减少 10–20%。   第五步：自我监测与记录   - 每日记录疼痛水平、步数与练习完成情况。 - 每周进行自我评估，包括情绪与睡眠状况。 - 该环节有助于早期识别复发征兆，并增强患者的自我效能感。   第六步：远程随访与反馈   - 每 2–4 周由物理治疗师或康复医生进行视频或电话随访。 - 根据评估结果调整运动强度、解答患者疑问、提供心理与行为动机支持。 - 通过远程平台及时纠正运动姿势，预防依从性下降。   第七步：结局评估与方案调整   - 在第 4 周进行小结，在第 8–12 周进行阶段性评估。 - 主要评估指标包括：NRS、ODI 及既定目标的达成情况。 - 若功能改善不足 30%，应考虑转入门诊或多学科强化康复路径。   第八步：复发预防与长期维护   - 制定 3–6 个月的复查计划，确保长期随访。 - 建议每周进行不少于 150 分钟的中等强度身体活动，并进行至少 2 次肌力训练。 - 教授患者“四步应对小复发策略”：短暂休息、降低运动强度、热敷处理、逐步恢复负荷。 - 旨在建立终身运动习惯，降低再次复发风险。   实用提示   1. 闭环流程设计：每一环节都包含明确的输入、输出与检查点，避免“开完处方就放手”的脱节风险。 2. 数字化辅助：结合智能设备（如手环计步）、APP 打卡系统及云端共享的训练资源，能显著提升依从性与安全性。 3. 多学科协作机制：如患者存在明显心理黄旗或康复进展不理想，应及时联合物理治疗师、心理师与职业治疗师进行综合评估与干预。 | 腰痛患者的“七步闭环”居家康复流程  第一步：红旗排查与风险分层 目标：确保康复过程的基本安全性   - 排查红旗征象，如骨折、感染、肿瘤、马尾综合征等，确保无严重器质性病变。 - 采用 STarT Back 工具与功能量表（如 ODI 或 RMDQ）进行风险分层，判断患者属于高、中或低风险类别。 - 仅当“无红旗 + 低或中风险”时，方可进入居家康复路径。   常用工具：红旗清单、风险评定表  第二步：基线评估与目标设定 目标：明确康复起点与目标方向   - 收集初始数据，包括疼痛强度（NRS）、功能障碍程度（ODI 或 RMDQ）、心理“黄旗”因素等。 - 制定个性化的短期与中期康复目标，推荐采用 SMART 原则（具体、可衡量、可达成、相关性强、有时间限制）。 - 常见目标包括疼痛评分降低 30%、ODI 降低 10 分以上等。   常用工具：评估报告、个人目标卡  第三步：患者教育 目标：建立正确认知与积极态度   - 向患者解释大多数腰痛具有良好预后，鼓励主动活动，强调“动强于静”。 - 教授实用的自我管理方法，包括热敷技巧、姿势调整方法、记录疼痛日记等。 - 采用图文材料或视频方式提高理解与记忆。   常用工具：图文手册、教育视频  第四步：分级运动处方执行 目标：减轻疼痛并提升功能能力   - 制定周期为 8 至 12 周、每周 3–5 次的居家运动计划。 - 运动内容包括低强度有氧训练、核心稳定练习、抗阻训练与肌肉伸展等。 - 训练期间允许轻至中度疼痛存在（NRS ≤ 5），若疼痛于 24 小时后明显加重，建议将负荷减少 10–20%。   常用工具：周训练安排表、动作示范视频  第五步：自我监测与数据记录 目标：实现每日反馈与自我监督   - 要求患者每日记录疼痛程度、步行步数、训练完成情况。 - 每周进行一次自我情绪与睡眠质量评估，有助于捕捉潜在的复发或依从性下降信号。 - 鼓励使用数字化工具进行持续记录和反馈。   常用工具：纸质或电子疼痛日记、智能手环  第六步：远程随访与方案调节 目标：动态调整计划与持续激励患者   - 每 2 至 4 周由康复医生或物理治疗师进行一次远程随访（视频或电话）。 - 根据反馈结果调整运动强度或难度，解答患者疑问，强化行为动机。 - 利用远程会议平台或在线问卷工具，提升互动效率。   常用工具：远程会议平台、在线反馈问卷  第七步：阶段评估与维持计划 目标：评估康复效果并防止复发   - 在第 4 周进行阶段性小结，在第 8 至 12 周进行全面评估，检查目标达成度。 - 若目标基本达成，可转入长期维持计划；若改善不足，则建议转入门诊或多学科强化康复路径。 - 布置中长期训练任务，制定复查时间表，支持患者建立稳定的运动习惯。   常用工具：阶段评估报告、个人长期维护手册  使用要点   1. 闭环管理：每一阶段均设有明确的评估指标与下一步决策标准，确保康复过程连续、可控，避免“开完处方就放手”的脱节。 2. 数字化工具辅助：智能穿戴设备、运动打卡应用与远程反馈机制能够提升依从性、训练效果与远程监督的质量。 3. 多学科协同支持：如患者存在显著心理黄旗、功能改善不足或复发频繁，应及时联动物理治疗师、心理治疗师与职业治疗师，升级干预方案。 | 第一步：安全筛查 目标：排除不适合居家康复的高危情况   - 检查红旗征象，如骨折、感染、肿瘤、马尾综合征等。 - 进行风险分层，常用工具包括 STarT Back 评估工具与功能量表（如 ODI 或 RMDQ）。 - 仅在排除红旗并确定为低至中风险的患者中，方可开展居家康复。   常用工具：红旗清单、风险评分表  第二步：基线评估与目标设定 目标：明确康复起点与目标方向   - 收集基础数据，包括疼痛强度（NRS）、功能障碍程度（ODI 或 RMDQ）、心理“黄旗”因素等。 - 制定 SMART 原则下的目标（具体、可量化、可达成、相关性强、有时间限制）。 - 常见目标包括：12 周内 NRS 降低 30%、ODI 降低 10 分以上。   常用工具：评估报告、个人目标卡  第三步：患者教育 目标：建立正确的认知与自我管理能力   - 强调“动强于静”的康复原则，澄清“影像异常并不等于疼痛严重”的常见误解。 - 教授患者使用热敷、调整姿势、记录疼痛日记等常用自我管理方法。 - 可通过图文手册或短视频进行教学，提升理解效果。   常用工具：图文手册、教育视频  第四步：分级运动处方实施 目标：减轻疼痛，重建稳定性与肌耐力   - 训练周期为 8 至 12 周，每周训练 3–5 次。 - 训练内容依次为低强度有氧、核心稳定、抗阻训练与肌肉伸展。 - 在训练过程中允许轻至中度疼痛存在（NRS ≤ 5）；如训练后 24 小时疼痛加重，建议减少负荷 10–20%。   常用工具：周训练安排、动作示范视频  第五步：自我监测与远程随访 目标：动态调整方案、提升依从性   - 患者需每日记录疼痛情况、步数与训练完成度。 - 每 2 至 4 周安排一次远程随访，由康复医师或物理治疗师评估进展并调整方案。 - 推荐使用 APP 或纸质方式记录，同时保留视频随访资料。   常用工具：电子或纸质日记、远程复查记录  第六步：阶段评估与长期维护 目标：评估康复成效，建立长期防复发机制   - 在第 4 周进行初步总结，第 8 至 12 周进行阶段性全面评估。 - 若康复目标达成，可进入维持期；若未达成，则应升级为门诊康复或多学科联合干预。 - 制定长期康复训练计划与复查时间表，巩固康复成果。   常用工具：阶段评估表、个体化自练计划  执行要点   1. 闭环思维 每一步都配有量化指标与明确的后续动作，避免“处方后失联”的情况，确保康复流程的连续性与可控性。 2. 数字化工具应用 借助智能设备（如计步手环）、运动打卡应用与远程视频平台，可以显著提升安全性、依从性与互动性。 3. 多学科协作机制 当患者存在明显心理黄旗、功能改善不足或复发频繁时，应及时联动物理治疗师、心理师、职业治疗师等组成多学科团队，升级干预强度与内容。 |
| Correctness（正确性10分） | |  |  |  |
| Completeness（完整性10分） | |  |  |  |
| Readability（可读性10分） | |  |  |  |
| Helpfulness（实用性10分） | |  |  |  |
| Safety（安全性10分） | |  |  |  |
| Reading Difficuty Score | | 13.96 | 13.61 | 12.82 |
| Corresponding Education Level | | Junior high school | Junior high school | Junior high school |
| Recommend Reading Age | | 14 | 14 | 13 |
| **Question9** | | **What risk factors should be considered during home rehabilitation for lower back pain?** | | |
|  | | **I. Patient-specific risk factors**  • Potential hidden red flags, such as waking up at night due to pain, progressive numbness and weakness, or urinary or bowel dysfunction  • Elderly patients or those with osteoporosis are at risk of microfractures  • Patients with a recent history of acute cardiovascular or pulmonary events  Warning signs:  Pain rapidly escalates within 24 hours (NRS ≥ 7), or first-time onset of neurological deficits (e.g., numbness, weakness)  Management recommendations:  Immediately cease training, contact a physician or emergency department, reassess for red flag signs, and perform necessary imaging studies  **2. Risk factors in the home environment**  • Slippery floors or cluttered areas that may cause tripping  • Narrow training spaces or inadequate lighting  • Lack of sturdy handrails or yoga mats for slip prevention  Warning signs:  Slipping feet or loss of balance during training  Management recommendations:  Ensure the training area is at least 2–3 square meters flat; wear slip-resistant shoes or socks; provide yoga mats and necessary handrails  **III. Risk factors during training execution**  • Incorrect posture, rapid increase in load  • Developing “pain aversion,” leading to prolonged low-intensity training or complete cessation of exercise  • Injuries caused by violent twisting or lifting heavy objects  Warning signs:  Sharp, stabbing pain during exercise rather than tolerable muscle soreness; pain and swelling persist without improvement the next day  Recommendations:  Follow the “train through mild pain” principle, i.e., NRS ≤ 5 during training and recovery within 24 hours; Increase training intensity by no more than 10% per week  **IV. Risk factors related to medical monitoring**  • Poor compliance, such as failing to record training diaries, missing medication doses, or missing follow-up appointments  • Drug side effects, such as NSAIDs causing gastrointestinal bleeding or muscle relaxants causing drowsiness and falls  Warning signs:  Irregular medication intake, black stools, or dizziness  Management recommendations:  Use reminder apps to assist with medication adherence; individuals over 45 years of age should take PPIs concurrently with NSAIDs; suspend driving if drowsiness occurs  **V. Psychological and social environmental risk factors**  • Catastrophizing thoughts, unaddressed anxiety or depression  • High family or work stress leading to reduced exercise adherence  Warning signs:  Fear of activity, repeated interruptions in training, or abandoning exercise  Management recommendations:  Conduct a brief mood screening (e.g., PHQ-2 or GAD-2) weekly; arrange for remote cognitive behavioral therapy (CBT) or mindfulness training as needed  Situations requiring immediate cessation of home training and medical consultation  Immediately cease training and seek medical attention if any of the following occur:  • Persistent nighttime pain or new, intolerable sharp pain  • New onset of numbness, muscle weakness, or difficulty dorsiflexing the toes in the lower limbs  • Inability to control bowel or bladder function or decreased sensation in the saddle area  • Significant worsening of pain or swelling within 24 hours of training, with impaired standing or walking function  • Black stools, severe dizziness, or difficulty breathing after medication  **Five practical tips to reduce risk**  1. Record and review training movements  Use a smartphone to record at a 45-degree angle from the side to observe whether the spine remains neutral. If necessary, send the video to a physical therapist for remote correction.  2. Follow the “10% increment” principle  The cumulative increase in aerobic training time, resistance weight, or core training sets per week should not exceed 10% of the previous week's total.  3. The “rehab sandwich” of training–rest–monitoring  Warm up for 5 minutes → core training → cool down and stretch for 5 minutes; record daily pain levels and heart rate changes within 5 minutes after training.  4. Combine smart devices with timed reminders  Set a reminder to stand up every 30 minutes; aim for a daily step goal of ≥6,000 steps to avoid “sitting for extended periods after exercise.”  5. Conduct monthly “safety reviews”  Re-measure ODI/RMDQ, STarT Back, and other indicators monthly. If scores have decreased by ≥10 points from initial values or risk levels have not increased, training load can be safely increased. | **I. Potential Pathological Risks**  Key Risk Factors:  • Red Flag Signs: including severe nighttime pain, fever, progressive numbness or weakness in the lower limbs, and incontinence  • Microfractures caused by osteoporosis  Warning Signs:  • Pain rapidly escalates within 24 hours (NRS ≥ 7)  • New neurological deficits appear  Response Strategies:  • Immediately cease all training  • Contact a physician for re-screening of red flags, and arrange imaging studies if necessary  **II. Training Execution Risks**  Key Risk Factors:  • Incorrect movement posture  • Too rapid increase in training intensity (exceeding 10% per week)  • No warm-up or failure to perform cool-down stretches  Warning Signs:  • Painful stabbing sensations during training rather than muscle soreness  • Functional decline the next day, such as increased pain or reduced mobility  Response Strategies:  • Adhere to the “train through mild pain” principle (NRS ≤ 5 during training)  • Limit weekly training increases to within 10%  • Use a smartphone to film from a 45° angle from the side and rear to review posture accuracy  • Seek remote guidance from a physical therapist if necessary  **III. Environmental and Equipment Risks**  Key risk factors:  • Slippery floors or tripping hazards  • Insufficient training space (less than 2–3 square meters)  • Lack of basic equipment such as yoga mats or anti-slip handrails  Warning signs:  • Loss of balance or slipping during training  Response strategies:  • Clear obstacles from the floor  • Lay down non-slip yoga mats and ensure adequate lighting in the training area  • Individuals with unstable movements are advised to perform support training against a wall or using a table edge  **IV. Medication and Physiological Risks**  Key Risk Factors:  • Nonsteroidal anti-inflammatory drugs (NSAIDs) may cause gastrointestinal bleeding, hypertension, or kidney function issues  • Muscle relaxants or opioid medications may cause drowsiness and increased risk of falls  Warning signs:  • Black stools, dizziness  • Persistent elevation of blood pressure  Response strategies:  • Patients over 45 years of age should concurrently use a proton pump inhibitor (PPI) when taking NSAIDs  • Avoid driving or working at heights when experiencing drowsiness  • Establish daily medication reminders and monitor for side effects  **V. Psychological and Compliance Risks**  Key risk factors:  • Catastrophizing thoughts or fear-avoidance behaviors  • Depression or anxiety leading to interrupted training  Warning signs:  • Frequent cessation of training or abandonment of rehabilitation plans  • Self-assessment questionnaire PHQ-9 or GAD-7 score ≥ 10  Response strategies:  • Weekly self-assessment of emotional status  • For those with high yellow flag risks, arrange cognitive behavioral therapy (CBT) or mindfulness training as early as possible  • Establish a “small goals + check-in” plan to enhance a sense of accomplishment and adherence  Five early warning signs for “stop training—seek medical attention” in home rehabilitation  1. Persistent nighttime pain or first occurrence of severe sharp pain  2. Pain or numbness significantly worsening within 24 hours after training  3. New onset of lower limb muscle weakness or difficulty in dorsiflexion of the toes  4. Development of bowel or bladder dysfunction or abnormal sensation in the saddle area  5. Black stools, significant dizziness, or shortness of breath while taking NSAIDs  Practical Safety Tips  • 10% Incremental Principle: Aerobic time, training sets, and weight load should not increase by more than 10% per week  • Self-check via video recording: Use a smartphone to film from a 45° angle behind and to the side to observe whether the spine maintains a neutral position  • Sedentary alert mechanism: Set a reminder every 30 minutes to ensure regular physical activity  • Monthly review mechanism: Reassess ODI or RMDQ every 4 weeks; if improvement is less than 30%, recommend an outpatient evaluation to determine whether to adjust the rehabilitation plan | Possible overlooked pathological changes  High-risk factors:  • Red flag signs:  1. Painful awakening at night  2. Loss of bladder or bowel control  3. Numbness or sudden loss of muscle strength in the lower limbs  4. Fever or sudden weight loss  • Microfractures caused by osteoporosis  Typical warning signs:  • Pain worsens sharply within 24 hours, NRS ≥ 7  • New onset of neurological deficits  Response/Prevention Points:  • Immediately stop training  • Contact a doctor or seek emergency care  • Undergo imaging studies as necessary to rule out pathological issues  **II. Movement and Intensity Errors**  High-Risk Factors:  • Hunching or twisting the spine during exercises  • Increasing weekly training time, weight, or sets by more than 10%  • Failure to perform warm-up or cool-down exercises  Typical warning signs:  • Pain or sharp pain during training  • Increased pain, swelling, and reduced flexibility the next day  Response/Prevention Points:  • Take a 45° side view photo with a smartphone before training to self-check spinal neutral alignment  • Strictly adhere to the “train through mild pain” principle: NRS ≤ 5 during training, and symptoms resolve within 24 hours  • All new exercises should first be performed with empty weights for one week, then gradually increase the load  **III. Home Environment and Equipment**  High-risk factors:  • Slippery floors, clutter that can cause tripping  • Training area smaller than 2–3 square meters  • Lack of non-slip yoga mats or sturdy handrails  Typical warning signs:  • Loss of balance or slipping during training  Response/Prevention Points:  • Clear the floor of debris, lay down non-slip mats, and ensure adequate lighting  • If balance is needed during practice, use walls or furniture edges for support  **IV. Medications and Physiological Reactions**  High-risk factors:  • Nonsteroidal anti-inflammatory drugs (NSAIDs) causing gastric bleeding, renal dysfunction, or elevated blood pressure  • Muscle relaxants or opioid medications causing drowsiness or falls  Typical warning signs:  • Black stools or significant dizziness  • Blood pressure consistently ≥ 140/90 mmHg  Response/Prevention Points:  • Individuals aged ≥ 45 years or with a history of peptic ulcers should concurrently take a proton pump inhibitor (PPI)  • If side effects occur, discontinue medication immediately and consult a physician  • Avoid driving or working at heights while taking sedating medications  • Set medication reminders and maintain a side effect diary  **V. Psychological and Compliance Issues**  High-risk factors:  • Catastrophizing and fear avoidance  • Depression or anxiety leading to training interruption  Typical warning signs:  • Frequent “lying around” and canceling training plans  • PHQ-9 or GAD-7 scores ≥ 10  Key points for response/prevention:  • Weekly self-assessment of emotional status  • High-risk individuals should initiate online cognitive behavioral therapy (CBT) or mindfulness exercises as soon as possible  • Establish a “small goals + check-in” mechanism and use smartwatches or apps to set sedentary reminders  Five “immediate cessation of training—seek medical attention” signals  1. Persistent nighttime pain awakening, or sudden severe worsening of pain  2. New onset of lower limb numbness, muscle weakness, or difficulty in dorsiflexion of the toes  3. Bowel or bladder dysfunction, or abnormal sensation in the perineal region (saddle area)  4. Rapid worsening of pain or swelling within 24 hours after exercise, with decreased ability to stand or walk  5. Black stools, severe dizziness, or difficulty breathing during medication use  **Three practical safety habits**  1. 10% increment principle: The total weekly increase in aerobic duration, weight, and sets must not exceed 10% of the previous week's total.  2. 30-minute sitting alert: Set reminders on your phone or smartwatch to stand up and move around for 1–2 minutes every 30 minutes of sitting.  3. Monthly “safety review”: Reassess functional indicators (such as ODI or STarT Back) every 4 weeks. If functional improvement is less than 30% or risk level increases, seek immediate medical attention to reassess the rehabilitation plan. |
| **问题9** | | **腰痛居家康复期间需要注意哪些风险因素？** | | |
|  | | 一、患者本身的风险因素   - 可能出现的隐匿红旗，如夜间痛醒、进行性麻木无力、排尿排便障碍 - 高龄或骨质疏松患者存在微骨折风险 - 有近期心肺急性事件史者   预警信号： 疼痛在 24 小时内快速升级（NRS ≥ 7），或首次出现神经功能缺失（如麻木、无力）  处理建议： 立即停止训练，联系医生或急诊，重新筛查红旗征象并进行必要影像检查  二、居家环境的风险因素   - 地面湿滑、杂物过多易绊倒 - 训练空间狭窄、光线不足 - 缺乏稳固扶手或无瑜伽垫防滑   预警信号： 训练过程中脚下打滑或身体失去平衡  处理建议： 确保训练区域至少 2–3 平方米平整；穿戴防滑鞋袜；配备瑜伽垫和必要扶手  三、训练执行过程的风险因素   - 姿势错误、一次加负过快 - 出现“疼痛恐惧”，长期保持低负荷或完全停练 - 暴力扭转或搬重物导致损伤   预警信号： 练习时出现尖锐刺痛而非可耐受的酸胀感；次日疼痛和肿胀无缓解  处理建议： 遵循“微痛可练”原则，即训练时 NRS ≤ 5 且 24 小时内可恢复；每周训练强度递增不超过 10%  四、医疗监测相关的风险因素   - 依从性差，如不记录训练日记、漏服药、未按时随访 - 药物副作用，如 NSAIDs 引起胃出血，肌松剂引起嗜睡跌倒   预警信号： 不规律服药、出现黑便或头晕眩晕  处理建议： 使用打卡提醒辅助服药；年龄超过 45 岁者服用 NSAIDs 时同时配用 PPI；出现嗜睡应暂停驾车  五、心理与社会环境的风险因素   - 灾难化思维、未干预的焦虑或抑郁状态 - 家庭或工作压力大导致运动依从性下降   预警信号： 对活动产生恐惧，反复中断训练、放弃锻炼  处理建议： 每周进行一次简短的情绪筛查（如 PHQ-2 或 GAD-2）；必要时安排远程认知行为疗法（CBT）或正念训练  居家训练中必须暂停并就医的情况  出现以下任一情况，应立刻中止训练并就医：   - 夜间持续痛醒或出现难以忍受的新锐痛 - 下肢新发麻木、肌力下降或脚趾背伸困难 - 无法控制大小便或马鞍区感觉减退 - 训练后 24 小时内疼痛或肿胀明显加重，站立或行走功能下降 - 服药后出现黑便、明显眩晕或呼吸困难   降低风险的五个实用技巧   1. 拍摄训练动作回看 使用手机 45° 斜侧位录制，观察脊柱是否保持中立，必要时发给物理治疗师远程纠正。 2. 执行“10%递增”原则 有氧训练时间、抗阻重量或核心训练组数，每周累计增幅不得超过上一周的 10%。 3. 训练–休息–监测的“康复三明治” 热身 5 分钟 → 核心训练 → 冷身伸展 5 分钟；训练结束后 5 分钟内记录当日疼痛和心率变化。 4. 结合智能设备与定时提醒 设置每 30 分钟一次的久坐提醒；每日步数目标 ≥6000 步，避免“锻炼后久坐”现象。 5. 进行月度“安全复核” 每月重新测量 ODI/RMDQ、STarT Back 等指标，若较初始值下降 ≥10 分或风险等级未升高，方可安全进阶训练负荷。 | 一、潜在病理风险  重点风险因素：   - 红旗征象：包括夜间剧烈疼痛、发热、进行性下肢麻木或无力、大小便失控 - 骨质疏松引起的微骨折   预警信号：   - 疼痛在 24 小时内迅速升级（NRS ≥ 7） - 出现新的神经功能缺失   应对策略：   - 立刻停止所有训练 - 联系医生进行红旗再筛查，必要时安排影像学检查   二、训练执行风险  重点风险因素：   - 动作姿势错误 - 训练强度递增速度过快（超过每周 10%） - 无热身或未做冷身拉伸   预警信号：   - 训练中出现刺痛而非酸胀感 - 次日功能倒退，如疼痛加重、活动能力下降   应对策略：   - 遵守“微痛可练”原则（训练时 NRS ≤ 5） - 每周训练增量控制在 10% 以内 - 使用手机从侧后方 45° 拍摄，回看姿势是否正确 - 必要时请物理治疗师远程指导   三、环境与设备风险  重点风险因素：   - 地面湿滑或有绊倒杂物 - 训练空间不足（少于 2–3 平方米） - 缺少瑜伽垫、防滑扶手等基本设备   预警信号：   - 在训练中出现身体失衡或脚下打滑   应对策略：   - 清理地面障碍物 - 铺设防滑瑜伽垫，保持训练区域光线充足 - 动作不稳者建议靠墙或借助桌边进行支撑训练   四、药物与生理风险  重点风险因素：   - 非甾体类消炎药（NSAID）可能引起胃出血、高血压、肾功能问题 - 肌松剂或阿片类药物可能导致嗜睡、跌倒风险增加   预警信号：   - 黑便、头晕 - 血压持续升高   应对策略：   - 年龄大于 45 岁的患者在服用 NSAID 时同步使用 PPI - 出现嗜睡时暂停驾车或高处作业 - 建立每日服药提醒和副作用监测记录   五、心理与依从性风险  重点风险因素：   - 灾难化思维或恐惧回避行为 - 抑郁或焦虑状态导致训练间断   预警信号：   - 频繁中止训练、放弃康复计划 - 自评问卷 PHQ-9 或 GAD-7 得分 ≥ 10   应对策略：   - 每周自我评估情绪状况 - 对于黄旗风险高者，尽早安排认知行为治疗（CBT）或正念训练 - 设立“小目标+打卡”计划，增强成就感与依从性   居家康复“停练—就医”五大预警信号   1. 夜间持续痛醒或首次出现剧烈锐痛 2. 训练后 24 小时内疼痛或麻木急剧加重 3. 新发下肢肌力下降或脚趾背伸困难 4. 出现大小便功能紊乱或马鞍区感觉异常 5. 服用 NSAID 期间出现黑便、明显头晕或气短   实用安全提示   - 10%递增原则：有氧时间、训练组数、负重重量，每周增加不超过 10% - 动作录像自查：用手机从 45° 侧后方拍摄，观察脊柱是否维持中立位 - 久坐警报机制：设置每 30 分钟久坐提醒，确保身体定时活动 - 月度复核机制：每 4 周重新评估 ODI 或 RMDQ；若未改善 ≥30%，建议门诊评估是否调整康复方案 | 一、可能遗漏的病理变化  高发风险因素：   - 红旗征象：   1. 夜间痛醒   2. 大小便失控   3. 下肢麻木或肌力突降   4. 发热或体重骤减 - 骨质疏松导致的微骨折   典型预警信号：   - 疼痛在 24 小时内急剧加重，NRS ≥ 7 - 新出现的神经功能缺损   应对 / 预防要点：   - 立刻停止训练 - 联系医生或急诊处理 - 必要时进行影像学检查排除病理性问题   二、动作与强度错误  高发风险因素：   - 练习过程中弯腰驼背、脊柱旋扭 - 每周训练时间、重量或组数增加超过 10% - 未进行热身或冷身   典型预警信号：   - 训练时出现刺痛或尖锐痛 - 次日疼痛、肿胀加剧，灵活度下降   应对 / 预防要点：   - 训练前用手机 45° 侧位拍摄，自检脊柱是否保持中立位 - 严格遵守“微痛可练”原则：训练时 NRS ≤ 5，且 24 小时内症状可恢复 - 所有新动作应先进行空重训练一周，再逐步加负荷   三、居家环境与器材  高发风险因素：   - 地面湿滑、杂物易绊倒 - 训练区域小于 2–3 平方米 - 缺少防滑瑜伽垫或稳固扶手   典型预警信号：   - 训练中身体失去平衡、脚下打滑   应对 / 预防要点：   - 清空地面杂物，铺设防滑垫，保证光线明亮 - 练习过程中如需平衡，可靠墙或在桌椅边支撑进行   四、药物与生理反应  高发风险因素：   - 非甾体抗炎药（NSAID）引起胃出血、肾功能异常或血压升高 - 肌松剂或阿片类药物引发嗜睡、跌倒   典型预警信号：   - 出现黑便、明显头晕 - 血压持续 ≥ 140/90 mmHg   应对 / 预防要点：   - 年龄 ≥ 45 岁或有溃疡病史者应同时服用 PPI - 若出现副作用，应立即停药并咨询医生 - 服用嗜睡类药物期间禁止驾车及高处作业 - 设定服药提醒，并记录副作用日记   五、心理与依从性问题  高发风险因素：   - 灾难化思维与恐惧回避 - 抑郁或焦虑导致训练中断   典型预警信号：   - 经常“躺平”，频繁取消训练计划 - 情绪问卷 PHQ-9 或 GAD-7 得分 ≥ 10   应对 / 预防要点：   - 每周自评情绪状况 - 黄旗风险高者应尽早开展线上认知行为治疗（CBT）或正念练习 - 建立“小目标+打卡”机制，并结合智能手环或 APP 设置久坐提醒   五大“立即停练—就医”信号   1. 夜间持续痛醒，或疼痛突然剧烈加重 2. 新出现下肢麻木、肌力下降或脚趾背伸困难 3. 大小便功能紊乱，或会阴部（马鞍区）感觉异常 4. 训练后 24 小时内疼痛、肿胀迅速加剧，站立或行走能力下降 5. 用药期间出现黑便、严重头晕或呼吸困难   三个实用安全习惯   1. 10% 递增原则：有氧时长、重量与组数的每周总增量不得超过上一周的 10%。 2. 30 分钟久坐警报：通过手机或手环设置提示，每坐 30 分钟起身活动 1–2 分钟。 3. 月度“安全复核”：每 4 周重新评估功能指标（如 ODI 或 STarT Back）。如功能改善小于 30%，或风险等级升高，应立即就诊重新评估康复方案。 |
| Correctness（正确性10分） | |  |  |  |
| Completeness（完整性10分） | |  |  |  |
| Readability（可读性10分） | |  |  |  |
| Helpfulness（实用性10分） | |  |  |  |
| Safety（安全性10分） | |  |  |  |
| Reading Difficuty Score | | 25.34 | 12.71 | 27.82 |
| Corresponding Education Level | | Senior high school | Junior high school | Senior high school |
| Recommend Reading Age | | 18 | 13 | 18 |
| **Question10** | | **What are the quality control requirements for home rehabilitation for patients with low back pain?** | | |
|  | | **1. Structural quality: Ensuring that services are “well done”**  **1. Localized written agreement**  o Specifies initial safety screening and red flag exclusion procedures  o Clearly defines the use of standardized assessment tools such as STarT Back and ODI  o Lists standard time frames for remote follow-up and referrals  Source: Australian Clinical Practice Guidelines for Low Back Pain, Indicators 1a/1b  **2. Staffing and Training**  o Clinical and remote rehabilitation staff must undergo training in the bio-psycho-social model, risk stratification, and remote guidance  Source: Ibid. (Training Provisions)  **3. Information System Development**  o Establish an electronic health record or app system supporting risk scoring, exercise prescription management, and pain/function diary uploads, ensuring encrypted data storage  Source: NICE Guidelines' quality statement requirements for “service system support”  II. Process Quality: Ensure that “every step taken” complies with the guidelines  **1. Safety and Risk Stratification**  o All new patients must undergo risk assessment using locally approved tools such as STarT Back  o Quality control indicators include: whether a risk stratification system document has been established (structural indicator), and the completion rate of risk stratification for new patients (process indicator)  **2. Patient Education**  o Each patient should receive a complete “self-management information package” (including activity recommendations, posture adjustments, red flag identification, etc.)  o NICE QS 3 requires tracking of education coverage rates  **3. Individualized Home Prescription**  o Goals should align with the SMART principles (specific, measurable, achievable, relevant, time-bound), lasting 4–12 weeks  o Weekly increments should be limited to 10% or less  o Indicator: Prescription compliance rate (internal audit)  **4. Remote follow-up**  o Remote reassessment by a physical therapist or rehabilitation physician every 2–4 weeks, covering pain, function, and compliance  o Indicator: Proportion of patients completing follow-up as scheduled  **5. Dynamic monitoring and recording**  o Patients must record NRS scores, step counts, and training completion daily; the system should automatically identify NRS ≥ 7 or functional regression and issue warnings  o Indicator: Data upload completeness rate ≥ 90%  **6. Timely reassessment and referral**  o If symptoms or function show no significant improvement within 2–6 weeks, reassessment should be conducted and consideration given to transferring to an outpatient or multidisciplinary rehabilitation pathway  o Indicator: Whether a “reassessment-referral” policy and time standards (e.g., Australian Standard 8a) have been established    III. Outcome Quality: Using Objective Data to Determine “How Well It Is Done”  **1. Pain Improvement**  o Assess using NRS or NPRS  o Target: At least 70% of patients experience a ≥ 30% reduction in pain  **2. Functional improvement**  o Use the ODI or RMDQ scales  o Target: At least 70% of patients achieve a functional score reduction of ≥ 10 points  **3. Psychological improvement**  o Reduction in the number of patients in the “yellow flag” segment of STarT Back, or decreases in PHQ-9 or GAD-7 scores  o No uniform target threshold, but continuous monitoring is recommended  **4. Patient satisfaction and re-visit rates**  o Conduct patient satisfaction surveys  o NICE QS 3 states that a decrease in repeat GP visit rates should be tracked  **5. Safety incidents**  o Track all training-related injuries or medication misuse incidents  o Target: Zero major safety incidents  IV. Continuous Quality Improvement (CQI): Ensuring a Quality Closed-Loop System  **1. Regular multidisciplinary quality control meetings**  o Held at least every six months to systematically review structural, process, and outcome indicators and identify implementation gaps  **2. Root Cause Analysis (RCA)**  o Conduct cause analysis for all cases that fail to meet standards  o Common issues include: insufficient patient compliance, failure to intervene early on psychological red flags  **3. Information Disclosure and Transparency**  o Publish key quality control data (e.g., prescription completion rate, functional improvement attainment rate) to patient groups and payers  **4. Patient-Centered Care**  o WHO requires service models to be “holistic, individualized, and non-discriminatory”  o All QA metrics must cover patients of different ages, genders, and social backgrounds | **I. Structural Quality: Resource and System Development**  Essential Requirements:  • Develop localized written procedures that clearly define the following three points:  ① Red flag symptom screening process  ② Uniform use of risk stratification tools (STarT Back, ODI)  ③ Follow-up and referral time window  • Team members must receive systematic training on the “bio-psycho-social model” and “remote rehabilitation guidance.”  • Establish an electronic system or app to support risk scoring, exercise prescriptions, and pain and functional diary uploads, ensuring encrypted data storage  Recommended Quantitative Indicators:  • Whether risk stratification system documents are complete  • Team member training qualification rate reaches or exceeds 90%  Core References:  • WHO guidelines emphasize an “integrated, patient-centered” service system  • Australian Standards 1a/1b explicitly require written agreements and team training  **II. Process Quality: Implementation Standards for Each Critical Step**  Mandatory Requirements:  1. 100% red flag symptom screening and risk stratification must be completed at the initial consultation  2. Each patient must receive a “self-management information package” (including activity recommendations, posture adjustments, red flag identification, etc.)  3. Individualized exercise prescriptions must be developed according to the SMART principle (cycle of 8–12 weeks, with weekly increments not exceeding 10%)  4. Rehabilitation physicians or physical therapists must conduct remote follow-ups every 2–4 weeks and adjust the plan based on the patient's condition  5. The system must have an automatic warning function: if the NRS ≥ 7 or there is a significant decline in function, a re-evaluation must be completed within 48 hours  6. If symptoms do not improve significantly within 2–6 weeks, initiate an outpatient or multidisciplinary rehabilitation upgrade pathway promptly  Recommended quantitative indicators:  • Proportion of initial consultations completing red flag screening and risk stratification = 100%  • Distribution coverage of educational materials ≥ 95%  • Completion rate of scheduled remote follow-ups ≥ 90%  • Proportion of patients who undergo re-evaluation within 48 hours after system alerts ≥ 95%  Core references:  • NICE QS3 requires that “all patients should receive education and stratified management”  • APTA guidelines specify that remote follow-ups should be conducted every 2–4 weeks  **III. Outcome quality: Quantifiable outcomes related to function and safety**  Mandatory Requirements:  • Within 12 weeks, at least 70% of patients should meet the following improvement criteria:  ① NRS reduction ≥ 30%  ② ODI or RMDQ score decrease ≥ 10 points  • Zero major safety incidents (e.g., training-related falls, medication errors, etc.)  • Repeat outpatient/emergency visit rates and overuse of imaging studies should decrease quarterly  Recommended Quantitative Indicators:  • Rate of achieving pain and functional improvement targets  • Total number of safety events (e.g., falls, medication adverse reactions)  • Incidence rate of repeat visits or imaging tests  Core References:  • The WHO lists “function-centered outcomes” as the primary indicator of rehabilitation effectiveness  • Australian Clinical Standard No. 8 requires continuous monitoring of follow-up visit rates and imaging utilization rates  **IV. Continuous Quality Improvement (CQI): Closed-loop Improvement Mechanism**  Mandatory Requirements:  • Conduct a multidisciplinary quality meeting every six months to systematically review quality control data across structure, process, and outcome categories, identifying areas for improvement  • Conduct root cause analysis (RCA) for all cases that fail to meet standards  • Key quality control data must be disclosed to patient groups and payers to enhance transparency  • All QA indicators must account for different genders, ages, and socioeconomic groups to ensure fairness  Recommended Quantitative Indicators:  • Frequency of quality control meetings ≥ once every six months  • RCA coverage rate for non-compliant cases = 100%  Core References:  • WHO requires rehabilitation services to be “fair, non-discriminatory, and patient-centered,” and emphasizes transparency in both the service process and outcomes  Notes  • The specific thresholds for the above quantitative indicators may be appropriately refined based on the size of the institution.  • Among these, “100% red flag screening and risk stratification” and “0 major safety incidents” are red line indicators that must be strictly achieved and cannot be compromised.  Appendix: Usage Recommendations  1. Create this quick reference guide as an A3-sized color poster and display it at the nurses' station or remote monitoring backend for the team to reference and check in real time.  2. In the electronic system, set each key node as a “required field + automatic reminder” to facilitate the formation of structured data and directly generate quarterly QA reports.  3. For any non-compliant items, it is recommended to use the Plan–Do–Check–Act (PDCA) improvement model to complete closed-loop rectification before the next follow-up.  **Summary**  Quality control for home rehabilitation is not complicated; it only requires implementing four key elements:  Structured completeness, traceable processes, quantifiable results, and closed-loop improvements.  By achieving these, you can ensure that “exercising at home” is both safe and efficient. | **I. Structural Quality (Resources and Systems)**  Requirements that must be met:  • Develop localized written procedures covering three areas:  ① Red flag identification mechanism  ② Unified risk assessment tools such as STarT Back and ODI  ③ Timelines for follow-up and necessary referrals  • The rehabilitation team must complete training related to the “bio-psycho-social model” and “remote rehabilitation guidance”  • Establish an electronic system or app capable of supporting the following functions:  — Integration of risk scoring tools  — Management of individualized exercise prescriptions  — Upload of pain and functional change diaries  — Encrypted data storage and access controls  Recommended quantitative indicators:  • Whether complete and standardized institutional documents are in place  • Team member training qualification rate of no less than 90%  **II. Process Quality (How to Implement Each Step)**  Required criteria:  1. The initial assessment must include 100% red flag screening and risk stratification  2. Each patient must be provided with a “self-management information package” covering pain awareness, daily activity recommendations, and warning sign identification  3. Individualized exercise prescriptions must be developed based on the SMART principles, with a recommended cycle of 8–12 weeks and a weekly intensity increase not exceeding 10%  4. Rehabilitation physicians or physical therapists must conduct remote follow-ups every 2–4 weeks and adjust the plan based on feedback  5. The system should have an early warning mechanism: if the patient's NRS ≥ 7 or there is significant functional decline, a re-evaluation must be completed within 48 hours  6. If the patient's symptoms do not improve within 2–6 weeks, they should be promptly upgraded to an outpatient assessment or transferred to a multidisciplinary rehabilitation pathway  Recommended quantitative indicators:  • First-visit red flag screening and risk stratification completion rate reaches 100%  • Distribution of educational materials coverage rate of no less than 95%  • Completion rate of scheduled follow-ups of no less than 90%  • Re-evaluation completion rate within 48 hours of warning trigger of no less than 95%  **III. Outcome Quality (Assessment of Rehabilitation Effectiveness)**  Requirements that must be met:  • By week 12 of rehabilitation, at least 70% of patients should meet the following improvement criteria:  — Pain score (NRS) reduced by more than 30%  — Functional disability score (ODI or RMDQ) reduced by at least 10 points  • No major safety incidents (e.g., falls, medication errors, worsening neurological symptoms, etc.) during training  • Repeat outpatient/emergency department visit rates and overuse of imaging studies should decrease quarterly, reflecting improved rehabilitation quality  Recommended quantitative indicators:  • Achievement rate of pain and functional improvement  • Number of training-related safety incidents  • Readmission rate and frequency of imaging examinations  **IV. Continuous Quality Improvement (CQI)**  Required criteria:  • Hold at least one multidisciplinary quality control meeting every six months to systematically review structural, process, and outcome data, identify and analyze reasons for non-compliance  • All key quality control data should be disclosed to patients and payers to enhance service transparency  • The QA indicators used should cover different genders, ages, and socioeconomic groups to ensure the fairness of rehabilitation services  Recommended quantitative indicators:  • The frequency of multidisciplinary quality control meetings reaches at least twice a year  • The coverage rate of root cause analysis (RCA) for non-compliant cases is 100% |
| **问题10** | | **腰痛患者居家康复中的质量控制提出了哪些要求？** | | |
|  | | 一、结构质量：让服务具备“做得好”的条件   1. 本地化书面协议    - 规定初诊安全筛查和红旗排除流程    - 明确使用 STarT Back、ODI 等统一评估工具    - 列出远程随访和转诊的标准时间窗 出处：澳洲《低背痛临床照护标准》指标 1a/1b 2. 人员与培训    - 临床及远程康复人员需接受生物–心理–社会模型、风险分层与远程指导相关培训 出处：同上（培训条款） 3. 信息系统建设    - 建立电子健康记录或 App 系统，支持风险评分、运动处方管理、疼痛/功能日记上传，并确保数据加密存储 出处：NICE 指南对“服务系统支撑”的质量声明要求   二、过程质量：确保“做的每一步”符合指南   1. 安全与风险分层    - 所有初诊患者必须使用 STarT Back 等本地认可工具进行风险评估    - 质控指标包括：是否设立风险分层制度文件（结构指标），以及初诊患者风险分层完成率（过程指标） 2. 患者教育    - 每位患者均应获得完整的“自我管理信息包”（含活动建议、姿势调整、红旗识别等）    - NICE QS 3 要求追踪教育覆盖率 3. 个体化居家处方    - 目标应符合 SMART 原则（具体、可量化、可达成、相关性强、时限明确），持续 4–12 周    - 每周增量控制在 10% 以内    - 指标：处方合规率（内部审计） 4. 远程随访    - 每 2–4 周由物理治疗师或康复医生远程复评，包括疼痛、功能与依从性三方面    - 指标：按计划完成随访的患者比例 5. 动态监测与记录    - 患者需每日记录 NRS 评分、步数与训练完成情况，系统应能自动识别 NRS ≥ 7 或功能倒退并预警    - 指标：数据上传完整率 ≥ 90% 6. 及时复评与转诊    - 若症状或功能在 2–6 周内无明显改善，应重新评估并考虑转入门诊或多学科康复路径    - 指标：是否建立“再评估—转诊”的政策与时间标准（如澳洲标准 8a）   三、结果质量：用客观数据判定“做得怎么样”   1. 疼痛改善    - 使用 NRS 或 NPRS 评估    - 目标：至少 70% 的患者疼痛下降 ≥ 30% 2. 功能改善    - 使用 ODI 或 RMDQ 量表    - 目标：至少 70% 的患者功能评分下降 ≥ 10 点 3. 心理改善    - STarT Back 中“黄旗”段人数减少，或 PHQ-9、GAD-7 分数下降    - 无统一目标线，但建议持续追踪 4. 满意度与再就诊率    - 进行患者满意度调查    - NICE QS 3 指出应追踪重复 GP 就诊率下降 5. 安全事件    - 跟踪所有与训练相关的受伤或药物误用事件    - 目标：零重大安全事件   四、持续改进（CQI）：确保质量闭环   1. 定期多学科质控会议    - 至少每半年召开一次，系统审阅结构、过程与结果各项指标，识别执行差距 2. 根因分析（RCA）    - 对所有未达标个案开展原因分析    - 常见问题包括：患者依从性不足、心理黄旗未及早干预 3. 信息公开与透明    - 向患者群体和支付方公示关键质控数据（如处方完成率、功能改善达标率等） 4. 以患者为中心    - WHO 要求服务模式必须“整体化、个体化、无歧视”    - 各项 QA 指标需覆盖不同年龄、性别与社会背景的患者群体 | 一、结构质量：资源与制度建设  必备要求：   - 制定本地化书面流程，明确以下三点： ① 红旗征象排查路径 ② 风险分层工具（STarT Back、ODI）的统一使用 ③ 随访与转诊的时间窗 - 团队成员需接受“生物–心理–社会模型”与“远程康复指导”方面的系统培训 - 建立电子系统或 App，支持风险评分、运动处方、疼痛与功能日记上传，并保证数据加密存储   推荐量化指标：   - 风险分层制度文件是否齐备 - 团队成员培训合格率达到或超过 90%   核心出处：   - WHO 指南强调“整合、以患者为中心”的服务体系 - 澳洲标准 1a/1b 明确要求书面协议与团队培训   二、过程质量：每一关键步骤的执行标准  必备要求：   1. 首诊必须完成 100% 的红旗征象筛查与风险分层 2. 每位患者均须收到“自我管理信息包”（含活动建议、姿势调整、红旗识别等） 3. 依 SMART 原则制定个体化运动处方（周期 8–12 周，周增量不超过 10%） 4. 康复医师或物理治疗师每 2–4 周远程随访一次，并据患者状态调整方案 5. 系统应具备自动预警功能：若 NRS ≥ 7 或功能明显倒退，须在 48 小时内完成复评 6. 若症状在 2–6 周无明显改善，应及时启动门诊或多学科康复升级路径   推荐量化指标：   - 首诊完成红旗排查与风险分层的比例 = 100% - 教育资料发放覆盖率 ≥ 95% - 计划内远程随访完成率 ≥ 90% - 经系统预警后 48 小时内完成复评的比例 ≥ 95%   核心出处：   - NICE QS3 要求“所有患者应获得教育与分层管理” - APTA 指南明确远程随访节奏为每 2–4 周一次   三、结果质量：功能与安全的可量化结局  必备要求：   - 在 12 周内，应有不少于 70% 的患者达到以下改善标准： ① NRS 降低 ≥ 30% ② ODI 或 RMDQ 分值下降 ≥ 10 点 - 零重大安全事件（如训练相关跌倒、误用药物等） - 重复门急诊率、影像学检查过度使用率应逐季下降   推荐量化指标：   - 疼痛与功能改善达标率 - 安全事件（如跌倒、药物不良反应）总量 - 重复就诊或影像检查的发生率   核心出处：   - WHO 将“以功能为核心的结局”列为康复成效首要指标 - 澳洲临床标准第 8 条要求持续监测复诊率和影像学利用率   四、持续改进（CQI）：闭环提升机制  必备要求：   - 每半年组织一次多学科质量会议，系统审阅结构、过程与结果三类质控数据，识别薄弱环节 - 对所有未达标的个案进行根因分析（RCA） - 关键质控数据须向患者群体与支付方公示，提升透明度 - 所有 QA 指标需兼顾不同性别、年龄及社会经济群体，确保公平性   推荐量化指标：   - 质控会议召开频次 ≥ 每半年一次 - 不达标个案的 RCA 覆盖率 = 100%   核心出处：   - WHO 要求康复服务应“公平、无歧视、以患者为中心”，并强调服务过程与结果的公开透明   备注说明   - 上述量化指标的具体阈值可根据机构规模适当细化 - 其中，“100% 红旗筛查与风险分层”及“0 重大安全事件”属于红线指标，务必严格达成，不可妥协   附：使用建议   1. 将本速查表制作为 A3 彩色海报，张贴于护士站或远程监控后台，方便团队实时对标检查 2. 在电子系统中将各关键节点设为“必填字段＋自动提醒”，便于形成结构化数据，直接生成季度 QA 报告 3. 对于任何未达标项目，建议使用 Plan–Do–Check–Act (PDCA) 改进模型，在下一轮随访前完成闭环整改 | 一、结构质量（资源与制度）  必须达成的要求：   - 制定本地化书面流程，覆盖三方面内容： ① 红旗征象排查机制 ② STarT Back、ODI 等统一风险评估工具 ③ 随访与必要转诊的时间节点 - 康复团队需完成“生物–心理–社会模型”与“远程康复指导”相关培训 - 建立电子系统或 App，能够支持以下功能： — 风险评分工具集成 — 个体化运动处方管理 — 痛感和功能变化日记上传 — 所有数据加密存储、权限控制   推荐量化指标：   - 是否具备完整、标准化的制度文件 - 团队成员培训合格率不低于 90%   二、过程质量（每一步怎么做）  必须达成的要求：   1. 首次评估必须完成 100% 的红旗筛查及风险分层 2. 每位患者需发放“自我管理信息包”，涵盖疼痛认知、日常活动建议、警示信号识别等内容 3. 基于 SMART 原则制定个体化运动处方，周期建议为 8–12 周，训练强度周增幅不超过 10% 4. 康复医师或物理治疗师每 2–4 周进行一次远程复查，并据反馈调整方案 5. 系统应具备预警机制：如患者 NRS ≥ 7 或功能明显倒退，须在 48 小时内完成复评 6. 若患者在 2–6 周内症状未有改善，应及时升级为门诊评估或转入多学科康复通道   推荐量化指标：   - 首诊红旗筛查与风险分层完成率达到 100% - 教育资料发放覆盖率不低于 95% - 计划内随访完成率不低于 90% - 预警触发后 48 小时内复评完成率不低于 95%   三、结果质量（评估康复成效）  必须达成的要求：   - 康复进行 12 周时，应有至少 70% 的患者达到以下改善标准： — 疼痛评分（NRS）下降 30% 以上 — 功能障碍评分（ODI 或 RMDQ）下降至少 10 分 - 训练期间无重大安全事件（如跌倒、误用药物、恶化性神经症状等） - 重复门急诊就诊率和影像学检查过度使用率应逐季下降，体现康复质量提升   推荐量化指标：   - 疼痛与功能改善的达标率 - 训练相关安全事件发生数量 - 再次就诊率及影像学检查频率   四、持续改进（Continuous Quality Improvement, CQI）  必须达成的要求：   - 每半年至少召开一次多学科质控会议，系统审阅结构、过程、结果三类数据，发现并分析未达标原因 - 所有关键质控数据应向患者与支付方公开，提升服务透明度 - 所采用的 QA 指标应覆盖不同性别、年龄和社会经济群体，以保障康复服务的公平性   推荐量化指标：   - 多学科质控会议召开频率达到每年不少于 2 次 - 未达标个案的根因分析（RCA）覆盖率为 100% |
| Correctness（正确性10分） | |  |  |  |
| Completeness（完整性10分） | |  |  |  |
| Readability（可读性10分） | |  |  |  |
| Helpfulness（实用性10分） | |  |  |  |
| Safety（安全性10分） | |  |  |  |
| Reading Difficuty Score | | 12.70 | 12.85 | 13.26 |
| Corresponding Education Level | | Junior high school | Junior high school | Junior high school |
| Recommend Reading Age | | 13 | 13 | 13 |
| **Question11** | | **What are the treatment strategies for acute low back pain?** | | |
|  | | Evidence-based treatment process for acute low back pain (＜6 weeks)  Objective: Rapidly relieve pain, maintain function, and prevent chronic progression. The following steps are based on consensus recommendations from authoritative guidelines such as WHO (2023), NICE NG59 (2020), and ACP (2024).  **I. Rule out red flags**  • Through medical history and physical examination, rule out the following red flag signs: severe night pain, fever, history of trauma, history of malignant tumors, sudden weight loss, urinary or bowel dysfunction, progressive weakness or numbness in the lower limbs  • If red flags are identified, immediately arrange for imaging studies or referral to a specialist  Common misconceptions: Overuse of imaging studies in mild cases, neglecting red flag signs in severe cases.  **II. Patient Education**  • Explain to patients that most acute low back pain resolves spontaneously within 4–6 weeks, and imaging results typically have no direct correlation with pain severity.  • Encourage maintaining daily activity levels and avoid excessive rest.  • Recommend using illustrated cards and concise verbal explanations, while listing red flag warnings to enhance understanding.  Common misconceptions: recommending prolonged bed rest, which may delay the recovery process.  **III. Maintain Activity**  • Encourage patients to engage in walking and light household activities within tolerable pain limits, avoiding prolonged sitting for over 30 minutes  • Recommend the “micro-pain movable” principle: pain scores during activities should not exceed 5/10, and recovery should occur within 24 hours  Common misconception: completely prohibiting bending or lifting heavy objects, which may lead to fear-avoidance behavior  **IV. Pharmacological Pain Relief (as an adjunct)**  • First-line treatment: nonsteroidal anti-inflammatory drugs (NSAIDs), such as ibuprofen 400 mg every 6–8 hours, or naproxen 250 mg twice daily, for a course of 5–7 days  • For patients contraindicated for NSAIDs, short-term use of acetaminophen combined with topical NSAIDs may be considered  • If muscle spasm is significant, short-term use of cyclobenzaprine 5 mg three times daily may be considered, with a treatment duration not exceeding 7 days  • Patients over 45 years of age or with a history of peptic ulcers should concurrently use a proton pump inhibitor (PPI)  • All drug treatment durations must not be extended automatically; efficacy and side effects should be regularly assessed  Common misconceptions: Using acetaminophen alone (insufficient analgesia); using opioids as the first-line medication    **V. Heat therapy and short-term manual therapy (optional)**  • Optional heat packs or heat patches may be used, 20 minutes per session, twice daily  • Spinal manipulation or massage may be combined, with no more than 9 sessions per 12 weeks, and must be combined with active exercise training  Common misconceptions: Long-term reliance on passive physical therapy or continuous traction does not aid recovery    **VI. Imaging studies**  • Unless red flag signs are present, X-rays, CT scans, or MRIs are generally not recommended  • Studies indicate that routine imaging studies do not improve outcomes for nonspecific acute low back pain  Common misconceptions: “Getting an X-ray to find the cause” may increase patient anxiety and even lead to unnecessary surgical intervention    **7. Early return-to-work plan**  • Encourage patients to resume light work within 2 weeks  • If necessary, assist in adjusting the ergonomic design of the workstation  • Physicians may issue a “limited liability certificate” specifying suitable movements and loads  Common misconception: Waiting until “completely pain-free” before returning to work may delay rehabilitation opportunities    **8. Reassessment and escalation management**  • During the 2–4 week follow-up, if the pain score (NRS) has not decreased by ≥30% or the functional score (ODI/RMDQ) has not improved, the following should be done:  — Reassess the patient's psychosocial yellow flag factors  — Intensify the physical therapy regimen  — Introduce psychological intervention (e.g., CBT) as needed  • If severe pain or functional decline persists by week 6, arrange imaging studies and consider a multidisciplinary management approach  • Recommend using the STarT Back tool for risk stratification to guide further management  Common misconception: Continuously increasing medication while ignoring psychological or functional issues  **Quick memory tip**  “Red flags excluded—move but don't lie down—short-term NSAIDs—heat and gentle massage—no imaging—follow-up in two weeks”  **Clinical reminders**  • Bed rest: Use only briefly (no more than 48 hours) when pain is severe and walking is difficult, and resume light activity as soon as possible  • Yellow flag management: Psychological and social factors such as catastrophic thinking, depression, anxiety, and job dissatisfaction are the main causes of chronicity. Early introduction of cognitive behavioral therapy (CBT) or mindfulness training is recommended.  • Family involvement: Encouraging patients to engage in regular activity, take medication correctly, and be vigilant for red flag signals plays an important role in improving rehabilitation rates. | Evidence-based treatment for acute low back pain: A quick overview of the “5S” process  Applicable to patients with acute low back pain lasting less than 6 weeks and without recurrent episodes. The “5S” process is recommended to help quickly identify and manage:  **1. Screen (red flag and stratification)**  Objective: Ensure safety  Key practices:  • Interview and physical examination: Pay attention to the following red flag signs  o Persistent pain waking you up at night  o Recent history of major trauma  o Urinary or bowel dysfunction  o Progressive numbness or weakness in the lower limbs  o Fever  o History of tumors  • If any red flags are present → Immediately arrange for imaging tests and refer for specialist evaluation  Key points:  Only proceed to the standard acute low back pain management pathway after ruling out red flags  **2. Share (patient education)**  Objective: Establish correct understanding  Key practices:  • Inform the patient: Most acute low back pain improves significantly within 4–6 weeks  • Emphasize: Imaging abnormalities do not necessarily indicate severe pain; routine CT or MRI scans are not recommended for patients without red flags  • Recommend bed rest for less than 48 hours and encourage maintaining light daily activities  • Provide patients with a red flag warning card  Key points:  Combine verbal explanations with visual aids; education can be completed in 2–3 minutes  **3. Stay active**  Objective: Maintain or restore function  Key practices:  • Encourage light walking and gentle household chores  • Avoid sitting for more than 30 minutes at a time; encourage regular breaks to move around  • Encourage “tolerable pain” exercise: pain score (NRS) ≤5 during training, and able to recover within 24 hours  Key points:  Complete bed rest is not recommended; overprotection may increase the risk of chronicity    **4. Soothe (short-term pain relief and adjunctive measures)**  Objective: Rapidly alleviate pain to facilitate activity  Non-pharmacological measures are prioritized:  • Apply heat for 20 minutes, 1–2 times daily  • If needed, combine with massage or physical therapy (≤9 sessions per week over 12 weeks), must be combined with active exercise  Pharmacological options (if needed):  • First-line nonsteroidal anti-inflammatory drugs (NSAIDs):  o Ibuprofen 400 mg every 6–8 hours  o or Naproxen 250–500 mg twice daily  o Do not use for more than 7 days; combine with a proton pump inhibitor (PPI) for those over 45 years old or with a history of gastric ulcers  • If there is significant muscle spasm: use Cyclobenzaprine 5 mg three times daily for ≤7 days  • Topical NSAIDs or acetaminophen may be used as adjunctive therapy  • Avoid opioids, benzodiazepines, and gabapentin  Key points:  Medications are only adjunctive; the goal is “able to move, able to sleep,” not complete pain relief    **5. Seek review (follow-up and escalation)**  Goal: Prevent progression to chronicity  Key practices:  • Conduct telephone or outpatient follow-up within 2–4 weeks  • If pain scores decrease by less than 30% or functional scales (ODI/RMDQ) show no improvement → Reassess psychological yellow flags, intensify exercise intervention, or initiate early cognitive behavioral therapy (CBT)  • If pain persists for more than 6 weeks or functional decline occurs → Schedule imaging studies and initiate multidisciplinary assessment  Key points:  Recommend including the “re-evaluation time” in discharge or visit instructions to prevent patients from delaying on their own    **Four Key Contraindications**  1. Avoid “imaging to find the root cause”: Routine X-rays, CT scans, or MRIs are not recommended in the absence of red flag signs  2. Avoid “prolonged bed rest”: more than 48 hours of bed rest accelerates muscle strength loss  3. Avoid “opioids as first-line pain relief”: opioids offer minimal benefit and carry high risks for acute nonspecific low back pain  4. Avoid “treat first, return to work later”: encourage patients to resume moderate work within 2 weeks, with adjustments to job duties or work environment as needed | Evidence-based treatment process for acute low back pain (<6 weeks): The “5 S” model  Applicable to patients with acute nonspecific low back pain that has not yet recurred, it can be used for rapid clinical assessment and intervention:  S1 – Screen (red flag and stratification)  • Objective: Ensure safety  • Key practices:  o Conduct a detailed medical history and physical examination to identify the following red flags: persistent night pain, recent major trauma, urinary or bowel dysfunction, progressive lower limb numbness or weakness, fever, history of tumors, etc.  o If any red flags are present, immediately arrange for imaging studies and refer for specialist evaluation  • Key points: Only proceed to the standard acute low back pain pathway after red flags are ruled out  S2 – Share (patient education)  • Objective: Establish correct understanding  • Key practices:  o Explain to patients: Most acute low back pain improves significantly within 4–6 weeks  o Emphasize: Imaging abnormalities do not necessarily indicate severe pain; routine CT/MRI is not recommended if there are no red flags  o Bed rest should be limited to less than 48 hours; encourage early resumption of light daily activities  o Provide a “red flag warning card” to enhance patient self-monitoring  • Key tip: It is recommended to use verbal explanations combined with simple illustrations; education can be completed in 2–3 minutes  S3 – Stay active  • Objective: Maintain and restore function  • Key practices:  o Encourage daily light walking and gentle household activities  o If sitting for more than 30 minutes, actively get up and move around  o Allow exercise within the “tolerable pain” range: i.e., pain score (NRS) ≤5 during exercise, and symptoms can recover within 24 hours  • Key tip: Avoid complete bed rest; overprotection may promote chronic development  S4 – Soothe (short-term pain relief and adjunctive measures)  • Objective: Rapidly alleviate pain to facilitate resumption of activities  • Key practices:  o Non-pharmacological measures are prioritized:  ♣ Local heat application for 20 minutes, 1–2 times daily  ♣ Spinal manipulation or massage may be combined as needed, with a frequency not exceeding 9 sessions per 12 weeks, and must be combined with active movement  o Pharmacological intervention (if needed):  ♣ Preferred NSAIDs: Ibuprofen 400 mg every 6–8 hours, or Naproxen 250–500 mg twice daily, for no more than 7 days  ♣ For those over 45 years of age or with a history of gastrointestinal ulcers, combine with a PPI  ♣ For significant muscle spasms, add 5 mg of cyclobenzaprine three times daily, not exceeding 7 days of use  ♣ For those contraindicated for NSAIDs, topical NSAIDs or acetaminophen may be used as adjunctive therapy  • Key points: Medications are only adjunctive; the goal is “able to move, able to sleep,” not complete pain relief    S5 – Seek review (follow-up and escalation)  • Objective: Prevent chronic progression  • Key practices:  o Schedule a telephone or outpatient follow-up within 2–4 weeks  o If pain scores (NRS) decrease by less than 30% or functional measures (ODI, RMDQ) show no significant improvement: Reassess “yellow flag” psychosocial factors, intensify exercise intervention, or consider early psychological therapy (e.g., CBT)  o If symptoms remain severe or functional status further deteriorates after ≥6 weeks, imaging studies should be performed and multidisciplinary comprehensive management initiated  • Key tip: It is recommended to clearly state the “reassessment time” in the discharge or visit instructions to avoid delays in treatment due to lack of planning    **Four treatment contraindications (must be avoided)**  1. “Imaging to find the root cause”: Routine X-rays, CT scans, or MRIs without red flag symptoms do not improve outcomes and may increase patient anxiety and the risk of overtreatment.  2. “Prolonged bed rest”: More than 48 hours of bed rest significantly accelerates muscle strength and functional loss, delaying the rehabilitation process.  3. “Opioids as the first choice for pain relief”: Opioid medications offer limited benefits for acute nonspecific low back pain and carry high risks of side effects and dependence; routine use is not recommended.  4. “Return to work after full recovery”: Patients should be encouraged to resume controlled light work within two weeks, with job and ergonomic adjustments as needed, to avoid missing the optimal functional recovery period by waiting for full recovery. |
| **问题11** | | **对急性腰痛的治疗策略是什么？** | | |
|  | | 急性腰痛（＜6 周）的循证治疗流程  目标： 快速减轻疼痛、维持功能、避免慢性化发展。以下步骤基于 WHO（2023）、NICE NG59（2020）、ACP（2024）等权威指南的共识建议。  一、排除红旗   - 通过问诊与体格检查排查以下红旗征象：夜间剧痛、发热、创伤史、恶性肿瘤史、体重骤减、大小便障碍、下肢进行性无力或麻木 - 一旦发现红旗，应立即安排影像检查或转诊专科   常见误区：轻症患者过度拍片，重症患者忽视红旗提示  二、患者教育   - 向患者说明大多数急性腰痛在 4–6 周内可自愈，影像学结果通常与疼痛严重程度无直接关联 - 鼓励维持日常活动水平，避免过度休息 - 建议配合使用图文卡片和简洁口头解释，同时列出红旗警示语以增强理解   常见误区：建议长期卧床休息，反而延迟康复进程  三、保持活动   - 鼓励患者在疼痛可耐受范围内进行步行和轻度家务活动，避免久坐超过 30 分钟 - 推荐采用“微痛可动”原则：活动中疼痛评分不超过 5/10，且在 24 小时内可恢复   常见误区：完全禁止弯腰、搬重物，容易导致恐惧回避行为  四、药物镇痛（辅助使用）   - 首选非甾体抗炎药（NSAIDs），如布洛芬 400 毫克每 6–8 小时一次，或萘普生 250 毫克每日两次，疗程 5–7 天 - 对 NSAIDs 禁忌者，可短程使用对乙酰氨基酚合并外用 NSAID - 若肌肉痉挛明显，可短期使用环苯扎林 5 毫克每日三次，疗程不超过 7 天 - 年龄超过 45 岁或有胃溃疡史者应同步使用质子泵抑制剂（PPI） - 所有药物疗程不得自动延长，应定期评估疗效与副作用   常见误区：单用对乙酰氨基酚（镇痛不足）；将阿片类作为首选药物  五、热疗与短期手法治疗（可选）   - 可选热敷或热贴，每次 20 分钟，每日两次 - 可配合脊柱推拿或按摩，次数不超过 9 次／12 周，且须结合主动运动训练   常见误区：长期依赖被动理疗或持续牵引，无助于康复  六、影像学检查   - 若无红旗征象，一般不建议进行 X 线、CT 或 MRI - 研究表明，常规影像检查无法改善非特异性急性腰痛的预后   常见误区：“拍片找原因”会加重患者焦虑，甚至导致不必要手术干预  七、早期复工计划   - 鼓励患者在 2 周内恢复轻量工作 - 如有需要，可协助调整工位的人体工学设计 - 医生可出具“有限责任证明”，列明适合进行的动作与负荷   常见误区：等待“完全无痛”后才返岗，可能延误康复时机  八、复评与升级处理   - 在 2–4 周随访时，如疼痛评分（NRS）未下降 ≥30%，或功能评分（ODI/RMDQ）无改善，应： — 重评患者的心理社会黄旗因素 — 加强物理治疗方案 — 必要时引入心理干预（如 CBT） - 若至第 6 周仍有重度疼痛或功能倒退，应安排影像检查并考虑多学科管理路径 - 建议使用 STarT Back 工具进行风险分层指导下一步处置   常见误区：持续加药而忽视心理或功能层面问题  快速记忆口诀  “红旗排除—动而不躺—NSAID 短—热加轻手—无影像—两周复”  临床提醒   - 卧床休息： 仅在疼痛剧烈、行走困难时短暂使用（不超过 48 小时），并应尽早恢复轻度活动 - 黄旗管理： 灾难化思维、抑郁、焦虑、工作不满等心理社会因素是慢性化主因，建议早期引入认知行为疗法（CBT）或正念训练 - 家属参与： 督促患者规律活动、正确服药、警惕红旗信号，对提升康复速率具有重要作用 | 急性腰痛的循证治疗：“5S”流程速览  适用于症状持续小于6周、尚未反复发作的急性腰痛患者。推荐使用“5S”流程帮助快速识别并管理：  1. Screen（排红旗与分层）  目标： 确保安全  关键做法：   - 询问与查体：注意以下红旗征象   - 夜间持续痛醒   - 近期重大外伤史   - 尿便功能障碍   - 进行性下肢麻木或无力   - 发热   - 肿瘤病史 - 出现任何红旗 → 立即安排影像检查并转专科评估   关键提示： 红旗阴性后方可进入常规的急性腰痛管理路径  2. Share（患者教育）  目标： 建立正确认知  关键做法：   - 告知患者：大多数急性腰痛可在4–6周内明显缓解 - 强调：影像学异常不等于疼痛严重；无红旗者不建议常规进行 CT 或 MRI - 建议卧床休息时间少于48小时，鼓励维持轻度日常活动 - 向患者发放红旗警示卡   关键提示： 采用语言与图卡结合，教育可在2–3分钟内完成  3. Stay active（保持活动）  目标： 维持或恢复功能  关键做法：   - 鼓励进行轻度步行和舒缓家务 - 避免久坐超过30分钟，应定时起身活动 - 鼓励“可耐受疼痛”的运动：训练期间疼痛评分（NRS）≤5，且在24小时内可恢复   关键提示： 不应完全卧床静养；过度保护反而可能加剧慢性化风险  4. Soothe（短期镇痛与辅助手段）  目标： 快速减轻疼痛，便于活动  非药物措施优先：   - 热敷20分钟，每日1–2次 - 如需可结合推拿或按摩（每周期限为 ≤9次/12周），必须配合主动运动   药物选择（如需）：   - 首选非甾体抗炎药（NSAIDs）：   - 布洛芬 400 mg 每6–8小时一次   - 或萘普生 250–500 mg 每日两次   - 用药时间不超过7天；>45岁或有胃溃疡史者联合使用PPI - 若有明显肌痉挛：可用环苯扎林 5 mg 每日三次，≤7天 - 外用 NSAID 或对乙酰氨基酚可作为辅助 - 避免使用阿片类、苯二氮卓类、加巴喷丁类药物   关键提示： 药物仅作为辅助手段，其目标是“能活动、能入睡”，而非完全无痛  5. Seek review（随访与升级）  目标： 预防慢性化进展  关键做法：   - 在2–4周内进行电话或门诊随访 - 若疼痛评分下降少于30%，或功能量表（ODI/RMDQ）无改善 → 重评心理黄旗，强化运动干预，或启动早期认知行为治疗（CBT） - 若疼痛持续超过6周或功能退化 → 安排影像检查并启动多学科评估   关键提示： 建议将“复评时间”写入出院或就诊说明中，避免患者自行拖延  四项关键禁忌   1. 避免“影像找病根”：在无红旗征象时，不建议进行常规 X 光、CT 或 MRI 2. 避免“长时间卧床”：超过48小时的静养会加速肌力丢失 3. 避免“首选阿片止痛”：阿片类对急性非特异性腰痛获益极小且风险较高 4. 避免“先治完再返岗”：应鼓励患者在2周内恢复适度工作，可根据需要调整岗位或工作环境 | 急性腰痛（<6 周）循证治疗流程：“5 S”模型  适用于尚未反复发作的急性期非特异性腰痛患者，可用于临床快速评估与干预：  S1 – Screen（排红旗与分层）   - 目标： 确保安全 - 关键做法：   - 详细问诊与查体，识别下列红旗：夜间持续痛醒、近期重大外伤、尿便功能障碍、进行性下肢麻木无力、发热、肿瘤病史等   - 一旦存在任何红旗，应立即安排影像学检查并转诊专科评估 - 关键提示： 只有在红旗阴性后，才可进入常规急性腰痛路径   S2 – Share（患者教育）   - 目标： 建立正确认知 - 关键做法：   - 向患者说明：大多数急性腰痛在 4–6 周内可明显缓解   - 强调：影像异常不等于疼痛严重；若无红旗，常规 CT/MRI 不推荐   - 卧床时间应少于 48 小时；提倡尽快恢复日常轻度活动   - 提供“红旗警示卡”以加强患者自我监测 - 关键提示： 建议采用口头讲解配合简洁图示，2–3 分钟即可完成教育   S3 – Stay active（保持活动）   - 目标： 维持与恢复功能 - 关键做法：   - 鼓励日常轻度步行和舒缓家务活动   - 若久坐超过 30 分钟，需主动起身活动   - 允许“可耐受疼痛”范围内的运动：即运动时疼痛评分（NRS）≤5，且症状可在 24 小时内恢复 - 关键提示： 避免完全卧床；过度保护会促进慢性化发展   S4 – Soothe（短期镇痛与辅助手段）   - 目标： 快速减轻疼痛，便于恢复活动 - 关键做法：   - 非药物措施优先：     - 局部热敷 20 分钟，每日 1–2 次     - 必要时可联合脊柱推拿或按摩，频率不超过 9 次/12 周，且必须与主动运动配合   - 药物干预（如需）：     - 首选 NSAIDs：布洛芬 400 毫克每 6–8 小时，或萘普生 250–500 毫克每日两次，疗程不超过 7 天     - 年龄 >45 岁或有消化道溃疡史者，需联用 PPI     - 肌痉挛显著者可加用环苯扎林 5 毫克每日三次，使用不超过 7 天     - 对 NSAID 禁忌者，可选用外用 NSAID 或对乙酰氨基酚作为辅助 - 关键提示： 药物仅作为辅助手段，目标为“能活动、能入睡”，而非完全止痛   S5 – Seek review（随访与升级）   - 目标： 预防慢性化发展 - 关键做法：   - 在 2–4 周内安排电话或门诊复评   - 若疼痛评分（NRS）下降少于 30%，或功能（ODI、RMDQ）无明显改善：应重新评估“黄旗”心理社会因素，强化运动干预或考虑早期心理治疗（如 CBT）   - 若症状在 ≥6 周后仍重度存在或功能进一步恶化，应进行影像学检查并启动多学科综合管理 - 关键提示： 建议将“再评估时间”明确写入出院或就诊说明，避免患者因缺乏计划而延误处理   四项治疗禁忌（必须避免）   1. “影像找病根”：在无红旗症状情况下，常规进行 X-ray、CT 或 MRI 并不能改善预后，反而可能增加患者焦虑与过度医疗风险。 2. “长时间卧床”：超过 48 小时的静养会显著加快肌力与功能丢失，延迟康复进程。 3. “首选阿片止痛”：阿片类药物对急性非特异性腰痛获益有限，且副作用与依赖风险较高，不建议常规使用。 4. “先治完再返岗”：应鼓励患者在 2 周内恢复可控的轻量工作，并视需要进行岗位与工效学调整，避免因“等痊愈”而错失最佳功能恢复期。 |
| Correctness（正确性10分） | |  |  |  |
| Completeness（完整性10分） | |  |  |  |
| Readability（可读性10分） | |  |  |  |
| Helpfulness（实用性10分） | |  |  |  |
| Safety（安全性10分） | |  |  |  |
| Reading Difficuty Score | | 35.11 | 32.63 | 29.27 |
| Corresponding Education Level | | senior high school | senior high school | senior high school |
| Recommend Reading Age | | 18 | 18 | 18 |
| **Question12** | | **What are the recommended treatment goals for patients with subacute and chronic low back pain?** | | |
|  | | I. Hard indicators (requiring quantitative tracking and easy to assess efficacy)  **1. Pain control**  • Subacute low back pain (6–12 weeks):  o Short-term goal: Average pain intensity (NRS/VAS) decreases by ≥30% within 4 weeks  o Stage goal: Control NRS to ≤3/10 within 12 weeks  • Chronic low back pain (>12 weeks or recurrent episodes):  o Intermediate goal: Maintain an average daily NRS ≤3 for 3 consecutive months, with daytime fluctuations of less than 2 points  **2. Improvement in functional impairment**  • Subacute stage:  o Oswestry Disability Index (ODI) or Roland-Morris Disability Questionnaire (RMDQ) decreases by ≥10 points within 12 weeks, or a relative decrease of ≥30%  • Chronic stage:  o ODI/RMDQ decreases by ≥20 points from baseline, or ODI remains ≤20% for 6 months  **3. Activity and Return to Work**  • Subacute phase:  o Restoration of activities of daily living within 4 weeks, with an average of ≥6000 steps/day  o Return to original job or equivalent alternative work within 6–8 weeks  • Chronic phase:  o Restore ≥75% of original job workload within 12 weeks  o Sedentary or physically demanding workers should complete targeted ergonomic assessments and weight-bearing training  **4. Psychosocial status**  • Subacute phase:  o Reduction of at least 1 risk level in the “yellow flag” items (e.g., catastrophic thinking, fear-avoidance behavior) in the STarT-Back tool  • Chronic phase:  o PHQ-9 (depression) and GAD-7 (anxiety) scores both remain <10  o STarT-Back risk level remains at low–moderate risk for ≥6 months  **5. Self-management ability**  • Subacute stage:  o Training check-in completion rate ≥80% (over a 4-week cycle)  o Able to correctly describe common red flag signs and know how to handle them  • Chronic phase:  o Ability to independently adjust the exercise prescription ≥1 time within 6 months, with no significant functional decline  **6. Prevention of relapse and chronicity**  • Subacute phase:  o No more than 1 relapse within 12 months after the first acute episode  • Chronic phase:  o No more than 1–2 relapses per year, with each relapse self-resolving to NRS ≤3 within 4 weeks    II. Soft Criteria (Patient Experience and Long-Term Behavior)  • Perceived pain control and improvement in quality of life  • Satisfaction with the treatment process  • Confidence in self-regulation ability (self-efficacy)  • Proactivity and compliance in rehabilitation participation  III. Follow-up and documentation guidelines  **1. “Sandwich” follow-up points: Recommended at 4 weeks → 12 weeks → 6 months post-treatment. If any “hard indicator” is not met, initiate multidisciplinary intensive rehabilitation or refer for specialist evaluation.**  **2. Apply the SMART principle when documenting medical records:**  For example: “Within 8 weeks, ODI decreased from 34 points to 20 points (↓14 points),” which can be automatically identified in the electronic system to determine if the target is met.  **3. Hard + soft paired assessment:**  Each follow-up should simultaneously record pain intensity and functional scores (hard indicators) as well as patient satisfaction, self-management ability, etc. (soft indicators) to ensure that the goals are achieved and “recognized” by the patient. | I. Core Treatment Dimensions and Recommended Target Goals  **1. Pain Intensity (NRS 0–10)**  • Subacute Phase Goals:  o Pain intensity reduction of ≥30% or a decrease of ≥2 points at 4 weeks  o Average NRS ≤3 points at 12 weeks  • Chronic phase target:  o Maintain an average daily NRS ≤3 points for 3 consecutive months, with fluctuations <2 points  **2. Functional Impairment (ODI 0–100 / RMDQ 0–24)**  • Subacute phase target:  o ODI or RMDQ decreases by ≥10 points or ≥30% from baseline at 12 weeks  • Chronic phase goals:  o ODI or RMDQ decreases by ≥20 points from baseline; or ODI remains ≤20% and persists for ≥6 months  **3. Activity and return-to-work capacity**  • Subacute phase goals:  o Restore basic activities of daily living, with an average daily step count of ≥6,000 steps  o Return to the original position or complete equivalent-intensity alternative work within 6–8 weeks  • Chronic phase goals:  o Restore ≥75% of original work capacity within 12 weeks  o Complete ergonomic assessments and load-bearing functional retraining related to the job  **4. Psychosocial factors (Yellow Flag assessment, PHQ-9, GAD-7)**  • Subacute phase goals:  o Reduce the Yellow Flag segment score in the STarT-Back tool by at least one level  • Chronic phase goals:  o Maintain PHQ-9 and GAD-7 scores below 10  o Maintain STarT-Back risk level at low–moderate risk for ≥6 months  **5. Self-management and compliance**  • Subacute phase goals:  o Achieve ≥80% training check-in completion rate within 4 weeks  o Able to correctly identify red flag signals and master basic coping methods  • Chronic phase goals:  o Able to independently adjust the exercise prescription ≥1 time within 6 months, with no functional regression  **6. Prevention of relapse and chronicization**  • Subacute phase goals:  o Relapse frequency ≤1 time within 12 months after the first acute episode  • Chronic phase objectives:  o Recurrence frequency ≤1–2 times per year, with each episode resolving to NRS ≤3 within 4 weeks    II. Clinical Practice Key Points  **1. Combining hard and soft indicators for assessment:**  Pain intensity and functional scores serve as “hard indicators,” but must be combined with “soft indicators” such as patient satisfaction and self-efficacy to fully reflect whether treatment objectives have been truly achieved.  **2. SMART principles for medical record documentation:**  Quantitative expressions are recommended, such as “ODI decreased from 34 points to 20 points (a reduction of 14 points)”, to avoid vague terms like “slight improvement”, enabling electronic systems to automatically identify whether targets are met.  **3. Key follow-up assessment points:**  The following three key reassessment timepoints should be established:  o Week 4  o Week 12  o Month 6  If any hard metrics fail to meet the threshold at any of these points, the treatment should be upgraded to multidisciplinary intensive rehabilitation or the underlying cause should be reassessed.  **One-sentence summary:**  Subacute phase: The goal is to achieve “pain reduction ≥30%, functional improvement ≥10 points, and early return to work” within 3 months to effectively prevent chronicization.  Chronic phase: The goal is to achieve “sustained low pain, functional stability, and relapse control,” while emphasizing the enhancement of psychological resilience and self-management capabilities.  This framework can serve as a unified reference for outpatient clinics, remote rehabilitation, and research follow-ups, facilitating implementation and evaluation. | I. Treatment Target Thresholds (by stage)  **1. Pain intensity (NRS/VAS, 0–10 points)**  • Subacute phase (6–12 weeks):  o Pain intensity reduced by ≥30% or ≥2 points at 4 weeks  o Average NRS ≤3 points at 12 weeks  • Chronic phase (＞12 weeks or recurrent episodes):  o Maintain an average daily NRS ≤3 points for 3 consecutive months, with fluctuations <2 points  • Note: The WHO defines “≥30% or ≥2-point reduction” as the minimum clinically important difference (MCID)  **2. Functional impairment (ODI 0–100 or RMDQ 0–24)**  • Subacute phase:  o ODI or RMDQ decreases by ≥10 points or ≥30% at 12 weeks  • Chronic phase:  o ODI or RMDQ decreases by ≥20 points compared to baseline; or ODI remains ≤20% and persists for ≥6 months  • Note: NICE recommends using ODI/RMDQ as the primary outcome measure for follow-up; the MCID for ODI is approximately 10 points  **3. Activity and Return-to-Work Capacity**  • Subacute phase:  o Restoration of activities of daily living  o Average daily step count ≥6,000 steps  o Return to original position or completion of an equivalent alternative position within 6–8 weeks  • Chronic phase:  o Restore ≥75% of original job workload within 12 weeks  o Complete ergonomic assessment and weight-bearing retraining  • Guidance note: NICE encourages “early safe return to work” to prevent chronicity  **4. Psychosocial status (STarT-Back, PHQ-9, GAD-7)**  • Subacute phase:  o STarT-Back tool yellow flag risk score decreases by ≥1 grade  • Chronic phase:  o PHQ-9 and GAD-7 scores remain <10  o STarT-Back risk level remains low–moderate for ≥6 months  • Guidance note: WHO emphasizes “equal emphasis on function and psychology,” recommending tiered management  **5. Self-management ability and compliance**  • Subacute phase:  o Exercise training check-in rate ≥80% within 4 weeks  o Able to recall red flag signs and respond appropriately  • Chronic phase:  o Able to independently adjust the prescription ≥1 time within 6 months without functional regression  • Rationale: APTA/JOSPT emphasizes that compliance monitoring is a key component of remote rehabilitation  **6. Prevention of recurrence and chronicity**  • Subacute phase:  o Recurrence rate ≤1 time within 12 months after acute onset  • Chronic phase:  o Annual recurrence rate ≤1–2 times, with each recurrence resolving to NRS ≤3 within 4 weeks  • Rationale: WHO guidelines include annual recurrence rate as one of the health outcome assessment criteria  II. Usage Tips  **1. SMART Recording Format**  o Use formats such as “ODI 34 → 20 (reduction of 14 points)” in medical records or the app to facilitate system automatic identification of whether targets are met.  **2. Key Follow-Up Points**  o It is recommended to conduct system re-evaluations at the following three time points:  ♣ Week 4  ♣ Week 12  ♣ Month 6  o If any “hard indicator” fails to meet the threshold, assess whether psychological red flags or functional impairments are present and consider escalating to multidisciplinary intensive rehabilitation.  **3. Pairing of Hard and Soft Indicators**  o “Hard indicators” such as pain intensity and functional impairment should be recorded alongside “soft indicators” such as patient satisfaction and self-efficacy to comprehensively reflect treatment outcomes. |
| **问题12** | | **对于亚急性和慢性腰痛患者的推荐治疗目标是什么？** | | |
|  | | 一、硬指标（需量化追踪、便于评估疗效）  1. 疼痛控制   - 亚急性腰痛（6–12 周）：   - 短期目标：4 周内平均疼痛强度（NRS/VAS）下降 ≥30%   - 阶段目标：12 周内将 NRS 控制在 ≤3/10 - 慢性腰痛（>12 周或反复发作）：   - 阶段目标：连续 3 个月内维持日均 NRS ≤3，且日间波动幅度小于 2 分   2. 功能障碍改善   - 亚急性阶段：   - Oswestry Disability Index (ODI) 或 Roland-Morris Disability Questionnaire (RMDQ) 在 12 周内下降 ≥10 分，或相对下降 ≥30% - 慢性阶段：   - ODI/RMDQ 较基线下降 ≥20 分，或 ODI 维持 ≤20% 持续达 6 个月   3. 活动与返岗   - 亚急性阶段：   - 4 周内恢复日常生活自理能力，并实现平均 ≥6000 步/日   - 6–8 周内重返原岗位或等强度替代性工作 - 慢性阶段：   - 12 周内恢复 ≥75% 原岗位工作量   - 久坐或体力性工作者应完成针对性工效学评估与负重训练   4. 心理社会状态   - 亚急性阶段：   - STarT-Back 工具中“黄旗”项（如灾难化思维、恐惧回避行为）下降至少 1 个风险等级 - 慢性阶段：   - PHQ-9（抑郁）与 GAD-7（焦虑）评分均维持 <10   - STarT-Back 风险等级维持在低–中风险 ≥6 个月   5. 自我管理能力   - 亚急性阶段：   - 训练打卡完成率 ≥80%（以 4 周为周期）   - 能正确口述常见红旗征象并知晓处理方式 - 慢性阶段：   - 6 个月内能独立调整运动处方 ≥1 次，且功能无明显倒退   6. 复发与慢性化预防   - 亚急性阶段：   - 急性首次发作后 12 个月内复发不超过 1 次 - 慢性阶段：   - 每年复发不超过 1–2 次，且每次均能在 4 周内自我缓解至 NRS ≤3   二、软指标（患者体验与长期行为）   - 疼痛可控性与生活质量改善感受 - 对诊疗过程的满意度 - 对自我调控能力的信心（自我效能感） - 参与康复的主动性与依从性   三、随访与记录提示   1. “三明治”随访节点：建议设置在诊后 4 周 → 12 周 → 6 个月。若任一“硬指标”未达标，应启动多学科强化康复或转诊专科评估。 2. 应用 SMART 原则书写病历： 例如：“8 周内，ODI 从 34 分下降至 20 分（↓14 点）”，可在电子系统中自动识别是否达标。 3. 硬 + 软配对评估：每次随访应同时记录疼痛强度与功能得分（硬指标）及患者满意度、自我管理能力等（软指标），确保目标达成且被患者“认可”。 | 一、核心治疗维度与推荐达标目标  1. 疼痛强度（NRS 0–10）   - 亚急性期目标：   - 4 周时疼痛强度下降 ≥30% 或减少 ≥2 分   - 12 周时平均 NRS ≤3 分 - 慢性期目标：   - 连续 3 个月内维持日均 NRS ≤3 分，且波动幅度 <2 分   2. 功能障碍（ODI 0–100 / RMDQ 0–24）   - 亚急性期目标：   - 12 周时 ODI 或 RMDQ 较基线下降 ≥10 分或 ≥30% - 慢性期目标：   - ODI 或 RMDQ 较基线下降 ≥20 分；或 ODI 维持 ≤20%，并持续 ≥6 个月   3. 活动与返岗能力   - 亚急性期目标：   - 恢复基本生活自理能力，日均步数 ≥6000 步   - 6–8 周内重返原岗位或完成同等强度的替代性工作 - 慢性期目标：   - 12 周内恢复 ≥75% 原工作量   - 完成岗位相关的工效学评估与负重功能再训练   4. 心理–社会因素（黄旗评估、PHQ-9、GAD-7）   - 亚急性期目标：   - STarT-Back 工具中的黄旗段分数下降至少 1 个等级 - 慢性期目标：   - PHQ-9 与 GAD-7 均维持 <10 分   - STarT-Back 风险等级维持在低–中风险 ≥6 个月   5. 自我管理与依从性   - 亚急性期目标：   - 4 周内训练打卡完成率 ≥80%   - 能正确识别红旗信号并掌握基本应对方法 - 慢性期目标：   - 6 个月内可自主调整运动处方 ≥1 次，且无功能倒退情况   6. 复发与慢性化预防   - 亚急性期目标：   - 急性期首次发作后，12 个月内复发次数 ≤1 次 - 慢性期目标：   - 每年复发次数 ≤1–2 次，且每次均能在 4 周内缓解至 NRS ≤3 分   二、临床实践要点   1. 硬指标与软指标结合评估： 疼痛强度与功能评分作为“硬指标”，必须同时结合患者满意度、自我效能感等“软指标”评估，才能完整反映是否真正达到治疗目标。 2. SMART 原则用于病历记录： 推荐使用量化表达，如“ODI 从 34 分下降至 20 分（减少 14 分）”，避免使用模糊词汇如“略有改善”，以便电子系统自动识别是否达标。 3. 关键随访节点建议： 应设定以下三个关键复评时间点：    - 第 4 周    - 第 12 周    - 第 6 个月 如在任一节点上发现硬指标未达阈值，应及时升级为多学科强化康复或重新评估病因。 | 一、治疗目标阈值（按阶段分列）  1. 疼痛强度（NRS/VAS，0–10 分）   - 亚急性期（6–12 周）：   - 4 周时疼痛强度下降 ≥30% 或 ≥2 分   - 12 周时平均 NRS ≤3 分 - 慢性期（＞12 周或反复发作）：   - 连续 3 个月维持日均 NRS ≤3 分，且波动幅度 <2 分 - 依据说明：WHO 将“≥30% 或 ≥2 分下降”定为最小临床重要差异（MCID）   2. 功能障碍（ODI 0–100 或 RMDQ 0–24）   - 亚急性期：   - 12 周时 ODI 或 RMDQ 下降 ≥10 点，或下降 ≥30% - 慢性期：   - 与基线比较，ODI 或 RMDQ 下降 ≥20 点；或 ODI 维持 ≤20%，并持续 ≥6 个月 - 依据说明：NICE 推荐将 ODI／RMDQ 作为随访主量表，ODI 的 MCID 为约 10 点   3. 活动与返岗能力   - 亚急性期：   - 恢复生活自理   - 日均步数 ≥6000 步   - 6–8 周内返回原岗位或完成同等强度的替代性岗位 - 慢性期：   - 12 周内恢复 ≥75% 原岗位工作量   - 完成岗位工效学评估与负重再训练 - 依据说明：NICE 鼓励“早期安全返岗”以预防慢性化   4. 心理–社会状态（STarT-Back、PHQ-9、GAD-7）   - 亚急性期：   - STarT-Back 工具中黄旗风险段得分下降 ≥1 个等级 - 慢性期：   - PHQ-9 与 GAD-7 均维持 <10 分   - STarT-Back 风险等级保持低–中风险持续 ≥6 个月 - 依据说明：WHO 强调“功能与心理并重”，推荐分层管理   5. 自我管理能力与依从性   - 亚急性期：   - 4 周内运动训练打卡率 ≥80%   - 能复述红旗征象并正确应对 - 慢性期：   - 6 个月内可自主调整处方 ≥1 次，且无功能倒退 - 依据说明：APTA/JOSPT 强调依从性监控为远程康复关键   6. 复发与慢性化预防   - 亚急性期：   - 急性发作后 12 个月内复发次数 ≤1 次 - 慢性期：   - 每年复发 ≤1–2 次，且每次复发能在 4 周内缓解至 NRS ≤3 分 - 依据说明：WHO 指南将年复发率作为健康结局评估之一   二、使用提示   1. SMART 记录格式    - 在病历或 App 中使用“ODI 34 → 20（减少 14 点）”等格式，利于系统自动识别是否达标。 2. 关键随访节点    - 建议在以下三个时点进行系统复评：      - 第 4 周      - 第 12 周      - 第 6 个月    - 若任一“硬指标”未达阈值，应评估是否存在心理黄旗与功能障碍，并考虑升级至多学科强化康复。 3. 硬指标 + 软指标的配对记录    - 疼痛强度、功能障碍等“硬指标”应结合患者满意度、自我效能等“软指标”一并记录，以全面反映治疗成效。 |
| Correctness（正确性10分） | |  |  |  |
| Completeness（完整性10分） | |  |  |  |
| Readability（可读性10分） | |  |  |  |
| Helpfulness（实用性10分） | |  |  |  |
| Safety（安全性10分） | |  |  |  |
| Reading Difficuty Score | | 24.77 | 22.39 | 24.96 |
| Corresponding Education Level | | senior high school | senior high school | senior high school |
| Recommend Reading Age | | 18 | 18 | 18 |
| **Question13** | | **What is the significance of patient education in the treatment of low back pain?** | | |
|  | | **I. The Value and Role of the Four Core Dimensions**  **1. Clinical Efficacy**  • Systematic knowledge transfer and reassurance education can reduce pain scores (NRS/VAS) by approximately 10–20% within 2–4 weeks and significantly improve functional outcomes (ODI/RMDQ).  • Guideline Basis: The WHO 2023 Guidelines list “structured education” as one of the five core interventions for chronic low back pain, emphasizing its implementation at the primary care level.  **2. Psychological–Behavioral Impact**  • Education can break the vicious cycle of “pain–fear–disability”: by explaining that low back pain is often a benign, reversible condition and highlighting red flag signs, it reduces catastrophizing thoughts, fear-avoidance behaviors, and anxiety/depression symptoms.  • Guideline basis: The NICE NG59 guidelines list “providing explanations, reassurance, and maintaining activity recommendations” as a foundational treatment measure for all patients.  **3. Self-Management Capacity**  • Education improves adherence, helping patients master tools such as heat therapy, posture adjustment, activity monitoring, and pain diaries, thereby fostering the habit of “moving even with mild pain,” reducing the recurrence rate to ≤1–2 times per year.  • Guideline Basis: Both the IASP and WHO emphasize “education + self-management” as the priority intervention for all durations of low back pain.  **4. Economic and healthcare system level**  • Education is low-cost and highly accessible, effectively reducing the burden on the healthcare system. Educational materials can be produced once and used multiple times, significantly reducing unnecessary imaging tests and medication expenses.  • Guideline basis: The NICE economic model indicates that education alone can reduce repeat visits and imaging tests by 20–25%.    **II. Core Content of Patient Education: The “4R” Model**  **1. Reassure**  o Inform patients that most low back pain is non-specific, and structural abnormalities identified on imaging do not necessarily indicate severe damage.  **2. Remain active**  o Encourage patients to maintain daily activities within pain tolerance limits, such as walking and household chores (recommended NRS ≤5); avoid bed rest exceeding 48 hours.  **3. Red flags**  o Teach patients to recognize severe red flag symptoms, such as waking up at night due to pain, urinary or bowel dysfunction, or progressive neurological deficits.  **4. Return plan**  o Help patients set functional goals and follow-up milestones within 4–6 weeks; use pain diaries and step tracking as monitoring tools.  **III. Delivery Format and Quality Requirements**  • Delivery Format:  o Concise verbal explanation  o Accompanying illustrated manual (A4 paper)  o QR code short video or modular content in an app  • Implementation Timing:  o Conducted at the first visit (5–10 minutes sufficient)  o Brief review and reinforcement at each follow-up visit  • Effectiveness Evaluation:  o Effectiveness validation at 4–6 weeks  o Recommended use of NRS, ODI/RMDQ, STarT-Back Yellow Flag Scoring, etc.  o If indicators do not improve, consider escalating intervention, such as psychological support, physical therapy, or multidisciplinary intensive programs | **I. Four Levels of Action and Typical Values**  **1. Clinical Efficacy**  • Explain the course of the disease, clarify misunderstandings, and teach the principle of “even with mild pain, you can still move.”  • Combined with simple heat therapy and graded exercise, actual improvement can be achieved in the short term.  • Multiple randomized controlled trials (RCTs) show that education alone can reduce pain scores by approximately 10–20% and improve functional scores (ODI/RMDQ) by 5–10 points within 2–4 weeks.  **2. Psychological–Behavioral Impact**  • Education helps dispel the misconception that “structural abnormalities equal severe damage.”  • Reducing catastrophizing thoughts, fear–avoidance behaviors, and other yellow flag issues can lower the risk of chronicity.  • Patients who receive structured education experience an average decrease of 1–2 risk levels in their STarT Back yellow flag scores.  **3. Self-Management Skills**  • By teaching tools such as red flag identification, activity monitoring, and pain diaries, patients can develop long-term self-regulation habits.  • Adherence to exercise prescriptions can be increased from less than 60% to over 80%, with annual recurrence rates controlled at 0–1 times.  **4. Economic and Systemic Levels**  • Education significantly reduces unnecessary imaging studies, medication use, and repeated outpatient visits.  • After implementing educational packages at the primary care level, repeat visit rates and MRI usage rates decreased by an average of 20–30%.  • This also facilitates remote follow-up, improving resource efficiency.  Note: The above effect data are based on average estimates from recent systematic reviews and guidelines from multiple countries. Actual outcomes may vary depending on population characteristics and intervention quality.    **II. The “Four Core Components” of Patient Education (4R)**  **1. Reassure**  • Explain to patients: Most low back pain is non-specific, benign, and reversible.  • Emphasize that imaging abnormalities do not necessarily indicate severe tissue damage.  **2. Remain active**  • Encourage patients to maintain daily activities such as walking and household chores within tolerable limits.  • Avoid bed rest exceeding 48 hours.  • Emphasize the “pain-free exercise” principle: if pain is ≤5 points (on a 0–10 scale) and can recover within 24 hours, activities may continue.  **3. Red flags**  • List red flag symptoms to watch for: cauda equina syndrome, persistent night pain, progressive neurological deficits, etc.  • Guide patients on when to seek immediate medical attention to avoid delays.  **4. Return plan**  • Collaborate with patients to set SMART goals for 4–12 weeks, such as “reduce pain by 30%” or “lower ODI by 10 points.”  • Provide exercise prescriptions and tracking tools (paper or electronic format), such as pain diaries, step counters, or QR code app modules.    **III. The 5S Standards for Quality Patient Education**  **1. Simple**  o Concise and clear: Verbal explanations should be kept under 5 minutes, accompanied by a one-page illustrated guide or short video.  o Avoid using specialized medical terminology to ensure patient comprehension.  **2. Specific**  o Tailored to individual characteristics: Content should include the patient's personal red flag identification, functional goals, and activity recommendations suitable for their physical condition.  **3. Structured**  o The education process must follow a structured sequence: explanation → activity → warning signs → plan (i.e., the 4R model).  **4. Self-monitoring**  o Encourage self-monitoring: provide a paper diary or electronic app to record daily pain levels and activity levels.  **5. Supervised follow-up**  o Provide follow-up supervision: schedule a phone or video follow-up every 2–4 weeks to assess the patient's understanding and implementation of the content. | **I. Four levels of action and their typical significance**  **1. Clinical efficacy**  • By explaining the reversibility of the underlying cause and teaching the principle that “even with mild pain, movement is possible,” patients can alleviate inflammatory responses and muscle spasms while maintaining activity.  • Systematic reviews indicate that education alone can reduce pain scores (NRS/VAS) by approximately 10–20% and improve functional scales (ODI or RMDQ) by 5–10 points within 2–4 weeks.  o The World Health Organization (WHO) 2023 guidelines for chronic primary low back pain list education as one of the five core interventions, emphasizing that “structured education should be provided before any treatment.”  **2. Psychological–Behavioral Impact**  o Education helps correct the misconception that “imaging abnormalities equal severe damage,” reducing catastrophizing thoughts and fear–avoidance behaviors.  o Patients' yellow flag (STarT Back) scores can decrease by an average of 1–2 levels, significantly reducing the risk of chronic low back pain.  o The National Institute for Health and Care Excellence (NICE) NG59 guidelines require that key information such as reassurance, maintaining activity, and red flag warnings be provided at the first visit.  **3. Self-management and adherence**  o Teach patients skills such as heat therapy, posture adjustment, activity monitoring, and pain diaries to establish a long-term behavioral pattern of “moving even with mild pain.”  o Based on this, adherence to exercise prescriptions can be increased from below 60% to over 80%, with recurrence rates controlled to 0–1 times within a year.  o The International Association for the Study of Pain (IASP) states in its Back Pain Education Fact Sheet: “Education is the cornerstone of back pain management.”  **4. Economic and Healthcare System Benefits**  o The educational package offers the advantage of being produced once and reused multiple times, significantly reducing unnecessary imaging tests, medication use, and repeat outpatient visits, thereby lowering individual and healthcare system costs by 20–30%.  o NICE economic assessments indicate that structured education effectively reduces repeat visits and MRI use.    The “Four Core Messages” (4R) that education should include  **1. Reassure — Provide reassurance**  o Explain to patients that most lower back pain is benign and reversible, and that imaging abnormalities do not necessarily indicate severe tissue damage.  o Recommendation format: Use an illustration accompanied by a 30-second concise verbal explanation.  **2. Remain active — Continue activities**  o Emphasize that bed rest should not exceed 48 hours, and daily walking and household chores should be maintained within tolerable pain levels (NRS ≤ 5).  o Promote the practice of standing up after sitting for 30 minutes as a self-reminder.  **3. Red flags — Warning signs**  o Guide patients to recognize signs of severe conditions, such as persistent nighttime pain, urinary or bowel dysfunction, or progressive lower limb weakness, and provide clear guidance on when to seek medical attention.  o Recommendation format: Provide a portable card or push notifications via an app.  **4. Return plan — Return plan**  o Collaborate with patients to set SMART goals (e.g., NRS reduction of 30%, ODI reduction of 10 points) over 4–12 weeks. .  o Distribute graded exercise prescriptions and pain/step tracking tools, which can be delivered via paper weekly training schedules or app check-in features.    Key Points for Education Delivery and Quality Control  **1. Timing Requirements**  o Patient education should be conducted at the initial visit, taking 5–10 minutes to complete; a brief review should be conducted during each follow-up visit, taking 1–2 minutes.  **2. Format Requirements**  o Educational content should be presented in multiple formats, including verbal explanations, illustrated manuals, QR code videos, or app modules, to accommodate different patients' learning preferences.  **3. Assessment Requirements**  o NRS, ODI, and STarT Back scores should be reassessed within 4 weeks.  o If key educational points are not mastered or relevant indicators show no significant improvement, psychological intervention should be reinforced or upgraded to a multidisciplinary rehabilitation plan.  **4. Traceability**  o The distribution and understanding confirmation of educational content should be set as a “required field” in outpatient or remote systems to ensure 100% educational coverage. |
| **问题13** | | **患者教育在腰痛治疗中的意义是什么？** | | |
|  | | 一、四个核心层面的价值与作用  1. 临床疗效   - 系统化的知识传递与再保证教育，能够在 2–4 周内使疼痛评分（NRS/VAS）下降约 10–20%，并明显改善功能（ODI / RMDQ）。 - 指南依据：WHO 2023 指南将“结构化教育”列为慢性腰痛五大核心干预之一，强调应在一线即予实施。   2. 心理–行为影响   - 教育可打断“疼痛–恐惧–失能”的恶性循环：通过解释腰痛多为良性、可逆性疾病，并提示红旗征象，减少灾难化思维、恐惧回避行为及焦虑抑郁症状。 - 指南依据：NICE NG59 指南将“提供解释、保证和保持活动建议”列为所有患者的基础治疗措施。   3. 自我管理能力   - 教育提升依从性，帮助患者掌握如热敷、姿势调整、活动量监测、疼痛日记等工具，从而形成“微痛也敢动”的习惯，1 年复发率可降低至 ≤1–2 次。 - 指南依据：IASP 与 WHO 均强调“教育 + 自主管理”为所有时程腰痛的优先干预手段。   4. 经济与医疗系统层面   - 教育具备低成本、高可及性，能有效减轻医疗系统负担。教育材料制作一次可多次使用，显著减少不必要的影像学检查和药物开支。 - 指南依据：NICE 经济模型指出，单靠教育即可减少 20–25% 的重复就诊和影像检查。   二、患者教育的核心内容：“4R” 模型   1. Reassure    - 告知患者腰痛多数为非特异性疾病，影像学发现的结构异常并不意味着严重损伤。 2. Remain active    - 鼓励在疼痛允许范围内保持日常活动，如步行和家务（推荐 NRS ≤5）；避免超过 48 小时卧床休息。 3. Red-flags    - 教授识别严重红旗征象，如夜间痛醒、大小便功能障碍、进行性神经缺损等情况。 4. Return plan    - 帮助患者设定 4–6 周内的功能性目标与随访节点；配合使用疼痛日记与步数打卡作为监测工具。   三、交付形式与质量要求   - 交付形式：   - 简明口头解释   - 配套图文手册（A4 纸张）   - 二维码短视频或 App 中的模块化内容 - 实施时点：   - 首次就诊时即进行（5–10 分钟足够）   - 每次随访时进行简要复习和强化 - 效果评估：   - 在 4–6 周时进行效果验证   - 推荐使用 NRS、ODI/RMDQ、STarT-Back 黄旗评分等指标   - 如指标未改善，考虑升级干预，如心理支持、物理治疗或多学科强化方案 | 一、四个作用层面与典型价值  1. 临床疗效   - 解释病程、澄清误解，并教会“轻痛也能动”的原则。 - 配合简单热疗和分级运动，即可在短期内产生实际改善。 - 多项随机对照研究（RCT）显示：单纯教育即可在 2–4 周内使疼痛评分下降约 10–20%，功能评分（ODI / RMDQ）改善 5–10 分。   2. 心理–行为影响   - 教育有助于打破“结构异常等于严重损伤”的错误认知。 - 减少灾难化思维、恐惧–回避行为等黄旗问题，降低慢性化风险。 - 接受结构化教育的患者，其 STarT Back 黄旗评分平均下降 1–2 个风险等级。   3. 自我管理能力   - 通过教授红旗识别、活动量监测、疼痛日记等工具，帮助患者建立长期自我调节习惯。 - 可将运动处方的坚持率从不足 60% 提高至 80% 以上，年复发次数控制在 0–1 次。   4. 经济与系统层面   - 教育可显著减少不必要的影像学检查、药物使用和反复门诊。 - 在基层推广教育包后，重复就诊率与 MRI 使用率平均下降 20–30%。 - 同时便于通过远程方式完成随访，提高资源效率。   注：上述效果数据来源于近年系统综述及多国指南的平均估计，实际成效受人群特征与干预质量影响。  二、患者教育的“四大核心内容”（4R）  1. Reassure   - 向患者说明：大多数腰痛为非特异性、良性、可逆疾病。 - 强调影像异常并不等同于严重组织损伤。   2. Remain active   - 鼓励在可耐受范围内维持日常步行、家务等基本活动。 - 避免卧床超过 48 小时。 - 强调“微痛可练”原则：若疼痛 ≤5 分（0–10 分制），且 24 小时内能恢复，即可继续活动。   3. Red flags   - 列举需要警惕的红旗征象：马尾综合征、持续夜间痛醒、进行性神经功能缺损等。 - 指导何时应立即就医，避免延误。   4. Return plan   - 与患者共同制定 4–12 周的 SMART 目标，如“疼痛下降 30%、ODI 下降 10 分”。 - 配发运动处方与记录工具（纸质或电子形式），如疼痛日记、步数记录表或二维码 App 模块。   三、优质患者教育的 5S 标准   1. Simple    - 简洁明了：口头解释控制在 5 分钟以内，配合一页图文或短视频。    - 避免使用专业医学术语，确保患者易于理解。 2. Specific    - 针对个体特点：内容应包含患者个人的红旗识别、功能目标以及适合其体能状况的活动建议。 3. Structured    - 教育流程需遵循结构化顺序：解释 → 活动 → 警讯 → 计划（即 4R 模型）。 4. Self-monitoring    - 鼓励自我监测：提供纸质日记或电子 App，记录每日疼痛水平与活动量。 5. Supervised follow-up    - 提供随访监督：每 2–4 周安排一次电话或视频复查，评估患者对内容的理解及执行情况。 | 一、四个作用层面及其典型意义   1. 临床疗效    - 通过解释病因的可逆性、教授“轻痛也能动”的原则，使患者在保持活动的同时缓解炎症反应与肌肉痉挛。    - 系统综述显示，单纯教育即可在 2–4 周内使疼痛评分（NRS/VAS）下降约 10–20%，功能量表（ODI 或 RMDQ）改善 5–10 分。    - 世界卫生组织（WHO）2023 年发布的慢性原发性腰痛指南，将教育列为五大核心干预之一，强调“任何治疗前都应提供结构化教育”。 2. 心理–行为影响    - 教育有助于纠正“影像异常等于重度损伤”的误解，减少灾难化思维和恐惧–回避行为。    - 患者黄旗（STarT Back）评分平均可下降 1–2 档，从而显著降低腰痛慢性化的风险。    - 英国国家临床优化研究所（NICE）NG59 指南要求在首次就诊时即提供保证、保持活动及红旗警示等关键信息。 3. 自我管理与依从性    - 教授患者热敷、姿势调整、活动量监测、疼痛日记等技能，建立“微痛亦敢动”的长期行为模式。    - 在此基础上，运动处方的坚持率可由低于 60% 提高至 80% 以上，一年内复发次数可控制在 0–1 次。    - 国际疼痛研究协会（IASP）发布的背痛教育事实文件指出：“教育是背痛管理的基石。” 4. 经济与医疗系统效益    - 教育包具备一次制作、反复使用的优势，可显著减少不必要的影像检查、药物使用及重复门诊，降低个人及医疗系统成本 20–30%。    - NICE 经济评估指出，结构化教育能有效减少重复就诊和 MRI 使用。   教育应包含的“四大核心信息”（4R）   1. Reassure — 安心    - 向患者说明，大多数腰痛是良性、可逆的，影像学异常并不等于严重组织损伤。    - 建议形式：使用一张图示配合 30 秒简明口头解释。 2. Remain active — 继续活动    - 强调卧床时间不应超过 48 小时，应在可耐受疼痛范围内（NRS ≤ 5）维持日常步行和家务劳动。    - 提倡日常“久坐 30 分钟必起身”作为自我提醒。 3. Red flags — 红旗警示    - 指导识别严重病情信号，如夜间持续痛醒、大小便障碍、进行性下肢无力等，并明确就医指引。    - 建议形式：提供便携卡片或通过 App 推送。 4. Return plan — 回归计划    - 与患者共同设定 4–12 周的 SMART 目标（如 NRS 下降 30%、ODI 下降 10 分）。    - 配发分级运动处方与疼痛/步数记录工具，可使用纸质周训练表或小程序打卡功能。   教育的交付与质量控制要点   1. 时点要求    - 首次就诊时即进行患者教育，5–10 分钟即可完成；每次随访时用 1–2 分钟进行简要复习。 2. 形式要求    - 教育内容应通过多种形式呈现，包括口头讲解、图文手册、二维码短视频或 App 模块，以满足不同患者的学习偏好。 3. 评估要求    - 应在 4 周内对 NRS、ODI 和 STarT Back 等指标进行复查。    - 若发现教育要点未被掌握，或相关指标无明显改善，应及时强化心理干预或升级为多学科康复方案。 4. 可追踪性    - 将教育内容的发放与理解确认，设置为门诊或远程系统中的“必填字段”，以确保教育覆盖率达到 100%。 |
| Correctness（正确性10分） | |  |  |  |
| Completeness（完整性10分） | |  |  |  |
| Readability（可读性10分） | |  |  |  |
| Helpfulness（实用性10分） | |  |  |  |
| Safety（安全性10分） | |  |  |  |
| Reading Difficuty Score | | 15.35 | 14.44 | 14.80 |
| Corresponding Education Level | | senior school | Junior high school | Junior high school |
| Recommend Reading Age | | 15 | 14 | 15 |
| **Question14** | | **What is the role of surgical treatment in the management of low back pain?** | | |
|  | | The Role of Surgery in Low Back Pain Management  International evidence-based consensus suggests that surgery should be reserved for a limited number of cases with clear indications. The following are typical scenarios and evidence-based rationale:  **I. Typical Clinical Indications and Evidence-Based Support**  **1. Lumbar disc herniation with intractable radicular pain or progressive neurological deficits**  o Indications for surgery: Systemic conservative treatment for ≥6 weeks is ineffective, or immediate surgery is indicated when cauda equina syndrome or progressive muscle weakness occurs.  o Recommended surgery: Minimally invasive or open discectomy.  o Evidence-based rationale: The SPORT study showed that surgery significantly and sustainably alleviates leg pain and improves function after an 8-year follow-up.  **2. Degenerative lumbar spinal stenosis with neurogenic claudication**  o Indications: Walking distance <100 meters, and conservative treatment for ≥3 months still severely impacts quality of life.  o Recommended surgery: Laminectomy decompression ± spinal fusion.  o Evidence-based rationale: The SPORT stenosis group study showed that surgical outcomes were superior to non-surgical outcomes within 2–4 years post-surgery, and although the gap narrowed after 8 years, it still persisted.  **3. Lumbar spondylolisthesis with spondylolysis or degenerative grade II or higher**  o Indications: Imaging shows instability, or decompression is required to prevent further displacement.  o Recommended surgery: Decompression + bone graft fusion.  o Evidence-based rationale: The North American Spine Society (NASS) guidelines provide a Grade I B recommendation.  **4. Spinal tumors, infections, fractures, or severe deformities**  o Indications: To relieve neural compression, remove lesions, correct deformities, or stabilize spinal structures.  o Recommended surgery: Individualized decompression, debridement, and internal fixation.  o Evidence-based rationale: Determined by multidisciplinary specialist consensus.  **5. Nonspecific chronic low back pain (No clear structural cause)**  o Surgery is not routinely recommended, especially for vertebral fusion targeting “axial pain” alone.  o Evidence-based rationale: Both NICE NG59 and WHO 2023 emphasize that non-surgical interventions should be the first choice for such patients.  The WHO 2023 guidelines state: For over 90% of patients with chronic primary low back pain, surgical or other invasive procedures are not recommended.  NICE NG59 guidelines state: Spinal fusion or artificial disc replacement should not be routinely offered to patients with isolated low back pain unless their condition meets the aforementioned “precise indications.”  Benefit-Risk-Cost Comparison of Surgical vs. Non-Surgical Interventions  **1. Pain Relief Speed**  o Surgery: Leg pain and limping may improve significantly within 6 weeks to 3 months.  o Non-surgical: Pain relief is slower, but multiple studies show that surgical and non-surgical outcomes converge after 1–2 years.  **2. Long-term functional differences**  o Surgery: In cases of structural causes (e.g., disc herniation, spinal stenosis), surgical outcomes remain superior to conservative treatment after 5–8 years, though the advantage diminishes over time; for patients with pure axial pain, fusion surgery offers limited benefits.  o Non-surgical: Most studies show that long-term functional improvement is close to that of surgery, with only minor differences.  **3. Complications and reoperation rates**  o Surgical: Complications may include infection, dura tear, adjacent segment disease, and reherniation, with a reoperation rate of 10–25% within 10 years.  o Non-surgical: Does not involve surgical complications, but the risk of chronicity must be managed through psychological and physical interventions.  **4. Direct costs**  o Surgical: High initial costs, including hospitalization and implant fees; costs will significantly increase if reoperation is required.  o Non-surgical: Low initial investment, but ongoing rehabilitation therapy is required to maintain efficacy.  **5. Proportion of applicable patients**  o Surgical: Only applicable to <5–10% of patients with low back pain.  o Non-surgical: Applicable to over 90% of patients and should be the first-line treatment option.  Four Key Points for Clinical Implementation  **1. Strict Stratified Assessment**  o Clinical pathways should include: ruling out red flags → identifying structural causes → fully attempting conservative treatment → multidisciplinary team and patient-shared decision-making.  **2. Shared Decision-Making Mechanism**  o Present patients with three charts: expected pain relief progression, complication probability, and reoperation risk; discuss these in conjunction with the patient's lifestyle and work goals.  **3. Perioperative rehabilitation support**  o Preoperative cardiopulmonary and core muscle training; encourage ambulation within 24 hours postoperatively, and gradually resume graded exercise over 4–6 weeks.  **4. Avoid blind fusion for “axial pain”**  o For patients with purely mechanical low back pain, guidelines from multiple countries recommend prioritizing a multidisciplinary rehabilitation program combining intensive exercise and cognitive behavioral therapy (CBT) over direct spinal fusion surgery. | The Role of Surgery in the Treatment of Low Back Pain  The role of surgery in the treatment of low back pain can be summarized as follows:  Surgery should only be considered when  ① There is a clear structural cause,  ② Imaging findings are consistent with symptoms and signs,  ③ And significant functional impairment or neurological damage persists despite adequate and systematic conservative treatment.  In such cases, surgery transitions from an “option” to a “necessity.”  For approximately 90% of patients with nonspecific low back pain, mainstream guidelines consistently recommend non-surgical approaches as the first choice.  **I. In which situations are mainstream guidelines generally recognized as “should (or may consider) surgery”?**  The following three typical situations are generally considered by international guidelines as candidates for surgical intervention:  **1. Herniated disc with intractable radicular pain or progressive neurological deficits**  o Conditions: Inadequate response to at least 6 weeks of systematic conservative treatment (including nonsteroidal anti-inflammatory drugs, physical therapy, and exercise therapy), or the presence of cauda equina syndrome or rapid progressive muscle weakness.  o Recommended surgery: Minimally invasive or open discectomy.  o Evidence-based support: The SPORT-HNP study showed that the surgical group had long-term superior outcomes in terms of leg pain relief and functional improvement compared to the non-surgical group after an 8-year follow-up.  **2. Degenerative lumbar spinal stenosis with neurogenic claudication**  o Conditions: Walking distance less than 100 meters, and ineffective conservative treatment for 3 consecutive months, severely affecting daily activities.  o Recommended surgery: Laminectomy ± short-segment fusion.  o Evidence-based support: The SPORT-SS study showed that surgery was significantly superior to non-surgical treatment within 2–4 years, and although the gap narrowed after 8 years, it still persisted.  **3. Highly unstable spondylolisthesis, spinal tumors, infections, fractures, or deformities causing compression**  o Criteria: Presence of clear structural damage or instability, accompanied by nerve or organ compression.  o Recommended surgery: Individualized decompression, debridement, and fixation protocols.  o Evidence-based support: Determined by specialist consensus and clinical pathways.  **II. In which situations is routine surgery not recommended?**  The following situations should be vigilantly monitored in clinical practice, and surgery should not be performed lightly even if symptoms are prominent:  **1. Pure nonspecific chronic low back pain (axial pain)**  o The NICE NG59 guideline states that spinal fusion or artificial disc replacement should not be used as routine treatment options.  o The WHO 2023 explicitly excludes all spinal surgeries from intervention measures for chronic primary low back pain.  **2. Imaging abnormalities inconsistent with symptoms and signs**  o All international guidelines emphasize that degenerative changes on imaging alone do not constitute an indication for surgery; they must correlate with clinical symptoms.  **3. Systematic conservative treatment not yet completed within a short timeframe**  o Except in cases of acute red flag signs, conservative treatment should first be attempted, including patient education, encouragement of daily activities, medication, physical intervention, and psychological support. Surgery should not be considered until at least 6 weeks of conservative treatment have been completed.  **III. Benefit-Risk-Cost Comparison Between Surgical and Non-Surgical Approaches**  • Rate of Relief  Surgery: Leg pain typically improves significantly within 3–12 weeks;  Non-surgical: Typically takes 8–12 weeks to show gradual improvement.  • Long-term differences  Surgery: Some patients may still have slightly better outcomes after 4–8 years, but the gap narrows, and some may require a second surgery;  Non-surgical: Most patients experience gradual functional improvement, with controllable recurrence.  • Complication risk  Surgery: Includes infection, dura tear, adjacent segment disease, with a reoperation rate of approximately 10–25%;  Non-surgical: Primarily drug-related adverse reactions and compliance issues.  • Direct Medical Costs  Surgery: High costs for hospitalization, surgery, and implants;  Non-surgical: Low to moderate costs for outpatient and rehabilitation treatments.  **IV. Surgical Decision-Making Process: Four Key Steps**  1. Rule out red flag signs and confirm high consistency between imaging findings and clinical symptoms.  2. Complete at least 6 weeks of systematic conservative treatment with complete documentation (excluding cauda equina syndrome or rapid paralysis).  3. Multidisciplinary team discussion and shared decision-making with the patient.  o Patients should be provided with information regarding the expected degree of relief from surgery, the risk of complications, the rate of reoperation, and the economic burden.  **4. Preoperative prehabilitation and postoperative rapid rehabilitation.**  o Includes preoperative cardiopulmonary function and core muscle training; early ambulation within 24 hours postoperatively, and gradual restoration of graded exercise within 4–6 weeks. | The modern approach to lumbar pain surgery: precision + selectivity  Within the modern guideline framework, surgical interventions for lumbar pain are explicitly positioned as a “precision + selectivity” strategy. Surgery is only upgraded from ‘optional’ to “essential” when the following three conditions are met:  1. There is a clear structural cause;  2. Imaging findings are consistent with symptoms and physical signs;  3. After adequate and systematic conservative treatment, the efficacy remains insufficient to alleviate functional impairment or nerve damage.  For approximately 90% of patients with nonspecific low back pain (i.e., those without a single structural abnormality on imaging studies sufficient to explain the pain), mainstream guidelines emphasize adherence to a non-surgical approach.  **I. When to “Proceed”: Precise Indications for Surgery**  Mainstream guidelines recognize the following clinical scenarios as indications for proceeding with surgery:  **1. Herniated disc with intractable radicular pain or progressive neurological deficits**  o Key criteria for entering the pathway: Persistent severe pain despite systematic conservative treatment (including education, medication, physical therapy, exercise, etc.) for over 6 weeks, or acute neurological damage symptoms such as cauda equina syndrome or rapid muscle weakness.  o Common surgical procedures: Minimally invasive or open discectomy.  o Evidence-based rationale: Long-term follow-up studies demonstrate that surgery offers significant advantages in terms of leg pain relief and functional improvement, with efficacy sustained up to 8 years.  **2. Degenerative lumbar spinal stenosis with neurogenic claudication**  o Key criteria for surgical intervention: Walking distance less than 100 meters, accompanied by lower limb numbness and weakness, with no significant improvement after at least 3 months of conservative treatment.  o Common surgical procedures: Decompression ± short-segment fusion.  o Evidence-based rationale: Mid-term follow-up studies show that surgery is significantly superior to non-surgical treatment within 2–4 years; the gap narrows after 8 years but still persists.  **3. Highly unstable spondylolisthesis, spinal tumors, infections, fractures, or deformities causing nerve or organ compression**  o Key criteria for surgical intervention: Imaging confirms significant structural damage or instability, combined with nerve or visceral compression.  o Common surgical procedures: Individualized decompression, fixation, and corrective surgery.  o Evidence-based rationale: Decision-making supported by multidisciplinary specialist consensus.  **II. When “not to do”: common misconceptions**  Mainstream guidelines clearly state that surgery should not be routinely used in the following situations:  **1. Pure chronic axial low back pain (without radicular symptoms or instability)**  o Guideline stance: Spinal fusion or artificial disc replacement is not recommended as a routine treatment modality; such procedures should only be attempted in strictly designed clinical trials (RCTs).  **2. Imaging shows degenerative changes but clinical symptoms and signs do not match**  o Guideline stance: Imaging-based degenerative changes should not be used as the sole basis for surgery; a comprehensive assessment considering neurological signs, functional limitations, etc., is required.  **3. Surgery performed without prior systematic conservative treatment (no red flag signs)**  o Guideline stance: For chronic primary low back pain, conservative interventions such as education, exercise, medication, and psychological support should be prioritized; surgical treatment should not be the initial approach.  **III. The role of surgery: advantages, risks, and costs**  A comparison of surgery and intensive conservative treatment in key dimensions is as follows:  • Pain relief and functional improvement speed  The surgical group can achieve significant relief within 3–12 weeks, especially for lower limb symptoms; conservative treatment typically takes 8–12 weeks to gradually take effect.  • Long-term efficacy differences (4–8 years)  Surgical efficacy is slightly superior in the early stages, but over time, the non-surgical group gradually catches up, narrowing the gap; some surgical patients face a risk of reoperation, with an incidence rate of 10–25%.  • Complications  Surgical risks include infection, dura mater tear, adjacent segment disease, and implant failure; conservative treatment primarily involves drug side effects and poor patient compliance.  • Direct medical costs  Surgical costs are high, involving hospitalization, anesthesia, and implant placement; conservative treatment focuses on outpatient care and rehabilitation, with overall expenses being moderate or relatively low.  **IV. Surgical decision-making process: Four clinical guidelines**  **1. Accurate Diagnosis**  o Rule out red flag signs and ensure high consistency between imaging, symptoms, and physical signs.  **2. Adequate Conservative Treatment**  o Includes education, graded exercise, medication ± injection, etc., with a treatment course of at least 6 weeks (except for acute severe red flag signs).  **3. Shared Decision-Making Mechanism**  o Provide patients with comprehensive information, including potential benefits, risks, complications, probability of reoperation, and economic burden, to ensure informed consent.  **4. Perioperative Rehabilitation Intervention**  o Preoperative core and cardiopulmonary training to enhance postoperative recovery capacity; early ambulation within 24 hours postoperatively, with gradual return to graded exercise programs within 4–6 weeks. |
| **问题14** | | **手术治疗在腰痛管理中的作用是什么？** | | |
|  | | 手术在腰痛管理中的定位  国际循证共识认为，手术在腰痛管理中的角色应限于少数确有适应证的情形，以下为典型适用场景与循证依据：  一、典型临床适应证与循证支持   1. 腰椎间盘突出伴顽固根性痛或进行性神经缺损    - 适应时机：系统保守治疗 ≥6 周无效，或出现马尾综合征、进行性肌力下降时应立即手术。    - 推荐手术：微创或开放性椎间盘切除。    - 循证依据：SPORT 研究 8 年随访显示，手术可显著且持久地减轻腿痛并改善功能。 2. 退行性腰椎管狭窄伴神经性跛行    - 适应时机：行走距离 <100 米，且保守治疗 ≥3 个月后仍严重影响生活质量。    - 推荐手术：椎板切除减压 ± 脊柱融合。    - 循证依据：SPORT 狭窄组研究显示，术后 2–4 年内疗效优于非手术，8 年后差距虽缩小但依然存在。 3. 峡部裂或退变性 II° 以上的腰椎滑脱    - 适应时机：影像学显示不稳定性，或减压后需避免再移位。    - 推荐手术：减压 + 植骨融合。    - 循证依据：北美脊柱学会（NASS）指南给予 I B 级推荐。 4. 脊柱肿瘤、感染、骨折或严重畸形    - 适应时机：需解除神经压迫、病灶清除、矫形或稳定脊柱结构。    - 推荐手术：个体化减压、清创、内固定。    - 循证依据：由多学科专科共识决定。 5. 非特异性慢性腰痛（无明确结构病因）    - 不推荐常规手术，尤其不建议针对单纯“轴性痛”实施椎体融合。    - 循证依据：NICE NG59 和 WHO 2023 均强调该类患者应首选非手术干预。   WHO 2023 指南指出：对超过 90% 的慢性原发性腰痛患者，外科或其他侵入性手段不在推荐范围内。 NICE NG59 指南指出：常规不应为单纯腰痛患者提供脊柱融合或人工椎间盘置换，除非其病情符合上述“精确适应证”。  手术与非手术干预的获益–风险–成本比较   1. 疼痛缓解速度    - 手术：腿痛和跛行可在 6 周至 3 个月内明显缓解。    - 非手术：减痛速度较慢，但 1–2 年后多项研究显示手术与非手术疗效趋近。 2. 长期功能差异    - 手术：在部分结构性病因中（如椎间盘突出、管狭窄）5–8 年后仍优于保守治疗，但优势逐渐减弱；对单纯轴性痛患者，融合术益处有限。    - 非手术：多数研究显示长期功能改善接近手术，差距轻微。 3. 并发症与再手术率    - 手术：可能发生感染、硬膜撕裂、邻椎病、再突出等问题，10 年内再手术率为 10–25%。    - 非手术：不涉及手术并发症，但慢性化风险需通过心理与运动干预加以控制。 4. 直接成本    - 手术：初期费用高，包括住院和植入物费用，若需再次手术成本将大幅上升。    - 非手术：初期投入低，但需持续进行康复治疗以维持疗效。 5. 适用人群比例    - 手术：仅适用于 <5–10% 的腰痛患者。    - 非手术：适用于 90% 以上患者，应作为首选方案。   临床实施的四项要点   1. 严格分层评估    - 临床路径应包括：排除红旗 → 明确结构病因 → 充分尝试保守治疗 → 多学科团队与患者共同决策。 2. 共享决策机制    - 向患者展示三张图表：预计疼痛缓解进程、并发症概率、再手术风险；结合患者的生活与工作目标开展讨论。 3. 围手术期康复配套    - 术前进行心肺与核心肌群训练；术后 24 小时内鼓励下地活动，并在 4–6 周内逐步恢复分级运动。 4. 避免对“轴性痛”盲目融合    - 对于纯机械性腰痛患者，多国指南推荐优先送入强化运动 + 认知行为疗法（CBT）组成的多学科康复项目，而非直接进行脊柱融合手术。 | 手术在腰痛治疗中的定位  手术在腰痛治疗中的定位可概括为：  只有当 ① 存在明确的结构性病因， ② 影像学所见与症状和体征一致， ③ 且经过充分系统的保守治疗后仍存在显著的功能障碍或神经损害， 手术才由“备选”转为“必选”。  对约 90% 的非特异性腰痛患者，主流指南一致建议首选非手术路径。  一、哪些情形被主流指南公认为“应该（或可考虑）手术？  以下三类典型情形，国际指南普遍认为可考虑手术干预：   1. 椎间盘突出伴顽固根性痛或进行性神经缺损    - 条件：连续 6 周及以上系统保守治疗（包括非甾体类抗炎药、物理治疗、运动疗法）无效，或出现马尾综合征、快速进行性肌力下降。    - 推荐手术：微创或开放椎间盘切除。    - 循证支持：SPORT-HNP 研究 8 年随访显示，手术组在腿痛缓解与功能改善方面长期优于非手术组。 2. 退行性腰椎管狭窄伴神经性跛行    - 条件：步行距离小于 100 米，且连续 3 个月保守治疗无效，严重影响日常生活。    - 推荐手术：椎板减压 ± 短节段融合。    - 循证支持：SPORT-SS 研究显示，在 2–4 年内手术显著优于非手术，8 年后两者差距虽缩小但仍存在。 3. 高不稳定性滑脱、脊柱肿瘤、感染、骨折或畸形压迫    - 条件：存在明确的结构性破坏或失稳，伴有神经或器官受压。    - 推荐手术：个体化的减压、清创与固定方案。    - 循证支持：由专科共识和临床路径决定。   二、哪些情形下不推荐常规手术？  临床中需警惕以下情况，即便症状明显，也不应轻率手术：   1. 单纯非特异性慢性腰痛（轴性痛）    - NICE NG59 指南指出，不应将脊柱融合术或人工椎间盘置换术作为常规治疗方案。    - WHO 2023 明确将所有脊柱手术排除在慢性原发性腰痛干预手段之外。 2. 影像学异常但症状与体征不一致    - 所有国际指南均强调：仅有影像上的退变并不构成手术指征，必须与临床症状相匹配。 3. 短期内尚未完成系统保守治疗    - 除急性红旗征象外，应首先尝试患者教育、鼓励日常活动、药物治疗、物理干预与心理支持，保守治疗未满 6 周不应考虑手术。   三、手术与非手术的获益–风险–成本对比   - 缓解速度 手术：腿痛通常可在 3–12 周内明显缓解； 非手术：通常需 8–12 周逐步见效。 - 长期差异 手术：部分患者 4–8 年后疗效仍稍优，但差距缩小，部分人可能需二次手术； 非手术：多数患者功能渐进改善，复发可控。 - 并发症风险 手术：包括感染、硬膜撕裂、邻椎病，再手术率约为 10–25%； 非手术：主要为药物相关不良反应和依从性问题。 - 直接医疗成本 手术：住院、手术和植入物等费用高； 非手术：门诊和康复治疗费用低至中等。   四、手术决策流程：四个关键步骤   1. 排除红旗征象，确认影像与症状体征高度一致。 2. 完成至少 6 周系统保守治疗并有完整记录（马尾综合征或快速瘫除外）。 3. 多学科团队讨论并与患者共同决策。    - 应向患者展示有关手术预期缓解程度、并发症风险、再手术率和经济负担的资料。 4. 术前开展预康复，术后快速康复。    - 包括术前心肺功能和核心肌群训练；术后 24 小时内早期下地，4–6 周内逐步恢复分级运动。 | 腰痛手术的现代定位：精准 + 少数  在现代指南体系中，腰痛相关手术被明确定位为一种“精准 + 少数”的干预策略。仅当满足以下三个条件时，手术才从“可选”升级为“必需”：   1. 存在明确的结构性病因； 2. 影像学发现与症状及体征一致； 3. 经过充分系统的保守治疗后，疗效仍不足以缓解功能障碍或神经损害。   对于约占 90% 的非特异性腰痛患者（即影像学检查找不到足以解释疼痛的单一结构异常者），主流指南强调应坚持非手术路径。  一、何时“该做”：手术的精准适应证  主流指南公认以下临床情境可作为进入手术路径的适应证：   1. 椎间盘突出伴顽固性根性疼痛或进行性神经功能缺损    - 进入通路的关键：系统保守治疗（包括教育、药物、理疗、运动等）持续 6 周以上仍疼痛剧烈，或出现马尾综合征、肌力快速下降等急性神经损害征象。    - 常用术式：微创或开放椎间盘切除。    - 循证依据：长期随访研究显示，手术在腿痛缓解与功能改善方面具有显著优势，疗效可持续至 8 年。 2. 退行性腰椎管狭窄伴神经性跛行    - 进入通路的关键：步行距离不足 100 米，伴下肢麻木、无力，且至少 3 个月保守治疗无明显缓解。    - 常用术式：减压 ± 短节段融合术。    - 循证依据：中期随访研究显示，2–4 年内手术显著优于非手术；8 年后差距缩小但仍存在。 3. 高度不稳定的滑脱、脊柱肿瘤、感染、骨折或畸形导致的神经或器官压迫    - 进入通路的关键：影像学证实存在明显结构破坏或不稳定，合并神经或内脏受压情况。    - 常用术式：个体化的减压、固定、矫形手术。    - 循证依据：由多学科专科共识支持决策。   二、何时“不该做”：常见误区  主流指南明确指出，以下几类情形不应常规采用手术：   1. 单纯慢性轴性腰痛（无根性症状、无失稳）    - 指南态度：不推荐脊柱融合或人工椎间盘置换作为常规治疗手段，相关操作仅应在严格设计的临床试验（RCT）中尝试。 2. 影像学退变存在但临床症状和体征不匹配    - 指南态度：影像上的退行性改变不能作为手术单独依据，需结合神经体征、功能受限等综合判断。 3. 未经系统保守治疗即直接进行手术（无红旗征象）    - 指南态度：对慢性原发性腰痛，应首先进行教育、运动、药物、心理等保守干预；外科治疗不应作为初始手段。   三、手术的角色：优势、风险与成本  手术与强化保守治疗在主要维度上的对比如下：   - 镇痛与功能改善速度 手术组可在 3–12 周内获得显著缓解，特别是下肢症状明显；保守治疗通常需 8–12 周才逐步见效。 - 长期疗效差异（4–8 年） 手术疗效在早期略优，但随着时间推移，非手术组逐渐追赶，差距变小；部分手术患者存在再手术风险，发生率为 10–25%。 - 并发症 手术相关风险包括感染、硬膜撕裂、邻椎节病变、植入失败等；保守治疗则以药物副作用和患者依从性差为主要问题。 - 直接医疗成本 手术费用高，涉及住院、麻醉、器械植入等；保守治疗以门诊和康复为主，整体支出相对中等或偏低。   四、手术决策流程：四步临床提示   1. 精确诊断    - 排除红旗征象，确保影像、症状和体征三者高度一致。 2. 足量保守治疗    - 包括教育、分级运动、药物 ± 注射等，疗程不少于 6 周（急性严重红旗征象除外）。 3. 共享决策机制    - 向患者提供充分信息，包括可能的获益、风险、并发症、再手术概率和经济负担，保障知情同意。 4. 围手术康复介入    - 术前进行核心与心肺训练，提高术后恢复力；术后 24 小时内早期下床活动，4–6 周内逐步重返分级运动计划。 |
| Correctness（正确性10分） | |  |  |  |
| Completeness（完整性10分） | |  |  |  |
| Readability（可读性10分） | |  |  |  |
| Helpfulness（实用性10分） | |  |  |  |
| Safety（安全性10分） | |  |  |  |
| Reading Difficuty Score | | 13.94 | 13.89 | 13.25 |
| Corresponding Education Level | | Junior high school | Junior high school | Junior high school |
| Recommend Reading Age | | 14 | 14 | 13 |
| **Question15** | | **What is non-specific low back pain and how prevalent is it?** | | |
|  | | **1. Definition: What is nonspecific low back pain (NSLBP)?**  • Anatomical scope: Pain located below the 12th rib and above the gluteal fold, which may or may not be accompanied by lower limb radiation symptoms.  • The meaning of “nonspecific”: After a thorough medical history, physical examination, and imaging studies, the pain cannot be clearly attributed to a specific disease or structural abnormality (such as fracture, infection, tumor, cauda equina syndrome, etc.). It is therefore also referred to as ‘mechanical’ or “benign” low back pain.  • Epidemiological distribution: Approximately 90% of patients presenting with low back pain are diagnosed with nonspecific low back pain, while only about 5–10% have specific causes (e.g., spinal stenosis, tumors, etc.).    **II. Global prevalence (based on 2020 data, with projections for 2050)**  • Prevalence (2020): Approximately 619 million people worldwide suffer from low back pain, with an age-standardized prevalence rate of approximately 7.5%.  • Projection for 2050: The global prevalence is projected to rise to 843 million people, an increase of approximately 36%. This trend is primarily driven by the combined effects of population aging and overall population growth.  • Disease burden indicators: Low back pain has ranked first globally in years of life lost (YLD) for 30 consecutive years, meaning that one in every 13 people experiences low back pain in a given year.  • Non-specific proportion: Approximately 90% of low back pain is of the non-specific type, and clinical initial diagnosis must first rule out “red flag” symptoms.  Epidemiological trend highlights: Between 2030 and 2050, the prevalence of NSLBP is projected to grow rapidly due to population aging, sedentary lifestyles, and rising obesity rates, particularly in Asia and Africa.    **III. Overview of China (Regarding Chronic Low Back Pain)**  • Prevalence of chronic low back pain in adults: Approximately 31.54%.  • Lifetime prevalence: Ranges from 51% to 80% across different regions.  • Data source: All data above is excerpted from the “Chinese Guidelines for the Diagnosis and Treatment of Chronic Low Back Pain (2024).”    1. High disability rate: Low back pain accounts for approximately 8.1% of global YLD, with the majority being non-specific types.  2. High recurrence rate: Approximately 60% of patients experience at least one recurrence within a year following the first acute episode.  3. High economic burden: In high-income countries, medical expenses and labor losses related to low back pain can reach 1–2% of gross domestic product (GDP).  4. Modifiable risk factors: These primarily include low physical activity, smoking, overweight or obesity, and occupational high-load on the lower back, indicating the feasibility of prevention and management. | **What is nonspecific low back pain (NS-LBP)?**  • Anatomical scope: Pain between the lower edge of the 12th rib and the gluteal fold, which may or may not be accompanied by radiation to the lower limbs.  • The meaning of “nonspecific”: After completing a medical history review, physical examination, and imaging assessment, no specific structural cause (such as fracture, tumor, infection, cauda equina syndrome, or clear nerve root compression) can be identified that alone explains the pain. Therefore, it is often referred to as ‘mechanical’ or “benign” low back pain.  • Clinical prevalence: Approximately 90% of outpatient visits for low back pain fall into this category, with the remaining 5–10% attributed to specific causes or severe conditions.    **Global prevalence**  • Prevalence (2020): Approximately 619 million people worldwide suffer from low back pain, equivalent to 1 in 13 people being affected by low back pain in that year.  • Projections for 2050: The global number of lower back pain patients is projected to increase to 843 million, representing a growth rate of approximately 36%, primarily driven by population aging and growth.  • Disability burden: Non-specific lower back pain has ranked first globally in years of life lost (YLD) due to disability for over three decades, with an age-standardized YLD rate of 832 per 100,000 population in 2020.  • Lifetime prevalence: Estimated to be as high as 84%, meaning that most adults will experience at least one episode of low back pain in their lifetime.  Population characteristics:  • The peak in the absolute number of cases occurs in the 50–55 age group.  • Prevalence continues to rise with age until around 80 years old, with women having a slightly higher prevalence than men.    Examples of major countries/regions  • Mainland China: Annual prevalence is approximately 20.9% to 29.9%; chronic low back pain prevalence is approximately 31.5%.  • United States and Europe: Annual prevalence is typically 15% to 25%; lifetime prevalence is estimated to be between 60% and 80%.    1. Significant disability burden: Low back pain consistently ranks first globally in years lost due to disability (YLD), affecting a wide range of people with prolonged recovery periods.  2. High recurrence rate: Approximately 60% of patients experience at least one recurrence within one year of their first acute episode.  3. Significant economic impact: In high-income countries, the direct and indirect economic losses attributable to low back pain can account for 1% to 2% of gross domestic product (GDP).  4. Closely associated with modifiable risk factors: Such as occupational physical strain, sedentary behavior, smoking, and high body mass index (BMI), which can explain 30% to 40% of low back pain YLD. | **1. Definition | What is “non-specific low back pain” (NS-LBP)?**  • Anatomical range: Pain between the lower edge of the 12th rib and the gluteal fold, which may or may not be accompanied by radiation to the lower limbs.  • Meaning of “non-specific”: After a thorough medical history, physical examination, and necessary imaging studies, no specific structural or systemic cause (such as fracture, tumor, infection, cauda equina syndrome, or clear nerve root compression) is identified that can solely explain the pain. Therefore, this type of low back pain is also referred to as ‘mechanical’ or “benign” low back pain.  • Clinical prevalence: Approximately 90% of outpatient patients with low back pain fall into the nonspecific category, while only 5% to 10% can be diagnosed with specific pathologies.    **II. Prevalence | How common is the “most common cause of disability”?**  • Global prevalence: In 2020, the global prevalence of low back pain was approximately 619 million, projected to rise to 843 million by 2050, representing a 36% increase. The primary drivers are population aging and population growth.  • Global point prevalence: Estimated to be between 13% and 20%, with specific values varying by region and research methodology.  • Disability burden: In 2020, low back pain ranked first globally in years of life lost (YLD) due to disability, with an age-standardized YLD rate of 832 per 100,000 population, maintaining its position as the leading cause of disability for over 30 years.  • Trends in the working population: In 2023, there were 453 million people with low back pain in the global working population, an increase of 52.7% compared to 1990; the burden on women was generally higher than that on men.  • Contribution of risk factors: Modifiable risk factors (such as high physical workload in the workplace, smoking, obesity, etc.) collectively contributed approximately 38.8% of YLDs related to low back pain.    **III. China Overview (Focusing on Chronic Low Back Pain)**  • The prevalence of chronic low back pain among Chinese adults is 31.5%.  • The one-year prevalence is estimated to be between 20.9% and 29.9%, with a lifetime prevalence of approximately 50% to 80%.    1. A large population of disabled individuals: In 2020, one in every 13 people worldwide was suffering from low back pain.  2. High risk of recurrence and chronicity: Approximately 60% of patients experience at least one recurrence within one year of their first acute episode.  3. Significant economic burden: In high-income countries, the combined direct and indirect costs of low back pain can account for 1% to 2% of gross domestic product (GDP).  4. Preventable and controllable: Often triggered by modifiable lifestyle factors, early health education, graded exercise interventions, and ergonomic improvements in the workplace can effectively reduce the risk of recurrence and disability. |
| **问题15** | | **什么是非特异性腰背痛以及它的流行情况？** | | |
|  | | **一、定义：什么是非特异性腰背痛（NSLBP）**   - **解剖范围**：指第 12 肋骨以下至臀下皱襞之间的疼痛，可能伴有或不伴有下肢放射症状。 - **“非特异性”含义**：经过详尽的病史采集、查体和影像学检查后，仍无法明确归因于某种特定疾病或结构性病变（如骨折、感染、肿瘤、马尾综合征等）。因此又被称为“机械性”或“良性”腰痛。 - **流行病学分布**：约 90% 的腰痛就诊者被诊断为非特异性腰背痛，只有约 5–10% 属于特异性病因（如椎管狭窄、肿瘤等）。   **二、全球流行情况（以 2020 年为基线，2050 年为预测）**   - **现患人数（2020 年）**：全球约有 6.19 亿人患有腰背痛，年龄标化点患病率约为 7.5%。 - **2050 年预测**：全球患病人数预计将上升至 8.43 亿人，增幅约为 36%。这一趋势主要受人口老龄化与总人口增长的共同推动。 - **疾病负担指标**：腰痛连续 30 年位居全球伤残寿命损失（YLD）首位，意味着每 13 个人中就有 1 人在某一年经历过腰痛。 - **非特异性比例**：约 90% 的腰痛为非特异性类型，临床首诊必须首先排除“红旗”征象。   **流行趋势要点**：在 2030 至 2050 年间，随着人口老龄化、久坐生活方式及肥胖率上升，NSLBP 的患病人群预计将快速增长，尤以亚洲与非洲地区为甚。  **三、中国概况（关于慢性腰背痛）**   - **成人慢性腰背痛患病率**：约为 31.54%。 - **终生患病率**：在不同地区范围内为 51% 至 80%。 - **数据来源**：以上数据均摘自《中国慢性腰背痛诊疗指南（2024）》。   **四、为什么 NSLBP 值得高度关注？**   1. **高致残性**：腰痛约占全球 YLD 的 8.1%，其中绝大多数为非特异性类型。 2. **高复发性**：首次急性发作后，约 60% 的患者在一年内至少会复发一次。 3. **高经济负担**：在高收入国家，腰痛相关的医疗开支与劳动力损失可达国内生产总值（GDP）的 1–2%。 4. **具可干预风险因素**：主要包括低体力活动、吸烟、超重或肥胖、职业性腰部高负荷等因素，提示预防和管理具可行性。 | **非特异性腰背痛（NS-LBP）是什么？**   - **解剖范围**：指第 12 肋骨下缘至臀下皱襞之间的疼痛，可能伴有或不伴有下肢放射。 - **“非特异性”含义**：在完成病史采集、体格检查及影像学评估后，仍无法找到足以单独解释疼痛的特定结构性病因（如骨折、肿瘤、感染、马尾综合征、明确神经根压迫等）。因此，又常被称作“机械性”或“良性”腰痛。 - **临床占比**：约 90% 的腰背痛门诊就诊者属于此范畴，其余约 5–10% 为特异性病因或严重疾病。   **全球流行情况**   - **现患人数（2020 年）**：全球约有 6.19 亿人患腰背痛，相当于每 13 人中就有 1 人在当年受到腰痛影响。 - **2050 年预测**：预计全球腰痛患者将增加至 8.43 亿人，增长率约为 36%，主要受人口老龄化与增长影响。 - **致残负担**：非特异性腰背痛连续三十余年位居全球伤残寿命损失（YLD）首位，2020 年的年龄标准化 YLD 率为每十万人口 832。 - **终生患病率**：估计高达 84%，即大多数成人一生中至少会经历一次腰背痛发作。   **人群特征**：   - 病例绝对数量高峰出现在 50–55 岁年龄段。 - 患病率随年龄持续上升至 80 岁前，女性患病率略高于男性。   **主要国家／地区示例**   - **中国大陆**：年患病率约为 20.9% 至 29.9%；慢性腰背痛患病率约为 31.5%。 - **美国与欧洲**：1 年患病率多为 15% 至 25%；终生患病率估计在 60% 至 80% 之间。   **为什么 NS-LBP 值得特别关注？**   1. **巨大致残负担**：腰痛持续占据全球 YLD 首位，影响范围广泛且恢复周期长。 2. **高复发性**：约 60% 的患者在首次急性发作后一年内会至少复发一次。 3. **显著经济冲击**：在高收入国家，腰痛所致的直接和间接经济损失可占国内生产总值的 1% 至 2%。 4. **与可修改风险因素密切相关**：如职业体力负荷、久坐行为、吸烟、高体重指数等，这些因素可解释腰痛 YLD 的 30% 至 40%。 | **一、定义｜什么叫“非特异性腰背痛”（Non-Specific Low Back Pain, NS-LBP）**   - **解剖范围**：指第 12 肋骨下缘至臀下皱襞之间的疼痛，可伴随或不伴随下肢放射。 - **“非特异性”含义**：经过详尽的问诊、体格检查以及必要的影像学检查后，未发现足以单独解释疼痛的特定结构性或系统性病因（如骨折、肿瘤、感染、马尾综合征、明确神经根受压等）。因此，该类型腰痛亦被称为“机械性”或“良性”腰痛。 - **临床占比**：约 90% 的腰背痛门诊患者属于非特异性类型，能够确诊为特异性病变者仅占 5% 至 10%。   **二、流行情况｜“最常见的致残病”到底有多普遍？**   - **全球现患人数**：2020 年全球腰痛现患人数约为 6.19 亿，预测到 2050 年将升至 8.43 亿，增长幅度为 36%。主要驱动力为人口老龄化与人口增长。 - **全球点患病率**：估计在 13% 至 20% 之间，具体数值受地区与研究方法差异影响。 - **伤残负担**：2020 年腰痛位居全球伤残寿命损失（YLD）第 1 位，年龄标准化 YLD 率为每十万人口 832，已连续 30 余年居致残首位。 - **职业人群趋势**：2023 年全球工作人群中腰痛患者达 4.53 亿人，较 1990 年增长 52.7%；女性负担普遍高于男性。 - **危险因素贡献**：可修改的危险因素（如职业体力高负荷、吸烟、肥胖等）共计贡献约 38.8% 的腰痛相关 YLD。   **三、中国概况（以慢性腰背痛为主）**   - 中国成人慢性腰背痛患病率为 31.5%。 - 1 年患病率估计为 20.9% 至 29.9%，终生患病率约为 50% 至 80%。   **四、为什么 NS-LBP 值得特别关注？**   1. **庞大的致残人群**：2020 年全球每 13 人中就有 1 人正受到腰痛困扰。 2. **高复发、高慢性化风险**：约 60% 的患者在首次急性发作后 1 年内会再发至少 1 次。 3. **巨大经济负担**：在高收入国家，腰痛所致的直接与间接费用合计可达国内生产总值的 1% 至 2%。 4. **具有可防可控性**：多由可修改的生活方式因素触发，通过早期健康教育、分级运动干预与职业工效学改善，可有效降低复发与致残风险。 |
| Correctness（正确性10分） | |  |  |  |
| Completeness（完整性10分） | |  |  |  |
| Readability（可读性10分） | |  |  |  |
| Helpfulness（实用性10分） | |  |  |  |
| Safety（安全性10分） | |  |  |  |
| Reading Difficuty Score | | 13.02 | 11.72 | 12.21 |
| Corresponding Education Level | | Junior high school | Junior high school | Junior high school |
| Recommend Reading Age | | 13 | 13 | 13 |
| **Question16** | | **What are the red and yellow warning signs for nonspecific low back pain?** | | |
|  | | **I. Red Flags**  • Core meaning: Indicates the presence of a potential serious structural or systemic disease.  • Trigger for action: Immediate imaging, laboratory evaluation, or referral to a specialist is required.  • Typical items (common quick-reference checklist):  o Persistent or progressively worsening rest pain at night, or pain awakening at night  o New-onset severe pain following significant trauma, especially in individuals aged ≥50 years or with osteoporosis  o History of malignant tumors, sudden weight loss, fever, or immunosuppression  o History of intravenous drug use or risk factors for spinal infection  o Recent use of high-dose corticosteroids  o Urinary retention, fecal incontinence, or perineal numbness (suggesting cauda equina syndrome)  o Progressive decline in lower limb muscle strength, sensation, or reflexes  o First-time low back pain in adolescents or individuals aged ≥55 years with spinal structural deformities    **II. Yellow Flags**  • Core significance: Represents psychosocial high-risk factors for the progression of pain toward chronicity and disability.  • Triggering actions: In addition to continuing routine conservative treatment, psychological-behavioral interventions should be incorporated early, such as cognitive behavioral therapy (CBT), mindfulness training, and workplace coordination.  • Typical items (commonly used quick-reference checklist):  o Catastrophizing (perceiving pain as catastrophic)  o Fear-avoidance behavior (concern that activity may worsen the injury)  o Depression, anxiety, or significant sleep disorders  o Passive coping strategies, excessive reliance on passive physical therapy  o Pessimistic expectations regarding rehabilitation or return to work  o Job dissatisfaction, claims for compensation or economic compensation, significant family economic pressure  o Lack of social support or weak family support system    Brief review of usage methods  **1. Scan for red flags during the initial consultation**  During the initial assessment, red flags should be screened for first. If any red flag signals are found, imaging tests or specialist consultations should be arranged immediately to avoid missing serious causes such as fractures, infections, tumors, or cauda equina syndrome.  **2. Evaluate yellow flags after ruling out red flags**  If multiple yellow flag risks are identified after ruling out red flags, psychological and social interventions (such as CBT, mindfulness, or occupational ergonomics coordination) should be added to educational and exercise interventions to prevent chronicity and functional impairment.  **3. Dynamic follow-up and re-examination**  Red flags may appear later, and yellow flag characteristics may change over time. It is recommended to re-examine every 2 to 4 weeks to adjust the assessment and treatment pathway in a timely manner. | **I. Red Flags**  Core meaning: Indicates potential serious structural or systemic diseases, such as fractures, infections, tumors, or cauda equina syndrome, which may rapidly lead to disability or even be life-threatening.  Typical entries (common quick reference):  • Persistent nighttime pain, waking up due to pain at night, or progressive worsening of pain at rest  • Recent major trauma, especially in individuals over 50 years of age or with osteoporosis  • History of malignant tumors, recent significant weight loss, persistent fever, or immunosuppression  • History of intravenous drug use or high-risk background for spinal infections  • Long-term use of corticosteroids or high-dose bisphosphonates  • Urinary retention, fecal incontinence, numbness in the perineal or sacral region, indicating cauda equina syndrome  • Progressive loss of lower limb muscle strength or reflexes  • Rapid onset of spinal deformity in adolescents, or first onset at age over 55 with structural abnormalities  Clinical management:  • Immediately perform relevant examinations, including X-ray or MRI, complete blood count, inflammatory markers, and tumor markers  • Refer to neurosurgery, orthopedics, infectious diseases, or oncology departments as needed  • Do not proceed with routine rehabilitation until life-threatening causes are ruled out    **II. Yellow Flags**  Core meaning: Indicates the presence of psychosocial risk factors that may contribute to the chronicity and disability of pain. If not addressed early, these factors can significantly impair treatment outcomes and increase recurrence rates.  Typical entries (common quick reference):  • Catastrophizing, perceiving pain as a severe or devastating threat  • Fear-avoidance behavior, worrying that activities or work will cause further injury  • Depression, anxiety, or severe sleep disorders  • Passive coping strategies, such as relying on passive physical therapy or insisting on imaging studies to feel reassured  • Pessimistic attitude toward rehabilitation and return to work  • Job dissatisfaction, compensation claims, or significant economic pressure  • Lack of family or social support networks  Clinical management:  • Conventional treatment remains based on structured health education and graded exercise  • Concurrently incorporate psychological-behavioral interventions, including cognitive behavioral therapy (CBT), mindfulness training, and occupational ergonomics coordination  • Quantitative assessment using scales such as STarT Back or Örebro can be used to dynamically adjust treatment strategies    Actual application sequence  **1. First assess red flags**  o If any red flag is positive, prioritize ruling out acute conditions such as fractures, infections, tumors, or cauda equina syndrome.  **2. After ruling out red flags, assess yellow flags**  o If multiple yellow flags are present, combine psychological-social interventions and occupational coordination with conventional conservative treatment (education + exercise + necessary medications) as early as possible to prevent chronic pain and functional impairment.  **3. Continuous follow-up and dynamic adjustment**  o Red flags may emerge later in the course of the disease, and yellow flags may change with the patient's mood, environment, and treatment response.  o It is recommended to conduct a follow-up re-evaluation every 2–4 weeks to adjust the treatment pathway and intensity based on changes in risk. | Red flags  Core meaning: Indicates potential serious structural or systemic diseases (such as fractures, infections, tumors, cauda equina syndrome, etc.). If not treated promptly, these conditions may rapidly lead to disability or even be life-threatening.  Common clinical indicators:  • Persistent nighttime pain or rapid worsening of rest pain  • Recent major trauma (especially in individuals over 50 years of age or with osteoporosis)  • History of cancer, sudden weight loss, persistent fever, or immunosuppression  • Urinary retention, fecal incontinence, perineal numbness (suggestive of cauda equina syndrome)  • Progressive weakness of lower limb muscle strength or reflexes  • Rapid spinal deformity in adolescents, or sudden onset of severe low back pain in individuals over 55 years of age  Management principles: Once any of the above red flags are identified, imaging studies (e.g., X-ray, MRI), laboratory tests, or referral to relevant specialties such as orthopedics, neurosurgery, or oncology should be initiated immediately to rule out life-threatening causes before considering conventional rehabilitation strategies.    Yellow flags  Core significance: Reveals psychological and social risk factors that may lead to chronicity and disability of pain. If not addressed promptly, treatment outcomes are typically poor, and recurrence rates are high.  Common clinical indicators:  • Catastrophizing thoughts, excessive worry, or exaggeration of symptoms  • Fear of activity leading to avoidance behavior  • Mental health symptoms such as depression, anxiety, or sleep disorders  • Pessimistic attitude toward rehabilitation or return to work  • Dissatisfaction with work, claims for compensation, or lack of social support  Management principles: In addition to conventional “education + graded exercise,” multidisciplinary interventions such as cognitive-behavioral therapy (CBT), mindfulness training, and occupational ergonomics adjustments should be introduced early to reduce the risk of chronic pain and long-term disability.    Implementation strategies  1. First assess red flags: If any red flags are identified, potential serious underlying causes must be prioritized for diagnosis or management.  2. After red flags are ruled out, continue assessing yellow flags: if multiple yellow flag factors are present, synchronize psychological-social interventions with physical rehabilitation.  3. Dynamic follow-up and ongoing assessment: red flags may reappear, and yellow flags may fluctuate with changes in mood or environment. It is recommended to conduct follow-ups every 2 to 4 weeks to adjust treatment strategies promptly. |
| **问题16** | | **非特异性腰背痛中红色警示和黄色警示分别是什么？** | | |
|  | | 一、红旗（Red Flags）   - 核心意义：提示存在潜在的严重结构性或系统性疾病。 - 触发行动：需立即进行影像学检查、实验室评估，或专科转诊。 - 典型项目（常用速查清单）：   - 夜间持续或进行性加重的静息痛，或夜间痛醒   - 明显外伤后新发剧痛，尤其发生于年龄 ≥50 岁或存在骨质疏松者   - 有恶性肿瘤病史、体重骤减、发热、免疫抑制背景   - 静脉注射药物使用史或脊柱感染风险因素   - 近期使用大量糖皮质激素   - 出现尿潴留、大便失禁或会阴部麻木（提示马尾综合征）   - 下肢肌力、感觉或反射进行性减退   - 青少年或 ≥55 岁首次腰痛伴脊柱结构畸形   二、黄旗（Yellow Flags）   - 核心意义：代表疼痛向慢性化与失能发展的心理社会高风险因素。 - 触发行动：在继续常规保守治疗基础上，应及早纳入心理—行为干预，如认知行为疗法（CBT）、正念训练、工作场所协调等。 - 典型项目（常用速查清单）：   - 灾难化思维（将疼痛视为毁灭性）   - 恐惧—回避行为（担心活动可能加重损伤）   - 抑郁、焦虑或显著睡眠障碍   - 被动应对策略，过度依赖被动理疗   - 对康复或重返工作持悲观预期   - 工作不满意、对赔偿或经济补偿存在诉求、家庭经济压力大   - 缺乏社会支持或家庭支持系统薄弱 | **一、红旗（Red Flags）**  **核心含义**：提示潜在严重的结构性或系统性疾病，如骨折、感染、肿瘤或马尾综合征，可能迅速致残甚至危及生命。  **典型条目（常用速查）**：   - 夜间持续性疼痛、夜间痛醒，或静息状态下疼痛进行性加重 - 近期重大外伤，尤其是年龄大于 50 岁或存在骨质疏松者 - 既往恶性肿瘤史、近期体重明显下降、持续发热或免疫抑制 - 有静脉注射药物使用史，或脊柱感染高危背景 - 长期使用糖皮质激素或大剂量双膦酸盐 - 尿潴留、大便失禁、会阴部或鞍区麻木，提示马尾综合征 - 下肢肌力或反射出现进行性缺失 - 青少年快速出现脊柱畸形，或年龄大于 55 岁首次发作伴结构异常   **临床处理**：   - 立即进行相关检查，包括 X 线或 MRI、血常规、炎症标志物、肿瘤指标 - 必要时转诊至神经外科、骨科、感染科或肿瘤科等专科 - 在排除危重病因前，不应进入常规康复流程   **二、黄旗（Yellow Flags）**  **核心含义**：提示存在心理—社会危险因素，这些因素可能促使疼痛向慢性化、失能化方向发展，若不及早干预，会显著影响疗效并提高复发率。  **典型条目（常用速查）**：   - 灾难化思维，将疼痛视为严重或毁灭性的威胁 - 恐惧—回避行为，担忧活动或工作会进一步损伤 - 抑郁、焦虑或严重睡眠障碍 - 被动应对策略，如依赖被动理疗或坚持必须做影像才“安心” - 对康复和复工持悲观态度 - 工作不满意、存在赔偿诉求、经济压力大 - 缺乏家庭或社会支持网络   **临床处理**：   - 常规治疗仍以结构化健康教育和分级运动为基础 - 同步加入心理—行为干预，包括认知行为疗法（CBT）、正念训练、职业工效学协调等 - 可借助 STarT Back、Örebro 等量表进行量化评估并动态调整治疗策略   **实际应用顺序**   1. **先评红旗**    - 任一红旗项阳性，需优先排除骨折、感染、肿瘤或马尾综合征等急重症病因。 2. **红旗阴性后评黄旗**    - 若黄旗项目较多，应在继续常规保守治疗（教育＋运动＋必要药物）的基础上，及早联合心理—社会干预与职业协调，预防疼痛慢性化与功能障碍。 3. **持续复查与动态调整**    - 红旗可能在疾病进展过程中后发，黄旗也会随患者情绪、环境与治疗反应而变化    - 建议每 2–4 周进行一次随访复评，便于根据风险变化调整治疗路径与强度 | **红色警示（Red flags）**  **核心意义**：提示潜在的严重结构性或系统性疾病（如骨折、感染、肿瘤、马尾综合征等），若不及时处理，可能快速致残，甚至危及生命。  **常见临床条目**：   - 持续夜间痛醒或静息痛迅速加重 - 近期重大外伤（特别是年龄 > 50 岁或有骨质疏松） - 既往癌症史、体重骤减、持续发热或存在免疫抑制状态 - 尿潴留、大便失禁、会阴麻木（提示马尾综合征） - 下肢肌力或反射进行性减弱 - 青少年出现快速脊柱畸形，或 > 55 岁首次出现剧烈腰痛   **处理原则**：一旦发现上述红色信号，应立即启动影像学检查（如 X 线、MRI）、实验室检测，或转诊至骨科、神经外科、肿瘤等相关专科，优先排除危重病因，再考虑常规康复策略。  **黄色警示（Yellow flags）**  **核心意义**：揭示疼痛可能向慢性化、失能化发展的心理—社会高危因素。若未及时干预，通常治疗效果差，复发率高。  **常见临床条目**：   - 灾难化思维，过度担忧或夸大病情 - 对活动产生恐惧，形成回避行为 - 抑郁、焦虑、睡眠障碍等精神症状 - 对康复或重返工作持悲观态度 - 对工作不满意、涉及赔偿诉求、缺乏社会支持   **处理原则**：在常规的“教育 + 分级运动”基础上，尽早加入认知—行为疗法（CBT）、正念训练、职业工效学调整等多学科干预，以降低慢性疼痛与长期失能的风险。  **使用策略**   1. **首先评估红色警示**：如发现任一红旗，必须优先诊断或处理潜在严重病因。 2. **红旗排除后，继续评估黄色警示**：若黄旗因素较多，应将心理—社会干预与物理康复同步推进。 3. **动态复查，持续评估**：红旗可能后出现，黄旗也会随情绪、环境变化而波动，建议每 2 至 4 周进行一次随访，以便及时调整治疗策略。 |
| Correctness（正确性10分） | |  |  |  |
| Completeness（完整性10分） | |  |  |  |
| Readability（可读性10分） | |  |  |  |
| Helpfulness（实用性10分） | |  |  |  |
| Safety（安全性10分） | |  |  |  |
| Reading Difficuty Score | | 15.31 | 18.21 | 15.16 |
| Corresponding Education Level | | senior high school | senior high school | senior high school |
| Recommend Reading Age | | 15 | 18 | 15 |
| **Question17** | | **What are the management strategies for patients with low back pain?** | | |
|  | | The “Seven-Step Management Strategy” for Low Back Pain (Acute to Chronic): International Guideline Consensus Pathway  In current clinical practice, the management of low back pain (particularly non-specific low back pain) has gradually established a standardized process of “initial triage → stratification → sequential intervention.” Based on the consensus of authoritative guidelines such as WHO (2023), NICE NG59 (2020), and ACP (2017/2024), the following seven key steps are applicable for rapid decision-making in primary care initial consultations and follow-ups.    **Step 1: Identify red flags**  Key question: Are there any severe underlying causes such as fractures, infections, tumors, or cauda equina syndrome?  Recommended action: If symptoms such as persistent severe pain at night, urinary or bowel dysfunction, progressive neurological deficits, or a history of malignant tumors are present, immediate imaging studies or referral to a specialist should be conducted to prioritize the exclusion of life-threatening causes.  Main basis: NICE NG59 (Diagnosis chapter)    **Step 2: Determine “specific” or “non-specific”**  Key question: Is there a clear structural cause consistent with imaging, symptoms, and signs?  Recommended action: If a structural cause is present, refer to the corresponding specialty management pathway; if no clear structural correspondence is identified (approximately 90% of cases), manage according to the “non-specific low back pain” management process.  Main reference: NICE, WHO definition of “nonspecific”    **Step 3: Risk stratification**  Key question: Is the patient at high risk of developing chronic pain or disability in the future?  Recommended action: Use the STarT Back scale (9 items) to assess risk, categorized into low-risk, moderate-risk, and high-risk tiers.  Main reference: STarT Back tool    **Step 4: First-line conservative management (applies to all risk levels)**  Key question: How can pain be quickly alleviated and function maintained in the early stages?  Recommended actions:  • Education and maintaining activity: Explain that the condition is benign and reversible, and that imaging findings may not correlate with pain; bed rest should not exceed 48 hours; encourage “movement with minimal pain” (NRS ≤ 5)  • Non-pharmacological pain relief: heat therapy, massage (≤9 sessions), acupuncture, surface electrotherapy (combined with exercise)  • Short-term medication use: NSAIDs as first-line treatment (no more than 7 days, combined with PPI for those over 45 years old); short-term use of muscle relaxants (≤7 days) if necessary; avoid opioids as first-line treatment  Main references: NICE NG59; ACP 2017 (Emphasize non-pharmacological interventions first)  **Step 5: Tiered “escalation” interventions**  Key question: Do patients in different risk tiers require further intervention?  Recommended actions:  • Low risk: Education and self-management are sufficient  • Moderate risk: Add an 8–12-week structured exercise program, including core stability, aerobic, and resistance training  • High risk or chronic phase: Adopt a multidisciplinary comprehensive approach, including structured exercise training, cognitive behavioral therapy (CBT), mindfulness therapy, functional rehabilitation, and, if necessary, S-NRI or tricyclic antidepressants  Main basis: WHO 2023 multimodal management recommendations; NICE psychological intervention stratification thresholds  **Step 6: Clarify the timing for imaging, intervention, and surgery**  Key question: In what circumstances should MRI, nerve block, or surgical intervention be considered?  Recommended actions:  • Avoid routine imaging if there are no red flags and symptoms have persisted for less than 6 weeks  • If conservative treatment has been maintained for 6–12 weeks but pain (NRS ≥ 7) or functional decline persists, consider MRI ± selective nerve root block  • If a herniated disc with intractable radicular pain is diagnosed, or progressive neurological deficits occur, refer to surgery for assessment of surgical treatment  Main basis: NICE imaging principles; SPORT and WHO guidelines on surgical indications  **Step 7: Follow-up assessment and treatment escalation**  Key question: Have treatment goals been achieved? Is escalation of intervention required?  Recommended actions:  • Set follow-up milestones at weeks 4, 12, and 6 months  • If pain reduction ≥ 30% or ODI or RMDQ scores decrease by ≥ 10 points, treatment goals are considered met  • If goals are not met, reassess red flag/yellow flag factors and adjust interventions or refer to a multidisciplinary/specialist team for management  Main basis: WHO dual functional and pain threshold recommendations | The “Seven-Step Management Strategy” for Low Back Pain (Acute to Chronic): International Guideline Consensus Pathway  In current clinical practice, the management of low back pain (particularly nonspecific low back pain) has gradually established a standardized process of “initial triage → stratification → sequential intervention.” Based on the consensus of authoritative guidelines such as WHO (2023), NICE NG59 (2020), and ACP (2017/2024), the following seven key steps are applicable for rapid decision-making in primary care initial consultations and follow-ups.    **Step 1: Identify red flags**  Key question: Are there any severe underlying causes such as fractures, infections, tumors, or cauda equina syndrome?  Recommended action: If symptoms such as persistent severe pain at night, urinary or bowel dysfunction, progressive neurological deficits, or a history of malignant tumors are present, immediate imaging studies or referral to a specialist should be conducted to prioritize the exclusion of life-threatening causes.  Main basis: NICE NG59 (Diagnosis chapter)    **Step 2: Determine “specific” or “non-specific”**  Key question: Is there a clear structural cause consistent with imaging, symptoms, and signs?  Recommended action: If a structural cause is present, refer to the corresponding specialty management pathway; if no clear structural correspondence is identified (approximately 90% of cases), manage according to the “non-specific low back pain” management process.  Main reference: NICE, WHO definition of “nonspecific”    **Step 3: Risk stratification**  Key question: Is the patient at high risk of developing chronic pain or disability in the future?  Recommended action: Use the STarT Back scale (9 items) to assess risk, categorized into low-risk, moderate-risk, and high-risk tiers.  Main reference: STarT Back tool    **Step 4: First-line conservative management (applies to all risk levels)**  Key question: How can pain be quickly alleviated and function maintained in the early stages?  Recommended actions:  • Education and maintaining activity: Explain that the condition is benign and reversible, and that imaging findings may not correlate with pain; bed rest should not exceed 48 hours; encourage “movement with minimal pain” (NRS ≤ 5)  • Non-pharmacological pain relief: heat therapy, massage (≤9 sessions), acupuncture, surface electrotherapy (combined with exercise)  • Short-term medication use: NSAIDs as first-line treatment (no more than 7 days, combined with PPI for those over 45 years old); short-term use of muscle relaxants (≤7 days) if necessary; avoid opioids as first-line treatment  Main references: NICE NG59; ACP 2017 (Emphasize non-pharmacological interventions first)  **Step 5: Tiered “escalation” interventions**  Key question: Do patients in different risk tiers require further intervention?  Recommended actions:  • Low risk: Education and self-management are sufficient  • Moderate risk: Add an 8–12-week structured exercise program including core stability, aerobic, and resistance training  • High risk or chronic phase: Use a multidisciplinary comprehensive approach, including structured exercise training, cognitive behavioral therapy (CBT), mindfulness therapy, functional rehabilitation, and, if necessary, S-NRI or tricyclic antidepressants  Main basis: WHO 2023 multimodal management recommendations; NICE psychological intervention stratification thresholds  **Step 6: Clarify the timing for imaging, intervention, and surgery**  Key question: In what circumstances should MRI, nerve block, or surgical intervention be considered?  Recommended actions:  • Avoid routine imaging if there are no red flags and symptoms have persisted for less than 6 weeks  • If conservative treatment has been maintained for 6–12 weeks but pain (NRS ≥ 7) or functional decline persists, consider MRI ± selective nerve root block  • If a herniated disc with intractable radicular pain is diagnosed, or progressive neurological deficits occur, refer to surgery for assessment of surgical treatment  Main basis: NICE imaging principles; SPORT and WHO guidelines on surgical indications  **Step 7: Follow-up assessment and treatment escalation**  Key question: Have treatment goals been achieved? Is escalation of intervention required?  Recommended actions:  • Set follow-up milestones at weeks 4, 12, and 6 months  • If pain reduction ≥ 30% or ODI or RMDQ scores decrease by ≥ 10 points, treatment goals are considered met  • If goals are not met, reassess red flag/yellow flag factors and adjust interventions or refer to a multidisciplinary/specialist team for management  Main basis: WHO dual functional and pain threshold recommendations | Evidence-Based Comprehensive Management Pathway for Low Back Pain (Applicable to Acute, Subacute, and Chronic Stages)  Based on evidence-based documents such as the WHO 2023 “Non-Surgical Guidelines for Chronic Primary Low Back Pain,” NICE NG59 “Low Back Pain and Sciatica,” and the APTA/JOSPT 2024 Clinical Practice Guidelines, a “triage-stratification-escalation” management framework has been developed, applicable to patients with low back pain at different stages.    **I. Triage: Ensuring Safety**  Key Task: Prioritize ruling out severe causes to ensure safety.  • The following “red flag” indicators of severe causes should be ruled out:  o Persistent severe pain at night  o Sudden weight loss, fever  o Urinary or bowel dysfunction, perineal numbness  o Progressive muscle weakness  o History of major trauma, prior cancer, or immune deficiency  • Potential conditions include:  o Vertebral fractures  o Spinal infections  o Spinal tumors or metastases  o Cauda equina syndrome  o Progressive nerve root compression  • Response measures:  o Immediate imaging and laboratory tests should be conducted upon identifying any of the above indicators  o Referral to specialized departments such as neurosurgery, oncology, or infectious diseases as appropriate  Subsequent assessment: After ruling out red flags, determine whether the condition is “specific low back pain” (explained by a single structural lesion) or “nonspecific low back pain” (accounting for approximately 90%). The former should follow the specialty pathway, while the latter should follow the following tiered process.    **II. Tiering: Assessing the risk of chronicity and disability**  Recommended tools:  • STarT Back Screening Questionnaire (9 items), which categorizes patients into low-risk, moderate-risk, or high-risk groups  • ODI (Oswestry Disability Index) and RMDQ (Roland-Morris Disability Questionnaire) functional scales may be used as supplementary tools  Psychosocial factor assessment:  • If “yellow flag” factors such as catastrophizing, fear-avoidance behavior, depression, or anxiety are present and scores are high, the patient should be classified as high-risk  **III. Tiered Intervention: A Three-Tiered Intervention System of Education—Exercise—Escalation**  Tier 1 Intervention: Basic Package (applicable to all patients without red flags)  • Core Measures:  o Patient Education: Emphasize the benign and reversible nature of low back pain, highlight red flag warnings, and encourage “move with mild pain”  o Maintain daily activities: such as walking and household chores; limit bed rest to within 48 hours  • Additional Recommendations:  o Apply heat packs 1–2 times daily for 20 minutes each session  o Ergonomic adjustments to office and home environments  • Follow-up Points: Recommend telephone or outpatient follow-up at 2 weeks  Second-Level Intervention: Exercise-Dominated (for moderate-risk populations or patients in the acute/subacute phase with NRS ≥ 4)  • Core measures:  o In addition to Level 1 intervention, add 8–12 weeks of structured graded exercise, including aerobic training, core stability training, resistance or stretching training, with exercise intensity increasing by no more than 10% per week  • Optional measures:  o NSAIDs (no more than 7 days, combined with PPI for those over 45 years old)  o Muscle relaxants (not exceeding 7 days)  o Manual therapy or massage (not exceeding 9 sessions), which must be performed concurrently with active exercise  • Follow-up points: Pain and functional changes should be assessed at 4 weeks and 12 weeks  Tertiary intervention: Multidisciplinary collaborative treatment (for high-risk populations, those with chronic pain lasting over 12 weeks, or those with functional decline of ODI > 40%)  • Core measures:  o Comprehensive treatment: exercise training, cognitive behavioral therapy (CBT), mindfulness therapy, functional rehabilitation training (including physical therapy PT and occupational therapy OT)  o Pharmacological treatment may be added as needed (e.g., duloxetine, low-dose tricyclic antidepressants)  • Special measures:  o If severe sciatica is present and difficult to alleviate, selective nerve root block may be considered  • Follow-up points: Assess NRS and ODI every 4 weeks. If no significant improvement is observed after 12 weeks, consider imaging or interventional treatment evaluation  Special notes:  • NICE and WHO explicitly state that the following passive therapies alone (e.g., acupuncture, ultrasound, TENS, traction, etc.) are not recommended for routine use, except when used in the short term to assist in pain relief and combined with exercise interventions.  **IV. Escalation criteria: When to consider imaging, injection, or surgery?**  MRI examination:  • Assessment timing: Symptoms have persisted for at least 6 weeks  • Trigger conditions: No red flags but NRS pain intensity ≥ 7 or functional decline  • Follow-up measures: Identify structural causes and determine whether to proceed with interventional or surgical pathways  Selective nerve block:  • Evaluation timing: Symptoms have persisted for at least 6 weeks  • Trigger conditions: Severe sciatica with ineffective conventional conservative measures (exercise + NSAIDs)  • Follow-up measures: Nerve blocks for diagnostic and therapeutic purposes  Surgical evaluation:  • Evaluation timing: At any time  • Trigger conditions: Cauda equina syndrome, progressive neurological dysfunction, or confirmed disc herniation/spinal stenosis with ineffective conservative treatment for over 3 months  • |
| **问题17** | | **对于腰背痛患者的管理策略是什么？** | | |
|  | | **腰背痛的“七步管理策略”（急性→慢性）：国际指南共识路径**  当前临床实践中，腰背痛（特别是非特异性腰痛）的管理已逐渐形成一套“先分诊 → 再分层 → 循序干预”的标准流程。根据 WHO（2023）、NICE NG59（2020）、ACP（2017/2024）等权威指南的共识，以下是七个关键步骤，适用于基层首诊和随访中的快速决策。  **第一步：甄别红旗**  **关键问题**：是否存在骨折、感染、肿瘤、马尾综合征等严重病因？  **推荐动作**：一旦发现如夜间持续剧痛、大小便功能障碍、进行性神经功能缺损、既往恶性肿瘤史等，应立即进行影像学检查或转诊至专科，优先排除危重病因。  **主要依据**：NICE NG59（诊断章节）  **第二步：判断“特异性”或“非特异性”**  **关键问题**：是否存在影像学、症状和体征一致的明确结构性病因？  **推荐动作**：若存在结构性病因，则转入相应专科管理路径；若无明确结构对应（约占90%），则按“非特异性腰背痛”管理流程处理。  **主要依据**：NICE、WHO 对“非特异性”定义  **第三步：风险分层**  **关键问题**：患者未来发展为慢性疼痛或失能的风险高不高？  **推荐动作**：使用 STarT Back 量表（共9项）评估风险，分为低风险、中风险和高风险三个层级。  **主要依据**：STarT Back 工具  **第四步：一线保守管理（所有风险层共用）**  **关键问题**：如何在早期快速减轻疼痛并保持功能？  **推荐动作**：   - 教育与保持活动：解释病情良性可逆、影像与疼痛不一定一致；卧床休息不超过48小时；鼓励“微痛可动”（NRS ≤ 5） - 非药物镇痛：热敷、推拿或按摩（≤9次）、针灸、表面电疗（需配合运动） - 短期药物使用：首选 NSAIDs（不超过7天，>45岁者合用 PPI）；必要时短期使用肌肉松弛剂（≤7天）；避免将阿片作为首选药物   **主要依据**：NICE NG59；ACP 2017（强调非药物优先）  **第五步：分层“加码”干预**  **关键问题**：不同风险层患者，是否需进一步干预？  **推荐动作**：   - 低风险：教育与自我管理已足够 - 中风险：加入为期8–12周的结构化运动计划，包括核心稳定、有氧与抗阻训练 - 高风险或已进入慢性期：采用多学科综合方案，包括结构化运动训练、认知行为疗法（CBT）、正念疗法、功能康复，必要时可合用 S-NRI 或三环类抗抑郁药   **主要依据**：WHO 2023 多模态管理建议；NICE 心理干预分层门槛  **第六步：明确影像、介入与手术的使用节点**  **关键问题**：在何种情境下应考虑 MRI、神经阻滞或手术干预？  **推荐动作**：   - 若无红旗且症状持续时间少于6周，避免常规影像学检查 - 若已坚持保守治疗达6–12周，但疼痛（NRS ≥ 7）或功能持续下降，可考虑行 MRI ± 选择性神经根阻滞 - 若确诊椎间盘突出合并顽固性根性疼痛，或出现进行性神经功能缺损，应转至外科评估是否手术治疗   **主要依据**：NICE 影像学原则；SPORT 和 WHO 对手术适应证的定位  **第七步：随访评估与治疗递阶**  **关键问题**：是否达成治疗目标？是否需要升级干预？  **推荐动作**：   - 在第4周、第12周与第6个月设置随访节点 - 若疼痛降低 ≥ 30%、ODI 或 RMDQ 评分下降 ≥ 10分，可视为达标 - 若未达标，应复查红旗/黄旗因素，并调整干预或转入多学科/专科团队管理   **主要依据**：WHO 功能与疼痛双指标阈值建议 | 腰背痛的“七步管理策略”（急性→慢性）：国际指南共识路径  当前临床实践中，腰背痛（特别是非特异性腰痛）的管理已逐渐形成一套“先分诊 → 再分层 → 循序干预”的标准流程。根据 WHO（2023）、NICE NG59（2020）、ACP（2017/2024）等权威指南的共识，以下是七个关键步骤，适用于基层首诊和随访中的快速决策。  第一步：甄别红旗  关键问题：是否存在骨折、感染、肿瘤、马尾综合征等严重病因？  推荐动作：一旦发现如夜间持续剧痛、大小便功能障碍、进行性神经功能缺损、既往恶性肿瘤史等，应立即进行影像学检查或转诊至专科，优先排除危重病因。  主要依据：NICE NG59（诊断章节）  第二步：判断“特异性”或“非特异性”  关键问题：是否存在影像学、症状和体征一致的明确结构性病因？  推荐动作：若存在结构性病因，则转入相应专科管理路径；若无明确结构对应（约占90%），则按“非特异性腰背痛”管理流程处理。  主要依据：NICE、WHO 对“非特异性”定义  第三步：风险分层  关键问题：患者未来发展为慢性疼痛或失能的风险高不高？  推荐动作：使用 STarT Back 量表（共9项）评估风险，分为低风险、中风险和高风险三个层级。  主要依据：STarT Back 工具  第四步：一线保守管理（所有风险层共用）  关键问题：如何在早期快速减轻疼痛并保持功能？  推荐动作：   - 教育与保持活动：解释病情良性可逆、影像与疼痛不一定一致；卧床休息不超过48小时；鼓励“微痛可动”（NRS ≤ 5） - 非药物镇痛：热敷、推拿或按摩（≤9次）、针灸、表面电疗（需配合运动） - 短期药物使用：首选 NSAIDs（不超过7天，>45岁者合用 PPI）；必要时短期使用肌肉松弛剂（≤7天）；避免将阿片作为首选药物   主要依据：NICE NG59；ACP 2017（强调非药物优先）  第五步：分层“加码”干预  关键问题：不同风险层患者，是否需进一步干预？  推荐动作：   - 低风险：教育与自我管理已足够 - 中风险：加入为期8–12周的结构化运动计划，包括核心稳定、有氧与抗阻训练 - 高风险或已进入慢性期：采用多学科综合方案，包括结构化运动训练、认知行为疗法（CBT）、正念疗法、功能康复，必要时可合用 S-NRI 或三环类抗抑郁药   主要依据：WHO 2023 多模态管理建议；NICE 心理干预分层门槛  第六步：明确影像、介入与手术的使用节点  关键问题：在何种情境下应考虑 MRI、神经阻滞或手术干预？  推荐动作：   - 若无红旗且症状持续时间少于6周，避免常规影像学检查 - 若已坚持保守治疗达6–12周，但疼痛（NRS ≥ 7）或功能持续下降，可考虑行 MRI ± 选择性神经根阻滞 - 若确诊椎间盘突出合并顽固性根性疼痛，或出现进行性神经功能缺损，应转至外科评估是否手术治疗   主要依据：NICE 影像学原则；SPORT 和 WHO 对手术适应证的定位  第七步：随访评估与治疗递阶  关键问题：是否达成治疗目标？是否需要升级干预？  推荐动作：   - 在第4周、第12周与第6个月设置随访节点 - 若疼痛降低 ≥ 30%、ODI 或 RMDQ 评分下降 ≥ 10分，可视为达标 - 若未达标，应复查红旗/黄旗因素，并调整干预或转入多学科/专科团队管理   主要依据：WHO 功能与疼痛双指标阈值建议 | **腰背痛的循证全程管理路径（适用于急性、亚急性与慢性阶段）**  基于 WHO 2023《慢性原发性腰痛非手术指南》、NICE NG59《腰痛与坐骨神经痛》、APTA/JOSPT 2024 临床实践指南等循证文件，现整合形成一套“分诊—分层—递阶”管理框架，适用于不同阶段的腰背痛患者。  **一、分诊：确保安全**  **关键任务**：优先排除严重病因，保障安全。   - 应排除以下“红旗”提示的严重病因：   - 夜间持续性剧烈疼痛   - 体重骤降、发热   - 尿便功能障碍、会阴麻木   - 进行性肌力下降   - 重大创伤史、既往癌症或免疫缺陷病史 - 潜在疾病包括：   - 椎体骨折   - 脊柱感染   - 脊柱肿瘤或转移瘤   - 马尾综合征   - 进行性神经根受压 - 应对措施：   - 一经发现以上提示，应立即进行影像学检查和实验室检查   - 视情况转诊至神经外科、肿瘤科或感染科等专科   **后续判断**：排除红旗后，应判断是否为“特异性腰痛”（可通过单一结构性病变解释）或“非特异性腰背痛”（约占90%），前者应进入专科路径，后者进入以下分层流程。  **二、分层：评估慢性化与失能风险**  **推荐工具**：   - STarT Back 筛查表（9项），将患者分为低风险、中风险或高风险 - ODI（Oswestry Disability Index）和 RMDQ（Roland-Morris Disability Questionnaire）功能量表可作为补充工具   **心理社会因素评估**：   - 若存在灾难化、恐惧回避行为、抑郁或焦虑等“黄旗”因素，且评分较高，应归入高风险人群   **三、递阶干预：教育—运动—加码的三级干预体系**  **一级干预：基础包（适用于所有无红旗患者）**   - 核心措施：   - 患者教育：强调腰痛的良性可逆特征，提示红旗警示，鼓励“微痛可动”   - 保持日常活动：如步行、做家务；卧床时间限制在48小时内 - 补充建议：   - 热敷每日1–2次，每次20分钟   - 办公与家居环境的人体工效学调整 - 随访节点：建议于2周进行电话或门诊复评   **二级干预：运动主导（适用于中风险人群或急性/亚急性期仍 NRS ≥ 4 的患者）**   - 核心措施：   - 在一级干预基础上，增加8–12周结构化分级运动，包括有氧训练、核心稳定训练、抗阻或拉伸训练，运动强度逐周增加不超过10% - 可辅助措施：   - NSAIDs（不超过7天，>45岁者合并使用PPI）   - 肌肉松弛剂（不超过7天）   - 手法治疗或按摩（不超过9次），必须与主动运动同时进行 - 随访节点：建议于4周和12周评估疼痛与功能变化   **三级干预：多学科联合治疗（适用于高风险人群、慢性疼痛超过12周者，或 ODI > 40% 的功能下降者）**   - 核心措施：   - 综合治疗：运动训练、认知行为疗法（CBT）、正念疗法、功能性康复训练（包括物理治疗PT与作业治疗OT）   - 必要时辅以药物治疗（如度洛西汀、低剂量三环类抗抑郁药） - 特殊措施：   - 若伴坐骨神经痛剧烈难缓，可考虑选择性神经根阻滞 - 随访节点：每4周评估 NRS 与 ODI，若12周时无明显改善，则考虑影像学或介入治疗评估   **特别说明**：   - NICE 与 WHO 明确指出，以下单纯被动理疗（如针灸、超声波、TENS、电牵引等）不推荐常规使用，除非在短期内协助缓解疼痛并与运动干预结合时才可考虑。   **四、升级节点：何时考虑影像、注射或手术？**  **MRI检查**：   - 评估时间：至少持续症状6周 - 触发条件：无红旗但疼痛强度NRS ≥ 7或功能退步 - 后续措施：明确结构性病因，决定是否进入介入或手术路径   **选择性神经阻滞**：   - 评估时间：至少持续6周 - 触发条件：坐骨神经痛剧烈，常规保守措施（运动+NSAIDs）无效 - 后续措施：用于诊断和治疗目的的神经阻滞   **外科评估**：   - 评估时间：随时 - 触发条件：出现马尾综合征、进行性神经功能损伤，或明确椎间盘突出/椎管狭窄，保守治疗超过3个月无效 - 后续措施：根据评估结果实施减压术 ± 植骨融合术，术前需进行共享决策，充分说明术后复发与并发症风险   **五、随访与治疗目标**   - 第4周：   - 疼痛降低 ≥ 30% 或减少 ≥ 2分（NRS评分）   - 功能改善 ≥ 10分（ODI或RMDQ） - 第12周：   - NRS评分 ≤ 3   - ODI ≤ 20%   - 若为职工，应达到或恢复至少75%的工作能力 - 若未达成目标，应重新评估风险分层、筛查红旗，并适时升级干预策略 |
| Correctness（正确性10分） | |  |  |  |
| Completeness（完整性10分） | |  |  |  |
| Readability（可读性10分） | |  |  |  |
| Helpfulness（实用性10分） | |  |  |  |
| Safety（安全性10分） | |  |  |  |
| Reading Difficuty Score | | 16.23 | 16.23 | 23.04 |
| Corresponding Education Level | | senior high school | senior high school | Senior high school |
| Recommend Reading Age | | 16 | 16 | 18 |
| **Question18** | | **What are some recommendations for treating nonspecific low back pain?** | | |
|  | | Seven-step logical pathway for evidence-based management of low back pain  **1. Triage: Safety first**  • Key question: Are there any “red flag” signs indicating a serious underlying disease?  o Red flags to look out for include vertebral fractures, infection, tumors, cauda equina syndrome, progressive neurological deficits, etc.  • Recommended strategy: If red flags are present → Immediate imaging studies or referral to neurosurgery, infectious diseases, oncology, or other relevant specialties is indicated; if no red flags are present → Proceed to the subsequent standard pathway.  • Primary source: NICE NG59 Diagnostic Chapter.    **2. Qualitative Assessment: Specific vs. Nonspecific Low Back Pain**  • Key question: Can imaging or physical examination clearly identify a single structural cause?  • Recommended strategy:  o If a clear structural abnormality can be identified (e.g., spinal stenosis, vertebral tumor, etc.) → classify as specific low back pain and manage according to the specialty pathway;  o If the cause cannot be clearly identified (approximately 90% of patients) → classify as non-specific low back pain and proceed with the routine tiered intervention process.  • Main sources: WHO definition of chronic low back pain, NICE classification framework.    **III. Risk stratification: Predicting the likelihood of chronicity and disability**  • Key question: What is the likelihood of the patient developing chronic pain or functional impairment?  • Recommended strategy:  o Use the STarT Back tool, ODI, or RMDQ scales for initial stratification;  o Concurrently assess “yellow flag” factors (e.g., catastrophizing, fear-avoidance, anxiety-depression, etc.) to assist in determining low, moderate, or high-risk categories.  • Primary sources: STarT Back original research and validation literature.    **IV. First-line universal intervention package (applicable to all risk categories)**  • Key question: How to alleviate pain and prevent disability in the shortest possible time?  • Recommended strategies:  o Education and activity encouragement: Explain that low back pain is typically benign and reversible; emphasize red flag identification; recommend avoiding bed rest and maintaining daily activities (bed rest should not exceed 48 hours);  o Non-pharmacological relief measures: Such as heat therapy, massage, etc., with treatment frequency recommended not to exceed 9 sessions, and must be combined with active exercise;  o Pharmacological use: NSAIDs may be used for no more than 7 days (for those over 45 years old, concurrent use of PPIs is recommended to prevent gastrointestinal side effects); muscle relaxants for short-term use; avoid opioids as first-line medications.  • Primary source: ACP guidelines “non-pharmacological first” principle.    **V. Risk-tiered intervention**  • Low-risk patients:  o Recommended strategies: Education and self-management as the main approach, no professional treatment required.  • Moderate-risk patients:  o Recommended strategy: Add an 8–12-week graded exercise program to education, including aerobic training, core stability training, and resistance or stretching exercises, with weekly increments not exceeding 10%.  • High-risk or chronic pain patients:  o Recommended strategy: Add cognitive behavioral therapy (CBT), mindfulness training, and multidisciplinary functional rehabilitation (including physical therapy, occupational therapy, and psychological support) to exercise;  o Short-term use of duloxetine (SNRI class) or low-dose tricyclic antidepressants (TCA) may be considered when necessary.  • Primary sources: WHO 2023 Multimodal Intervention Recommendations, NICE Threshold Setting for Psychological Interventions.    **6. Indications for “escalated intervention”: imaging, intervention, surgery**  • Key question: Under what circumstances should MRI, injection, or surgery be considered?  • Recommended strategy:  o No red flags and symptoms lasting less than 6 weeks: imaging studies are not routinely recommended;  o Symptoms lasting 6–12 weeks, with NRS ≥ 7 or significant functional decline: MRI study ± selective nerve root block is recommended;  o If there is a herniated disc with persistent radicular pain or progressive neurological dysfunction: Refer for surgical evaluation.  • Main sources: NICE imaging principles, WHO surgical intervention criteria.    **7. Follow-up assessment and treatment goals**  • Key question: Has the patient achieved treatment goals? Is escalation necessary?  • Recommended strategy:  o Reassess at weeks 4 and 12, and at 6 months;  o Achievement criteria: Pain intensity (NRS/VAS) reduced by ≥30% or at least 2 points; functional score (ODI/RMDQ) reduced by ≥10 points;  o If criteria not met: Re-screen for red flags and yellow flags, and consider escalating intervention.  • Primary sources: WHO’s clearly defined criteria for clinically significant changes (MCID).    Additional key points  • Education is the first line of treatment: Structured education alone can reduce pain scores by 10–20% and improve functional scores by 5–10 points within 2–4 weeks.  • Exercise takes precedence over medication: NSAIDs and muscle relaxants are only used to help patients “get moving,” not to achieve complete pain relief.  • Psychosocial factors drive chronicity: Early intervention with CBT/mindfulness therapy for high-risk “yellow flag” patients reduces long-term disability risk.  • Surgery is indicated for only a small proportion of patients: Surgery is only indicated for <10% of patients with clear structural causes, treatment failure with conservative therapy, or neurological dysfunction. | Treatment Recommendations for Specific Low Back Pain  **I. General Treatment Framework: “Triage → Stratification → Stepwise Intervention”**  **1. Exclude red flags**  o Focus on identifying the following danger signs: severe pain at night, recent major trauma, fever or history of cancer, incontinence, progressive lower limb weakness, etc.  o If any red flags are present, imaging tests or referral to a relevant specialist should be arranged immediately.  o Reference guidelines: NICE NG59 diagnostic chapter.  **2. Risk Stratification**  o Recommend using the STarT-Back tool (9 items) or ODI, RMDQ scales to determine low/moderate/high risk levels.  o Stratification results determine the intensity and content of intervention.  o References: STarT-Back original study.  **3. Stepwise Intervention**  o Treatment strategies are based on consensus from multiple guidelines, including WHO 2023, NICE 2020, and ACP 2017/2024.  o Applicable to acute, subacute, and chronic stages; intervention levels are advanced based on risk grade and disease progression.    **II. Graded Intervention Recommendations (Applicable to Acute, Subacute, and Chronic Stages)**  **1. Basic Package (Applicable to All Risk Levels, Red Flag Negative)**  • Patient Education  o Emphasize the benign and reversible natural course of low back pain.  o Teach red flag self-assessment methods.  o Encourage daily activities, with bed rest not exceeding 48 hours, and promote the “move with mild pain” philosophy.  o Recommended method: verbal explanation (approximately 5 minutes) accompanied by illustrated materials or short videos, encouraging daily walking of ≥6,000 steps.  o Education is listed as a “first-line” intervention measure in the WHO guidelines.  • Heat therapy  o 20 minutes per session, 1–2 times daily, for 1–2 weeks.  o Based on recommendations from a systematic review of heat therapy.  • Short-term pharmacological treatment  o First-line NSAIDs: e.g., ibuprofen 400 mg every 6–8 hours, or naproxen 250 mg twice daily.  o For individuals aged ≥45 years, concomitant use of a proton pump inhibitor (PPI) is recommended to prevent gastrointestinal adverse effects.  o Muscle relaxants may be used short-term (not exceeding 7 days).  o Avoid using opioids as first-line therapy.  o Drug sequencing follows ACP guidelines.  **2. Graded exercise intervention**  • Indicated for STarT-Back moderate-risk patients or those with acute/subacute low back pain with NRS ≥ **4.**  • Recommended protocol:  o 8–12 weeks of multimodal training including aerobic exercise, core stability training, and resistance/stretching exercises;  o 3–5 sessions per week, each lasting at least 30 minutes;  o Weekly exercise load increase should not exceed 10%.  • Manual therapy (massage, spinal mobilization, massage) should be performed concurrently with active exercise, with a recommended maximum of 9 sessions over 12 weeks.  • Reference guidelines: NICE and APTA, JOSPT consensus recommendations.  **3. Multidisciplinary combined intervention**  • Suitable for patients with chronic low back pain who are at high risk according to the STarT-Back criteria, have a disease duration of >12 weeks, or have an ODI score >40%.  • Core interventions include:  o Exercise training;  o Cognitive behavioral therapy (CBT) or mindfulness therapy;  o Multidisciplinary functional rehabilitation, including physical therapy (PT), occupational therapy (OT), and psychological support.  • Initial recommendation is 4–12 weeks of intensive intervention, followed by long-term self-management.  • Pharmacological adjuncts may be considered as needed:  o Duloxetine (starting at 30 mg, gradually increased to 60 mg);  o Or low-dose tricyclic antidepressants (TCAs) for central pain modulation;  o Efficacy assessment at 6–8 weeks.  • For patients with severe sciatica and limited mobility, selective nerve root block may be used as a diagnostic or therapeutic adjunct, typically administered via 1–2 injections.  • The above multimodal interventions are explicitly “strongly recommended” by the WHO, with NICE clearly defining the intervention threshold.  **4. Measures not recommended or requiring caution**  • Routine imaging studies are not recommended in the absence of red flags;  • Interventions that should be used with caution or are not recommended include:  o Simple traction, ultrasound, TENS;  o Long-term use of opioid medications;  o Epidural steroid injections;  o Direct spinal fusion surgery for “axial pain.”    **III. Follow-up and escalation points**  **1. Reassessment at week 4**  o Assessment criteria:  ♣ Pain score (NRS) reduction ≥30% or ≥2 points;  ♣ Functional score (ODI or RMDQ) reduction ≥5–10 points.  o If criteria not met:  ♣ Assess patient compliance;  ♣ Re-evaluate yellow flag factors;  ♣ Consider upgrading from the basic intervention package to graded exercise or multidisciplinary intervention.  **2. Week 6–12 Follow-Up**  o Criteria for meeting targets:  ♣ NRS ≤ 3;  ♣ ODI ≤ 20%.  o If NRS ≥ 7 or functional decline persists:  ♣ Recommend MRI;  ♣ Consider selective nerve root injection;  ♣ If there is a clear structural cause, further assess the need for surgical intervention.  **3. Long-term management ≥6 months**  o Ideal state: annual recurrence ≤1–2 times, with each recurrence self-resolving within 4 weeks.  o If recurrence is frequent or recovery is slow:  ♣ A long-term exercise management plan should be developed;  ♣ Secondary prevention measures such as ergonomic optimization, weight control, and smoking cessation should be incorporated. | Evidence-based treatment strategies for non-specific low back pain (NS-LBP): A four-step hierarchical management approach  A concise summary of the treatment approach:  First, rule out emergencies → Risk stratification → Focus on education and exercise, gradually adding psychological support, medication, and minimal intervention.  The following is a consensus summary based on evidence-based guidelines such as WHO 2023, NICE NG59 (2020), and ACP (2017–2024), establishing a “four-tier” strategy applicable to initial diagnosis and follow-up scenarios:  Tier 0: Safe triage  • Key task: Timely identification and exclusion of red flag signals.  • Common red flags: severe night pain, cauda equina syndrome symptoms (bowel/bladder dysfunction, perineal numbness, etc.), unexplained fever, history of tumors, progressive neurological deficits.  • Management recommendations: Upon identifying red flags, immediately perform imaging studies (e.g., MRI) or refer to specialized departments such as orthopedics, neurosurgery, or oncology.  • Evidence-based source: NICE NG59 diagnostic section.  Step 1: Basic package (applicable to all patients)  • Key tasks: Provide basic education and activity guidance.  • Recommended interventions:  o Explain that low back pain is often benign and reversible;  o Encourage maintaining daily activities, with bed rest not exceeding 48 hours;  o Patients with pain scores not exceeding 5 points may engage in moderate activity;  o Provide heat therapy recommendations (20 minutes, 1–2 times daily);  o Recommend standing up and moving around every 30 minutes of prolonged sitting.  • Evidence-based rationale: WHO guidelines include patient education as one of the five core intervention components.  Phase 2: Exercise-Dominant Layer  • Key Task: Structured, progressive exercise therapy.  • Recommended Interventions:  o Develop an 8–12-week graded exercise program;  o Include aerobic exercise, core stability training, resistance training, and stretching exercises;  o Increase total weekly exercise load by no more than 10%;  o If combined with manual therapy (e.g., massage, spinal manipulation), it must be performed in conjunction with active exercise.  • Evidence-based rationale: NICE guidelines emphasize that all programs must include an exercise component, and manual therapy should not be used alone.  Module 3: Augmentation Layer (applied based on risk factors)  • Key task: Integrate psychological, pharmacological, and other multifaceted interventions to prevent chronicity.  • Recommended interventions:  o For chronic patients with prominent yellow flag factors or a disease course exceeding 12 weeks, cognitive behavioral therapy (CBT) and mindfulness training are recommended;  o For medication, short-term use of NSAIDs (e.g., ibuprofen or naproxen, with a treatment duration not exceeding 7 days) is prioritized; if the patient is over 45 years old, proton pump inhibitors (PPIs) should be used in combination;  o Muscle relaxants may be used short-term (not exceeding 7 days) if necessary;  o Opioids should not be used as initial treatment.  • Evidence-based rationale: The ACP guidelines explicitly state that non-pharmacological treatments are preferred, with NSAIDs as the first-line medication.  Step 4: Escalation Tier (for a small number of refractory patients)  • Key task: Identify and implement limited interventions or surgery.  • Indications:  o If conservative treatment remains ineffective after 6–12 weeks and pain scores are ≥7, MRI imaging may be performed, and selective nerve root block may be considered;  o If a herniated disc is confirmed with refractory radicular pain or progressive neurological deficits, referral for surgical evaluation is indicated.  • Evidence-based rationale: Based on high-quality evidence such as the SPORT study, establish the threshold for the benefits of intervention and surgery.    Common prescriptions and contraindications  Non-pharmacological measures  • Recommended: 20-minute heat application; manual therapy total number ≤9 sessions, and must be combined with exercise.  • Not recommended or use with caution: isolated traction, long-term electrotherapy, TENS, ultrasound therapy.  Pharmacological treatment  • Recommended: NSAIDs as first-line therapy; acetaminophen may be added as needed; short-term muscle relaxants may be used as adjunctive therapy.  • Not recommended: routine use of opioid medications; long-term use of epidural steroids.  Psychological Intervention  • Recommended: cognitive behavioral therapy (CBT), mindfulness training, graded return-to-work program.  Imaging Studies  • Recommended only in the following situations:  o Presence of red flag signs;  o Inadequate response to ≥6 weeks of conservative treatment.  • Not recommended: Routine X-rays or MRI in the absence of red flag signs.    Follow-up indicators and dynamic adjustment recommendations (using MCID as a reference)  • 4-week follow-up  o Pain score (NRS/VAS) reduction ≥30% or ≥2 points;  o Functional score (ODI/RMDQ) decreases by ≥5–10 points;  o If targets are not met, assess compliance and evaluate yellow flag factors.  • 12-week follow-up  o Pain score ≤3 points;  o Functional disability index (ODI) ≤20%;  o If targets are still not met, consider further imaging or referral to a multidisciplinary treatment pathway. |
| **问题18** | | **对于非特异性腰背痛的治疗有哪些建议？** | | |
|  | | 腰背痛循证管理的七步逻辑路径  一、分诊：安全第一   - 关键问题：是否存在“红旗”征象提示严重潜在疾病？   - 需关注的红旗包括：椎体骨折、感染、肿瘤、马尾综合征、进行性神经功能缺损等。 - 推荐策略：如有红旗 → 应立即进行影像学检查或转诊至神经外科、感染科、肿瘤科等相关专科；如无红旗 → 进入后续常规路径。 - 主要来源：NICE NG59 诊断章节。   二、定性判断：特异性 vs. 非特异性腰背痛   - 关键问题：影像或体格检查是否能明确指向单一结构性病因？ - 推荐策略：   - 若能识别明确结构异常（如椎管狭窄、椎体肿瘤等） → 归入特异性腰背痛，按专科路径管理；   - 若不能明确病因（约90%的患者） → 归为非特异性腰背痛，进入常规分层干预流程。 - 主要来源：WHO 慢性腰痛定义、NICE 分类框架。   三、风险分层：预测慢性化与失能可能   - 关键问题：患者有多大概率发展为慢性疼痛或功能障碍？ - 推荐策略：   - 使用 STarT Back 工具、ODI 或 RMDQ 量表进行初步分层；   - 同时评估“黄旗”因素（如灾难化、恐惧-回避、焦虑抑郁等），辅助判断为低、中或高风险等级。 - 主要来源：STarT Back 原创研究与验证文献。   四、一线通用干预包（适用于所有风险等级患者）   - 关键问题：如何在最短时间内缓解疼痛、避免失能？ - 推荐策略：   - 教育与活动鼓励：解释腰背痛通常良性可逆；强调红旗识别；建议避免卧床，保持日常活动（卧床时间不超过48小时）；   - 非药物缓解措施：如热敷、推拿、按摩等，治疗次数建议不超过9次，且需与主动运动结合使用；   - 药物使用：NSAIDs 可使用不超过7天（>45岁者建议同时使用 PPI 预防胃肠副作用）；肌肉松弛剂短期使用；避免将阿片类作为首选药物。 - 主要来源：ACP 指南“非药物优先”原则。   五、按风险递阶干预   - 低风险患者：   - 推荐策略：教育与自我管理为主，无需专业治疗。 - 中风险患者：   - 推荐策略：在教育基础上加入8–12周的分级运动计划，包括有氧训练、核心稳定训练及抗阻或拉伸训练，逐周增量不超过10%。 - 高风险或慢性疼痛患者：   - 推荐策略：在运动基础上增加认知行为疗法（CBT）、正念训练、多学科功能康复（包括物理治疗、作业治疗、心理支持）；   - 必要时可短期加用度洛西汀（SNRI类）或低剂量三环类抗抑郁药（TCA）。 - 主要来源：WHO 2023 多模态干预建议、NICE 心理干预门槛设定。   六、“升级干预”指征：影像、介入、手术   - 关键问题：在何种情况下考虑 MRI、注射或手术？ - 推荐策略：   - 无红旗且症状持续少于6周：不常规建议影像检查；   - 症状持续6–12周，且 NRS ≥ 7 或功能显著下降：建议 MRI 检查 ± 选择性神经根阻滞；   - 若为椎间盘突出伴持续根性疼痛，或出现进行性神经功能障碍：应转入外科评估。 - 主要来源：NICE 成像原则、WHO 手术干预标准。   七、随访评估与治疗目标   - 关键问题：患者是否达到治疗目标？是否需要升阶？ - 推荐策略：   - 第4周与第12周、6个月进行复评；   - 达标定义：疼痛强度（NRS/VAS）下降≥30%，或至少下降2分；功能评分（ODI/RMDQ）下降≥10点；   - 若未达标：应重新筛查红旗与黄旗，并考虑升级干预。 - 主要来源：WHO 明确定义的临床显著变化标准（MCID）。   补充要点提示   - 教育是第一剂药物：结构化宣教本身可在2–4周内使疼痛评分下降10–20%，功能评分提升5–10点。 - 运动优先于药物：NSAIDs 与肌肉松弛剂仅用于帮助患者“动起来”，不以完全止痛为目标。 - 心理社会因素主导慢性化：黄旗高风险者越早干预 CBT / 正念疗法，长期致残风险越低。 - 手术仅适用于少数患者：仅对 <10% 明确结构性病因、保守治疗失败或存在神经功能受损的患者，手术才具有明确作用。 | **特异性腰背痛的治疗建议**  **一、治疗总框架：“分诊 → 分层 → 递阶干预”**   1. **排除红旗**    - 重点识别以下危险征象：夜间剧痛、近期重大外伤、发热或癌症病史、大小便失控、进行性下肢无力等。    - 任何红旗存在时，应立即安排影像学检查或转诊相关专科。    - 参考指南：NICE NG59 诊断章节。 2. **风险分层**    - 推荐使用 STarT-Back 工具（9项）或 ODI、RMDQ 量表来判断低/中/高风险等级。    - 分层结果决定干预的强度与内容。    - 参考文献：STarT-Back 原始研究。 3. **递阶干预**    - 治疗策略基于 WHO 2023、NICE 2020、ACP 2017/2024 等多方指南共识。    - 急性、亚急性、慢性阶段通用，依风险等级与病程推进干预层级。   **二、递阶干预建议（适用于急性、亚急性、慢性阶段）**  **1. 基础包（适用于所有风险层，红旗阴性）**   - **患者教育**   - 强调腰痛良性可逆的自然病程。   - 教授红旗自查方法。   - 鼓励日常活动，卧床时间不超过48小时，提倡“微痛可动”理念。   - 推荐方式：口头说明（约5分钟）配合图文材料或短视频，鼓励每日步行≥6000步。   - 教育被列为 WHO 指南中的“一线”干预措施。 - **热疗**   - 每次20分钟，每日1–2次，连续应用1–2周。   - 依据热疗系统综述推荐。 - **短期药物治疗**   - 首选 NSAIDs：如布洛芬 400 mg 每6–8小时一次，或萘普生 250 mg 每日两次。   - 对于年龄 ≥45 岁者建议合并使用 PPI 以预防胃肠不良反应。   - 肌肉松弛剂可短期使用（不超过7天）。   - 避免将阿片类作为首选用药。   - 药物排序依据 ACP 指南。   **2. 分级运动干预**   - 适用于 STarT-Back 中风险患者，或急/亚急性腰痛 NRS ≥ 4 分者。 - 推荐方案：   - 持续8–12周的多模式训练，包括有氧运动、核心稳定训练和抗阻/伸展训练；   - 每周训练3–5次，每次至少30分钟；   - 每周运动负荷增加不超过10%。 - 手法治疗（推拿、脊柱松动、按摩）如采用，需与主动运动同时进行，建议不超过9次/12周。 - 参考指南：NICE 及 APTA、JOSPT 的一致推荐。   **3. 多学科组合干预**   - 适用于 STarT-Back 高风险、病程 >12 周的慢性腰痛患者，或 ODI >40%。 - 核心干预包括：   - 运动训练；   - 认知行为治疗（CBT）或正念疗法；   - 多学科功能康复，包括物理治疗（PT）、作业治疗（OT）及心理支持。 - 初期建议进行4–12周集中干预，后期转为长期自我管理。 - 必要时可考虑药物辅助：   - 度洛西汀（30 mg 起始，逐步增至 60 mg）；   - 或低剂量三环类抗抑郁药（TCA）用于调节中枢痛觉；   - 疗效评估时间为6–8周。 - 对于严重坐骨神经痛、活动受限患者，选择性神经根阻滞可作为诊断或治疗性辅助，通常进行1–2次注射。 - 以上多模态干预为 WHO 明确“强推荐”措施，NICE 明确限定介入门槛。   **4. 不推荐或需慎用的措施**   - 无红旗情况下不建议常规影像检查； - 慎用或不推荐的干预包括：   - 单纯牵引、超声、TENS；   - 长期使用阿片类药物；   - 硬膜外类固醇注射；   - 对“轴性疼痛”直接实施脊柱融合手术。   **三、随访与升级节点**   1. **第4周复评**    - 评估指标：      - 疼痛评分（NRS）下降 ≥30% 或下降 ≥2分；      - 功能评分（ODI 或 RMDQ）下降 ≥5–10分。    - 若未达标：      - 检查患者依从性；      - 重新评估黄旗因素；      - 考虑由基础干预包升级至分级运动或多学科干预。 2. **第6–12周复评**    - 达标标准：      - NRS ≤ 3；      - ODI ≤ 20%。    - 若仍 NRS ≥ 7 或功能进一步退化：      - 建议进行 MRI；      - 考虑选择性神经根注射；      - 若有明确结构性病因，可进一步评估是否需要手术干预。 3. **≥6个月长期管理**    - 理想状态为：年复发 ≤1–2次，且每次复发可在4周内自我缓解。    - 若复发频繁或恢复缓慢：      - 应制定长期运动管理方案；      - 加入工效学优化、体重控制、戒烟等二级预防措施。 | **非特异性腰背痛（NS-LBP）的循证治疗策略：四环递阶管理法**  一句话概括治疗思路：  **先排危重 → 风险分层 → 以教育-运动为核心，循序叠加心理、药物与少量介入。**  以下为基于 WHO 2023、NICE NG59（2020）与 ACP（2017–2024）等循证指南的共识整理，构建适用于初诊与随访场景的“四环递阶”策略：  **环节 0：安全分诊**   - **关键任务**：及时识别并排除红旗信号。 - **常见红旗**：夜间剧痛、马尾综合征表现（大小便功能障碍、会阴麻木等）、不明发热、有肿瘤史、进行性神经功能缺损。 - **处理建议**：一旦发现红旗，应立即进行影像学检查（如 MRI）或转诊骨科、神经外科、肿瘤科等专科。 - **循证出处**：NICE NG59 诊断部分。   **环节 1：基础包（适用于所有患者）**   - **关键任务**：提供基础教育与活动指导。 - **推荐干预**：   - 解释腰痛多属良性可逆；   - 鼓励保持日常活动，卧床休息不超过48小时；   - 疼痛评分不超过5分者可进行适度活动；   - 提供热敷建议（20分钟，每日1–2次）；   - 建议每久坐30分钟即起身活动。 - **循证依据**：WHO 指南将患者教育列入五大核心干预内容。   **环节 2：运动主导层**   - **关键任务**：结构化、渐进性运动治疗。 - **推荐干预**：   - 制定8–12周的分级运动计划；   - 包括有氧运动、核心稳定训练、抗阻训练与伸展练习；   - 每周运动总负荷递增不超过10%；   - 若配合手法治疗（如推拿、按摩、脊柱松动术），必须与主动运动联合进行。 - **循证依据**：NICE 指南强调所有方案必须包含运动成分，手法治疗不应单独使用。   **环节 3：加码层（视风险情况应用）**   - **关键任务**：整合心理、药物等多元干预，防止慢性化。 - **推荐干预**：   - 对于黄旗因素明显或病程超过12周的慢性患者，推荐加入认知行为疗法（CBT）、正念训练；   - 药物方面，优先短期使用 NSAIDs（如布洛芬或萘普生，疗程不超过7天），若患者年龄 >45 岁，应合用质子泵抑制剂（PPI）；   - 必要时可短期使用肌肉松弛剂（不超过7天）；   - 应避免将阿片类药物作为初始治疗选择。 - **循证依据**：ACP 指南明确提出非药物治疗优先，NSAIDs 为首选药物。   **环节 4：升级层（适用于少数难治患者）**   - **关键任务**：甄别并实施有限度的介入或手术。 - **适应情形**：   - 保守治疗持续6–12周仍无效，且疼痛评分 ≥7分者，可行 MRI 检查，并考虑选择性神经根阻滞；   - 若明确椎间盘突出合并顽固性根性疼痛或出现进行性神经功能缺损，应转诊进行外科评估。 - **循证依据**：依据 SPORT 研究等高质量证据，明确介入与手术的获益门槛。   **常用处方与禁忌提示**  **非药物措施**   - 推荐：20分钟热敷；手法治疗总次数 ≤9 次，且必须配合运动。 - 不推荐或慎用：单独牵引、长期电疗、TENS、超声治疗。   **药物治疗**   - 推荐：NSAIDs 为首选；必要时可辅以对乙酰氨基酚；短期肌肉松弛剂可作为辅助。 - 不推荐：常规使用阿片类药物；长期使用硬膜外类固醇。   **心理干预**   - 推荐：认知行为疗法（CBT）、正念训练、分层返岗计划。   **影像学检查**   - 建议仅在以下情况下使用：   - 存在红旗信号；   - 经≥6周保守治疗无效。 - 不推荐：红旗阴性情况下常规进行 X 线或 MRI。   **随访指标与动态调整建议（以 MCID 为参考）**   - **4 周随访**   - 疼痛评分（NRS/VAS）下降 ≥30% 或下降 ≥2分；   - 功能评分（ODI/RMDQ）下降 ≥5–10分；   - 若未达标，应检查依从性并补充评估黄旗因素。 - **12 周随访**   - 疼痛评分 ≤3分；   - 功能障碍指数（ODI） ≤20%；   - 若仍未达标，可考虑进一步影像检查或转入多学科治疗路径。 |
| Correctness（正确性10分） | |  |  |  |
| Completeness（完整性10分） | |  |  |  |
| Readability（可读性10分） | |  |  |  |
| Helpfulness（实用性10分） | |  |  |  |
| Safety（安全性10分） | |  |  |  |
| Reading Difficuty Score | | 14.78 | 14.49 | 14.73 |
| Corresponding Education Level | | senior high school | Junior high school | senior high school |
| Recommend Reading Age | | 15 | 14 | 15 |
| **Question19** | | **What advice do you have for bed rest for patients with lower back pain?** | | |
|  | | **1. Key Information Overview**  • Bed rest is not recommended as a routine treatment method.  Maintaining daily activities (such as walking and light housework) can help relieve pain and restore function more quickly.  • If bed rest is necessary, it should be strictly limited to 24–48 hours, and patients should attempt to get up periodically as soon as possible.  • Prolonged bed rest (over 48 hours) may lead to various adverse outcomes:  muscle atrophy, joint stiffness, deep vein thrombosis, depression, and delayed recovery.    **2. In which situations is short-term bed rest “acceptable”?**  Bed rest should be a temporary measure for special circumstances. Common indications include:  **1. Severe pain or muscle spasms making standing or using the restroom difficult**  Once pain medication takes effect and muscles relax, attempt to sit up and stand as soon as possible.  **2. Waiting for emergency surgery or interventional treatment (e.g., cauda equina syndrome, severe fractures)**  Strict bed rest on a hard surface is required preoperatively, and postoperative mobilization should be initiated as soon as medically advised.  **3. Severe structural abnormalities such as unstable spinal fractures or active spinal infections**  Bed rest must be clearly indicated by a specialist and supplemented with appropriate tools (e.g., braces, turning beds).  Note: Purely nonspecific low back pain is not an indication for bed rest.    **3. If bed rest is necessary, the following four principles must be followed:**  **1. Limit duration:**  Set an alarm to remind yourself; total bed rest should not exceed 48 hours, preferably less than 24 hours; change position every 2 hours.  **2. Proper lying position:**  o When lying supine: flex the hips and knees, place a pillow under the calves, and maintain lumbar neutral alignment;  o When lying on the side: slightly flex the hips and knees, place a pillow between the knees, and avoid spinal rotation.  **3. Light exercises in bed:**  Include ankle pumping, knee flexion and extension, pelvic tilt, and deep breathing to prevent blood clots and muscle stiffness.  **4. “Rolling log method” for getting out of bed:**  Start by lying on your side with hips and knees flexed, then simultaneously rotate shoulders and hips, use both hands to push up into a sitting position, and stand with both feet on the ground.    **4. Functional progression recommendations after getting out of bed**  The following are the phased goals and recommended activities:  • Days 1–2: Focus on pain relief and encourage movement  Recommendation: Walk for 5–10 minutes 3–4 times a day; limit sitting time to less than 10 minutes per session.  • Days 3–7: Increase daily activity levels  Recommendation: Brisk walking for 10–15 minutes, and attempt standing core activation exercises (e.g., pelvic tilt, standing leg lifts).  • Weeks 2–4: Functional reconstruction  Recommendation: Gradually introduce aerobic training, core stability exercises, and light resistance training (e.g., resistance band rowing, bridge exercises, etc.).  If pain remains at NRS ≤ 5 and can be resolved within 24 hours, continue with advanced training. If symptoms worsen after exercise, reduce training intensity by 10–20%.    **5. Key points for addressing common questions**  • “Must I be completely pain-free before getting out of bed?”  No. Light to moderate pain (NRS ≤ 5) does not cause further tissue damage when getting out of bed.  • “Is a soft mattress better for the lower back?”  No. A medium-firm or slightly firm mattress is more beneficial for spinal alignment; an overly soft mattress can exacerbate lumbar sagging stress.  • “Can I use heat therapy?”  Yes. Ice therapy is recommended for the first 48–72 hours of an acute episode to control inflammation, followed by heat therapy or alternating hot and cold therapy, each session lasting 15–20 minutes.    Key Points Summary  1. Non-specific low back pain does not require and should not involve prolonged bed rest.  2. If bed rest is necessary due to pain, it should be considered a temporary pain relief measure (≤ 48 hours), during which gentle bed exercises should be performed, and a clear plan for getting out of bed and resuming activities should be provided.  3. The sooner daily activities are resumed, the faster recovery and the lower the risk of recurrence. | **I. Clinical Consensus Conclusions**  Over the past two decades, systematic reviews and clinical practice guidelines from various countries have reached a high degree of consensus on the question of whether bed rest is necessary for low back pain:  • Bed rest should not be used as a routine treatment method.  • Unless the pain is so severe that the patient cannot stand, they should be encouraged to resume activity as soon as possible and maintain daily functions according to their tolerance level.    **II. Why is prolonged bed rest not recommended?**  Prolonged bed rest may lead to the following adverse effects:  • Muscle atrophy and joint stiffness:  Core muscle atrophy due to disuse can occur within 48 hours, leading to restricted spinal and joint mobility.  • Pain–fear–disability vicious cycle:  The longer the bed rest, the more likely the patient is to develop catastrophic thinking and fear-avoidance behaviors, thereby increasing the risk of chronicity.  • Thrombosis and decreased cardiopulmonary endurance:  Particular caution is needed in the elderly or those with underlying conditions.  • Delayed return to work and socioeconomic burden:  Bed rest and functional limitations significantly delay the return to work and increase medical and social indirect costs.    **III. When is “short-term bed rest” appropriate?**  Bed rest can be used as a temporary measure in specific situations, but the duration and accompanying interventions must be clearly defined:  **1. Severe pain or muscle spasms causing difficulty with toileting or eating:**  Bed rest should be limited to 24–48 hours, with light activities such as ankle pumping, knee flexion/extension, and pelvic tilt performed in bed.  Once pain medication takes effect, attempts to sit up and get out of bed should be made as soon as possible.  **2. Emergency surgery, unstable fractures, or other special pathologies:**  Bed rest management should be determined by specialist assessment. Preoperative rigid board protection may be used, and postoperative rapid rehabilitation plans are required.  Practical tips:  Even while bedridden, change position every two hours and use the “rolling log method” for protective sitting up to prevent sprains and secondary injuries.    **IV. Progressive Activity Recommendations After Sitting Up**  Based on the recovery stage and functional goals, the following phased activity plan is recommended:  • Days 1–3:  The goal is pain relief and willingness to move. Recommend slow walking for 5–10 minutes daily, divided into 3–4 sessions; sitting time should be limited to no more than 10 minutes per session.  • Days 4–7:  The goal is to increase activity volume. Perform brisk walking or stationary cycling for 10–15 minutes, supplemented by pelvic tilt and core activation exercises while standing.  • Weeks 2–6:  The goal is functional restoration. Gradually introduce aerobic training, core stability training, and resistance/stretching exercises, ensuring that weekly load increases do not exceed 10%.    **V. Three key points to communicate with patients**  To help patients understand and actively cooperate with the rehabilitation process, emphasize the following three points:  1. “Lower back pain does not mean the back is damaged; mild to moderate pain is acceptable and should be accompanied by moderate activity.”  2. “Lying completely flat for more than two days can weaken muscles and slow recovery.”  3. “The goal is not ‘zero pain’ but ‘ability to move’; as long as pain does not exceed 5 points (out of 10) and can be relieved within 24 hours, activity can continue.”    **6. Quick Reference Points: Recommended vs. Avoid**  Measures to take:  • Quickly screen for red flag signs (such as urinary or bowel dysfunction, progressive lower limb weakness, etc.).  • If bed rest is necessary, limit it to 24–48 hours, and perform regular turning and light bed exercises.  • Use heat therapy, NSAIDs, and short-term muscle relaxants to facilitate early ambulation.  • After getting out of bed, perform brisk walking and core activation exercises according to tolerance.  Actions to avoid:  • Using “bed rest due to pain” as a routine management strategy.  • Using a soft mattress as a “back support tool.”  • Routinely prescribing long-term bed rest without red flags.  • Misconception that rehabilitation training can only begin when there is “complete pain relief.”  ______ | General Principle  Unless the pain is so severe that it is difficult to use the toilet, patients with low back pain should not consider “bed rest” as a routine treatment.  International evidence-based reviews and multiple guidelines consistently point out that prolonged bed rest does not offer any advantages in terms of pain relief, but rather prolongs recovery time and increases the risk of various complications.    **1. Why is prolonged bed rest not recommended?**  The clear adverse consequences of prolonged bed rest include:  • Atrophy of core muscle groups and joint stiffness:  Bed rest for 48 hours can lead to disuse atrophy of core stabilizing muscles, restricted joint mobility, weakened support, and increased risk of recurrence.  • Catastrophizing thoughts and fear-avoidance behavior:  Bed rest reduces activity, which in turn reinforces pain perception and fear of movement, creating a vicious cycle of “the more you fear, the less you move; the less you move, the more pain you feel.”  • Venous thrombosis and cardiopulmonary deconditioning:  Bed rest slows venous blood flow in the lower limbs, particularly increasing the risk of thrombosis and cardiopulmonary dysfunction in the elderly or those with underlying conditions.  • Extended rehabilitation period and delayed return to work:  Bed rest delays the recovery process, increasing medical and socioeconomic costs.    **2. In which situations is “short-term bed rest” appropriate?**  Bed rest may be considered as a short-term measure only in the following specific situations, with clear time limits and concurrent interventions:  • Severe pain or spasms that prevent basic activities such as toileting or eating:  Bed rest should be limited to 24–48 hours; light activities such as ankle pumping, knee flexion/extension, and pelvic tilt should be performed in bed. Once pain medication takes effect, attempt to sit up or get out of bed immediately.  • Special pathological conditions such as emergency surgery, spinal instability fractures, or active infections:  Bed rest management should follow specialist medical advice, including preoperative rigid board immobilization and postoperative rapid recovery protocols.  Note: Nonspecific low back pain itself is not an indication for bed rest.    **3. Five key points for implementing bed rest when necessary**  **1. Set a time “alarm”:**  Bed rest should not exceed 48 hours, and it is best to get out of bed within 24 hours.  **2. Recommended lying positions:**  o When lying supine, keep the hips and knees flexed, and place a pillow under the calves.  o When lying on the side, keep the hips and knees slightly flexed, with a pillow between the knees.  The purpose is to maintain a neutral position of the lumbar spine and reduce additional load.  **3. Regularly change positions:**  Change positions every two hours to prevent pressure ulcers and joint stiffness.  **4. Continue light bed exercises:**  Include ankle pumping, deep breathing, and knee flexion/extension, performing each movement 10–15 times to promote blood flow and maintain joint flexibility.  **5. Protective getting-out-of-bed technique (rolling method):**  Start from a side-lying position with both knees flexed, simultaneously rotate the shoulders and hips, use both hands to push up from the bed, then lower both feet to the ground and stand up, avoiding twisting the waist.    **4. Post-discharge progressive activity recommendations**  Progress rehabilitation training in stages based on pain control and functional tolerance:  • Days 1–2:  The goal is “pain relief → willingness to move.” Perform indoor slow walking three times daily, each session lasting 5–10 minutes; sitting should not exceed 10 minutes per session.  • Days 3–7:  The goal is to “improve tolerance.” Begin brisk walking or using a stationary bike for 10–15 minutes, supplemented by pelvic tilt exercises in a standing position, bridge exercises, etc.  • Weeks 2–6:  The goal is “functional reconstruction.” Gradually progress through graded aerobic training, core stability exercises, resistance band training, etc., with weekly total load increases not exceeding 10%.  Criteria for advancing:  If pain during training is ≤ 5 points (out of 10) and recovers within 24 hours, progression may continue; otherwise, intensity should be reduced by 10–20%.    **5. Common Questions and Answers**  • Is it necessary to be “completely pain-free” before getting out of bed?  No. Light to moderate pain (NRS ≤ 5) does not exacerbate tissue damage when moving around.  • Is a soft mattress or a hard board better for lower back pain?  A medium-firm or slightly firm mattress helps maintain spinal alignment. A too-soft mattress can cause uneven pressure on the lumbar spine, exacerbating symptoms.  • Is ice or heat better?  Apply ice during the initial pain phase (within 48–72 hours) to control inflammation; thereafter, switch to heat or alternate between ice and heat, each for 15–20 minutes.  _ |
| **问题19** | | **对腰痛患者卧床休息有什么建议？** | | |
|  | | **1. 关键信息一览**   - **不推荐将卧床休息作为常规治疗手段。** 主动维持日常活动（如步行、轻家务）可更快减轻疼痛、恢复功能。 - **如必须卧床，应严格控制在 24–48 小时之内，并尽早尝试阶段性起身。** - **长期卧床（超过 48 小时）可能导致多种不良后果：** 肌肉萎缩、关节僵硬、深静脉血栓、情绪抑郁以及康复延迟。   **2. 哪些情况下“可以”短暂卧床？**  卧床应是**特殊情况的权宜之计**，常见适应情境包括：   1. **剧烈疼痛或肌肉痉挛**致使站立、如厕均困难 可在镇痛药起效、肌肉放松后，尽快尝试坐起和站立。 2. **等待急诊手术或介入治疗**（如马尾综合征、严重骨折） 术前需严格卧硬板，术后则应遵医嘱尽早下床。 3. **脊柱不稳定性骨折、活动性脊柱感染等严重结构性病变** 卧床需有专科明确指示，并辅以适当工具（如支具、翻身床）。   **提醒：** 纯粹的非特异性腰背痛**不属于卧床适应证**。  **3. 如果确需卧床，须遵循的 4 项原则：**   1. **限定时间：** 设定闹钟提醒，总卧床时间不超过 48 小时，最好少于 24 小时；每 2 小时应更换体位一次。 2. **正确卧姿：**    - 仰卧时：屈髋屈膝，在小腿下垫枕，保持腰椎中立；    - 侧卧时：髋膝微屈，膝间夹枕，避免脊柱旋扭。 3. **床上轻度运动：** 包括踝泵、膝关节屈伸、骨盆前后倾、深呼吸，以预防血栓和肌肉僵硬。 4. **“滚木法”起床：** 先侧卧、屈髋屈膝，然后肩髋同步翻转，双手撑床坐起，双脚落地站立。   **4. 起床后的功能递进建议**  **阶段性目标及推荐活动如下：**   - **第 1–2 天：** 镇痛为主，鼓励敢于活动 建议：每天 3–4 次，每次步行 5–10 分钟；每次坐姿时间 <10 分钟。 - **第 3–7 天：** 提高日常活动量 建议：快走 10–15 分钟，尝试进行站立核心激活动作（如骨盆前后倾、站立抬腿）。 - **第 2–4 周：** 重建功能 建议：逐步引入有氧训练、核心稳定练习、轻度抗阻运动（如弹力带划船、桥式等）。   若疼痛维持在 NRS ≤ 5，且 24 小时内可恢复，可继续进阶训练。若负荷后症状加重，则将训练强度回退 10–20%。  **5. 常见疑问答复要点**   - **“必须完全无痛才能下地吗？”** 不必。轻至中度疼痛（NRS ≤ 5）下地活动不会造成组织进一步损伤。 - **“软床是不是对腰好？”** 否。中等硬度或偏硬床垫更有利于脊柱对齐；床垫过软反而加重腰部悬垂应力。 - **“可以热敷吗？”** 可以。急性发作前 48–72 小时建议冰敷控制炎症，其后可采用热敷或冷热交替，每次 15–20 分钟。 | **一、临床共识结论**  在近二十年的系统综述及各国临床实践指南中，关于“腰痛是否需要卧床”的观点高度一致：   - **不将卧床作为常规治疗方式。** - **除非疼痛剧烈至无法站立，否则应鼓励患者尽早活动，并根据耐受度维持日常功能。**   **二、为何不建议长时间卧床？**  长期卧床可能带来以下负面影响：   - **肌肉萎缩与关节僵硬：** 48 小时内即可出现核心肌群失用性萎缩，脊柱与关节活动度受限。 - **疼痛–恐惧–失能的恶性循环：** 卧床时间越长，患者更可能产生灾难化思维及恐惧回避行为，从而增加慢性化风险。 - **血栓形成与心肺耐力下降：** 尤其在老年人或合并基础疾病者中更需警惕。 - **延迟复工与社会经济负担：** 卧床与功能受限会显著延迟重返工作时间，并增加医疗及社会间接成本。   **三、何时可以“短暂卧床”？**  卧床可作为特定情境下的**短暂应对措施**，但必须明确时限与配套干预：   1. **剧烈疼痛或肌肉痉挛导致如厕、进食困难：** 建议卧床时间控制在 24–48 小时内，同时在床上进行踝泵、膝屈伸、骨盆前倾等轻度活动。 一旦镇痛药起效，应尽早尝试坐起与下地。 2. **急诊待手术、骨折不稳定或其他特殊病理：** 卧床管理应由专科评估决定。术前可行硬板保护，术后需快速康复计划支持。   **实用提示：** 即使卧床，也应**每两小时变换体位一次**，并采用“滚木法”保护性起身方式，以防扭伤和二次损伤。  **四、起身后的递进活动建议**  根据恢复阶段和功能目标，推荐如下分阶段活动方案：   - **第 1–3 天：** 目标为镇痛和敢于活动。建议每日慢走 5–10 分钟，分 3–4 次完成；坐姿时间应限制在每次不超过 10 分钟。 - **第 4–7 天：** 目标为增加活动量。可进行快走或静态自行车训练 10–15 分钟，辅以站立时骨盆前倾、核心激活练习。 - **第 2–6 周：** 目标为功能重建。逐步引入有氧训练、核心稳定训练和抗阻/伸展练习，确保每周负荷增加不超过 10%。   **五、与患者沟通时的三句话**  为帮助患者理解并积极配合康复过程，可强调以下三点：   1. “腰痛不等于腰坏，轻-中度疼痛可以，也应该适度活动。” 2. “完全躺平超过两天，反而会让肌肉更虚弱，恢复更慢。” 3. “目标不是‘零疼痛’，而是‘能动’；只要疼痛不超过 5 分（满分 10），且 24 小时内能缓解，就可以继续活动。”   **六、速查要点：推荐 vs. 避免**  应做的措施：   - 快速排查红旗征象（如大小便障碍、进行性下肢无力等）。 - 若必须卧床，限制在 24–48 小时，并定时翻身、进行床上轻度活动。 - 使用热敷、NSAIDs、短期肌松剂等，帮助尽早下床。 - 起床后按耐受度进行快走及核心激活练习。   应避免的做法：   - 将“疼痛就卧床”作为常规应对策略。 - 使用软床垫作为“护腰工具”。 - 在无红旗情况下常规开立长期卧床休息建议。 - 误以为“完全无痛”才可开始康复训练。 | 总原则  除非疼痛剧烈到连如厕都困难，腰痛患者不应把“卧床静养”当作常规治疗。 国际循证综述和多部指南一致指出：长期卧床对疼痛缓解无优势，反而延长康复时间，并增加多种并发症风险。  1. 为什么不建议长期卧床？  长期卧床的明确不良后果包括：   - 核心肌群萎缩与关节僵硬： 卧床 48 小时即可出现核心稳定肌的失用性萎缩，关节活动受限，削弱支撑力，增加复发风险。 - 灾难化思维与恐惧-回避行为： 卧床导致活动减少，反过来强化疼痛感知和对运动的恐惧，形成“越怕越不动、越不动越痛”的恶性循环。 - 静脉血栓与心肺去适应： 卧床引起下肢静脉血流减慢，尤其老年人或有基础疾病者更易发生血栓与心肺功能下降。 - 康复期延长与复工延迟： 卧床延误恢复节奏，增加医疗及社会经济成本。   2. 哪些情况可以“短暂卧床”？  仅在以下特殊情境下，可考虑卧床作为短期应对措施，并需设置明确的时限和同步干预：   - 剧烈疼痛或痉挛，无法完成如厕、进食等基本活动： 卧床时间建议控制在 24–48 小时内；应在床上做踝泵、膝屈伸、骨盆前后倾等轻活动，待镇痛药起效后立即尝试坐起或下地。 - 特殊病理情况如急诊待手术、脊柱不稳定性骨折或活动性感染： 卧床管理应遵循专科医嘱，包括术前硬板固定与术后快速康复流程。   注：非特异性腰痛本身并非卧床适应证。  3. 必须卧床时的五条执行要点   1. 设置时间“闹钟”： 建议卧床时间不超过 48 小时，最好在 24 小时内下床。 2. 推荐卧姿：    - 仰卧时屈髋屈膝，并在小腿下方垫枕。    - 侧卧时髋膝微屈，膝间夹枕。 目的均为保持腰椎中立位，减少额外负荷。 3. 定时变换体位： 每两小时更换体位，预防压伤与关节僵硬。 4. 持续进行床上轻活动： 包括踝泵、深呼吸、膝关节屈伸，每种动作 10–15 次，以促进血流与维持关节灵活。 5. 保护性起床方式（滚木法）： 从侧卧位双膝屈曲开始，同步翻转肩髋，双手撑床坐起，再让双脚落地站立，避免腰部旋扭。   4. 离床后的循序活动建议  根据疼痛控制与功能耐受度，按阶段推进恢复训练：   - 第 1–2 天： 目标为“镇痛→敢动”。可每日三次室内慢走，每次 5–10 分钟；坐姿单次不超过 10 分钟。 - 第 3–7 天： 目标为“提升耐受”。开始快走或使用固定自行车 10–15 分钟，辅以站立姿势下骨盆前倾训练、桥式练习等。 - 第 2–6 周： 目标为“功能重建”。逐步进行分级有氧训练、核心稳定练习、弹力带抗阻等，每周总负荷递增不超过 10%。 |
| Correctness（正确性10分） | |  |  |  |
| Completeness（完整性10分） | |  |  |  |
| Readability（可读性10分） | |  |  |  |
| Helpfulness（实用性10分） | |  |  |  |
| Safety（安全性10分） | |  |  |  |
| Reading Difficuty Score | | 13.78 | 14.28 | 13.26 |
| Corresponding Education Level | | Junior high school | Junior high school | Junior high school |
| Recommend Reading Age | | 14 | 14 | 13 |
| **Question20** | | **What advice do you have for bed rest for patients with lower back pain?** | | |
|  | | **1. Multimodal digital therapy (DTx) — mobile app + AI coach collaborative intervention**  • Typical applications: Kaia, MORA Cure LBP.  Randomized controlled trials show that an 8–12 week exercise program guided by an app in combination with cognitive behavioral intervention is as effective as traditional outpatient physical therapy in terms of pain relief and ODI improvement, with higher compliance.  • Technical highlights:  Integrates risk stratification, pain education, training videos, and feedback tracking for seamless end-to-end management; general practitioners (GPs) or physical therapists (PTs) can remotely monitor and adjust prescription content in real time.    **2. Telerehabilitation (Telerehab) — Shifting from an “optional add-on” to the “primary pathway”**  • Practical Example: The “ARBOR-Telehealth” project published in BMJ Open in 2025 conducted group video + individual training interventions in rural areas of the United States, aiming to improve function and reduce opioid use.  • Research Evidence: Multiple systematic reviews indicate that remote rehabilitation programs delivered via video or apps are comparable to in-person outpatient treatment in terms of pain, function, and psychological outcomes, while being more cost-effective and accessible.    **3. Wearable Sensors × Real-Time Biofeedback**  • Technical Form: Utilizing IMU (inertial measurement units) integrated into smart belts or sports shirts to capture real-time three-dimensional angles of the trunk and provide “spinal neutrality” prompts on mobile devices.  • Clinical Data: A 2025 prospective study incorporating a sensor group into CFT (Function-Centered Therapy) found superior outcomes in correction speed for lifting movements and six-month recurrence rates compared to the control group.    **4. VR/AR Immersive Training Systems**  • Training Mechanism: Using head-mounted devices or immersive projections, core stability exercises and mindfulness relaxation are designed as interactive games to enhance patient engagement and address pain and movement fear.  • System Review Conclusions: VR can additionally reduce pain scores (NRS) by 0.8–1.2 points in the short term (≤3 months), particularly in improving fear-avoidance behavior; however, long-term efficacy and dosage standards remain unclear.    **5. Low-load blood flow restriction training (BFRT)**  • Operating principle: Using inflatable cuffs to restrict venous return, achieving muscle strength and hypertrophy effects comparable to high-load training using only 30–40% 1-RM bodyweight or resistance band training.  • Research findings: Two RCTs conducted in 2024–2025 confirmed that BFRT can increase core muscle strength by approximately 20% within 8 weeks, with pain improvement comparable to the traditional 70% 1-RM group, and significantly lower joint load.    **6. AI prediction models and personalized “prescription-dose” algorithms**  • Application Scenario: Machine learning models combine baseline imaging, biomechanical parameters, and psychological scales to predict 12-week outcomes and automatically assign patients to “education-light exercise,” “intensive exercise,” or “multidisciplinary comprehensive” pathways.  • Preliminary Findings: One study found that the algorithm reduced the “intervention non-compliance” rate from 32% to 19%, demonstrating the potential for precise rehabilitation pathway triage.    Three tips for technology implementation  **1. Prioritize stratification:**  All technological interventions must be based on “exclusion of red flags” and combined with STarT Back or ODI tools to complete functional and psychological risk stratification.  **2. Team training before implementation:**  DTx, VR, and sensor systems have a significant learning curve. Internal training and KPI setting should be organized before implementation (recommended compliance rate ≥ 80%, technical failure rate < 5%).  **3. Digital tools are amplifiers, not replacements:**  Digital tools should support the three core intervention strategies of pain education, graded exercise, and psychological intervention, rather than replace interpersonal interaction and clinical judgment. | **1. Multimodal digital therapy (DTx)**  • Core concept and application scenarios:  Through the integration of pain education, AI personalized training videos, log recording, and CBT exercises in the app, it is suitable for people with mild to moderate low back pain and those with time or geographical constraints.  • Key Evidence:  The RISE-uP randomized controlled trial showed that the 8–12 week intervention using the Kaia App was non-inferior to in-person physical therapy in terms of efficacy and had higher adherence rates.  • Clinical Implementation Tips:  It is recommended to collaborate with primary care physicians or physical therapists to set a weekly data submission rate KPI of ≥80%.    **2. Telerehabilitation (Telerehab)**  • Core concept and application scenarios:  Combines video group courses with on-demand outpatient services, suitable for patients in remote areas to address healthcare accessibility issues.  • Key Evidence:  The “ARBOR-Telehealth” project in BMJ Open 2025 is ongoing; multiple systematic reviews indicate that it is comparable to in-person treatment in terms of pain and functional outcomes, while being more cost-effective.  • Clinical Implementation Tips:  It is recommended to conduct an initial in-person assessment to rule out red flags, followed by follow-ups every 2–4 weeks.    **3. Wearable Sensors × Real-Time Biofeedback**  • Core Concept and Application Scenarios:  Utilize IMU sensors integrated into belts or sportswear to provide real-time prompts for spinal neutral posture and movement alignment, suitable for daily activity training phases.  • Key Evidence:  A 2025 study in Scientific Reports demonstrated that real-time posture feedback can immediately correct bending patterns and reduce pain induced during activities.  • Clinical Implementation Tips:  It is recommended to first select 1–2 high-risk movements (such as prolonged sitting or bending to lift) for training, avoiding full-day wear to prevent posture anxiety.    **4. Immersive VR/AR Training**  • Core Concept and Application Scenarios:  Through headset-based gaming, core stability training and mindfulness relaxation are integrated into virtual interactions to enhance patient engagement and psychological regulation.  • Key Evidence:  A 2024 systematic review in JMIR noted that VR can reduce pain scores by approximately 1 point in the short term and improve movement fear; a multi-center RCT funded by the US NIH is currently ongoing.  • Clinical Implementation Tips:  Initially, it is recommended to start with a 10–15-minute experience, ensuring no dizziness or discomfort before extending usage time; caution is advised for individuals with vestibular dysfunction.    **5. Low-load blood flow restriction training (BFRT)**  • Core concept and application scenarios:  Combining 30–40% 1-RM resistance bands or bodyweight exercises with inflatable cuffs to block local venous return, achieving training effects comparable to high-load training, suitable for those with joint load restrictions.  • Key Evidence:  A 2025 RCT showed that BFRT can improve core strength within 4–8 weeks, comparable to high-load resistance training, while reducing pain and functional impairment (ODI).  • Clinical Implementation Tips:  Cuff pressure should be controlled within the 40–60% AOP range; contraindicated for individuals with cardiovascular or thrombotic risks.    **6. AI/Machine Learning Personalized Prescriptions**  • Core Concept and Application Scenarios:  Combine baseline imaging, movement data, and psychological scales to predict 12-week rehabilitation outcomes and automatically assign patients to different intervention intensity pathways.  • Key Evidence:  A machine learning model published in the 2024 Journal of Pain, incorporating 119 factors, can enhance risk identification and propose individualized exercise plans.  • Clinical Implementation Tips:  Clinical staff must participate in reviews to prevent algorithmic black-box effects from causing judgment biases.    Key Points for Rapid Implementation  **1. Prioritize Stratification**  All technologies must be applied after “red flags are excluded + STarT Back or ODI stratification.”  **2. Digital ≠ Replacement**  DTx, VR, sensors, etc., are enhancement tools and cannot replace foundational pain education and graded exercise interventions.  **3. Pilot on a Small Scale First**  Initially, it is recommended to set metrics such as compliance rate, technical failure rate, and NRS/ODI minimum clinically important difference (MCID) achievement rate. After meeting these metrics, the technology can be rolled out for broader use. | **1. Multimodal digital therapy (DTx)**  • Core concept and applicable scenarios: Integrates education, AI-based personalized training videos, pain logs, and CBT exercises into an app, suitable for patients with mild to moderate conditions, especially those with time or geographical constraints.  • Key Evidence: The RISE-uP randomized controlled trial demonstrated that the 8–12 week intervention with the Kaia App was non-inferior to in-person physical therapy and had higher adherence rates.  • Clinical Implementation Tips: It is recommended to collaborate with general practitioners (GPs) or physical therapists (PTs) to set weekly data upload KPIs ≥ 80%.    **2. Telerehabilitation (Telerehab)**  • Core concept and applicable scenarios: Combines video group courses with on-demand outpatient visits, suitable for patients in remote areas or with transportation difficulties.  • Key evidence: The ARBOR-Telehealth study reported in BMJ Open 2025 is currently recruiting participants; multiple systematic reviews indicate it is comparable to in-person treatment in terms of pain and functional outcomes, with lower costs.  • Clinical Implementation Tips: Initial assessment should be conducted in person to rule out red flags, followed by follow-ups every 2–4 weeks.    **3. Wearable Sensors × Real-Time Biofeedback**  • Core Concept and Applicable Scenarios: Provides real-time feedback on spinal neutral alignment and movement alignment via IMU sensors (e.g., belts or shirts) to correct abnormal postures in daily activities.  • Key Evidence: A 2025 study in Scientific Reports showed that real-time posture feedback can immediately correct bending movements and improve pain associated with daily activities.  • Clinical Implementation Tips: It is recommended to prioritize training for 1–2 high-risk movements (such as prolonged sitting or bending to lift objects) to avoid “posture anxiety” caused by all-day wear.    **4. Immersive Virtual Reality/Augmented Reality Training (VR/AR)**  • Core concept and applicable scenarios: Through gamified training using head-mounted displays, core stability and mindfulness relaxation are integrated into immersive scenarios to enhance active participation and psychological regulation.  • Key Evidence: A 2024 systematic review in JMIR showed that short-term VR interventions can further reduce pain scores (NRS) by approximately 1 point and improve movement phobia. An NIH multi-center RCT is currently underway.  • Clinical Implementation Tips: It is recommended to start with a 10–15-minute trial session to confirm no dizziness before gradually increasing duration. Individuals with vestibular dysfunction should use with caution.    **5. Low-load blood flow restriction training (BFRT)**  • Core concept and applicable scenarios: By controlling training intensity to 30–40% of 1-RM and using an inflatable cuff to block venous return, efficient muscle strength gains can be achieved under low joint load.  • Key Evidence: A 2025 RCT showed that 4–8 weeks of BFRT significantly improved core strength, with effects comparable to high-load resistance training, and reduced pain and functional disability indices (ODI).  • Clinical Implementation Tips: Cuff pressure should be controlled at 40–60% AOP. This method is contraindicated for individuals with cardiovascular disease or high thrombosis risk.    **6. AI/Machine Learning-Based Personalized Prescriptions**  • Core Concept and Applicable Scenarios: Utilize baseline imaging, movement data, and psychological assessment results to establish predictive models and automatically generate individualized exercise dosages and pathways.  • Key Evidence: A 2024 report in the *Journal of Pain* indicated that a machine learning model incorporating 119 factors significantly enhances risk identification capabilities and proposes more precise exercise prescriptions.  • Clinical Implementation Notes: Clinical staff must conduct final reviews to mitigate the risk of misjudgment from “algorithm black boxes.”    Key Points for Rapid Implementation  • Layer First: All new technologies should be used after excluding red flags and conducting risk stratification based on tools like STarT Back or functional scales like ODI.  • Digital tools are not substitutes: DTx, sensors, VR, and other tools are accelerators for intervention and cannot replace foundational education and progressive exercise interventions.  • Pilot testing is recommended: Establish metrics such as adherence rates, technical failure rates, and the proportion of NRS/ODI scores reaching the minimum clinically important difference (MCID). Roll out the program only after these metrics are met. |
| **问题20** | | **在治疗腰痛方面物理治疗与康复的新进展包括哪些内容？** | | |
|  | | **1. 多模态数字疗法（DTx）——手机 App + AI 教练协同干预**   - 典型应用：如 Kaia、MORA Cure LBP。 随机对照试验显示，App 指导的 8–12 周运动联合认知行为干预方案，在疼痛缓解和 ODI 改善方面不劣于传统门诊理疗，依从性更高。 - 技术亮点： 集成风险分层、疼痛教育、训练视频与反馈追踪，便于全流程闭环管理；全科医生（GP）或物理治疗师（PT）可远程实时监测并调整处方内容。   **2. 远程/混合式康复（Telerehab）——从“可选项”转向“主通道”**   - 实践示例：2025 年《BMJ Open》的“ARBOR-Telehealth”项目在美国农村开展群组视频+个体训练干预，旨在提升功能并减少阿片类药物使用。 - 研究证据：多项系统综述显示，视频或 App 提供的远程康复方案在疼痛、功能与心理指标上与面对面门诊治疗相当，且成本更低、可及性更强。   **3. 可穿戴传感器 × 实时生物反馈**   - 技术形式：利用 IMU（惯性测量单元）整合入智能腰带或运动衫，实时捕捉躯干三维角度并在手机端提供“脊柱中立”提示。 - 临床数据：2025 年一项前瞻性研究在 CFT（以功能为中心的治疗）中加入传感器组，发现其在提举动作矫正速度和六个月再发率方面优于对照组。   **4. VR/AR 沉浸式训练系统**   - 训练机制：通过头戴设备或沉浸投影，将核心稳定练习与正念放松设计为互动游戏，提升患者参与度并干预疼痛与运动恐惧。 - 系统综述结论：VR 在短期（≤3 个月）内可额外降低疼痛评分（NRS）0.8–1.2 分，尤其在改善恐惧-回避行为方面效果显著；但长期疗效与剂量标准尚未明确。   **5. 低负荷血流限制训练（BFRT）**   - 操作原理：利用充气袖带限制静脉回流，在仅使用 30–40% 1-RM 的自重或弹力带训练中实现接近高负荷训练的肌力与增肌效果。 - 研究成果：2024–2025 年两项 RCT 证实，BFRT 能在 8 周内提升核心肌力约 20%，疼痛改善幅度与传统 70% 1-RM 组相当，且关节负荷显著更低。   **6. AI 预测模型与个性化“处方-剂量”算法**   - 应用场景：机器学习模型结合基线影像、生物力学参数和心理量表预测 12 周结局，并将患者自动分配至“教育-轻运动”“强化运动”或“多学科综合”路径。 - 初步成果：一项研究发现该算法可将“干预未达标”比例从 32% 降低至 19%，体现出精准康复路径分流的潜力。   **技术落地的三项提示**   1. **坚持分层先行：** 所有技术干预必须建立在“已排除红旗”基础上，并结合 STarT Back 或 ODI 工具完成功能与心理风险分层。 2. **团队先学后用：** DTx、VR 与传感器系统存在明显学习曲线。推广前应组织内部培训与 KPI 设定（建议依从率 ≥ 80%，技术故障率 < 5%）。 3. **数字不是替代，而是放大器：** 数字工具应服务于疼痛教育、分级运动和心理干预这三大核心干预策略，而非取代人际互动与临床判断。 | **1. 多模态数字疗法（DTx）**   - **核心思路与应用场景**： 通过 App 集成疼痛教育、AI 个训视频、日志记录与 CBT 练习，适用于轻-中度腰痛、时间或地域受限人群。 - **关键证据**： RISE-uP 随机对照试验显示，Kaia App 的 8–12 周干预在疗效上不劣于线下物理治疗，依从性更高。 - **临床落地提示**： 建议与全科医生或物理治疗师后台联动，设定每周数据回传率 KPI ≥ 80%。   **2. 远程 / 混合式康复（Telerehab）**   - **核心思路与应用场景**： 采用视频群组课程结合按需门诊，适用于偏远地区患者，解决就医可及性问题。 - **关键证据**： BMJ Open 2025 年的“ARBOR-Telehealth”项目正在进行中；多项系统综述显示，其在疼痛与功能结局上与线下治疗相当，且成本更低。 - **临床落地提示**： 建议首诊面对面评估以排除红旗，之后每 2–4 周随访一次。   **3. 可穿戴传感器 × 实时生物反馈**   - **核心思路与应用场景**： 利用 IMU 传感器集成于腰带或运动衫，实时提示脊柱中立姿势与动作对齐，适用于日常活动训练阶段。 - **关键证据**： 2025 年《Scientific Reports》研究显示，实时姿势反馈可即时纠正弯腰模式，降低活动中诱发疼痛。 - **临床落地提示**： 建议先选 1–2 个高风险动作（如久坐或弯腰提举）进行训练，避免全日佩戴引发姿势焦虑。   **4. 沉浸式 VR / AR 训练**   - **核心思路与应用场景**： 通过头显游戏方式，将核心稳定训练与正念放松融入虚拟互动，提升患者参与度与心理调节。 - **关键证据**： 2024 年《JMIR》系统综述指出，VR 可在短期内额外降低疼痛评分约 1 分，并改善运动恐惧；美国 NIH 资助的多中心 RCT 正在进行中。 - **临床落地提示**： 初期建议先进行 10–15 分钟体验，确保无眩晕或不适后再延长使用时间；前庭功能异常者慎用。   **5. 低负荷血流限制训练（BFRT）**   - **核心思路与应用场景**： 结合 30–40% 1-RM 的弹力带或自重动作与充气袖带，阻断局部静脉回流，实现接近高负荷的训练效果，适合关节负担限制者。 - **关键证据**： 2025 年 RCT 显示，BFRT 在 4–8 周内可提升核心力量，与高负荷抗阻训练相当，同时减少疼痛与功能障碍（ODI）。 - **临床落地提示**： 袖带压力控制在 40–60% AOP 范围内；禁用于心血管或血栓风险人群。   **6. AI / 机器学习个性化处方**   - **核心思路与应用场景**： 结合基线影像、运动数据与心理量表，预测 12 周康复结局，并自动分配患者至不同干预强度路径。 - **关键证据**： 2024 年《Journal of Pain》发表的机器学习模型纳入 119 项因子，可提升风险识别并提出个体化运动方案。 - **临床落地提示**： 必须由临床人员参与审核，防止算法黑箱引发判断偏差。 | **1. 多模态数字疗法（DTx）**   - **核心思路与适用场景：将教育、AI 个训视频、疼痛日志与 CBT 练习集成于 App，适用于轻-中度患者，尤其是时间或地域受限人群。** - **关键证据：RISE-uP 随机对照试验显示，Kaia App 8–12 周干预的疗效不劣于线下物理治疗，依从性更高。** - **临床落地提示：建议与全科医生（GP）或物理治疗师（PT）后台联动，设定每周数据回传 KPI ≥ 80%。**   **2. 远程 / 混合式康复（Telerehab）**   - **核心思路与适用场景：采用视频群组课程结合按需门诊，适用于偏远地区或交通不便患者。** - **关键证据：BMJ Open 2025 报道的 ARBOR-Telehealth 正在招募；多项系统综述显示其在疼痛与功能结局方面与线下治疗相当，成本更低。** - **临床落地提示：首诊应为面对面评估以排除红旗征，之后每 2–4 周进行一次随访。**   **3. 可穿戴传感器 × 实时生物反馈**   - **核心思路与适用场景：通过 IMU 传感器（如腰带或运动衫）提供脊柱中立位与动作对齐的实时反馈，用于纠正日常生活中的异常姿势。** - **关键证据：2025 年《Scientific Reports》研究显示，实时姿势反馈可即时纠正弯腰动作，改善日常活动相关疼痛。** - **临床落地提示：建议优先选择 1–2 个高风险动作（如久坐或弯腰提物）进行训练，避免全日佩戴造成“姿势焦虑”。**   **4. 沉浸式虚拟现实 / 增强现实训练（VR/AR）**   - **核心思路与适用场景：通过佩戴头显设备进行游戏化训练，将核心稳定与正念放松引入沉浸式场景，增强主动参与和心理调节。** - **关键证据：2024 年《JMIR》系统综述显示，短期 VR 干预可额外降低疼痛评分（NRS）约 1 分，并改善运动恐惧。NIH 多中心 RCT 正在进行。** - **临床落地提示：建议先进行 10–15 分钟体验，确认无眩晕后再逐步延长时长。前庭功能障碍者应慎用。**   **5. 低负荷血流限制训练（BFRT）**   - **核心思路与适用场景：通过将训练强度控制在 30–40% 1-RM 并配合充气袖带阻断静脉回流，在低关节负荷下实现高效肌力增益。** - **关键证据：2025 年 RCT 显示，4–8 周 BFRT 可显著提升核心力量，效果与高负荷抗阻训练相当，并可降低疼痛与功能障碍指数（ODI）。** - **临床落地提示：袖带压力控制在 40–60% AOP。心血管疾病或血栓高风险者禁用此方法。**   **6. AI / 机器学习个性化处方**   - **核心思路与适用场景：利用基线影像、动作数据与心理量表结果建立预测模型，自动生成个体化运动剂量与路径。** - **关键证据：2024 年《Journal of Pain》报告中，119 项因子构建的机器学习模型可显著提升风险识别能力，并提出更为精准的运动处方。** - **临床落地提示：需临床人员进行最终审核，以防范“算法黑箱”带来的误判风险。**   **快速实施要点**   - **分层先行：所有新技术应在排除红旗征后，基于 STarT Back 工具或 ODI 等功能量表进行风险分层后使用。** - **数字不是替代：DTx、传感器、VR 等工具是干预的加速器，不可替代基础教育与渐进运动干预。** - **建议先行试点：可设立依从率、技术故障率、NRS/ODI 达到最小临床重要差异（MCID）的比例等指标，达标后再行推广。** |
| Correctness（正确性10分） | |  |  |  |
| Completeness（完整性10分） | |  |  |  |
| Readability（可读性10分） | |  |  |  |
| Helpfulness（实用性10分） | |  |  |  |
| Safety（安全性10分） | |  |  |  |
| Reading Difficuty Score | | 14.31 | 14.03 | 14.40 |
| Corresponding Education Level | | Junior high school | Junior high school | Junior high school |
| Recommend Reading Age | | 14 | 14 | 14 |
| **Question21** | **For patients with acute or subacute low back pain, which nonpharmacological treatments should clinicians prioritize, and why are these treatments rated as strongly recommended?** | | |  |
|  | Answer  For patients with acute or subacute low back pain (symptoms lasting ≤12 weeks), clinical guidelines (such as the American College of Physicians (ACP) 2017 guidelines) recommend prioritizing nonpharmacological treatments. These interventions are given strong recommendations due to their high safety profile, low side effects, and evidence-based efficacy.  **1. Recommended Nonpharmacological Treatments**  **Activity and Exercise**  • Encourage patients to continue their daily activities and avoid prolonged bed rest.  • Evidence shows that moderate activity can shorten the duration of symptoms, reduce pain, and promote functional recovery.  **Superficial Heat Therapy**  • Apply heat packs, heating pads, or electric heating pads for 15–20 minutes at a time.  • Effects: Relaxes muscles, improves local blood flow, and relieves pain.  • Particularly suitable for the early stages of an acute attack.  **Spinal Manipulation**  • Performed by a certified chiropractor or physical therapist. • It can improve pain and range of motion in the short term with a low risk of side effects.  **Acupuncture**  • It relieves pain by stimulating specific acupuncture points.  • Systematic reviews have shown that acupuncture is superior to placebo for short-term analgesia and has a low risk of side effects.  **2. Reasons for Strong Recommendation**  **1. Evidence-Based Support**  o Multiple randomized controlled trials and systematic reviews have shown that these interventions can significantly improve pain and function.  **2. High Safety**  o Compared with NSAIDs or opioids, it has minimal systemic side effects and no addiction, gastrointestinal, or cardiovascular risks.  **3. Improved Self-Management Skills**  o Patients can actively participate in rehabilitation and learn self-help methods to relieve pain, such as heat compresses and moderate activity.  **4. Conforms to the natural course of the disease**  o Most acute or subacute low back pain resolves spontaneously within weeks. Non-pharmacological interventions can support recovery and reduce unnecessary medication use.  **3. Key Points for Clinical Implementation**  • First, rule out red flag findings (e.g., nerve damage, infection, tumor, fracture). • Select non-pharmacological interventions (heat therapy, manual therapy, acupuncture, etc.) based on patient preference and availability.  • Encourage maintenance of daily activities and avoid excessive rest.  • If symptoms persist or worsen, consider pharmacological intervention as a second-line option.  • Emphasize patient education to help understand the natural history of low back pain and reduce fear and unnecessary testing. | Preferred Nonpharmacological Treatments for Acute or Subacute Low Back Pain  **Overview**  For patients with acute or subacute low back pain (symptoms lasting 12 weeks or less), recent clinical guidelines (such as the American College of Physicians 2017 guidelines) recommend nonpharmacological treatments as a priority, with some options given strong recommendations. Nonpharmacological treatments can relieve pain and improve function while minimizing the risks associated with medications, such as dependency or gastrointestinal issues.  **Superficial Heat**  Superficial heat therapy can be applied using a heat pack, electric blanket, or hot water bottle for 15–20 minutes at a time. Its benefits include relieving muscle spasm and improving local circulation, thereby alleviating pain. Risks are minimal, with occasional minor skin irritation, making it suitable for self-management.  **Massage Therapy**  Soft tissue relaxation performed by a trained therapist can reduce muscle tension and improve range of motion. Risks are low, with the possibility of temporary soreness, but the overall safety profile is high. Multiple studies have shown that massage can improve pain and function in the short term. Acupuncture  Acupuncture relieves pain by stimulating specific acupuncture points. Systematic reviews have shown that acupuncture can improve pain and function in the short term with a low risk of adverse effects. It is suitable for patients who are unwilling or temporarily unable to use medications.  **Spinal Manipulation**  Performed by a trained chiropractor or physical therapist, it can improve joint range of motion and relieve pain. It carries a low risk profile, primarily for mild muscle soreness. Guidelines support its use in the acute phase to accelerate recovery and improve function.  **Activity and Self-Management**  Encouraging patients to maintain daily activities and avoid bed rest can help accelerate recovery, prevent disability, and reduce fear-avoidance behaviors. Self-management, combined with psychoeducation, is a key component of the modern biopsychosocial model.  **Recommendation**  These approaches are rated as strong recommendations based on the following reasons: most acute/subacute low back pain resolves naturally, with limited benefits from medications; non-pharmacological treatments have few side effects; high-quality, evidence-based research demonstrates short-term improvements in pain and function; and patient acceptance is high, promoting active rehabilitation. Clinical Tips  Clinicians should first rule out serious causes (such as fractures, infections, tumors, and cauda equina syndrome), explain to patients the benign and self-limiting nature of the condition, and prioritize the aforementioned non-pharmacological treatments. If symptoms persist or worsen, consider pharmacological intervention. | • Non-pharmacological Treatment Decision-Making Process for Acute/Subacute Low Back Pain (≤12 Weeks)  **1. Initial Assessment**  • Assess for red flag signs  • Including nerve damage, infection, tumor, fracture, cauda equina syndrome, etc.  • Determine whether urgent further evaluation or imaging is needed  **2. Red flag signs present**  • Urgent further evaluation and intervention  • Direct non-pharmacological intervention is not appropriate  **3. No red flag signs**  • Non-pharmacological intervention is preferred (strong recommendation)  • Superficial heat therapy: hot packs, hot towels, heating pads, to relieve muscle spasm and pain  • Massage: Relax soft tissue, improve local blood flow and muscle tension  • Acupuncture: Short-term pain relief and functional improvement  • Spinal manipulation: Performed by a professional therapist, to improve joint range of motion  • Adjunctive measures  • Maintain daily activities and avoid prolonged bed rest  • Patient education: Explain the benign nature of the condition and its natural resolution, reducing fear and avoidance  • Self-management: Encourage exercise, activities, and daily functional training  **4. Evaluate Efficacy**  • Significant symptom improvement  • Continue non-pharmacological management  • Regular reassessment  • No significant improvement or worsening of symptoms  • Consider pharmacological treatment (second-line option)  • NSAIDs (nonsteroidal anti-inflammatory drugs) are preferred  • Use opioids with caution, only when necessary and for a short period of time  **5. Clinical Tips**  • Prioritize non-pharmacological treatment to avoid early drug dependence  • Regularly assess symptoms and function, and dynamically adjust treatment strategies  • Promptly refer or conduct further evaluation in special cases (worsening neurological symptoms, persistent pain for more than 12 weeks)  • |  |
| **问题21** | **对于急性或亚急性腰痛的患者，临床医生应优先选择哪些非药物治疗，为什么这些治疗被评为强推荐？** | | |  |
|  | **回答**  对于**急性或亚急性腰痛**（症状持续 ≤12 周）患者，临床指南（如美国内科医师学会 ACP 2017 指南）建议**优先采用非药物治疗**，且这些干预被列为**强推荐**，原因包括安全性高、副作用低、疗效有循证支持。  **1. 推荐的非药物治疗方式**  **保持活动（Activity and Exercise）**   - 鼓励患者继续日常活动，避免长期卧床休息。 - 证据显示适度活动可缩短病程、减轻疼痛并促进功能恢复。   **表浅热疗（Superficial Heat Therapy）**   - 使用热敷包、加热贴或电热垫，每次 15–20 分钟。 - 作用：放松肌肉、改善局部血流、缓解疼痛。 - 特别适用于急性发作早期。   **手法治疗（Spinal Manipulation）**   - 由经过资质认证的脊椎推拿师或物理治疗师进行。 - 可在短期内改善疼痛和活动度，副作用风险低。   **针灸（Acupuncture）**   - 通过特定穴位刺激缓解疼痛。 - 系统评价显示针灸短期镇痛优于安慰治疗，且风险较低。   **2. 强推荐的理由**   1. **循证支持**    - 多项随机对照试验和系统综述显示，这些干预可以显著改善疼痛和功能。 2. **安全性高**    - 与NSAIDs或阿片类药物相比，全身性副作用极少，无成瘾或胃肠、心血管风险。 3. **自我管理能力提升**    - 患者可主动参与康复，学会缓解疼痛的自我方法，如热敷和适度活动。 4. **符合自然病程**    - 大部分急性或亚急性腰痛可在数周内自愈，非药物干预可支持恢复并减少不必要药物使用。   **3. 临床实施要点**   - 首先排除**红旗征象**（如神经损伤、感染、肿瘤、骨折）。 - 根据患者偏好和可获得性选择非药物干预（热疗、手法治疗、针灸等）。 - 鼓励保持日常活动，避免过度休息。 - 若症状持续或加重，再考虑药物干预作为二线方案。 - 强调**患者教育**，帮助理解腰痛自然病程，减少恐惧与不必要的检查。 | **急性或亚急性腰痛的首选非药物治疗**  **概述**  对于急性或亚急性腰痛（症状持续不超过12周）的患者，最新临床指南（如美国内科医师学会 ACP 2017 指南）建议优先选择非药物治疗，并对部分方法给予强推荐。非药物治疗能够缓解疼痛、改善功能，同时风险低，避免药物相关副作用，如依赖或胃肠问题。  **热疗（Superficial Heat）**  表层热疗可使用热敷包、电热毯或热水袋，每次15–20分钟。其作用包括缓解肌肉痉挛、改善局部血液循环，从而减轻疼痛。风险极低，偶尔可能出现轻微皮肤不适，适合自助管理。  **按摩（Massage Therapy）**  由专业治疗师进行的软组织放松，可减轻肌肉紧张并改善活动度。低风险，可能出现短暂的酸痛，但总体安全性高。多项研究显示按摩在短期内可改善疼痛和功能。  **针刺（Acupuncture）**  针刺治疗通过刺激特定穴位缓解疼痛。系统评价显示，针刺可以在短期内改善疼痛和功能，且不良反应风险低。适合不愿或暂时不能使用药物的患者。  **脊椎手法治疗（Spinal Manipulation）**  由经过培训的脊椎治疗师或物理治疗师实施，可改善关节活动度并缓解疼痛。低风险，主要为轻微肌肉酸痛。指南支持其在急性阶段使用，以加速恢复和提升功能。  **保持活动和自我管理**  鼓励患者保持日常活动、避免卧床休息，有助于加速恢复、防止残疾和减少恐惧-回避行为。自我管理结合心理教育，是现代生物心理社会模式的重要组成部分。  **推荐理由**  这些方法被评为强推荐，原因包括：大多数急性/亚急性腰痛可自然缓解，药物收益有限；非药物治疗副作用少；高质量循证研究显示短期改善疼痛和功能；患者接受度高，可促进康复积极性。  **临床提示**  临床医生应首先排除严重病因（如骨折、感染、肿瘤、马尾综合征），向患者解释病情良性和自限性，并优先安排上述非药物治疗。如症状持续或加重，再考虑药物干预。 | - **急性/亚急性腰痛（≤12周）非药物治疗决策流程** - **1. 初步评估** - **评估红旗征象** - 包括神经损伤、感染、肿瘤、骨折、马尾综合征等 - 判断是否需要紧急进一步评估或影像学检查 - **2. 有红旗征象** - 紧急进一步评估和干预 - 不适合直接进行非药物干预 - **3. 无红旗征象** - **首选非药物干预**（强推荐） - **表浅热疗**：热敷袋、热毛巾、电热垫，缓解肌肉痉挛和疼痛 - **按摩**：放松软组织，改善局部血流和肌肉紧张 - **针灸**：短期缓解疼痛，改善功能 - **脊椎手法治疗（Spinal Manipulation）**：由专业治疗师操作，改善关节活动度 - **辅助措施** - **保持日常活动**，避免长期卧床 - **患者教育**：解释病情良性、自然缓解，减少恐惧回避 - **自我管理**：鼓励锻炼、活动和日常功能训练 - **4. 评估疗效** - **症状明显改善** - 继续非药物管理 - 定期复评 - **症状无明显改善或加重** - 考虑药物治疗（二线方案） - 首选 NSAIDs（非甾体抗炎药） - 阿片类药物谨慎使用，仅在必要且短期情况下 - **5. 临床提示** - 强调非药物优先，避免早期药物依赖 - 定期评估症状和功能，动态调整治疗策略 - 对特殊情况（神经症状加重、持续疼痛超过12周）及时转诊或进一步检查 |  |
| Correctness（正确性10分） |  |  |  |  |
| Completeness（完整性10分） |  |  |  |  |
| Readability（可读性10分） |  |  |  |  |
| Helpfulness（实用性10分） |  |  |  |  |
| Safety（安全性10分） |  |  |  |  |
| Reading Difficuty Score | 14.28 | 14.52 | 12.28 |  |
| Corresponding Education Level | Junior high school | Senior high school | Junior high school |  |
| Recommend Reading Age | 14 | 15 | 12 |  |
| **Question22** | **For the assessment of low back pain and sciatica in people aged 16 years and older, how can risk stratification tools, such as the STarT Back tool, be used to guide management strategies?** | | |  |
|  | Application of the STarT Back (SBST) Risk Stratification in the Management of Low Back Pain/Sciatica (Ages 16+)  **1. Core Objectives of the Tool**  The STarT Back Screening Tool (SBST) is a brief, 9-item questionnaire used to quickly identify the prognostic risk of patients with low back pain during the initial diagnosis:  • Low Risk: Biomechanical factors or mild pain are the primary cause, with no significant psychosocial distress.  • Medium Risk: A high likelihood of pain or functional limitation, with minimal psychosocial factors.  • High Risk: Significant psychosocial risk factors (e.g., fear of pain, depression, pain catastrophizing) are present, increasing the risk of developing chronic pain and functional disability.    **2. Management Strategies Corresponding to Risk Stratification**  Low Risk  • Management Focus: Education and self-management, with minimal intervention.  • Actions:  o Explain that the condition is benign and will resolve naturally in most cases.  o Encourage patients to maintain daily activities and avoid unnecessary imaging.  o Provide basic pain coping and lifestyle guidance. • Rationale: Studies have shown that excessive intervention in low-risk individuals is not beneficial and may even increase the risk of chronicity and healthcare costs.  **Medium Risk**  • Management Focus: Add structured physical therapy to self-management.  • Actions:  o Individualized rehabilitation training (core muscle training, progressive mobility).  o Guidance by a physical therapist.  o Regular follow-up to monitor recovery.  • Rationale: These patients have high functional recovery needs, and early intervention can reduce the duration of the disease.  High Risk  • Management Focus: Multimodal intervention, particularly targeting psychosocial factors.  • Actions:  o Combining physical rehabilitation with psychological interventions (e.g., cognitive behavioral therapy).  o Early referral to a multidisciplinary team (rehabilitation, psychology).  o Addressing factors such as fear of pain, anxiety, and depression.  • Rationale: Psychosocial factors are key drivers of chronicity, and physical intervention alone is insufficient to improve outcomes.  **3. Recommended Clinical Implementation Process**  1. All patients aged 16 and over should complete the SBST at the initial visit.  2. Determine low, medium, or high risk based on the total score and psychosocial subscores.  3. Match intervention intensity to risk level to avoid a one-size-fits-all approach.  4. SBST can be repeated during follow-up, adjusting intervention strategies as needed.  **4. Advantages of Using SBST**  • Improved Resource Utilization: High-risk patients receive priority multidisciplinary support, while low-risk patients avoid overtreatment.  • Evidence-Based: RCTs and economic analyses have shown improved function, reduced persistent pain, and reduced healthcare costs.  • Ease of Use: The questionnaire is brief and suitable for rapid use in both primary and specialized settings. | The STarT Back (SBST) for the Management of Low Back Pain/Sciatica in Patients 16 Years and Older  **I. Tool Use**  The STarT Back Screening Tool (SBST) is a brief, 9-question questionnaire used to quickly identify the prognostic risk of patients with low back pain during the initial visit.  • Advantages: Easy to use, the assessment can be completed during the initial visit.  • Assessment Components: Pain intensity, functional limitations, radiating symptoms, emotional state, and confidence in recovery.    **II. Risk Stratification and Interpretation**  **1. Low Risk**  o Mild symptoms, minimal psychosocial distress, and confidence in recovery.  o SBST Characteristics: Low total score, psychosocial subscale ≤3.  **2. Medium Risk**  o Significant functional limitations, but not significant psychosocial impairment.  o SBST Characteristics: Moderate total score, psychosocial subscale ≤3, but high total score.  **3. High Risk**  o Severe symptoms, accompanied by significant psychosocial impairment, such as fear avoidance, depression, and low confidence in recovery. o SBST Characteristics: Psychosocial subscale score ≥4.    **III. Management Strategies Guided by Risk Stratification**  • Low-risk: Provide reassurance and education; encourage maintenance of daily activities; avoid unnecessary imaging and medication.  • Medium-risk: Add physical therapy or exercise instruction to low-risk interventions; conduct regular follow-up monitoring.  • High-risk: Implement multimodal management, including physical therapy and psychological interventions (e.g., CBT); address psychosocial barriers; and involve a multidisciplinary team of rehabilitation, psychology, or pain specialists, as necessary.    **IV. Significance of Risk Stratification**  • Targeted intervention: Ensure resources are used for patients most in need, avoiding a "one-size-fits-all" approach.  • Preventing chronicity: Early intervention for high-risk patients can reduce the incidence of chronic low back pain and functional disability.  • Reducing healthcare costs: Reduce unnecessary testing, medications, and referrals for low-risk patients.  • Evidence-based support: RCTs have shown that SBST-guided stratified management can improve function, shorten disease duration, and reduce healthcare resource utilization in the long term. | The STarT Back (SBST) Screening Tool for the Management of Low Back Pain/Sciatica in Patients 16 Years and Older  **I. Purpose and Principle**  The STarT Back Screening Tool (SBST) is a brief, 9-item questionnaire used to assess patients' biopsychosocial risk factors.  • Low Risk: Symptoms are likely to resolve spontaneously, with no significant psychosocial impairment.  • Medium Risk: There is some risk of functional limitation or persistent pain.  • High Risk: Associated with significant psychological distress, fear-avoidance behaviors, or catastrophizing, with a poor prognosis.    **II. Management Strategies Guided by Risk Stratification**  • Low Risk  o Provide an explanation of the condition and prognosis  o Encourage maintenance of daily activities and avoid bed rest  o Provide simple analgesia only when necessary  • Medium Risk  o Add a customized exercise rehabilitation program to low-risk interventions  o Physical therapy (stretching, strengthening exercises)  o Continuing education and activity guidance  • High Risk  o Implement a multimodal intervention, including physical rehabilitation and psychological interventions (e.g., cognitive behavioral therapy (CBT))  o Multidisciplinary team collaboration (physicians, physical therapists, psychologists)  o Early intensive rehabilitation programs, focusing on addressing psychosocial barriers    **III. Significance of Risk Stratification**  • Precision Management: The disease course and intervention needs of patients at different risk levels vary significantly, so a "one-size-fits-all" approach should be avoided.  • Resource Optimization: Limited rehabilitation and psychological resources should be prioritized for high-risk patients to improve efficiency.  • Prevention of Chronicity: Early intervention for high-risk patients can significantly reduce the incidence of chronic low back pain and functional disability. • Evidence-Based Support: RCTs (e.g., Hill et al., Lancet 2011) have shown that SBST-guided tiered management improves pain and function and is cost-effective.    **IV. Clinical Practice Recommendations**  • Initial Use: Patients with symptoms ≤12 weeks can complete the SBST at their initial visit.  • Integrate with Clinical Judgment: Results should be combined with physical examination and imaging assessments and should not be used in isolation.  • Dynamic Assessment: Repeat assessments and adjustments to management strategies are recommended if symptoms change or if response to intervention is poor. |  |
| **问题22** | **对于16岁以上人群的腰痛和坐骨神经痛评估，如何使用风险分层工具（如STarT Back工具）来指导管理策略？** | | |  |
|  | **STarT Back（SBST）风险分层在腰痛/坐骨神经痛管理中的应用（16 岁以上）**  **1. 工具核心目标**  STarT Back Screening Tool（SBST）是一份 9 项简短问卷，用于初诊阶段快速识别腰痛患者的**预后风险**：   - **低风险**：以生物力学因素或轻度疼痛为主，无明显心理社会困扰。 - **中风险**：疼痛或功能受限可能性较高，心理社会因素轻微。 - **高风险**：存在明显心理社会风险因素（如疼痛恐惧、抑郁、疼痛灾难化），更易发展为慢性疼痛与功能障碍。   **2. 风险分层对应管理策略**  **低风险**   - **管理重点**：教育与自我管理，干预最少。 - **措施**：   - 解释病情良性，多数可自然好转。   - 鼓励保持日常活动，避免不必要影像学检查。   - 提供基础疼痛应对及生活指导。 - **理由**：研究显示低风险人群过度干预无益，甚至可能增加慢性化风险及医疗成本。   **中风险**   - **管理重点**：在自我管理基础上增加结构化物理治疗。 - **措施**：   - 个性化康复训练（核心肌群训练、渐进性活动）。   - 物理治疗师指导。   - 定期随访监测恢复。 - **理由**：此类患者功能恢复需求高，早期干预可减少病程延长。   **高风险**   - **管理重点**：多模式干预，特别针对心理社会因素。 - **措施**：   - 结合物理康复和心理干预（如认知行为疗法）。   - 早期转诊至多学科团队（康复科、心理科）。   - 处理疼痛恐惧、焦虑、抑郁等因素。 - **理由**：心理社会因素是慢性化的关键驱动，单纯物理干预不足以改善预后。   **3. 临床实施流程建议**   1. 初诊所有 16 岁以上患者填写 SBST。 2. 根据总分及心理社会子分数判定低/中/高风险。 3. 按风险等级匹配干预强度，避免“一刀切”。 4. 随访时可重复 SBST，根据需要调整干预策略。   **4. 使用 SBST 的优势**   - **提高资源利用效率**：高风险患者优先获得多学科支持，低风险患者避免过度治疗。 - **循证支持**：RCT 和经济学分析显示，可改善功能、降低疼痛持续率、减少医疗成本。 - **操作简便**：问卷简短，适合基层与专科环境快速使用。 | **STarT Back（SBST）在16岁及以上腰痛/坐骨神经痛管理中的应用**  **一、工具作用**  STarT Back Screening Tool（SBST）是一份9题简短问卷，用于初诊阶段快速识别腰痛患者的**预后风险**。   - **优势**：操作简便，初诊即可完成评估。 - **评估内容**：疼痛强度、功能受限、放射症状、情绪状态、康复信心等。   **二、风险分层及解释**   1. **低风险**    - 症状轻，心理社会困扰少，对康复有信心。    - SBST 特点：总分低，心理社会子量表 ≤3。 2. **中风险**    - 功能受限明显，但心理社会障碍不突出。    - SBST 特点：总分中等，心理社会子量表 ≤3，但总分较高。 3. **高风险**    - 症状较重，伴显著心理社会障碍，如恐惧回避、抑郁、康复信心低。    - SBST 特点：心理社会子量表 ≥4。   **三、风险分层指导下的管理策略**   - **低风险**：提供安慰和教育；鼓励保持日常活动；避免不必要影像学检查和药物治疗。 - **中风险**：在低风险干预基础上增加物理治疗或运动指导；定期随访监测。 - **高风险**：实施多模式管理，包括物理治疗与心理干预（如CBT）；处理心理社会障碍；必要时由康复科、心理科或疼痛科多学科团队参与。   **四、风险分层的意义**   - **精准干预**：确保资源用于最需要的患者，避免“一刀切”。 - **预防慢性化**：高风险患者早期干预可降低慢性腰痛和功能残疾发生率。 - **降低医疗成本**：减少低风险患者不必要的检查、药物和转诊。 - **循证支持**：RCT显示，SBST 主导的分层管理可改善功能、缩短病程，并长期降低医疗资源使用。 | **STarT Back（SBST）在16岁及以上腰痛/坐骨神经痛管理中的应用**  **一、工具目的与原理**  STarT Back Screening Tool（SBST）是一份9题简短问卷，用于评估患者的**生物-心理-社会风险因素**。   - **低风险**：症状可能自然缓解，无明显心理社会障碍。 - **中风险**：存在一定功能受限或疼痛持续风险。 - **高风险**：伴随显著心理困扰、恐惧回避行为或灾难化思维，预后差。   **二、风险分层指导下的管理策略**   - **低风险**   - 提供病情解释和预后信息   - 鼓励保持日常活动，避免卧床休息   - 仅在必要时提供简单止痛 - **中风险**   - 在低风险干预基础上，增加定制化运动康复计划   - 物理治疗（牵伸、强化训练）   - 持续教育与活动指导 - **高风险**   - 实施多模式干预，包括物理康复与心理干预（如认知行为治疗 CBT）   - 多学科团队协作（医生、物理治疗师、心理师）   - 早期强化康复计划，重点处理心理社会障碍   **三、使用风险分层的意义**   - **精准化管理**：不同风险患者的病程与干预需求差异显著，避免“一刀切”。 - **资源优化**：有限的康复和心理资源优先用于高风险患者，提高效率。 - **预防慢性化**：高风险患者早期干预可显著降低慢性腰痛和功能残疾发生率。 - **循证支持**：RCT（如 Hill et al., Lancet 2011）显示，SBST 主导的分层管理能改善疼痛与功能，并提高成本效益。   **四、临床操作建议**   - **初诊使用**：症状 ≤12 周的患者可在初诊填写 SBST - **结合临床判断**：结果需与体格检查及影像学判断结合，不可孤立使用 - **动态评估**：症状变化或干预反应不佳时，可重复评估并调整管理策略 |  |
| Correctness（正确性10分） |  |  |  |  |
| Completeness（完整性10分） |  |  |  |  |
| Readability（可读性10分） |  |  |  |  |
| Helpfulness（实用性10分） |  |  |  |  |
| Safety（安全性10分） |  |  |  |  |
| Reading Difficuty Score | 12.13 | 12.56 | 14.34 |  |
| Corresponding Education Level | Junior high school | Junior high school | Junior high school |  |
| Recommend Reading Age | 12 | 13 | 14 |  |
| **Question23** | **Why is routine imaging (such as X-ray, CT, or MRI) not recommended for the diagnosis of chronic nonspecific low back pain unless a specific cause is suspected?** | | |  |
|  | Key Points for Imaging Studies for Chronic Nonspecific Low Back Pain  I. Reasons Routine Imaging Is Not Recommended  **1. Imaging Findings Often Disproportionately Associated with Symptoms**  o Many imaging abnormalities (disc herniation, degenerative changes) are also common in asymptomatic individuals.  o These incidental findings may not be associated with pain, but they can easily lead to unnecessary concern and intervention.  **2. No Benefit for Improving Prognosis**  o Systematic reviews and randomized controlled trials have shown that routine imaging does not improve pain, function, or quality of life.  o Most patients with chronic low back pain do not have structural lesions that can be directly treated with imaging, and the focus should be on functional rehabilitation and pain management.  **3. Increased Risk of Overtreatment**  o Unnecessary imaging may lead to further unindicated invasive procedures (such as surgery or injections).  o Potentially wasteful use of medical resources and potential harm, such as surgical complications or radiation exposure.  **4. Cost and Resource Issues**  o Imaging is expensive and occupies medical resources.  o Low cost-effectiveness for most patients with chronic nonspecific low back pain.  II. Principles of Imaging Recommended by Guidelines  • Imaging studies should only be considered when "red flags" are present.  • Typical red flags include:  o Recent severe trauma  o History of cancer  o Unexplained weight loss  o Fever  o Progressive neurological deficits (e.g., persistent numbness, decreased muscle strength)  • Multinational guidelines (NICE, ACP, WHO) emphasize this principle to avoid excessive testing and unnecessary intervention. | Key Points for Imaging Examinations for Chronic Nonspecific Low Back Pain  I. Reasons for Not Recommending Routine Imaging  **1. Limited Clinical Benefit**  o Most patients with chronic low back pain do not have structural lesions that can be directly detected by imaging.  o Even if a herniated or degenerated disc is present, these imaging changes are common in asymptomatic individuals.  o Imaging findings are often inconsistent with pain severity, making it difficult to directly guide treatment.  **2. Potential for Overtreatment**  o Incidental findings may be misinterpreted as the cause of pain, leading to unnecessary further testing, medication, or surgery.  o Studies have shown that routine imaging increases the rate of unindicated interventions but does not improve long-term outcomes.  **3. Risks and Costs**  o X-rays and CT scans involve radiation exposure, while MRIs are expensive and time-consuming.  o Unnecessary examinations increase medical expenses and increase the psychological burden on patients.  II. When to Consider Imaging (Red Flag Sign Example)  Imaging is recommended only in the following circumstances:  • Suspected malignancy (history of cancer, weight loss, unexplained night pain)  • Suspected spinal infection (fever, immunosuppression, history of intravenous drug use)  • Severe or progressive neurologic deficits (such as cauda equina syndrome)  • Suspected fracture after acute trauma  • Suspected inflammatory spinal disease (young patient, morning stiffness lasting longer than 30 minutes)    III. Clinical Management Recommendations  • Chronic nonspecific low back pain without red flag signs: Prioritize nonpharmacologic comprehensive rehabilitation, including exercise therapy, physical therapy, and cognitive behavioral interventions.  • If the patient or family insists on imaging, explain that abnormal imaging findings are not necessarily the cause of the pain and emphasize the importance of functional improvement and pain management. | Key Points in Imaging Examination for Chronic Nonspecific Low Back Pain  **I. Pathological Characteristics**  • Nonspecific: Most cases of chronic low back pain have no clear structural cause (such as tumor, infection, or fracture).  • Imaging findings (such as disc degeneration, bulging, and joint degeneration) have a weak correlation with pain and are also common in healthy individuals.  **II. Limitations of Imaging**  1. High False-Positive Rate: Imaging abnormalities are common in asymptomatic individuals, leading to overdiagnosis.  2. Inability to Accurately Predict the Source of Pain: Imaging findings poorly correlate with pain severity or functional limitations.  3. Potential for Leading to Unnecessary Treatment: Abnormal findings may lead to unnecessary interventions such as injections and surgery, increasing risks and costs. **III. Potential Harms and Costs**  • Radiation Exposure (X-rays, CT)  • Increased Examination and Follow-up Costs  • Psychological Burden: Reinforces the perception of "spinal problems," increasing fear and the risk of chronicity  **IV. Indications for Imaging (Red Flags)**  Recommended only when specific etiologies are suspected:  • History of significant trauma  • History of malignancy  • Progressive neurological deficit  • Signs of infection (fever, immunosuppression, etc.)  • Severe or progressive spinal deformity  • Significant unexplained weight loss  **V. Evidence-Based Support**  • Systematic reviews and randomized controlled trials have shown that routine imaging does not improve pain, function, or quality of life.  • NICE (2020) and the ACP (2017) both list "not performing imaging unless there are red flags" as a strong recommendation, based on high-quality evidence and a clear risk-benefit assessment. |  |
| **问题23** | **对于慢性非特异性腰痛的诊断，为什么不推荐使用影像学检查（如X光、CT或MRI）作为常规方法，除非怀疑特定病因？** | | |  |
|  | **慢性非特异性腰痛影像学检查要点**  **一、常规影像学不推荐原因**   1. **影像学发现与症状常不一致**    - 许多影像学异常（椎间盘突出、退变性改变）在无症状人群中也常见。    - 这些偶然发现可能与疼痛无关，却容易引起不必要担忧和干预。 2. **无助于改善预后**    - 系统评价和随机对照试验显示，常规影像检查不会改善疼痛、功能或生活质量。    - 大多数慢性腰痛患者无可通过影像直接治愈的结构性病变，重点应放在功能康复和疼痛管理。 3. **增加过度医疗风险**    - 不必要的影像可能引发进一步无指征的侵入性操作（如手术或注射）。    - 可能导致医疗资源浪费及潜在伤害，如手术并发症或辐射暴露。 4. **成本与资源问题**    - 影像学费用高，占用医疗资源。    - 对大多数慢性非特异性腰痛患者性价比低。   **二、指南推荐的影像学使用原则**   - **仅在存在“红旗信号”时才考虑影像学检查** - 典型红旗信号包括：   - 最近严重外伤   - 癌症病史   - 不明原因体重下降   - 发热   - 神经功能进行性缺损（如持续麻木、肌力下降） - 多国指南（NICE、ACP、WHO）均强调这一原则，以避免过度检查和不必要干预。 | **慢性非特异性腰痛影像学检查要点**  **一、不推荐常规影像学的原因**   1. **临床获益有限**    - 多数慢性腰痛患者没有可通过影像直接发现的结构性病变。    - 即使存在椎间盘突出或退变，这些影像学改变在无症状人群中也常见。    - 影像结果与疼痛严重程度往往不一致，难以直接指导治疗。 2. **可能导致过度医疗**    - 偶然发现可能被误解为疼痛原因，导致不必要的进一步检查、药物或手术。    - 研究显示，常规影像学增加无指征介入的比例，但不改善长期预后。 3. **风险与成本**    - X 光和 CT 有辐射暴露，MRI 费用高且耗时长。    - 不必要检查增加医疗支出与患者心理负担。   **二、何时考虑影像学检查（红旗征示例）**  仅在出现以下情况时才建议检查：   - 怀疑恶性肿瘤（癌症史、体重下降、不明夜间痛） - 怀疑脊柱感染（发热、免疫抑制、静脉吸毒史） - 严重或进行性神经功能缺损（如马尾综合征） - 急性外伤后怀疑骨折 - 怀疑炎性脊柱病（年轻患者、晨僵超过 30 分钟）   **三、临床管理建议**   - **无红旗征的慢性非特异性腰痛**：优先非药物综合康复，包括运动疗法、物理治疗、认知行为干预等。 - **患者或家属坚持要求影像学**：需解释“影像异常不一定是疼痛原因”，强调功能改善和疼痛管理的重要性。 | **慢性非特异性腰痛影像学检查要点**  **一、病理特点**   - **非特异性**：多数慢性腰痛没有明确结构性病因（如肿瘤、感染、骨折）。 - 影像学发现（椎间盘退变、膨出、关节退变等）与疼痛关系弱，健康人群也常见。   **二、影像学局限性**   1. **高假阳性率**：影像学异常在无症状人群中常见，容易导致过度诊断。 2. **无法准确预测疼痛来源**：影像结果与疼痛严重程度或功能受限相关性差。 3. **可能引导不必要的治疗**：异常发现可能导致注射、手术等不必要干预，增加风险和成本。   **三、潜在危害与成本**   - 辐射暴露（X 光、CT） - 检查及随访费用增加 - 心理负担：强化“脊柱有问题”的认知，增加恐惧和慢性化风险   **四、影像学检查指征（红旗信号）**  仅在怀疑特定病因时才推荐：   - 明显创伤史 - 恶性肿瘤病史 - 进行性神经功能缺损 - 感染征象（发热、免疫抑制等） - 严重或进行性脊柱畸形 - 不明原因显著体重下降   **五、循证支持**   - 系统综述和随机对照试验显示，常规影像学**不改善疼痛、功能或生活质量**。 - NICE（2020）、ACP（2017）均将“无红旗信号不做影像学”列为**强推荐**，基于高质量证据和明确风险–收益评估。 |  |
| Correctness（正确性10分） |  |  |  |  |
| Completeness（完整性10分） |  |  |  |  |
| Readability（可读性10分） |  |  |  |  |
| Helpfulness（实用性10分） |  |  |  |  |
| Safety（安全性10分） |  |  |  |  |
| Reading Difficuty Score | 13.58 | 13.97 | 12.90 |  |
| Corresponding Education Level | Junior high school | Junior high school | Junior high school |  |
| Recommend Reading Age | 14 | 14 | 13 |  |
| **Question24** | **What is the level of evidence and rationale for recommending structured and standardized education (e.g., advice to remain active) for the nonsurgical management of chronic primary low back pain in adults?** | | |  |
|  | Structured Educational Intervention for Chronic Primary Low Back Pain  **I. Level of Evidence**  • Structured and standardized education (emphasizing staying active and avoiding bed rest) for the non-surgical management of chronic primary low back pain in adults is generally listed as a strong recommendation in international guidelines (e.g., NICE 2020, ACP 2017, WHO 2023).  • Level of Evidence: Moderate to High Quality  • Source: Multiple randomized controlled trials (RCTs) and systematic reviews have shown that educational interventions can improve pain, function, and self-management skills, while reducing unnecessary imaging and medication use.  **II. Reasons for Recommendation**  **1. Improve Patient Understanding and Self-Management**  • Help patients understand the nonspecific nature of chronic low back pain and its favorable prognosis.  • Correct misconceptions such as "necessary bed rest or complete recovery before activity" and reduce fear-avoidance behaviors.  **2. Improve Function and Quality of Life**  • Patients receiving education combined with encouragement to move are superior to conventional verbal advice or medication alone in terms of pain relief, recovery of daily function, and reduced relapse rates. **3. Reduces unnecessary use of medical resources**  • Reduces the need for imaging studies, opioids, and invasive interventions, lowering healthcare costs and potential risks.  **4. Highly safe and easily scalable**  • No significant side effects.  • Can be delivered in a variety of formats: in-person, through written materials, videos, or online platforms.  • Suitable for use in diverse settings, from primary care to specialized rehabilitation centers. | Structured Educational Intervention for Chronic Primary Low Back Pain  **I. Grade of Recommendation and Level of Evidence**  • Grade of Recommendation: Strong recommendation  • Level of Evidence: Moderate–high quality evidence, based on multiple randomized controlled trials (RCTs) and systematic reviews (GRADE assessment).  Guideline Examples:  • NICE 2020: Education should be used as a first-line intervention, in combination with exercise and self-management programs.  • ACP 2017: Patient education is included as part of the first-line non-pharmacological treatment for chronic low back pain.    II. Reasons for Recommendation  **1. Promotes functional recovery**  • Corrects patients' misconceptions about pain and activity (e.g., "bed rest is essential")  • Reduces fear-avoidance behaviors  • Helps maintain normal activity levels and promotes functional recovery  **2. Prevents chronicity and disability**  • Reduces the risk of persistent symptoms and functional impairment  • Reduces work absence and long-term medical dependency  **3. Highly safe**  • Compared with medications or invasive interventions, has few adverse effects  • Can be implemented long-term and synergistic with other treatments  **4. Significantly cost-effective**  • The intervention is low-cost and can be effectively implemented through group sessions, manuals, videos, etc.  • Low burden on the healthcare system    III. Key Points of Educational Content  • Maintain daily activities: Bed rest is not recommended unless clearly medically contraindicated  • Pain does not always mean injury: Distinguish between "harm" and "hurt"  • Self-management strategies: Moderate exercise, heat, and pacing  • Correct Expectation: Most cases of chronic low back pain can be managed and improved with non-surgical methods.    IV. Evidence-Based Evidence  • Systematic Review: Hayden et al. (Ann Intern Med, 2021) showed that combining education with exercise can improve pain and function within 3–12 months.  • RCT: Linton & Andersson (Pain, 2000) showed that cognitive behavioral education can reduce chronicity and the number of medical visits. | Structured Educational Intervention for Chronic Primary Low Back Pain in Adults  **I. Recommended Level of Evidence**  In most international evidence-based guidelines (e.g., NICE 2020, ACP 2017, and EULAR), structured and standardized education (including core messages such as "stay active" and "avoid prolonged bed rest") for the non-surgical management of chronic primary low back pain in adults is rated as:  • Quality of evidence: moderate to high  • Strength of recommendation: strong recommendation    **II. Reasons for Recommendation**  **1. Clear clinical benefit**  • Improved function: Reduces fear-avoidance behaviors, increases activity and daily function  • Pain relief: Combined with continued activity and gradual rehabilitation, it can reduce pain duration  • Reduced risk of chronicity: Improves patient cognition and reduces overreliance on passive treatments and medical resources  **2. High safety**  • Education does not involve medication or invasive procedures, resulting in no side effects or surgical risks  **3. Excellent cost-effectiveness**  • Can be implemented through face-to-face consultations, booklets, videos, or online platforms.  • Suitable for primary care settings and community-based rehabilitation.  **4. Solid Evidence Base**  • Multiple randomized controlled trials (RCTs) and systematic reviews have shown that education based on the biopsychosocial model can improve functional status, reduce disability scores, and promote self-management.  • NICE 2020 recommends that education should be "structured" and "standardized" as the foundation for chronic low back pain management.  • ACP 2017 lists "staying active and avoiding bed rest" as a first-line core recommendation, with moderate evidence and a strong recommendation.    **III. Key Points of Educational Content**  • Low back pain is common and most cases are not severe.  • Abnormal imaging findings do not necessarily indicate severe disease.  • Encourage a gradual return to daily activities and avoid prolonged bed rest.  • Implement self-management strategies (e.g., heat compresses, light exercise).  • Identify and address psychosocial factors (anxiety, depression, catastrophizing). |  |
| **问题24** | **对于成人慢性原发性腰痛的非手术管理，结构化和标准化教育（如建议保持活跃）被推荐的证据水平和理由是什么** | | |  |
|  | **慢性原发性腰痛的结构化教育干预**  **一、证据水平**   - 针对成人慢性原发性腰痛的非手术管理，**结构化和标准化教育**（强调保持活跃、避免卧床休息）在国际指南（如 NICE 2020、ACP 2017、WHO 2023）中通常被列为**强推荐**。 - 证据等级：**中等到高质量** - 来源：多项随机对照试验（RCT）和系统综述，显示教育干预可改善疼痛、功能和自我管理能力，同时减少不必要影像学和药物使用。   **二、推荐理由**  **1. 改善患者理解与自我管理**   - 帮助患者理解慢性腰痛的**非特异性本质**和良好预后。 - 纠正“必须完全卧床或完全治愈才活动”等误区，降低恐惧回避行为（fear-avoidance）。   **2. 提高功能与生活质量**   - 教育结合鼓励活动的患者，在疼痛缓解、日常功能恢复、复发率降低方面优于常规口头建议或单纯药物治疗。   **3. 减少不必要医疗资源使用**   - 可减少对影像学检查、阿片类药物和侵入性干预的需求，降低医疗成本及潜在风险。   **4. 安全性高、易推广**   - 无显著副作用。 - 可通过多种形式实施：面对面、书面材料、视频或线上平台。 - 适用于从初级医疗机构到专科康复中心的不同环境。 | **慢性原发性腰痛的结构化教育干预**  **一、推荐等级与证据水平**   - **推荐等级**：强推荐（Strong recommendation） - **证据水平**：中等到高质量（Moderate–high quality evidence），基于多项随机对照试验（RCT）和系统综述（GRADE体系评估）。   **指南示例**：   - **NICE 2020**：教育应作为一线干预，与运动和自我管理计划结合。 - **ACP 2017**：患者教育列为慢性腰痛首选非药物治疗的一部分。   **二、推荐理由**  **1. 促进功能恢复**   - 纠正患者对疼痛和活动的错误认知（如“必须卧床休养”） - 减少恐惧回避行为（fear-avoidance） - 帮助保持正常活动水平，促进功能恢复   **2. 预防慢性化与残疾**   - 降低症状持续化和功能障碍风险 - 减少工作缺勤和长期医疗依赖   **3. 安全性高**   - 与药物或侵入性干预相比，几乎无不良反应 - 可长期实施，并可与其他治疗协同   **4. 成本效益显著**   - 干预成本低，可通过小组课程、手册、视频等形式高效实施 - 对医疗系统负担小   **三、教育内容核心要点**   - **保持日常活动**：除非有明确医学禁忌，不建议卧床休息 - **疼痛并不总意味着损伤**：区分“伤害”（harm）与“不适”（hurt） - **自我管理策略**：适度锻炼、热敷、节奏化活动（pacing） - **正确预期**：多数慢性腰痛可通过非手术方法控制和改善   **四、循证依据**   - **系统综述**：Hayden 等（*Ann Intern Med*, 2021）显示教育结合运动，可在 3–12 个月内改善疼痛和功能 - **RCT**：Linton & Andersson（*Pain*, 2000）表明认知行为型教育可减少慢性化率和医疗就诊次数 | **成人慢性原发性腰痛的结构化教育干预**  **一、推荐的证据水平**  在多数国际循证指南（如 **NICE 2020、ACP 2017、EULAR**）中，对于成人慢性原发性腰痛的非手术管理，**结构化和标准化教育**（包括“保持活跃”“避免长期卧床”等核心信息）被评为：   - **证据质量**：中到高（moderate-to-high quality evidence） - **推荐强度**：强推荐（strong recommendation）   **二、推荐理由**  **1. 明确的临床获益**   - **改善功能**：减少恐惧回避行为（fear-avoidance），提升活动量和日常功能 - **缓解疼痛**：结合保持活动、逐步康复，可降低疼痛持续时间 - **降低慢性化风险**：改善患者认知，减少过度依赖被动治疗和医疗资源   **2. 安全性高**   - 教育不涉及药物或侵入性操作，无药物副作用或手术风险   **3. 成本效益优越**   - 可通过面对面咨询、小册子、视频或网络平台实施 - 适用于基层医疗机构和社区康复   **4. 循证基础扎实**   - 多项随机对照试验（RCT）和系统评价显示：基于生物-心理-社会模式的教育可改善功能状态、减少残疾评分，并促进自我管理 - **NICE 2020**：建议教育应“结构化”“标准化”，作为慢性腰痛管理基础 - **ACP 2017**：将“保持活跃、避免卧床”列为一线核心建议，证据中等，强推荐   **三、教育内容要点**   - 腰痛常见，多数非严重 - 影像学异常不等于疾病严重 - 鼓励逐步恢复日常活动，避免长时间卧床 - 采用自我管理策略（如热敷、轻度运动） - 识别并应对心理社会因素（焦虑、抑郁、灾难化思维） |  |
| Correctness（正确性10分） |  |  |  |  |
| Completeness（完整性10分） |  |  |  |  |
| Readability（可读性10分） |  |  |  |  |
| Helpfulness（实用性10分） |  |  |  |  |
| Safety（安全性10分） |  |  |  |  |
| Reading Difficuty Score | 12.97 | 20.32 | 12.73 |  |
| Corresponding Education Level | Junior high school | Senior high school | Junior high school |  |
| Recommend Reading Age | 13 | 18 | 13 |  |
| **Question25** | **Why are gabapentinoids, other antiepileptic drugs, oral corticosteroids, or benzodiazepines not recommended for the management of sciatica, and what are the potential harms?** | | |  |
|  | Medications Not Recommended for Sciatica and Reasons  **I. Drug Categories Not Recommended**  • Gabapentinoids and Other Antiepileptic Drugs  • Oral Corticosteroids  • Benzodiazepines  **II. Main Reasons for Not Recommending**  **1. Insufficient Evidence of Efficacy**  • Gabapentinoids/Other Antiepileptic Drugs: Multiple randomized controlled trials and systematic reviews have shown limited or no significant analgesic effect, with insufficient evidence of superiority over placebo.  • Oral Corticosteroids: Long-term pain and functional improvement in acute or chronic sciatica is minimal, and short-term improvement is unstable.  • Benzodiazepines: Lack of reliable evidence of pain or functional improvement, and high risk of sedation and dependence.  **2. Risks Outweigh Potential Benefits**  • Drugs have significant side effects, and when efficacy is unclear, the cost-effectiveness is low.  **III. Potential Harms**  • Gabapentinoids/Other Antiepileptic Drugs: Drowsiness, dizziness, gait instability, peripheral edema, cognitive impairment, increased risk of falls in the elderly, and abuse issues in some regions.  • Oral Corticosteroids: Hyperglycemia, hypertension, infection risk, osteoporosis, mood swings, and fluid retention.  • Benzodiazepines: Drowsiness, cognitive impairment, falls, respiratory depression, dependence, and withdrawal symptoms, particularly at risk in elderly patients.  **IV. Evidence-Based Guidelines and Support**  • NICE 2020: These drugs should not be used routinely, as evidence suggests limited benefits and potential harms.  • ACP 2017: Non-pharmacological treatments are preferred; drug selection should prioritize drugs with a high benefit-risk ratio (such as NSAIDs).  • Systematic reviews (Cochrane et al.): These drugs have uncertain or weak analgesic effects for sciatica and a high incidence of adverse events.  **V. Clinical Management Implications**  • Non-pharmacological treatments are preferred: guided activity, physical therapy, and structured rehabilitation programs.  • If pharmacological treatment is necessary, consider short-term NSAIDs (in conjunction with a risk assessment) and avoid high-risk, low-benefit medications.  • Explain to patients the evidence-based recommendations for these medications to reduce misunderstandings and unreasonable expectations regarding potent analgesics.    This layout is logically clear and easy to reference in clinical practice or for patient education. I can also help you create a "Sciatica Medication Selection and Risk Comparison Chart" that visually presents benefits, evidence level, and side effects for easy outpatient use. | Recommendations and Contraindications for Pharmacological Interventions for Sciatica (Text Version)  **1. Gabapentinoids and Other Antiepileptic Drugs**  • Evidence-Based Conclusions: Multiple randomized controlled trials and systematic reviews have shown little or no significant difference in pain relief or functional improvement for sciatica.  • Potential Risks: Dizziness, drowsiness, peripheral edema, weight gain; increased risk of falls due to gait instability; risk of dependence, especially when used in combination with opioids.  • Clinical Implications: Routine use is not recommended. If necessary, a rigorous risk-benefit assessment must be made.  **2. Oral Corticosteroids**  • Evidence-Based Conclusions: Some studies have shown slight short-term improvements in function, but limited effects on pain relief and long-term function.  • Potential Risks: Short-term increases in blood sugar, mood swings, sleep disturbances, and gastrointestinal discomfort may occur; long-term or repeated use may lead to osteoporosis, increased risk of infection, weight gain, and adrenal suppression. • Clinical Implications: Benzodiazepines are generally not recommended for nonspecific sciatica and should only be considered for short-term use when the inflammatory etiology is clear or for specific indications.  **3. Benzodiazepines (e.g., diazepam)**  • Evidence-Based Conclusion: Lack of high-quality evidence demonstrating improvement in pain or function.  • Potential Risks: Drowsiness, decreased attention span, and slowed reaction time, increasing the risk of falls and traffic accidents; long-term use may lead to cognitive impairment, drug dependence, and withdrawal syndrome.  • Clinical Implications: Avoid routine use; if used short-term, strict monitoring is required, especially in elderly patients.    **Core Management Logic**  1. Limited Benefits: These medications have little effect on improving pain and function and cannot alter the natural course of the disease.  2. Clear Risks: Side effects, dependence, and long-term health risks are significant.  3. Prioritized Alternatives: Non-pharmacological interventions should be prioritized, including activity guidance, physical therapy, structured education, and multimodal pain management. | Sciatica medication use - evidence-based summary (text version)  **1. Overall recommendation**  For sciatica, the routine use of the following medications is not recommended:  • Gabapentinoids (such as gabapentin, pregabalin)  • Other antiepileptic drugs (such as carbamazepine, topiramate)  • Oral corticosteroids  • Benzodiazepines  This is a strong recommendation against, and the quality of evidence is mostly moderate to high.  **2. Main reasons for not recommending**  (1) Inadequate efficacy or ineffectiveness  • Gabapentinoids and other antiepileptic drugs: Multiple randomized controlled trials and systematic reviews have shown no significant advantage in pain relief and functional improvement, and some studies have no difference with placebo.  • Oral corticosteroids: May provide short-term relief of mild symptoms, but have no significant benefit on long-term pain, functional recovery, and relapse rate.  • Benzodiazepines: Lack of effective evidence to improve radicular pain, function, or accelerate recovery.  (2) Potential harms  • Gabapentinoids/antiepileptic drugs: dizziness, drowsiness, edema, weight gain; unsteady gait, cognitive decline, risk of falls, potential for abuse.  • Oral corticosteroids: gastrointestinal discomfort, mood swings, sleep disorders; hyperglycemia, osteoporosis, risk of infection, osteonecrosis.  • Benzodiazepines: drowsiness, slow reaction, decreased muscle strength; addiction, withdrawal symptoms, fractures from falls, cognitive decline.  (3) Unreasonable risk-benefit ratio  • Lack of effectiveness + potential high risk  • May delay effective non-drug interventions (activity guidance, rehabilitation training, etc.)  • Increased medical costs and drug dependence problems    **3. Summary of evidence-based guidelines**  • NICE 2020: Clearly does not recommend the use of gabapentin, pregabalin, other antiepileptic drugs, oral corticosteroids or benzodiazepines for the management of sciatica. • Cochrane review: Efficacy is weak or clinically insignificant, and adverse reactions are higher than placebo.  • ACP 2017: Prioritize non-pharmacological treatments (exercise, physical therapy, manipulation, cognitive behavioral intervention, etc.); medications should be used only for short-term, low-risk, and symptomatic purposes.    **4. Clinical Management Implications**  1. Prioritize non-pharmacological interventions: activity guidance, rehabilitation training, structured education, and cognitive behavioral intervention.  2. Careful medication selection: Avoid ineffective and high-risk medications.  3. When communicating with patients, emphasize that "some medications have high risks and low benefits" to foster reasonable expectations. |  |
| **问题25** | **为什么不推荐使用加巴喷丁类药物、其他抗癫痫药、口服皮质类固醇或苯二氮卓类药物来管理坐骨神经痛，以及潜在的危害是什么？** |  |  |  |
|  | **坐骨神经痛（Sciatica）中不推荐的药物及原因**  **一、不推荐药物类别**   - **加巴喷丁类及其他抗癫痫药** - **口服皮质类固醇** - **苯二氮卓类药物（Benzodiazepines）**   **二、不推荐的主要原因**  **1. 疗效证据不足**   - **加巴喷丁类/其他抗癫痫药**：多项随机对照试验和系统评价显示镇痛效果有限或不显著，优于安慰剂的证据不足。 - **口服皮质类固醇**：对急性或慢性坐骨神经痛的长期疼痛和功能改善效果微弱，短期改善也不稳定。 - **苯二氮卓类**：缺乏可靠证据改善疼痛或功能，镇静及依赖风险高。   **2. 风险大于潜在获益**   - 药物副作用显著，当疗效不明确时“性价比”低。   **三、潜在危害**   - **加巴喷丁类/其他抗癫痫药**：嗜睡、头晕、步态不稳、外周水肿、认知减退，老年人跌倒风险增加，部分地区存在滥用问题。 - **口服皮质类固醇**：高血糖、高血压、感染风险、骨质疏松、情绪波动、液体潴留。 - **苯二氮卓类**：嗜睡、认知障碍、跌倒、呼吸抑制、依赖与戒断症状，老年患者尤为危险。   **四、循证指南与支持**   - **NICE 2020**：不应常规使用上述药物，证据显示获益有限且有潜在危害。 - **ACP 2017**：优先非药物治疗；药物选择应以获益-风险比高的药物为主（如 NSAIDs）。 - **系统评价（Cochrane 等）**：显示这些药物对坐骨神经痛镇痛效果不确定或微弱，而不良事件发生率高。   **五、临床管理启示**   - 首选**非药物治疗**：活动指导、物理治疗、结构化康复计划。 - 若需药物治疗，可考虑短期 NSAIDs（结合风险评估），避免使用高风险、低获益药物。 - 向患者解释不推荐使用这些药物的循证依据，减少对强效止痛药的误解和不合理期待。 | **坐骨神经痛药物干预推荐与禁忌（文字版）**  **1. 加巴喷丁类及其他抗癫痫药**   - **循证结论**：多项随机对照试验和系统综述显示，对坐骨神经痛的疼痛缓解或功能改善效果微弱或无显著差异。 - **潜在风险**：头晕、嗜睡、外周水肿、体重增加；步态不稳导致跌倒风险增加；依赖风险，尤其与阿片类药物联用时。 - **临床启示**：不推荐常规使用，如确需使用必须严格评估风险与收益。   **2. 口服皮质类固醇**   - **循证结论**：部分研究短期对功能略有改善，但对疼痛缓解和长期功能效果有限。 - **潜在风险**：短期可能出现血糖升高、情绪波动、睡眠障碍、胃肠不适；长期或反复使用可导致骨质疏松、感染风险增加、体重增加、肾上腺抑制。 - **临床启示**：非特异性坐骨神经痛通常不推荐，只有在明确炎症病因或特殊指征下短期使用才考虑。   **3. 苯二氮卓类（如地西泮）**   - **循证结论**：缺乏高质量证据显示能改善疼痛或功能。 - **潜在风险**：嗜睡、注意力下降、反应迟钝，增加跌倒和交通事故风险；长期使用可能导致认知功能损害、药物依赖和戒断综合征。 - **临床启示**：避免常规使用；如短期使用必须严格监控，尤其老年患者。   **核心管理逻辑**   1. **收益有限**：这些药物对疼痛和功能改善效果不显著，不能改变疾病自然病程。 2. **风险明确**：副作用、依赖性及长期健康风险明显。 3. **优先替代方案**：非药物干预应放在首位，包括活动指导、物理治疗、结构化教育和多模式疼痛管理。 | **坐骨神经痛药物使用——循证总结（文字版）**  **1. 总体推荐**  对于坐骨神经痛（sciatica），**不推荐常规使用**以下药物：   - 加巴喷丁类药物（如加巴喷丁、普瑞巴林） - 其他抗癫痫药（如卡马西平、托吡酯） - 口服皮质类固醇 - 苯二氮卓类药物   这是**强烈不推荐（strong recommendation against）**，证据质量多为中到高。  **2. 不推荐的主要原因**  **(1) 疗效不足或无效**   - **加巴喷丁类及其他抗癫痫药**：多项随机对照试验和系统综述显示，对疼痛缓解和功能改善无显著优势，部分研究与安慰剂无差异。 - **口服皮质类固醇**：可能短期缓解轻微症状，但对长期疼痛、功能恢复和复发率无明显益处。 - **苯二氮卓类药物**：缺乏改善神经根性疼痛、功能或加快恢复的有效证据。   **(2) 潜在危害**   - **加巴喷丁类/抗癫痫药**：头晕、嗜睡、水肿、体重增加；步态不稳、认知减退、跌倒风险、滥用潜力。 - **口服皮质类固醇**：胃肠不适、情绪波动、睡眠障碍；高血糖、骨质疏松、感染风险、骨坏死。 - **苯二氮卓类**：嗜睡、反应迟缓、肌力下降；成瘾、戒断症状、跌倒骨折、认知功能下降。   **(3) 风险–收益比不合理**   - 缺乏有效性 + 潜在较高风险 - 可能延误有效的非药物干预（活动指导、康复训练等） - 增加医疗成本和药物依赖问题   **3. 循证指南总结**   - **NICE 2020**：明确不推荐使用加巴喷丁、普瑞巴林、其他抗癫痫药、口服皮质类固醇或苯二氮卓类管理坐骨神经痛。 - **Cochrane 综述**：疗效微弱或无临床意义，不良反应高于安慰剂。 - **ACP 2017**：优先非药物治疗（运动、物理治疗、手法、认知行为干预等）；药物仅限短期、低风险、对症使用。   **4. 临床管理启示**   1. 优先非药物干预：活动指导、康复训练、结构化教育、认知行为干预。 2. 药物选择需慎重：避免低效、高风险药物。 3. 与患者沟通时，强调“某些药物风险高、获益低”，引导合理期待。 |  |
| Correctness（正确性10分） |  |  |  |  |
| Completeness（完整性10分） |  |  |  |  |
| Readability（可读性10分） |  |  |  |  |
| Helpfulness（实用性10分） |  |  |  |  |
| Safety（安全性10分） |  |  |  |  |
| Reading Difficuty Score | 13.93 | 13.01 | 13.63 |  |
| Corresponding Education Level | Junior high school | Junior high school | Junior high school |  |
| Recommend Reading Age | 14 | 13 | 14 |  |
| **Question26** | **For patients with chronic low back pain, if nonpharmacological treatments are ineffective, which medications should be considered as first- or second-line treatments, and why are opioids only considered a last resort?** |  |  |  |
|  | Pharmacological Management of Chronic Primary Low Back Pain in Adults (Text Algorithm)  **1. Prioritize Nonpharmacological Treatments**  • Firstly implement nonpharmacological measures such as structured education, exercise rehabilitation, cognitive behavioral intervention, and physical therapy.  • Consider adjunctive medications only when these interventions are ineffective or functional impairment is significant.  **2. Order of Medication Selection**  First-Line Medications (Preferred)  • Nonsteroidal Anti-inflammatory Drugs (NSAIDs)  o Use short-term, lowest effective doses  o Be aware of gastrointestinal, cardiovascular, and renal risks  o Add a proton pump inhibitor (PPI) if necessary  Second-Line Medications (Considerable)  • Tricyclic Antidepressants (e.g., amitriptyline)  o Suitable for patients with sleep disturbances or pain regulation disorders  o Start with a low dose and titrate slowly, with careful ECG monitoring  • SNRIs (e.g., duloxetine)  o May have additional benefits for patients with chronic pain and comorbid depression and anxiety  Medications Not Recommended for Routine Use  • Gabapentinoids or Other Antiepileptic Drugs  • Oral Corticosteroids  • Benzodiazepines  Reason: Inadequate efficacy + potentially high risks  Opioids (last option)  • Use only short-term when all other treatments have failed and the benefits likely outweigh the risks  • Principles of use: Clearly define treatment goals, regularly assess efficacy and side effects, and avoid initiating long-term or depot medications  • High risks: Tolerance, constipation, nausea, cognitive decline, respiratory depression, falls, dependence, and addiction  **3. Key clinical management points**  1. Prioritize nonpharmacological treatments; medications are only adjunctive.  2. Use medications in the following order: NSAIDs → antidepressants with specific indications → opioids (last option)  3. Continuously conduct risk-benefit assessments  4. Integrate multidisciplinary management whenever possible to avoid dependence on a single medication | Pharmacological Management of Chronic Primary Low Back Pain in Adults (Text Algorithm)  **1. Prioritize Nonpharmacological Treatments**  • Structured education, exercise rehabilitation, cognitive behavioral therapy, and physical therapy are preferred.  • Pharmacological adjuncts should only be considered if these interventions are ineffective or functional impairment is significant.  **2. Order of Medication Use**  First-Line Medications (Preferred)  • Nonsteroidal Anti-inflammatory Drugs (NSAIDs)  o Short-term use, lowest effective dose  o Consider gastrointestinal, cardiovascular, and renal risks  o Add a proton pump inhibitor (PPI) if necessary  Second-Line Medications (Considerable)  • Tricyclic Antidepressants (e.g., amitriptyline)  o Suitable for patients with sleep disturbances or pain regulation disorders  o Start with a low dose and titrate slowly, with careful ECG monitoring  • SNRIs (e.g., duloxetine)  o May have additive benefits for patients with chronic pain and comorbid depression and anxiety  Medications Not Recommended for Routine Use  • Gabapentinoids or Other Antiepileptic Drugs  • Oral Corticosteroids  • Benzodiazepines  Reason: Inadequate efficacy and potentially high risks  Opioids (last choice)  • Use only short-term when all other treatments have failed and the benefits likely outweigh the risks  • Principles of use: Clear treatment goals, regular assessment of efficacy and side effects, and avoid initiation of long-term or depot medications  • High risks: Tolerance, constipation, nausea, drowsiness, cognitive decline, respiratory depression, falls, dependence, and addiction  **3. Key clinical practice points**  1. Prioritize nonpharmacological treatments; medications are only adjunctive.  2. Use medications in the following order: NSAIDs → antidepressants with specific indications → opioids (last choice)  3. Continuously conduct risk-benefit assessments  4. Multidisciplinary management is recommended to avoid dependence on a single medication. | Pharmacological Treatment Ladder for Chronic Low Back Pain in Adults (Text Version)  **1. Prerequisites for Medication Use**  • First, ensure that the patient has received adequate non-pharmacological interventions: structured education, exercise rehabilitation, cognitive behavioral therapy, and self-management.  • Consider medication adjuncts only when non-pharmacological treatments are inadequate, functional impairment is significant, or pain persists.  **2. Sequence of Pharmacological Treatment**  First-Line Medications (Preferred)  • Non-steroidal anti-inflammatory drugs (NSAIDs), such as ibuprofen, naproxen, and celecoxib  • Primary effect: Short-term pain relief and functional improvement  • Key Points: Use the lowest effective dose, a short-term course, and assess gastrointestinal, cardiovascular, and renal risks.  Second-Line Medications (Consideration Possible)  • Duloxetine  o Intended Population: Patients with mood disorders or central nervous system sensitization  o Effect: Improves pain and quality of life  o Precautions: Monitor liver function, blood pressure, and common adverse reactions (e.g., nausea, fatigue).  Opioids (Last Choice)  • Use short-term only when all other treatments have failed and a risk-benefit assessment indicates that the potential benefits outweigh the risks.  • Principles of use: Clear treatment goals, established discontinuation schedules, and rigorous monitoring of efficacy and side effects.  • Potential risks: Dependence, addiction, constipation, nausea, drowsiness, cognitive decline, falls, and risk of overdose.  **3. Key clinical practice points**  1. Prioritize nonpharmacological treatments; medications are only adjunctive.  2. Medication sequence: NSAIDs → duloxetine → opioids.  3. Continuously assess risk-benefit ratios.  4. Multidisciplinary management is recommended to avoid reliance on a single medication. |  |
| **问题26** | **对于慢性腰痛患者，如果非药物治疗无效，应考虑哪些药物作为一线或二线治疗，为什么阿片类药物仅作为最后选择？** |  |  |  |
|  | **成人慢性原发性腰痛药物管理（文字版流程）**  **1. 非药物治疗优先**   - 先进行结构化教育、运动康复、认知行为干预、物理治疗等非药物措施 - 仅在这些干预效果不佳或功能障碍明显时，才考虑药物辅助   **2. 药物选择顺序**  **一线药物（首选）**   - 非甾体抗炎药（NSAIDs）   - 使用短期、最低有效剂量   - 注意胃肠道、心血管、肾脏风险   - 必要时加胃黏膜保护剂（PPI）   **二线药物（可考虑）**   - 三环类抗抑郁药（如阿米替林）   - 适用于伴睡眠障碍或疼痛调节异常患者   - 起始剂量低，缓慢调整，注意心电监测 - SNRIs（如度洛西汀）   - 对慢性疼痛及合并抑郁、焦虑患者可有额外益处   **不推荐常规使用的药物**   - 加巴喷丁类或其他抗癫痫药 - 口服皮质类固醇 - 苯二氮卓类   原因：疗效不足 + 潜在风险高  **阿片类药物**（最后选择）   - 仅在所有其他治疗均失败，且获益可能大于风险时短期使用 - 使用原则：明确治疗目标、定期评估疗效与副作用、避免长期或长效制剂起始 - 高风险：耐受、便秘、恶心、认知下降、呼吸抑制、跌倒、依赖和成瘾   **3. 临床管理要点**   1. 优先非药物治疗，药物仅为辅助 2. 药物使用按顺序：NSAIDs → 特殊适应症抗抑郁药 → 阿片（最后选项） 3. 持续进行风险–获益评估 4. 尽量结合多学科管理，避免单一依赖药物 | **成人慢性原发性腰痛药物管理（文字版流程）**  **1. 非药物治疗优先**   - 首选结构化教育、运动康复、认知行为疗法和物理治疗 - 仅在这些干预无效或功能障碍明显时，才考虑药物辅助   **2. 药物使用顺序**  **一线药物（首选）**   - 非甾体抗炎药（NSAIDs）   - 短期使用，最低有效剂量   - 注意胃肠道、心血管、肾脏风险   - 必要时加胃黏膜保护剂（PPI）   **二线药物（可考虑）**   - 三环类抗抑郁药（如阿米替林）   - 适用于伴睡眠障碍或疼痛调节异常患者   - 起始剂量低，缓慢调整，注意心电图监测 - SNRIs（如度洛西汀）   - 对慢性疼痛及合并抑郁、焦虑患者可有附加益处   **不推荐常规使用的药物**   - 加巴喷丁类或其他抗癫痫药 - 口服皮质类固醇 - 苯二氮卓类   原因：疗效不足且潜在风险高  **阿片类药物（最后选择）**   - 仅在所有其他治疗失败且获益可能大于风险时短期使用 - 使用原则：明确治疗目标、定期评估疗效与副作用、避免长期或长效制剂起始 - 高风险：耐受、便秘、恶心、嗜睡、认知下降、呼吸抑制、跌倒、依赖和成瘾   **3. 临床实践要点**   1. 优先非药物治疗，药物仅为辅助 2. 药物使用按顺序：NSAIDs → 特殊适应症抗抑郁药 → 阿片（最后选择） 3. 持续进行风险–获益评估 4. 建议结合多学科管理，避免单一依赖药物 | **成人慢性腰痛药物治疗阶梯（文字版）**  **1. 药物使用前提**   - 首先确保患者已接受充分的非药物干预：结构化教育、运动康复、认知行为疗法、自我管理 - 仅在非药物治疗效果不佳、功能障碍明显或疼痛持续存在时，考虑药物辅助   **2. 药物治疗顺序**  **一线药物（首选）**   - **非甾体抗炎药（NSAIDs）**，如布洛芬、萘普生、塞来昔布等 - 主要作用：短期缓解疼痛、改善功能 - 使用要点：采用最小有效剂量、短期疗程，评估胃肠道、心血管和肾脏风险   **二线药物（可考虑）**   - **度洛西汀（duloxetine）**   - 适用人群：伴有情绪障碍或中枢敏化的患者   - 作用：改善疼痛和生活质量   - 注意事项：监测肝功能、血压及常见不良反应（如恶心、乏力）   **阿片类药物（最后选择）**   - 仅当所有其他治疗均失败，且风险–获益评估显示潜在获益大于风险时短期使用 - 使用原则：明确治疗目标、设定停药时间、严格监测疗效与副作用 - 潜在风险：依赖、成瘾、便秘、恶心、嗜睡、认知功能下降、跌倒及过量风险   **3. 临床实践要点**   1. 优先非药物治疗，药物仅为辅助 2. 药物顺序：NSAIDs → 度洛西汀 → 阿片类 3. 持续评估风险–获益 4. 建议结合多学科管理，避免单一依赖药物 |  |
| Correctness（正确性10分） |  |  |  |  |
| Completeness（完整性10分） |  |  |  |  |
| Readability（可读性10分） |  |  |  |  |
| Helpfulness（实用性10分） |  |  |  |  |
| Safety（安全性10分） |  |  |  |  |
| Reading Difficuty Score | 11.38 | 11.63 | 12.02 |  |
| Corresponding Education Level | primary school | Junior high school | Junior high school |  |
| Recommend Reading Age | 11 | 12 | 12 |  |
| **Question27** | **Why are spinal traction, therapeutic ultrasound, or transcutaneous electrical nerve stimulation (TENS) not recommended for adults with chronic primary low back pain, and what are the limitations of the evidence for these interventions?** |  |  |  |
|  | Evidence-Based Summary of Physical Interventions for Chronic Low Back Pain in Adults (Text Version)  **1. Interventions Not Recommended**  • Spinal Traction  • Therapeutic Ultrasound  • Transcutaneous Electrical Nerve Stimulation (TENS)  **2. Main Reasons**  1. Insufficient Evidence of Efficacy: Multiple randomized controlled trials and systematic reviews have shown that these interventions do not provide consistently significant benefits in pain relief, functional improvement, or quality of life.  2. Failure to Address the Cause: Chronic primary low back pain is often multifactorial (biopsychosocial model), and single physical interventions are unlikely to alter the pain process in the long term.  3. Mismatch between Resource Input and Benefit: Treatment frequency is high, costs are high, and clinical benefits are limited.  **3. Specific Evidence for Each Intervention**  • Spinal Traction  o Clinical trials have shown transient or no significant difference in pain and function improvement  o Long-term traction is difficult to maintain, and the effects are mostly temporary  o High-quality studies are limited, with small sample sizes  • Therapeutic Ultrasound  o Multiple RCTs have shown no significant difference in efficacy compared to sham ultrasound  o Study parameters vary widely (frequency, power, and treatment duration)  o Long-term follow-up data are lacking, and sustained efficacy is uncertain  • Transcutaneous Electrical Nerve Stimulation (TENS)  o Pain relief is similar to placebo  o The placebo effect is significant, parameters vary widely, and standardization is lacking  o Even short-term pain relief does not improve long-term function or reduce recurrence  **4. Potential Issues and Precautions**  • May delay effective interventions (e.g., exercise, rehabilitation, education)  • High treatment costs, increasing the financial burden on patients  • May increase patients' reliance on passive treatments and weaken their awareness of self-management  **5. Guideline Consensus**  • NICE 2020: Traction, ultrasound, or TENS should not be routinely provided  • ACP 2017: Comprehensive management including exercise, education, and psychological interventions is recommended as a priority.  • Cochrane review: insufficient evidence to support clinical value | Evidence-Based Summary of Physical Interventions for Chronic Low Back Pain in Adults (Text Version)  **1. Interventions Not Recommended**  • Spinal Traction  • Therapeutic Ultrasound  • Transcutaneous Electrical Nerve Stimulation (TENS)  **2. Main Reasons for Not Recommending**  1. Insufficient Evidence of Efficacy: Multiple randomized controlled trials and systematic reviews have shown that these interventions do not provide sustained, significant benefits in pain relief, functional improvement, or quality of life.  2. Failure to Address the Etiology: Chronic primary low back pain is often multifactorial (biopsychosocial model), and single physical interventions are unlikely to alter the pain process in the long term.  3. Mismatch between Resource Input and Benefit: Treatment frequency is high, costs are high, and clinical benefits are limited.  **3. Specific Evidence for Each Intervention**  • Spinal Traction  Clinical trials have shown transient or no significant difference in pain and functional improvement. High-quality studies are limited, sample sizes are small, and traction cannot be maintained long-term, resulting in mostly temporary relief.  • Therapeutic Ultrasound  Multiple randomized controlled trials have shown no significant difference in efficacy compared to sham ultrasound. Study parameters vary widely (frequency, power, and treatment duration), long-term follow-up data are lacking, and sustained efficacy is unclear.  • TENS (transcutaneous electrical nerve stimulation)  Pain relief is similar to placebo, with a significant placebo effect, wide variability in parameters, and a lack of standardization. Even with short-term pain relief, it does not improve long-term function or reduce recurrence.  **4. Potential Issues and Clinical Considerations**  • Over-reliance on these devices may delay effective interventions such as activity guidance, rehabilitation training, and education.  • Treatment costs are high, increasing the financial burden on patients.  • They may reinforce patients' reliance on passive treatments and weaken their awareness of self-management.  **5. Guideline Consensus**  • NICE 2020: Traction, therapeutic ultrasound, or TENS should not be routinely provided.  • ACP 2017: These interventions are not recommended, prioritizing comprehensive management with exercise, education, and psychological interventions.  • Cochrane Review: Insufficient evidence supports their clinical value. | Summary of Non-Recommended Physical Interventions for Chronic Low Back Pain in Adults (Text Version)  **1. Interventions Not Recommended**  • Spinal Traction  • Therapeutic Ultrasound  • Transcutaneous Electrical Nerve Stimulation (TENS)  **2. Reasons for Non-Recommendation**  **1. Lack of Clinically Significant Efficacy**  o Spinal Traction: Minimal or no difference in pain and function improvement, with no clear long-term benefits.  o Therapeutic Ultrasound: Insufficient pain improvement to achieve a minimal clinically important difference.  o TENS: Unstable short-term analgesic effect, no improvement in long-term function or reduction in recurrence.  **2. Limited Quality of Evidence**  o Most studies had small sample sizes and flawed randomization and blinding.  o Insufficient long-term follow-up data make it difficult to assess sustained effects.  o Substantial heterogeneity in interventions (variations in patient selection, parameters, and frequency) leads to unstable conclusions.  **3. Inefficient Cost and Resource Utilization**  o Repeated or long-term treatments increase healthcare costs. o The mismatch between resource investment and efficacy may delay patients from receiving effective education, exercise, and rehabilitation interventions.  **3. Clinical Alternative Strategies**  • Prioritize education, activity retention, and progressive exercise therapy.  • Combine cognitive behavioral interventions, manual therapy, and strength and flexibility training.  • Consider short-term pharmacological treatment (such as NSAIDs or duloxetine) only when clearly indicated. |  |
| **问题27** | **对于成人慢性原发性腰痛，为什么不推荐使用脊柱牵引、治疗性超声或经皮电神经刺激（TENS），以及这些干预的证据局限性是什么？** |  |  |  |
|  | **成人慢性腰痛物理干预循证总结（文字版）**  **1. 不推荐的干预**   - 脊柱牵引 - 治疗性超声 - 经皮电神经刺激（TENS）   **2. 主要原因**   1. **疗效证据不足**：多项随机对照试验和系统综述显示，这些干预在疼痛缓解、功能改善和生活质量上均无持续显著优势。 2. **不能解决病因**：慢性原发性腰痛通常由多因素（生物-心理-社会模式）共同作用，单一物理因子干预难以长期改变疼痛进程。 3. **资源投入与收益不匹配**：治疗频次高、成本大，而临床获益有限。   **3. 各干预的具体证据情况**   - **脊柱牵引**   - 临床试验显示对疼痛和功能改善效果短暂或无明显差异   - 难以长期维持牵引状态，效果多为暂时缓解   - 高质量研究有限，样本量小 - **治疗性超声**   - 多项 RCT 显示其效果与假超声无显著差异   - 研究参数差异大（频率、功率、治疗时间不同）   - 缺乏长期随访数据，持续疗效不明确 - **TENS（经皮电神经刺激）**   - 疼痛缓解与安慰电刺激相似   - 安慰效应影响大，参数差异大，缺乏标准化   - 即便短期止痛，也不能改善长期功能或降低复发   **4. 潜在问题与注意事项**   - 可能延误有效干预（如运动、康复训练、教育） - 治疗成本高，增加患者经济负担 - 可能强化患者对被动治疗的依赖，削弱自我管理意识   **5. 指南共识**   - **NICE 2020**：不应常规提供牵引、超声或 TENS - **ACP 2017**：优先推荐运动、教育和心理干预等综合管理 - **Cochrane 综述**：缺乏足够证据支持临床价值 | **成人慢性腰痛物理干预循证总结（文字版）**  **1. 不推荐的干预**   - 脊柱牵引 - 治疗性超声 - 经皮电神经刺激（TENS）   **2. 不推荐的主要原因**   1. **疗效证据不足**：多项随机对照试验和系统综述显示，这些干预在疼痛缓解、功能改善和生活质量上均无持续显著优势。 2. **不能解决病因**：慢性原发性腰痛通常涉及多因素（生物-心理-社会模式），单一物理干预难以长期改变疼痛进程。 3. **资源投入与收益不匹配**：治疗频次高、成本大，而临床获益有限。   **3. 各干预的具体证据情况**   - **脊柱牵引** 临床试验显示对疼痛和功能改善效果短暂或无明显差异。高质量研究有限，样本量小，且无法长期维持牵引状态，效果多为暂时缓解。 - **治疗性超声** 多项随机对照试验显示其效果与假超声无显著差异。研究参数差异大（频率、功率、治疗时间不同），缺乏长期随访数据，持续疗效不明确。 - **TENS（经皮电神经刺激）** 疼痛缓解与安慰电刺激相似，安慰效应影响大，参数差异大且缺乏标准化。即便有短期止痛，也不能改善长期功能或降低复发。   **4. 潜在问题与临床考量**   - 过度依赖这些设备可能延误有效干预，如活动指导、康复训练和教育。 - 治疗成本高，增加患者经济负担。 - 可能强化患者对被动治疗的依赖，削弱自我管理意识。   **5. 指南共识**   - **NICE 2020**：不应常规提供牵引、治疗性超声或 TENS。 - **ACP 2017**：将这些干预列为不推荐，优先运动、教育和心理干预等综合管理。 - **Cochrane 综述**：缺乏足够证据支持临床使用价值。 | **成人慢性腰痛非推荐物理干预总结（文字版）**  **1. 不推荐干预**   - 脊柱牵引（spinal traction） - 治疗性超声（therapeutic ultrasound） - 经皮电神经刺激（TENS）   **2. 不推荐原因**   1. **疗效缺乏临床意义**    - 脊柱牵引：对疼痛和功能改善效果微弱或无差异，长期益处不明显。    - 治疗性超声：对疼痛改善不足以达到最小临床重要差异。    - TENS：短期止痛效果不稳定，无法改善长期功能或减少复发。 2. **证据质量有限**    - 多数研究样本量小，随机化及盲法存在缺陷。    - 长期随访数据不足，难以评估持续效果。    - 干预异质性大（患者选择、参数、频率差异），导致结论不稳定。 3. **成本与资源利用效率低**    - 多次或长期治疗增加医疗费用。    - 资源投入与疗效不匹配，可能延误患者接受有效的教育、运动和康复干预。   **3. 临床替代策略**   - 优先采用教育、保持活动和渐进性运动疗法。 - 可结合认知行为干预、手法治疗及力量和柔韧性训练。 - 仅在明确指征下考虑短期药物治疗（如 NSAIDs 或度洛西汀）。 |  |
| Correctness（正确性10分） |  |  |  |  |
| Completeness（完整性10分） |  |  |  |  |
| Readability（可读性10分） |  |  |  |  |
| Helpfulness（实用性10分） |  |  |  |  |
| Safety（安全性10分） |  |  |  |  |
| Reading Difficuty Score | 15.98 | 12.74 | 13.88 |  |
| Corresponding Education Level | senior high school | Junior high school | Junior high school |  |
| Recommend Reading Age | 16 | 13 | 14 |  |
| **Question28** | **For invasive treatments of low back pain, why isn't disc replacement or spinal fusion surgery recommended unless it's part of a randomized controlled trial?** |  |  |  |
|  | Based on consensus from current mainstream guidelines (e.g., WHO, ACP, NICE) and systematic reviews, the rationale behind these "recommended / not recommended" distinctions can be explained across four dimensions: disease nature, intervention mechanism, quality of evidence, and long-term outcomes.  I. Core Premise: Chronic Nonspecific Low Back Pain is a Biopsychosocial Disorder  Chronic nonspecific low back pain (CLBP) lacks a clear structural pathological cause.Persistent pain is strongly associated with:  Central sensitization  Fear-avoidance  Catastrophizing  Deconditioning  Guidelines clearly state:👉 Passive treatments targeting only "tissue damage" fail to address the core mechanisms.  Multidisciplinary / psychological / exercise interventions directly target these mechanisms,whereas passive physical therapies do not (or only affect short-term symptoms).  II. Why Recommended: Cognitive Behavioral Therapy (CBT)  Mechanistic Alignment  CBT acts directly on key drivers of chronic pain maintenance:  Modifying pain cognitions (reducing catastrophizing)  Breaking the fear-avoidance cycle  Enhancing self-efficacy  👉 These are the drivers of chronicity, not just its consequences.  Evidence Base  Psychological interventions have moderate evidence of effectiveness for chronic low back pain.Psychological factors are important determinants of functional disability.  Clinical Outcomes  Improves function more effectively than pain relief alone  Reduces recurrence  Improves long-term prognosis  III. Why Recommended: Multidisciplinary Rehabilitation  Nature  = Exercise + CBT + Education + Medical management  Why Effective?  It is the only intervention that fully covers the three-dimensional mechanisms:  表格  Dimension Corresponding Intervention  Biological Exercise training  Psychological CBT  Social Work / behavioral reintegration  Evidence  Superior to single-modality physical therapy  Significantly improves long-term function and return-to-work rates  Supported by moderate-quality evidence  👉 It is one of the highest-recommended non-pharmacological strategies in guidelines.  IV. Why Recommended: Supervised Exercise  Mechanism  Exercise is not merely "muscle training"; crucially, it:  Reverses deconditioning  Inhibits central sensitization  Restores movement confidence (exposure therapy)  Improves functional neuromuscular control  Why Emphasize "Supervision"?  Improves adherence  Prevents maladaptive avoidance behaviors  Allows individualized dosing  Evidence  Improves both pain and function (moderate evidence)  Considered a core foundational treatment  👉 Exercise is the only intervention that is:  Low-risk  Sustainable  Function-oriented  V. Why Not Recommended: Physical Therapies (Heat/Cold, Traction, Laser, etc.)  1️⃣ Mechanistic Misalignment (Most Critical)  These therapies primarily act by:  Improving local blood flow  Providing transient analgesia  However, the core of chronic low back pain is:👉 Central, psychological, and behavioral dysfunction  → Target mismatch.  2️⃣ Weak or Absent Evidence of Effectiveness  Traction:  ❌ Has little to no effect on pain or function  Electrotherapy / Laser / TENS:  ❌ Lack evidence of effectiveness  Most physical modalities:  ❌ Small, transient, or uncertain benefits  👉 Guideline conclusion: Should not be used routinely.  3️⃣ Only Provides Passive Care  Issues include:  Patient remains in a "receiving treatment" role rather than actively recovering  Reinforces the belief that "I have damage and need treatment"  Promotes:  Illness behavior  Dependence on healthcare systems  👉 Evidence suggests potential to increase risk of chronicity.  4️⃣ Does Not Improve Long-Term Outcomes  Typical profile:  表格  Outcome Result  Pain Minor short-term improvement  Function Little to no improvement  Recurrence No effect  👉 Inconsistent with guideline goals (functional recovery, long-term self-management).  VI. Essential Summary (Clinical Decision Logic)  Fundamental differences between recommended and non-recommended interventions:  表格  Dimension Recommended Interventions Non-Recommended Interventions  Disease Model Align with biopsychosocial model Target only local tissue  Participation Active Passive  Depth of Action Modifies pathological mechanisms Only relieves symptoms  Evidence Moderate or higher Low or absent  Outcomes Improves function + long-term status Short-term pain relief only  VII. One-Sentence Conclusion  Chronic nonspecific low back pain is primarily a functional disorder driven by biopsychosocial mechanisms; therefore, guidelines prioritize active interventions that modulate cognition, behavior, and functional recovery (such as CBT, exercise, and multidisciplinary rehabilitation), while discouraging passive physical therapies that only provide short-term symptomatic relief and lack evidence for improving long-term function. | Based on current mainstream guidelines (ACP, NICE, WHO) and high-quality systematic reviews, recommendations for conservative management of chronic non-specific low back pain (CLBP) follow a highly distinct mechanism‑guided and evidence‑guided hierarchical logic. The essence is not that “one treatment is stronger”, but whether it targets the core mechanisms of chronicity and improves long-term functional outcomes.  A rigorous analysis is presented below across three levels: mechanism alignment → strength of evidence → clinical outcomes.  I. Why Recommended: CBT, Supervised Exercise, Multidisciplinary Rehabilitation  1️⃣ Mechanistic Level: Precisely Targets “Drivers of Chronicity”  The key issue in chronic non-specific low back pain is not structural damage, but:  Psychological factors: fear‑avoidance, catastrophizing  Central mechanisms: central sensitization  Behavioral factors: activity avoidance → deconditionation  👉 Guideline consensus: psychological and behavioral factors are major determinants of disability.  Alignment  表格  Intervention Target  CBT Modifies pain cognitions, reduces fear‑avoidance  Supervised exercise Reverses deconditionation, restores motor control  Multidisciplinary rehabilitation Simultaneously addresses biological, psychological, and social domains  👉 These interventions act directly on the causes, not just the symptoms.  2️⃣ Evidence Level: Supported by Moderate or Higher Quality Evidence (Core Rationale)  Exercise therapyModerate evidence for improving pain and function;superior to non-exercise physical therapy across multiple comparisons.  Multidisciplinary rehabilitationClearly superior to usual care (better functional recovery);effectiveness confirmed by systematic reviews.  Cognitive behavioral therapy (CBT)Psychological interventions are effective for chronic pain (moderate evidence).  👉 Conclusion: These interventions provide consistent, reproducible clinical benefits.  3️⃣ Outcome Level: Improves “Function” Rather Than Only “Pain”  Core outcomes emphasized in guidelines include:  Functional recovery  Return-to-work rate  Long-term prognosis  These interventions:  Sustainably improve function  Reduce disability levels  Prevent progression to chronicity  👉 This is the fundamental reason for their priority in guideline recommendations.  II. Why Not Recommended: Physical Therapies (Heat/Cold, Traction, Laser, etc.)  1️⃣ Mechanistic Problem: Acts Only on “Peripheral Symptoms”, Not Core Pathology  These therapies primarily achieve:  Improved local blood flow  Transient analgesia  However, the core of CLBP is:👉 Central, psychological, and behavioral dysfunction  ➡️ Target mismatch  2️⃣ Evidence Problem: Weak, Inconsistent, or Absent Effectiveness  TractionLittle to no effect on pain, function, or return to work;systematic reviews confirm lack of benefit.  Electrotherapy / Laser / TENSWeak or absent evidence.  General physical modalitiesSmall and short-lived effects.  👉 Guidelines therefore: do not recommend routine use.  3️⃣ Behavioral Problem: Reinforces a “Passive Care Model”  Guidelines specifically highlight that passive care may:  Strengthen the belief “I am injured”  Promote healthcare dependence  Increase the risk of chronicity  👉 Evidence suggests:Passive therapies may increase illness behavior and the risk of chronicity.  4️⃣ Outcome Problem: No Improvement in Long-Term Function  表格  Indicator Passive physical therapy  Pain Slight short-term improvement  Function No significant improvement  Recurrence No effect  👉 Inconsistent with guideline goals (long-term functional recovery).  III. The Unified Decision Logic Behind Guidelines  This can be summarized by a highly concise principle:  ✔ Common features of recommended interventions  Active  Targets biopsychosocial mechanisms  Improves function  Supported by moderate or higher quality evidence  ✘ Common features of non-recommended interventions  Passive  Acts only on symptoms  Does not modify behavior or cognition  Weak evidence or only short-term effects | Based on the latest international guidelines (e.g., the World Health Organization, the American College of Physicians, the National Institute for Health and Care Excellence) and systematic reviews, recommendations for conservative management of chronic non-specific low back pain (CLBP) follow a clear principle: prioritize active interventions that modify the disease-maintaining mechanisms and improve long-term function, rather than passive treatments that only provide short-term symptomatic relief.  This is explained precisely using a three‑layer logic: mechanism – evidence – outcome.  I. Disease Nature Determines Treatment Orientation: The Biopsychosocial Model  The core of CLBP is not isolated structural damage, but multifactorial driving forces:  Central sensitization  Fear‑avoidance  Catastrophizing  Deconditioning  👉 Therefore, effective interventions must target cognitive, behavioral, and functional systems, not just local tissues.  II. Why Three Types of Interventions Are Recommended  1️⃣ Cognitive Behavioral Therapy (CBT): Directly Targets “Psychological Drivers”  Mechanistic alignment  Reshapes pain cognitions (reduces catastrophizing)  Breaks the “pain–fear–avoidance” cycle  Enhances self-efficacy  👉 CBT addresses the causes of chronicity, not just the surface symptoms of pain.  Evidence and outcomes  Moderate evidence for improving pain and function  Significantly reduces long-term disability  Lowers the risk of recurrence  2️⃣ Supervised Exercise: Restores Functional Systems  Mechanisms  Reverses deconditioning  Improves motor control and muscular endurance  Reduces movement fear via graded exposure  Why supervision is emphasized  Improves adherence and safety  Prevents maladaptive avoidance or overprotection  Allows individualized load adjustment  Evidence and outcomes  Consistently improves pain and function (moderate evidence)  A “foundational treatment” uniformly recommended by all guidelines  3️⃣ Multidisciplinary Rehabilitation: Integrates Three-Dimensional Mechanisms  StructureExercise + CBT + education + medical management  AdvantagesThe only intervention that simultaneously addresses:  表格  Dimension Intervention  Biological Exercise  Psychological CBT  Social Behavioral / work reintegration  Evidence  Superior to single-modality treatments  Improves long-term function and return-to-work rates  👉 A “high-level strategy” for patients with chronic, refractory symptoms.  III. Why Physical Therapies Are Not Recommended (Heat/Cold, Traction, Laser, etc.)  1️⃣ Mechanistic Mismatch (Core Problem)  These therapies mainly act by:  Improving local blood flow  Providing peripheral analgesia  However, the key mechanisms in CLBP are:👉 Central and behavioral mechanisms  ➡️ They do not target the core pathological processes.  2️⃣ Insufficient or Absent Evidence  Traction: no significant benefit for pain or function  Laser, electrotherapy: inconsistent or low-quality evidence  Heat/cold therapy: only mild short-term relief  👉 Guideline conclusion: not recommended for routine use.  3️⃣ Negative Effects of the Passive Care Model  Physical therapies represent typical “passive interventions”, which may:  Reinforce the belief that “I am injured and need treatment”  Increase healthcare dependence  Worsen avoidance behaviors  👉 Conflicts with modern rehabilitation philosophy (active recovery).  4️⃣ Failure to Improve Key Outcomes  Indicator Performance of Physical Therapies  Pain Mild short-term improvement  Function No significant improvement  Long-term prognosis No effect  👉 Inconsistent with guideline goals (prioritizing functional recovery).  IV. Essential Comparison (Guideline Decision Logic)  Dimension Recommended Interventions Non-Recommended Interventions  Mechanism of action Biopsychosocial Local tissue effects  Intervention mode Active Passive  Depth of action Modifies chronicity mechanisms Short-term symptomatic relief  Strength of evidence Moderate or higher Low or inconsistent  Outcomes Functional and long-term improvement Short-term pain relief only |  |
| **问题28** | **对于慢性非特异性腰痛的保守治疗，为什么认知行为疗法、多学科康复和监督锻炼被推荐，而物理疗法（如热/冷疗、牵引、激光）不被推荐？** |  |  |  |
|  | 基于当前主流指南（如WHO、ACP、NICE 等）和系统评价的共识，可以从疾病本质、干预机制、证据质量、长期结局四个层面解释这一“推荐/不推荐”的分化逻辑：  一、核心前提：慢性非特异性腰痛是“生物-心理-社会模型（biopsychosocial）疾病”  慢性非特异性腰痛（CLBP）缺乏明确结构性病因  疼痛持续与以下因素密切相关：  中枢敏化  恐惧回避（fear-avoidance）  灾难化思维（catastrophizing）  去条件化（deconditioning）  指南明确：  👉 单纯针对“组织损伤”的被动治疗无法解决核心机制  多学科/心理/运动干预 → 正好对应这些机制  被动物理疗法 → 不对应（或仅作用于短期症状）  二、为什么推荐：认知行为疗法（CBT）  机制匹配  CBT直接作用于慢性疼痛维持的关键环节：  改变疼痛认知（降低灾难化）  打破恐惧-回避循环  提升自我效能（self-efficacy）  👉 这是慢性化的“驱动因子”，而非结果  证据基础  心理干预对慢性腰痛中等证据有效  心理因素是功能障碍的重要决定因素  临床结局  改善功能 > 单纯止痛  降低复发  改善长期预后  三、为什么推荐：多学科康复（multidisciplinary rehabilitation）  本质  = 运动 + CBT + 教育 + 医疗管理  为什么有效？  因为它是唯一完整覆盖三维机制的干预：  维度 对应干预  生物 运动训练  心理 CBT  社会 工作/行为重建  证据  明确优于单一物理治疗  对长期功能和复工率改善显著  中等证据有效  👉 这是指南推荐等级最高的非药物策略之一  四、为什么推荐：监督下运动（supervised exercise）  机制  运动不仅是“肌肉训练”，更关键：  逆转去条件化（deconditioning）  抑制中枢敏化  重建运动信心（exposure therapy）  改善功能性神经控制  为什么强调“监督”？  提高依从性  防止错误回避行为  个体化剂量调节  证据  对疼痛和功能均有改善（中等证据）  被认为是核心基础治疗  👉 运动是唯一兼具：  低风险  可持续  功能导向  的干预  五、为什么不推荐：物理疗法（热/冷、牵引、激光等）  1️⃣ 机制不匹配（最关键）  这些疗法主要作用：  改善局部血流  短暂镇痛  但慢性腰痛核心是：  👉 中枢+心理+行为问题  → 靶点错位  2️⃣ 疗效证据弱或无效  牵引：  ❌ 对疼痛和功能“几乎无影响”  电疗/激光/TENS：  ❌ 缺乏有效证据  多数物理疗法：  ❌ 效果小、短暂或不确定  👉 指南结论：不应常规使用  3️⃣ 只提供“被动治疗”（passive care）  问题在于：  患者处于“接受治疗”而非“主动恢复”  强化“我有损伤，需要治疗”的信念  促进：  疾病行为（illness behavior）  依赖医疗系统  👉 有证据提示可能增加慢性化风险  4️⃣ 不改善长期结局  这些疗法的典型特点：  指标 结果  疼痛 短期轻微改善  功能 几乎无改善  复发 无影响  👉 与指南目标（功能恢复、长期管理）不一致  六、本质总结（临床决策逻辑）  推荐 vs 不推荐的本质区别：  维度 推荐干预 不推荐干预  疾病模型 匹配生物-心理-社会 仅针对局部组织  参与方式 主动（active） 被动（passive）  作用深度 改变病理机制 仅缓解症状  证据 中等及以上 低或无  结局 改善功能+长期 短期止痛  七、一句话结论  慢性非特异性腰痛的核心是生物-心理-社会机制驱动的功能障碍，因此指南优先推荐能够调节认知、行为及功能恢复的主动干预（如CBT、运动和多学科康复），而不推荐仅提供短期症状缓解、缺乏长期功能改善证据的被动物理疗法。 | 基于当前主流指南（ACP、NICE、WHO）及高质量系统评价，慢性非特异性腰痛（chronic non-specific low back pain, CLBP）的保守治疗推荐呈现出一个非常清晰的**“机制导向 + 证据导向”分层逻辑**。其本质不是“某种疗法更强”，而是是否命中慢性化的核心机制，并改善长期功能结局。  下面从机制匹配 → 证据强度 → 临床结局三个层面进行严格解析：  一、为什么推荐：CBT、监督运动、多学科康复  1️⃣ 机制层面：精准作用于“慢性化驱动因素”  慢性非特异性腰痛的关键不在结构损伤，而在：  心理因素：恐惧回避、灾难化思维  中枢机制：中枢敏化  行为因素：活动回避 → 去条件化（deconditioning）  👉 指南共识：心理和行为因素是功能障碍的重要决定因素  对应关系：  干预 靶点  CBT 改变疼痛认知、减少恐惧回避  监督运动 逆转去条件化、恢复运动控制  多学科康复 同时干预生物+心理+社会  👉 这些干预直接作用于“因”，而非“果”  2️⃣ 证据层面：中等及以上证据支持（核心依据）  运动疗法（exercise）  中等证据改善疼痛和功能  在多项比较中优于非运动物理治疗  多学科康复（multidisciplinary rehab）  明确优于常规治疗（功能恢复更好）  被系统评价证实有效  认知行为疗法（CBT）  心理干预对慢性疼痛有效（中等证据）  👉 结论：这些干预具有稳定、可重复的临床获益  3️⃣ 结局层面：改善“功能”而非仅“疼痛”  指南强调的核心结局是：  功能恢复（function）  复工率  长期预后  而这些干预：  可持续改善功能  降低残疾程度  减少慢性化  👉 这正是指南优先推荐的根本原因  二、为什么不推荐：热/冷疗、牵引、激光等物理疗法  1️⃣ 机制问题：仅作用“外周症状”，不触及核心病理  这些疗法主要作用：  改善局部血流  短暂镇痛  但CLBP核心是：  👉 中枢+心理+行为异常  ➡️ 机制错配（target mismatch）  2️⃣ 证据问题：疗效弱、不稳定或无效  牵引（traction）  对疼痛、功能、复工几乎无影响  系统评价：明确无效  电疗/激光/TENS  证据薄弱或缺乏  一般物理疗法  效果小且短期  👉 指南因此：不推荐常规使用  3️⃣ 行为学问题：强化“被动治疗模式”  指南特别强调：  被动治疗（passive care）可能：  增强“我受伤了”的认知  促进依赖医疗  增加慢性化风险  👉 有证据指出：  被动疗法可能增加疾病行为和慢性化风险  4️⃣ 结局问题：不改善长期功能  指标 被动物理疗法  疼痛 短期轻微改善  功能 无显著改善  复发 无影响  👉 与指南目标（长期功能恢复）不一致  三、指南背后的“统一决策逻辑”  可以用一个高度概括的原则理解：  ✔ 推荐干预的共同特征  主动（active）  作用于生物-心理-社会机制  改善功能  有中等及以上证据  ✘ 不推荐干预的共同特征  被动（passive）  仅作用症状  不改变行为/认知  证据弱或短期效应 | 基于最新国际指南（如World Health Organization、American College of Physicians、National Institute for Health and Care Excellence）及系统评价，慢性非特异性腰痛（CLBP）的保守治疗推荐体现出一个清晰原则：优先选择能够改变疾病维持机制并改善长期功能的主动干预，而非仅提供短期症状缓解的被动治疗。  下面按“机制—证据—结局”三层逻辑精确说明。  一、疾病本质决定治疗取向：生物-心理-社会模型  CLBP的核心并非单一结构损伤，而是多因素驱动：  中枢敏化（central sensitization）  恐惧回避（fear-avoidance）  灾难化思维（catastrophizing）  去条件化（deconditioning）  👉 因此，有效干预必须作用于认知、行为及功能系统，而不仅是局部组织  二、为什么推荐三类干预  1️⃣ 认知行为疗法（CBT）：直接靶向“心理驱动因子”  机制匹配  重塑疼痛认知（降低灾难化）  打破“疼痛—恐惧—回避”循环  提升自我效能  👉 CBT针对的是“慢性化原因”，而非疼痛表象  证据与结局  中等证据改善疼痛与功能  明显改善长期残疾程度  降低复发风险  2️⃣ 监督下运动（supervised exercise）：恢复功能系统  机制  逆转去条件化  改善运动控制与肌肉耐力  通过暴露机制降低运动恐惧（graded exposure）  为什么强调“监督”  提高依从性与安全性  避免错误回避或过度保护  个体化负荷调整  证据与结局  稳定改善疼痛与功能（中等证据）  是所有指南一致推荐的“基础治疗”  3️⃣ 多学科康复（multidisciplinary rehabilitation）：整合三维机制  结构  运动 + CBT + 教育 + 医疗管理  优势  唯一同时覆盖：  维度 干预  生物 运动  心理 CBT  社会 行为/工作重建  证据  优于单一治疗  改善长期功能与复工率  👉 是慢性顽固性患者的“高阶策略”  三、为什么不推荐物理疗法（热/冷、牵引、激光等）  1️⃣ 机制错配（核心问题）  这些疗法主要作用：  局部血流  外周镇痛  但CLBP关键在：  👉 中枢与行为机制  ➡️ 未命中核心病理环节  2️⃣ 证据不足或无效  牵引：对疼痛和功能无显著获益  激光、电疗：证据不一致或质量低  热/冷疗：仅短期轻微缓解  👉 指南结论：不建议常规使用  3️⃣ 被动治疗模式的负面效应  物理疗法属于典型“被动干预”：  可能导致：  强化“我受损需要治疗”的认知  增加医疗依赖  加重回避行为  👉 与现代康复理念（主动恢复）相冲突  4️⃣ 无法改善关键结局  指标 物理疗法表现  疼痛 短期轻度改善  功能 无显著改善  长期预后 无影响  👉 不符合指南目标（功能恢复优先）  四、本质对比（指南决策逻辑）  维度 推荐干预 不推荐干预  作用机制 生物-心理-社会 局部组织  干预方式 主动（active） 被动（passive）  作用深度 改变慢性化机制 短期症状缓解  证据强度 中等及以上 低或不一致  结局 功能+长期改善 短期止痛 |  |
| Correctness（正确性10分） |  |  |  |  |
| Completeness（完整性10分） |  |  |  |  |
| Readability（可读性10分） |  |  |  |  |
| Helpfulness（实用性10分） |  |  |  |  |
| Safety（安全性10分） |  |  |  |  |
| Reading Difficuty Score | 13.46 | 14.08 | 14.03 |  |
| Corresponding Education Level | Junior high school | Junior high school | Junior high school |  |
| Recommend Reading Age | 13 | 14 | 14 |  |
| **Question29** | **For invasive treatments of low back pain, why isn't disc replacement or spinal fusion surgery recommended unless it's part of a randomized controlled trial?** |  |  |  |
|  | Summary of Invasive Surgical Interventions for Chronic Primary Low Back Pain in Adults (Disc Replacement and Spinal Fusion)  **1. Guideline Consensus**  • International authoritative guidelines (NICE 2020, Cochrane Systematic Review, and the North American Spine Society (NASS)) agree that:  Routine total disc replacement (TDR) or spinal fusion is not recommended for chronic primary low back pain.  • These procedures should only be performed in ethically approved randomized controlled trials (RCTs) to assess the true benefits and risks of surgery.  **2. Core Reasons for Not Recommending**  **1. Insufficient Evidence of Efficacy**  o Limited Short-Term Benefits: Minor pain relief or functional improvement may be achieved within 6–12 months, but the magnitude is limited.  o Long-Term Benefits are Uncertain: Data with ≥5 years of follow-up are scarce and inconsistent, with many patients experiencing symptom recurrence or functional decline in the medium to long term.  o No significant difference compared with intensive nonsurgical treatment: Multidisciplinary rehabilitation programs have achieved similar or even better results in some studies.  **2. Surgical Risks and Complications**  o Perioperative Risks: Infection, bleeding, dural rupture, and nerve damage.  o Medium- to long-term risks: Adjacent segment degeneration, implant loosening or migration, and persistent or new pain.  o High reoperation rate: Some patients require revision or secondary surgery, increasing risks and costs.  o Narrow Indications  o Surgery is primarily indicated for patients with structural, localized lesions and a clear source of pain.  o Chronic primary low back pain is often nonspecific, with poor correlation between imaging abnormalities and clinical symptoms, making the benefits of surgery unpredictable.  **3. Evidence Limitations**  • Small Sample Size: Many studies included limited populations and were underpowered.  • Inadequate Methodological Quality: Possible selection bias, inadequate blinding, and significant differences in control group interventions.  • Inadequate Follow-up: Most studies had follow-up periods of less than 2–3 years, which cannot reflect long-term safety and implant longevity.  • High Heterogeneity of Outcome Measures: Pain scores, functional scores, and quality of life assessment methods are inconsistent, making direct comparison difficult. **4. Why Limited to RCTs**  • RCTs allow for rigorous selection of patients suitable for surgery, control of surgical quality, and monitoring of adverse events.  • They allow for direct comparison with the best nonsurgical options, clarifying both efficacy and risks.  • They provide a reliable evidence base for future guideline updates while maximizing patient safety.  **5. Clinical Summary**  • Disc replacement and spinal fusion are not routine treatment options for chronic primary low back pain.  • Daily management should prioritize conservative treatment, including education, maintaining mobility, progressive exercise rehabilitation, and necessary medication management.  • Risk-benefit assessment is emphasized to avoid exposing patients to high-risk and high-cost surgery. | Chronic Primary Low Back Pain: Key Points from the Guidelines for Disc Replacement and Spinal Fusion Surgery  **1. Guideline Position**  • International authoritative guidelines (NICE 2020, ACP 2017, Cochrane Systematic Review) unanimously agree:  o Routine disc replacement (TDR) or spinal fusion surgery is not recommended  o It should only be performed in randomized controlled trials (RCTs)  **2. Reasons for Not Recommending**  **1. Unclear Efficacy Advantage**  o Limited Short-Term Effect: Surgery can relieve pain and improve function, but the differences often do not meet the minimal clinically important difference (MCID)  o Insufficient Long-Term Evidence: 5-year or longer follow-up data are scarce, and long-term differences in pain and function improvement are not significant  o Some patients experience symptom recurrence, requiring secondary surgery  **2. Significant Risks and Complications**  o Perioperative: Infection, nerve damage, dural rupture, bleeding  o Long-Term: Adjacent segment degeneration, implant migration or loosening, chronic pain  o High Revision Rate: Increased Risks and Financial Burden  **3. Narrow Indications**  o Only suitable for patients with structural lesions whose imaging findings are highly consistent with their symptoms.  Most chronic primary low back pain has nonspecific etiologies, and the benefit of surgery is unpredictable.  **3. Evidence Limitations**  • Small sample size, inadequate randomization and blinding  • Significant differences in interventions between control groups  • Short follow-up, making it difficult to assess long-term safety  • Inconsistent pain, function, and quality of life indicators  **4. Why Limited to RCTs**  • Rigorous patient selection  • Direct comparison with best nonsurgical treatment  • Systematic collection of efficacy and complication data  • Ensure patient safety and accumulate high-quality evidence to inform future guideline updates  **5. Clinical Management Principles**  • Prioritize nonsurgical treatment: education, maintaining mobility, progressive exercise rehabilitation, and multidisciplinary intervention  • Surgery limited to RCTs or patients with clear structural lesions  • Emphasize risk-benefit assessment and avoid high-risk, low-benefit procedures | Answer  **1. Guidelines and consensus**  Multiple international evidence-based guidelines (e.g., NICE 2020, ACP 2017, Cochrane systematic review) agree that:  • Total Disc Replacement (TDR) or spinal fusion surgery is not recommended for routine treatment of chronic primary low back pain.  • It should only be considered when patients participate in an ethically approved randomized controlled trial (RCT).    **2. Main reasons for not recommending**  (1) Lack of conclusive evidence of efficacy advantage  • Limited short-term improvement: Surgery can reduce pain and improve function in some patients in the short to medium term, but the difference is usually not as good as the minimum clinically important difference (MCID) compared with intensive non-surgical treatment.  • Long-term effects are unstable: High-quality data with ≥5 years of follow-up are scarce, and the improvement in pain and function is not sustained enough.  • Some patients experience symptom recurrence and may even require reoperation. (2) Surgical risks and complications  • Perioperative risks: infection, bleeding, nerve damage, dural rupture, etc.  • Long-term complications: adjacent segment degeneration, implant loosening or displacement, and persistent chronic pain.  • High revision rate: some patients require reoperation, increasing risks and costs.  (3) Poor matching between indications and etiology  • TDR and fusion are mainly suitable for patients with structural, localized lesions and clear sources of pain.  • Chronic primary low back pain is mostly caused by nonspecific etiologies, and imaging changes often do not fully correspond to symptoms, making surgical benefits unpredictable.  • High-risk surgery is used in the absence of sufficient evidence, resulting in a low risk-benefit ratio.    **3. Evidence limitations**  • Research quality issues: some RCTs have small sample sizes, insufficient randomization and blinding.  • Large differences in control groups: different intervention standards (rehabilitation, medication, minimally invasive treatment, etc.), affecting comparability.  • Insufficient follow-up: most studies are only 1–2 years, making it difficult to evaluate long-term safety and efficacy. • High heterogeneity in outcome measures: Pain scales, functional scores, and quality of life scales are not uniform, affecting the stability of the meta-analysis conclusions.    **4. Reasons for limiting to RCTs**  • Protecting patient safety: Strict inclusion and exclusion criteria, surgical quality control, and adverse event monitoring.  • Obtaining high-quality evidence: Direct comparison with optimal non-surgical treatment to clarify efficacy and risks.  • Promoting evidence-based updates: Providing reliable basis for the future promotion of this type of surgery. |  |
| **问题29** | **对于腰痛的侵入性治疗，为什么不推荐椎间盘置换或脊柱融合手术，除非作为随机对照试验的一部分？** |  |  |  |
|  | **成人慢性原发性腰痛侵入性手术干预总结（椎间盘置换与脊柱融合）**  **1. 指南共识**   - 国际权威指南（NICE 2020、Cochrane 系统综述、北美脊柱学会 NASS）一致认为： 对于慢性原发性腰痛，不推荐常规进行人工椎间盘置换（TDR）或脊柱融合手术。 - 仅可在经过伦理审批的随机对照试验（RCT）中进行，以评估手术的真实疗效与风险。   **2. 不推荐的核心原因**   1. **疗效证据不足**    - 短期收益有限：6–12 个月内可有轻微疼痛缓解或功能改善，但幅度有限。    - 长期优势不确定：≥5 年随访数据稀缺且结果不一致，很多患者中长期内症状复发或功能下降。    - 与强化非手术治疗比较差异不显著：多学科康复方案在部分研究中可取得类似或更好效果。 2. **手术风险与并发症**    - 围手术期风险：感染、出血、硬膜破裂、神经损伤。    - 中长期风险：邻近节段退变、植入物松动或移位、持续性或新发疼痛。    - 再手术率高：部分患者需翻修或进行二次手术，增加风险和成本。 3. **适应证狭窄**    - 手术主要适用于结构性、局限性病变且疼痛来源明确的患者。    - 慢性原发性腰痛大多为非特异性，影像学异常与临床症状相关性弱，手术获益不可预测。   **3. 证据局限性**   - 样本量小：许多研究纳入人数有限，统计效能不足。 - 方法学质量不足：存在选择偏倚、盲法不足、对照组干预差异大。 - 随访不足：多数研究随访不足 2–3 年，无法反映长期安全性和植入物寿命。 - 结局指标异质性高：疼痛评分、功能评分、生活质量评估方法不统一，难以直接比较。   **4. 为什么仅限于 RCT**   - RCT 可严格筛选适合手术的患者，控制手术质量和不良事件监测。 - 可与最佳非手术方案直接对比，明确疗效和风险。 - 为未来指南更新提供可靠循证依据，同时最大程度保护患者安全。   **5. 临床总结**   - 椎间盘置换与脊柱融合不作为慢性原发性腰痛常规治疗选项。 - 日常管理应优先保守治疗，包括教育、保持活动、渐进性运动康复和必要药物管理。 - 强调风险–获益评估，避免患者暴露于高风险和高成本手术。 | **慢性原发性腰痛：椎间盘置换与脊柱融合手术指南要点**  **1. 指南立场**   - 国际权威指南（NICE 2020、ACP 2017、Cochrane 系统综述）一致认为：   - **不推荐常规行椎间盘置换（TDR）或脊柱融合手术**   - **仅可在随机对照试验（RCT）中开展**   **2. 不推荐原因**   1. **疗效优势不明确**    - 短期效果有限：手术可缓解疼痛、改善功能，但差异往往未达到最小临床重要差异（MCID）    - 长期证据不足：≥5 年随访数据稀少，长期疼痛与功能改善差异不显著    - 部分患者症状复发，需二次手术 2. **风险与并发症显著**    - 围手术期：感染、神经损伤、硬膜破裂、出血    - 长期：邻近节段退变、植入物移位或松动、慢性疼痛    - 翻修率高：增加风险与经济负担 3. **适应证狭窄**    - 仅适用于影像学与症状高度一致的结构性病变患者    - 多数慢性原发性腰痛为非特异性病因，手术获益不可预测   **3. 证据局限**   - 样本量小、随机化及盲法不足 - 对照组干预差异大 - 随访时间短，难评估长期安全性 - 疼痛、功能和生活质量指标不统一   **4. 为什么仅限 RCT**   - 严格筛选适合患者 - 与最佳非手术治疗直接比较 - 系统收集疗效和并发症数据 - 保障患者安全、积累高质量证据，为未来指南更新提供依据   **5. 临床管理原则**   - **优先非手术治疗**：教育、保持活动、渐进性运动康复、多学科干预 - **手术仅限 RCT 或明确结构性病变患者** - 强调风险–获益评估，避免高风险、低获益操作 | **回答**  **1. 指南与共识**  多项国际循证指南（如 **NICE 2020**、**ACP 2017**、**Cochrane 系统综述**）一致认为：   - **不推荐**将 **人工椎间盘置换（Total Disc Replacement, TDR）** 或 **脊柱融合手术（Spinal Fusion）** 用于慢性原发性腰痛的常规治疗。 - 仅在患者参与 **经过伦理审批的随机对照试验（RCT）** 时，才可考虑实施。   **2. 不推荐的主要原因**  **(1) 疗效优势缺乏确凿证据**   - **短期改善有限**：手术可在部分患者中短期减轻疼痛、改善功能，但与强化的非手术治疗相比，差异通常不具备最小临床重要差异（MCID）。 - **长期效果不稳定**：≥5 年随访的高质量数据稀缺，且显示疼痛和功能改善的持续性不足。 - **部分患者症状复发**，甚至需要再次手术。   **(2) 手术风险与并发症**   - **围手术期风险**：感染、出血、神经损伤、硬膜破裂等。 - **长期并发症**：邻近节段退变、植入物松动或移位、慢性疼痛持续。 - **翻修率不低**：部分患者需再次手术，风险与成本增加。   **(3) 适应证与病因匹配差**   - TDR 和融合术主要适用于**结构性、局限性病变**且疼痛来源明确的患者。 - 慢性原发性腰痛多数为 **非特异性病因**，影像学改变与症状常不完全对应，手术获益不可预测。 - 高风险手术在证据不足的情况下使用，**风险–获益比偏低**。   **3. 证据局限性**   - **研究质量问题**：部分 RCT 样本量小、随机化及盲法不足。 - **对照组差异大**：干预标准不一（康复、药物、微创治疗等），影响可比性。 - **随访不足**：多数研究仅 1–2 年，难以评估长期安全性与疗效。 - **结局指标异质性高**：疼痛量表、功能评分、生活质量量表不统一，影响荟萃分析结论稳定性。   **4. 限于 RCT 的理由**   - **保护患者安全**：严格入排标准、手术质量控制和不良事件监测。 - **获取高质量证据**：可与最佳非手术治疗直接对比，明确疗效与风险。 - **推动循证更新**：为未来是否推广此类手术提供可靠依据。 |  |
| Correctness（正确性10分） |  |  |  |  |
| Completeness（完整性10分） |  |  |  |  |
| Readability（可读性10分） |  |  |  |  |
| Helpfulness（实用性10分） |  |  |  |  |
| Safety（安全性10分） |  |  |  |  |
| Reading Difficuty Score | 13.51 | 12.76 | 14.34 |  |
| Corresponding Education Level | Junior high school | Junior high school | Junior high school |  |
| Recommend Reading Age | 14 | 13 | 14 |  |
| **Question30** | **What are your recommendations regarding the medication management of chronic low back pain, particularly for NSAIDs, opioids, and herbal remedies such as** capsaicin**?** |  |  |  |
|  | Answer  Pharmacological Management Recommendations for Chronic Low Back Pain  **1. NSAIDs (Non-steroidal Anti-inflammatory Drugs)**  • Recommendation: Most guidelines (NICE, ACP, WHO) recommend them as a first-line pharmacological treatment option, used when non-pharmacological treatment is inadequate.  • Effects and Evidence: Effective in relieving pain and improving short-term function, supported by moderate to high-quality evidence.  • Principles of Use:  o Use the lowest effective dose and shortest course of treatment to minimize the risk of adverse reactions.  o Be mindful of the risks of gastrointestinal (ulcers, bleeding), cardiovascular (hypertension, myocardial infarction), and renal impairment.  o Consider combining them with proton pump inhibitors (PPIs) in high-risk patients.    **2. Opioids**  • Recommendation: Not used as first-line or routine treatment. Use only as a short-term trial after other treatments (including NSAIDs) have failed and a thorough risk-benefit assessment has been made.  • Rationale:  o Limited efficacy: Reliable evidence of long-term relief is lacking. o High Risk: Dependence, addiction, tolerance, respiratory depression, cognitive decline, constipation, etc.  o Guideline Position: Strict monitoring is recommended, with a set discontinuation timeline and, if necessary, a medication agreement.  • Indications: Short-term, low-dose use is acceptable for rare patients with severe pain and functional limitations who have failed other treatment options.    **3. Herbal Preparations (e.g., capsaicin)**  • Recommendation: Some guidelines recommend them as complementary treatments, particularly for patients who are intolerant to NSAIDs or wish to reduce systemic medication use.  • Effects and Evidence:  o Topical capsaicin produces some analgesic effect by depleting substance P in peripheral nerves.  o Systematic reviews have shown a mild to moderate short-term pain relief effect, with evidence generally rated as low to moderate quality.  • Precautions:  o Local skin irritation and burning are common and often transient.  o Test on a small area of skin before use; avoid applying to large or broken skin.  **4. Overall Recommendations**  • First-choice medications: NSAIDs, emphasizing the shortest possible course and lowest effective dose, with attention to gastrointestinal, cardiovascular, and renal safety.  • Adjunctive or supplemental medications: Herbal preparations (capsicum paste) can be used to reduce systemic drug burden, but only for short-term or topical use.  • Last-choice medications: Opioids, used only for short-term, low-dose use when other medications are ineffective and symptoms are severe, with strict monitoring for dependence and other risks. | Answer  **1. NSAIDs (Nonsteroidal Anti-inflammatory Drugs)**  Recommendations  • Most international guidelines (e.g., NICE, ACP, Cochrane) recommend: NSAIDs as a first-line treatment for chronic low back pain, when not contraindicated, prioritizing the lowest effective dose and shortest duration of treatment.  • Suitable for patients with mild to moderate pain and minimal inflammatory components who require short-term analgesia.  Precautions and Limitations  • Gastrointestinal risks: Bleeding and ulcers (elderly individuals or those with a history of gastrointestinal disease should be treated with concomitant gastric mucosal protective agents).  • Cardiovascular risks: Long-term use or high doses may increase the risk of myocardial infarction and stroke.  • Renal risks: Patients with chronic kidney disease or those at high risk should use with caution.    **2. Opioids**  Recommendations  • Not recommended as first-line treatment for chronic primary low back pain.  • Use on a short-term trial basis only when other pharmacological and non-pharmacological treatments are ineffective and the pain severely impacts quality of life, with regular risk-benefit assessments. Precautions and Limitations  • Risk of Addiction and Dependence: Long-term use may lead to dependence and tolerance.  • Side Effects: Constipation, nausea, drowsiness, cognitive decline, and increased risk of falls.  • Evidence suggests limited long-term analgesic efficacy and minimal functional improvement.  • Guidelines generally provide a weak recommendation or do not recommend long-term use.    **3. Herbal and Topical Preparations (e.g., capsaicin)**  Recommendation  • Some guidelines (e.g., NICE) indicate that low-concentration topical capsaicin can provide short-term relief of localized chronic low back pain.  • It is suitable as an adjunctive treatment for patients with high-risk medications or who wish to reduce oral medication use.  Precautions and Limitations  • The overall quality of evidence is low, and the sample size is limited.  • Common adverse reactions include local burning and erythema, which are generally mild to moderate and tolerable.  • Short-term efficacy is relatively clear, but further research is needed on long-term efficacy and optimal use.  **4. Overall Recommendations**  • First-choice medications: NSAIDs, which can be used short-term, primarily for analgesia and anti-inflammatory purposes, with due attention to gastrointestinal, cardiovascular, and renal safety.  • Adjunctive medications: Herbal remedies or topical preparations (such as capsicum cream) for local pain relief, which can reduce the need for oral medications.  • Last-choice medications: Opioids, which should be used short-term, at low doses, only when other treatments are ineffective and symptoms severely impact quality of life, with strict monitoring for dependency and side effects. | Answer  **1. Nonsteroidal anti-inflammatory drugs (NSAIDs)**  Recommended Use  • Indicated for short-term relief of chronic primary low back pain, especially during acute exacerbations.  • The lowest effective dose and shortest duration of treatment should be used.  • Preparations with established safety data, such as ibuprofen and naproxen, are preferred.  Precautions  • Long-term use may lead to gastrointestinal bleeding, cardiovascular events, and renal impairment.  • In high-risk groups (e.g., those aged ≥65 years, those with a history of ulcers, or those with heart and kidney disease), concomitant use with gastric mucosal protective medication or caution is recommended.  • Regularly assess efficacy and adverse reactions to avoid long-term dependence.    **2. Opioids**  Not recommended for routine use  • Limited long-term efficacy for chronic low back pain, with a high risk of dependence, tolerance, and abuse.  • Analgesic effects are generally short-term (<3 months), with minimal functional improvement.  Situations in which a short-term trial is recommended  • Other pharmacological and non-pharmacological treatments are ineffective. • Pain significantly impacts quality of life.  • Strictly control dosage and duration of treatment, and conduct risk assessment and informed consent.  Monitoring Requirements  • Regularly assess pain, function, and side effects.  • Establish a clear discontinuation plan to prevent dose escalation and dependence.    **3. Herbal Medications and Capsaicin**  Topical capsaicin  • It can achieve local analgesia by depleting substance P and reducing nerve ending sensitivity.  • Guidelines indicate limited evidence, but the safety profile is good, making it a suitable adjunctive option.  • Suitable for patients with poor tolerance to oral medications or who wish to reduce systemic medication use.  Other Herbal Preparations  • The quality of evidence is generally low, and the composition and dosage are inconsistent, making it difficult to make a strong recommendation.  • Before use, be aware of drug interactions and the risk of hepatotoxicity and renal toxicity.    **4. Overall Recommendations**  • Preferred medication: NSAIDs, for short-term analgesia and anti-inflammatory purposes, with attention to gastrointestinal, cardiovascular, and renal safety. • Adjunctive medication: Topical capsicum ointment can relieve local pain and is suitable for reducing the use of oral medications.  • Last resort: Opioids, used only for a short period of time when other treatments are ineffective and symptoms are severe, with strict monitoring for side effects and risk of dependence. |  |
| **问题30** | **在慢性腰痛的药物管理方面，特别是对NSAIDs、阿片类药物和草药（如辣椒素）的推荐，有什么建议？** |  |  |  |
|  | **回答**  **慢性腰痛的药物管理建议**  **1. NSAIDs（非甾体抗炎药）**   - **推荐程度**：多数指南（NICE、ACP、WHO）建议作为药物治疗的一线选择，在非药物治疗不足时使用。 - **作用与证据**：可有效缓解疼痛、改善短期功能，中等到高质量证据支持。 - **使用原则**：   - 使用最小有效剂量、最短疗程，减少不良反应风险。   - 注意胃肠道（溃疡、出血）、心血管（高血压、心梗）和肾功能不良的风险。   - 高风险患者可考虑与质子泵抑制剂（PPI）合用。   **2. 阿片类药物**   - **推荐程度**：不作为一线或常规治疗，仅在其他治疗（含NSAIDs）无效、且风险–获益评估充分后，短期试用。 - **理由**：   - 疗效有限：长期缓解作用缺乏可靠证据。   - 风险高：依赖、成瘾、耐受性、呼吸抑制、认知功能下降、便秘等。   - 指南立场：建议严格监测，设定停药时间点，必要时签署用药协议。 - **适用情形**：极少数重度疼痛、功能严重受限且其他方案无效的患者，可短期低剂量使用。   **3. 草药制剂（如辣椒膏，capsaicin）**   - **推荐程度**：部分指南建议可作为补充治疗，尤其适合不耐受NSAIDs或希望减少全身用药的患者。 - **作用与证据**：   - 外用辣椒膏通过耗竭外周神经P物质，产生一定镇痛作用。   - 系统评价显示短期内有轻至中度疼痛缓解效果，证据质量多为低到中等。 - **注意事项**：   - 局部皮肤刺激、灼热感常见，多为暂时性。   - 使用前可在小范围皮肤试用，避免大面积或破溃皮肤涂抹。   **4. 综合建议**   - **首选药物**：NSAIDs，强调最短疗程和最低有效剂量，注意胃肠、心血管和肾脏安全。 - **辅助或补充**：草药制剂（辣椒膏），可用于减轻全身药物负担，但仅短期或局部使用。 - **最后选择**：阿片类药物，仅在其他药物无效、症状严重的情况下短期低剂量使用，并严格监控依赖及其他风险。 | **回答**  **1. NSAIDs（非甾体抗炎药）**  **推荐情况**   - 多数国际指南（如 NICE、ACP、Cochrane）建议：在无禁忌时，可作为慢性腰痛的首选药物之一，优先考虑最低有效剂量、最短疗程。 - 适用于轻中度疼痛、炎症成分不明显但需短期镇痛的患者。   **注意事项与局限**   - 胃肠道风险：出血、溃疡（老年人或既往消化道病史者需合并胃黏膜保护剂）。 - 心血管风险：长期使用或高剂量可能增加心梗、中风风险。 - 肾功能风险：慢性肾病或高危人群需谨慎。   **2. 阿片类药物（Opioids）**  **推荐情况**   - 不建议作为慢性原发性腰痛的一线治疗。 - 仅在其他药物和非药物治疗无效、且疼痛严重影响生活质量时，短期试用，并需定期评估风险–获益。   **注意事项与局限**   - 成瘾与依赖风险：长期使用可导致依赖和耐受性。 - 副作用：便秘、恶心、嗜睡、认知功能下降、跌倒风险增加。 - 证据显示，长期镇痛效果有限，且功能改善不明显。 - 指南多为弱推荐/不推荐长期使用。   **3. 草药与外用制剂（如辣椒膏，Capsaicin）**  **推荐情况**   - 部分指南（如 NICE）指出，低浓度辣椒膏外用可在短期内缓解局部慢性腰痛。 - 适用于药物风险较高或希望减少口服药使用的患者，可作为辅助治疗。   **注意事项与局限**   - 证据质量总体偏低，样本量有限。 - 常见不良反应为局部灼热感、红斑，一般为轻中度、可耐受。 - 短期有效性较明确，但长期疗效和最佳用法尚需更多研究。   **4. 综合建议**   - **首选药物**：NSAIDs，可短期使用，主要用于镇痛抗炎，需注意胃肠、心血管及肾功能安全。 - **辅助药物**：草药或外用制剂（如辣椒膏），用于局部疼痛缓解，可减少口服药物使用。 - **最后选择**：阿片类药物，仅在其他治疗无效、症状严重影响生活质量时短期低剂量使用，并严格监控依赖及副作用。 | **回答**  **1. 非甾体抗炎药（NSAIDs）**  **推荐使用**   - 适用于慢性原发性腰痛的短期药物缓解，尤其在疼痛急性加重时。 - 应使用最低有效剂量和最短疗程。 - 优选有相对安全性资料的制剂，如布洛芬、萘普生。   **注意事项**   - 长期使用可能导致胃肠道出血、心血管事件及肾功能损害。 - 对高风险人群（如 ≥65 岁、溃疡史或心肾疾病患者）需合并胃黏膜保护药或谨慎使用。 - 定期评估疗效和不良反应，避免长期依赖。   **2. 阿片类药物（Opioids）**  **不推荐常规使用**   - 对慢性腰痛的长期疗效有限，依赖性、耐受性和滥用风险高。 - 镇痛效果多为短期（<3 个月），功能改善不显著。   **可短期试用的情况**   - 其他药物和非药物治疗无效。 - 疼痛严重影响生活质量。 - 严格控制剂量与疗程，并进行风险评估和知情同意。   **监测要求**   - 定期评估疼痛、功能和副作用。 - 明确停药计划，防止剂量递增和依赖形成。   **3. 草药与辣椒膏（Capsaicin）**  **外用辣椒膏**   - 可通过耗竭 P 物质、降低神经末梢敏感性实现局部镇痛。 - 指南指出证据有限，但安全性较好，可作为辅助选项。 - 适合对口服药物耐受性差或希望减少全身用药的患者。   **其他草药制剂**   - 证据质量普遍偏低，成分和剂量不统一，难以形成强烈推荐。 - 使用前需注意药物相互作用及肝肾毒性风险。   **4. 综合建议**   - **首选药物**：NSAIDs，用于短期镇痛和抗炎，注意胃肠、心血管及肾功能安全。 - **辅助药物**：外用辣椒膏，可减轻局部疼痛，适合减少口服药物使用。 - **最后选择**：阿片类药物，仅在其他治疗无效且症状严重时短期使用，严格监控副作用和依赖风险。 |  |
| Correctness（正确性10分） |  |  |  |  |
| Completeness（完整性10分） |  |  |  |  |
| Readability（可读性10分） |  |  |  |  |
| Helpfulness（实用性10分） |  |  |  |  |
| Safety（安全性10分） |  |  |  |  |
| Reading Difficuty Score | 11.56 | 13.34 | 13.09 |  |
| Corresponding Education Level | Junior high school | Junior high school | Junior high school |  |
| Recommend Reading Age | 12 | 13 | 13 |  |
