# Supplementary material for: Deficiencies in clinical reasoning of LLMs in low back pain management and remediation via prompt engineering: from performance evaluation to error diagnosis
Source: Front Artif Intell. 2026 May 25;9:1811701. doi: 10.3389/frai.2026.1811701 (PMC13243384; doi:10.3389/frai.2026.1811701)
Supplement: Supplementary file 1 [file Data_Sheet_1.zip › 补充材料/Supplementary materials 3/S2(Grok4).docx]

| **Table S2: Grok-4 answers to 30** | | | | |
| --- | --- | --- | --- | --- |
| **Question1** | | **How to define and classify low back pain?** | | |
|  | | **Definition of Low Back Pain**  Low back pain (also known as lower back pain or lumbar pain, abbreviated as LBP) is a common musculoskeletal disorder characterized by pain in the lower back region, which extends from the lower edge of the ribs to the gluteal fold. The nature of the pain varies and may manifest as dull pain, stabbing pain, burning sensation, or radiating pain, often accompanied by muscle tension, stiffness, or limited mobility. Most cases of low back pain are non-specific, meaning that a specific cause cannot be identified, and are typically associated with injuries to muscles, ligaments, or intervertebral discs.  **Classification of Low Back Pain**  There are various methods for classifying low back pain, primarily based on duration, cause, and clinical characteristics. The following are common classification criteria:  **I. Classification by Duration**  Low back pain can be categorized into three types based on the duration of pain, which aids in guiding treatment and prognosis assessment:  • Acute lower back pain: Pain lasting less than six weeks. Typically caused by sudden injury, such as muscle strain or sprain, with a high rate of spontaneous resolution  • Subacute lower back pain: Pain lasting six to twelve weeks. Intermediate between acute and chronic, may require intervention to prevent progression to chronic  • Chronic low back pain: Pain lasting longer than twelve weeks. Often associated with lifestyle factors, psychological factors, or underlying diseases, making treatment more complex.  **2. Classification by etiology or specificity**  Based on whether the cause can be clearly identified, low back pain can be classified as:  • Nonspecific low back pain: Accounts for approximately 85–90% of all low back pain cases. Pain is localized to the lower back with no clear pathological cause, often associated with poor posture, muscle fatigue, or stress.  • Specific low back pain: Has a clear cause and can be further subdivided into:  o Mechanical low back pain: The most common, accounting for approximately 90%, including herniated discs, spinal stenosis, spondyloarthritis, fractures, or muscle and ligament injuries.  o Non-mechanical low back pain: Such as inflammatory (e.g., ankylosing spondylitis), infectious (e.g., discitis), neoplastic (e.g., spinal tumors), or metabolic (e.g., osteoporosis)  o Radiating or metastatic low back pain: Pain may extend to the buttocks or lower limbs, often caused by nerve root compression, or may originate from visceral organs such as kidney stones or endometriosis  **III. Other classification methods**  • By clinical presentation: simple low back pain (only lower back pain), radicular low back pain (with lower limb radiation pain), neurogenic low back pain (with sensory abnormalities or muscle weakness)  • By severity: mild (does not affect daily activities), moderate (affects work but tolerable), severe (requires bed rest or surgery)  These classifications are based on international medical standards, such as the World Health Organization's International Classification of Diseases, including the 10th edition M 54.5 and the 11th edition ME 84.**2. In clinical practice, diagnosis is often made in conjunction with imaging tests (such as MRI) or medical history. It is recommended that patients with low back pain consult a physician early to avoid self-diagnosis.** | **Definition of Low Back Pain**  Low back pain (LBP), also known as lower back pain or lumbar pain, refers to pain symptoms in the area between the lower rib margin and the gluteal fold (the crease at the buttocks). It is not a single disease but a symptom that may be accompanied by the following manifestations:  • Muscle tension  • Stiffness  • Limited mobility  • Pain radiating to the legs (e.g., sciatica)  The nature of the pain varies and may include:  • Dull pain  • Stabbing pain  • Burning sensation  Most low back pain is nonspecific, meaning that a specific pathological cause cannot be identified, and is often associated with the following factors:  • Poor posture  • Muscle strain  • Mental stress  • Age-related degenerative changes  According to the World Health Organization (WHO), low back pain is one of the leading causes of disability worldwide, significantly impacting daily life and work.  **Classification of low back pain**  There are various methods for classifying low back pain, primarily based on the following dimensions:  • Duration  • Etiology  • Clinical presentation  • Treatment orientation  **I. Classification by duration**  This is the most basic classification method, which helps assess prognosis and develop treatment strategies:  • Acute low back pain: lasting less than 6 weeks  o Typically caused by sudden injury (e.g., muscle strain or sprain)  o High self-resolution rate (approximately 70%–90%)  o Usually does not require special intervention  • Subacute low back pain: lasting 6 to 12 weeks  o Intermediate between acute and chronic  o May require early intervention (e.g., physical therapy) to prevent chronicity  • Chronic low back pain: lasting more than 12 weeks  o Often recurrent  o Associated with psychological and social factors  o Treatment is complex and may require multidisciplinary management  • Recurrent low back pain: intermittent and recurrent  o Typically reoccurs after a period of remission  o Common during acute exacerbations in patients with chronic low back pain  **II. Classification by etiology or specificity**  Based on whether the cause can be clearly identified, low back pain is divided into two major categories: nonspecific and specific:  • Nonspecific low back pain  o Accounts for 85%–95% of all low back pain  o No clear structural or organic lesions  o Often caused by abnormal posture, muscle strain, or mechanical stress  • Specific low back pain  o Has a clear cause, which requires imaging or laboratory tests to confirm  o Includes the following categories:  o Mechanical low back pain: The most common type, originating from the spine, intervertebral discs, or surrounding soft tissues, such as herniated discs, spinal stenosis, fractures, or muscle or ligament injuries  o Inflammatory low back pain: such as ankylosing spondylitis, characterized by morning stiffness and nighttime pain  o Infectious low back pain: such as discitis or spinal tuberculosis, often accompanied by fever and systemic symptoms  o Neoplastic low back pain: such as primary or metastatic spinal tumors, typically worsening at night  o Neurogenic low back pain: caused by nerve root compression, commonly seen in sciatica, accompanied by radiating pain or sensory abnormalities in the lower limbs  o Visceral-related low back pain: caused by diseases of the kidneys, gynecological system, or gastrointestinal system, such as kidney stones or endometriosis  o Nonociceptive low back pain (classified based on pain mechanisms): including discogenic pain, facet joint pain, sacroiliac joint pain, and myofascial pain, etc.  **III. Classification based on treatment or clinical orientation**  This classification system is primarily used to guide clinical treatment, especially the development of physical therapy and rehabilitation plans:  • Treatment-based classification (TBC system)  o Patients are divided into the following subgroups based on symptoms and signs: stabilization group, manipulation group, specific exercise group, traction group  o Helps in developing individualized intervention plans  • Clinical diagnosis classification  o Radicular low back pain: accompanied by lower limb radicular pain, indicating nerve root compression  o Simple low back pain: pain localized to the lower back without radicular symptoms  o Neurological deficit type: accompanied by muscle weakness, sensory loss, or abnormal reflexes  • Lumbar Spine Functional Impairment Classification  o Includes movement control disorders, directional preference disorders, etc.  o Applicable for assessment and management during rehabilitation and exercise therapy phases | **Definition of Low Back Pain**  Low back pain (LBP), also known as lower back pain or lumbar pain, refers to pain symptoms in the area between the lower rib margin and the gluteal fold (the crease at the buttocks). It is not a single disease but a common symptom that may be accompanied by muscle tension, stiffness, limited mobility, or pain radiating to the lower limbs. The nature of the pain varies, including dull pain, stabbing pain, burning sensation, or shooting pain. Most low back pain is nonspecific, meaning that a specific pathological cause cannot be identified, and is often associated with poor posture, muscle strain, obesity, smoking, or work-related stress.  According to the World Health Organization (WHO) and the International Association for the Study of Pain (IASP), low back pain is one of the leading causes of disability worldwide, affecting the quality of life and work capacity of people of all ages.  As of 2025, the definition has not undergone significant updates, but it emphasizes that it is a symptom rather than a disease.  Classification of low back pain  There are various methods for classifying low back pain, primarily based on duration, etiology (specificity), pain mechanisms, clinical manifestations, or risk stratification. The following are common classification criteria, referenced from international clinical guidelines (such as WHO, IASP, NICE) and the latest research (as of 2025).  Classification aids in guiding diagnosis, treatment, and prognosis assessment.  **1. Classification by Duration**  This is the most basic classification method, with slight variations across different guidelines, but it helps assess prognosis. Common criteria include:  Classification Type: Acute Low Back Pain  Duration: <6 weeks (some guidelines specify <4 weeks or <3 months)  Characteristics and Prognosis: Typically caused by sudden injury (e.g., muscle strain, sprain), with a high self-resolution rate (70%-90%) and no need for special intervention.  Classification Type: Subacute Low Back Pain  Duration: 6–12 weeks  Characteristics and Prognosis: Intermediate between acute and chronic, may require early intervention (e.g., physical therapy) to prevent progression to chronicity.  Classification Type: Chronic Low Back Pain  Duration: >12 weeks (some guidelines >3 months)  Characteristics and prognosis: Often recurrent, associated with psychological and social factors, complex to treat, may require multidisciplinary management (e.g., psychological intervention).  Classification type: Recurrent low back pain  Duration: Intermittent episodes  Characteristics and prognosis: Recurs after periods of remission, commonly seen in acute episodes on a chronic background.  Note: The NICE guidelines define acute as <3 months and chronic as ≥3 months, while the WHO further subdivides it into subacute.  **2. Classification by etiology or specificity**  Based on whether the etiology can be clearly identified, it is divided into nonspecific and specific low back pain. Among these, nonspecific accounts for 85%-90%, and imaging or laboratory tests are required to confirm specificity.  • Nonspecific low back pain: No clear pathological cause, pain is limited to the lower back, often caused by mechanical stress, soft tissue injury, or lifestyle factors.  • Specific low back pain: Has a clear cause, which can be further subdivided:  o Mechanical low back pain: The most common type, originating from spinal structures such as herniated discs, spinal stenosis, fractures, or muscle/ligament injuries.  o Inflammatory low back pain: Such as ankylosing spondylitis, accompanied by morning stiffness and night pain.  o Infectious low back pain: Such as discitis or spinal tuberculosis, accompanied by fever.  o Neoplastic low back pain: such as spinal tumors or metastatic cancer, often worsening at night.  o Visceral-related low back pain: originating from kidney, gynecological, or gastrointestinal diseases, such as kidney stones or endometriosis.  o Other: such as osteoporosis or aneurysms.  **3. Classification by pain mechanism (emerging classification)**  Based on the latest research in 2025, low back pain is classified by pain mechanism to guide precise treatment:  • Nociceptive: caused by tissue damage, such as muscle strain or arthritis.  • Neuropathic: caused by nerve damage, such as sciatica with radiating pain.  • Nociplastic: No clear injury, but central nervous system sensitization, such as chronic nonspecific low back pain.  **4. Other classification methods**  • By clinical presentation: Simple low back pain (only lower back), radicular low back pain (with lower limb radiating pain, such as sciatica), neurogenic low back pain (with sensory abnormalities or muscle weakness).  • Risk stratification: Use tools such as STarT Back to assess low, moderate, or high risk to guide treatment intensity (low risk: self-management; high risk: comprehensive intervention). |
| **问题1** | | **如何定义和分类腰痛？** | | |
|  | | **腰痛的定义**  腰痛（也称下背痛或低背痛，英文為 Low Back Pain，LBP）是一种常见的肌肉骨骼系统疾病，指位于肋骨下缘至臀褶之间的下背部区域出现疼痛。疼痛性质多样，可表现为钝痛、刺痛、烧灼感或放射性疼痛，常伴随肌肉紧张、僵硬或活动受限。大多数腰痛为非特异性，即无法明确特定病因，通常与肌肉、韧带或椎间盘的损伤相关。  **腰痛的分类**  腰痛的分类方法多样，主要基于持续时间、病因和临床特征等维度。以下是常见的分类标准：  **一 按持续时间分类**  腰痛可根据疼痛持续的时间分为三类，有助于指导治疗和预后评估：   - 急性腰痛：疼痛持续时间少于六周。通常由突发性损伤引起，如肌肉拉伤或扭伤，自愈率高 - 亚急性腰痛：疼痛持续六至十二周。介于急性和慢性之间，可能需干预以防转为慢性 - 慢性腰痛：疼痛持续超过十二周。常与生活方式、心理因素或潜在疾病相关，治疗更复杂   **二 按病因或特异性分类**  根据是否能明确病因，腰痛可分为：   - 非特异性腰痛：约占所有腰痛的百分之八十五至九十，疼痛限于下背部，无明确病理学原因，常与姿势不良、肌肉疲劳或压力相关 - 特异性腰痛：有明确病因，可进一步细分为   - 机械性腰痛：最常见，约占百分之九十，包括椎间盘突出、椎管狭窄、脊柱关节炎、骨折或肌肉韧带损伤   - 非机械性腰痛：如炎症性（如强直性脊柱炎）、感染性（如椎间盘炎）、肿瘤性（如脊柱肿瘤）或代谢性（如骨质疏松）   - 放射性或转移性腰痛：疼痛可延伸至臀部或下肢，常由神经根压迫引起，也可能源于内脏器官，如肾结石、子宫内膜异位症等   **三 其他分类方式**   - 按临床表现：单纯性腰痛（仅下背部疼痛）、根性腰痛（伴下肢放射痛）、神经源性腰痛（伴感觉异常或肌无力） - 按严重程度：轻度（不影响日常活动）、中度（影响工作但可忍受）、重度（需卧床休息或手术）   这些分类基于国际医疗标准，如世界卫生组织的国际疾病分类，包括第十版 M 五四点五 及第十一版 ME 八四点二。临床实践中常结合影像学检查（如磁共振）或病史进行诊断。建议腰痛患者及早咨询医师，避免自行判断。 | **腰痛的定义**  腰痛（Low Back Pain，简称LBP），也称为下背痛或低背痛，是指位于下肋骨边缘至臀褶（臀部皱褶）之间区域的疼痛症状。它不是单一疾病，而是一种症状，可能伴随以下表现：   - 肌肉紧张 - 僵硬 - 活动受限 - 放射至腿部的疼痛（如坐骨神经痛）   疼痛性质多样，包括：   - 钝痛 - 刺痛 - 烧灼感   大多数腰痛为非特异性，即无法明确特定病理原因，常与以下因素有关：   - 姿势不良 - 肌肉劳损 - 精神压力 - 年龄相关的退行性改变   据世界卫生组织（WHO）描述，腰痛是全球主要的致残原因之一，严重影响日常生活和工作。  **腰痛的分类**  腰痛的分类方法多种多样，主要基于以下维度：   - 持续时间 - 病因 - 临床表现 - 治疗导向   **一、按持续时间分类**  这是最基本的分类方式，有助于评估预后与制定治疗策略：   - 急性腰痛：持续少于6周   - 通常由突发损伤（如肌肉拉伤或扭伤）引起   - 自愈率高（约70%–90%）   - 通常无需特殊干预 - 亚急性腰痛：持续6至12周   - 介于急性与慢性之间   - 可能需要早期干预（如物理治疗）以防慢性化 - 慢性腰痛：持续超过12周   - 常反复发作   - 与心理、社会因素相关   - 治疗复杂，可能需要多学科管理 - 复发性腰痛：间歇性反复发作   - 通常在缓解期后再次出现   - 多见于慢性腰痛患者的急性加重期   **二、按病因或特异性分类**  根据是否能明确病因，腰痛分为非特异性和特异性两大类：   - 非特异性腰痛   - 占所有腰痛的85%–95%   - 无明确结构或器质性病变   - 多因姿势异常、肌肉劳损或机械应力引起 - 特异性腰痛   - 具有明确病因，需影像学或实验室检查确认   - 包括以下几类：   - 机械性腰痛：最常见，源于脊柱、椎间盘或周围软组织，如椎间盘突出、椎管狭窄、骨折、肌肉或韧带损伤   - 炎症性腰痛：如强直性脊柱炎，表现为晨僵和夜间疼痛   - 感染性腰痛：如椎间盘炎或脊柱结核，常伴发热和全身症状   - 肿瘤性腰痛：如原发或转移性脊柱肿瘤，通常夜间加重   - 神经源性腰痛：因神经根受压，常见于坐骨神经痛，伴下肢放射痛或感觉异常   - 内脏相关腰痛：由肾脏、妇科或胃肠系统疾病引起，如肾结石、子宫内膜异位症等   - 伤害感受性腰痛（基于疼痛机制分类）：包括椎间盘源性痛、椎小关节痛、骶髂关节痛和肌筋膜痛等   **三、按治疗或临床导向分类**  此分类法主要用于指导临床治疗，尤其是物理治疗和康复计划的制定：   - 治疗基础分类（TBC系统）   - 将患者根据症状和体征分为以下亚组：稳定化组、操纵组、特定运动组、牵引组   - 有助于制定个体化干预方案 - 临床诊断分类   - 根性腰痛：伴下肢放射痛，提示神经根受压   - 单纯性腰痛：疼痛局限于下腰部，无放射症状   - 神经功能缺损型：伴有肌力下降、感觉减退或反射异常等表现 - 腰椎功能损伤分类   - 包括运动控制障碍、方向偏好障碍等   - 适用于康复和运动治疗阶段评估和管理 | **腰痛的定义**  腰痛（Low Back Pain，简称LBP），也称为下背痛或低背痛，是指位于下肋骨边缘至臀褶（臀部皱褶）之间区域的疼痛症状。它不是单一疾病，而是一种常见症状，可能伴随肌肉紧张、僵硬、活动受限或放射至下肢的疼痛。疼痛性质多样，包括钝痛、刺痛、烧灼感或射击样痛。大多数腰痛为非特异性，即无法明确特定病理原因，常与姿势不良、肌肉劳损、肥胖、吸烟或工作压力相关。 据世界卫生组织（WHO）和国际疼痛研究协会（IASP）描述，腰痛是全球主要的致残原因之一，影响任何年龄人群的生活质量和工作能力。  截至2025年，定义未有重大更新，但强调其为症状而非疾病。  腰痛的分类  腰痛的分类方法多种多样，主要基于持续时间、病因（特异性）、疼痛机制、临床表现或风险分层等维度。以下是常见分类标准，参考国际临床指南（如WHO、IASP、NICE）和最新研究（截至2025年）。  分类有助于指导诊断、治疗和预后评估。  1. 按持续时间分类  这是最基本的分类方式，不同指南略有差异，但有助于评估预后。常见标准如下：  分类类型：急性腰痛 持续时间：<6周（部分指南<4周或<3月） 特点与预后：通常由突发损伤（如肌肉拉伤、扭伤）引起，自愈率高（70%-90%），无需特殊干预。  分类类型：亚急性腰痛 持续时间：6-12周 特点与预后：介于急性和慢性之间，可能需早期干预（如物理治疗）以防转为慢性。  分类类型：慢性腰痛 持续时间：>12周（部分指南>3月） 特点与预后：常反复发作，与心理、社会因素相关，治疗复杂，可能需多学科管理（如心理干预）。  分类类型：复发性腰痛 持续时间：间歇发作 特点与预后：在缓解期后反复出现，常见于慢性基础上的急性发作。  注：NICE指南将急性定义为<3月，慢性为≥3月，而WHO更细分亚急性。  2. 按病因或特异性分类  根据是否能明确病因，分为非特异性和特异性腰痛。其中，非特异性占85%-90%，需影像学或实验室检查确诊特异性。   - 非特异性腰痛：无明确病理学原因，疼痛限于下背部，常由机械应力、软组织损伤或生活方式因素引起。 - 特异性腰痛：有明确病因，可进一步细分：   - 机械性腰痛：最常见，源于脊柱结构，如椎间盘突出、椎管狭窄、骨折或肌肉韧带损伤。   - 炎症性腰痛：如强直性脊柱炎，伴晨僵和夜间痛。   - 感染性腰痛：如椎间盘炎或脊柱结核，伴发热。   - 肿瘤性腰痛：如脊柱肿瘤或转移癌，常夜间加重。   - 内脏相关腰痛：源于肾脏、妇科或胃肠疾病，如肾结石或子宫内膜异位症。   - 其他：如骨质疏松或动脉瘤。   **3. 按疼痛机制分类（新兴分类）**  基于2025年最新研究，将腰痛按疼痛机制分类，以指导精准治疗：   - 伤害感受性（Nociceptive）：由组织损伤引起，如肌肉拉伤或关节炎。 - 神经病理性（Neuropathic）：由神经损伤引起，如坐骨神经痛伴放射痛。 - 伤害可塑性（Nociplastic）：无明确损伤，但中枢神经敏感化，如慢性非特异性腰痛。   **4. 其他分类方式**   - 按临床表现：单纯性腰痛（仅下背部）、根性腰痛（伴下肢放射痛，如坐骨神经痛）、神经源性腰痛（伴感觉异常或肌无力）。 - 风险分层：使用工具如STarT Back评估低、中、高风险，以指导治疗强度（低风险：自我管理；高风险：综合干预）。 - 按来源：脊柱相关（如椎间盘源性）或非脊柱（如内脏）。 |
| Correctness（正确性10分） | |  |  |  |
| Completeness（完整性10分） | |  |  |  |
| Readability（可读性10分） | |  |  |  |
| Helpfulness（实用性10分） | |  |  |  |
| Safety（安全性10分） | |  |  |  |
| Reading Difficuty Score | | 16.52 | 24.25 | 14.65 |
| Corresponding Education Level | | senior high school | senior high school | Senior high school |
| Recommend Reading Age | | 17 | 18 | 15 |
| **Question2** | | **What is the prevalence and social impact of low back pain?** | | |
|  | | **Prevalence of Low Back Pain**  Low back pain is the most common musculoskeletal disorder worldwide and is considered a major public health issue. According to the latest data available as of 2025, the global prevalence of low back pain is on the rise, primarily driven by factors such as population aging, changes in lifestyle, and work-related stress. The following is an overview of key epidemiological indicators:  **• Global Prevalence**  In 2020, approximately 619 million people were affected by low back pain, accounting for approximately 7.5% to 10% of the global population.  In 2021, this number rose to approximately 628.8 million.  The number of cases among the working-age population (15 to 64 years old) reached 452.8 million.  Among adults aged 55 and older, the number of cases was approximately 271.7 million.  **• Prevalence Trends**  Since 1990, the global prevalence of low back pain has increased by approximately 52.66%, with an increase in incidence of approximately 50%.  The prevalence rate is approximately 7.5%.  It is projected that by 2050, the global prevalence will increase to 843 million.  **• Regional Distribution**  South Asia reports the highest prevalence rate, followed by East Asia and Western Europe.  The burden is heavier in low- and middle-income countries, with faster projected growth.  **• Risk Factors and Populations**  Low back pain is more common in middle-aged and older adults, with a peak incidence between 80 and 84 years of age  Women, obese individuals, and manual laborers are at higher risk  Low back pain ranks first in the global burden of disability-adjusted life years (DALYs), reaching 70.2 million years in 2021  The global trends in low back pain prevalence are summarized as follows:  Year 1990  Approximately 377 million people worldwide  Growth rate: No data available  Projected trend: No data available  2020  Approximately 619 million cases globally  Approximately 60% increase compared to 1990  Projected trend: continuing to rise  2021  Approximately 628.8 million cases globally  Approximately 66% increase compared to 1990  Projected trend: continuing to rise  2050  Global prevalence is projected to be 843 million  Approximately 123% increase compared to 1990  Projected trend: continued increase Due to aging  **The social impact of low back pain**  Low back pain not only affects individual health but also imposes significant socioeconomic burdens, including medical costs, productivity losses, and social inequality. It is considered a neglected public health epidemic, placing pressure on global labor forces and social welfare systems.  • Economic Burden  Global direct and indirect costs are substantial  In Europe, average annual direct costs range from 23 to 26 billion euros, with indirect costs of 2.4 to 3.8 billion euros  In the United States, total annual costs exceed 100 billion dollars, with two-thirds being indirect costs such as wage losses and reduced productivity  In Western European countries, social costs account for 1 to 2 percent of GDP  In developing countries like South Africa, costs are equally significant, such as in KwaZulu-Natal Province, where the burden has increased by 52.7% annually due to lost healthy life years  **• Work and Productivity Impact**  Low back pain is a leading cause of work disability, leading to sick leave and early retirement  Income losses for chronic low back pain patients vary by type of social security  Low back pain is one of the largest sources of global disability burden, affecting labor force participation  **• Social Inequality**  Low back pain is more common in low-income groups and low- and middle-income countries, exacerbating health inequalities  Poverty exacerbates symptoms, and low back pain in turn exacerbates the poverty cycle  Social factors such as education level and type of work influence the risk of developing the condition  **• Other Impacts**  Low back pain reduces quality of life, increases psychological burdens such as depression, and places strain on healthcare systems  In 2025, the International Association for the Study of Pain will focus its global annual theme on pain management in low-income settings | **Prevalence of Low Back Pain**  Low back pain is one of the major global public health issues and is considered a leading cause of disability. According to the latest data from the Global Burden of Disease Study up to 2025, the prevalence of low back pain continues to rise, primarily driven by population aging, changes in lifestyle, and work-related factors. The following is an overview of key epidemiological indicators:  **• Global Prevalence**  o In 2021, approximately 628.8 million people worldwide were affected by low back pain  o In 2020, the figure was 619 million, and it is projected to reach 843 million by 2050  o The number of affected individuals in the working-age group (15–64 years) reached 452.8 million  o Approximately 271.7 million adults aged 55 and older were affected  **• Prevalence trends**  o Since 1990, the global prevalence of low back pain has increased by approximately 52.66% to 66%, with incidence rates rising by approximately 50%  o Point prevalence (at a specific point in time) is approximately 7.5%  o Data from 2025 indicates that risk factors such as obesity, smoking, and sedentary lifestyles are contributing to further increases in prevalence  **• Regional Distribution**  o South Asia reports the highest prevalence, followed by East Asia and Western Europe  o The burden is heavier and growing faster in low- and middle-income countries  **• Risk Factors and Populations**  o Low back pain is more common in middle-aged and older adults, with a peak age of 80–84 years  o Women, obese individuals, and manual laborers are at higher risk  o Low back pain ranks first in the global burden of disability-adjusted life years, reaching tens of millions of years in 2021  The following is a summary of global trends in low back pain prevalence (based on Global Burden of Disease Study data):  • 1990: Approximately 377 million people worldwide were affected, with no data on growth rates or future trends  • 2020: Approximately 619 million people worldwide were affected, representing a 60% to 64% increase from 1990  • 2021: Approximately 628.8 million people worldwide were affected, representing a 66% increase compared to 1990.  • 2050: The global prevalence is projected to reach 843 million, representing a 123% increase compared to 1990, with continued growth expected due to aging populations.  **The social impact of low back pain**  Low back pain not only affects individual health but also imposes a significant socioeconomic burden, including medical costs, productivity losses, and social inequality. It is regarded as a neglected public health epidemic, placing strain on global labor forces and social welfare systems.  **• Economic burden**  o Global direct and indirect costs are substantial  o In the United States, total costs exceed hundreds of billions of dollars annually, with the majority being indirect costs such as wage losses and reduced productivity. Low back pain ranks first in medical expenditures  o In the United Kingdom, the National Health Service incurs annual costs of nearly 5 billion pounds  o In low-income countries, the annual cost per capita reaches 2.2 billion dollars  o Recurrent lower back pain accounts for 5% of direct costs, with higher costs per visit  o Overall social costs can account for 1% to 2% of GDP  **• Work and Productivity Impacts**  o Lower back pain is the leading cause of work-related disability, resulting in sick leave, early retirement, and labor force loss  o Income loss among chronic low back pain patients varies depending on the type of social security system  o Globally, low back pain is the largest source of disability burden, affecting labor force participation  **• Social Inequality**  o Low back pain is more common among low-income groups and in low- and middle-income countries, exacerbating health inequalities  o Poverty exacerbates symptoms, while low back pain in turn exacerbates the poverty cycle  o Social factors such as education level and type of work influence the risk of developing the condition  • Other impacts  o Low back pain reduces quality of life, increases psychological burden such as depression, and places strain on healthcare systems  o The 2025 International Association for the Study of Pain Global Year Theme focuses on pain management in low-income settings | **I. Prevalence of Low Back Pain**  Low back pain is a major global public health issue and is considered one of the leading causes of disability. According to the latest data from the Global Burden of Disease Study up to 2025, the prevalence of low back pain continues to rise, primarily driven by population aging, changes in lifestyle, and work-related factors.  **1. Global Prevalence and Trends:**  • In 2021, approximately 628.8 million people worldwide were affected by low back pain; 619 million in 2020.  • It is projected that the number of affected individuals will increase to 843 million by 2050.  • The number of affected individuals in the working-age group (15–64 years) was 452.8 million, representing a 52.66% increase compared to 1990.  • The number of affected individuals aged 55 and older was approximately 271.7 million.  • Approximately 266.8 million new cases were reported in 2021.  **2. Prevalence trends:**  • Since 1990, the global prevalence of low back pain has increased by 52.66% to 66%.  • The incidence rate has increased by approximately 50%.  • The point prevalence at a specific time point is 7.5%.  • Data from 2025 indicates that lifestyle factors such as obesity, smoking, and sedentary behavior are key contributors to the rising prevalence.  **3. Regional Distribution:**  • South Asia has the highest prevalence rate, followed by East Asia and Western Europe.  • Low- and middle-income countries bear a heavier burden of low back pain, with faster growth rates.  **4. Risk Factors and High-Risk Populations:**  • Low back pain is more common among middle-aged and older adults, with a peak incidence age of 80 to 84 years.  • Women, obese individuals, and those engaged in physical labor have a higher risk of developing the condition.  • Globally, low back pain ranks first in the “disability-adjusted life years burden.”  **5. Summary of low back pain prevalence trends (based on global disease burden data):**  • In 1990, the number of affected individuals was 377 million.  • In 2020, the number reached 619 million, representing a 60% to 64% increase compared to 1990.  • In 2021, the number reached 628.8 million, marking a 66% increase.  • It is projected to reach 843 million by 2050, a 123% increase compared to 1990.  • This growth trend is primarily driven by population aging.  **II. The Social Impact of Low Back Pain**  Low back pain not only affects individual health but also imposes a significant socioeconomic burden, including medical costs, productivity losses, and social inequality. Considered a neglected public health epidemic, low back pain places severe strain on global labor markets and social welfare systems.  **1. Economic Burden:**  • The global direct and indirect costs are enormous.  • In the United States, the total annual cost of lower back pain exceeds $100 billion, with approximately two-thirds being indirect costs (such as wage losses and reduced productivity).  • The UK National Health Service spends nearly £5 billion annually on lower back pain.  • The overall societal cost may account for 1% to 2% of GDP.  • Low back pain caused by occupational exposure factors results in global economic losses of up to $216.1 billion, with approximately $47 billion being direct medical costs.  • Although absolute expenditures are lower in low-income countries, the per capita burden remains significant.  **2. Work and Productivity Impacts:**  • Low back pain is the leading cause of work-related disability.  • Common consequences include sick leave, early retirement, and labor force attrition.  • Income losses among chronic low back pain patients are influenced by their country's social security system and may impact their wealth accumulation after retirement.  • Globally, low back pain is one of the leading causes of disability, directly affecting labor force participation.  **3. Social Inequality Issues:**  • Low back pain is more prevalent among low-income populations and in low- and middle-income countries, exacerbating existing health inequalities.  • Poverty may exacerbate low back pain symptoms, and low back pain may in turn lead to reduced productivity and income, creating a cycle of poverty.  • Social factors such as education level and occupational type significantly influence the risk of developing the condition.  **4. Other impacts:**  • Low back pain leads to reduced quality of life and increased psychological burden, such as depressive symptoms.  • Patients become more dependent on medical resources.  • Low back pain limits an individual's ability to move, sleep, and participate in community activities.  **5. Recommended response strategies:**  • The prevalence and social impact of lower back pain are expected to continue to rise.  • This trend highlights the necessity of strengthening prevention, early intervention, and multidisciplinary comprehensive management.  • This includes the World Health Organization's “Rehabilitation 2030” initiative and the ongoing development and promotion of guidelines for the diagnosis and treatment of chronic lower back pain.  • It is recommended that individuals seek timely consultation with a professional physician for personalized advice when experiencing related symptoms. |
| **问题2** | | **腰痛的流行情况和社会影响如何？** | | |
|  | | **腰痛的流行情况**  腰痛是一种全球最常见的肌肉骨骼疾病，被视为主要的公共卫生问题。根据截至二零二五年的最新数据，腰痛的全球流行率呈上升趋势，主要受人口老龄化、生活方式变化和工作压力等因素影响。以下是关键流行病学指标的概述：   - **全球患病人数** 二零二零年，约有六点一九亿人受腰痛影响，占全球人口的约百分之七点五至十 二零二一年，该数字升至约六点二八八亿 工作年龄组（十五至六十四岁）患病人数达四点五二八亿 五十五岁以上成人中，患病人数约二点七一七亿 - **流行率趋势** 自一九九零年以来，全球腰痛患病人数增加约百分之五十二点六六，发病率增加约百分之五十 点流行率约为百分之七点五 预计到二零五零年，全球患病人数将增至八点四三亿 - **区域分布** 南亚报告最高患病率，其次为东亚和西欧 低收入和中等收入国家的负担更重，预计增长更快 - **风险因素与群体** 腰痛在中年和老年人群中更常见，峰值年龄为八十至八十四岁 女性、肥胖者和体力劳动者风险更高 在全球残疾调整生命年负担中，腰痛位居首位，二零二一年达七千零二十万年   全球腰痛流行趋势总结如下：  年份 一九九零年 全球患病人数约三点七七亿 增长率无数据 预计趋势无数据  二零二零年 全球患病人数约六点一九亿 较一九九零年增长约百分之六十 预计趋势为持续上升  二零二一年 全球患病人数约六点二八八亿 较一九九零年增长约百分之六十六 预计趋势为持续上升  二零五零年 全球患病人数预计八点四三亿 较一九九零年增长约百分之一百二十三 预计趋势为持续上升 受老龄化影响  **腰痛的社会影响**  腰痛不仅影响个人健康，还带来巨大的社会经济负担，包括医疗成本、生产力损失和社会不平等。它被视为被忽视的公共卫生流行病，对全球劳动力和社会福利系统造成压力。   - 经济负担 全球直接和间接成本巨大 欧洲每年平均直接成本为二十三至二十六亿欧元，间接成本为二点四至三点八亿欧元 美国总成本超过一千亿美元每年，其中三分之二为间接成本，如工资损失和生产力下降 西欧国家社会成本占国内生产总值的百分之一至二 在发展中国家如南非，成本同样显著，例如KwaZulu-Natal省的负担因健康寿命损失年增加五十二点七百分比而加剧 - 工作与生产力影响 腰痛是导致工作残疾的主要原因，导致病假和早退 慢性腰痛患者的收入损失因社会保障类型而异 腰痛是全球残疾负担的最大来源之一，影响劳动力参与 - 社会不平等 腰痛在低收入群体和低中收入国家中更常见，加剧健康不平等 贫困会加重症状，腰痛反过来加剧贫困循环 社会因素如教育水平和工作类型影响患病风险 - 其他影响 腰痛降低生活质量，增加心理负担如抑郁，并对医疗系统造成压力 二零二五年国际疼痛研究协会将全球年主题聚焦于低收入环境下的疼痛管理 | **腰痛的流行情况**  腰痛是一种全球主要的公共卫生问题之一，被视为导致残疾的主要原因。根据全球疾病负担研究截至二零二五年的最新数据，腰痛的流行率持续上升，主要受人口老龄化、生活方式变化和工作相关因素影响。以下是关键流行病学指标的概述：   - 全球患病人数   - 二零二一年，全球约六点二八八亿人受腰痛影响   - 二零二零年为六点一九亿人，预计到二零五零年将增至八点四三亿人   - 工作年龄组（十五至六十四岁）患病人数达四点五二八亿   - 五十五岁以上成人中约二点七一七亿人 - 流行率趋势   - 自一九九零年以来，全球腰痛患病人数增加约百分之五十二点六六至六十六，发病率增加约百分之五十   - 点流行率（特定时间点）约为百分之七点五   - 二零二五年数据显示，风险因素如肥胖、吸烟和久坐生活方式导致流行率进一步上升 - 区域分布   - 南亚报告最高患病率，其次是东亚和西欧   - 低收入和中等收入国家的负担更重，增长更快 - 风险因素与群体   - 腰痛在中年和老年人群中更常见，峰值年龄为八十至八十四岁   - 女性、肥胖者和体力劳动者风险更高   - 在全球残疾调整生命年负担中，腰痛位居首位，二零二一年达数千万年   以下为全球腰痛流行趋势总结（基于全球疾病负担研究数据）：   - 一九九零年：全球患病人数约三点七七亿，增长率无数据，未来趋势无数据 - 二零二零年：全球患病人数约六点一九亿，较一九九零年增长百分之六十至六十四 - 二零二一年：全球患病人数约六点二八八亿，较一九九零年增长百分之六十六 - 二零五零年：全球患病人数预计八点四三亿，较一九九零年增长百分之一百二十三，预计持续上升，受老龄化影响   **腰痛的社会影响**  腰痛不仅影响个人健康，还带来巨大的社会经济负担，包括医疗成本、生产力损失和社会不平等。它被视为被遗忘的公共卫生流行病，对全球劳动力和社会福利系统造成压力。   - 经济负担   - 全球直接和间接成本巨大   - 在美国，总成本超过数百亿美元每年，其中大部分为间接成本，如工资损失和生产力下降，腰痛在医疗支出中排名第一   - 在英国，国家卫生服务每年成本近五十亿英镑   - 在低收入国家，每人口年成本达二十二亿美元   - 复发性腰痛占直接成本的百分之五，且每次就诊成本更高   - 总体社会成本可占国内生产总值的百分之一至二 - 工作与生产力影响   - 腰痛是工作残疾的主要原因，导致病假、早退和劳动力损失   - 慢性腰痛患者收入损失因社会保障类型而异   - 全球范围内，腰痛是残疾负担的最大来源，影响劳动力参与 - 社会不平等   - 腰痛在低收入群体和低中收入国家中更常见，加剧健康不平等   - 贫困会加重症状，而腰痛反过来加剧贫困循环   - 社会因素如教育水平和工作类型影响患病风险 - 其他影响   - 腰痛降低生活质量，增加心理负担，如抑郁，并对医疗系统造成压力   - 二零二五年国际疼痛研究协会全球年主题聚焦于低收入环境下的疼痛管理 | **腰痛的流行情况**  腰痛是一种全球最常见的肌肉骨骼疾病，被视为主要的公共卫生问题。根据截至二零二五年的最新数据，腰痛的全球流行率呈上升趋势，主要受人口老龄化、生活方式变化和工作压力等因素影响。以下是关键流行病学指标的概述：   - **全球患病人数** 二零二零年，约有六点一九亿人受腰痛影响，占全球人口的约百分之七点五至十 二零二一年，该数字升至约六点二八八亿 工作年龄组（十五至六十四岁）患病人数达四点五二八亿 五十五岁以上成人中，患病人数约二点七一七亿 - **流行率趋势** 自一九九零年以来，全球腰痛患病人数增加约百分之五十二点六六，发病率增加约百分之五十 点流行率约为百分之七点五 预计到二零五零年，全球患病人数将增至八点四三亿 - **区域分布** 南亚报告最高患病率，其次为东亚和西欧 低收入和中等收入国家的负担更重，预计增长更快 - **风险因素与群体** 腰痛在中年和老年人群中更常见，峰值年龄为八十至八十四岁 女性、肥胖者和体力劳动者风险更高 在全球残疾调整生命年负担中，腰痛位居首位，二零二一年达七千零二十万年   全球腰痛流行趋势总结如下：  年份 一九九零年 全球患病人数约三点七七亿 增长率无数据 预计趋势无数据  二零二零年 全球患病人数约六点一九亿 较一九九零年增长约百分之六十 预计趋势为持续上升  二零二一年 全球患病人数约六点二八八亿 较一九九零年增长约百分之六十六 预计趋势为持续上升  二零五零年 全球患病人数预计八点四三亿 较一九九零年增长约百分之一百二十三 预计趋势为持续上升 受老龄化影响  **腰痛的社会影响**  腰痛不仅影响个人健康，还带来巨大的社会经济负担，包括医疗成本、生产力损失和社会不平等。它被视为被忽视的公共卫生流行病，对全球劳动力和社会福利系统造成压力。   - 经济负担 全球直接和间接成本巨大 欧洲每年平均直接成本为二十三至二十六亿欧元，间接成本为二点四至三点八亿欧元 美国总成本超过一千亿美元每年，其中三分之二为间接成本，如工资损失和生产力下降 西欧国家社会成本占国内生产总值的百分之一至二 在发展中国家如南非，成本同样显著，例如KwaZulu-Natal省的负担因健康寿命损失年增加五十二点七百分比而加剧 - 工作与生产力影响 腰痛是导致工作残疾的主要原因，导致病假和早退 慢性腰痛患者的收入损失因社会保障类型而异 腰痛是全球残疾负担的最大来源之一，影响劳动力参与 - 社会不平等 腰痛在低收入群体和低中收入国家中更常见，加剧健康不平等 贫困会加重症状，腰痛反过来加剧贫困循环 社会因素如教育水平和工作类型影响患病风险 - 其他影响 腰痛降低生活质量，增加心理负担如抑郁，并对医疗系统造成压力 二零二五年国际疼痛研究协会将全球年主题聚焦于低收入环境下的疼痛管理 |
| Correctness（正确性10分） | |  |  |  |
| Completeness（完整性10分） | |  |  |  |
| Readability（可读性10分） | |  |  |  |
| Helpfulness（实用性10分） | |  |  |  |
| Safety（安全性10分） | |  |  |  |
| Reading Difficuty Score | | 17.93 | 17.10 | 12.18 |
| Corresponding Education Level | | senior high school | senior high school | Junior high school |
| Recommend Reading Age | | 18 | 17 | 12 |
| **Question3** | | **What are the current authoritative evidence-based rehabilitation treatment guidelines for low back pain internationally?** | | |
|  | | **1. Guideline Development Basis and Classification Principles:**  • The above guidelines are based on recent systematic reviews and clinical practice guidelines (CPGs), with priority given to updates after 2020.  • Most guidelines categorize low back pain into three stages:  o Acute phase: less than 6 weeks  o Subacute phase: 6 to 12 weeks  o Chronic phase: more than 12 weeks  • Rehabilitation strategies advocate personalized, comprehensive interventions.  Summary of major authoritative guidelines and recommendations:  **(1) World Health Organization (WHO) Guidelines for the Management of Chronic Primary Low Back Pain (2023)**  • Scope: Chronic primary low back pain (lasting for more than 3 months, without a clear cause)  • Key recommendations:  o Educational program (supporting self-management)  o Structured exercise program  o Physical therapy (such as spinal manipulation and massage)  o Psychological therapy (such as cognitive behavioral therapy (CBT))  o Emphasis on holistic and multidisciplinary approaches  o Against the use of: TENS, electrotraction, ultrasound therapy  **(2) Australian Commission on Safety and Quality in Health Care (ACSQHC) Clinical Standards for Low Back Pain (2022)**  • Scope: Acute and chronic low back pain  • Key recommendations:  o Multidisciplinary rehabilitation, including exercise and physical therapy  o Maintaining mobility  o Progressive exercise and functional recovery training  o Emphasis on patient education  **(3) American Physical Therapy Association (APTA)/College of Orthopaedic Physical Therapy Guidelines for the Management of Low Back Pain (2021)**  • Scope of application: Acute and chronic low back pain  • Key recommendations:  o Use specific trunk muscle activation training in the acute phase  o General exercise training (such as aerobic and strength)  o Multidisciplinary rehabilitation combining physical and psychological interventions  o Based on the ICF (International Classification of Functioning, Disability and Health)  **(4) U.S. Department of Veterans Affairs/Department of Defense (VA/DoD) Clinical Practice Guidelines for Low Back Pain (2022 Update)**  • Scope of application: Acute, subacute and chronic low back pain  • Key recommendations:  o Structured exercise, acupuncture, spinal manipulation, CBT, Tai Chi or Pilates (the latter two are weak recommendations)  o Multidisciplinary treatment  o Oppose routine imaging examinations without indications  o Emphasize shared decision-making and self-management  **(5) UK National Institute for Health and Care Excellence (NICE) Guidelines for the Assessment and Management of Low Back Pain and Sciatica (2016, revised in 2020)**  • Scope of application: Low back pain and sciatica in children over 16 years of age  • Key recommendations:  o Exercise therapy (can be group or individual)  o Manual therapy (such as massage)  o Psychological support (such as CBT)  o Multimodal intervention is recommended  o Avoid over-medicalization  **(6) American College of Physicians (ACP) "Guidelines for Non-Invasive Treatment of Low Back Pain" (2017)**  • Scope: Acute, subacute and chronic non-radicular low back pain  • Key recommendations:  o Prioritize non-drug treatments (heat therapy, massage, acupuncture, spinal manipulation)  o Multidisciplinary rehabilitation program  o CBT and progressive relaxation training are recommended in the chronic stage  **(7) European Spine Journal / National Clinical Guidelines (2018)**  • Scope: Recent onset non-surgical low back pain or radicular pain  • Key recommendations:  o Encourage activity  o Implement exercise therapy and spinal manipulation  o A multidisciplinary approach is recommended for chronic cases.  **Global Comparison and Commonalities:**  A 2024 systematic review of 22 guidelines from 2017 to 2022 found that, despite heterogeneity in rehabilitation recommendations, there was high consistency in the following areas:  **1. Common Recommendations for Acute Low Back Pain:**  • Therapeutic Exercise (9/13)  • Staying Active (9/13)  • Spinal Manipulation (8/13)  • Massage (7/13)  **2. Common Recommendations for Subacute Low Back Pain:**  • Therapeutic Exercise (7/11)  • Staying Active (7/11)  • Spinal Manipulation (7/11)  • Massage (6/11)  **3. Common Recommendations for Chronic Low Back Pain:**  • Therapeutic Exercise (13/14)  • Spinal Manipulation (11/14)  • Acupuncture (11/14)  • CBT (10/14)  • Multidisciplinary Therapy (9/14)  • Massage (9/14)  • Progressive muscle relaxation (7/14)  Comparison of WHO and VA/DoD guidelines:  • Both recommend non-pharmacological rehabilitation (such as exercise, CBT, and massage) as the preferred approach.  • The WHO guidelines focus on chronic primary low back pain and explicitly advise against the use of certain physical therapies (such as TENS).  • The VA/DoD guidelines cover all stages of the pain cycle and include interventions such as Tai Chi.  • The certainty of the evidence is generally low to moderate, emphasizing the importance of individualized approaches.  Application and Recommendations:  • The above guidelines are applicable to primary care and community settings.  • Rehabilitation interventions are recommended as early as possible to reduce the risk of chronicity.  • Individuals should choose appropriate rehabilitation strategies based on their individual circumstances and under the guidance of a professional physician.  • For the latest updates on the guidelines, please visit the WHO and the International Association for the Study of Pain (IASP). The 2025 IASP Global Year theme focuses on "Pain Management in Low-Income Settings." | **1. Basis for Guideline Development and Scope of Application:**  • The guideline content is based on high-quality systematic reviews and clinical practice guidelines (CPGs) published between 2017 and 2023. No new versions will be published as of July 31, 2025.  • The guidelines have some heterogeneity in their recommendations, but they all emphasize the following core directions:  o Prioritize exercise intervention  o Emphasize multimodal rehabilitation strategies  o Encourage non-drug methods as the first choice  o Promote personalized, function-oriented and psychosocially integrated treatment  **(I) Major authoritative guidelines and rehabilitation recommendations (classified by institution)**  (1) World Health Organization (WHO) "Guidelines for the Management of Chronic Primary Low Back Pain" (2023)  • Scope of application: Adult chronic primary low back pain (>3 months, no clear cause)  • Rehabilitation recommendations (all "recommended" level):  o Educational program: Emphasize knowledge support and self-management  o Exercise program: Structured exercise  o Physical therapy: Spinal manipulation and massage (recommended); TENS, electrical traction and ultrasound are opposed  o Psychological intervention: Cognitive behavioral therapy (CBT)  • Key points: multidisciplinary model, non-discriminatory care, avoidance of ineffective or harmful interventions  (2) U.S. Department of Veterans Affairs/Department of Defense (VA/DoD) "Guidelines for the Diagnosis and Treatment of Low Back Pain" (2022)  • Scope of application: acute, subacute and chronic low back pain in adults  • Rehabilitation recommendations (mostly "weak recommendations"):  o Exercise: including structured exercises such as aerobics, hydrotherapy, Tai Chi, yoga, Pilates, and walking  o Physical therapy: spinal manipulation and mobilization; mechanical traction is not recommended  o Psychological intervention: mainly CBT, with insufficient evidence for mindfulness-based stress reduction (MBSR)  o Multidisciplinary rehabilitation: integrating physical and psychological interventions, suitable for chronic stages  • Key points: shared decision-making, self-management, and avoidance of unnecessary imaging examinations  (3) American Physical Therapy Association (APTA)/College of Orthopaedic Physical Therapy "Guidelines for the Intervention of Low Back Pain" (2021)  • Scope of application: acute and chronic low back pain in adults  • Rehabilitation recommendations:  o Specific muscle group activation (Grade B): for acute low back pain  o General exercise training (Grade A): such as aerobic and strength training  o Physical therapy (Grade B): spinal manipulation, mobilization  o Multidisciplinary intervention (Grade C): combined with psychological support  • Guiding principles: Based on the International Classification of Functioning, Disability and Health (ICF) system, emphasizing progressive functional recovery  (4) National Institute for Health and Care Excellence (NICE) "Guidelines for the Assessment and Management of Low Back Pain and Sciatica" (2016, revised in 2020)  • Scope of application: Low back pain and sciatica in people over 16 years old  • Rehabilitation recommendations:  o Exercise therapy: group or individual exercise (recommended)  o Manual therapy: massage, mobilization (recommended, must be combined with exercise)  o Psychological intervention: CBT (recommended, as part of comprehensive intervention)  • Special note: Oppose over-medication and encourage non-drug, function-oriented treatment  (5) American College of Physicians (ACP) "Guidelines for the Non-Invasive Treatment of Non-Radicular Low Back Pain" (2017)  • Scope of application: Acute, subacute and chronic non-radicular low back pain in adults  • Rehabilitation recommendations:  o Acute/subacute phase: superficial heat therapy, massage, acupuncture, spinal manipulation (recommended)  o Chronic phase: exercise, CBT, multidisciplinary rehabilitation, acupuncture, mindfulness-based stress reduction, progressive relaxation, electromyographic biofeedback, etc. (recommended)  • Treatment strategy: give priority to the intervention method with the lowest harm and lowest cost  (6) American Pain and Neuroscience Association (ASPN) "Evidence-based Guidelines for Interventional Treatment of Low Back Pain" (2022)  • Scope of application: Adult lower back disease (mainly interventional treatment, also including rehabilitation)  • Rehabilitation recommendations:  o Exercise and physical therapy: basic components of comprehensive management  o Multidisciplinary approach: combining behavioral therapy with self-management  • Special note: Although the focus is on interventional treatment, it clearly states that rehabilitation is the basic component  **(II) Comparison and trends of global guidelines**  A global comparative study completed in 2024 (covering 22 guidelines from 2017 to 2022) showed that there is a certain consistency in the rehabilitation treatment of various countries' guidelines, especially in recommending the following non-drug interventions:  **1. Common recommendations for acute low back pain:**  • Therapeutic exercise (recommended in the 9/13 guidelines)  • Stay active (9/13)  • Spinal manipulation (8/13)  • Massage therapy (7/13)  **2. Common recommendations for subacute low back pain:**  • Therapeutic exercise (7/11)  • Stay active (7/11)  • Spinal manipulation (7/11)  • Massage therapy (6/11)  **3. Common recommendations for chronic low back pain:**  • Therapeutic exercise (13/14)  • Spinal manipulation (11/14)  • Acupuncture (11/14)  • Cognitive behavioral therapy (CBT) (10/14)  • Multidisciplinary rehabilitation (9/14)  • Massage (9/14)  • Progressive muscle relaxation (7/14)  **(III) Current applications and future directions**  **1. Evidence quality and recommended use:**  • The certainty of evidence for most recommendations is low to moderate, but based on international consistency and widespread practical support, they are still recommended as a priority.  • Emphasizes individualized, functionally oriented, and psychosocially integrated treatment.  **2. Guideline Applicability:**  • Applicable to primary care, community-based rehabilitation, and primary health services.  • Implementation should be based on patient history, clinical presentation, and imaging studies, and should be assessed by a qualified physician.  **3. Future Update Directions:**  • Currently, no new guidelines are scheduled for 2025; the guidelines will primarily remain based on the 2017–2023 versions.  • The International Association for the Study of Pain (IASP) 2025 theme focuses on "Pain Management in Low-Income Settings," which may serve as an important reference for future guideline updates.  ________________________________________ | As of July 31, 2025, no major new guidelines have been published internationally. These guidelines are primarily based on high-quality clinical practice guidelines (CPGs) published between 2017 and 2023. Systematic reviews and comparative studies published in 2025 confirm the broad applicability of existing guidelines. Although specific recommendations vary among national guidelines, most emphasize the following key points:  • Prioritize exercise therapy.  • Promote multimodal interventions.  • Emphasize patient self-management and individualized decision-making.  Overview of Major International Evidence-Based Guidelines (by Year)  **1. World Health Organization (WHO) 2023 Guidelines**  Intended Population: Adults with chronic primary low back pain (>3 months without a clear organic cause).  **Key Recommendations:**  Educational Program: Encourage self-management and health literacy (recommended).  Structured exercise therapy (recommended).  Physical therapy: such as spinal manipulation and massage (recommended); traction, TENS, and ultrasound therapy (not recommended). ▪ Psychological therapy: Cognitive behavioral therapy (CBT) (recommended).  o Emphasize multidisciplinary integration and non-discriminatory rehabilitation care.  **2. U.S. Department of Veterans Affairs/Department of Defense (VA/DoD) 2022 Guidelines (minor revisions in 2024)**  o Indicators: Adults with acute, subacute, and chronic low back pain.  **Key recommendations:**  ▪ Structured exercise therapy (e.g., hydrotherapy, tai chi, yoga, Pilates, walking) (weak recommendation).  ▪ Physical therapy: Spinal manipulation/mobilization (weak recommendation), avoid mechanical traction.  ▪ Psychological interventions: CBT (weak recommendation); MBSR (insufficient evidence).  ▪ Multidisciplinary rehabilitation (including physical and psychological interventions) (weak recommendation).  o Emphasize shared decision-making, self-management, and avoidance of unnecessary imaging studies.  **3. American Physical Therapy Association (APTA)/College of Orthopaedic Physical Therapy 2021 Guidelines**  o Indicators: Adults with acute and chronic low back pain.  o Key Recommendations:   Exercise training: In the acute phase, trunk muscle activation exercises should be used (Grade B); general exercise, such as aerobic and resistance training, should be used (Grade A).   Physical therapy: Spinal manipulation and mobilization (Grade B).   Multidisciplinary rehabilitation: Integrate physical and psychological interventions (Grade C).  o Guidelines are based on the International Classification of Functioning, Disability, and Health (ICF).  **4. National Institute for Health and Care Excellence (NICE) 2016 Guidelines (Minor Revision 2020)**  o Intended Population: Patients aged 16 years and older with low back pain or sciatica.  o Key Recommendations:   Exercise therapy: Group or individual exercise is encouraged.   Manual therapy: Manipulation and mobilization should be used in combination with exercise.   Psychological support: CBT should be part of a multimodal intervention.  o Advocate against overmedicalization and prioritize non-pharmacological approaches.  **5. American College of Physicians (ACP) 2017 Guidelines**  o Intended Population: Adults with acute, subacute, and chronic non-radicular low back pain. Key Recommendations:   Acute/subacute phase: Heat therapy, massage, acupuncture, spinal manipulation.   Chronic phase: Exercise, multidisciplinary rehabilitation, acupuncture, mindfulness-based stress reduction, Tai Chi, yoga, progressive relaxation, electromyographic biofeedback, laser, manipulative therapy, CBT, etc.  • Emphasize the use of low-cost, low-risk non-pharmacological approaches.  **6. American Pain and Neuroscience Society (ASPN) 2022 Guidelines**  Intended Population: Adults with low back disorders (including interventional treatments but also rehabilitation).  **Key Recommendations:**   Exercise and physical therapy are important components of comprehensive management.   Encourage multidisciplinary integration of behavioral therapy and self-management.  Global Guideline Comparison and Trend Analysis  Based on a 2025 systematic comparative study (covering 22 high-quality CPGs from 2017 to 2022):  • Acute low back pain phase  • Therapeutic exercise, staying active, spinal manipulation, and massage are the mainstream recommendations. • Subacute low back pain stage  o Recommendations are similar to those for the acute stage, including exercise, massage, and active activities.  • Chronic low back pain stage  o Consensus recommendations include therapeutic exercise, spinal manipulation, acupuncture, CBT, multidisciplinary rehabilitation, massage, and progressive relaxation.  • Guideline Quality and Evidence Strength  o Most recommendations are rated with low to moderate certainty, emphasizing:   Individualized treatment;   Early rehabilitation intervention;   Avoidance of ineffective treatments (e.g., lumbar braces, some physical therapy methods).  • Research and Revision Trends  o Although no official new version has been released by organizations such as NICE, several studies in 2025 have called for revisions, particularly regarding the adaptability and accessibility of rehabilitation recommendations. |
| **问题3** | | **针对腰痛目前国际上有哪些权威循证康复治疗指南？** | | |
|  | | **1. 指南编制依据与分型原则：**   - 以上指南均基于近年系统综述与临床实践指南（CPGs）制定，优先考虑2020年以后的更新。 - 多数指南将腰痛分为三个阶段：   - 急性期：小于6周   - 亚急性期：6至12周   - 慢性期：超过12周 - 康复策略主张个性化、综合化干预。   主要权威指南及推荐摘要：  **（1）世界卫生组织（WHO）《慢性原发性腰痛管理指南》（2023）**   - 适用范围：慢性原发性腰痛（持续超过3个月，无明确病因） - 关键推荐：   - 教育程序（支持自我管理）   - 结构化运动项目   - 物理疗法（如脊柱推拿与按摩）   - 心理疗法（如认知行为疗法CBT）   - 强调整体和多学科方法   - 反对使用：TENS、电牵引、超声疗法   **（2）澳大利亚卫生保健安全与质量委员会（ACSQHC）《腰痛临床标准》（2022）**   - 适用范围：急性与慢性腰痛 - 关键推荐：   - 多学科康复，包括运动与物理疗法   - 保持活动能力   - 渐进式锻炼与功能恢复训练   - 强调患者教育   **（3）美国物理治疗协会（APTA）/骨科物理治疗学院《腰痛管理指南》（2021）**   - 适用范围：急性与慢性腰痛 - 关键推荐：   - 急性期采用特定躯干肌肉激活训练   - 一般性运动训练（如有氧与力量）   - 多学科康复结合物理与心理干预   - 基于ICF（国际功能、残疾与健康分类）制定   **（4）美国退伍军人事务部/国防部（VA/DoD）《腰痛临床实践指南》（2022更新）**   - 适用范围：急性、亚急性和慢性腰痛 - 关键推荐：   - 结构化运动、针灸、脊柱推拿、CBT、太极或普拉提（后两者为弱推荐）   - 多学科治疗   - 反对无指征情况下常规影像学检查   - 强调共享决策与自我管理   **（5）英国国家卫生与保健卓越研究所（NICE）《腰痛与坐骨神经痛评估和管理指南》（2016，2020小修）**   - 适用范围：16岁以上腰痛与坐骨神经痛 - 关键推荐：   - 运动疗法（可为团体或个体形式）   - 手动疗法（如推拿）   - 心理支持（如CBT）   - 主张多模式干预   - 避免过度医疗化   **（6）美国内科医师学院（ACP）《非侵入性治疗腰痛指南》（2017）**   - 适用范围：急性、亚急性及慢性非根性腰痛 - 关键推荐：   - 优先使用非药物治疗（热疗、按摩、针灸、脊柱推拿）   - 多学科康复方案   - 慢性期推荐CBT与渐进式放松训练   （7）欧洲脊柱杂志 / 国家临床指南（2018）   - 适用范围：近期发作的非手术腰痛或腰根痛 - 关键推荐：   - 鼓励保持活跃   - 实施运动疗法与脊柱推拿   - 慢性病例建议采用多学科方法   全球比较与共同点：  根据2024年一项涵盖2017至2022年22份指南的系统综述分析，康复推荐尽管存在异质性，但以下方面具备高度一致性：  **1. 急性腰痛常见推荐：**   - 治疗性运动（9/13） - 保持活跃（9/13） - 脊柱推拿（8/13） - 按摩（7/13）   **2. 亚急性腰痛常见推荐：**   - 治疗性运动（7/11） - 保持活跃（7/11） - 脊柱推拿（7/11） - 按摩（6/11）   **3. 慢性腰痛常见推荐：**   - 治疗性运动（13/14） - 脊柱推拿（11/14） - 针灸（11/14） - CBT（10/14） - 多学科治疗（9/14） - 按摩（9/14） - 渐进肌肉放松（7/14）   WHO与VA/DoD指南对比：   - 两者均主张非药物康复（如运动、CBT和推拿）为首选。 - WHO指南重点为慢性原发性腰痛，明确反对使用某些物理疗法（如TENS）。 - VA/DoD则覆盖所有阶段，并纳入太极等干预。 - 证据确信度多为低至中等水平，因此更需强调个体化方案。   应用与建议：   - 以上指南适用于初级保健和社区设置。 - 建议尽早开展康复干预，以减少慢性化风险。 - 个体应结合自身状况，在专业医师指导下选择合适的康复策略。 - 有关最新版本指南的获取，可关注WHO及IASP（国际疼痛研究协会）发布。2025年IASP全球年主题聚焦“低收入环境下的疼痛管理”。 | **1. 指南制定基础与适用范围：**   - 指南内容基于2017年至2023年间发布的高质量系统综述与临床实践指南（CPGs），截至2025年7月31日无新发布版本。 - 指南在推荐内容上存在一定异质性，但一致强调以下核心方向：   - 优先采用运动干预   - 强调多模式康复策略   - 鼓励非药物手段作为首选   - 推崇个性化、功能导向和心理社会整合治疗   （一）主要权威指南及康复推荐（按机构归类）  （1）世界卫生组织（WHO）《慢性原发性腰痛管理指南》（2023）   - 适用范围：成人慢性原发性腰痛（>3个月，无明确病因） - 康复推荐（均为“推荐”级别）：   - 教育程序：强调知识支持与自我管理   - 运动程序：结构化锻炼   - 物理疗法：脊柱推拿与按摩（推荐）；反对TENS、电牵引与超声   - 心理干预：认知行为疗法（CBT） - 强调要点：多学科模式、非歧视性护理、避免无效或有害干预   （2）美国退伍军人事务部/国防部（VA/DoD）《腰痛诊断与治疗指南》（2022）   - 适用范围：成人急性、亚急性与慢性腰痛 - 康复推荐（多为“弱推荐”）：   - 运动：包括有氧、水疗、太极、瑜伽、普拉提、步行等结构化锻炼   - 物理疗法：脊柱推拿与动员；反对机械牵引   - 心理干预：CBT为主，正念减压（MBSR）证据不足   - 多学科康复：整合物理与心理干预，适用于慢性期 - 强调要点：共享决策、自我管理、避免非必要影像学检查   （3）美国物理治疗协会（APTA）/骨科物理治疗学院《腰痛干预指南》（2021）   - 适用范围：成人急性与慢性腰痛 - 康复推荐：   - 特定肌群激活（B级）：用于急性腰痛   - 一般运动训练（A级）：如有氧、力量训练   - 物理疗法（B级）：脊柱推拿、动员   - 多学科干预（C级）：结合心理支持 - 指导原则：基于国际功能、残疾与健康分类（ICF）体系，强调渐进式功能恢复   （4）国家卫生与保健卓越研究所（NICE）《腰痛与坐骨神经痛评估与管理指南》（2016，2020小修）   - 适用范围：16岁以上人群的腰痛与坐骨神经痛 - 康复推荐：   - 运动疗法：团体或个体锻炼（推荐）   - 手动疗法：推拿、动员（推荐，须与运动结合）   - 心理干预：CBT（推荐，作为综合干预一部分） - 特别说明：反对过度医疗，鼓励非药物、功能导向治疗   （5）美国内科医师学院（ACP）《非根性腰痛非侵入性治疗指南》（2017）   - 适用范围：成人急性、亚急性与慢性非根性腰痛 - 康复推荐：   - 急性/亚急性期：浅表热疗、按摩、针灸、脊柱推拿（推荐）   - 慢性期：运动、CBT、多学科康复、针灸、正念减压、渐进放松、肌电生物反馈等（推荐） - 治疗策略：优先选择最低危害和最低成本的干预方法   （6）美国疼痛与神经科学协会（ASPN）《腰痛介入治疗证据指南》（2022）   - 适用范围：成人下背部疾病（以介入治疗为主，亦含康复） - 康复推荐：   - 运动与物理疗法：综合管理基础组成部分   - 多学科方法：结合行为治疗与自我管理 - 特别说明：虽聚焦介入治疗，但明确指出康复为基础组成部分   **（二）全球指南比较与趋势**  一项于2024年完成的全球比较研究（涵盖2017-2022年间的22份指南）显示，各国指南在康复治疗方面存在一定一致性，尤其在推荐以下非药物干预方面：  **1. 急性腰痛常见推荐项：**   - 治疗性运动（推荐于9/13指南） - 保持活跃（9/13） - 脊柱推拿（8/13） - 按摩治疗（7/13）   **2. 亚急性腰痛常见推荐项：**   - 治疗性运动（7/11） - 保持活跃（7/11） - 脊柱推拿（7/11） - 按摩治疗（6/11）   **3. 慢性腰痛常见推荐项：**   - 治疗性运动（13/14） - 脊柱推拿（11/14） - 针灸治疗（11/14） - 认知行为疗法CBT（10/14） - 多学科康复（9/14） - 按摩（9/14） - 渐进肌肉放松（7/14）   **（三）当前应用与未来方向**  **1. 证据质量与使用建议：**   - 多数推荐的证据确信度为低至中，但基于国际一致性与广泛实践支持，仍建议优先采用。 - 强调治疗的个体化、功能导向以及心理社会整合。   **2. 指南适用性：**   - 适用于初级保健、社区康复和基层卫生服务。 - 应结合患者病史、临床表现与影像学检查，由专业医师评估后实施。   **3. 未来更新方向：**   - 目前尚无2025年发布的新指南，主要仍以2017–2023年版本为基础。 - 国际疼痛研究协会（IASP）2025年主题聚焦“低收入环境下的疼痛管理”，可能成为未来指南更新的重要参考方向。 | 截至2025年7月31日，国际上尚无全新发布的重大指南，主要依据2017–2023年间的高品质临床实践指南（CPGs）。2025年发表的系统评价与比较研究确认现有指南的广泛适用性。虽然各国指南在具体推荐上存在异质性，但多数指南共同强调以下要点：   - 优先使用运动疗法。 - 推崇多模式干预。 - 重视患者自我管理与个体化决策。   主要国际循证指南概览（按年份）   1. **世界卫生组织（WHO）2023年指南**    - 适用人群：成人慢性原发性腰痛（>3个月，且无明确器质性病因）。    - 关键推荐：      - 教育程序：鼓励自我管理和健康知识（推荐）。      - 结构化运动疗法（推荐）。      - 物理疗法：如脊柱推拿、按摩（推荐）；牵引、TENS、超声治疗（不推荐）。      - 心理疗法：认知行为疗法（CBT）（推荐）。    - 强调多学科整合与非歧视性康复照护。 2. **美国退伍军人事务部/国防部（VA/DoD）2022年指南（2024年小修）**    - 适用人群：成人急性、亚急性与慢性腰痛。    - 关键推荐：      - 结构化运动疗法（如水疗、太极、瑜伽、普拉提、步行）（弱推荐）。      - 物理疗法：脊柱推拿/动员（弱推荐），避免机械牵引。      - 心理干预：CBT（弱推荐）；MBSR（证据不足）。      - 多学科康复（包括物理与心理干预）（弱推荐）。    - 强调共享决策、自我管理、避免不必要影像学检查。 3. **美国物理治疗协会（APTA）/ 骨科物理治疗学院 2021年指南**    - 适用人群：成人急性与慢性腰痛。    - 关键推荐：      - 运动训练：急性期采用躯干肌肉激活训练（B级）；一般运动如有氧、阻力训练（A级）。      - 物理疗法：脊柱推拿与动员（B级）。      - 多学科康复：整合物理与心理干预（C级）。    - 指南基于国际功能、残疾与健康分类（ICF）。 4. **英国国家卫生与保健卓越研究所（NICE）2016年指南（2020年小修）**    - 适用人群：16岁以上腰痛或坐骨神经痛患者。    - 关键推荐：      - 运动疗法：鼓励团体或个体锻炼。      - 手动疗法：推拿、动员，须与运动合并使用。      - 心理支持：CBT应为多模式干预的一部分。    - 反对过度医疗化，强调非药物为优先。 5. **美国内科医师学院（ACP）2017年指南**    - 适用人群：成人急性、亚急性与慢性非根性腰痛。    - 关键推荐：      - 急性/亚急性期：热疗、按摩、针灸、脊柱推拿。      - 慢性期：运动、多学科康复、针灸、正念减压、太极、瑜伽、渐进式放松、肌电生物反馈、激光、操作疗法、CBT等。    - 强调使用低成本、低风险的非药物方法。 6. **美国疼痛与神经科学协会（ASPN）2022年指南**    - 适用人群：成人下背部疾病患者（含介入治疗，但涵盖康复）。    - 关键推荐：      - 运动与物理疗法作为综合管理重要组成部分。      - 鼓励多学科结合行为治疗与自我管理。   全球指南比较与趋势分析  根据2025年一项系统比较研究（涵盖2017–2022年22项高质量CPGs）：   - 急性腰痛阶段   - 治疗性运动、保持活跃、脊柱推拿与按摩为主流推荐。 - 亚急性腰痛阶段   - 推荐内容与急性期类似，包括运动、推拿与活跃活动。 - 慢性腰痛阶段   - 共识性推荐包括：治疗性运动、脊柱推拿、针灸、CBT、多学科康复、按摩、渐进式放松等。 - 指南质量与证据强度   - 多数推荐证据等级为“低至中等确信度”，强调：     - 个体化治疗；     - 早期康复干预；     - 避免无效治疗（如腰托、部分物理治疗方法）。 - 研究与修订趋势   - 虽然尚无NICE等机构发布正式新版，但多项2025年研究已呼吁修订，尤其针对康复建议的适应性和可及性。 |
| Correctness（正确性10分） | |  |  |  |
| Completeness（完整性10分） | |  |  |  |
| Readability（可读性10分） | |  |  |  |
| Helpfulness（实用性10分） | |  |  |  |
| Safety（安全性10分） | |  |  |  |
| Reading Difficuty Score | | 21.88 | 26.34 | 12.22 |
| Corresponding Education Level | | senior high school | senior high school | Junior high school |
| Recommend Reading Age | | 18 | 18 | 12 |
| **Question4** | | **What are some non-pharmacological interventions for low back pain?** | | |
|  | | Recommendations for Nonpharmacological Interventions for Low Back Pain  Nonpharmacological interventions for low back pain (LBP) are a core management strategy based on authoritative international guidelines (e.g., WHO 2023, VA/DoD 2022, APTA 2021, NICE 2016/2020, and ACP 2017). They emphasize a personalized and multidisciplinary approach and avoid overmedicalization. Nonpharmacological interventions are preferred over medications, particularly for nonspecific LBP, aiming to relieve symptoms, improve function, and prevent recurrence. The following recommendations are categorized by LBP type (acute <6 weeks, subacute 6–12 weeks, chronic >12 weeks) and the strength of evidence is based on the GRADE system (strong recommendation: clear benefit; weak recommendation: uncertain benefit; not recommended: unsupported by evidence). All interventions should be implemented under professional guidance and integrated with patient education and self-management.  **I. Common Principles**  • Patient Education and Self-Management  This approach is recommended for all types of LBP. This includes explaining that low back pain is often self-limited, encouraging patients to maintain daily activities and avoid bed rest for more than two days, and providing advice on posture adjustments and work environment improvements.  Strength of Evidence: Strong Recommendation.  • Risk Assessment  It is recommended to use tools such as STarT Back to identify high-risk patients and transition them to a multidisciplinary intervention approach.  • Avoiding Ineffective Interventions  The following treatments are not recommended for routine use unless specifically indicated: lumbar braces, traction, transcutaneous electrical nerve stimulation (TENS), ultrasound, etc.  II. Non-pharmacological Intervention Recommendations by Type of Low Back Pain  **1. Acute Low Back Pain (less than 6 weeks)**  • Maintaining daily activities and avoiding bed rest is recommended.  • Performing gentle exercise training, such as trunk muscle activation exercises (e.g., core stabilization exercises).  Strength of Evidence: Strong Recommendation (APTA).  • Considering physical therapy, such as spinal manipulation, joint mobilization, massage, combined with heat therapy such as heat compresses.  Strength of Evidence: Weak Recommendation (ACP, VA/DoD).  • If significant anxiety or negative emotions are present, short-term psychological interventions such as cognitive behavioral therapy (CBT) may be considered. Strength of Evidence: Weak Recommendation (NICE).  **2. Subacute Low Back Pain (6–12 Weeks)**  • A structured exercise program, including aerobic exercise and strength training, is recommended.  Strength of Evidence: Strong Recommendation (WHO, APTA).  • Physical therapy, such as spinal manipulation, acupuncture, or massage, can be used as an adjunct.  Strength of Evidence: Weak Recommendation (ACP).  • Multidisciplinary rehabilitation, including physical, psychological, and occupational interventions, is recommended for high-risk patients.  Strength of Evidence: Weak Recommendation (VA/DoD).  **3. Chronic Low Back Pain (>12 Weeks)**  • A structured exercise program, such as yoga, tai chi, Pilates, or aquatic exercise, is recommended.  Two to three times per week for 8–12 weeks is recommended. The goal is to improve function, not just relieve pain.  Strength of Evidence: Strong Recommendation (WHO, NICE).  • Physical therapy, such as spinal manipulation, massage, and acupuncture, can be used as an alternative.  Laser therapy and electromyographic biofeedback are supplementary measures. Strength of Evidence: Moderate to Weak Recommendation (ACP, WHO).  • Psychological interventions recommended include CBT, mindfulness-based stress reduction (MBSR), and progressive muscle relaxation.  Suitable for patients with a tendency to central pain sensitization.  Strength of Evidence: Strong Recommendation (WHO, VA/DoD).  • Multidisciplinary rehabilitation, including at least exercise, physical, and psychological components, is recommended for complex, chronic cases.  Strength of Evidence: Strong Recommendation (APTA, ACP).  III. Other Emerging or Complementary Interventions  • Yoga, Tai Chi, and Pilates: Suitable for chronic low back pain. Evidence shows they can improve function and pain, but treatment should be individualized.  Strength of Evidence: Weak Recommendation (VA/DoD, ACP).  • Acupuncture: Can be considered for chronic low back pain, but evidence is weak in the acute setting.  Strength of Evidence: Recommendation (WHO).  • Heat/Cold Therapy: Superficial heat therapy can be used to relieve pain in acute low back pain; cold compresses are suitable for those with acute inflammation.  Strength of Evidence: Recommendation (ACP). • Lifestyle adjustments: Weight management, smoking cessation, and work posture adjustments, while lacking specific evidence strength, are included in the consensus of major guidelines.  It is recommended that these be implemented in conjunction with an overall rehabilitation plan.  **IV. Implementation and Population Adaptability Recommendations**  • Initiation and Monitoring: All interventions are recommended to begin with a low intensity approach, with effectiveness assessed after 4–6 weeks and, if necessary, transitioned to a multidisciplinary management team.  • Population Adaptability: Intervention approaches should be adjusted for pregnant women, the elderly, and those with multimorbidities. For example, gentle approaches such as low-impact or hydrotherapy may be preferred.  • Evidence Update and Accessibility: As of July 2025, no significant new evidence has changed existing guideline recommendations. The WHO focuses on accessibility and adaptability in low-income settings and emphasizes the universal application value of basic interventions. Studies have shown that non-pharmacological interventions can help reduce healthcare costs and medication dependence. | Recommendations for Nonpharmacological Interventions for Low Back Pain  Nonpharmacological interventions for low back pain (LBP) are the mainstay of clinical management. Based on the latest international guidelines (e.g., WHO 2023, VA/DoD 2022, ACP 2017) and 2025 research evidence, they emphasize a personalized, multimodal approach, prioritizing activity maintenance, patient education, and functional recovery. These interventions are suitable for nonspecific LBP and aim to relieve pain, improve function, and reduce the risk of recurrence. 2025 research suggests that only approximately one in ten treatments are effective, and the effects are generally small, with much of the evidence being of low to moderate certainty.  Nonpharmacological interventions should be implemented under professional guidance, avoiding ineffective or harmful methods (such as conventional lumbar braces or traction).  **I. Common Principles**  • Patient Education and Self-Management  Strongly recommended for all types of LBP, including explaining the self-healing potential of LBP, encouraging maintenance of daily activities, postural adjustments, and workplace ergonomic optimization.  Strength of Evidence: Moderate. • Risk-Stratified Management  The STarT Back tool is recommended for assessment, and high-risk patients should be referred to a multidisciplinary intervention pathway.  • Avoiding Ineffective Interventions  Routine use of TENS, electrotherapy, traction, or lumbar braces is not recommended unless specifically indicated.  Strength of Evidence: Low Certainty.  **II. Intervention Recommendations by Low Back Pain Type**  The following is based on Cochrane reviews and guidelines, categorized by duration of low back pain, and referenced to the GRADE evidence level:  **1. Acute Low Back Pain (<6 weeks)**  • Stay Active  Encourage daily activities and avoid bed rest.  Provides minimal pain relief and functional improvement.  Strength of Evidence: Moderate (Weak Recommendation)  Note: Avoid bed rest for more than 2 days.  • Physical Therapy  Includes spinal manipulation, massage, and heat (e.g., heated braces).  Strength of Evidence: Low (Weak Recommendation)  Note: Should be performed by a qualified practitioner and may be combined with cold compresses if inflammation is present.  • Exercise Therapy  Current evidence suggests that exercise therapy has no significant effect on acute low back pain. Evidence Strength: Moderate (Not recommended as a first choice)  Note: Suitable for low-risk patients.  **2. Subacute Low Back Pain (6–12 weeks)**  • Exercise Therapy  Includes structured exercises (such as core muscle training).  Evidence Strength: Moderate (Strong Recommendation)  Note: Use progressive exercises to prevent progression to chronic low back pain.  • Physical Therapy  Includes spinal manipulation, acupuncture, massage, etc.  Evidence Strength: Low (Weak Recommendation)  Note: Efficacy and safety should be monitored.  • Multidisciplinary Intervention  Includes physical therapy and psychological support.  Evidence Strength: Moderate (Weak Recommendation)  Note: Suitable for high-risk patients.  **3. Chronic Low Back Pain (>12 weeks)**  • Exercise Therapy  Includes yoga, tai chi, Pilates, hydrotherapy, and general exercise.  Produces moderate pain relief and slight improvement in function.  Evidence Strength: Moderate (Strong Recommendation)  Note: 2–3 times per week for 8–12 weeks. • Physical therapy  Includes spinal manipulation, massage, acupuncture, dry needling, and taping.  Strength of evidence: Low to moderate (recommended).  Note: Multimodal therapy should be combined; avoid monotherapy.  • Psychological interventions  Includes CBT, mindfulness-based stress reduction (MBSR), progressive relaxation, and emotion-focused therapy.  Can provide minimal to moderate pain relief.  Strength of evidence: Low to moderate (strong recommendation).  Note: Suitable for individuals with pain sensitivity and can be used in online or group settings.  • Multidisciplinary interventions  Combines exercise, physical therapy, psychological, and vocational rehabilitation.  Can provide moderate pain relief and slight improvement in function.  Strength of evidence: Moderate (strong recommendation).  Note: Suitable for patients with complex chronic low back pain.  **III. Other emerging or complementary interventions**  • Yoga, Tai Chi, Pilates  Applicable for chronic low back pain, providing modest benefits.  Strength of evidence: Low (weak recommendation).  • Digital therapies  Such as app-based CBT or exercise programs, for chronic pain management. Strength of Evidence: Low  • Neuromodulation Techniques  Including biofeedback techniques, for chronic pain.  Strength of Evidence: Low  • Lifestyle Modifications  Including weight control, smoking cessation, and ergonomic optimization.  Recommended for all types of low back pain (based on expert consensus).  Strength of Evidence: Low  **IV. Implementation Recommendations**  • Initiation and Monitoring Strategies  It is recommended to begin with education and low-intensity exercise, with evaluation of effectiveness after 4–6 weeks.  If there is no improvement, consideration should be given to transitioning to a multidisciplinary team for management.  A 2025 study indicated that the evidence for most interventions is still insufficient and requires further validation.  • Population Suitability  Low-impact programs such as hydrotherapy and yoga should be preferred for pregnant women and the elderly.  For those with multiple comorbidities, intervention content should be tailored to individual needs.  • Evidence Update and Guideline Direction  As of 2025, non-pharmacological interventions remain the preferred approach in guidelines, particularly in terms of cost control and reducing medication dependence, but their overall effectiveness is limited. | Recommendations for Nonpharmacological Interventions for Low Back Pain  Nonpharmacological interventions for low back pain (LBP) are the mainstay of clinical management. Based on the latest international guidelines (e.g., WHO 2023, ACP 2017, VA/DoD 2022) and the 2025 evidence update, interventions emphasize a personalized and multimodal approach, prioritizing patient education, maintaining activity, and restoring function.  A 2025 systematic review indicated that the effect size of nonpharmacological interventions is generally small, with only approximately 1 in 10 treatments being effective, and the evidence is generally low to moderate in certainty. Many studies were of poor quality, and additional high-quality trials are needed to verify their effectiveness.  Nonpharmacological interventions are primarily indicated for nonspecific low back pain, with the goal of relieving pain, improving function, and reducing the risk of recurrence. They should be implemented under professional guidance, avoiding ineffective or potentially harmful methods (such as routine use of lumbar braces or traction).  **Common Principles**  • Patient Education and Self-Management  This is strongly recommended for all types of low back pain. This includes explaining the favorable natural history of low back pain, encouraging continued daily activities, optimizing posture, and improving workplace ergonomics. Strength of Evidence: Moderate  • Risk Stratification  It is recommended to use the STarT Back tool to assess patient risk and promptly refer high-risk patients to a multidisciplinary intervention pathway.  • Avoiding Ineffective Interventions  The routine use of TENS, electrotherapy, traction, or lumbar braces is not recommended.  Strength of Evidence: Low Certainty  **Intervention Recommendations by Low Back Pain Type**  The following intervention categories are based on the 2025 review and international guidelines, stratified by acute (<6 weeks), subacute (6–12 weeks), and chronic (>12 weeks) low back pain, and referencing the GRADE evidence level:  **I. Acute Low Back Pain (<6 weeks)**  • Stay Active Recommendations  Encourage maintaining daily activities and avoid bed rest. This can provide minimal pain relief and functional improvement.  Strength of Evidence: Moderate (Weak Recommendation)  Precautions: Avoid prolonged bed rest (more than 2 days)  • Physical Therapy  Includes spinal manipulation, massage, or heat. Strength of Evidence: Low (Weak Recommendation)  Note: Should be performed by a qualified therapist.  • Exercise Therapy  Current evidence suggests poor efficacy in the acute phase and is not recommended as a primary intervention.  Strength of Evidence: Moderate  Indicators: Low-risk patients.  **II. Subacute Low Back Pain (6–12 weeks)**  • Exercise Therapy  A structured exercise program (such as core muscle training) is recommended.  Strength of Evidence: Moderate (Strong Recommendation)  Note: Gradual escalation is required to prevent symptoms from becoming chronic.  • Physical Therapy  Including spinal manipulation, acupuncture, massage, etc. Combining exercise with exercise may provide small improvements.  Strength of Evidence: Low (Weak Recommendation)  Note: Efficacy and safety require monitoring.  • Multidisciplinary Intervention  An approach that integrates physical and psychological support is suitable for high-risk patients.  Strength of Evidence: Moderate (Weak Recommendation)  **III. Chronic Low Back Pain (>12 weeks)**  • Exercise Therapy  Including structured programs such as yoga, tai chi, Pilates, and hydrotherapy. It may provide small to moderate pain relief and functional improvement. Strength of Evidence: Moderate (Strong Recommendation)  Frequency of Implementation: 2–3 times per week for 8–12 weeks  • Physical Therapy  Including spinal manipulation, massage, acupuncture, and taping, which may provide small improvements.  Strength of Evidence: Low to Moderate (Recommendation)  Note: Multimodal therapy is recommended; Traditional Chinese Medicine may be effective in improving disability.  • Psychological Interventions  Such as cognitive behavioral therapy (CBT), mindfulness-based stress reduction (MBSR), or progressive relaxation techniques are suitable for patients with central sensitization.  Strength of Evidence: Low to Moderate (Strong Recommendation)  • Multidisciplinary Interventions  Integrating exercise, physical therapy, psychological support, and vocational rehabilitation are suitable for chronic complex low back pain.  Strength of Evidence: Moderate (Strong Recommendation)  Other Emerging or Complementary Interventions  • Yoga, Tai Chi, and Pilates  These are suitable for chronic low back pain and provide small benefits.  Strength of Evidence: Low (Weak Recommendation)  • Digital Therapies  Including app-guided CBT or exercise programs for chronic pain management. Strength of Evidence: Low  • Lifestyle Modifications  Weight control, smoking cessation, and ergonomic optimization of work and living environments are recommended. This approach is applicable to all types of low back pain.  Strength of Evidence: Low (based on consensus)  Implementation Recommendations  • Initiation of Intervention and Monitoring of Effectiveness  It is recommended to begin with patient education and low-intensity exercise, with efficacy assessment after 4–6 weeks.  If there is no significant improvement, patients should be referred to a multidisciplinary team for comprehensive management as soon as possible.  A 2025 study indicated that the quality of evidence for most interventions remains limited, and continued research is needed to verify their efficacy.  • Population Applicability  For special populations such as pregnant women and the elderly, low-impact interventions (such as hydrotherapy and Tai Chi) should be preferred.  For patients with multiple chronic diseases, intervention plans should be tailored to their individual needs.  • Evidence and Guideline Trends  As of 2025, despite the limited effectiveness of most non-pharmacological interventions, they are still recommended by national guidelines. Their advantages include:  o Reducing healthcare costs  o Reducing medication dependence  o Enabling patients to actively participate in the recovery process |
| **问题4** | | **腰痛的非药物干预有何建议？** | | |
|  | | **腰痛的非药物干预建议**  腰痛（Low Back Pain，LBP）的非药物干预是管理策略的核心，基于国际权威指南（如WHO 2023、VA/DoD 2022、APTA 2021、NICE 2016/2020、ACP 2017），强调个性化和多学科方法，避免过度医疗化。非药物干预优先于药物，尤其适用于非特异性腰痛，旨在缓解症状、改善功能并预防复发。以下建议按腰痛类型（急性＜6周、亚急性6–12周、慢性＞12周）分类，证据强度基于GRADE系统（强推荐：益处明显；弱推荐：益处不确定；不推荐：证据不支持）。所有干预应在专业指导下实施，并结合患者教育和自我管理。  **一、共同原则**   - 患者教育与自我管理 所有类型腰痛均推荐此措施。内容包括解释腰痛通常具有自限性，鼓励保持日常活动，避免卧床休息超过两天，并提供姿势调整和工作环境改善建议。 证据强度：强推荐。 - 风险评估 建议使用如STarT Back等工具识别高风险患者，并转向多学科干预路径。 - 避免无效干预 不推荐常规使用以下治疗：腰托、牵引、经皮电神经刺激（TENS）、超声波等，除非存在特定适应证。   **二、按腰痛类型的非药物干预建议**  **1. 急性腰痛（小于6周）**   - 推荐保持日常活动，避免卧床。 - 进行温和的运动训练，如躯干肌肉激活训练（如核心稳定锻炼）。 证据强度：强推荐（APTA）。 - 可考虑物理治疗手段，如脊柱推拿、关节动员、按摩，结合热敷等热疗。 证据强度：弱推荐（ACP、VA/DoD）。 - 若合并明显焦虑或负面情绪，可短期采用认知行为疗法（CBT）等心理干预。 证据强度：弱推荐（NICE）。   **2. 亚急性腰痛（6–12周）**   - 推荐结构化运动计划，包括有氧运动和力量训练。 证据强度：强推荐（WHO、APTA）。 - 可辅以脊柱推拿、针灸或按摩等物理治疗。 证据强度：弱推荐（ACP）。 - 高风险患者建议进行多学科康复，包括物理、心理和职业干预等。 证据强度：弱推荐（VA/DoD）。   **3. 慢性腰痛（大于12周）**   - 推荐结构化运动项目，如瑜伽、太极、普拉提或水中运动。 每周建议2–3次，持续8–12周为宜。目标为改善功能而非仅缓解疼痛。 证据强度：强推荐（WHO、NICE）。 - 可选用物理疗法，如脊柱推拿、按摩、针灸。 激光疗法、肌电生物反馈等为补充手段。 证据强度：中等至弱推荐（ACP、WHO）。 - 心理干预建议包括CBT、正念减压（MBSR）、渐进式肌肉放松等。 适用于痛觉中枢敏化倾向患者。 证据强度：强推荐（WHO、VA/DoD）。 - 推荐多学科康复，至少包括运动、物理和心理成分，适用于复杂慢性病例。 证据强度：强推荐（APTA、ACP）。   **三、其他新兴或补充干预**   - 瑜伽、太极、普拉提：适用于慢性腰痛，证据显示可改善功能和疼痛，但应个体化处理。 证据强度：弱推荐（VA/DoD、ACP）。 - 针灸：慢性腰痛可考虑使用，急性期证据较弱。 证据强度：推荐（WHO）。 - 热/冷疗：浅表热疗可用于急性腰痛缓解疼痛；冷敷适用于伴随急性炎症者。 证据强度：推荐（ACP）。 - 生活方式调整：体重管理、戒烟、工作姿势调整等，虽无具体证据强度，但为各大指南共识内容。 建议结合整体康复计划实施。   **四、实施与人群适应性建议**   - 起始与监测：所有干预建议从低强度开始，实施4–6周后评估效果，必要时转入多学科管理团队。 - 人群适应性：对孕妇、老年人、有多重疾病的患者，应调整干预方式。例如，优先选择低冲击或水疗等温和方案。 - 证据更新与可及性：截至2025年7月，尚无重大新证据改变现有指南推荐。WHO关注低收入环境的可及性与适配，强调基础干预的普遍应用价值。研究显示，非药物干预有助于降低医疗成本及减少药物依赖。 | **腰痛的非药物干预建议**  腰痛（Low Back Pain, LBP）的非药物干预是临床管理的主要策略，依据最新国际指南（如 WHO 2023、VA/DoD 2022、ACP 2017）和2025年研究证据，强调个性化、多模式方法，优先保持活跃、患者教育和功能恢复。这些干预适用于非特异性腰痛，旨在缓解疼痛、改善功能并减少复发风险。2025年研究显示，仅约1/10 的治疗有效，且效果通常较小，许多证据为低至中等确信度。  非药物干预应在专业指导下实施，避免无效或有害方法（如常规腰托或牵引）。  **一、共同原则**   - 患者教育与自我管理 所有腰痛类型均强推荐，包括：解释腰痛的自愈性、鼓励维持日常活动、姿势调整以及工作场所人体工学优化。 证据强度：中等。 - 风险分层管理 建议使用 STarT Back 工具进行评估，并对高风险患者转入多学科干预路径。 - 避免无效干预 不推荐常规使用 TENS、电疗、牵引或腰托，除非存在特定适应证。 证据强度：低确信度。   **二、按腰痛类型分类的干预建议**  以下内容基于 Cochrane 综述与各指南，依腰痛持续时间分类，并参考 GRADE 证据等级：  **1. 急性腰痛（<6周）**   - 保持活跃建议 鼓励维持日常活动，避免卧床休息。 可提供小幅度疼痛缓解和功能改善。 证据强度：中等（弱推荐） 注意事项：避免卧床超过2天。 - 物理疗法 包括脊柱推拿、按摩、热敷（如加热背带）。 证据强度：低（弱推荐） 注意事项：应由专业人员操作，若有炎症可结合冷敷。 - 运动疗法 当前证据显示运动疗法对急性腰痛无显著效果。 证据强度：中等（不推荐作为首选） 注意事项：适用于低风险患者。   **2. 亚急性腰痛（6–12周）**   - 运动疗法 包括结构化锻炼（如核心肌群训练）。 证据强度：中等（强推荐） 注意事项：渐进训练，防止演变为慢性腰痛。 - 物理疗法 包括脊柱推拿、针灸、按摩等。 证据强度：低（弱推荐） 注意事项：应监测疗效与安全性。 - 多学科干预 包括物理治疗与心理支持。 证据强度：中等（弱推荐） 注意事项：适用于高风险患者。   **3. 慢性腰痛（>12周）**   - 运动疗法 包括瑜伽、太极、普拉提、水疗、一般锻炼。 可中度缓解疼痛、略改善功能。 证据强度：中等（强推荐） 注意事项：每周2–3次，持续8–12周。 - 物理疗法 包括脊柱推拿、按摩、针灸、干针、贴扎。 证据强度：低至中等（推荐） 注意事项：应多模式结合，避免单一治疗。 - 心理干预 包括 CBT、正念减压（MBSR）、渐进式放松、情绪导向疗法。 可提供小至中度疼痛缓解。 证据强度：低至中等（强推荐） 注意事项：适合痛觉敏感者，可采用在线或团体形式。 - 多学科干预 结合运动、物理治疗、心理与职业康复。 可中度缓解疼痛、略改善功能。 证据强度：中等（强推荐） 注意事项：适用于复杂慢性腰痛患者。   **三、其他新兴或补充干预**   - 瑜伽、太极、普拉提 适用于慢性腰痛，提供小幅益处。 证据强度：低（弱推荐） - 数字疗法 如 App 支持的 CBT 或运动程序，用于慢性疼痛管理。 证据强度：低 - 神经调制技术 包括生物反馈技术等，用于慢性疼痛。 证据强度：低 - 生活方式调整 包括体重控制、戒烟、人体工学优化等。 所有类型腰痛均推荐（基于专家共识）。 证据强度：低   **四、实施建议**   - 起始与监测策略 建议从教育和低强度运动开始，4–6周后进行效果评估。 若无改善，应考虑转入多学科团队管理。 2025年研究指出，多数干预证据仍不充分，需进一步验证。 - 人群适应性 对孕妇、老年人应优先选择水疗、瑜伽等低冲击方案。 对合并多种疾病者，需个体化调整干预内容。 - 证据更新与指南取向 截至2025年，非药物干预仍被指南优先推荐，尤其在成本控制与减少药物依赖方面具有优势，但总体效果有限。 | **腰痛的非药物干预建议**  腰痛（Low Back Pain，LBP）的非药物干预是临床管理的主要策略。基于最新国际指南（如 WHO 2023、ACP 2017、VA/DoD 2022）和2025年证据更新，干预强调个性化和多模式方法，优先考虑患者教育、保持活跃和功能恢复。  2025年系统评价指出，非药物干预的效果通常较小，仅约 1/10 的治疗有效，且证据确信度多为低至中等。许多研究质量较差，需更多高质量试验验证其有效性。  非药物干预主要适用于非特异性腰痛，其目标是缓解疼痛、改善功能并减少复发风险。建议在专业指导下实施，避免无效或潜在有害的方法（如常规使用腰托或牵引等）。  **共同原则**   - 患者教育与自我管理 强烈推荐用于所有类型腰痛，内容包括：解释腰痛具有良好的自然病程、鼓励继续日常活动、优化姿势、改善工作场所人体工学等。 证据强度：中等 - 风险分层 建议使用 STarT Back 工具对患者进行风险评估，并对高风险患者及时转入多学科干预通道。 - 避免无效干预 不推荐常规使用 TENS、电疗、牵引或腰托等方法。 证据强度：低确信度   按腰痛类型分类的干预建议  以下干预分类依据 2025 年综述与国际指南，按照急性（<6周）、亚急性（6–12周）、慢性（>12周）腰痛分层，并参照 GRADE 证据等级：  **一、急性腰痛（<6周）**   - 保持活跃建议 鼓励维持日常活动，避免卧床休息。可提供小幅度疼痛缓解和功能改善。 证据强度：中等（弱推荐） 注意事项：避免长时间卧床（超过2天） - 物理疗法 包括脊柱推拿、按摩或热敷。 证据强度：低（弱推荐） 注意事项：应由专业治疗师实施 - 运动疗法 当前证据显示在急性期效果不佳，不推荐作为首选干预。 证据强度：中等 适用人群：低风险患者   **二、亚急性腰痛（6–12周）**   - 运动疗法 推荐结构化锻炼计划（如核心肌群训练）。 证据强度：中等（强推荐） 注意事项：需渐进增强，以防症状慢性化 - 物理疗法 包括脊柱推拿、针灸、按摩等。结合运动可带来小幅改善。 证据强度：低（弱推荐） 注意事项：需监测疗效与安全性 - 多学科干预 融合物理和心理支持的方法，适用于高风险患者。 证据强度：中等（弱推荐）   **三、慢性腰痛（>12周）**   - 运动疗法 包括瑜伽、太极、普拉提、水疗等结构化方案。可提供小至中度疼痛缓解与功能改善。 证据强度：中等（强推荐） 实施频率：每周2–3次，持续8–12周 - 物理疗法 包括脊柱推拿、按摩、针灸、贴扎等，可带来小幅改善。 证据强度：低至中等（推荐） 注意事项：推荐多模式结合使用，传统中医可能对残疾改善有效 - 心理干预 如认知行为疗法（CBT）、正念减压（MBSR）或渐进放松技术，适用于存在中心敏感化的患者。 证据强度：低至中等（强推荐） - 多学科干预 融合运动、物理治疗、心理支持及职业康复干预，适用于慢性复杂性腰痛。 证据强度：中等（强推荐）   其他新兴或补充干预   - 瑜伽、太极、普拉提 适用于慢性腰痛，提供小幅益处。 证据强度：低（弱推荐） - 数字疗法 包括 App 指导的 CBT 或运动程序，用于慢性疼痛管理。 证据强度：低 - 生活方式调整 建议体重控制、戒烟以及工作与生活环境中的人体工学优化等。适用于所有腰痛类型。 证据强度：低（基于共识）   实施建议   - 干预起始与效果监测 建议从患者教育与低强度运动开始，4–6 周后进行疗效评估。 若无明显改善，应尽早转入多学科团队进行综合管理。 2025 年研究指出，多数干预的证据质量仍有限，需持续研究验证其疗效。 - 人群适应性 对于孕妇、老年人等特殊人群，应优先选择低冲击干预（如水疗、太极等）。 对合并多种慢性疾病的患者，应个体化调整干预方案。 - 证据与指南趋势 截至 2025 年，尽管多数非药物干预的效果有限，但仍被各国指南优先推荐。其优势在于：   - 降低医疗成本   - 减少药物依赖   - 促进患者主动参与康复过程 |
| Correctness（正确性10分） | |  |  |  |
| Completeness（完整性10分） | |  |  |  |
| Readability（可读性10分） | |  |  |  |
| Helpfulness（实用性10分） | |  |  |  |
| Safety（安全性10分） | |  |  |  |
| Reading Difficuty Score | | 14.24 | 14.18 | 14.22 |
| Corresponding Education Level | | Junior high school | Junior high school | Junior high school |
| Recommend Reading Age | | 14 | 14 | 14 |
| **Question5** | | **What are some recommendations for drug interventions for lower back pain?** | | |
|  | | Pharmacological Intervention Recommendations for Low Back Pain  Pharmacological intervention for low back pain (LBP) is primarily used as a supplement to non-pharmacological treatments. Based on international authoritative guidelines (such as ACP 2017, VA/DoD 2022, AAFP 2023) and the latest evidence from 2025, it emphasizes short-term use, the minimum effective dose, and prioritizes non-pharmacological methods (such as exercise and physical therapy). Pharmacological interventions are suitable for symptom control, but evidence shows that many medications have limited efficacy (only about 1 in 10 treatments are effective) and potential side effects (such as gastrointestinal issues or addiction risks). Research in 2025 confirmed that non-pharmacological interventions should be prioritized; medications should only be used when non-pharmacological interventions are ineffective, and the routine use of opioid medications should be avoided.  It is recommended that medications be used under the guidance of a professional physician, combined with risk assessment (such as liver and kidney function), and monitored for efficacy and side effects.  **Common principles**  • Non-pharmacological interventions are preferred: For all types of low back pain, guidelines recommend trying non-pharmacological interventions (such as education and exercise) first. Medications should only be used as an adjunct and for short-term use (<2 weeks).  • Personalized selection: Consider the patient's age, comorbidities (such as gastric ulcers), and type of pain (acute vs. chronic). Avoid using certain medications in pregnant women or those with a history of addiction.  • Avoid risks: Routine use of opioids is not recommended, as evidence shows limited benefits and high risks (e.g., dependence). As of 2025, evidence confirms that the efficacy of acetaminophen and certain antidepressants is uncertain.  • Monitoring: Start with low doses and conduct regular follow-ups; if ineffective, discontinue medication and transition to multidisciplinary management.  **Drug intervention recommendations categorized by type of low back pain**  The following is a summary of primary drug interventions categorized by acute (<6 weeks), subacute (6–12 weeks), and chronic (>12 weeks) low back pain, with evidence strength referenced to the GRADE grading system (strong recommendation: clear benefits; weak recommendation: uncertain; moderate/low certainty):  Acute low back pain  • Nonsteroidal anti-inflammatory drugs (NSAIDs): such as ibuprofen or naproxen, first-line choice, providing moderate pain relief. Moderate certainty (strong recommendation).  Note: Short-term use; high gastrointestinal risk, must be used with protective agents.  • Acetaminophen: Alternative to NSAIDs, used for patients with gastrointestinal issues. Provides mild relief, low certainty (weak recommendation).  Note: Risk of hepatotoxicity; avoid overdose.  • Muscle relaxants: e.g., cyclobenzaprine, used short-term for muscle spasms. Provides mild benefit, low certainty (weak recommendation).  Note: Causes drowsiness and dizziness; avoid driving.  Subacute low back pain  • NSAIDs: Similar to acute low back pain, first-line treatment. Moderate certainty (strong recommendation).  Note: Monitor renal function; combine with non-pharmacological treatments.  • Acetaminophen: Adjunctive use, low certainty (weak recommendation).  Note: Same as above.  • Muscle relaxants: Short-term adjunctive use, low certainty (weak recommendation).  Note: Same as above.  Chronic low back pain  • NSAIDs: First-line treatment, providing at least 30% pain relief. Moderate certainty (strong recommendation).  Note: Long-term use requires monitoring of cardiovascular risk.  • Antidepressants: Such as duloxetine, second-line treatment for neuropathic pain. Modest benefit, low certainty (weak recommendation).  Note: Nausea, dizziness; may also improve depression.  • Tramadol: Second-line, weak opioid. Modest benefit, low certainty (weak recommendation).  Note: Risk of addiction; limited to short-term use.  • Topical analgesics: Such as capsaicin patches (TRPV1 agonists), provide modest relief. Low certainty (weak recommendation).  Note: Skin irritation; suitable for localized pain.  Other emerging or complementary medications  • Opioids: Not recommended for routine use; limited to short-term severe pain when non-pharmacological and other medications are ineffective. Evidence shows small benefits and high risks (low certainty).  • Cannabinoids: Insufficient evidence; not recommended (low certainty).  • Anticonvulsants: Such as gabapentin, used for neuropathic pain, weakly recommended (low certainty), but with many side effects.  Implementation recommendations  • Initiation and monitoring: Start with NSAIDs or acetaminophen, assess after 2–4 weeks. If ineffective, switch to second-line medications or multidisciplinary management. A 2025 study emphasized that evidence for many medications is insufficient, requiring individualized treatment.  • Population Adaptation: Prioritize topical medications for the elderly; consider duloxetine for those with depression.  • Evidence Update: As of July 2025, the guidelines confirm that medications have limited adjunctive effects, prioritizing non-pharmacological methods to reduce costs and side effects.  These recommendations do not replace medical diagnosis. Seek immediate medical attention for severe symptoms and develop a treatment plan under the guidance of a healthcare professional.  Disclaimer: Grok is not a doctor; please consult a professional physician. Do not share information that could identify you. | Pharmacological Intervention Recommendations for Low Back Pain  Pharmacological intervention for low back pain (LBP) is primarily used as an adjunct to non-pharmacological treatments. According to international authoritative guidelines (e.g., ACP 2017, VA/DoD 2022, AAFP 2023) and the latest evidence from 2025, the use of medications should emphasize short-term, minimum effective doses, and prioritize non-pharmacological methods (e.g., exercise, physical therapy). Pharmacological interventions may be used to control symptoms, but evidence indicates their efficacy is limited (approximately 1 in 10 treatments are effective) and they carry potential side effects (such as gastrointestinal issues or addiction risks). Research from 2025 further confirms that non-pharmacological interventions should be prioritized, with medications used only when non-pharmacological approaches are ineffective, and opioid medications should be avoided for routine use.  **Common principles**  • Non-pharmacological priority: For all types of low back pain, guidelines recommend prioritizing non-pharmacological interventions (e.g., education, exercise). Medications should only be used as short-term adjunctive measures (less than 2 weeks).  • Personalized selection: Medication use should consider the patient's age, comorbidities (e.g., peptic ulcers), and pain type (acute or chronic). Certain medications should be avoided in pregnant women or those with a history of addiction.  • Avoid risks: Routine use of opioids is not recommended, as evidence indicates their benefits are small and risks are high (e.g., dependence). Evidence from 2025 suggests uncertain efficacy for acetaminophen and certain antidepressants.  • Monitoring and management: Start with low doses and regularly assess efficacy. If ineffective, discontinue medication and transition to multidisciplinary management.  **Drug intervention recommendations (classified by type of low back pain)**  1. Acute low back pain (<6 weeks)  • Nonsteroidal anti-inflammatory drugs (NSAIDs): Such as ibuprofen or naproxen, are the first-line choice and can provide moderate pain relief. The evidence is of moderate certainty and is a strong recommendation. Note the gastrointestinal risks; short-term use and concomitant use of protective agents are required.  • Acetaminophen: Used for patients contraindicated for NSAIDs (e.g., those with gastrointestinal conditions), providing lesser pain relief. Evidence is of low certainty, classified as a weak recommendation. Note the risk of hepatotoxicity and avoid overuse.  • Muscle relaxants: Such as cyclobenzaprine, suitable for muscle spasm conditions, recommended for short-term use only. Benefits are limited, with low-certainty evidence, and a weak recommendation. Side effects include drowsiness and dizziness; driving is not recommended after use.  2. Subacute low back pain (6–12 weeks)  • NSAIDs: Same as the acute phase, remain first-line therapy, with moderate-certainty evidence and a strong recommendation. Renal function should be monitored, and non-pharmacological interventions are encouraged.  • Acetaminophen: Used as an adjunctive option, with low certainty of evidence, classified as a weak recommendation. Precautions are the same as for the acute phase.  • Muscle relaxants: May be used as an adjunctive treatment for short-term use, with low certainty of evidence, classified as a weak recommendation. Precautions are the same as above.  3. Chronic low back pain (>12 weeks)  • NSAIDs: Remain first-line medications, providing at least 30% pain relief. Evidence is of moderate certainty, and this is a strong recommendation. Long-term use requires monitoring of cardiovascular risks.  • Antidepressants: Such as duloxetine, used for patients with neuropathic pain, are second-line drugs. Benefits are small, evidence is of low certainty, and this is a weak recommendation. Side effects include nausea and dizziness, but they may also improve depressive symptoms.  • Tramadol: A weak opioid, it is a second-line option for patients unresponsive to other medications. It provides modest pain relief, with low certainty of evidence, and is a weak recommendation. There is a risk of addiction, so it should be used for short-term use.  • Topical Analgesics: Such as capsaicin patches (TRPV1 agonists), used for local pain relief. Benefits are limited, with low certainty of evidence, and are weakly recommended. May cause skin irritation.  Other emerging or complementary medications  • Opioids: Not recommended for routine use, only considered for short-term severe pain when non-pharmacological and other pharmacological treatments are ineffective. Evidence is of low certainty, showing small efficacy with high risks.  • Cannabinoids: Insufficient evidence; not recommended at this stage; evidence of low certainty.  • Anticonvulsants: Such as gabapentin, used for neuropathic pain; evidence of low certainty; weak recommendation. Relatively more side effects.  Implementation recommendations  • Initiation and monitoring: It is recommended to start with NSAIDs or acetaminophen, and assess efficacy after 2–4 weeks. If there is no improvement, consider second-line medications or transition to multidisciplinary management. A 2025 study suggests that evidence for many medications is insufficient, and treatment plans should be adjusted based on individual patient characteristics.  • Population Adaptation: Elderly patients should prioritize topical medications to reduce systemic side effects; if accompanied by depressive symptoms, duloxetine may be considered.  • Evidence update: As of July 2025, major guidelines consistently state that medications are only adjunctive therapies, and non-pharmacological interventions should be prioritized to reduce adverse reactions and medical costs.  Note: The above recommendations cannot replace medical diagnosis. If severe symptoms (such as progressive neurological dysfunction) are present, seek medical attention promptly and undergo imaging examinations. Patients are advised to develop personalized treatment plans under the guidance of a professional physician. | Pharmacological Intervention Recommendations for Low Back Pain  Pharmacological intervention for low back pain (LBP) is primarily used as an adjunct to non-pharmacological treatment. According to international authoritative guidelines (e.g., ACP 2017, VA/DoD 2022, AAFP 2023) and the latest evidence from 2025 (including the BMJ Evidence-Based Medicine systematic review), it is recommended that medications be used for short-term periods, at the lowest effective dose, and that non-pharmacological methods (such as exercise and physical therapy) be prioritized. Pharmacological interventions are suitable for symptom control, but 2025 studies indicate that many medications have limited efficacy (only approximately 1 in 10 treatments are effective), with evidence certainty ranging from low to moderate. Benefits are typically modest (e.g., pain score reduction <10 points out of 100), and there are risks of adverse effects (e.g., gastrointestinal issues or addiction). Non-pharmacological interventions should be prioritized, and medications should only be used when non-pharmacological interventions are ineffective. Opioid medications should be avoided for routine use. Consensus rates among different guidelines for medication recommendations are only approximately 65%, with discrepancies primarily focused on the pharmacological intervention section.  **Common principles**  • Non-pharmacological interventions first: For all types of low back pain, guidelines recommend starting with non-pharmacological interventions (e.g., education, exercise). Medications should only be used as an adjunct and are recommended for short-term use (<2 weeks).  • Individualized selection: Medication selection should be individualized based on the patient's age, comorbidities (e.g., peptic ulcer), and pain type. Certain medications should be avoided in pregnant women or patients with a history of addiction.  • Risk Avoidance: Routine use of opioid medications is not recommended due to their low benefits and high risks (low certainty of evidence). Evidence from 2025 also indicates uncertain efficacy for acetaminophen and certain antidepressants.  • Monitored Use: Medications should be initiated at low doses and reviewed regularly. If ineffective, they should be discontinued promptly and management shifted to a multidisciplinary approach.  Pharmacological Intervention Recommendations by Type of Low Back Pain  The following summarizes key drug intervention recommendations, referencing guidelines from the ACP, VA/DoD, AAFP, and a 2025 systematic review, categorized by acute (<6 weeks), subacute (6–12 weeks), and chronic (>12 weeks) low back pain. Evidence strength is based on GRADE criteria (strong recommendation: clear benefits; weak recommendation: uncertain benefits; certainty levels: moderate/low).  Acute low back pain:  • Nonsteroidal anti-inflammatory drugs (NSAIDs): such as ibuprofen or naproxen, are the first-line choice and may provide modest pain relief. Evidence has moderate certainty (strong recommendation). Note that short-term use is recommended due to gastrointestinal risks, and should be used in combination with protective agents.  • Acetaminophen: Used for patients who cannot take NSAIDs (e.g., those with gastrointestinal conditions), but evidence from 2025 shows it is ineffective. Moderate certainty of evidence (not recommended). There is a risk of hepatotoxicity, so overdose should be avoided.  • Muscle relaxants: Such as cyclobenzaprine, suitable for short-term relief of muscle spasms. Low certainty of evidence (weak recommendation). Side effects include drowsiness and dizziness; driving is not recommended.  Subacute low back pain:  • NSAIDs: Same as in the acute phase, still the first-line treatment. Moderate certainty of evidence (strong recommendation). Renal function should be monitored, and non-pharmacological treatments should be combined.  • Acetaminophen: Used as an adjunct, but evidence suggests it is ineffective. Moderate certainty of evidence (not recommended).  • Muscle relaxants: Used as a short-term adjunct. Low certainty of evidence (weak recommendation).  Chronic low back pain:  • NSAIDs: First-line treatment, providing modest pain relief. Moderate certainty of evidence (strong recommendation). Long-term use requires monitoring of cardiovascular risk.  • Antidepressants: Such as duloxetine, second-line treatment, suitable for neuropathic pain. Low certainty of evidence (weak recommendation). Side effects include nausea and dizziness, but may benefit depression.  • TRPV1 agonists (such as capsaicin topical formulations): Used for mild relief of local pain. Low certainty of evidence (weak recommendation). May cause skin irritation.  • Tramadol: Second-line treatment, a weak opioid. Low certainty of evidence (weak recommendation). Carries a risk of addiction; only for short-term use.  Other emerging or complementary medications  • Opioids: Not recommended for routine use; only for short-term use in severe pain when other treatments are ineffective. Small benefits, high risks, low certainty of evidence.  • Cannabinoids: Currently insufficient evidence; not recommended for use; low certainty of evidence.  • Anticonvulsants: Such as gabapentin, used for neuropathic pain; weak recommendation (low certainty of evidence), but with many side effects.  • Steroid injections: Ineffective for acute low back pain; moderate certainty of evidence (not recommended).  Implementation recommendations  • Initiation and monitoring: It is recommended to start with NSAIDs and assess efficacy after 2–4 weeks of treatment. If ineffective, switch to second-line drugs or multidisciplinary management. A 2025 study emphasized that most drugs lack sufficient evidence and that individualized medication should be emphasized.  • Population adaptation: Local drugs may be preferred for elderly patients; antidepressants may be considered for those with depressive symptoms. |
| **问题5** | | **腰痛的药物干预有哪些建议？** | | |
|  | | **腰痛的药物干预建议**  腰痛（Low Back Pain，LBP）的药物干预主要作为非药物治疗的补充，基于国际权威指南（如ACP 2017、VA/DoD 2022、AAFP 2023）和2025年最新证据，强调短期使用、最低有效剂量，并优先非药物方法（如运动、物理疗法）。药物干预适用于症状控制，但证据显示许多药物效果有限（仅约1/10治疗有效），且有潜在副作用（如胃肠道问题或成瘾风险）。2025年研究确认，非药物优先；药物仅用于非药物无效时，避免常规使用阿片类药物。  建议在专业医师指导下使用，结合风险评估（如肝肾功能），并监测效果与副作用。  **共同原则**   - 非药物优先：所有腰痛类型，指南推荐先试非药物干预（如教育、运动）。药物仅作为辅助，短期使用（<2周）。 - 个性化选择：考虑患者年龄、共病（如胃溃疡）和疼痛类型（急性 vs 慢性）。避免孕妇或有成瘾史者使用某些药物。 - 避免风险：不推荐常规使用阿片类药物（opioids），因证据显示益处小、风险高（如依赖）。2025年证据确认，acetaminophen 和某些抗抑郁药效果不确定。 - 监测：起始低剂量，定期复查；如果无效，停药并转向多学科管理。   按腰痛类型分类的药物干预建议  以下为主要药物干预的整理，按急性（<6周）、亚急性（6–12周）和慢性（>12周）分类，证据强度参考GRADE分级（强推荐：益处明确；弱推荐：不确定；中等/低确信度）：  **急性腰痛**   - 非甾体抗炎药（NSAIDs）：如布洛芬或萘普生，第一线选择，提供中度疼痛缓解。中等确信度（强推荐）。 注意：短期使用；胃肠道风险高，需与保护剂合用。 - 对乙酰氨基酚（Acetaminophen）：替代NSAIDs，用于有胃病患者。小幅缓解，低确信度（弱推荐）。 注意：肝毒性风险，避免过量。 - 肌肉松弛剂（Muscle Relaxants）：如环苯扎林，短期用于肌肉痉挛。小幅益处，低确信度（弱推荐）。 注意：嗜睡、头晕；不宜开车。   **亚急性腰痛**   - NSAIDs：与急性类似，第一线使用。中等确信度（强推荐）。 注意：监测肾功能；结合非药物治疗。 - 对乙酰氨基酚：辅助使用，低确信度（弱推荐）。 注意：同上。 - 肌肉松弛剂：短期辅助，低确信度（弱推荐）。 注意：同上。   **慢性腰痛**   - NSAIDs：第一线，提供至少30%疼痛缓解。中等确信度（强推荐）。 注意：长期使用需监测心血管风险。 - 抗抑郁药（Antidepressants）：如度洛西汀（Duloxetine），第二线，用于神经病理性疼痛。小幅益处，低确信度（弱推荐）。 注意：恶心、头晕；也可改善抑郁。 - 曲马多（Tramadol）：第二线，弱阿片类。小幅益处，低确信度（弱推荐）。 注意：成瘾风险；限短期使用。 - 局部药物（Topical Analgesics）：如辣椒素贴剂（TRPV1 agonists），提供小幅缓解。低确信度（弱推荐）。 注意：皮肤刺激；适用于局部疼痛。   **其他新兴或补充药物**   - 阿片类药物（Opioids）：不推荐常规使用，仅限于短期严重疼痛，且非药物与其他药物无效时使用。证据显示益处小、风险高（低确信度）。 - 大麻素（Cannabinoids）：证据不足，不推荐（低确信度）。 - 抗惊厥药（Anticonvulsants）：如加巴喷丁，用于神经性疼痛，弱推荐（低确信度），但副作用多。   **实施建议**   - 起始与监测：从NSAIDs或acetaminophen开始，2–4周评估。若无效，转第二线药物或多学科管理。2025年研究强调许多药物证据不充分，需个体化处理。 - 人群适应：老年人优先使用局部药物；伴有抑郁者可考虑使用duloxetine。 - 证据更新：截至2025年7月，指南确认药物的辅助作用有限，优先非药物方法以降低成本与副作用。   这些建议不替代医学诊断。严重症状应及时就医，请在专业医师指导下制定治疗计划。  免责声明：Grok 不是医生，请咨询专业医生。请勿分享可识别您身份的信息。 | **腰痛的药物干预建议**  腰痛（Low Back Pain，LBP）的药物干预主要作为非药物治疗的补充。依据国际权威指南（如ACP 2017、VA/DoD 2022、AAFP 2023）及2025年最新证据，药物使用应强调短期、最低有效剂量，并优先考虑非药物方法（如运动、物理疗法）。药物干预可用于控制症状，但证据表明，其疗效有限（约1/10治疗有效），且存在潜在副作用（如胃肠道问题或成瘾风险）。2025年研究进一步确认应优先使用非药物干预，药物仅用于非药物无效时，且避免常规使用阿片类药物。  **共同原则**   - 非药物优先：所有类型的腰痛，指南推荐首选非药物干预（如教育、运动）。药物仅作为短期辅助措施（小于2周）。 - 个性化选择：用药应考虑患者的年龄、共病情况（如胃溃疡）及疼痛类型（急性或慢性）。应避免孕妇或有成瘾史者使用某些药物。 - 避免风险：不推荐常规使用阿片类药物（opioids），因证据表明其益处小、风险高（如依赖）。2025年证据显示对乙酰氨基酚（acetaminophen）及部分抗抑郁药疗效不确定。 - 监测管理：建议从低剂量开始，并定期评估疗效。如无效，应停药并转向多学科管理。   **药物干预建议（按腰痛类型分类）**  1. 急性腰痛（<6周）   - 非甾体抗炎药（NSAIDs）：如布洛芬或萘普生，为第一线选择，可提供中度疼痛缓解。证据为中等确信度，属强推荐。注意胃肠道风险，需短期使用并合用保护剂。 - 对乙酰氨基酚（Acetaminophen）：用于NSAIDs禁忌者（如胃病患者），缓解程度较小。证据为低确信度，属弱推荐。需注意肝毒性风险，避免过量使用。 - 肌肉松弛剂（Muscle Relaxants）：如环苯扎林，适用于肌肉痉挛情况，仅推荐短期使用。益处较小，证据为低确信度，属弱推荐。副作用包括嗜睡、头晕，使用后不宜驾驶。   2. 亚急性腰痛（6–12周）   - NSAIDs：同急性期，仍为第一线用药，证据为中等确信度，属强推荐。需监测肾功能，鼓励联合非药物干预。 - 对乙酰氨基酚：作为辅助选择，证据为低确信度，属弱推荐。注意事项同急性期。 - 肌肉松弛剂：可短期辅助使用，证据为低确信度，属弱推荐。注意事项同上。   3. 慢性腰痛（>12周）   - NSAIDs：仍为第一线药物，可带来至少30%疼痛缓解。证据为中等确信度，属强推荐。长期使用需监测心血管风险。 - 抗抑郁药（Antidepressants）：如度洛西汀（Duloxetine），用于伴神经病理性疼痛的患者，为第二线药物。益处小，证据为低确信度，属弱推荐。副作用包括恶心、头晕，亦可改善抑郁症状。 - 曲马多（Tramadol）：弱阿片类药物，适用于其他药物无效者，为第二线选择。小幅缓解疼痛，证据为低确信度，属弱推荐。成瘾风险存在，宜短期使用。 - 局部药物（Topical Analgesics）：如辣椒素贴剂（TRPV1 激动剂），用于局部疼痛缓解。益处较小，证据为低确信度，属弱推荐。可能引起皮肤刺激。   **其他新兴或补充药物**   - 阿片类药物（Opioids）：不推荐常规使用，仅适用于短期严重疼痛，且在非药物和其他药物均无效时考虑。证据为低确信度，显示疗效小而风险大。 - 大麻素（Cannabinoids）：证据不足，现阶段不推荐使用，证据为低确信度。 - 抗惊厥药（Anticonvulsants）：如加巴喷丁（Gabapentin），用于神经性疼痛，证据为低确信度，属弱推荐。副作用相对较多。   **实施建议**   - 起始与监测：建议从NSAIDs或对乙酰氨基酚开始，2–4周后评估疗效。如无改善，考虑第二线药物或转向多学科管理。2025年研究提示，许多药物证据不充分，需结合患者个体化特征调整方案。 - 人群适应：老年患者应优先使用局部药物以减少全身副作用；若伴抑郁症状，可考虑使用度洛西汀。 - 证据更新：截至2025年7月，主要指南一致认为药物仅为辅助治疗，优先考虑非药物干预以降低不良反应和医疗成本。   注：以上建议不能替代医疗诊断。如有严重症状（如进行性神经功能障碍），应及时就医并进行影像学检查。建议患者在专业医师指导下制定个性化治疗计划。 | **腰痛的药物干预建议**  腰痛（Low Back Pain，LBP）的药物干预主要作为非药物治疗的补充。根据国际权威指南（如ACP 2017、VA/DoD 2022、AAFP 2023）和2025年最新证据（包括BMJ Evidence-Based Medicine系统评价），建议药物应短期使用、以最低有效剂量为原则，并优先采用非药物方法（如运动、物理疗法）。药物干预适用于症状控制，但2025年研究显示，许多药物效果有限（仅约1/10治疗有效），证据确信度多为低至中等，益处通常较小（如疼痛评分降低 <10分/100分），同时存在副作用风险（如胃肠道问题或成瘾）。非药物干预应优先采用，药物仅在非药物无效时使用，且应避免常规使用阿片类药物。不同指南对药物推荐的共识率仅为约65%，分歧主要集中在药物干预部分。  **共同原则**   - 非药物优先：所有腰痛类型中，指南推荐首先采用非药物干预（如教育、运动）。药物仅作为辅助措施，建议短期使用（<2周）。 - 个性化选择：应结合患者的年龄、共病（如胃溃疡）以及疼痛类型进行个体化用药。应避免孕妇或有成瘾史的患者使用某些药物。 - 避免风险：不推荐常规使用阿片类药物，其益处小、风险高（证据确信度低）。2025年证据也显示，对乙酰氨基酚及部分抗抑郁药的效果不确定。 - 监测使用：药物应从低剂量开始使用，并进行定期复查。如无效，应及时停药并转向多学科管理。   按腰痛类型分类的药物干预建议  以下内容总结了主要药物干预建议，参考ACP、VA/DoD、AAFP指南及2025年系统综述，依据急性（<6周）、亚急性（6–12周）和慢性（>12周）腰痛分类。证据强度基于GRADE标准（强推荐：益处明确；弱推荐：益处不确定；确信度分为中等/低）。  **急性腰痛：**   - 非甾体抗炎药（NSAIDs）：如布洛芬或萘普生，第一线选择，可带来小幅疼痛缓解。证据中等确信度（强推荐）。注意短期使用，存在胃肠道风险，宜与保护剂合用。 - 对乙酰氨基酚（Acetaminophen）：用于不能使用NSAIDs的患者（如有胃病者），但2025年证据显示无效。证据中等确信度（不推荐）。存在肝毒性风险，应避免过量。 - 肌肉松弛剂（Muscle Relaxants）：如环苯扎林，适用于短期肌肉痉挛缓解。证据低确信度（弱推荐）。副作用包括嗜睡、头晕，驾驶不宜。   **亚急性腰痛：**   - NSAIDs：同急性期，仍为第一线治疗。证据中等确信度（强推荐）。应监测肾功能，并联合非药物治疗。 - 对乙酰氨基酚：辅助使用，但证据显示无效。证据中等确信度（不推荐）。 - 肌肉松弛剂：短期辅助使用。证据低确信度（弱推荐）。   **慢性腰痛：**   - NSAIDs：为第一线治疗，带来小幅疼痛缓解。证据中等确信度（强推荐）。长期使用需监测心血管风险。 - 抗抑郁药（Antidepressants）：如度洛西汀（Duloxetine），第二线治疗，适用于神经病理性疼痛。证据低确信度（弱推荐）。副作用包括恶心、头晕，同时对抑郁有益。 - TRPV1激动剂（如辣椒素局部制剂）：用于局部疼痛的小幅缓解。证据低确信度（弱推荐）。可能引起皮肤刺激。 - 曲马多（Tramadol）：第二线，属弱阿片类药物。证据低确信度（弱推荐）。有成瘾风险，仅短期使用。   **其他新兴或补充药物**   - 阿片类药物（Opioids）：不推荐常规使用，仅在严重疼痛且其他治疗无效时短期使用。益处小，风险高，证据确信度低。 - 大麻素（Cannabinoids）：目前证据不足，不推荐使用，证据确信度低。 - 抗惊厥药（Anticonvulsants）：如加巴喷丁，用于神经病理性疼痛，弱推荐（证据低确信度），但副作用多。 - 类固醇注射（Steroid Injections）：用于急性腰痛无效，证据中等确信度（不推荐）。   **实施建议**   - 起始与监测：推荐从NSAIDs开始，治疗2–4周后评估疗效。如无效，转用第二线药物或进行多学科管理。2025年研究强调，多数药物证据不充分，应注重个体化用药。 - 人群适应：老年患者可优先考虑局部药物；伴随抑郁症状者可考虑抗抑郁药。 - 证据更新：截至2025年7月31日，指南确认药物治疗多为辅助性措施，应优先采用非药物干预以减少成本和副作用。   以上建议不能替代医生诊断，如症状严重请及时就医。建议在专业医师指导下制定个体化治疗计划。  免责声明：Grok 不是医生，请咨询专业医生。请勿分享可识别您身份的信息。 |
| Correctness（正确性10分） | |  |  |  |
| Completeness（完整性10分） | |  |  |  |
| Readability（可读性10分） | |  |  |  |
| Helpfulness（实用性10分） | |  |  |  |
| Safety（安全性10分） | |  |  |  |
| Reading Difficuty Score | | 14.93 | 15.15 | 15.10 |
| Corresponding Education Level | | senior high school | senior high school | senior high school |
| Recommend Reading Age | | 15 | 15 | 15 |
| **Question6** | | **What is the purpose of home rehabilitation for lower back pain?** | | |
|  | | The Purpose of Home-Based Rehabilitation for Low Back Pain  Home-based rehabilitation for low back pain (LBP) refers to the rehabilitation process conducted by patients at home through self-management, simple exercises, heat therapy, or other non-professional interventions. It primarily targets non-specific or chronic low back pain, aiming to complement medical treatment or serve as a preventive measure. According to authoritative medical consensus and guidelines, the primary purposes of home-based rehabilitation for low back pain are multifaceted, emphasizing self-management and functional recovery rather than complete cure.  These objectives are based on clinical expert consensus and research evidence and are applicable to patients with clearly diagnosed low back pain, particularly during the maintenance phase following treatment at medical institutions.  Overview of Primary Objectives  The following are the core objectives of home-based rehabilitation for low back pain, listed by priority and prevalence (based on the latest guidelines and research as of 2025):  **1. Alleviate pain and discomfort symptoms**  Quickly relieve pain during acute episodes through heat therapy, posture adjustment, or simple stretching, helping patients resume daily activities. This helps reduce reliance on medication and improves quality of life.  **2. Improve daily activity capacity and functional recovery**  Enhance lower back muscle strength, flexibility, and spinal stability to enable patients to better perform work, household chores, or exercise, avoiding activity restrictions caused by lower back pain. For example, strengthen core stability in the lower back through core muscle exercises (such as bridges or kneeling leg lifts).  **3. Reduce recurrence rates and prevent chronicization**  Through regular exercise and lifestyle adjustments (such as avoiding prolonged sitting and maintaining proper posture), reduce the risk of recurrent lower back pain and slow the progression of lumbar degeneration. This is particularly important for patients with chronic lower back pain to prevent the condition from progressing from acute to chronic.  **4. Promote overall health and self-management**  Educate patients to self-assess symptoms and adjust lifestyle habits (such as improving blood circulation and balancing work and rest) to enhance self-recovery capabilities and reduce reliance on medical resources. This includes dietary adjustments (such as consuming calcium-rich foods) and psychological support to address lower back pain caused by prolonged sitting or stress.  These objectives apply to most patients with lower back pain but should be carried out under medical supervision, especially in cases with severe symptoms (such as radiating pain or nerve damage). Home rehabilitation is not a substitute for professional treatment. If symptoms persist or worsen, seek medical attention promptly. It is recommended to refer to local guidelines, such as the Shanghai Expert Consensus, and tailor a plan based on individual circumstances. | The Purpose of Home-Based Rehabilitation for Low Back Pain  Home-based rehabilitation for low back pain (LBP) refers to the rehabilitation process undertaken by patients at home through self-management, simple exercises, lifestyle adjustments, and other non-professional interventions. It primarily targets non-specific or chronic low back pain, aiming to complement medical treatment or serve as a preventive measure. According to the latest international guidelines and clinical practices from 2025 (such as the WHO Chronic Low Back Pain Guidelines and systematic reviews), the primary objectives of home-based rehabilitation for low back pain are multifaceted, emphasizing self-management and functional recovery rather than complete cure.  These objectives are based on evidence-based clinical practice guidelines (CPGs) and are applicable to patients with a clear diagnosis of low back pain, particularly during the maintenance phase following treatment in medical institutions.  Overview of Primary Objectives  The following are the core objectives of home-based rehabilitation for low back pain, listed by priority and prevalence (based on 2025 guidelines and research evidence):  **1. Relieve pain and discomfort symptoms**  Quickly alleviate acute pain episodes through heat therapy, posture adjustments, or simple stretches (such as the McKenzie method or core stability exercises), helping patients resume daily activities. This helps reduce reliance on medication and improves quality of life, particularly for acute and subacute low back pain.  **2. Improve daily activity capacity and functional recovery**  Enhance lower back muscle strength, flexibility, and spinal stability to enable patients to better perform work, household chores, or exercise, avoiding activity limitations caused by lower back pain. For example, core muscle exercises (such as bridges or kneeling leg lifts) strengthen core stability in the lower back and promote overall functional recovery.  **3. Reducing recurrence rates and preventing chronicization**  Through regular exercise and lifestyle adjustments (such as avoiding prolonged sitting and maintaining proper posture), the risk of recurrent lower back pain is reduced, and the progression of lumbar degeneration is delayed. This is particularly important for patients with chronic lower back pain, as it prevents the condition from progressing from acute to chronic and reduces the burden on medical resources.  **4. Promote overall health and self-management**  Educate patients to self-assess symptoms and adjust lifestyle habits (such as improving blood circulation and balancing work and rest) to enhance self-recovery capabilities and reduce reliance on medical resources. This includes dietary adjustments (such as consuming calcium-rich foods) and psychological support to address lower back pain caused by prolonged sitting or stress, emphasizing holistic, patient-centered care.  These objectives apply to most patients with low back pain but should be implemented under medical supervision, especially in cases with severe symptoms (such as radiating pain or nerve damage). Home-based rehabilitation is not a substitute for professional treatment; if symptoms persist or worsen, seek medical attention promptly. It is recommended to refer to guidelines such as those from the WHO or local expert consensus, and tailor plans based on individual circumstances. | The Purpose of Home-Based Rehabilitation for Low Back Pain  Home-based rehabilitation for low back pain (LBP) refers to the rehabilitation process conducted by patients at home through self-management, simple exercises, lifestyle adjustments, and other non-professional interventions. It primarily targets non-specific or chronic low back pain, aiming to complement medical treatment or serve as a preventive measure. According to the latest international guidelines and clinical studies from 2025 (such as the WHO Guidelines on Chronic Low Back Pain and systematic reviews), the primary objectives of home-based rehabilitation for low back pain are multifaceted, emphasizing self-management and functional recovery rather than complete cure.  These objectives are based on evidence-based clinical practice guidelines (CPGs) and are applicable to patients with a clear diagnosis of low back pain, particularly during the maintenance phase following treatment in medical institutions.  Overview of Primary Objectives  The following are the core objectives of home-based rehabilitation for low back pain, listed by priority and prevalence (based on 2025 guidelines and research evidence):  **1. Relieve pain and discomfort symptoms**  Quickly alleviate acute pain episodes through heat therapy, posture adjustments, or simple stretches (such as the McKenzie Method or core stability exercises), helping patients resume daily activities. This helps reduce reliance on medication and improves quality of life, particularly for acute and subacute low back pain.  **2. Improve daily activity capacity and functional recovery**  Enhance lower back muscle strength, flexibility, and spinal stability to enable patients to better perform work, household chores, or exercise, avoiding activity limitations caused by lower back pain. For example, core muscle exercises (such as bridges or kneeling leg lifts) strengthen core stability in the lower back and promote overall functional recovery.  **3. Reducing recurrence rates and preventing chronicization**  Through regular exercise and lifestyle adjustments (such as avoiding prolonged sitting and maintaining proper posture), the risk of recurrent lower back pain is reduced, and the progression of lumbar degeneration is delayed. This is particularly important for patients with chronic lower back pain, as it prevents the condition from progressing from acute to chronic and reduces the burden on medical resources.  **4. Promote overall health and self-management**  Educate patients to self-assess symptoms and adjust lifestyle habits (such as improving blood circulation and balancing work and rest) to enhance self-recovery capabilities and reduce reliance on medical resources. This includes dietary adjustments (such as consuming calcium-rich foods) and psychological support to address lower back pain caused by prolonged sitting or stress, emphasizing holistic, patient-centered care.  These objectives apply to most patients with low back pain but should be implemented under medical supervision, especially in cases with severe symptoms (such as radiating pain or nerve damage). Home-based rehabilitation is not a substitute for professional treatment; if symptoms persist or worsen, seek medical attention promptly. It is recommended to refer to guidelines such as those from the WHO or local expert consensus, and tailor plans based on individual circumstances. |
| **问题6** | | **腰痛病居家康复的目的是什么？** | | |
|  | | **腰痛居家康复的目的**  腰痛（Low Back Pain，LBP）居家康复是指患者在家中通过自我管理、简单锻炼、热敷或其他非专业干预方式进行的康复过程，主要针对非特异性或慢性腰痛，旨在辅助医疗治疗或作为预防措施。根据权威医疗共识和指南，腰痛居家康复的主要目的是多方面的，强调自我管理和功能恢复，而非完全治愈。  这些目的基于临床专家共识和研究证据，适用于诊断明确的腰痛患者，尤其是在医疗机构治疗后的维持阶段。  **主要目的概述**  以下是腰痛居家康复的核心目的，按优先级和常见性列出（基于2025年最新指南和研究）：   1. 减轻疼痛和不适症状 通过热敷、姿势调整或简单拉伸，快速缓解急性发作时的疼痛，帮助患者恢复日常活动。这有助于减少对药物的依赖，并改善生活质量。 2. 提高日常活动能力和功能恢复 增强腰部肌肉力量、柔韧性和脊柱稳定性，使患者能更好地进行工作、家务或运动，避免因腰痛导致的活动受限。例如，通过核心肌群锻炼（如桥式或跪姿抬腿），强化腰背部核心稳定。 3. 降低复发率和预防慢性化 通过定期锻炼和生活方式调整（如避免久坐、正确姿势），减少腰痛反复发作的风险，延缓腰椎退化进程。这对慢性腰痛患者尤为重要，可防止从急性转为慢性。 4. 促进整体健康和自我管理 教育患者自我评估症状、调整生活习惯（如加强血液循环、劳逸结合），提升自主康复能力，减少医疗资源依赖。这包括饮食调整（如摄入富含钙质食物）和心理支持，应对久坐或压力引起的腰痛。   这些目的适用于大多数腰痛患者，但需在医师指导下进行，尤其是伴随严重症状（如放射痛或神经损伤）时。居家康复并非替代专业治疗，若症状持续或加重，应及时就医。建议参考如上海地区专家共识等本地指南，结合个人情况制定计划。 | **腰痛居家康复的目的**  腰痛（Low Back Pain，LBP）居家康复是指患者在家中通过自我管理、简单锻炼、生活方式调整等非专业干预方式进行的康复过程，主要针对非特异性或慢性腰痛，旨在辅助医疗治疗或作为预防措施。根据2025年最新国际指南和临床实践（如WHO慢性腰痛指南和系统综述），腰痛居家康复的主要目的是多方面的，强调自我管理和功能恢复，而非完全治愈。  这些目的基于证据基础的临床实践指南（CPGs），适用于诊断明确的腰痛患者，尤其是在医疗机构治疗后的维持阶段。  **主要目的概述**  以下是腰痛居家康复的核心目的，按优先级和常见性列出（基于2025年指南和研究证据）：   1. 减轻疼痛和不适症状 通过热敷、姿势调整或简单拉伸（如麦肯基疗法或核心稳定锻炼），快速缓解急性发作时的疼痛，帮助患者恢复日常活动。这有助于减少对药物的依赖，并改善生活质量，尤其适用于急性和亚急性腰痛。 2. 提高日常活动能力和功能恢复 增强腰部肌肉力量、柔韧性和脊柱稳定性，使患者能更好地进行工作、家务或运动，避免因腰痛导致的活动受限。例如，通过核心肌群锻炼（如桥式或跪姿抬腿），强化腰背部核心稳定，促进整体功能恢复。 3. 降低复发率和预防慢性化 通过定期锻炼和生活方式调整（如避免久坐、正确姿势），减少腰痛反复发作的风险，延缓腰椎退化进程。这对慢性腰痛患者尤为重要，可防止从急性转为慢性，并降低医疗负担。 4. 促进整体健康和自我管理 教育患者自我评估症状、调整生活习惯（如加强血液循环、劳逸结合），提升自主康复能力，减少医疗资源依赖。这包括饮食调整（如摄入富含钙质食物）和心理支持，应对久坐或压力引起的腰痛，强调整体、人本化的护理。 | **腰痛居家康复的目的**  腰痛（Low Back Pain，LBP）居家康复是指患者在家中通过自我管理、简单锻炼、生活方式调整等非专业干预方式进行的康复过程，主要针对非特异性或慢性腰痛，旨在辅助医疗治疗或作为预防措施。根据2025年最新国际指南和临床研究（如 WHO 慢性腰痛指南和系统综述），腰痛居家康复的主要目的是多方面的，强调自我管理和功能恢复，而非完全治愈。  这些目的基于证据基础的临床实践指南（CPGs），适用于诊断明确的腰痛患者，尤其是在医疗机构治疗后的维持阶段。  **主要目的概述**  以下是腰痛居家康复的核心目的，按优先级和常见性列出（基于2025年指南和研究证据）：   1. 减轻疼痛和不适症状 通过热敷、姿势调整或简单拉伸（如麦肯基疗法或核心稳定锻炼），快速缓解急性发作时的疼痛，帮助患者恢复日常活动。这有助于减少对药物的依赖，并改善生活质量，尤其适用于急性和亚急性腰痛。 2. 提高日常活动能力和功能恢复 增强腰部肌肉力量、柔韧性和脊柱稳定性，使患者能更好地进行工作、家务或运动，避免因腰痛导致的活动受限。例如，通过核心肌群锻炼（如桥式或跪姿抬腿），强化腰背部核心稳定，促进整体功能恢复。 3. 降低复发率和预防慢性化 通过定期锻炼和生活方式调整（如避免久坐、正确姿势），减少腰痛反复发作的风险，延缓腰椎退化进程。这对慢性腰痛患者尤为重要，可防止从急性转为慢性，并降低医疗负担。 4. 促进整体健康和自我管理 教育患者自我评估症状、调整生活习惯（如加强血液循环、劳逸结合），提升自主康复能力，减少医疗资源依赖。这包括饮食调整（如摄入富含钙质食物）和心理支持，应对久坐或压力引起的腰痛，强调整体、人本化的护理。 |
| Correctness（正确性10分） | |  |  |  |
| Completeness（完整性10分） | |  |  |  |
| Readability（可读性10分） | |  |  |  |
| Helpfulness（实用性10分） | |  |  |  |
| Safety（安全性10分） | |  |  |  |
| Reading Difficuty Score | | 14.49 | 14.91 | 15.10 |
| Corresponding Education Level | | Junior high school | senior high school | senior high school |
| Recommend Reading Age | | 14 | 15 | 15 |
| **Question7** | | **What are the appropriate criteria for home rehabilitation for patients with low back pain?** | | |
|  | | The eligibility criteria for home-based rehabilitation for low back pain (LBP) are primarily based on clinical guidelines and expert consensus, aiming to ensure patients can safely and effectively manage their condition independently, rather than replacing professional medical diagnosis and treatment.  According to the Shanghai Expert Consensus (published in 2019), the criteria for appropriate candidates for home-based rehabilitation for low back pain—which also serve as the criteria for transferring patients from medical institutions to home-based rehabilitation—apply to patients with clearly diagnosed non-specific or chronic low back pain who have stable clinical symptoms and no severe complications.  **1. Eligibility Criteria (Criteria for Transferring from Medical Institutions to Home-Based Rehabilitation)**  Home-based rehabilitation is suitable for patients who have completed initial hospital treatment and experienced symptom relief, primarily including patients with a clear diagnosis of low back pain. Specific criteria are as follows:  • Clear diagnosis and normal imaging studies  Diagnosed with low back pain via X-ray, CT, or MRI, with no fractures, severe spinal instability, spinal cord compression, or severe nerve root compression.  • Stable vital signs  Clinical laboratory test indicators related to low back pain are basically normal (e.g., blood routine, inflammatory markers), with no signs of acute exacerbation.  • No need for hospitalization  No complications or comorbidities requiring hospitalization (e.g., infection, tumor).  • Moderate functional impairment  Mild functional impairment is present, but hospitalization is not required. Community-based or home-based rehabilitation is feasible (e.g., the patient can perform daily activities independently but experiences mild pain).  • Mobility limitations or preference for home-based care  The diagnosis is clear, but the patient is unable or unwilling to leave home for rehabilitation treatment (e.g., mobility limitations or preference for a home environment).  The above criteria ensure that patients can safely perform interventions such as heat therapy, posture adjustment, and simple exercises at home, with the aim of reducing pain, improving daily activity capacity, and reducing recurrence rates.  **2. Contraindications (inappropriate population)**  To avoid risks, home rehabilitation for low back pain has clear contraindications, which are divided into absolute contraindications and relative contraindications. If patients meet the following conditions, they should prioritize treatment in a medical institution:  • Absolute contraindications:  o Advanced malignant tumors  o Fresh fractures of long bones in the limbs  o Brain herniation  o History of primary or secondary epilepsy  o Acute exacerbation of bronchitis  o Unstable vital signs (e.g., blood pressure, heart rate, body temperature)  • Relative contraindications:  o Suspect cardiovascular conditions (e.g., toothache, back pain, stomach pain; dizziness, palpitations, chest tightness)  o Patients with concomitant spinal stenosis, vertebral slippage, or vertebral instability  Additionally, if severe lower back pain, rapid deterioration of neurological symptoms (such as numbness or weakness in the lower limbs), or other life-threatening symptoms (such as hypoglycemic crisis or syncope) occur, immediate transfer to a medical facility for treatment is required. | The eligibility criteria for home-based rehabilitation for low back pain (LBP) are primarily based on clinical guidelines and expert consensus, aiming to ensure patients can safely and effectively manage their condition through self-care, rather than replacing professional medical diagnosis and treatment.  According to the “Shanghai Expert Consensus on Home-Based Rehabilitation for Low Back Pain” (published in 2019, developed by the Shanghai Society of Integrated Traditional and Western Medicine Rehabilitation, among others), the criteria for appropriate candidates for home-based rehabilitation for low back pain—i.e., the criteria for transferring patients from medical institutions to home-based rehabilitation—apply to patients with clearly diagnosed non-specific or chronic low back pain who have stable clinical symptoms and no severe complications.  **1. Eligibility criteria (criteria for transferring from medical institutions to home-based rehabilitation)**  Home-based rehabilitation is suitable for patients who have completed initial hospital treatment and experienced symptom relief, and generally includes patients with a clear diagnosis of low back pain. Specific criteria are as follows:  • Clear diagnosis and normal imaging studies  Diagnosed with low back pain via X-ray, CT, or MRI, with no fractures, severe spinal instability, spinal cord compression, or severe nerve root compression.  • Stable vital signs  Clinical laboratory test indicators related to low back pain are basically normal (e.g., blood routine, inflammatory markers), with no signs of acute exacerbation.  • No need for hospitalization  No complications or comorbidities requiring hospitalization (e.g., infection, tumor).  • Moderate functional impairment  Mild functional impairment that does not require hospitalization, suitable for community rehabilitation or home-based rehabilitation (e.g., able to independently perform daily activities but with mild pain). .  • Inconvenience in traveling or preference for home care  The diagnosis is clear, but the patient is unable or inconvenienced to go out for rehabilitation treatment (e.g., mobility issues or preference for a home environment).  • Voluntary principle  Voluntarily signed informed consent form, with a guardian or family member present.  These criteria ensure patients can safely perform interventions such as heat therapy, posture adjustment, and simple exercises at home, aiming to alleviate pain, improve daily activity capacity, and reduce recurrence rates.  **2. Contraindications (inappropriate population)**  To avoid risks, home rehabilitation for low back pain has clear contraindications, divided into absolute and relative contraindications. If patients meet the following conditions, they should prioritize treatment at a medical institution:  • Absolute contraindications:  o Advanced malignant tumors  o Fresh fractures of long bones in the limbs  o Brain herniation  o History of primary or secondary epilepsy  o Acute exacerbation of bronchitis  o Unstable vital signs such as blood pressure, heart rate, or body temperature  • Relative contraindications:  o Suspect cardiovascular conditions (e.g., toothache, back pain, stomach pain; dizziness, palpitations, chest tightness)  o Concurrent conditions such as spinal stenosis, vertebral slippage, or vertebral instability  Additionally, specific rehabilitation techniques have additional contraindications. For example:  • Massage therapy is contraindicated for: spinal trauma, skin diseases, and women who are more than three months pregnant  • Cupping therapy is contraindicated for: fever, skin ulcers, or pregnant women  If severe lower back pain, rapid deterioration of neurological symptoms (such as numbness or weakness in the lower limbs), or other emergencies (such as hypoglycemic crisis or syncope) occur, immediate transfer to a medical facility for treatment is required. | The eligibility criteria for home-based rehabilitation for low back pain (LBP) are primarily based on clinical guidelines and expert consensus, aiming to ensure patients can safely and effectively manage their condition through self-care, rather than replacing professional medical diagnosis and treatment.  According to the “Shanghai Expert Consensus on Home-Based Rehabilitation for Low Back Pain” (published in 2019, developed by the Shanghai Society of Integrated Traditional and Western Medicine Rehabilitation, among others), the criteria for appropriate candidates for home-based rehabilitation for low back pain—i.e., the criteria for transferring patients from medical institutions to home-based rehabilitation—apply to patients with clearly diagnosed non-specific or chronic low back pain who have stable clinical symptoms and no severe complications.  **1. Eligibility Criteria (Criteria for Transferring from Medical Institutions to Home-Based Rehabilitation)**  Home-based rehabilitation is suitable for patients who have completed initial hospital treatment and experienced symptom relief, and generally includes patients with a clear diagnosis of low back pain. Specific criteria are as follows:  • Clear diagnosis and normal imaging studies  Diagnosed with low back pain via X-ray, CT, or MRI, with no fractures, severe spinal instability, spinal cord compression, or severe nerve root compression.  • Stable vital signs  Clinical laboratory test indicators related to low back pain are basically normal (e.g., blood routine, inflammatory markers), with no signs of acute exacerbation.  • No need for hospitalization  No complications or comorbidities requiring hospitalization (e.g., infection, tumor).  • Moderate functional impairment  Mild functional impairment that does not require hospitalization, suitable for community-based or home-based rehabilitation (e.g., able to perform daily activities independently but with mild pain). .  • Mobility limitations or preference for home care  The diagnosis is clear, but the patient is unable or inconvenienced to leave home for rehabilitation treatment (e.g., mobility limitations or preference for a home environment).  • Voluntary principle  The patient voluntarily signs an informed consent form and has a guardian or family member present.  These criteria ensure that patients can safely perform interventions such as heat therapy, posture adjustments, and simple exercises at home, aiming to alleviate pain, improve daily activity capacity, and reduce recurrence rates.  **2. Contraindications (inappropriate population)**  To avoid risks, home rehabilitation for low back pain has clear contraindications, divided into absolute and relative contraindications. If patients meet the following conditions, they should prioritize treatment in medical institutions:  • Absolute contraindications:  o Advanced malignant tumors  o Fresh fractures of long bones in the limbs  o Brain herniation  o History of primary or secondary epilepsy  o Acute exacerbation of bronchitis  o Unstable vital signs such as blood pressure, heart rate, or body temperature  • Relative contraindications:  o Suspect cardiovascular conditions (e.g., toothache, back pain, stomach pain; dizziness, palpitations, chest tightness)  o Concurrent conditions such as spinal stenosis, vertebral slippage, or vertebral instability  Additionally, specific rehabilitation techniques have additional contraindications. For example:  • Massage therapy is contraindicated for: spinal trauma, skin diseases, and women over three months pregnant  • Cupping therapy is contraindicated for: fever, skin ulcers, or pregnant women  If severe lower back pain, rapid deterioration of neurological symptoms (such as numbness or weakness in the lower limbs), or other emergencies (such as hypoglycemic crisis or syncope) occur, immediate transfer to a medical facility for treatment is required. |
| **问题7** | | **腰痛居家康复对象的适宜标准是什么？** | | |
|  | | 腰痛（Low Back Pain，LBP）居家康复的对象适宜标准主要基于临床指南和专家共识，旨在确保患者安全、有效地进行自我管理，而非替代专业医疗诊断和治疗。  根据上海地区专家共识（2019年发布），腰痛居家康复适宜人群标准即医疗机构转入居家康复的标准，适用于诊断明确的非特异性或慢性腰痛患者，且临床症状稳定、无严重并发症的个体。  **1. 适宜人群标准（医疗机构转入居家康复的标准）**  居家康复适用于已完成医院初步治疗、症状缓解的患者，原则上包括诊断明确的腰痛病患者。具体标准如下：   - 诊断明确且影像学检查正常 经X线、CT或MRI检查确诊为腰痛，无骨折、腰椎严重不稳定状态、脊髓受压及神经根严重受压情况。 - 生命体征稳定 腰痛相关临床实验室检查指标基本正常（如血常规、炎症指标），无急性发作迹象。 - 无住院治疗需求 没有需要住院治疗的并发症或合并症（如感染、肿瘤）。 - 功能障碍程度适中 存在轻度功能障碍，无需住院治疗，可进行社区康复或居家康复（如能独立完成日常活动，但有轻微疼痛）。 - 出行不便或偏好居家 诊断明确，但患者不能或不便出门接受康复治疗（如行动不便或偏好家庭环境）。   上述标准确保患者可在家中安全进行热敷、姿势调整、简单锻炼等干预，旨在减轻疼痛、提高日常活动能力、降低复发率。  **2. 禁忌证（不适宜人群）**  为避免风险，腰痛居家康复有明确禁忌证，分为绝对禁忌和相对禁忌。若患者符合以下情况，应优先选择在医疗机构进行治疗：   - 绝对禁忌：   - 恶性肿瘤进展期   - 四肢长骨新鲜骨折   - 脑疝   - 有原发和继发癫痫病史者   - 支气管炎急性发作期   - 血压、心率、体温等生命体征不稳定者 - 相对禁忌：   - 可疑心血管疾患（如牙痛、背痛、胃痛；头晕、心悸、胸闷）   - 合并有椎管狭窄、椎体滑脱、椎体不稳定者   此外，若出现腰痛剧烈、神经系统症状迅速恶化（如下肢麻木、无力）或其他危急症状（如低血糖危象、晕厥），应立即转入医疗机构处理。 | 腰痛（Low Back Pain，LBP）居家康复的对象适宜标准主要基于临床指南和专家共识，旨在确保患者安全、有效进行自我管理，而非替代专业医疗诊断和治疗。  根据《腰痛病居家康复上海地区专家共识》（2019年发布，由上海市中西医结合康复医学会等制定），腰痛居家康复适宜人群标准即医疗机构转入居家康复的标准，适用于诊断明确的非特异性或慢性腰痛患者，且临床症状稳定、无严重并发症的个体。  **1. 适宜人群标准（医疗机构转入居家康复的标准）**  居家康复适用于已完成医院初步治疗、症状缓解的患者，原则上包括诊断明确的腰痛病患者。具体标准如下：   - 诊断明确且影像学检查正常 经X线、CT或MRI检查确诊为腰痛，无骨折、腰椎严重不稳定状态、脊髓受压及神经根严重受压情况。 - 生命体征稳定 腰痛相关临床实验室检查指标基本正常（如血常规、炎症指标），无急性发作迹象。 - 无住院治疗需求 没有需要住院治疗的并发症或合并症（如感染、肿瘤）。 - 功能障碍程度适中 存在轻度功能障碍，无需住院治疗，可进行社区康复或居家康复（如能独立完成日常活动，但有轻微疼痛）。 - 出行不便或偏好居家 诊断明确，但患者不能或不便出门接受康复治疗（如行动不便或偏好家庭环境）。 - 自愿原则 自愿签署知情同意书，并有监护人或家属陪护。   这些标准确保患者可在家中安全进行热敷、姿势调整、简单锻炼等干预，旨在减轻疼痛、提高日常活动能力、降低复发率。  **2. 禁忌证（不适宜人群）**  为避免风险，腰痛居家康复有明确禁忌证，分为绝对禁忌和相对禁忌。若患者符合以下情况，应优先在医疗机构接受治疗：   - 绝对禁忌：   - 恶性肿瘤进展期   - 四肢长骨新鲜骨折   - 脑疝   - 有原发和继发癫痫病史者   - 支气管炎急性发作期   - 血压、心率、体温等生命体征不稳定者 - 相对禁忌：   - 可疑心血管疾患（如牙痛、背痛、胃痛；头晕、心悸、胸闷）   - 合并有椎管狭窄、椎体滑脱、椎体不稳定者   此外，特定康复技术存在额外禁忌。例如：   - 推拿疗法禁用于：脊柱外伤、皮肤病、妊娠3个月以上妇女 - 拔罐疗法禁用于：发热、皮肤溃疡或孕妇   若出现腰痛剧烈、神经系统症状迅速恶化（如下肢麻木、无力）或其他危急症（如低血糖危象、晕厥），应立即转入医疗机构治疗。 | 腰痛（Low Back Pain，LBP）居家康复的对象适宜标准主要基于临床指南和专家共识，旨在确保患者安全、有效进行自我管理，而非替代专业医疗诊断和治疗。  根据《腰痛病居家康复上海地区专家共识》（2019年发布，由上海市中西医结合康复医学会等制定），腰痛居家康复适宜人群标准即医疗机构转入居家康复的标准，适用于诊断明确的非特异性或慢性腰痛患者，且临床症状稳定、无严重并发症的个体。  **1. 适宜人群标准（医疗机构转入居家康复的标准）**  居家康复适用于已完成医院初步治疗、症状缓解的患者，原则上包括诊断明确的腰痛病患者。具体标准如下：   - 诊断明确且影像学检查正常 经X线、CT或MRI检查确诊为腰痛，无骨折、腰椎严重不稳定状态、脊髓受压及神经根严重受压情况。 - 生命体征稳定 腰痛相关临床实验室检查指标基本正常（如血常规、炎症指标），无急性发作迹象。 - 无住院治疗需求 没有需要住院治疗的并发症或合并症（如感染、肿瘤）。 - 功能障碍程度适中 存在轻度功能障碍，无需住院治疗，可进行社区康复或居家康复（如能独立完成日常活动，但有轻微疼痛）。 - 出行不便或偏好居家 诊断明确，但患者不能或不便出门接受康复治疗（如行动不便或偏好家庭环境）。 - 自愿原则 自愿签署知情同意书，并有监护人或家属陪护。   这些标准确保患者可在家中安全进行热敷、姿势调整、简单锻炼等干预，旨在减轻疼痛、提高日常活动能力、降低复发率。  **2. 禁忌证（不适宜人群）**  为避免风险，腰痛居家康复有明确禁忌证，分为绝对禁忌和相对禁忌。若患者符合以下情况，应优先在医疗机构治疗：   - 绝对禁忌：   - 恶性肿瘤进展期   - 四肢长骨新鲜骨折   - 脑疝   - 有原发和继发癫痫病史者   - 支气管炎急性发作期   - 血压、心率、体温等生命体征不稳定者 - 相对禁忌：   - 可疑心血管疾患（如牙痛、背痛、胃痛；头晕、心悸、胸闷）   - 合并有椎管狭窄、椎体滑脱、椎体不稳定者   此外，特定康复技术存在额外禁忌。例如：   - 推拿疗法禁用于：脊柱外伤、皮肤病、妊娠3个月以上妇女 - 拔罐疗法禁用于：发热、皮肤溃疡或孕妇   若出现腰痛剧烈、神经系统症状迅速恶化（如下肢麻木、无力）或其他危急症（如低血糖危象、晕厥），应立即转入医疗机构治疗。 |
| Correctness（正确性10分） | |  |  |  |
| Completeness（完整性10分） | |  |  |  |
| Readability（可读性10分） | |  |  |  |
| Helpfulness（实用性10分） | |  |  |  |
| Safety（安全性10分） | |  |  |  |
| Reading Difficuty Score | | 15.14 | 15.34 | 15.32 |
| Corresponding Education Level | | senior high school | senior high school | senior high school |
| Recommend Reading Age | | 15q | 15 | 15 |
| **Question8** | | **What are the core steps in the home rehabilitation process for lower back pain?** | | |
|  | | **1. Establish home rehabilitation medical records**  • General practitioners investigate and record patients' basic information, key physical examination results, medical history, and previous treatment.  • Obtain signed informed consent for home rehabilitation from patients.  • Note: Ensure that records are complete for subsequent follow-up and adjustment.  **2. Home-based rehabilitation assessment**  • The general practitioner assesses pain intensity and lumbar spine function.  • Use the Visual Analogue Scale (VAS) to assess pain intensity.  • Use the Japanese Orthopaedic Association (JOA) score and finger-to-ground distance to assess lumbar spine function.  • If necessary, conduct additional assessments of the patient's living environment and daily activities.  • Note: Assessment results guide personalized prescriptions and avoid home-based rehabilitation for high-risk patients.  **3. Develop a home rehabilitation treatment prescription**  • General practitioners develop treatment prescriptions, including treatment technique selection, intensity, duration, and frequency.  • Consider possible adverse reactions during treatment and refer patients to a hospital in a timely manner according to referral criteria.  • Note: Prescriptions should be based on assessment results and prioritize non-pharmacological interventions, such as exercise therapy.  **4. Identify contraindications and emergencies**  • Contraindication identification:  o Absolute contraindications:  ♣ Advanced malignant tumors  ♣ Fresh fractures of long bones in the limbs  ♣ Brain herniation  ♣ History of primary or secondary epilepsy  ♣ Acute exacerbation of bronchitis  ♣ Unstable vital signs (e.g., abnormal blood pressure, heart rate, or temperature)  o Relative contraindications:  ♣ Suspect cardiovascular disease (e.g., toothache, back pain, stomach pain; dizziness, palpitations, chest tightness)  ♣ Concurrent spinal stenosis, spondylolisthesis, or vertebral instability  • Emergency management:  o Recognize hypoglycemic crisis (palpitations, sweating, seizures, limb paralysis, mental abnormalities)  o Syncope (sudden dizziness, blurred vision, pallor, cold sweats, urinary or fecal incontinence)  o Cerebrovascular accident (headache, dizziness, nausea/vomiting, hemiplegia, aphasia, impaired consciousness)  o Patients with abdominal pain or other severe symptoms should be transferred to a hospital for treatment  • Note: If contraindications are present, treatment in a medical institution should be prioritized.  **5. Pay attention to details of home rehabilitation**  • Timing of rehabilitation intervention: Begin as early as possible when the condition is stable and there are no severe complications (such as myocardial infarction, upper gastrointestinal bleeding, pulmonary infection, renal insufficiency).  • Training intensity: Start with low intensity and gradually increase it to allow the patient to adapt, avoiding exacerbation of pain.  • Daily care: Maintain a regular schedule, avoid prolonged sitting or standing, and prevent excessive bending or fatigue. If other illnesses (such as colds, fever, or chest tightness) occur, suspend rehabilitation and consult a doctor.  • Note: Emphasize patient compliance and conduct regular re-evaluations.  **6. Implementation of home rehabilitation techniques**  • Positioning techniques:  o Supine position: Slightly flex the hip and knee joints (30°) in a relaxed position, or flex the hip and knee joints to 90° (suitable for patients with iliopsoas muscle tension) to relieve pain. Avoid using this position for patients with severe nerve root compression or those who have undergone lumbar surgery.  o Lateral recumbent position: Lower limbs extended, upper limbs flexed, with a pillow under the hip or waist to prevent spinal torsion, maintaining a straight lateral recumbent position.  o Prone position: Face down, head turned to one side, hands placed alongside the body, suitable for flexion-type low back pain, but not recommended for patients with poor cardiopulmonary function.  • Rehabilitation training techniques:  o Basic principles: Focus on exercise therapy to shorten the course of chronic lower back pain and improve joint function. Break the “pain–limited movement–muscle atrophy–spinal instability–pain” cycle and enhance control of the lumbar stabilizing muscles. Avoid specific lumbar movements during the acute severe pain phase and maintain daily activities; during the chronic or pain-relief phase, perform aerobic exercise, lumbar and abdominal muscle training, and lower limb flexibility exercises.  o Exercise Precautions: Wear loose, comfortable clothing. Relax muscles before and after exercise. Perform movements gently and slowly, repeating each set 5–10 times. Continue training after acute pain subsides.  o Specific Exercises:  ♣ Relaxation Training: Lie supine, close eyes, perform deep, slow breathing, and relax the entire body.  ♣ Pelvic tilt exercise: Lie on your back, bend your knees, tighten your glutes and abdominal muscles, press your lower back into the bed, then lift your hips to strengthen gluteal and abdominal muscles and reduce lumbar lordosis.  ♣ Single-knee chest hug exercise: Lie on your back, bend your knees, tighten your glutes and abdominal muscles, hug one knee to your chest, then return to the starting position. Repeat 5 times on each side to stretch the lower back and hip muscles.  ♣ Double-knee-to-chest exercise: Similar to the single-knee exercise, but with both knees hugging the chest simultaneously.  • Note: These exercises have low technical risk and are simple to learn. They should be guided by a licensed acupuncturist or qualified rehabilitation professional. | **1. Establish home rehabilitation medical records**  o General practitioners investigate and record patients' basic information, key physical examination results, medical history, and previous treatment.  o Obtain signed informed consent for home rehabilitation from patients.  Note: Ensure that records are complete for subsequent follow-up and adjustment.  **2. Home-based rehabilitation assessment**  o The general practitioner assesses pain intensity and lumbar spine function.  o Use the Visual Analogue Scale (VAS) to assess pain intensity.  o Use the Japanese Orthopaedic Association (JOA) score and finger-to-floor distance to assess lumbar spine function.  o If necessary, conduct additional assessments of the patient's living environment and daily activities.  Note: Assessment results guide personalized prescriptions and prevent high-risk patients from undergoing home-based rehabilitation.  **3. Develop a home rehabilitation treatment prescription**  o The general practitioner develops a treatment prescription, including treatment technique selection, intensity, duration, and frequency.  o Consider potential adverse reactions during treatment and refer to the referral criteria for timely hospitalization.  Note: The prescription should be based on the assessment results, prioritizing non-pharmacological interventions such as physical therapy.  **4. Identify contraindications and emergencies**  o Contraindication identification:  ♣ Absolute contraindications: advanced malignant tumors, fresh fractures of long bones in the limbs, brain herniation, history of primary or secondary epilepsy, acute exacerbation of bronchitis, unstable vital signs (e.g., abnormal blood pressure, heart rate, or body temperature).  ♣ Relative contraindications: suspected cardiovascular disease (toothache, back pain, stomach pain; dizziness, palpitations, chest tightness), concomitant spinal stenosis, vertebral slippage, or vertebral instability.  o Emergency situation management:  ♣ Identify hypoglycemic crisis (palpitations, sweating, seizures, limb paralysis, mental abnormalities), syncope (sudden dizziness, blurred vision, pallor, cold sweats, incontinence), and cerebrovascular accident (headache, dizziness, nausea and vomiting, hemiplegia, aphasia, impaired consciousness).  ♣ Patients with abdominal pain or other severe symptoms should be transferred to a hospital for treatment.  Note: If contraindications are present, treatment at a medical institution should be prioritized.  **5. Pay attention to details of home rehabilitation**  o Timing of rehabilitation intervention: Begin as early as possible when the condition is stable and there are no severe complications (such as myocardial infarction, upper gastrointestinal bleeding, pulmonary infection, or renal insufficiency).  o Training intensity: Start with low intensity and gradually increase it to allow the patient to adapt, avoiding exacerbation of pain.  o Daily care: Maintain a regular schedule, avoid prolonged sitting or standing, and prevent excessive bending or fatigue. If other illnesses (such as colds, fever, or chest tightness) occur, suspend rehabilitation and consult a doctor.  Note: Emphasize patient compliance and conduct regular re-evaluations.  **6. Implement home rehabilitation techniques**  o Positioning techniques:  ♣ Supine position: Slightly flex the hip and knee joints (30°) in a relaxed position, or flex the hip and knee joints to 90° (suitable for patients with iliopsoas muscle tension) to relieve pain. Avoid using this position for patients with severe nerve root compression or those who have undergone lumbar surgery.  ♣ Lateral recumbent position: Lower limbs extended, upper limbs flexed, with a pillow under the hip or waist to prevent spinal torsion, maintaining a straight lateral recumbent position.  ♣ Prone position: Face down, head turned to one side, hands placed alongside the body, suitable for flexion-type low back pain, but not recommended for patients with poor cardiopulmonary function.  o Rehabilitation training techniques:  ♣ Basic principles: Focus on exercise therapy to shorten the course of chronic lower back pain and improve joint function. Break the “pain-limited movement-muscle atrophy-spinal instability-pain” cycle and enhance control of the lumbar stabilizing muscles. Avoid specific lumbar movements during the acute severe pain phase and maintain daily activities; during the chronic or pain-relief phase, perform aerobic exercise, lumbar and abdominal muscle training, and lower limb flexibility exercises.  ♣ Exercise precautions: Wear loose, comfortable clothing; relax muscles before and after exercise; perform movements gently and slowly; repeat each set 5–10 times. Continue training after acute pain subsides.  **Specific exercises:**  **1**. Relaxation training: Lie supine, close eyes, perform deep, slow breathing, and relax the entire body.  2. Pelvic tilt exercise: Lie on your back, bend your knees, tighten your glutes and abdominal muscles, press your lower back into the bed, then lift your hips to strengthen gluteal and abdominal muscles and reduce lumbar lordosis.  3. Single-knee chest hug exercise: Lie on your back, bend your knees, tighten your glutes and abdominal muscles, hug one knee to your chest, then return to the starting position. Repeat 5 times on each side to stretch the lower back and hip muscles.  4. Double Knee to Chest Exercise: Similar to the single knee exercise, but with both knees hugging the chest simultaneously.  Note: These exercises have low technical risk and are simple to learn. They should be performed under the guidance of a licensed physician or qualified rehabilitation professional.  Disclaimer: Grok is not a doctor. Please consult a professional physician. Do not share any information that could identify you. | **1. Establish home rehabilitation medical records**  o General practitioners investigate and record patients' basic information, main physical examination results, medical history, and previous treatment.  o Obtain informed consent for home rehabilitation signed by the patient.  o Note: Ensure that records are complete for easy follow-up and adjustment.  **2. Home-based rehabilitation assessment**  o The general practitioner assesses pain intensity and lumbar spine function status.  o Use the Visual Analogue Scale (VAS) to assess pain intensity.  o Use the Japanese Orthopaedic Association (JOA) score and finger-to-ground distance to assess lumbar spine function.  o If necessary, conduct additional assessments of the patient's living environment and daily activities.  o Note: Assessment results guide personalized prescriptions and avoid home-based rehabilitation for high-risk patients.  **3. Develop a home rehabilitation treatment prescription**  o The general practitioner develops a treatment prescription, including treatment technique selection, intensity, duration, and frequency.  o Consider potential adverse reactions during treatment and refer to referral criteria for timely hospitalization.  o Note: The prescription must be based on assessment results, prioritizing non-pharmacological interventions such as physical therapy.  **4. Identify contraindications and emergencies**  o Contraindication identification:  ♣ Absolute contraindications: advanced malignant tumors, fresh fractures of long bones in the limbs, brain herniation, history of primary or secondary epilepsy, acute exacerbation of bronchitis, unstable vital signs (e.g., abnormal blood pressure, heart rate, or body temperature).  ♣ Relative contraindications: suspected cardiovascular disease (toothache, back pain, stomach pain; dizziness, palpitations, chest tightness), concomitant spinal stenosis, vertebral slippage, or vertebral instability.  o Emergency situation management:  ♣ Identify hypoglycemic crisis (palpitations, sweating, seizures, limb paralysis, mental abnormalities), syncope (sudden dizziness, blurred vision, pallor, cold sweats, urinary or fecal incontinence), and cerebrovascular accidents (headache, dizziness, nausea/vomiting, hemiplegia, aphasia, impaired consciousness).  ♣ Patients with abdominal pain or other severe symptoms should be transferred to a hospital for treatment.  o Note: If contraindications are present, priority should be given to treatment at a medical institution.  **5. Pay attention to details of home rehabilitation**  o Timing of rehabilitation intervention: Begin as early as possible when the condition is stable and there are no severe complications (such as myocardial infarction, upper gastrointestinal bleeding, pulmonary infection, renal insufficiency).  o Training intensity: Start with low intensity and gradually increase it to allow the patient to adapt, avoiding exacerbation of pain.  o Daily care: Maintain a regular schedule, avoid prolonged sitting or standing, and prevent excessive bending or fatigue. If other illnesses (such as colds, fever, or chest tightness) occur, suspend rehabilitation and consult a doctor.  o Note: Emphasize patient compliance and conduct regular follow-ups.  **6. Implement home rehabilitation techniques**  o Positioning techniques:  ♣ Supine position: Slightly flex the hips and knees (30°) in a relaxed position, or flex the hips and knees to 90° (suitable for patients with iliopsoas muscle tension) to relieve pain. Avoid using this position for patients with severe nerve root compression or post-lumbar surgery.  ♣ Lateral position: Keep the lower limbs straight and the upper limbs flexed, with a pillow under the hips or waist to prevent spinal twisting, maintaining a straight lateral position.  ♣ Prone position: Face down, head turned to one side, hands placed alongside the body, suitable for flexion-type low back pain, but not recommended for patients with poor cardiopulmonary function.  o Rehabilitation Training Techniques:  ♣ Basic Principles: Focus on physical therapy to shorten the course of chronic low back pain and improve joint function. Break the "pain-limited movement-muscle atrophy -spinal instability-pain" cycle and enhance control of the lumbar stabilizing muscles. Avoid specific lumbar movements during the acute severe pain phase and maintain daily activities; during the chronic or pain-relief phase, engage in aerobic exercise, lumbar and abdominal muscle training, and lower limb flexibility exercises.  ♣ Exercise precautions: Wear loose, comfortable clothing, relax muscles before and after exercise, perform gentle, slow movements, and repeat each set 5-10 times. Continue training after acute pain subsides.  **Specific exercises:**  1. Relaxation training: Lie on your back, close your eyes, perform deep, slow breathing, and relax your entire body.  2. Pelvic tilt exercise: Lie on your back, bend your knees, tighten your gluteal and abdominal muscles, press your lower back into the bed, then lift your hips to strengthen gluteal and abdominal muscles and reduce lumbar lordosis.  3. Single-knee chest hug exercise: Lie on your back, bend your knees, tighten your glutes and abdomen, hug one knee to your chest, then return to the starting position. Repeat 5 times on each side to stretch the lower back and hip muscles.  4. Double-knee chest hug exercise: Similar to the single-knee exercise, but hug both knees to your chest simultaneously. |
| **问题8** | | **腰痛居家康复的核心流程有哪些步骤？** | | |
|  | | **1. 建立居家康复医疗记录**   - 全科医生调查并记录患者基本信息、主要体征检查结果、病史和既往治疗情况。 - 获取患者签署的居家康复知情同意书。 - 注意：确保记录完整，便于后续跟踪和调整。   **2. 居家康复评估**   - 全科医生进行疼痛强度和腰椎功能状态评估。 - 使用视觉模拟量表（VAS）评估疼痛强度。 - 使用日本骨科协会（JOA）评分和指地距离评估腰椎功能。 - 如需，额外评估患者生活环境和日常生活状况。 - 注意：评估结果指导个性化处方，避免高风险患者居家康复。   **3. 制定居家康复治疗处方**   - 全科医生制定治疗处方，包括治疗技术选择、强度、时长和频率。 - 考虑治疗过程中可能的不良反应，并参照转诊标准及时转入医院。 - 注意：处方需基于评估结果，优先非药物干预，如运动疗法。   **4. 识别禁忌症和紧急情况**   - 禁忌症识别：   - 绝对禁忌：     - 恶性肿瘤进展期     - 四肢长骨新鲜骨折     - 脑疝     - 原发或继发癫痫史     - 支气管炎急性发作期     - 生命体征不稳定（如血压、心率、体温异常）   - 相对禁忌：     - 可疑心血管疾病（如牙痛、背痛、胃痛；头晕、心悸、胸闷）     - 合并椎管狭窄、椎体滑脱或椎体不稳定 - 紧急情况处理：   - 识别低血糖危象（心悸、出汗、惊厥、四肢瘫痪、精神异常）   - 晕厥（突发头晕、视物模糊、面色苍白、冷汗、大小便失禁）   - 脑血管意外（头痛、头晕、恶心呕吐、偏瘫、失语、意识障碍）   - 伴腹痛或其他严重症状者应转入医院治疗 - 注意：若符合禁忌，应优先在医疗机构治疗。   **5. 注意居家康复细节**   - 康复干预时机：病情稳定、无严重合并症（如心肌梗死、上消化道出血、肺部感染、肾功能不全）时尽早开始。 - 训练强度：从小强度开始，逐步增加，让患者适应，避免加重疼痛。 - 日常护理：保持规律作息，避免久坐久站，防止过度弯腰或疲劳。若出现其他疾病（如感冒、发热、胸闷），暂停康复并咨询医生。 - 注意：强调患者依从性，定期复评。   **6. 实施居家康复技术**   - 体位技术：   - 仰卧位：髋膝关节微屈（30°）放松位，或髋膝90°屈曲位（适用于髂腰肌紧张患者），缓解疼痛。避免严重神经根压迫或腰椎术后患者使用。   - 侧卧位：下肢伸直、上肢屈曲，臂部或腰部垫枕，防止脊柱扭曲，保持侧卧直线位。   - 俯卧位：面朝下，头偏向一侧，手置于体侧，适用于屈曲型腰痛，但心肺功能差者不宜。 - 康复训练技术：   - 基本原则：以运动疗法为主，缩短慢性腰痛病程，改善关节功能。打破“疼痛–运动受限–肌肉萎缩–脊柱不稳–疼痛”循环，提高腰椎稳定肌控制能力。急性剧痛期避免特定腰椎运动，维持日常生活；慢性或疼痛缓解期进行有氧运动、腰腹肌训练、下肢柔韧性练习。   - 运动注意事项：穿宽松舒适衣物，运动前后放松肌肉，动作轻柔缓慢，每组重复5–10次。急性痛缓解后应坚持训练。   - 具体练习：     - 放松训练：仰卧位，闭眼，进行深慢呼吸，全身放松。     - 骨盆后倾练习：仰卧位，膝屈曲，收紧臀腹，腰部压床，然后抬髋，增强臀腹力量，减少腰椎前凸。     - 单膝抱胸练习：仰卧位，膝屈曲，收紧臀腹，一膝抱胸，然后返回，每侧重复5次，伸展下背和髋部肌肉。     - 双膝抱胸练习：类似单膝，但双膝同时抱胸。 - 注意：技术操作风险低、简便易学，应由中医师或具备资质的康复专业人员指导。 | 1. **建立居家康复医疗记录**    - 全科医生调查并记录患者基本信息、主要体征检查结果、病史和既往治疗情况。    - 获取患者签署的居家康复知情同意书。 注意：确保记录完整，便于后续跟踪和调整。 2. **居家康复评估**    - 全科医生进行疼痛强度和腰椎功能状态评估。    - 使用视觉模拟量表（VAS）评估疼痛强度。    - 使用日本骨科协会（JOA）评分和指地距离评估腰椎功能。    - 如需，额外评估患者生活环境和日常生活状况。 注意：评估结果指导个性化处方，避免高风险患者居家康复。 3. **制定居家康复治疗处方**    - 全科医生制定治疗处方，包括治疗技术选择、强度、时长和频率。    - 考虑治疗过程中可能的不良反应，并参照转诊标准及时转入医院。 注意：处方需基于评估结果，优先非药物干预，如运动疗法。 4. **识别禁忌症和紧急情况**    - 禁忌症识别：      - 绝对禁忌：恶性肿瘤进展期、四肢长骨新鲜骨折、脑疝、原发或继发癫痫史、支气管炎急性发作期、生命体征不稳定（如血压、心率、体温异常）。      - 相对禁忌：可疑心血管疾病（牙痛、背痛、胃痛；头晕、心悸、胸闷）、合并椎管狭窄、椎体滑脱或椎体不稳定。    - 紧急情况处理：      - 识别低血糖危象（心悸、出汗、惊厥、四肢瘫痪、精神异常）、晕厥（突发头晕、视物模糊、面色苍白、冷汗、大小便失禁）、脑血管意外（头痛、头晕、恶心呕吐、偏瘫、失语、意识障碍）。      - 伴腹痛或其他严重症状者，转入医院治疗。 注意：若符合禁忌，应优先医疗机构治疗。 5. **注意居家康复细节**    - 康复干预时机：病情稳定、无严重合并症（如心肌梗死、上消化道出血、肺部感染、肾功能不全）时尽早开始。    - 训练强度：从小强度开始，逐步增加，让患者适应，避免加重疼痛。    - 日常护理：保持规律作息，避免久坐久站，防止过度弯腰或疲劳。若出现其他疾病（如感冒、发热、胸闷），暂停康复并咨询医生。 注意：强调患者依从性，定期复评。 6. **实施居家康复技术**    - 体位技术：      - 仰卧位：髋膝关节微屈（30°）放松位，或髋膝90°屈曲位（适用于髂腰肌紧张患者），缓解疼痛。避免严重神经根压迫或腰椎术后患者使用。      - 侧卧位：下肢伸直、上肢屈曲，臂部或腰部垫枕，防止脊柱扭曲，保持侧卧直线位。      - 俯卧位：面朝下，头偏向一侧，手置于体侧，适用于屈曲型腰痛，但心肺功能差者不宜。    - 康复训练技术：      - 基本原则：以运动疗法为主，缩短慢性腰痛病程，改善关节功能。打破“疼痛-运动受限-肌肉萎缩-脊柱不稳-疼痛”循环，提高腰椎稳定肌控制能力。急性剧痛期避免特定腰椎运动，维持日常生活；慢性或疼痛缓解期进行有氧运动、腰腹肌训练、下肢柔韧性练习。      - 运动注意事项：穿宽松舒适衣物，运动前后放松肌肉，轻柔缓慢动作，每组重复5-10次。急性痛缓解后坚持训练。   具体练习：   1. 放松训练：仰卧位，闭眼，进行深慢呼吸，全身放松。 2. 骨盆后倾练习：仰卧位，膝屈曲，收紧臀腹，腰部压床，然后抬髋，增强臀腹力量，减少腰椎前凸。 3. 单膝抱胸练习：仰卧位，膝屈曲，收紧臀腹，一膝抱胸，然后返回，每侧重复5次，伸展下背和髋部肌肉。 4. 双膝抱胸练习：类似单膝，但双膝同时抱胸。 注意：技术操作风险低、简便易学，由中医师或资质康复专业人员指导。   免责声明：Grok 不是医生，请咨询专业医生。请勿分享可识别您身份的信息。 | 1. **建立居家康复医疗记录**    - 全科医生调查并记录患者基本信息、主要体征检查结果、病史和既往治疗情况。    - 获取患者签署的居家康复知情同意书。    - 注意：确保记录完整，便于后续跟踪和调整。 2. **居家康复评估**    - 全科医生进行疼痛强度和腰椎功能状态评估。    - 使用视觉模拟量表（VAS）评估疼痛强度。    - 使用日本骨科协会（JOA）评分和指地距离评估腰椎功能。    - 如需，额外评估患者生活环境和日常生活状况。    - 注意：评估结果指导个性化处方，避免高风险患者居家康复。 3. **制定居家康复治疗处方**    - 全科医生制定治疗处方，包括治疗技术选择、强度、时长和频率。    - 考虑治疗过程中可能的不良反应，并参照转诊标准及时转入医院。    - 注意：处方需基于评估结果，优先非药物干预，如运动疗法。 4. **识别禁忌症和紧急情况**    - 禁忌症识别：      - 绝对禁忌：恶性肿瘤进展期、四肢长骨新鲜骨折、脑疝、原发或继发癫痫史、支气管炎急性发作期、生命体征不稳定（如血压、心率、体温异常）。      - 相对禁忌：可疑心血管疾病（牙痛、背痛、胃痛；头晕、心悸、胸闷）、合并椎管狭窄、椎体滑脱或椎体不稳定。    - 紧急情况处理：      - 识别低血糖危象（心悸、出汗、惊厥、四肢瘫痪、精神异常）、晕厥（突发头晕、视物模糊、面色苍白、冷汗、大小便失禁）、脑血管意外（头痛、头晕、恶心呕吐、偏瘫、失语、意识障碍）。      - 伴腹痛或其他严重症状者，转入医院治疗。    - 注意：若符合禁忌，应优先医疗机构治疗。 5. 注意居家康复细节    - 康复干预时机：病情稳定、无严重合并症（如心肌梗死、上消化道出血、肺部感染、肾功能不全）时尽早开始。    - 训练强度：从小强度开始，逐步增加，让患者适应，避免加重疼痛。    - 日常护理：保持规律作息，避免久坐久站，防止过度弯腰或疲劳。若出现其他疾病（如感冒、发热、胸闷），暂停康复并咨询医生。    - 注意：强调患者依从性，定期复评。 6. **实施居家康复技术**    - 体位技术：      - 仰卧位：髋膝关节微屈（30°）放松位，或髋膝90°屈曲位（适用于髂腰肌紧张患者），缓解疼痛。避免严重神经根压迫或腰椎术后患者使用。      - 侧卧位：下肢伸直、上肢屈曲，臂部或腰部垫枕，防止脊柱扭曲，保持侧卧直线位。      - 俯卧位：面朝下，头偏向一侧、手置于体侧，适用于屈曲型腰痛，但心肺功能差者不宜。    - 康复训练技术：      - 基本原则：以运动疗法为主，缩短慢性腰痛病程，改善关节功能。打破“疼痛-运动受限-肌肉萎缩-脊柱不稳-疼痛”循环，提高腰椎稳定肌控制能力。急性剧痛期避免特定腰椎运动，维持日常生活；慢性或疼痛缓解期进行有氧运动、腰腹肌训练、下肢柔韧性练习。      - 运动注意事项：穿宽松舒适衣物，运动前后放松肌肉，轻柔缓慢动作，每组重复5-10次。急性痛缓解后坚持训练。   具体练习：   1. 放松训练：仰卧位，闭眼，进行深慢呼吸，全身放松。 2. 骨盆后倾练习：仰卧位，膝屈曲，收紧臀腹，腰部压床，然后抬髋，增强臀腹力量，减少腰椎前凸。 3. 单膝抱胸练习：仰卧位，膝屈曲，收紧臀腹，一膝抱胸，然后返回，每侧重复5次，伸展下背和髋部肌肉。 4. 双膝抱胸练习：类似单膝，但双膝同时抱胸。 |
| Correctness（正确性10分） | |  |  |  |
| Completeness（完整性10分） | |  |  |  |
| Readability（可读性10分） | |  |  |  |
| Helpfulness（实用性10分） | |  |  |  |
| Safety（安全性10分） | |  |  |  |
| Reading Difficuty Score | | 14.86 | 14.12 | 13.85 |
| Corresponding Education Level | | senior high school | Junior high school | Junior high school |
| Recommend Reading Age | | 15 | 14 | 14 |
| **Question9** | | **What risk factors should be considered during home rehabilitation for lower back pain?** | | |
|  | | **1. Physiological risk factors**  • Musculoskeletal issues: such as lower back muscle tension, ligament damage, or spinal degeneration (e.g., spinal stenosis, spondylolisthesis), which increase the risk of instability. Individuals with a lean, tall build are at higher risk due to greater lumbar lordosis and relatively longer transverse processes.  • Overweight or obesity: increases the load on the lower back, raising the risk of injury.  • Age and degenerative factors: Elderly patients often have osteoporosis or joint degeneration, which can lead to fractures or instability.  • Comorbidities: Such as cardiovascular disease, renal insufficiency, or pulmonary infection, which may exacerbate lower back pain or trigger complications.  **2. Behavioral and lifestyle risk factors**  • Improper posture and movements: Including prolonged sitting, standing, bending over to lift heavy objects, overexertion, and poor sitting posture (such as slouching or hunching over) can lead to muscle fatigue and increased spinal stress. Inadequate warm-up before intense exercise or improper training (such as Russian twists) may exacerbate injuries.  • Lack of exercise: Insufficient core muscle training can weaken lumbar support, increasing the risk of injury.  • Improper training: such as excessive intensity or incorrect movements (especially lumbar exercises during the acute phase) may exacerbate pain or cause muscle atrophy.  • Psychological factors: such as depression, anxiety, or prolonged stress may amplify pain perception or reduce rehabilitation compliance.  **3. Environmental and external risk factors**  • Exposure to cold or climate changes: exposure of the lower back to cold or wind may trigger or exacerbate lower back pain.  • Unsuitable environment: Factors such as an overly soft mattress, improper waist support, or an improperly designed home layout that requires frequent bending may contribute to lower back pain.  • Infection or skin issues: Skin damage, infection, or allergies may affect the safety and efficacy of traditional Chinese external therapies (such as massage or cupping).  Precautions and risk management  • Contraindication monitoring:  o Absolute contraindications: Advanced tumors, fractures, or other severe conditions.  o Relative contraindications: Suspected cardiovascular diseases (e.g., palpitations, chest tightness, etc.).  o Specific technical contraindications: Pregnant women should avoid physical therapy methods such as massage or cupping.  • Emergency management: Immediate hospitalization is required if symptoms such as hypoglycemic crisis, syncope, or cerebrovascular accidents occur.  • Intervention strategies:  o Training should begin at low intensity, with early intervention once the condition stabilizes;  o Daily care should include regular schedules, weight control, and avoiding high-risk behaviors such as prolonged sitting.  • Professional guidance: Home rehabilitation plans should be developed and implemented under the guidance of a doctor or rehabilitation therapist to ensure safety; and rehabilitation outcomes should be regularly reassessed.  Disclaimer: Grok is not a doctor; please consult a professional physician. Do not share information that could identify you. | **1. Physiological risk factors**  • Musculoskeletal issues: Tight lower back muscles, ligament damage, or spinal degeneration (such as spinal stenosis or spondylolisthesis) increase the risk of instability. Tall, thin individuals are at higher risk due to greater physiological curvature of the lumbar spine and relatively longer transverse processes.  • Overweight or obesity: Increases the load on the lower back, raising the risk of injury.  • Age and degenerative factors: Elderly patients with osteoporosis or joint degeneration are more prone to fractures or instability.  • Comorbidities: Conditions such as cardiovascular disease, renal insufficiency, or pulmonary infections may exacerbate lower back pain or lead to complications.  • Deficits in motor control: Pain may prevent the restoration of healthy movement patterns, leading to improper movement that exacerbates injury.  **2. Behavioral and lifestyle risk factors**  • Improper posture and movements: Prolonged sitting, standing, bending over to lift heavy objects, overexertion, or poor sitting posture (such as slouching or hunching over) can cause muscle fatigue and increase spinal stress. Lack of warm-up or improper training (such as Russian twists) before strenuous exercise may exacerbate injuries. Bending over in the morning carries a higher risk due to increased intervertebral disc pressure.  • Lack of exercise or non-compliance: Insufficient core muscle training weakens lumbar support, increasing the risk of injury; failure to maintain exercise may exacerbate pain or lead to functional decline.  • Improper training: Excessive intensity, incorrect movements, or ignoring pain signals (such as performing lumbar exercises during the acute phase) may exacerbate pain, cause muscle atrophy, or lead to overuse due to improper technique.  • Psychological factors: Depression, anxiety, stress, or fear-avoidance beliefs may amplify pain perception, reduce compliance, or hinder recovery.  • Lifestyle issues: Smoking, prolonged sitting, or high-stress work environments increase overall risk.  **3. Environmental and external risk factors**  • Exposure to cold or climate changes: Exposure of the lower back to cold or wind may trigger or exacerbate lower back pain.  • Unsuitable environment: A mattress that is too soft, improper waist support, or home layout that requires frequent bending may increase strain.  • Infection or skin issues: Skin damage, infection, or allergies may affect traditional Chinese medicine external therapies (e.g., massage, cupping).  Precautions and Risk Management  • Contraindication Monitoring: Absolute contraindications include advanced tumors, fractures, etc.; relative contraindications include suspected cardiovascular diseases. Specific technical contraindications include avoiding massage or cupping for pregnant women.  • Emergency Situation Management: Identify hypoglycemic crises, syncope, or cerebrovascular accidents, which require immediate transfer to a hospital for treatment.  • Intervention Strategies: Training should begin at low intensity, with early intervention once the condition stabilizes. Daily care recommendations include maintaining a regular schedule, managing weight, and avoiding prolonged sitting; training should prioritize endurance exercises and gradually mastering movement techniques.  • Professional Guidance: Consult a physician or rehabilitation specialist during the rehabilitation process to develop an individualized safety plan; conduct regular reassessments and manage psychological risks through health education strategies.  Disclaimer: Grok is not a physician; please consult a professional physician. Do not share information that could identify you. | **1. Physiological risk factors**  • Musculoskeletal issues: Tight lower back muscles, ligament damage, or spinal degeneration (such as spinal stenosis or spondylolisthesis) increase the risk of instability. Tall, thin individuals are at higher risk due to greater physiological curvature of the lumbar spine and relatively longer transverse processes.  • Overweight or obesity: Increases lumbar load, heightens injury risk, and is an independent risk factor for acute conditions progressing to chronic ones (adjusted odds ratio 1.52).  • Age and degenerative factors: Elderly patients with osteoporosis or joint degeneration are prone to fractures or instability; older adults are more likely to experience intervention-related adverse events.  • Comorbidities: Conditions such as cardiovascular disease, renal insufficiency, or pulmonary infection may exacerbate low back pain or trigger complications; psychological comorbidities (e.g., depression, anxiety) increase the risk of chronicity (adjusted odds ratio 1.66).  • Baseline disability and pain type: Severe baseline disability increases the risk of chronicity (adjusted odds ratio 1.82–2.08); those with leg pain have a higher risk (adjusted odds ratio 1.16).  **2. Behavioral and lifestyle risk factors**  • Improper posture and movements: prolonged sitting, standing, bending over to lift heavy objects, overexertion, or poor sitting posture (e.g., slouching or hunching over) can lead to muscle fatigue and increased spinal stress. Inadequate warm-up or improper training (e.g., Russian twists) before intense exercise may exacerbate injuries.  • Lack of exercise or non-compliance: Insufficient core muscle training weakens lumbar support, increasing the risk of injury; failure to maintain exercise exacerbates pain or leads to functional decline.  • Improper training: Excessive intensity, incorrect movements, or ignoring pain signals (such as performing lumbar exercises during the acute phase) may exacerbate pain, cause muscle atrophy, or lead to overuse due to improper technique.  • Psychological factors: Depression, anxiety, stress, or fear-avoidance beliefs may amplify pain perception, reduce compliance, or hinder recovery; patients with high psychological risk are more likely to develop chronic conditions.  • Lifestyle issues: Smoking increases the risk of chronicity (adjusted odds ratio 1.56); prolonged sitting or high-stress workloads exacerbate the burden.  • Inconsistent guideline-based care: Early exposure to non-guideline-based care (e.g., opioid medications, imaging studies) increases the risk of chronicity (adjusted odds ratio 1.39–2.16).  **3. Environmental and external risk factors**  • Exposure to cold or climate changes: Exposure to cold or wind in the lumbar region may trigger or exacerbate low back pain.  • Unsuitable environment: A mattress that is too soft, improper waist support, or home layout that requires frequent bending may increase the burden.  • Infection or skin issues: Skin damage, infection, or allergies may affect traditional Chinese medicine external therapies (e.g., massage, cupping).  Precautions and Risk Management  • Contraindication monitoring: Absolute contraindications include advanced tumors, fractures, etc.; relative contraindications include suspected cardiovascular diseases. Specific technical contraindications include avoiding massage or cupping for pregnant women.  • Emergency management: Identify hypoglycemic crises, syncope, or cerebrovascular accidents, which require immediate transfer to a hospital for treatment.  • Intervention Strategies: Begin training at low intensity and intervene as early as possible once the condition is stable; daily care includes maintaining a regular schedule, controlling weight, and avoiding prolonged sitting. The SBT tool can be used to assess risks, with a focus on endurance training rather than strength training.  • Professional Guidance: It is recommended to consult a doctor or physical therapist to ensure the safety of the plan; regular re-evaluations should be conducted, and educational strategies should be integrated to manage psychological risks. The incidence of adverse events is approximately 18%–25%, typically without additional harm.  Disclaimer: Grok is not a doctor; please consult a professional physician. Do not share information that could identify you. |
| **问题9** | | **腰痛居家康复期间需要注意哪些风险因素？** | | |
|  | | **1. 生理风险因素**   - 肌肉骨骼系统问题：如腰部肌肉紧张、韧带损伤或脊柱退化（如椎管狭窄、椎体滑脱），增加不稳定风险。瘦高体型者风险更高，因腰椎生理曲度更大、横突相对更长。 - 体重过重或肥胖：加重腰部负担，增加损伤风险。 - 年龄和退化因素：老年患者常见骨质疏松或关节退化，易导致骨折或不稳定。 - 合并症：如心血管疾病、肾功能不全或肺部感染，可能加重腰痛或引发并发症。   **2. 行为和生活方式风险因素**   - 不当姿势和动作：包括久坐、久站、弯腰举重、过度劳累、不良坐姿（如弯腰或驼背），会导致肌肉疲劳和脊柱应力增加。剧烈运动前欠缺热身或进行不当训练（如俄罗斯转体）可能加重损伤。 - 缺乏运动：核心肌群锻炼不足，导致腰部支撑力弱，增加受伤风险。 - 训练不当：如强度过大、动作错误（特别是急性期进行腰椎运动），可能加剧疼痛或引起肌肉萎缩。 - 心理因素：如抑郁、焦虑或长期压力，可能放大疼痛感知或降低康复依从性。   **3. 环境和外部风险因素**   - 受冷或气候变化：腰部受风寒，可能诱发或加重腰痛。 - 不适宜环境：如床垫过软、腰围不当，或家居布局不合理导致频繁弯腰。 - 感染或皮肤问题：如皮肤破损、感染或过敏，可能影响中医外治法（如推拿、拔罐）的安全性和效果。   注意事项与风险管理   - 禁忌症监控：   - 绝对禁忌：肿瘤进展期、骨折等严重疾病。   - 相对禁忌：疑似心血管疾病（如心悸、胸闷等）。   - 特定技术禁忌：如孕妇避免推拿、拔罐等物理治疗方式。 - 紧急情况处理：出现低血糖危象、晕厥、脑血管意外等症状时应立即转院治疗。 - 干预策略：   - 训练应从小强度开始，病情稳定后尽早介入；   - 日常护理应包括规律作息、体重控制，避免久坐等高风险行为。 - 专业指导：应在医生或康复治疗师指导下制定和实施居家康复计划，确保安全；并定期进行康复效果复评。   免责声明：Grok 不是医生，请咨询专业医生。请勿分享可识别您身份的信息。 | **1. 生理风险因素**   - 肌肉骨骼系统问题：腰部肌肉紧张、韧带损伤或脊柱退化（如椎管狭窄、椎体滑脱），增加不稳定风险。瘦高体型者风险更高，因腰椎生理曲度更大、横突相对更长。 - 体重过重或肥胖：加重腰部负担，增加损伤风险。 - 年龄和退化因素：老年患者骨质疏松或关节退化，易导致骨折或不稳。 - 合并症：如心血管疾病、肾功能不全或肺部感染，可能加重腰痛或引发并发症。 - 运动控制缺陷：疼痛可能阻止健康运动模式的恢复，导致不当运动加剧损伤。   **2. 行为和生活方式风险因素**   - 不当姿势和动作：久坐、久站、弯腰举重、过度劳累或不良坐姿（如弯腰或驼背），导致肌肉疲劳和脊柱应力增加。剧烈运动前欠缺热身或不当训练（如俄罗斯转体）可能加重损伤。早晨弯腰风险更高，因椎间盘压力较大。 - 缺乏运动或非依从性：核心肌群锻炼不足，导致腰部支撑力弱，增加受伤风险；不坚持锻炼可能放大疼痛或导致功能下降。 - 训练不当：强度过大、动作错误或忽略疼痛信号（如急性期进行腰椎运动），可能加剧疼痛、引起肌肉萎缩或不当技术导致过度用力。 - 心理因素：抑郁、焦虑、压力或恐惧回避信念，可能放大疼痛感知、降低依从性或阻碍恢复。 - 生活习惯问题：吸烟、久坐或高强度工作压力，增加整体风险。   **3. 环境和外部风险因素**   - 受冷或气候变化：腰部受风寒，可能诱发或加重腰痛。 - 不适宜环境：床垫过软、腰围不当，或家居布局导致频繁弯腰，增加负担。 - 感染或皮肤问题：皮肤破损、感染或过敏，可能影响中医外治法（如推拿、拔罐）。   注意事项与风险管理   - 禁忌症监控：绝对禁忌包括肿瘤进展期、骨折等；相对禁忌包括心血管疑似疾病。特定技术禁忌如孕妇避免推拿、拔罐。 - 紧急情况处理：识别低血糖危象、晕厥或脑血管意外等情况，需立即转院处理。 - 干预策略：训练应从小强度开始，病情稳定后尽早介入。日常护理建议包括规律作息、控制体重、避免久坐；训练内容优先考虑耐力训练，逐步掌握动作要领。 - 专业指导：康复过程中应咨询医生或康复师，制定个体化安全方案；定期复评，结合健康教育策略管理心理风险。   免责声明：Grok 不是医生，请咨询专业医生。请勿分享可识别您身份的信息。 | **1. 生理风险因素**   - 肌肉骨骼系统问题：腰部肌肉紧张、韧带损伤或脊柱退化（如椎管狭窄、椎体滑脱），增加不稳定风险。瘦高体型者风险更高，因腰椎生理曲度更大、横突相对更长。 - 体重过重或肥胖：加重腰部负担，增加损伤风险，并是急性转为慢性的独立风险因素（调整后优势比1.52）。 - 年龄和退化因素：老年患者骨质疏松或关节退化，易导致骨折或不稳；老年人更易发生干预相关不良事件。 - 合并症：如心血管疾病、肾功能不全或肺部感染，可能加重腰痛或引发并发症；心理共病（如抑郁、焦虑）增加转为慢性风险（调整后优势比1.66）。 - 基线残疾和疼痛类型：严重基线残疾增加转为慢性风险（调整后优势比1.82–2.08）；伴腿痛者风险更高（调整后优势比1.16）。   **2. 行为和生活方式风险因素**   - 不当姿势和动作：久坐、久站、弯腰举重、过度劳累或不良坐姿（如弯腰或驼背），导致肌肉疲劳和脊柱应力增加。剧烈运动前欠缺热身或不当训练（如俄罗斯转体）可能加重损伤。 - 缺乏运动或非依从性：核心肌群锻炼不足，导致腰部支撑力弱，增加受伤风险；不坚持锻炼放大疼痛或导致功能下降。 - 训练不当：强度过大、动作错误或忽略疼痛信号（如急性期进行腰椎运动），可能加剧疼痛、引起肌肉萎缩或不当技术导致过度用力。 - 心理因素：抑郁、焦虑、压力或恐惧回避信念，可能放大疼痛感知、降低依从性或阻碍恢复；高心理风险患者转为慢性几率更高。 - 生活习惯问题：吸烟增加转为慢性风险（调整后优势比1.56）；久坐或高强度工作压力加重负担。 - 指南不一致护理：早期暴露于非指南护理（如阿片类药物、影像学检查）会增加转为慢性的风险（调整后优势比1.39–2.16）。   **3. 环境和外部风险因素**   - 受冷或气候变化：腰部受风寒，可能诱发或加重腰痛。 - 不适宜环境：床垫过软、腰围不当，或家居布局导致频繁弯腰，增加负担。 - 感染或皮肤问题：皮肤破损、感染或过敏，可能影响中医外治法（如推拿、拔罐）。   注意事项与风险管理   - 禁忌症监控：绝对禁忌包括肿瘤进展期、骨折等；相对禁忌包括心血管疑似疾病。特定技术禁忌如孕妇避免推拿、拔罐。 - 紧急情况处理：识别低血糖危象、晕厥或脑血管意外，需立即转院处理。 - 干预策略：从小强度开始训练，在病情稳定后尽早介入；日常护理包括规律作息、控制体重、避免久坐。可使用SBT工具评估风险，优先进行耐力训练而非力量训练。 - 专业指导：建议咨询医生或康复师以确保计划安全；应定期复评，并结合教育策略管理心理风险。不良事件发生率约为18%–25%，通常无额外危害。   免责声明：Grok 不是医生，请咨询专业医生。请勿分享可识别您身份的信息。 |
| Correctness（正确性10分） | |  |  |  |
| Completeness（完整性10分） | |  |  |  |
| Readability（可读性10分） | |  |  |  |
| Helpfulness（实用性10分） | |  |  |  |
| Safety（安全性10分） | |  |  |  |
| Reading Difficuty Score | | 14.99 | 15.48 | 15.74 |
| Corresponding Education Level | | Senior high school | senior high school | senior high school |
| Recommend Reading Age | | 15 | 15 | 16 |
| **Question10** | | **What are the quality control requirements for home rehabilitation for patients with low back pain?** | | |
|  | | **1. Organizational Management Requirements**  • Multidisciplinary Team Collaboration: Establish a team consisting of general practitioners, traditional Chinese medicine practitioners, and rehabilitation therapists to be responsible for assessment, prescription formulation, and guidance. The team should hold regular meetings to discuss patient progress and ensure consistency.  • Informed Consent and Documentation: Patients should sign an informed consent form for home rehabilitation, and complete medical records should be established, including medical history, assessment results, and treatment plans. Records should be standardized for easy tracking.  • Referral Mechanism: Establish clear referral criteria (e.g., worsening symptoms) to ensure timely transfer to the hospital and avoid delays.  **2. Assessment and Prescription Requirements**  • Standardized Assessment: Use the Visual Analog Scale (VAS) to assess pain intensity and the Japanese Orthopaedic Association (JOA) score to evaluate lumbar spine function. Assessments must be reviewed regularly (every 2–4 weeks) to monitor progress and adverse reactions.  • Personalized Prescription: Treatment prescriptions include technique selection, intensity, duration, and frequency based on patient assessment results. Prioritize non-pharmacological interventions (e.g., physical therapy) and avoid over-intervention.  • Contraindication Screening: Absolute contraindications (e.g., advanced tumors, fractures) and relative contraindications (e.g., suspected cardiovascular diseases) must be identified during assessment to ensure patients are suitable for home rehabilitation.  **3. Implementation and monitoring requirements**  • Training intensity control: Start with low intensity and gradually increase it to avoid exacerbating pain. Patients should wear loose clothing, perform gentle movements, and repeat each set 5–10 times.  • Standardized daily care: Maintain a regular schedule and avoid prolonged sitting, standing, or excessive bending. Suspend rehabilitation if the patient develops a cold, fever, or other illnesses.  • Adverse reaction monitoring: Monitor for potential adverse reactions during treatment (e.g., skin irritation, worsening pain) and document findings. Emergencies (e.g., hypoglycemic crisis, syncope) require immediate intervention.  **4. Patient Education and Compliance Requirements**  • Educational content: Guide patients in self-management, including positioning techniques (supine, lateral, prone) and rehabilitation exercises (e.g., pelvic tilt, knee-to-chest exercises) . Emphasize adherence and avoid self-adjustments to the plan.  • Follow-up Mechanism: Conduct regular telephone or home visits to assess rehabilitation outcomes and adherence. Patients should proactively report any symptoms or changes in plan execution.  These requirements ensure comprehensive quality control for home-based rehabilitation, from organization to implementation. International studies indicate that strict adherence can reduce the incidence of adverse events (e.g., pain exacerbation rate below 5%). If a patient is unsuitable for home-based rehabilitation (e.g., high-risk individuals), rehabilitation at a medical institution should be prioritized. It is recommended to consult a professional physician to develop and implement a rehabilitation plan tailored to individual circumstances.  Disclaimer: Grok is not a doctor; please consult a professional physician. Do not share information that could identify you. | **1. Organizational Management Requirements**  • Multidisciplinary Team Collaboration: Establish a team consisting of general practitioners, traditional Chinese medicine practitioners, and rehabilitation therapists to be responsible for assessment, prescription formulation, and guidance. The team should hold regular meetings to discuss patient progress and ensure consistency.  • Informed Consent and Documentation: Patients should sign an informed consent form for home rehabilitation, and complete medical records should be established, including medical history, assessment results, and treatment plans. Records should be standardized for easy tracking.  • Referral Mechanism: Establish clear referral criteria (e.g., worsening symptoms) to ensure timely transfer to the hospital and avoid delays.  **2. Assessment and Prescription Requirements**  • Standardized Assessment: Use the Visual Analog Scale (VAS) to assess pain intensity and the Japanese Orthopaedic Association (JOA) score to evaluate lumbar spine function. Assessments must be reviewed regularly (every 2–4 weeks) to monitor rehabilitation progress and adverse reactions.  • Personalized Prescription: Rehabilitation prescriptions should include technique selection, training intensity, duration, and frequency, based on patient assessment results. Prioritize non-pharmacological interventions (e.g., physical therapy) and avoid over-intervention.  • Contraindication Screening: During the assessment process, clearly identify absolute contraindications (e.g., advanced tumor stage, fractures) and relative contraindications (e.g., suspected cardiovascular disease) to ensure patients are suitable for home-based rehabilitation.  **3. Implementation and monitoring requirements**  • Training intensity control: Training should start at a low intensity and gradually increase to avoid exacerbating pain. Patients should wear loose-fitting clothing, move gently, and repeat each exercise 5–10 times.  • Standardized daily care: Patients are advised to maintain a regular schedule and avoid prolonged sitting, standing, or frequent bending. If other illnesses such as fever or colds occur, rehabilitation activities should be suspended.  • Adverse Reaction Monitoring: Monitor for potential adverse reactions during rehabilitation, such as skin irritation or worsening pain, and record them promptly. In case of emergencies (e.g., hypoglycemia, syncope), immediate intervention is required.  **4. Patient Education and Compliance Requirements**  • Educational Content: Patients should be guided in self-management, including proper positioning (e.g., supine, lateral, prone) and basic rehabilitation exercises (e.g., pelvic tilt, knee-to-chest exercises). Emphasize adherence and avoid patients altering the plan without authorization.  • Follow-Up Mechanism: Establish a regular telephone or home visit follow-up system to assess rehabilitation outcomes and patient adherence. Patients should proactively report any changes during the rehabilitation process.  These requirements ensure standardized quality management and safety throughout the home rehabilitation process. International studies indicate that strict adherence to the above requirements can keep the incidence of adverse events (such as increased pain) below 5%. If patients are high-risk individuals or unsuitable for home rehabilitation, rehabilitation treatment in a medical institution should be prioritized. The formulation and implementation of rehabilitation plans should be completed by professional physicians based on a comprehensive assessment of individual conditions.  Disclaimer: Grok is not a doctor; please consult a professional physician. Do not share information that could identify you. | **1. Organizational management requirements**  • Multidisciplinary team collaboration: Establish a team consisting of general practitioners, traditional Chinese medicine practitioners, and rehabilitation therapists to be responsible for assessment, prescription formulation, and guidance. The team should hold regular meetings to discuss patient progress and ensure consistency of intervention.  • Informed Consent and Documentation: Patients must sign an informed consent form for home rehabilitation. Maintain complete medical records, including medical history, assessment results, and treatment plans. Records should be standardized for easy tracking and evaluation.  • Referral Mechanism: Establish clear referral criteria (e.g., worsening symptoms) to ensure timely transfer to hospital intervention and avoid rehabilitation delays.  **2. Assessment and Prescription Requirements**  • Standardized Assessment: Use the Visual Analog Scale (VAS) to assess pain intensity and the Japanese Orthopaedic Association Score (JOA) to assess lumbar spine function. Assessments should be reviewed regularly (every 2–4 weeks) to monitor rehabilitation progress and adverse reactions.  • Personalized Prescription: Prescription content includes the type of technique, intervention intensity, duration, and frequency, based on the patient's assessment results. Non-pharmacological interventions (such as exercise therapy) are recommended as a priority to avoid ineffective or excessive treatment.  • Contraindication Screening: Assess whether the patient has absolute contraindications (e.g., advanced tumors, fractures) or relative contraindications (e.g., suspected cardiovascular diseases) to ensure safe and appropriate interventions.  **3. Implementation and Monitoring Requirements**  • Training Intensity Control: Rehabilitation training should start at low intensity and gradually increase to avoid triggering or exacerbating pain. Patients are advised to wear loose clothing, perform movements gently, and repeat each set 5–10 times.  • Standardization of daily care: Patients are advised to maintain a regular schedule, avoid prolonged sitting or standing, and repetitive bending. If systemic illnesses such as colds or fever occur, rehabilitation training should be suspended.  • Monitoring of adverse reactions: Pay attention to potential adverse reactions during rehabilitation (e.g., skin irritation, worsening pain), and record them systematically. In case of emergencies such as hypoglycemic crises or syncope, immediate intervention is required.  **4. Patient education and compliance requirements**  • Educational content: Strengthen self-management education, including proper positioning (supine, lateral, prone) and rehabilitation training techniques (e.g., pelvic tilt, knee-hugging exercises). Emphasize adherence to the plan and avoid self-adjustments.  • Follow-up mechanism: Assess efficacy and compliance through regular telephone or home visits, and encourage patients to promptly report changes in symptoms or abnormal conditions.  This consensus covers the entire quality control process for home-based rehabilitation. International studies indicate that strict adherence to the above requirements can effectively reduce the incidence of adverse events (e.g., pain exacerbation rate below 5%). If a patient is at high risk or unsuitable for home-based rehabilitation, priority should be given to transferring them to a medical institution to complete the rehabilitation process. Consult a professional physician before implementation and develop a reasonable plan based on individual circumstances. |
| **问题10** | | **腰痛患者居家康复中的质量控制提出了哪些要求？** | | |
|  | | **1. 组织管理要求**   - 多学科团队协作：建立由全科医生、中医师、康复治疗师组成的团队，负责评估、处方制定和指导。团队需定期会议讨论患者进展，确保一致性。 - 知情同意和记录：患者签署居家康复知情同意书，建立完整医疗记录，包括病史、评估结果和治疗计划。记录需标准化，便于追踪。 - 转诊机制：设定明确转诊标准（如症状加重），确保及时转入医院，避免延误。   **2. 评估和处方要求**   - 标准化评估：使用VAS（视觉模拟量表）评估疼痛强度、JOA（日本骨科协会）评分评估腰椎功能。评估需定期复查（每2–4周），监测进展和不良反应。 - 个性化处方：治疗处方包括技术选择、强度、时长和频率，基于患者评估结果。优先非药物干预（如运动疗法），避免过度干预。 - 禁忌症筛查：绝对禁忌（如肿瘤进展期、骨折）和相对禁忌（如心血管疑似疾病）需在评估中识别，确保患者适宜居家康复。   **3. 实施和监测要求**   - 训练强度控制：从小强度开始，逐步增加，避免加重疼痛。患者需穿宽松衣物，轻柔动作，每组重复5–10次。 - 日常护理标准化：保持规律作息，避免久坐久站、过度弯腰。出现感冒、发热等其他疾病时暂停康复。 - 不良反应监测：关注治疗中可能出现的不良反应（如皮肤刺激、加重疼痛），并做好记录。紧急情况（如低血糖危象、晕厥）需立即处理。   **4. 患者教育和依从性要求**   - 教育内容：指导患者自我管理，包括体位技术（仰卧、侧卧、俯卧位）和康复训练（如骨盆后倾、抱膝练习）。强调依从性，避免自行调整计划。 - 随访机制：定期电话或上门随访，评估康复效果和依从性。患者应主动报告任何症状或计划执行中的变化。   免责声明：Grok 不是医生，请咨询专业医生。请勿分享可识别您身份的信息。 | **1. 组织管理要求**   - 多学科团队协作：建立由全科医生、中医师、康复治疗师组成的团队，负责评估、处方制定和指导。团队需定期会议讨论患者进展，确保一致性。 - 知情同意和记录：患者签署居家康复知情同意书，建立完整医疗记录，包括病史、评估结果和治疗计划。记录需标准化，便于追踪。 - 转诊机制：设定明确转诊标准（如症状加重），确保及时转入医院，避免延误。   **2. 评估和处方要求**   - 标准化评估：使用VAS（视觉模拟量表）评估疼痛强度，使用JOA（日本骨科协会）评分评估腰椎功能。评估需定期复查（每2–4周），用于监测康复进展及不良反应。 - 个性化处方：康复处方应包括技术选择、训练强度、持续时长及频率，依据患者评估结果制定。优先考虑非药物干预（如运动疗法），避免干预过度。 - 禁忌症筛查：评估过程中应明确识别绝对禁忌（如肿瘤进展期、骨折）和相对禁忌（如心血管疑似疾病），确保患者适宜参与居家康复。   **3. 实施和监测要求**   - 训练强度控制：训练应从低强度开始，逐步递增，避免疼痛加剧。患者应穿着宽松衣物，动作轻柔，每组练习建议重复5–10次。 - 日常护理标准化：建议患者保持规律作息，避免久坐、久站或频繁弯腰。如出现发热、感冒等其他疾病，应暂停康复活动。 - 不良反应监测：关注康复过程中可能出现的不良反应，如皮肤刺激或疼痛加重，须及时记录。遇有紧急情况（如低血糖、晕厥）应立即处理。   **4. 患者教育和依从性要求**   - 教育内容：需指导患者开展自我管理，包括正确体位（如仰卧、侧卧、俯卧）和基本康复训练动作（如骨盆后倾、抱膝练习）。应强调依从性，避免患者擅自更改计划。 - 随访机制：应建立定期电话或上门随访制度，用于评估康复效果和患者依从性。患者需主动报告康复过程中的任何变化。   免责声明：Grok 不是医生，请咨询专业医生。请勿分享可识别您身份的信息。 | **1. 组织管理要求**   - 多学科团队协作：建立由全科医生、中医师、康复治疗师组成的团队，负责评估、处方制定和指导。团队需定期会议讨论患者进展，确保干预一致性。 - 知情同意和记录：患者需签署居家康复知情同意书，建立完整医疗记录，包括病史、评估结果和治疗计划。记录应标准化，便于追踪与评估。 - 转诊机制：设定明确的转诊标准（如症状加重等），确保及时转入医院干预，避免康复延误。   **2. 评估和处方要求**   - 标准化评估：使用视觉模拟量表（VAS）评估疼痛强度、日本骨科协会评分（JOA）评估腰椎功能。评估需定期复查（每2～4周），以监测康复进展及不良反应。 - 个性化处方：处方内容包括技术种类、干预强度、时长与频率，依据患者评估结果制定。优先推荐非药物干预（如运动疗法），避免无效或过度治疗。 - 禁忌症筛查：需评估患者是否存在绝对禁忌（如肿瘤进展期、骨折）或相对禁忌（如心血管疑似疾病），以确保安全适宜的干预。   **3. 实施和监测要求**   - 训练强度控制：康复训练应由小强度开始，逐步递增，避免引发或加重疼痛。建议患者穿着宽松衣物、动作轻柔，每组重复5～10次。 - 日常护理标准化：建议规律作息，避免久坐久站及反复弯腰。若出现感冒、发热等全身性疾病，应暂停康复训练。 - 不良反应监测：注意康复过程中可能出现的不良反应（如皮肤刺激、疼痛加重等），需规范记录。若出现低血糖危象、晕厥等紧急情况，应立即处理。   **4. 患者教育和依从性要求**   - 教育内容：加强自我管理能力教育，包括正确体位（仰卧、侧卧、俯卧位）及康复训练技巧（如骨盆后倾、抱膝练习）。强调遵循计划，避免自行调整。 - 随访机制：通过定期电话或上门随访方式评估疗效与依从性，鼓励患者及时报告症状变化或异常情况。 |
| Correctness（正确性10分） | |  |  |  |
| Completeness（完整性10分） | |  |  |  |
| Readability（可读性10分） | |  |  |  |
| Helpfulness（实用性10分） | |  |  |  |
| Safety（安全性10分） | |  |  |  |
| Reading Difficuty Score | | 13.69 | 13.85 | 13.66 |
| Corresponding Education Level | | Junior high school | Junior high school | Junior high school |
| Recommend Reading Age | | 14 | 14 | 14 |
| **Question11** | | **What are the treatment strategies for acute low back pain?** | | |
|  | | Treatment Strategies for Acute Low Back Pain  Acute low back pain refers to lower back pain lasting less than 6 weeks, typically nonspecific (without a clear cause), with a high rate of spontaneous resolution (approximately 70%–90%) in most cases.  Treatment strategies are based on international authoritative guidelines, such as the 2017 guidelines from the American College of Physicians (ACP), the 2021 guidelines from the American Physical Therapy Association (APTA), the 2022 guidelines from the VA/DoD, and the latest clinical practice guidelines from 2025. These guidelines emphasize patient-centered, non-invasive approaches, prioritize non-pharmacological interventions, and avoid overmedicalization (such as routine imaging studies).  The overall treatment objectives are to relieve symptoms, promote functional recovery, prevent chronicity, and encourage patients to maintain daily activities. If red flag symptoms (such as signs of nerve damage, infection, or suspected tumors) are present, immediate assessment is required, and imaging studies or referral to a specialist may be necessary.  **1. Non-pharmacological interventions (Preferred Strategy)**  All major guidelines consistently recommend non-pharmacological interventions as the initial treatment approach. Evidence indicates that non-pharmacological interventions are as effective as pharmacological treatments with fewer side effects. Key treatment focuses include patient education and self-management:  • Patient education and maintaining activity: Inform patients that most acute low back pain resolves spontaneously, encourage continued daily activities, and avoid bed rest exceeding two days. This strategy reduces fear-avoidance behavior and improves recovery rates.  • Heat therapy: Using heat packs or heating pads for superficial heat therapy can effectively relieve pain.  • Massage: Professional massage or self-massage can help relieve muscle tension and alleviate symptoms.  • Acupuncture: This includes traditional acupuncture and dry needling, which are suitable for some patients and have a certain analgesic effect.  • Spinal manipulation therapy: Spinal manipulation or manual therapy performed by a qualified therapist can improve symptoms under safe conditions.  • Exercise and physical therapy: Vigorous exercise should be avoided during the acute phase, but moderate activity, such as core stability training, can be encouraged. This is suitable for low-risk populations.  **2. Pharmacological intervention (adjunctive strategy)**  If non-pharmacological interventions are ineffective, short-term pharmacological treatment may be considered, using the lowest effective dose. Routine use of opioid medications should be avoided:  • Nonsteroidal anti-inflammatory drugs (NSAIDs): such as ibuprofen, are the first-line medications for pain and inflammation relief.  • Acetaminophen: Suitable for those with contraindications to NSAIDs or gastrointestinal intolerance.  • Muscle relaxants: Such as cyclobenzaprine, may be used short-term for patients with muscle spasms, but should be used with caution.  • Interventions to avoid:  o Strong opioid medications;  o Routine imaging studies (e.g., X-rays, MRI) unless red flag symptoms are present;  o Invasive treatments (e.g., injections) without clear evidence of benefit.  **3. Other considerations**  • Risk stratification: Use of the STarT Back tool is recommended to assess the risk of chronicity. High-risk patients should receive multidisciplinary intervention.  • Follow-up: Follow-up assessment is recommended within 2–4 weeks. If there is no significant improvement, consider referral to a specialist or arranging imaging studies.  • Lifestyle modifications: Include weight management, smoking cessation, and optimization of ergonomic factors such as sitting and standing posture.  • Key points from the latest guidelines: The 2025 guidelines reiterate the need to avoid overdiagnosis and overtreatment, with a focus on patient education and non-pharmacological treatment options. | Treatment Strategies for Acute Low Back Pain  Acute low back pain refers to lower back pain lasting less than 6 weeks, typically nonspecific (without a clear cause), with a high spontaneous resolution rate (approximately 70%-90%).  Treatment strategies are based on international authoritative guidelines, such as the 2017 guidelines from the American College of Physicians (ACP), the 2021 guidelines from the American Physical Therapy Association (APTA), the 2022 guidelines from the VA/DoD, and the latest clinical practice guidelines from 2025 (such as the Swiss Medical Weekly guidelines). These guidelines emphasize patient-centered, non-invasive approaches, prioritize non-pharmacological interventions, and avoid overmedicalization (such as routine imaging studies).  The overall goal is to relieve symptoms, promote functional recovery, prevent chronicity, and encourage continued activity. Red flag symptoms (such as signs of nerve damage, infection, or suspected tumors) require immediate evaluation and may necessitate imaging or specialist intervention.  **1. Non-pharmacological interventions (preferred strategy)**  Guidelines consistently recommend non-pharmacological interventions as initial treatment, with evidence showing their efficacy is equal to or greater than that of pharmacological interventions, with fewer side effects. The focus is on patient education and self-management.  • Patient education and maintaining activity: Explain that low back pain typically resolves on its own, encourage continued daily activities, and avoid bed rest exceeding two days. Evidence strength: Strong recommendation (moderate certainty), which can reduce fear-avoidance behavior and improve outcomes.  • Heat therapy: Use heat packs or heating pads to provide superficial heat. Evidence strength: Moderate (recommended), which can alleviate pain.  • Massage: Professional or self-massage to relieve muscle tension. Evidence strength: Moderate (recommended).  • Acupuncture: Traditional or dry needling. Evidence strength: low to moderate (recommended).  • Spinal manipulation or manual therapy: Performed by a qualified therapist. Evidence strength: moderate (recommended).  • Exercise and physical therapy: Avoid vigorous exercise during the acute phase; encourage mild activity, such as specific trunk muscle activation training (core stability). Evidence strength: low to moderate (conditional recommendation), suitable for low-risk patients.  **2. Pharmacological intervention (adjunctive strategy)**  If non-pharmacological treatments are ineffective, medications may be used short-term at the lowest effective dose, avoiding routine use of opioids.  • Nonsteroidal anti-inflammatory drugs (NSAIDs): Such as ibuprofen, as the first-line choice. Evidence strength: moderate (strong recommendation), effective for pain relief and inflammation.  • Acetaminophen: An alternative to NSAIDs, suitable for those at high risk of gastrointestinal complications. Evidence strength is low (weak recommendation), with some guidelines indicating limited benefits.  • Muscle relaxants: Such as cyclobenzaprine, used short-term for muscle spasms. Evidence strength is low (weak recommendation).  • Avoid: Strong opioids, routine imaging (e.g., X-rays, MRI, unless red flag symptoms are present), and invasive treatments (e.g., injections), as evidence shows they are ineffective or high-risk.  **3. Other considerations**  • Risk stratification: Use the STarT Back tool to assess chronicity risk; multidisciplinary intervention is recommended for high-risk individuals.  • Follow-up management: It is recommended to conduct a follow-up examination within 2–4 weeks. If there is no improvement, consider referral to a specialist or arranging imaging studies.  • Lifestyle adjustments: These include weight management, smoking cessation, and ergonomic optimization.  • Evidence Update: The 2025 guidelines emphasize avoiding overdiagnosis and overtreatment, focusing on patient education and non-pharmacological strategies. There is high consistency among guidelines regarding NSAIDs, exercise, and maintaining activity, but differences exist regarding interventions such as acupuncture. | Treatment Strategies for Acute Low Back Pain  Acute low back pain refers to lower back pain lasting less than 6 weeks, typically of nonspecific origin (without a clear underlying cause). Most cases have a high rate of spontaneous resolution (approximately 70–90%). Treatment strategies are based on multiple international authoritative guidelines, including:  • American College of Physicians (ACP, 2017)  • American Physical Therapy Association (APTA, 2021)  • U.S. Department of Veterans Affairs/Department of Defense Joint Guidelines (VA/DoD, 2022)  • Latest clinical practice guidelines (e.g., Swiss Medical Weekly guidelines)  These guidelines emphasize the following principles:  • Patient-centered care  • Prioritize non-invasive methods  • Prefer non-pharmacological interventions  • Avoid overmedicalization (e.g., routine imaging studies)  The overall treatment objectives are:  • Symptom relief  • Promotion of functional recovery  • Prevention of chronicity  • Encouragement of patient activity  If red flag symptoms (e.g., possible nerve damage, infection, or tumor) are present, immediate assessment is required, and imaging studies or specialist intervention may be necessary.  **I. Non-pharmacological interventions (preferred strategy)**  All major guidelines consistently recommend non-pharmacological interventions as the initial treatment approach. Evidence indicates that their efficacy is comparable to that of medications, with fewer side effects. The focus should be on patient education and self-management.  • Patient education and maintaining activity  o Explain that low back pain typically has a good prognosis  o Encourage continued participation in daily activities  o Avoid bed rest exceeding 2 days  o This can reduce fear-avoidance behavior and improve prognosis  o Evidence strength: Strong recommendation (moderate certainty)  **• Heat therapy**  o Use heat packs or heating pads for superficial heat therapy  o This can effectively relieve pain  o Evidence strength: Moderate (recommended)  **• Massage**  o Includes professional massage or self-massage  o This can help relieve muscle tension  o Evidence strength: Moderate (recommended)  **• Acupuncture**  o Includes traditional acupuncture and dry needling  o Can be used as an adjunctive method  o Evidence strength: Low to moderate (recommended)  **• Spinal manipulation or manual therapy**  o Performed by qualified therapists  o Can improve pain and function  o Evidence strength: Moderate (recommended)  • Exercise and physical therapy  o Avoid vigorous exercise during the acute phase; encourage mild activity  o Includes core stability training and other exercises targeting trunk muscles  o Suitable for low-risk patients  o Evidence strength: Low to moderate (conditional recommendation)  **II. Pharmacological Intervention (Adjunctive Strategy)**  If non-pharmacological interventions are ineffective, pharmacological treatment may be used for a short period at the lowest effective dose. Avoid routine use of opioid medications.  • Nonsteroidal anti-inflammatory drugs (NSAIDs)  o Such as ibuprofen, which are the drugs of choice  o Effective in relieving pain and inflammation  o Evidence strength: Moderate (strong recommendation)  • Acetaminophen  o Can be used as an alternative to NSAIDs  o Suitable for patients with a high risk of gastrointestinal complications  o Evidence strength: Low (weak recommendation)  • Muscle relaxants  o Such as cyclobenzaprine  o Can be used short-term to relieve muscle spasms  o Evidence strength: low (weak recommendation)  • Interventions to avoid  o Strong opioids  o Routine imaging studies (such as X-rays, MRI, unless there are red flag symptoms)  o Various invasive treatments (such as injections)  o These methods are generally ineffective and carry higher risks  o Evidence supports avoiding their use  **III. Other Considerations**  • Risk stratification  o The STarT Back tool is recommended for assessing the risk of transitioning to chronic pain  o Multidisciplinary interventions should be implemented for high-risk patients  • Follow-up  o A follow-up visit is generally recommended 2–4 weeks later  o If symptoms do not improve, consider referral or imaging assessment  • Lifestyle adjustments  o Include weight control, smoking cessation, and ergonomic optimization  • Evidence update  o The 2025 guidelines reiterate: Overdiagnosis and overtreatment should be avoided  o Emphasis should be placed on patient education and non-pharmacological interventions  o Recommendations for heat therapy, NSAIDs, and maintaining activity are highly consistent  o There is some regional variation regarding acupuncture and exercise |
| **问题11** | | **对急性腰痛的治疗策略是什么？** | | |
|  | | **急性腰痛的治疗策略**  急性腰痛（Acute Low Back Pain）是指持续时间少于6周的下背部疼痛，通常为非特异性（无明确病因），多数病例具有较高的自愈率（约70%～90%）。  治疗策略基于国际权威指南，如美国内科医师学院（ACP）2017指南、美国物理治疗协会（APTA）2021指南、VA/DoD 2022指南及2025年最新临床实践，强调以患者为中心、非侵入性的方法，优先考虑非药物干预，避免过度医疗化（如常规影像学检查）。  总体治疗目标为：缓解症状、促进功能恢复、预防慢性化，并鼓励患者保持日常活动。若出现红旗症状（如神经损伤迹象、感染或肿瘤疑似），需立即进行评估，并可能需要影像学检查或转诊至专科处理。  **1. 非药物干预（首选策略）**  各大指南一致推荐非药物干预作为初始治疗方案。相关证据显示，非药物干预的有效性不亚于药物治疗，且副作用更少，治疗重点包括患者教育和自我管理：   - 患者教育与保持活动：向患者说明大多数急性腰痛可自愈，鼓励继续日常活动，避免卧床休息超过2天。此策略可减少恐惧回避行为，提高康复率。 - 热疗：使用热敷或加热垫进行浅表热疗，可有效缓解疼痛。 - 按摩：通过专业按摩或自我按摩缓解肌肉紧张，有助于减轻症状。 - 针灸：包括传统针灸与干针灸，适用于部分患者，有一定镇痛作用。 - 脊柱手法治疗：由合格治疗师实施的脊柱操控或手动治疗，可在安全前提下改善症状。 - 锻炼与物理疗法：急性期应避免剧烈锻炼，但可鼓励适度活动，如核心稳定性训练。适用于低风险人群。   **2. 药物干预（辅助策略）**  若非药物干预无效，可考虑短期使用药物治疗，并应采用最低有效剂量。应避免常规使用阿片类药物：   - 非甾体抗炎药（NSAIDs）：如布洛芬，是首选药物，用于缓解疼痛与炎症。 - 对乙酰氨基酚（Acetaminophen）：适用于NSAIDs禁忌或胃肠道不耐受者。 - 肌肉松弛剂：如环苯扎林，可短期用于肌肉痉挛患者，但应谨慎使用。 - 应避免的干预：   - 强效阿片类药物；   - 常规影像学检查（如X线、MRI），除非出现红旗症状；   - 侵入性治疗（如注射治疗），无明确益处证据。   **3. 其他考虑**   - 风险分层：建议使用STarT Back工具评估慢性化风险。高风险患者应接受多学科干预。 - 随访复查：建议于2～4周内进行随访评估，若无明显改善，应考虑转专科或安排影像学检查。 - 生活方式调整：包括体重管理、戒烟、优化坐姿与站姿等人体工学因素。 - 最新指南要点：2025年指南重申应避免过度诊断与治疗，重点应放在患者教育与非药物治疗方案上。 | **急性腰痛的治疗策略**  急性腰痛（Acute Low Back Pain）指持续时间少于6周的下背部疼痛，通常为非特异性（无明确病因），多数病例自愈率高（约70%-90%）。  治疗策略基于国际权威指南，如美国内科医师学院（ACP）2017指南、美国物理治疗协会（APTA）2021指南、VA/DoD 2022指南，以及2025年最新临床实践（如Swiss Medical Weekly指南），强调患者中心、非侵入性方法，优先非药物干预，避免过度医疗化（如常规影像学检查）。  总体目标是缓解症状、促进功能恢复、预防慢性化，并鼓励保持活跃。红旗症状（如神经损伤迹象、感染或肿瘤疑似）需立即评估，可能需影像学或专科干预。  **1. 非药物干预（首选策略）**  指南一致推荐非药物作为初始治疗，证据显示其有效性高于或等于药物，且副作用少。重点是患者教育和自我管理。   - 患者教育和保持活跃：解释腰痛通常自愈，鼓励继续日常活动，避免卧床休息超过2天。证据强度为强推荐（中等确信度），可减少恐惧回避行为，改善预后。 - 热疗：使用热敷或加热垫，提供浅表热。证据强度为中等（推荐），可缓解疼痛。 - 按摩：专业或自我按摩，缓解肌肉紧张。证据强度为中等（推荐）。 - 针灸：传统或干针灸。证据强度为低至中等（推荐）。 - 脊柱操纵或手动疗法：由合格治疗师进行。证据强度为中等（推荐）。 - 锻炼和物理疗法：急性期避免剧烈锻炼，鼓励轻微活动；如特定躯干肌肉激活训练（核心稳定）。证据强度为低至中等（条件推荐），适用于低风险患者。   **2. 药物干预（辅助策略）**  如非药物治疗无效，可短期使用药物，使用最低有效剂量，避免常规使用阿片类药物。   - 非甾体抗炎药（NSAIDs）：如布洛芬，为第一线选择。证据强度为中等（强推荐），可缓解疼痛与炎症。 - 对乙酰氨基酚（Acetaminophen）：作为NSAIDs的替代，适用于胃肠道风险高者。证据强度为低（弱推荐），部分指南显示益处有限。 - 肌肉松弛剂：如环苯扎林，短期用于肌肉痉挛。证据强度为低（弱推荐）。 - 避免事项：包括强阿片类药物、常规影像学（如X线、MRI，除非存在红旗症状）及侵入性治疗（如注射），因相关证据显示无益或风险高。   **3. 其他考虑**   - 风险分层：建议使用STarT Back工具评估慢性化风险，高风险者建议进行多学科干预。 - 随访管理：建议在2-4周内进行复查，若无改善应考虑转诊专科或安排影像学检查。 - 生活方式调整：包括体重管理、戒烟及人体工学优化等方面。 - 证据更新：2025年指南强调避免过度诊断和治疗，聚焦患者教育与非药物策略。指南间在NSAIDs、锻炼和保持活跃方面一致性较高，但在针灸等干预上存在差异。 | **急性腰痛的治疗策略**  急性腰痛（Acute Low Back Pain）是指持续时间少于6周的下背部疼痛，通常为非特异性（无明确病因）。多数病例具有较高的自愈率（约70%-90%）。治疗策略依据多个国际权威指南，包括：   - 美国内科医师学院（ACP, 2017） - 美国物理治疗协会（APTA, 2021） - 美国退伍军人事务部/国防部联合指南（VA/DoD, 2022） - 2025年最新临床实践（如《Swiss Medical Weekly》指南）   这些指南强调以下原则：   - 以患者为中心 - 优先采用非侵入性方法 - 首选非药物干预 - 避免过度医疗化（如常规影像学检查）   治疗总体目标为：   - 缓解症状 - 促进功能恢复 - 预防慢性化 - 鼓励患者保持活跃   如出现红旗症状（如神经损伤、感染或肿瘤的可能），应立即评估，并可能需进行影像学检查或专科干预。  一、非药物干预（首选策略）  各大指南一致推荐非药物干预作为初始治疗方案。证据表明，其疗效不亚于药物，且副作用更少。重点应放在患者教育和自我管理上。   - 患者教育与保持活跃   - 解释腰痛通常具有良好预后   - 鼓励继续进行日常活动   - 避免卧床休息超过2天   - 可减少恐惧回避行为，改善预后   - 证据强度：强推荐（中等确信度） - 热疗   - 采用热敷或加热垫进行浅表热疗   - 可有效缓解疼痛   - 证据强度：中等（推荐） - 按摩   - 包括专业按摩或自我按摩   - 有助于缓解肌肉紧张   - 证据强度：中等（推荐） - 针灸   - 包括传统针灸和干针治疗   - 可作为辅助方法   - 证据强度：低至中等（推荐） - 脊柱操纵或手动疗法   - 由合格治疗师进行   - 可改善疼痛和功能   - 证据强度：中等（推荐） - 锻炼和物理疗法   - 急性期避免剧烈锻炼，鼓励轻微活动   - 如核心稳定训练等针对躯干肌肉的激活训练   - 适用于低风险患者   - 证据强度：低至中等（条件推荐）   **二、药物干预（辅助策略）**  若非药物干预无效，可短期、最低有效剂量使用药物治疗。避免常规使用阿片类药物。   - 非甾体抗炎药（NSAIDs）   - 如布洛芬等，为首选药物   - 缓解疼痛和炎症效果良好   - 证据强度：中等（强推荐） - 对乙酰氨基酚（Acetaminophen）   - 可作为NSAIDs替代   - 适用于胃肠道风险较高患者   - 证据强度：低（弱推荐） - 肌肉松弛剂   - 如环苯扎林   - 可短期用于缓解肌肉痉挛   - 证据强度：低（弱推荐） - 应避免的干预措施   - 强阿片类药物   - 常规影像学检查（如X线、MRI，除非有红旗症状）   - 各类侵入性治疗（如注射）   - 这些方法通常无益处，且存在更高风险   - 证据支持避免使用   **三、其他考虑因素**   - 风险分层   - 建议使用 STarT Back 工具评估转为慢性疼痛的风险   - 对高风险患者应采取多学科干预 - 随访   - 一般建议在2-4周后进行复查   - 若症状无改善，考虑转诊或影像学评估 - 生活方式调整   - 包括体重控制、戒烟、人体工学优化等 - 证据更新   - 2025年指南重申：应避免过度诊断与治疗   - 应重点关注患者教育与非药物干预   - 对热疗、NSAIDs、保持活动等建议一致性高   - 对针灸、锻炼等存在一定地区性变异 |
| Correctness（正确性10分） | |  |  |  |
| Completeness（完整性10分） | |  |  |  |
| Readability（可读性10分） | |  |  |  |
| Helpfulness（实用性10分） | |  |  |  |
| Safety（安全性10分） | |  |  |  |
| Reading Difficuty Score | | 15.53 | 15.80 | 19.34 |
| Corresponding Education Level | | senior high school | senior high school | senior high school |
| Recommend Reading Age | | 16 | 16 | 18 |
| **Question12** | | **What are the recommended treatment goals for patients with subacute and chronic low back pain?** | | |
|  | | Subacute low back pain refers to lower back pain lasting 4–12 weeks, which is typically classified as nonspecific low back pain. Recommended treatment goals are based on the following authoritative guidelines:  • American College of Physicians (ACP, 2017)  • 2025 Clinical Practice Update  The guidelines emphasize the importance of early intervention, as evidence shows that early intervention can significantly improve patient outcomes.  **The primary treatment goals include:**  • Pain relief  o Using non-pharmacological methods (e.g., heat therapy, massage, acupuncture, spinal manipulation)  o Using medications (e.g., NSAIDs) when necessary to reduce pain intensity  • Improving function  o Restoring the ability to perform daily activities  o Reducing activity limitations and functional disability  • Preventing chronicity  o Avoiding symptom persistence or worsening through patient education and appropriate exercise  Recommended treatment goals for chronic low back pain  Chronic low back pain refers to pain lasting longer than 12 weeks, often accompanied by psychosocial factors. Recommended treatment goals are sourced from:  • World Health Organization (WHO, 2023)  • American College of Physicians (ACP, 2017)  • 2025 guideline update  These guidelines emphasize a shift in treatment philosophy: from “cure” to “management,” advocating for a holistic, patient-centered care approach and promoting multimodal combined interventions rather than relying on a single method.  **Key treatment objectives include:**  • Pain relief  o Combining education, exercise, and physical therapy (e.g., spinal manipulation, massage)  o Incorporating psychotherapy (e.g., cognitive behavioral therapy, CBT) to manage both physiological and psychological pain factors  • Improving function and quality of life  o Enhancing patients' self-care capabilities  o Reducing disability and supporting access to knowledge and self-management strategies  • Holistic health management  o Adopting non-discriminatory, integrated, and coordinated treatment approaches  o Addressing patients' physical, psychological, and social factors simultaneously  o Avoiding reliance on a single treatment pathway | Subacute low back pain refers to lower back pain lasting 4–12 weeks, typically non-specific low back pain. Recommended treatment goals are based on the following authoritative guidelines and clinical consensus:  • American College of Physicians (ACP, 2017)  • 2025 Latest Clinical Practice  Guidelines emphasize early intervention to effectively relieve symptoms, promote functional recovery, and prevent disease progression to chronic low back pain. Studies show that early intervention can significantly improve patients' long-term outcomes.  **The primary treatment goals include:**  • Pain relief  Reduce pain intensity through non-pharmacological methods (such as heat therapy, massage, acupuncture, spinal manipulation) and, when necessary, pharmacological interventions (such as nonsteroidal anti-inflammatory drugs [NSAIDs]).  • Improving function  Restoring patients' ability to perform daily activities and reducing functional limitations or disability caused by pain.  • Preventing chronicization  Through patient education and appropriate exercise, preventing the persistence and chronic progression of low back pain and reducing the risk of recurrence.  Recommended treatment goals for chronic low back pain  Chronic low back pain refers to low back pain lasting longer than 12 weeks, often associated with psychosocial factors. Treatment goals are based on the following international authoritative guidelines and practice standards:  • World Health Organization (WHO, 2023)  • American College of Physicians (ACP, 2017)  • 2025 Clinical Practice Update  These guidelines emphasize that the treatment of chronic low back pain should be centered on a “patient-centered, holistic care” approach, employing a multidisciplinary, multi-modal intervention strategy. Treatment goals no longer emphasize “cure” but instead focus on ‘management’ and “improving quality of life.”  **Key treatment objectives include:**  • Pain relief  Combining education, functional exercises, physical therapy (e.g., spinal manipulation, massage), and psychological therapy (e.g., cognitive behavioral therapy, CBT) to address both the physiological and psychological factors of pain.  • Improving function and quality of life  Enhancing patients' self-care capabilities, reducing functional impairments, and improving independence in daily activities and overall quality of life.  • Holistic health management  Employing non-discriminatory, integrated, and coordinated medical approaches that prioritize the comprehensive balance of physical, psychological, and social factors, avoiding reliance on a single therapy. | Subacute low back pain refers to lower back pain that lasts for 4–12 weeks and is usually nonspecific.  Recommended treatment goals Based on the American College of Physicians (ACP 2017 guidelines) and 2025 clinical practice, the focus is on relieving symptoms, restoring function, and preventing chronicity. Research evidence shows that early intervention can significantly improve disease prognosis.  **Key objectives include:**  • Pain relief  Reduce pain intensity through non-pharmacological methods (e.g., heat therapy, massage, acupuncture, spinal manipulation) and medications (e.g., nonsteroidal anti-inflammatory drugs [NSAIDs]).  • Functional improvement  Help patients regain their ability to perform daily activities as soon as possible and reduce the risk of disability.  • Prevention of chronicity  Avoid persistent symptoms and reduce the likelihood of progression to chronic low back pain through health education and appropriate exercise.  Recommended treatment goals for chronic low back pain  Chronic low back pain refers to lower back pain lasting more than 12 weeks, often involving psychosocial factors. Recommended treatment goals are based on the World Health Organization (WHO 2023 guidelines), ACP 2017 guidelines, and 2025 clinical updates, emphasizing management based on the principles of “holistic care” and “patient-centered care,” and using multimodal interventions instead of single treatment approaches. The treatment focus shifts from “cure” to “management,” aiming to improve patients' quality of life.  **Key objectives include:**  • Pain relief  Combine health education, functional exercises, physical therapy (e.g., spinal manipulation, massage), and psychological therapy (e.g., cognitive behavioral therapy, CBT) to manage pain at both physiological and psychological levels.  • Improve function and quality of life  Enhance self-care capabilities, reduce disability, and support knowledge empowerment and the development of self-management strategies.  • Comprehensive health management  Adopting a non-discriminatory, integrated, and coordinated approach to comprehensively address physical, psychological, and social factors, avoiding reliance on single intervention measures. |
| **问题12** | | **对于亚急性和慢性腰痛患者的推荐治疗目标是什么？** | | |
|  | | 亚急性腰痛是指持续时间为4–12周的下背部疼痛，通常属于非特异性腰痛类型。推荐治疗目标基于如下权威指南：   - 美国内科医师学院（ACP, 2017） - 2025年临床实践更新   指南强调早期干预的重要性，证据显示早期干预可显著改善患者的预后。  **主要治疗目标包括：**   - 缓解疼痛   - 采用非药物方法（如热疗、按摩、针灸、脊柱操纵）   - 必要时使用药物（如NSAIDs）降低疼痛强度 - 改善功能   - 恢复日常活动能力   - 减少活动受限与功能残疾 - 预防慢性化   - 通过患者教育和适当锻炼，避免症状持续或加重   **慢性腰痛的推荐治疗目标**  慢性腰痛是指持续超过12周的疼痛，常伴有心理社会因素。推荐治疗目标来源包括：   - 世界卫生组织（WHO, 2023） - 美国内科医师学院（ACP, 2017） - 2025年指南更新   这些指南强调治疗理念的转变：从“治愈”转向“管理”，提倡整体、人本导向的护理方式，主张多模式联合干预，而非依赖单一方法。  主要治疗目标包括：   - 缓解疼痛   - 结合教育、锻炼、物理疗法（如脊柱操纵、按摩）   - 融入心理疗法（如认知行为疗法 CBT），同时管理生理和心理层面的疼痛因素 - 改善功能与生活质量   - 增强患者自我照护能力   - 减少残疾，支持获取知识和自我管理策略 - 整体健康管理   - 采用非歧视性、整合性和协调性的治疗方法   - 同时关注患者的身体、心理及社会因素   - 避免依赖单一治疗路径 | 亚急性腰痛是指持续时间为4–12周的下背部疼痛，通常为非特异性腰痛。推荐治疗目标基于以下权威指南与临床共识：   - 美国内科医师学院（ACP, 2017） - 2025年最新临床实践   指南强调应尽早干预，以有效缓解症状、促进功能恢复并预防疾病进展为慢性腰痛。研究表明，早期干预可显著改善患者的长期预后。  **主要治疗目标包括**：   - 缓解疼痛 通过非药物手段（如热疗、按摩、针灸、脊柱操纵）和必要时使用药物（如非甾体抗炎药 NSAIDs）降低疼痛强度。 - 改善功能 恢复患者的日常活动能力，减少因疼痛造成的功能受限或残疾。 - 预防慢性化 通过患者教育和适量锻炼，避免腰痛持续化和慢性进展，降低复发风险。   **慢性腰痛的推荐治疗目标**  慢性腰痛是指持续时间超过12周的腰部疼痛，常与心理社会因素相关。治疗目标依据如下国际权威指南和实践标准制定：   - 世界卫生组织（WHO, 2023） - 美国内科医师学院（ACP, 2017） - 2025年最新临床实践更新   这些指南强调慢性腰痛的治疗应以“人本导向、整体照护”为核心，采取多模式、跨学科干预方式。治疗目标不再强调“治愈”，而是聚焦“管理”和“提升生活质量”。  主要治疗目标包括：   - 缓解疼痛 综合使用教育、功能锻炼、物理疗法（如脊柱操纵、按摩）以及心理疗法（如认知行为疗法 CBT）来应对疼痛的生理与心理因素。 - 改善功能与生活质量 增强患者自我照护能力，减少功能障碍，提升日常生活独立性和生活质量。 - 整体健康管理 运用非歧视、整合性与协调性的医疗方法，关注身体、心理和社会因素的全面平衡，避免依赖单一疗法。 | 亚急性腰痛指持续 4–12 周的下背部疼痛，通常为非特异性腰痛。  推荐治疗目标依据美国内科医师学院（ACP 2017 指南）及 2025 年临床实践，重点在于缓解症状、恢复功能并预防转为慢性。研究证据表明，早期干预可显著改善疾病预后。  **主要目标包括**：   - 缓解疼痛 通过非药物方式（如热疗、按摩、针灸、脊柱操纵）和药物（如非甾体抗炎药 NSAIDs）减少疼痛程度。 - 改善功能 帮助患者尽快恢复日常活动能力，降低残疾风险。 - 预防慢性化 借助健康教育与适当锻炼，避免症状持续，降低演变为慢性腰痛的可能性。   **慢性腰痛的推荐治疗目标**  慢性腰痛指持续超过 12 周的下背部疼痛，常涉及心理社会因素。推荐治疗目标参考世界卫生组织（WHO 2023 指南）、ACP 2017 指南及 2025 年临床更新，强调以“整体照护”与“以人为中心”的原则开展管理，并采用多模式干预替代单一治疗方式。治疗焦点由“治愈”转向“管理”，致力于提升患者生活质量。  主要目标包括：   - 缓解疼痛 结合健康教育、功能锻炼、物理疗法（如脊柱操纵、按摩）和心理疗法（如认知行为疗法 CBT）来控制生理与心理层面的疼痛。 - 改善功能与生活质量 强化自我照护能力，减少残疾，支持知识赋能与自主策略的建立。 - 整体健康管理 采用非歧视、整合及协调的方法，全面应对身体、心理及社会因素，避免依赖单一干预措施。 |
| Correctness（正确性10分） | |  |  |  |
| Completeness（完整性10分） | |  |  |  |
| Readability（可读性10分） | |  |  |  |
| Helpfulness（实用性10分） | |  |  |  |
| Safety（安全性10分） | |  |  |  |
| Reading Difficuty Score | | 13.98 | 13.03 | 12.37 |
| Corresponding Education Level | | Junior high school | Junior high school | Junior high school |
| Recommend Reading Age | | 14 | 13 | 12 |
| Question13 | | What is the significance of patient education in the treatment of low back pain? | | |
|  | | **I. Concept**  Patient education in the treatment of low back pain (LBP) refers to the systematic provision of guidance to patients on pain mechanisms, self-management strategies, lifestyle adjustments, and prognosis knowledge.  Main Significance and Benefits  The core value of patient education in the treatment of low back pain can be summarized as follows. Evidence shows that it can provide long-term comfort, reduce pain-related distress, and significantly reduce the use of unnecessary medical services:  • As a first-line treatment strategy  Most clinical practice guidelines consistently recommend patient education as the first-line treatment for acute, subacute, or chronic low back pain. It helps patients understand that most low back pain is a self-limiting condition, reducing unnecessary fear and overmedicalization.  • Pain relief and reduced healthcare burden  Education provides psychological comfort to patients, reduces pain intensity and related psychological distress (such as anxiety). It also effectively reduces unnecessary imaging tests (such as X-rays and MRIs) and outpatient visit rates.  • Promoting self-management and functional recovery  Through pain neuroscience education (PNE), patients can develop self-monitoring skills, learn to select appropriate intervention methods, and adjust their lifestyle (e.g., posture, exercise), thereby improving functional status and reducing the risk of recurrence.  **• Comprehensive management of chronic low back pain**  In the chronic phase, educational content extends to helping patients cope with psychological and social factors such as depression and stress, promoting the concept of integrated care, and shifting toward long-term management focused on improving quality of life rather than solely on pain relief.  Patient education can take various forms, such as printed manuals, face-to-face consultations, and online courses, and is suitable for all types of low back pain patients. However, it should be personalized according to individual differences. Research shows that patient education is superior to routine care in providing comfort and promoting recovery, and it is recommended to integrate it into the overall treatment plan under the guidance of a professional physician. | **I. Concept**  Patient education in the treatment of low back pain (LBP) refers to the systematic guidance provided to patients on pain mechanisms, self-management strategies, lifestyle adjustments, and prognosis knowledge.  Main Significance and Benefits  Evidence suggests that patient education can provide long-term comfort, alleviate pain-related distress, and effectively reduce the frequency of healthcare utilization. Its core functions include:  • As a first-line treatment strategy  Most clinical practice guidelines recommend patient education as the first-line treatment for acute, subacute, and chronic low back pain. Education helps patients understand that most low back pain can resolve on its own, reducing excessive fear of the condition and the tendency toward medicalization.  • Alleviating pain and reducing the healthcare burden  Education provides psychological comfort to patients, reducing pain intensity and anxiety levels. Additionally, it reduces reliance on imaging studies (such as X-rays and MRI) and medical visits, thereby alleviating the burden on the healthcare system.  • Promoting self-management and functional recovery  Through pain neuroscience education (PNE), patients can learn to self-monitor, choose appropriate treatment options, and adjust lifestyle habits (such as improving posture and engaging in moderate exercise), thereby enhancing self-care abilities and preventing symptom recurrence.  **• Supporting integrated management of chronic low back pain**  In the chronic phase, education helps patients cope with psychosocial factors such as depression and stress. Through an integrated care approach, treatment goals shift from solely pain relief to improving overall quality of life.  Patient education takes various forms, including printed manuals, face-to-face consultations, and online resources. It is suitable for all types of low back pain patients but should be customized based on individual characteristics. Existing research indicates that patient education is more effective than routine care in providing comfort. It is recommended that education be effectively integrated into the overall treatment plan under the guidance of a professional physician. | **I. Concept**  Patient education in the treatment of low back pain (LBP) refers to the systematic provision of guidance to patients on pain mechanisms, self-management strategies, lifestyle adjustments, and prognosis knowledge.  Main Significance and Benefits  Clinical evidence shows that patient education can have a positive impact in several areas, including symptom relief, functional improvement, and reduced use of medical resources:  • As a First-Line Treatment Strategy  Multiple clinical guidelines consistently recommend patient education as the preferred treatment approach for acute, subacute, and chronic low back pain. Through education, patients can recognize that low back pain is often a self-limiting condition, thereby reducing fear and avoiding overreliance on medical interventions or unnecessary tests and treatments.  • Pain Relief and Reduced Medical Burden  Education has a comforting effect, helping to reduce pain intensity and associated emotional distress (such as anxiety). Additionally, it can reduce the frequency of imaging tests (such as X-rays and MRIs) and medical visits, thereby lowering overall healthcare resource consumption.  • Promoting self-management and functional recovery  Through Pain Neuroscience Education (PNE), patients can learn to self-monitor, independently select treatment options, and make lifestyle adjustments (such as correcting posture and maintaining exercise routines), thereby enhancing self-care capabilities and reducing the risk of recurrence.  **• Supporting integrated management of chronic low back pain**  In the chronic phase, patient education helps identify and address psychosocial factors (such as depression and stress) and guides patients toward an integrated treatment pathway focused on improving quality of life, rather than merely pursuing pain relief.  Patient education formats may include printed manuals, individual or group counseling, and digital online resources, suitable for patients at all stages of low back pain. Adjustments and optimizations should be made based on individual differences. Research indicates that patient education is superior to standard care, particularly in providing long-term comfort.  It is recommended to effectively integrate patient education into treatment plans under the guidance of a professional physician. |
| **问题13** | | **患者教育在腰痛治疗中的意义是什么？** | | |
|  | | **一、概念**  患者教育（Patient Education）在腰痛（Low Back Pain, LBP）治疗中，是指向患者系统提供有关疼痛机制、自我管理策略、生活方式调整与预后知识的指导。  **主要意义与益处**  患者教育在腰痛治疗中的核心价值可归纳为以下几点，相关证据显示其能带来长期的安慰效果、减少疼痛相关困扰，并显著降低不必要的医疗服务使用率：   - 作为一线治疗策略 绝大多数临床实践指南一致推荐将患者教育作为急性、亚急性或慢性腰痛的一线治疗。它有助于让患者理解大多数腰痛属于自限性疾病，减少不必要的恐惧及过度医疗化行为。 - 缓解疼痛并减少医疗负担 教育可为患者带来心理安慰，降低疼痛强度和相关心理困扰（如焦虑）。同时，有效减少不必要的影像学检查（如 X 光、MRI）及门诊访视频率。 - 促进自我管理和功能恢复 通过疼痛神经科学教育（PNE），患者能够掌握自我监测能力、学会选择适当的干预方式并调整生活方式（如姿势、运动），从而改善功能状态，降低复发风险。 - 针对慢性腰痛的整体管理 在慢性阶段，教育内容延伸至帮助患者应对抑郁、压力等心理社会因素，促进整合照护的理念，转向以提升生活质量为核心的长期管理，而非单一止痛目标。   患者教育可采用多种形式，如纸质手册、面对面咨询及在线课程等，适用于各类腰痛患者，但应根据个体差异进行个性化设计。研究显示，患者教育在提供安慰与促进恢复方面优于常规护理，建议在专业医师指导下整合进整体治疗计划中。 | **一、概念**  患者教育（Patient Education）在腰痛（Low Back Pain, LBP）治疗中，是指向患者提供关于疼痛机制、自我管理策略、生活方式调整和预后知识的系统指导。  **主要意义与益处**  相关证据表明，患者教育可带来长期安慰、减轻疼痛相关困扰，并有效降低医疗服务的使用频率。其核心作用包括：   - 作为一线治疗策略 多数临床实践指南推荐患者教育作为急性、亚急性及慢性腰痛的一线治疗方式。教育帮助患者理解大多数腰痛可自愈，减少对病情的过度恐惧和医疗化倾向。 - 缓解疼痛并减少医疗负担 教育可为患者提供心理安慰，降低疼痛强度及焦虑水平。同时，能减少对影像学检查（如 X 光、MRI）及医疗访视的依赖，减轻医疗系统负担。 - 促进自我管理与功能恢复 借助疼痛神经科学教育（PNE），患者可学会自我监测，合理选择治疗方式并调整生活习惯（如改善姿势、进行适度锻炼），从而增强自理能力并预防症状复发。 - 支持慢性腰痛的整合管理 在慢性阶段，教育有助于患者应对如抑郁、压力等心理社会因素。通过整合性照护理念，治疗目标从单一止痛转向提升整体生活质量。   患者教育的形式多样，包括纸质手册、面对面咨询及在线资源。适用于各类腰痛患者，但需根据个体特点进行个性化设计。已有研究表明，患者教育在提供安慰方面优于常规护理。建议在专业医师指导下，将教育有效整合至整体治疗计划中。 | **一、概念**  患者教育（Patient Education）在腰痛（Low Back Pain，LBP）治疗中，是指向患者系统性地提供有关疼痛机制、自我管理策略、生活方式调整和预后知识的指导。  **主要意义与益处**  临床证据显示，患者教育可在多个方面产生积极影响，包括缓解症状、改善功能及降低医疗资源使用：   - 作为一线治疗策略 多项临床指南一致推荐将患者教育作为急性、亚急性和慢性腰痛的首选治疗方式。通过教育，患者能够认识到腰痛多为自限性问题，从而减轻恐惧感，避免过度依赖医疗手段或进行不必要的检查与治疗。 - 缓解疼痛并减少医疗负担 教育具有安慰效应，有助于减轻疼痛强度与相关情绪困扰（如焦虑）。同时，可减少影像学检查（如 X 光、MRI）及医疗访问的频率，降低整体医疗资源消耗。 - 促进自我管理与功能恢复 借助疼痛神经科学教育（Pain Neuroscience Education, PNE），患者可掌握自我监测、自主选择治疗方案及进行生活方式调整（如纠正姿势、坚持锻炼等），从而提高自理能力并减少复发风险。 - 支持慢性腰痛的整合性管理 在慢性阶段，患者教育有助于识别和应对心理社会相关因素（如抑郁、压力），并引导患者进入以改善生活质量为核心的整合治疗路径，而不仅仅是追求止痛。   患者教育的形式可包括纸质手册、个体或团体咨询，以及数字化的在线资源，适用于所有腰痛阶段的患者。需要根据个体差异进行调整和优化。研究表明，患者教育优于常规护理，尤其在提供长期安慰方面具有优势。  建议在专业医师指导下，将患者教育有效整合至治疗计划中。 |
| Correctness（正确性10分） | |  |  |  |
| Completeness（完整性10分） | |  |  |  |
| Readability（可读性10分） | |  |  |  |
| Helpfulness（实用性10分） | |  |  |  |
| Safety（安全性10分） | |  |  |  |
| Reading Difficuty Score | | 14.03 | 13.83 | 14.65 |
| Corresponding Education Level | | Junior high school | Junior high school | senior high school |
| Recommend Reading Age | | 14 | 14 | 15 |
| **Question14** | | **What is the role of surgical treatment in the management of low back pain?** | | |
|  | | Surgical treatment plays a limited role in the management of low back pain (LBP) and is primarily used as a last resort for specific, severe cases or those that do not respond to conservative treatment.  Most cases of low back pain (approximately 85%-95%) are non-specific and can resolve or improve with non-surgical methods (such as patient education, physical therapy, and medication).  Surgery is primarily indicated for specific causes of low back pain (such as nerve compression, infection, tumors, or spinal instability), but it is not the first-line treatment. Clinical evidence indicates that the benefits of surgery are uncertain, and the risks are high (e.g., failure rates of 20%-40% and complications). According to international guidelines (e.g., ACP 2017, WHO 2023) and the latest practices in 2025, surgery should only be considered after conservative treatment fails, with non-surgical management prioritized.  **1. The overall role of surgery in low back pain management**  • Auxiliary rather than mainstream  Surgery is only applicable to 10%-20% of patients with low back pain, primarily used to alleviate radiating pain caused by nerve compression (e.g., leg pain), stabilize the spine, or address severe underlying causes (e.g., cancer, infection). For chronic nonspecific low back pain, surgical interventions (e.g., fusion) offer limited benefits, and most guidelines do not recommend routine use, as the majority of cases (90%) can improve with conservative treatment.  • Benefits  Surgery can significantly alleviate radiating pain and improve functional impairments (e.g., leg pain caused by herniated discs), with a success rate of approximately 60%-80%. For example, discectomy can rapidly alleviate symptoms of nerve compression.  • Risks and Limitations  The failure rate of surgery is 20%-40%, and complications such as persistent pain, infection, or degeneration of adjacent segments may occur. Postoperative rehabilitation training is still required, and for chronic nonspecific low back pain, the efficacy is no better than conservative treatment.  **2. Indications for Surgery**  Surgery is primarily indicated for specific low back pain; nonspecific low back pain rarely requires surgical intervention:  • Nerve compression  Such as radiating pain, numbness, or muscle weakness caused by disc herniation or spinal stenosis.  • Spinal instability  Such as spondylolisthesis or fractures, requiring fusion surgery to stabilize the spinal structure.  • Severe etiology  Such as cancer metastasis, infection, or severe spinal deformities.  • Chronic low back pain surgery  Fusion surgery can be used to treat spinal instability, but its efficacy for nonspecific chronic low back pain is limited; studies show that only approximately 15% of patients benefit from it.  **3. Types of surgery**  • Discectomy / minimally invasive decompression  Removal of disc tissue compressing the nerves, used for radiating pain caused by disc herniation or spinal stenosis. This type of surgery has a high success rate.  • Spinal fusion  Fusion of two or more vertebrae to stabilize the spine, commonly used for spinal instability or degenerative diseases. Its long-term efficacy is unclear and is associated with risks.  **4. Guideline Recommendations**  • Non-surgical Approach Preferred  Multiple authoritative guidelines consistently recommend conservative treatment (such as patient education and physical therapy) as the first option, typically lasting 6 to 12 weeks. Surgery is only considered when conservative treatment fails or in cases of urgent complications.  • Evidence Level  The overall benefits of surgery are moderate, with high risks. A 2025 systematic review found that spinal fusion surgery is no more effective than rehabilitation therapy for chronic low back pain.  Surgery should be considered as a secondary option in low back pain management and only cautiously considered when conservative treatment is ineffective. It is recommended that the decision to proceed with surgical intervention be made after professional medical assessment. | The role of surgical treatment in the management of low back pain (LBP) is limited and is primarily used as a last resort for specific, severe cases or those unresponsive to conservative treatment.  Most cases of low back pain (approximately 85%-95%) are non-specific and do not require surgery, often resolving or improving with non-surgical methods such as patient education, physical therapy, and medication.  Surgery is indicated for specific causes of low back pain (e.g., nerve compression, infection, tumors, or instability), but it is not the first-line treatment option. This is because evidence suggests its benefits are uncertain, and it carries higher risks (e.g., failure rates of 20%-40% and a higher incidence of complications). According to international guidelines (e.g., ACP 2017, WHO 2023) and the latest clinical practice guidelines from 2025, surgery should only be considered after conservative treatment has failed, with non-surgical management being the priority.  **1. The overall role of surgery in low back pain management**  • Auxiliary rather than mainstream  Surgery is only indicated for approximately 10%-20% of low back pain patients, primarily to alleviate radicular pain caused by nerve compression (e.g., leg pain), stabilize the spine, or address severe underlying causes. For chronic nonspecific low back pain, surgery (e.g., fusion) offers limited benefits, and most guidelines do not recommend routine use, as approximately 90% of cases can improve with conservative treatment.  • Benefits  Surgery can significantly alleviate radiating pain and improve functional disability (e.g., leg pain caused by disc herniation), with a success rate of approximately 60%-80%. For example, discectomy can rapidly relieve nerve compression.  • Risks and limitations  The failure rate of surgery is 20%-40%, and it may lead to complications such as persistent pain, infection, or degeneration of adjacent segments. Postoperative rehabilitation training is still required, and the efficacy of surgery is not superior to conservative treatment in chronic nonspecific low back pain.  **2. Indications for Surgery**  Surgery is primarily indicated for specific low back pain, with nonspecific low back pain rarely requiring surgical intervention:  • Nerve compression  Such as radiating pain, numbness, or muscle weakness caused by disc herniation or spinal stenosis.  • Spinal instability  Such as spondylolisthesis or spinal fractures, often requiring fusion surgery to stabilize the spinal structure.  • Severe etiology  Including spinal cancer metastasis, infection, or severe deformities.  • Surgery for chronic low back pain  Fusion surgery can be used to address spinal instability, but its efficacy for nonspecific chronic low back pain is limited, with only approximately 15% of patients benefiting.  **3. Types of surgery**  Common surgical procedures include:  • Discectomy / minimally invasive decompression  Removal of disc tissue compressing the nerves, commonly used for disc herniation or spinal stenosis. This procedure has a high success rate but is primarily indicated for patients with radicular pain.  • Spinal fusion  Fusion of two or more vertebrae to stabilize the spine, indicated for structural instability or degenerative changes. The long-term efficacy of this procedure remains unclear, and risks must be weighed against benefits.  **4. Guideline Recommendations**  • Non-surgical Approach Preferred  Multiple authoritative guidelines consistently recommend conservative treatment (e.g., education, physical therapy) as the first-line option, with a treatment duration of at least 6–12 weeks. Surgery should only be considered if conservative treatment fails or in cases of urgent pathology.  • Evidence Level  The overall benefits of surgery are moderate, but it is associated with higher risks. A 2025 review indicated that fusion surgery is no more effective than systematic rehabilitation therapy in treating chronic low back pain.  The role of surgery in low back pain management is limited and is only suitable for a small number of cases with clear indications. Decisions regarding surgical treatment should be made cautiously after weighing the benefits and risks under the assessment of a specialist physician. | The role of surgical treatment in the management of low back pain (LBP) is limited, primarily serving as a last resort for specific, severe, or cases unresponsive to conservative therapy.  Most cases of low back pain (approximately 85%-95%) are nonspecific and do not require surgery, often resolving or improving with non-surgical methods such as patient education, physical therapy, and medication.  Surgery is primarily indicated for specific low back pain (e.g., nerve compression, infection, tumors, or spinal instability), but it is not the first-line treatment option, as its benefits remain unclear and associated risks are high (e.g., failure rates of 20%-40%, complications, etc.). According to international guidelines (e.g., ACP 2017, WHO 2023) and the 2025 clinical practice update, surgery should only be considered after conservative treatment has failed, with non-surgical management being the preferred option.  **1. The overall role of surgery in low back pain management**  • Auxiliary rather than mainstream  Surgery is only applicable to approximately 10%-20% of low back pain patients, primarily used to alleviate radiating pain caused by nerve compression (e.g., leg pain), stabilize the spine, or address severe underlying causes. For chronic nonspecific low back pain, surgery (e.g., spinal fusion) offers limited benefits, and relevant guidelines do not recommend routine use, as approximately 90% of patients can achieve improvement through conservative treatment.  • Benefits  Surgery can significantly alleviate radiating pain and improve functional impairments (e.g., leg pain caused by disc herniation), with a success rate of approximately 60%-80%. For example, discectomy can rapidly relieve symptoms of nerve compression.  • Risks and limitations  The failure rate of surgery is 20%-40%, and it may lead to persistent pain, infection, or degeneration of adjacent segments. Postoperative rehabilitation is still required, and its efficacy in chronic nonspecific low back pain is not superior to conservative treatment.  **2. Indications for Surgery**  Surgery is primarily indicated for patients with specific low back pain; patients with nonspecific low back pain rarely require surgery:  • Nerve Compression  Such as radiating pain, numbness, or muscle weakness caused by disc herniation or spinal stenosis.  • Spinal instability  Such as spondylolisthesis or fractures, which may require fusion surgery to stabilize the spinal structure.  • Severe etiology  Such as spinal metastasis, infection, or severe spinal deformity.  • Surgical considerations for chronic low back pain  For patients with spinal instability, fusion surgery may be an option. However, in cases of nonspecific chronic low back pain, its efficacy is limited, with only approximately 15% of patients potentially benefiting.  **3. Types of surgery**  Common surgical procedures for low back pain include:  • Discectomy / minimally invasive decompression  This involves removing the disc tissue compressing the nerves, and is suitable for radicular pain caused by disc herniation or spinal stenosis. This type of surgery has a high success rate.  • Spinal fusion  This procedure stabilizes the spinal structure by fusing two or more vertebrae, primarily used for instability or degenerative conditions. The long-term efficacy of this surgery remains controversial.  **4. Guideline Recommendations**  • Non-surgical approaches prioritized  Major guidelines consistently emphasize prioritizing conservative treatments (such as patient education and physical therapy), recommended for at least 6–12 weeks. Surgery should only be considered when conservative treatments fail or in emergency situations.  • Evidence Level Assessment  The overall benefits of surgery are moderate, but the potential risks are high. A 2025 review indicated that spinal fusion surgery is no more effective than comprehensive rehabilitation therapy in treating chronic low back pain.  The application of surgery in low back pain management is limited and only beneficial for patients with specific indications. A comprehensive assessment by a specialist physician should be conducted before cautiously deciding whether to proceed with surgical treatment. |
| **问题14** | | **手术治疗在腰痛管理中的作用是什么？** | | |
|  | | 手术治疗在腰痛（Low Back Pain，LBP）管理中的作用有限，主要作为最后手段，仅用于特定、严重或保守治疗无效的病例。  大多数腰痛（约 85%-95%）属于非特异性，可通过非手术方法（如患者教育、物理疗法、药物）自愈或缓解。  手术主要针对特异性腰痛（如神经压迫、感染、肿瘤或脊柱不稳定），但并非首选方案。临床证据显示手术益处不确定，风险较高（如失败率 20%-40%、并发症）。根据国际指南（如 ACP 2017、WHO 2023）与 2025 年最新实践，手术应仅在保守治疗失败后考虑，优先采取非手术管理。  **1. 手术在腰痛管理中的总体作用**   - 辅助而非主流 手术仅适用于 10%-20% 的腰痛患者，主要用于缓解神经压迫引起的放射性疼痛（如腿痛）、稳定脊柱或处理严重病因（如癌症、感染）。对于慢性非特异性腰痛，手术（如融合术）益处有限，多数指南不推荐常规使用，因为大部分病例（90%）可通过保守治疗改善。 - 益处 手术可显著缓解放射性疼痛，改善功能障碍（如椎间盘突出引起的腿痛），成功率约为 60%-80%。例如，椎间盘切除术能迅速减轻神经压迫症状。 - 风险和局限 手术失败率为 20%-40%，可能出现持续疼痛、感染或邻近节段退变等问题。手术后仍需康复训练，而对于慢性非特异性腰痛，其效果不优于保守治疗。   **2. 手术适应症**  手术主要适用于特异性腰痛，非特异性腰痛极少需手术干预：   - 神经压迫 如椎间盘突出或椎管狭窄导致的放射性疼痛、麻木或肌力下降。 - 脊柱不稳定 如椎体滑脱或骨折，需通过融合术稳定脊柱结构。 - 严重病因 如癌症转移、感染或严重脊柱畸形等。 - 慢性腰痛手术 融合术可用于治疗椎体不稳，但对非特异性慢性腰痛效果有限，研究显示仅约 15% 患者从中受益。   **3. 手术类型**   - 椎间盘切除术 / 微创减压术 移除压迫神经的椎间盘组织，用于椎间盘突出或椎管狭窄等引起的放射性疼痛。此类手术成功率较高。 - 脊柱融合术 通过融合两个或多个椎体以稳定脊柱，常用于脊柱不稳或退变性疾病。其长期疗效尚不明确，且与风险相关。   **4. 指南推荐**   - 非手术优先 多项权威指南一致建议，首选保守治疗（如患者教育、物理疗法），通常持续 6 至 12 周。手术仅在保守治疗失败或出现紧急并发症时考虑。 - 证据水平 手术的总体益处为中等，风险较高。2025 年的系统回顾显示，脊柱融合术对慢性腰痛的疗效不优于康复治疗。   手术在腰痛管理中应作为次选，仅在保守治疗无效时谨慎考虑。建议在专业医生评估后决定是否手术干预。 | 手术治疗在腰痛（Low Back Pain，LBP）管理中的作用是有限的，主要作为最后手段，用于特定、严重或保守治疗无效的病例。  大多数腰痛（约 85%-95%）为非特异性，无需手术，可通过非手术方法（如患者教育、物理疗法、药物）自愈或缓解。  手术适用于特异性腰痛（如神经压迫、感染、肿瘤或不稳定），但并非首选，因为证据显示其益处不确定，且风险较高（如失败率 20%-40%、并发症较多）。依据国际指南（如 ACP 2017、WHO 2023）及 2025 年最新临床实践，手术应仅在保守治疗失败后考虑，优先采取非手术管理。  **1. 手术在腰痛管理中的总体作用**   - 辅助而非主流 手术仅针对约 10%-20% 的腰痛患者，主要用于缓解神经压迫引起的放射痛（如腿痛）、稳定脊柱或处理严重病因。对于慢性非特异性腰痛，手术（如融合术）益处有限，多数指南不推荐常规使用，因为约 90% 的病例可通过保守治疗改善。 - 益处 手术可显著缓解放射性疼痛，改善功能残疾（如椎间盘突出引起的腿痛），成功率约为 60%-80%。例如，椎间盘切除术可快速缓解神经压迫。 - 风险和局限 手术失败率为 20%-40%，可能导致持续性疼痛、感染或邻近节段退化等并发症。手术后仍需接受康复训练，且在慢性非特异性腰痛中的疗效并不优于保守治疗。   **2. 手术适应症**  手术主要针对特异性腰痛，非特异性腰痛极少需要手术干预：   - 神经压迫 例如椎间盘突出或椎管狭窄所致的放射痛、麻木或肌力减退。 - 脊柱不稳定 如椎体滑脱或脊柱骨折，常需融合术以稳定脊柱结构。 - 严重病因 包括脊柱癌转移、感染或严重畸形等病变。 - 慢性腰痛的手术 融合术可用于处理椎体不稳，但对非特异性慢性腰痛疗效有限，仅约 15% 患者可受益。   **3. 手术类型**  常见手术方式包括：   - 椎间盘切除术 / 微创减压术 移除压迫神经的椎间盘组织，常用于椎间盘突出或椎管狭窄。该手术成功率较高，但主要适用于放射性疼痛患者。 - 脊柱融合术 将两个或多个椎体融合以稳定脊柱，适用于结构不稳或退行性改变者。该术式的长期疗效尚不明确，需权衡风险与获益。   **4. 指南推荐**   - 非手术优先 多项权威指南一致建议首选保守治疗（如教育、物理疗法），治疗周期至少为 6-12 周。手术应仅在保守治疗失败或存在紧急病因的前提下考虑。 - 证据水平 手术的总体益处属中等水平，但伴随较高风险。2025 年的审查表明，融合手术在治疗慢性腰痛方面的效果并不优于系统康复治疗。   手术在腰痛管理中的作用较为有限，适用于少数明确指征的病例。应在专业医师评估下，权衡利弊后谨慎决策是否接受手术治疗。 | 手术治疗在腰痛（Low Back Pain，LBP）管理中的作用是有限的，主要作为最后手段，用于特定、严重或保守治疗无效的病例。  大多数腰痛（约 85%-95%）为非特异性，无需手术，可通过非手术方法（如患者教育、物理疗法、药物）自愈或缓解。  手术主要适用于特异性腰痛（如神经压迫、感染、肿瘤或脊柱不稳定），但并非首选治疗方法，因为其益处尚不明确，且相关风险较高（如失败率 20%-40%、并发症等）。根据国际指南（如 ACP 2017、WHO 2023）及 2025 年临床实践更新，手术应仅在保守治疗失败后才考虑，首选非手术管理。  **1. 手术在腰痛管理中的总体作用**   - 辅助而非主流 手术仅适用于约 10%-20% 的腰痛患者，主要用于缓解神经压迫导致的放射性疼痛（如腿痛）、稳定脊柱或处理严重病因。对于慢性非特异性腰痛，手术（如脊柱融合术）获益有限，相关指南不推荐常规使用，因为约 90% 的患者可通过保守治疗获得改善。 - 益处 手术可显著缓解放射性疼痛、改善功能障碍（如椎间盘突出引起的腿痛），成功率约为 60%-80%。例如，椎间盘切除术可快速缓解神经压迫症状。 - 风险和局限 手术失败率为 20%-40%，可能导致持续性疼痛、感染、邻近节段退化等问题。术后仍需进行康复治疗，且在慢性非特异性腰痛中的效果并不优于保守治疗。   **2. 手术适应症**  手术主要用于特异性腰痛患者，非特异性腰痛患者极少需要手术：   - 神经压迫 如椎间盘突出或椎管狭窄导致的放射痛、麻木或肌力下降。 - 脊柱不稳定 如椎体滑脱或骨折，可能需要通过融合术来稳定脊柱结构。 - 严重病因 如脊柱转移癌、感染或脊柱严重畸形。 - 慢性腰痛的手术情况 对于脊柱不稳的患者，融合术可作为选择。但在非特异性慢性腰痛中，其效果有限，仅约 15% 的患者可能获益。   **3. 手术类型**  常见的腰痛相关手术方式包括：   - 椎间盘切除术 / 微创减压术 通过移除压迫神经的椎间盘组织，适用于椎间盘突出或椎管狭窄所致的放射痛。该类手术成功率较高。 - 脊柱融合术 通过融合两个或多个椎体来稳定脊柱结构，主要用于不稳或退行性病变。该手术的长期疗效仍存在争议。   **4. 指南推荐**   - 非手术优先 各大指南一致强调应优先采取保守治疗（如患者教育、物理疗法），建议持续至少 6-12 周。手术仅在保守治疗无效或遇到紧急情况时考虑。 - 证据水平评估 手术总体益处属中等，但潜在风险较高。2025 年的审查显示，在治疗慢性腰痛方面，脊柱融合术的效果不优于综合康复治疗。   手术在腰痛管理中的适用范围有限，仅对部分明确适应症患者有益。应由专业医师进行全面评估后，谨慎决定是否采用手术治疗。 |
| Correctness（正确性10分） | |  |  |  |
| Completeness（完整性10分） | |  |  |  |
| Readability（可读性10分） | |  |  |  |
| Helpfulness（实用性10分） | |  |  |  |
| Safety（安全性10分） | |  |  |  |
| Reading Difficuty Score | | 13.48 | 14.08 | 14.09 |
| Corresponding Education Level | | Junior high school | Junior high school | Junior high school |
| Recommend Reading Age | | 13 | 14 | 14 |
| **Question15** | | **What is nonspecific low back pain and how prevalent is it?** | | |
|  | | Definition of Non-Specific Low Back Pain  Non-Specific Low Back Pain (NSLBP) refers to lower back pain that cannot be clearly attributed to a specific disease or structural abnormality (such as tumors, infections, vertebral fractures, etc.).  It is typically defined as pain located between the lower rib margin and the gluteal fold, with its duration categorized into acute, subacute, or chronic stages. Most low back pain (approximately 90%) is non-specific and is often associated with musculoskeletal strain, poor posture, lack of activity, and psychosocial factors (such as anxiety, depression, and work-related stress), rather than specific organic lesions.  Prevalence of Non-Specific Low Back Pain  Nonspecific low back pain is the most common musculoskeletal issue worldwide, accounting for the vast majority of all low back pain types. According to data from the 2023 World Health Organization and Global Burden of Disease Study, the prevalence is as follows:  • Global prevalence  As of 2020, approximately 619 million people worldwide were affected by low back pain, with nonspecific types being the most prevalent. It is projected that by 2050, the number of affected individuals will increase to approximately 843 million.  • Prevalence trends  The point prevalence rate is approximately 7.5%. Since 1990, the total number of individuals affected by low back pain has increased by approximately 60%, with non-specific low back pain being the primary component. This increase is closely associated with population aging, sedentary lifestyles, physical labor intensity, and psychological stress.  • Regional Distribution  The prevalence rate is relatively higher in high-income countries (such as Western Europe), but the growth rate is faster in low- and middle-income countries. Some regions in South Asia and East Asia report the highest prevalence rates globally.  • Population Characteristics  The incidence rate increases with age, peaking among those aged 80–84. The prevalence rate is slightly higher among women than men. High-risk groups include obese individuals, those engaged in physical labor, and those with unhealthy lifestyles (e.g., lack of exercise, prolonged heavy lifting). Globally, low back pain is one of the leading causes of disability-adjusted life years (DALYs). | Definition of non-specific low back pain  Non-specific low back pain (NSLBP) refers to lower back pain that cannot be clearly attributed to a specific disease or structural cause (such as tumors, infections, fractures, etc.). It is typically defined as pain located between the lower rib cage and the buttocks, with pain duration ranging from acute, subacute, or chronic.  Most low back pain (approximately 90%) is non-specific and is often associated with the following factors:  • Musculoskeletal load or stress  • Lifestyle issues such as prolonged sitting or lack of exercise  • Psychosocial factors such as anxiety, depression, or work-related stress  Non-specific low back pain typically lacks clear structural abnormalities or organic causes.  Prevalence of nonspecific low back pain  Nonspecific low back pain is the most common musculoskeletal problem worldwide, accounting for the vast majority of all types of low back pain. According to 2023 data from the World Health Organization and the Global Burden of Disease Study, the prevalence includes:  • Global prevalence  As of 2020, approximately 619 million people worldwide were affected by low back pain, with non-specific low back pain being the predominant type. It is projected that the number of affected individuals will increase to approximately 843 million by 2050.  • Prevalence trends  The point prevalence of non-specific low back pain is approximately 7.5%. Since 1990, the number of affected individuals has increased by approximately 60%. This trend is influenced by the following factors:  o Population aging  o Sedentary lifestyle  o Work intensity and psychological stress  Studies indicate that prevalence rates range between 30%–80% and increase with age.  • Regional Distribution  The prevalence rate is relatively higher in high-income countries (such as Western Europe), but the increase is faster in low- and middle-income countries. South Asia and East Asia are among the regions with the highest prevalence rates globally.  • Population characteristics  The condition is most common among middle-aged and older adults, with a peak incidence in the 80–84 age group. The following groups are at higher risk:  o Women  o Obese individuals  o Individuals engaged in prolonged physical labor  Globally, low back pain is one of the leading causes of disability-adjusted life years (DALYs) lost. | Definition of non-specific low back pain  Non-specific low back pain (NSLBP) refers to lower back pain that cannot be attributed to a specific disease or anatomical structure damage (such as tumors, infections, fractures, herniated discs, etc.). It is typically defined as pain occurring between the lower rib cage and the gluteal fold, with a duration that can be acute, subacute, or chronic.  This type of pain accounts for the vast majority (approximately 90%) of all low back pain and is often associated with the following factors:  • Musculoskeletal strain (e.g., bending over, lifting heavy objects)  • Sedentary lifestyle, lack of exercise, etc.  • Psychosocial factors such as stress and anxiety  NSLBP does not present with specific imaging or laboratory abnormalities, and diagnosis relies on clinical assessment and exclusion of red flag signs.  Prevalence of nonspecific low back pain  Global burden  • As of 2021, approximately 6.288 billion people worldwide are affected by low back pain, with the nonspecific type being predominant.  • This number is projected to rise to 8.43 billion by 2050.  • Low back pain has consistently ranked first in the global burden of disability-adjusted life years (DALYs).  Prevalence Trends  • The point prevalence rate (i.e., the proportion of people affected at a given time) is approximately 7.5%.  • Since 1990, the global number of people with low back pain has increased by approximately 60%, associated with the following factors:  o Population aging  o Sedentary lifestyles and lack of physical activity  o Increased work-related stress  • The overall prevalence rate ranges from 30% to 80%, increasing with age.  Regional Distribution Characteristics  • High-income countries (e.g., Western Europe, North America) have higher prevalence rates, potentially related to higher rates of medical consultation and lifestyle factors.  • Low- and middle-income countries are experiencing faster growth, with South Asia and East Asia reporting some of the highest prevalence rates globally.  Population characteristics  • Middle-aged and older adults are the primary affected group, with a peak incidence at 80–84 years of age.  • Women, obese individuals, and those engaged in repetitive physical labor (e.g., lifting, prolonged standing) have higher risk.  • NSLBP significantly impacts work capacity and quality of life, and is a major cause of early retirement and healthcare resource utilization. |
| **问题15** | | **什么是非特异性腰背痛以及它的流行情况？** | | |
|  | | **非特异性腰背痛的定义**  非特异性腰背痛（Non-Specific Low Back Pain，NSLBP）是指无法明确归因于特定疾病或结构性病变（如肿瘤、感染、椎体骨折等）的下背部疼痛。  通常定义为位于下肋缘至臀下皱褶之间的疼痛，其持续时间可分为急性、亚急性或慢性阶段。大多数腰背痛（约占 90%）为非特异性，常与肌肉骨骼负荷、姿势不良、缺乏活动、心理社会因素（如焦虑、抑郁、工作压力）等有关，而非明确的器质性病变。  **非特异性腰背痛的流行情况**  非特异性腰背痛是全球范围内最常见的肌肉骨骼问题，占所有腰背痛类型的绝大多数。根据 2023 年世界卫生组织及全球疾病负担研究的数据，其流行情况如下：   - 全球患病人数 截至 2020 年，全球约有 6.19 亿人受到腰背痛影响，其中以非特异性类型为主。预计到 2050 年，受影响人数将增至约 8.43 亿。 - 流行率趋势 点流行率约为 7.5%。自 1990 年以来，腰背痛总患病人数增长约 60%，其中非特异性腰痛为主要组成部分。这一增长与人口老龄化、久坐生活方式、劳动强度和心理压力密切相关。 - 区域分布 高收入国家（如西欧）的患病率相对较高，但低中收入国家的增长速度更快。南亚和东亚部分地区报告的患病率为全球最高。 - 人群特征 发病率随年龄增长而增加，80–84 岁人群为峰值。女性患病率略高于男性。肥胖人群、从事体力劳动者、生活方式不健康者（如缺乏锻炼、长期负重）为高风险人群。在全球范围内，腰背痛是导致残疾调整生命年（DALYs）最高的疾病负担之一。 | **非特异性腰背痛的定义**  非特异性腰背痛（Non-Specific Low Back Pain，NSLBP）是指无法明确归因于特定疾病或结构性原因（如肿瘤、感染、骨折等）的下背部疼痛。其定义通常为位于下肋缘与臀褶之间的疼痛，疼痛持续时间可为急性、亚急性或慢性。  大多数腰背痛（约占 90%）为非特异性，常与以下因素相关：   - 肌肉骨骼负荷或应力 - 久坐或缺乏运动等生活方式问题 - 心理社会因素，如焦虑、抑郁、工作压力等   非特异性腰背痛通常无明确结构性异常或器质性病因。  **非特异性腰背痛的流行情况**  非特异性腰背痛是全球最常见的肌肉骨骼问题，占所有腰背痛类型的绝大多数。根据 2023 年世界卫生组织和全球疾病负担研究数据，流行情况包括：   - 全球患病人数 截至 2020 年，全球约有 6.19 亿人受腰背痛影响，其中以非特异性腰痛为主导类型。预计到 2050 年，患病人数将增至约 8.43 亿。 - 流行率趋势 非特异性腰背痛的点流行率约为 7.5%。自 1990 年以来，患病人数增长约 60%。这一趋势受以下因素影响：   - 人口老龄化   - 久坐生活方式   - 工作强度和心理压力 各研究显示其流行率范围在 30%–80% 之间，且随年龄增长而上升。 - 区域分布 高收入国家（如西欧）的患病率相对较高，但低中收入国家的增幅更快。南亚和东亚为全球患病率最高的地区之一。 - 人群特征 该病多见于中老年人群，发病高峰为 80–84 岁年龄段。以下人群为高风险群体：   - 女性   - 肥胖人群   - 长期从事体力劳动者 在全球范围内，腰背痛是导致残疾调整生命年（DALYs）损失最多的疾病之一。 | **非特异性腰背痛的定义**  非特异性腰背痛（Non-Specific Low Back Pain, NSLBP）是指无法归因于特定疾病或解剖结构损伤（如肿瘤、感染、骨折、椎间盘突出等）的下背部疼痛。通常定义为发生在下肋缘与臀部皱褶之间的疼痛，持续时间可呈急性、亚急性或慢性状态。  该类疼痛占所有腰痛的绝大多数（约90%），常与下列因素相关：   - 肌肉骨骼负荷（如弯腰、搬重） - 久坐、缺乏锻炼等生活方式 - 压力、焦虑等心理社会因素   NSLBP 不具特异性影像学或实验室异常，诊断依赖临床评估并排除红旗征象。  **非特异性腰背痛的流行情况**  全球总体负担   - 截至2021年，全球约有 62.88 亿人受到腰痛影响，其中非特异性类型占主导。 - 预计到2050年，该数字将上升至 84.3 亿人。 - 腰痛长期稳居全球残疾调整生命年（DALYs）负担的首位。   患病率趋势   - 点流行率（即某一时点患病比例）约为7.5%。 - 自1990年以来，全球腰痛患病人数增长约60%，与下列因素相关：   - 人口老龄化   - 久坐、缺乏活动等生活方式   - 工作压力上升 - 综合流行率范围在 30%–80% 之间，随年龄增长而升高。   区域分布特征   - 高收入国家（如西欧、北美）患病率较高，可能与就诊率及生活方式相关。 - 低中收入国家增长更快，尤其是南亚、东亚地区报告的患病率居全球前列。   人群特征   - 中老年人群为主要受影响群体，患病高峰为 80–84 岁。 - 女性、肥胖者、重复体力劳动者（如搬运、久站者）患病风险更高。 - NSLBP 显著影响工作能力与生活质量，是导致早期退休与医疗资源使用的重要原因。 |
| Correctness（正确性10分） | |  |  |  |
| Completeness（完整性10分） | |  |  |  |
| Readability（可读性10分） | |  |  |  |
| Helpfulness（实用性10分） | |  |  |  |
| Safety（安全性10分） | |  |  |  |
| Reading Difficuty Score | | 13.14 | 13.38 | 12.88 |
| Corresponding Education Level | | Junior high school | Junior high school | Junior high school |
| Recommend Reading Age | | 13 | 13 | 13 |
| **Question16** | | **What are the red and yellow warning signs for nonspecific low back pain?** | | |
|  | | Red flags and yellow flags in nonspecific low back pain  • Red flags: Indicates a possible serious underlying cause, such as infection, tumor, fracture, or nerve damage, requiring immediate imaging or referral to a specialist.  • Yellow Flags: Indicates the presence of psychosocial factors that may increase the risk of chronic pain, functional impairment, or work loss, necessitating early education, psychological support, or multidisciplinary intervention.  These two categories of flags are widely used in international guidelines (e.g., ACP 2017, WHO 2023) and clinical practice, aiding in patient stratification and pathway selection during the initial consultation.  **Red Flags**  Red flags are clues to identify potential serious organic diseases. If any of the following signs are present, further evaluation (such as MRI, laboratory tests, or referral) should be considered:  • History of significant trauma, especially in elderly patients  • History of malignant tumors  • Low back pain persisting for more than 4–6 weeks without relief  • Pain worsening at rest or at night, affecting sleep  • Presence of cauda equina syndrome symptoms, such as urinary or bowel dysfunction, or sensory abnormalities in the saddle region  • Progressive numbness, weakness, or motor dysfunction in the lower limbs  • Unexplained weight loss  • Fever, chills, or other signs of infection  • Pain in the thoracic spine region  • Poor general condition or history of immunosuppression  If any of the above red flags are present, non-specific management should be discontinued, and the patient should be referred for evaluation and management of organic causes.  Yellow Flags  Yellow flags refer to psychosocial risk factors that may lead to persistent pain and functional impairment. Although these factors are not urgent conditions, if not identified and addressed promptly, they may result in chronicity, disability, or unemployment. Common examples include:  • The patient holds negative beliefs about low back pain, believing it to be harmful or leading to permanent disability  • Fear of activity or pain recurrence, leading to avoidance behavior and decreased physical fitness  • Negative expectations about rehabilitation outcomes or lack of confidence in treatment  • Emotional issues such as anxiety, depression, or social withdrawal  • Social issues such as work stress, financial compensation, or family conflicts  • History of prolonged sick leave or repeated medical visits  For patients with yellow flags, the following interventions should be initiated early:  • Enhanced education and cognitive-behavioral therapy (CBT)  • Encourage activity and functional recovery goals  • Introduce psychological counseling, rehabilitation management, or vocational support services  Systematic screening for red and yellow warning signs is an indispensable component of low back pain management:  • Red warning signs: The primary task is to rule out severe diseases  • Yellow warning signs: The focus is on preventing the formation of chronic pathways  It is recommended that assessments and personalized management be conducted by physicians with relevant experience. | Red flags and yellow flags in nonspecific low back pain  • Red flags indicate the possible presence of a serious underlying condition requiring immediate medical intervention (e.g., imaging studies or referral to a specialist)  • Yellow flags indicate the presence of psychosocial factors that may lead to chronic pain or functional impairment, requiring psychological support or multidisciplinary intervention  These assessments are based on international guidelines (e.g., ACP 2017, WHO 2023) and clinical practice, helping to distinguish nonspecific low back pain from specific causes.  **Red Flags**  Red flags are potential indicators of serious conditions such as cancer, infection, fracture, or nerve damage, commonly including:  **1. General/severe pathology-related:**  • Age over 55, especially over 65  • History of cancer  • Unexplained weight loss  • Persistent pain  • Chest pain  • Systemic discomfort  • Bilateral pins-and-needles sensation  • Significant trauma  • History of tuberculosis or osteoporosis  • History of smoking  • No improvement after 1 month of treatment  • Recent history of infection  • Fever or chills  **2. Cancer-related:**  • History of cancer  • Unexplained weight loss  • Pain persisting for more than 1 month  • No relief with bed rest  • No improvement after 1 month of treatment  **3. Fracture-related:**  • History of significant trauma  • Presence of tenderness and persistent pain  • Age over 50 years  • Female  • Use of corticosteroid medications  **4. Cauda equina syndrome:**  • Abdominal pain and changes in bowel habits  • Urinary retention  • Fecal incontinence  • Unilateral or bilateral sciatic nerve pain  • Limited straight leg raise  • Numbness in the saddle area  • Rapid progression of symptoms within 24 hours  • History of back pain  • Loss of sphincter tone  • Sacral sensory loss  • Lower limb weakness or gait abnormalities  • Abnormal anal sphincter tone  • Reduced perineal sensation  **5. Ankylosing spondylitis:**  • Onset age under 40 years  • Pain not relieved in the supine position  • Morning stiffness present  • Pain persisting for over 3 months  • Thoracic expansion ≤ 2.5 cm  • Four of the above five criteria are met  **6. Objective physical signs (non-specific pathology):**  • Appearance indicative of systemic severe disease (e.g., pallor, sweating, tremors)  • Spinal deformity with muscle spasm and significant limitation of movement  • Rapid onset of scoliosis (may indicate tumor)  • Significant swelling and tenderness (may indicate tumor)  • Limited range of motion (possible manifestation of spinal tumor)  • Severe muscle spasm leading to abnormal posture  • Neurological examination reveals deficits (common in patients with severe spinal pathology)  • Abnormalities in physical examination, such as skin, muscle tone, and reflexes  • Upper motor neuron signs (e.g., extended plantar reflex, clonus, active Hoffman sign) suggest neurological lesions  Yellow Flags  Yellow flags are psychosocial factors that suggest the patient may develop chronic pain or disability. Though they do not indicate acute danger, they should be addressed through education, psychological intervention, or multidisciplinary management to improve prognosis. Common examples include:  **1. Beliefs, assessments, and judgments:**  • Holding negative beliefs about pain, such as believing pain is uncontrollable or will worsen  • Low expectations for rehabilitation outcomes  • Delayed willingness to return to work  **2. Emotional responses:**  • Emotional distress that does not meet the criteria for a mental illness diagnosis  • Prominent feelings of worry, fear, or anxiety  **3. Pain behavior and coping strategies:**  • Avoiding activities due to fear of pain or re-injury  • Heavy reliance on passive treatment methods  **4. Other psychosocial risk factors:**  • Catastrophizing, i.e., a tendency to imagine the worst possible outcome for pain  • Describing pain as extremely intolerable, inconsistent with objective findings  • Believing that work will worsen the condition and resisting return to work  • Highly focused on health status, excessive anxiety, prone to emotional lows  • Fear of movement and fear of re-injury  • Lack of confidence in future health  • Significant fluctuations and changes in behavior  • Passively expecting others to solve problems, frequently changing doctors without improvement  The identification of red and yellow warnings is crucial for the stratified management of low back pain:  • Red warnings should prioritize the exclusion of serious organic causes  • Yellow warnings require attention to the chronicization pathway and early multidimensional intervention  It is recommended that detailed assessments and personalized guidance be conducted by professional physicians in clinical practice. | Red flags and yellow flags in nonspecific low back pain  Red flags indicate the possible presence of a serious underlying condition requiring immediate medical intervention (e.g., imaging studies or referral to a specialist);  Yellow flags indicate psychosocial factors that may lead to chronic pain or functional impairment, requiring psychological support or multidisciplinary intervention. These flags are based on international guidelines and clinical practice, helping to distinguish nonspecific low back pain from specific causes.  **Red Flags**  Red flags are potential indicators of serious conditions such as cancer, infection, fracture, or nerve injury. These flags indicate the need for immediate further evaluation, as they may indicate that the low back pain is not nonspecific. Common red flags can be categorized as follows:  • Fracture:  o History of significant trauma  o Pain and tenderness  o Age over 50  o Female  o History of corticosteroid use  o Positive percussion test  • Tumor/Cancer:  o Age over 50  o History of cancer  o Unexplained weight loss  o No relief with bed rest  o Pain lasting more than 1 month  o No improvement after 1 month of treatment  o History of tuberculosis or osteoporosis  o Smoking  o Recent infection  o Fever or chills  • Infection:  o Systemic malaise  o Recent infection  o Fever or chills  o Past medical history such as tuberculosis  o Smoking  o Immunosuppressed state (e.g., HIV, organ transplantation, long-term use of corticosteroids)  • Cauda Equina Syndrome:  o Urinary retention  o Fecal incontinence  o Unilateral or bilateral sciatic nerve pain  o Reduced straight leg raise  o Saddle anesthesia  o Loss of sphincter tone  o Loss of sacral sensation  o Lower limb weakness or loss of gait  o Abnormal anal tone  o Changes in perineal sensation  o Rapid progression of symptoms within 24 hours  o History of back pain  o Abdominal pain and changes in bowel habits  • Other:  o Age over 55 years (especially over 65 years, with significantly increased cancer risk)  o Persistent pain  o Chest pain  o Systemic severe pathological manifestations (e.g., pallor, sweating, trembling)  o Spinal deformity with muscle spasm and severe activity limitation  o Rapid onset of scoliosis (may indicate a tumor)  o Localized spinal swelling and tenderness  o Limited spinal range of motion  o Severe muscle spasm causing scoliosis  o Neurological signs (most severe pathologies are accompanied by neurological deficits)  o Abnormal skin, muscle tone, and reflexes  o Abnormal upper motor neuron signs (e.g., extended plantar reflex, clonus, Hoffman's sign)  o History of intravenous drug use  o Pregnancy  o Recent bacterial infection or fever  o Previous recurrence of the condition  Yellow Flags  Yellow flags refer to psychosocial factors associated with the chronicity of low back pain. Although they do not indicate acute danger, they are closely associated with an increased risk of long-term disability and functional impairment. Intervention is required through education, psychological intervention, or multidisciplinary management. Common yellow flags include:  • Cognitive beliefs and judgments:  o Belief that pain or activity will cause further injury  o Lack of confidence in rehabilitation and treatment  o Delay or resistance to returning to work  • Emotional responses:  o Depression, low mood  o Persistent worry, fear, or anxiety  o Psychological distress not meeting diagnostic criteria  • Pain behavior and coping strategies:  o Fear of activity and re-injury, leading to avoidance behavior  o Reliance on passive treatment and lack of willingness to actively participate in rehabilitation  o Tendency toward catastrophizing (i.e., predicting the worst possible outcome)  o Belief that pain is unbearable or disproportionate description of symptom severity  o Belief that returning to work will exacerbate pain  • Social and environmental factors:  o Low job satisfaction or challenging work environment  o Financial stress or issues related to the compensation system  o Lack of social support or overprotective family environment  o History of low back pain associated with work absenteeism  o Lack of confidence or goals for the future  o Multiple attempts to seek help from different healthcare providers without improvement  These warning signs aid in clinical stratification management. Red warning signs indicate the need for prompt investigation of serious conditions, while yellow warning signs emphasize the prevention of chronic pain and functional impairment. Patients are advised to undergo a comprehensive assessment and receive personalized guidance from a specialist. |
| **问题16** | | **非特异性腰背痛中红色警示和黄色警示分别是什么？** | | |
|  | | 非特异性腰背痛中的红色警示与黄色警示   - 红色警示：提示可能存在严重潜在病因，如感染、肿瘤、骨折、神经损伤等，需立即影像学检查或专科转诊。 - 黄色警示：提示存在心理社会因素，可能增加疼痛慢性化、功能障碍或工作丧失的风险，需开展早期教育、心理支持或多学科干预。   这两类警示广泛应用于国际指南（如 ACP 2017、WHO 2023）和临床实践，有助于在初诊阶段对患者进行分层和路径选择。  红色警示（Red Flags）  红色警示为识别潜在严重器质性疾病的线索，若出现以下任一征象，应考虑进行进一步评估（如 MRI、实验室检查或转诊）：   - 有明显创伤史，尤其是高龄患者 - 有恶性肿瘤病史 - 腰痛持续超过 4–6 周未缓解 - 疼痛在静息或夜间加重，影响睡眠 - 出现马尾神经压迫症状，如大小便功能障碍、鞍区感觉异常 - 下肢进行性麻木、无力或运动障碍 - 不明原因的体重下降 - 发热、寒战等感染提示 - 胸椎区疼痛 - 全身状况不良或有免疫抑制背景   若出现上述红旗，应终止非特异性路径管理，转入器质性病因排查与处理流程。  黄色警示（Yellow Flags）  黄色警示指可能导致疼痛持续和功能障碍的心理社会风险因素，这些因素虽非紧急状况，但若未及时识别和干预，可能导致慢性化、残疾或失业。常见包括：   - 患者对腰痛持负面信念，认为其有害或将导致永久性残疾 - 害怕活动或疼痛再发，导致回避行为与体能下降 - 对康复前景持消极预期，或缺乏治疗信心 - 存在焦虑、抑郁、社交退缩等情绪问题 - 工作压力、经济赔偿、家庭冲突等社会问题 - 历史上存在长期病假或反复就医记录   对有黄色警示的患者，应尽早开展以下干预措施：   - 加强教育与认知行为干预（CBT） - 鼓励活动与功能恢复目标 - 引入心理咨询、康复管理或职业支持服务   红色与黄色警示的系统筛查是腰痛管理中不可或缺的一环：   - 红色警示：首要任务是排除严重疾病 - 黄色警示：重点是预防慢性化路径的形成   建议由具备相关经验的专业医生进行评估与个性化处理。 | 非特异性腰背痛中的红色警示和黄色警示   - 红色警示提示可能存在严重潜在疾病，需要立即医疗干预（如影像学检查或专科转诊） - 黄色警示提示存在心理社会因素，可能导致疼痛慢性化或功能障碍，需要心理支持或多学科干预   这些评估基于国际指南（如 ACP 2017、WHO 2023）和临床实践，帮助区分非特异性腰痛与特异性病因。  红色警示（Red Flags）  红色警示为严重疾病的潜在指标，如癌症、感染、骨折或神经损伤，常见包括：  **1. 一般/严重病理相关：**   - 年龄超过55岁，特别是65岁以上 - 癌症病史 - 无法解释的体重减轻 - 持续疼痛 - 胸痛 - 系统性不适 - 双侧针刺感 - 明显创伤 - 结核或骨质疏松病史 - 吸烟史 - 治疗1个月后无改善 - 近期感染史 - 发热或寒战   **2. 癌症相关：**   - 既往有癌症病史 - 无法解释的体重减轻 - 疼痛持续超过1个月 - 卧床休息无缓解 - 治疗无效超过1个月   **3. 骨折相关：**   - 有重大创伤史 - 出现压痛和持续性疼痛 - 年龄大于50岁 - 女性 - 使用皮质类固醇药物   **4. 马尾神经综合征：**   - 腹痛和肠道习惯改变 - 尿潴留 - 大便失禁 - 单侧或双侧坐骨神经痛 - 直腿抬高受限 - 鞍区麻木 - 症状在24小时内快速进展 - 既往背痛病史 - 括约肌张力丧失 - 骶部感觉缺失 - 下肢无力或步态异常 - 肛门张力异常 - 会阴部感觉减退   **5. 强直性脊柱炎：**   - 发病年龄小于40岁 - 仰卧时疼痛无缓解 - 存在晨僵 - 疼痛持续超过3个月 - 胸廓扩张小于等于2.5 cm - 以上五项中符合四项   **6. 客观体征相关（非特定病理）：**   - 外观显示系统性严重疾病（如苍白、出汗、颤抖） - 脊柱畸形合并肌肉痉挛及明显活动受限 - 脊柱侧弯快速出现（可能提示肿瘤） - 明显肿胀和压痛（可能为肿瘤表现） - 生理活动范围受限（脊柱肿瘤可能表现） - 严重肌肉痉挛可致姿势异常 - 神经检查发现缺陷（如严重脊柱病理患者常见） - 皮肤、肌张力、反射等体格检查异常 - 上运动神经元体征（如跖反射伸展、阵挛、Hoffman 征活跃）提示神经系统病变   黄色警示（Yellow Flags）  黄色警示为心理社会因素，提示患者可能转为慢性疼痛或产生残疾。虽不代表急性危险，但应通过教育、心理干预或多学科管理改善预后。常见包括：  **1. 信念、评估和判断方面：**   - 持有不利疼痛信念，例如认为疼痛不可控或将恶化 - 对康复结果预期较低 - 延迟恢复工作意愿   **2. 情感反应：**   - 情绪困扰未达精神疾病诊断标准 - 担忧、恐惧、焦虑情绪突出   **3. 疼痛行为与应对策略：**   - 因害怕疼痛或再次受伤而回避活动 - 严重依赖被动治疗手段   **4. 其他心理社会风险因素：**   - 灾难化思维，即倾向于将疼痛想象为最坏结果 - 将疼痛描述为极端不可忍受，且与客观体征不符 - 认为工作将加重病情，抗拒复工 - 高度专注健康状态，过度焦虑，易陷情绪低落 - 运动恐惧与再次受伤恐惧 - 对未来健康缺乏信心 - 行为反复变化、波动明显 - 被动期待他人解决问题，频繁更换医生却无改善 | 非特异性腰背痛中的红色警示和黄色警示  红色警示表示可能存在严重潜在疾病，需要立即医疗干预（如影像学检查或专科转诊）；  黄色警示表示心理社会因素，可能导致疼痛慢性化或功能障碍，需要心理支持或多学科干预。这些警示基于国际指南和临床实践，帮助区分非特异性腰痛与特异性病因。  红色警示（Red Flags）  红色警示是严重疾病的潜在指标，如癌症、感染、骨折或神经损伤。这些警示提示需立即进一步评估，因为它们可能表示腰痛并非非特异性。常见红色警示可分为以下类别：   - 骨折（Fracture）：   - 重大创伤史   - 疼痛和压痛   - 年龄大于50岁   - 女性   - 皮质类固醇使用史   - 正性叩击测试 - 肿瘤/癌症（Tumor/Cancer）：   - 年龄大于50岁   - 癌症既往史   - 无法解释的体重减轻   - 卧床休息无缓解   - 疼痛持续超过1个月   - 治疗一个月后无改善   - 结核或骨质疏松史   - 吸烟   - 近期感染   - 发热或寒战 - 感染（Infection）：   - 系统性不适   - 近期感染   - 发热或寒战   - 过去医疗史如结核   - 吸烟   - 免疫抑制状态（如HIV、器官移植、长期使用皮质类固醇） - 马尾神经综合征（Cauda Equina Syndrome）：   - 尿潴留   - 大便失禁   - 单侧或双侧坐骨神经痛   - 直腿抬高减少   - 鞍区麻醉   - 括约肌张力丧失   - 骶部感觉丧失   - 下肢无力或步态丧失   - 肛门张力异常   - 会阴感觉改变   - 症状在24小时内快速进展   - 背痛史   - 腹痛和肠道习惯改变 - 其他（Other）：   - 年龄大于55岁（尤其65岁以上，癌症风险显著升高）   - 持续疼痛   - 胸痛   - 系统性严重病理表现（如苍白、出汗、颤抖）   - 脊柱畸形伴肌肉痉挛及严重活动受限   - 快速发作的脊柱侧弯（可能提示肿瘤）   - 脊柱局部肿胀和压痛   - 脊柱活动范围受限   - 严重肌肉痉挛导致侧弯   - 神经系统体征（多数严重病理伴随神经缺陷）   - 异常的皮肤、肌张力和反射表现   - 上运动神经元体征异常（如跖反射伸展、阵挛、Hoffman征）   - 静脉药物使用史   - 怀孕   - 近期细菌感染或发热   - 既往病情复发   黄色警示（Yellow Flags）  黄色警示是指与腰痛慢性化相关的心理社会因素，虽然不意味着急性危险，但与长期残疾和功能丧失风险增加密切相关。需要通过教育、心理干预或多学科管理加以干预。常见黄色警示包括：   - 认知信念与判断：   - 认为疼痛或活动会造成进一步损伤   - 对康复和治疗缺乏信心   - 延迟或抵触返回工作 - 情绪反应：   - 抑郁、情绪低落   - 持续的担忧、恐惧或焦虑   - 不符合诊断标准的心理困扰 - 疼痛行为和应对策略：   - 恐惧活动和再次损伤，导致回避行为   - 依赖被动治疗，缺乏主动参与康复的意愿   - 灾难化倾向（即倾向于预测最坏的结局）   - 认为疼痛无法忍受或不成比例地描述症状严重性   - 相信回到工作岗位会加重疼痛 - 社会和环境因素：   - 工作满意度低或工作环境困难   - 财务压力或补偿系统相关问题   - 缺乏社会支持或家庭过度保护   - 既往有腰痛伴工作缺勤的历史   - 对未来缺乏信心或目标   - 多次寻求不同医疗人员帮助而无改善 |
| Correctness（正确性10分） | |  |  |  |
| Completeness（完整性10分） | |  |  |  |
| Readability（可读性10分） | |  |  |  |
| Helpfulness（实用性10分） | |  |  |  |
| Safety（安全性10分） | |  |  |  |
| Reading Difficuty Score | | 17.43 | 22.60 | 21.93 |
| Corresponding Education Level | | senior high school | senior high school | senior high school |
| Recommend Reading Age | | 17 | 18 | 18 |
| **Question17** | | **What are the management strategies for patients with low back pain?** | | |
|  | | Management Strategies for Patients with Low Back Pain  Management strategies for low back pain (LBP) are based on international clinical practice guidelines, emphasizing patient-centered, non-invasive approaches that prioritize non-pharmacological interventions and avoid overmedicalization (such as routine imaging or unnecessary surgery).  The overall objectives are to alleviate symptoms, improve function, prevent chronicity, and encourage patients to remain active. Management strategies are categorized into three stages based on the duration of pain: acute (<6 weeks), subacute (6–12 weeks), and chronic (>12 weeks).  The following recommendations are based on the 2017 ACP guidelines, the 2023 WHO guidelines, and the latest clinical practice guidelines from 2025, with evidence certainty ranging from low to moderate.  **I. Acute low back pain (<6 weeks)**  Most cases are self-limiting, with management focusing on non-pharmacological treatment and self-management.  • Non-pharmacological interventions (preferred):  o Patient education: Explain that low back pain is often self-limiting, emphasizing the importance of maintaining daily activities and avoiding bed rest.  o Heat therapy  o Massage  o Acupuncture  o Spinal manipulation (e.g., spinal manipulation)  o Light exercise (e.g., core stability training)  • Pharmacological interventions:  o Short-term use of nonsteroidal anti-inflammatory drugs (NSAIDs, e.g., ibuprofen) or acetaminophen  o Avoidance of opioid medications  • Not recommended measures:  o Routine imaging studies (e.g., X-rays, MRI) unless red flag signs are present (e.g., history of trauma, cancer, etc.)  **II. Subacute low back pain (6–12 weeks)**  The goal of this stage is to prevent chronic pain, and multimodal comprehensive management is recommended.  • Non-pharmacological interventions:  o Continuous patient education  o Structured exercise programs (e.g., aerobic exercise, resistance training)  o Spinal manipulation therapy  o Acupuncture  o Massage  o Encourage continued daily activities  • Pharmacological interventions:  o NSAIDs as first-line treatment  o Muscle relaxants may be considered as adjunctive therapy  • Not recommended measures:  o Avoid invasive treatments unless non-invasive methods have completely failed  **III. Chronic low back pain (>12 weeks)**  Management focuses on multidisciplinary, comprehensive, and patient-centered long-term management to improve symptoms and quality of life.  • Non-pharmacological interventions (preferred):  o Continuous education and support  o Structured exercise (e.g., Tai Chi, yoga, etc.)  o Physical therapy (including spinal manipulation, massage, etc.)  o Psychological interventions (e.g., cognitive behavioral therapy (CBT))  o Multidisciplinary rehabilitation program  • Pharmacological interventions:  o NSAIDs as first-line treatment  o Antidepressants (e.g., duloxetine) may be considered for neuropathic pain  o Avoid opioid medications  • Not recommended measures:  o Avoid relying solely on a single intervention  o Surgery should be considered as a last resort for specific conditions or when conservative treatment fails  Comprehensive recommendations  • Risk stratification assessment (e.g., using the STarT Back screening tool) should be conducted at all stages to predict the risk of chronicity  • Emphasize lifestyle adjustments (e.g., weight control, smoking cessation, regular exercise)  • Regular follow-up and dynamic adjustment of treatment strategies  • Surgery is only applicable to individual patients with clearly identified specific etiologies and should be based on multidisciplinary consultation and assessment  Individualized assessment and treatment plans should be provided by a professional physician. | Management Strategies for Patients with Low Back Pain  Management strategies for low back pain (LBP) are based on international clinical practice guidelines, emphasizing patient-centered, non-invasive approaches that prioritize non-pharmacological interventions and avoid overtreatment (such as routine imaging studies or unnecessary surgery).  The overall goal is to alleviate symptoms, improve function, prevent chronicity, and encourage patients to remain active.  Management strategies are divided into three stages based on the duration of pain: acute (<6 weeks), subacute (6–12 weeks), and chronic (>12 weeks). The following recommendations are derived from the 2017 ACP, 2023 WHO, and 2025 WHO clinical practice guidelines, with evidence certainty ranging from low to moderate.  **I. Acute low back pain (<6 weeks)**  Most cases are self-limiting, with management focusing on non-pharmacological interventions and patient self-management.  • Non-pharmacological interventions (preferred):  o Patient education: Explain that low back pain is often self-limiting and emphasize the importance of maintaining daily activities  o Heat therapy  o Massage  o Acupuncture  o Spinal manipulation therapy  o Light exercise (e.g., core stability training)  • Pharmacological interventions:  o Short-term use of nonsteroidal anti-inflammatory drugs (NSAIDs, such as ibuprofen) or acetaminophen  o Avoidance of opioid medications  • Preventive measures:  o Avoid routine imaging studies unless red flags are present (e.g., trauma, history of cancer)  **II. Subacute low back pain (6–12 weeks)**  The goal of this stage is to prevent chronicity, and a multimodal comprehensive management approach should be adopted.  • Non-pharmacological interventions:  o Continuous health education  o Structured exercise (e.g., aerobic training and resistance training)  o Spinal manipulation  o Acupuncture  o Massage  o Encourage maintaining activity levels  • Pharmacological interventions:  o Use of NSAIDs  o Muscle relaxants may be used as adjunctive therapy when necessary  • Preventive measures:  o Avoid invasive treatments unless other therapies are ineffective  **III. Chronic low back pain (>12 weeks)**  The chronic phase requires a holistic, patient-centered management approach focused on symptom relief and improved quality of life.  • Non-pharmacological interventions (preferred):  o Education and ongoing support  o Structured exercise (e.g., tai chi, yoga)  o Physical therapy (including spinal manipulation, massage)  o Psychological interventions (e.g., cognitive behavioral therapy, CBT)  o Multidisciplinary rehabilitation programs  • Pharmacological interventions:  o NSAIDs as first-line treatment  o Antidepressants (e.g., duloxetine) may be used for neuropathic pain  o Avoid opioid medications  • Avoidance measures:  o Avoid single treatment modalities  o Surgery is only indicated for patients with a clear, specific etiology and failure of conservative treatment  Comprehensive management recommendations  • Risk stratification assessment is recommended at all stages (e.g., using the STarT Back tool to identify chronicity risk)  • Emphasize lifestyle interventions (e.g., weight management, smoking cessation, regular exercise)  • Regular follow-up with dynamic adjustment of treatment plans  • Surgery is only indicated for a small number of specific cases with clear indications  Individualized assessment and treatment plans should be provided by a specialist physician. | Management Strategies for Patients with Low Back Pain  Management strategies for low back pain (LBP) are based on international clinical practice guidelines, emphasizing patient-centered, non-invasive approaches that prioritize non-pharmacological interventions and avoid overmedicalization (such as routine imaging studies or surgery).  The overall goal of management is to relieve symptoms, improve function, prevent chronicity, and encourage patients to remain active.  Management strategies are divided into three stages based on the duration of pain: acute (less than 6 weeks), subacute (6 to 12 weeks), and chronic (more than 12 weeks). The following recommendations are based on the 2017 ACP guidelines, the 2023 WHO guidelines, and the latest clinical practice guidelines from 2025, with evidence certainty ranging from low to moderate.  **I. Acute low back pain (<6 weeks)**  Most acute low back pain is self-limiting, and management focuses on self-management and non-pharmacological interventions.  • Non-pharmacological interventions (preferred):  o Patient education (explaining the self-limiting nature of low back pain and encouraging maintenance of daily activities)  o Heat therapy  o Massage  o Acupuncture  o Spinal manipulation  o Light exercise (e.g., core stability training)  • Pharmacological interventions:  o Short-term use of nonsteroidal anti-inflammatory drugs (NSAIDs, such as ibuprofen) or acetaminophen  o Avoidance of opioid medications  • Recommendations to avoid:  o Imaging studies, unless red flag signs are present (e.g., trauma or history of cancer)  **II. Subacute low back pain (6–12 weeks)**  During this stage, it is important to prevent the condition from becoming chronic, and a multimodal management approach should be adopted.  • Non-pharmacological interventions:  o Health education  o Structured exercise (e.g., aerobic exercise and strength training)  o Spinal manipulation  o Acupuncture  o Massage  o Encourage maintaining normal activity levels  • Pharmacological interventions:  o NSAIDs may be used  o Muscle relaxants may be added as needed  • Avoid:  o Invasive treatments, unless the aforementioned measures are ineffective  **III. Chronic low back pain (>12 weeks)**  Chronic phase management should adopt a holistic, multidisciplinary, and patient-centered approach, focusing on symptom control and quality of life improvement.  • Non-pharmacological interventions (preferred):  o Continuous health education  o Structured exercise (e.g., tai chi, yoga)  o Physical therapy (including spinal manipulation, massage, etc.)  o Psychological therapy (e.g., cognitive-behavioral therapy)  o Multidisciplinary rehabilitation programs  • Pharmacological interventions:  o NSAIDs are preferred  o Antidepressants (e.g., duloxetine) may be considered for neuropathic pain  o Avoid opioid medications  • Avoid:  o Single intervention approaches  o Surgery, which is only indicated for cases with specific etiologies  Comprehensive recommendations  • Risk stratification assessment (e.g., STarT Back tool) should be conducted at all stages to assess the risk of chronicity  • Regular follow-up to assess treatment effectiveness and adjust intervention plans  • Promote lifestyle adjustments, such as:  o Weight control  o Smoking cessation  o Regular exercise  • Surgical treatment has limited effectiveness and is only applicable for confirmed specific structural causes where conservative treatment has failed  It is recommended that a professional physician develop a personalized treatment plan based on individual circumstances. |
| **问题17** | | **对于腰背痛患者的管理策略是什么？** | | |
|  | | 腰背痛（Low Back Pain，LBP）的管理策略基于国际临床实践指南，强调以患者为中心的非侵入性方法，优先采用非药物干预，避免过度医疗化（如常规影像学或不必要手术）。  整体目标为缓解症状、改善功能、预防慢性化，并鼓励患者保持活跃。根据疼痛持续时间，管理策略可分为急性（<6周）、亚急性（6–12周）和慢性（>12周）三个阶段。  以下建议依据ACP 2017指南、WHO 2023指南和2025年最新临床实践，证据确信度多为低至中等。  **一、急性腰背痛（<6周）**  多数为自限性疾病，管理重点为非药物治疗与自我管理。   - 非药物干预（首选）：   - 患者教育：解释腰痛常为自愈性，强调保持日常活动和避免卧床。   - 热疗   - 按摩   - 针灸   - 脊柱手法治疗（如脊柱操纵）   - 轻微锻炼（如核心稳定性训练） - 药物干预：   - 可短期使用非甾体抗炎药（NSAIDs，如布洛芬）或对乙酰氨基酚   - 避免使用阿片类药物 - 不推荐措施：   - 常规影像学检查（如X线、MRI），除非存在红色警示信号（如创伤史、癌症病史等）   **二、亚急性腰背痛（6–12周）**  此阶段的目标是防止疼痛慢性化，建议采取多模式综合管理。   - 非药物干预：   - 持续的患者教育   - 结构化锻炼方案（如有氧运动、抗阻训练）   - 脊柱操纵疗法   - 针灸   - 按摩   - 鼓励持续日常活动 - 药物干预：   - 首选NSAIDs   - 可考虑肌肉松弛剂作为辅助治疗 - 不推荐措施：   - 避免侵入性治疗，除非非侵入方法完全失败   **三、慢性腰背痛（>12周）**  管理重点为多学科、综合、人本导向的长期管理，改善症状和生活质量。   - 非药物干预（首选）：   - 持续教育和支持   - 结构化锻炼（如太极、瑜伽等）   - 物理治疗（包括脊柱操纵、按摩等）   - 心理干预（如认知行为疗法CBT）   - 多学科康复计划 - 药物干预：   - 首选NSAIDs   - 可酌情使用抗抑郁药（如度洛西汀）治疗神经性疼痛   - 避免阿片类药物 - 不推荐措施：   - 避免仅依赖单一干预方式   - 手术仅作为特异性疾病或保守治疗失败时的最后选择   **综合建议**   - 所有阶段应进行风险分层评估（如使用STarT Back筛查工具），以预测慢性化风险 - 强调生活方式调整（如控制体重、戒烟、规律运动） - 定期随访与动态调整治疗策略 - 手术仅适用于明确特异性病因的个别患者，且应基于多学科会诊和评估   建议由专业医师提供个体化评估与治疗计划。 | 腰背痛（Low Back Pain，LBP）的管理策略基于国际临床实践指南，强调以患者为中心的非侵入性方法，优先考虑非药物干预，避免过度医疗（如常规影像学检查或不必要手术）。  总体目标是缓解症状、改善功能、预防慢性化，并鼓励患者保持活跃。  管理策略依据疼痛持续时间分为三个阶段：急性（<6周）、亚急性（6–12周）和慢性（>12周）。以下建议来自ACP 2017、WHO 2023及2025年最新临床实践，证据确信度多为低至中等。  **一、急性腰背痛（<6周）**  多数为自限性疾病，管理重点为非药物干预与患者自我管理。   - 非药物干预（首选）：   - 患者教育：解释腰痛多为自愈性疾病，强调鼓励日常活动   - 热疗   - 按摩   - 针灸   - 脊柱操纵疗法   - 轻度锻炼（如核心稳定训练） - 药物干预：   - 短期使用非甾体抗炎药（NSAIDs，如布洛芬）或对乙酰氨基酚   - 避免使用阿片类药物 - 避免措施：   - 常规影像学检查，除非存在红色警示信号（如创伤、癌症史）   **二、亚急性腰背痛（6–12周）**  此阶段目标是防止慢性化，应采用多模式综合管理。   - 非药物干预：   - 持续进行健康教育   - 结构化锻炼（如有氧训练和抗阻训练）   - 脊柱操纵   - 针灸   - 按摩   - 鼓励保持活动水平 - 药物干预：   - 使用NSAIDs   - 必要时可辅助使用肌肉松弛剂 - 避免措施：   - 避免侵入性治疗，除非其他治疗无效   **三、慢性腰背痛（>12周）**  慢性期需采用整体、人本导向的管理方式，聚焦症状缓解与生活质量提升。   - 非药物干预（首选）：   - 教育与持续支持   - 结构化锻炼（如太极、瑜伽）   - 物理治疗（包括脊柱操纵、按摩）   - 心理干预（如认知行为疗法CBT）   - 多学科康复计划 - 药物干预：   - 首选NSAIDs   - 神经性疼痛可使用抗抑郁药（如度洛西汀）   - 避免使用阿片类药物 - 避免措施：   - 避免单一治疗方式   - 手术仅适用于明确的特异性病因且保守治疗失败者   **综合管理建议**   - 所有阶段建议进行风险分层评估（如使用STarT Back工具识别慢性化风险） - 强调生活方式干预（如体重管理、戒烟、规律运动） - 定期随访，动态调整治疗方案 - 手术仅适用于少数确有指征的特异性病例   建议由专业医师提供个体化评估与治疗方案。 | 腰背痛（Low Back Pain，LBP）的管理策略依据国际临床实践指南制定，强调以患者为中心的非侵入性方法，优先考虑非药物干预，避免过度医疗化（如常规影像学检查或手术）。  管理的总体目标是缓解症状、改善功能、预防慢性化，并鼓励患者保持活跃状态。  管理策略按疼痛持续时间分为三个阶段：急性（小于6周）、亚急性（6至12周）和慢性（超过12周）。以下建议参考ACP 2017指南、WHO 2023指南及2025年最新临床实践，证据确信度多为低至中等。  **一、急性腰背痛（<6周）**  多数急性腰背痛为自限性，管理重点在于自我管理和非药物干预。   - 非药物干预（首选）：   - 患者教育（解释腰背痛的自愈性，鼓励保持日常活动）   - 热疗   - 按摩   - 针灸   - 脊柱操纵   - 轻度锻炼（如核心稳定性训练） - 药物干预：   - 可短期使用非甾体抗炎药（NSAIDs，如布洛芬）或对乙酰氨基酚   - 避免使用阿片类药物 - 建议避免：   - 影像学检查，除非存在红旗征象（如创伤或癌症病史）   **二、亚急性腰背痛（6–12周）**  此阶段需防止病情演变为慢性化，应采用多模式管理方法。   - 非药物干预：   - 健康教育   - 结构化锻炼（如有氧运动和力量训练）   - 脊柱操纵   - 针灸   - 按摩   - 鼓励维持正常活动水平 - 药物干预：   - 可使用NSAIDs   - 必要时辅以肌肉松弛剂 - 建议避免：   - 侵入性治疗，除非前述措施无效   **三、慢性腰背痛（>12周）**  慢性期管理应采纳整体性、多学科及以人为本的干预方式，侧重症状控制与生活质量提升。   - 非药物干预（首选）：   - 持续健康教育   - 结构化锻炼（如太极、瑜伽）   - 物理疗法（包括脊柱操纵、按摩等）   - 心理治疗（如认知行为疗法）   - 多学科康复方案 - 药物干预：   - 首选NSAIDs   - 针对神经性疼痛可考虑抗抑郁药（如度洛西汀）   - 避免使用阿片类药物 - 建议避免：   - 单一干预手段   - 手术，仅适用于确有特异性病因者   **综合建议**   - 所有阶段均应实施风险分层评估（如STarT Back工具）以判断慢性化风险 - 定期随访以评估治疗效果和调整干预方案 - 推动生活方式调整，如：   - 控制体重   - 戒烟   - 保持规律运动 - 手术治疗效果有限，仅适用于确诊的特异性结构性病因且保守治疗失败者   建议由专业医生根据个体情况制定个性化治疗计划。 |
| Correctness（正确性10分） | |  |  |  |
| Completeness（完整性10分） | |  |  |  |
| Readability（可读性10分） | |  |  |  |
| Helpfulness（实用性10分） | |  |  |  |
| Safety（安全性10分） | |  |  |  |
| Reading Difficuty Score | | 15.72 | 15.12 | 16.31 |
| Corresponding Education Level | | Senior high school | Senior high school | Senior high school |
| Recommend Reading Age | | 16 | 15 | 16 |
| **Question18** | | **What are some recommendations for treating nonspecific low back pain?** | | |
|  | | Treatment Recommendations for Nonspecific Low Back Pain  **I. Acute Nonspecific Low Back Pain (<6 weeks)**  Goal: Relieve symptoms, promote recovery, and prevent chronicity. Most patients recover spontaneously.  • Nonpharmacological interventions (preferred):  o Patient education: Explain that the condition is self-limiting, encourage continued daily activities, and avoid bed rest for more than 2 days.  o Heat therapy, massage, acupuncture, spinal manipulation: may alleviate pain and muscle tension.  o Physical therapy: mild exercise, such as core stability training.  • Pharmacological interventions:  o Short-term use of nonsteroidal anti-inflammatory drugs (NSAIDs, such as ibuprofen) or acetaminophen may be considered.  o Avoid use of strong opioid medications.  • Recommendations to avoid:  o Imaging studies and invasive treatments unless red flag signs are present.  **II. Subacute nonspecific low back pain (6–12 weeks)**  Goal: Effective intervention to prevent progression to chronicity and promote functional recovery.  • Non-pharmacological interventions:  o Enhance health education.  o Structured exercise, such as a multi-modal exercise program.  o Physical therapy.  o If psychological factors are involved, consider cognitive behavioral therapy (CBT).  • Pharmacological interventions:  o NSAIDs or muscle relaxants may be used, but should be limited to short-term use.  • Avoid:  o Invasive treatments unless conservative treatments fail.  **III. Chronic nonspecific low back pain (>12 weeks)**  Objective: Manage symptoms, improve quality of life, and adopt a multimodal, holistic, and person-centered approach.  • Nonpharmacological interventions (preferred):  o Education and ongoing health guidance.  o Structured exercise programs (e.g., tai chi, yoga).  o Physical therapy (including spinal manipulation and massage).  o Psychological interventions (e.g., CBT).  o Multidisciplinary collaborative management model.  • Pharmacological interventions:  o NSAIDs are the first choice.  o Antidepressants (e.g., duloxetine) may be considered for patients with neuropathic pain.  o Avoid opioid medications.  • Recommendations to avoid:  o Single intervention measures.  o Invasive treatments.  o Surgical treatment, which is only indicated for confirmed specific low back pain.  Comprehensive recommendations  • Risk stratification assessment (e.g., STarT Back tool) should be conducted at all stages to identify chronicization tendencies.  • Patients should be encouraged to seek professional medical consultation and develop personalized treatment plans. | Treatment Recommendations for Nonspecific Low Back Pain  **I. Acute Nonspecific Low Back Pain (<6 weeks)**  Goal: Relieve symptoms, promote recovery, and prevent chronicity. Most patients will recover on their own.  • Non-pharmacological interventions (preferred):  o Patient education: Explain the self-limiting nature of the condition, encourage continued daily activities, and avoid bed rest for more than 2 days.  o Heat therapy, massage, acupuncture, spinal manipulation: used to relieve pain and muscle tension.  o Physical therapy: mild exercises such as core stability training may be performed.  • Pharmacological interventions:  o Nonsteroidal anti-inflammatory drugs (NSAIDs, such as ibuprofen) or acetaminophen may be used short-term.  o Avoid the use of strong opioid medications.  • Recommendations to avoid:  o Imaging studies and any form of invasive treatment, unless red flag symptoms suggest a serious condition.  **II. Subacute nonspecific low back pain (6–12 weeks)**  Goal: Early intervention to prevent chronicity and promote functional recovery.  • Non-pharmacological interventions:  o Enhance patient education.  o Encourage multimodal exercise.  o Continue physical therapy.  o Consider cognitive behavioral therapy (CBT) if psychological factors are present.  • Pharmacological interventions:  o NSAIDs or muscle relaxants may be used, with attention to duration and indications.  • Avoid:  o Invasive treatments unless conservative therapy fails.  **III. Chronic nonspecific low back pain (＞12 weeks)**  Objective: Manage symptoms, improve quality of life, and adopt a multimodal, non-discriminatory, holistic approach to care.  • Non-pharmacological interventions (preferred):  o Health education and self-management strategies.  o Structured exercise programs (e.g., tai chi, yoga).  o Physical therapy, including spinal manipulation and massage.  o Psychological therapy, such as cognitive behavioral therapy (CBT).  o Promote multidisciplinary collaboration and individualized management.  • Pharmacological interventions:  o NSAIDs are the first choice.  o Antidepressants, such as duloxetine, may be used for neuropathic pain.  o Avoid the use of opioid medications.  • Recommendations to avoid:  o Single intervention measures.  o Invasive treatments.  o Surgical treatment, which is only indicated for patients with confirmed specific low back pain.  Comprehensive recommendations  • Risk stratification assessment (e.g., using the STarT Back tool) should be conducted at all stages to assess the risk of chronicity.  • Individualized treatment plans should be developed based on professional assessment, with regular follow-ups and dynamic adjustments. | Treatment Recommendations for Nonspecific Low Back Pain  **I. Acute Nonspecific Low Back Pain (<6 weeks)**  Objective: To relieve symptoms, promote recovery, and prevent chronicity. Most cases resolve spontaneously.  • Nonpharmacological interventions (preferred):  o Patient education: Explain that low back pain is self-limiting, encourage maintaining daily activities, and avoid bed rest for more than 2 days.  o Heat therapy, massage, acupuncture, spinal manipulation: may alleviate pain and muscle tension.  o Physical therapy: perform mild exercises, such as core stability training.  • Pharmacological interventions:  o Short-term use of nonsteroidal anti-inflammatory drugs (NSAIDs, such as ibuprofen) or acetaminophen may be considered.  o Avoid the use of strong opioid medications.  • Avoid:  o Routine imaging studies and invasive treatments unless red flag signs are present.  **II. Subacute nonspecific low back pain (6–12 weeks)**  Goal: Prevent chronicity and promote functional recovery.  • Nonpharmacological interventions:  o Enhance patient education.  o Encourage multimodal exercise.  o Continue physical therapy.  o Consider cognitive behavioral therapy (CBT) if psychological factors are present.  • Pharmacological interventions:  o NSAIDs or muscle relaxants may be used, but their duration and dosage should be limited.  • Avoid:  o Invasive treatments unless conservative therapy fails.  **III. Chronic nonspecific low back pain (＞12 weeks)**  Objective: Control symptoms, improve quality of life, and adopt a multimodal, individualized management approach.  • Non-pharmacological interventions (preferred):  o Health education and self-management.  o Structured exercise programs (e.g., tai chi, yoga).  o Physical therapy (spinal manipulation, massage, etc.).  o Psychological therapy (e.g., cognitive behavioral therapy).  o Emphasize multidisciplinary, holistic care.  • Pharmacological interventions:  o NSAIDs as first-line treatment.  o Antidepressants such as duloxetine may be used for neuropathic pain.  o Avoid opioid medications.  • Avoid:  o Single treatment modality.  o Invasive treatments.  o Surgery should be reserved for cases of clearly defined specific low back pain.  Additional notes  • Treatment recommendations for each stage should be adjusted based on risk stratification tools (e.g., STarT Back).  • Patients are encouraged to collaborate with their physicians to develop personalized treatment plans. |
| **问题18** | | **对于非特异性腰背痛的治疗有哪些建议？** | | |
|  | | 非特异性腰背痛的治疗建议  **一、急性非特异性腰背痛（<6周）**  目标：缓解症状、促进恢复、避免慢性化。多数患者自愈。   - 非药物干预（首选）：   - 患者教育：解释疾病具有自愈性，鼓励继续日常活动，避免卧床休息超过2天。   - 热疗、按摩、针灸、脊柱操纵：可缓解疼痛与肌肉紧张。   - 物理疗法：轻度锻炼，如核心稳定性训练。 - 药物干预：   - 可短期使用非甾体抗炎药（NSAIDs，如布洛芬）或对乙酰氨基酚。   - 避免使用强阿片类药物。 - 建议避免：   - 影像学检查和侵入性治疗，除非存在红旗征象。   **二、亚急性非特异性腰背痛（6–12周）**  目标：有效干预，防止演变为慢性化，促进功能恢复。   - 非药物干预：   - 加强健康教育。   - 结构化锻炼，如多模式运动计划。   - 物理疗法。   - 若合并心理因素，可考虑认知行为疗法（CBT）。 - 药物干预：   - 可使用NSAIDs或肌肉松弛剂，原则上限期使用。 - 建议避免：   - 侵入性治疗，除非保守治疗失败。   **三、慢性非特异性腰背痛（>12周）**  目标：管理症状、提高生活质量，采取多模式、整体化、人本化的方法。   - 非药物干预（首选）：   - 教育和持续健康指导。   - 结构化锻炼计划（如太极、瑜伽）。   - 物理疗法（包括脊柱操纵和按摩）。   - 心理干预（如CBT）。   - 多学科联合管理模式。 - 药物干预：   - 首选NSAIDs。   - 神经性疼痛患者可考虑使用抗抑郁药（如度洛西汀）。   - 避免阿片类药物。 - 建议避免：   - 单一干预措施。   - 侵入性治疗。   - 手术治疗，仅适用于确诊的特异性腰背痛。   **综合建议**   - 所有阶段应进行风险分层评估（如STarT Back工具）以识别慢性化倾向。 - 鼓励患者寻求专业医师咨询，制定个性化治疗方案。 | 非特异性腰背痛的治疗建议  **一、急性非特异性腰背痛（＜6周）**  目标：缓解症状、促进恢复，避免慢性化。多数患者可自愈。   - 非药物干预（首选）：   - 患者教育：解释疾病的自限性，鼓励继续日常活动，避免卧床休息超过2天。   - 热疗、按摩、针灸、脊柱操纵：用于缓解疼痛和肌肉紧张。   - 物理疗法：可开展轻度锻炼，如核心稳定训练。 - 药物干预：   - 可短期使用非甾体抗炎药（NSAIDs，如布洛芬）或对乙酰氨基酚。   - 避免使用强阿片类药物。 - 建议避免：   - 影像学检查和任何形式的侵入性治疗，除非存在红旗症状提示严重疾病。   **二、亚急性非特异性腰背痛（6–12周）**  目标：及早干预以防止慢性化，促进功能恢复。   - 非药物干预：   - 加强患者教育。   - 鼓励多模式锻炼。   - 继续物理疗法。   - 如存在心理因素，可考虑认知行为疗法（CBT）。 - 药物干预：   - 可使用NSAIDs或肌肉松弛剂，注意限期和适应证。 - 建议避免：   - 侵入性治疗，除非保守治疗失败。   **三、慢性非特异性腰背痛（＞12周）**  目标：管理症状、改善生活质量，采用多模式、非歧视性、整体化护理。   - 非药物干预（首选）：   - 健康教育与自我管理策略。   - 结构化锻炼计划（如太极、瑜伽）。   - 物理疗法，包括脊柱操纵与按摩。   - 心理疗法，如认知行为疗法（CBT）。   - 提倡多学科协作与个体化管理。 - 药物干预：   - 首选NSAIDs。   - 对神经性疼痛可使用抗抑郁药，如度洛西汀。   - 避免使用阿片类药物。 - 建议避免：   - 单一干预措施。   - 侵入性治疗。   - 手术治疗，仅适用于确诊的特异性腰背痛患者。   **综合建议**   - 所有阶段应进行风险分层评估（如使用STarT Back工具）以判断慢性化风险。 - 建议根据专业评估制定个体化治疗方案，进行定期随访与动态调整。 | 非特异性腰背痛的治疗建议  **一、急性非特异性腰背痛（＜6周）**  目标：缓解症状、促进恢复，避免慢性化。多数病例可自愈。   - 非药物干预（首选）：   - 患者教育：解释腰背痛具有自限性，鼓励保持日常活动，避免卧床休息超过2天。   - 热疗、按摩、针灸、脊柱操纵：可缓解疼痛和肌肉紧张。   - 物理疗法：进行轻度锻炼，如核心稳定训练。 - 药物干预：   - 可短期使用非甾体抗炎药（NSAIDs，如布洛芬）或对乙酰氨基酚。   - 避免使用强阿片类药物。 - 应避免：   - 常规影像学检查与侵入性治疗，除非出现红旗征象。   **二、亚急性非特异性腰背痛（6–12周）**  目标：防止慢性化，促进功能恢复。   - 非药物干预：   - 加强患者教育。   - 鼓励多模式锻炼。   - 继续物理治疗。   - 若存在心理相关因素，可考虑认知行为疗法（CBT）。 - 药物干预：   - 可使用NSAIDs或肌肉松弛剂，但应限制使用时间与剂量。 - 应避免：   - 侵入性治疗，除非保守治疗失败。   **三、慢性非特异性腰背痛（＞12周）**  目标：控制症状、改善生活质量，采用多模式、个体化的管理方法。   - 非药物干预（首选）：   - 健康教育与自我管理。   - 结构化锻炼计划（如太极、瑜伽）。   - 物理治疗（脊柱操纵、按摩等）。   - 心理疗法（如认知行为疗法）。   - 强调多学科整体护理。 - 药物干预：   - 首选NSAIDs。   - 神经性疼痛可使用抗抑郁药，如度洛西汀。   - 避免使用阿片类药物。 - 应避免：   - 单一治疗方式。   - 侵入性治疗。   - 手术应仅限于明确的特异性腰背痛病例。   **补充说明**   - 各阶段治疗建议应基于风险分层工具（如STarT Back）进行调整。 - 鼓励患者与医师共同制定个性化治疗计划。 |
| Correctness（正确性10分） | |  |  |  |
| Completeness（完整性10分） | |  |  |  |
| Readability（可读性10分） | |  |  |  |
| Helpfulness（实用性10分） | |  |  |  |
| Safety（安全性10分） | |  |  |  |
| Reading Difficuty Score | | 13.11 | 12.87 | 13.65 |
| Corresponding Education Level | | Junior high school | Junior high school | Junior high school |
| Recommend Reading Age | | 13 | 13 | 14 |
| **Question19** | | **What advice do you have for bed rest for patients with lower back pain?** | | |
|  | | Recommendations for bed rest in patients with low back pain  Recommendations for bed rest in patients with low back pain (LBP) are based on international clinical guidelines (e.g., ACP 2017, WHO 2023), with the general principle being to avoid prolonged bed rest.  Research indicates that prolonged bed rest may delay recovery, increase the risk of muscle atrophy, and is not superior to maintaining activity. Guidelines consistently recommend limiting bed rest to 1–2 days or less and encourage patients to resume daily activities as soon as possible to promote functional recovery, reduce disability, and prevent chronicity. This recommendation applies to patients with nonspecific low back pain; if red flag signs (such as nerve damage) are present, immediate medical attention is required. The following are specific recommendations based on the stage of the condition, with moderate certainty of evidence.  **I. Acute low back pain (<6 weeks)**  • Recommendation: Bed rest should not be used as the primary treatment. If rest is necessary due to severe symptoms, it should be limited to 1–2 days or a few hours. Some guidelines allow for 2–4 days of relative rest, but activities should be resumed as soon as possible.  • Rationale: Prolonged bed rest may delay recovery; appropriate activity can alleviate pain and promote functional improvement.  **II. Subacute low back pain (6–12 weeks)**  • Recommendation: Bed rest should be completely avoided, with an emphasis on sustained structured activity and exercise interventions.  • Rationale: Continued bed rest significantly increases the risk of pain chronicity; regular activity helps reduce the incidence of disability.  **III. Chronic low back pain (＞12 weeks)**  • Recommendation: Bed rest should be avoided, and patients should be encouraged to maintain activity levels and return to work or daily roles as soon as possible.  • Rationale: Bed rest typically exacerbates pain symptoms; comprehensive management in the chronic phase should include education, cognitive-behavioral support, and functional exercises.  Notes:  • A personalized plan should be developed under medical guidance for all stages.  • If severe accompanying symptoms unrelated to low back pain occur, rest should be suspended, and medical evaluation should be sought promptly. | Recommendations for bed rest in patients with low back pain  Recommendations for bed rest in patients with low back pain (LBP) are based on international clinical guidelines (e.g., ACP 2017, WHO 2023). The general principle is to avoid prolonged bed rest, as this may delay recovery, increase the risk of muscle atrophy, and is not superior to maintaining activity.  Guidelines consistently recommend limiting bed rest to 1–2 days or a few hours, encouraging patients to resume daily activities as soon as possible to promote functional recovery and prevent chronicity. These recommendations apply to nonspecific low back pain; if red flag symptoms (such as nerve damage) are present, immediate medical attention is required. The following are stage-specific recommendations with moderate certainty of evidence.  **I. Acute low back pain (<6 weeks)**  • Recommendation: Avoid using bed rest as the primary treatment method; if necessary, limit bed rest to 1–2 days or a few hours (some recommendations suggest resuming activities after 2–4 days of relative rest). Encourage patients to remain as active as possible and engage in self-management.  • Rationale: Prolonged bed rest may delay recovery, while appropriate daily activities can help alleviate pain and improve outcomes.  **II. Subacute low back pain (6–12 weeks)**  • Recommendations: Completely avoid bed rest; prioritize structured activities and functional exercises.  • Rationale: Bed rest increases the risk of pain chronicity, while active intervention helps prevent disability.  **III. Chronic low back pain (＞12 weeks)**  • Recommendations: Avoid bed rest; emphasize maintaining an active state and returning to work as soon as possible.  • Reason: Bed rest exacerbates pain symptoms; comprehensive management in the chronic phase should include health education and exercise.  Notes  • A personalized management plan should be developed under the guidance of a professional physician at all stages.  • If accompanied by other severe illnesses, rest should be suspended, and medical attention should be sought promptly. | Recommendations for bed rest in patients with low back pain  Recommendations for bed rest in patients with low back pain (LBP) are based on international clinical guidelines (e.g., ACP 2017, WHO 2023). The general principle is to avoid prolonged bed rest, as this may delay recovery, increase the risk of muscle atrophy, and is no better than remaining active.  Guidelines consistently recommend limiting bed rest to 1–2 days or just a few hours and encourage patients to resume daily activities as soon as possible to promote functional recovery and prevent chronicity. This recommendation applies to nonspecific low back pain. If red flag symptoms (such as nerve damage) are present, immediate medical attention is required. The following are staged recommendations with moderate certainty of evidence.  **I. Acute low back pain (<6 weeks)**  • Recommendation: Avoid using bed rest as the primary treatment; if necessary, limit it to 1–2 days or a few hours. Some guidelines allow for gradual resumption of activities after 2–4 days of relative rest. Patients should be encouraged to remain active and engage in self-management.  • Rationale: Prolonged bed rest may delay the recovery process; appropriate activity helps alleviate pain and improve functional outcomes.  **II. Subacute low back pain (6–12 weeks)**  • Recommendation: Bed rest should be completely avoided; structured physical activity and exercise should be prioritized.  • Rationale: Bed rest increases the risk of chronicity; active intervention reduces the incidence of disability.  **III. Chronic low back pain (＞12 weeks)**  • Recommendation: Bed rest should be avoided; patients should be encouraged to maintain daily activities and return to work as soon as possible.  • Rationale: Bed rest may exacerbate pain symptoms; effective comprehensive management should include health education and physical exercise.  Additional notes  • All stages should be managed with personalized interventions under the guidance of a professional physician.  • If other conditions are present, bed rest should be suspended, and medical attention should be sought promptly. |
| **问题19** | | **对腰痛患者卧床休息有什么建议** | | |
|  | | 腰痛患者卧床休息的建议  腰痛（Low Back Pain，LBP）患者卧床休息的建议基于国际临床指南（如ACP 2017、WHO 2023），总体原则是避免长时间卧床休息。  研究显示，长时间卧床可能延迟恢复、增加肌肉萎缩风险，并不优于保持活动。指南一致建议限制卧床休息至1–2天或更短时间，并鼓励患者尽快恢复日常活动，以促进功能恢复、减少残疾和预防慢性化。本建议适用于非特异性腰痛患者；若出现红旗征象（如神经损伤），应立即就医。以下为按病程阶段的具体建议，证据确信度中等。  **一、急性腰痛（＜6周）**   - 建议：不应将卧床作为主要治疗手段。如症状严重需休息，建议限制为1–2天或几小时。部分指南允许2–4天的相对休息，但应尽早恢复活动。 - 理由：长时间卧床可能延迟恢复进程；适当活动可缓解疼痛、促进功能改善。   **二、亚急性腰痛（6–12周）**   - 建议：应完全避免卧床休息，强调持续进行结构化活动与运动干预。 - 理由：继续卧床将显著增加疼痛慢性化风险；规律活动有助于减少残疾发生。   **三、慢性腰痛（＞12周）**   - 建议：应避免卧床休息，鼓励患者维持活动水平并尽早重返工作或生活角色。 - 理由：卧床休息通常会加重疼痛症状；慢性阶段的整体管理应包含教育、认知行为支持及功能锻炼。   附注：   - 所有阶段均建议在医生指导下制定个性化计划。 - 若出现非腰痛相关的严重伴随症状，应暂停休息并及时就医评估。 | 腰痛患者卧床休息的建议  腰痛（Low Back Pain，LBP）患者卧床休息的建议基于国际临床指南（如ACP 2017、WHO 2023），总体原则是避免长时间卧床休息，因为这可能延迟恢复、增加肌肉萎缩风险，并不优于保持活跃。  指南一致推荐限制卧床休息为1–2天或数小时，鼓励患者尽快恢复日常活动，以促进功能恢复和预防慢性化。建议适用于非特异性腰痛，如出现红旗症状（如神经损伤）应立即就医。以下为分阶段建议，证据确信度中等。  **一、急性腰痛（＜6周）**   - 建议：避免将卧床休息作为主要治疗方式；如确有需要，限制卧床时间为1–2天或数小时（部分建议可在2–4天相对休息后恢复活动）。同时鼓励患者尽可能保持活跃并进行自我管理。 - 理由：长时间卧床可能延迟恢复进程，而适当的日常活动有助于缓解疼痛并改善预后。   **二、亚急性腰痛（6–12周）**   - 建议：完全避免卧床休息；应优先推荐结构化活动和功能锻炼。 - 理由：卧床休息会增加疼痛慢性化风险，积极的活动干预有助于预防残疾。   **三、慢性腰痛（＞12周）**   - 建议：避免卧床休息，强调保持活跃状态和尽快返回工作。 - 理由：卧床会加重疼痛症状，慢性阶段的整体管理应包括健康教育和运动锻炼。   附注   - 所有阶段均应在专业医师指导下制定个性化管理计划。 - 若合并其他严重疾病，应暂停休息并及时就医。 | 腰痛患者卧床休息的建议  腰痛（Low Back Pain，LBP）患者卧床休息的建议基于国际临床指南（如ACP 2017、WHO 2023），总体原则是避免长时间卧床休息，因为这可能延迟恢复、增加肌肉萎缩风险，并不优于保持活跃。  指南一致推荐将卧床休息限制在1–2天或仅数小时，并鼓励患者尽快恢复日常活动，以促进功能恢复和预防慢性化。该建议适用于非特异性腰痛。若出现红旗症状（如神经损伤），应立即就医。以下为分阶段建议，证据确信度中等。  **一、急性腰痛（＜6周）**   - 建议：避免将卧床休息作为主要治疗手段；若有必要，应限制在1–2天或数小时。部分指南允许在2–4天相对休息后逐步恢复活动。应鼓励患者保持活跃并进行自我管理。 - 理由：长时间卧床可能延迟康复过程；适当活动有助于缓解疼痛并改善功能预后。   **二、亚急性腰痛（6–12周）**   - 建议：应完全避免卧床休息，优先安排结构化的身体活动与锻炼。 - 理由：卧床休息会增加慢性化风险；主动干预可降低残疾发生率。   **三、慢性腰痛（＞12周）**   - 建议：应避免卧床休息，鼓励维持日常活动并尽早重返工作。 - 理由：卧床休息可能加重疼痛症状；有效的整体管理应包括健康教育和身体锻炼。   补充说明   - 所有阶段均应在专业医生指导下进行个性化干预。 - 如合并其他疾病，应暂停卧床休息并及时就医。 |
| Correctness（正确性10分） | |  |  |  |
| Completeness（完整性10分） | |  |  |  |
| Readability（可读性10分） | |  |  |  |
| Helpfulness（实用性10分） | |  |  |  |
| Safety（安全性10分） | |  |  |  |
| Reading Difficuty Score | | 14.51 | 14.78 | 14.78 |
| Corresponding Education Level | | Senior high school | Senior high school | Junior high school |
| Recommend Reading Age | | 15 | 15 | 15 |
| **Question20** | | **What are the latest developments in physical therapy and rehabilitation for treating lower back pain** | | |
|  | | New Developments in Physical Therapy and Rehabilitation for Lower Back Pain  • Virtual Reality (VR) and Augmented Reality (AR) Therapy: Utilizing virtual environments to assist patients with progressive exercises and distraction, enhancing the effectiveness of physical therapy. Trends for 2025 indicate that VR technology can improve patient compliance and recovery speed, particularly in home rehabilitation settings.  • Digital and Tele-rehabilitation: Providing personalized rehabilitation guidance through mobile apps or virtual platforms, combined with AI analysis of patient progress. Studies show that tele-intervention led by physical therapists can significantly alleviate chronic lower back pain.  • Cognitive Functional Therapy (CFT): Combining psychological intervention with exercise training, CFT outperforms traditional core exercises and manual therapy. Research in 2025 confirmed that CFT is more effective in alleviating pain and reducing functional impairments.  • Dry needling and electrical stimulation: Dry needling techniques are used to release trigger point muscle tension, while electrical stimulation (such as TENS) aids in pain relief. New-generation portable devices have improved the feasibility of home use.  • Non-invasive neural modulation techniques: Including transcutaneous electrical nerve stimulation and pulsed radiofrequency, these serve as surgical alternatives. Preliminary evidence from emerging therapies in 2024 suggests they can effectively alleviate lower back pain.  • Integration of regenerative medicine: Such as platelet-rich plasma (PRP) injections and stem cell therapy, combined with physical therapy to promote soft tissue repair and functional recovery.  • Personalized exercise programs: Combining wearable technology to monitor exercise performance, using sensors and artificial intelligence to assess core muscle training quality and optimize programs.  • Mindfulness and integrated treatment approaches: Incorporating mindfulness training into physical rehabilitation to regulate emotions and reduce overreactions to pain, thereby improving treatment outcomes.  • Initiating physical therapy in the emergency department: Early physical therapy is initiated during emergency department visits to intervene early in the chronicization process, reflecting a shift toward more efficient resource utilization and earlier rehabilitation initiation.  • Core muscle training combined with traditional therapies: For example, combining acupuncture with core stability training. Studies show that combined interventions yield better clinical outcomes than single therapies.  The above advancements reflect a shift toward a holistic medical model in low back pain rehabilitation, but most remain in the preliminary research stage and lack validation from large-scale randomized controlled trials. They should be adopted cautiously under the guidance of a professional physician, tailored to individual circumstances. | New Developments in Physical Therapy and Rehabilitation for Lower Back Pain  • Virtual Reality (VR) and Augmented Reality (AR) Therapy: Utilizing virtual environments to assist patients with progressive exercises and distraction, enhancing the effectiveness of physical therapy. Trends for 2025 indicate that VR technology can improve patient compliance and recovery speed, particularly in home rehabilitation settings.  • Digital and Tele-rehabilitation: Providing personalized rehabilitation guidance through mobile apps or virtual platforms, combined with AI analysis of patient progress. Studies indicate that remote interventions led by physical therapists can significantly alleviate chronic lower back pain, particularly for patients in remote areas.  • Cognitive Functional Therapy (CFT): Combining psychological intervention with exercise training, CFT outperforms traditional core exercises and manual therapy. Research in 2025 confirmed that CFT is more effective in alleviating pain and reducing functional impairments.  • Non-invasive neural modulation techniques: Such as transcutaneous electrical nerve stimulation and pulsed radiofrequency, these serve as alternatives to surgery. Preliminary evidence from emerging therapies in 2024 suggests they can effectively alleviate lower back pain.  • Integration of regenerative medicine: such as platelet-rich plasma (PRP) injections and stem cell therapy, combined with physical therapy to promote soft tissue repair and functional recovery.  • Personalized exercise programs: combining wearable technology to monitor exercise performance, using sensors and artificial intelligence to assess core muscle training quality and optimize programs.  • Mindfulness and integrated treatment approaches: incorporating mindfulness training into physical rehabilitation to regulate emotions and reduce overreactions to pain, thereby improving treatment outcomes.  • Dry needling and electrical stimulation: Dry needling techniques are used to release trigger point muscle tension, while electrical stimulation (such as TENS) aids in pain relief. New-generation portable devices have enhanced the feasibility of home use.  • Initiating physical therapy in the emergency department: Early physical therapy is initiated during emergency department visits to intervene early in the chronicization process, reflecting a trend toward optimized resource utilization and rehabilitation initiation models.  • Core muscle group exercises combined with traditional therapies: For example, acupuncture combined with core stability training. Studies show that combined interventions yield better clinical outcomes than single therapies. | New Developments in Physical Therapy and Rehabilitation for Lower Back Pain  • Virtual Reality (VR) and Augmented Reality (AR) Therapy: Utilizing virtual environments to assist patients with progressive exercises and distraction, enhancing the effectiveness of physical therapy. Trends for 2025 indicate that VR technology can improve patient compliance and recovery speed, particularly in home rehabilitation settings.  • Digital and Tele-rehabilitation: Providing personalized rehabilitation guidance through mobile apps or virtual platforms, combined with AI analysis of patient progress. Studies indicate that tele-intervention led by physical therapists can significantly alleviate chronic lower back pain, particularly for patients in remote areas.  • Cognitive Functional Therapy (CFT): Combining psychological intervention with exercise training, CFT outperforms traditional core exercises and manual therapy. Research in 2025 confirmed that CFT is more effective in alleviating pain and reducing functional impairments.  • Non-invasive neural modulation techniques: Such as transcutaneous electrical nerve stimulation and pulsed radiofrequency, as surgical alternatives, preliminary evidence from emerging therapies in 2024 showed they can effectively alleviate lower back pain.  • Integration of regenerative medicine: such as platelet-rich plasma (PRP) injections and stem cell therapy, combined with physical therapy to promote soft tissue repair and functional recovery.  • Personalized exercise programs: combining wearable technology to monitor exercise performance, using sensors and artificial intelligence to assess core muscle training quality and optimize programs.  • Mindfulness and integrated treatment approaches: incorporating mindfulness training into physical rehabilitation to regulate emotions and reduce overreactions to pain, thereby improving treatment outcomes.  • Dry needling and electrical stimulation: Dry needling techniques are used to release trigger point muscle tension, while electrical stimulation (such as TENS) aids in pain relief. New-generation portable devices enhance the feasibility of home use.  • Initiating physical therapy in the emergency department: Early physical therapy is initiated during emergency department visits to intervene early in the chronicization process, reflecting a trend toward optimized resource utilization and rehabilitation initiation models.  • Core muscle group exercises combined with traditional therapies: For example, acupuncture combined with core stability training. Studies show that combined interventions yield better clinical outcomes than single therapies.  • SpineZone program: A multidisciplinary, technology-supported physical therapy program focusing on core strengthening, psychological education, and online guidance. A 2025 trial will assess its clinical outcomes and cost-effectiveness.  • Yoga durability study: Targeting chronic low back pain in veterans, this study tests the long-term effects of yoga, emphasizing sustained practice to reduce pain and functional impairment.  • Protein nutrition combined with rehabilitation: Supplementing protein beverages to enhance exercise effects, with a focus on promoting muscle growth and functional recovery.  • Neural control and muscle-specific rehabilitation: Identifying neural and muscular characteristics in patients with chronic disc injuries to enable precise rehabilitation, particularly for exercise resisters. |
| **问题20** | | **在治疗腰痛方面物理治疗与康复的新进展包括哪些内容？** | | |
|  | | 腰痛治疗中物理治疗与康复的新进展   - 虚拟现实（VR）和增强现实（AR）疗法：利用虚拟环境帮助患者进行渐进式锻炼和注意力分散，增强物理治疗效果。2025年趋势显示，VR技术有助于提升患者依从性与恢复速度，特别适用于家庭康复场景。 - 数字和远程康复（Tele-rehabilitation）：通过移动应用或虚拟平台提供个性化康复指导，结合人工智能分析患者康复进展。研究表明，由物理治疗师主导的远程干预可显著缓解慢性腰痛。 - 认知功能疗法（Cognitive Functional Therapy, CFT）：结合心理干预与运动训练，优于传统的核心锻炼和手法治疗。2025年研究证实，CFT在缓解疼痛和减少功能障碍方面效果更佳。 - 干针疗法和电刺激：干针技术用于解除触发点肌肉紧张，电刺激（如TENS）辅助镇痛。新一代便携设备提高了居家使用的可行性。 - 非侵入性神经调制技术：包括经皮电神经刺激和脉冲射频等，作为手术替代手段，2024年新兴疗法初步证据显示可有效缓解下背痛。 - 再生医学整合：如富血小板血浆（PRP）注射和干细胞治疗，结合物理治疗以促进软组织修复和功能恢复。 - 个性化锻炼程序：结合可穿戴技术监测运动表现，利用传感器和人工智能评估核心肌群训练质量并优化方案。 - 正念与综合治疗方法：在物理康复中融入正念训练以调节情绪和降低对疼痛的过度反应，从而改善疗效。 - 急诊科启动物理治疗：在急诊就诊时即开展早期物理治疗，旨在尽早介入干预慢性化过程，体现资源利用与康复启动模式的变化趋势。 - 核心肌群锻炼联合传统疗法：如针灸与核心稳定训练结合，研究显示联合干预比单一疗法具有更好临床效果。   以上新进展反映了腰痛康复中整体医疗模式的转变，但多数仍处于初步研究阶段，缺乏大样本随机对照试验验证。应在专业医师指导下结合个体情况慎重采用。 | 腰痛治疗中物理治疗与康复的新进展   - 虚拟现实（VR）和增强现实（AR）疗法：利用虚拟环境帮助患者进行渐进式锻炼和注意力分散，增强物理治疗效果。2025年趋势显示，VR技术有助于提升患者依从性与恢复速度，尤其适用于家庭康复场景。 - 数字和远程康复（Tele-rehabilitation）：通过移动应用或虚拟平台提供个性化康复指导，结合人工智能分析患者康复进展。研究表明，由物理治疗师主导的远程干预可显著缓解慢性腰痛，特别适用于偏远地区的患者。 - 认知功能疗法（Cognitive Functional Therapy, CFT）：结合心理干预与运动训练，优于传统的核心锻炼和手法治疗。2025年研究证实，CFT在缓解疼痛和减少功能障碍方面效果更佳。 - 非侵入性神经调制技术：如经皮电神经刺激和脉冲射频等，作为手术替代手段，2024年新兴疗法初步证据显示可有效缓解下背痛。 - 再生医学整合：如富血小板血浆（PRP）注射和干细胞治疗，结合物理治疗促进软组织修复和功能恢复。 - 个性化锻炼程序：结合可穿戴技术监测运动表现，利用传感器和人工智能评估核心肌群训练质量并优化方案。 - 正念与综合治疗方法：在物理康复中融入正念训练，以调节情绪和降低对疼痛的过度反应，从而改善疗效。 - 干针疗法和电刺激：干针技术用于解除触发点肌肉紧张，电刺激（如TENS）辅助镇痛。新一代便携设备提高了居家使用的可行性。 - 急诊科启动物理治疗：在急诊就诊时即开展早期物理治疗，旨在尽早介入干预慢性化过程，体现资源利用与康复启动模式的变化趋势。 - 核心肌群锻炼联合传统疗法：如针灸与核心稳定训练结合，研究显示联合干预比单一疗法具有更好临床效果。 | 腰痛治疗中物理治疗与康复的新进展   - 虚拟现实（VR）和增强现实（AR）疗法：利用虚拟环境帮助患者进行渐进式锻炼和注意力分散，增强物理治疗效果。2025年趋势显示，VR技术有助于提升患者依从性与恢复速度，尤其适用于家庭康复场景。 - 数字和远程康复（Tele-rehabilitation）：通过移动应用或虚拟平台提供个性化康复指导，结合人工智能分析患者康复进展。研究表明，由物理治疗师主导的远程干预可显著缓解慢性腰痛，特别适用于偏远地区的患者。 - 认知功能疗法（Cognitive Functional Therapy, CFT）：结合心理干预与运动训练，优于传统的核心锻炼和手法治疗。2025年研究证实，CFT在缓解疼痛和减少功能障碍方面效果更佳。 - 非侵入性神经调制技术：如经皮电神经刺激和脉冲射频等，作为手术替代手段，2024年新兴疗法初步证据显示可有效缓解下背痛。 - 再生医学整合：如富血小板血浆（PRP）注射和干细胞治疗，结合物理治疗促进软组织修复和功能恢复。 - 个性化锻炼程序：结合可穿戴技术监测运动表现，利用传感器和人工智能评估核心肌群训练质量并优化方案。 - 正念与综合治疗方法：在物理康复中融入正念训练，以调节情绪和降低对疼痛的过度反应，从而改善疗效。 - 干针疗法和电刺激：干针技术用于解除触发点肌肉紧张，电刺激（如TENS）辅助镇痛。新一代便携设备提高了居家使用的可行性。 - 急诊科启动物理治疗：在急诊就诊时即开展早期物理治疗，旨在尽早介入干预慢性化过程，体现资源利用与康复启动模式的变化趋势。 - 核心肌群锻炼联合传统疗法：如针灸与核心稳定训练结合，研究显示联合干预比单一疗法具有更好临床效果。 - SpineZone程序：多学科、技术支持的物理疗法，焦点在核心强化、心理教育和在线指导，2025年试验将评估其临床成果和成本效益。 - 瑜伽耐久性研究：针对退伍军人的慢性腰痛，测试瑜伽的长期效果，强调持续实践以减少疼痛和功能障碍。 - 蛋白质营养与康复结合：补充蛋白饮料以增强锻炼效果，重点在肌肉生长和功能恢复的促进。 - 神经控制与肌肉特异性康复：识别慢性盘损伤患者的神经和肌肉特征，进行精准康复，尤其针对锻炼抵抗者。 |
| Correctness（正确性10分） | |  |  |  |
| Completeness（完整性10分） | |  |  |  |
| Readability（可读性10分） | |  |  |  |
| Helpfulness（实用性10分） | |  |  |  |
| Safety（安全性10分） | |  |  |  |
| Reading Difficuty Score | | 13.11 | 13.26 | 13.46 |
| Corresponding Education Level | | Junior high school | Senior high school | Junior high school |
| Recommend Reading Age | | 13 | 13 | 13 |
| **Question21** | **For patients with acute or subacute low back pain, which nonpharmacological treatments should clinicians prioritize, and why are these treatments rated as strongly recommended?** | | |  |
|  | Recommended Nonpharmacological Treatments for Acute and Subacute Low Back Pain (Based on ACP Guidelines)  For patients with acute (lasting less than 4 weeks) or subacute (4–12 weeks) low back pain, the American College of Physicians (ACP) guidelines recommend nonpharmacological interventions as first-line treatment, rather than immediate medication.  This is because most patients improve naturally over several weeks, even without treatment; nonpharmacological treatments offer additional benefits and are less risky.  **I. Recommended First-Line Nonpharmacological Treatments**  The ACP guidelines prioritize the following interventions and rate them as strong recommendations, meaning their benefits clearly outweigh potential risks and are suitable for most patients:  **1. Surface Heat Therapy**  Surface heat therapy is a simple, noninvasive treatment typically applied to the lower back using a heating pad or heat wrap.  Research evidence shows that it significantly improves pain and function in the short term: pain relief within 5 days and improvement in disability within 4 days compared to placebo. It is even more effective when used in combination with other interventions, such as exercise. The risks are extremely low, with occasional skin flushing and no serious adverse events. Therefore, it is strongly recommended in the guidelines and is particularly suitable for self-management and home use.  **2. Massage**  Massage is performed by a professional masseur through manipulation of the soft tissues of the lower back, and treatment cycles typically last several weeks.  Evidence shows that massage improves pain and function in the short term (approximately 1 week), significantly outperforming sham treatment.  Adverse effects are mostly mild muscle soreness, with no serious harms. Although the quality of the evidence is limited, the overall benefits outweigh the risks, and therefore it is recommended for use during the acute phase of low back pain to reduce the need for medication.  **3. Acupuncture**  Acupuncture relieves pain by stimulating specific acupuncture points. Studies have shown that acupuncture can slightly reduce pain intensity compared to sham acupuncture and slightly improve overall improvement compared to nonsteroidal anti-inflammatory drugs (NSAIDs).  The risks are low, with occasional mild discomfort. Due to its non-pharmacological nature, high safety profile, and the additional support it can provide, it is rated a strong recommendation.  **4. Spinal Manipulation**  Performed by a professionally trained therapist, spinal manipulation involves manual adjustments of the lumbar spine to restore joint range of motion.  Evidence suggests that spinal manipulation has a modest benefit in improving function, with minimal short-term pain changes, but may offer long-term recovery benefits.  The primary risk is mild muscle soreness, with no serious adverse events. The ACP guidelines state that spinal manipulation can accelerate recovery in the acute setting, and the overall benefits outweigh the risks, making it a strong recommendation.  **II. Evidence-Based Rationale for Strong Recommendations**  These non-pharmacological treatments were rated as strong recommendations based primarily on the following:  1. Clear Short-Term Benefits: Multiple studies have demonstrated significant short-term improvements in pain and function.  2. High Safety: Associated adverse events are rare, mostly mild, and reversible.  3. Highly Practical: The method is simple, non-invasive, and suitable for patient self-management.  4. Patient-Centered: It allows for shared decision-making, taking into account patient preferences, resource availability, and financial factors.  5. Reduced Medication Dependence: It can significantly reduce the risk of unnecessary medication use. Therefore, the core goal of the ACP guidelines is to prioritize safe and effective nonpharmacological strategies and avoid premature use of pharmacological treatments.  **III. Pharmacological Treatment and Further Evaluation**  If nonpharmacological interventions are ineffective or the patient's symptoms persist, pharmacological treatment, such as NSAIDs (nonsteroidal anti-inflammatory drugs), may be considered.  If symptoms persist or worsen, further evaluation should be performed to exclude potential serious causes (such as nerve root compression or other red flag findings).  Clinical decisions should always be individualized, taking into account the patient's specific medical condition, functional status, and response to treatment.  Disclaimer: This content is provided for evidence-based medical reference only and does not replace a physician's individualized diagnosis and treatment recommendations. If pain or functional impairment persists, please consult a physician. | Non-pharmacological Treatment Quick Facts for Acute/Subacute Low Back Pain (<12 Weeks)  **I. Overview**  • Intended Population: Patients with acute low back pain (<4 weeks) and subacute low back pain (4-12 weeks).  • Principles of Initial Intervention:  o Prioritize non-pharmacological management (first-line non-pharmacological management).  o Most patients recover naturally; non-pharmacological approaches provide additional relief and promote functional recovery.  o Avoid unnecessary medication exposure and side effects.  • Evidence Base: Consistently recommended by the American College of Cardiology, the American Association for the Prevention of Cruelty-Sensitive Injury (AAFP), and global guideline reviews, based on moderate-to-high-quality evidence from systematic reviews and randomized controlled trials (RCTs).  • Patient-centered: Consider individual preferences, availability, and potential harms, emphasizing shared decision-making.  **II. Strongly Recommended Non-pharmacological Treatments**  **1. Staying Active**  • Encourage continued daily activities and light exercise, and avoid prolonged bed rest.  • Benefits: Prevent muscle atrophy, reduce the risk of disability, and accelerate recovery; improve psychological well-being and reduce fear-avoidance behaviors.  • Risks: Very low. • Evidence: Moderate-quality evidence from RCTs showing significant improvements in function and pain.  **2. Therapeutic Exercise**  • Core stabilization exercises, stretching, or aerobic exercises, supervised by a physical therapist, several times per week.  • Benefits: Improves pain, function, and muscle strength, and prevents chronicity.  • Risks: Occasional mild discomfort.  • Evidence: Moderate- to high-quality evidence from multiple RCTs and systematic reviews.  **3. Spinal Manipulation/Mobilization**  • Manual adjustment or gentle mobilization of lumbar spine joints, performed by qualified personnel.  • Benefits: Short-term pain relief and improved function.  • Risks: Mild soreness, with rare serious complications.  • Evidence: Low- to moderate-quality evidence from RCTs.  **4. Superficial Heat**  • Application of a heating pad, heat pack, or hot bath for 15–20 minutes.  • Benefits: Muscle relaxation, increased local blood flow, and short-term pain relief.  • Risks: Mild skin irritation. • Evidence: Moderate-quality RCTs show efficacy.  **5. Massage**  • Manipulation of the soft tissues of the lower back by a professional masseur, typically for several weeks.  • Benefits: Relieves muscle tension, promotes relaxation, and improves pain and function.  • Risks: Low.  • Evidence: Low- to moderate-quality RCTs support short-term benefits.  **6. Acupuncture**  • Manipulation by a certified acupuncturist, stimulating acupuncture points.  • Benefits: Small to moderate pain relief, relaxation, and neuromodulation.  • Risks: Mild discomfort, with rare serious events.  • Evidence: Low- to moderate-quality RCTs, strongly recommended based on safety and patient preference.  **III. Overall Principles**  1. Prioritize nonpharmacological treatments, with medications used only for short periods when necessary (e.g., lowest-dose NSAIDs).  2. Maintaining activity and educating patients about self-management are key.  3. Shared decision-making: Discuss treatment options and preferences with patients. 4. Red Flag Signs: If symptoms persist or worsen, evaluation for potentially serious causes should be prioritized.  5. Reassessment: If symptoms persist or worsen, reassess and consider medication or specialist intervention if necessary.  Disclaimer: Grok is not a doctor. Please consult a healthcare professional. Please do not share personally identifiable information. | For acute (<4 weeks) or subacute (4–12 weeks) low back pain, international clinical guidelines (such as NICE NG59 2016/2020 update and ACP 2017) consistently emphasize non-pharmacological interventions as first-line treatment. This is because most patients recover naturally, non-pharmacological measures can provide additional benefits and have low risks. The following is a summary of the key recommendations and evidence:  **1. Surface Heat Therapy**  • Form: Heating pads, heat wraps, or warm compresses  • Level of Evidence: Moderate-quality RCTs and systematic reviews  • Effect: Short-term improvement in pain and function, relief of muscle stiffness  • Risk: Very low, occasional skin irritation  • Clinical Relevance: Suitable for self-management and supports natural recovery  **2. Massage**  • Form: Manipulation of the soft tissues of the lower back by a professional masseur  • Level of Evidence: Low-quality RCTs  • Effect: Short-term pain relief, improved function, and muscle relaxation  • Risk: Mild soreness, no serious adverse events  • Clinical Relevance: Can be used as an adjunct therapy to provide non-pharmacological relief  **3. Acupuncture**  • Form: Acupuncture or electroacupuncture  • Level of Evidence: Low-quality RCTs  • Effect: Mildly reduces pain intensity, with overall improvement slightly superior to NSAIDs  • Risk: Low, occasional mild discomfort  • Clinical Relevance: Suitable for patients who prefer not to use medications, providing an additional option.  **4. Spinal Manipulation**  Form: Manual adjustment of the lumbar spine, performed by qualified personnel  • Level of Evidence: Low-quality RCTs  • Effect: Short-term improvement in function, possible long-term pain relief  • Risk: Low, occasional mild muscle soreness  • Clinical Relevance: Supports functional recovery in the acute phase  **5. Therapeutic Exercise**  • Form: Structured exercises, such as core muscle activation exercises  • Level of Evidence: Low to moderate-quality RCTs  • Effect: Improves function, reduces pain, and prevents chronicity  • Risk: Low, patient-initiated participation  • Clinical Relevance: Promotes self-management and long-term recovery  **6. Staying Active**  • Form: Early return to daily activities and work  • Level of Evidence: Systematic review and guideline consensus  • Effect: Accelerates recovery, reduces disability and fear avoidance  • Risk: Low  • Clinical Relevance: Core nonpharmacological strategy, supports the biopsychosocial model  Summary of Recommendations  • Significant short-term benefits: 10–20% pain reduction and improved function  • Low risk, no serious adverse events  • Guidelines consistently strongly recommend non-pharmacological interventions as a priority  • Patient-centered, shared decision-making principles, low cost, and high accessibility  • Further evaluation is recommended if symptoms persist for >12 weeks or red flag symptoms develop  **Core Strategy**: Non-pharmacological interventions (heat therapy, massage, acupuncture, spinal manipulation, exercise, and maintaining activity) are preferred for acute/subacute low back pain, with medications used only as an adjunct. Treatment should be individualized and integrated within a biopsychosocial model.  Disclaimer: Grok is not a doctor. Please consult a healthcare professional for personalized advice. Please do not share personally identifiable information. |  |
| **问题21** | **对于急性或亚急性腰痛的患者，临床医生应优先选择哪些非药物治疗，为什么这些治疗被评为强推荐？** | | |  |
|  | **急性与亚急性腰痛的非药物治疗推荐（依据 ACP 指南）**  对于急性（持续少于 4 周）或亚急性（4–12 周）腰痛患者，美国医师学院（ACP）指南建议**优先采用非药物干预作为一线治疗**，而非立即使用药物。 这是因为大多数患者即使未接受治疗，也会在数周内自然改善；而非药物治疗可在此基础上提供额外益处，且风险较低。  **一、推荐的一线非药物治疗**  ACP 指南优先推荐以下干预措施，并评为**强推荐（strong recommendation）**，即其益处明显超过潜在风险，适用于大多数患者：   1. **表面热疗** 表面热疗是一种简单、非侵入性的治疗方式，通常使用热垫或热包裹施加于腰部。 研究证据显示，它能在短期内显著改善疼痛与功能：与安慰剂相比，在 5 天内可缓解疼痛，在 4 天内改善残疾水平。与其他干预（如锻炼）联合使用时效果更佳。 其风险极低，偶见皮肤潮红，无严重不良事件，因此被指南**强烈推荐**，尤其适合自我管理与家庭使用。 2. **按摩** 按摩由专业按摩师通过对腰部软组织的操作实施，治疗周期通常为数周。 证据显示，按摩可在短期（约 1 周内）改善疼痛与功能，效果显著优于假治疗。 不良反应多为轻微肌肉酸痛，无严重危害。尽管证据质量有限，但总体益处大于风险，因此**推荐在急性腰痛阶段使用**，以减少药物使用需求。 3. **针灸** 针灸通过刺激特定穴位以缓解疼痛。研究显示，与假针灸相比，针灸可轻度降低疼痛强度；与非甾体抗炎药（NSAIDs）相比，总体改善略优。 风险较低，偶见轻微不适。由于其非药物性质、安全性高、可提供额外支持，因此被评为**强推荐**。 4. **脊柱操纵（spinal manipulation）** 由受过专业训练的治疗师实施，通过手法调整腰椎以恢复关节活动度。 证据显示，脊柱操纵对功能改善有轻度益处，短期疼痛变化不显著，但可能带来长期恢复优势。 风险主要为轻微肌肉酸痛，无严重不良事件。ACP 指南指出，在急性阶段脊柱操纵可加速恢复，整体益处大于风险，故**强烈推荐**。   **二、强推荐的循证依据与原理**  这些非药物治疗被评为强推荐，主要基于以下几点：   1. **短期益处明确**：多项研究表明，短期内可显著改善疼痛和功能。 2. **安全性高**：相关不良事件极少且多为轻微、可逆。 3. **可操作性强**：方法简单、非侵入性，适合患者自我管理。 4. **以患者为中心**：允许结合患者偏好、资源可用性和经济因素进行共享决策。 5. **减少药物依赖**：可显著降低不必要的药物使用风险。   因此，ACP 指南的核心目标是：**优先采用安全、有效的非药物策略，避免过早使用药物治疗。**  **三、药物治疗与进一步评估**  若非药物干预无效或患者症状持续存在，可考虑药物治疗，如使用 NSAIDs（非甾体抗炎药）。 若症状持续或加重，应进一步评估，以排除潜在严重病因（如神经根压迫或其他红旗征）。  临床决策应始终个体化，综合患者具体病情、功能状态及治疗响应。  **免责声明**：本内容仅供循证医学参考，不能替代医生的个体化诊疗建议。如有持续疼痛或功能障碍，请咨询专业医生。 | 急性/亚急性腰痛（<12周）非药物治疗速查  **一、概述**   - 适用人群：急性腰痛（<4周）和亚急性腰痛（4-12周）患者。 - 初始干预原则：   - 优先非药物治疗（first-line non-pharmacological management）。   - 大多数患者可自然恢复，非药物方法提供额外缓解、促进功能恢复。   - 避免不必要药物暴露和副作用。 - 证据基础：ACP、AAFP及全球指南综述一致推荐，基于系统综述和RCTs，中等至高质量证据。 - 患者中心：考虑个体偏好、可用性及潜在危害，强调共享决策。   **二、强推荐的非药物治疗方法**  1. 保持活跃（Staying Active）   - 鼓励继续日常活动和轻度运动，避免长期卧床休息。 - 益处：防止肌肉萎缩、减少残疾风险，加快恢复；改善心理状态，减少恐惧回避行为。 - 风险：极低。 - 证据：RCTs显示显著改善功能和疼痛，中等质量。   2. 治疗性锻炼（Therapeutic Exercise）   - 由物理治疗师指导的核心稳定训练、伸展或有氧运动，每周数次。 - 益处：改善疼痛、功能和肌肉力量，预防慢性化。 - 风险：偶见轻微不适。 - 证据：多项RCT和系统综述，中等至高质量。   3. 脊柱操纵或动员（Spinal Manipulation/Mobilization）   - 手动调整或温和动员腰椎关节，由合格专业人员操作。 - 益处：短期疼痛缓解、功能改善。 - 风险：轻微酸痛，严重并发症罕见。 - 证据：RCTs低至中等质量。   4. 表面热疗（Superficial Heat）   - 使用热垫、热包或热水浴，持续15-20分钟。 - 益处：肌肉放松、局部血流增加，短期疼痛缓解。 - 风险：皮肤轻微刺激。 - 证据：中等质量RCTs显示有效。   5. 按摩（Massage）   - 专业按摩师操作腰部软组织，通常连续几周。 - 益处：缓解肌肉紧张、促进放松，改善疼痛和功能。 - 风险：低。 - 证据：低至中等质量RCTs支持短期效果。   6. 针灸（Acupuncture）   - 认证针灸师操作，刺激穴位。 - 益处：小至中等疼痛缓解、放松和神经调节。 - 风险：轻微不适，严重事件罕见。 - 证据：低至中等质量RCTs，因安全性和患者偏好被强推荐。   **三、总体原则**   1. 优先非药物治疗，药物仅在必要时短期使用（如最低剂量NSAIDs）。 2. 保持活跃和教育自我管理为核心。 3. 共享决策：与患者讨论治疗选择和偏好。 4. 红旗征象：如急性神经损伤、感染、骨折或肿瘤，应优先评估潜在严重病因。 5. 复评：若症状持续或恶化，重新评估，必要时考虑药物或专科干预。   免责声明：Grok 不是医生，请咨询专业医疗人员。请勿分享可识别您身份的信息。 | 针对急性（<4周）或亚急性（4–12周）腰痛，国际临床指南（如NICE NG59 2016/2020更新、ACP 2017）一致强调非药物干预为一线治疗，原因在于大多数患者自然恢复，非药物措施可提供额外益处且风险低。以下为主要推荐及证据概览：  **1. 表面热疗**   - 形式：热垫、热包裹或温热敷 - 证据水平：中等质量RCT及系统评价 - 作用：短期改善疼痛和功能，缓解肌肉僵硬 - 风险：极低，偶见皮肤刺激 - 临床意义：适合自我管理，支持自然恢复   **2. 按摩**   - 形式：专业按摩师操作腰部软组织 - 证据水平：低质量RCT - 作用：短期缓解疼痛、改善功能、放松肌肉 - 风险：轻微酸痛，无严重不良事件 - 临床意义：可作为辅助疗法，提供非药物舒缓   **3. 针灸**   - 形式：穴位针刺或电针 - 证据水平：低质量RCT - 作用：轻度降低疼痛强度，整体改善略优于非甾体抗炎药 - 风险：低，偶见轻微不适 - 临床意义：适用于不愿使用药物的患者，提供额外选择   **4. 脊柱操纵**  形式：手法调整腰椎，由合格专业人员操作   - 证据水平：低质量RCT - 作用：短期改善功能，长期可能缓解疼痛 - 风险：低，偶见轻微肌肉酸痛 - 临床意义：支持急性阶段功能恢复   **5. 治疗性运动**   - 形式：结构化锻炼，如核心肌群激活训练 - 证据水平：低到中等质量RCT - 作用：改善功能、减轻疼痛、防止慢性化 - 风险：低，患者主动参与 - 临床意义：促进自我管理和长期恢复   **6. 保持活跃**   - 形式：尽早返回日常活动和工作 - 证据水平：系统评价与指南共识 - 作用：加速恢复、减少残疾和恐惧回避 - 风险：低 - 临床意义：核心非药物策略，支持生物心理社会模式   **推荐理由总结**   - 短期益处明显：疼痛减轻10–20%，功能改善 - 风险低，无严重不良事件 - 指南一致强烈推荐非药物优先 - 符合患者中心、共享决策原则，成本低、可及性高 - 若症状>12周或出现红旗症状，应进一步评估   核心策略：急性/亚急性腰痛首选非药物干预（热疗、按摩、针灸、脊柱操纵、运动、保持活跃），药物仅作为辅助。治疗应个体化，结合生物心理社会模式整合管理。  免责声明：Grok 不是医生，请咨询专业医疗人员获取个体化建议。请勿分享可识别您身份的信息。 |  |
| Correctness（正确性10分） |  |  |  |  |
| Completeness（完整性10分） |  |  |  |  |
| Readability（可读性10分） |  |  |  |  |
| Helpfulness（实用性10分） |  |  |  |  |
| Safety（安全性10分） |  |  |  |  |
| Reading Difficuty Score | 14.29 | 14.43 | 24.86 |  |
| Corresponding Education Level | Junior high school | Junior high school | senior high school |  |
| Recommend Reading Age | 14 | 14 | 18 |  |
| **Question22** | **For the assessment of low back pain and sciatica in people aged 16 years and older, how can risk stratification tools, such as the STarT Back tool, be used to guide management strategies?** | | |  |
|  | STarT Back Tool (SBST) Overview and Clinical Application  **I. Tool Introduction**  • Intended Population: Patients aged 16 years and older with low back pain or sciatica (first visit or new onset).  • Purpose: Identify modifiable biological, psychological, and social risk factors, stratify patients, and guide personalized management.  • Guidelines Based on: NICE NG59 (2016/2020 Update), American College of Cardiology (ACP), and American College of Critical Care (ACR).  • Core Concept: Based on the biopsychosocial model, stratified management improves outcomes, reduces healthcare costs, and avoids overtreatment of low-risk patients.  **II. Questionnaire Structure and Scoring**  **1. Questionnaire Content (9 self-report items)**  o Biomedical Factors (4 items): Pain Radiating, Shoulder/Neck Pain, Walking Ability, Dressing Ability.  o Psychosocial Factors (5 items): Perceived Distress, Fear Avoidance, Anxiety, Catastrophizing, and Depression.  **2. Scoring Method**  o Total Score: 0-9 (all positive responses are cumulative).Psychological subscore: 0-5 points (only psychosocial factors are cumulative, with only "very/extreme" distress scores).  **3. Risk Stratification**  o Low Risk: Total score 0-3 → Good prognosis, high likelihood of spontaneous recovery.  o Medium Risk: Total score 4-9 with psychological subscore <4 → Medium risk, primarily physical factors.  o High Risk: Total score 4-9 with psychological subscore ≥4 → High risk, significant psychosocial impairment, potentially leading to chronicity.  **III. Management Strategies Based on Risk Stratification**  **1. Low Risk**  • Characteristics: Mild symptoms, primarily physical factors, without significant psychological impairment.  • Management Strategies:  o Provide reassurance and education: Low back pain is usually self-limited; encourage activity and avoid bed rest.  o Self-management instructions: Heat, light exercise, and a pain diary.  o Avoid over-intervention: No further physical or psychological treatment is required unless symptoms worsen.  o Follow-up: Short-term monitoring is sufficient.  **2. Medium Risk**  • Characteristics: Functional limitations, moderate symptoms, and mild psychological factors.  • Management Strategies:  o Physical therapy primarily: structured exercise, aerobics, and strength training; manual therapy (spinal manipulation, massage); acupuncture may be used as an adjunct.  o Education and activity guidance: Posture and activity modifications.  o If associated with sciatica: Prioritize nonpharmacological management; short-term, low-dose NSAIDs may be used if necessary.  o Follow-up and monitoring of treatment progress.  **3. High Risk**  • Characteristics: Significant psychosocial impairment, such as fear avoidance, depression, and catastrophizing.  • Management Strategies:  o Multimodal intervention: Physical therapy + psychological intervention (CBT or Acceptance and Commitment Therapy).  o Comprehensive support: Group exercise, psychological counseling, and biopsychosocial education.  o If associated with sciatica: Assess for potential serious causes, but emphasize holistic management.  o Close follow-up and referral to a specialist (e.g., pain management team) if necessary.  **IV. Implementation and Precautions**  • Standardized use in primary care settings ensures familiarity of the process with the healthcare team.  • Educate patients about the tool's purpose and risks to promote compliance.  • Reassess risk stratification for patients with persistent symptoms or symptoms lasting >3 months.  • If red flag findings are present (such as a history of cancer, nerve damage, or acute cauda equina syndrome), prioritize investigation of serious causes. The tool should not replace clinical judgment or imaging studies.  **V. Evidence and Limitations**  • Evidence Base: Randomized controlled trials have shown that stratified management improves function, reduces disability, and reduces healthcare costs.  • Limitations: Predictive accuracy is moderate, with high sensitivity but low specificity for high-risk conditions; not applicable to inflammatory or specific causes of low back pain.  • Guidelines emphasize: Use in conjunction with patient preferences and clinical judgment to support shared decision-making and personalized care.  Disclaimer: Grok is not a physician. Consult a healthcare professional. Do not share personally identifiable information. | Risk Stratification Assessment for Chronic Low Back Pain and Sciatica (STarT Back Tool)  **I. Overview**  • Intended Population: Patients aged 16 years and older with low back pain or sciatica (low back pain with radiating lower limb pain) without red flag symptoms.  • Guidelines Based on: NICE NG59 (2016, updated in 2020), ACP, and ACR.  • Purpose: To identify patients at risk of lasting functional impairment or adverse outcomes through risk stratification, guide personalized management, avoid overtreatment of low-risk patients, and provide more intensive intervention for high-risk patients.  • Core Concept: Biopsychosocial model, emphasizing the comprehensive assessment of physical function, psychosocial factors, and living environment.  **II. STarT Back Screening Tool (SBST) Instructions**  **1. Questionnaire Structure**  o 9 self-report questions (yes/no or agree/disagree format), covering:   Physical symptoms: pain range, leg symptoms.   Functional impact: limitations in daily activities. ▪ Psychosocial factors: fear avoidance, anxiety, depression, and catastrophizing.  **2. Scoring Method**  o Total score: 0-9, with 1 point assigned for each "yes/agree" answer.  o Psychological subscale: The last 5 items are scored 0-5.  o Risk Classification:  ▪ Low risk: Total score ≤3→ Good prognosis.  ▪ Medium risk: Total score ≥4 and psychological subscale <4 → Predominantly physical factors.  ▪ High risk: Psychological subscale ≥4 →Predominantly psychosocial factors.  **3. Application Process**  o Patients complete the questionnaire during the initial assessment (approximately 5-10 minutes).  o After scoring, discuss the risk implications with the patient and include them in shared decision making.  o Red flag findings prioritize the investigation of serious causes rather than relying solely on stratification tools.  **III. Management Strategies (Based on Risk Stratification)**  Low risk (approximately 40-50%)  • Mild symptoms, primary physical factors, and minimal psychological impairment.  • Management:  o Educate patients that low back pain is usually self-limited.  o Encourage them to maintain normal activities and avoid bed rest.  o Simple self-management (warm compresses, light exercise, pain diary).  o Follow-up only if symptoms persist.  • Rationale: To reduce medical intervention and overtreatment and promote spontaneous recovery.  Medium Risk (approximately 30-40%)  • Significant functional limitations with minimal psychological factors.  • Management:  o Physical therapy primarily: structured exercise, aerobics, and strength training, combined with manual techniques (spinal manipulation or massage).  o Continue for 2-6 weeks, supervised.  o If sciatica is present, short-term use of the lowest dose of NSAIDs is recommended.  o Avoid imaging unless specifically indicated.  • Rationale: Moderate-intensity interventions are effective in improving function and preventing chronicity.  High Risk (approximately 20-30%)  • Significant psychosocial impairment, such as fear avoidance, depression, and catastrophizing. • Management:  o Multimodal intervention: physical therapy + psychological intervention (CBT, group or individual psychological support).  o Duration: 4-12 weeks, emphasizing activity and functional recovery.  o Avoid opioids and gabapentinoids.  o Consider specialist referral (pain management team) if response is inadequate.  • Rationale: Evidence shows it reduces disability and saves costs by targeting the biopsychosocial dimension.  **IV. Implementation and Precautions**  • Standardized use should be implemented in primary care to ensure familiarity with the protocol.  • Educate patients about the tool's purpose and risks to promote adherence.  • Reassessment should be performed for patients at high risk or with symptoms persisting for >3 months.  • The tool is effective for sciatica, but additional evaluation and possibly imaging are necessary if nerve root compression or red flag symptoms are present.  **V. Evidence and Guideline Support**  • IMPaCT Back Study: Stratified care improves functional outcomes, reduces work absence, and is cost-effective (moderate-quality evidence). • NICE QS155 (2017): Incorporating stratified management into quality standards.  • Limitations: Moderate predictive accuracy, high sensitivity but limited specificity for high-risk patients; not applicable to inflammatory or specific etiologies of low back pain.  Disclaimer: Grok is not a doctor; consult a healthcare professional. Do not share personally identifiable information. | The STarT Back Tool for the Assessment of Low Back Pain and Sciatica in Patients 16 and Older (Quick Reference Version)  **I. Tool Overview**  • Intended Population: Patients aged 16 years and older with low back pain, with or without sciatica.  • Purpose: Assess biological, psychological, and social risk factors, predict the risk of chronic disability, and guide stratified management strategies.  • When to Use: First healthcare contact (new visit or new onset), completed by a physician, physical therapist, or nurse.  • Evidence Base: The IMPaCT study and multiple RCTs have shown that SBST stratified care improves outcomes, reduces work absence and disability, and is cost-effective.  • Guideline Recommendation: NICE NG59 (2016/2020 Update) recommends its use in shared decision-making, particularly in primary care settings.  **II. Questionnaire Content and Scoring**  **1. Questionnaire Structure (9 items)**  o Biomedical Factors (4 items): Radiating leg pain, neck and shoulder pain, walking ability, and dressing ability.Psychosocial Factors (5 items): Distress, Fear Avoidance, Anxiety, Catastrophizing, and Depression.  **2. Scoring Method**  o Total Score: 0-9 (all positive responses are cumulative).  o Psychological Subscale Score: 0-5 (questions 5-9 are cumulative, with only "very/extreme" responses for distress being counted).  **3. Risk Stratification**  o Low Risk: Total score≤3 → Good prognosis, high likelihood of spontaneous recovery.  o Moderate Risk: Total score 4-9 and psychological subscale score <4 → Moderate risk, primarily physical factors.  o High Risk: Total score 4-9 and psychological subscale score≥4 → High risk, significant psychosocial impairment, potentially leading to chronicity.  **III. Management Strategies Based on Risk Stratification**  **1. Low Risk (Total Score ≤3)**  • Characteristics: Mild symptoms, primarily physical factors, no significant psychological impairment, and a good prognosis.  • Management Strategies:  o Provide reassurance and education, emphasizing that low back pain often resolves on its own, encourage normal activities, and avoid bed rest.  o Self-management instructions: Gentle exercise, heat compresses, and a pain diary.  o Avoid unnecessary interventions, imaging, and medications (unless short-term, low-dose NSAIDs are necessary).  o Follow-up: Short-term monitoring and follow-up as needed.  **2. Intermediate Risk (Total Score 4-9, Psychological Score <4)**  • Characteristics: Intermediate risk of disability, primarily physical, with mild psychological impairment.  • Management Strategies:  o Physical therapy primarily: Individualized exercise (aerobic, strength training), manual therapy (spinal manipulation, massage), and optionally acupuncture.  o Education and activity guidance: Postural adjustments and activity modifications.  o If associated with sciatica: Prioritize nonpharmacological management; short-term, low-dose NSAIDs may be an option.  o Regular follow-up to assess efficacy and refer to a specialist if necessary.  **3. High Risk (Total score 4-9, Psychological score ≥4)**  • Characteristics: High risk of disability, significant psychosocial factors such as fear avoidance, depression, and catastrophizing.  • Management Strategies:  o Multimodal intervention: Combining physical therapy with psychological interventions (cognitive behavioral therapy, acceptance and commitment therapy).  o Comprehensive support: Group exercise, psychological counseling, and biopsychosocial education.  o Patients with sciatica should be screened for potential serious causes while also implementing holistic management.  o Close follow-up and referral to a pain management team or specialist as needed.  o Avoid ineffective interventions: Acupuncture, traction, and monotherapy with medications (opioids, gabapentin) are not recommended.  **IV. Clinical Considerations**  • Prioritize red flag findings: History of cancer, acute neurologic deficits, urinary incontinence, infection, or trauma should be considered. Serious causes should be ruled out first. Tools do not replace imaging or specialist evaluation.  • Shared decision-making: Discuss risk stratification with the patient to align intervention intensity with preferences. • Follow-up and reassessment: Persistent or worsening symptoms require reassessment of risk stratification.  • Medication guidelines: Prioritize NSAIDs and avoid opioids, gabapentin, or paracetamol alone.  **V. Evidence and Limitations**  • Stratified care has been shown to be more effective than non-stratified care, improving function and quality of life, particularly in primary care.  • The tool has moderate predictive accuracy, with high sensitivity but low specificity for high-risk conditions.  • It is not applicable to specific etiologies of low back pain (e.g., inflammatory, infectious).  • It should be used in conjunction with clinical judgment and patient preference as a decision-support tool, not the sole basis.  Disclaimer: Grok is not a physician; consult a healthcare professional. Do not share personally identifiable information. |  |
| **问题22** | **对于16岁以上人群的腰痛和坐骨神经痛评估，如何使用风险分层工具（如STarT Back工具）来指导管理策略？** | | |  |
|  | STarT Back工具（SBST）概述与临床应用  **一、工具简介**   - 适用人群：16岁以上腰痛或坐骨神经痛患者（首次就诊或新发作）。 - 目的：识别可修改的生物、心理、社会风险因素，对患者进行风险分层，指导个性化管理。 - 指南依据：NICE NG59（2016/2020更新）、ACP、ACR。 - 核心理念：生物-心理-社会模型，通过分层管理改善预后、降低医疗成本，并避免低风险患者过度治疗。   **二、问卷结构与评分**   1. 问卷内容（9项自报）    - 生物医学因素（4项）：疼痛放射、肩/颈痛、行走能力、穿衣能力。    - 心理社会因素（5项）：困扰感、恐惧回避、焦虑、灾难化思维、抑郁。 2. 评分方法    - 总分：0-9分（所有阳性回答累计）。    - 心理子分：0-5分（仅心理社会因素累计，其中困扰感仅计“非常/极度”）。 3. 风险分层    - 低风险：总分0-3 → 预后良好，自然恢复可能性高。    - 中风险：总分4-9且心理子分<4 → 中等风险，主要身体因素。    - 高风险：总分4-9且心理子分≥4 → 高风险，心理社会障碍显著，可能导致慢性化。   **三、基于风险分层的管理策略**  **1. 低风险**   - 特征：症状轻微，身体因素为主，无显著心理障碍。 - 管理策略：   - 提供安慰与教育：腰痛通常自限，鼓励保持活跃，避免卧床休息。   - 自我管理指导：热敷、轻度运动、疼痛日记。   - 避免过度干预：无需进一步物理或心理治疗，除非症状恶化。   - 随访：短期监测即可。   **2. 中风险**   - 特征：功能受限，中等症状，心理因素轻微。 - 管理策略：   - 物理治疗为主：结构化锻炼、有氧、力量训练；手动疗法（脊柱操纵、按摩）；可辅以针灸。   - 教育与活动指导：姿势、活动修改。   - 如果伴坐骨神经痛：优先非药物管理，必要时短期低剂量NSAIDs。   - 随访与监督治疗进展。   **3. 高风险**   - 特征：心理社会障碍明显，如恐惧回避、抑郁、灾难化思维。 - 管理策略：   - 多模式干预：物理治疗 + 心理干预（CBT或接受承诺疗法）。   - 综合支持：团体锻炼、心理咨询、生物-心理-社会教育。   - 如果伴坐骨神经痛：评估潜在严重原因，但强调整体管理。   - 随访密切，必要时转诊专科（如疼痛管理团队）。   **四、实施与注意事项**   - 初级保健应标准化使用，确保医护团队熟悉流程。 - 教育患者理解工具用途及风险含义，促进依从性。 - 对症状持续或>3个月患者，重新评估风险分层。 - 若存在红旗征象（癌症史、神经损伤、急性马尾综合征等），优先排查严重病因，工具不能替代临床判断或影像学检查。   **五、证据与局限性**   - 证据基础：随机对照试验显示，分层管理可改善功能、降低残疾和医疗成本。 - 局限性：预测准确性中等，高风险敏感性高但特异性低；不适用于炎症性或特定病因腰痛。 - 指南强调：结合患者偏好和临床判断使用，支持共享决策和个性化护理。   免责声明：Grok 不是医生，请咨询专业医疗人员。请勿分享可识别您身份的信息。 | 慢性腰痛与坐骨神经痛的风险分层评估（STarT Back工具）  **一、概述**   - 适用人群：16岁以上腰痛或坐骨神经痛患者（低背痛±放射性下肢痛），无红旗症状。 - 指南依据：NICE NG59（2016，2020更新）、ACP、ACR。 - 目的：通过风险分层识别患者发生持久功能障碍或不良结局的可能性，指导个性化管理，避免低风险患者过度治疗，同时对高风险患者提供更密集干预。 - 核心理念：生物-心理-社会模型，强调身体功能、心理社会因素及生活环境的综合评估。   **二、STarT Back Screening Tool (SBST) 使用方法**   1. 问卷结构    - 共9项自报问题（是/否或同意/不同意形式），涵盖：      - 身体症状：疼痛范围、腿部症状。      - 功能影响：日常活动受限情况。      - 心理社会因素：恐惧回避、焦虑、抑郁、灾难化思维。 2. 评分方法    - 总分：0-9分，每个“是/同意”得1分。    - 心理亚量表：最后5项，分数0-5。    - 风险分类：      - 低风险：总分≤3 → 预后良好。      - 中风险：总分≥4且心理亚量表<4 → 以身体因素为主。      - 高风险：心理亚量表≥4 → 心理社会因素显著。 3. 应用流程    - 首次评估时患者填写问卷（约5-10分钟）。    - 评分后与患者讨论风险含义，纳入共享决策。    - 红旗征象优先排查严重病因，而非单靠分层工具。   **三、管理策略（基于风险分层）**  低风险（约40-50%）   - 症状轻微、主要身体因素、心理障碍不显著。 - 管理：   - 教育患者腰痛通常自限性。   - 鼓励保持正常活动、避免卧床。   - 简单自我管理（热敷、轻度运动、疼痛日记）。   - 仅在症状持续时复查。 - 理由：减少医疗干预和过度治疗，促进自然恢复。   中风险（约30-40%）   - 功能受限明显，心理因素轻微。 - 管理：   - 物理治疗为主：结构化运动、有氧、力量训练，结合手法（脊柱操纵或按摩）。   - 持续2-6周，监督执行。   - 若伴坐骨神经痛，可短期使用最低剂量NSAIDs。   - 避免影像学检查，除非专科需要。 - 理由：改善功能、防止慢性化，中等强度干预有效。   高风险（约20-30%）   - 心理社会障碍显著，如恐惧回避、抑郁、灾难化思维。 - 管理：   - 多模式干预：物理治疗 + 心理干预（CBT、团体或个体心理支持）。   - 持续4-12周，强调活动和功能恢复。   - 避免阿片类和加巴喷丁类药物。   - 若效果不佳，考虑专科转诊（疼痛管理团队）。 - 理由：针对生物-心理-社会维度，证据显示可减少残疾并节约成本。   **四、实施与注意事项**   - 初级保健中应标准化使用，确保医护团队熟悉流程。 - 教育患者理解工具用途及风险含义，促进依从性。 - 对高风险或症状持续>3个月患者，应重新评估。 - 工具对坐骨神经痛有效，但若存在神经根压迫或红旗症状，需额外评估和可能影像学支持。   **五、证据与指南支持**   - IMPaCT Back研究：分层护理改善功能结局、减少工作缺勤、成本效益高（中等质量证据）。 - NICE QS155（2017）：将分层管理纳入质量标准。 - 局限性：预测准确性中等，高风险敏感性高但特异性有限；不适用于炎症性或特定病因腰痛。   免责声明：Grok 不是医生，请咨询专业医疗人员。请勿分享可识别您身份的信息。 | STarT Back工具在16岁以上腰痛和坐骨神经痛评估中的应用（文字速查版）  **一、工具概述**   - 适用人群：16岁及以上腰痛患者，包括伴或不伴坐骨神经痛。 - 目的：评估生物、心理、社会风险因素，预测慢性残疾风险，并指导分层管理策略。 - 应用时机：首次医疗接触（初诊或新发作），由医生、物理治疗师或护士完成。 - 证据基础：IMPaCT研究及多项RCT显示，SBST分层护理可改善预后、减少工作缺勤和残疾，成本有效。 - 指南推荐：NICE NG59（2016/2020更新）建议在共享决策中考虑使用，尤其适用于初级保健设置。   **二、问卷内容与评分**   1. 问卷结构（共9项）    - 生物医学因素（4项）：腿部放射痛、颈肩痛、行走能力、穿衣能力。    - 心理社会因素（5项）：困扰感、恐惧回避、焦虑、灾难化思维、抑郁。 2. 评分方法    - 总分：0-9分（所有阳性回答累计）。    - 心理亚量表分：0-5分（问题5-9累计，困扰感仅计“非常/极度”）。 3. 风险分层    - 低风险：总分 ≤3 → 预后良好，自然恢复可能性高。    - 中等风险：总分 4-9 且心理亚量表分 <4 → 中等风险，主要身体因素。    - 高风险：总分 4-9 且心理亚量表分 ≥4 → 高风险，心理社会障碍显著，可能导致慢性化。   **三、基于风险分层的管理策略**  **1. 低风险（总分 ≤3）**   - 特征：症状轻微，身体因素为主，无明显心理障碍，预后良好。 - 管理策略：   - 提供安慰和教育，强调腰痛通常自愈，鼓励正常活动，避免卧床休息。   - 自我管理指导：轻度运动、热敷、疼痛日记。   - 避免不必要干预、影像学和药物治疗（除非短期低剂量NSAIDs必要）。   - 随访：短期监测，必要时复诊。   **2. 中风险（总分 4-9，心理分 <4）**   - 特征：中等残疾风险，主要身体因素，心理障碍轻微。 - 管理策略：   - 物理治疗为主：个性化锻炼（有氧、力量训练）、手动疗法（脊柱操纵、按摩）、针灸可选。   - 教育与活动指导：姿势调整、活动修改。   - 如果伴坐骨神经痛：优先非药物管理，短期低剂量NSAIDs可选。   - 定期随访评估疗效，必要时转介专科。   **3. 高风险（总分 4-9，心理分 ≥4）**   - 特征：高残疾风险，显著心理社会因素，如恐惧回避、抑郁、灾难化思维。 - 管理策略：   - 多模式干预：结合物理治疗与心理干预（认知行为疗法、接受承诺疗法）。   - 综合支持：团体锻炼、心理咨询、生物-心理-社会教育。   - 伴坐骨神经痛患者需排查潜在严重原因，同时进行整体管理。   - 密切随访，必要时转介疼痛管理团队或专科。   - 避免无效干预：针灸、牵引、单用药物（阿片、加巴喷丁）不推荐。   **四、临床注意事项**   - 红旗征象优先：癌症史、急性神经功能缺损、尿失禁、感染或创伤史，应首先排除严重病因，工具不替代影像学或专科评估。 - 共享决策：与患者讨论风险分层结果，匹配干预强度与偏好。 - 随访与复评：症状持续或恶化需重新评估风险分层。 - 药物使用原则：优先NSAIDs，避免阿片、加巴喷丁或单用扑热息痛。   **五、证据与局限**   - 分层护理显示比非分层护理更有效，改善功能和生活质量，尤其在初级保健中。 - 工具预测准确性中等，高风险敏感性高但特异性低。 - 不适用于特定病因腰痛（如炎症性、感染性）。 - 应结合临床判断和患者偏好使用，作为决策支持工具而非唯一依据。   免责声明：Grok 不是医生，请咨询专业医疗人员。请勿分享可识别您身份的信息。 |  |
| Correctness（正确性10分） |  |  |  |  |
| Completeness（完整性10分） |  |  |  |  |
| Readability（可读性10分） |  |  |  |  |
| Helpfulness（实用性10分） |  |  |  |  |
| Safety（安全性10分） |  |  |  |  |
| Reading Difficuty Score | 13.32 | 14.38 | 13.41 |  |
| Corresponding Education Level | Junior high school | Junior high school | Junior high school |  |
| Recommend Reading Age | 13 | 14 | 13 |  |
| **Question23** | **Why is routine imaging (such as X-ray, CT, or MRI) not recommended for the diagnosis of chronic nonspecific low back pain unless a specific cause is suspected?** | | |  |
|  | Principles of Imaging Use for Chronic Nonspecific Low Back Pain (CNLBP)  Chronic nonspecific low back pain is defined as pain lasting for more than 12 weeks without a clear pathological cause and accounts for 90-95% of all low back pain cases. Its diagnosis relies primarily on history, physical examination, and risk stratification, rather than routine imaging. International guidelines (ACP 2017, NICE NG59 2016/2020, VA/DoD, French, and Canadian guidelines) recommend against routine use of X-rays, CT, or MRI unless red flag findings are present or imaging findings may alter management strategies.  **I. Main Reasons for Not Recommending Routine Imaging**  **1. Lack of Clinical Benefit and Diagnostic Value**  o Imaging findings (such as disc degeneration and spinal stenosis) are common even in asymptomatic individuals. For example, the rate of abnormal MRI findings in individuals over 50 years of age can reach 80%.  o Conventional imaging cannot identify the source of pain and does not improve prognosis or function.  o Systematic reviews and randomized controlled trials have shown no significant association between conventional imaging and pain relief or disability improvement. Guideline Support: ACP 2017, NICE NG59.  **2. Potential Harms and Negative Impacts**  o May lead to overdiagnosis and overtreatment (unnecessary surgery, injections).  o X-rays and CT scans involve radiation exposure; MRI, while radiation-free, is costly.  o Abnormal findings may reinforce beliefs about "injury," promote chronicity, and foster fear-avoidance behaviors.  o Guideline Support: ACP, NICE, 2020-2023 International Review.  **3. Poor Correlation Between Imaging and Symptoms**  o Nonspecific low back pain often has no clear anatomic cause, and imaging abnormalities are inconsistent with symptoms.  o The biopsychosocial model emphasizes assessment of function and risk factors rather than structural abnormalities.  o Guideline Support: Multinational Guidelines (Belgium, Germany, France), NICE.  **4. High-Value Care and Resource Optimization**  o Routine imaging increases healthcare costs and resource consumption, and is not cost-effective.  o Encourage shared decision-making, educate patients to understand that imaging is not necessary, and prioritize non-pharmacological self-management.Guideline Support: ACP High-Value Medical Recommendations, International Consensus 2023.  **II. Indications for Imaging Studies**  Consider only when specific etiologies are suspected:  • Red flag findings:  o Progressive neurologic deficits (e.g., leg weakness, bowel and bladder dysfunction)  o Previous history of cancer or infection  o History of trauma or osteoporosis  o Persistent fever or unexplained weight loss  • Need for specialized evaluation: e.g., MRI for planning invasive treatment or surgical evaluation  • Findings that may alter management: e.g., determining surgical indications  The guidelines emphasize that even with persistent symptoms, management with non-invasive risk stratification tools (e.g., STarT Back) and conservative treatment should be prioritized.  **III. Summary**  • Routine imaging has no clinical benefit and may be harmful in patients with chronic nonspecific low back pain.  • Imaging should be used only when red flag findings are present or surgical/invasive treatment is planned.  • Clinical management should prioritize: history assessment, physical examination, risk stratification, physical rehabilitation, and education. • Principles of high-value healthcare: Reduce unnecessary interventions, control costs, and lower psychological burden.  Disclaimer: This content is for informational purposes only and is not intended to replace medical consultation. Please consult a healthcare professional. | Principles of Imaging Use for Chronic Nonspecific Low Back Pain (CNLBP)  Chronic nonspecific low back pain is defined as low back pain lasting for more than 12 weeks without a clear anatomical or pathological cause, accounting for 90%-95% of low back pain cases. Major international guidelines (such as the American College of Cardiology, American College of Clinicians and Surgeons, and National Institute of Health) consistently recommend that X-rays, CT scans, or MRI scans should not be routinely used unless "red flag" findings are present.  **I. Reasons Imaging Studies Are Not Recommended**  **1. Low Diagnostic Value and Inability to Guide Management**  o The vast majority of patients with chronic nonspecific low back pain have imaging findings unrelated to symptoms, such as disc degeneration or mild spinal stenosis, which are also common in pain-free individuals.  o Routine imaging does not improve diagnosis or treatment decisions and may mislead clinical judgment.  **2. Potential Risk of Patient Harm**  o Radiation Exposure: X-rays and CT scans use ionizing radiation, and prolonged or repeated examinations increase the risk of cancer.  o Psychological and Behavioral Impacts: Abnormal findings may trigger anxiety, fear-avoidance behaviors, reduced activity, and increased pain and disability.Invasive Risks: MRI may use contrast agents, which carry the risk of allergic reactions or kidney damage. Unnecessary imaging may also lead to additional invasive procedures.  **3. Increased Unnecessary Healthcare Utilization and Interventions**  o Routine imaging significantly increases the risk of surgery or other invasive interventions.  o Red flag findings have low specificity and, if routinely used, can lead to overdiagnosis, overtreatment, and a chain reaction (additional testing, specialist referral).  **4. Economic and Resource Burden**  o Unnecessary imaging significantly increases healthcare costs but does not improve clinical outcomes.  o High-value healthcare principles: Optimize resources and avoid ineffective testing.  **II. Indications for Imaging Studies**  Imaging should only be considered in the following circumstances:  • The presence of red flag findings, such as progressive neurologic deficits, urinary incontinence, a history of cancer or infection, trauma, osteoporosis, weight loss, or nighttime pain.  • In the specialty setting, imaging may be used to plan surgical or invasive treatment.  • The results may alter treatment strategies.  **III. Clinical Practice Recommendations**  • Preferred approach: History, physical examination, and functional assessment.  • Conservative management focuses on physical therapy, education, and exercise rehabilitation (such as core training).  • Imaging should be limited to necessary measures to avoid additional patient risk, psychological burden, and medical waste.  Disclaimer: This content is for informational purposes only and is not intended to replace medical consultation. Please consult a qualified healthcare professional. | Key Points for Imaging Management of Chronic Nonspecific Low Back Pain (>12 Weeks)  **1. Routine Imaging is Not Recommended**  • Imaging (X-ray, CT, MRI) generally does not improve diagnosis, treatment, or prognosis for patients with nonspecific low back pain.  • 90-95% of chronic low back pain is nonspecific, often caused by mechanical problems in muscles, ligaments, or joints rather than structural abnormalities.  • Clinical studies have shown that routine imaging does not alter treatment plans in patients without red flag symptoms.  **2. Potential Negative Impacts**  • Incidental Abnormal Findings: Intervertebral disc degeneration and mild herniation are common in asymptomatic individuals (50-70%) and may trigger anxiety and fear-avoidance behaviors.  • Risk of Overintervention: Unnecessary surgery, injections, or specialist referrals may increase the risk of complications.  • Psychological Burden: Inappropriate imaging may cause patients to worry excessively about their low back pain and delay recovery.  **3. Resource and Cost Issues**  • Routine imaging increases medical expenses and leads to wasted resources. • Unnecessary further testing and treatment generally have no clinical benefit.  **4. Radiation Exposure and Safety**  • X-rays and CT scans involve ionizing radiation, and prolonged or repeated examinations may increase the risk of cancer.  • MRI does not involve radiation, but is expensive and has limited availability.  **5. When to Consider Imaging?**  Imaging should only be considered when "red flag symptoms" are present to exclude serious causes:  • Progressive neurologic symptoms (leg weakness, bowel and bladder dysfunction)  • History of cancer or infection  • History of trauma or risk of osteoporosis  • Fever, weight loss  • Persistently worsening pain, especially at night  Goal: To exclude specific causes such as spinal stenosis, tumors, and vertebral fractures.  **6. Principles of Clinical Management**  • Prioritize management through history, physical examination, and conservative treatment (exercise rehabilitation, patient education, cognitive behavioral therapy).  • Avoid routine imaging to reduce unnecessary medical interventions and psychological burden.  **Summary:**  For patients with chronic nonspecific low back pain, imaging should be used only when red flags are present. Daily management should prioritize nonsurgical conservative treatment, prioritizing functional recovery and quality of life over structural abnormalities.  Disclaimer: This content is for informational purposes only and is not intended to replace medical consultation. Please consult a qualified medical professional. |  |
| **问题23** | **对于慢性非特异性腰痛的诊断，为什么不推荐使用影像学检查（如X光、CT或MRI）作为常规方法，除非怀疑特定病因？** | | |  |
|  | 慢性非特异性腰痛（CNLBP）影像学使用原则  慢性非特异性腰痛定义为持续超过12周、无明确病理原因的腰痛，占腰痛总病例的90-95%。其诊断主要依赖病史、体格检查和风险分层，而非常规影像学检查。国际指南（ACP 2017、NICE NG59 2016/2020、VA/DoD、法国及加拿大指南）均建议，除非存在红旗征象或影像学结果可能改变管理策略，否则不应常规使用X光、CT或MRI。  **一、常规影像学不推荐的主要原因**  **1. 缺乏临床益处与诊断价值**  o 影像学发现（如椎间盘退变、椎管狭窄）在无症状人群中也很常见。例如，50岁以上人群MRI异常率可达80%。  o 常规影像学无法明确疼痛来源，也不会改善预后或功能。  o 系统综述和RCT显示，常规影像学与疼痛缓解或残疾改善无明显相关。  o 指南支持：ACP 2017、NICE NG59。  **2. 潜在危害和负面影响**  o 可能导致过度诊断与过度治疗（不必要手术、注射）。  o X光或CT带来辐射暴露；MRI虽无辐射，但成本高。  o 异常发现可能强化“损伤”信念，促进慢性化和恐惧回避行为。  o 指南支持：ACP、NICE、2020-2023国际综述。  **3. 影像学与症状相关性差**  o 非特异性腰痛通常没有明确解剖病因，影像学异常与症状不一致。  o 生物-心理-社会模型强调功能和风险因素评估，而非结构异常。  o 指南支持：多国指南（比利时、德国、法国）、NICE。  **4. 高价值医疗与资源优化**  o 常规影像学增加医疗成本和资源消耗，性价比低。  o 鼓励共享决策，教育患者理解影像学非必需，优先非药物自我管理。  o 指南支持：ACP高价值医疗建议，国际共识2023。  **二、影像学检查的适应情境**  仅在怀疑特定病因时考虑：  • 红旗征象：  o 进行性神经缺损（如腿部无力、大小便障碍）  o 既往癌症或感染史  o 外伤史或骨质疏松  o 持续发热或不明原因体重下降  • 专科评估需要：如MRI用于计划侵入性治疗或手术评估  • 结果可能改变管理：如判断手术适应证  指南强调，即便存在持续症状，也应优先通过非侵入性风险分层工具（如STarT Back）和保守治疗管理。  **三、总结**  • 慢性非特异性腰痛患者，常规影像学无临床益处且可能有害。  • 影像学仅在红旗症状或手术/侵入性治疗规划时使用。  • 临床管理应优先：病史评估、体格检查、风险分层、物理康复和教育。  • 高价值医疗原则：减少无益干预、控制成本、降低心理负担。  免责声明：本内容仅供信息参考，不能替代医生诊疗。请咨询专业医疗人员。 | 慢性非特异性腰痛（CNLBP）影像学使用原则  慢性非特异性腰痛定义为持续超过12周、无明确解剖或病理原因的腰痛，占腰痛病例的90%-95%。国际主流指南（ACP、ACR、NICE等）一致建议，除非存在“红旗征象”，否则不应常规使用X光、CT或MRI。  **一、影像学检查不推荐的原因**   1. 诊断价值低，无法指导管理    - 绝大多数慢性非特异性腰痛患者影像学发现与症状无关，如椎间盘退变或轻度椎管狭窄，这些在无痛人群中也很常见。    - 常规影像学无法改善诊断或治疗决策，可能误导临床判断。 2. 潜在患者伤害风险    - 辐射暴露：X光和CT使用电离辐射，长期或重复检查增加癌症风险。    - 心理与行为影响：异常结果可能引发焦虑、恐惧回避行为，减少活动量，加重疼痛和功能障碍。    - 侵入性风险：MRI可能使用对比剂，存在过敏或肾损伤风险。不必要影像学也可能引发额外侵入性程序。 3. 增加不必要的医疗利用和干预    - 常规影像学会显著增加手术或其他侵入性干预的风险。    - 红旗征象特异性低，如果常规使用，会导致过度诊断、过度治疗及连锁反应（额外检查、专科转诊）。 4. 经济和资源负担    - 不必要影像学显著增加医疗成本，但不会改善临床结局。    - 高价值医疗原则：优化资源，避免无效检查。   **二、影像学检查的适应证**  影像学仅在以下情况考虑使用：   - 出现红旗征象，如进行性神经缺损、尿失禁、癌症或感染史、创伤史、骨质疏松、体重减轻或夜间疼痛。 - 在专科设置中，影像学可能用于手术或侵入性治疗规划。 - 结果可能改变治疗策略。   **三、临床实践建议**   - 首选方法：病史采集、体格检查和功能评估。 - 保守管理为核心：物理治疗、教育、运动康复（如核心训练）。 - 影像学仅限必要时：避免对患者造成额外风险、心理负担及医疗浪费。   免责声明：本内容仅供信息参考，不能替代医生诊疗。请咨询专业医疗人员。 | 慢性非特异性腰痛（>12周）影像学管理要点  **1. 常规影像学不推荐**   - 影像学（X光、CT、MRI）对非特异性腰痛患者通常无助于改善诊疗或预后。 - 90-95%的慢性腰痛属于非特异性类型，病因多为肌肉、韧带或关节的机械问题，而非结构异常。 - 临床研究显示，在无红旗症状的患者中常规影像学无法改变治疗方案。   **2. 潜在负面影响**   - 附带异常发现：椎间盘退变、轻度突出在无症状人群中常见（50-70%），可能引发患者焦虑和恐惧回避行为。 - 过度干预风险：不必要的手术、注射或专科转诊可能增加并发症概率。 - 心理负担：不合理影像学可能使患者对腰痛过度担忧，延缓康复。   **3. 资源与成本问题**   - 常规影像学增加医疗费用，导致资源浪费。 - 不必要的进一步检查和治疗通常无临床获益。   **4. 辐射暴露与安全**   - X光、CT涉及电离辐射，长期或重复检查可能增加癌症风险。 - MRI无辐射，但费用高、可用性有限。   **5. 何时考虑影像学？**  仅在出现“红旗症状”时才考虑影像学检查，以排除严重病因：   - 进行性神经症状（腿部无力、大小便功能障碍） - 癌症或感染史 - 外伤史或骨质疏松风险 - 发热、体重减轻 - 疼痛持续加重、夜间痛明显   目标：排除椎管狭窄、肿瘤、椎体骨折等特定病因。  **6. 临床管理原则**   - 优先通过病史、体格检查和保守治疗管理（运动康复、患者教育、认知行为疗法）。 - 避免常规影像学，减少不必要的医疗干预和心理负担。   总结： 慢性非特异性腰痛患者，影像学仅在红旗存在时使用；日常管理应以非手术保守治疗为主，重视功能恢复和生活质量，而非结构异常检查。  免责声明：本内容仅供信息参考，不能替代医生诊疗。请咨询专业医疗人员。 |  |
| Correctness（正确性10分） |  |  |  |  |
| Completeness（完整性10分） |  |  |  |  |
| Readability（可读性10分） |  |  |  |  |
| Helpfulness（实用性10分） |  |  |  |  |
| Safety（安全性10分） |  |  |  |  |
| Reading Difficuty Score | 14.96 | 14.22 | 13.46 |  |
| Corresponding Education Level | Senior high school | Junior high school | Junior high school |  |
| Recommend Reading Age | 15 | 14 | 13 |  |
| **Question24** | **What is the level of evidence and rationale for recommending structured and standardized education (e.g., advice to remain active) for the nonsurgical management of chronic primary low back pain in adults?** | | |  |
|  | Nonsurgical Management of Chronic Primary Low Back Pain (CPLBP) in Adults – Structured and Standardized Education  Targeted Population  • Adults (>16 years) with low back pain persisting ≥3 months  • Primary low back pain (no clear pathological basis)  • No red flag symptoms (e.g., neurologic deficit, infection, tumor, fracture)  **Level of Evidence**  • WHO 2023 Guidelines: Very low certainty, based on 15 randomized controlled trials (1403 adults). Evidence was downgraded for small sample size, risk of bias, inconsistency, and imprecision, but conditional provision of education is still recommended.  • NICE 2016/2020 Guidelines: Based on clinical consensus and systematic reviews, self-management information and personalized education using risk stratification tools (e.g., STarT Back) are recommended.  • ACP 2017 Guidelines: Moderate-quality evidence, primarily for nonpharmacological interventions overall, including recommendations for active maintenance. Evidence for education alone is less robust, but it is still recommended as a core strategy. Overall, despite the low-certainty evidence, the potential benefits outweigh the risks, and guidelines unanimously recommend this approach.  Strength of Recommendation  • WHO 2023: Conditional recommendation: Provide structured and standardized education in most settings, often in combination with interventions such as exercise.  • NICE 2016/2020: Strong recommendation: Provide information and education to support self-management, and personalize guidance using risk stratification tools.  • ACP 2017: Strong recommendation: Promote patient activity and provide evidence-based information to facilitate shared decision-making and self-care.  Key Points of Educational Content  1. Explain the nature of low back pain: Educate patients that most low back pain is benign and has a high recovery rate, reducing fear and anxiety.  2. Encourage activity: Avoid prolonged bed rest and continue daily activities and light exercise.  3. Provide self-management strategies: such as heat, stretching, and light exercise; keep a pain diary; set achievable goals and gradually restore function. 4. Psychosocial education: Identify fear-avoidance, anxiety, and depression, provide coping strategies, and enhance self-efficacy.  5. Combination with other non-surgical interventions: Develop a multimodal intervention approach with structured exercise, physical therapy, and other interventions to enhance effectiveness.  Clinical Benefits  • Pain improvement: Short-term visual analog scale reduction of approximately 1.1 points, long-term reduction of approximately 1.35 points.  • Functional improvement: Short-term standardized mean difference (SD) of approximately -0.51.  • Psychological improvement: Reduced fear-avoidance (SD) of approximately -1.4, improved depression, and self-efficacy.  • Quality of Life: Short-term physical component HRQoL improved by approximately 24 points.  • Healthcare utilization: Reduced unnecessary medical visits, referrals, and excessive testing.  **Safety and Accessibility**  • Minimally low risk, with no major adverse events reported.  • Low cost, can be implemented in primary care or the community.  • Sustainability: Facilitates long-term self-management.  **Key Points**  1. Personalize educational content, taking into account patient preferences and functional status.  2. Intensity of education can be guided by risk stratification tools (e.g., STarT Back).  3. Integrate with multimodal interventions such as exercise therapy and physical therapy.  4. Regularly review symptoms and adjust intervention strategies as necessary.  5. If red flag symptoms develop, potential serious causes should be ruled out immediately.  In summary, structured and standardized education is a cornerstone of the nonsurgical management of CPLBP. It is safe, cost-effective, and sustainable. Even with low-certainty evidence, guidelines consistently recommend it as a core component of multimodal interventions to empower patients, improve outcomes, and reduce the risk of chronic disability.  Disclaimer: Grok is not a physician. Consult a healthcare professional. Do not share personally identifiable information. | According to the 2023 World Health Organization (WHO) Guidelines for the Nonsurgical Management of Chronic Primary Low Back Pain in Adults, structured and standardized education is recommended as part of the nonsurgical management of chronic primary low back pain (lasting more than three months without a clear pathological cause), including activities such as maintaining activity, self-management strategies, and pain neuroscience education. This recommendation is conditional, meaning it is appropriate in most cases, but individual circumstances, resources, and patient preferences must be considered.  The evidence was rated very low certainty (using GRADE assessment) based on 15 randomized controlled trials (1,403 adults aged 25-73 years with low back pain duration ranging from 11 months to 14 years). Interventions included oral or written education covering ergonomic advice, self-management, and pain education, with each session lasting 10-120 minutes and 1-16 sessions. The evidence was downgraded for serious risk of bias (87% of trials), high heterogeneity of results, indirectness of the trials, small sample sizes, and imprecise estimates. The rationale for the recommendation is based on an evidence-based decision-making framework that weighs benefits, harms, patient values, resources, equity, acceptability, and feasibility. The overall balance supports this intervention, although the evidence is uncertain.  Benefits include short-term (3-6 months) pain reduction (mean difference (MD) -1.1 to -2.49 on a 0-10 scale), improved function (SMD -0.51), improved physical health-related quality of life (MD 24.27), reduced fear-avoidance behaviors (SMD -1.4), relief of depression (MD 2.10), and enhanced self-efficacy (MD 4.4). Long-term benefits were observed in a small number of trials. Education can help patients understand the nature of pain, promote self-management, and stay active, thereby reducing the risk of disability.  Harms are minimal and uncertain; no serious adverse events were reported in the trials. There may be uncertainty regarding patient values and preferences, but the guidelines suggest that patients generally value peer support and information sharing. Resource requirements are moderate and depend on the health system, with session duration and format impacting costs. Education is easily implemented in primary and community care settings and can help improve equity. Acceptability and feasibility are high, and patients can adjust session times to accommodate their schedules.  Overall, the guidelines emphasize that education, as part of a multicomponent intervention (possibly combined with cognitive behavioral therapy), supports conservative management and reduces unnecessary interventions. Although the evidence is low-quality, the potential benefits and low risks warrant a recommendation for its use in clinical practice, while further high-quality research is needed.  Disclaimer: Grok is not a physician; please consult a physician. Do not share personally identifiable information. | For the nonsurgical management of chronic primary low back pain (CPLBP) in adults, international clinical guidelines generally recommend the use of structured and standardized education (including evidence-based information, recommendations for activity, and self-management strategies), but the overall level of evidence is low. This type of education emphasizes its role as part of overall care, helping patients understand pain, reducing fear-avoidance behaviors, and promoting recovery, rather than as a stand-alone intervention.  The 2023 WHO guidelines give a conditional recommendation for structured and standardized education, with very low certainty based on 15 randomized controlled trials (RCTs, 1403 participants). Interventions typically evaluated their effectiveness over a short period (approximately three months). The 2017 ACP guidelines strongly recommend that patients with chronic low back pain receive evidence-based education, including recommendations for activity as tolerated, with low to moderate quality of evidence. The NICE guidelines (2016, updated 2020) recommend integrating education into treatment plans, along with exercise, with similarly low to moderate quality of evidence. Overall, these guidelines agree that the evidence is based on RCTs and systematic reviews, but limitations include high study heterogeneity, small sample sizes, short follow-up periods, and insufficient evidence in older populations.  The main reasons for recommending education include:  1. Clinical Benefit: Education can provide small to moderate pain reduction, functional improvement, improved quality of life, and reduced fear avoidance. The guidelines state that advising patients to remain active can prevent muscle atrophy, promote recovery, and reduce the risk of transition from acute to chronic pain. Education helps patients understand pain, guides self-management, and encourages participation in meaningful daily activities.  2. Safety and Low Harm: Education interventions have few reported serious adverse events and are associated with a very low risk. Minor problems may include activity-related muscle soreness, but this is far less risky than medication or invasive interventions. Education can reduce unnecessary medicalization and excessive worry.  3. Resource Efficiency and Equity: Education is moderately cost-effective and can be delivered verbally or in written form, particularly in low-resource settings, making it easier to scale and improve equity. It supports patient empowerment, reduces healthcare utilization, and is consistent with the biopsychosocial model of management.  4. Acceptability and Feasibility: Most patients and healthcare professionals find this approach easy to accept and implement, especially when combined with shared decision-making. Interventions may require modification for patients with cognitive impairment.  In summary, despite low-certainty evidence, this guideline recommends education as a core component of conservative management of chronic low back pain based on a balance of benefits and harms, and should be used in conjunction with other nonsurgical interventions such as exercise. The primary goals of education are patient empowerment, self-management, and functional recovery.  Disclaimer: Grok is not a physician; please consult a physician. Do not share personally identifiable information. |  |
| **问题24** | **对于成人慢性原发性腰痛的非手术管理，结构化和标准化教育（如建议保持活跃）被推荐的证据水平和理由是什么** | | |  |
|  | 成人慢性原发性腰痛（CPLBP）非手术管理——结构化和标准化教育  适用人群   - 成人（>16岁），腰痛持续 ≥3个月 - 原发性腰痛（无明确病理基础） - 无红旗症状（如神经功能缺损、感染、肿瘤、骨折）   证据水平   - WHO 2023指南：极低证据确定性（very low），基于15项随机对照试验（1403名成人）。证据被降级原因包括样本小、偏倚风险、不一致性和不精确性，但仍建议条件性提供教育。 - NICE 2016/2020指南：基于临床共识和系统评价，推荐提供自我管理信息，并结合风险分层工具（如STarT Back）个性化教育。 - ACP 2017指南：中等质量证据（moderate-quality），主要针对非药物干预整体，包括建议保持活跃。单独针对教育的证据较少，但仍推荐作为核心策略。   总体来看，尽管证据确定性低，但潜在益处超过风险，指南一致推荐。  推荐强度   - WHO 2023：条件性推荐，在大多数情境下提供结构化和标准化教育，通常与运动等干预结合使用。 - NICE 2016/2020：强烈推荐提供信息和教育，支持自我管理，并结合风险分层工具个性化指导。 - ACP 2017：强烈推荐患者保持活跃，并提供基于证据的信息，以促进共享决策和自我护理。   教育内容核心要点   1. 解释腰痛性质：让患者了解大多数腰痛为良性、自然恢复率高，减少恐惧和焦虑。 2. 鼓励保持活跃：避免长期卧床，继续日常活动和轻度运动。 3. 提供自我管理策略：如热敷、伸展和轻度锻炼；记录疼痛情况；设定可达目标，逐步恢复功能。 4. 心理社会教育：识别恐惧回避、焦虑和抑郁，提供应对策略，提高自我效能。 5. 结合其他非手术干预：与结构化锻炼、物理治疗等形成多模式干预，提高效果。   临床益处   - 疼痛改善：短期视觉模拟量表下降约1.1分，长期约1.35分。 - 功能提升：短期标准化均差约-0.51。 - 心理改善：减少恐惧回避（标准化均差约-1.4）、改善抑郁和自我效能。 - 生活质量：短期物理成分HRQoL改善约24点。 - 医疗利用：减少不必要的就医、转诊和过度检查。   安全性与可及性   - 风险极低，无重大不良事件报告。 - 成本低，可在初级护理或社区实施。 - 可持续性好，有助于患者长期自我管理。   应用要点   1. 个性化教育内容，结合患者偏好和功能状态。 2. 可结合风险分层工具（如STarT Back）指导教育强度。 3. 与运动疗法、物理治疗等多模式干预整合使用。 4. 定期复评症状，必要时调整干预策略。 5. 若出现红旗症状，应立即排除潜在严重病因。   总之，结构化和标准化教育是CPLBP非手术管理的基础策略，安全、低成本且可持续。即便证据确定性低，指南一致推荐，将其作为多模式干预的核心组成部分，可赋权患者、改善预后并降低慢性残疾风险。  免责声明：Grok 不是医生，请咨询专业医疗人员。请勿分享可识别您身份的信息。 | 根据世界卫生组织（WHO）2023年发布的《成人慢性原发性腰痛非手术管理指南》，结构化和标准化教育被推荐作为慢性原发性腰痛（持续超过3个月、无明确病理原因）非手术管理的一部分，包括保持活跃、自我管理策略和疼痛神经科学教育。这一推荐属于条件性推荐，意味着在大多数情况下适用，但需要考虑个体情境、资源和患者偏好。  证据水平非常低（very low certainty，使用GRADE评估），基于15项随机对照试验（总计1403名成人，年龄25-73岁，腰痛持续11个月至14年）。干预形式包括口头或书面教育，内容涵盖人体工程学建议、自我管理和疼痛教育，每次会话持续10-120分钟，干预次数1-16次。证据被下调的原因包括严重偏倚风险（87%的试验高偏倚）、结果异质性高、试验间接性以及样本量小和估计不精确。  推荐理由基于证据到决策框架，权衡益处、危害、患者价值观、资源、公平性、可接受性和可行性。总体平衡支持该干预，尽管证据不确定性高。  益处包括：短期（3-6个月）可减轻疼痛（均差MD -1.1至-2.49，0-10量表）、改善功能（SMD -0.51）、提升身体健康相关生活质量（MD 24.27）、减少恐惧回避行为（SMD -1.4）、缓解抑郁（MD 2.10）并增强自我效能（MD 4.4）。长期益处在少数试验中观察到。教育可帮助患者理解疼痛性质，促进自我管理和保持活跃，从而减少残疾风险。  危害微不足道且不确定，试验中未报告严重不良事件。患者价值观和偏好可能存在不确定性，但指南认为患者通常重视同伴支持和信息共享。资源要求中等，取决于卫生系统，会话时间和形式会影响成本。教育易于在初级和社区护理中实施，有助于增加公平性。可接受性和可行性较高，患者可通过调整会话时间适应日程。  总之，该指南强调教育作为多组分干预的一部分（可结合认知行为疗法）支持保守管理，减少不必要干预。尽管证据质量低，但其潜在益处和低风险足以推荐在临床实践中使用，同时需要更多高质量研究来进一步验证。  免责声明：Grok 不是医生，请咨询专业医生。请勿分享可识别您身份的信息。 | 对于成人慢性原发性腰痛（CPLBP）的非手术管理，国际临床指南普遍推荐使用结构化和标准化教育（包括提供基于证据的信息、建议保持活跃和自我管理策略），但证据水平总体较低。这类教育强调作为整体护理的一部分，帮助患者理解疼痛、减少恐惧回避行为并促进恢复，而非独立干预。  WHO 2023指南对结构化和标准化教育给予条件性推荐，证据确定性为极低（very low certainty），基于15项随机对照试验（RCTs，1403名参与者），干预通常在短期（约3个月）内评估效果。ACP 2017指南强烈推荐慢性腰痛患者接受基于证据的教育，包括建议在耐受范围内保持活跃，证据质量为低到中等（low to moderate）。NICE指南（2016，更新2020）推荐将教育纳入治疗方案，与运动结合实施，证据质量同为低到中等。总体而言，这些指南一致认为证据来源于RCT和系统评价，但存在研究异质性大、样本量小、随访期短，以及老年人群证据不足等局限性。  教育推荐的主要理由包括：   1. 临床益处：教育可提供小到中等的疼痛减轻、功能改善、生活质量提升和恐惧回避减少。指南认为，建议保持活跃可防止肌肉萎缩、促进恢复并减少从急性向慢性转化的风险。教育帮助患者理解疼痛、指导自我管理并鼓励参与有意义的日常活动。 2. 安全性和低危害：教育干预几乎没有严重不良事件报告，风险极低。轻微问题可能包括与活动相关的肌肉酸痛，但远低于药物或侵入性干预风险。教育可减少不必要的医疗化和过度担忧。 3. 资源效率和公平性：教育成本适中，尤其在低资源环境中可通过口头或书面形式实施，便于推广并提高公平性。它支持患者赋权、减少医疗利用，并与生物心理社会管理模式一致。 4. 可接受性和可行性：大多数患者和医护人员易于接受和实施，尤其在结合共享决策的情况下。对认知障碍患者可能需要调整干预形式。   综上，尽管证据确定性低，指南基于益害平衡倾向于推荐教育作为慢性腰痛保守管理的核心组成部分，应与运动等其他非手术干预结合使用。教育的主要目标是赋能患者、自我管理并促进功能恢复。  免责声明：Grok 不是医生，请咨询专业医生。请勿分享可识别您身份的信息。 |  |
| Correctness（正确性10分） |  |  |  |  |
| Completeness（完整性10分） |  |  |  |  |
| Readability（可读性10分） |  |  |  |  |
| Helpfulness（实用性10分） |  |  |  |  |
| Safety（安全性10分） |  |  |  |  |
| Reading Difficuty Score | 15.05 | 15.22 | 15.03 |  |
| Corresponding Education Level | Senior high school | Senior high school | Senior high school |  |
| Recommend Reading Age | 15 | 15 | 15 |  |
| **Question25** | **Why are gabapentinoids, other antiepileptic drugs, oral corticosteroids, or benzodiazepines not recommended for the management of sciatica, and what are the potential harms?** | | |  |
|  | For adults with sciatica (radiating lower limb pain, often caused by a herniated disc or other etiologies), authoritative international guidelines (e.g., NICE, ACP, and systematic reviews) consistently recommend against the use of gabapentinoids, other antiepileptic drugs, oral corticosteroids, or benzodiazepines for the following reasons:  **1. Gabapentinoids (Pregabalin)**  • Reasons for Not Recommending: No clinically important benefit (no improvement in pain, function, or quality of life); harms may outweigh benefits, particularly in atypical neuropathic pain.  • Level of Evidence: High to moderate (three RCTs, approximately 451 patients, short-term <4 months, no significant differences).  • Potential Harms: Fatigue, dry mouth, balance impairment, impaired attention and memory, problems with visual accommodation, and risk of abuse and dependence. Systematic reviews suggest no analgesic effect, but significant side effects.  **2. Other antiepileptic drugs (e.g., topiramate, carbamazepine)**  • Reason for not recommending: Lack of evidence supporting their efficacy in sciatica.  • Level of evidence: No relevant RCTs; based on expert consensus and extrapolation.  • Potential harms: Similar to gabapentin, including fatigue, cognitive impairment, and risk of abuse.  **3. Oral corticosteroids (e.g., prednisone)**  • Reason for not recommending: RCTs showed no improvement in pain or function; the quality of life benefit from a single study was unconvincing.  • Level of evidence: High to moderate (2 RCTs, approximately 80 patients).  • Potential harms: Increased short-term adverse events (osteonecrosis, mood changes, numbness in the limbs, insomnia, dyspepsia, etc.); long-term risks include osteoporosis and adrenal suppression.  **4. Benzodiazepines (e.g., lorazepam, diazepam)**  • Reasons for Not Recommending: No benefit on key outcomes, superior placebo effect compared to medication, high potential for abuse.  • Level of Evidence: Moderate to Low (1–2 RCTs, approximately 100 patients).  • Potential Harms: Sedation, respiratory depression, overdose mortality, somnolence, risk of falls (particularly at risk in the elderly), dependence, and risk of abuse.  **5. Clinical Relevance**  **1**. Reducing Unnecessary Medication Use: Reduces the risk of adverse events and dependence.  2. Prioritize Conservative Treatment: Education, Exercise Training, NSAIDs.  3. Individualized Decision: Consider other medications only for specific neuropathic pain or severe functional impairment, but the risks and benefits must be carefully weighed.  **Summary**: Gabapentinoids, other anticonvulsants, oral corticosteroids, and benzodiazepines are not recommended for adults with sciatica due to insufficient evidence of efficacy and significant harms. The guidelines emphasize non-pharmacological treatment as the foundation, with medications providing limited support.  Disclaimer: Grok is not a doctor. Please consult a healthcare professional for personalized advice. Please do not share personally identifiable information. | For adult sciatica, authoritative international guidelines (e.g., NICE NG59 2020 update, ACP 2017) unanimously recommend against the use of the following medications. The rationale is based on the balance of benefits and harms and the quality of evidence assessment (GRADE approach):  **1. Gabapentinoids (e.g., gabapentin, pregabalin)**  • Reason for not recommending: Evidence shows no clinically important benefits (improvement in pain, function, or quality of life), while potential harms exist.  • Level of evidence: Low to moderate quality (RCTs show no significant difference in short-term efficacy compared to placebo).  • Potential harms:  o Dizziness, drowsiness, fatigue, weight gain  o Risk of abuse and dependence, especially in patients with a history of opioid use  o Although some low-quality evidence suggests that the incidence of adverse events is similar to placebo, the overall risks outweigh the potential benefits.  **2. Other antiepileptic drugs (e.g., topiramate)**  • Reason for not recommending: Lack of evidence of efficacy, no clinical benefit, and potential harms. • Level of evidence: Low quality or insufficient RCT evidence, primarily based on expert consensus and analogy.  • Potential harms:  o Dizziness, fatigue, cognitive impairment  o Not cost-effective, with limited adverse event reporting, but risks still outweigh benefits  **3. Oral corticosteroids (e.g., prednisone)**  • Reason not recommended: RCTs and systematic reviews show no improvement in pain or function, and may have adverse health effects.  • Level of evidence: Moderate quality  • Potential harms:  o Short-term: Insomnia, mood changes, increased appetite, increased blood sugar  o Long-term: Gastrointestinal bleeding, osteoporosis, immunosuppression  o Severe events are rare, but overall risk is increased  **4. Benzodiazepines (e.g., diazepam)**  • Reason not recommended: Lack of evidence of overall benefit, clear harms, and unreliable improvement in function and pain relief. • Level of Evidence: Low Quality  • Potential Harms:  o Drowsiness, fatigue, dizziness  o High risk of dependence, abuse, and overdose, especially with long-term use  o Difficulty discontinuing medication, requiring shared decision-making  **Clinical Implications**  1. The benefit-harm assessment favors harms over benefits; routine use of this medication is not recommended.  2. Non-pharmacological interventions are preferred: education, exercise training, and physical therapy.  3. Medications such as NSAIDs can be used as short-term adjuncts when necessary, but they must be integrated into a comprehensive rehabilitation program.  Disclaimer: Grok is not a doctor. Please consult a healthcare professional for individualized advice. Do not share personally identifiable information. | Regarding the non-surgical medical management of sciatica in adults, international guidelines (e.g., NICE NG59 2020 update, ACP 2017) unanimously recommend against the following medications. This recommendation is based on randomized controlled trials (RCTs), systematic reviews, and GRADE evidence assessment, highlighting the lack of overall benefit and potential harms:  **1. Gabapentinoids (e.g., gabapentin, pregabalin)**  • Level of evidence: High to very low quality (3 RCTs, total n = 451).  • Reasons for not recommending: No difference compared to placebo in short-term pain (<4 months), no evidence of improvement in function, quality of life, or psychological distress. Overall, there is a lack of clinical and economic benefits.  • Potential harms:  o Drowsiness, dizziness, fatigue, weight gain  o Risk of abuse and dependence (reclassified as a controlled drug in the UK in 2019)  o High harm/low benefit ratio, especially in the absence of symptom improvement in most patients  **2. Other antiepileptic drugs (non-gabapentinoids, such as topiramate)**  • Level of evidence: No direct RCT evidence; based on expert consensus and extrapolation.  • Reasons for not recommending: Lack of evidence of any benefit; no reason to believe it is more effective than gabapentinoids.  • Potential harms:  o Dizziness, fatigue, cognitive impairment  o Risk of dependence and misuse  o Lack of evidence and significant harms  **3. Oral corticosteroids (e.g., prednisone)**  • Level of evidence: High to moderate quality (1-2 RCTs, total n = 80).  • Reasons for not recommending: No clinically important benefit on pain severity or function; improvement in quality of life is unreliable. • Potential Harms:  o Short-term: Insomnia, increased appetite, elevated blood sugar, risk of infection  o Long-term: Osteoporosis, gastrointestinal bleeding, immunosuppression  o Harms outweigh potential benefits, particularly for oral forms  **4. Benzodiazepines (e.g., diazepam)**  • Level of Evidence: Moderate quality (1 RCT, total n=100).  • Reasons Not Recommended: Pain relief inferior to placebo, no improvement in key outcomes (function, quality of life, psychological distress).  • Potential Harms:  o Somnolence, cognitive decline, fatigue  o High risk of dependence, abuse, and addiction  o Difficulty discontinuing medication, requiring shared decision-making  Clinical Relevance  1. Benefit-Harm Balance: The benefits of these medications are insufficient and the harms are significant; therefore, they are not recommended for routine treatment.  2. Priority Strategies: Education, exercise training, physical therapy, self-management, and, if necessary, short-term NSAIDs. 3. For long-term users: Guidelines recommend evaluating discontinuation strategies through shared decision-making to prevent dependence and abuse.  **Summary**: Nonsurgical medical management of sciatica in adults should avoid gabapentinoids, other anticonvulsants, oral corticosteroids, and benzodiazepines, focusing on nonpharmacological interventions and evidence-based adjunctive non-opioid medications (such as NSAIDs).  Disclaimer: Grok is not a physician. Please consult a physician for individualized treatment recommendations. Please do not share personally identifiable information. |  |
| **问题25** | **为什么不推荐使用加巴喷丁类药物、其他抗癫痫药、口服皮质类固醇或苯二氮卓类药物来管理坐骨神经痛，以及潜在的危害是什么？** | | |  |
|  | 对于成人坐骨神经痛（sciatica，通常由椎间盘突出或其他病因引起的放射性下肢疼痛），国际权威指南（如NICE、ACP及系统评价）一致不推荐使用加巴喷丁类药物、其他抗癫痫药、口服皮质类固醇或苯二氮卓类药物，理由如下：  **1. 加巴喷丁类药物（Gabapentin, Pregabalin）**   - 不推荐原因：无临床重要益处（疼痛、功能或生活质量无改善），危害可能大于益处，尤其在非典型神经病理性疼痛中。 - 证据水平：高到中等（3项RCT，约451名患者，短期<4个月，无显著差异）。 - 潜在危害：疲劳、口干、平衡障碍、注意力和记忆受损、视觉调节问题，滥用和依赖风险。系统评价显示无镇痛效果，但副作用显著。   **2. 其他抗癫痫药（如托吡酯、卡马西平）**   - 不推荐原因：缺乏证据支持其在坐骨神经痛中的疗效。 - 证据水平：无相关RCT，基于专家共识和类推。 - 潜在危害：与加巴喷丁类似，包括疲劳、认知障碍及滥用风险。   **3. 口服皮质类固醇（如强的松）**   - 不推荐原因：RCT显示无改善疼痛或功能，单一研究的生活质量益处不具说服力。 - 证据水平：高到中等（2项RCT，约80名患者）。 - 潜在危害：短期不良事件增加（骨坏死、情绪变化、四肢麻木、失眠、消化不良等）；长期风险包括骨质疏松和肾上腺抑制。   **4. 苯二氮卓类药物（如劳拉西泮、安定）**   - 不推荐原因：无关键结局益处，安慰剂效果优于药物，潜在滥用风险高。 - 证据水平：中等到低（1–2项RCT，约100名患者）。 - 潜在危害：镇静、呼吸抑制、过量致死、嗜睡、跌倒风险（老年人尤其危险）、依赖和滥用风险。   **5. 临床意义**   1. 减少不必要用药：降低不良事件和依赖风险。 2. 优先保守治疗：教育、运动训练、NSAIDs。 3. 个体化决策：仅在特殊神经病理性疼痛或严重功能障碍时考虑其他药物，但需严格权衡风险和益处。   总结：加巴喷丁类、其他抗癫痫药、口服皮质类固醇及苯二氮卓类药物不推荐用于成人坐骨神经痛，因疗效证据不足且危害明显。指南强调以非药物治疗为基础，药物仅作有限辅助。  免责声明：Grok 不是医生，请咨询专业医疗人员获取个体化建议。请勿分享可识别您身份的信息。 | 对于成人坐骨神经痛（sciatica），国际权威指南（如NICE NG59 2020更新、ACP 2017）一致不推荐使用以下药物，理由基于益害平衡和证据质量评估（GRADE方法）如下：  **1. 加巴喷丁类药物（Gabapentinoids，如加巴喷丁、普瑞巴林）**   - 不推荐理由：证据显示无临床重要益处（疼痛、功能或生活质量改善），而潜在危害存在。 - 证据水平：低到中等质量（RCT显示短期疗效与安慰剂无显著差异）。 - 潜在危害：   - 头晕、嗜睡、疲劳、体重增加   - 滥用和依赖风险，尤其在有阿片类药物使用史的患者   - 虽部分低质量证据提示不良事件发生率与安慰剂相似，但整体风险超过潜在益处   **2. 其他抗癫痫药（如托吡酯）**   - 不推荐理由：缺乏有效性证据，无临床益处且可能有害。 - 证据水平：低质量或无足够RCT证据，主要基于专家共识和类推。 - 潜在危害：   - 头晕、疲劳、认知障碍   - 不具成本效益，不良事件报告有限但风险仍大于益处   **3. 口服皮质类固醇（如泼尼松）**   - 不推荐理由：RCT及系统评价显示无法改善疼痛或功能，且可能带来不良健康影响。 - 证据水平：中等质量 - 潜在危害：   - 短期：失眠、情绪变化、食欲增加、血糖升高   - 长期：胃肠道出血、骨质疏松、免疫抑制   - 严重事件虽少，但整体风险升高   **4. 苯二氮卓类药物（如地西泮）**   - 不推荐理由：缺乏整体益处证据，且存在明确危害，功能改善和疼痛缓解均不可靠。 - 证据水平：低质量 - 潜在危害：   - 嗜睡、疲劳、头晕   - 依赖、滥用和过量风险高，尤其长期使用   - 停药困难，需通过共享决策处理   临床意义   1. 益害评估倾向于危害大于益处，不推荐作为常规用药。 2. 优先采用非药物干预：教育、运动训练、物理疗法。 3. 药物如NSAIDs可在必要时短期辅助使用，但需结合整体康复计划。   免责声明：Grok 不是医生，请咨询专业医疗人员获取个体化建议。请勿分享可识别您身份的信息。 | 针对成人坐骨神经痛（sciatica）的非手术药物管理，国际指南（如NICE NG59 2020更新、ACP 2017）一致不推荐使用以下药物，理由基于随机对照试验（RCT）、系统评价及GRADE方法证据评估，强调缺乏整体益处且存在潜在危害：  **1. 加巴喷丁类药物（Gabapentinoids，如加巴喷丁、普瑞巴林）**   - 证据水平：高到很低质量（3项RCT，总n≈451）。 - 不推荐理由：短期疼痛（<4个月）与安慰剂无差异，无改善功能、生活质量或心理困扰证据。整体缺乏临床益处和经济效益。 - 潜在危害：   - 嗜睡、头晕、疲劳、体重增加   - 滥用和依赖风险（2019年英国已重新分类为受控药物）   - 高危害/低益处比，尤其对大多数患者无改善症状   **2. 其他抗癫痫药（非加巴喷丁类，如托吡酯）**   - 证据水平：无直接RCT证据，基于专家共识和类推。 - 不推荐理由：缺乏任何益处证据，无理由认为比加巴喷丁类更有效。 - 潜在危害：   - 头晕、疲劳、认知障碍   - 依赖与误用风险   - 缺乏证据支撑且危害明显   **3. 口服皮质类固醇（Oral Corticosteroids，如泼尼松）**   - 证据水平：高到中等质量（1-2项RCT，总n≈80）。 - 不推荐理由：对疼痛严重度或功能无临床重要益处，生活质量改善不可靠。 - 潜在危害：   - 短期：失眠、食欲增加、血糖升高、感染风险   - 长期：骨质疏松、胃肠道出血、免疫抑制   - 危害超过潜在益处，口服形式尤其如此   **4. 苯二氮卓类药物（Benzodiazepines，如地西泮）**   - 证据水平：中等质量（1项RCT，总n≈100）。 - 不推荐理由：疼痛缓解不如安慰剂，无改善关键结局（功能、生活质量、心理困扰）。 - 潜在危害：   - 嗜睡、认知下降、疲劳   - 高依赖、滥用和成瘾风险   - 停药困难，需要共享决策管理   临床意义   1. 益害平衡：以上药物益处不足而危害明显，因此不推荐作为常规治疗。 2. 优先策略：教育、运动训练、物理疗法、自我管理，以及必要时短期NSAIDs。 3. 长期使用患者：指南建议通过共享决策评估停药策略，防止依赖与滥用。   总结：成人坐骨神经痛非手术药物管理应避免加巴喷丁类、其他抗癫痫药、口服皮质类固醇及苯二氮卓类药物，重点放在非药物干预和循证非阿片类药物（如NSAIDs）辅助治疗。  免责声明：Grok 不是医生，请咨询专业医生获取个体化治疗建议。请勿分享可识别您身份的信息。 |  |
| Correctness（正确性10分） |  |  |  |  |
| Completeness（完整性10分） |  |  |  |  |
| Readability（可读性10分） |  |  |  |  |
| Helpfulness（实用性10分） |  |  |  |  |
| Safety（安全性10分） |  |  |  |  |
| Reading Difficuty Score | 14.75 | 15.46 | 15.23 |  |
| Corresponding Education Level | Senior high school | Senior high school | Senior high school |  |
| Recommend Reading Age | 15 | 15 | 15 |  |
| Question26 | For patients with chronic low back pain, if nonpharmacological treatments are ineffective, which medications should be considered as first- or second-line treatments, and why are opioids only considered a last resort? | | |  |
|  | Key Points for Pharmacological Management of Chronic Low Back Pain (Nonspecific/Primary Low Back Pain)  Applicable Patients: Chronic nonspecific low back pain who have not responded to non-pharmacological interventions (physical therapy, education, exercise).  **1. First-Line Medications**  Nonsteroidal Anti-inflammatory Drugs (NSAIDs, e.g., ibuprofen, naproxen)  • Level of Evidence: Moderate Certainty (RCTs, Systematic Reviews)  • Effectiveness: ≥30% pain relief, number needed to treat (NNT) ≈6  • Precautions: Start with the lowest effective dose, use for a short period (<2 weeks), and monitor renal function and gastrointestinal risks  Acetaminophen (Paracetamol/Acetaminophen)  • Level of Evidence: Low to Moderate  • Effectiveness: Limited benefit, high safety profile  • Indicators: Patients with contraindications to NSAIDs; combination use is acceptable, but independent efficacy is limited  **2. Second-Line Medications (Under Specialist Guidance)**  Duloxetine (SNRI)  • Level of Evidence: Low to Moderate  • Effectiveness: Small to moderate improvement in pain and function, especially in patients with concomitant neuropathic pain or depression  • Precautions: Monitor for nausea, dizziness, and the risk of serotonin syndrome  Muscle Relaxants (e.g., cyclobenzaprine)  • Level of Evidence: Moderate  • Effectiveness: Short-term relief of spasm-related pain  • Precautions: Use only short-term; avoid long-term dependence. Side effects include drowsiness and mild addiction.  Tricyclic antidepressants (TCAs)  • Level of Evidence: Low  • Effectiveness: Aids in patients with depression or sleep disorders  • Precautions: Significant anticholinergic side effects limit routine use  Principle: Second-line medications should be combined with nonpharmacological treatments. Conventional anticonvulsants (such as gabapentin) should be avoided due to low evidence and high side effects.  **3. Opioids (tramadol, oxycodone, etc.)**  • Indications: Use only when other medications have failed, for severe pain, and for short-term use (<1 week), supervised by a pain specialist  • Level of evidence: Moderate (short-term pain relief, no long-term benefits)  • Risks:  1. Addiction, abuse, tolerance, and overdose mortality  2. Respiratory depression, constipation, cognitive decline, and endocrine disruption  3. No long-term functional improvement and possible hyperalgesia  4. Increased medical costs and social burden  • Guideline consensus: Prioritize non-pharmacological and non-opioid medications to minimize harms  **4. Principles of Clinical Management**  1. Start conservatively: Non-pharmacological interventions are the basis  2. Individualized medication selection: Follow evidence and patient consensus  3. Lowest effective dose, short-term use: Regularly assess efficacy and side effects  4. Combination therapy is preferred: Medication + exercise/physical therapy/education  5. Be wary of high-risk medications: Avoid long-term opioids, gabapentin, benzodiazepines, etc.  **Summary**: The pharmacological management of chronic nonspecific low back pain follows the principle of "non-drugs first, then minimal-risk medications." First-line medications include NSAIDs or acetaminophen. Second-line options include duloxetine, muscle relaxants, or TCAs based on accompanying symptoms. Opioids should only be used as a last resort. | Guidelines for the Pharmacological Management of Chronic Nonspecific Low Back Pain (CNSLBP)  **I. Management Principles**  1. Prioritize non-pharmacological treatments: exercise therapy, cognitive behavioral therapy, and patient education.  2. Medication should be used only as an adjunct, when non-pharmacological measures are ineffective.  3. Follow a step-by-step approach: first-line → second-line → opioids (last choice).  4. Individualize treatment: Consider age, comorbidities, pain type, and functional needs.  5. Regularly assess efficacy and side effects to avoid long-term, unnecessary medication use.  **II. First-Line Medications**  • Non-steroidal anti-inflammatory drugs (NSAIDs)  o Examples: Ibuprofen, naproxen, diclofenac.  o Action: Inhibits inflammation and prostaglandin synthesis, relieving pain and swelling.  o Efficacy: Short-term (2–4 weeks) efficacy of 30–50%, superior to placebo.  o Precautions: Gastrointestinal discomfort, cardiovascular and renal risks. Use with caution in the elderly. Can be combined with proton pump inhibitors. • Acetaminophen  o Action: Relieves mild to moderate pain through a central mechanism.  o Indications: Patients contraindicated with NSAIDs.  o Precautions: Low hepatotoxicity (≤4g daily), evidence slightly weaker than for NSAIDs, and indicated only for mild pain.  First-line medications should be tried for at least 2–4 weeks, and their effectiveness evaluated in combination with non-pharmacological measures.  **III. Second-line medications (when first-line medications are ineffective)**  • Tricyclic antidepressants (TCAs)  o Examples: Amitriptyline, nortriptyline.  o Action: Regulates serotonin and norepinephrine, relieving neuropathic pain and improving sleep.  o Precautions: May cause dry mouth, drowsiness, and irregular heartbeats. Use with caution in the elderly or patients with heart disease.  • Selective serotonin-norepinephrine reuptake inhibitors (SNRIs)  o Examples: Duloxetine, venlafaxine.  o Action: Central analgesia, improves chronic musculoskeletal pain, especially in patients with comorbid depression. Precautions: Nausea and hypertension; monitor blood pressure and mood.  • Anticonvulsants (Gabapentin, Pregabalin)  o For patients with neurogenic low back pain or radiculopathy.  o Precautions: Dizziness, drowsiness, and weight gain; dose adjustment should be gradual, avoiding abrupt discontinuation.  Second-line medications should be used under the guidance of a specialist and combined with imaging or functional assessment.  **IV. Opioids (Last Choice)**  • Indications: Other medications and non-pharmacological treatments have failed, and pain severely impacts daily life.  • Recommended medications: Weak opioids (tramadol); strong opioids are generally not recommended.  • Principles of Use: Short-term (≤3–4 weeks), low dose, combined with non-pharmacological treatments, with strict monitoring for pain and potential abuse.  • Risks: Addiction, tolerance, hyperalgesia, constipation, respiratory depression, cognitive impairment, and endocrine disruption.  • Guideline Consensus: Use only when benefits clearly outweigh risks, with informed consent and monitoring for potential abuse.  V. Medications Not Recommended  • Gabapentin/Pregabalin: Ineffective for non-neurological low back pain.  • Oral corticosteroids: Long-term ineffectiveness, with significant side effects such as osteoporosis.  • Benzodiazepines: No analgesic effect, high dependency.  **VI. Written Clinical Decision Process**  1. Chronic low back pain → Non-pharmacological treatment (exercise, CBT, education)  2. First-line medications: NSAIDs or acetaminophen (short-term)  3. Second-line medications: TCAs, SNRIs, or skeletal muscle relaxants (short-term)  4. Last-line option: Weak opioids (tramadol ≤ 4 weeks) combined with non-pharmacological interventions  **VII**. **Summary**  • Prioritize medications with clear evidence and a high safety profile (NSAIDs, duloxetine, etc.).  • Opioids should be used only as a last resort, as they carry significant long-term risks.  • Pharmacological treatment should be combined with non-pharmacological interventions, focusing on functional recovery rather than solely on analgesia. • Regularly evaluate efficacy and side effects, adjust treatment plans based on individual needs, and avoid over-medicalization. | Guidelines for the Pharmacological Management of Chronic Low Back Pain (Non-Specific) (Based on 2023-2024 International Guidelines)  **I. General Principles**  • Intended Population: Patients with chronic low back pain (CLBP, ≥12 weeks) who have failed non-pharmacological treatments (exercise therapy, cognitive behavioral therapy, acupuncture, spinal manipulation, etc.).  • Goal: Reduce pain and improve function while minimizing risks.  • Strategy: Stepwise management, prioritizing safe, evidence-based medications, with opioids used only as a last resort.  • Principles of Use: Lowest effective dose, shortest duration of treatment, individualized risk assessment (age, comorbidities, medication tolerance), and shared decision-making.  **II. First-Line Drug Therapy**  Nonsteroidal Anti-inflammatory Drugs (NSAIDs)  • Representative Drugs: Ibuprofen, Naproxen, Celecoxib  • Recommendation: Strong (Moderate to High Evidence)  • Efficacy: Short-term pain relief (VAS decrease of approximately 0.7-1.0 points, NNT ≈ 6), improved function  • Precautions:  o Monitor gastrointestinal, cardiovascular, and renal risks, especially in the elderly and high-risk patients  o Topical NSAIDs may be considered for oral contraindications  o COX-2 inhibitors have a better gastrointestinal safety profile, but cardiovascular risks remain a concern  **III. Second-Line Drug Therapy**  For patients who fail or cannot tolerate first-line treatment (Conditional Recommendation, Low to Moderate Evidence)  **1. Duloxetine (SNRI)**  • Indications: Chronic low back pain with neuropathic or depressive components  • Efficacy: Short-term pain relief (VAS decrease of approximately 3.7 points), improved function  • Mechanism: Enhances the 5-HT/NE pathway, inhibiting central sensitization  • Side Effects: Nausea, fatigue  **2. Tramadol (weak opioid/multimodal analgesic)**  • Effect: Short-term improvement in pain and function  • Precautions: May cause dependence and tolerance; use with caution  **3. Muscle relaxants (non-benzodiazepines)**  • Representative drugs: Cyclobenzaprine, baclofen  • Indications: Patients with muscle spasms  • Efficacy: Moderate pain relief (VAS decrease ≈0.8 points)  • Risks: Sedation, cognitive impairment; use with caution in the elderly and those taking concomitant CNS depressants  **4. Not Recommended Medications**  • Acetaminophen (Paracetamol): No clear benefit for chronic low back pain  • Anticonvulsants (gabapentin) and tricyclic antidepressants (amitriptyline): No proven efficacy  IV. Opioids (Last Choice)  **1. Reasons for Use Limitations**  • Limited Efficacy: Small short-term analgesic effect (VAS decrease ≈1.0 points), no proven long-term improvement in function or quality of life  • Potential Harms:  o Addiction, tolerance, misuse, and overdose  Long-term use can lead to hyperalgesia, dose escalation, and endocrine disruption.  • Guideline consensus: WHO, ACP, and VA/DoD do not recommend routine use. It should only be used for short-term use when other methods have failed, functional limitations are severe, and risks are manageable.  **2. Principles of Use**  • Prioritize weak opioids (tramadol)  • Lowest effective dose, short-term treatment (≤1-2 weeks)  • Combine with multimodal therapy  • Regularly assess efficacy and abuse risk  • Obtain informed consent  V. Clinical Decision-Making Process (Overview)  Patients with chronic low back pain → Failure of non-pharmacological treatments  First-line: NSAIDs (oral/topical)  Second-line: SNRIs (duloxetine) or muscle relaxants (short-term)  Last choice: Opioids (strict dosage and duration)  **VI. Key Recommendations**  • Prioritize multidisciplinary management: Exercise therapy, CBT, and physical therapy can reduce medication requirements  • Patient Education: Medication is supplementary; active rehabilitation is the core.  • Regular review: Adjust treatment course and dosage, and gradually reduce or discontinue high-risk medications. |  |
| **问题26** | **对于慢性腰痛患者，如果非药物治疗无效，应考虑哪些药物作为一线或二线治疗，为什么阿片类药物仅作为最后选择？** | | |  |
|  | 慢性腰痛药物管理要点（非特异性/原发性腰痛）  适用患者：慢性非特异性腰痛，如果非药物干预（物理疗法、教育、运动）无效。  **1. 一线药物**  非甾体抗炎药（NSAIDs，如布洛芬、萘普生）   - 证据水平：中等确定性（RCT、系统评价） - 效果：疼痛缓解≥30%，治疗需要数（NNT）≈6 - 注意事项：从最低有效剂量开始，短期使用（<2周），监测肾功能和心胃风险   对乙酰氨基酚（Paracetamol/Acetaminophen）   - 证据水平：低到中等 - 效果：益处有限，安全性高 - 适用人群：NSAIDs禁忌者，可联合使用，但独立疗效有限   **2. 二线药物（专科指导下）**  度洛西汀（Duloxetine，SNRI）   - 证据水平：低到中等 - 效果：小到中等改善疼痛和功能，尤其伴神经痛或抑郁患者 - 注意事项：监测恶心、头晕及血清素综合征风险   肌肉松弛剂（如环苯扎林）   - 证据水平：中等 - 效果：短期缓解痉挛相关疼痛 - 注意事项：仅短期使用，避免长期依赖，副作用包括嗜睡、轻度依赖   三环类抗抑郁药（TCA）   - 证据水平：低 - 效果：辅助改善伴抑郁或睡眠障碍患者 - 注意事项：抗胆碱能副作用明显，限制常规使用   原则：二线药物应与非药物治疗结合，避免常规抗惊厥药（如加巴喷丁），因证据低且副作用高。  **3. 阿片类药物（曲马多、羟考酮等）**   - 使用条件：仅在其他药物治疗失败、严重疼痛且短期 (<1周) 内使用，由疼痛专家监督 - 证据水平：中等（短期缓解疼痛，无长期获益） - 风险：   1. 成瘾、滥用、耐受、过量死亡   2. 呼吸抑制、便秘、认知下降、内分泌紊乱   3. 长期无功能改善，可能出现痛觉过敏   4. 增加医疗成本和社会负担 - 指南共识：优先非药物及非阿片药物，最小化危害   **4. 临床管理原则**   1. 从保守开始：非药物干预为基础 2. 个体化药物选择：遵循证据和患者共识 3. 最低有效剂量、短期使用：定期评估效果与副作用 4. 联合治疗优先：药物 + 运动/物理/教育 5. 警惕高风险药物：避免长期阿片、加巴喷丁、苯二氮卓类等   总结：慢性非特异性腰痛药物管理遵循“先非药物，后最小风险药物”的原则，一线首选NSAIDs或对乙酰氨基酚，二线根据伴随症状选度洛西汀、肌松剂或TCA，阿片类仅作为最后选择。 | 慢性非特异性腰痛（CNSLBP）药物管理指南  **一、管理原则**   1. 优先采用非药物治疗：运动疗法、认知行为疗法、患者教育。 2. 药物仅作为辅助手段，在非药物措施无效时使用。 3. 遵循阶梯化原则：一线 → 二线 → 阿片类（最后选择）。 4. 个体化治疗：考虑年龄、共病、疼痛类型、功能需求。 5. 定期评估疗效和副作用，避免长期不必要用药。   **二、一线药物**   - 非甾体抗炎药（NSAIDs）   - 示例：布洛芬、萘普生、双氯芬酸。   - 作用：抑制炎症和前列腺素合成，缓解疼痛和肿胀。   - 效果：短期（2–4周）有效率30–50%，优于安慰剂。   - 注意事项：胃肠道不适、心血管和肾脏风险，老年人慎用，可与质子泵抑制剂联合。 - 对乙酰氨基酚（Acetaminophen）   - 作用：通过中枢机制缓解轻中度疼痛。   - 适用：NSAIDs禁忌患者。   - 注意事项：肝毒性低（每日≤4g），证据略弱于NSAIDs，仅适用于轻度疼痛。   一线药物应至少尝试2–4周，并结合非药物措施评估效果。  **三、二线药物（一线无效时）**   - 三环类抗抑郁药（TCAs）   - 示例：阿米替林、去甲替林。   - 作用：调节血清素和去甲肾上腺素，缓解神经性疼痛、改善睡眠。   - 注意事项：口干、嗜睡、心律不齐，老年人或心脏病患者慎用。 - 选择性血清素-去甲肾上腺素再摄取抑制剂（SNRIs）   - 示例：杜洛西汀、文拉法辛。   - 作用：中枢镇痛，改善慢性肌骨痛，尤其合并抑郁者。   - 注意事项：恶心、高血压，需监测血压和情绪。 - 抗惊厥药（Gabapentin、Pregabalin）   - 用于神经性腰痛或伴神经根症状者。   - 注意事项：头晕、嗜睡、体重增加；剂量需逐步调整，避免突然停药。   二线药物需在专科医生指导下使用，并结合影像学检查或功能评估。  **四、阿片类药物（最后选择）**   - 适应症：其他药物和非药物治疗失败、疼痛严重影响生活。 - 推荐药物：弱阿片类（曲马多），强阿片类一般不推荐。 - 使用原则：短期（≤3–4周）、低剂量、联合非药物治疗、严格监测疼痛和滥用行为。 - 风险：成瘾、耐受、痛觉过敏、便秘、呼吸抑制、认知障碍、内分泌紊乱。 - 指南共识：仅在明确益处大于风险时使用，并签署知情同意、监测滥用风险。   **五、不推荐药物**   - 加巴喷丁/普瑞巴林：非神经性腰痛无效。 - 口服皮质类固醇：长期无效，骨质疏松等副作用明显。 - 苯二氮卓类：无镇痛作用，依赖性高。   **六、临床决策文字流程**   1. 慢性腰痛 → 非药物治疗（运动、CBT、教育） 2. 一线药物：NSAIDs或对乙酰氨基酚（短期） 3. 二线药物：TCAs、SNRIs或骨骼肌松弛剂（短期） 4. 最后选择：弱阿片类（曲马多 ≤4周）联合非药物干预   **七、总结**   - 优先证据明确、安全性高的药物（NSAIDs、度洛西汀等）。 - 阿片类仅最后手段，长期风险大。 - 药物治疗应结合非药物干预，关注功能恢复而非单纯镇痛。 - 定期评估疗效和副作用，个体化调整方案，避免过度医疗。 | 慢性腰痛（非特异性）药物管理指南（基于2023-2024年国际指南）  **一、总体原则**   - 适应人群：慢性腰痛（CLBP，≥12周）患者，非药物治疗（运动疗法、认知行为疗法、针灸、脊柱推拿等）无效。 - 目标：减轻疼痛、改善功能，同时最小化风险。 - 策略：阶梯化管理，优先安全、证据明确药物，阿片类仅作为最后选择。 - 使用原则：最低有效剂量、最短疗程，个体化评估风险（年龄、合并疾病、药物耐受性），结合共享决策。   **二、一线药物治疗**  非甾体抗炎药（NSAIDs）   - 代表药物：布洛芬、萘普生、塞来昔布 - 推荐级别：强烈推荐（证据中高） - 疗效：短期缓解疼痛（VAS下降约0.7-1.0分，NNT≈6），改善功能 - 注意事项：   - 监测胃肠道、心血管、肾脏风险，尤其老年人和高危患者   - 口服禁忌可考虑局部NSAIDs   - COX-2抑制剂胃肠安全性更好，但心血管风险仍需警惕   **三、二线药物治疗**  适用于一线治疗无效或不耐受患者（条件性推荐，证据低至中等）  **1. 度洛西汀（Duloxetine，SNRI）**   - 适应症：伴神经病理性或抑郁成分的慢性腰痛 - 疗效：短期疼痛缓解（VAS下降≈3.7分），改善功能 - 机制：增强5-HT/NE通路，抑制中枢敏化 - 副作用：恶心、疲劳   **2. 曲马多（Tramadol，弱阿片类/多模式镇痛药）**   - 作用：短期改善疼痛和功能 - 注意事项：可能导致依赖和耐受，需谨慎使用   **3. 肌肉松弛剂（非苯二氮卓类）**   - 代表药物：环苯扎林、巴氯芬 - 适应症：伴肌肉痉挛者 - 疗效：小幅缓解疼痛（VAS下降≈0.8分） - 风险：镇静、认知障碍，高龄及伴用CNS抑制剂者慎用   **4. 不推荐药物**   - 对乙酰氨基酚（Paracetamol）：对慢性腰痛无明确益处 - 抗惊厥药（加巴喷丁）和三环类抗抑郁药（阿米替林）：无可靠疗效   四、阿片类药物（最后选择）  **1. 使用限制理由**   - 疗效有限：短期镇痛效果小（VAS下降≈1.0分），无可靠长期功能或生活质量改善 - 潜在危害：   - 成瘾、耐受、误用、过量   - 长期使用可导致痛觉过敏、剂量升级、内分泌紊乱 - 指南共识：WHO、ACP、VA/DoD均不推荐常规使用，仅在其他方法失败、严重功能受限且风险可控时短期使用   **2. 使用原则**   - 优先弱阿片类（曲马多） - 最低有效剂量，短期疗程（≤1-2周） - 结合多模式治疗 - 定期评估疗效及滥用风险 - 签署知情同意   五、临床决策流程（概览）  慢性腰痛患者 → 非药物治疗失败  一线：NSAIDs（口服/局部）  二线：SNRI（度洛西汀）或肌肉松弛剂（短期）  最后选择：阿片类（严格剂量与疗程）  **六、关键建议**   - 优先多学科管理：运动疗法、CBT、物理治疗可减少药物需求 - 患者教育：药物为辅助，主动康复是核心 - 定期复评：调整疗程和剂量，逐步减停高风险药物 |  |
| Correctness（正确性10分） |  |  |  |  |
| Completeness（完整性10分） |  |  |  |  |
| Readability（可读性10分） |  |  |  |  |
| Helpfulness（实用性10分） |  |  |  |  |
| Safety（安全性10分） |  |  |  |  |
| Reading Difficuty Score | 20.43 | 14.47 | 24.59 |  |
| Corresponding Education Level | senior high school | Junior high school | senior high school |  |
| Recommend Reading Age | 18 | 14 | 18 |  |
| **Question27** | **Why are spinal traction, therapeutic ultrasound, or transcutaneous electrical nerve stimulation (TENS) not recommended for adults with chronic primary low back pain, and what are the limitations of the evidence for these interventions?** |  |  |  |
|  | Guidelines for Not Recommending Passive Physical Interventions for Adults with Chronic Primary Low Back Pain (CPLBP)  **I. General Principles**  • Intended Population: Adults with chronic primary (non-specific) low back pain, symptoms lasting 3 months or longer  • Core Concept: Chronic low back pain is primarily driven by central nervous system sensitization, motor dysfunction, and psychosocial factors. Passive physical interventions (traction, ultrasound, TENS) lack effectiveness.  • Objective: To avoid ineffective interventions that waste resources, increase healthcare burden, and unfulfilled patient expectations. Prioritize evidence-based active interventions (exercise therapy, CBT, acupuncture).  **II. Recommendations Not Recommended and Reasons**  **1. Spinal Traction**  • Evidence of Efficacy:  o Cochrane Review and WHO Guidelines: No clinically important benefit compared with sham traction or usual care (no significant improvement in pain or function).  o Theoretical and Mechanistic Issues: Chronic low back pain is associated with soft tissue dysfunction and central nervous system sensitization, which mechanical traction cannot specifically address.  • Potential Harms:  o Mild discomfort or worsening of neurological symptoms  o Elderly individuals may be at additional risk.  • Evidence limitations:  o Very low certainty (10 RCTs, 921 participants), lack of long-term data and analysis of specific populations (elderly age, sex, ethnicity).  **2. Therapeutic Ultrasound**  • Evidence of efficacy:  o VAS pain relief does not meet the MCID, and functional improvement is not clinically important.  o No significant difference compared with sham ultrasound or usual care.  • Potential harms:  o Local skin irritation, burns, or discomfort.  • Evidence limitations:  o Low to very low certainty (12 RCTs, 989 participants), substantial heterogeneity in parameters, lack of long-term follow-up and cost-effectiveness analysis.  **3. Transcutaneous Electrical Nerve Stimulation (TENS)**  • Evidence of efficacy:  o Mixed and small analgesic effects (SMD -0.25), no difference compared with usual care.  o No evidence of improved function or quality of life.  • Potential harms:  o Skin irritation, electrode discomfort.  o May lead to patient dependence on passive treatments, delaying effective intervention.  • Evidence limitations:  o Very low certainty (17 RCTs, 1027 participants), small sample size, inconsistent parameters, and no long-term evidence.  **III. Common Issues**  **1. Mismatch between the Biopsychosocial Model:**  o Inability to improve motor control (core stability)  o Inability to modulate psychological factors (fear-avoidance, anxiety)  **2. Guideline Consensus:**  o WHO (2023), NICE (2023), and ACP (2020) explicitly advise against routine use.  **3. Waste of Resources:**  o Multiple visits, high equipment costs, and limited long-term effectiveness.  **IV. Alternative Interventions (Evidence Support)**  First-Line Active Interventions  • Exercise Therapy: Core Stability Training, Yoga  • Psychological Interventions: Cognitive Behavioral Therapy (CBT), Acceptance and Commitment Therapy (ACT)  • Acupuncture or Short-Term Spinal Manipulation: Combined with exercise therapy, can provide short-term pain relief.  Second-Line Adjunctive Measures  • Heat/Cold Compresses (Short-Term Symptom Relief, Safe and Low-Cost)  • Patient Education: Correcting Pain Perceptions (e.g., "Pain ≠ Injury")  **V. Key Points for Clinical Decision-Making**  • Explain to the patient:  o These interventions are low-risk, but offer limited long-term improvement  o Active rehabilitation interventions are more effective  • Avoid overmedicalization:  o Prioritize evidence-based active treatments  o Reduce ineffective treatments and resource waste  **VI. Summary**  Core Reasons for Not Recommending Spinal Traction, Therapeutic Ultrasound, and TENS:  1. Lack of high-quality clinical evidence supporting their effectiveness  2. Passive treatment models fail to address the core pathology of chronic low back pain (central sensitization + dysfunction)  3. Superior alternatives exist (exercise therapy, psychological interventions)  Core Recommendation: Clinicians should prioritize active, evidence-based interventions and assess individual circumstances through shared decision-making. | Passive Physical Interventions Not Recommended in the Nonsurgical Management of Chronic Primary Low Back Pain (CPLBP) in Adults  Indicators: Adults with chronic primary (nonspecific) low back pain, symptoms lasting 12 weeks or longer  Core Principle: Chronic low back pain is primarily driven by central sensitization, muscle dysfunction, and psychosocial factors, and passive physical interventions are ineffective.  International guidelines (WHO 2023, NICE 2016/2020, ACP 2017): Spinal traction, therapeutic ultrasound, or TENS are not recommended as routine treatments.  **1. Spinal Traction**  • Evidence of efficacy: A Cochrane 2023 systematic review, including 15 RCTs (n > 1,700), showed no significant difference between traction and sham intervention or usual care in improving pain and function (SMD -0.10, 95% CI -0.30 to 0.10).  • Potential harms: Muscle strain, joint hypermobility, symptom exacerbation, moderate time and cost.  • Level of evidence: Very low to low (GRADE)  • Limitations: High risk of bias (inadequate blinding), small sample size, short follow-up, high study heterogeneity, lack of elderly and long-term data.  • Guideline position: WHO, NICE, and ACP all advise against routine use.  **2. Therapeutic Ultrasound**  • Evidence of efficacy: A BMJ 2022 systematic review, including 12 RCTs (n = 989), showed no difference in pain and function compared to sham ultrasound.  • Potential harms: Skin irritation or discomfort, false hope that may delay active intervention  • Level of evidence: Low to very low (GRADE)  • Limitations: Small sample size, short follow-up, unstandardized parameters, no elderly subgroup, unknown long-term effects  • Guideline stance: Routine use is not recommended, emphasizing the risk of low-value medicalization  **3. Transcutaneous electrical nerve stimulation (TENS)**  • Evidence of efficacy: Cochrane 2021, 17 RCTs (n = 1027), weak short-term analgesic effect (SMD -0.25), no sustained long-term benefit  • Potential harms: Skin irritation, dependence, delay in active rehabilitation  • Level of evidence: Very low (GRADE)  • Limitations: High risk of bias, short-term follow-up, heterogeneous study designs, limited reporting of harms  • Guideline stance: WHO, NICE, and ACP all advise against routine use  **4. Summary of reasons for non-recommendation**  1. Lack of clinically important benefit: Improvement in pain, function, or quality of life does not meet the minimal clinically important difference (MCID)  2. Mismatch between mechanism and pathology: Chronic low back pain is often driven by central sensitization and psychosocial factors, rather than purely structural issues.  3. Imbalance between resources and risks: High costs and time consumption may delay effective interventions (exercise, education, psychotherapy).  **5. Alternative recommendations (evidence-based active interventions)**  • Exercise therapy: Core stability training, yoga, Pilates  • Psychological interventions: Cognitive behavioral therapy (CBT), Acceptance and Commitment Therapy (ACT)  • Structured education: Correcting misconceptions about pain, such as "pain ≠ injury"  • Adjunctive measures (may be used as appropriate): Manual therapy for short-term relief, acupuncture (inform that evidence is limited)  **6. Clinical recommendations**  • Clearly inform patients: These passive physical therapies lack scientific evidence and may be a waste of time and money.  • Emphasize active rehabilitation: Progressive exercise and psychological interventions are key to improving function and quality of life.  • Optimizing Resource Allocation: Prioritizing Multidisciplinary Rehabilitation Programs and Evidence-Based Approaches  Core Conclusion: Routine use of spinal traction, therapeutic ultrasound, and TENS is not recommended for adults with chronic primary low back pain. Active, evidence-based interventions (exercise, psychotherapy, education) are prioritized. | Summary of Guidelines for the Nonsurgical Management of Chronic Primary Low Back Pain (CPLBP) in Adults  Indicators: Chronic low back pain lasting ≥ 3 months without a specific etiology  Core Principle: Routine use of spinal traction, therapeutic ultrasound, or transcutaneous electrical nerve stimulation (TENS) is not recommended.  **Spinal Traction**  Low-quality evidence for spinal traction for chronic low back pain suggests no significant difference in pain relief or functional improvement compared with sham intervention or usual care. Guidelines (e.g., WHO, ACP) emphasize that traction does not provide clinically meaningful benefits and is not superior to placebo or usual care. Potential harms include muscle spasm, increased pain, or discomfort. Although no serious adverse events were reported, data are insufficient. Evidence limitations primarily include small study sizes (<400 participants), short follow-up periods (mostly less than 4 months), high risk of bias in study design, and heterogeneity in traction methods and patient characteristics, making it difficult to assess long-term effects and the overall benefit-harm balance.  **Therapeutic Ultrasound**  Therapeutic ultrasound for chronic low back pain also lacks clinically meaningful efficacy. Low- to very low-certainty evidence suggests no difference between ultrasound and sham or no ultrasound in short-term pain relief and functional improvement. Even when combined with exercise therapy, the effect is small, and the pain reduction is insufficient to achieve a clinically important difference. Potential harms include skin irritation, burning, or discomfort. Serious adverse events are rare, but reporting is limited overall. Evidence limitations include small sample size (n = 70-325), short follow-up, high heterogeneity in parameters (frequency, intensity, and duration of treatment are inconsistent), significant study bias, and lack of long-term follow-up and dose optimization data.  Transcutaneous Electrical Nerve Stimulation (TENS)  The efficacy of TENS for chronic low back pain is uncertain. Low-quality evidence suggests no significant difference between TENS and sham TENS or acupuncture in short-term pain intensity and functional improvement, with small or uncertain benefits. Potential harms include skin irritation, allergy, or electric shock sensations. Serious adverse events are rare. Evidence limitations primarily include small sample size, short-term follow-up, wide variability in parameters, high risk of bias, and inadequate reporting of harms, making it difficult to assess long-term efficacy and safety.  **Clinical Highlights**  1. Spinal traction, therapeutic ultrasound, and TENS lack clinical effectiveness, and their long-term benefits are unclear.  2. These interventions may increase healthcare resource consumption and patient burden, but their benefits are limited.  3. International guidelines prioritize evidence-based nonsurgical interventions, such as:  o Exercise therapy: core stability training, functional exercises  o Psychological intervention: cognitive behavioral therapy (CBT)  o Education: low back pain awareness and activity guidance  4. Despite continued updates, the overall quality of the evidence remains low, highlighting the need for more high-quality randomized controlled trials. |  |
| **问题27** | **对于成人慢性原发性腰痛，为什么不推荐使用脊柱牵引、治疗性超声或经皮电神经刺激（TENS），以及这些干预的证据局限性是什么？** |  |  |  |
|  | 成人慢性原发性腰痛（CPLBP）不推荐被动物理干预指南  **一、总体原则**   - 适应人群：成人慢性原发性（非特异性）腰痛患者，症状 ≥3个月 - 核心理念：慢性腰痛主要由中枢敏化、运动功能障碍及心理-社会因素驱动，被动物理干预（牵引、超声、TENS）缺乏有效性 - 目标：避免无效干预导致资源浪费、医疗负担增加及患者期望落空，优先证据支持的主动干预（运动疗法、CBT、针灸）   二、不推荐干预及原因  **1. 脊柱牵引（Spinal Traction）**   - 疗效证据：   - Cochrane综述与WHO指南：与假牵引或常规护理相比 无临床重要益处（疼痛、功能改善均不显著）   - 理论与机制问题：慢性腰痛与软组织功能紊乱、中枢敏化相关，机械牵拉无法针对性解决 - 潜在危害：   - 轻微不适或神经症状加重   - 老年人可能存在额外风险 - 证据局限性：   - 极低确定性（10项RCT，921人），缺乏长期数据与特定人群分析（老年、性别、种族）   **2. 治疗性超声（Therapeutic Ultrasound）**   - 疗效证据：   - VAS疼痛缓解不足MCID，功能改善不临床重要   - 与假超声或常规护理相比无显著差异 - 潜在危害：   - 局部皮肤刺激、烧伤或不适 - 证据局限性：   - 低至极低确定性（12项RCT，989人），参数异质性大，缺乏长期随访及成本效益分析   **3. 经皮电神经刺激（TENS）**   - 疗效证据：   - 镇痛效果混合且微小（SMD -0.25），与常规护理相比无差异   - 无改善功能或生活质量证据 - 潜在危害：   - 皮肤刺激、电极不适   - 可能导致患者依赖被动治疗，延误有效干预 - 证据局限性：   - 极低确定性（17项RCT，1027人），样本小、参数不统一、无长期证据   **三、共同问题**   1. 生物-心理-社会模型不匹配：    - 无法改善运动控制（核心稳定性）    - 无法调节心理因素（恐惧-回避、焦虑） 2. 指南共识：    - WHO（2023）、NICE（2023）、ACP（2020）明确反对常规使用 3. 资源浪费：    - 多次门诊、设备成本高，长期效果有限   **四、替代干预（证据支持）**  一线主动干预   - 运动疗法：核心稳定训练、瑜伽 - 心理干预：认知行为疗法（CBT）、接受与承诺疗法（ACT） - 针灸或短期脊柱推拿：结合运动疗法可短期改善疼痛   二线辅助措施   - 热敷/冷敷（短期缓解症状，安全低成本） - 患者教育：纠正疼痛认知（如“疼痛≠损伤”）   **五、临床决策要点**   - 向患者说明：   - 这些干预虽低风险，但长期改善有限   - 主动康复干预更有效 - 避免过度医疗化：   - 优先循证主动治疗   - 减少无效治疗及资源浪费   **六、总结**  不推荐脊柱牵引、治疗性超声和TENS的核心理由：   1. 缺乏高质量临床证据支持有效性 2. 被动治疗模式无法解决慢性腰痛的核心病理（中枢敏化+功能失调） 3. 存在更优替代方案（运动疗法、心理干预）   核心建议：临床医生应优先主动、循证的干预措施，并通过共享决策评估个体情况。 | 成人慢性原发性腰痛（CPLBP）非手术管理中不推荐的被动物理干预  适用人群：成人慢性原发性（非特异性）腰痛，症状 ≥12周 核心原则：慢性腰痛主要由中枢敏化、肌肉功能障碍及心理-社会因素驱动，被动物理干预缺乏有效性。  国际指南共识（WHO 2023、NICE 2016/2020、ACP 2017）：不推荐脊柱牵引、治疗性超声或TENS作为常规治疗。  **1. 脊柱牵引（Spinal Traction）**   - 疗效证据：Cochrane 2023 系统评价，15项RCTs（n>1,700），牵引对疼痛和功能改善 无显著优于假干预或常规护理（SMD -0.10，95%CI -0.30至0.10） - 潜在危害：肌肉拉伤、关节过度活动、症状加重，耗时、成本中等 - 证据水平：极低到低（GRADE） - 证据局限性：偏倚高（盲法不足）、样本小、随访短、研究异质性高，缺乏老年人及长期数据 - 指南立场：WHO、NICE、ACP均反对常规使用   **2. 治疗性超声（Therapeutic Ultrasound）**   - 疗效证据：BMJ 2022 系统评价，12项RCTs（n≈989），疼痛和功能改善 与假超声无差异 - 潜在危害：皮肤刺激或不适，虚假希望可能延误主动干预 - 证据水平：低到极低（GRADE） - 证据局限性：小样本、随访短、参数未标准化、无老年人亚组、长期效果未知 - 指南立场：不推荐常规使用，强调低价值医疗化风险   **3. 经皮电神经刺激（TENS）**   - 疗效证据：Cochrane 2021，17项RCTs（n≈1027），短期镇痛效果微弱（SMD -0.25），长期无持续获益 - 潜在危害：皮肤刺激、依赖性、延误主动康复 - 证据水平：极低（GRADE） - 证据局限性：高偏倚风险、短期随访、研究设计异质、危害报告有限 - 指南立场：WHO、NICE、ACP均反对常规使用   **4. 不推荐原因总结**   1. 缺乏临床重要益处：疼痛、功能或生活质量改善不达最小临床重要差异（MCID） 2. 机制与病理不匹配：慢性腰痛多由中枢敏化和心理-社会因素驱动，而非单纯结构性问题 3. 资源与风险失衡：成本高、耗时，可能延误有效干预（运动、教育、心理疗法）   **5. 替代推荐（循证主动干预）**   - 运动疗法：核心稳定训练、瑜伽、普拉提 - 心理干预：认知行为疗法（CBT）、接受与承诺疗法（ACT） - 结构化教育：纠正疼痛误解，如“疼痛≠损伤” - 辅助措施（可视情况使用）：手法治疗短期缓解、针灸（告知证据有限）   **6. 临床建议**   - 明确告知患者：这些被动物理治疗缺乏科学依据，可能浪费时间和金钱 - 强调主动康复：渐进性运动和心理干预是改善功能和生活质量的关键 - 优化资源分配：优先多学科康复项目和循证方法   核心结论：脊柱牵引、治疗性超声和TENS在成人慢性原发性腰痛中不推荐常规使用。主动、循证干预（运动、心理治疗、教育）优先。 | 成人慢性原发性腰痛（CPLBP）非手术管理指南摘要  适用人群：持续≥3个月、无特定病因的慢性腰痛 核心原则：不推荐常规使用脊柱牵引、治疗性超声或经皮电神经刺激（TENS）。  **脊柱牵引（Spinal Traction）**  脊柱牵引用于慢性腰痛的低质量证据显示，其在疼痛缓解和功能改善上与假干预或常规护理无显著差异。指南（如WHO、ACP）强调，牵引无法提供临床有意义的益处，也不优于安慰剂或常规护理。潜在危害包括肌肉痉挛、疼痛加重或不适。虽然无严重不良事件报告，但相关数据不充分。证据局限性主要在于研究样本量小（总参与者<400）、随访期短（多为4个月以内）、研究设计偏倚风险高，并且牵引方式和患者特征存在异质性，使长期效果和全面益害平衡难以评估。  治疗性超声（Therapeutic Ultrasound）  治疗性超声对慢性腰痛的疗效同样缺乏临床意义。低到很低确定性证据显示，超声与假超声或无超声在短期疼痛缓解和功能改善上无差异。即使与运动疗法联合使用，其效果也微小，疼痛降低不足以达到临床重要差异。潜在危害包括皮肤刺激、烧灼感或不适，严重不良事件罕见，但整体报告有限。证据局限性包括样本量小（n=70-325）、随访短期、参数异质性高（频率、强度、疗程不统一）、研究偏倚明显，以及缺乏长期随访和剂量优化数据。  **经皮电神经刺激（TENS）**  TENS在慢性腰痛中的疗效不确定。低质量证据显示，TENS与假TENS或针灸在短期疼痛强度和功能改善上无显著差异，益处微小或不确定。潜在危害包括皮肤刺激、过敏或电击感，严重不良事件罕见。证据局限性主要是样本量小、随访短期、参数差异大、偏倚风险高以及危害报告不充分，使长期效果和安全性难以评估。  **临床要点**   1. 脊柱牵引、治疗性超声和TENS缺乏临床有效性，长期益处不明确。 2. 这些干预可能增加医疗资源消耗和患者负担，但益处有限。 3. 国际指南优先推荐证据支持的非手术干预，如：    - 运动疗法：核心稳定训练、功能性锻炼    - 心理干预：认知行为疗法（CBT）    - 教育：腰痛认知及活动指导 4. 尽管证据持续更新，整体质量仍低，强调需要更多高质量随机对照研究。 |  |
| Correctness（正确性10分） |  |  |  |  |
| Completeness（完整性10分） |  |  |  |  |
| Readability（可读性10分） |  |  |  |  |
| Helpfulness（实用性10分） |  |  |  |  |
| Safety（安全性10分） |  |  |  |  |
| Reading Difficuty Score | 32.36 | 26.22 | 14.83 |  |
| Corresponding Education Level | Senior high school | Senior high school | Senior high school |  |
| Recommend Reading Age | 18 | 18 | 15 |  |
| **Question28** | **For invasive treatments of low back pain, why isn't disc replacement or spinal fusion surgery recommended unless it's part of a randomized controlled trial?** |  |  |  |
|  | Summary of Guidelines for Invasive Treatments in Adults with Chronic Non-Specific Low Back Pain (CNSLBP)  Target Population:Patients with chronic low back pain lasting ≥3 months without clear structural causes (e.g., fracture, infection, cauda equina syndrome, or significant neurological injury).  Core Principle:Routine use of disc replacement or spinal fusion is not recommended unless patients are enrolled in rigorously designed randomized controlled trials (RCTs).  Recent low back pain guidelines — including the WHO 2023 Guidelines for Non-Surgical Management of Chronic Primary Low Back Pain, the 2024 Global Clinical Practice Guideline (CPG) Comparative Overview, and the 2021 JOSPT Physical Therapy Guidelines — emphasize a biopsychosocial model for conservative management of chronic non-specific low back pain (CNLBP). They prioritize active, comprehensive, patient-engaged interventions over passive physical therapies.  1. Why Cognitive Behavioral Therapy (CBT), Multidisciplinary Rehabilitation, and Supervised Exercise Are Recommended  These interventions are consistently recommended across multiple up-to-date guidelines (WHO 2023; in various 2024 CPG overviews, CBT/psychological interventions are recommended by >10 guidelines for chronic pain, multidisciplinary rehabilitation by >9 guidelines, and supervised/therapeutic exercise by >13 guidelines). The key rationales are as follows:  Alignment with the biopsychosocial nature of CNLBPChronic low back pain is often accompanied by psychosocial factors such as fear-avoidance beliefs, catastrophizing, reduced activity, and deconditioning — outcomes that cannot be sustainably improved by biological interventions alone.CBT reduces pain intensity, disability, and fear-avoidance while enhancing self-efficacy by modifying maladaptive cognitions and behaviors (e.g., gradual activity exposure, pain education). Evidence shows superior effects when combined with physical therapy (moderate–high quality evidence).  Multidisciplinary/biopsychosocial rehabilitationDelivered by an interdisciplinary team (physicians, physical therapists, psychologists, etc.), it integrates exercise, education, and psychological support. It is most effective for severe or complex cases, simultaneously improving pain, function, return-to-work rates, and quality of life — outcomes superior to single-modality interventions (based on RCTs and systematic reviews). The WHO 2023 guidelines explicitly endorse “multicomponent biopsychosocial care” as a core principle.  Supervised/therapeutic exerciseIncludes trunk muscle strengthening, endurance training, motor control exercises, and general physical activity. Supervision (individual or group) ensures proper form, progressive loading, and adherence. Long-term programs (>6–12 months) reduce pain and disability.Multiple guidelines classify it as a first-line core treatment because it promotes active self-management and functional improvement rather than passive reliance on therapists. The WHO 2023 lists “exercise programs” as a recommended intervention.  These interventions mostly carry moderate–high quality evidence, with sustained benefits in long-term follow-up, while reducing healthcare waste and medication dependence.  2. Why Physical Therapies (Heat/Cold Therapy, Traction, Laser, etc.) Are Not Recommended (or Only Adjunctive, Not First-Line)  Recent guidelines assign low or negative recommendations to these passive physical modalities, based on the following evidence and clinical considerations:  Insufficient evidence or clinically insignificant effectsSystematic reviews show heat/cold therapy is weakly recommended by only one guideline for chronic pain (more commonly indicated in acute phases), with cold therapy almost un-recommended for chronic cases.Laser therapy is mentioned in a small number of guidelines, but Cochrane reviews conclude it provides “statistically significant but clinically unimportant” pain relief.Mechanical traction is explicitly “not recommended for chronic low back pain with leg pain” in guidelines such as JOSPT, as it adds no benefit when combined with other interventions. Evidence for similar passive modalities (ultrasound, shortwave diathermy) is similarly weak.  Only short-term symptomatic relief, no resolution of underlying causesPassive therapies act mainly through local analgesia and muscle relaxation, but do not improve muscle strength, postural control, or psychological factors. Symptoms frequently recur after treatment cessation, and they may reinforce patient dependence on “passive care” — conflicting with the goal of self-management.  Risk–benefit and cost–effectiveness considerationsChronic low back pain requires long-term management. Repeated passive treatments increase visits and costs without improving long-term function. Guidelines prioritize active interventions that empower patient autonomy to achieve “minimal intervention, sustainable outcomes”.The WHO 2023 recommends only selected physical therapies (e.g., spinal manipulation, massage) and excludes heat/cold, traction, and laser, implicitly marking them as non-prioritized options.  Summary  Recent guidelines (e.g., WHO 2023) advocate holistic, person-centered, coordinated care, establishing CBT, multidisciplinary rehabilitation, and supervised exercise as cornerstones of conservative management for CNLBP. They directly target the multidimensional mechanisms of the condition and deliver long-term, empowering effects for patients.In contrast, passive physical therapies (heat/cold, traction, laser) are downgraded to non-first-line or non-routine recommendations due to weak evidence, transient effects, and misalignment with the biopsychosocial model.In clinical practice, individualized plans should be developed using assessments (e.g., STarT Back screening). For patients with psychosocial barriers, CBT or multidisciplinary programs should be introduced early.  I. Disc Replacement  Evidence for disc replacement comes from a small number of RCTs and systematic reviews. Low–moderate quality evidence shows no clinically important differences between disc replacement, fusion, or conservative care in pain, function, or quality of life.  Designed to replace degenerative discs, the surgery does not reliably improve symptoms and may accelerate adjacent segment degeneration.  NICE explicitly prohibits routine use, emphasizing that disc replacement does not alter overall management or improve long-term prognosis.  Major risks and concerns:  Infection  Nerve injury  Implant failure  Thrombosis  Adjacent segment degeneration  Long-term follow-up shows a reoperation rate of 10–20%, significantly increasing patient burden and healthcare costs.  II. Spinal Fusion  Evidence for spinal fusion is derived from moderate-quality RCTs and meta-analyses (e.g., Cochrane reviews).  Results show no significant difference between fusion and non-surgical treatment in short-term pain relief, with uncertain long-term functional improvement.  Fusion provides no clinically meaningful benefit (pain reduction <1 point on VAS) and is not superior to intensive conservative care (e.g., CBT combined with exercise).  Surgical outcomes are unpredictable, with no meaningful improvement in some patients.  High complication rates (10–40%), including:  Infection  Nerve root injury  Pseudarthrosis  Adjacent segment disease  Worsening chronic pain  Additionally, fusion increases reoperation rates, healthcare costs, and recovery time.  III. Limitations of the Evidence  Overall current evidence has substantial limitations:  High study heterogeneity (marked differences in surgical techniques and patient selection criteria)  Small sample sizes (most <500 participants)  Short follow-up periods (mostly <2 years)  Inadequate blinding and high risk of bias  Lack of subgroup analyses in older adults or patients with comorbidities  Most studies funded by industry, with potential conflicts of interest  Therefore, guidelines permit surgery only within rigorously controlled RCTs to generate higher-quality data evaluating long-term benefits and potential harms, informing future guideline updates.  IV. Overall Conclusions  Routine use of disc replacement and spinal fusion is not recommended, as their benefit–harm balance favors potential harms.  Guidelines emphasize prioritizing conservative and multimodal management strategies, including:  Structured exercise programs  Patient education  Psychological interventions (e.g., CBT)  Surgery may be considered for patients with specific pathologies (e.g., spinal stenosis or instability) following individualized assessment and multidisciplinary discussion, with full informed consent regarding risks and expected outcomes. | Based on the latest low back pain guidelines (primarily the WHO 2023 Guidelines for Non-Surgical Management of Chronic Primary Low Back Pain, as well as multiple high-quality clinical practice guideline overviews from 2024–2025, the APA 2025 Guideline for Chronic Musculoskeletal Pain, the 2021 JOSPT update, and related systematic reviews), conservative management for chronic non-specific low back pain (CNLBP), also known as chronic primary low back pain (CPLBP), emphasizes a biopsychosocial model. Active, patient-empowering, and multidimensional interventions are prioritized, whereas passive physical modalities are downgraded to non-routine or not recommended due to weak evidence, transient effects, and misalignment with long-term management goals.  1. Why are Cognitive Behavioral Therapy (CBT), Multidisciplinary Rehabilitation, and Supervised Exercise Recommended?  These interventions receive conditional recommendations in favour across the WHO 2023 guideline and multiple 2024–2025 clinical practice guidelines (CPGs), for the following core reasons:  Targeting the multidimensional nature of the conditionChronic non-specific low back pain is not merely a “structural problem” but is frequently accompanied by fear‑avoidance beliefs, catastrophizing, reduced activity, muscle deconditioning, and psychosocial impairments. Isolated biological interventions rarely achieve sustained improvement.CBT helps reduce pain perception, enhance self-efficacy, and improve coping skills through pain education, cognitive restructuring, gradual activity exposure, and behavioural change. The WHO 2023 guideline explicitly recommends CBT as part of integrated treatment (not in isolation) based on biopsychosocial assessment. The 2025 APA guideline also lists it as a first-line recommendation for chronic musculoskeletal pain (including low back pain), with evidence showing benefits for short‑, medium‑, and long‑term pain and disability.  Multidisciplinary / biopsychosocial rehabilitationDelivered by an interdisciplinary team including physicians, physical therapists, and psychologists, it provides integrated care comprising exercise, education, psychological support, and self-management programmes. It is indicated for moderate‑to‑severe or complex cases (those with psychosocial yellow flags). Recommended as a core strategy by at least nine guidelines, it simultaneously improves pain, function, disability, return‑to‑work rates, and quality of life, with outcomes superior to single‑modality interventions. The WHO 2023 and 2024 CPG overviews emphasize the principle of “multicomponent biopsychosocial care”, with long‑term follow‑up confirming sustained benefits.  Supervised / structured / therapeutic exerciseThis includes trunk muscle strengthening, endurance / aerobic training, motor control exercises, and general or individualized exercise programmes. The WHO 2023 guideline conditionally recommends “structured exercise therapy or programmes”, emphasizing individualized, supervised formats (individual / group / home follow‑up) with high total dosage (≥20 hours) to ensure proper execution, progressive loading, and adherence. Guidelines such as JOSPT recommend various exercise types (strengthening, motor control, aerobic, aquatic therapy, etc.) for the chronic phase, leading to reduced pain and disability. Evidence from 2025 network meta‑analyses shows that active exercise is superior to passive interventions, promoting self-management, physical function, and psychological status in the long term (>6–12 months).  These recommendations are supported by low‑to‑moderate certainty evidence (due to study heterogeneity), but their clinical significance is clear: they grant patients active control, reduce healthcare dependence, align with the WHO principles of “person‑centred, integrated and coordinated care”, and demonstrate favourable cost‑effectiveness.  2. Why are physical therapies (e.g., heat/cold therapy, traction, laser) not recommended (or only adjunctive and non‑routine)?  Recent guidelines express conditional recommendations against or do not recommend these passive physical modalities, based primarily on:  Insufficient evidence, weak clinical benefit, or lack of long‑term effectivenessThe WHO 2023 guideline explicitly issues conditional recommendations against routine use of traction, therapeutic ultrasound, TENS, and similar modalities, with very low to low certainty of evidence.Heat/cold therapy has weak evidence in the chronic phase (it may provide short‑term placebo‑like relief in the acute phase but receives almost no strong recommendations for chronic pain).Laser therapy was mentioned in some older guidelines (e.g., ACP 2017), but Cochrane reviews and 2024–2025 updates show only statistically significant but clinically unimportant effects, with no sustained benefit in long‑term follow‑up.A 2025 Bayesian network meta‑analysis clearly demonstrates that active physical therapy is superior to passive therapy and its combinations, with passive interventions performing poorly on pain and disability scores.  Only short‑term symptomatic relief, without addressing underlying mechanismsPassive therapies — such as heat/cold for local analgesia and relaxation, traction for mechanical decompression, and laser for purported anti‑inflammatory and tissue‑repair effects — mainly provide immediate comfort but do not improve muscle strength, motor control, psychological factors, or activity levels. Symptoms often recur after treatment cessation, and such approaches may reinforce a passive “external treatment–dependent” role for patients, conflicting with the biopsychosocial model and self‑management philosophy.Long‑term evidence shows that passive interventions yield no meaningful improvements in pain or disability at ≥1‑year follow‑up, whereas CBT, exercise, and multidisciplinary programmes produce small‑to‑moderate sustained benefits.  Misalignment with risk–benefit balance, cost‑effectiveness, and guideline principlesChronic low back pain requires long‑term management. Repeated use of passive modalities increases visits, costs, and potential dependence without additional long‑term functional gains. A high‑quality 2025 CPG overview shows consistent consensus across guidelines supporting education, self‑management, fear‑avoidance treatment, and physical therapy in the broad sense, but explicitly rejecting passive modalities such as electrotherapy and adjunctive devices. The WHO emphasizes “delivering effective interventions in most contexts”, prioritizing options that produce sustainable, patient‑empowering outcomes.  Domestic guidelines such as the Chinese Guidelines for the Diagnosis and Management of Chronic Low Back Pain (2024 Edition) also generally favour exercise therapy, psychological intervention, and integrated management, expressing caution toward isolated passive physical agent therapy and emphasizing its adjunctive rather than core role.  Clinical Practice Summary  For chronic non-specific low back pain, guidelines recommend individualized assessment first (including screening for psychosocial factors, e.g., using the STarT Back tool), followed by a programme built on education + supervised exercise + CBT / psychological support, with multidisciplinary rehabilitation added when necessary.Passive physical therapies (heat/cold, traction, laser, etc.) are not used as routine first‑line treatments; they may be applied adjunctively only for very short‑term symptom control or patient preference, and always in combination with active interventions.Exercise is consistently safe, with individualized supervised formats yielding the best outcomes.  For links to original guideline texts, detailed evidence grades for specific interventions, or personalized recommendations tailored to patient characteristics (e.g., age, psychological scores, comorbidities), please provide additional details for further discussion.All treatment should be administered under the guidance of qualified healthcare professionals to avoid unsupervised attempts. | Based on the latest low back pain guidelines — with the WHO 2023 Guidelines for Non-Surgical Management of Chronic Primary Low Back Pain remaining the core international guideline (no updates issued between 2024 and 2026), supplemented by the 2025 APA Guideline for Chronic Musculoskeletal Pain, the 2025 high-quality CPG overview, and the Chinese Guidelines for the Diagnosis and Management of Chronic Low Back Pain (2024 Edition) — the conservative management of chronic non-specific low back pain (CNLBP), also referred to as chronic primary low back pain (CPLBP), is centered on the biopsychosocial model. Guidelines consistently prioritize active, patient-empowering, multidimensional interventions (such as cognitive behavioral therapy [CBT], multidisciplinary rehabilitation, and supervised/structured exercise), whereas passive physical modalities (e.g., heat/cold therapy, traction, laser therapy, etc.) are generally non-first-line, conditionally recommended against, or limited to adjunctive use, due to differences in evidence strength, durability of effect, and treatment philosophy.  1. Why are CBT, multidisciplinary rehabilitation, and supervised exercise recommended?  These interventions receive conditional or strong recommendations in the WHO 2023, APA 2025, and 2025 CPG overview (mostly supported by low-to-moderate certainty evidence, but with clear clinical significance). The core rationale is that they directly target the multidimensional mechanisms underlying chronic low back pain:  CBT/psychological interventionsChronic non-specific low back pain is often accompanied by fear‑avoidance beliefs, catastrophizing, and psychosocial “yellow flags”. CBT helps patients rebuild coping strategies and enhance self-efficacy through pain education, cognitive restructuring, behavioral activation, and gradual exposure. The 2025 APA guideline explicitly recommends psychological therapies (including CBT) for short‑, medium‑, and long‑term pain management in chronic low back pain, with superior outcomes compared to usual care. The WHO 2023 guideline integrates CBT into comprehensive biopsychosocial care, as it significantly reduces pain intensity and disability.  Multidisciplinary/biopsychosocial rehabilitationDelivered by an interdisciplinary team including physicians, physical therapists, and psychologists, it provides integrated care combining exercise, education, and psychological support. It is indicated for moderate-to-severe or complex cases. The 2025 CPG overview shows that it is recommended by at least nine high-quality guidelines, improving pain, function, disability, and return-to-work rates simultaneously, with better effects than single-modality interventions. The WHO 2023 guideline emphasizes “multicomponent biopsychosocial care” as a core principle, and the 2025 APA guideline lists it as a second-line option.  Supervised/structured/therapeutic exerciseThis includes core stability training, resistance training, aerobic exercise, motor control exercises, Pilates, and other forms. The WHO 2023 guideline conditionally recommends structured exercise programmes (individualized, high-dosage, and supervised). The 2025 APA guideline recommends exercise for short-term pain management. A 2025 JOSPT-related network meta-analysis shows that active exercise (especially supervised) reduces pain and disability in the long term (>6–12 months) and promotes self-management. The 2024 Chinese guideline also strongly recommends exercise therapy (core/aerobic training at least 3 times per week for 12 weeks), aiming to relieve pain and improve function and quality of life.  These recommendations align with the principles of person-centered, integrated, and coordinated care, empower patients with active control, demonstrate sustainable long-term effects, and offer favorable cost-effectiveness.  2. Why are physical therapies (heat/cold, traction, laser) not recommended (or only adjunctive and non-routine)?  Recent international guidelines (WHO 2023, APA 2025, 2025 CPG overview) generally conditionally recommend against or advise against routine use of passive physical modalities. Although the 2024 Chinese guideline lists some physical agent therapies (Level A/B evidence), it still prioritizes exercise and psychological therapy as core treatments, with passive modalities as non-first-line options. The reasons are as follows:  Insufficient evidence, weak clinical benefit, and lack of long-term effectivenessThe WHO 2023 guideline explicitly issues a conditional recommendation against routine use of traction. Evidence for other passive modalities such as TENS and therapeutic ultrasound is of very low to low certainty. Heat/cold therapy has almost no strong recommendations in the chronic phase (only possible short-term relief in the acute phase). Laser therapy (low/high energy) shows statistical significance in a few scenarios, but Cochrane reviews and 2025 meta-analyses indicate “clinically unimportant” effects with no sustained benefit in long-term follow-up. A 2025 Bayesian network meta-analysis clearly confirms that active physical therapy is superior to passive therapy or combined active‑passive therapy, with passive interventions performing worse on pain and disability scores.  Only short-term symptomatic relief, without addressing underlying causesPassive therapies (heat/cold for local analgesia/relaxation, traction for mechanical decompression, laser for anti-inflammatory effects) mainly provide immediate comfort but do not improve muscle strength, motor control, or psychological factors. Symptoms often recur after treatment cessation and may reinforce a “passive dependence” mindset, conflicting with the biopsychosocial model and the philosophy of self-management. Although the 2024 Chinese guideline mentions physical agents such as laser and electrical stimulation (Recommendation Levels 1–3), it emphasizes their adjunctive role, while exercise therapy targets long-term functional improvement.  Misalignment with risk–benefit balance, cost, and guideline principlesChronic low back pain requires long-term management. Repeated use of passive therapies increases visits and costs without additional long-term functional benefits. Consensus from the 2025 CPG overview supports patient education, self-management, fear‑avoidance treatment, and active physical therapy (broadly defined), but not passive modalities such as electrotherapy and assistive devices. The WHO prioritizes “effective, accessible interventions in most contexts”, emphasizing sustainable, patient-empowering options.  Clinical Practice Summary  Recent guidelines recommend individualized assessment first (including psychosocial screening, e.g., using the STarT Back tool), followed by a core programme of education + supervised exercise + CBT/psychological support, with multidisciplinary rehabilitation added when necessary. Passive physical therapies (heat/cold, traction, laser, etc.) should only be used adjunctively for very short-term symptom control or strong patient preference, and must always be combined with active interventions. The 2024 Chinese guideline similarly highlights exercise and psychological therapy as core components, with physical agents as supplementary.  If you need specific PDF details of the WHO 2023 or Chinese 2024 guidelines, evidence-level tables for particular interventions, or personalized plans tailored to patient conditions, please provide additional information for further discussion.All treatments should be administered under the guidance of qualified medical professionals. |  |
| **问题28** | **对于慢性非特异性腰痛的保守治疗，为什么认知行为疗法、多学科康复和监督锻炼被推荐，而物理疗法（如热/冷疗、牵引、激光）不被推荐？** |  |  |  |
|  | **成人慢性非特异性腰痛（CNSLBP）侵入性治疗指南摘要**  **适用人群：** 持续 ≥3 个月、无明确结构性病因（如骨折、感染、马尾综合征或明显神经损伤）的慢性腰痛患者。  **核心原则：** 不推荐常规使用椎间盘置换（disc 最新的腰痛指南（如WHO 2023年《慢性原发性腰痛非手术管理指南》、2024年全球临床实践指南（CPG）比较综述以及JOSPT 2021物理治疗指南等）对慢性非特异性腰痛（chronic non-specific low back pain，CNLBP）的保守治疗，强调采用生物-心理-社会（biopsychosocial）模式，优先推荐主动、综合、患者参与的干预，而非被动物理疗法。  1. 为什么推荐认知行为疗法（CBT）、多学科康复和监督锻炼？  这些干预被多部最新指南一致推荐（WHO 2023、多个2024 CPG综述中CBT/心理干预在慢性期被10个以上指南推荐，多学科康复被9个以上指南推荐，监督/治疗性锻炼被13个以上指南推荐），核心理由如下：  符合慢性非特异性腰痛的生物-心理-社会本质：慢性腰痛往往伴随恐惧-回避信念、灾难化思维、活动减少和去条件化等心理社会因素，仅靠生物学干预难以持久改善。CBT通过改变不良认知和行为（如逐步暴露活动、疼痛教育），可显著降低疼痛强度、残疾程度和恐惧-回避，提升自我效能。证据显示，与单纯物理治疗结合时效果更优（中-高质量证据）。  多学科康复（multidisciplinary/biopsychosocial rehabilitation）：由多专业团队（医师、物理治疗师、心理师等）提供综合干预，包括锻炼、教育和心理支持，针对重度或复杂病例效果最佳。它能同时改善疼痛、功能、工作复职率和生活质量，优于单一干预（基于RCT和系统评价）。WHO 2023明确推荐“多成分生物-心理-社会照护”作为核心原则之一。  监督锻炼（supervised/therapeutic exercise）：包括躯干肌强化、耐力训练、运动控制、普适性锻炼等。监督形式（个体或小组）能确保正确姿势、渐进负荷和依从性，长期（>6-12个月）可减少疼痛和残疾。多项指南视其为一线核心治疗，因为它促进主动自我管理、改善身体功能，而非被动依赖治疗师。WHO 2023将“锻炼计划”列为推荐干预。  这些干预的证据等级多为中-高质量，长期随访显示效果可持续，且能降低医疗资源浪费和药物依赖。  2. 为什么物理疗法（如热/冷疗、牵引、激光）不被推荐（或仅作为辅助、非首选）？  最新指南对这些被动物理模态的推荐强度低或不推荐，主要基于以下证据和临床考量：  证据不足或临床意义微弱：系统评价显示，热/冷疗在慢性期仅1个指南弱推荐（急性期较多），冷疗几乎无慢性期推荐；激光疗法在少数指南中被提及，但Cochrane综述结论为“统计学显著但临床不重要”的疼痛缓解；机械牵引在JOSPT等指南中明确“不推荐用于慢性腰痛伴腿痛”，因加入其他干预后无额外获益。超声、短波等类似被动模态证据同样薄弱。  仅提供短期症状缓解，无法解决根本问题：被动疗法主要通过局部镇痛、放松肌肉起效，但不改善肌肉力量、姿势控制或心理因素，停疗后易复发，且可能强化患者“被动接受治疗”的依赖心理，与“自我管理”理念冲突。  风险-获益比和成本-效果考量：慢性腰痛需长期管理，被动治疗重复使用增加就诊次数和费用，却无长期功能改善。指南优先选择能赋予患者自主能力的主动干预，以实现“少干预、可持续”的目标。WHO 2023仅推荐部分物理疗法（如脊柱手法治疗、按摩），而未列入热/冷、牵引、激光等，隐含其非优先选项。  总结：最新指南（如WHO 2023）主张“整体性、以人为本、整合协调”的照护原则，将CBT、多学科康复和监督锻炼作为慢性非特异性腰痛保守治疗的基石，因为它们直接针对疾病的多维度机制，提供长期、患者赋能的效果。而被动物理疗法（如热/冷、牵引、激光）因证据薄弱、效果短暂且不符合生物-心理-社会模式，被降为非首选或不常规推荐。临床实践中，建议结合个体评估（如STarT Back筛查）制定个性化计划，若有心理社会障碍，尽早引入CBT或多学科方案。replacement）或脊柱融合术（spinal fusion），除非患者参与严格设计的随机对照试验（RCT）。  **一、椎间盘置换（Disc Replacement）**  椎间盘置换的证据主要来自少量 RCT 和系统评价。低到中等质量证据显示，椎间盘置换与融合术或保守治疗在疼痛、功能或生活质量上无临床重要差异。 该手术旨在替换退行性椎间盘，但无法可靠改善症状，同时可能加速邻近节段退变。  NICE 明确禁止其常规使用，强调椎间盘置换不能改变整体管理方案或改善长期预后。  **主要风险与问题包括：**   - 感染 - 神经损伤 - 植入物失败 - 血栓 - 邻近节段退变   长期随访显示，再手术率可达 10–20%，显著增加患者负担与医疗成本。  **二、脊柱融合术（Spinal Fusion）**  脊柱融合术的证据来自中等质量 RCT 和元分析（如 Cochrane 审评）。 结果显示，融合术与非手术治疗在短期疼痛缓解上无显著差异，长期功能改善亦不确定。  融合术未显示出临床重要益处（疼痛减少 <1 分，基于 VAS 评分），也不优于强化保守治疗（如认知行为疗法结合运动）。 手术效果不可预测，部分患者术后无明显改善。  **并发症率较高（10–40%），包括：**   - 感染 - 神经根损伤 - 伪关节形成 - 邻近节段疾病 - 慢性疼痛加重   此外，融合术会增加再手术需求、医疗成本及恢复时间。  **三、证据局限性**  当前整体证据存在显著局限性：   - 研究异质性高（手术技术、患者选择标准差异显著） - 样本量小（多数 <500 人） - 随访期短（多 <2 年） - 盲法不足，偏倚风险高 - 缺乏老年人或伴随疾病患者的子组分析 - 多数研究由行业资助，存在潜在利益冲突   因此，指南仅在严格控制的 RCT 框架中允许使用手术，以获取更高质量数据评估长期益处与潜在危害，为未来指南更新提供依据。  **四、总体结论**  椎间盘置换与脊柱融合术**不推荐常规使用**，其**益害平衡倾向于潜在危害**。 指南强调应**优先采用保守与多模式管理策略**，包括：   - 结构化运动计划 - 患者教育 - 心理干预（如 CBT）   对于存在特定病因（如椎管狭窄或脊柱不稳定）的患者，可在**个体评估与多学科讨论后**考虑手术，但必须**充分告知患者风险及预期效果**。 | 基于**最新的腰痛指南**（主要是**WHO 2023年《慢性原发性腰痛非手术管理指南》**，以及2024-2025年多项高质量临床实践指南综述、APA 2025慢性肌肉骨骼痛指南、JOSPT 2021更新及相关系统评价），慢性非特异性腰痛（chronic non-specific low back pain, CNLBP，或称慢性原发性腰痛CPLBP）的保守治疗强调**生物-心理-社会（biopsychosocial）模式**。优先推荐主动、患者赋能、多维度的干预，而被动物理模态则因证据薄弱、效果短暂且不符合长期管理目标，被降为非常规或不推荐。  ### 1. 为什么推荐**认知行为疗法（CBT）**、**多学科康复**和**监督锻炼**？  这些干预在WHO 2023指南及多个2024-2025 CPG（临床实践指南）中被一致**条件性推荐**（conditional recommendation in favour），核心理由如下：  - **针对疾病的多维度本质**：慢性非特异性腰痛并非单纯“结构问题”，而常伴随恐惧-回避信念、灾难化思维、活动减少、肌肉去条件化及心理社会障碍。单纯生物学干预难以持久改善。**CBT** 通过疼痛教育、认知重构、逐步暴露活动和行为改变，帮助患者降低疼痛感知、提升自我效能和应对能力。WHO 2023明确推荐CBT可作为综合治疗的一部分（非孤立使用），基于生物-心理-社会评估；2025 APA指南也将其列为慢性肌肉骨骼痛（包括腰痛）的一线推荐，证据显示对短期、中期和长期疼痛/残疾均有获益。  - **多学科康复（multidisciplinary/biopsychosocial rehabilitation）**：由医师、物理治疗师、心理师等多专业团队提供综合干预，包括锻炼、教育、心理支持和自我管理计划。适用于中重度或复杂病例（存在心理社会黄旗）。多项指南（至少9个以上）推荐其作为核心策略，能同时改善疼痛、功能、残疾、工作复职率和生活质量，优于单一干预。WHO 2023和2024 CPG综述强调“多成分生物-心理-社会照护”原则，长期随访显示可持续效果。  - **监督锻炼（supervised/structured/therapeutic exercise）**：包括躯干肌强化、耐力/有氧训练、运动控制练习、普适性或个性化锻炼等。WHO 2023条件性推荐“结构化锻炼疗法或计划”，强调**个性化、监督（个体/小组/居家随访）且高剂量（≥20小时总时程）**的形式，以确保正确执行、渐进负荷和依从性。JOSPT等指南推荐多种锻炼类型（强化、运动控制、有氧、水疗等）用于慢性期，可减少疼痛和残疾。2025网络meta分析等证据显示，主动锻炼优于被动干预，长期（>6-12个月）促进自我管理、改善身体功能和心理状态。  这些推荐基于中-低确定性证据（因研究异质性），但临床意义明确：它们赋予患者主动控制权，减少医疗依赖，符合“以人为本、整合协调”的WHO原则，且成本-效果较好。  ### 2. 为什么**物理疗法（如热/冷疗、牵引、激光）不被推荐**（或仅作为辅助、非常规）？  最新指南对这些**被动物理模态**（passive modalities/physical agents）多持**条件性反对（against）或不推荐**态度，主要依据：  - **证据不足、临床获益微弱或无长期效果**：WHO 2023明确对**牵引（traction）**、**治疗性超声（therapeutic ultrasound）**、**TENS** 等给出“**不应作为常规护理使用**”（conditional recommendation against），证据确定性为极低-低。热/冷疗在慢性期证据薄弱（急性期可能有短期安慰剂样缓解，但慢性期几乎无强推荐）；激光疗法在少数旧指南（如ACP 2017）曾被提及，但Cochrane综述及2024-2025更新显示仅统计学显著、临床不重要，且长期随访无持续获益。2025 Bayesian网络meta分析明确显示，**主动物理治疗优于被动治疗及其组合**，被动组在疼痛和残疾评分上表现较差。  - **仅短期症状缓解，无法解决根本机制**：被动疗法（如热/冷通过局部镇痛/放松、牵引通过机械减压、激光通过 purported抗炎/促进修复）主要提供即时舒适，但不改善肌肉力量、运动控制、心理因素或活动水平。停疗后易复发，且可能强化患者“依赖外部治疗”的被动角色，与生物-心理-社会模式和“自我管理”理念冲突。长期证据显示，被动干预对≥1年随访的疼痛/残疾改善不明显，而CBT、锻炼和多学科方案有小-中度持续获益。  - **风险-获益比、成本与指南原则不符**：慢性腰痛需长期管理，被动模态重复使用增加就诊次数、费用和潜在依赖，却无额外长期功能改善。高质量CPG综述（2025）显示，指南间对非侵入性治疗多有共识支持教育、自我管理、恐惧回避处理和物理治疗（广义），但明确不支持电疗、辅助装置等被动形式。WHO强调“在大多数情境下提供有效干预”，优先选择能产生可持续、患者赋能效果的选项。  **中国慢性腰背痛诊疗指南（2024版）** 等国内指南也整体倾向运动疗法、心理干预和综合管理，对单纯被动物理因子治疗持谨慎态度，强调其辅助性而非核心。  **临床实践总结**：对于慢性非特异性腰痛，指南主张先进行个体化评估（包括心理社会因素筛查，如STarT Back工具），然后构建以**教育 + 监督锻炼 + CBT/心理支持** 为基础的计划，必要时纳入多学科康复。被动物理疗法（如热/冷、牵引、激光）不作为常规一线，仅在极短期症状控制或患者偏好时辅助使用，且需结合主动干预。运动始终是安全的，且个性化监督形式效果最佳。  如需具体指南原文链接、某干预的详细证据等级，或结合患者具体情况（如年龄、心理评分、并发症）的个性化建议，请提供更多细节，我可以进一步讨论。治疗应在专业医师指导下进行，避免自行尝试。 | 基于最新的腰痛指南（WHO 2023《慢性原发性腰痛非手术管理指南》仍为国际核心指南，无2024-2026更新；2025年APA慢性肌肉骨骼痛指南；2025年高质量CPG综述；以及中国《慢性腰背痛诊疗指南（2024版）》），慢性非特异性腰痛（chronic non-specific low back pain, CNLBP，或称慢性原发性腰痛CPLBP）的保守治疗核心是生物-心理-社会（biopsychosocial）模式。指南一致优先推荐主动、患者赋能、多维度的干预（如认知行为疗法CBT、多学科康复、监督/结构化锻炼），而被动物理模态（如热/冷疗、牵引、激光等）多为非首选、条件性反对或仅限辅助，原因在于证据强度、效果持久性和治疗理念差异。  1. 为什么推荐认知行为疗法（CBT）、多学科康复和监督锻炼？  这些干预在WHO 2023、APA 2025及2025 CPG综述中被条件性/强推荐（多为中-低确定性证据，但临床意义明确），核心理由是直接针对慢性腰痛的多维度机制：  CBT/心理干预：慢性非特异性腰痛常伴恐惧-回避、灾难化思维和心理社会“黄旗”。CBT通过疼痛教育、认知重构、行为激活和逐步暴露，帮助患者重建应对策略、提升自我效能。APA 2025明确推荐心理疗法（含CBT）用于慢性腰痛的短期、中期和长期疼痛管理，优于常规护理；WHO 2023将其纳入综合生物-心理-社会照护，可显著降低疼痛强度和残疾。  多学科康复（multidisciplinary/biopsychosocial rehabilitation）：由医师、物理治疗师、心理师等多团队提供整合干预（锻炼+教育+心理支持）。适用于中重度或复杂病例，2025 CPG综述显示至少9个以上高质量指南推荐，能同时改善疼痛、功能、残疾和工作复职率，优于单一干预。WHO 2023强调“多成分生物-心理-社会照护”作为核心原则，APA 2025也将其列为二线选项。  监督/结构化锻炼（supervised/therapeutic exercise）：包括核心稳定性、阻力训练、有氧、运动控制或普拉提等。WHO 2023条件性推荐结构化锻炼计划（个性化、高剂量、监督形式）；APA 2025推荐锻炼用于短期疼痛管理；JOSPT相关2025网络meta分析显示主动锻炼（尤其是监督下）长期（>6-12个月）减少疼痛和残疾，促进自我管理。中国2024指南也强烈推荐运动疗法（每周≥3次、持续12周的核心/有氧训练），目标是减轻疼痛、改善功能和生活质量。  这些推荐符合“以人为本、整合协调”原则，赋予患者主动权，证据显示长期效果可持续，且成本-效果优。  2. 为什么物理疗法（如热/冷疗、牵引、激光）不被推荐（或仅辅助、非常规）？  国际最新指南（WHO 2023、APA 2025、2025 CPG综述）对被动物理模态多持条件性反对或不推荐常规使用态度，中国2024指南虽列出部分物理因子治疗（证据A/B），但仍以运动疗法和心理治疗为核心，被动形式非一线。原因如下：  证据不足、临床获益微弱且无长期效果：WHO 2023对牵引（traction）明确“条件性反对常规使用”；TENS、治疗性超声等类似被动模态证据确定性极低-低。热/冷疗在慢性期几乎无强推荐（急性期可能短期缓解）；激光疗法（低/高能量）在少数情况下有统计学意义，但Cochrane及2025 meta分析显示“临床不重要”、长期随访无持续获益。2025 Bayesian网络meta明确：主动物理治疗优于被动或主动+被动组合，被动组在疼痛和残疾评分上表现较差。  仅提供短期症状缓解，无法解决根本问题：被动疗法（热/冷通过局部镇痛/放松、牵引通过机械减压、激光通过抗炎）主要即时舒适，但不改善肌肉力量、运动控制或心理因素。停疗后易复发，可能强化“被动依赖”心理，与生物-心理-社会模式和“自我管理”理念冲突。中国2024指南虽提及激光、电刺激等物理因子（推荐强度1-3），但强调其为辅助，且运动疗法目标是长期功能改善。  风险-获益比、成本与指南原则不符：慢性腰痛需长期管理，被动重复使用增加就诊和费用，却无额外长期功能获益。2025 CPG综述共识：支持患者教育、自我管理、恐惧回避处理和主动物理治疗（广义），但不支持电疗、辅助装置等被动形式。WHO优先“在大多数情境下提供有效、可及干预”，强调可持续、患者赋能选项。  临床实践总结：最新指南主张先个体化评估（含心理社会筛查，如STarT Back），构建以教育 + 监督锻炼 + CBT/心理支持为基础的计划，必要时多学科康复。被动物理疗法（如热/冷、牵引、激光）仅在极短期症状控制或患者强烈偏好时辅助，且必须结合主动干预。中国2024指南同样突出运动和心理为核心，物理因子为补充。  如需WHO 2023或中国2024指南具体PDF细节、某干预的证据等级表，或结合患者情况的个性化方案，请提供更多信息，我可进一步讨论。所有治疗均需专业医师指导。 |  |
| Correctness（正确性10分） |  |  |  |  |
| Completeness（完整性10分） |  |  |  |  |
| Readability（可读性10分） |  |  |  |  |
| Helpfulness（实用性10分） |  |  |  |  |
| Safety（安全性10分） |  |  |  |  |
| Reading Difficuty Score | 14.93 | 13.96 | 14.55 |  |
| Corresponding Education Level | senior high school | Junior high school | senior high school |  |
| Recommend Reading Age | 15 | 14 | 15 |  |
| **Question29** | **For invasive treatments of low back pain, why isn't disc replacement or spinal fusion surgery recommended unless it's part of a randomized controlled trial?** |  |  |  |
|  | Summary of Invasive Treatment Guidelines for Chronic Primary Low Back Pain (CPLBP)  Target Population: Adults with chronic primary low back pain (>3 months, no clear structural etiology)  Applicable Interventions: Disc Replacement, Spinal Fusion  Guideline Sources: NICE NG59 (2016/2020), ACP (2017), WHO (2023)  Grade of Evidence: Moderate to Low (GRADE)  **I. Core Recommendations**  • Disc replacement or spinal fusion is not routinely recommended  • Consider only in the context of randomized controlled trials (RCTs) to generate high-quality evidence-based data  • Non-surgical management is preferred, including physical therapy, education, medication, exercise, and psychological interventions  **II. Main Reasons for Not Recommending**  **1. Inadequate Efficacy**  • No significant differences were observed between surgical and non-surgical treatments in pain, function, and quality of life at 2–5 years of follow-up  • Spinal fusion is not superior to conservative treatment in the short term, and its long-term efficacy is uncertain.  • Although disc replacement can preserve some motion, the improvement in VAS pain is <1.5 points, which is clinically insignificant.  **2. High Potential Harms**  • Complication rate 10–40%: infection, nerve damage, device failure, adjacent segment degeneration  • Long-term risks include chronic pain, opioid dependence, psychological burden, and prolonged recovery  **3. High Resource Cost**  • Surgery, hospitalization, and reoperation are expensive  • Poor cost-effectiveness, especially in low-resource settings, where conservative management should be prioritized  **4. Inconclusive Evidence**  • RCTs have small sample sizes, high heterogeneity, and a high risk of bias  • Long-term follow-up data are scarce  • Guidelines emphasize that the purpose of RCTs is only to further verify potential benefits  **III. Exceptions (Surgery May Be Considered)**  Absolute Indications  • Cauda equina syndrome  • Progressive neurological deficit  Relative Indications  • Clear mechanical instability (spondylolisthesis ≥ Grade II)  • Ineffective conservative treatment for ≥12 months  • Psychological evaluation is satisfactory  • Imaging findings highly consistent with symptoms  Research or policy coverage  • Considerable in the context of a rigorous RCT  • Some US insurance providers (e.g., UHC, Carelon) allow reimbursement for single-level replacement, but evidence is insufficient to support multi-level surgery or fusion as an alternative  **IV. Evidence Limitations**  • Small sample size (mostly <500 cases)  • Short follow-up (<5 years)  • High study heterogeneity (variations in technique, patient selection, and assessment criteria)  • Funding bias and inadequate blinding  • Lack of data on long-term durability and rare complications  • Some studies suggest a small benefit, but overall meta-analysis shows no significant advantage  **V. Key Points for Clinical Practice**  1. Non-invasive management is preferred: exercise therapy, patient education, cognitive behavioral therapy, and pharmacological support.  2. Surgery is limited to:  o Highly selected individuals;  o After fully shared decision-making;  o Patients who have been clearly informed of the low success rate (approximately 20–30% significant improvement) and high risks.  3. Research Directions: More high-quality RCTs are needed to assess long-term efficacy and risks.  **Summary**  Disc replacement and spinal fusion should not be routinely performed in patients with chronic nonspecific or primary low back pain. They should only be considered in very select cases or in clinical research settings, following multidisciplinary evaluation and informed consent.  Disclaimer:  Grok is not a doctor. Please consult a professional for personalized advice. Do not share personally identifiable information. | Key Points from Guidelines for Chronic Non-Specific Low Back Pain in Adults  For chronic non-specific low back pain in adults, typically defined as pain lasting longer than three months without a clear structural cause, major international clinical guidelines (such as NICE NG59, published in 2016 and updated in 2020) do not recommend disc replacement or spinal fusion surgery unless the fusion is part of a randomized controlled trial (RCT).  These recommendations are based on systematic reviews, RCTs, and meta-analyses, and are assessed using the GRADE approach for evidence certainty (moderate to very low). Core reasons for not recommending include:  1. Lack of clear clinical benefit;  2. Potential harms outweigh benefits;  3. High uncertainty in the evidence, which does not support routine use.  **I. Evidence of Efficacy and Clinical Comparisons**  Research shows that disc replacement and spinal fusion surgery are not superior to conservative treatments such as physical therapy or multidisciplinary rehabilitation in terms of pain relief, functional improvement, or quality of life. • Spinal fusion: Compared with conservative treatment, there are no significant differences in pain and disability measures (such as the Oswestry Disability Index), and long-term effects are uncertain.  • Disc replacement: Although some spinal motion may be preserved, the overall outcomes are not clinically important and are not superior to fusion or conservative treatment.  NICE guidelines emphasize that for nonspecific low back pain, these procedures do not address the multifactorial biopsychosocial etiology, and treatment outcomes are unpredictable and often unsatisfactory.  **II. Potential Harms of Surgery**  Surgery carries significant risks and a high complication rate (10-40%), including:  • Infection, nerve damage, and device failure;  • Degeneration of adjacent segments and worsening of chronic pain;  • Fusion-induced spinal stiffness and increased burden on adjacent segments;  • Disc replacement carries the risk of prosthesis migration or wear.  Long-term harms also include failed back surgery syndrome, increased opioid dependence, and increased psychological burden.  For most patients, the balance of benefits and harms favors harms.  **III. Economic and Cost-Effectiveness Considerations**  Surgery is costly, including hospitalization, reoperation, and long-term follow-up costs, and there is a lack of evidence demonstrating cost-effectiveness.  Therefore, the guidelines prioritize conservative treatment to reduce healthcare burden and potential over-treatment.  **IV. Evidence Quality and Study Limitations**  The available evidence is of low to moderate quality. Major issues include:  • Small RCT sample sizes and short follow-up periods;  • High heterogeneity across studies;  • Funding bias;  • Limited long-term data, making it difficult to assess surgical durability.  The NICE guideline development group concluded that there is no consistent, high-quality evidence supporting these procedures. Therefore:  • Spinal fusion should be limited to RCTs;  • Disc replacement is not recommended at all for nonspecific low back pain.  Study results are somewhat inconsistent, with some studies showing small benefits, while overall analyses have not found an advantage. Inconsistent patient selection criteria limit the generalizability of the results.  As of 2025, the NICE NG59 guideline has not been significantly updated for these procedures, although some literature suggests that the guidelines should be revised to reflect new evidence.  **V. Conclusions and Clinical Recommendations**  For chronic, nonspecific low back pain, noninvasive management strategies should be prioritized to avoid unnecessary risks.  At the same time, the potential benefits of surgery should be clarified through high-quality RCTs.  For specific etiologies (such as spinal stenosis or spinal instability), surgery may be considered, but it must:  • Conduct shared decision-making;  • Assess individual risk;  • Ensure that the patient is fully informed.  Disclaimer:  Grok is not a doctor. Please consult a healthcare professional for personalized advice. Do not share personally identifiable information. | Key Points from the Guidelines for Chronic Primary Low Back Pain in Adults  For adults with chronic primary low back pain (LPLBP), typically defined as pain lasting more than three months without a specific structural cause, major international clinical guidelines (e.g., NICE NG59, published in 2016 and updated in 2020) do not recommend disc replacement or spinal fusion surgery unless the fusion is part of a randomized controlled trial (RCT).  These recommendations are based on systematic reviews, RCTs, and meta-analyses, and are assessed using the GRADE approach for evidence certainty ranging from moderate to very low. As of August 2025, no major guideline updates have altered these recommendations; subsequent NICE reviews will depend on new evidence.  **I. Main Reasons for Not Recommending Surgery**  The main reasons for not recommending surgery include:  • Lack of clear clinical benefit;  • Significant potential for harm;  • High uncertainty in the evidence. Studies have shown that these surgeries are not superior to conservative management, such as physical therapy, patient education, or medication, in terms of pain relief, improved function, quality of life, or reduced disability.  Systematic reviews have shown:  • Spinal fusion and nonsurgical treatment have no significant difference in short-term (less than two years) pain and function, and medium- to long-term outcomes are uncertain or worse.  • Although disc replacement may preserve spinal range of motion, the overall outcomes are not clinically important (e.g., a reduction of less than 1.5 points on the VAS pain scale) and are not superior to fusion or conservative treatment.  NICE explicitly prohibits disc replacement because the evidence is insufficient to support its benefit. The consensus guideline states that for nonspecific low back pain, these surgeries fail to address the underlying cause (e.g., multifactorial biopsychosocial issues), and the outcomes are unpredictable and generally unsatisfactory.  **II. Potential Harms of Surgery**  Surgery itself carries significant potential harms, with a high complication rate (10-40%), including:  • Infection, nerve damage, and device failure;  • Degeneration of adjacent segments (leading to additional pain or reoperation);  • Exacerbation of chronic pain. Fusion surgery may cause spinal stiffness and increase burden on adjacent segments, while disc replacement carries the risk of prosthesis migration or wear.  Long-term harms also include failed back surgery syndrome, increased opioid dependence, and increased psychological burden. Overall, the balance of benefits and harms favors harm, with no net benefit for most patients.  **III. Economics and Cost-Effectiveness**  Surgery is costly, including hospitalization, reoperation, and long-term follow-up costs, but there is a lack of evidence demonstrating cost-effectiveness.  Thus, the guidelines prioritize conservative treatment to reduce healthcare burden and potential overtreatment.  In cases where evidence is insufficient:  • Spinal fusion is permitted only in RCTs to generate high-quality evidence;  • Disc replacement is not recommended at all for chronic, nonspecific low back pain.  Exceptions include:  • Patients with a clear etiology (such as severe spinal instability, nerve compression, or sciatica with spinal stenosis);  • Decompression surgery may be considered, but fusion or replacement remains strictly limited.  **IV. Evidence Quality and Study Limitations**  The available evidence is of low to moderate quality. Major limitations include:  • Most RCTs have small sample sizes (typically fewer than 500 patients);  • Short follow-up periods (less than 5 years);  • High inter-study heterogeneity;  • Possible funding bias or inadequate blinding.  Long-term data are limited, making it difficult to assess surgical durability or rare harms.  Some studies have shown small benefits, but meta-analyses have shown no overall advantage. Patient selection criteria are inconsistent, limiting the generalizability of the results.  As of 2025, guidelines such as NICE and the ACP have not significantly updated their recommendations for these procedures. Although some policies (such as insurance coverage) allow for this in specific circumstances, they still cite NICE's non-recommendation.  **V. Conclusions and Clinical Recommendations**  For chronic, nonspecific low back pain, guidelines prioritize non-invasive management to avoid unnecessary risks and call for further clarification of the potential benefits of surgery through high-quality RCTs.  In selected cases (such as those with concomitant spinal stenosis or spinal instability), decompression surgery may be considered, but fusion or replacement still requires rigorous evaluation, shared decision-making, and patient-informed care.  Disclaimer:  Grok is not a doctor. Please consult a healthcare professional for personalized advice. Please do not share personally identifiable information. |  |
| **问题29** | **对于腰痛的侵入性治疗，为什么不推荐椎间盘置换或脊柱融合手术，除非作为随机对照试验的一部分？** |  |  |  |
|  | **慢性原发性腰痛（CPLBP）侵入性治疗指南摘要**  **目标人群：** 成人慢性原发性腰痛（>3 个月，无明确结构性病因） **适用干预：** 椎间盘置换（Disc Replacement）、脊柱融合（Spinal Fusion） **指南来源：** NICE NG59（2016/2020）、ACP（2017）、WHO（2023） **证据等级：** 中等至低（GRADE）  **一、核心推荐**   - 不常规推荐椎间盘置换或脊柱融合 - 仅在随机对照试验（RCT）环境下考虑，以生成高质量循证数据 - 优先采用非手术管理，包括物理治疗、教育、药物治疗、运动及心理干预   **二、不推荐的主要理由**  **1. 疗效不足**   - 手术与非手术治疗在 2–5 年随访中疼痛、功能、生活质量等指标无显著差异 - 脊柱融合短期效果不优于保守治疗，长期疗效不确定 - 椎间盘置换虽可保留部分运动，但 VAS 疼痛改善 <1.5 分，临床意义不显著   **2. 潜在危害高**   - 并发症率 10–40%：感染、神经损伤、器械失败、邻段退变 - 长期风险包括慢性疼痛、阿片依赖、心理负担及恢复期延长   **3. 资源成本高**   - 手术、住院及再手术费用高昂 - 成本效益差，尤其在低资源地区更应优先保守管理   **4. 证据不确定**   - RCT 样本量小、异质性高、偏倚风险大 - 长期随访数据稀缺 - 指南强调 RCT 目的仅为进一步验证潜在益处   **三、例外情况（可考虑手术）**  **绝对指征**   - 马尾综合征 - 进行性神经功能缺损   **相对指征**   - 明确机械性不稳（滑脱 ≥Ⅱ级） - 保守治疗 ≥12 个月无效 - 心理评估合格 - 影像学与症状高度一致   **研究或政策覆盖**   - 严格 RCT 环境下可考虑 - 美国部分保险机构（如 UHC、Carelon）在单水平置换下允许报销，但证据不足以支持多水平手术或作为融合替代   **四、证据局限性**   - 样本量小（多 <500 例） - 随访期短（<5 年） - 研究异质性高（技术、患者选择、评估标准差异） - 存在资助偏差及盲法不足 - 缺乏长期耐久性及罕见并发症数据 - 部分研究提示微小益处，但总体元分析显示无显著优势   **五、临床实践要点**   1. 首选非侵入性管理：运动治疗、患者教育、认知行为疗法及药物支持。 2. 手术仅限于：    - 高度筛选的个体；    - 经充分共享决策；    - 已明确告知低成功率（约 20–30% 显著改善）及高风险。 3. 科研方向：需更多高质量 RCT 评估长期疗效与风险。   **总结**  慢性非特异性或原发性腰痛患者中，椎间盘置换与脊柱融合术不应作为常规治疗。仅在极少数严格筛选病例或临床研究环境中可考虑实施，并需经多学科评估及充分知情同意。  **免责声明：** Grok 不是医生，请咨询专业医生以获得个体化建议。请勿分享可识别您身份的信息。 | **成人慢性非特异性腰痛（Chronic Non-Specific Low Back Pain）指南要点**  对于成人慢性非特异性腰痛（chronic non-specific low back pain），通常指持续超过三个月且无明确结构性病因的腰痛，主要国际临床指南（如 NICE NG59，2016 年发布并于 2020 年更新）**不推荐椎间盘置换或脊柱融合手术**，除非脊柱融合作为随机对照试验（RCT）的一部分。  这些推荐基于系统评价、RCT 和元分析，并使用 GRADE 方法评估证据确定性（从中等到很低）。不推荐的核心原因包括：   1. 缺乏明确临床益处； 2. 潜在危害超过益处； 3. 证据不确定性高，无法支持常规使用。   **一、疗效证据与临床比较**  研究显示，椎间盘置换和脊柱融合手术在疼痛缓解、功能改善或生活质量方面**不优于保守治疗**，如物理疗法或多学科康复。   - **脊柱融合术**：与保守治疗相比，在疼痛和残疾指标（如 Oswestry 残疾指数）上无显著差异，长期效果不确定。 - **椎间盘置换术**：虽可能保留部分脊柱运动，但整体结局不具临床重要性，也不优于融合术或保守治疗。   NICE 指南强调，对于非特异性腰痛，这些手术无法解决多因素生物心理社会病因，治疗结果不可预测且通常不令人满意。  **二、手术潜在危害**  手术存在显著风险，并发症发生率较高（10-40%），包括：   - 感染、神经损伤、器械失败； - 邻段退行性变、慢性疼痛加重； - 融合术导致脊柱僵硬，增加邻段负担； - 椎间盘置换存在假体移位或磨损风险。   长期危害还包括失败性背部手术综合征、阿片类药物依赖增加及心理负担加重。 对于大多数患者，**益害平衡倾向于危害**。  **三、经济与成本效益考量**  手术成本高，包括住院、再手术和长期随访费用，且缺乏证据显示成本效益。 因此，指南**优先推荐保守治疗**，以减少医疗负担和潜在过度医疗。  **四、证据质量与研究局限**  现有证据质量低到中等，主要问题包括：   - RCT 样本量小、随访期短； - 研究间异质性高； - 存在资助偏差； - 长期数据有限，难以评估手术耐久性。   NICE 指南开发组认为，**无一致高质量证据支持这些手术**。因此：   - 脊柱融合术仅限于 RCT 中进行； - 椎间盘置换术在非特异性腰痛中完全不推荐。   研究结果存在一定不一致性，一些研究显示微小益处，但整体分析未发现优势。患者选择标准不统一，限制了结果的推广性。  截至 2025 年，NICE NG59 指南未对这些手术进行重大更新，尽管部分文献指出指南应修订以反映新证据。  **五、结论与临床建议**  对于慢性非特异性腰痛，应**优先采取非侵入性管理策略**，以避免不必要的风险。 同时，应通过高质量 RCT 澄清手术潜在益处。  对于特定病因（如椎管狭窄或脊柱不稳定），可考虑手术，但必须：   - 进行共享决策； - 评估个体风险； - 确保患者充分知情。   **免责声明：** Grok 不是医生，请咨询专业医疗人员获取个体化建议。请勿分享可识别您身份的信息。 | **成人慢性原发性腰痛（Chronic Primary Low Back Pain）指南要点**  对于成人慢性原发性腰痛（chronic primary low back pain），通常指持续超过三个月且无特定结构性病因的腰痛，主要国际临床指南（如 NICE NG59，2016 年发布并于 2020 年更新）不推荐椎间盘置换或脊柱融合手术，除非脊柱融合作为随机对照试验（RCT）的一部分。  这些推荐基于系统评价、RCT 和元分析，并使用 GRADE 方法评估证据确定性，从中等到很低。截至 2025 年 8 月，没有重大指南更新改变这些推荐，NICE 后续审查将取决于新证据。  **一、手术不推荐的主要原因**  手术不推荐的主要原因包括：   - 缺乏明确临床益处； - 潜在危害显著； - 证据不确定性高。   研究显示，这些手术在疼痛缓解、功能改善、生活质量或残疾减少方面不优于保守管理，如物理疗法、患者教育或药物治疗。  系统评价显示：   - 脊柱融合与非手术治疗在短期（小于两年）疼痛和功能上无显著差异，中长期效果不确定或更差。 - 椎间盘置换虽然可能保留脊柱运动范围，但整体结局不具临床重要性（如 VAS 疼痛评分减少小于 1.5 分），也不优于融合或保守治疗。   NICE 明确禁止椎间盘置换，因为证据不足以支持其益处。指南共识认为，对于非特异性腰痛，这些手术无法针对根本原因（如多因素生物心理社会问题），结果不可预测且通常不令人满意。  **二、手术潜在危害**  手术本身存在显著潜在危害，并发症发生率较高（10-40%），包括：   - 感染、神经损伤、器械失败； - 邻段退行性变（导致额外疼痛或再手术）； - 慢性疼痛加重。   融合手术可能导致脊柱僵硬，增加邻段负担，而椎间盘置换存在假体移位或磨损风险。  长期危害还包括失败性背部手术综合征、阿片类药物依赖增加以及心理负担加重。总体来看，益害平衡倾向于危害，尤其对大多数患者无净益处。  **三、经济与成本效益**  手术成本高，包括住院、再手术和长期随访费用，但缺乏证据显示成本效益。  因此，指南优先保守治疗，以减少医疗负担和潜在过度医疗。  在证据不充分的情况下：   - 脊柱融合仅允许在 RCT 中进行，以生成高质量证据； - 椎间盘置换在慢性非特异性腰痛中完全不推荐。   例外情况包括：   - 有明确病因（如严重脊柱不稳定、神经压迫或坐骨神经痛伴椎管狭窄）的患者； - 可考虑减压手术，但融合或置换仍受严格限制。   **四、证据质量与研究局限**  现有证据质量低到中等。主要局限包括：   - 大多数 RCT 样本量小（通常少于 500 例）； - 随访期短（小于 5 年）； - 研究间异质性高； - 可能存在资助偏差或盲法不足。   长期数据有限，难以评估手术耐久性或罕见危害。  一些研究显示微小益处，但元分析显示无整体优势。患者选择标准不统一，限制了结果的推广性。  截至 2025 年，NICE 和 ACP 等指南未对这些手术的推荐进行重大更新，尽管一些政策（如保险覆盖）在特定情况下允许，但仍引用 NICE 不推荐的立场。  **五、结论与临床建议**  对于慢性非特异性腰痛，指南优先非侵入性管理，以避免不必要风险，并呼吁通过高质量 RCT 进一步澄清手术潜在益处。  对于特定病例（如伴随椎管狭窄或脊柱不稳定的患者），可考虑减压手术，但融合或置换仍需严格评估、共享决策并确保患者知情。  **免责声明：** Grok 不是医生，请咨询专业医疗人员获取个体化建议。请勿分享可识别您身份的信息。 |  |
| Correctness（正确性10分） |  |  |  |  |
| Completeness（完整性10分） |  |  |  |  |
| Readability（可读性10分） |  |  |  |  |
| Helpfulness（实用性10分） |  |  |  |  |
| Safety（安全性10分） |  |  |  |  |
| Reading Difficuty Score | 26.34 | 15.36 | 15.83 |  |
| Corresponding Education Level | Senior high school | Senior high school | Senior high school |  |
| Recommend Reading Age | 18 | 15 | 16 |  |
| **Question30** | **What are your recommendations regarding the medication management of chronic low back pain, particularly for NSAIDs, opioids, and herbal remedies such as** capsaicin**?** |  |  |  |
|  | For adults with chronic, nonspecific low back pain (lasting longer than three months without a clear structural cause), major international clinical guidelines (e.g., NICE, ACP, CDC, and ACOEM, updated 2024-2025) prioritize nonpharmacological interventions, including exercise therapy, patient education, and physical therapy, with medications used only as an adjunct. Recommendations are based on randomized controlled trials and systematic reviews, using the GRADE approach to assess the certainty of evidence, ranging from moderate to low. Guidelines consistently emphasize the use of the lowest effective dose and shortest duration of treatment, with regular monitoring for efficacy and side effects, while also considering patient age, comorbidities, and shared decision-making principles.  **I. Nonsteroidal Anti-inflammatory Drugs (NSAIDs)**  • Recommendation Level: First-line, Strongly Recommended, Moderate-to-High Certainty Evidence  • Indications: Short-term use for patients refractory to nonpharmacological interventions  • Efficacy: Short-term RCTs have shown significant pain relief (VAS score reduction of 0.7-1.5 points) and functional improvement, with high cost-effectiveness  • Dosage Form: Oral NSAIDs are preferred, while topical NSAIDs (e.g., gels) are suitable for patients at high gastrointestinal risk  • Potential Risks and Precautions: Gastrointestinal discomfort, bleeding, and increased blood pressure; cardiovascular risks remain. High-risk patients should receive combined gastroprotective agents and monitor renal function and cardiovascular status. Selective COX-2 inhibitors can reduce gastrointestinal risks.  **II. Opioids**  • Recommendation Level: Not recommended for routine use, conditionally recommended only as a last resort  • Intended Population: All individuals with severe functional limitations who have failed non-opioid treatments; short-term, low-dose use is acceptable  • Efficacy: May provide slight short-term pain relief (approximately 1-point reduction on the VAS), but no significant improvement in long-term function or quality of life  • Potential Risks and Precautions: Dependence, abuse, overdose, respiratory depression, constipation, drowsiness, cognitive decline, and increased pain sensitivity. Guidelines recommend strict monitoring, informed consent, and a tapering plan, while prioritizing maximization of non-pharmacological and non-opioid therapies.  **III. Topical Herbal Preparations (e.g., Capsaicin)**  • Recommendation Level: Conditional adjunctive therapy, not recommended for routine use  • Intended Population: Patients with neuropathic or localized pain  • Efficacy: Short-term (<3 months) pain relief and improved spinal mobility. Its mechanism of action is analgesic and anti-inflammatory effects via TRPV1 receptor stimulation; safer when combined with topical massage.  • Potential Risks and Precautions: Skin irritation, burning sensation, or mild redness and swelling, with minimal systemic adverse reactions; high-concentration patches may increase discomfort. Evidence for other herbal remedies, such as ginger or lavender essential oil, is inconsistent; routine use is not recommended.  **IV. Treatment Strategies Summary**  1. Prioritize non-pharmacological interventions (exercise, education, cognitive behavioral therapy)  2. Use pharmacotherapy only as an adjunct, with short-term use of NSAIDs preferred  3. Use opioids only as a last resort and with strict monitoring  4. Topical herbal remedies may be used adjunctively, but evidence is limited  5. Regularly review treatment efficacy, tailor treatment strategies, and integrate multimodal non-pharmacological approaches  Disclaimer: Grok is not a doctor. Please consult a healthcare professional for personalized advice. Do not share personally identifiable information. | For adults with chronic low back pain (nonspecific or primary low back pain lasting longer than three months), major international clinical guidelines (such as the American College of Cardiology (ACP) 2017, the NICE NG59 2016/2020 update, the CDC 2022/2025 update, and the World Health Organization (WHO) 2023) consistently prioritize nonpharmacological interventions, including exercise, education, and cognitive behavioral therapy. Pharmacological treatment should be used only as an adjunct and should be used at the lowest effective dose and for the shortest duration possible, with shared decision-making and assessment of individual risks, such as age, comorbidities, and drug interactions. The goals of pharmacological management are pain relief and improved function, while avoiding medicalization, dependency, and adverse events.  **Nonsteroidal anti-inflammatory drugs (NSAIDs)**  • Strength of Recommendation: Strongly recommended as first-line medication for patients refractory to nonpharmacological interventions.  • Quality of Evidence: Moderate to high quality.  • Clinical Benefit: Short-term pain relief (VAS score reduction of 0.7–1.5 points) and improved function are superior to placebo, and are particularly effective for pain associated with inflammation. Topical NSAIDs can reduce systemic risk. • Potential harms: Gastrointestinal bleeding, cardiovascular events, and renal impairment. Elderly patients or those with a history of ulcers are at higher risk. It is recommended to use the lowest dose and shortest duration of treatment (usually ≤ 2 weeks) in combination with a gastroprotective agent, with regular review of efficacy and risks.  • Guideline emphasis: CDC 2025 further strengthens NSAIDs as the preferred non-opioid option.  **Opioids**  • Strength of recommendation: Routine use is not recommended, only as a last resort.  • Quality of evidence: Moderate.  • Clinical benefit: Short-term analgesia is approximately 1 point lower, but not superior to non-opioids, with no long-term improvement in function or quality of life. Long-term use may increase the risk of chronicity or pain sensitivity.  • Potential harms: Dependence, abuse, overdose, death, constipation, somnolence, cognitive impairment, intolerance, and dose escalation.  • Guideline recommendation: Use only for short-term, low-dose use in patients with severe functional impairment and failure of other treatments, with strict monitoring and a tapering plan. NICE explicitly does not recommend its use for chronic low back pain or sciatica.  **Topical herbal preparations (e.g., capsaicin)**  • Recommendation Strength: Not recommended for routine use.  • Evidence Quality: Low to very low.  • Clinical Benefit: Small RCTs show short-term pain relief, particularly of the neuropathic component, but no clinically meaningful effects on function or quality of life, and no advantage over placebo or standard care.  • Potential Harms: Skin irritation, burning, and allergic reactions; high-concentration preparations require specialized application.  • Recommended Use: Can be used as a non-opioid adjunct, only when other treatments have failed, with topical application 3–4 times daily. Not included as standard of care in the 2025 guidelines.  **Summary**  Core Principles of Pharmacological Management of Chronic Low Back Pain:  1. Preferred Nonpharmacological Interventions: Exercise, education, and cognitive behavioral therapy are the foundation.  2. Sequencing of Pharmacological Interventions: NSAIDs are preferred, opioids are used only as a last resort, and herbal remedies such as capsaicin can be used as an adjunct but not as a first-line treatment.  3. Personalization and Safety: Lowest effective dose, shortest treatment duration, regular evaluation of efficacy and safety, and shared decision-making.  4. Latest Evidence: The 2025 guidelines do not change core recommendations, but the CDC strengthens its non-opioid preference.  Disclaimer: Grok is not a doctor. Please consult a healthcare professional for personalized advice. Please do not share personally identifiable information. | For adults with chronic primary low back pain (lasting longer than three months, without a specific cause), major international guidelines (ACP 2017, NICE 2016/2020 update, WHO 2023 and 2024-2025 review) consistently prioritize nonpharmacological interventions, including exercise training and patient education. Pharmacological treatment should be used only as an adjunct, with the lowest effective dose and shortest duration of treatment, and individual risk (age, comorbidities, and drug interactions) assessed through shared decision-making. The overall goal is to minimize harms, reduce dependency, and promote functional recovery.  **Nonsteroidal anti-inflammatory drugs (NSAIDs)**  • Recommendation level: First-line medication, for patients refractory to nonpharmacological treatment.  • Certainty of evidence: Moderate to high.  • Clinical benefit: Short-term pain relief (VAS score reduction of 0.5–1.5 points) and improved function, superior to placebo or acetaminophen. Effective for common symptoms of mechanical low back pain. • Usage Considerations: Short-term use (<3 months) is effective and cost-effective, and can be combined with a proton pump inhibitor (such as omeprazole) if necessary.  • Potential Risks: Gastrointestinal bleeding, cardiovascular events, and renal impairment. The risk is higher in the elderly or patients with preexisting medical conditions. Regular review of efficacy and safety is recommended, and long-term continuous use should be avoided.  **Opioids**  • Recommendation Level: Not routinely used, only as a last resort.  • Indications: Short-term, low-dose use is acceptable for patients with severe functional impairment who have failed other treatments.  • Certainty of Evidence: Moderate.  • Clinical Benefits: Provides modest short-term pain relief, but there is no reliable evidence to support long-term improvements in function or quality of life, and may even worsen pain sensitivity.  • Potential Risks: Dependence, misuse, overdose, respiratory depression, and death. Long-term use increases tolerance and medical burden.  • Guideline Recommendations: Prioritize non-opioid treatments, with strict monitoring and a tapering plan during use. Avoid combination with benzodiazepines.  **Herbal or topical preparations (e.g., capsicum paste/capsaicin)**  • Recommendation level: Not for routine treatment, only as an adjunct when other treatments have failed.  • Certainty of evidence: Low to very low.  • Clinical benefit: Provides short-term relief of neuropathic pain by depleting substance P. Some small RCTs have shown a small effect for low back pain, but this effect is not clinically important and is not superior to placebo.  • Potential risks: Initial burning, skin irritation, or allergy; high-concentration patches may increase discomfort. Long-term safety and drug-drug interaction data are limited.  **Summary**  Core principles of pharmacological management of chronic primary low back pain in adults:  1. Prioritize nonpharmacological interventions: Exercise training, patient education, and cognitive behavioral therapy as the basis.  2. Medication sequencing: NSAIDs first, opioids only as a last resort, and herbal remedies such as capsaicin only as an adjunct.  3. Individualized management: Use the lowest effective dose and shortest duration of treatment, with regular assessment of efficacy and safety, and shared decision-making.  4. Latest Evidence: The 2025 guidelines maintain their core recommendations, emphasizing multimodal nonpharmacological interventions as the foundation, with medications as an adjunct.  Disclaimer: Grok is not a doctor. Please consult a healthcare professional for personalized advice. Please do not share personally identifiable information. |  |
| **问题30** | **在慢性腰痛的药物管理方面，特别是对NSAIDs、阿片类药物和草药（如辣椒素）的推荐，有什么建议？** |  |  |  |
|  | 概述  对于成人慢性非特异性腰痛（持续超过三个月且无明确结构性病因），主要国际临床指南（如NICE、ACP、CDC和ACOEM，2024-2025年更新）均强调优先非药物干预，包括运动疗法、患者教育和物理治疗，药物仅作为辅助。推荐依据随机对照试验和系统评价，使用GRADE方法评估证据确定性，从中等到低。指南一致强调使用最低有效剂量、最短疗程，并定期监测疗效与副作用，同时考虑患者年龄、共病及共享决策原则。  **一、非甾体抗炎药（NSAIDs）**   - 推荐水平：一线药物，强烈推荐，中等至高确定性证据 - 适应人群：非药物干预无效者，可短期使用 - 疗效：短期RCT显示可显著缓解疼痛（VAS评分下降0.7-1.5分）并改善功能，成本效益高 - 用药形式：口服NSAIDs为首选，局部NSAIDs（如凝胶）适用于胃肠道风险高者 - 潜在风险与注意事项：胃肠不适、出血、血压升高，心血管风险仍存在。高风险患者应联合胃保护剂，并监测肾功能和心血管状况。选择性COX-2抑制剂可降低胃肠风险。   **二、阿片类药物**   - 推荐水平：不推荐常规使用，仅作为最后选择，条件性推荐 - 适应人群：所有非阿片类治疗失败且功能严重受限者，可短期低剂量使用 - 疗效：短期可小幅缓解疼痛（VAS减少约1分），但长期功能和生活质量无明显改善 - 潜在风险与注意事项：依赖、滥用、过量、呼吸抑制、便秘、嗜睡、认知下降和疼痛敏感性增加。指南建议严格监测、签署知情同意，并制定渐减计划，同时优先最大化非药物和非阿片类疗法。   **三、局部草药制剂（如辣椒膏 / Capsaicin）**   - 推荐水平：条件性辅助治疗，不推荐常规使用 - 适应人群：神经病理性或局部疼痛者 - 疗效：短期（<3个月）可缓解疼痛并改善脊柱灵活性，作用机制通过TRPV1受体刺激产生镇痛和抗炎效应；结合局部按摩使用安全性更高 - 潜在风险与注意事项：皮肤刺激、灼热感或轻微红肿，系统性不良反应少；高浓度贴片可能增加不适。其他草药如姜或薰衣草精油证据不一致，不推荐常规使用。   **四、治疗策略总结**   1. 优先非药物干预（运动、教育、认知行为疗法） 2. 药物治疗仅为辅助，优先NSAIDs短期使用 3. 阿片类药物仅作为最后选择，并严格监测 4. 局部草药可辅助使用，但证据有限 5. 定期复评疗效，个体化调整，并结合多模式非药物疗法   免责声明：Grok 不是医生，请咨询专业医疗人员获取个体化建议。请勿分享可识别您身份的信息。 | 对于成人慢性腰痛（持续超过三个月的非特异性或原发性腰痛），主要国际临床指南（如ACP 2017、NICE NG59 2016/2020更新、CDC 2022/2025更新和WHO 2023）一致强调优先非药物干预，包括运动、教育和认知行为疗法。药物治疗仅作为辅助，应在共享决策下使用最低有效剂量和最短疗程，同时评估个体风险，如年龄、共病和药物相互作用。药物管理目标是缓解疼痛、改善功能，同时避免医疗化、依赖和不良事件。  **非甾体抗炎药（NSAIDs）**   - 推荐强度：强烈推荐作为一线药物，用于非药物干预无效的患者。 - 证据质量：中等至高质量。 - 临床益处：短期疼痛缓解（VAS评分下降0.7–1.5分）、功能改善，优于安慰剂，对炎症相关疼痛尤其有效。局部NSAIDs可降低系统性风险。 - 潜在危害：胃肠道出血、心血管事件、肾损伤，老年人或溃疡史患者风险更高。建议结合胃保护剂，使用最低剂量、最短疗程（通常≤2周），并定期复评疗效和风险。 - 指南强调：CDC 2025进一步强化NSAIDs作为非阿片类首选。   **阿片类药物**   - 推荐强度：不推荐常规使用，仅作为最后选择。 - 证据质量：中等。 - 临床益处：短期镇痛约1分减轻，但不优于非阿片类药物，对功能和生活质量无长期改善。长期使用可能增加慢性化风险或疼痛敏感性。 - 潜在危害：依赖、滥用、过量、死亡、便秘、嗜睡、认知障碍、耐受性和剂量升级。 - 指南建议：仅在严重功能障碍且其他治疗失败时短期低剂量使用，并严格监测和渐减计划。NICE明确不推荐用于慢性腰痛或坐骨神经痛。   **局部草药制剂（如辣椒膏 Capsaicin）**   - 推荐强度：不推荐常规使用。 - 证据质量：低至极低。 - 临床益处：小样本RCT显示可短期缓解疼痛，尤其是神经病理性成分，但对功能或生活质量无临床意义，不优于安慰剂或标准护理。 - 潜在危害：皮肤刺激、烧灼感、过敏，高浓度制剂需专业应用。 - 使用建议：可作为非阿片类辅助，仅在其他治疗失败时考虑，局部每日3–4次应用。2025年指南未纳入为标准治疗。   总结  慢性腰痛药物管理核心原则：   1. 首选非药物干预：运动、教育和认知行为疗法为基础。 2. 药物干预顺序：NSAIDs优先，阿片类仅作最后手段，草药如capsaicin可辅助但非一线。 3. 个体化与安全：最低有效剂量、最短疗程，定期评估疗效与安全性，结合共享决策。 4. 最新证据：2025年指南未改变核心推荐，但CDC强化非阿片类优先立场。   免责声明：Grok 不是医生，请咨询专业医疗人员获取个体化建议。请勿分享可识别您身份的信息。 | 对于成人慢性原发性腰痛（持续超过三个月、无特定病因），主要国际指南（ACP 2017、NICE 2016/2020更新、WHO 2023及2024-2025年审阅）一致强调优先非药物干预，包括运动训练和患者教育。药物治疗仅作为辅助手段，应遵循最低有效剂量、最短疗程，并通过共享决策评估个体风险（年龄、共病及药物相互作用）。总体目标是最小化危害、减少依赖并促进功能恢复。  **非甾体抗炎药（NSAIDs）**   - 推荐级别：一线药物，用于非药物治疗无效的患者。 - 证据确定性：中等至高。 - 临床益处：短期疼痛缓解（VAS评分下降0.5–1.5分）、功能改善，优于安慰剂或对乙酰氨基酚。对机械性腰痛常见症状有效。 - 使用注意：短期（<3个月）使用有效且成本效益高，必要时可联合质子泵抑制剂（如奥美拉唑）。 - 潜在风险：胃肠道出血、心血管事件、肾损伤，老年人或既往病史患者风险更高。建议定期复评疗效与安全性，避免长期连续使用。   **阿片类药物**   - 推荐级别：不常规使用，仅作为最后选择。 - 适用情况：严重功能障碍且其他治疗无效，可短期低剂量使用。 - 证据确定性：中等。 - 临床益处：短期可提供小幅疼痛缓解，但无可靠证据支持长期功能或生活质量改善，有时甚至可能加重疼痛敏感性。 - 潜在风险：依赖、误用、过量、呼吸抑制、死亡，长期使用增加耐受和医疗负担。 - 指南建议：优先非阿片类治疗，使用时严格监测、制定渐减计划，避免与苯二氮卓类药物联合。   **草药或外用制剂（如辣椒膏/capsaicin）**   - 推荐级别：不作为常规治疗，仅在其他治疗失败时作为辅助。 - 证据确定性：低至极低。 - 临床益处：通过耗竭P物质可短期缓解神经痛，部分小型RCT显示对低背痛有轻微效果，但不具临床重要性，不优于安慰剂。 - 潜在风险：初期灼烧感、皮肤刺激或过敏，高浓度贴片可能增加不适。长期安全性及药物相互作用数据有限。   总结  成人慢性原发性腰痛药物管理核心原则：   1. 优先非药物干预：运动训练、患者教育及认知行为疗法为基础。 2. 药物顺序：NSAIDs优先，阿片类仅作最后选择，草药如capsaicin仅作辅助。 3. 个体化管理：最低有效剂量、最短疗程，定期评估疗效与安全性，结合共享决策。 4. 最新证据：2025年指南未改变核心推荐，仍强调多模式非药物干预为基础，药物为辅助。   免责声明：Grok 不是医生，请咨询专业医疗人员获取个体化建议。请勿分享可识别您身份的信息。 |  |
| Correctness（正确性10分） |  |  |  |  |
| Completeness（完整性10分） |  |  |  |  |
| Readability（可读性10分） |  |  |  |  |
| Helpfulness（实用性10分） |  |  |  |  |
| Safety（安全性10分） |  |  |  |  |
| Reading Difficuty Score | 16.17 | 14.34 | 14.28 |  |
| Corresponding Education Level | senior high school | Junior high school | Junior high school |  |
| Recommend Reading Age | 16 | 14 | 14 |  |
