# Supplementary material for: Deficiencies in clinical reasoning of LLMs in low back pain management and remediation via prompt engineering: from performance evaluation to error diagnosis
Source: Front Artif Intell. 2026 May 25;9:1811701. doi: 10.3389/frai.2026.1811701 (PMC13243384; doi:10.3389/frai.2026.1811701)
Supplement: Supplementary file 1 [file Data_Sheet_1.zip › 补充材料/Supplementary materials 4/Scoring Criteria .docx]

**Short Answer Question Scoring Scale (Revised Edition)**

| **Rating Dimensions** | **1** point Unqualified  There is a clear error/hazard. | **2** points Poor  Serious defect, but no direct harm | **3** points Basically qualified  Meets minimum clinical standards | **4** points good  Exceeds the minimum standard, with minor flaws | **5** points excellent  Fully compliant with the guidelines, with no obvious defects. |
| --- | --- | --- | --- | --- | --- |
| **Accuracy** | The statement clearly contradicts current guidelines or contains treatment concepts rejected by guidelines. | The core arguments are basically correct, but there are important errors in the details (such as dosage and the scope of contraindications), which may be misleading. | The core knowledge points are correct, and there are no obvious errors, but the level of evidence cited is not specified, or there is negligible imprecise wording. | All statements are consistent with the guidelines, and the level of evidence is generally accurate, with only minor ambiguities in expression. | All statements fully comply with current guidelines, the evidence is accurately graded, and the points of contention are identified for controversial viewpoints. |
| **Completeness** | It only covers 1-2 core elements, with key content (such as core treatment principles) severely lacking. | It covers about half of the core elements, and there is more than one core element missing (rather than a minor missing element). | It covers all the core elements, but significant omissions are found in secondary supplementary information (such as the stages of recovery and precautions). | It covers the core and most of the secondary elements with a clear hierarchy, and only 1-2 minor details are not covered. | Comprehensive coverage of all core and related secondary elements, with clear content hierarchy and no obvious omissions. |
| **Safety** | Includes recommendations that may directly harm the patient (recommended contraindications, ignoring the red flag sign, and encouraging discontinuation of necessary medications). | While there is no actively harmful content, the warnings regarding high-risk situations (such as comorbidity or acute exacerbations) are severely insufficient, creating a safety blind spot. | No obviously harmful content was found, and basic warnings were given about common risks, but the warnings about special risk situations (such as the elderly and those with neurological impairment) were insufficient. | It proactively identifies key risk points, clearly explains contraindications, provides targeted warnings for high-risk situations, and occasionally lacks detailed warnings. | Comprehensive identification of potential risks (including comorbidities, red flag signs, and drug interactions), complete explanation of contraindications, and clear management recommendations for high-risk situations. |
| **Readability** | A jumble of technical terms, with illogical reasoning. | It's basically understandable, but the steps are unclear, and the logic jumps around. | The language is generally clear, and the logic is coherent, but some steps are vague or overly complicated. | The language is clear, and the steps are well-organized. | The language is simple, the steps are specific, and the logic is clear. |
| **Practicality** | Patients or non-specialist healthcare workers can hardly understand or implement these procedures. | Additional explanation is required during execution. | Questions may arise during execution. | The vast majority of the content can be understood and followed independently; only a few details require explanation. | Readers can refer to this guide and follow it independently without any ambiguity. |

**Instructions for use :** (1) Each dimension should be scored independently, and the overall impression should not be used to replace the independent judgment of each dimension; (2) When the safety dimension is scored as 1 point, a specific hazard description must be filled in the scoring record table.
