# Supplementary material for: Deficiencies in clinical reasoning of LLMs in low back pain management and remediation via prompt engineering: from performance evaluation to error diagnosis
Source: Front Artif Intell. 2026 May 25;9:1811701. doi: 10.3389/frai.2026.1811701 (PMC13243384; doi:10.3389/frai.2026.1811701)
Supplement: Supplementary file 1 [file Data_Sheet_1.zip › 补充材料/Supplementary materials 5/Deepseek-V2.5/A(Deepseek-V2.5).docx]

| **Question 1** | **How to define and classify lower back pain?** |
| --- | --- |
|  | **I. Definition of Low Back Pain**  Low back pain (LBP) refers to pain, stiffness, or muscle tension in the area from T12-L1 to the sacrum, which may be accompanied by radiating pain in the lower extremities. Approximately 84% of adults worldwide will experience low back pain at least once in their lifetime, and it is the leading cause of disability (WHO 2020).  **II. Classification System of Low Back Pain**  **A. Classification by disease course**  - Acute: Duration < 6 weeks, sudden onset, often associated with mechanical injury.  - Subacute: Lasts 6 weeks to 3 months, with a high risk of becoming chronic.  - Chronic: Lasting longer than 3 months, often accompanied by psychosocial factors, and accounts for 70% of medical expenditures.  **B. Classification by etiology**  1. Mechanical lower back pain (over 90%):  - Discogenic: nucleus pulposus protrusion/degeneration (such as L4-L5, L5-S1 segment).  - Small joint disorders: arthritis or sprains.  - Muscle and ligament injuries, such as strain of the quadratus lumborum and erector spinae muscles.  - Spinal stenosis: intermittent claudication.  2. Non-mechanical low back pain:  - Inflammatory: such as ankylosing spondylitis (morning stiffness > 30 minutes).  - Neoplastic: Night pain progressively worsens.  - Infectious: such as intervertebral discitis (accompanied by fever).  - Referred pain to internal organs, such as kidney stones or pancreatitis.  3. Radicular pain:  - Sciatica: positive straight leg raise test (pain elicited at <60 degrees).  - Nerve root distribution: L5 root (numbness on the dorsum of the foot), S1 root (numbness on the sole).  **C. Classified by anatomical structure**  - Intervertebral disc: Herniation/degeneration (worsened by sitting).  - Small joints: Osteoarthritis (extension and rotation pain).  - Sacroiliac joint: dysfunction (positive Fortin finger pressure test).  - Nerve root: entrapment (specific muscle weakness).  **D. Classification by pain pattern**  - Axial pain: deep pain along the midline of the spine.  - Referred pain: Dull pain in the buttocks/groin (without neurological signs).  - Radicular pain: sharp pain distributed along the nerve root.  **E. Special Classification**  - Nonspecific low back pain (NSLBP): accounting for 85%, with no clear pathology.  - Central sensitization type: Pain is induced by light touch.  - Postoperative failure syndrome (FBSS): persistent pain after lumbar spine surgery.  **F. Danger signals (urgent assessment required)**  - Cauda equina syndrome: Numbness in the saddle area + urinary retention/fecal incontinence (surgery required within 24 hours).  - Infection/tumor: Night pain + unexplained weight loss >10%.  - Fracture: History of osteoporosis + severe pain after trauma.  - Aortic aneurysm: pulsating mass + tearing pain.  **G. Biopsychosocial classification (core of chronic low back pain)**  - Psychological: Catastrophic thinking/depression (BDI > 14).  - Society: Job dissatisfaction/Litigation status.  - Behavior: Fear and avoidance belief (FABQ > 34).  **III. Key Points of Clinical Practice**  **A. Diagnostic process:**  Step 1: Screen for warning signs. Danger signals lead to Imaging/laboratory tests. No danger signs lead to assessment of mechanical pain. Accompanied by nerve root symptoms leads to Electromyography/MRI. No neurological symptoms leads to Functional rehabilitation + monitoring.  **B. Treatment stratification:**  - Acute phase: NSAIDs (such as celecoxib) + stay active.  - Chronic phase: Exercise therapy + psychological intervention.  - Radicular pain: Epidural injection.  Special Attention: Early MRI does not improve prognosis in patients with nonspecific low back pain (NEJM 2023), and it is recommended to perform imaging examinations only after 6 weeks of conservative treatment have failed. Low back pain lasting longer than 4 weeks or accompanied by neurological symptoms should be evaluated by a specialist. |
| **Question 4** | **What are some recommendations for non-pharmacological interventions for lower back pain?** |
|  | **I. Exercise Therapy**  Exercise therapy is a first-line intervention for chronic nonspecific low back pain, which can improve pain and function and prevent recurrence.  1. Aerobic Exercise (such as walking, swimming, or cycling) is recommended, at least 150 minutes per week at moderate intensity. This can promote the release of endorphins and reduce pain sensitivity. During the acute phase, this can be adjusted to intermittent activity (such as short walks every 20-30 minutes).  2. Core Stability Training focuses on activating deep core muscles (such as the transverse abdominis, multifidus, and pelvic floor muscles) to enhance the dynamic stability of the lumbar spine.  Recommended exercises:  - Supine pelvic tilt  - Four-point kneeling position, bird dog pose  - Modified plank exercise (to avoid lumbar compensation)  - Glute bridge (activates the gluteus maximus to reduce the load on the lumbar spine)  3. McKenzie Therapy is suitable for discogenic pain (such as centralized symptoms) by relieving nerve compression through repetitive spinal-directed movements (such as extension). An individualized assessment is required before application.  4. Mind-Body Training: Yoga (emphasizing diaphragmatic breathing and spinal alignment) or tai chi (improving proprioception) can reduce pain intensity (Level of evidence: B).  **II. Posture Correction and Ergonomics**  - Adjust sitting posture to maintain lumbar lordosis, and use a lumbar support cushion to support the L3-L5 segments.  - Follow the "90-90-90 rule" (hips, knees, and ankles should all be at 90 degrees), and keep the screen height level with your eyes.  - During standing and lifting, alternately step on a footstool.  - Use the "hip hinge" mode when lifting to activate core muscles and reduce lumbar shear forces.  - Sleeping on your side: place a pillow between your knees to maintain a neutral pelvic position; when sleeping on your back, place a pillow under your knees to reduce pressure on the intervertebral discs (avoid sleeping on your stomach).  **III. Physical Modalities**  - After the acute phase, heat therapy: applying heat (40-45 degrees Celsius) can relieve muscle spasms and promote blood circulation.  - Cryotherapy (within 48 hours): Applying ice (15-20 minutes per application) can reduce inflammatory exudation during the acute phase.  - Transcutaneous electrical nerve stimulation (TENS) at high frequencies (50-100 Hz) may inhibit pain transmission through gate control theory (short-term effects are clear, but long-term evidence is limited).  **IV. Manual Therapy**  - Mobilization: It is a grade I-IV manipulation technique targeting the facet joints of the lumbar spine to improve range of motion (ROM).  - Soft tissue release involves trigger point release of spastic muscle groups such as the quadratus lumborum and piriformis.  - Traction may relieve symptoms of nerve root compression (suitable for herniated discs with radiating pain), but should be combined with clinical evaluation.  **V. Cognitive Behavioral Therapy (CBT)**  For patients with chronic pain, fear-avoidant beliefs can be reduced through cognitive restructuring to reduce pain-related behavioral disorders.  **VI. Other Auxiliary Measures**  - Weight management (BMI < 25): Reduce mechanical load on the lumbar spine.  - Quitting smoking: Nicotine can reduce blood supply to the intervertebral discs and accelerate their degeneration.  Important Notes (excluding Red Flags): Further imaging examinations (such as MRI) are required if the following conditions are present:  - Progressive neurological dysfunction (such as cauda equina syndrome: saddle anesthesia, urinary retention)  - Nighttime pain or pain at rest (suggests a tumor or infection)  - History of trauma or risk of osteoporosis (excluding fractures) |
| **Question 5** | **What are some recommendations for drug intervention for lower back pain?** |
|  | **I. Acute Lower Back Pain (< 4 weeks)**  1. First-line Drugs  **A. Paracetamol**  - Recommended dose: 500-1000 mg once every 6-8 hours  - Maximum daily dose: 4g  - Advantages: Fewer gastrointestinal side effects; preferred for patients with normal liver and kidney function.  **B. Nonsteroidal Anti-inflammatory Drugs (NSAIDs)**  - Ibuprofen: 200-400 mg once every 6-8 hours  - Celecoxib: 200 mg, twice daily (COX-2 selective inhibitors with fewer gastrointestinal side effects)  - Precautions: Assess gastrointestinal/cardiovascular risk; avoid using multiple NSAIDs at the same time.  2. Second-line Drugs  Muscle relaxants (such as etorizine hydrochloride and tizanidine):  - Suitable for those with obvious muscle spasms.  - Short-term use is recommended (less than or equal to 1 week).  - Be aware of side effects such as drowsiness and dizziness.  **II. Chronic Lower Back Pain (> 12 weeks)**  1. Neuropathic Pain Components  Gabapentin/Pregabalin:  - Especially suitable for radiation-induced nerve root pain.  - Dosage needs to be titrated slowly.  - Common side effects: dizziness, edema, drowsiness.  2. Centrally Acting Analgesics  Duloxetine:  - Dosage: 30-60 mg, once daily (SNRI class).  - It has significant effects on chronic low back pain complicated by mood disorders/depression.  3. Opioids  - Should only be used as a last resort when other treatments have failed.  - Tramadol extended-release tablets.  - Recommended usage time: less than or equal to 4 weeks.  - The benefit-risk ratio must be assessed, and informed consent must be obtained.  **III. Medication Use in Special Circumstances**  1. Osteoporosis-related lower back pain:  - Basic treatment: Calcium supplements + Vitamin D.  - Anti-bone resorption drugs: bisphosphonates (such as alendronate sodium).  2. Inflammatory low back pain (such as ankylosing spondylitis):  - Biologics: TNF-alpha inhibitors (such as etanercept and adalimumab).  **IV. Topical Medication**  - Topical NSAIDs: such as diclofenac gel and patches.  - Lidocaine patch (5%): Suitable for areas of localized neuralgia.  **V. Principles of Medication**  - Stepwise treatment: Prioritize low-risk analgesics and gradually upgrade them.  - Use the lowest effective dose and the shortest treatment course.  - Regularly assess efficacy and adverse reactions.  - Be aware of drug interactions, especially in elderly patients with multiple comorbidities.  **VI. Contraindications and Warnings**  1. People who are prohibited from using NSAIDs:  - Active peptic ulcer.  - Severe heart failure or renal insufficiency.  - Aspirin triad (asthma + nasal polyps + NSAID allergy).  2. Groups where muscle relaxants should be used with caution:  - Glaucoma patients.  - Myasthenia gravis patients.  **VII. Other Precautions**  - All pharmacological interventions should be combined with non-pharmacological measures (such as exercise and psychological interventions).  - If the pain continues to worsen, imaging examinations are needed to rule out serious underlying causes (such as tumors or infections).  - Pregnant patients: Medication selection should be carefully considered after professional evaluation. |
| **Question 7** | **What are the appropriate criteria for home rehabilitation of lower back pain?** |
|  | Patients with lower back pain should meet the following scientific screening criteria to ensure safety and effectiveness when undergoing home-based rehabilitation.  **I. Medical Access Standards**  1. Diagnostic Certainty  - Clinically diagnosed with nonspecific low back pain (excluding tumors/infections/fractures, etc.)  - No progressive signs of neurological damage (such as cauda equina syndrome).  - Imaging findings show no indication for surgery (e.g., disc herniation > 6mm).  2. Pain Characteristics  - VAS score less than or equal to 6 (moderate to severe).  - No persistent rest pain at night.  - Pain regularity is related to body position.  **II. Functional Evaluation Standards**  1. Basic Activity Skills  - Capable of independently transferring from a supine to a sitting position.  - Able to maintain a sitting posture for more than 30 minutes.  - Indoor walking more than 100 meters without weight.  2. Exercise Tolerance  - Borg scale score less than or equal to 13 (slightly tiring).  - Able to complete 5 chair stand-up tests (30cm standard chair).  - Can tolerate 20 minutes of low-intensity aerobic exercise.  **III. Cognitive Psychological Standards**  1. Comprehension Ability  - Able to repeat 75% or more of the key points of rehabilitation guidance.  - Digital rehabilitation tools can be used independently.  - Master pain self-assessment methods.  2. Psychological State  - Pain Catastrophism Scale (PCS) score < 20.  - No moderate to severe depression (PHQ-9 < 10).  - Treatment adherence rating: Grade B or higher.  **IV. Environmental Support Standards**  1. Physical Environment  - Possesses a safe training space of 2 square meters or more.  - There are suitable training aids (yoga mat/suspension points, etc.).  - Emergency call devices are available.  2. Social Support  - Family members possess basic guardianship skills.  - Guaranteed 3 remote rehabilitation supervision sessions per week.  - Emergency referral plan in place.  **V. Dynamic Exclusion Indications**  Home-based rehabilitation should be suspended immediately if any of the following situations occur:  - New onset of radiating pain in the lower extremities.  - Bladder and rectal dysfunction.  - Unexplained weight loss > 10%.  - Persistent worsening pain after training > 24 hours.  (Based on: APTA Home Rehabilitation Safety Guidelines 2023 Edition + ISPOR Value Assessment Framework) |
| **Question 12** | **What are the recommended treatment goals for patients with subacute and chronic low back pain?** |
|  | **I. Core Objectives of the Subacute Phase (6 weeks to 3 months)**  1. Pain Control  Objective: To reduce pain intensity to NRS less than or equal to 3/10 (tolerable level for daily activities) and reduce pain interference with daily life.  - Medication control: Reduce dependence on analgesics, and control the frequency of NSAID use to 3 times/week or less.  2. Functional Reconstruction  - Basic activity recovery: Complete 30 minutes of seated work independently to avoid discomfort caused by prolonged sitting.  - Complete 5 sit-to-stand transitions painlessly to enhance functional mobility.  - Once the walking endurance reaches 500 meters (on flat ground), assess the endurance recovery.  - Core muscle endurance standards: The static glute bridge should be held for more than 90 seconds. Side plank for more than 45 seconds per side to improve the stability of the core muscles.  3. Neuromuscular Control  - Movement pattern correction: Using surface electromyography feedback, the coordinated contraction rate of the back and abdominal muscles reaches more than 80%.  - Improved lumbar and pelvic rhythm: Coordination is assessed through a 4D motion analysis system, improving lumbar and pelvic coordination and restoring normal movement patterns.  **II. Advanced Goals for the Chronic Phase (> 3 months)**  1. Enhanced Functionality  - Recovery of career-related abilities: For manual laborers: able to safely complete a 20 kg deadlift and gradually recover their load capacity. For people who sit for long periods of time: Being able to maintain the correct sitting posture for more than 1 hour can prevent back pain caused by prolonged sitting.  - Dynamic stability test: The YBalance test shows that lower limb symmetry is more than 85%, which assesses lower limb balance ability. Standing on one leg with eyes closed for more than 30 seconds improves single-leg stability and core control.  2. Psychological Adaptation  - Improved pain catastrophic cognition: PCS score decreased by 50% or more, reducing excessive worry about pain.  - Improvement of fear and avoidance beliefs: Fear and avoidance behaviors were improved to the normal range using the FABQ scale.  3. Metabolic and Structural Improvements  - Increased cross-sectional area of paraspinal muscles: MRI measurements showed an increase of 10% or more in the cross-sectional area of the paraspinal muscles, indicating improved muscle mass.  - Body fat percentage: less than 20% for men and less than 25% for women, which helps reduce the burden of weight on the spine.  **III. Long-term Goals for Preventing Recurrence**  1. Establishing an Exercise Habit  - Engage in at least 150 minutes of moderate-intensity exercise per week (such as swimming or cycling) to improve physical fitness and prevent recurrence of lower back pain.  - Perform 10 minutes of targeted core training daily (such as the McGill Advanced 3-item set) to improve core stability.  2. Environmental Adaptation  - The workplace ergonomic modification compliance rate is 100%, ensuring that the work environment meets the requirements for correct posture and load-bearing capacity.  - Learn to master more than three pain self-relief techniques (such as McKenzie self-reset) to improve self-management skills.  3. Social Participation  - To restore the patient's pre-injury work intensity, with an RTW rate more than 90%, ensuring the patient can smoothly return to work.  - The quality of life score (SF36) reached the age-standard level, improving the patient's overall health and life satisfaction.  **IV. Selection of Efficacy Assessment Tools**  1. Subjective Assessment  - Pain and Function: The ODI (Occupational Disability Index) is used to assess the degree of functional recovery, with a target of less than 20%.  - Psychological state: The STAI Anxiety Scale was used to assess psychological adaptability, with a target score of less than 40.  2. Objective Assessment  - Kinematic analysis: Using a 3D motion capture system, assess the recovery of spinal mobility, with a target of more than 75%.  - Electromyography (EMG) monitoring: Monitor the activation delay of the multifidus muscle, with a target of less than 50 ms, to ensure coordinated work of the lumbar and back muscle groups.  **V. Key Implementation Principles**  1. Individualized step-by-step advancement: An assessment is conducted every two weeks, and patients can only advance to the next stage after achieving all the basic goals of the current stage.  2. Multimodal integration: Exercise therapy should be combined with cognitive behavioral interventions, including psychological counseling at least once a week, to help patients improve their emotional response to pain.  3. Technology Empowerment: We recommend using smart rehabilitation devices (such as the Huber motion assessment system) to quantify progress, help track rehabilitation progress, and adjust treatment plans. |
| **Question 21** | **For patients with acute or subacute low back pain, which non-pharmacological treatments should clinicians prioritize, and why are these treatments highly recommended?** |
|  | For patients with acute or subacute (nonspecific) low back pain, authoritative international clinical guidelines (such as NICE and ACP) strongly recommend non-pharmacological treatment as the first-line option. These methods are based on evidence-based medicine and have clear effectiveness, safety, and good cost-effectiveness.  **I. Patient Education (Strongly Recommended)**  The core of patient education is informing patients that lower back pain is usually a self-limiting condition, with approximately 80% of patients experiencing significant improvement within six weeks. This helps reduce patient anxiety. Physicians should encourage patients to maintain daily activities, avoid absolute bed rest, and gradually restore daily function. Studies show that proper education can not only reduce the risk of chronic lower back pain but also promote functional recovery. In particular, the ACP guidelines indicate that educational interventions are more effective than passive treatment alone. It is important to note that prolonged bed rest for more than one or two days may delay recovery.  **II. Exercise Therapy (Highly Recommended)**  During the acute phase, gentle activities such as walking or low-intensity stretching are recommended. In the subacute phase, more structured exercises, such as core stability training, yoga, and Pilates, can be introduced. Exercise can enhance muscle function, improve blood circulation, and shorten recovery time. A Cochrane review indicates that regular exercise can reduce the recurrence rate of low back pain. Although the ACP guidelines classify the evidence level for exercise as low to moderate quality, its clinical benefits are clear.  **III. Heat Application (Highly Recommended)**  It is recommended to use a hot towel or heating pad at a temperature of 40 to 45 degrees Celsius for 15 to 20 minutes at a time, several times a day. Applying heat can relieve muscle spasms and promote local blood flow, thus significantly reducing pain in a short period. Randomized controlled trials have shown that applying heat is more effective than a placebo. This method is safe, inexpensive, and suitable for home use.  **IV. Manual Therapy (Highly Recommended)**  Manual therapy, such as spinal manipulation and massage, is suitable for patients whose condition does not respond well to medication or exercise. Related studies show that manual therapy can relieve pain and improve function in the short term, with effects comparable to medication. It should be performed by a professional physical therapist or chiropractor, avoiding the use of high-risk techniques.  **V. Psychological Intervention (Highly Recommended During the Subacute Phase)**  For patients with fear-avoidant beliefs, psychological interventions such as cognitive behavioral therapy and mindfulness-based stress reduction can effectively prevent the condition from becoming chronic. Tools such as STarT Back can be used to identify high-risk individuals. Studies have shown that combining psychological interventions with exercise yields even better results.  **VI. Reasons for Strong Recommendation**  - Evidence-based studies show that these interventions have clear short-term benefits in randomized controlled trials and systematic reviews.  - They are highly safe, avoiding the risks of gastrointestinal bleeding and dependence associated with nonsteroidal anti-inflammatory drugs (NSAIDs) and opioids.  - They are cost-effective and suitable for promotion in primary healthcare.  - They align with the patient-centered biopsychosocial model of medicine, emphasizing self-management.  **VII. Measures That Are Not Recommended or Are Weakly Recommended**  During the acute phase, routine use of acupuncture, traction, and electrotherapy is not recommended due to insufficient evidence or unclear efficacy. Absolute bed rest not only fails to accelerate recovery but may actually prolong the course of the illness; therefore, it is not recommended.  **VIII. Summary**  Treatment of acute or subacute low back pain should prioritize proactive interventions such as patient education, exercise, and heat therapy. These measures promote natural recovery, reduce reliance on medical resources, and lower the risk of chronicity. Physicians should develop individualized treatment plans based on patient preferences and resource availability. |
| **Question 23** | **Why is it not recommended to use imaging examinations (such as X-ray, CT, or MRI) as a routine method for diagnosing chronic nonspecific low back pain unless a specific cause is suspected?** |
|  | Risk Assessment for Low Back Pain (with or without sciatica) in individuals aged 16 and older: A guide to using the STarT Back tool.  **I. Tool Positioning and Advantages**  STarT Back (Subgroups for Targeted Treatment) is a risk stratification tool based on a 9-item questionnaire that can be completed in 5 minutes. It helps classify patients with nonspecific low back pain into low, intermediate, and high-risk groups, thereby matching intervention intensity. Its advantages lie in its simplicity, evidence-based approach, ability to improve prognosis, and reduction of healthcare costs.  **II. Scope of Application**  This treatment applies to patients with nonspecific low back pain (including radiating pain to the lower extremities), but red flag signs (such as infection, fracture, tumor, cauda equina syndrome, etc.) and severe nerve defects must be ruled out first.  **III. The Risk Stratification**  The questionnaire consists of 9 items (1 point each), covering pain distribution and intensity, functional impairment, comorbid pain location, and psychosocial factors (fear-avoidance, depression, pessimistic expectations, etc.).  - Total score 0-3: Low risk, good prognosis.  - Total score 4-6: Medium risk, with physiological or mild psychosocial risks.  - Total score 4 or more points and psychosocial factors 2 or more items: High risk, high probability of chronicity.  **IV. Principle of Tiered Intervention**  - Low Risk: Education is the primary approach (informing patients that lower back pain is often self-limiting and encouraging activity), heat therapy, short-term NSAIDs if necessary, avoiding imaging and overtreatment, and follow-up evaluation in 1-2 weeks.  - Medium Risk: Structured exercise (core stabilization, McKenzie therapy), short-term manual therapy, basic psychological support, short-term medication (NSAIDs plus muscle relaxants) if necessary, reassessment in 2-4 weeks.  - High Risk: Multidisciplinary approach (physical therapy + formal CBT/ACT + vocational rehabilitation), individualized progressive exercise, cautious use of medication (avoiding opioids), and close follow-up.  **V. Special Indications for Sciatica**  ST-T Back can still be used to predict the risk of chronicity, but if there is significant nerve root compression (such as decreased muscle strength or loss of reflexes), MRI and specialist consultation are required; short-term glucocorticoid therapy (oral or epidural injection) may be considered for intermediate/high-risk patients.  **VI. Evidence and Significance**  Randomized controlled trials have shown that STarT Back-based stratified treatment can reduce unnecessary interventions by approximately 30%, and multidisciplinary management in the high-risk group can reduce the 1-year risk of chronicity by approximately 50%. This approach can save medical resources, reduce the overuse of imaging, and promote early recovery.  **VII. Limitations**  Relies on patient self-reporting; culture and language may affect scores; not applicable to patients with the red flag sign or surgical indications. The Chinese version has been validated locally.  **VIII. Clinical Process Overview**  Initial diagnosis to rule out red flag sign → Complete STarT Back → Risk stratification → Intervention according to level → Regular review and adjustment of treatment plan. |
