# Supplementary material for: Deficiencies in clinical reasoning of LLMs in low back pain management and remediation via prompt engineering: from performance evaluation to error diagnosis
Source: Front Artif Intell. 2026 May 25;9:1811701. doi: 10.3389/frai.2026.1811701 (PMC13243384; doi:10.3389/frai.2026.1811701)
Supplement: Supplementary file 1 [file Data_Sheet_1.zip › 补充材料/Supplementary materials 5/Deepseek-V2.5/B(Deepseek-V2.5).docx]

| **Question 1** | **How to define and classify lower back pain? (With Safety Precautions and Evidence-Based Details)** |
| --- | --- |
|  | Important Note: The following information is compiled based on evidence-based medicine guidelines and is intended to provide a professional reference framework. It cannot replace face-to-face consultation with a licensed physician. Any new, persistent, or worsening lower back pain should be evaluated by a professional medical individual.  **I. Definition of Low Back Pain (Based on Clinical Guidelines)**  In clinical guidelines, lower back pain is generally defined as pain, muscle tension, or stiffness in the area below the lower ribcage and above the gluteal crease, with or without radiating or referred pain in the lower extremities (legs).  Core Feature: The location of the pain is the core definition. It is not a disease in itself, but a symptom.  Disease Course Classification (crucial for guiding treatment decisions):  - Acute low back pain: Duration of illness less than 6 weeks.  - Subacute low back pain: Duration of 6 to 12 weeks.  - Chronic lower back pain: Duration of illness more than 12 weeks.  **II. Classification of Low Back Pain (Based on Etiology/Pathological Mechanism)**  1. Specific Lower Back Pain (approximately 15%)  This refers to lower back pain caused by a clear pathophysiological reason. Identifying these conditions is a primary task of clinical assessment, as some require urgent intervention.  Severe pathological diseases (indicated by the "red flag" sign):  - Infections: Such as intervertebral discitis, epidural abscess.  - Tumor: Primary or metastatic spinal tumor.  - Fractures: Especially osteoporotic compression fractures or post-traumatic fractures.  - Cauda equina syndrome: This is a true orthopedic emergency.  - Inflammatory arthritis such as ankylosing spondylitis.  Radicular Pain (Sciatica):  - Definition: Radiating pain along the nerve distribution area caused by nerve root compression or irritation, usually extending below the knee.  - Common causes: Lumbar disc herniation, spinal stenosis, osteophyte compression, etc.  Other Specific Structural Lesions:  - Spinal stenosis: Characteristic manifestation is neurogenic claudication (pain/weakness in the lower limbs after walking, which can be relieved by sitting down or bending forward).  - Vertebral slippage: One vertebra slides forward or backward relative to another vertebra.  2. Nonspecific Lower Back Pain (approximately 85%)  This refers to lower back pain without a clear specific pathological structural change. The pain may originate from strain or dysfunction of structures such as muscles, ligaments, facet joints, and intervertebral discs, but there is no clear corresponding finding on conventional imaging. This is the most common type, and its management focuses on functional assessment, rehabilitation, and intervention for psychosocial factors.  3. Classification Based on Influencing Factors (focus on "yellow flag", "blue flag", and "black flag")  Modern guidelines emphasize the biopsychosocial model, considering the following when classifying:  - Mechanical pain: Worsens with activity, is relieved by rest, and is posture-related.  - Nociceptive pain: Associated with tissue damage or potential damage.  - Neuropathic pain: Caused by nerve damage or disease, often described as burning, electric shock, or numbness.  - Accompanied by relevant psychosocial factors ("yellow flag"): Such as fear of pain, avoidance behavior, depression, anxiety, job dissatisfaction, or compensation issues, these are important risk factors for chronicity and dysfunction.  **III. Core Safety Precautions and Verification of "Red Flag" Signs**  **A. "Red Flag" Signs Requiring Immediate Medical Attention (Emergency Room):**  1. Signs of cauda equina syndrome:  - Saddle area numbness: Progressive numbness in the inner thigh, perineum, and perianal area.  - New or worsening bladder dysfunction: Urinary retention (inability to urinate) or urinary incontinence (inability to control urination).  - New or worsening bowel dysfunction: Fecal incontinence.  - Progressive loss of lower limb motor function: such as foot drop (inability to lift the ankle), limping, or progressive weakness in both legs.  2. Signs of serious infection or tumor:  - Progressively worsening nighttime or resting pain.  - Unexplained fever, chills, and a recent history of bacterial infection.  - Unexplained and significant weight loss.  - History of cancer, new onset of back pain.  3. Signs of severe trauma:  - High-risk post-traumatic injury: Such as severe lower back pain following a car accident or fall from a height.  - Severe pain following minor injuries (such as slipping) in older adults or those at risk of osteoporosis.  **B. Signs Requiring Further Medical Evaluation (Non-Urgent, But Requiring Medical Attention):**  1. Age factor: First onset after 55 years or before 20 years.  2. Pain characteristics: Severe pain that cannot be relieved by any posture.  3. Disease progression: The pain continues to worsen and shows no signs of relief.  4. History of systemic diseases: History of long-term steroid use, immunodeficiency, or intravenous drug use.  5. Persistent neurological symptoms: Progressive numbness and weakness in one or both lower limbs.  **IV. Detailed Actions for Patients/Assessors**  1. Initial Assessment Checklist:  - Step 1 (Safety Screening): Systematically ask about and verify all the above "red flag" signs. If any are present, immediately stop self-treatment and go to the emergency room.  - Step 2 (Classification): Inquire about the nature of the pain (sharp, dull, radiating pain?), aggravating and relieving factors (activity/rest? morning stiffness?), and duration of the illness.  - Step 3 (Functional Impact): Understand the extent to which pain affects walking, sitting, working, and sleeping.  - Step 4 (Psychosocial Factors): Gain a preliminary understanding of work stress, perceptions of pain, and emotional state ("yellow flag").  2. Initial Self-Management for Nonspecific Low Back Pain (after evaluation by a doctor and without red flag signs):  - Maintain activity: The guidelines strongly recommend maintaining normal activity as much as possible within the limits of pain tolerance, and avoiding absolute bed rest.  - Gentle exercise: Engage in low-impact activities such as walking or swimming.  - Posture Adjustment: Avoid prolonged sitting and change your posture regularly; use a lumbar support when sitting.  - Short-term pain control: Over-the-counter nonsteroidal anti-inflammatory drugs (NSAIDs) such as ibuprofen (provided there are no contraindications) or acetaminophen may be considered, and used strictly according to the instructions for short-term use.  - Hot compress: For most patients with acute or chronic low back pain, hot compresses can relieve muscle tension.  **V. Evidence-Based Basis and Uncertainty Explanation**  - Main Guidelines Based On: This answer comprehensively references the latest clinical practice guidelines on low back pain management from the American College of Physicians (ACP), the National Institute for Health and Care Excellence (NICE) in the UK, the American Physical Therapy Association (APTA), and a series of reviews in The Lancet.  - Standard for Imaging Examinations: Guidelines generally emphasize that routine X-ray, CT, or MRI examinations are not recommended in the early stages of nonspecific low back pain without the "red flag" sign (usually within 6 weeks), as their findings often do not match the pain symptoms and may lead to unnecessary treatment and anxiety.  - Uncertainty and Individual Differences: Lower back pain presents with extreme complexity. This answer provides a general framework. Specific diagnosis and treatment plans must be individualized by a physician based on the patient's complete medical history, physical examination (including neurological examination), and any possible ancillary examinations.  Summary: Defining and classifying low back pain is a systematic clinical reasoning process that begins with eliminating warning signs (red flags), then distinguishing between specific and nonspecific symptoms, and finally developing a patient-centered treatment plan aimed at functional recovery within a biopsychosocial framework. Safety is always the top priority. |

| **Question 4** | **What are some recommendations for non-pharmacological interventions for lower back pain? (With Safety Precautions and Evidence-Based Details)** |
| --- | --- |
|  | Based on current major international clinical guidelines (such as NICE, ACP, APTA, and Lancet reviews), the following are non-pharmacological intervention recommendations for low back pain (especially nonspecific low back pain), including specific details and safety checks.  Core Principles: Patient-centered, proactive, and based on the biopsychosocial model.  **I. Core First-Line Intervention Measures (Most Evidently Supported)**  1. Patient Education and Reassurance (Eliminating Fear)  Content:  - Explain the symptoms: Clearly state (after ruling out serious illnesses) that "lower back pain is very common and the vast majority of cases are not caused by serious illnesses," and that tissues can usually heal or adapt on their own.  - Relieve the "alarm": Emphasize that pain does not mean that the tissue is being damaged in the absence of "red flag" signs, and avoid excessive fear of pain.  - Encourage continued activity: Clearly inform the public that prolonged bed rest is harmful, potentially delaying recovery, causing muscle atrophy, and leading to fear-avoidant behavior.  - Management Expectations: This indicates that the recovery process may have ups and downs, but continuous improvement is the goal.  Executable Details: Provide verbal explanations in conjunction with written materials or links to reliable sources (such as patient education pages from authoritative medical institutions).  2. Exercise Therapy and Physical Activity  General Recommendation: Within tolerable pain levels, return to daily activities as soon as possible and maintain a regular exercise routine. Any form of exercise is better than no exercise.  Specific Plan (needs to be individualized, best started under the guidance of a professional):  - Acute phase (less than 6 weeks): Primarily engage in gentle activities, such as walking. Start with short distances and gradually increase.  - Subacute and chronic phases (more than 6 weeks): Perform structured exercise therapy, which is the cornerstone of chronic low back pain management.  - Core Muscle (Truncal) Stability Training/Motor Control Training: Focus on activating and training deep stabilizing muscles such as the transverse abdominis and multifidus. Examples include: Bird Dog Pose (alternating arm and leg raises in a four-point kneeling position), Plank (starting from a kneeling position), and a modified crunch. The principle is to perform the movements slowly and with control, avoiding holding your breath.  - Aerobic exercise: such as brisk walking, swimming, and stationary cycling, 150 minutes of moderate-intensity exercise per week to improve overall health and pain tolerance.  - Mind-body exercises: Pilates, Yoga, and Tai Chi have moderate-level evidence to support their effectiveness in improving chronic lower back pain, increasing flexibility, and relieving stress.  Safety and Implementation: The "no pain or minimal pain" principle. Mild muscle soreness from exercise is normal, but it should not cause severe pain or radiating pain. Start with low intensity and low volume, and gradually increase the intensity.  **II. Other Effective Non-Pharmacological Interventions (Select as Needed)**  1. Psychological Intervention (especially suitable for those with chronic low back pain or those with "yellow flag" factors)  - Cognitive Behavioral Therapy: Helps patients identify and change negative thoughts, fears, and avoidance behaviors related to pain, and establish positive coping strategies.  - Acceptance and Commitment Therapy: Helps patients continue to pursue the life values they cherish, even while experiencing pain.  - Execution Details: Can be performed by a registered psychotherapist, a partial physical therapist, or a validated digital CBT program.  2. Manual Therapy  Includes: Spinal manipulation/mobilization techniques, massage therapy.  Evidence:  - It can provide short-term pain relief and functional improvement for patients with acute low back pain.  - For chronic low back pain, it is often used as part of a combination therapy regimen (such as combining exercise therapy) rather than as a single long-term treatment.  Safety and Implementation: This procedure must be performed by a properly trained medical professional (such as an orthopedic surgeon, physical therapist, or chiropractor). A "red flag" sign screening should be conducted beforehand. Manipulation in the neck area carries a risk of vertebral artery injury and requires extreme caution.  3. Multidisciplinary Rehabilitation Program  - Suitable for: Patients with complex chronic low back pain, severely limited function, inability to work due to pain, and significant psychosocial factors.  - Content: A collaborative effort involving a team of doctors, physical therapists, psychotherapists, occupational therapists, and others, integrating physical, psychological, vocational, and social interventions.  4. Physical Factors and Tools (Auxiliary Role)  - Heat therapy: Effective for relieving muscle tension and post-acute pain. Use a heating pad or hot water bottle, wrapped in a towel, for 15-20 minutes each time, avoiding burns.  - Low-dose laser therapy and transcutaneous electrical nerve stimulation: Evidence is limited, or efficacy is inconsistent, and guidelines generally do not recommend them routinely. They may be tried in multimodal treatments.  - Supportive tools: Such as ergonomic support chairs and adjustable standing desks. Regular use of lumbar support belts/back braces is not recommended; use them only for short periods (e.g., a few days) during severe acute pain to prevent disuse of core muscles.  **III. Key Safety Precautions and Risk Mitigation**  **A. Pre-intervention Security Check (must be repeated)**  The "red flag" sign must be checked again before any non-pharmacological intervention begins or when pain changes. If it appears, stop immediately and seek medical attention.  **B. Safety Details for Specific Interventions**  1. Exercise Therapy:  - Contraindications: During periods of active disease, such as confirmed unstable fractures, acute infections, or tumors, regular exercise should be avoided, and medical advice should be followed.  - Warning signs: If you experience increased or worsened radiating pain, numbness, or weakness in your lower limbs during or after exercise, you should stop exercising and consult a therapist or doctor.  - Personalized: Patients with lumbar spinal stenosis are better suited to exercises that involve leaning forward (such as cycling) rather than stretching exercises that involve leaning backward.  2. Manual Therapy:  - Absolute contraindications: Presence or suspicion of cauda equina syndrome, spinal infection, tumor, fracture, severe osteoporosis, vertebral artery insufficiency, etc.  - Relative contraindications: Patients with bleeding disorders, taking anticoagulants, or who are pregnant should inform the operator.  - Adverse Reactions: Temporary local soreness, stiffness, or fatigue may occur after treatment, usually subsiding within 24-48 hours. If new neurological symptoms or severe pain occur, contact the operator immediately and seek medical attention.  3. Psychological Intervention:  - This must be conducted in a professional and confidential environment. Patients with severe mental illness must first be assessed and managed by a psychiatrist.  **C. Common Methods to Avoid or Use with Caution (Based on Guidelines)**  - Long-term passive treatment: If one relies on massage, electrotherapy, etc. for a long time without engaging in active exercise, it may lead to dependence and functional decline.  - Absolute bed rest (more than 1-2 days): has been shown to delay recovery.  - Routine imaging examinations (without a "red flag"): Abnormal findings on X-rays/CT/MRI are often unrelated to symptoms and may lead to unnecessary fear and invasive treatments.  - Complex interventions based on non-evidence-based diagnosis: Such as implementing expensive, long-term immobilization programs based solely on imaging diagnoses of "herniated disc" or "spinal instability" without functional assessment and exercise rehabilitation.  - Ineffective or weakly supported interventions: Such as traction and ultrasound therapy (for low back pain), these are generally not recommended by guidelines.  **IV. Executable Steps Flowchart (For Patient/Primary Healthcare Reference)**  1. Step 1: Safety Self-Check. Check for "red flag" signs. If present, seek immediate emergency care.  2. Step 2: Initiating Self-Management (No "Red Flags"). Maintain activity. Gentle exercise: Start with short walks each day. Short-term hot compress: Relieves muscle tension. Adjust your posture: Avoid prolonged sitting and change your posture regularly.  3. Step 3: Seek professional help (if the pain persists for more than 1-2 weeks or affects your daily life). Seeking medical attention: Consult a general practitioner, rehabilitation physician, or physical therapist. Obtain an individualized plan: Conduct a functional assessment to obtain a targeted exercise prescription (such as core training), and consider combining it with short-term manual therapy to relieve symptoms. Mental Health Considerations: If you experience worry, fear, or low mood, proactively talk to your doctor and seek psychological support or CBT.  4. Step 4: Persistence and Adjustment. Maintain active exercise: Treat it as a lifestyle habit, like brushing your teeth. Regular follow-up visits: Communicate with the therapist about progress and adjust the treatment plan. Managing Recurrence: Understand that back pain may recur, but mastering self-management skills can help you cope more easily.  **V. Evidence-Based Basis and Uncertainty Explanation**  - Strong evidence supports: Patient education, maintaining activity, and targeted exercise therapy (especially for chronic low back pain) are Grade A recommendations in the vast majority of international guidelines.  - Moderate evidence supports: Manual therapy provides short-term relief for acute low back pain; psychological intervention is effective for chronic low back pain with psychosocial factors.  - Limited evidence or mixed results: Acupuncture, dry needling, and most physical therapy methods (such as TENS and ultrasound) are not routinely recommended by guidelines.  - Uncertainty: There is no absolute consensus on the "best" type of exercise; the key is that the patient can stick to and enjoy the exercise. The plan must be individualized.  Final Reminder: This recommendation primarily addresses nonspecific low back pain. If diagnosed with specific low back pain (such as spinal stenosis, herniated disc with radicular symptoms), the treatment plan will need to be adjusted based on the specific pathology, but exercise rehabilitation will remain a core component. All interventions should be conducted under the guidance of a qualified medical professional. |

| **Question 5** | **What are some recommendations for medication interventions for lower back pain? (With Safety Precautions and Evidence-Based Details)** |
| --- | --- |
|  | Based on current major international clinical guidelines (such as NICE, ACP, and the European Spine Journal guidelines), the following are recommendations for a step-by-step approach to pharmacological interventions for nonspecific low back pain, including specific details, safety checks, and evidence-based support.  Pharmacological treatment should always be combined with non-pharmacological interventions (such as exercise and education) and is typically used when pain severely impacts function or quality of life.  Core Principles: Stepped Treatment, Individualized Approach, Short Treatment Course, Minimum Effective Dose.  **I. Drug Selection Ladder and Specific Recommendations**  First Tier: First-line Selection (for mild to moderate pain)  1. Topical Nonsteroidal Anti-inflammatory Drugs  - Examples: Diclofenac gel/patch, flurbiprofen gel patch.  - Evidence and Details: Recommended as a first-line treatment for both acute and chronic low back pain. Topical application results in minimal systemic absorption and significantly lower gastrointestinal and cardiovascular risks compared to oral formulations. Apply directly to the most painful area.  - Specifics: Use 2-4 times daily as directed. Treatment duration is typically no more than 2 weeks. Wash hands after use and avoid contact with eyes and mucous membranes.  2. Oral Acetaminophen  - Evidence and Details: Although recent reviews have suggested that its efficacy for lower back pain is limited, guidelines still often use it as one of the initial options due to its relatively good safety profile, especially for patients who cannot use NSAIDs.  - Execution Details: The usual adult dose is 500-1000 mg every 4-6 hours, with a total dose not exceeding 3000-4000 mg in 24 hours (follow your doctor's instructions or the package insert for specific dosage). Be aware of the risk of hepatotoxicity.  Second Tier: Second-line/Combined Selection (for moderate to severe pain, when first-line treatment is ineffective)  1. Oral Nonsteroidal Anti-inflammatory Drugs (NSAIDs)  - Examples: Ibuprofen, naproxen, diclofenac, celecoxib.  - Evidence and Details: It has a clear effect on relieving pain and improving function in acute low back pain, superior to acetaminophen. It is one of the core drug choices for acute cases.  - Executable Details: Short course of treatment: For use in the acute phase, generally no more than 5-7 days. If a longer course of treatment is required, a doctor's evaluation is necessary. Take with meals to reduce stomach irritation. Selection Principles: Individuals without cardiovascular risk may choose non-selective NSAIDs (such as ibuprofen); individuals with gastrointestinal risk may choose COX-2 inhibitors (such as celecoxib) or a combination of proton pump inhibitors (such as omeprazole).  2. Short-term Oral Muscle Relaxants  - Examples: Cyclobenzaprine, Tizanidine, Eperisone Hydrochloride.  - Evidence and Details: In the early stages of acute low back pain (less than 72 hours), combination therapy with NSAIDs may provide additional short-term (within 1 week) pain relief and muscle relaxation. It is ineffective for chronic low back pain, and sedation is a common side effect.  - Execution Details: Strictly limited to short-term use (3-7 days or less). Due to significant drowsiness, it is recommended to take it at bedtime. Driving or operating machinery is prohibited during the course of treatment.  Third Tier: Tertiary/Specialist Selection (for chronic low back pain, requires specialist evaluation)  1. Duloxetine  - Evidence and Details: An SNRI antidepressant. Recommended by guidelines in multiple countries for the treatment of chronic nonspecific low back pain, especially in patients with neuropathic pain components or comorbid depression/anxiety. Its analgesic effect is independent of its antidepressant effect.  - Execution Details: Prescription required. Usually starts with a low dose (e.g., 30 mg/day) and is slowly titrated to an effective dose (commonly 60 mg/day). Onset of action takes 2-4 weeks. Abrupt cessation is not permitted.  2. Other Anti-neuropathic Pain Medications  - Examples: Pregabalin, Gabapentin.  - Evidence and Details: Consider only when there is a clear indication of radicular pain (such as sciatica) or a clear neuropathic pain pattern. Its effectiveness for nonspecific low back pain is unclear and carries a high risk of side effects (drowsiness, dizziness, weight gain).  - Implementation Details: Not recommended as a first-line or routine treatment for nonspecific low back pain. Use should be prescribed by a pain specialist or neurologist after evaluation and should begin with a low titration.  Fourth Tier: Strictly Restricted Choices (High Risk, Requires Extreme Caution)  1. Opioids  - Examples: Tramadol, Hydrocodone, Oxycodone.  - Evidence and Details: Acute low back pain: Only considered for severe, acute pain that is unresponsive to other medications, and strictly limited to the shortest possible course of treatment (usually 3 days or less). Chronic low back pain: Due to a lack of long-term efficacy evidence and extremely high risks of addiction, overdose, tolerance, and endocrine disorders, most guidelines strongly oppose it as a treatment option for chronic low back pain.  - Implementation Details: Prescription must be prescribed by a physician after a thorough evaluation and discussion of the risks and benefits with the patient. A clear discontinuation plan must be established. Avoid concurrent use with benzodiazepines.  2. Epidural Steroid Injection  - Evidence and Details: Applicable only to patients with clear symptoms of nerve root compression (such as sciatica) who have not responded to conservative treatment. It may provide short-term (weeks to months) pain relief to help patients participate in rehabilitation training. Ineffective for non-radiating low back pain.  **II. Key Safety Precautions and Risk Mitigation**  **A. General Principles of Drug Safety**  1. Prioritize "Red Flag" Signs: Before administering medication, it is essential to reconfirm the absence of any "red flag" signs requiring immediate attention (cauda equina syndrome, infection, tumors, etc.). Medication should not mask the diagnosis of a serious illness.  2. Lowest effective dose, shortest treatment duration: The goal is to "control pain to enable activity and rehabilitation," rather than to eliminate pain.  3. Regular Assessment: After starting medication, the effects and side effects should be assessed regularly (e.g., every 1-2 weeks) to determine whether to continue, adjust, or discontinue the medication.  4. Be aware of interactions: Inform your doctor about all medications you are using (including traditional Chinese medicine and health supplements) to prevent interactions.  **B. Safety Warnings and Contraindications by Drug Category**  1. Acetaminophen: Risk of liver toxicity: Do not exceed 4000 mg daily. Patients with liver disease or who drink 3 or more drinks daily should use a lower dose or avoid this medication altogether. Avoid alcohol consumption while taking this medication.  2. Nonsteroidal Anti-inflammatory Drugs:  - Gastrointestinal Risks: May cause ulcers and bleeding. Contraindications: Active peptic ulcer/bleeding. High-risk groups (elderly, those with a history of ulcers, and those using anticoagulants/hormones) require the addition of a gastric protectant (such as a PPI).  - Cardiovascular Risks: May increase the risk of myocardial infarction and stroke. Contraindications: Severe heart failure, perioperative period after coronary artery bypass grafting. Use with caution in patients with a history of cardiovascular disease.  - Risk of kidney injury: May cause acute kidney injury, especially in cases of dehydration, chronic kidney disease, or concurrent use of diuretics or ACE inhibitors.  - Selectivity: Celecoxib (a COX-2 inhibitor) has a lower gastrointestinal risk, but its cardiovascular risk needs to be monitored.  3. Muscle Relaxants: Central nervous system depression: May cause severe drowsiness and dizziness. Driving and drinking alcohol are prohibited while taking this medication. Dependency: This product has the potential to cause dependence; use beyond the prescribed course of treatment is strictly prohibited.  4. Duloxetine: Withdrawal syndrome: Do not stop abruptly; gradually reduce the dosage over several weeks. Other: May cause nausea, dry mouth, and excessive sweating; initial adaptation is required. Use with caution in individuals with a history of glaucoma or liver disease.  5. Opioids: Addiction and Abuse: Risks exist even with short-term use. Tolerance and Hyperalgesia: Long-term use may require increased dosage and may lead to increased pain sensitivity. Respiratory depression: Overdose can be fatal, especially when used in combination with alcohol or sedatives. Constipation: Almost inevitable, requires preventative use of laxatives.  **C. Common Drugs/Methods That Should Be Avoided or for Which There Is Very Weak Evidence**  - Systemic glucocorticoids (oral or intravenous): Their effectiveness for acute sciatica is unclear, they have significant side effects, and are not routinely recommended.  - Combined acetaminophen and opioid preparations: Because they contain opioids, they also pose a risk of addiction and can easily lead to acetaminophen overdose.  - Benzodiazepines (such as diazepam): There is no evidence to support their effectiveness for lower back pain. They have a high risk of dependence and many side effects, and should be avoided.  - Most dietary supplements (such as glucosamine and chondroitin): are ineffective for lower back pain.  **III. Executable Tiered Management Flowchart**  1. Step One: Assessment and Education. Exclude the "red flag" sign. Confirm nonspecific lower back pain. Initiate core non-pharmacological therapies (education, maintaining activity).  2. Step Two: First-line drug intervention (acute phase or mild to moderate pain). Preferred choice: Topical NSAIDs (such as diclofenac gel). Alternative/Combination: Oral acetaminophen (note the upper limit of dosage). Assessment takes 3-5 days.  3. Step Three: Second-line drug intervention (pain persists or is severe). Add or switch to short-course oral NSAIDs (7 days or less). If muscle spasms are significant and in the acute phase, a muscle relaxant can be used for a very short period (3 nights or less) before bedtime.  4. Step Four: Chronic Pain Management (Pain more than 12 Weeks). Emphasize the central role of non-pharmacological methods (exercise therapy, psychological intervention). If medication is needed, duloxetine may be considered (prescription required). Strictly avoid routine use of opioids.  5. Step Five: Specialist Referral. If accompanied by persistent radicular symptoms, epidural injection may be considered for evaluation. Complex cases are referred to the pain management department or multidisciplinary rehabilitation team.  **IV. Evidence-Based Basis and Uncertainty Explanation**  - Strong evidence supports: Topical/oral NSAIDs are effective for acute low back pain; duloxetine is effective for chronic low back pain.  - Supporting evidence but with clear limitations: Muscle relaxants are effective for acute low back pain in the short term, but side effects limit their use.  - Evidence against: Opioids are ineffective and carry extremely high risks with long-term use for chronic low back pain; benzodiazepines are ineffective.  - Uncertainties/Controversies: The efficacy of acetaminophen has been questioned in recent years by high-quality reviews, but its safety (within strict dosage limits) keeps it in some guidelines.  Final Reminder: This recommendation is a general framework. Specific medication selection should be individualized by a physician based on the patient's age, comorbidities (stomach, heart, kidney, liver), allergy history, concomitant medications, pregnancy status, and specific characteristics of pain. Patients should not prescribe or adjust their own medications. |

| **Question 7** | **What are the appropriate criteria for home rehabilitation of lower back pain? (With Safety Precautions and Evidence-Based Details)** |
| --- | --- |
|  | Based on mainstream international clinical guidelines (such as NICE, ACP, APTA, etc.), the "appropriate criteria" for home-based rehabilitation of low back pain are not a single indicator, but a clinical decision-making process based on safety screening, risk stratification, and functional assessment.  **I. Core Appropriate Standards for Home-Based Rehabilitation**  Individuals suitable for home-based rehabilitation should meet all of the following conditions, which can be summarized as "three no's" and "three yes's."  **A. "Three No's" - Absolutely Safe Prerequisite (Excluding Contraindications)**  1. No signs of a "red flag" appearing:  This includes: signs of cauda equina syndrome (saddle area numbness, bowel and bladder dysfunction), signs of infection/tumor (fever, unexplained weight loss, severe nocturnal pain), and risk of severe post-traumatic fractures. This is the primary and essential condition to meet.  2. Non-progressive neurological deficits:  No new or progressively worsening lower limb muscle weakness (such as foot drop, knee buckling when walking), sensory loss, or abnormal reflexes.  3. Severe specific pathologies that do not require urgent or specialist intervention:  For example, mild to moderate lumbar spinal stenosis or old disc herniation (non-acute phase) that has been diagnosed but is stable can be treated with home rehabilitation under the guidance of a doctor. However, if symptoms severely affect walking ability (e.g., neurogenic claudication distance less than 100 meters), a professional evaluation is required first.  **B. "Three Essentials" - Prerequisites for Function and Capability**  1. Possesses basic self-management skills and good compliance:  - Able to understand rehabilitation instructions and committed to maintaining activity and regular exercise within tolerable limits.  - It can recognize warning signals and know when to stop and seek help.  2. A suitable home environment and start-up instructions are available:  - Environmental safety (non-slip floor, sufficient space for activities).  - Key Points: Even when working at home, the rehabilitation program (especially the initial exercises, intensity, and frequency) should ideally originate from a professional medical assessment (such as one provided by a doctor or physical therapist) or follow a highly evidence-based standardized program.  3. Relatively controllable pain level:  Pain scores (0-10) are usually below 7 and can be partially relieved by rest, adjusting posture, or simple self-treatment (such as applying heat), without affecting basic daily activities (such as washing, walking short distances).  **II. Specific Classification and Suitability Judgment**  - Acute nonspecific low back pain (less than 6 weeks): Highly suitable. Guideline-recommended core management scenario. Home self-management (education, gentle activity, short-term medication) should begin immediately after ruling out the "red flag" syndrome.  - Subacute nonspecific low back pain (6-12 weeks): Suitable if conditions permit. If the pain becomes chronic, it is strongly recommended to undergo at least one professional evaluation before starting home rehabilitation to obtain an individualized exercise prescription and avoid solidifying incorrect patterns.  - Chronic nonspecific low back pain (more than 12 weeks): Suitable if available. Home-based rehabilitation is the cornerstone of its long-term management, but it must be based on a professionally developed, gradual plan. It often requires integration of pain cognitive management (psychological techniques).  - Accompanied by radicular symptoms (sciatica): Requires careful evaluation before proceeding. Suitable for: Symptoms are stable or improving (no worsening of numbness or pain), and muscle strength is normal. Unsuitable for: Symptoms progressively worsen, and muscle strength declines.  - A clearly diagnosed specific disease (such as stable spinal stenosis): Must be performed under specialist guidance. Rehabilitation plans must be highly individualized (e.g., flexion/cycling exercises are more suitable for patients with spinal stenosis than extension exercises).  **III. Safety Precautions and "Stop" Warning Signals**  **A. Pre-Initiation Safety Checklist (Executor/Patient must confirm each item)**  1. I have reviewed and verified all signs of "red flag" and none of them exist.  2. My pain and symptoms have not progressively worsened in the past 24-48 hours.  3. I did not experience any new weakness or worsening of instability in my ankles or toes.  4. I have consulted a doctor or therapist, or my condition fully meets the characteristics of "acute nonspecific low back pain."  5. I understand that the core of home rehabilitation is active exercise, rather than absolute rest.  **B. "Stop Signals" That Require Immediate Stop and Medical Attention During Rehabilitation**  1. Change in pain: The pain suddenly becomes unusually intense, or changes from a dull ache to a sharp, stabbing, or electric shock-like pain.  2. The onset or worsening of neurological symptoms: Numbness or weakness in the lower limbs (especially on one side) increases in area or worsens in severity. The appearance of "foot drop" (i.e., a significant weakening of the upward flexion of the ankle and toes) that was not present before.  3. Functional Loss: Difficulty urinating or defecating, incontinence, or numbness in the perineum (saddle area). (Emergency Indications)  4. Systemic symptoms: Unexplained fever, chills, or pain that is significantly aggravated at rest at night.  5. Abnormal reaction after exercise: After a certain exercise, pain or discomfort persistently worsens for more than 2 hours, or causes radiating pain down to the lower limbs.  **C. Safety Details for Home-Based Rehabilitation Implementation**  1. Follow the "no pain or minimal pain" principle: Any discomfort during or after exercise should return to baseline levels within 24 hours. Do not exercise in severe pain.  2. Progress Gradually: Start with low intensity and low volume (e.g., 5 minutes of walking, 5 repetitions of core exercises per set), and gradually increase. Avoid "overcompensation" when you feel good.  3. Focus on quality of movement rather than quantity: Core stability training emphasizes slow, controlled movements and proper breathing (exhale when exerting force, avoid holding your breath).  4. Environmental Safety: Ensure the exercise area is dry and free of obstructions; use a yoga mat if necessary. Chairs should be sturdy and at an appropriate height.  **IV. Specific and Actionable Steps to Initiate Home-Based Rehabilitation**  1. Step 1: Education and Mindset Building (Day 1). Read authoritative educational materials on lower back pain, understand that "pain is not equal to injury," and build confidence that "maintaining activity is beneficial."  2. Step 2: Gentle Start-up (Days 1-3). Activity: Get up and move around for 2-3 minutes every hour, doing gentle walking (such as walking back and forth indoors). Hot compress: Apply a hot compress to the painful area for 15-20 minutes, 2-3 times daily, to relieve muscle tension. Posture Adjustment: Avoid prolonged sitting or standing; use a lumbar support.  3. Step 3: Introduce Basic Exercises (Days 3-7, after pain begins to subside). Continue walking: Gradually increase to 10-15 minutes each time, twice a day. Basic Core Activation: Transverse abdominis activation - Lie on your back with your knees bent, slowly contract your lower abdomen (imagine gently pulling your navel towards your spine), hold for 5-10 seconds, then relax. Repeat 10 times. Cat-Cow Pose: Kneel on all fours, slowly and alternately arching and lowering your back to move your spine, repeat 10 times. Bird Dog Pose (Basic Version): Kneel on all fours, slowly and alternately extend the opposite arm and leg, keeping your body balanced and still, 5 times on each side.  4. Step 4: Forming Patterns and Advancement (1-2 weeks later). Regularize the above activities and consider seeking guidance from a physical therapist to learn more individualized advanced exercises.  **V. Evidence-Based Basis and Uncertainty Explanation**  - Strong evidence: For acute nonspecific low back pain without warning signs, home self-management centered on education and encouragement activities is the first-line standard of care (strongly recommended by the NICE guidelines).  - Individual Differences: The pain threshold (e.g., 7 points) and the definition of "mild" neurological symptoms in the "appropriate criteria" are subjective. When any uncertainty exists, an initial assessment by a healthcare professional should be prioritized.  - Effectiveness Prerequisites: The success of home-based rehabilitation highly depends on the patient's self-efficacy and compliance. For patients lacking confidence in self-management or harboring fear or avoidance beliefs, even without contraindications, the results may be poor, requiring further professional support.  Summary Flowchart: Start - Strictly check for "red flags" and "stop signals" - If present, seek medical attention immediately - If absent, determine if the "three conditions" are met - If not, seek professional evaluation - If met, begin home rehabilitation according to the "starting steps" - Continuously monitor warning signals during the process - If there is no improvement or worsening after 2-4 weeks, refer to professional evaluation. |

| **Question 12** | **What are the recommended treatment goals for patients with subacute and chronic low back pain? (With Safety Precautions and Evidence-Based Details)** |
| --- | --- |
|  | For patients with subacute and chronic low back pain, the treatment goals of modern evidence-based medicine guidelines (such as NICE, ACP, and the European Spine Journal guidelines) have undergone a fundamental shift: from focusing on "pain relief" to focusing on "functional restoration, improved quality of life, and self-management."  **I. Core Treatment Goal: Paradigm Shift**  Overall Goal: To help patients, while coexisting with pain, maximize their ability to participate in work, family life, social activities, and recreation, and reduce the physical and mental burden of pain.  Key Goal During the Subacute Phase (6-12 weeks of illness): To halt the progression to chronicity  This period is the golden intervention window for preventing acute low back pain from becoming chronic. The goal has a dual nature:  1. Functional Recovery: Gradually resume normal daily activities and work.  2. Risk Factor Management: Identify and intervene in "yellow flag" factors (psychosocial risk factors) that lead to chronicity, such as fear-avoidant behavior, negative cognition, and job dissatisfaction.  Key Goals in the Chronic Phase (Disease Duration more than 12 Weeks): Long-Term Functional Optimization and Self-Efficacy Building  Pain itself can become a long-term condition, and the goal is to manage rather than cure:  1. Functional Reconstruction and Maintenance: Reconstructing physical functions, social roles, and activities lost or reduced due to pain.  2. Self-efficacy enhancement: Enables patients to become "experts" in their own pain management, mastering self-management skills and gaining confidence.  3. Reduce pain-related disorders: Reduce the negative impact of pain on mood, sleep, and overall well-being.  **II. Specific and Actionable Recommended Treatment Goals (SMART Principle Guidance)**  Target Domain 1: Physical Function and Activity Participation  - Job: "Within 4 weeks, be able to return from part-time work to full-time office work and master the coping strategy of getting up and moving around for 2 minutes every 30 minutes of sitting."  - Daily Life: "Within two weeks, I will be able to independently complete a 20-minute supermarket shopping trip, including carrying 5 kilograms of weight home."  - Specific Activity: "Under the guidance of a physical therapist, I was able to resume swimming three times a week for 30 minutes each time within 8 weeks without fear."  - Implementation Details: Use activity diaries and functional assessment scales (such as the Roland-Maurice Disability Questionnaire) as a baseline to work with the patient to set progressive, measurable activity goals.  Target Domain 2: Pain Cognition and Behavioral Modification  - Reducing Fear and Avoidance Beliefs: "Through cognitive behavioral therapy, after 6 sessions, the patient's belief score on 'activity leads to injury' (such as the TSK scale) decreased from high to moderate."  - Establishing healthy coping behaviors: "When the pain score reaches 5/10, patients can proactively use deep breathing relaxation techniques or perform gentle stretching instead of immediately taking painkillers or resting in bed."  - Implementation Details: This will be achieved through psychoeducation, cognitive restructuring, and behavioral experiments (such as trying "terrible" activities within safe limits to verify their consequences).  Target Domain 3: Athletic Ability and Physical Fitness  - Core Stability: "Within 6 weeks, you will be able to correctly and painlessly complete 3 sets of 10 repetitions of the 'bird dog' exercise and apply this stability in your daily life."  - Aerobic Endurance: "Increase your walking endurance from 10 minutes to 25 minutes per session, 5 times a week, over 4 weeks."  - Flexibility: "Stretch your hamstrings and hip flexors daily to achieve a 2-centimeter improvement in the sit-and-reach test after 2 weeks."  - Implementation Details: An individualized, graded (gradual) exercise prescription will be developed by a physical therapist and adjusted regularly.  Target Domain 4: Rationalization of Drug and Medical Resource Use  - Reduce dependence on acute-phase medications: "Within 4 weeks, reduce the frequency of NSAID use from twice daily to no more than twice weekly."  - Avoid unnecessary medical contact: "Educate patients to understand that pain fluctuations are normal, intending to reduce the number of follow-up/emergency visits for nonspecific low back pain by 50% within the next 6 months."  - Implementation details: Establish a clear medication tapering plan and teach non-pharmacological analgesia strategies as alternatives.  **III. Core Safety Considerations (Throughout the Process)**  **A. Continuous Safety Screening (even in the chronic phase)**  Principle: "Chronic" does not equal "safe." Patients must be repeatedly educated to recognize and report new "red flag" signs. During each follow-up visit or self-assessment, you should quickly check: "Has the pain pattern undergone a fundamental change? (e.g., turning into persistent nocturnal pain)?" and "Have any new neurological symptoms appeared? (e.g., new foot drop, saddle area numbness)?"  **B. Risk Factor Identification and Intervention ("Yellow Flag" Management)**  Objective: Proactively screening and managing psychosocial factors is key to preventing chronicity and successful treatment.  Specific examples of "yellow flags" and intervention directions:  1. Negative cognitions and emotions: "My back is ruined, and it will never be cured." - Goal: To correct catastrophic thinking through cognitive behavioral therapy.  2. Fear-Avoidance Behavior: Avoiding all bending over and lifting activities due to fear of pain. - Goal: Implement graded exposure therapy, gradually reintroducing the activity in a safe environment.  3. Unrealistic expectations of recovery: Pursuing a "complete cure" and "complete painlessness." - Goal: Provide realistic education to establish reasonable expectations of "living with pain while improving function."  4. Workplace Issues: Lack of support, low job satisfaction. - Goal: Provide career counseling or communicate with the employer for adjustments if necessary.  **C. Safety Principles for Advanced Exercise and Activity**  - The "painless or minimally painful" principle: Mild discomfort caused by activity or exercise should return to baseline within 24 hours. It should not be done while in pain.  - "Graded Exposure" and "Starting Line" Principles: Start with an intensity/amount far below what triggers fear or severe pain (e.g., start with a 5-minute walk), and gradually increase the intensity as you adapt.  - Be alert for abnormal reactions: If an activity causes pain to radiate to the distal lower extremities, worsening numbness or weakness, or symptoms to persist for more than 2 hours, you should return to the previous stage and consult a therapist.  **IV. Integration of Evidence-Based Treatment Pathways and Goals**  1. Initial/Review Stage:  - Action: Eliminate "red flags." Assess functional limitations. Screen for "yellow flags" (using short questionnaires or structured consultations). Work with the patient to set 1-2 of the most pressing short-term functional goals.  - Goal: To establish a treatment alliance and complete the cognitive shift from "curing pain" to "managing function."  2. Proactive Intervention Phase (lasting several weeks to several months):  - Action: Prescribe and supervise individualized exercise therapy (core stability, aerobic exercise). Provide pain science education, explaining the non-linear relationship between pain and tissue damage. Introduce cognitive-behavioral strategies to cope with fear and negative thoughts. Review progress toward goals regularly (e.g., every 2 weeks) and make adjustments together.  - Objective: To achieve specific functional improvements and observe improvements in the "yellow flag" factor.  3. Self-Management Maintenance Stage (Long-Term):  - Action: Gradually transfer the initiative in rehabilitation to the patient. Develop a "relapse prevention plan": Identify early warning signs (such as high stress, reduced activity) and develop coping steps (such as restarting core exercises, adjusting pace). Schedule follow-up visits at longer intervals.  - Goal: Patients gain self-management skills and confidence, and the use of medical resources is reduced to a reasonable level.  **V. Evidence-Based Basis and Uncertainty Explanation**  - Strong evidence supports: Function-oriented treatment goals, multimodal rehabilitation (exercise + psychological), and intervention for the "yellow flag" are Grade A recommendations for the management of chronic low back pain.  - Goal setting tools: Using tools like the Goal Achievement Scale to quantify functional progress is supported by research.  - Uncertainty/Individualization: There is no single, universally applicable best exercise program (e.g., yoga vs. Pilates vs. strength training). Key factors are patient preference and long-term adherence. Treatment goals must be collaboratively developed by both doctor and patient to ensure relevance and feasibility.  - Role of medication: In the chronic phase, medications (such as duloxetine) are used only as adjunctive therapy to help patients better participate in recovery, rather than as the primary treatment goal.  Summary: For patients with subacute and chronic low back pain, the sign of success is not a pain score dropping to zero, but rather "Despite the pain, I can live and work normally." The core treatment process is: Ensuring safety - Setting functional goals - Achieving goals through proactive intervention (exercise + psychological support) - Empowering patients with self-management. |

| **Question 21** | **For patients with acute or subacute low back pain, which nonpharmacological treatments should clinicians prioritize, and why are these treatments highly recommended? (With Safety Precautions and Evidence-Based Details)** |
| --- | --- |
|  | Based on mainstream international clinical guidelines (especially the NICE guidelines, the American College of Physicians guidelines, and the Lancet low back pain series of reviews), for patients with acute (less than 6 weeks) and subacute (6-12 weeks) low back pain, the non-pharmacological treatments that clinicians should prioritize, along with the strong reasons for their recommendation and safety details, are as follows.  Core Security Considerations (First Steps):  Before considering any treatment, a clinical safety screening must be completed:  1. Systematic screening for "red flag" signs: including but not limited to signs of cauda equina syndrome (saddle area numbness, bowel and bladder dysfunction), infection, tumors, fractures, etc. If present, immediately transfer to the appropriate emergency or specialist treatment pathway.  2. Assess "Yellow Flag" Factors: Conduct a preliminary assessment of psychosocial risk factors (such as catastrophic fear of pain, job dissatisfaction, and depressed mood). These factors significantly impact prognosis and are important criteria for selecting the intensity of intervention.  3. Explaining the "Nonspecific" Diagnosis: After ruling out serious diseases, the meaning of "nonspecific low back pain" should be clearly explained to the patient, that is, the pain is not caused by a serious disease, and most cases can heal on their own or be improved through self-management.  **I. Two Strongly Recommended Non-Drug Treatments and Reasons**  The following two interventions have been rated as highest level of recommendation (strong recommendation) by top guidelines from multiple countries and should be used as core frontline strategies.  1. Patient Education and Fear Relief  Specific content:  - Reassurance: Clearly informs you that "your back pain is common, no serious illness has been found, and it usually improves within a few weeks."  - Pain Neuroscience Education: Explaining in layman's terms that "pain is not always directly proportional to the degree of tissue damage," that acute pain is a protective signal, but that excessive fear and avoidance of activity may prolong the pain.  - Set positive expectations: Emphasize that "staying active is crucial for recovery, and bed rest is often harmful."  Why it's highly recommended:  - High-quality evidence: Multiple randomized controlled trials and meta-analyses have shown that targeted education can effectively reduce pain fear, improve function, and shorten sick leave.  - Core Mechanism: It directly targets the key risk factor for the chronicity of acute low back pain - fear and avoidance beliefs. Changing cognition can fundamentally change behavior (from bedridden to active).  - Extremely cost-effective: No special equipment is required; it can be completed in a short time within the examination room and forms the basis for all subsequent treatments.  2. Encourage Continued Activity and Gradual Resumption of Exercise  Specific suggestions:  - Explicitly oppose bed rest: It is recommended that even during periods of pain, one should maintain daily activities (such as walking or light housework) as much as possible.  - Provide a specific activity prescription: "Start with short, slow walks, such as 2-3 times a day for 5-10 minutes each time, and gradually increase the duration according to your tolerance."  - Posture Adjustment Guidance: Avoid prolonged sitting, and it is recommended to change your posture regularly, but there is no need to pursue an "absolutely correct" posture.  Why it's highly recommended:  - Overwhelming Evidence: A large body of high-level evidence (including the Cochrane review) consistently demonstrates that, compared to recommending bed rest, recommending activity leads to faster pain relief, faster functional recovery, less progression to chronicity, and less work absence.  - Physiological basis: Gentle activity promotes blood circulation, maintains muscle strength and spinal segment stability, prevents disuse atrophy, and helps the brain establish safe movement patterns.  - Preventing chronicity: This is the most effective behavioral intervention to prevent the acute phase from developing into the chronic phase.  **II. Specific and Feasible Clinical Procedures**  Core interventions that can be completed during the initial consultation (5-10 minutes):  1. Safety Notice: "The inspection showed no serious problems, which allows us to focus on restoring functionality with peace of mind."  2. Provide key educational information: "Pain does not mean you are 'adding insult to injury.' Moving within safe limits is part of the recovery process. The goal is to manage pain, not to eliminate it. Some discomfort is normal during the recovery process."  3. Provide clear and actionable activity suggestions: "Starting today, try to get up and move around for 2 minutes every 30 minutes of sitting." "Starting tomorrow, add two 5-minute walks each day. If that feels okay, increase it to 10 minutes each time next week."  4. Management Expectations: "The recovery process may have ups and downs, but the overall trend is upward. If there is no significant improvement after 2-4 weeks, we will have a follow-up appointment to adjust the plan."  Intensive Intervention for Patients with "Yellow Flag" Factors or in the Subacute Phase:  - Action: Consider early referral to a physical therapist.  - Reasons and Details: Physical therapists can provide: Graded Exercise Prescriptions (instruct safe, progressive, individualized exercises such as beginner-level core stability training movements), Manual therapy (optional, as a short-term adjunct to symptom relief, e.g., no more than 2-4 sessions, to help patients begin activities more easily; note: Manual therapy is not recommended as a long-term or monotherapy approach), More in-depth behavioral guidance on-site for fear avoidance.  **III. Common Measures to Avoid or Weakly Recommend (Based on Evidence)**  1. Routine imaging examinations (X-ray, MRI): Reason: In the absence of "red flag" signs, degeneration found on imaging (such as disc herniation and bone hyperplasia) is often not directly related to the symptoms and may increase patient fear and lead to overtreatment. Recommendation: Only consider when a specific pathology is suspected or symptoms persist and are unresponsive to treatment.  2. Primarily passive physical therapy: Reason: Long-term reliance on passive therapies such as electrotherapy, ultrasound, and traction, coupled with a lack of active movement, is detrimental to functional recovery and the establishment of self-efficacy. Recommendation: This treatment should only be used as an adjunct to relieve severe pain during the first few days of the acute phase; a rapid transition to active treatment is essential.  3. Prescription of strict bed rest: Reason: Evidence clearly shows that it is harmful and not beneficial. Recommendation: Absolutely avoid.  **IV. Uncertainty and Clinical Decision Points Explained**  - The Role of Manual Therapy: While guidelines permit it as a short-term symptom relief option, its effectiveness is controversial and highly dependent on the operator's skill. The consensus is that it should not replace education and exercise, but rather serve as a bridge to encourage patient participation in active therapy.  - Optimal type of exercise: For the acute phase, any well-tolerated aerobic activity (such as walking) is beneficial. For the subacute phase, there is stronger evidence to begin introducing individualized core stability training, but there is no universally accepted definition of the "optimal" approach.  - Intervention Threshold for the "Yellow Flag": There is no precise standard for how to accurately identify and when to implement intensive psychological interventions (such as formal CBT). The clinician's experiential judgment and the patient's wishes are crucial.  Summary Flowchart: When receiving patients with acute/subacute low back pain: 1. Check for the "red flag" (lower back pain marker) - 2. Assess the "yellow flag" (lower back pain marker) - 3. Inform the patient of the "nonspecific" diagnosis - 4. Strongly recommend and implement "educational and encouragement activities" - 5. For complex cases (significant "yellow flag" but no improvement in the subacute phase), refer the patient to physical therapy early - 6. Avoid ineffective measures. |

| **Question 23** | **Why is it not recommended to use imaging studies (such as X-rays, CT scans, or MRIs) as a routine method for diagnosing chronic nonspecific low back pain unless a specific cause is suspected? (With Safety Precautions and Evidence-Based Details)** |
| --- | --- |
|  | This question touches upon a core evidence-based principle in modern low back pain diagnosis and treatment. For chronic nonspecific low back pain, the routine use of imaging examinations (X-ray, CT, MRI) is not recommended, which is a strong recommendation of mainstream international clinical guidelines (such as NICE, ACP, and the European Spine Society).  **I. Core Reason: Why Are Routine Imaging Examinations Not Recommended?**  1. Evidence-based medicine: Imaging findings have an extremely weak correlation with clinical symptoms  Key Fact: In the general population who are asymptomatic, radiographic "abnormalities" are extremely common and become almost the norm with age.  - Intervertebral disc degeneration/protrusion: In asymptomatic individuals aged 30-50, the proportion of intervertebral disc herniation detected by MRI is as high as 30-40%; in individuals over 60 years of age, more than 90% have intervertebral disc degeneration or bulging.  - Osteophyte formation (bone hyperplasia) and facet joint degeneration: are also very common in asymptomatic elderly people.  Core Inference: For patients with chronic nonspecific low back pain, any "abnormalities" found on imaging are most likely age-related, asymptomatic, incidental findings, rather than the exact cause of the pain. Directly linking them to pain symptoms is a logical fallacy (correlation does not equal causation).  2. Clinical Risks: The "Harm" of Routine Imaging Outweighs the Benefits  - Triggering or exacerbating harmful beliefs and fear-avoidance behaviors: When patients are told they have a herniated disc, bone spurs, or spinal stenosis, even if these are common age-related changes, they may be misinterpreted as "spinal structural damage" or "fragility," leading to a fear of activity ("moving more will make it worse"), thereby exacerbating functional disability and chronic pain. This is the most concerning psychological harm.  - Leading to Unnecessary Invasive Treatments: Based on incidental imaging findings, both patients and physicians may be inclined to pursue more aggressive treatments, such as surgery or epidural injections. However, numerous studies have confirmed that for nonspecific low back pain without specific neurological signs, surgery is not superior to high-quality non-surgical treatments (such as structured motor rehabilitation) and carries associated risks.  - Increased healthcare costs and radiation exposure: Unnecessary examinations waste healthcare resources. X-rays and CT scans also introduce unnecessary ionizing radiation.  3. No benefit to treatment decision-making: Imaging does not change the first-line treatment plan  Guideline consensus: Regardless of imaging findings, the first-line basic treatment for chronic nonspecific low back pain is the same: comprehensive rehabilitation based on a biopsychosocial model, including: patient education and fear relief; active rehabilitation centered on exercise therapy (such as core stability and aerobic exercise); psychological intervention (such as cognitive behavioral therapy) may be necessary when required. Imaging studies do not provide additional guidance for these core treatments. On the contrary, they may mislead both doctors and patients away from these evidence-based, proactive rehabilitation pathways.  **II. Safety Precautions: When Should Imaging Examinations Be Used?**  Imaging examinations have specific applications, primarily used to exclude or confirm "specific low back pain."  A. Indications for mandatory imaging examinations (presence of a "red flag" or suspected specific cause):  1. Suspected serious underlying disease: Medical history or physical examination findings suggest tumor, infection, or fracture (such as unexplained weight loss, fever, immunosuppression, severe trauma, severe pain after minor trauma in elderly people with osteoporosis, or night pain). Preferred examination: MRI (most sensitive to soft tissue and bone marrow lesions) or CT (best for showing fracture details).  2. Progressive or severe neurological deficits: New or worsening of lower limb muscle weakness (such as foot drop, knee buckling). Symptoms consistent with cauda equina syndrome (saddle area numbness, bowel and bladder dysfunction) - this is an absolute indication for emergency MRI.  3. As a necessary assessment before invasive treatment: Before considering invasive treatments such as spinal surgery, epidural injection, or nerve block, imaging studies must be performed to clarify anatomical structures, locate lesions, and corroborate clinical symptoms.  4. If symptoms do not improve or even worsen after a period of regular non-surgical treatment: General guidelines usually recommend that if symptoms are still severe and affect daily life after at least 6 weeks of high-quality, well-behaved non-surgical treatment, an MRI may be considered to reassess whether there are previously unidentified specific causes.  **III. Specific and Actionable Clinical Decision-Making Pathways**  1. Step 1: Comprehensive Clinical Assessment (This is the most important "diagnostic tool")  - Detailed medical history: Understand the pain pattern, influencing factors, degree of functional limitation, and systematically screen for all "red flag" signs.  - A thorough physical examination: This includes a neurological examination (muscle strength, sensation, reflexes), spinal mobility assessment, and specific provocation tests. The purpose of the physical examination is to assess functional and neurological status, rather than simply looking for "structural damage."  - Assess "Yellow Flag" Factors: Identify psychosocial factors such as fear and avoidance beliefs, depression and anxiety, and work-related stress.  2. Step 2: Making a Preliminary Clinical Diagnosis  - If there is no "red flag" sign and the pain is not significantly related to neurological examination, the diagnosis should be "chronic nonspecific low back pain."  - Clearly explain to the patient: "Your pain is real, but the current assessment has not revealed any serious problems requiring urgent treatment, such as infection, tumors, or nerve compression requiring surgery. This situation is very common, and our treatment focuses on helping you restore function and reduce the impact of pain on your life."  3. Step 3: Initiating First-Line Treatment and Setting Expectations  - Prescription-based proactive rehabilitation program.  - Clearly stated: "Based on current international guidelines and extensive research evidence, taking imaging (X-ray/MRI) at this stage generally will not change our core treatment plan and may instead cause unnecessary concern. We recommend starting active rehabilitation training first."  4. Step 4: Define the Referral Criteria for Imaging Examinations  - It is agreed with the patient that if the above-mentioned new "red flag" signs appear, or if the function shows no improvement or even deteriorates after 6-12 weeks of standard non-surgical treatment, a reassessment and imaging examination will be conducted.  **IV. Explanation of Uncertainties and Special Circumstances**  - Uncertainty: Currently, there is no precise "pain atlas" that can completely correlate specific imaging findings with a patient's pain experience. Pain is the result of a complex interplay of neurological, psychological, and social factors.  - Challenges in Doctor-Patient Communication: Patients often feel confused about the lack of examinations or believe that doctors don't take them seriously. This requires doctors to spend more time explaining and educating patients, building trust.  - Special Populations: For patients with a history of cancer, severe immunosuppression, or unexplained systemic symptoms, the threshold for imaging examinations should be significantly lowered.  Summary: Routine imaging for chronic nonspecific low back pain is not recommended based on solid evidence (most findings are irrelevant), clear clinical risks (leading to fear and overtreatment), and efficient treatment logic (it does not affect first-line treatment). Clinical decisions should always be patient-centered and based on thorough clinical evaluation, not incidental imaging findings. Reserving imaging for patients who truly need it to rule out serious diseases or plan invasive treatments is the most responsible and safest way to use medical resources. |
