# Supplementary material for: Deficiencies in clinical reasoning of LLMs in low back pain management and remediation via prompt engineering: from performance evaluation to error diagnosis
Source: Front Artif Intell. 2026 May 25;9:1811701. doi: 10.3389/frai.2026.1811701 (PMC13243384; doi:10.3389/frai.2026.1811701)
Supplement: Supplementary file 1 [file Data_Sheet_1.zip › 补充材料/Supplementary materials 5/GPT-4o/A(GPT-4o).docx]

| **Question 1** | **How to define and classify lower back pain?** |
| --- | --- |
|  | 1. Definition  Low back pain refers to pain located in the area between the lower edge of the 12th rib and the gluteal fold, which may or may not be accompanied by radiating pain in the lower limbs. It does not refer to a specific cause and can be acute, subacute, or chronic.  2. Classification Method  The classification of low back pain is mainly based on three aspects: etiology, duration of symptoms, and clinical manifestations.  **(a) Classification by etiology (most common)**  1. Nonspecific low back pain (LBP) — accounts for 85%-90%. Imaging or physical examinations fail to identify a clear structural cause. Typical symptoms include muscle, ligament, and fascia strain, and poor posture.  2. Specific LBP — There is a clear cause, and structural damage is visible: Herniated disc, lumbar spinal stenosis, vertebral spondylolisthesis; Spinal infections (such as tuberculosis), tumors, fractures; Inflammatory diseases (such as ankylosing spondylitis).  3. Radicular low back pain/sciatica — Caused by nerve root compression or irritation, accompanied by radiating pain, numbness, and abnormal reflexes in the lower limbs.  **(ii) Classification by duration of disease**  - Acute low back pain: less than 6 weeks  - Subacute low back pain: 6–12 weeks  - Chronic low back pain: greater than 12 weeks (chronic cases are often accompanied by functional impairment and psychosocial factors)  **(iii) According to the nature/distribution of pain**  - Axial (localized) low back pain — limited to the lower back and does not radiate.  - Radiating lower back pain — pain radiates along the sciatic nerve to the lower limbs.  - Mixed type — presents with both localized and radiating pain.  **(iv) Three common clinical classifications used in international guidelines (simplified)**  - Simple back pain — without neurological symptoms and without serious underlying diseases.  - Accompanied by nerve root pain.  - Serious spinal pathology (red flag sign) — should raise suspicion of fractures, tumors, infections, or inflammatory diseases. |

| **Question 4** | **What are some recommendations for non-pharmacological interventions for lower back pain?** |
| --- | --- |
|  | **I. Education and Self-Management**  - Educate patients to understand back pain correctly: most back pain is nonspecific and self-limiting, and does not necessarily indicate serious injury.  - Keep active and avoid prolonged bed rest. Short-term rest is only recommended during the acute phase; prolonged bed rest will delay recovery.  - Lifestyle adjustment: maintain a regular sleep schedule, control weight, avoid prolonged sitting, and quit smoking.  **II. Exercise Therapy**  The guidelines unanimously agree that exercise is the most effective and evidence-based intervention. Suitable for subacute or chronic lower back pain, and to prevent recurrence.  Type recommendations:  - Core stability training, Pilates, yoga, Tai Chi  - Aerobic exercise (walking, swimming, cycling)  - Strength and flexibility training  Key points: individualized planning, gradual approach, at least 6–12 weeks.  **III. Physical Therapy and Rehabilitation Techniques**  Spinal manipulation therapy (spinal adjustment/massage): can provide short-term relief of symptoms, especially for subacute lower back pain. Combining exercise guidance with manual techniques yields better results.  Conventional ultrasound, traction, passive electrotherapy: due to insufficient supporting evidence, these are not the first choice for routine use.  **IV. Psychological Intervention (especially for chronic low back pain)**  - Cognitive Behavioral Therapy (CBT): helps patients overcome fear-avoidant behaviors and improve chronic pain.  - Mindfulness-based stress reduction: has an auxiliary effect on chronic lower back pain and accompanying anxiety and depression.  **V. Multidisciplinary Rehabilitation**  For patients with chronic, recurrent, or severely limited function, it is recommended that a team consisting of doctors, rehabilitation therapists, and mental health professionals implement: functional training + education + psychological intervention + pain management.  **VI. Acupuncture, Yoga, Tai Chi**  International guidelines (ACP, WHO) recognize that acupuncture, yoga, and tai chi can improve pain and function in some populations as complementary therapies. |
| **Question 5** | **What are some recommendations for drug intervention for lower back pain?** |
|  | **I. General Principles**  - Medication is only an adjunct measure. Medication cannot replace core treatments such as exercise, rehabilitation, and education.  - Assess the patient's underlying diseases, comorbidities, and risk of adverse reactions before use.  - Short-term, lowest effective dose — avoid long-term dependence, especially on opioids.  - Non-pharmacological interventions should be considered first. Medication should only be considered when non-pharmacological interventions are ineffective or when pain significantly impairs function.  **II. Recommended Commonly Used Drugs**  1. Nonsteroidal anti-inflammatory drugs (NSAIDs)  The first-line drug (with the best evidence support for acute and subacute low back pain). Usage principles: use for the shortest possible time, and combine selective COX-2 inhibitors or traditional NSAIDs with gastric mucosal protection. Be aware of cardiovascular, digestive, and kidney risks.  2. Muscle relaxants (short-term)  Can be used for a short period during the acute phase when muscle spasms occur (such as tizanidine). Long-term or unindicated use is not recommended.  3. Opioids (use with caution)  Use only for severe acute pain, when other medications are ineffective, and for a short period (a few days or less). Routine use is not recommended for chronic lower back pain, as the risks outweigh the benefits.  4. Antidepressants (for chronic lower back pain accompanied by mood problems)  The main drugs are tricyclic antidepressants (such as amitriptyline) or SNRIs (duloxetine), used for chronic low back pain accompanied by depression, anxiety, or central sensitization. Not a first-line drug.  5. Other drugs (not recommended for regular use)  - Oral or injectable glucocorticoids  - Acetaminophen monotherapy (evidence shows limited efficacy)  - Anticonvulsants (such as gabapentin and pregabalin) — should be used only when there is a clear neuropathic pain indication.  **III. Combined or Injection Therapy**  Epidural injection/nerve block: should only be considered when radicular symptoms (sciatica) are severe and conservative treatment is ineffective, and only under strict indications. |
| **Question 7** | **What are the appropriate criteria for home rehabilitation of lower back pain?** |
|  | According to international guidelines (NICE 2020, WHO 2023, ACP 2017) and principles of rehabilitation medicine, patients with low back pain who are suitable for home-based rehabilitation mainly meet the following criteria:  1. Disease Types  - Nonspecific low back pain (without serious structural lesions)  - Mild to moderate radicular low back pain, with relatively stable symptoms  - Chronic or recurrent low back pain requiring long-term functional exercises and self-management  2. Disease Stability  - Vital signs are stable, and pain level is manageable (e.g., VAS score is mild to moderate)  - No obvious worsening trend, no acute and severe deterioration  - Emergency indications that do not require hospitalization or surgery  3. Exclude Red Flag Signs (situations unsuitable for home-based rehabilitation)  The following situations require treatment by a specialist doctor first and are not suitable for home rehabilitation:  - Progressive neurological dysfunction (such as progressive weakness of lower limb muscles and bowel and bladder dysfunction)  - Suspected fracture after severe trauma  - Accompanied by high fever, infection, or history of tumors  - Other life-threatening or functional emergencies  4. Patient Compliance and Safety  - Cognitive and learning abilities are normal; able to understand rehabilitation methods  - Has a certain degree of self-monitoring ability and can follow medical advice  - The home environment is relatively safe and allows for basic rehabilitation training  5. Rehabilitation Goals  - The goal is to restore daily living functions, improve mobility, and prevent relapse  - Willing to maintain long-term exercise and self-management |
| **Question 12** | **What are the recommended treatment goals for patients with subacute and chronic low back pain?** |
|  | 1. Restore and Improve Functionality  To restore daily living, working, and exercising abilities and reduce functional impairment. Improve core stability, flexibility, and endurance, and enhance the load tolerance of the spine and trunk.  2. Control Symptoms and Reduce the Impact of Pain  Pain can be alleviated through multimodal interventions (exercise, psychological, and pharmacological support), thereby reducing the limitations on daily life caused by pain. Change the fear of pain and build confidence in activity.  3. Prevent Chronicity and Relapse  Reduce the recurrence rate of lower back pain through regular exercise, posture adjustment, and lifestyle interventions. Break the vicious cycle of "pain - fear of movement - decreased function - increased pain."  4. Improved Psychosocial Functioning  Address accompanying anxiety, depression, and sleep disorders, and restore social participation. Reduce fear-avoidant behaviors caused by chronic pain.  5. Improve Patients' Self-Management Abilities  To help patients learn self-regulation, scientific exercise, and self-monitoring, thereby reducing their long-term dependence on medications and medical resources. |
| **Question 21** | **For patients with acute or subacute low back pain, which non-pharmacological treatments should clinicians prioritize, and why are these treatments highly recommended?** |
|  | Non-pharmacological treatment recommendations for acute or subacute low back pain  Clinical guidelines (such as NICE and ACP) recommend prioritizing non-pharmacological treatments for acute or subacute low back pain. The main measures and rationale for these recommendations are as follows:  1. Keep Active  - Objective: To promote healing and prevent muscle stiffness and atrophy.  - Approach: Encourage patients to continue their daily activities and avoid prolonged bed rest.  - Effects: Reduces pain and speeds up recovery.  2. Exercise Therapy  - Objective: To enhance lumbar flexibility and core muscle strength, providing support for the spine.  - Method: Targeted stretching and strengthening exercises.  - Effects: Reduces pain and improves function.  3. Physical Therapy  - Objective: To relieve pain and restore normal activity through various means.  - Methods: manual therapy, ultrasound, low-frequency electrical stimulation, etc.  - Effects: Quickly relieves pain and improves mobility in a short period of time.  4. Educational and Cognitive Behavioral Therapy (CBT)  - Objective: To help patients understand the causes and recovery process of lower back pain and to adjust psychological factors.  - Methods: Pain education and cognitive behavioral intervention.  - Effects: Reduces anxiety and fear, and enhances patients' active participation in rehabilitation.  5. Heat Therapy or Cold Therapy  - Objective: To provide short-term pain relief.  - Methods: Apply heat to relax muscles, and apply cold to reduce inflammation and swelling.  - Effects: Short-term pain relief and improved comfort.  Overall Recommendation Reasons  - High safety: Non-drug methods have fewer side effects and are safer and more reliable than drugs.  - Increase patient engagement: encourage active participation in rehabilitation, improve self-management skills, and adherence.  - Evidence-based support: Multiple studies and guidelines have demonstrated effectiveness in improving function and reducing pain.  - Improve quality of life: Promote the return to normal activities and reduce the negative impact of back pain on life.  These non-pharmacological treatments combine physical and psychological interventions to provide a comprehensive management approach that not only relieves pain but also improves function and quality of life for patients. |
| **Question 23** | **Why is it not recommended to use imaging examinations (such as X-ray, CT, or MRI) as a routine method for diagnosing chronic nonspecific low back pain unless a specific cause is suspected?** |
|  | Guidelines for the Application of Imaging Examinations in Chronic Nonspecific Low Back Pain  Imaging examinations (X-ray, CT, MRI) are not used as routine diagnostic methods for chronic nonspecific low back pain for the following reasons:  1. Non-specific Characteristics  - Limitations: Nonspecific low back pain usually has no clear anatomical lesions, and imaging examinations are difficult to provide a clear cause.  - Inconsistent imaging findings with symptoms: Even with mild disc herniation or degenerative changes, these abnormalities may be unrelated to symptoms, as similar imaging changes are common in asymptomatic individuals.  2. Abuse and Overuse  - Resource consumption: Imaging examinations are expensive, and over-reliance on them may lead to a waste of medical resources.  - Increased costs: Ineffective tests increase medical expenses without necessarily improving patient outcomes.  3. Psychological and Behavioral Effects  - Increased anxiety: Over-interpretation of imaging abnormalities may trigger worry or anxiety in patients.  - Reduced confidence: In the absence of a clear lesion, over-reliance on imaging examinations may undermine the confidence that patients gain through education and self-management.  4. Limited Guidance for Treatment  - Minimal impact on decision-making: Treatment of nonspecific low back pain relies primarily on symptom improvement rather than correction of imaging abnormalities.  - Natural prognosis: Most chronic low back pain can be gradually relieved through conservative treatment (such as exercise therapy and physical therapy), and imaging examinations do not change the natural recovery process.  5. Recommended Usage Scenarios (per guideline)  Imaging should only be used when a serious underlying cause is suspected, such as:  - Unexplained weight loss or fever  - Significant neurological dysfunction (such as decreased muscle strength, sensory abnormalities)  - Accompanied by trauma or severe structural abnormalities  Objective: To investigate potential serious causes such as infection, tumors, or fractures.  Summary  Routine imaging examinations are not recommended unless there is a clear clinical indication. Management focus: Improve patients' quality of life and treatment outcomes through comprehensive clinical assessment, functional rehabilitation, education, and self-management guidance, while avoiding unnecessary examinations and overtreatment. |
