# Supplementary material for: Deficiencies in clinical reasoning of LLMs in low back pain management and remediation via prompt engineering: from performance evaluation to error diagnosis
Source: Front Artif Intell. 2026 May 25;9:1811701. doi: 10.3389/frai.2026.1811701 (PMC13243384; doi:10.3389/frai.2026.1811701)
Supplement: Supplementary file 1 [file Data_Sheet_1.zip › 补充材料/Supplementary materials 5/GPT-4o/B(GPT-40).docx]

| **Question 1** | **How to define and classify lower back pain? (With safety precautions and evidence-based details)** |
| --- | --- |
|  | The following is a systematic, actionable explanation of the definition and classification of low back pain, based on the consensus of mainstream international clinical guidelines (ACP, NICE, WHO, North American Spine Society, etc.).  **I. Standard Definition of Low Back Pain (clinically applicable)**  Low back pain (LBP) refers to pain, discomfort, or tension located between the lower edge of the 12th rib and the gluteal cleft, which may or may not be accompanied by radiating pain in the lower limbs.  Key points: The location of the pain is the key factor in defining the condition, not the imaging or the underlying cause. Whether the pain radiates to the legs does not determine whether it is "lower back pain"; it only affects the classification.  **II. Classification by "Etiology Clarity" (The Most Important Clinical Classification)**  This is the most frequently used classification method in the guidelines, directly determining whether emergency treatment, imaging, or referral is required.  1. Nonspecific lower back pain  Definition: No clear, singular, specific pathological structure or disease could be found to explain the pain. They account for approximately 85–90% of low back pain cases (evidence is highly consistent).  Clinical features: related to activity, posture, and load; no severe neurological deficits; no systemic disease manifestations.  Common situations: muscle/fascia dysfunction; limited joint mobility; pain sensitization (especially in the chronic phase).  Safety tip: This is a "diagnosis of exclusion" — the red flag must be ruled out first. This does not mean "no problem," but rather "no evidence of a dangerous cause."  2. Specific low back pain  Definition: Caused by a clear, identifiable pathological reason.  Common causes: Vertebral fracture; Infections (intervertebral discitis, vertebral osteomyelitis); tumors; Inflammatory spinal diseases (such as ankylosing spondylitis); Pain associated with visceral diseases (such as an aortic aneurysm).  Safety red lines (any one of which requires medical evaluation): Fever, chills; Nocturnal progressive pain; Unexplained weight loss; Previous history of malignant tumors; Severe or minor trauma plus osteoporosis.  3. Radicular pain / lower back and leg pain  Definition: Lower back pain accompanied by radiating pain in the lower limbs along the nerve distribution; mostly caused by nerve root irritation or compression.  Typical manifestations: Radiation to the buttocks, thighs, calves, or feet; coughing, sneezing, and straining worsen the symptoms; may be accompanied by numbness, tingling sensations, or decreased muscle strength.  Common causes: Herniated disc; Spinal stenosis.  Safety tip: If progressive muscle weakness, sensory disturbances, or bowel and bladder abnormalities occur, emergency treatment is required. Not all radiating pain requires surgery.  **III. Classification by "Disease Duration" (for treatment strategy selection)**  1. Acute lower back pain — 6 weeks or less  Most cases can self-limit and recover.  Safety point: If symptoms worsen significantly or a red flag appears within 2–4 weeks, reassess.  2. Subacute lower back pain — 6–12 weeks  This is a critical window of opportunity to prevent chronicity.  Safety point: During this stage, avoid simply waiting for it to heal on its own. Active functional recovery and psychological risk screening are needed.  3. Chronic lower back pain — greater than 12 weeks  Often accompanied by pain sensitization, psychological and social factors.  Safety point: Persistent pain does not mean persistent structural damage. Treatment goals should shift towards function and quality of life. The time boundaries (6 weeks and 12 weeks) are a guideline consensus, not absolute biological limits.  **IV. Classification by "Pain Mechanism" (especially important for chronic low back pain)**  1. Mechanical lower back pain — closely related to activity, posture, and load; rest can alleviate the symptoms.  2. Inflammatory lower back pain  Typical characteristics: Night pain; Morning stiffness lasting more than 30 minutes; Symptoms relieved after activity but worsened after rest; Onset at a young age (under 40 years old).  Safety tip: Meeting the above characteristics — consider rheumatological and immunological assessment.  3. Neuropathic pain — burning, electric shock-like, needle prick-like; often accompanied by sensory abnormalities.  4. Pain sensitization/central pain (moderate evidence)  The degree of pain is disproportionate to the peripheral damage. Commonly seen in chronic nonspecific low back pain. Diagnostic criteria are still under development (evidence is not yet fully conclusive).  **V. Clinically Mandatory Safety Checklist**  Red flag symptoms (any one of these = not treated as ordinary lower back pain):  - Bowel and bladder dysfunction  - Abnormal sensation in the saddle area  - Progressive muscle weakness  - Fever, signs of infection  - History of cancer  - Severe or high-risk trauma  Imaging precautions: Nonspecific low back pain does not routinely indicate X-ray. Abnormal imaging findings do not identify the source of pain. Imaging is only used for: red flag assessment, preoperative/interventional evaluation, and reassessment after long-term conservative treatment has failed.  **VI. A Clinically Applicable Classification Execution Sequence**  1. First, ask about red flag signs — if present, immediately refer the patient.  2. No red flag — determine if there are nerve root symptoms.  3. If not, classify as nonspecific low back pain.  4. Further stratify by disease course (acute/subacute/chronic).  5. In the chronic phase, supplement with pain mechanism assessment.  Summary: Lower back pain is first classified according to "whether there is a dangerous underlying cause," and then stratified according to time and mechanism; imaging and labeling cannot replace clinical judgment; safety screening always takes precedence over detailed subtyping. |
| **Question 4** | **What are some recommendations for non-pharmacological interventions for lower back pain? (With safety precautions and evidence-based details)** |
|  | Based on the consistent conclusions of mainstream international clinical guidelines (ACP, NICE, WHO, North American Spine Society, etc.), the following systematically answers non-pharmacological intervention recommendations for low back pain.  **I. General Principles**  Before implementing any non-pharmacological intervention, the following safety checks must be completed:  1. The "red flag" must be eliminated first.  If any of the following conditions are present, they are not treated as standard lower back pain, and an immediate medical evaluation is required:  - Progressive lower limb weakness and sensory disturbance  - Numbness in the saddle area and abnormal bowel and bladder function  - Fever, signs of infection  - Unexplained weight loss, history of cancer  - Severe or minor trauma plus osteoporosis  Non-pharmacological interventions are not an alternative in the red flag state.  2. Prioritization of Non-pharmacological Intervention Targets (Guideline Consensus)  - Recovery of function and activity  - Reduce pain-related fear and avoidance  - Prevent chronicity  Note: Complete disappearance of pain is not the primary goal.  **II. Basic Non-Drug Interventions Applicable to All Stages**  1. Stay active and avoid bed rest (highly recommended)  How to do it: Avoid bed rest for more than 24–48 hours. Continue daily activities, walking, and light physical activity.  Evidence-based reasoning: Clearly shortens the course of the disease and reduces the risk of chronicity. Bed rest is not helpful and may even delay recovery.  Safety precautions: Discomfort is allowed during the activity. If the pain continues to worsen significantly or neurological symptoms appear, stop and reassess.  2. Patient education and expectation management (highly recommended)  How to do it: Clearly inform: most back pain is benign and reversible; pain does not equal structural "damage"; explain why imaging is not done routinely.  Evidence-based reasoning: Can reduce fear-avoidant behavior; reduces unnecessary tests and overtreatment.  Safety precautions: Avoid catastrophic or threatening language. Information should be matched to the patient's cognitive level.  **III. Non-pharmacological intervention recommendations based on disease stage**  **(a) Acute and subacute low back pain (12 weeks or less)**  3. Superficial heat therapy (highly recommended for short-term use)  How to do it: Apply heat for 15–20 minutes, 1–3 times daily.  Evidence-based reasoning: Consistent evidence for short-term pain relief. Low cost, feasible at home.  Safety precautions: Prevent burns. Use with caution in patients with diabetes or sensory impairment.  4. Early, low-intensity exercise guidance (highly recommended)  How to do it: Walking, activity range practice, light core control (non-strength training).  Evidence-based reasoning: Early activity can reduce the risk of chronicity.  Safety precautions: If radiating pain worsens significantly or muscle strength decreases, stop.  5. Spinal manipulation therapy (highly recommended under certain conditions)  How to do it: Performed by a formally trained physician or physical therapist. Short-term use.  Evidence-based reasoning: Moderate-quality evidence for short-term improvement in pain and function.  Safety red line — disabled for: Fractures, infections, tumors; Severe osteoporosis; Progressive neurological deficits.  **(b) Chronic nonspecific low back pain (greater than 12 weeks)**  6. Exercise therapy (the most crucial intervention, highly recommended)  Recommended format: Aerobic exercise (walking, cycling, swimming); Core stability training; Strength and endurance training; Tai Chi, Yoga, Pilates.  Evidence-based reasoning: All guidelines agree that any regular exercise is better than no exercise. There is no single "best type of exercise."  Safety precautions: Start with low intensity and gradually increase. A temporary increase in pain does not necessarily mean danger.  7. Cognitive Behavioral Therapy (CBT) — highly recommended  How to do it: Implemented by professionals, combining motor and functional goals.  Evidence-based reasoning: Can improve function and reduce pain catastrophizing. There is sufficient evidence for chronic low back pain.  Safety precautions: This does not mean "psychological problems equal faking illness." Should be combined with biomechanical intervention.  8. Multidisciplinary comprehensive rehabilitation (highly recommended)  Composition: Exercise + Education + Psychological Intervention, targeting work and life functional goals.  Evidence-based reasoning: For intractable chronic low back pain, it is superior to a single treatment.  Safety precautions: Needs to be individualized, not a "one-size-fits-all" approach.  **IV. Non-pharmacological interventions with limited or conditional evidence**  - Acupuncture: Moderate evidence — can be used as an auxiliary means.  - Massage: Short-term effective — does not replace active rehabilitation.  - Traction: Insufficient evidence — not recommended as a regular practice.  - Lumbar support: Limited evidence — for short-term use only.  - Physical agents (electrotherapy, ultrasound): Weak evidence — not as a core treatment.  **V. Safety Principles (Must Be Emphasized Repeatedly)**  - Non-pharmacological intervention does not mean no medical evaluation is required.  - Increased pain does not mean worsened structural damage (especially in the chronic phase).  - Long-term passive treatment does not equal recovery.  - Functional recovery takes precedence over pain score.  Summary: Non-pharmacological interventions for low back pain focus on "activity, exercise, and education," with safety screening always taking priority, and passive treatment cannot replace active rehabilitation. |
| **Question 5** | **What are some recommendations for medication interventions for lower back pain? (With safety precautions and evidence-based details)** |
|  | Based on the highly consistent conclusions of mainstream international clinical guidelines (ACP, UK NICE, WHO, North American Spine Society, etc.), the following systematically explains the recommendations for pharmacological interventions for low back pain.  Important premise: Medication plays a supporting role in low back pain management and cannot replace non-pharmacological interventions (activity, exercise, education). All medication decisions must be made only after ruling out red flag causes.  **I. General Principles of Drug Therapy (Guidelines Consensus)**  - Prioritize non-pharmacological interventions, with medication as a supplement.  - Short-term, lowest effective dose, and a clear discontinuation plan.  - The goal is to improve function, not to eliminate pain.  - Extreme caution is needed when using medication for chronic lower back pain long-term.  **II. Medication Recommendations for Acute and Subacute Low Back Pain (12 weeks or less)**  1. Nonsteroidal anti-inflammatory drugs (NSAIDs) — First-line recommendation  Applicable to: Acute/subacute low back pain patients without obvious contraindications; pain that affects activity or sleep.  Common choices: Ibuprofen, naproxen, diclofenac, etc. (Guidelines do not recommend any one NSAID as "absolutely better.")  Evidence-based basis: Moderate-quality evidence regarding pain and functional improvement. Clearly superior to placebo.  Execution details: Use the lowest effective dose. Continuous use should ideally not exceed 1–2 weeks. Take as needed, rather than taking it long-term.  Key safety precautions: Gastrointestinal bleeding, kidney damage, cardiovascular risk. The following groups should use with caution or avoid: history of peptic ulcer; chronic kidney disease; high cardiovascular disease risk groups; elderly patients. Combination with gastric mucosal protectants (such as PPIs) may be considered, but this does not eliminate all risks.  2. Acetaminophen (paracetamol) — no longer recommended as a first-line treatment alone  Current status: Multiple high-quality studies have shown that the efficacy of this treatment for acute low back pain is no better than that of a placebo.  Clinical positioning: If NSAIDs are contraindicated, it can be used as a short-term alternative. Long-term use alone is not recommended.  Safety precautions: Risk of liver toxicity. Strictly avoid exceeding the recommended dosage or using it in combination with alcohol.  3. Skeletal muscle relaxants — can be considered for short-term use  Applicable to: Obvious muscle spasms; patients with inadequate or intolerable NSAID efficacy.  Evidence-based basis: Evidence for short-term pain relief; no improvement in long-term outcomes.  Execution details: For short-term use only (usually 1 week or less).  Key safety precautions: Drowsiness, dizziness, cognitive impairment. Risk of falls increases significantly in older adults. Do not use with alcohol or sedatives.  **III. Medication Recommendations for Chronic Nonspecific Low Back Pain (greater than 12 weeks)**  4. NSAIDs — Conditional recommendation  Applicable to: Insufficient efficacy of non-pharmacological interventions; no obvious contraindications.  Evidence-based basis: Still has a certain analgesic effect on chronic lower back pain. However, long-term safety issues remain prominent.  Implementation principles: Intermittent, short-term use. Long-term continuous use is not recommended.  Safety priorities: Long-term risks outweigh short-term benefits. Kidney function and gastrointestinal risk need to be assessed regularly.  5. Antidepressants (such as duloxetine) — recommended in specific situations  Applicable to: Chronic low back pain combined with significant pain sensitization; depression or anxiety; widespread pain characteristics.  Evidence-based basis: Moderate evidence for improvement in function and pain in patients with chronic low back pain.  Safety precautions: Nausea, insomnia, blood pressure changes. Medication should be started and stopped gradually. Not suitable for all patients with chronic lower back pain.  **IV. Medications Explicitly Not Recommended or Highly Restricted**  NSAIDs: Not recommended for routine use for lower back pain. Limited to a very small number of strictly selected short-term users. Reason: limited improvement in functionality; risks of addiction, tolerance, and overdose are clear.  Glucocorticoids (oral or injectable, not for specific indications): No clear benefit for nonspecific low back pain. Regular use is not recommended.  Antiepileptic drugs (such as gabapentin, pregabalin): Generally ineffective for lower back pain or sciatica. Many adverse reactions. Most guidelines explicitly do not recommend.  **V. Mandatory Safety Checklist for Drug Therapy**  Before prescribing any medication, it must be confirmed that:  - Is there a red flag cause (if so — treat the cause first)?  - Have non-pharmacological interventions been tried or implemented concurrently?  - Are there any contraindications to the medication, or is the patient in a high-risk group?  - Is it clear: purpose of medication; use period; discontinuation criteria?  **VI. Simplified Drug Ladder for Direct Clinical Use**  1. Non-pharmacological basis.  2. Acute/subacute: NSAIDs (preferred), plus or minus short-term muscle relaxants.  3. Chronic: Intermittent NSAIDs.  4. Opioids: Avoid in principle. Consider antidepressants in specific situations.  Summary: Drug treatment for lower back pain should be short-term, cautious, and function-oriented; no medication can replace activity and exercise. |
| **Question 7** | **What are the appropriate criteria for home rehabilitation of lower back pain? (With safety precautions and evidence-based details)** |
|  | The following content is compiled based on authoritative clinical guidelines and expert consensus (including the Chinese rehabilitation medicine community's statements and international guidelines for the management of chronic low back pain), clearly stating the criteria for suitable home rehabilitation and all safety precautions.  **I. Criteria for Suitable Candidates for Home-Based Rehabilitation**  The following standards are applicable to assessing whether patients with nonspecific low back pain can begin rehabilitation training at home when their condition is stable:  1. The diagnosis is clear, and there are no serious structural abnormalities  - The patient has been diagnosed with lower back pain at a regular medical institution.  - Imaging studies (X-ray/CT/MRI) have ruled out dangerous structural lesions such as severe fractures, vertebral instability, or severe compression of the spinal cord or nerve roots.  2. The patient's condition is stable, and there are no signs of acute danger  - Vital signs are normal, with no fever or signs of acute infection.  - No unexplained, severe nighttime pain that prevents sleep.  - No severe pain that cannot be relieved by conventional conservative treatments.  3. No complications or comorbidities requiring hospitalization  - No other serious illnesses requiring emergency medical monitoring (such as severe cardiopulmonary disease, unstable diabetes, acute inflammation).  - The patient has mild to moderate functional impairment with relatively good daily living activities, and the patient/caregiver can cooperate with the rehabilitation plan.  4. The patient is unable or finds it inconvenient to go to a medical institution  For example: limited mobility, difficulty in accessing remote medical care, or inability to access continuous rehabilitation services. Even so, it is still necessary to confirm that the aforementioned basic safety conditions are met.  Simplified Standards for Home-Based Rehabilitation:  - Suitable: Diagnosed with lower back pain, no serious structural abnormalities, symptoms are stable and pain is controllable, no serious complications, mild to moderate functional impairment.  - Unsuitable (transfer to another facility): Suspected fracture, spinal cord/nerve root compression, acute uncontrollable pain, severe pain at night, other illnesses requiring hospital treatment, severe functional loss or limitation of daily life.  **II. Scope of Application and Basic Principles**  International guidelines (ACP and WHO) emphasize that rehabilitation training (exercise, education, and behavioral modification) is an important component of chronic low back pain management and support its implementation in community and home settings.  Examples of rehabilitation types suitable for home-based care:  - Light to moderate intensity aerobic exercise (such as walking)  - Core stability training/motor control training  - Flexibility and stretching training  - Patient education and posture correction exercises  **III. Safety Precautions for Home-Based Rehabilitation**  1. Training intensity and pain feedback control  When starting rehabilitation training, begin with low intensity and short duration, gradually increasing the intensity. If you experience a significant increase in pain, worsening of neurological symptoms (such as numbness/tingling in the lower extremities, decreased muscle strength), or persistent pain after exercise, immediately stop training and contact your doctor for a reassessment.  2. Red flag sign monitoring (training must be stopped and medical attention sought)  If a patient experiences any of the following symptoms during home rehabilitation, all training should be stopped and medical attention sought as soon as possible:  - Symptoms associated with acute cauda equina syndrome (such as saddle area paresthesia and bowel and bladder control disorders)  - Significant deterioration of neurological function (e.g., gait instability, new-onset significant decrease in muscle strength)  - Significant weight loss, signs of infection (high fever), or unexplained, persistent, severe pain  3. Personalized adaptation and safety support  If the patient has osteoporosis, joint instability, severe cardiopulmonary disease, or other chronic systemic diseases, individual adjustments should be made to the home rehabilitation plan, or remote supervision/in-person assessment should be considered. For individuals with cognitive impairment or those whose mobility is affected by pain, they should be accompanied and supervised by family members or caregivers.  4. Suggestions for postures and activities in daily life  - Avoid prolonged sitting or standing; recommended to change position and move around slightly every 30–45 minutes.  - Avoid large forward bends, excessive twisting of the waist, or strenuous physical activities, especially in the early stages of rehabilitation.  - Maintain good sitting and sleeping postures (recommended to keep the spine neutral) and use appropriate support.  **IV. Clear Indications for Transfer to Another Medical Institution**  The following conditions are not suitable for self-recovery at home and require prompt medical evaluation and treatment at a hospital:  - Moderate to severe pain that persists and cannot be relieved  - Acute neurological dysfunction occurs or worsens  - Accompanied by severe systemic symptoms (fever, signs of infection, unexplained weight loss)  - Significant loss of function (e.g., complete inability to walk independently or perform daily activities)  **V. Summary of Executable Process (Home Version)**  1. Clinical assessment and exclusion of risk factors (physician/rehabilitation therapist)  2. Develop individualized home-based rehabilitation plans (including type, duration, and intensity)  3. Conduct training and monitor pain and functional changes  4. Follow the safety red flag signals — stop training immediately and seek medical attention |
| **Question 12** | **What are the recommended treatment goals for patients with subacute and chronic low back pain? (With safety precautions and evidence-based details)** |
|  | Based on the consensus of mainstream international evidence-based guidelines (ACP, NICE, WHO, North American Spine Society, etc.), the following systematically answers the recommended treatment goals for patients with subacute and chronic low back pain.  **I. A Core Premise (Very Important)**  The goal of treating subacute and chronic low back pain is not to "eliminate the pain," but to "restore function and reduce the risk of chronicity and disability." This is a highly consistent conclusion from all authoritative guidelines, and it is also the biggest difference from acute low back pain.  **II. Recommended Treatment Goals for Subacute Low Back Pain (6–12 weeks)**  The subacute phase is a critical window of opportunity to prevent chronicity.  1. Primary goal: Restore or maintain daily function (highly recommended)  Specific goals: Can it continue or gradually recover? Work/Study; Daily chores; Basic physical activity (e.g., walking 20–30 minutes or more).  Evidence-based basis: Functional level is a better predictor of whether chronic low back pain will develop than pain intensity. Excessive "waiting until the pain subsides before starting to move" can significantly increase the risk of chronicity.  Safety precautions: Functional recovery can be performed "in pain." If functional activities lead to persistent deterioration for more than 24–48 hours — the treatment plan needs to be adjusted.  2. Reduce fear-avoidance behavior (highly recommended)  Specific goals: The patient can understand and accept that the activity is safe; pain does not necessarily mean worsening of the injury.  Executable metrics: The patient is willing to gradually increase the amount of activity; no longer avoiding activities for extended periods due to fear of "injuring my back."  Evidence-based basis: Fear-avoidance is one of the strongest predictors of chronic low back pain.  Safety precautions: Not a forced activity. A reassessment is required if neurological symptoms worsen.  3. Pain control goal: Sufficient to support activity (not completely pain-free)  Specific goals: Pain levels are reduced to a point where they do not interfere with daily activities and rehabilitation training.  Evidence: Pursuing complete painlessness during the subacute phase does not improve long-term outcomes. Functional improvement can be achieved even before the pain has completely disappeared.  **III. Recommended Treatment Goals for Chronic Low Back Pain (greater than 12 weeks)**  4. Core Goal 1: Improve functionality and quality of life (Highest Recommendation)  Specific goals — improve at least one of the following: Ability to walk, sit for long periods, and stand for long periods; Work or social participation; Self-care ability.  Quantifiable tools (common but not mandatory): ODI (Oswestry): improvement of 10 points or more; RMDQ: improvement of 5 points or more. These are commonly used minimum clinically important differences (MCID) reference values, not hard targets.  Safety precautions: The target may be appropriately lowered for elderly patients or patients with multiple coexisting diseases.  5. Core Goal 2: Develop long-term self-management skills (highly recommended)  Specific goals: The patient can maintain regular exercise; actively respond to pain fluctuations; avoid relying on passive treatment or frequent medical visits.  Evidence-based basis: Self-management ability is one of the most important factors for the long-term prognosis of chronic low back pain.  Safety precautions: Self-management does not mean tolerating dangerous symptoms. If new red flag symptoms appear, medical attention is still required.  6. Pain goal: Reduce the level of pain, not eliminate pain  Recommended wording: "Pain is controllable, predictable, and no longer dominates my life."  Evidence-based basis: In chronic lower back pain, complete pain relief is neither a realistic nor a necessary goal. An excessive pursuit of painlessness can actually increase medical dependence and feelings of frustration.  7. Psychological and social goals (highly recommended, but often overlooked)  Specific goals: Reduce pain catastrophizing thinking; improve sleep; reduce the amplifying effect of anxiety and depression on pain.  Evidence-based basis: Psychosocial factors have an impact on chronic low back pain that is no less significant than that of biomechanical factors.  Safety precautions: Psychological intervention is not about "the pain being fake." Must be combined with movement and functional goals.  **IV. Subacute vs. Chronic Low Back Pain: Target Comparison**  - Subacute: Core objective = prevent chronicity; Pain target = support activities; Functional expectation = restored to near baseline; Management focus = early intervention.  - Chronic: Core objective = improve functionality and quality of life; Pain target = reduce pain; Functional expectation = accepting "high-functioning pain management"; Management focus = long-term self-management.  **V. Mandatory Safety Boundaries for Patients**  Regardless of whether it is subacute or chronic, if any of the following conditions occur, the treatment goals should be adjusted immediately and the patient referred for medical evaluation:  - Progressive lower limb weakness  - Abnormal sensation in the saddle area, changes in bowel and bladder function  - The pain progressively worsens at night  - Fever, signs of infection  - Pain or function continues to deteriorate under standard intervention  Summary: The goal of subacute low back pain is to "prevent it from becoming chronic," while the goal of chronic low back pain is to "get life back on track," not to "pursue pain-free living." |

| **Question 21** | **For patients with acute or subacute low back pain, which nonpharmacological treatments should clinicians prioritize, and why are these treatments highly recommended? (With evidence-based details)** |
| --- | --- |
|  | Based on mainstream international clinical guidelines (ACP 2017, NICE, WHO, and multiple systematic reviews), the following summarizes the strongly recommended non-pharmacological treatments for acute/subacute low back pain and their evidence-based rationale.  **I. Which Non-pharmacological Treatments Are "Preferred and Strongly Recommended"?**  Based on ACP (2017), NICE, WHO, and multiple systematic reviews, the non-pharmacological treatments consistently rated as strong recommendations for acute/subacute low back pain include the following five categories:  1. Stay active and avoid bed rest  Do what: Not bedridden, or only bedridden for very short periods (24–48 hours or less). Continue daily activities, walking, and light physical activity.  Why is it a strong recommendation?  - Clearly shortens the course of the disease and reduces the risk of chronicity.  - Bed rest offers no additional benefits and may even delay recovery.  - Extremely low risk and widest applicability.  Safety red line: If progressive neurological deficits occur or pain continues to worsen after activity — stop treatment and reassess.  2. Patient education and expectation management  Do what: To be clear: most acute/subacute lower back pain is benign and self-limiting; explain that "pain does not equal worsening of structural damage"; explain why imaging is not done routinely.  Why is it a strong recommendation?  - Consistent evidence shows it can significantly reduce fear-avoidant behavior.  - Reduces unnecessary dependence on imaging, drugs, and medical treatment.  - Almost no risk.  Safety points: Avoid catastrophic language (such as "the intervertebral disc is broken"). Information needs to match the patient's cognitive level.  3. Superficial heat therapy (short term)  Do what: Apply heat for 15–20 minutes, 1–3 times daily.  Why is it a strong recommendation?  - Evidence is consistent for short-term analgesia in acute/subacute phases.  - Low cost, feasible at home, and high compliance.  Safety points: Prevent burns. Patients with diabetes or sensory disturbances should exercise special caution.  4. Spinal manipulation therapy (when conditions are suitable)  Do what: Short-term manual therapy performed by trained professionals.  Why is it a strong recommendation?  - Moderate-quality evidence for short-term improvement in pain and function.  - Serious adverse events are rare (provided that red flags are excluded).  Safety red line: Contraindicated in fractures, infections, tumors, severe osteoporosis, and progressive neurological deficits.  5. Early, low-intensity exercise guidance  Do what: Walking, range of motion exercises, basic motor control — instead of waiting until "completely painless" before moving.  Why is it a strong recommendation?  - Can reduce the risk of chronicity.  - The risks are significantly lower than those of medications and invasive treatments.  Safety points: If there is a significant increase in radiating pain and decreased muscle strength, stop immediately.  **II. Why Are These Treatments Unanimously Rated as "Strongly Recommended"?**  From an evidence-based medicine perspective, they simultaneously meet four conditions:  - Clear benefits: improved function, shortened disease course, and prevention of chronicity.  - Extremely low risk: far lower than NSAIDs, opioids, and invasive procedures.  - Highly cost-effective: no complex equipment or inspection required.  - High consistency of evidence: conclusions from different countries and guidelines are highly consistent.  **III. A Crucial Clinical Reminder (Very Important)**  Strong recommendation does not mean indiscriminate use for all patients. A red flag screening must be completed before implementing any of the above.  If any of the following occurs:  - Progressive neurological dysfunction  - Abnormal sensation in the saddle area, abnormal bowel and bladder function  - Nocturnal progressive pain, fever, and weight loss  Immediately stop non-pharmacological pathways and proceed with medical evaluation. |
| **Question 23** | **Why is it not recommended to use imaging studies (such as X-rays, CT scans, or MRIs) as a routine method for diagnosing chronic nonspecific low back pain unless a specific cause is suspected? (With evidence-based details)** |
|  | For patients with chronic nonspecific low back pain (low back pain without a clear and serious pathological cause such as infection, tumor, fracture, cauda equina syndrome, etc.), clinical guidelines explicitly discourage the routine use of imaging examinations as initial or routine diagnostic methods unless a specific cause is suspected.  **I. Why Are Routine Imaging Examinations Not Recommended?**  1. No improvement in clinical outcomes  Multiple guidelines and evidence-based reviews indicate that routine imaging examinations (X-ray, CT, MRI) for patients with nonspecific low back pain do not improve key clinical outcomes such as pain intensity, functional recovery, or quality of life. Even if structural changes are found, they do not change the treatment plan or improve the prognosis. This means that routine imaging does not provide additional clinical benefit to the vast majority of patients with chronic nonspecific low back pain.  2. Imaging findings have a weak correlation with symptoms  Common MRI/CT/X-ray abnormalities (such as intervertebral disc degeneration, mild herniation, etc.) are also common in asymptomatic individuals and do not necessarily mean that these changes are the true cause of the pain. The imaging abnormalities are poorly correlated with the severity or persistence of pain. This can lead to "structural labeling," where patients interpret structural abnormalities as "damage" or "vulnerability," increasing pain, fear, and functional withdrawal. This phenomenon not only does not help with treatment but may also increase the patient's psychological burden and pain experience.  3. Potential adverse consequences include overtreatment  Routine imaging examinations can lead to:  - Over-medicalization (such as unnecessary disc surgery or invasive interventional treatments)  - Significantly increased medical costs  - Delayed diagnosis that hinders truly effective rehabilitation interventions (such as exercise rehabilitation and behavioral therapy)  Evidence-based research shows that patients with low back pain who undergo routine imaging experience significantly increased costs without any improvement in quality of life or function.  4. Guideline consistency  Several authoritative organizations, including the American Academy of Family Physicians (AAFP), the American College of Physicians (ACP), and the North American Spine Society, recommend: imaging should not be routinely performed for nonspecific low back pain in the absence of the "red flag sign." These recommendations are based on the consideration that "there is a lack of evidence to prove the benefits of conventional imaging, while there are clear potential risks."  **II. When Should Imaging Examinations Be Considered?**  Although routine use is not recommended, imaging evaluation should be given priority in the following situations:  1. Appearance of the red flag sign or a high degree of suspicion:  - Progressive neurological deficits (such as decreased muscle strength and worsening numbness)  - Abnormal sensation in the saddle area, loss of urinary and fecal control  - History of primary malignant tumor or strong suspicion of tumor  - Signs of infection (fever, chills, immunosuppression)  - History of severe trauma or high suspicion of fracture  These findings suggest a possible serious pathological cause, requiring urgent imaging evaluation.  2. Pre-operative evaluation for interventional or surgical procedures: When considering interventional/surgical treatments such as discectomy or spinal decompression, imaging studies are used for pre-operative assessment and treatment planning.  3. After 6–12 weeks of conservative treatment (physical therapy, exercise rehabilitation, behavioral therapy) without improvement: Imaging evaluation may be considered based on clinical judgment.  **III. Safety Precautions (Related to Imaging)**  1. Radiation risks associated with X-rays and CT scans  X-rays and CT scans both emit ionizing radiation, with CT scans especially delivering a higher radiation dose. Unnecessary radiation exposure can accumulate risks, especially in younger patients and those undergoing repeated examinations. Clinical implementation: ensure that imaging examinations are scheduled only when there is a clear indication; choose the lowest radiation method when necessary.  2. Safety and contraindications of MRI examination  MRI is radiation-free, but may be contraindicated for patients with metal implants, pacemakers, etc. For individuals with claustrophobia or those unable to remain still, alternative options need to be evaluated.  3. Avoid overdiagnosis and overtreatment  Clinicians should clearly explain: "Imaging findings" do not equate to "establishing the cause of the disease." Many imaging abnormalities are normal aging or structural changes and are not necessarily directly related to pain. This helps prevent patients from misunderstanding their condition or becoming overly reliant on interventional treatment.  **IV. Suggested Implementation Guidelines (Clinically Applicable)**  - Prioritize collecting detailed medical history and conducting physical examinations, paying attention to red flag symptoms.  - Imaging examinations should only be considered when there is a clear clinical indication.  - For patients undergoing initial treatment, wait at least 6–12 weeks for conservative treatment before assessing whether imaging is necessary (unless a red flag is present).  - Provide patient education: inform patients why imaging studies are not used first, and explain that many structural changes are not related to symptoms.  Summary: Routine imaging is not recommended for chronic nonspecific low back pain because it does not improve clinical outcomes, has poor correlation with symptoms, may lead to overdiagnosis, and has potential side effects (such as radiation). Examination should only be considered in specific situations such as red flag signs, reassessment after ineffective conservative treatment, or pre-intervention/operative assessment. |
